# Ancient Gene Duplicates in *Gossypium* (Cotton) Exhibit Near-Complete Expression Divergence

**DOI:** 10.1093/gbe/evu037

**Published:** 2014-02-19

**Authors:** Simon Renny-Byfield, Joseph P. Gallagher, Corrinne E. Grover, Emmanuel Szadkowski, Justin T. Page, Joshua A. Udall, Xiyin Wang, Andrew H. Paterson, Jonathan F. Wendel

**Affiliations:** ^1^Department of Ecology, Evolution and Organismal Biology, Iowa State University, Ames, Iowa; ^2^Plant and Wildlife Science Department, Brigham Young University, Provo, Utah; ^3^Plant Genome Mapping Laboratory, University of Georgia, Athens, Georgia

## Abstract

Whole genome duplication (WGD) is widespread in flowering plants and is a driving force in angiosperm diversification. The redundancy introduced by WGD allows the evolution of novel gene interactions and functions, although the patterns and processes of diversification are poorly understood. We identified ∼2,000 pairs of paralogous genes in *Gossypium raimondii* (cotton) resulting from an approximately 60 My old 5- to 6-fold ploidy increase. Gene expression analyses revealed that, in *G. raimondii*, 99.4% of the gene pairs exhibit differential expression in at least one of the three tissues (petal, leaf, and seed), with 93% to 94% exhibiting differential expression on a per-tissue basis. For 1,666 (85%) pairs, differential expression was observed in all tissues. These observations were mirrored in a time series of *G. raimondii* seed, and separately in leaf, petal, and seed of *G. arboreum*, indicating expression level diversification before species divergence. A generalized linear model revealed 92.4% of the paralog pairs exhibited expression divergence, with most exhibiting significant gene and tissue interactions indicating complementary expression patterns in different tissues. These data indicate massive, near-complete expression level neo- and/or subfunctionalization among ancient gene duplicates, suggesting these processes are essential in their maintenance over *∼*60 Ma.

## Introduction

**Fig. 1.—:**
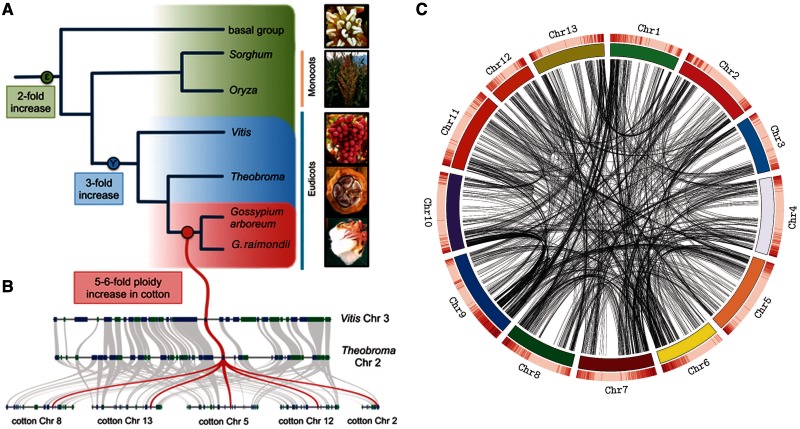
WGD in angiosperms. (*A*) A reconstructed phylogeny of representative angiosperms. Phylogenetic analysis of gene duplicates has revealed two ancient WGD events, one (ζ) at the root of the seed plants (not shown) and another (ε) at the base of the angiosperms (Jiao et al. 2011). More derived duplications at the base of the eudicots (Υ [Vision et al. 2000; Jaillon et al. 2007]), and several in the monocots (σ [Tang et al. 2010] and ρ [Paterson et al. 2004; Wang et al. 2005]) have also been inferred, in addition to multitude of other lineage-specific WGD events (not shown). Sequencing of the *G. raimondii* genome revealed a penta- or hexaploid duplication event (or series of temporally adjacent events) that occurred in the *Gossypium* lineage ∼60 Ma (red circle in *A*). It is important to note that this WGD event is not shared with *T. cacao* or *V. vinifera*. (*B*) A schematic representation of syntenic regions duplicated in *Gossypium* relative to *T. cacao* and *V. vinifera*. Modified with permission from Paterson et al. (2012). (*C*) Circos plot detailing the position and distribution of strictly duplicated genes along chromosomes of the *G. raimondii* genome sequence. A heat map of gene density (dark red is high density, light red low density) is given above each ideogram. Pictures are from top to bottom: *G. hirsutum, T. cacao, V. vinifera, Sorghum bicolor* (Mike Peel; www.mikepeel.net, last accessed February 28, 2014) and *Amborella trichopoda* (Scott Zona).

The role of gene duplication in the genesis of evolutionary novelty and complexity has long been recognized (Stephens 1951; Ohno 1970). Whole genome duplication (WGD or polyploidy) introduces genome-wide genetic redundancy and is considered a driving force in angiosperm evolution (Jiao et al. 2011). WGD is ubiquitous in flowering plants, with recent phylogenetic analyses of gene duplicates revealing two ancient WGD events, one (ζ) occurring at the root of the seed plants and another (ε) occurring at the base of the angiosperms (fig. 1 here; Jiao et al. 2011, fig. 3). Earlier analyses, using assembled plant genomes or collections of expressed sequence tags, also indicate more recent duplications at the base on the eudicots (Υ; Vision et al. 2000 and Jaillon et al. 2007) and several in the monocots (σ and ρ; Tang et al. 2010, Paterson et al. 2004, and Wang et al. 2005). Relatively recent (neopolyploid) events are also well known (Ashton and Abbott 1992; Ainouche, Baumel, Salmon 2004; Ainouche, Baumel, Salmon, Yannic, et al. 2004; Pires et al. 2004; Soltis et al. 2004; Renny-Byfield et al. 2010) and characterize many crop plants, including wheat, tobacco, *Brassica*, apple, banana, sugar cane, and cotton (Wendel and Cronn 2003; Leitch AR and Leitch IJ 2008). The ubiquity of WGD and gene duplication in land plants suggests a crucial role for this process in their evolution and diversification (Jaillon et al. 2007; Leitch AR and Leitch IJ 2008; Soltis et al. 2009; Jiao et al. 2011).

Although the importance of WGD in evolution has long been recognized (Stebbins 1950; Stephens 1951; Stebbins 1971), it has been historically challenging to infer ancient duplication events. This reflects the tendency of polyploid species to undergo diploidization, a suite of processes that return the genome to a more diploid-like state. These diploidization processes include genome downsizing (Leitch and Bennett 2004; Leitch et al. 2008; Renny-Byfield et al. 2011, 2013), establishment of disomic inheritance (Le Comber et al. 2010), chromosomal rearrangement (Clarkson et al. 2005; Lim et al. 2006, 2007; Weiss-Schneeweiss et al. 2008; Kovarik et al. 2011; Chester et al. 2012; Renny-Byfield et al. 2012), chromosome number reduction (Mandakova et al. 2010) and fractionation, and the reciprocal loss of paralogous genes among subgenomes (Langham et al. 2004; Freeling 2009; Freeling et al. 2012). The genomic changes induced during ancient diploidization frequently obscure the signatures of WGD in extant taxa; fractionation, for example, leaves a relatively small number of duplicated genes within paleopolyploid genomes (Langham et al. 2004; Paterson et al. 2012), where, intriguingly, retention is nonrandom (Blanc and Wolfe 2004a; Paterson et al. 2006; Barker et al. 2008, 2009; Buggs et al. 2012; De Smet et al. 2013).

The observation of nonrandom loss of genes following WGD has stimulated much discussion regarding the patterns of loss versus retention and the evolutionary processes that influence these outcomes. Many of these arguments trace to the seminal works of Haldane (1932), Ohno, and others, who posited there must exist a reason some gene duplicates escape mutational obliteration and eventual deletion. Early work invoked a neutral form of neofunctionalization. Following duplication, the possibility of relaxed selection on one of the duplicates allows one of the copies to acquire mutations, and by chance, one or more of these may result in new protein function (Ohno 1970). Subsequent to the emergence of a new function in one of the duplicates, selection or drift can lead to fixation of that function.

A complementary perspective emerged from the work of Lynch and colleagues, who proposed a model of duplication, degeneration, and complementation, the DDC model (Force et al. 1999; Lynch and Conery 2000; Lynch and Force 2000), whereby retention of duplicates is achieved when both genes are rendered essential by a process of subfunctionalization. In this case, the function of an ancestral gene is partitioned between the two duplicates via complementary and degenerate mutations so that both duplicates are needed to maintain the original function of the single ancestral gene (Force et al. 1999; Lynch and Conery 2000; Lynch and Force 2000; Prince and Pickett 2002). Subfunctionalization can take the form of partitioning protein function between duplicates or, perhaps more commonly, partitioning of gene expression, so that duplicates have complementary expression patterns (Prince and Pickett 2002).

Recent research in neopolyploids has elucidated the importance of subfunctionalization in the context of polyploidy, particularly in angiosperms where several accounts of rapid subfunctionalization via tissue-specific reciprocal silencing have been described (Adams et al. 2003, 2004; Buggs et al. 2010). These studies were limited in scope when compared with the potential of modern high-throughput sequencing, and only a few cases of tissue-specific reciprocal silencing were demonstrated. Because most genes duplicated by WGD are subsequently lost, the subfunctionalization observed in neopolyploids may not reflect evolutionary processes that operate over longer time frames. Relatively little is known about the long-term balance between the processes of gene loss and sub- and neofunctionalization. Understanding the molecular, functional, and expression level divergence of retained gene duplicates is needed to appreciate the role of gene duplication in the generation of evolutionary complexity.

To understand the forces that govern the maintenance of gene duplicates following WGD, we took advantage of the recently published genome sequence of *Gossypium raimondii* (a D-genome cotton), which revealed a striking signal of a 5- or 6-fold ploidy increase that occurred approximately 60 Ma (fig. 1). Here, we assess expression level neo- and subfunctionalization following this ancient polyploidization. Using ∼2,000 pairs of strictly duplicated genes, whose origin traces to the *Gossypium*-specific ancient polyploidy event(s) (Paterson et al. 2012), we compare sequence and expression-level divergence among these duplicates in three tissues of *G. raimondii* and a sister species, *G. arboreum*. The data demonstrate massive, near-complete expression-level divergence among duplicates, consistent with regulatory neo- and/or subfunctionalization, and provide a genome-scale view of expression level evolution tracing to ancient polyploidy.

## Materials and Methods

### Identification of Paralogous Gene Pairs

We identified groups of paralogous genes in *G. raimondii* originating from the *Gossypium*-specific whole genome multiplication event identified by Paterson et al. (2012) using both syntenic information and sequence similarity between genes in *G. raimondii* and their orthologs in *Vitis vinifera* (Jaillon et al. 2007) and *Theobroma cacao* (Argout et al. 2011). As the *V. vinifera*, *T. cacao*, and *G. raimondii* genomes share an even more ancient triplication event (fig. 1), we identified strictly duplicated genes in the *G. raimondii* genome as those present in duplicate syntenic regions in *G. raimondii,* but which traced to only a single genomic region in the *T. cacao* and *V. vinifera* genomes. We then used chromosome coordinates for all paralogous pairs to visualize the distribution of paralogs over the *Gossypium raimondii* genome assembly using the program Circos (Krzywinski et al. 2009). For each paralogous pair, coding domain sequences of their primary transcripts were aligned using ClustalW (Chenna et al. 2003), and d*N*/d*S* ratios were measured using custom BioPerl scripts and a Jukes–Cantor substitution model (Jukes and Cantor 1969).

### RNA-seq Data, Quality Control, and Read Mapping

Gene expression analysis in several tissues and time points was used to assess the expression patterns of the ∼2,000 strictly duplicated gene pairs. Transcriptomic RNA-seq data from previous analyses were retrieved from the NCBI SRA database for three *G. raimondii* tissues: leaf (Yoo et al. 2013; SRX172483-SRX172485), seed (Paterson et al. 2012; SRX204399-SRX204401, SRX204405-SRX204407, and SRX204429-SRX204434), and petal (Rambani et al. 2014; SRX328344). Similarly, leaf (Yoo et al. 2013; SRX170955, SRX172454, SRX172473), seed (SRX204555-SRX204558), and petal (Rambani et al. 2014; SRX328344) RNA-seq data for *G. arboreum* were also retrieved. The assembled data set consisted of three biological replicates per tissue and/or time points for both *G. raimondii* and *G. arboreum*.

Each RNA-seq library was screened for quality using the program sickle (https://github.com/najoshi/sickle, last accessed February 28, 2014) with default parameters, and low quality reads were excluded from further analysis. The remaining reads were mapped to the *G. raimondii* genome using GSNAP (Wu and Nacu 2010), allowing for mapping across splice junctions*.* A *Gossypium*-specific single nucleotide polymorphism (SNP) index (Page et al. 2013) was used to reduce biases in the mapping of *G. raimondii* and *G. arboreum* reads to the *G. raimondii* genome. Mapping results were subsequently sorted and indexed with samtools (Li, Handsaker, et al. 2009). RNA-seq coverage of the ∼37,000 published gene annotations (Paterson et al. 2012) was calculated using custom perl scripts that considered only uniquely mapped reads. Read counts were subsequently normalized by reads per kilobase per million (RPKM) and, separately, using upper-quartile (UQ) normalization (Bullard et al. 2010).

### Analysis of Differential Expression between Paralogs

To evaluate expression divergence, we assessed differential expression between paralogous genes, for within and between tissue comparisons, assuming equal expression upon duplication. We note that this simplifying assumption may not be true for all genes, particularly if the ancient WGD events involved wide allopolyploidization. As it is impossible to determine the nature of such an ancient WGDs, we consider differential gene expression to indicate departure from the ancestral state of equal expression, as this should hold true for the majority of genes. Using this logic, we assessed differential gene expression using Student’s *t*-test of the log ratio of RPKM and separately UQ-normalized data. Here we calculate the log ratio of expression between two paralogous genes using:

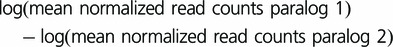



The distribution of log ratios among the paralogs was visually inspected for deviation from normality in both RPKM and UQ data sets. The resulting *P* values were corrected for a false discovery rate (FDR) of 5% using the method of Benjamini and Hochberg (1995). To examine the data for subfunctionalization, we identified tissue-specific reciprocal silencing among differentially expressed paralogs in cases where 1) one of the paralogous gene pair accounted for 95% or more of the total RPKM attributed to both paralogs and 2) this pattern was reversed in one or more tissues/time points.

Although the statistical analysis described above can tell us about differential expression of duplicates between and among tissues, additional insight into expression level divergence may derive from the use of a generalized linear model (GLM). Such a model can estimate gene and tissue effects and their interaction, allowing us to statistically identify patterns of expression consistent with sub and/or neofunctionalization. We therefore fitted a GLM with a negative binomial distribution (implemented in R, using UQ normalized data) to RNA-seq data in petal, seed, and leaf tissue of *G. raimondii*. Our model estimated gene effects, tissue effects, and their interactions, given the equation:

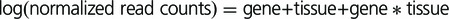



We utilized this GLM to test for gene, tissue, and gene by tissue interaction effects for each of the specific paralog pairs, using the contrasts package in R. A statistically significant gene effect (G effect) indicates that two paralogs differ in mean expression when combined across all three tissues, whereas a significant tissue effect (T effect) indicates that the mean expression of both paralogs together is different between at least two tissues. The effect of these factors combined can be assessed by testing for an interaction between gene and tissue (G×T), which indicates that expression differences between paralogs are not statistically equivalent among tissues. We also performed contrasts to examine differential expression on a per tissue basis, a G | T effect where paralog pairs are differentially expressed within a tissue, irrespective of expression in other tissues. In addition, we identified paralog pairs with complementary expression patterns, again utilizing the contrast analysis. We define complementary expression patterns as cases where paralogs were differentially expressed in both tissues A and B, and additionally, where there is a biased use of one paralog in tissue A and the other paralog in tissue B. Complementary expression patterns are similar, in principle, to tissue-specific reciprocal silencing but do not require actual silencing of one of the paralogs. Resulting *P* values were corrected for FDR of 5% using the method of Benjamini and Hochberg (1995). This use of a GLM is similar to that performed by Duarte et al. (2006) and can reveal patterns of expression level complementation as well as neo- and/or subfunctionalization. We subsequently grouped paralogous gene pairs by patterns of G, T, G×T effects, and assessed differences in mean d*N*/d*S* ratios between groups using a Wilcoxon signed-rank test.

## Results

### Paralogous Gene Identification

We identified groups of paralogous genes in *G. raimondii* originating from the *Gossypium*-specific 5- to 6-fold ploidy increase (fig. 1*A* and *B*; Paterson et al. 2012) via sequence similarity and synteny with genes in *T. cacao* and *V. vinifera* (supplementary file S1, Supplementary Material online). We selected genes surviving as duplicates that trace to this genome multiplication by identifying regions of synteny that were duplicated in *G. raimondii* but corresponded to only a single genomic region in both *T. cacao* and *V. vinifera*. This allowed us to identify 1,971 strictly duplicated paralogous gene pairs (supplementary file S2, Supplementary Material online). We investigated the genomic distribution of retained genes by visual inspection of their distribution along the chromosome scaffolds of *G. raimondii* (fig. 1*C*) using Circos (Krzywinski et al. 2009). Not surprisingly, genes retained as duplicates were broadly distributed without apparent bias with respect to location, apart from the observation that they were most dense in regions having a high overall gene density, subtelomeric regions for example.

### Differential Expression of Paralogous Genes among Tissues and Time Points

**Fig. 2.—:**
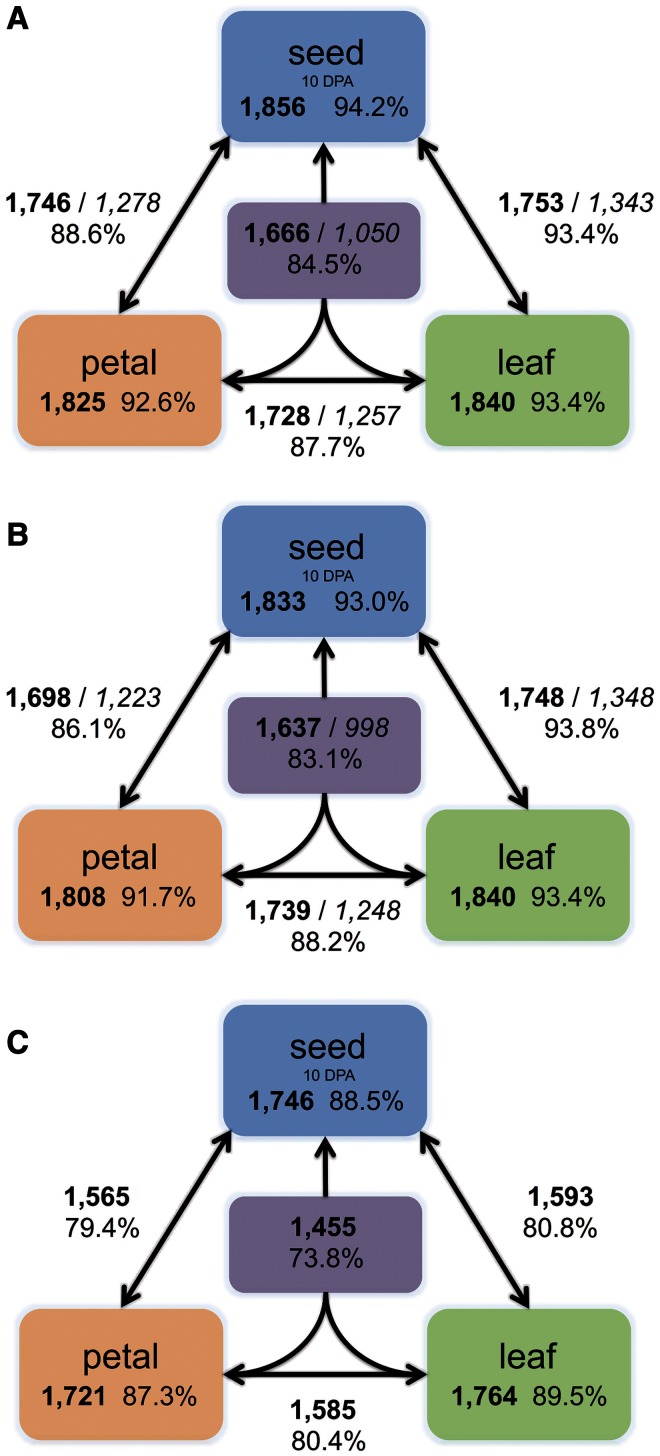
Differential gene expression of ancient paralogous gene pairs in three tissues. (*A*) For *G. raimondii* the number of differentially expressed paralogous gene pairs is indicated in each tissue while the number shared between tissues is indicated next to the lines connecting each pair of tissues. For example 1,825 gene pairs are differentially expressed in petal and 1,746 of these are also differentially expressed in seed (10 DPA). In the middle, connected to all three tissues is the number of pairs showing transcriptional divergence in all three tissues (1,666). The percentage of differentially expressed genes is given and italicized numbers represent the number of gene pairs biased in the same direction in the two tissues connected (e.g., in A 1,278 genes pairs are biased in the same direction in both petal and seed). (*B*) The same diagram as in (*A*) but for expression divergence in *G. arboreum*. (*C*) The number of paralogous gene pairs differentially expressed in both *G. raimondii* and *G. arboreum* in various tissue comparisons.

The 1,971 paralogous gene pairs were subjected to gene expression analysis using RNA-seq data. Reads from both species (*G. raimondii* and *G. arboreum*) and all three tissues (petal, leaf, and seed) were mapped to the *G. raimondii* genome. Gene expression was assessed by evaluating the coverage of uniquely mapped reads over the published gene annotations (Paterson et al. 2012). Statistically significant expression level divergence between paralogs was detected in at least one of the three tissues examined (petal, leaf, and seed) for nearly all pairs (99.4%) in *G. raimondii*, with 93% to 94% of gene pairs exhibiting differential expression on a per tissue basis (fig. 2*A*). Furthermore, 85% of duplicate genes were differentially expressed in all tissues (petal, leaf, and seed), with expression divergence detected for two of the three tissues in 88% to 89% of the paralogs. In *G. arboreum,* the patterns of expression divergence were similar to those observed in *G. raimondii* (fig. 2*B*); nearly all (1,962; 99.5%) paralogs exhibited evidence of transcriptional divergence in at least one tissue, congruent with the observations in *G. raimondii*. Similarly, the range in differential expression, both on a per tissue basis (92–95%) or in at least two of the tissues (86–88%), was also consistent. These analyses were repeated using the UQ normalized data, with nearly identical results (supplementary fig. S1, Supplementary Material online).

We assessed the distribution of fold change between differentially expressed paralogs within tissues of *G. raimondii* (table 1 and supplementary fig. S2, Supplementary Material online). Between 1,379 and 1,462 pairs have a fold change greater than 1.5 on a per tissue basis, with a majority (1,809 out of 1,971) displaying statistically significant and substantial transcriptional divergence in at least one tissue (supplementary fig. S2*D*, Supplementary Material online). Similarly, in *G. arboreum*, between 1,403 and 1,481 pairs exhibit expression fold change greater than 1.5, depending on the tissue (table 1 and supplementary fig. S3, Supplementary Material online), and 1,811 of the 1,971 cases display significant and substantial transcriptional divergence in at least one tissue. Perhaps more biologically meaningful, in all tissues in all species, at least 25% of all paralogs displayed at least a 5-fold difference in expression.

**Table 1:** The Number of the 1,971 Paralogous Gene Pairs That Show Equal or Greater than 1.5-, 2- or 5-Fold Change in Expression Level in Different Tissues

		Petal				Leaf				Seed				Maximum in Any Tissue		
Fold change		1.5	2	5		1.5	2	5		1.5	2	5		1.5	2	5
*G. raimondii*^a^		1,462	1,237	715		1,380	1,076	496		1,379	1,119	551		1,809	1,644	1,026
*G. arboreum*^b^		1,481	1,259	742		1,403	1,087	508		1,409	1,134	568		1,811	1,645	1,027

^a^A histogram of fold change is in supplementary file S3, Supplementary Material online.

^b^A histogram of fold change is in supplementary file S4, Supplementary Material online.

We extended our analysis to investigate possible positional affects. Using a binomial test, we assessed whether duplicates at a given chromosomal region were more likely to be either over- or underexpressed relative to their duplicated counterpart (supplementary fig. S4, Supplementary Material online). After correction for an FDR of 5% we found no significant departure from expectation in leaf, seed, or petal tissue.

We identified paralogs that were differentially expressed in both *G. raimondii* and *G. arboreum* to assess the overlap in the two species (fig. 2*C*). All but two paralog pairs (1,969 of 1,971) were differentially expressed in both *G. raimondii* and *G. arboreum* for at least one tissue. For example, 87% and 90% of pairs were differentially expressed in petal and leaf, respectively, in both species. Moreover, 74% of gene pairs were transcriptionally divergent in all tissues of both species. Importantly, there is a strong linear relationship in expression fold change between paralogs in *G. raimondii* and *G. arboreum* (supplementary fig. S5, Supplementary Material online), suggesting expression divergence likely occurring in the common ancestor of both species.

**Fig. 3.—:**
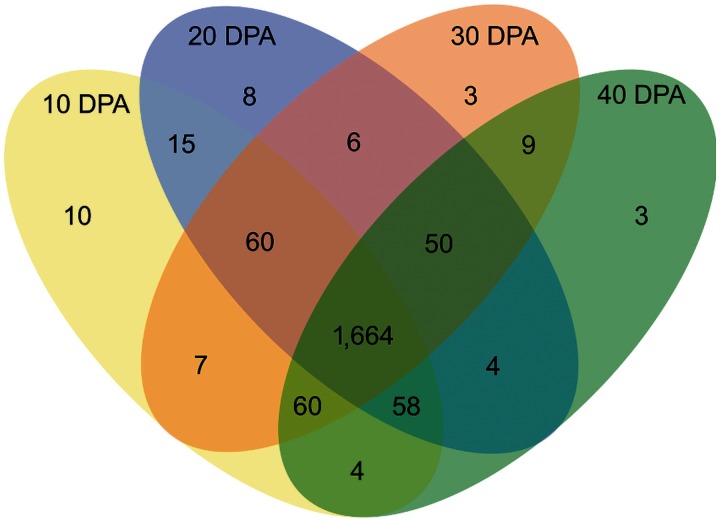
Differential expression of paralogs during seed development in *G. raimondii*. Shown are differentially expressed paralogous gene pairs for stages 10, 20, 30, and 40 days postanthesis and their intersections across stages. A total of 1,971 gene pairs were considered in the analysis.

We extended our analysis to evaluate the effects of development on paralog usage by characterizing differential expression in a developmental time series of *G. raimondii* seed (10–40 DPA). Congruent with the tissue-specific results, most paralogs were differentially expressed in at least one developmental stage (1,961; 99.5%), and the total number of paralogous pairs demonstrating expression divergence was approximately the same at all four time points (between 1,854 and 1,878 pairs; supplementary fig. S6, Supplementary Material online). Most paralog pairs (1,664; 84.4%) displayed transcriptional divergence in all stages of development (fig. 3), indicative of substantial expression level divergence of ancient paralogs throughout seed development.

### Tissue-Specific Reciprocal Silencing

**Fig. 4.—:**
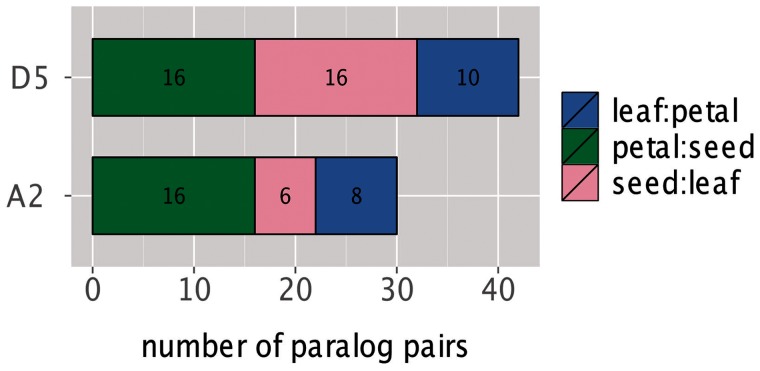
Tissue-specific reciprocal silencing among ancient gene duplicates. Bar plot detailing the number of gene pairs exhibiting tissue-specific reciprocal silencing in three tissue comparisons in both D5 (*G. raimondii*) and A2 (*G. arboreum*). The total number of cases of reciprocal silencing is indicated by bar height, and the number within each tissue comparison is indicated.

Tissue-specific reciprocal silencing is a special case of expression level divergence that can occur immediately after polyploid formation (Adams et al. 2003, 2004) and represents a striking and obvious form of expression level subfunctionalization. We looked for similar patterns of divergence by examining both tissue- and time point-specific reciprocal silencing (i.e., regulatory expression level neo- and/or subfunctionalization) by identifying paralogous gene pairs that show a 95% or greater bias in usage in one tissue/time point, and the opposite pattern of bias in another tissue/time point. Few examples of reciprocal silencing were detected in either *G. raimondii* or *G. arboreum*, ranging from 6 to 16 cases in the three tissue comparisons for each species (fig. 4). Among all comparisons, a maximum of 0.8% of the paralogous pairs were reciprocally silenced, despite most paralogs having substantial expression level divergence. Reciprocal silencing was detected at a slightly higher frequency among time-point comparisons of developing seed of *G. raimondii*. All-way comparisons of the four developmental time points revealed that between 6 and 25 paralog pairs are reciprocally silenced, depending on the time points compared, with 20 versus 30 DPA and 10 versus 40 DPA exhibiting the highest number of reciprocally silenced gene pairs (supplementary fig. S7, Supplementary Material online). Again, a relatively small number (39) of the 1,971 paralog pairs were reciprocally silenced in at least one comparison.

### GLM of Expression Divergence

**Fig. 5.—:**
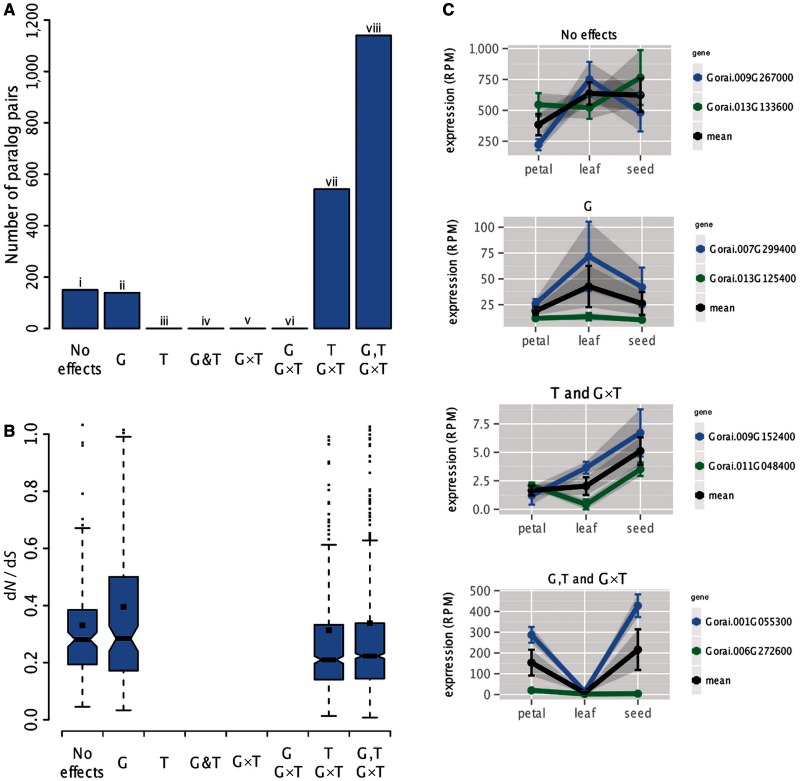
Expression level- and sequence divergence patterns between ancient paralogs in *Gossypium*. (*A*) Paralogous gene pairs categorized according to statistically significant effects following GLM analysis. All possible combinations of G, T, and G×T effects are shown; groups are exclusive, meaning that a given gene pair may only contribute to a single group. (*B*) The same groups as in (*A*) but displaying box plots of d*N*/d*S* ratios between paralogous gene pairs. Horizontal lines and black squares indicate the median and mean of each group, respectively. (*C*) Examples of expression profiles from each category are given.

We used a GLM to examine expression divergence of the 1,971 paralogous gene pairs in petal, seed, and leaf of *G. raimondii*. Two-way analysis of variance (ANOVA) revealed all factors (and their interactions) to be significant (*P* < 0.0005; table 2). For each paralogous gene pair, we examined expression divergence by performing contrasts to detect 1) specific gene effects (G), indicating two paralogs differ in mean expression across all three tissues, 2) tissue effects (T) where the mean expression of both paralogs together is different between at least two tissues, and 3) gene and tissue interactions (G×T) where expression differences between paralogs are not statistically equivalent among tissues, the latter a hallmark of expression level sub- and/or neofunctionalization (Duarte et al. 2006). Most genes exhibited statistically significant G, T, and G×T effects (1,141; 57.9%; fig. 5, column viii), whereas only 150 (7.6%) of the gene pairs exhibited no statistically significant effects (fig. 5*A*, column i). A total of 543 (27.5%) gene pairs exhibited T and G×T effects (fig. 5*A*, column vii). When considering just the G effect, we found that most paralogous genes pairs (1,281; 65.0%) exhibited statistically significant differential expression across all three tissues combined, but this was mostly in conjunction with other measurable effects. Similarly, although 1,684 gene pairs had a statistically significant tissue effect, it was always with other significant effects; we found no cases of T effect alone. Importantly, we did not assign a single gene pair to categories iii–vi.

**Table 2:** Two-Way ANOVA of Gene Expression among 1,971 Ancient Paralogous Gene Pairs in Three Tissues of *G. raimondii*

	df	Sum of Squares	Mean Sq	*F* Value	*P* Value
Gene (G)	3,941	4.82 × 10^10^	12,239,778	23.02	<0.00005
Tissue (T)	2	1.38 × 10^9^	688,414,651	1294.52	<0.00005
Gene:tissue(G×T)	7,882	6.61 × 10^10^	8,384,732	15.77	<0.00005
Residuals	23,652	1.26 × 10^10^	531,792		

### Molecular Divergence of Paralogous Gene Pairs

We examined the possibility of differential selection among paralogs within each of the expression categories by grouping gene pairs based on the outcome of the contrasts analysis described above (i.e., patterns of expression level divergence) and displayed their collective d*N*/d*S* ratios as box plots (fig. 5*B*). Regardless of the pattern of expression differences, all groups had mean and median d*N*/d*S* ratios of less than 0.5 (black squares and black lines, respectively, fig. 5*B*). Using a Wilcoxon signed rank test, we found that mean d*N*/d*S* ratio among pairs with no significant effect (fig. 5*B*, column i) was significantly greater than for two other groups, columns vii (G and G×T; *W* = 46,086, *P* < 0.0005) and viii (G, T, and G×T; *W* = 93,399, *P* < 0.0005). Similarly, d*N*/d*S* ratios for the group with just gene effects (column ii) was statistically greater than the groups in columns vii (T and G×T; *W* = 43,623, *P* < 0.0005) and viii (G, T, and G×T; *W* = 88,798, *P* < 0.0005). No significant differences in d*N*/d*S* ratios were detected for other comparisons.

### Complementary Tissue-Specific Partitioning of Paralogous Gene Expression

**Fig. 6.—:**
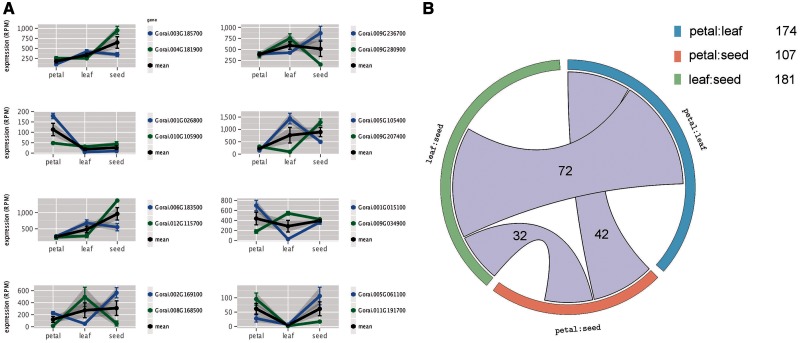
Ancient paralog pairs exhibiting complementary expression profiles. (*A*) Eight representative examples of paralog pairs that exhibit complementary expression level divergence. It is important to note that, in all cases, there is an exchange in paralog bias where one of the gene pair accounts for the majority of combined expression in one tissue, but that this is reversed in another tissue or time point. (*B*) A Circos plot detailing the number of gene pairs with complimentary patterns in different tissue comparisons. The numbers contained within connecting ribbons indicates the number of paralog pairs with complimentary patterns in the connected tissue comparisons. The bar length for each tissue comparisons is scaled relative to the total number of complimentary expression patterns in that tissue.

Using the GLM, we assessed complementary expression patterns between paralogs. Similar to tissue-specific reciprocal silencing described above, we did not require actual silencing of genes but rather reciprocal bias in paralog usage between tissues and differential expression of gene, as estimated by the GLM. Thus, complementary expression is a less stringent form of tissue-specific reciprocal expression, when compared with silencing. This analysis revealed that 314 (15.9%) of the paralogous gene pairs have complementary expression patterns, and the number of gene demonstrating such expression patterns varied between tissue comparisons (fig. 6). For example, there were 75 cases of complementary expression patterns between paralogous genes in leaf and seed, whereas 33 and 58 paralog pairs showed complementary expression patterns in petal and leaf and petal and seed, respectively. Interestingly, we found examples of overlap between complementary expression patterns in different tissue combinations. The greatest overlap was between petal and leaf versus leaf and seed, with 74 paralogous gene pairs showing complementary expression in both of these comparisons.

## Discussion

### Expression Divergence between Paralogs Is the Rule Rather than the Exception

Most genes duplicated by WGD events are subsequently lost during the process of diploidization, although a fraction are retained as duplicates over millions of years (Langham et al. 2004; Thomas et al. 2006; Woodhouse et al. 2010; Freeling et al. 2012). Importantly, the loss or retention of duplicated genes is a nonrandom process (Blanc and Wolfe 2004a; Paterson et al. 2006; Barker et al. 2008, 2009; Buggs et al. 2012; De Smet et al. 2013), reflecting a number of different evolutionary processes. Analyses of recently derived allopolyploids have provided information on homeolog loss shortly following polyploid formation (Langham et al. 2004; Buggs et al. 2012); however, the increasing availability of genome sequences has provided the ability to detect ancient polyploidy events (Paterson et al. 2000; Bowers et al. 2003; Blanc and Wolfe 2004b; Van de Peer et al. 2009; Jiao et al. 2011; Murat et al. 2012) and the attendant opportunity to analyze the properties of homeologs ultimately retained as paralogs. Recent studies have indicated that certain categories of genes are more likely to be retained during diploidization (Blanc and Wolfe 2004a; Paterson et al. 2006; Barker et al. 2008, 2009; Buggs et al. 2012; De Smet et al. 2013), but few studies have examined the role of expression divergence in long-term paralog retention in paleopolyploid plants (Duarte et al. 2006; Throude et al. 2009; Guo et al. 2013; Roulin et al. 2013).

Here we present an analysis of long-term expression divergence of ∼2,000 duplicate genes in the cotton genus, originating from an ancient 5-to-6-fold whole genome multiplication *∼*60 Ma (fig. 1; Paterson et al. 2012). The most striking result is that expression divergence among paralog pairs is nearly complete, in the sense that almost all paralog pairs exhibit expression level divergence on a per tissue and developmental basis; in *G. raimondii*, 99.4% of the paralog pairs are differentially expressed in at least one of the three tissues examined, and 93–94% of gene pairs are differentially expressed on a per tissue basis (fig. 2*A*). Importantly, the extensive expression divergence observed in *G. raimondii* was mirrored in a separate analysis of a second cotton species, *G. arboreum* (fig. 2*B*). This indicates that expression divergence occurred in the period between the ancient polyploidization (*∼*60 Ma) and the divergence of the cotton genome groups 5–10 Ma (Wendel et al. 2009). Given that 1) the signatures of polyploidy typically erode relatively quickly (Mandakova et al. 2010), 2) the rapid expression evolution of some homeologs has been documented in 1–2 My old neoallopolyploids in cotton (Adams et al. 2003, 2004; Flagel et al. 2008; Yoo et al. 2013), and 3) a well-developed theoretical framework substantiating the evolutionary race between mutational loss and neutral or selective retention (Force et al. 1999; Lynch and Conery 2000; Lynch and Force 2000), we suggest that expression divergence likely occurs fairly rapidly and is subsequently maintained over millions of years. Although we cannot be sure that individual expression differences between paralog pairs are functionally meaningful, the weight of numbers and ubiquity in our data set suggest that regulatory divergence in expression is a key process in gene retention following duplication. The results for *Gossypium* are even more impressive when one considers that only several tissues are examined here, from the scores of possibilities, and that for at least 25% of gene pairs in all comparisons in all tissues, transcript abundances for paralogs were more than 5-fold different (table 1). An interesting dimension to this pattern is the observation of complementarity in expression patterns of about one-sixth of all paralog pairs (fig. 6).

Collectively, the data indicate that expression divergence of ancient paralog pairs is the rule rather than the exception, and that this divergence may be evident among developmental stages and/or across tissues (figs. 2 and 3). The observation of only a tiny (<1%) fraction of paralog pairs where expression-level divergence had not occurred (or was not detected) suggests that gene pairs lacking such divergence are generally not maintained as duplicates over the long term. This indicates that the process of expression level divergence is complete or nearly complete on a genome-wide scale. Similarly, in a recent study of ancient duplicates in *Arabidopsis*, changes in interacting gene partners revealed that as many as 97% of paralog pairs showed evidence of functional diversification (encompassing both neo- and subfunctionalization; Guo et al. 2013), mirroring the gene expression data in this study and supporting the notion that regulatory and/or functional diversification are almost universal among ancient gene duplicates. In addition, analysis of d*N*/d*S* ratios in this study and in *Arabidopsis* (Guo et al. 2013) indicates extensive purifying selection on duplicate genes. These results are wholly consistent with theory, indicating that retention of duplicate genes on a long-term basis requires selective maintenance (Force et al. 1999; Lynch and Conery 2000; Lynch and Force 2000; Kafri et al. 2008).

It is important to note that in young synthetic allopolyploids of cotton most homeologs displayed more or less equivalent patterns of expression across several tissue types (Adams et al. 2004), only 5% of genes were silenced or downregulated following allopolyploidy. Similarly in 1–2 Ma allopolyploid *G. hirsutum* (upland cotton), 25% of homeologs displayed varying expression pattern differences (Adams et al. 2003). Our results, where almost all duplicates display divergent expression (fig. 2*A* and *B*), are in stark contrast with those of younger polyploids. The work of Adams et al. (2003, 2004) assessed gene expression changes by RT-PCR, cDNA-single-stranded conformation polymorphism, and AFLP-cDNA display screens, whereas our analysis uses RNA-seq data, perhaps accounting for greater sensitivity and higher rates of expression divergence between duplicates. On the other hand, a greater divergence time (60 Ma compared with 1–2 Ma) may account for such differences, as seen in rice (Li, Zhang, et al. 2009).

### Expression-Level Divergence and Regulatory Neo- and/or Subfunctionalization

Despite the pervasiveness of expression-level differences among paralogs, we found few cases of tissue-specific reciprocal silencing, as seen for a handful of genes in cotton allopolyploids (Adams et al. 2003, 2004). To examine more subtle regulatory level divergence over several tissues, we analyzed expression data for gene pairs via a GLM in petal, leaf, and seed of *G. raimondii* (table 2 and fig. 5*A*). This analysis revealed complex expression level divergence patterns among paralogs, but similar to all other analyses, the GLM revealed that few paralogs have escaped expression partitioning; in total, 92.4% of gene pairs had at least one statistically significant effect (fig. 5*A*), even after correcting *P* values for an FDR of 5%. Furthermore, 85.4% of paralogs showed significant G×T interaction effects, indicating complimentary alterations to expression level in different tissues. Similar to Duarte et al. (2006), we interpret G×T interaction effects as evidence of regulatory level sub- and/or neofunctionalization (Force et al. 1999; Lynch and Conery 2000; Lynch and Force 2000). Interestingly, GLM analysis indicated that more than 300 of the 1,971 gene pairs exhibited a less stringent form of tissue-specific reciprocal silencing (fig. 6). We propose that many of these paralogs represent examples of bona fide regulatory-level sub- and/or neofunctionalization.

The now classical model of Ohno (1970) posits that genes will be retained in duplicate if one of the duplicates is released from evolutionary constraint and, under a neutral mutational model, acquires a new function. Under this relaxed selection regime, one might expect the accumulation of nonsynonymous substitutions to occur at an accelerated rate compared with the duplicate that remains under purifying selection. The results of neofunctionalization under Ohno’s neutral model might be expected to produce d*N*/d*S* ratios greater than one, at least for some paralog pairs. Here, for the vast majority of genes however, d*N*/d*S* ratios were less than 0.5 (fig. 5*B*), indicative of purifying selection. These observations seem to exclude gene retention via a classical neutral model of neofunctionalization for the majority of duplicates in cotton, as also reported for a small sampling of duplicate genes in tetraploid *Xenopus* (Hughes MK and Hughes AL 1993). Interestingly, mean d*N*/d*S* ratios between genes with no measurable effects (fig. 5 category i) and between those pairs with only G effects (fig. 5, category ii) were significantly higher than those with T and G×T effects (fig. 5, category vii) and G, T, and G×T effects (fig. 5, category viii). This suggests a general trend of greater purifying selection among gene pairs exhibiting complex patterns of expression level divergence. Mirroring our results, similar patterns of increased purifying selection in functionally diverged duplicates was also observed in *Arabidopsis* (Guo et al. 2013).

Although the majority of ancient gene duplicates in *Gossypium* appear to have been under a regime of purifying selection and exhibit expression differences consistent with subfunctionalization, with the available data, we cannot distinguish a process of subfunctionalization with that of a more nuanced process of neofunctionalization. For example, new function could be brought about by a single amino acid substitution that, via our analysis of d*N*/d*S* ratios, would be undetectable. Similarly, novel expression of one duplicate in a new tissue or developmental time point might constitute new function, and this may occur independently of molecular divergence in coding regions, making it undetectable by the analysis in figure 5*B*. Thus, we cannot absolutely exclude the process of neofunctionalization, even when d*N*/d*S* ratios are lower than 1. Although the scenarios described above are possible, it seems unlikely to be the case for the majority of gene pairs.

There are also a number of other explanations for gene retention that our data set does not allow us to investigate, but are nonetheless possibilities for many of these duplicates. These include: 1) gene dosage effects (Freeling and Thomas 2006; Birchler and Veitia 2007), where copy number is maintained following duplication as subsequent deletion perturbs the stoichiometric balance of gene networks, 2) genetic buffering (Chapman et al. 2006), where complex, slowly evolving genes are preferentially retained as duplicates in *Arabidopsis* and *Oryza* as a way of buffering mutations and 3) functional redundancy (Gu et al. 2003; Kafri et al. 2008), where hub genes seem to retain complimentary duplicates over long time frames. These processes may all play a role in gene retention over ∼60 Ma and warrant further investigation. In any case, a combination of factors is likely to be at play. For example, it has been proposed that in *Populus* gene duplicates are maintained by a combination of purifying selection in favor of maintaining gene balance and subfunctionalization (Rodgers-Melnick et al. 2012).

Although we and others (Throude et al. 2009) have identified extensive divergence between genes duplicated by ancient whole genome multiplication, several others have noted that various modes of duplication seem to drive different rates of expression diversification (Wang et al. 2012). For example, single small-scale duplications typically result in greater expression-level diversification relative to duplications via WGD in *Arabidopsis* (Casneuf et al. 2006). In rice, Li, Zhang, et al. (2009) observed that genes duplicated in tandem or maintained in long syntenic blocks after duplication were more correlated in their expression compared with those maintained as dispersed duplicates. Similarly, in a study of six varying duplication modes by Wang et al. (2011), it was observed that genes duplicated by whole genome multiplication and tandem duplication exhibited more conserved expression when compared with all other modes of duplication. Considering that WGD seems to result in slower rates of expression-level divergence, it is perhaps surprising to see that many paralog pairs examined in this study exhibit quite different patterns of expression. 

## Conclusions 

Long-term retention of duplicate genes following WGD is a complex process likely involving the operation and interaction of diverse mechanisms and a panoply of evolutionary forces and thus is difficult to comprehensively describe. Here we detailed an analysis of expression level changes subsequent to ancient polyploidization to elucidate the role of expression divergence in gene retention. We show 1) retention of duplicates over 60 My; 2) nearly complete expression divergence of duplicates; and 3) statistical inference of complimentary expression patterns consistent with regulatory expression level neo- and/or subfunctionalization. Thus, our data demonstration that genes retained in duplicate have experienced near universal and often substantial expression divergence. Although we note the limitations of our analysis, particularly in distinguishing neo- and subfunctionalization, the data are congruent with theory and are supported by evidence from other systems (Duarte et al. 2006; Throude et al. 2009; Guo et al. 2013; Roulin et al. 2013), including more recent polyploids (Adams et al. 2004; Buggs et al. 2010). We are also aware that our analysis is informative only at the level of transcription, and that there are multiple steps between this window into the evolutionary process and demonstrations of sub- and neofunctionalization at the protein and metabolic levels. Future work involving multiple approaches, including manipulative experiments involving individual paralogs and functional assays, are required to further elucidate the patterns and processes leading to duplicate gene retention.

## Supplementary Material

Supplementary files S1 and S2 and figures S1–S7 are available at *Genome Biology and Evolution* online (http://www.gbe.oxfordjournals.org/).

Supplementary Data
