# Supplementary material for: Ancient Gene Duplicates in Gossypium (Cotton) Exhibit Near-Complete Expression Divergence
Source: Genome Biol Evol. 2014 Feb 19;6(3):559–71. doi: 10.1093/gbe/evu037 (PMC3971588; doi:10.1093/gbe/evu037)
Supplement: Supplementary Data [file supp_6_3_559__index.html]

Ancient gene duplicates in Gossypium (cotton) exhibit near-complete expression divergence — Ancient Gene Duplicates in Gossypium (Cotton) Exhibit Near-Complete Expression Divergence — Supplementary Data 

# Ancient Gene Duplicates in *Gossypium* (Cotton) Exhibit Near-Complete Expression Divergence

## Supplementary Data

files

**Files in this Data Supplement:**

- Supplementary Data - pdf file
- Supplementary Data - jpg file
- Supplementary Data - jpg file
- Supplementary Data - jpg file
- Supplementary Data - tiff file
- Supplementary Data - tiff file
- Supplementary Data - jpg file
- Supplementary Data - jpg file
- Supplementary Data - txt file
