# Supplementary figures and images for: Ancient Gene Duplicates in Gossypium (Cotton) Exhibit Near-Complete Expression Divergence
Source: Genome Biol Evol. 2014 Feb 19;6(3):559–71. doi: 10.1093/gbe/evu037 (PMC3971588; doi:10.1093/gbe/evu037)

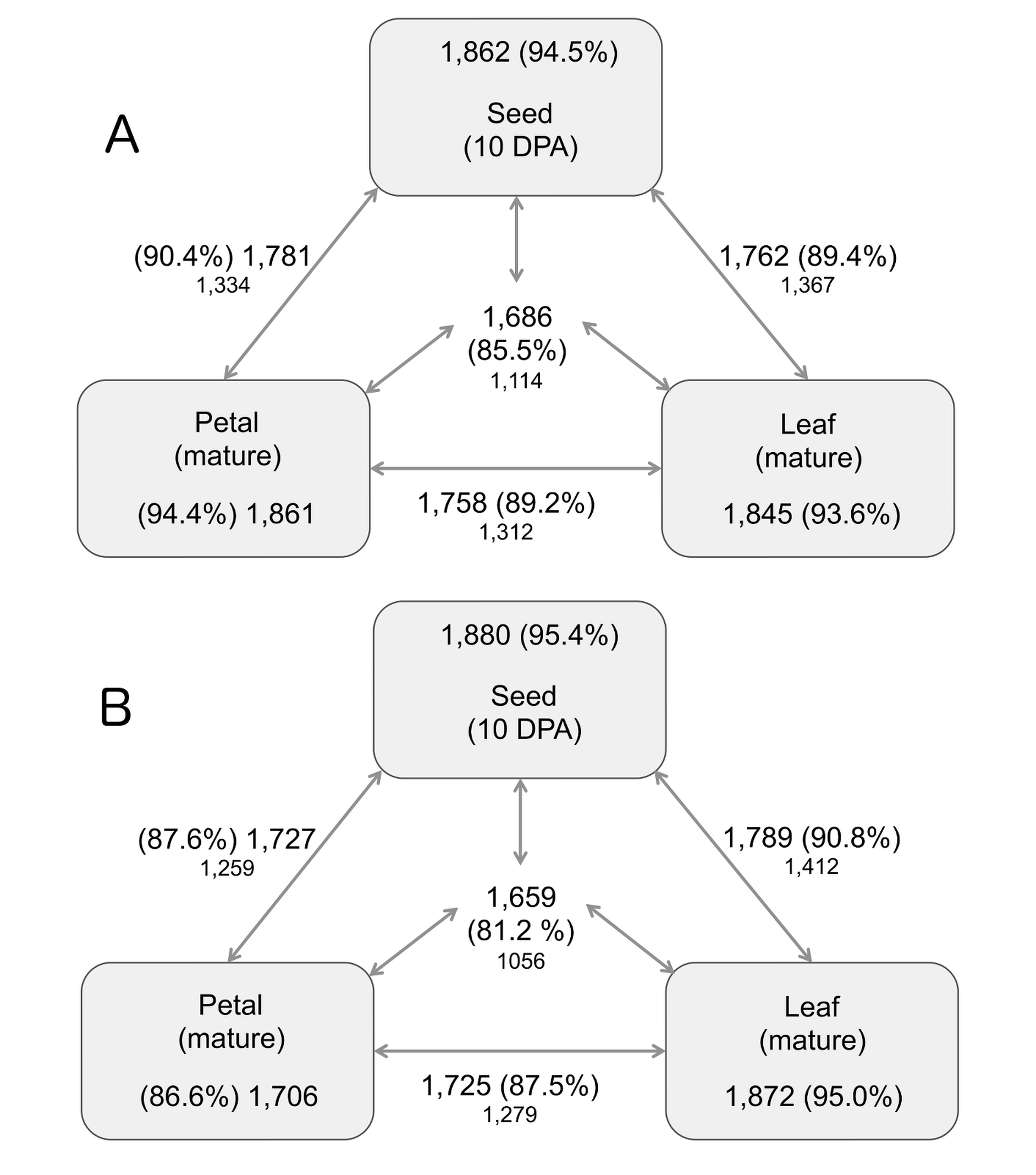

Supplement: Supplementary Data [file supp_evu037_Supplementary_Fig_1.jpg]

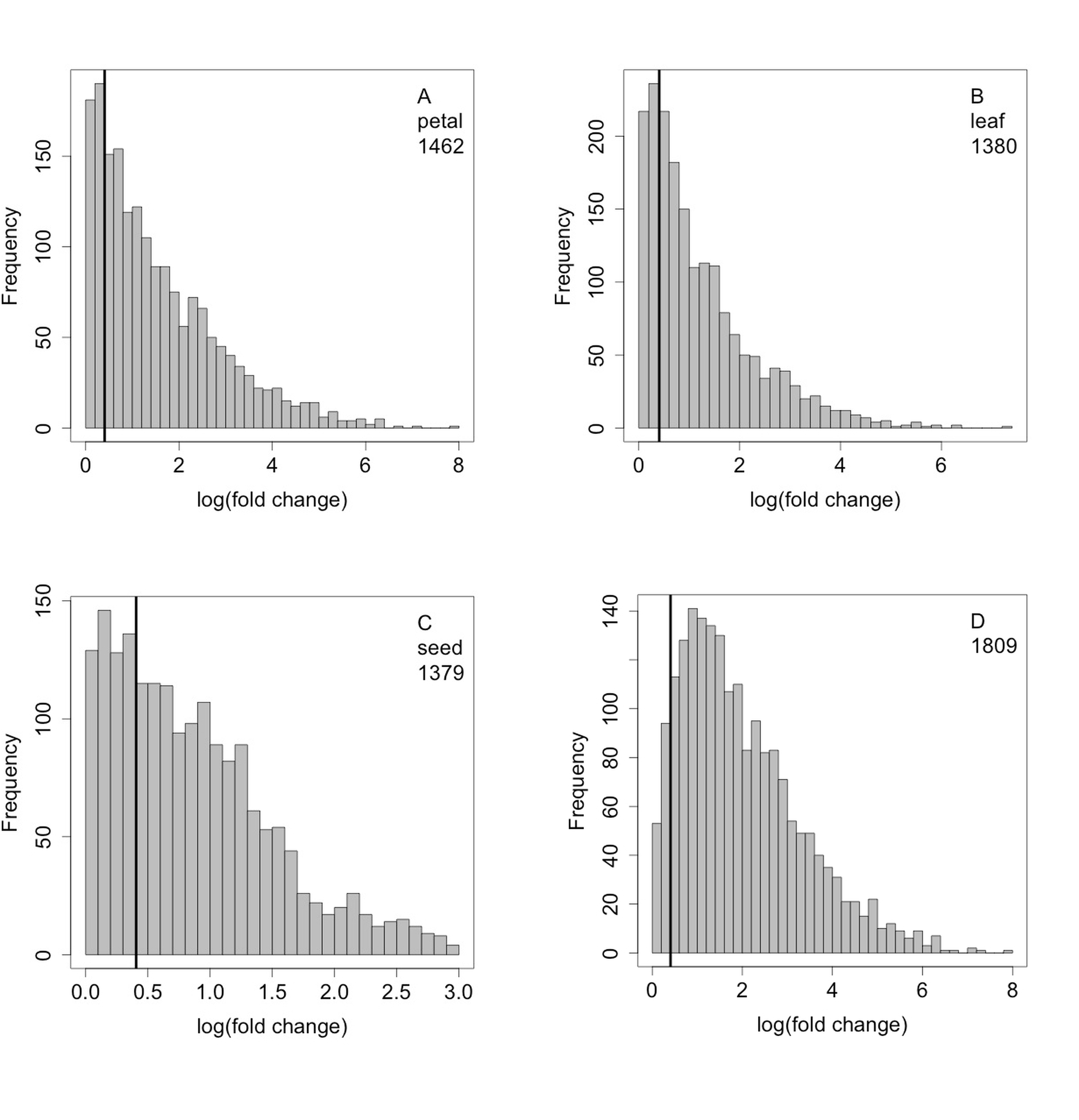

Supplement: Supplementary Data [file supp_evu037_Supplementary_Fig_2.jpg]

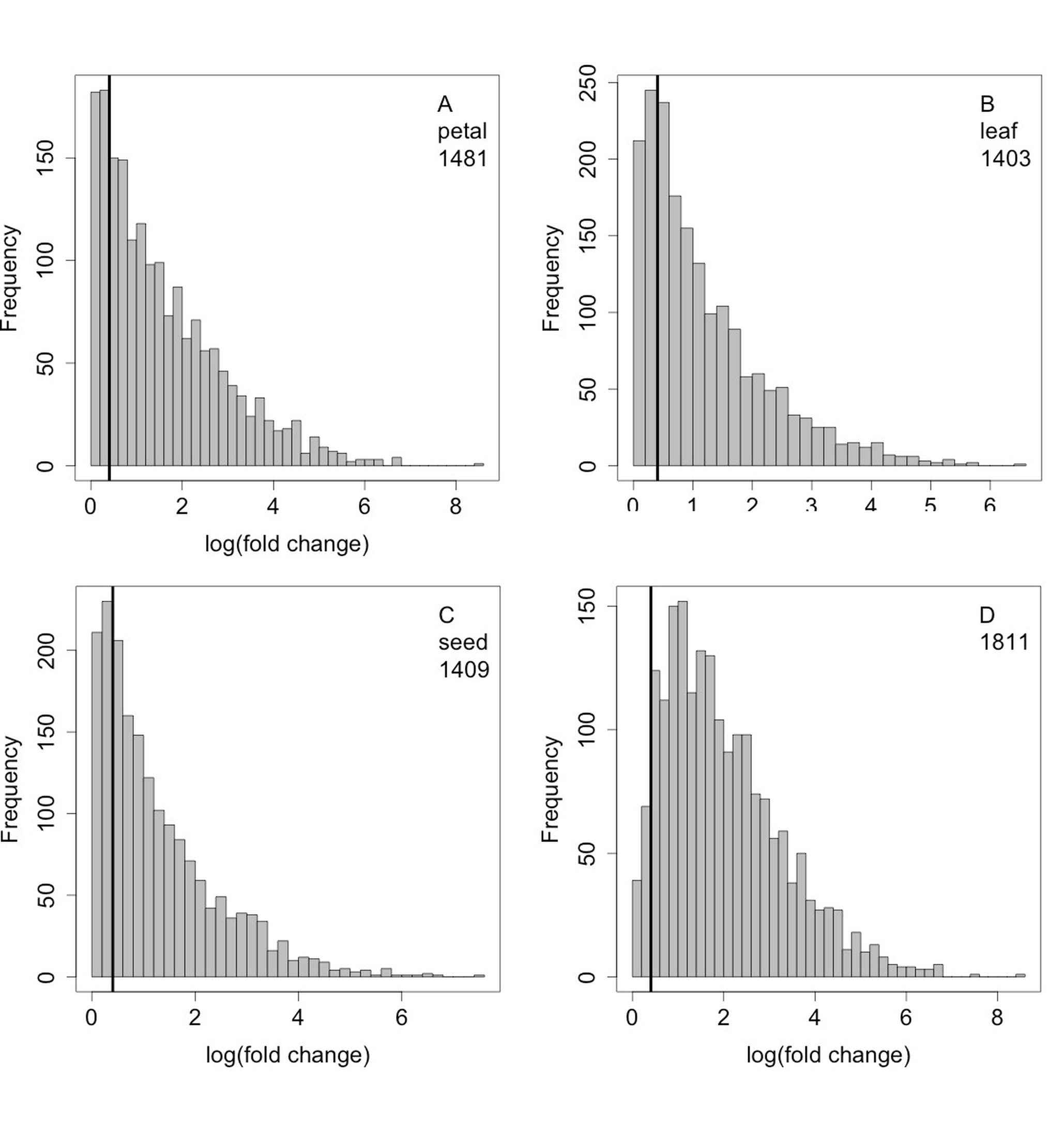

Supplement: Supplementary Data [file supp_evu037_Supplementary_Fig_3.jpg]

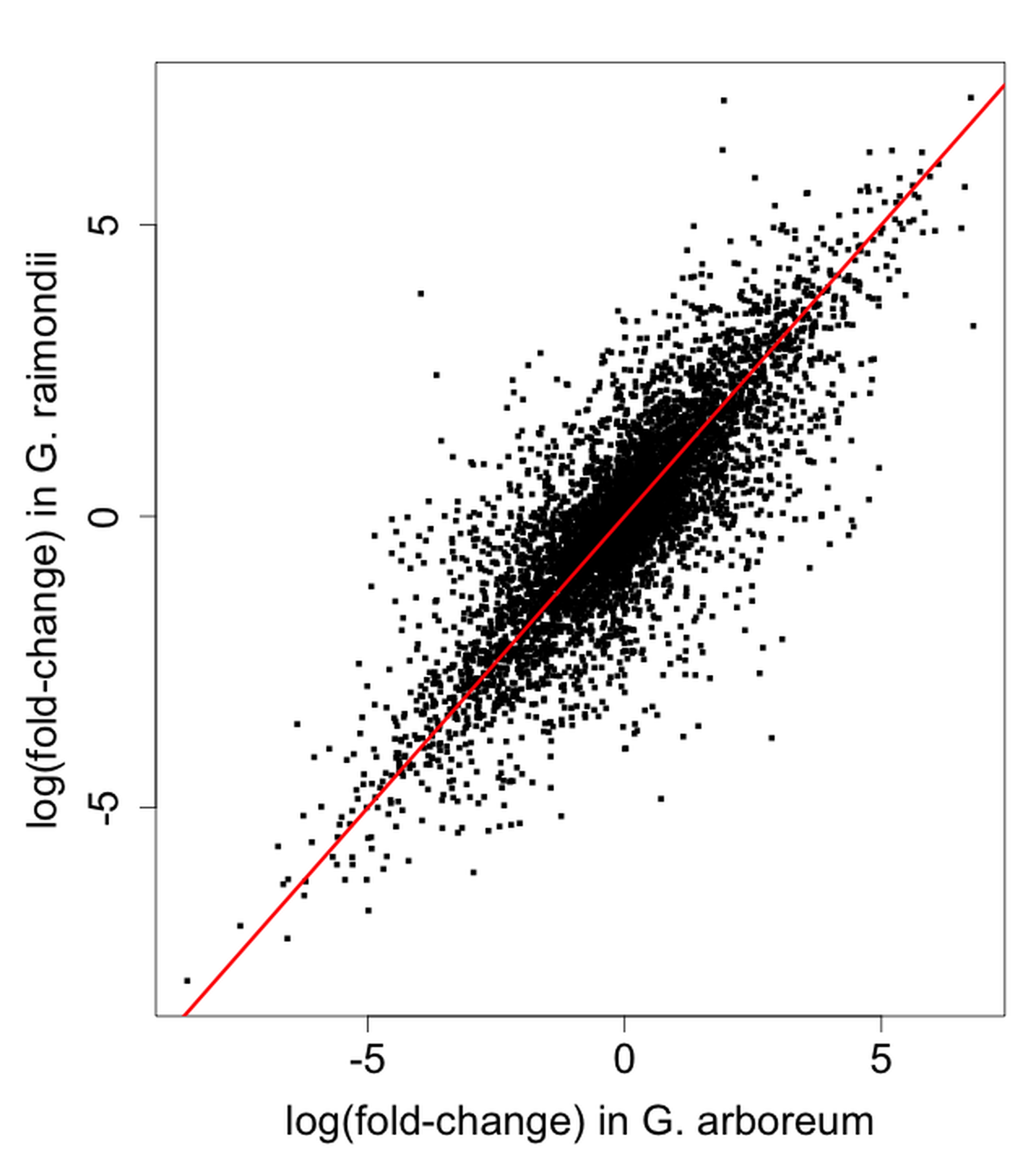

Supplement: Supplementary Data [file supp_evu037_Supplementary_Fig_5.tif]

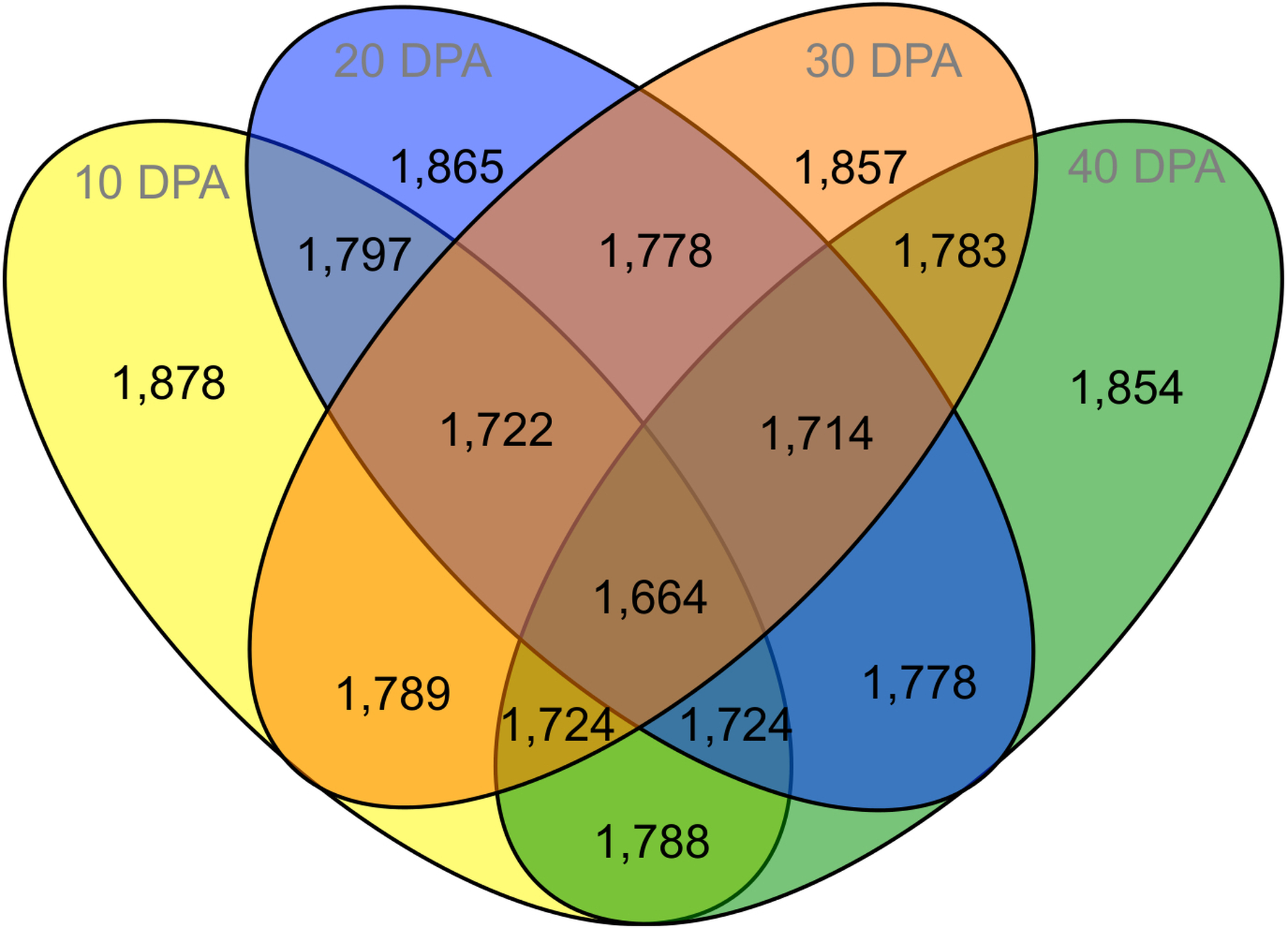

Supplement: Supplementary Data [file supp_evu037_Supplementary_Fig_6.jpg]

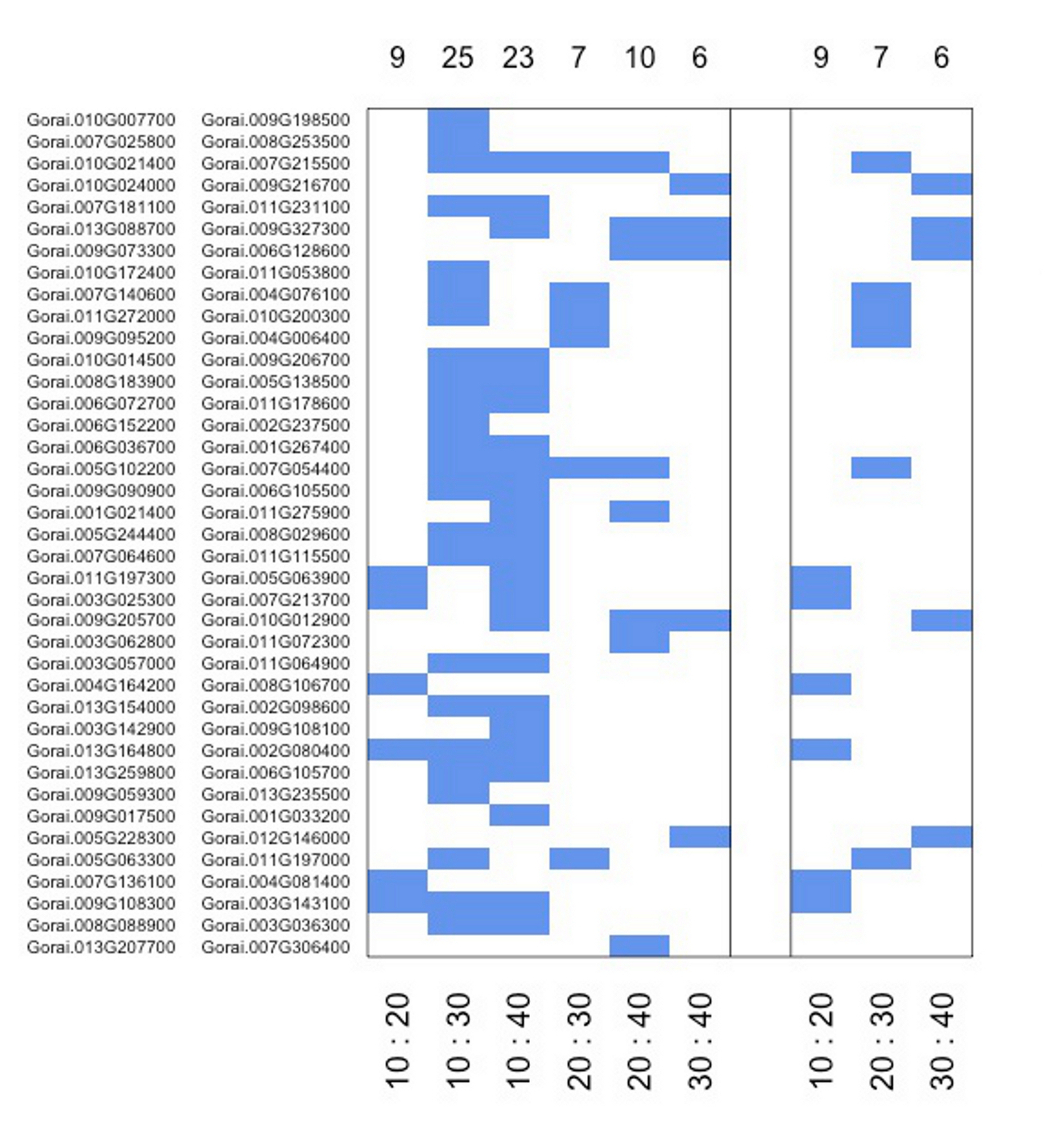

Supplement: Supplementary Data [file supp_evu037_Supplementary_Fig_7.jpg]
