# Supplementary material for: Ancient Gene Duplicates in Gossypium (Cotton) Exhibit Near-Complete Expression Divergence
Source: Genome Biol Evol. 2014 Feb 19;6(3):559–71. doi: 10.1093/gbe/evu037 (PMC3971588; doi:10.1093/gbe/evu037)
Supplement: Supplementary Data [file supp_evu037_Supplementary_File_1.pdf]

```
V1       T1      G11       G12     G13       G14     G15       V2      T2
         G21     G22       G23     G24       G25     V3        T3      G31
         G32     G33       G34     G35
Vv01g0001        .         .       .         .       .         .
Vv14g0896        .         .       .         .       .         .       .
         .       .         .       .         .       .
Vv01g0002        .         .       .         .       .         .       .
         .       .         .       .         .       .         .       .
         .       .         .       .         .
Vv01g0003        .         .       .         .       .         .       .
         .       .         .       .         .       .         .       .
         .       .         .       .         .
Vv01g0004        .         .       Gorai.005G147400.1          .
Gorai.008G175500.1         .       .         .       .         .       .
         .       .         .       .         .       .         .       .
         .
Vv01g0005        Tc02_g031480      .         .       Gorai.009G327100.1
         .       .         .       .         .       .         .       .
         .       .         .       .         .       .         .       .
Vv01g0006        .         .       .         .       .         .       .
         .       .         .       .         .       .         .       .
         .       .         .       .         .
Vv01g0007        Tc02_g031640      .         .       .         .       .
         .       .         .       .         .       .         .       .
         .       .         .       .         .       .
Vv01g0008        Tc02_g031650      Gorai.013G096300.1          .
Gorai.009G327000.1         .       .         .       .         .       .
         .       .         .       .         .       .         .       .
         .       .
Vv01g0009        Tc02_g031660      Gorai.013G096200.1          .       .
         .       .         .       .         .       .         .       .
         .       .         .       .         .       .         .       .
Vv01g0010        .         .       .         .       .         .       .
         .       .         .       .         .       .         .       .
         .       .         .       .         .
Vv01g0011        .         .       .         .       .         .       .
         .       .         .       .         .       .         .       .
         .       .         .       .         .
Vv01g0012        .         .       .         .       .         .       .
         .       .         .       .         .       .         .       .
         .       .         .       .         .
Vv01g0013        .         .       .         .       .         .       .
         .       .         .       .         .       .         .       .
         .       .         .       .         .
Vv01g0014        Tc02_g031670      Gorai.013G096100.1          Gorai.
005G147200.1     .         .       .         Vv03g0885
Tc02_g002300     Gorai.002G213500.1          .       .         .       .
         Vv14g0934         Tc05_g031790      .       .         .       .
         .
Vv01g0015        .         .       .         .       .         .       .
```

. . . . . . . .
. . . . .
Vv01g0016 . . . . . . .
. . . . . . . .
. . . . .
Vv01g0017 Tc02_g031690 Gorai.013G096000.1 . .
. . Vv03g0882 Tc02_g010670 . .
. Gorai.008G017200.1 . Vv14g0936 .
. . . . .
Vv01g0018 Tc02_g031720 Gorai.013G095700.1 Gorai.
005G146800.1 . . . . . . .
. . . . . . . .
. .
Vv01g0019 Tc02_g031740 . Gorai.005G146700.1 .
. . . . . . . .
. . . . . . . .
Vv01g0020 Tc02_g031750 . . . Gorai.
008G175800.1 . Vv03g0857 Tc02_g031750 . .
. . . . . . . .
. .
Vv01g0021 Tc02_g031760 Gorai.013G095500.1 . .
Gorai.008G200200.1 . . . . .
. . . Vv14g0941 . .
Gorai.001G208000.1 Gorai.007G039800.1 . .
Vv01g0022 Tc02_g031770 Gorai.013G095400.1 . .
. . . . . . . .
. . . . . . . .
Vv01g0023 . . . . . . .
. . . . . . . .
. . . . .
Vv01g0024 . . . . . . .
. . . . . . . .
. . . . .
Vv01g0025 . . . . . . .
. . . . . . . .
. . . . .
Vv01g0026 Tc02_g031830 Gorai.013G095300.1 Gorai.
005G146500.1 . . . . . . .
. . . . . . . .
. .
Vv01g0027 Tc02_g031850 . . . Gorai.
008G176100.1 . . . . . . .
. . . . . . . .
Vv01g0028 Tc02_g031860 . . . Gorai.
008G176200.1 . . . . . . .
. . . . . . . .
Vv01g0029 Tc02_g031870 Gorai.013G095200.1 Gorai.
005G146400.1 . . . . . . .
. . . . . . . .
. .

Vv01g0030 . . . . . . .
. . . . . . Vv14g0960
. . Gorai.001G208900.1 . . .
Vv01g0031 Tc02_g031880 Gorai.013G095100.1 . .
. . . . . . . .
. . . . . . . .
Vv01g0032 Tc02_g031890 . Gorai.005G146200.1 .
Gorai.008G176300.1 . . . . .
. . . . . . . .
. .
Vv01g0033 . . . . . . .
. . . . . . . .
. . . . .
Vv01g0034 Tc02_g031900 Gorai.013G095000.1 Gorai.
005G146000.1 . Gorai.008G176400.1 . . .
. . . . . . . .
. . . .
Vv01g0035 . . . . . . .
. . . . . . . .
. . . . .
Vv01g0036 Tc02_g031930 . . . . .
. . . . . . . .
. . . . . .
Vv01g0037 Tc02_g031960 . . . Gorai.
008G176600.1 . . . . . . .
. . . . . . . .
Vv01g0038 Tc02_g031970 Gorai.013G094700.1 . .
Gorai.008G199600.1 . . . . .
. . . . . . . .
. .
Vv01g0039 Tc02_g031980 Gorai.013G094600.1 . .
Gorai.008G176700.1 . . . . .
. . . Vv14g0984 . . .
. Gorai.006G010000.1 .
Vv01g0040 Tc02_g032000 Gorai.013G094500.1 . .
Gorai.008G176800.1 . . . . .
. . . . . . . .
. .
Vv01g0041 Tc02_g032010 Gorai.013G094300.1 .
Gorai.009G326900.1 . . . . . .
. . . . . . . .
. .
Vv01g0042 Tc02_g032020 . . . Gorai.
008G177000.1 . Vv03g0826 Tc02_g032020 Gorai.
013G051100.1 . Gorai.002G010900.1 . . .
. . . . . .
Vv01g0043 . . . . Gorai.008G199200.1
. . . . . . . .
. . . . . . .
Vv01g0044 Tc02_g032040 Gorai.013G094200.1 . .

```
        Gorai.008G177100.1          .       .       .       .       .
        .       .       .       .       .       .       .       .
        .       .
Vv01g0045       Tc02_g032050        .       .       .       .       .
        .       .       .       .       .       .       .       .
        .       .       .       .       .       .
Vv01g0046       Tc02_g032060    Gorai.013G094000.1          .       .
        .       .       .       .       .       .       .       .
        .       .       .       .       .       .       .       .
Vv01g0047       .       .       .       .       .       .       .
        .       .       .       .       .       .       .       .
        .       .       .       .       .
Vv01g0048       .       .       .       .       Gorai.008G177200.1
        .       .       .       .       .       .       .       .
        .       .       .       .       .       .       .
Vv01g0049       Tc02_g032070        .       .       .       .       .
        .       .       .       .       .       .       .       .
        .       .       .       .       .       .
Vv01g0050       .       .       .       .       .       .       .
        .       .       .       .       .       .       .       .
        .       .       .       .       .
Vv01g0051       .       .       .       .       .       .       .
        .       .       .       .       .       .       .       .
        .       .       .       .       .
Vv01g0052       Tc02_g032120    Gorai.013G093800.1          .       .
        .       .       .       .       .       .       .       .
        .       .       .       .       .       .       .       .
Vv01g0053       Tc02_g032150    Gorai.013G093700.1          .       .
        Gorai.008G177400.1          .       .       .       .       .
        .       .       .       .       .       .       .       .
        .       .
Vv01g0054       Tc02_g032160        .       .       .       Gorai.
008G177500.1    .       .       .       .       .       .       .
        .       Vv14g0990       .       .       .       .       .
        .
Vv01g0055       .       .       .       .       .       .       .
        .       .       .       .       .       .       .       .
        .       .       .       .       .       .
Vv01g0056       Tc02_g032210        .       Gorai.005G145100.1      .
        .       .       .       .       .       .       .       .
        .       .       .       .       .       .       .       .
Vv01g0057       .       .       .       Gorai.009G326400.1      .
        .       .       .       .       .       .       .       .
        .       .       .       .       .       .       .
Vv01g0058       Tc02_g032220        .       .       .       .       .
        .       .       .       .       .       .       .       .
        .       .       .       .       .       .
Vv01g0059       Tc02_g032230    Gorai.013G093500.1          .       .
        .       .       .       .       .       .       .       .
        .       .       .       .       .       .       .       .
```

Vv01g0060 . Gorai.013G093400.1 . . .
. . . . . . . .
. . . . . . .
Vv01g0061 . . . . . . .
. . . . . . . .
. . . . .
Vv01g0062 Tc02_g007240 . . . . .
. . . . . . . .
. . . . . .
Vv01g0063 Tc02_g000140 . . . . .
. . . . . . . .
. . . . . .
Vv01g0064 . . . . . . .
. . . . . . . .
. . . . .
Vv01g0065 Tc02_g007550 . . . . .
. . . . . . . .
. . . . . .
Vv01g0066 . . . . . . .
. . . . . . . .
. . . . .
Vv01g0067 . . . . Gorai.008G196700.1
. . . . . . . .
. . . . . . .
Vv01g0068 Tc02_g032310 . . . . .
. . . . . . . .
. . . . . .
Vv01g0069 Tc02_g032320 Gorai.013G092600.1 Gorai.
005G144700.1 . Gorai.008G177900.1 . . .
. . . . . . . .
. . . .
Vv01g0070 Tc02_g032330 Gorai.013G092500.1 Gorai.
005G144600.1 . . . . . . .
. . . . . . . .
. .
Vv01g0071 Tc02_g032340 Gorai.013G092400.1 .
Gorai.009G326300.1 Gorai.008G178000.1 . . .
. . . . . . . .
. . . .
Vv01g0072 Tc02_g032350 Gorai.013G092300.1 Gorai.
005G144500.1 . Gorai.008G178100.1 . . .
. . . . . . . .
. . . .
Vv01g0073 Tc02_g032360 Gorai.013G092200.1 . .
. . . . . . . .
. . . . . . . .
Vv01g0074 . . . . . . .
. . . . . . . .
. . . . .
Vv01g0075 . . . . . . .

. . . . . . . .
. . . . .
Vv01g0076 . . . . . . .
. . . . . . . .
. . . . .
Vv01g0077 . . . . . . .
. . . . . . . .
. . . . .
Vv01g0078 . . Gorai.005G141600.1 . .
. . . . . . . .
. . . . . . .
Vv01g0079 . . . . . . .
. . . . . . . .
. . . . .
Vv01g0080 . . . . . . .
. . . . . . . .
. . . . .
Vv01g0081 . . . . . .
Vv03g0789 Tc02_g010190 . . . .
Gorai.012G147500.1 . . . . . .
.
Vv01g0082 Tc02_g032420 . . . Gorai.
008G196400.1 . . . . . . .
. . . . . . . .
Vv01g0083 . . . . . . .
. . . . . . . .
. . . . .
Vv01g0084 . . . Gorai.009G326200.1 .
. . . . . . . .
. . . . . . .
Vv01g0085 Tc02_g032460 . . . . .
. . . . . . . .
. . . . . .
Vv01g0086 Tc02_g032490 . . Gorai.009G326100.1
. . . . . . . .
. . . . . . . .
Vv01g0087 Tc02_g032500 Gorai.013G091300.1 .
Gorai.009G326000.1 . . . . . .
. . . . . . . .
. .
Vv01g0088 Tc02_g032510 Gorai.013G091200.1 . .
. . . . . . . .
. . . . . . . .
Vv01g0089 Tc02_g032520 . . . . .
. . . . . . . .
. . . . . .
Vv01g0090 Tc02_g032530 . . . . .
. . . . . . . .
. . . . . .
Vv01g0091 Tc02_g032540 Gorai.013G090900.1 .

Gorai.009G325900.1 Gorai.008G193600.1 . . .
. . . . . . . .
. . . .
Vv01g0092 Tc02_g000190 . . . . .
. . . . . . . .
. . . . . .
Vv01g0093 . . . . . . .
. . . . . . . .
. . . . .
Vv01g0094 Tc02_g000200 . . . . .
. . . . . . . .
. . . . . .
Vv01g0095 . . . . . . .
. . . . . . . .
. . . . .
Vv01g0096 . . . . . . .
. . . . . . . .
. . . . .
Vv01g0097 . . . . . . .
. . . . . . . .
. . . . .
Vv01g0098 . . . . . . .
. . . . . . . .
. . . . .
Vv01g0099 . . . . . . .
. . . . . . . .
. . . . .
Vv01g0100 . . . . . . .
. . . . . . . .
. . . . .
Vv01g0101 Tc02_g005790 . . . . .
. . . . . . . .
. . . . . .
Vv01g0102 Tc02_g005760 . . . . .
. . . . . . . .
. . . . . .
Vv01g0103 . . . . . . .
. . . . . . . .
. . . . .
Vv01g0104 Tc02_g000220 Gorai.013G088700.1 . .
. . Vv03g0788 Tc02_g010170 Gorai.
013G050300.1 Gorai.005G231800.1 Gorai.002G010300.1 .
. . . . . . . .
Vv01g0105 Tc02_g007590 . . . . .
. . . . . . . .
. . . . . .
Vv01g0106 Tc02_g032850 . . Gorai.009G323300.1
. . . . . . . .
. . . . . . . .
Vv01g0107 . . . . . . .

```
                 .          .          .          .          .          .          .          .
                 .          .          .          .          .
Vv01g0108            Tc02_g000240          .          .          .          .          .
                 .          .          .          .          .          .          .          .
                 .          .          .          .          .          .
Vv01g0109              .          .          .          .          .          .          .
                 .          .          .          .          .          .          .          .
                 .          .          .          .          .
Vv01g0110              .          .          .          .          .          .          .
                 .          .          .          .          .          .          .          .
                 .          .          .          .          .
Vv01g0111            Tc02_g000250          .          .          .          .          .
                 .          .          .          .          .          .          .          .
                 .          .          .          .          .          .
Vv01g0112            Tc02_g000260     Gorai.002G162700.1          .          .
                 .          .     Vv03g0773        Tc02_g010020          .          .
                 .          .     Gorai.012G148400.1          .          .          .
                 .          .          .          .
Vv01g0113            Tc02_g000270          .          .          .          .          .
                 .          .          .          .          .          .          .          .
                 .          .          .          .          .          .
Vv01g0114              .          .          .          .          .          .          .
                 .          .          .          .          .          .          .          .
                 .          .          .          .          .
Vv01g0115            Tc02_g000310          .          .          .          .          .
                 .          .          .          .          .          .          .          .
                 .          .          .          .          .          .
Vv01g0116            Tc02_g032930          .          .          .       Gorai.
008G179600.1           .          .          .          .          .          .          .
                 .          .          .          .          .          .          .          .
Vv01g0117            Tc02_g032940          .          .          .          .          .
                 .          .          .          .          .          .          .          .
                 .          .          .          .          .          .
Vv01g0118              .          .          .          .          .          .          .
                 .          .          .          .          .          .          .          .
                 .          .          .          .          .
Vv01g0119              .          .          .          .          .          .          .
                 .          .          .          .          .          .          .          .
                 .          .          .          .          .
Vv01g0120            Tc02_g000340          .          .          .          .          .
                 .          .          .          .          .          .          .          .
                 .          .          .          .          .          .
Vv01g0121            Tc02_g000350          .          .          .          .          .
                 .          .          .          .          .          .          .          .
                 .          .          .          .          .          .
Vv01g0122            Tc02_g000360          .          .          .          .          .
                 .          .          .          .          .          .          .          .
                 .          .          .          .          .          .
Vv01g0123              .          .          .          .          .          .          .
                 .          .          .          .          .          .          .          .
```

| | | | | | | | |
|---|---|---|---|---|---|---|---|
| Vv01g0124 | . | . | . | . | . | . | . |
| Vv01g0125 | Tc02_g000370 | | Gorai.002G163400.1 | | | . | . |
| Vv01g0126 | Tc02_g000380 | | . | . | . | . | . |
| Vv01g0127 | Tc02_g000400 | | . | . | . | . | . |
| Vv01g0128 | Tc02_g000440 | | . | . | . | . | . |
| Vv01g0129 | Tc02_g000450 | | . | . | . | . | . |
| Vv01g0130 | . | . | . | . | . | . | . |
| Vv01g0131 | . | . | . | . | . | . | . |
| Vv01g0132 | Tc02_g000460 | | Gorai.002G163800.1 | | | . | . |
| Vv01g0133 | . | . | . | . | . | . | . |
| Vv01g0134 | Tc02_g000490 | | . | . | . | . | . |
| Vv01g0135 | . | . | . | . | . | . | . |
| Vv01g0136 | . | . | . | . | . | . | . |
| Vv01g0137 | Tc02_g000510 | | . | . | . | . | . |
| Vv01g0138 | . | . | . | . | . | . | . |
| Vv01g0139 | Tc02_g000520 | | . | . | . | . | . |
| Vv01g0140 | Tc02_g000530 | | . | . | . | . | . |

```
        .        .       .        .       .        .       .        .
        .        .       .        .       .        .
Vv01g0141        Tc02_g000540     .       .        .       .        .
        .        .       .        .       .        .       .        .
        .        .       .        .       .        .
Vv01g0142        Tc02_g000550     .       .        .       .        .
        .        .       .        .       .        .       .        .
        .        .       .        .       .        .
Vv01g0143        .       Gorai.002G163900.1        .       .        .
        .        .       .        .       .        .       .        .
        .        .       .        .       .        .       .
Vv01g0144        Tc02_g000580     .       .        .       .        .
        .        .       .        .       .        .       .        .
        .        .       .        .       .        .
Vv01g0145        Tc02_g007620     .       .        .       .        .
        .        .       .        .       .        .       .        .
        .        .       .        .       .        .
Vv01g0146        Tc02_g000590     .       .        .       .        .
        .        .       .        .       .        .       .        .
        .        .       .        .       .        .
Vv01g0147        Tc02_g000600     .       .        .       .        .
        .        .       .        .       .        .       .        .
        .        .       .        .       .        .
Vv01g0148        Tc02_g000630     .       .        .       .        .
        .        .       .        .       .        .       .        .
        .        .       .        .       .        .
Vv01g0149        Tc02_g000680     .       .        .       .        .
        .        .       .        .       .        .       .        .
        .        .       .        .       .        .
Vv01g0150        .       .        .       .        .       .        .
        .        .       .        .       .        .       .        .
        .        .       .        .       .
Vv01g0151        Tc02_g033130     .       .        .       .        .
        .        .       .        .       .        .       .        .
        .        .       .        .       .        .
Vv01g0152        .       .        .       .        .       .        .
        .        .       .        .       .        .       .        .
        .        .       .        .       .
Vv01g0153        .       .        .       .        .       .        .
        .        .       .        .       .        .       .        .
        .        .       .        .       .
Vv01g0154        Tc02_g000690     Gorai.002G164000.1       .        .
        .        .       .        .       .        .       .        .
        .        .       .        .       .        .       .        .
Vv01g0155        Tc02_g000700     .       .        .       .        .
        .        .       .        .       .        .       .        .
        .        .       .        .       .        .
Vv01g0156        Tc02_g000710     .       .        .       .        .
        .        .       .        .       .        .       .        .
        .        .       .        .       .        .
```

Vv01g0157 . . . . . . .
. . . . . . . .
. . . . .
Vv01g0158 Tc02_g026550 . . . . .
. . . . . . . .
. . . . . .
Vv01g0159 Tc02_g000730 . . . . .
. . . . . . . .
. . . . . .
Vv01g0160 Tc02_g000740 . . . . .
. . . . . . . .
. . . . . .
Vv01g0161 Tc02_g000750 . . . . .
. . . . . . . .
. . . . . .
Vv01g0162 Tc02_g000760 Gorai.002G164100.1 . .
. . . . . . . .
. . . . . . . .
Vv01g0163 . . . . . . .
. . . . . . . .
. . . . .
Vv01g0164 . . . . . . .
. . . . . . . .
. . . . .
Vv01g0165 . . . . . . .
. . . . . . . .
. . . . .
Vv01g0166 Tc02_g000770 . . . . .
. . . . . . . .
. . . . . .
Vv01g0167 . . . . . . .
. . . . . . . .
. . . . .
Vv01g0168 . . . . . . .
. . . . . . . .
. . . . .
Vv01g0169 . . . . . . .
. . . . . . . .
. . . . .
Vv01g0170 . . . . . . .
. . . . . . . .
. . . . .
Vv01g0171 . . . . . . .
. . . . . . . .
. . . . .
Vv01g0172 . . . . . . .
. . . . . . . .
. . . . .
Vv01g0173 . . . . . . .
. . . . . . . .

. . . . .
Vv01g0174 . . . . . . .
. . . . . . . .
. . . . .
Vv01g0175 . . . . . . .
. . . . . . . .
. . . . .
Vv01g0176 . . . . . . .
. . . . . . . .
. . . . .
Vv01g0177 . . . . . . .
. . . . . . . .
. . . . .
Vv01g0178 . . . . . . .
. . . . . . . .
. . . . .
Vv01g0179 . . . . . . .
. . . . . . . .
. . . . .
Vv01g0180 Tc02_g007670 . . . . .
. . . . . . . .
. . . . . .
Vv01g0181 . . . . . . .
. . . . . . . .
. . . . .
Vv01g0182 . . . . . . .
. . . . . . . .
. . . . .
Vv01g0183 . Gorai.013G069000.1 . . .
. . . . . . . .
. . . . . . .
Vv01g0184 . . . . . . .
. . . . . . . .
. . . . .
Vv01g0185 . . . . . . .
. . . . . . . .
. . . . .
Vv01g0186 . . . . . . .
. . . . . . . .
. . . . .
Vv01g0187 . . . . . . .
. . . . . . . .
. . . . .
Vv01g0188 . . . . . . .
. . . . . . . .
. . . . .
Vv01g0189 . . . . . . .
. . . . . . . .
. . . . .
Vv01g0190 . . . . . . .

. . . . . . . .
. . . . .
Vv01g0191 . . . . . . .
. . . . . . . .
. . . . .
Vv01g0192 Tc02_g028890 . . . Gorai.
008G168700.1 . . . . . . .
. . . . . . . .
Vv01g0193 . . . . . . .
. . . . . . . .
. . . . .
Vv01g0194 . . . . . . .
. . . . . . . .
. . . . .
Vv01g0195 . . . . . . .
. . . . . . . .
. . . . .
Vv01g0196 Tc02_g026760 . . . . .
. . . . . . . .
. . . . . .
Vv01g0197 . . . . . . .
. . . . . . . .
. . . . .
Vv01g0198 . . . . . . .
. . . . . . . .
. . . . .
Vv01g0199 . . . . . . .
. . . . . . . .
. . . . .
Vv01g0200 . . . . . . .
. . . . . . . .
. . . . .
Vv01g0201 . . . . . . .
. . . . . . . .
. . . . .
Vv01g0202 Tc02_g028420 . . . . .
. . . . . . . .
. . . . . .
Vv01g0203 . . . . . . .
. . . . . . . .
. . . . .
Vv01g0204 . . . . . . .
. . . . . . . .
. . . . .
Vv01g0205 . . . . . . .
. . . . . . . .
. . . . .
Vv01g0206 . Gorai.013G070000.1 . . .
. . . . . . . .
. . . . . . .

```
Vv01g0207          .         .         .         .         .         .         .
         .         .         .         .         .         .         .         .
         .         .         .         .         .
Vv01g0208          .         .         .         .         .         .         .
         .         .         .         .         .         .         .         .
         .         .         .         .         .
Vv01g0209          .         Gorai.013G070100.1          .         .
Gorai.008G148400.1           .         .         .         .         .         .
         .         .         .         .         .         .         .         .
         .
Vv01g0210          .         .         .         .         .         .         .
         .         .         .         .         .         .         .         .
         .         .         .         .         .
Vv01g0211          .         .         .         .         .         .         .
         .         .         .         .         .         .         .         .
         .         .         .         .         .
Vv01g0212          .         .         .         .         .         .         .
         .         .         .         .         .         .         .         .
         .         .         .         .         .
Vv01g0213          .         Gorai.013G070300.1          .         .
Gorai.008G148600.1           .         .         .         .         .         .
         .         .         .         .         .         .         .         .
         .
Vv01g0214          .         .         .         .         .         .         .
         .         .         .         .         .         .         .         .
         .         .         .         .         .
Vv01g0215          .         .         .         .         Gorai.008G149000.1
         .         .         .         .         .         .         .         .
         .         .         .         .         .         .         .
Vv01g0216          Tc02_g028310        .         .         .         .         .
         .         .         .         .         .         .         .         .
         .         .         .         .         .         .
Vv01g0217          .         .         .         .         .         .         .
         .         .         .         .         .         .         .         .
         .         .         .         .         .
Vv01g0218          .         .         .         .         Gorai.008G149200.1
         .         .         .         .         .         .         .         .
         .         .         .         .         .         .         .
Vv01g0219          .         Gorai.013G070600.1          .         .         .
         .         .         .         .         .         .         .         .
         .         .         .         .         .         .         .
Vv01g0220          .         .         .         .         .         .         .
         .         .         .         .         .         .         .         .
         .         .         .         .         .
Vv01g0221          .         .         .         .         .         .         .
         .         .         .         .         .         .         .         .
         .         .         .         .         .
Vv01g0222          .         .         .         .         Gorai.008G149600.1
         .         .         .         .         .         .         .         .
         .         .         .         .         .         .         .
```

Vv01g0223 . Gorai.013G070800.1 . . .
. . . . . . . .
. . . . . . .
Vv01g0224 . . . . . . .
. . . . . . . .
. . . . .
Vv01g0225 . . . . . . .
. . . . . . . .
. . . . .
Vv01g0226 . . . . . . .
. . . . . . . .
. . . . .
Vv01g0227 . . . . . . .
. . . . . . . .
. . . . .
Vv01g0228 . . . . Gorai.008G149700.1
. . . . . . . .
. . . . . . .
Vv01g0229 . . . . . . .
. . . . . . . .
. . . . .
Vv01g0230 . . . . . . .
. . . . . . . .
. . . . .
Vv01g0231 . . . . . . .
. . . . . . . .
. . . . .
Vv01g0232 . . . . . . .
. . . . . . . .
. . . . .
Vv01g0233 . . . . . . .
. . . . . . . .
. . . . .
Vv01g0234 . . . . . . .
. . . . . . . .
. . . . .
Vv01g0235 . . . . . . .
. . . . . . . .
. . . . .
Vv01g0236 . . . . . . .
. . . . . . . .
. . . . .
Vv01g0237 . . . . . . .
. . . . . . . .
. . . . .
Vv01g0238 . . . . . . .
. . . . . . . .
. . . . .
Vv01g0239 . . . . . . .
. . . . . . . .

. . . . .
Vv01g0240 . . . . . . .
. . . . . . . .
. . . . .
Vv01g0241 . . . . . . .
. . . . . . . .
. . . . .
Vv01g0242 . . . . . . .
. . . . . . . .
. . . . .
Vv01g0243 . . . . . . .
. . . . . . . .
. . . . .
Vv01g0244 . . . . . . .
. . . . . . . .
. . . . .
Vv01g0245 . . . . . . .
. . . . . . . .
. . . . .
Vv01g0246 . . . . . . .
. . . . . . . .
. . . . .
Vv01g0247 Tc02_g034940 . . . . .
. . . . . . . .
. . . . . .
Vv01g0248 . Gorai.013G078900.1 . . .
. . . . . . . .
. . . . . . .
Vv01g0249 Tc02_g034930 . . . . .
. . . . . . . .
. . . . . .
Vv01g0250 Tc02_g034910 . . . Gorai.
008G188600.1 . . . . . . .
. . . . . . . .
Vv01g0251 Tc02_g034900 . . . Gorai.
008G188500.1 . Vv17g0468 Tc03_g013460 . .
. . . . . . . .
. .
Vv01g0252 Tc02_g034890 . . . . .
. . . . . . . .
. . . . . .
Vv01g0253 . . . . Gorai.008G188400.1
. . . . . . . .
. . . . . . .
Vv01g0254 Tc02_g034870 . . . Gorai.
008G188300.1 . . . . . . .
. . . . . . . .
Vv01g0255 Tc02_g034860 . . . Gorai.
008G188200.1 . . . . . . .
. . . . . . . .

Vv01g0256 Tc02_g034850 Gorai.013G079100.1 . .
. . . . . . . .
. . . . . . . .
Vv01g0257 . . . . . . .
. . . . . . . .
. . . . .
Vv01g0258 . . . . . . .
. . . . . . . .
. . . . .
Vv01g0259 . . . . . . .
. . . . . . . .
. . . . .
Vv01g0260 . . . . . . .
. . . . . . . .
. . . . .
Vv01g0261 . . . . . . .
. . . . . . . .
. . . . .
Vv01g0262 . . . . . . .
. . . . . . . .
. . . . .
Vv01g0263 . . . . . . .
. . . . . . . .
. . . . .
Vv01g0264 . . . . . . .
. . . . . . . .
. . . . .
Vv01g0265 . . . . . . .
. . . . . . . .
. . . . .
Vv01g0266 . . . . . . .
. . . . . . . .
. . . . .
Vv01g0267 . . . . . . .
. . . . . . . .
. . . . .
Vv01g0268 . . . . . . .
. . . . . . . .
. . . . .
Vv01g0269 Tc02_g034810 . . . Gorai.
008G188000.1 . . . . . . .
. . . . . . . .
Vv01g0270 . . . . . . .
. . . . . . . .
. . . . .
Vv01g0271 Tc02_g034800 . . . Gorai.
008G187900.1 . . . . . . .
. . . . . . . .
Vv01g0272 Tc02_g034780 Gorai.013G079300.1 . .
. . . . . . . .

. . . . . . . .
Vv01g0273 . . . . . . .
. . . . . Vv14g0852
Tc04_g015480 . . . . .
Vv01g0274 Tc02_g034770 . . . Gorai.
008G187800.1 . Vv17g0457 Tc03_g013490 . .
Gorai.007G041200.1 Gorai.008G187800.1 . .
. . . . . .
Vv01g0275 . . . . . . .
. . . . . . . .
. . . . .
Vv01g0276 Tc02_g034750 . . . Gorai.
008G187600.1 . . . . . . .
. . . . . . . .
Vv01g0277 Tc02_g034740 . . . Gorai.
008G187500.1 . . . . . . .
. . . . . . . .
Vv01g0278 Tc02_g034730 . Gorai.005G137500.1 .
Gorai.008G187400.1 . . . . .
. . . . . . . .
. .
Vv01g0279 Tc02_g034720 . . . Gorai.
008G187300.1 . . . . . . .
. . . . . . . .
Vv01g0280 Tc02_g034710 . . . Gorai.
008G187200.1 . . . . . . .
. Vv14g0848 Tc04_g015320 . . .
. .
Vv01g0281 Tc02_g034700 . . . Gorai.
008G187100.1 . . . . . . .
. . . . . . . .
Vv01g0282 Tc02_g034690 . . . Gorai.
008G187000.1 . Vv17g0444 . . . .
Gorai.008G187000.1 . . . . .
. . .
Vv01g0283 Tc02_g034680 . Gorai.005G137600.1 .
. . . . . . . .
. . . . . . . .
Vv01g0284 Tc02_g000260 . . . . .
. . . . . . . .
. . . . . .
Vv01g0285 Tc02_g034670 Gorai.013G079400.1 . .
. . . . . . . .
. . . . . . . .
Vv01g0286 Tc02_g034660 . . . Gorai.
008G186900.1 . . . . . . .
. . . . . . . .
Vv01g0287 Tc02_g034650 . . . Gorai.
008G186800.1 . Vv17g0440 . . . .
Gorai.008G186800.1 . Vv14g0842

Tc04_g015290 . . . . .
Vv01g0288 . . . . . . .
. . . . . . . .
. . . . .
Vv01g0289 Tc02_g034640 Gorai.013G079600.1 . .
Gorai.008G186700.1 . . . . .
. . . . . . . .
. .
Vv01g0290 Tc02_g034630 Gorai.013G079900.1 Gorai.
005G137800.1 . . . Vv17g0437 . .
Gorai.006G063200.1 . . . . .
. . . . .
Vv01g0291 Tc02_g034610 . . . Gorai.
008G186500.1 . Vv17g0436 . . . .
Gorai.008G186500.1 . . . . .
. . .
Vv01g0292 . . . . . . .
. . . . . . . .
. . . . .
Vv01g0293 Tc02_g034600 . . . Gorai.
008G186300.1 . . . . . . .
. . . . . . . .
Vv01g0294 Tc02_g034590 . . . Gorai.
008G186200.1 . . . . . . .
. Vv14g0841 Tc04_g015260 . . .
. .
Vv01g0295 Tc02_g034580 . . . . .
. . . . . . . .
. . . . . .
Vv01g0296 Tc02_g000600 . . . . .
. . . . . . . .
. . . . . .
Vv01g0297 . . . . . . .
. . . . . . . .
. . . . .
Vv01g0298 . . . . . . .
. . . . . . . .
. . . . .
Vv01g0299 . . Gorai.005G187600.1 . .
. . . . . . . .
. . . . . . .
Vv01g0300 Tc02_g034570 . Gorai.005G138000.1 .
. . . . . . . .
. . . . . . . .
Vv01g0301 Tc02_g034560 Gorai.013G080000.1 Gorai.
005G138100.1 . Gorai.008G185900.1 . . .
. . . . . . . .
. . . .
Vv01g0302 Tc02_g034540 . . . Gorai.
008G185700.1 . . . . . . .

. . . . . . . .
Vv01g0303 Tc02_g034530 . . . Gorai.
008G185600.1 . . . . . . .
. . . . . . . .
Vv01g0304 . Gorai.013G080100.1 . . .
. . . . . . . .
. . . . . . .
Vv01g0305 Tc02_g034510 Gorai.013G080200.1 . .
Gorai.008G185500.1 . . . . .
. . . . . . . .
. .
Vv01g0306 Tc02_g034500 . . . Gorai.
008G185400.1 . . . . . . .
. . . . . . . .
Vv01g0307 Tc02_g034490 . . . Gorai.
008G185300.1 . . . . . . .
. . . . . . . .
Vv01g0308 . . . . . . .
. . . . . . . .
. . . . .
Vv01g0309 Tc02_g034480 Gorai.013G080300.1 Gorai.
005G138200.1 . . . Vv17g0427 . .
. . . . . . . .
. . .
Vv01g0310 Tc02_g034470 Gorai.013G080400.1 . .
. . . . . . . .
. . . . . . . .
Vv01g0311 Tc02_g034460 Gorai.013G080600.1 . .
. . . . . . . .
. . . . . . . .
Vv01g0312 Tc02_g034450 Gorai.013G080800.1 . .
. . . . . . . .
. . . . . . . .
Vv01g0313 . . . . . . .
. . . . . . . .
. . . . .
Vv01g0314 Tc02_g034440 . . . . .
Vv17g0421 Tc03_g031200 . . . .
. . . . . . . .
Vv01g0315 Tc02_g034430 Gorai.013G081000.1 . .
Gorai.008G185100.1 . . . . .
. . . . . . . .
. .
Vv01g0316 . . . . . . .
. . . . . . . .
. . . . .
Vv01g0317 Tc02_g034420 Gorai.002G219800.1 . .
Gorai.008G185000.1 . . . . .
. . . . . . . .
. .

Vv01g0318 Tc02_g034400 . . . Gorai.
008G184800.1 . Vv17g0417 . . Gorai.
006G062300.1 . Gorai.008G184700.1 Gorai.006G195300.1
. . . . . . .
Vv01g0319 Tc02_g034390 . . . . .
. . . . . . .
Vv14g0817 . . . . . .
Vv01g0320 Tc02_g034380 . . . Gorai.
008G184700.1 . . . . . . .
. . . . . . . .
Vv01g0321 Tc02_g034360 . . . Gorai.
008G184600.1 . . . . . . .
. Vv14g0816 Tc04_g015100 . . .
. .
Vv01g0322 Tc02_g034340 Gorai.013G081300.1 . .
Gorai.008G184500.1 . Vv17g0406 . .
. . Gorai.008G184500.1 . . .
. . . . .
Vv01g0323 Tc02_g034330 . . . Gorai.
008G184400.1 . Vv17g0404 Tc03_g023370 . .
. Gorai.008G184400.1 Gorai.006G195100.1 .
. . . . . .
Vv01g0324 Tc02_g034320 . . . Gorai.
008G184300.1 . . . . . . .
. . . . . . . .
Vv01g0325 Tc02_g034310 . Gorai.005G138300.1 .
. . . . . . . .
. Vv14g0810 . . . Gorai.
007G180900.1 . .
Vv01g0326 Tc02_g034300 Gorai.013G081400.1 . .
. . . . . . . .
. . . . . . . .
Vv01g0327 Tc02_g034290 Gorai.013G081500.1 . .
. . . . . . . .
. . . . . . . .
Vv01g0328 Tc02_g034280 Gorai.013G081600.1 Gorai.
005G138400.1 . . . . . . .
. . . Vv14g0808 Tc04_g014920 .
. . . .
Vv01g0329 . . . . . . .
. . . . . . . .
. . . . .
Vv01g0330 Tc02_g034270 Gorai.013G081700.1 . .
. . . . . . . .
. . . . . . . .
Vv01g0331 Tc02_g034250 Gorai.013G082000.1 . .
Gorai.008G184000.1 . . . . .
. . . . . . . .
. .
Vv01g0332 . . . . . . .

```
         .        .        .        .        .        .        .        .
         .        .        .        .        .
Vv01g0333         Tc02_g034240      Gorai.013G077100.1         .        .
         .        .        .        .        .        .        .        .
         .        .        .        .        .        .        .        .
Vv01g0334         Tc02_g034230      .        Gorai.005G138500.1         .
         Gorai.008G183900.1         .        .        .        .        .
         .        .        .        .        .        .        .        .
         .        .
Vv01g0335         .        .        .        .        .        .        .
         .        .        .        .        .        .        .        .
         .        .        .        .        .
Vv01g0336         Tc02_g034220      .        Gorai.005G138600.1         .
         .        .        .        .        .        .        .        .
         .        .        .        .        .        .        .        .
Vv01g0337         Tc02_g034190      Gorai.013G076800.1         .        .
         .        .        .        .        .        .        .        .
         .        Vv14g0806         Tc04_g014900      .        .        .
         .        .
Vv01g0338         .        .        .        .        .        .        .
         .        .        .        .        .        .        .        .
         .        .        .        .        .
Vv01g0339         .        .        .        .        .        .        .
         .        .        .        .        .        .        .        .
         .        .        .        .        .
Vv01g0340         Tc02_g034150      Gorai.013G076300.1         .        .
         Gorai.008G183500.1         .        .        .        .        .
         .        .        .        .        .        .        .        .
         .        .
Vv01g0341         .        Gorai.013G076100.1         .        .
Gorai.008G183300.1         .        .        .        .        .        .
         .        .        .        .        .        .        .        .
         .
Vv01g0342         .        .        .        .        Gorai.008G180600.1
         .        .        .        .        .        .        .        .
         .        .        .        .        .        .        .
Vv01g0343         .        .        .        .        .        .        .
         .        .        .        .        .        .        .        .
         .        .        .        .        .
Vv01g0344         .        Gorai.002G218500.1         .        .        .
         .        .        .        .        .        .        .        .
         Vv14g0801         .        .        .        .        .        .
Vv01g0345         .        .        Gorai.005G190200.1         .        .
         .        .        .        .        .        .        .        .
         .        .        .        .        .        .        .
Vv01g0346         .        .        .        .        .        .        .
         .        .        .        .        .        .        .        .
         .        .        .        .        .
Vv01g0347         .        Gorai.002G218400.1         .        .        .
         .        Vv17g0394         .        .        .        .        .
```

. . . . . . . .
Vv01g0348 . . . . . . .
. . . . . . . .
. . . . .
Vv01g0349 . . . . . . .
. . . . . . . .
. . . . .
Vv01g0350 . . . . . . .
. . . . . . . .
. . . . .
Vv01g0351 Tc02_g010100 Gorai.002G217800.1 Gorai.
005G190300.1 . . . Vv17g0390 . .
Gorai.006G061200.1 . . . Vv14g0795
Tc04_g014830 . . . . .
Vv01g0352 . Gorai.002G216000.1 Gorai.005G190500.1
. . . . . . . .
. . . . . . . .
.
Vv01g0353 . . Gorai.005G190600.1 . .
. Vv17g0383 . . . . .
. Vv14g0790 Tc04_g014790 . .
Gorai.007G181500.1 . .
Vv01g0354 . . Gorai.005G190700.1 . .
. . . . . . . .
. . . . . . .
Vv01g0355 . . Gorai.005G191500.1 . .
. . . . . . . .
. . . . . . .
Vv01g0356 . . Gorai.005G191700.1 . .
. . . . . . . .
. . . . . . .
Vv01g0357 . . Gorai.005G191800.1 . .
. . . . . . . .
Vv14g0788 Tc04_g014770 . . . .
.
Vv01g0358 Tc02_g009800 . . . . .
. . . . . . . .
. . . . . .
Vv01g0359 . . . Gorai.009G347400.1 .
. . . . . . . .
. . . . . . .
Vv01g0360 . . Gorai.005G192000.1 . .
Gorai.012G122000.1 . . . . .
. . . . . . . .
.
Vv01g0361 . . . . . . .
. . . . . . . .
. . . . .
Vv01g0362 . . . . . . .
. . . . . . . .

. . . . .
Vv01g0363 . . . . . . .
. . . . . . . .
. . . . .
Vv01g0364 . . . . . Gorai.
012G122100.1 . . . . . . .
. . . . . . .
Vv01g0365 . . . . . Gorai.
012G122200.1 . . . . . . .
. . . . . . .
Vv01g0366 . . . . . . .
. . . . . . . .
. . . . .
Vv01g0367 . . . . . Gorai.
012G122300.1 . . . . . . .
. . . . . . .
Vv01g0368 . . . . . . .
. . . . . . . .
. . . . .
Vv01g0369 . . . . . . .
. . . . . . . .
. . . . .
Vv01g0370 . . . Gorai.009G347600.1 .
. . . . . . . .
. . . . . . .
Vv01g0371 . . . . . . .
. . . . . . . .
. . . . .
Vv01g0372 . . . . . . .
. . . . . . Vv14g0779
Tc04_g014670 . . Gorai.007G181800.1 .
.
Vv01g0373 . . . . . . .
. . . . . . . .
. . . . .
Vv01g0374 . . . . . .
Vv17g0355 . Gorai.001G081000.1 . . .
. Vv14g0771 Tc04_g014610 . . .
. .
Vv01g0375 . . . . . . .
. . . . . . . .
. . . . .
Vv01g0376 . . . . . . .
. . . . . . . .
. . . . .
Vv01g0377 Tc02_g009740 . . . . .
. . . . . . .
Vv14g0767 Tc04_g014560 . . . . .
Vv01g0378 Tc02_g011150 . . Gorai.009G347800.1
. Gorai.012G123200.1 . . . .

. . . . . . . .
. .
Vv01g0379 . . . . . . .
. . . . . . . .
. . . . .
Vv01g0380 . . . . . . .
. . . . . . . .
. . . . .
Vv01g0381 . . . . . .
Vv17g0353 . . . . . . .
. . . . . .
Vv01g0382 . . . . . . .
. . . . . . . .
. . . . .
Vv01g0383 . . . . . . .
. . . . . . . .
. . . . .
Vv01g0384 . . Gorai.005G192100.1 Gorai.
009G347900.1 . Gorai.012G124300.1 Vv17g0351 .
Gorai.001G081300.1 . . . .
Vv14g0764 Tc04_g014420 . . . Gorai.
011G228800.1 .
Vv01g0385 . . . . . . .
. . . . . . . .
. . . . .
Vv01g0386 . . . . . . .
. . . . . . . .
. . . . .
Vv01g0387 . . . . . Gorai.
012G124200.1 Vv17g0350 . Gorai.001G081500.1 .
. . . . . . . .
. .
Vv01g0388 . . . . . . .
. . . . . . . .
. . . . .
Vv01g0389 . . . . . . .
. . . . . . Vv14g0759
Tc04_g014350 . . . . .
Vv01g0390 . . . . . . .
. . . . . . . .
. . . . .
Vv01g0391 . . . . . . .
. . . . . . . .
. . . . .
Vv01g0392 . . . . . . .
. . . . . . . .
. . . . .
Vv01g0393 Tc02_g010900 . . . . .
. . . . . . . .
. . . . . .

Vv01g0394 . . Gorai.005G192600.1 . .
. . . . . . . .
. . . . . . .
Vv01g0395 . . . . . . .
. . . . . . . .
. . . . .
Vv01g0396 . . . . . . .
. . . . . . . .
. . . . .
Vv01g0397 . . . . . . .
. . . . . . . .
. . . . .
Vv01g0398 . . . . . . .
. . . . . . . .
. . . . .
Vv01g0399 Tc02_g024240 . . . . .
. . . . . . . .
. . . . . .
Vv01g0400 . . . . . . .
. . . . . . . .
. . . . .
Vv01g0401 . . . . . . .
. . . . . . . .
. . . . .
Vv01g0402 . . . . . . .
. . . . . . . .
. . . . .
Vv01g0403 . . . . . . .
. . . . . . . .
. . . . .
Vv01g0404 . . . . . . .
. . . . . . . .
. . . . .
Vv01g0405 . . Gorai.005G193200.1 . .
. . . . . . . .
. . . . . . .
Vv01g0406 . . . . . .
Vv17g0337 . . . . . . .
. . . . . .
Vv01g0407 . . . . . . .
. . . . . . Vv14g0746
Tc04_g013970 . . . . .
Vv01g0408 . . Gorai.005G193300.1 . .
. . . . . . . .
. . . . . . .
Vv01g0409 Tc02_g009450 . . . . .
Vv17g0333 . Gorai.001G075400.1 Gorai.
006G059000.1 . . . . . . .
. . .
Vv01g0410 . . Gorai.005G193400.1 . .

. . . . . . . .
. . . . . . .
Vv01g0411 . . . . . . .
. . . . . . . .
. . . . .
Vv01g0412 . . . . . . .
. . . . . . . .
. . . . .
Vv01g0413 . . . Gorai.009G348100.1 .
. . . . . . . .
. . . . . . .
Vv01g0414 . . . . . . .
. . . . . . . .
. . . . .
Vv01g0415 . . . Gorai.009G348200.1 .
Gorai.012G124000.1 Vv17g0325 . . .
. . . . . . . .
. .
Vv01g0416 . . . . . . .
. . . . . . . .
. . . . .
Vv01g0417 . . . . . . .
. . . . . . . .
. . . . .
Vv01g0418 . . . Gorai.009G348900.1 .
. Vv17g0323 . Gorai.001G068000.1 .
. . . . . . . .
. .
Vv01g0419 . . . . . . .
. . . . . . . .
. . . . .
Vv01g0420 . . . . . . .
. . . . . . . .
. . . . .
Vv01g0421 . . . . . . .
. . . . . . Vv14g0736
Tc04_g013830 . . . . .
Vv01g0422 . . . . . .
Vv17g0322 . Gorai.001G084000.1 . . .
. . . . . . . .
Vv01g0423 . . . . . . .
. . . . . . . .
. . . . .
Vv01g0424 . . . . . Gorai.
012G123900.1 . . . . . . .
. . . . . . .
Vv01g0425 . . . . . . .
. . . . . . . .
. . . . .
Vv01g0426 . . . . . . .

. . . . . . . .
. . . . .
Vv01g0427 . . Gorai.005G193700.1 . .
. . . . . . . .
Vv14g0732 . . . . . .
Vv01g0428 . . . . . . .
. . . . . . . .
. . . . .
Vv01g0429 . . . . . . .
. . . . . . . .
. . . . .
Vv01g0430 Tc02_g024250 . . . . .
. . . . . . . .
. . . . . .
Vv01g0431 . . . . . . .
. . . . . . . .
. . . . .
Vv01g0432 Tc02_g024420 . . Gorai.009G343100.1
. . . . . . . .
. . . . . . . .
Vv01g0433 . . . . . .
Vv17g0318 . . . . . Gorai.
006G193600.1 . . . . . . .
Vv01g0434 . . . . . .
Vv17g0317 . . . . . . .
. . . . . .
Vv01g0435 Tc02_g017750 . . Gorai.009G342900.1
. . . . . . . .
. . . . . . . .
Vv01g0436 Tc02_g017610 . . . . .
Vv17g0308 . . . . . .
Vv08g0040 . . . . . .
Vv01g0437 Tc02_g017590 . . . . .
Vv17g0307 . . . . . .
. . . . . . .
Vv01g0438 Tc02_g017580 . Gorai.005G194200.1 .
. . . . . . . .
. . . . . . . .
Vv01g0439 Tc02_g027740 . . . Gorai.
008G168700.1 . . . . . . .
. . . . . . . .
Vv01g0440 . . . . . . .
. . . . . . . .
. . . . .
Vv01g0441 . . . . . . .
. . . . . . . .
. . . . .
Vv01g0442 . . . Gorai.009G342800.1 .
. . . . . . . .
. . . . . . .

Vv01g0443 Tc02_g024580 . Gorai.005G179100.1 .
. . Vv17g0305 Tc03_g004720 . .
. . . Vv08g0076 . . .
. . .
Vv01g0444 . . . . . . .
. . . . . . . .
. . . . .
Vv01g0445 Tc02_g024630 . Gorai.005G178900.1 .
. . Vv17g0303 Tc03_g004730 . .
. . . Vv08g0084 Tc05_g023190 .
. . . .
Vv01g0446 . . . . . .
Vv17g0300 Tc03_g004760 . . . . .
Vv08g0088 Tc05_g023210 . . . .
.
Vv01g0447 . . . . . . .
. . . . . . Vv08g0096
. . . . . .
Vv01g0448 . . . . . . .
. . . . . . . .
. . . . .
Vv01g0449 . . . . . Gorai.
012G123700.1 Vv17g0292 . . . . .
. . . . . . . .
Vv01g0450 . . . Gorai.009G341900.1 .
. . . . . . . .
Vv08g0110 Tc05_g023460 . . . .
.
Vv01g0451 . . . . . . .
. . . . . . . .
. . . . .
Vv01g0452 . . . . . . .
. . . . . . . .
. . . . .
Vv01g0453 . . . . . . .
. . . . . . . .
. . . . .
Vv01g0454 . . . . . Gorai.
012G123600.1 . . . . . . .
. . . . . . .
Vv01g0455 Tc02_g025080 . Gorai.005G177000.1 .
. . . . . . . .
. . . . . . . .
Vv01g0456 . . . Gorai.009G341800.1 .
. . . . . . . .
. . . . . . .
Vv01g0457 . . . . . .
Vv014rg0081 . . . . . . .
. . . . . .
Vv01g0458 . Gorai.002G181200.1 . . .

. Vv014rg0093 . . . . .
. . . . . . . .
Vv01g0459 . . Gorai.005G163600.1 Gorai.
009G338800.1 . . . . . . .
. . . . . . . .
.
Vv01g0460 . . . . . . .
. . . . . . . .
. . . . .
Vv01g0461 . . Gorai.005G163400.1 .
Gorai.008G166100.1 . . . . . .
. . . . . . . .
.
Vv01g0462 . Gorai.002G181100.1 Gorai.005G163100.1
. Gorai.008G166200.1 . Vv014rg0099 .
. . . . . . . .
. . . .
Vv01g0463 . Gorai.002G181000.1 Gorai.005G163000.1
. . Gorai.012G120900.1 Vv014rg0104 .
. . . . . . . .
. . . .
Vv01g0464 . . . . . . .
. . . . . . . .
. . . . .
Vv01g0465 . Gorai.002G180500.1 Gorai.005G162900.1
. . . . . . . .
. . Vv08g0143 Tc05_g004750 . .
. . .
Vv01g0466 . . . . . . .
. . . . . . . .
. . . . .
Vv01g0467 Tc02_g027610 . . . Gorai.
008G166300.1 . Vv014rg0119 . . . .
. . . . . . . .
.
Vv01g0468 . . . . . .
Vv014rg0121 . . . . . . .
. . . . . .
Vv01g0469 . . . . . . .
. . . . . . . .
. . . . .
Vv01g0470 . . . . . . .
. . . . . . . .
. . . . .
Vv01g0471 . . . . . . .
. . . . . . . .
. . . . .
Vv01g0472 . . Gorai.005G162700.1 . .
. . . . . . . .
. . . . . . .

Vv01g0473 . . . . Gorai.008G166400.1
. . . . . . . .
. . . . . . .
Vv01g0474 Tc02_g027590 . . . . .
. . . . . . . .
. . . . . .
Vv01g0475 Tc02_g027570 . . . . .
. . . . . . . .
. . . . . .
Vv01g0476 . . . . . . .
. . . . . . . .
. . . . .
Vv01g0477 . . . . . . .
. . . . . . . .
. . . . .
Vv01g0478 Tc02_g027560 Gorai.002G180300.1 . .
. . . . . . . .
. . . . . . . .
Vv01g0479 Tc02_g027540 Gorai.002G180200.1 . .
. . . . . . . .
. . . . . . . .
Vv01g0480 Tc02_g027530 Gorai.002G180100.1 Gorai.
005G162500.1 . Gorai.008G166500.1 . Vv014rg0144
. . . . . . . .
. . . . .
Vv01g0481 Tc02_g027520 Gorai.002G180000.1 Gorai.
005G162400.1 . . . . . . .
. . . . . . . .
. .
Vv01g0482 . . . . . . .
. . . . . . . .
. . . . .
Vv01g0483 Tc02_g027510 Gorai.002G179900.1 . .
. . . . . . . .
. . . . . . . .
Vv01g0484 . . . . . .
Vv014rg0147 . . . . . . .
. . . . . .
Vv01g0485 Tc02_g027390 Gorai.002G179700.1 . .
. . . . . . . .
. . . . . . . .
Vv01g0486 . . . . . .
Vv014rg0183 . . . . . .
Vv12g0901 . . . . . .
Vv01g0487 Tc02_g027380 . Gorai.005G162300.1 .
. . . . . . . .
. . . . . . . .
Vv01g0488 Tc02_g027370 Gorai.002G179300.1 . .
. . . . . . . .
. . . . . . . .

Vv01g0489 Tc02_g027350 . Gorai.005G162000.1 .
Gorai.008G166700.1 . . . . .
. . . . . . . .
. .
Vv01g0490 . . . . . . .
. . . . . . . .
. . . . .
Vv01g0491 Tc02_g027340 Gorai.002G179000.1 Gorai.
005G161800.1 . . . . . . .
. . . . . . . .
. .
Vv01g0492 Tc02_g027300 Gorai.002G178800.1 .
Gorai.009G331300.1 . . . . . .
. . . . . . . .
. .
Vv01g0493 Tc02_g027290 . . . . .
. . . . . . . .
. . . . . .
Vv01g0494 . . Gorai.005G161600.1 . .
. . . . . . . .
. . . . . . .
Vv01g0495 . . . . . . .
. . . . . . . .
. . . . .
Vv01g0496 . . . . . . .
. . . . . . . .
. . . . .
Vv01g0497 . . . . . . .
. . . . . . . .
. . . . .
Vv01g0498 . . . . . . .
. . . . . . . .
. . . . .
Vv01g0499 . . . . . . .
. . . . . . . .
. . . . .
Vv01g0500 Tc02_g027270 . . . . .
. . . . . . . .
. . . . . .
Vv01g0501 . . Gorai.005G161500.1 . .
. . . . . . . .
. . . . . . .
Vv01g0502 . . . . . . .
. . . . . . . .
. . . . .
Vv01g0503 . . . . . . .
. . . . . . . .
. . . . .
Vv01g0504 Tc02_g027260 Gorai.002G178700.1 . .
. . . . . . . .

. . . . . . . .
Vv01g0505 . . . . . . .
. . . . . . . .
. . . . .
Vv01g0506 . . . . . . .
. . . . . . . .
. . . . .
Vv01g0507 Tc02_g027240 . . . .
Gorai.012G181200.1 . . . . . .
. . . . . . . .
Vv01g0508 . . . . . . .
. . . . . . . .
. . . . .
Vv01g0509 . . . . . . .
. . . . . . Vv12g0872
. . . . . .
Vv01g0510 Tc02_g027230 . Gorai.005G161200.1 .
. . . . . . . .
. . . . . . . .
Vv01g0511 Tc02_g027220 . . . . .
. . . . . . . .
. . . . . .
Vv01g0512 Tc02_g027200 Gorai.002G178600.1 . .
Gorai.008G166800.1 . . . . .
. . . . . . . .
. .
Vv01g0513 Tc02_g027190 . . . . .
. . . . . . . .
. . . . . .
Vv01g0514 Tc02_g027180 . . . . .
. . . . . . . .
. . . . . .
Vv01g0515 Tc02_g027170 . . . . .
. . . . . . . .
. . . . . .
Vv01g0516 Tc02_g027160 . . . Gorai.
008G289800.1 . Vv17g0246 Tc03_g005580 . .
. . . . . . . .
. .
Vv01g0517 Tc02_g027140 Gorai.002G178500.1 .
Gorai.009G330900.1 . . . . . .
. . . . . . . .
. .
Vv01g0518 . . . . . . .
. . . . . . . .
. . . . .
Vv01g0519 . . . . . . .
. . . . . . . .
. . . . .
Vv01g0520 . . . . . .

Vv17g0243 . . . Gorai.005G131900.1 .
. . . . . . . .
Vv01g0521 Tc02_g027060 . . . . .
. . . . . . . .
. . . . . .
Vv01g0522 . . . . . . .
. . . . . . . .
. . . . .
Vv01g0523 . . . . . . .
. . . . . . . .
. . . . .
Vv01g0524 . . . . . .
Vv05g0089 Tc04_g028900 . Gorai.005G064700.1 .
Gorai.011G197500.1 . . . . .
. . .
Vv01g0525 Tc02_g027000 . Gorai.005G160800.1 .
. . Vv05g0087 . . Gorai.
005G064500.1 . Gorai.011G197400.1 . . .
. . . . .
Vv01g0526 Tc02_g028660 . . . . .
Vv05g0083 . . . . . .
. . . . . . .
Vv01g0527 . . . . . .
Vv05g0080 Tc04_g028820 . . . . .
. . . . . . .
Vv01g0528 . . . . . . .
. . . . . . . .
. . . . .
Vv01g0529 Tc02_g028620 . . . . .
Vv05g0078 Tc04_g028790 . Gorai.005G063900.1
. Gorai.011G197300.1 . . . .
. . . .
Vv01g0530 . . . . . . .
. . . . . . . .
. . . . .
Vv01g0531 . . Gorai.005G160700.1 . .
. Vv05g0076 . . Gorai.005G063400.1
. Gorai.011G197200.1 . . . .
. . . .
Vv01g0532 . . . . . . .
. . . . . . . .
. . . . .
Vv01g0533 Tc02_g028610 . . . . .
Vv05g0074 . . Gorai.005G063300.1 .
Gorai.011G197000.1 . . . . .
. . .
Vv01g0534 . . . . . . .
. . . . . . . .
. . . . .
Vv01g0535 . Gorai.002G178000.1 . . .

```
        .       Vv05g0071       .       .       Gorai.005G063200.1
        .       Gorai.011G196900.1      .       .       .       .
        .       .       .       .
Vv01g0536       .       .       .       .       .       .       .
        .       .       .       .       .       .       .       .
        .       .       .       .       .
Vv01g0537       .       .       .       .       .       .       .
        .       .       .       .       .       .       .       .
        .       .       .       .       .
Vv01g0538       Tc02_g028590    Gorai.002G177600.1      .       .
        .       .       Vv05g0069       Tc04_g028750    .
Gorai.005G062800.1      .       Gorai.011G196800.1      .       .
        .       .       .       .       .       .
Vv01g0539       .       .       .       .       .       .       .
        .       .       .       .       .       .       .       .
        .       .       .       .       .
Vv01g0540       Tc02_g028580    .       .       .       .       .
        .       .       .       .       .       .       .       .
        .       .       .       .       .       .
Vv01g0541       .       Gorai.002G177300.1      .       .       .
        .       .       .       .       .       .       .       .
        .       .       .       .       .       .       .
Vv01g0542       .       .       .       .       Gorai.008G289900.1
        .       Vv17g0242       Tc03_g003550    .       .
Gorai.005G132000.1      .       .       .       .       .       .
        .       .       .
Vv01g0543       .       .       .       .       .       .       .
        .       .       .       .       .       .       .       .
        .       .       .       .       .
Vv01g0544       Tc02_g028560    .       Gorai.005G160400.1      .
        Gorai.008G290100.1      Gorai.012G181500.1      .       .
        .       .       .       .       .       .       .       .
        .       .       .       .
Vv01g0545       .       .       .       .       .       .       .
        .       .       .       .       .       .       .       .
        .       .       .       .       .
Vv01g0546       Tc02_g028530    Gorai.002G177100.1      .       .
        Gorai.008G167200.1      .       .       .       .       .
        .       .       .       .       .       .       .       .
        .       .
Vv01g0547       Tc02_g028520    Gorai.002G177000.1      .       .
        .       .       .       .       .       .       .       .
        .       Vv12g0842       Tc01_g018470    Gorai.007G167000.1
        .       .       .       .
Vv01g0548       .       .       .       .       .       .       .
        .       .       .       .       .       .       .       .
        .       .       .       .       .
Vv01g0549       Tc02_g028490    Gorai.002G176900.1      .       .
        .       .       .       .       .       .       .       .
        .       .       .       .       .       .       .       .
```

```
Vv01g0550       .       .       .       .       .       .       .
       .        .       .       .       .       .       .       .
       .        .       .       .       .
Vv01g0551       Tc02_g028440    .       .       .       .       .
       .        .       .       .       .       .       .       .
       .        .       .       .       .       .
Vv01g0552       .       Gorai.002G176700.1      .       .       .
       .        .       .       .       .       .       .       .
       .        .       .       .       .       .       .
Vv01g0553       .       .       .       .       .       .       .
       .        .       .       .       .       .       .       .
       .        .       .       .       .
Vv01g0554       Tc02_g028410    .       .       Gorai.009G330700.1
       .        Gorai.012G181700.1      .       .       .       .
       .        .       .       .       .       .       .       .
       .        .
Vv01g0555       Tc02_g028400    Gorai.002G176500.1      .       .
       .        .       .       .       .       .       .       .
       .        .       .       .       .       .       .       .
Vv01g0556       .       .       .       .       .       .       .
       .        .       .       .       .       .       .       .
       .        .       .       .       .
Vv01g0557       Tc02_g028380    Gorai.002G176300.1      .       .
       .        .       .       .       .       .       .       .
       .        .       .       .       .       .       .       .
Vv01g0558       Tc02_g028370    .       .       .       Gorai.
008G167300.1    .       .       .       .       .       .       .
       .        .       .       .       .       .       .       .
Vv01g0559       Tc02_g028360    Gorai.002G176200.1      .       .
       .        Gorai.012G181800.1      .       .       .       .
       .        .       .       .       .       .       .       .
       .        .
Vv01g0560       Tc02_g028350    Gorai.002G176100.1      .       .
       .        .       .       .       .       .       .       .
       .        .       .       .       .       .       .       .
Vv01g0561       .       .       .       .       .       .       .
       .        .       .       .       .       .       .       .
       .        .       .       .       .
Vv01g0562       .       .       .       .       .       .       .
       .        .       .       .       .       .       .       .
       .        .       .       .       .
Vv01g0563       Tc02_g028340    Gorai.002G175900.1      Gorai.
005G160200.1    .       Gorai.008G167500.1      .       .       .
       .        .       .       .       .       .       .       .
       .        .       .       .
Vv01g0564       Tc02_g026800    .       .       .       .       .
       .        .       .       .       .       .       .       .
       .        .       .       .       .       .
Vv01g0565       Tc02_g028250    Gorai.002G175600.1      .       .
       .        .       .       .       .       .       .       .
```

. . . . . . . .
Vv01g0566 Tc02_g028240 Gorai.002G175300.1 . .
. . . . . . . .
. . . . . . . .
Vv01g0567 . . . . . . .
. . . . . . . .
. . . . .
Vv01g0568 Tc02_g028210 Gorai.002G175100.1 . .
. . . . . . . .
. . . . . . . .
Vv01g0569 . . . . . . .
. . . . . . . .
. . . . .
Vv01g0570 Tc02_g028170 Gorai.002G174700.1 . .
. . . . . . . .
. . . . . . . .
Vv01g0571 . . . . . . .
. . . . . . . .
. . . . .
Vv01g0572 Tc02_g028160 Gorai.002G174600.1 . .
. . . . . . . .
. . . . . . . .
Vv01g0573 . . . . . . .
. . . . . . . .
. . . . .
Vv01g0574 Tc02_g028130 Gorai.002G174500.1 Gorai.
005G159200.1 . . . . . . .
. . . . . . . .
. .
Vv01g0575 . . . . . . .
. . . . . . . .
. . . . .
Vv01g0576 . . . . . . .
. . . . . . . .
. . . . .
Vv01g0577 Tc02_g028100 Gorai.002G174400.1 . .
. . . . . . . .
. . . . . . . .
Vv01g0578 Tc02_g028080 Gorai.002G174200.1 . .
. . . . . . . .
. . . . . . . .
Vv01g0579 Tc02_g028070 Gorai.002G174100.1 Gorai.
005G159100.1 . . . . . . .
. . . Vv12g0829 . . .
. . .
Vv01g0580 Tc02_g028060 Gorai.002G174000.1 Gorai.
005G158900.1 . . . . . . .
. . . . . . . .
. .
Vv01g0581 Tc02_g028050 Gorai.002G173700.1 .

Gorai.009G330500.1 Gorai.008G291000.1 . Vv17g0213
Tc03_g006820 . . . . . .
. . . . . .
Vv01g0582 Tc02_g028040 . Gorai.005G158800.1 .
. . . . . . . .
. . . . . . . .
Vv01g0583 Tc02_g026770 . . . . .
. . . . . . . .
. . . . . .
Vv01g0584 Tc02_g028030 Gorai.002G173500.1 . .
. . . . . . . .
. . . . . . . .
Vv01g0585 Tc02_g028020 Gorai.002G173300.1 Gorai.
005G158500.1 . . Gorai.012G181900.1 . .
. . . . . . . .
. . . .
Vv01g0586 Tc02_g028000 Gorai.002G173200.1 . .
Gorai.008G167700.1 Gorai.012G182000.1 . .
. . . . . . . .
. . . .
Vv01g0587 . . . . . . .
. . . . . . . .
. . . . .
Vv01g0588 Tc02_g027940 . . . . .
. . . . . . . .
. . . . . .
Vv01g0589 Tc02_g027930 Gorai.002G172700.1 . .
. . . . . . . .
. . . . . . . .
Vv01g0590 Tc02_g027880 Gorai.002G172600.1 . .
Gorai.008G291200.1 . Vv17g0189 . .
. Gorai.005G133500.1 . . . .
. . . . .
Vv01g0591 Tc02_g027860 . . . . .
. . . . . . . .
. . . . . .
Vv01g0592 . . . . Gorai.008G168000.1
. . . . . . . .
. . . . . . .
Vv01g0593 Tc02_g027810 . . . .
Gorai.012G182300.1 . . . . . .
. . . . . . . .
Vv01g0594 Tc02_g027800 . . . . .
. . . . . . . .
. . . . . .
Vv01g0595 . Gorai.002G172300.1 . Gorai.
009G330200.1 Gorai.008G168200.1 Gorai.012G182400.1 .
. . . . . . . .
. . . . .
Vv01g0596 Tc02_g027780 . Gorai.005G157100.1 .

. . . . . . . .
. . . . . . . .
Vv01g0597 . . . . . . .
. . . . . . . .
. . . . .
Vv01g0598 . . . . . . .
. . . . . . . .
. . . . .
Vv01g0599 Tc02_g027770 Gorai.002G172200.1 Gorai.
005G157000.1 Gorai.009G330000.1 . . . .
. . . . . . . .
. . . .
Vv01g0600 Tc02_g027750 . . . .
Gorai.012G182600.1 . . . . . .
. . . . . . . .
Vv01g0601 Tc02_g027730 Gorai.002G171800.1 .
Gorai.009G329800.1 Gorai.008G168300.1 Gorai.012G182700.1
. . . . . . . .
. . . . . .
Vv01g0602 Tc02_g027720 Gorai.002G171700.1 . .
Gorai.008G168400.1 . . . . .
. . . . . . . .
. .
Vv01g0603 Tc02_g027700 Gorai.002G169100.1 . .
Gorai.008G168500.1 . . . . .
. . . . . . . .
. .
Vv01g0604 . . . . . . .
. . . . . . . .
. . . . .
Vv01g0605 Tc02_g027650 . . . .
Gorai.012G182800.1 . . . . . .
. . . . . . . .
Vv01g0606 Tc02_g033190 Gorai.013G086300.1 Gorai.
005G156900.1 . . . . . . .
. . . . . . . .
. .
Vv01g0607 . . . . . .
Vv04g1184 Tc01_g002610 . . Gorai.007G200700.1
Gorai.003G013000.1 Gorai.011G100700.1 . .
. . . . .
Vv01g0608 . Gorai.013G028900.1 . . .
. . . . . . . .
. . . . . . .
Vv01g0609 . . . . . . .
. . . . . . . .
. . . . .
Vv01g0610 . . . . . . .
. . . . . . . .
. . . . .

Vv01g0611 . Gorai.013G029000.1 . .
Gorai.008G296900.1 . . . . . .
. . . . . . . .
.
Vv01g0612 . . . . . .
Vv04g1170 . . . . . . .
. . . . . .
Vv01g0613 . Gorai.013G029200.1 . . .
. Vv04g1169 Tc01_g002740 . . .
. . . . . . . .
.
Vv01g0614 . . . . . .
Vv04g1168 . . . . . . .
. . . . . .
Vv01g0615 . . . . . . .
. . . . . . . .
. . . . .
Vv01g0616 . Gorai.013G029300.1 . . .
. Vv04g1166 Tc01_g002750 . . .
. . Vv12g0791 Tc01_g001380 . .
. . .
Vv01g0617 . . . . . .
Vv04g1164 . . . . . .
Vv12g0790 Tc01_g001370 . . . . .
Vv01g0618 . Gorai.013G029400.1 . . .
. . . . . . . .
. . . . . . .
Vv01g0619 . . Gorai.005G183800.1 . .
. . . . . . . .
. . . . . . .
Vv01g0620 . . . . Gorai.008G225200.1
. . . . . . . .
. . . . . . .
Vv01g0621 . Gorai.013G029700.1 . . .
. . . . . . . .
. . . . . . .
Vv01g0622 . . . . . . .
. . . . . . . .
. . . . .
Vv01g0623 . Gorai.013G029800.1 . Gorai.
009G328400.1 . . . . . . .
. . . . . . . .
.
Vv01g0624 . . . . . . .
. . . . . . . .
. . . . .
Vv01g0625 . . . . . . .
. . . . . . . .
. . . . .
Vv01g0626 . . . . . . .

. . . . . . . .
. . . . .
Vv01g0627 . . . Gorai.009G325800.1 .
. . . . . . . .
. . . . . . .
Vv01g0628 Tc02_g033580 . . Gorai.009G328600.1
Gorai.008G224700.1 . . . . .
. . . . . . . .
. .
Vv01g0629 . Gorai.013G029900.1 . . .
. . . . . . . .
. . . . . . .
Vv01g0630 . Gorai.013G030000.1 . . .
. . . . . . . .
. . . . . . .
Vv01g0631 . . . . . . .
. . . . . . . .
. . . . .
Vv01g0632 . . . . . . .
. . . . . . . .
. . . . .
Vv01g0633 . . . . . . .
. . . . . . . .
. . . . .
Vv01g0634 . . . . . . .
. . . . . . . .
. . . . .
Vv01g0635 . . . . . . .
. . . . . . . .
. . . . .
Vv01g0636 . . . . . . .
. . . . . . . .
. . . . .
Vv01g0637 . Gorai.013G030100.1 . .
Gorai.008G294400.1 . Vv04g1129 Tc01_g003080 .
. Gorai.007G202100.1 Gorai.003G014000.1
Gorai.011G101500.1 . . . . . .
.
Vv01g0638 Tc02_g033810 . . . . .
. . . . . . . .
. . . . . .
Vv01g0639 . . . . . . .
. . . . . . . .
. . . . .
Vv01g0640 . Gorai.013G031200.1 . . .
. . . . . . . .
. . . . . . .
Vv01g0641 . Gorai.013G031500.1 . . .
. . . . . . . .
. . . . . . .

```
Vv01g0642          .       .       .       .       .       .       .
        .          .       .       .       .       .       .       .
        .          .       .       .       .
Vv01g0643          .       Gorai.013G031700.1      .       .       .
        .          .       .       .       .       .       .       .
        .          .       .       .       .       .       .
Vv01g0644          .       .       .       .       .       .       .
        .          .       .       .       .       .       .       .
        .          .       .       .       .
Vv01g0645          .       .       .       .       .       .       .
        .          .       .       .       .       .       .       .
        .          .       .       .       .
Vv01g0646          .       Gorai.013G031900.1      .       .       .
        .          .       .       .       .       .       .       .
        .          .       .       .       .       .       .
Vv01g0647          .       Gorai.013G032000.1      .       .
Gorai.008G223600.1         .       .       .       .       .       .
        .          .       .       .       .       .       .       .
        .
Vv01g0648          .       Gorai.013G032100.1      .       .
Gorai.008G169900.1         .       .       .       .       .       .
        .          .       .       .       .       .       .       .
        .
Vv01g0649          .       .       .       .       .       .       .
        .          .       .       .       .       .       .       .
        .          .       .       .       .
Vv01g0650          .       .       .       Gorai.009G328700.1      .
        .          .       .       .       .       .       .       .
        .          .       .       .       .       .       .
Vv01g0651          .       .       .       .       .       Gorai.
012G183700.1       .       .       .       .       .       .       .
        .          .       .       .       .       .       .
Vv01g0652          .       .       .       .       .       .       .
        .          .       .       .       .       .       .       .
        .          .       .       .       .
Vv01g0653          .       .       .       .       .       .       .
        .          .       .       .       .       .       .       .
        .          .       .       .       .
Vv01g0654          .       .       .       Gorai.009G328800.1
Gorai.008G292200.1         .       .       .       .       .       .
        .          .       .       .       .       .       .       .
        .
Vv01g0655          .       .       .       Gorai.009G329000.1      .
        .          .       .       .       .       .       .       .
        .          .       .       .       .       .       .
Vv01g0656          .       .       Gorai.005G153900.1      .       .
        .          Vv12g0754       .       Gorai.007G164400.1      .
        Gorai.001G189600.1         .       .       .       .       .
        .          .       .       .
Vv01g0657          .       .       .       .       .       .       .
```

. . . . . . . .
. . . . .
Vv01g0658 . . . . . . .
. . . . . . . .
. . . . .
Vv01g0659 . . . . . . .
. . . . . . . .
. . . . .
Vv01g0660 . Gorai.002G171300.1 . Gorai.
009G329100.1 . . . . . . .
. . . . . . . .
.
Vv01g0661 . Gorai.002G171200.1 . .
Gorai.008G220500.1 . . . . . .
. . . . . . . .
.
Vv01g0662 . . . . . . .
. . . . . . . .
. . . . .
Vv01g0663 . . . . . . .
. . . . . . . .
. . . . .
Vv01g0664 . . . . . .
Vv14g0658 . . . . . . .
. . . . . .
Vv01g0665 . . . . . . .
. . . . . . . .
. . . . .
Vv01g0666 . Gorai.002G171100.1 . Gorai.
009G329200.1 Gorai.008G293000.1 . . . .
. . . . . . . .
. . .
Vv01g0667 . . . . . . .
. . . . . . . .
. . . . .
Vv01g0668 . . Gorai.005G186700.1 .
Gorai.008G171500.1 . . . . . .
. . . . . . . .
.
Vv01g0669 . . . . . . .
. . . . . . . .
. . . . .
Vv01g0670 . Gorai.002G170900.1 Gorai.005G186800.1
. . . . . . . .
. . . . . . . .
.
Vv01g0671 . . . . . . .
. . . . . . . .
. . . . .
Vv01g0672 . . Gorai.005G186900.1 . .

. Vv14g0622 . Gorai.013G121000.1
Gorai.007G282000.1 . . . . . .
. . . .
Vv01g0673 . . . . . . .
. . . . . . . .
. . . . .
Vv01g0674 . Gorai.002G170700.1 Gorai.005G187000.1
. . . Vv14g0620 . .
Gorai.007G282100.1 . . . . . .
. . . .
Vv01g0675 . . . . . .
Vv14g0617 Tc04_g024410 . Gorai.007G282500.1 .
. . . . . . . .
.
Vv01g0676 . Gorai.002G170600.1 Gorai.005G187000.1
. . . Vv14g0614 Tc04_g024400 .
Gorai.007G282900.1 . . . . .
. . . . .
Vv01g0677 . . Gorai.005G186900.1 . .
. Vv14g0612 . . Gorai.007G283000.1
. . . . . . . .
. .
Vv01g0678 . . Gorai.005G186800.1 . .
. Vv14g0611 Tc04_g024390 . Gorai.
007G283200.1 . . . . . . .
. . .
Vv01g0679 . . Gorai.005G186700.1 . .
. Vv14g0609 Tc04_g024380 . Gorai.
007G283400.1 . . . . . . .
. . .
Vv01g0680 . . . . . . .
. . . . . . . .
. . . . .
Vv01g0681 Tc02_g007920 Gorai.002G170500.1 .
Gorai.009G329300.1 . . Vv12g0740 .
Gorai.007G163300.1 Gorai.001G225600.1 . .
Gorai.001G100100.1 . . . . . .
.
Vv01g0682 . . . . . . .
. . . . . . . .
. . . . .
Vv01g0683 . Gorai.002G170400.1 . .
Gorai.008G291400.1 . Vv17g1166 . . .
. . . . . . . .
. .
Vv01g0684 . . . . . . .
. . . . . . . .
. . . . .
Vv01g0685 . Gorai.002G170200.1 . . .
. . . . . . . .

```
          .       .       .       .       .       .       .
Vv01g0686         .       .       .       .       Gorai.008G293200.1
          .       .       .       .       .       .       .       .
          .       .       .       .       .       .       .
Vv01g0687         .       .       .       .       .       .       .
          .       .       .       .       .       .       .       .
          .       .       .       .       .
Vv01g0688         .       Gorai.002G169900.1      .       .       .
          .       .       .       .       .       .       .       .
          .       .       .       .       .       .       .
Vv01g0689         .       .       .       .       .       .       .
          .       .       .       .       .       .       .       .
          .       .       .       .       .
Vv01g0690         .       .       .       .       .       .       .
          .       .       .       .       .       .       .       .
          .       .       .       .       .
Vv01g0691         Tc02_g007490    .       .       .       .       .
          .       .       .       .       .       .       .       .
          .       .       .       .       .       .
Vv01g0692         .       .       .       .       .       .       .
          .       .       .       .       .       .       .       .
          .       .       .       .       .
Vv01g0693         .       .       .       .       .       .       .
          .       .       .       .       .       .       .       .
          .       .       .       .       .
Vv01g0694         .       .       .       .       .       .       .
          .       .       .       .       .       .       .       .
          .       .       .       .       .
Vv01g0695         .       .       .       .       .       .       .
          .       .       .       .       .       .       .       .
          .       .       .       .       .
Vv01g0696         .       .       .       .       .       .       .
          .       .       .       .       .       .       .       .
          .       .       .       .       .
Vv01g0697         Tc02_g025980    Gorai.002G169300.1      .
Gorai.009G329400.1        .       .       Vv17g1136       .       .
          .       .       .       .       .       .       .       .
          .       .       .
Vv01g0698         .       .       .       .       .       .       .
          .       .       .       .       .       .       .       .
          .       .       .       .       .
Vv01g0699         .       .       .       .       .       .       .
          .       .       .       .       .       .       .       .
          .       .       .       .       .
Vv01g0700         .       Gorai.002G166300.1      .       .
Gorai.008G294300.1        .       .       .       .       .       .
          .       .       .       .       .       .       .       .
          .
Vv01g0701         .       .       .       .       .       .       .
          .       .       .       .       .       .       .       .
```

. . . . .
Vv01g0702 . . . . . . .
. . . . . . . .
. . . . .
Vv01g0703 Tc02_g025850 . . . . .
. . . . . . . .
. . . . . .
Vv01g0704 . . . . . . .
. . . . . . . .
. . . . .
Vv01g0705 Tc02_g025800 . . . . .
. . . . . . . .
. . . . . .
Vv01g0706 Tc02_g025790 . Gorai.005G173900.1 .
. . . . . . . .
. . . . . . . .
Vv01g0707 . . Gorai.005G174100.1 . .
. . . . . . . .
. . . . . . .
Vv01g0708 Tc02_g025780 . Gorai.005G174200.1 .
. . . . . . . .
. . . . . . . .
Vv01g0709 Tc02_g025770 . Gorai.005G174300.1 .
. Gorai.012G173900.1 Vv17g1108
Tc03_g014070 . . . Gorai.008G204200.1 .
. . . . . . .
Vv01g0710 Tc02_g025760 Gorai.002G190000.1 . .
. . . . . . . .
. . . . . . . .
Vv01g0711 . . . . . . .
. . . . . . . .
. . . . .
Vv01g0712 Tc02_g025750 . . . Gorai.
008G162600.1 . . . . . . .
. . . . . . . .
Vv01g0713 Tc02_g025730 . Gorai.005G174500.1
Gorai.009G334300.1 . . . . . .
. . . . . . . .
. .
Vv01g0714 Tc02_g025720 Gorai.002G189900.1 Gorai.
005G174600.1 . Gorai.008G162500.1 . Vv17g1097
. . . . . . Vv14g1268
Tc04_g002320 . Gorai.001G267300.1 .
Gorai.006G036300.1 .
Vv01g0715 Tc02_g025710 Gorai.002G189700.1 .
Gorai.009G334400.1 Gorai.008G162400.1 . . .
. . . . . . . .
. . . .
Vv01g0716 . . . . . . .
. . . . . . . .

. . . . .
Vv01g0717 Tc02_g025690 . . . .
Gorai.012G173800.1 . . . . . . .
. . . . . . . .
Vv01g0718 Tc02_g025610 Gorai.002G189400.1 Gorai.
005G174800.1 . Gorai.008G162200.1 . . .
. . . . . . . .
. . . .
Vv01g0719 . . . . . . .
. . . . . . . .
. . . . .
Vv01g0720 Tc02_g025600 . . . . .
. . . . . . . .
. . . . . .
Vv01g0721 . . Gorai.005G174900.1 . .
. . . . . . . .
. . . . . . .
Vv01g0722 Tc02_g025590 . . . Gorai.
008G288600.1 . . . . . . .
. . . . . . . .
Vv01g0723 Tc02_g025580 . . . Gorai.
008G286100.1 . . . . . . .
. . . . . . . .
Vv01g0724 . . . . . . .
. . . . . . . .
. . . . .
Vv01g0725 Tc02_g025570 . Gorai.005G175000.1 .
Gorai.008G162000.1 . . . . .
. . . Vv14g1271 . . .
. . .
Vv01g0726 Tc02_g025520 . . . Gorai.
008G161800.1 . Vv17g1093 Tc03_g013960 . .
. Gorai.008G203800.1 . . . .
. . . .
Vv01g0727 Tc02_g025510 Gorai.002G189100.1 . .
Gorai.008G161700.1 . . . . .
. . . . . . . .
. .
Vv01g0728 Tc02_g025500 Gorai.002G188900.1 . .
. . . . . . . .
. . . . . . . .
Vv01g0729 Tc02_g025470 Gorai.002G188800.1 Gorai.
005G186600.1 . . . . . . .
. . . . . . . .
. .
Vv01g0730 Tc02_g025410 . Gorai.005G183600.1 .
. . . . . . . .
. Vv14g1276 . Gorai.011G093700.1 .
. . .
Vv01g0731 Tc02_g025400 . Gorai.005G175200.1 .

. . Vv17g1083 Tc03_g013840 . .
Gorai.007G041500.1 . . Vv14g1279 .
. . . . .
Vv01g0732 . . Gorai.005G175300.1 . .
. . . . . . . .
Vv14g1281 . . . . .
Gorai.012G066300.1
Vv01g0733 Tc02_g025380 . Gorai.005G175500.1 .
. . . . . . . .
. . . . . . . .
Vv01g0734 . . . . . . .
. . . . . . . .
. . . . .
Vv01g0735 Tc02_g025370 Gorai.002G188600.1 . .
. Gorai.012G173600.1 . . . .
. . . . . . . .
. .
Vv01g0736 Tc02_g025330 . . . .
Gorai.012G173500.1 . . . . . .
. . . . . . . .
Vv01g0737 Tc02_g025310 . Gorai.005G175700.1
Gorai.009G335200.1 . . Vv17g1077
Tc03_g013760 . . . Gorai.008G202700.1 .
Vv14g1306 . . . . Gorai.
006G034900.1 .
Vv01g0738 . . . . . . .
. . . . . . . .
. . . . .
Vv01g0739 Tc02_g025290 . Gorai.005G175900.1 .
. . . . . . . .
. . . . . . . .
Vv01g0740 . . . . . . .
. . . . . . . .
. . . . .
Vv01g0741 Tc02_g025280 . . . . .
. . . . . . . .
. . . . . .
Vv01g0742 . . . . . . .
. . . . . . . .
. . . . .
Vv01g0743 . . . . . . .
. . . . . . . .
. . . . .
Vv01g0744 . . . . . . .
. . . . . . . .
. . . . .
Vv01g0745 . . . . . . .
. . . . . . . .
. . . . .
Vv01g0746 Tc02_g025270 . Gorai.005G176000.1 .

. . . . . . . .
. . . . . . . .
Vv01g0747 . . . . .
Vv17g1058 Tc03_g013680 Gorai.001G063800.1 . .
. . . . . . . .
.
Vv01g0748 Tc02_g025260 . Gorai.005G176100.1 .
Gorai.008G161500.1 . . . . .
. . . . . . . .
. .
Vv01g0749 . . . . . . .
. . . . . . . .
. . . . .
Vv01g0750 . . . . . . .
. . . . . . . .
. . . . .
Vv01g0751 Tc02_g025240 . . . . .
. . . . . . . .
. . . . . .
Vv01g0752 Tc02_g025220 . Gorai.005G176400.1 .
. Gorai.012G173300.1 . . . .
. . . . . . . .
. .
Vv01g0753 Tc02_g025190 Gorai.002G188400.1 Gorai.
005G176500.1 . . . . . . .
. . . Vv14g1398 Tc04_g000990 .
Gorai.001G262500.1 . . .
Vv01g0754 Tc02_g025170 . . . . .
. . . . . . . .
. . . . . .
Vv01g0755 . Gorai.002G188300.1 . . .
. . . . . . . .
. . . . . . .
Vv01g0756 . . . . . . .
. . . . . . . .
. . . . .
Vv01g0757 Tc02_g025160 . . . Gorai.
008G161400.1 Gorai.012G173200.1 . . . .
. . . . . . . .
. .
Vv01g0758 Tc02_g025150 . Gorai.005G176600.1 .
. . . . . . . .
. . . . . . . .
Vv01g0759 . Gorai.002G216000.1 . . .
. . . . . . . .
. . . . . . .
Vv01g0760 . . . . . . .
. . . . . . . .
. . . . .
Vv01g0761 . . . . . . .

. . . . . . . .
. . . . .
Vv01g0762 . . . . . . .
. . . . . . . .
. . . . .
Vv01g0763 Tc02_g025140 . . Gorai.009G335400.1
. . Vv17g1055 Tc03_g013600 Gorai.
001G064000.1 . . . . . . .
. . . .
Vv01g0764 Tc02_g025130 Gorai.002G188100.1 Gorai.
005G176700.1 . . . . . . .
. . . . . . . .
. .
Vv01g0765 Tc02_g025120 . . . . .
. . . . . . . .
. . . . . .
Vv01g0766 Tc02_g025110 . Gorai.005G176800.1 .
. . . . . . . .
. . . . . . . .
Vv01g0767 Tc02_g025100 . Gorai.005G176900.1 .
. . . . . . . .
. . . . . . . .
Vv01g0768 . . . . . . .
. . . . . . . .
. . . . .
Vv01g0769 Tc02_g025080 . Gorai.005G177000.1 .
. . . . . . . .
. . . . . . . .
Vv01g0770 Tc02_g025050 . Gorai.005G177200.1 .
. . Vv17g1048 Tc03_g013560 Gorai.
001G064200.1 . . Gorai.008G201900.1 . .
. . . . . .
Vv01g0771 Tc02_g025040 . . . . .
. . . . . . . .
. . . . . .
Vv01g0772 Tc02_g024990 . Gorai.005G177300.1 .
. . . . . . . .
. . . . . . . .
Vv01g0773 . . . . . . .
. . . . . . . .
. . . . .
Vv01g0774 Tc02_g024980 . Gorai.005G177400.1 .
Gorai.008G161300.1 . Vv14g1308
Tc04_g001980 Gorai.011G094500.1 Gorai.001G266300.1 .
. Gorai.012G065300.1 . . . .
. . .
Vv01g0775 Tc02_g024970 Gorai.002G187900.1 . .
. . . . . . . .
. . . . . . . .
Vv01g0776 . . . . . . .

. . . . . . . .
. . . . .
Vv01g0777 Tc02_g024950 Gorai.002G187800.1 .
Gorai.009G335700.1 Gorai.008G161200.1 . Vv14g1312
Tc04_g001940 . . . Gorai.006G034700.1
Gorai.012G065000.1 . . . . .
. .
Vv01g0778 . . . . . . .
. . . . . . . .
. . . . .
Vv01g0779 . Gorai.002G167200.1 . .
Gorai.008G161100.1 . . . . . .
. . . . . . . .
.
Vv01g0780 . . . . . . .
. . . . . . . .
. . . . .
Vv01g0781 Tc02_g023430 . Gorai.005G182800.1 .
. . . . . . . .
. . . . . . . .
Vv01g0782 . . . . . . .
. . . . . . . .
. . . . .
Vv01g0783 Tc02_g023420 . . . Gorai.
008G158700.1 . . . . . . .
. . . . . . . .
Vv01g0784 . . . . . . .
. . . . . . . .
. . . . .
Vv01g0785 Tc02_g023410 . Gorai.005G182900.1 .
. . Vv14g1418 Tc04_g000830 . .
. . . . . . . .
. .
Vv01g0786 Tc02_g023390 . . . .
Gorai.012G171400.1 . . . . . .
. . . . . . . .
Vv01g0787 . . . . . . .
. . . . . . . .
. . . . .
Vv01g0788 . . . . . . .
. . . . . . . .
. . . . .
Vv01g0789 . . . . . . .
. . . . . . . .
. . . . .
Vv01g0790 Tc02_g023370 . Gorai.005G183000.1 .
. . . . . . . .
. . . . . . . .
Vv01g0791 Tc02_g000580 . . . . .
Vv14g1425 Tc04_g000760 . . .

Gorai.006G029600.1 . . . . . .
. .
Vv01g0792 . . . . Gorai.008G158600.1
. . . . . . . .
. . . . . . .
Vv01g0793 . . . . . . .
. . . . . . . .
. . . . .
Vv01g0794 Tc02_g023320 . . . . .
. . . . . . . .
. . . . . .
Vv01g0795 . . Gorai.005G183200.1 . .
. . . . . . . .
. . . . . . .
Vv01g0796 . Gorai.002G213300.1 . Gorai.
009G337400.1 . . . . . . .
. . . . . . . .
.
Vv01g0797 . . Gorai.005G183300.1 .
Gorai.008G158500.1 . . . . . .
. . . . . . . .
.
Vv01g0798 . . . . . . .
. . . . . . . .
. . . . .
Vv01g0799 . . . . . . .
. . . . . . . .
. . . . .
Vv01g0800 . . . . Gorai.008G155700.1
. Vv17g0896 Tc03_g000920 . . .
Gorai.008G158500.1 . Vv14g1435
Tc04_g000710 . Gorai.001G261100.1 . . .
Vv01g0801 . . Gorai.005G183400.1 . .
. . . . . . . .
. . . . . . .
Vv01g0802 . . Gorai.005G183500.1 . .
. Vv17g0893 Tc03_g000880 Gorai.001G068600.1
. . . . . . . .
. . .
Vv01g0803 . . . . . . .
. . . . . . . .
. . . . .
Vv01g0804 . Gorai.002G165700.1 Gorai.005G183600.1
. . . . . . . .
. . . . . . . .
.
Vv01g0805 Tc02_g000600 Gorai.002G213100.1 . .
. Gorai.012G171200.1 . . . .
. . . Vv14g1439 Tc04_g000670 .
. . Gorai.006G029100.1 Gorai.012G060500.1

Vv01g0806 . Gorai.002G213000.1 . Gorai.
009G343300.1 . . . . . . .
. . . . . . .
.
Vv01g0807 . . Gorai.005G183800.1 . .
. Vv17g0891 Tc03_g000820 . . .
. . . . . . . .
.
Vv01g0808 . . . . . .
Vv17g0882 Tc03_g000730 . . . . .
. . . . . . .
Vv01g0809 . . . . . . .
. . . . . . . .
. . . . .
Vv01g0810 . . . . . . .
. . . . . . . .
. . . . .
Vv01g0811 . . . . . . .
. . . . . . . .
. . . . .
Vv01g0812 . Gorai.002G164400.1 Gorai.005G183900.1
. . . . . . . .
. . . . . . . .
.
Vv01g0813 . . . . . . .
. . . . . . . .
. . . . .
Vv01g0814 . . . Gorai.009G343600.1 .
Gorai.012G171100.1 . . . . .
. . Vv14g1440 Tc04_g000660 .
Gorai.001G260900.1 . . .
Vv01g0815 . . . . . . .
. . . . . . . .
. . . . .
Vv01g0816 . . . . . . .
. . . . . . . .
. . . . .
Vv01g0817 . . . . . . .
. . . . . . . .
. . . . .
Vv01g0818 . . . . . . .
. . . . . . . .
. . . . .
Vv01g0819 . . . . . . .
. . . . . . . .
. . . . .
Vv01g0820 . . Gorai.005G184200.1 . .
. . . . . . . .
. . . . . . .
Vv01g0821 Tc02_g023300 . Gorai.005G184300.1 .

```
        .       .       Vv17g0877       Tc03_g000690    .       .
        .       .       .       Vv14g1446       Tc04_g000550    .
        .       .       .       Gorai.012G060000.1
Vv01g0822       .       .       .       .       .       .       .
        .       .       .       .       .       .       .       .
        .       .       .       .       .
Vv01g0823       .       .       .       .       .       .       .
        .       .       .       .       .       .       .       .
        .       .       .       .       .
Vv01g0824       .       .       .       .       .       .
Vv17g0873       Tc03_g000650    .       .       .       .
Gorai.006G187700.1      .       .       .       .       .       .
        .
Vv01g0825       .       .       .       .       .       .       .
        .       .       .       .       .       .       .       .
        .       .       .       .       .
Vv01g0826       .       .       .       .       .       .       .
        .       .       .       .       .       .       .       .
        .       .       .       .       .
Vv01g0827       Tc02_g000860    .       .       .       .       .
        .       .       .       .       .       .       .       .
        .       .       .       .       .       .
Vv01g0828       .       .       .       .       .       .       .
        .       .       .       .       .       .       .       .
        .       .       .       .       .
Vv01g0829       .       .       .       .       .       .       .
        .       .       .       .       .       .       .       .
        .       .       .       .       .
Vv01g0830       .       .       .       .       .       .       .
        .       .       .       .       .       .       .       .
        .       .       .       .       .
Vv01g0831       .       .       .       .       .       .       .
        .       .       .       .       .       .       .       .
        .       .       .       .       .
Vv01g0832       Tc02_g023290    .       Gorai.005G184400.1      .
        .       .       Vv04g1448       .       .       .       .
        .       .       Vv14g1447       Tc04_g000520    .       .
        .       .       Gorai.012G059900.1
Vv01g0833       .       .       .       .       .       .
Vv04g1446       .       .       .       .       .       .       .
        .       .       .       .       .       .
Vv01g0834       .       .       .       .       .       .
Vv04g1444       .       .       .       .       .       .       .
        .       .       .       .       .       .
Vv01g0835       .       .       .       .       .       .
Vv04g1443       Tc01_g010770    Gorai.007G063300.1      .       .
        .       .       .       .       .       .       .       .
        .
Vv01g0836       .       Gorai.002G164300.1      Gorai.005G184500.1
        Gorai.009G344100.1      .       .       Vv04g1441       .
```

. . . . . . . .
. . . .
Vv01g0837 . . . . . . .
. . . . . . . .
. . . . .
Vv01g0838 . . Gorai.005G184600.1 . .
. . . . . . . .
. . . . . . .
Vv01g0839 . . . . . . .
. . . . . . . .
. . . . .
Vv01g0840 . . Gorai.005G184900.1 Gorai.
009G344200.1 . . . . . . .
. . . . . . . .
.
Vv01g0841 . . . . . . .
. . . . . . . .
. . . . .
Vv01g0842 . . . . . .
Vv17g0862 . . . . . Gorai.
006G187800.1 . . . . . . .
Vv01g0843 . . . . . . .
. . . . . . . .
. . . . .
Vv01g0844 . . . . . . .
. . . . . . . .
. . . . .
Vv01g0845 . Gorai.002G164200.1 . . .
. . . . . . . .
. . . . . . .
Vv01g0846 . . . . . . .
. . . . . . . .
. . . . .
Vv01g0847 . . . . . . .
. . . . . . . .
. . . . .
Vv01g0848 Tc02_g023090 . . . . .
. . . . . . . .
. . . . . .
Vv01g0849 . . . . . . .
. . . . . . . .
. . . . .
Vv01g0850 Tc02_g023050 . Gorai.005G185700.1 .
. . . . . . . .
. Vv14g1462 Tc04_g000390 . .
Gorai.007G077300.1 . .
Vv01g0851 . . . . . . .
. . . . . . . .
. . . . .
Vv01g0852 . . . . . . .

. . . . . . . .
. . . . .
Vv01g0853 Tc02_g023030 . Gorai.005G185900.1 .
. . . . . . . .
. . . . . . . .
Vv01g0854 . . . . . . .
. . . . . . . .
. . . . .
Vv01g0855 . . . . . Gorai.
012G170700.1 . . . . . . .
. . . . . .
Vv01g0856 . Gorai.002G161300.1 . . .
. . . . . . . .
. . . . . . .
Vv01g0857 . . . . . . .
. . . . . . .
. . . . .
Vv01g0858 . . . . . . .
. . . . . . . .
. . . . .
Vv01g0859 . . . . . . .
. . . . . . . .
. . . . .
Vv01g0860 . . . . . . .
. . . . . . . .
. . . . .
Vv01g0861 . . . Gorai.009G344300.1 .
Gorai.012G170400.1 . . . . .
. . . . . . . .
.
Vv01g0862 . . . . . . .
. . . . . . . .
. . . . .
Vv01g0863 . . . . . . .
. . . . . . . .
. . . . .
Vv01g0864 Tc02_g022870 . . . .
Gorai.012G123400.1 . . . . . .
. . . . . . . .
Vv01g0865 . . . . . . .
. . . . . . . .
. . . . .
Vv01g0866 Tc02_g022840 . Gorai.005G186300.1 .
. Gorai.012G123200.1 . . . .
. . . . . . . .
. .
Vv01g0867 . . . . . . .
. . . . . . . .
. . . . .
Vv01g0868 . . . . . Gorai.

012G123100.1 Vv17g0857 . . . .
Gorai.008G158300.1 . Vv14g1477 Tc04_g000310 .
. . Gorai.006G028100.1 .
Vv01g0869 . . . . . . .
. . . . . . . .
. . . . .
Vv01g0870 . Gorai.002G159700.1 Gorai.005G186600.1
. . . . . . . .
. . . . . . . .
.
Vv01g0871 . . . Gorai.009G345000.1 .
. . . . . . . .
Vv14g1481 . . . .
Gorai.012G058700.1
Vv01g0872 . . . . . .
Vv17g0851 Tc03_g000370 Gorai.001G071200.1 . .
. . . . . . . .
.
Vv01g0873 . . . Gorai.009G345200.1 .
. . . . . . . .
. . . . . . .
Vv01g0874 . . Gorai.005G187100.1 . .
. . . . . . . .
. . . . . . .
Vv01g0875 . . . . . .
Vv17g0843 Tc03_g000310 . . . .
Gorai.006G187400.1 . . . . . .
.
Vv01g0876 . . Gorai.005G187300.1 . .
. Vv17g0842 Tc03_g000300 . . .
. . . . . . . .
.
Vv01g0877 . . Gorai.005G187400.1 . .
. . . . . . . .
. . . . . . .
Vv01g0878 . . . . . . .
. . . . . . . .
. . . . .
Vv01g0879 . . Gorai.005G187600.1 Gorai.
009G345300.1 . . . . . . .
. . . . . . . .
.
Vv01g0880 . . Gorai.005G187700.1 Gorai.
009G345400.1 . . . . . . .
. . . . . . . .
.
Vv01g0881 . . . . . . .
. . . . . . . .
. . . . .
Vv01g0882 . . . Gorai.009G345500.1 .

. . . . . . . .
. . . . . . .
Vv01g0883 . . Gorai.005G187800.1 . .
. Vv17g0839 . . . . .
. . . . . . . .
Vv01g0884 . . . . . . .
. . . . . . . .
. . . . .
Vv01g0885 . . Gorai.005G188000.1 Gorai.
009G345700.1 . . Vv17g0832 Tc03_g000240 .
. . . . . . . .
. . .
Vv01g0886 . . . Gorai.009G345800.1 .
. . . . . . . .
. . . . . . .
Vv01g0887 . . . Gorai.009G345900.1 .
. . . . . . . .
. . . . . . .
Vv01g0888 . . . . . . .
. . . . . . . .
. . . . .
Vv01g0889 . . . . . . .
. . . . . . . .
. . . . .
Vv01g0890 . . . . . . .
. . . . . . . .
. . . . .
Vv01g0891 . . . Gorai.009G346000.1 .
. . . . . . . .
. . . . . . .
Vv01g0892 . . . Gorai.009G346100.1
Gorai.008G189700.1 . Vv17g0576 . . .
. Gorai.008G189700.1 . Vv14g1504 .
. . . . .
Vv01g0893 . . . . . . .
. . . . . . . .
. . . . .
Vv01g0894 . . . . . . .
. . . . . . . .
. . . . .
Vv01g0895 Tc02_g022680 . Gorai.005G188300.1 .
. . . . . . . .
. . . . . . . .
Vv01g0896 . . . . . . .
. . . . . . . .
. . . . .
Vv01g0897 . . . . . . .
. . . . . . . .
. . . . .
Vv01g0898 Tc02_g022640 . Gorai.005G188700.1 .

```
         .       .       .       .       .       .       .       .
         .       .       .       .       .       .       .       .
Vv01g0899        .       .       .       .       .       .       .
         .       .       .       .       .       .       .       .
         .       .       .       .       .
Vv01g0900        Tc02_g022600    .       .       Gorai.009G346400.1
         .       .       .       .       .       .       .       .
         .       .       .       .       .       .       .       .
Vv01g0901        Tc02_g022580    .       .       Gorai.009G346600.1
         .       .       .       .       .       .       .       .
         .       .       .       .       .       .       .       .
Vv01g0902        .       .       .       .       .       .       .
         .       .       .       .       .       .       .       .
         .       .       .       .       .
Vv01g0903        Tc02_g022570    .       Gorai.005G188800.1      .
         .       .       .       .       .       .       .       .
         .       .       .       .       .       .       .       .
Vv01g0904        .       .       .       .       .       .       .
         .       .       .       .       .       .       .       .
         .       .       .       .       .
Vv01g0905        .       .       .       .       .       .       .
         .       .       .       .       .       .       .       .
         .       .       .       .       .
Vv01g0906        Tc02_g022550    .       Gorai.005G188900.1      .
         .       .       .       .       .       .       .       .
         .       .       .       .       .       .       .       .
Vv01g0907        Tc02_g022530    .       Gorai.005G189000.1      .
         .       .       Vv17g0583       .       .       .       .
         .       .       .       .       .       .       .       .
         .
Vv01g0908        Tc02_g022520    .       Gorai.005G189100.1      .
         .       .       .       .       .       .       .       .
         .       .       .       .       .       .       .       .
Vv01g0909        .       .       .       .       .       .       .
         .       .       .       .       .       .       .       .
         .       .       .       .       .
Vv01g0910        Tc02_g022460    .       Gorai.005G189200.1      .
         .       .       .       .       .       .       .       .
         .       .       .       .       .       .       .       .
Vv01g0911        .       .       .       .       .       .       .
         .       .       .       .       .       .       Vv14g1529
         Tc04_g007270    .       .       .       Gorai.006G025900.1
         .
Vv01g0912        .       .       .       .       .       .
Vv17g0589        Tc03_g009010    .       .       .       .       .
         .       .       .       .       .       .       .
Vv01g0913        .       Gorai.002G166900.1      .       .       .
         Gorai.012G121400.1      .       .       .       .       .
         .       .       .       .       .       .       .       .
         .
```

Vv01g0914 . . . . . Gorai.
012G121100.1 . . . . . . .
. . . . . . .
Vv01g0915 Tc02_g022310 . . . .
Gorai.012G121000.1 . . . . . .
. . . . . . . .
Vv01g0916 . . . . . Gorai.
012G120900.1 . . . . . . .
. . . . . . .
Vv01g0917 . . . . . . .
. . . . . . . .
. . . . .
Vv01g0918 Tc02_g022230 Gorai.002G165100.1 . .
. . . . . . . .
. . . . . . . .
Vv01g0919 . . . . . . .
. . . . . . . .
. . . . .
Vv01g0920 Tc02_g022220 . Gorai.005G189300.1 .
. . . . . . . .
. . . . . . . .
Vv01g0921 Tc02_g022210 . . . . .
. . . . . . .
Vv14g1531 Tc04_g007300 . . . . .
Vv01g0922 Tc02_g022200 Gorai.002G164600.1 Gorai.
005G189500.1 . . . . . . .
. . . Vv14g1532 . . .
. . .
Vv01g0923 . . . . . . .
. . . . . . . .
. . . . .
Vv01g0924 Tc02_g022190 . . . . .
. . . . . . . .
. . . . . .
Vv01g0925 Tc02_g022150 . Gorai.005G189600.1 .
. . . . . . . .
. . . . . . . .
Vv01g0926 Tc02_g022140 . . Gorai.009G346700.1
. . . . . . . .
. Vv14g1540 Tc04_g016040 . .
Gorai.007G198800.1 . .
Vv01g0927 . . . Gorai.009G346800.1 .
. . . . . . . .
. . . . . . .
Vv01g0928 . Gorai.002G162600.1 . . .
. . . . . . . .
. . . . . . .
Vv01g0929 . . . . . . .
. . . . . . . .
. . . . .

Vv01g0930 . . . . . . .
. . . . . . . .
. . . . .
Vv01g0931 Tc02_g034200 . . . . .
Vv17g0603 . . . . . .
. . . . . . .
Vv01g0932 . . . . . . .
. . . . . . . .
. . . . .
Vv01g0933 Tc02_g021900 . Gorai.005G189700.1 .
. . . . . . . .
. . . . . . . .
Vv01g0934 Tc02_g021880 Gorai.002G159700.1 . .
. . . . . . . .
. . . . . . . .
Vv01g0935 . . . . . . .
. . . . . . . .
. . . . .
Vv01g0936 Tc02_g021830 . . Gorai.009G347000.1
. . . . . . . .
. . . . . . . .
Vv01g0937 Tc02_g021810 . Gorai.005G189800.1 .
Gorai.008G190300.1 . Vv17g0615
Tc03_g009540 Gorai.001G073600.1 . . Gorai.
008G190300.1 . . . . . . .
.
Vv01g0938 . . . . . . .
. . . . . . . .
. . . . .
Vv01g0939 . . . . . . .
. . . . . . . .
. . . . .
Vv01g0940 Tc02_g034120 . . . . .
Vv17g0616 Tc03_g009550 . . . .
. Vv14g1543 Tc04_g025970 . . .
. .
Vv01g0941 Tc02_g034110 . . . Gorai.
008G183000.1 . . . . . . .
. . . . . . . .
Vv01g0942 Tc02_g034100 . . . . .
. . . . . . . .
. . . . . .
Vv01g0943 . . . . . . .
. . . . . . . .
. . . . .
Vv01g0944 Tc02_g034090 Gorai.013G077900.1 . .
Gorai.008G182900.1 . . . . .
. . . . . . . .
. .
Vv01g0945 Tc02_g034070 . . . . .

```
        .       .       .       .       .       .       .       .
        .       .       .       .       .       .
Vv01g0946       Tc02_g034060    Gorai.013G077700.1      .       .
        .       .       .       .       .       .       .       .
        .       .       .       .       .       .       .       .
Vv01g0947       .       .       .       .       .       .       .
        .       .       .       .       .       .       .       .
        .       .       .       .       .
Vv01g0948       .       .       .       .       .       .       .
        .       .       .       .       .       .       .       .
        .       .       .       .       .
Vv01g0949       .       .       .       .       .       .       .
        .       .       .       .       .       .       .       .
        .       .       .       .       .
Vv01g0950       Tc02_g034050    .       .       .       Gorai.
008G182800.1    .       Vv17g0620       Tc03_g009570    Gorai.
001G073500.1    .       .       Gorai.008G182800.1      .       .
        .       .       .       .       .       .
Vv01g0951       Tc02_g034040    .       .       .       Gorai.
008G190600.1    .       Vv17g0621       Tc03_g009580    .       .
        .       Gorai.008G182700.1      .       Vv14g1551       .
        .       .       .       .       .
Vv01g0952       Tc02_g034030    .       .       .       Gorai.
008G182600.1    .       .       .       .       .       .       .
        .       .       .       .       .       .       .       .
Vv01g0953       Tc02_g034020    .       .       .       .       .
        Vv17g0625       Tc03_g009670    .       .       .       .
        .       Vv14g1554       .       .       .       .       .
        .
Vv01g0954       .       .       .       .       .       .       .
        .       .       .       .       .       .       .       .
        .       .       .       .       .
Vv01g0955       Tc02_g034010    .       .       .       Gorai.
008G182500.1    .       .       .       .       .       .       .
        .       .       .       .       .       .       .       .
Vv01g0956       .       .       .       .       Gorai.008G182400.1
        .       .       .       .       .       .       .       .
        .       .       .       .       .       .       .
Vv01g0957       .       .       .       .       .       .       .
        .       .       .       .       .       .       .       .
        .       .       .       .       .
Vv01g0958       .       Gorai.013G077500.1      .       .       .
        .       .       .       .       .       .       .       .
        .       .       .       .       .       .       .
Vv01g0959       Tc02_g033990    Gorai.013G077400.1      .       .
        .       .       .       .       .       .       .       .
        .       .       .       .       .       .       .       .
Vv01g0960       .       .       .       .       .       .       .
        .       .       .       .       .       .       .       .
        .       .       .       .       .
```

Vv01g0961 Tc02_g033950 Gorai.013G074800.1 . .
Gorai.008G191200.1 Gorai.012G117800.1 Vv17g0634
Tc03_g009820 . . . Gorai.008G191200.1
. Vv14g1562 . . . . .
.
Vv01g0962 Tc02_g033940 Gorai.013G082400.1 . .
. . . . . . . .
. . . . . . . .
Vv01g0963 Tc02_g033930 Gorai.013G082500.1 . .
. . . . . . . .
. . . . . . . .
Vv01g0964 Tc02_g033920 . . . Gorai.
008G191300.1 . Vv17g0636 . . . .
Gorai.008G182300.1 . Vv14g1564
Tc05_g023640 . . . . .
Vv01g0965 Tc02_g033910 . . . . .
. . . . . . . .
. . . . . .
Vv01g0966 Tc02_g033900 Gorai.013G082700.1 .
Gorai.009G323400.1 . . Vv17g0638
Tc03_g009840 . . . . . Vv14g1565
Tc04_g017560 . . . . .
Vv01g0967 . . . . . . .
. . . . . . . .
. . . . .
Vv01g0968 . . . . . . .
. . . . . . . .
. . . . .
Vv01g0969 . . . . . . .
. . . . . . . .
. . . . .
Vv01g0970 . . . . . . .
. . . . . . . .
. . . . .
Vv01g0971 . . . . . . .
. . . . . . . .
. . . . .
Vv01g0972 . . . . . . .
. . . . . . . .
. . . . .
Vv01g0973 . . . . . . .
. . . . . . . .
. . . . .
Vv01g0974 . . . . . . .
. . . . . . . .
. . . . .
Vv01g0975 . . . . . . .
. . . . . . . .
. . . . .
Vv01g0976 . . . . . . .

. . . . . . . .
. . . . .
Vv01g0977 . . . . Gorai.008G179500.1
. . . . . . . .
. . . . . . .
Vv01g0978 . . . . . . .
. . . . . . . .
. . . . .
Vv01g0979 . . . . . . .
. . . . . . . .
. . . . .
Vv01g0980 . . . . . . .
. . . . . . . .
. . . . .
Vv01g0981 . . . . . . .
. . . . . . . .
. . . . .
Vv01g0982 . . . . . . .
. . . . . . . .
. . . . .
Vv01g0983 . . . . . . .
. . . . . . . .
. . . . .
Vv01g0984 . . . . . . .
. . . . . . . .
. . . . .
Vv01g0985 . . . . . . .
. . . . . . . .
. . . . .
Vv01g0986 . . . . . . .
. . . . . . . .
. . . . .
Vv01g0987 . . . . . . .
. . . . . . . .
. . . . .
Vv01g0988 . . . . . . .
. . . . . . . .
. . . . .
Vv01g0989 . . . . . . .
. . . . . . . .
. . . . .
Vv01g0990 . . . . . . .
. . . . . . . .
. . . . .
Vv01g0991 . . . . . . .
. . . . . . . .
. . . . .
Vv01g0992 . . . . . . .
. . . . . . . .
. . . . .

Vv01g0993 . . . . . . .
. . . . . . . .
. . . . .
Vv01g0994 . . . . . . .
. . . . . . . .
. . . . .
Vv01g0995 . . . . . . .
. . . . . . . .
. . . . .
Vv01g0996 . . . . . . .
. . . . . . . .
. . . . .
Vv01g0997 . . . . . . .
. . . . . . . .
. . . . .
Vv01g0998 . . . . . . .
. . . . . . . .
. . . . .
Vv01g0999 . . . . . . .
. . . . . . . .
. . . . .
Vv01g1000 . . . . . . .
. . . . . . . .
. . . . .
Vv01g1001 . . . . . . .
. . . . . . . .
. . . . .
Vv01g1002 . . . . . . .
. . . . . . . .
. . . . .
Vv01g1003 . . . . . . .
. . . . . . . .
. . . . .
Vv01g1004 . . . . . . .
. . . . . . . .
. . . . .
Vv01g1005 . . . . . . .
. . . . . . . .
. . . . .
Vv01g1006 . . . . . . .
. . . . . . . .
. . . . .
Vv01g1007 . . . . . . .
. . . . . . . .
. . . . .
Vv01g1008 . . . . . . .
. . . . . . . .
. . . . .
Vv01g1009 . . . . . . .
. . . . . . . .

. . . . .
Vv01g1010 Tc02_g030270 . Gorai.005G151100.1 .
. . . . . . . .
. . . . . . . .
Vv01g1011 . . . . . . .
. . . . . . . .
. . . . .
Vv01g1012 . . . . . . .
. . . . . . . .
. . . . .
Vv01g1013 . . . . . . .
. . . . . . . .
. . . . .
Vv01g1014 . . . . . . .
. . . . . . . .
. . . . .
Vv01g1015 . . . . . . .
. . . . . . . .
. . . . .
Vv01g1016 . . . . . . .
. . . . . . . .
. . . . .
Vv01g1017 . . . . . . .
. . . . . . . .
. . . . .
Vv01g1018 . . . . . . .
. . . . . . . .
. . . . .
Vv01g1019 . . . . . . .
. . . . . . . .
. . . . .
Vv01g1020 . . . . . . .
. . . . . . . .
. . . . .
Vv01g1021 . . . . . . .
. . . . . . . .
. . . . .
Vv01g1022 . . . . . . .
. . . . . . . .
. . . . .
Vv01g1023 . . . . . . .
. . . . . . . .
. . . . .
Vv01g1024 . . . . . . .
. . . . . . . .
. . . . .
Vv01g1025 . . . . . . .
. . . . . . . .
. . . . .
Vv01g1026 . . . . . . .

```
        .      .      .      .      .      .      .      .
        .      .      .      .      .
Vv01g1027      .      .      .      .      .      .      .
        .      .      .      .      .      .      .      .
        .      .      .      .      .
Vv01g1028      .      .      .      .      .      .      .
        .      .      .      .      .      .      .      .
        .      .      .      .      .
Vv01g1029      .      .      .      .      .      .      .
        .      .      .      .      .      .      .      .
        .      .      .      .      .
Vv01g1030      .      .      .      .      .      .      .
        .      .      .      .      .      .      .      .
        .      .      .      .      .
Vv01g1031      .      .      .      .      .      .      .
        .      .      .      .      .      .      .      .
        .      .      .      .      .
Vv01g1032      .      Gorai.013G090800.1      .      .      .
        .      .      .      .      .      .      .      .
        .      .      .      .      .      .      .
Vv01g1033      .      .      .      .      .      .      .
        .      .      .      .      .      .      .      .
        .      .      .      .      .
Vv01g1034      Tc02_g032570    Gorai.013G090700.1      .      .
        .      .      .      .      .      .      .      .
        .      .      .      .      .      .      .      .
Vv01g1035      .      .      .      .      .      .      .
        .      .      .      .      .      .      .      .
        .      .      .      .      .
Vv01g1036      Tc02_g032580    Gorai.013G090600.1      .      .
        .      .      .      .      .      .      .      .
        .      .      .      .      .      .      .      .
Vv01g1037      Tc02_g032590    Gorai.013G090500.1      .      .
        .      .      .      .      .      .      .      .
        .      .      .      .      .      .      .      .
Vv01g1038      .      .      .      .      .      .      .
        .      .      .      .      .      .      .      .
        .      .      .      .      .
Vv01g1039      Tc02_g030050    .      .      Gorai.009G328300.1
        .      .      .      .      .      .      .      .
        .      .      .      .      .      .      .      .
Vv01g1040      .      .      .      .      .      .      .
        .      .      .      .      .      .      .      .
        .      .      .      .      .
Vv01g1041      Tc02_g031320    Gorai.013G088700.1      .
Gorai.009G327300.1    .      .      .      .      .      .
        .      .      .      .      .      .      .      .
        .      .
Vv01g1042      .      .      .      .      .      .      .
        .      .      .      .      .      .      .      .
```

. . . . .
Vv01g1043 . . . . Gorai.008G175200.1
. . . . . . . .
. . . . . . .
Vv01g1044 . . . . . . .
. . . . . . . .
. . . . .
Vv01g1045 . . . . . . .
. . . . . . . .
. . . . .
Vv01g1046 . . . . . . .
. . . . . . . .
. . . . .
Vv01g1047 . . Gorai.005G147500.1 . .
. . . . . . . .
. . . . . . .
Vv01g1048 . . . . . . .
. . . . . . . .
. . . . .
Vv01g1049 . . . . . . .
. . . . . . . .
. . . . .
Vv01g1050 . . . . . . .
. . . . . . . .
. . . . .
Vv01g1051 . . . . . . .
. . . . . . . .
. . . . .
Vv01g1052 . . . . . . .
. . . . . . . .
. . . . .
Vv01g1053 Tc02_g031310 Gorai.013G098100.1 . .
Gorai.008G175000.1 . . . . .
. . . . . . . .
. .
Vv01g1054 Tc02_g031300 . . . Gorai.
008G174900.1 . . . . . . .
. . . . . . . .
Vv01g1055 . . . . . . .
. . . . . . . .
. . . . .
Vv01g1056 Tc02_g012240 . . . . .
. . . . . . . .
. . . . . .
Vv01g1057 . . . . . . .
. . . . . . . .
. . . . .
Vv01g1058 Tc02_g031290 . Gorai.005G147900.1 .
. . . . . . . .
. . . . . . . .

Vv01g1059 . . . . . . .
. . . . . . . .
. . . . .
Vv01g1060 . . . . . . .
. . . . . . . .
. . . . .
Vv01g1061 Tc02_g031260 . Gorai.005G148000.1 .
. . . . . . . .
. . . . . . . .
Vv01g1062 . . . . . . .
. . . . . . . .
. . . . .
Vv01g1063 . . . . . . .
. . . . . . . .
. . . . .
Vv01g1064 Tc02_g031250 Gorai.013G098300.1 Gorai.
005G148100.1 . . . . . . .
. . . . . . . .
. .
Vv01g1065 . . . . . . .
. . . . . . . .
. . . . .
Vv01g1066 . . . . Gorai.008G174700.1
. . . . . . . .
. . . . . . .
Vv01g1067 . . . . . . .
. . . . . . . .
. . . . .
Vv01g1068 Tc02_g026750 Gorai.013G098400.1 .
Gorai.009G332900.1 Gorai.008G174600.1 . . .
. . . . . . . .
. . . .
Vv01g1069 . . Gorai.005G148200.1 . .
. . . . . . . .
. . . . . . .
Vv01g1070 Tc02_g012570 . . . . .
. . . . . . . .
. . . . . .
Vv01g1071 . Gorai.013G098600.1 Gorai.005G148300.1
. . . . . . . .
. . . . . . . .
.
Vv01g1072 . Gorai.013G098800.1 . . .
. . . . . . . .
. . . . . . .
Vv01g1073 . . . . . . .
. . . . . . . .
. . . . .
Vv01g1074 . . . . . . .
. . . . . . . .

. . . . .
Vv01g1075 . . . . . . .
. . . . . . . .
. . . . .
Vv01g1076 . . . . . . .
. . . . . . . .
. . . . .
Vv01g1077 . . . . . . .
. . . . . . . .
. . . . .
Vv01g1078 . . . . . . .
. . . . . . . .
. . . . .
Vv01g1079 . . . . . . .
. . . . . . . .
. . . . .
Vv01g1080 . . . . . . .
. . . . . . . .
. . . . .
Vv01g1081 Tc02_g031110 . Gorai.005G148400.1 .
. . . . . . . .
. . . . . . . .
Vv01g1082 . . . . . . .
. . . . . . . .
. . . . .
Vv01g1083 Tc02_g031100 . . . . .
. . . . . . . .
. . . . . .
Vv01g1084 . . . . . . .
. . . . . . . .
. . . . .
Vv01g1085 . . . . . . .
. . . . . . . .
. . . . .
Vv01g1086 . . . . . . .
. . . . . . . .
. . . . .
Vv01g1087 . . . . . . .
. . . . . . . .
. . . . .
Vv01g1088 Tc02_g031080 . Gorai.005G148600.1 .
Gorai.008G174500.1 . . . . .
. . . . . . . .
. .
Vv01g1089 . . . . . . .
. . . . . . . .
. . . . .
Vv01g1090 . . . . . . .
. . . . . . . .
. . . . .

Vv01g1091 . . . . . . .
. . . . . . . .
. . . . .
Vv01g1092 Tc02_g030740 Gorai.013G102300.1 .
Gorai.009G327500.1 . . . . . .
. . . . . . . .
. .
Vv01g1093 . . . . . . .
. . . . . . . .
. . . . .
Vv01g1094 Tc02_g030430 . . . . .
. . . . . . . .
. . . . . .
Vv01g1095 . . . . . . .
. . . . . . . .
. . . . .
Vv01g1096 . . . . . . .
. . . . . . . .
. . . . .
Vv01g1097 . . . . . . .
. . . . . . . .
. . . . .
Vv01g1098 . . Gorai.005G149100.1 . .
. . . . . . . .
. . . . . . .
Vv01g1099 Tc02_g007110 . . Gorai.002G005900.1
. . . . . . . .
. . . . . . . .
Vv01g1100 . . . . . . .
. . . . . . . .
. . . . .
Vv01g1101 . . . . . . .
. . . . . . . .
. . . . .
Vv01g1102 . . . . . . .
. . . . . . . .
. . . . .
Vv01g1103 . . . . . . .
. . . . . . . .
. . . . .
Vv01g1104 . . . . . . .
. . . . . . . .
. . . . .
Vv01g1105 Tc02_g027210 . . . . .
. . . . . . . .
. . . . . .
Vv01g1106 Tc02_g027230 . . . . .
. . . . . . . .
. . . . . .
Vv01g1107 . . . . . . .

| | | | | | | | |
|---|---|---|---|---|---|---|---|
| . | . | . | . | . | . | . | . |
| . | . | . | . | . | | | |
| Vv01g1108 | Tc02_g030420 | | . | Gorai.005G150600.1 | | | . |
| . | . | . | . | . | . | . | . |
| . | . | . | . | . | . | . | . |
| Vv01g1109 | . | . | . | . | . | . | . |
| . | . | . | . | . | . | . | . |
| . | . | . | . | . | | | |
| Vv01g1110 | . | . | . | . | Gorai.008G174300.1 | | |
| . | . | . | . | . | . | . | . |
| . | . | . | . | . | . | . | |
| Vv01g1111 | . | . | . | . | . | . | . |
| . | . | . | . | . | . | . | . |
| . | . | . | . | . | | | |
| Vv01g1112 | . | . | . | . | . | . | . |
| . | . | . | . | . | . | . | . |
| . | . | . | . | . | | | |
| Vv01g1113 | . | . | . | . | . | . | . |
| . | . | . | . | . | . | . | . |
| . | . | . | . | . | | | |
| Vv01g1114 | . | . | . | . | . | . | . |
| . | . | . | . | . | . | . | . |
| . | . | . | . | . | | | |
| Vv01g1115 | . | . | . | . | . | . | . |
| . | . | . | . | . | . | . | . |
| . | . | . | . | . | | | |
| Vv01g1116 | . | . | . | . | . | . | . |
| . | . | . | . | . | . | . | . |
| . | . | . | . | . | | | |
| Vv01g1117 | Tc02_g030380 | | . | . | Gorai.009G328100.1 | | |
| . | . | . | . | . | . | . | . |
| . | . | . | . | . | . | . | . |
| Vv01g1118 | . | . | . | . | . | . | . |
| . | . | . | . | . | . | . | . |
| . | . | . | . | . | | | |
| Vv01g1119 | . | . | . | . | . | . | . |
| . | . | . | . | . | . | . | . |
| . | . | . | . | . | | | |
| Vv01g1120 | . | . | . | . | . | . | . |
| . | . | . | . | . | . | . | . |
| . | . | . | . | . | | | |
| Vv01g1121 | . | . | . | . | . | . | . |
| . | . | . | . | . | . | . | . |
| . | . | . | . | . | | | |
| Vv01g1122 | Tc02_g030350 | | . | Gorai.005G150900.1 | | | . |
| . | . | . | . | . | . | . | . |
| . | . | . | . | . | . | . | . |
| Vv01g1123 | . | . | . | . | . | . | . |
| . | . | . | . | . | . | . | . |
| . | . | . | . | . | | | |

Vv01g1124 Tc02_g028780 . . . . .
. . . . . . . .
. . . . . .
Vv01g1125 Tc02_g028790 Gorai.002G167400.1 . .
. . . . . . . .
. . . . . . . .
Vv01g1126 . . . . . . .
. . . . . . . .
. . . . .
Vv01g1127 . Gorai.002G167300.1 . . .
. . . . . . . .
. . . . . . .
Vv01g1128 . . . . . . .
. . . . . . . .
. . . . .
Vv01g1129 . . . . . . .
. . . . . . . .
. . . . .
Vv01g1130 . . . . . . .
. . . . . . . .
. . . . .
Vv01g1131 Tc02_g003670 . . . . .
. . . . . . . .
. . . . . .
Vv01g1132 . . . . . . .
. . . . . . . .
. . . . .
Vv01g1133 . . . . . . .
. . . . . . . .
. . . . .
Vv01g1134 . . . . . . .
. . . . . . . .
. . . . .
Vv01g1135 . . . . . . .
. . . . . . . .
. . . . .
Vv01g1136 Tc02_g006690 . . . . .
. . . . . . . .
. . . . . .
Vv01g1137 . . . . . . .
. . . . . . . .
. . . . .
Vv01g1138 . . . . . . .
. . . . . . . .
. . . . .
Vv01g1139 . . . . . . .
. . . . . . . .
. . . . .
Vv01g1140 . . . . . . .
. . . . . . . .

. . . . .
Vv01g1141 . . . . . . .
. . . . . . . .
. . . . .
Vv01g1142 . . . . . . .
. . . . . . . .
. . . . .
Vv01g1143 . Gorai.002G167200.1 . . .
. . . . . . . .
. . . . . . .
Vv01g1144 . Gorai.002G167000.1 . . .
. . . . . . . .
. . . . . . .
Vv01g1145 . . . . . . .
. . . . . . . .
. . . . .
Vv01g1146 . . . . . . .
. . . . . . . .
. . . . .
Vv01g1147 . . . . . . .
. . . . . . . .
. . . . .
Vv01g1148 Tc02_g030330 . . . Gorai.
008G172000.1 . . . . . . .
. . . . . . . .
Vv01g1149 . . . . . . .
. . . . . . . .
. . . . .
Vv01g1150 . Gorai.002G164000.1 . . .
. . . . . . . .
. . . . . . .
Vv01g1151 Tc02_g028820 . . . . .
. . . . . . . .
. . . . . .
Vv01g1152 Tc02_g003490 Gorai.013G028800.1 . .
. . . . . . . .
. . . . . . . .
Vv01g1153 . Gorai.013G028700.1 Gorai.005G152100.1
. Gorai.008G171000.1 . . . .
. . . . . . . .
. . .
Vv01g1154 . . . . . . .
. . . . . . . .
. . . . .
Vv01g1155 . . . . . . .
. . . . . . . .
. . . . .
Vv01g1156 . . . . . . .
. . . . . . . .
. . . . .

```
Vv01g1157        .       .        .       .        .       .
Vv17g0108        Tc03_g012600     .       .        .       .        .
        .        .       .        .       .        .       .
Vv01g1158        .       .        .       .        .       .        .
        .        .       .        .       .        .       .        .
        .        .       .        .       .
Vv01g1159        .       .        .       .        .       .        .
        .        .       .        .       .        .       .        .
        .        .       .        .       .
Vv01g1160        .       .        .       .        .       .        .
        .        .       .        .       .        .       .        .
        .        .       .        .       .
Vv01g1161        .       .        .       .        .       .        .
        .        .       .        .       .        .       .        .
        .        .       .        .       .
Vv01g1162        .       .        .       .        .       .        .
        .        .       .        .       .        .       .        .
        .        .       .        .       .
Vv01g1163        Tc02_g019490     .       .        .       .        .
        .        .       .        .       .        .       .        .
        .        .       .        .       .        .
Vv01g1164        .       .        .       .        .       .        .
        .        .       .        .       .        .       .        .
        .        .       .        .       .
Vv01g1165        Tc02_g030230     Gorai.013G028500.1       .        .
        Gorai.008G168000.1        .       .        .       .        .
        .        .       .        .       .        .       .        .
        .        .
Vv01g1166        .       .        .       .        .       .        .
        .        .       .        .       .        .       .        .
        .        .       .        .       .
Vv01g1167        .       .        .       .        .       .        .
        .        .       .        .       .        .       .        .
        .        .       .        .       .
Vv01g1168        .       .        .       .        .       .        .
        .        .       .        .       .        .       .        .
        .        .       .        .       .
Vv01g1169        Tc02_g019380     .       .        .       .        .
        .        .       .        .       .        .       .        .
        .        .       .        .       .        .
Vv01g1170        .       .        .       .        .       .        .
        .        .       .        .       .        .       .        .
        .        .       .        .       .
Vv01g1171        .       Gorai.013G028400.1        .       .        .
        .        .       .        .       .        .       .        .
        .        .       .        .       .        .       .
Vv01g1172        .       .        .       .        .       .        .
        .        .       .        .       .        .       .        .
        .        .       .        .       .
Vv01g1173        .       .        .       .        .       .        .
```

. . . . . . . .
. . . . .
Vv01g1174 Tc02_g006310 . Gorai.005G154500.1
Gorai.002G003900.1 . . . . . .
. . . . . . . .
. .
Vv01g1175 . Gorai.013G028100.1 . . .
. . . . . . . .
. . . . . . .
Vv01g1176 Tc02_g028860 Gorai.013G027500.1 . .
. . . . . . . .
. . . . . . . .
Vv01g1177 . . . . . . .
. . . . . . . .
. . . . .
Vv01g1178 . . . . . . .
. . . . . . . .
. . . . .
Vv01g1179 Tc02_g028880 Gorai.002G167900.1 . .
. . . . . . . .
. . . . . . . .
Vv01g1180 . . . . . . .
. . . . . . . .
. . . . .
Vv01g1181 Tc02_g028890 . . . Gorai.
008G168700.1 . . . . . . .
. . . . . . . .
Vv01g1182 . . . . . . .
. . . . . . . .
. . . . .
Vv01g1183 . . . . . . .
. . . . . . . .
. . . . .
Vv01g1184 . . . . . . .
. . . . . . . .
. . . . .
Vv01g1185 . . . . . . .
. . . . . . . .
. . . . .
Vv01g1186 . . . . . . .
. . . . . . . .
. . . . .
Vv01g1187 Tc02_g028900 Gorai.002G168100.1 . .
. . . . . . . .
. . . . . . . .
Vv01g1188 Tc02_g028910 Gorai.002G168200.1 . .
. . . . . . . .
. . . . . . . .
Vv01g1189 . . . . . . .
. . . . . . . .

. . . . .
Vv01g1190 Tc02_g028930 Gorai.002G168300.1 . .
. . . . . . . .
. . . . . . . .
Vv01g1191 . . . . . . .
. . . . . . . .
. . . . .
Vv01g1192 . . . . . . .
. . . . . . . .
. . . . .
Vv01g1193 . . . . . .
Vv17g0097 Tc03_g015160 Gorai.001G057100.1 . .
Gorai.008G207300.1 Gorai.006G272200.1 . .
. . . . .
Vv01g1194 . . . . . . .
. . . . . . . .
. . . . .
Vv01g1195 . . . . . Gorai.
012G124400.1 . . . . . . .
. . . . . . .
Vv01g1196 . . . . . . .
. . . . . . . .
. . . . .
Vv01g1197 . . Gorai.005G194000.1 . .
. . . . . . . .
. . . . . . .
Vv01g1198 . . . . . . .
. . . . . . . .
. . . . .
Vv01g1199 . . . . . . .
. . . . . . . .
. . . . .
Vv01g1200 . . . . . .
Vv17g0088 Tc03_g015290 Gorai.001G056500.1 . .
. . Vv14g1122 . . .
Gorai.007G068800.1 Gorai.006G043900.1 Gorai.012G084400.1
Vv01g1201 . . . . . . .
. . . . . . . .
. . . . .
Vv01g1202 . . . . . . .
. . . . . . . .
. . . . .
Vv01g1203 . . Gorai.005G194100.1 . .
. . . . . . . .
. . . . . . .
Vv01g1204 . . . . . . .
. . . . . . . .
. . . . .
Vv01g1205 . . . . . . .
. . . . . . Vv14g1119

. . . Gorai.007G071700.1 Gorai.
006G044200.1 .
Vv01g1206 . . . . . . .
. . . . . . . .
. . . . .
Vv01g1207 . . Gorai.005G194700.1 . .
. . . . . . . .
. . . . . . .
Vv01g1208 Tc02_g018500 . . . Gorai.
008G205800.1 . . . . . . .
. . . . . . . .
Vv01g1209 . . . . . . .
. . . . . . . .
. . . . .
Vv01g1210 . . Gorai.005G194900.1 .
Gorai.008G157200.1 . . . . . .
. . . . . . . .
.
Vv01g1211 Tc02_g018390 . Gorai.005G195000.1 .
Gorai.008G156900.1 Gorai.012G124500.1 . .
. . . . . Vv14g1110 .
. . . . Gorai.012G084000.1
Vv01g1212 Tc02_g020470 . . Gorai.002G016600.1
. . . . . . . .
. . . . . . . .
Vv01g1213 . . . . . . .
. . . . . . . .
. . . . .
Vv01g1214 . . Gorai.005G195100.1 .
Gorai.008G209000.1 Gorai.012G124700.1 Vv17g0072
Tc03_g015460 Gorai.001G055500.1 . . Gorai.
008G209100.1 . . . . . . .
.
Vv01g1215 . . . . . . .
. . . . . . . .
. . . . .
Vv01g1216 . . . . . . .
. . . . . . . .
. . . . .
Vv01g1217 . . . . . . .
. . . . . . . .
. . . . .
Vv01g1218 . . . . . . .
. . . . . . . .
. . . . .
Vv01g1219 . . . . . . .
. . . . . . . .
. . . . .
Vv01g1220 . . Gorai.005G195600.1 .
Gorai.008G156700.1 . Vv17g0060 Tc03_g015520 .

. . Gorai.008G156700.1 . . .
. . . . .
Vv01g1221 . . . . . . .
. . . . . . . .
. . . . .
Vv01g1222 . . Gorai.005G195700.1 .
Gorai.008G156600.1 . . . . . .
. . Vv14g1101 Tc04_g004700 . .
. . Gorai.012G083500.1
Vv01g1223 . . . . . . .
. . . . . . . .
. . . . .
Vv01g1224 . . . . . . .
. . . . . . . .
. . . . .
Vv01g1225 . . Gorai.005G195800.1 . .
. . . . . . . .
Vv14g1089 Tc04_g004790 . . . .
Gorai.012G083100.1
Vv01g1226 . . . . . .
Vv17g0050 Tc03_g015570 Gorai.001G055200.1 . .
. Gorai.006G272800.1 . . . .
. . .
Vv01g1227 . . Gorai.005G195900.1 .
Gorai.008G156500.1 . . . . . .
. . . . . . . .
.
Vv01g1228 . . . . . . .
. . . . . . . .
. . . . .
Vv01g1229 . . . . . . .
. . . . . . . .
. . . . .
Vv01g1230 . . . . . . .
. . . . . . . .
. . . . .
Vv01g1231 . . . . . . .
. . . . . . . .
. . . . .
Vv01g1232 . . . . Gorai.008G156400.1
. . . . . . . .
. . . . . . .
Vv01g1233 . . Gorai.005G196000.1 . .
. . . . . . . .
Vv14g1087 Tc04_g004840 . . . .
.
Vv01g1234 . . . . . . .
. . . . . . . .
. . . . .
Vv01g1235 . . . . . . .

```
        .       .       .       .       .       .       .       .
        .       .       .       .       .
Vv01g1236       .       .       .       .       .       .       .
        .       .       .       .       .       .       .       .
        .       .       .       .       .
Vv01g1237       .       .       .       .       .       .       .
        .       .       .       .       .       .       Vv14g1084
        Tc04_g004860    .       .       Gorai.007G072400.1      .
        .
Vv01g1238       .       .       .       .       .       .       .
        .       .       .       .       .       .       .       .
        .       .       .       .       .
Vv01g1239       Tc02_g019970    .       Gorai.005G196200.1      .
        .       .       .       .       .       .       .       .
        .       .       .       .       .       .       .       .
Vv01g1240       Tc02_g019960    .       .       .       .       .
        .       .       .       .       .       .       .       .
        .       .       .       .       .       .
Vv01g1241       Tc02_g019950    .       Gorai.005G196300.1      .
        Gorai.008G156200.1      .       .       .       .       .
        .       .       .       .       .       .       .       .
        .       .
Vv01g1242       .       .       .       .       Gorai.008G209700.1
        .       Vv17g0047       Tc03_g015600    Gorai.001G055000.1
        .       Gorai.007G044300.1      Gorai.008G209700.1
Gorai.006G272900.1      .       .       .       .       .       .
        .
Vv01g1243       .       .       .       .       .       .       .
        .       .       .       .       .       .       .       .
        .       .       .       .       .
Vv01g1244       Tc02_g019910    .       .       .       Gorai.
008G209900.1    .       .       .       .       .       .       .
        .       .       .       .       .       .       .       .
Vv01g1245       .       .       .       .       .       .       .
        .       .       .       .       .       .       .       .
        .       .       .       .       .
Vv01g1246       .       .       .       .       Gorai.008G210000.1
        .       .       .       .       .       .       .       .
        .       .       .       .       .       .       .
Vv01g1247       .       .       .       .       .       .       .
        .       .       .       .       .       .       .       .
        .       .       .       .       .
Vv01g1248       Tc02_g019780    .       .       .       .       .
        .       .       .       .       .       .       .       .
        .       .       .       .       .       .
Vv01g1249       Tc02_g019760    .       .       .       Gorai.
008G210100.1    .       Vv17g0039       Tc03_g015670    .       .
        Gorai.007G044600.1      Gorai.008G210100.1      .       .
        .       .       .       .       .       .
Vv01g1250       Tc02_g023750    .       .       Gorai.009G337800.1
```

. . . . . . . .
. . . . . . . .
Vv01g1251 Tc02_g019750 . Gorai.005G196500.1 .
. . Vv17g0038 Tc03_g015680 . .
Gorai.007G044700.1 . . . . .
. . . .
Vv01g1252 Tc02_g019740 . Gorai.005G196600.1 .
. . . . . . . .
. . . . . . . .
Vv01g1253 Tc02_g019710 . . . . .
. . . . . . . .
. . . . . .
Vv01g1254 . . . . . . .
. . . . . . . .
. . . . .
Vv01g1255 Tc02_g019700 . . . . .
. . . . . . .
Vv14g1069 Tc04_g005100 . Gorai.001G275700.1 .
Gorai.006G000400.1 .
Vv01g1256 Tc02_g019690 . . . Gorai.
008G210200.1 . . . . . . .
. . . . . . . .
Vv01g1257 . . . . . .
Vv17g0033 Tc03_g015720 Gorai.001G054600.1 .
Gorai.007G044900.1 . . . . . .
. . .
Vv01g1258 . . . . . . .
. . . . . . . .
. . . . .
Vv01g1259 . . . . . Gorai.
012G125000.1 . . . . . . .
Vv14g1066 Tc04_g005150 . . Gorai.
007G072700.1 . .
Vv01g1260 . . . . . . .
. . . . . . . .
. . . . .
Vv01g1261 Tc02_g024160 . . Gorai.009G337000.1
. . . . . . . .
. . . . . . . .
Vv01g1262 . . Gorai.005G196700.1 . .
. . . . . . . .
. . . . . . .
Vv01g1263 . . Gorai.005G196800.1 . .
. . . . . . . .
. . . . . . .
Vv01g1264 . . . . . .
Vv17g0026 Tc03_g015810 . . . . .
. . . . . . .
Vv01g1265 Tc02_g024380 . . . Gorai.
008G156100.1 . . . . . . .

```
         .       .        .       .        .       .        .       .
Vv01g1266        Tc02_g002960     .        Gorai.005G196900.1       .
         .       .        .       .        .       .        .       .
         .       Vv14g1058        Tc05_g025990     .        .       .
         .       .
Vv01g1267        .        .       .        .       .        .       .
         .       .        .       .        .       .        .       .
         .       .        .       .        .
Vv01g1268        .        .       .        .       Gorai.008G210400.1
         .       .        .       .        .       .        .       .
         .       .        .       .        .       .        .
Vv01g1269        .        .       Gorai.005G197000.1        .       .
         .       .        .       .        .       .        .       .
         .       .        .       .        .       .        .
Vv01g1270        .        .       .        .       .        .       .
         .       .        .       .        .       .        .       .
         .       .        .       .        .
Vv01g1271        .        .       .        .       .        .       .
         .       .        .       .        .       .        .       .
         .       .        .       .        .
Vv01g1272        .        .       .        .       .        .       .
         .       .        .       .        .       .        .       .
         .       .        .       .        .
Vv01g1273        Tc02_g024890     .        .       .        .       .
         .       .        .       .        .       .        .       .
         .       .        .       .        .       .
Vv01g1274        .        .       .        .       .        .       .
         .       .        .       .        .       .        .       .
         .       .        .       .        .
Vv01g1275        .        .       .        .       Gorai.008G210800.1
         .       .        .       .        .       .        .       .
         .       .        .       .        .       .        .
Vv01g1276        .        .       .        .       .        Gorai.
012G125800.1     Vv17g0017        Tc03_g015940     .        .       .
         .       .        .       .        .       .        .       .
         .
Vv01g1277        .        .       .        .       .        .       .
         .       .        .       .        .       .        Vv14g1054
         Tc04_g005260     .       .        Gorai.007G072800.1       .
         .
Vv01g1278        .        .       .        .       .        .
Vv17g0012        Tc03_g015970     Gorai.001G053800.1        .       .
         .       .        .       .        .       .        .       .
         .
Vv01g1279        .        .       .        .       .        .       .
         .       .        .       .        .       .        .       .
         .       .        .       .        .
Vv01g1280        .        .       .        .       Gorai.008G156000.1
         .       Vv17g0011        Tc03_g015980     .        .       .
         Gorai.008G156000.1       .        .       .        .       .
```

```
                    .       .       .
Vv01g1281           .       .       .       .       .       Gorai.
012G125900.1        .       .       .       .       .       .       .
                    .       .       .       .       .       .       .
Vv01g1282           .       .       .       .       .       Gorai.
012G126000.1        .       .       .       .       .       .       .
                    .       .       .       .       .       .       .
Vv01g1283           Tc02_g025270    .       .       .       Gorai.
008G155900.1        .       .       .       .       .       .       .
                    .       .       .       .       .       .       .
Vv01g1284           Tc02_g018910    .       .       .       .       .
                    .       .       .       .       .       .       .       .
                    .       .       .       .       .       .
Vv01g1285           .       .       .       .       .       .       .
                    .       .       .       .       .       .       .       .
                    .       .       .       .       .
Vv01g1286           Tc02_g018890    .       .       .       .       .
                    .       .       .       .       .       .       .       .
                    .       .       .       .       .       .
Vv01g1287           Tc02_g018880    .       .       .       .
Gorai.012G126100.1          .       .       .       .       .       .
                    .       .       .       .       .       .       .       .
Vv01g1288           .       .       .       .       .       .       .
                    .       .       .       .       .       .       .       .
                    .       .       .       .       .
Vv01g1289           Tc02_g018870    .       Gorai.005G197200.1      .
        Gorai.008G211200.1          .       .       .       .       .
                    .       .       .       .       .       .       .       .
                    .       .
Vv01g1290           .       .       .       .       .       .       .
                    .       .       .       .       .       .       .       .
                    .       .       .       .       .
Vv01g1291           Tc02_g018860    .       .       Gorai.002G021000.1
                    .       .       .       .       .       .       .       .
                    .       .       .       .       .       .       .       .
Vv01g1292           .       .       Gorai.005G197700.1      .       .
        Gorai.012G126200.1          .       .       .       .       .
                    .       .       .       .       .       .       .       .
                    .
Vv01g1293           .       .       .       .       .       .
Vv14g1041           Tc04_g005410    .       .       .       .       .
                    .       .       .       .       .       .       .
Vv01g1294           .       .       .       .       Gorai.008G155700.1
                    .       Vv14g1040       Tc04_g005460    .       .       .
                    .       .       .       .       .       .       .       .
                    .
Vv01g1295           .       .       Gorai.005G197900.1      .       .
                    .       .       .       .       .       .       .       .
                    .       .       .       .       .       .       .
Vv01g1296           Tc02_g002710    .       .       .       Gorai.
```

008G152800.1 Gorai.012G126300.1 . . . .
. . . . . . . .
. .
Vv01g1297 . . . . . . .
. . . . . . . .
. . . . .
Vv01g1298 . . . . . .
Vv14g1024 Tc04_g005560 . . . . .
. . . . . . .
Vv01g1299 . . . . . . .
. . . . . . . .
. . . . .
Vv01g1300 . . . . . . .
. . . . . . . .
. . . . .
Vv01g1301 . . . . Gorai.008G211600.1
. . . . . . . .
. . . . . . .
Vv01g1302 . . . . . . .
. . . . . . . .
. . . . .
Vv01g1303 . . . . . . .
. . . . . . . .
. . . . .
Vv01g1304 . . . . . . .
. . . . . . . .
. . . . .
Vv01g1305 . . . . Gorai.008G212100.1
. . . . . . . .
. . . . . . .
Vv01g1306 . . . . . . .
. . . . . . . .
. . . . .
Vv01g1307 . . . . . . .
. . . . . . . .
. . . . .
Vv01g1308 . . . . . . .
. . . . . . . .
. . . . .
Vv01g1309 . . . . . . .
. . . . . . . .
. . . . .
Vv01g1310 . . . . . . .
. . . . . . . .
. . . . .
Vv01g1311 . . . . . .
Vv14g1017 . Gorai.011G088800.1 Gorai.001G276400.1
Gorai.007G074000.1 Gorai.006G038900.1 . .
. . . . . .
Vv01g1312 . . . . . . .

| | | | | | | | |
|---|---|---|---|---|---|---|---|
| . | . | . | . | . | . | . | . |
| . | . | . | . | . | | | |
| Vv01g1313 | . | . | . | . | . | . | . |
| . | . | . | . | . | . | . | . |
| . | . | . | . | . | | | |
| Vv01g1314 | . | . | . | . | . | . | . |
| . | . | . | . | . | . | . | . |
| . | . | . | . | . | | | |
| Vv01g1315 | . | . | . | . | . | . | . |
| . | . | . | . | . | . | . | . |
| . | . | . | . | . | | | |
| Vv01g1316 | . | . | . | . | . | . | . |
| . | . | . | . | . | . | . | . |
| . | . | . | . | . | | | |
| Vv01g1317 | . | . | . | . | . | . | . |
| . | . | . | . | . | . | . | . |
| . | . | . | . | . | | | |
| Vv01g1318 | . | . | . | . | . | . | . |
| . | . | . | . | . | . | . | . |
| . | . | . | . | . | | | |
| Vv01g1319 | . | . | . | . | . | . | . |
| . | . | . | . | . | . | . | . |
| . | . | . | . | . | | | |
| Vv01g1320 | . | . | . | . | . | . | . |
| . | . | . | . | . | . | . | . |
| . | . | . | . | . | | | |
| Vv01g1321 | . | . | . | . | . | . | . |
| . | . | . | . | . | . | . | . |
| . | . | . | . | . | | | |
| Vv01g1322 | . | . | . | . | . | . | . |
| . | . | . | . | . | . | . | . |
| . | . | . | . | . | | | |
| Vv01g1323 | . | . | . | . | . | . | . |
| . | . | . | . | . | . | . | . |
| . | . | . | . | . | | | |
| Vv01g1324 | . | . | . | . | . | . | . |
| . | . | . | . | . | . | . | . |
| . | . | . | . | . | | | |
| Vv01g1325 | . | . | . | . | . | . | . |
| . | . | . | . | . | . | . | . |
| . | . | . | . | . | | | |
| Vv01g1326 | . | . | . | . | . | . | . |
| . | . | . | . | . | . | . | . |
| . | . | . | . | . | | | |
| Vv01g1327 | . | . | . | . | . | . | . |
| . | . | . | . | . | . | . | . |
| . | . | . | . | . | | | |
| Vv02g0001 | . | . | . | . | . | . | . |
| . | . | . | . | . | . | . | . |
| . | . | . | . | . | | | |

Vv02g0002 . . . . . . .
. . . . . . . .
. . . . .
Vv02g0003 . . . Gorai.007G101000.1 .
. . . . . . . .
. . . . . . .
Vv02g0004 . . . . . . .
. . . . . . . .
. . . . .
Vv02g0005 . . Gorai.003G157300.1 Gorai.
007G023700.1 Gorai.008G248000.1 . Vv015rg0193 .
. . . . . . . .
. . . .
Vv02g0006 Tc03_g027670 . Gorai.003G157100.1 .
Gorai.008G248200.1 Gorai.006G209100.1 . .
. . . . . . . .
. . . .
Vv02g0007 Tc03_g027680 . . . Gorai.
008G248300.1 . . . . . . .
. . . . . . . .
Vv02g0008 Tc03_g027730 . Gorai.003G157000.1 .
. . . . . . . .
. . . . . . . .
Vv02g0009 Tc03_g027740 . Gorai.003G156900.1
Gorai.007G023800.1 . . . . . .
. . . . . . . .
. .
Vv02g0010 Tc03_g027750 . . Gorai.007G023900.1
Gorai.008G248400.1 Gorai.006G209000.1 . .
. . . . . . . .
. . . .
Vv02g0011 Tc03_g027760 Gorai.004G139700.1 Gorai.
003G156800.1 . Gorai.008G248500.1 . . .
. . . . . . . .
. . . .
Vv02g0012 . . . . . . .
. . . . . . . .
. . . . .
Vv02g0013 . . . . . . .
. . . . . . . .
. . . . .
Vv02g0014 . . . . . . .
. . . . . . . .
. . . . .
Vv02g0015 Tc03_g027810 Gorai.004G139400.1 Gorai.
003G156100.1 . Gorai.008G248700.1 Gorai.006G208800.1
. . . . . . . .
. . . . . .
Vv02g0016 . . . . . . .
. . . . . . . .

. . . . .
Vv02g0017 Tc03_g027820 . . . Gorai.
008G248800.1 . . . . . . .
. . . . . . . .
Vv02g0018 Tc03_g027830 Gorai.004G139300.1 Gorai.
003G156000.1 . Gorai.008G248900.1 . . .
. . . . . Vv08g1047
Tc05_g003660 . Gorai.002G255800.1 . Gorai.
012G108300.1 .
Vv02g0019 Tc03_g027840 Gorai.004G139200.1 Gorai.
003G155900.1 Gorai.007G102100.1 Gorai.008G249300.1 .
. . . . . . .
Vv08g1008 Tc05_g004020 . . . . .
Vv02g0020 Tc03_g027850 . . . Gorai.
008G249400.1 . . . . . . .
. . . . . . . .
Vv02g0021 . . . . . . .
. . . . . . . .
. . . . .
Vv02g0022 Tc03_g027870 . . . Gorai.
008G274800.1 . . . . . . .
. . . . . . . .
Vv02g0023 Tc03_g027890 Gorai.004G139000.1 . .
. . . . . . . .
. . . . . . . .
Vv02g0024 Tc03_g027900 Gorai.004G138900.1 Gorai.
003G155800.1 Gorai.007G012900.1 Gorai.008G274700.1 .
. . . . . . . .
. . . . . .
Vv02g0025 Tc03_g027910 . Gorai.003G155700.1 .
. . . . . . . .
. . . . . . . .
Vv02g0026 . . . . . . .
. . . . . . . .
. . . . .
Vv02g0027 . . . . . . .
. . . . . . . .
. . . . .
Vv02g0028 Tc03_g027920 Gorai.004G138600.1 Gorai.
003G155600.1 . Gorai.008G274600.1 . . .
. . . . . . . .
. . . .
Vv02g0029 . . . . . . .
. . . . . . . .
. . . . .
Vv02g0030 Tc03_g027930 Gorai.004G138500.1 . .
. . . . . . . .
. . . . . . . .
Vv02g0031 Tc03_g027940 Gorai.004G138400.1 Gorai.
003G155500.1 . . Gorai.006G208700.1 . .

. . . . . . . .
. . . .
Vv02g0032 . . . . . . .
. . . . . . . .
. . . . .
Vv02g0033 Tc03_g027950 . Gorai.003G155300.1 .
Gorai.008G249900.1 . . . . .
. . . . . . . .
. .
Vv02g0034 Tc03_g027960 . . . Gorai.
008G250000.1 . . . . . . .
. . . . . . . .
Vv02g0035 Tc03_g027970 Gorai.004G138000.1 Gorai.
003G154200.1 . . Gorai.006G208600.1 Vv015rg0199
. . . . . . . .
. . . . .
Vv02g0036 . . . . . . .
. . . . . . . .
. . . . .
Vv02g0037 Tc03_g027980 Gorai.004G137900.1 Gorai.
003G085900.1 Gorai.007G024400.1 Gorai.008G250100.1 .
Vv015rg0200 . . . . . .
. . . . . . .
Vv02g0038 Tc03_g027990 Gorai.004G137800.1 Gorai.
003G085800.1 . . . . . . .
. . . . . . . .
. .
Vv02g0039 Tc03_g028000 Gorai.004G137700.1 Gorai.
003G085700.1 . . . . . . .
. . . . . . . .
. .
Vv02g0040 Tc03_g028010 . Gorai.003G085600.1 .
Gorai.008G250200.1 . . . . .
. . . . . . . .
. .
Vv02g0041 Tc03_g028020 Gorai.004G137600.1 . .
. . . . . . . .
. . . . . . . .
Vv02g0042 . . . . . . .
. . . . . . . .
. . . . .
Vv02g0043 Tc03_g028030 . Gorai.003G085500.1
Gorai.007G013300.1 Gorai.008G273700.1 . Vv015rg0207
. . . . . . Vv08g0980
Tc05_g004380 Gorai.006G171700.1 . .
Gorai.012G104800.1 .
Vv02g0044 Tc03_g028040 . . . Gorai.
008G250400.1 . . . . . . .
. . . . . . . .
Vv02g0045 Tc03_g028050 . Gorai.003G085400.1 .

. . . . . . . .
. . . . . . . .
Vv02g0046 . . . . Gorai.008G273400.1
. . . . . . . .
. . . . . . .
Vv02g0047 . . . . . . .
. . . . . . . .
. . . . .
Vv02g0048 . Gorai.004G137500.1 . . .
. Vv015rg0210 . . . . .
. . . . . . . .
Vv02g0049 Tc03_g028070 . Gorai.003G085300.1 .
. . . . . . . .
. . . . . . . .
Vv02g0050 Tc03_g028080 . . Gorai.007G024600.1
. . . . . . . .
. . . . . . . .
Vv02g0051 Tc03_g028090 Gorai.004G137400.1 Gorai.
003G085200.1 Gorai.007G024700.1 . . . .
. . . . . . . .
. . . .
Vv02g0052 . . . . . . .
. . . . . . . .
. . . . .
Vv02g0053 Tc03_g028110 Gorai.004G137300.1 Gorai.
003G085100.1 . Gorai.008G250500.1 . Vv015rg0231
. . . . . . . .
. . . . .
Vv02g0054 Tc03_g028180 . Gorai.003G084800.1 .
Gorai.008G273000.1 . . . . .
. . . . . . . .
. .
Vv02g0055 . . . . . . .
. . . . . . . .
. . . . .
Vv02g0056 . . . . . . .
. . . . . . . .
. . . . .
Vv02g0057 . . . . . . .
. . . . . . . .
. . . . .
Vv02g0058 . . . . . . .
. . . . . . . .
. . . . .
Vv02g0059 . . . . . . .
. . . . . . . .
. . . . .
Vv02g0060 . . . . . . .
. . . . . . . .
. . . . .

Vv02g0061 . . . . . . .
. . . . . . . .
. . . . .
Vv02g0062 . . . . . . .
. . . . . . . .
. . . . .
Vv02g0063 Tc03_g028190 Gorai.004G137000.1 Gorai.
003G084400.1 Gorai.007G013600.1 Gorai.008G272900.1
Gorai.006G208500.1 Vv015rg0246 . . . .
. . . . . . . .
.
Vv02g0064 Tc03_g028200 . Gorai.003G084300.1 .
. Gorai.006G208400.1 . . . .
. . . . . . . .
. .
Vv02g0065 Tc03_g028210 Gorai.004G136900.1 Gorai.
003G084200.1 Gorai.007G024900.1 . . . .
. . . . . . . .
. . . .
Vv02g0066 Tc03_g028220 . Gorai.003G084100.1 .
. . . . . . . .
. . . . . . . .
Vv02g0067 Tc03_g028230 . Gorai.003G084000.1
Gorai.007G025000.1 . . . . . .
. . . . . . . .
. .
Vv02g0068 Tc03_g028240 Gorai.004G136700.1 Gorai.
003G083800.1 . . . . . . .
. . . Vv08g0941 Tc05_g004820 .
Gorai.002G258600.1 . . .
Vv02g0069 Tc03_g028250 . Gorai.003G083700.1 .
. Gorai.006G208300.1 . . . .
. . . . . . . .
. .
Vv02g0070 . . . . Gorai.008G251200.1
. . . . . . . .
. . . . . . .
Vv02g0071 Tc03_g028260 . . . . .
. . . . . . . .
. . . . . .
Vv02g0072 Tc03_g028270 Gorai.004G136600.1 Gorai.
003G083600.1 . . . . . . .
. . . . . . . .
. .
Vv02g0073 Tc03_g028280 . . . . .
. . . . . . . .
. . . . . .
Vv02g0074 Tc03_g028290 . Gorai.003G083500.1 .
. . . . . . . .
. . . . . . . .

Vv02g0075 . . . . . . .
. . . . . . Vv08g0933
Tc05_g004900 . . . . .
Vv02g0076 Tc03_g028310 . Gorai.003G083300.1 .
. . . . . . . .
. Vv08g0932 Tc05_g004910 . Gorai.
002G255100.1 . Gorai.012G102300.1 .
Vv02g0077 . . . . . . .
. . . . . . Vv08g0930
Tc05_g004920 . . . .
Vv02g0078 Tc03_g028320 . Gorai.003G083100.1 .
. . . . . . . .
. . . . . . . .
Vv02g0079 . . Gorai.003G082900.1 . .
. . . . . . . .
. . . . . . .
Vv02g0080 . . . Gorai.007G104600.1 .
. Vv015rg0268 . . . . .
. . . . . . . .
Vv02g0081 Tc03_g028330 Gorai.004G136500.1 .
Gorai.007G104700.1 Gorai.008G251400.1 . Vv015rg0269
. . . . . . . .
. . . . .
Vv02g0082 Tc03_g028340 . . . Gorai.
008G251500.1 . . . . . . .
. . . . . . . .
Vv02g0083 Tc03_g028350 . . . Gorai.
008G251600.1 . . . . . . .
. . . . . . . .
Vv02g0084 . . . . . . .
. . . . . . . .
. . . . .
Vv02g0085 Tc03_g028360 . . Gorai.007G025100.1
Gorai.008G251700.1 . Vv015rg0271 . .
. . . . . . . .
. . .
Vv02g0086 . . . . . . .
. . . . . . . .
. . . . .
Vv02g0087 Tc03_g028380 . . . Gorai.
008G251800.1 . . . . . . .
. . . . . . . .
Vv02g0088 . . . . . . .
. . . . . . . .
. . . . .
Vv02g0089 . . . . . . .
. . . . . . . .
. . . . .
Vv02g0090 Tc03_g028390 . Gorai.003G082700.1 .
. . . . . . . .

| | | | | | | | | |
|---|---|---|---|---|---|---|---|---|
| | . | . | . | . | . | . | . | . |
| Vv02g0091 | | . | . | . | . | . | . | . |
| | . | . | . | . | . | . | . | . |
| | . | . | . | . | . | | | |
| Vv02g0092 | | . | . | . | . | . | . | . |
| | . | . | . | . | . | . | . | . |
| | . | . | . | . | . | | | |
| Vv02g0093 | | Tc03_g028400 | | . | . | . | . | . |
| | . | . | . | . | . | . | . | . |
| | . | . | . | . | . | . | | |
| Vv02g0094 | | . | . | . | . | . | . | . |
| | . | . | . | . | . | . | . | . |
| | . | . | . | . | . | | | |
| Vv02g0095 | | . | . | . | . | . | . | . |
| | . | . | . | . | . | . | . | . |
| | . | . | . | . | . | | | |
| Vv02g0096 | | . | . | . | . | . | . | . |
| | . | . | . | . | . | . | . | . |
| | . | . | . | . | . | | | |
| Vv02g0097 | | . | . | . | . | . | . | . |
| | . | . | . | . | . | . | . | . |
| | . | . | . | . | . | | | |
| Vv02g0098 | | . | . | . | . | . | . | . |
| | . | . | . | . | . | . | . | . |
| | . | . | . | . | . | | | |
| Vv02g0099 | | . | . | . | . | . | . | . |
| | . | . | . | . | . | . | . | . |
| | . | . | . | . | . | | | |
| Vv02g0100 | | . | . | . | . | . | . | . |
| | . | . | . | . | . | . | . | . |
| | . | . | . | . | . | | | |
| Vv02g0101 | | . | . | . | . | . | . | . |
| | . | . | . | . | . | . | . | . |
| | . | . | . | . | . | | | |
| Vv02g0102 | | . | . | . | . | . | . | . |
| | . | . | . | . | . | . | . | . |
| | . | . | . | . | . | | | |
| Vv02g0103 | | . | . | . | . | . | . | . |
| | . | . | . | . | . | . | . | . |
| | . | . | . | . | . | | | |
| Vv02g0104 | | . | . | . | . | . | . | . |
| | . | . | . | . | . | . | . | . |
| | . | . | . | . | . | | | |
| Vv02g0105 | | . | . | . | Gorai.007G014300.1 | | | . |
| | . | . | . | . | . | . | . | . |
| | . | . | . | . | . | . | . | |
| Vv02g0106 | | . | . | . | . | . | . | . |
| | . | . | . | . | . | . | . | . |
| | . | . | . | . | . | | | |
| Vv02g0107 | | Tc03_g028410 | | Gorai.004G136000.1 | | | . | |

Gorai.007G105200.1 Gorai.008G252000.1 . Vv015rg0277
. . . . . . . .
. . . . .
Vv02g0108 Tc03_g028420 . Gorai.003G082600.1 .
. . . . . . . .
. . . . . . . .
Vv02g0109 Tc03_g028430 Gorai.004G135900.1 Gorai.
003G082500.1 . Gorai.008G252200.1 . Vv015rg0280
. . . . . . . .
. . . . .
Vv02g0110 Tc03_g028450 Gorai.004G135700.1 Gorai.
003G082200.1 Gorai.007G105700.1 Gorai.008G271200.1
Gorai.006G208200.1 . . . . . .
. . . . . . . .
Vv02g0111 Tc03_g028460 . . . . .
. . . . . . . .
. . . . . .
Vv02g0112 . . . . . . .
. . . . . . . .
. . . . .
Vv02g0113 Tc03_g028480 . . . Gorai.
008G252500.1 . Vv015rg0285 . . . .
. . . . . . . .
.
Vv02g0114 Tc03_g028500 Gorai.004G135500.1 .
Gorai.007G106200.1 . Gorai.006G208100.1 . .
. . . . . . . .
. . . .
Vv02g0115 Tc03_g028510 . . Gorai.007G106300.1
Gorai.008G252600.1 . . . . .
. . . . . . . .
. .
Vv02g0116 Tc03_g028520 . Gorai.003G082000.1 .
. . . . . . . .
. . . . . . . .
Vv02g0117 Tc03_g028530 . . Gorai.007G025400.1
. . . . . . . .
. . . . . . . .
Vv02g0118 . . . . . . .
. . . . . . . .
. . . . .
Vv02g0119 . . . . . . .
. . . . . . . .
. . . . .
Vv02g0120 Tc03_g028550 Gorai.004G135400.1 Gorai.
003G081700.1 . . Gorai.006G207900.1 . .
. . . . . . . .
. . . .
Vv02g0121 Tc03_g028560 Gorai.004G135200.1 .
Gorai.007G106400.1 Gorai.008G252700.1 . . .

. . . . . . . .
. . . .
Vv02g0122 Tc03_g028580 . Gorai.003G081600.1
Gorai.007G106800.1 . . Vv015rg0292 . .
. . . . . . . .
. . .
Vv02g0123 Tc03_g028590 Gorai.004G135000.1 Gorai.
003G081500.1 . . . Vv015rg0294 . .
. . . . . . . .
. . .
Vv02g0124 . . . . . . .
. . . . . . . .
. . . . .
Vv02g0125 Tc03_g028600 . . Gorai.007G025500.1
. . . . . . . .
. . . . . . . .
Vv02g0126 Tc03_g028620 . Gorai.003G081400.1 .
Gorai.008G252900.1 . . . . .
. . . . . . . .
. .
Vv02g0127 Tc03_g028630 Gorai.004G134900.1 Gorai.
003G081300.1 . Gorai.008G253000.1 . . .
. . . . . . . .
. . . .
Vv02g0128 Tc03_g028650 . . . . .
. . . . . . . .
. . . . . .
Vv02g0129 Tc03_g028670 . . . Gorai.
008G253100.1 . . . . . . .
. . . . . . . .
Vv02g0130 Tc03_g028680 Gorai.004G134800.1 .
Gorai.007G107200.1 Gorai.008G253200.1 . . .
. . . . . . . .
. . . .
Vv02g0131 Tc03_g028690 Gorai.004G134700.1 . .
. . . . . . . .
. . . . . . . .
Vv02g0132 Tc03_g028700 Gorai.004G134600.1 .
Gorai.007G014600.1 Gorai.008G270600.1 . Vv015rg0302
. . . . . . . .
. . . . .
Vv02g0133 Tc03_g028780 . . Gorai.007G025600.1
. . . . . . . .
. . . . . . . .
Vv02g0134 Tc03_g028790 . . Gorai.007G025800.1
Gorai.008G253500.1 . . . . .
. . . . . . . .
. .
Vv02g0135 Tc03_g028800 Gorai.004G134300.1 . .
. . . . . . . .

. . . . . . . .
Vv02g0136 . . . . . . .
. . . . . . . .
. . . . .
Vv02g0137 Tc03_g028820 Gorai.004G134000.1 .
Gorai.007G026000.1 . . . . . .
. . . . . . . .
. .
Vv02g0138 Tc03_g028830 Gorai.004G133900.1 .
Gorai.007G026100.1 Gorai.008G270200.1 . Vv015rg0310
. . . . . . . .
. . . . .
Vv02g0139 Tc03_g028840 . . . Gorai.
008G253800.1 . . . . . . .
. . . . . . . .
Vv02g0140 Tc03_g028850 Gorai.004G133800.1 .
Gorai.007G026200.1 Gorai.008G253900.1 Gorai.006G207800.1
. . . . . . . .
. . . . . .
Vv02g0141 Tc03_g028860 . Gorai.003G080900.1 .
. Gorai.006G207700.1 . . . .
. . . . . . . .
. .
Vv02g0142 . . . . . . .
. . . . . . . .
. . . . .
Vv02g0143 Tc03_g028870 Gorai.004G133700.1 Gorai.
003G080800.1 . Gorai.008G254000.1 . . .
. . . . . . . .
. . . .
Vv02g0144 Tc03_g028880 . Gorai.003G080700.1 .
Gorai.008G270000.1 . . . . .
. . . . . . . .
. .
Vv02g0145 Tc03_g028890 . Gorai.003G080600.1 .
. . . . . . . .
. . . . . . . .
Vv02g0146 Tc03_g028900 . Gorai.003G080500.1
Gorai.007G026300.1 Gorai.008G254100.1 . . .
. . . . . . . .
. . . .
Vv02g0147 Tc03_g028910 . Gorai.003G080300.1
Gorai.007G026600.1 Gorai.008G254200.1 . . .
. . . . . . . .
. . . .
Vv02g0148 Tc03_g028930 Gorai.004G133400.1 Gorai.
003G080000.1 Gorai.007G014700.1 Gorai.008G254300.1 .
. . . . . . . .
. . . . . .
Vv02g0149 Tc03_g028960 . . . Gorai.

008G254400.1 . . . . . . .
. . . . . . . .
Vv02g0150 . . . . . . .
. . . . . . . .
. . . . .
Vv02g0151 Tc03_g028990 . . . Gorai.
008G254600.1 . . . . . . .
. . . . . . . .
Vv02g0152 Tc03_g029000 . . . . .
. . . . . . . .
. . . . . .
Vv02g0153 . . . Gorai.007G015000.1 .
. . . . . . . .
. . . . . . .
Vv02g0154 Tc03_g029010 Gorai.004G132800.1 . .
Gorai.008G254700.1 . . . . .
. . . . . . . .
. .
Vv02g0155 . . . . . . .
. . . . . . . .
. . . . .
Vv02g0156 Tc03_g029020 . . . Gorai.
008G254800.1 Gorai.006G207600.1 . . . .
. . . . . . . .
. .
Vv02g0157 . . . . . . .
. . . . . . . .
. . . . .
Vv02g0158 . . . . . . .
. . . . . . . .
. . . . .
Vv02g0159 Tc03_g029040 . . . Gorai.
008G255000.1 . . . . . . .
. . . . . . . .
Vv02g0160 . . . . . . .
. . . . . . . .
. . . . .
Vv02g0161 . . . . . . .
. . . . . . . .
. . . . .
Vv02g0162 Tc03_g029050 Gorai.004G132700.1 . .
. Gorai.006G207400.1 . . . .
. . . . . . . .
. .
Vv02g0163 Tc03_g029060 Gorai.004G132600.1 . .
Gorai.008G255100.1 Gorai.006G207300.1 Vv0Unrg2991
. . . . . . . .
. . . . .
Vv02g0164 . . Gorai.003G078800.1 . .
. . . . . . . .

. . . . . . .
Vv02g0165 Tc03_g029100 Gorai.004G132500.1 . .
Gorai.008G269100.1 . . . . .
. . . . . . . .
. .
Vv02g0166 Tc03_g029120 . Gorai.003G078700.1 .
Gorai.008G266400.1 . . . . .
. . . . . . . .
. .
Vv02g0167 Tc03_g029130 Gorai.004G132400.1 Gorai.
003G078600.1 Gorai.007G108300.1 Gorai.008G255600.1 .
Vv0Unrg2994 . . . . . .
Vv16g0644 . . . Gorai.007G108300.1
Gorai.008G255200.1 .
Vv02g0168 Tc03_g029140 . . . .
Gorai.006G207200.1 . . . . . .
. . . . . . . .
Vv02g0169 Tc03_g029150 . . Gorai.007G015400.1
Gorai.008G255800.1 Gorai.006G207100.1 . .
. . . . . . . .
. . . .
Vv02g0170 Tc03_g029160 . . . Gorai.
008G255900.1 . . . . . . .
. . . . . . . .
Vv02g0171 Tc03_g012780 Gorai.004G132300.1 . .
. . . . . . . .
. . . . . . . .
Vv02g0172 Tc03_g029170 . . . . .
. . . . . . . .
. . . . . .
Vv02g0173 . . . Gorai.007G108500.1 .
. Vv0Unrg2997 . . . . .
. Vv16g0639 Tc03_g020910 . .
Gorai.007G108500.1 . .
Vv02g0174 Tc03_g029190 . Gorai.003G078500.1 .
. . . . . . . .
. . . . . . . .
Vv02g0175 Tc03_g029200 . Gorai.003G078400.1 .
Gorai.008G256000.1 Gorai.006G207000.1 . .
. . . . . . . .
. . . .
Vv02g0176 Tc03_g029210 . . . Gorai.
008G256100.1 . . . . . . .
. . . . . . . .
Vv02g0177 Tc03_g029220 . Gorai.003G078300.1
Gorai.007G109200.1 . . . . . .
. . . . . . . .
. .
Vv02g0178 . . . . . . .
. . . . . . . .

```
         .       .        .       .        .
Vv02g0179        Tc03_g029260     Gorai.004G131900.1       .       .
        Gorai.008G256200.1        .        .       .        .       .
         .       .        .       .        .       .        .       .
         .       .
Vv02g0180        .        .       .        .       .        .       .
         .       .        .       .        .       .        .       .
         .       .        .       .        .
Vv02g0181        .       Gorai.004G131800.1       .        .       .
         .       .        .       .        .       .        .       .
         .       .        .       .        .       .        .
Vv02g0182        Tc03_g029270     .        .       .        .       .
         .       .        .       .        .       .        .       .
         .       .        .       .        .       .
Vv02g0183        Tc03_g029280     .        Gorai.003G078000.1
Gorai.007G026900.1        .       .        Vv0Unrg3009      .       .
         .       .        .       .        .       .        .       .
         .       .        .
Vv02g0184        .        .       .        .       .        .       .
         .       .        .       .        .       .        .       .
         .       .        .       .        .
Vv02g0185        Tc03_g029290     .        .       Gorai.007G027000.1
        Gorai.008G256300.1        .        .       .        .       .
         .       .        .       .        .       .        .       .
         .       .
Vv02g0186        .        .       .        .       .        .       .
         .       .        .       .        .       .        .       .
         .       .        .       .        .
Vv02g0187        .        .       .        .       .        .       .
         .       .        .       .        .       .        .       .
         .       .        .       .        .
Vv02g0188        Tc03_g029300     .        .       .        .
Gorai.006G206800.1        .       .        .       .        .       .
         .       .        .       .        .       .        .       .
Vv02g0189        Tc03_g029310     .        .       .        .       .
         .       .        .       .        .       .        .       .
         .       .        .       .        .       .
Vv02g0190        .        .       .        .       .        .       .
         .       .        .       .        .       .        .       .
         .       .        .       .        .
Vv02g0191        .        .       .        .       .        .       .
         .       .        .       .        .       .        .       .
         .       .        .       .        .
Vv02g0192        Tc03_g029320     .        .       .        .
Gorai.006G206700.1        .       .        .       .        .       .
         .       .        .       .        .       .        .       .
Vv02g0193        .        .       .        .       .        .       .
         .       .        .       .        .       .        .       .
         .       .        .       .        .
Vv02g0194        Tc03_g029330     .        .       .        .       .
```

. . . . . . . .
. . . . . .
Vv02g0195 . . . . . . .
. . . . . . . .
. . . . .
Vv02g0196 Tc03_g029340 . . . .
Gorai.006G206600.1 . . . . . .
. . . . . . . .
Vv02g0197 . . . . . . .
. . . . . . . .
. . . . .
Vv02g0198 Tc03_g012860 . Gorai.003G104000.1 .
. . . . . . . .
. . . . . . . .
Vv02g0199 . . . . . . .
. . . . . . . .
. . . . .
Vv02g0200 . . . . . . .
. . . . . . . .
. . . . .
Vv02g0201 . . . . . . .
. . . . . . . .
. . . . .
Vv02g0202 . . . . . . .
. . . . . . . .
. . . . .
Vv02g0203 . . . . . . .
. . . . . . . .
. . . . .
Vv02g0204 . . . . . . .
. . . . . . . .
. . . . .
Vv02g0205 . . . . . . .
. . . . . . . .
. . . . .
Vv02g0206 . . . . . . .
. . . . . . . .
. . . . .
Vv02g0207 Tc03_g029350 . . . Gorai.
008G256400.1 . . . . . . .
. . . . . . . .
Vv02g0208 . . . . . . .
. . . . . . . .
. . . . .
Vv02g0209 . . . . . . .
. . . . . . . .
. . . . .
Vv02g0210 . . . . . . .
. . . . . . . .
. . . . .

Vv02g0211 . . . . . . .
. . . . . . . .
. . . . .
Vv02g0212 Tc03_g029370 Gorai.004G131700.1 . .
. Gorai.006G206500.1 . . . .
. . . . . . . .
. .
Vv02g0213 . . . . . . .
. . . . . . Vv16g0627
Tc03_g021060 . Gorai.003G121600.1 . .
.
Vv02g0214 Tc03_g029390 Gorai.004G131600.1 Gorai.
003G119400.1 . Gorai.008G256600.1 . . .
. . . . . . . .
. . . .
Vv02g0215 Tc03_g029400 . . Gorai.007G110100.1
. Gorai.006G206300.1 Vv0Unrg3017 . .
. . . . Vv16g0621
Tc03_g021120 . . . . .
Vv02g0216 Tc03_g029420 . . . . .
. . . . . . . .
. . . . . .
Vv02g0217 Tc03_g029430 Gorai.004G131400.1 .
Gorai.007G020600.1 . . . . . .
. . . Vv16g0613 Tc03_g021180 .
. . . .
Vv02g0218 Tc03_g029440 Gorai.004G131300.1 . .
. . . . . . . .
. . . . . . . .
Vv02g0219 . . . . . . .
. . . . . . . .
. . . . .
Vv02g0220 Tc03_g029500 Gorai.004G131200.1 . .
. . . . . . . .
. . . . . . . .
Vv02g0221 . . . . . . .
. . . . . . . .
. . . . .
Vv02g0222 Tc03_g029590 . . Gorai.007G020500.1
. . . . . . . .
. . . . . . . .
Vv02g0223 Tc03_g029600 Gorai.004G131000.1 .
Gorai.007G020400.1 . . . . . .
. . . . . . . .
. .
Vv02g0224 . . . . Gorai.008G256700.1
. . . . . . . .
. . . . . . .
Vv02g0225 Tc03_g029670 Gorai.004G130900.1 . .
. . . . . . . .

```
        .       .       .       .       .       .       .       .
Vv02g0226       .       .       .       .       .       .       .
        .       .       .       .       .       .       .       .
        .       .       .       .       .
Vv02g0227       .       .       Gorai.003G122000.1      Gorai.
007G020200.1    .       .       .       .       .       .       .
        .       .       .       .       .       .       .       .
        .
Vv02g0228       .       .       .       .       .       .       .
        .       .       .       .       .       .       .       .
        .       .       .       .       .
Vv02g0229       Tc03_g012980    .       Gorai.003G076400.1      .
        Gorai.008G256800.1      Gorai.006G206100.1      Vv0Unrg3026
        .       .       .       .       .       .       Vv16g0603
        Tc03_g021260    .       .       .       Gorai.008G267000.1
        .
Vv02g0230       .       Gorai.004G130600.1      .       .
Gorai.008G256900.1      .       Vv0Unrg3027     .       .       .
        .       .       .       .       .       .       .       .
        .       .
Vv02g0231       .       .       .       .       Gorai.008G257000.1
        .       .       .       .       .       .       .       .
        .       .       .       .       .       .       .
Vv02g0232       .       .       .       .       .       .       .
        .       .       .       .       .       .       .       .
        .       .       .       .       .
Vv02g0233       .       Gorai.004G130500.1      Gorai.003G076000.1
        .       .       .       .       .       .       .       .
        .       .       .       .       .       .       .       .
        .
Vv02g0234       .       Gorai.004G130400.1      Gorai.003G075900.1
        .       .       .       Vv0Unrg3028     .       .       .
        .       .       .       .       .       .       .       .
        .       .
Vv02g0235       .       .       .       .       Gorai.008G257100.1
        .       .       .       .       .       .       .       .
        .       .       .       .       .       .       .
Vv02g0236       .       .       .       .       .       .       .
        .       .       .       .       .       .       .       .
        .       .       .       .       .
Vv02g0237       .       .       .       .       .       .       .
        .       .       .       .       .       .       .       .
        .       .       .       .       .
Vv02g0238       .       .       Gorai.003G075800.1      .
Gorai.008G257200.1      .       .       .       .       .       .
        .       .       .       .       .       .       .       .
        .
Vv02g0239       .       .       .       .       .       .       .
        .       .       .       .       .       .       .       .
        .       .       .       .       .
```

```
Vv02g0240       .       .       .       .       .       .       .
        .       .       .       .       .       .       .       .
        .       .       .       .       .
Vv02g0241       .       Gorai.004G130300.1      Gorai.003G122200.1
        .       Gorai.008G257400.1      .       Vv0Unrg3035     .
        .       .       .       .       .       Vv16g0599
Tc03_g021300    .       Gorai.003G122200.1      Gorai.007G016100.1
        Gorai.008G257400.1      .
Vv02g0242       .       Gorai.004G130200.1      .       .       .
        .       .       .       .       .       .       .       .
        Vv16g0597       .       .       .       Gorai.007G016300.1
        .       .
Vv02g0243       .       .       .       .       Gorai.008G257500.1
        .       .       .       .       .       .       .       .
        .       .       .       .       .       .       .
Vv02g0244       .       .       Gorai.003G075500.1      .       .
        .       Vv0Unrg3068     .       .       .       .       .
        .       .       .       .       .       .       .       .
Vv02g0245       .       Gorai.004G130100.1      .       .
Gorai.008G266400.1      .       Vv16g0591       Tc03_g021390    .
        .       Gorai.007G016500.1      Gorai.008G266400.1      .
        .       .       .       .       .       .       .
Vv02g0246       .       .       Gorai.003G075400.1      .       .
        .       .       .       .       .       .       .       .
        .       .       .       .       .       .       .
Vv02g0247       .       .       .       .       Gorai.008G257700.1
        Gorai.006G206000.1      .       .       .       .       .
        .       .       .       .       .       .       .       .
        .
Vv02g0248       .       .       .       .       Gorai.008G257800.1
        .       .       .       .       .       .       .       .
        .       .       .       .       .       .       .
Vv02g0249       Tc03_g025990    .       Gorai.003G072400.1      .
        .       .       .       .       .       .       .       .
        .       .       .       .       .       .       .       .
Vv02g0250       .       .       .       .       .       .       .
        .       .       .       .       .       .       .       .
        .       .       .       .       .
Vv02g0251       Tc03_g029770    .       .       .       .       .
        .       .       .       .       .       .       .       .
        .       .       .       .       .       .
Vv02g0252       .       .       .       .       .       .       .
        .       .       .       .       .       .       .       .
        .       .       .       .       .
Vv02g0253       .       Gorai.004G129900.1      .       .
Gorai.008G258200.1      .       .       .       .       .       .
        .       .       .       .       .       .       .       .
        .
Vv02g0254       .       .       Gorai.003G122700.1      .       .
        .       Vv16g0585       Tc03_g021450    .       Gorai.
```

003G122700.1 . . . . . . .
. . .
Vv02g0255 . . . . . . .
. . . . . . . .
. . . . .
Vv02g0256 . . . . . . .
. . . . . . . .
. . . . .
Vv02g0257 . Gorai.004G129800.1 . . .
Gorai.006G205900.1 . . . . .
. . . . . . . .
.
Vv02g0258 . . . Gorai.007G020000.1
Gorai.008G258300.1 . Vv16g0582 Tc03_g021470 .
. Gorai.007G016600.1 Gorai.008G258300.1 .
. . . . . . .
Vv02g0259 . . . Gorai.007G019900.1 .
. . . . . . . .
. . . . . . .
Vv02g0260 . . . Gorai.007G019800.1 .
. . . . . . . .
. . . . . . .
Vv02g0261 . . . . Gorai.008G258500.1
Gorai.006G205700.1 . . . . .
. . . . . . . .
.
Vv02g0262 Tc03_g026270 . . . . .
. . . . . . . .
. . . . . .
Vv02g0263 . . . . Gorai.008G258800.1
. . . . . . . .
. . . . . . .
Vv02g0264 Tc03_g029890 . . Gorai.007G111400.1
Gorai.008G258900.1 . Vv15g0552
Tc01_g033980 . . . . . Vv16g0578
Tc03_g021520 . . Gorai.007G111400.1
Gorai.008G258900.1 .
Vv02g0265 . . . . . Gorai.
006G205600.1 . . . . . . .
. . . . . . .
Vv02g0266 . . . . . . .
. . . . . . . .
. . . . .
Vv02g0267 Tc03_g015710 Gorai.004G129600.1 .
Gorai.007G111800.1 Gorai.008G259000.1 . . .
. . . . . . . .
. . . .
Vv02g0268 . . . Gorai.007G019700.1
Gorai.008G259100.1 Gorai.006G205500.1 . . .
. . . . . . . .

.	.	.
Vv02g0269	.	.	.	.	.	.	.
.	.	.	.	.	.	.	.
.	.	.	.	.
Vv02g0270	.	.	.	.	.	.	.
.	.	.	.	.	.	.	.
.	.	.	.	.
Vv02g0271	.	.	.	.	.	.	.
.	.	.	.	.	.	.	.
.	.	.	.	.
Vv02g0272	.	.	.	Gorai.007G019200.1
Gorai.008G259300.1	.	.	.	.	.	.
.	.	.	.	.	.	.	.
.
Vv02g0273	.	.	.	.	Gorai.008G259400.1
Gorai.006G205400.1	.	.	.	.	.
.	.	.	.	.	.	.	.
.
Vv02g0274	.	.	.	.	.	.	.
.	.	.	.	.	.	.	.
.	.	.	.	.
Vv02g0275	.	.	.	Gorai.007G019100.1
Gorai.008G259600.1	.	Vv15g0531	Tc01_g033790	.
.	.	.	.	Vv16g0567
Tc03_g021610	.	.	.	.	.
Vv02g0276	.	Gorai.004G129300.1	.	.	.
.	.	.	.	.	.	.	.
.	.	.	.	.	.	.
Vv02g0277	Tc03_g015490	.	.	Gorai.007G043900.1
.	.	.	.	.	.	.	.
.	.	.	.	.	.	.	.
Vv02g0278	.	Gorai.004G129100.1	.	Gorai.
007G112600.1	.	Gorai.006G205200.1	Vv15g0527
Tc01_g033750	.	.	.	.	.	.	.
.	.	.	.	.
Vv02g0279	.	.	.	.	.	.	.
.	.	.	.	.	.	.	.
.	.	.	.	.
Vv02g0280	.	Gorai.004G129000.1	.	.	.
.	.	.	.	.	.	.	.
.	.	.	.	.	.	.
Vv02g0281	.	Gorai.004G128900.1	.	Gorai.
007G016900.1	.	.	.	.	.	.	.
.	.	.	.	.	.	.	.
.
Vv02g0282	.	.	.	.	.	.	.
.	.	.	.	.	.	.	.
.	.	.	.	.
Vv02g0283	.	.	.	.	Gorai.008G259800.1
.	.	.	.	.	.	.	.

. . . . . . .
Vv02g0284 . . . . . Gorai.
006G205100.1 . . . . . . .
. . . . . . .
Vv02g0285 . . . . Gorai.008G259900.1
. . . . . . . .
. . . . . . .
Vv02g0286 . . . . . . .
. . . . . . . .
. . . . .
Vv02g0287 . . . . . . .
. . . . . . Vv16g0559
Tc03_g021690 . . . . .
Vv02g0288 . . . . . Gorai.
006G205000.1 . . . . . . .
. . . . . . .
Vv02g0289 . Gorai.004G128700.1 . .
Gorai.008G260100.1 . . . . . .
. . . . . . . .
.
Vv02g0290 . . . . . . .
. . . . . . . .
. . . . .
Vv02g0291 . . . . . . .
. . . . . . . .
. . . . .
Vv02g0292 . . . . Gorai.008G265200.1
Gorai.006G204800.1 . . . . .
. . . . . . . .
.
Vv02g0293 Tc03_g017700 . Gorai.003G156200.1
Gorai.007G014300.1 Gorai.008G260300.1 Gorai.006G204700.1
. . . . . . . .
. . . . . .
Vv02g0294 . . . . . . .
. . . . . . . .
. . . . .
Vv02g0295 Tc03_g026320 . . . . .
. . . . . . . .
. . . . . .
Vv02g0296 Tc03_g026330 . . . . .
. . . . . . . .
. . . . . .
Vv02g0297 Tc03_g026340 . . . . .
. . . . . . . .
. . . . . .
Vv02g0298 . . . . . . .
. . . . . . . .
. . . . .
Vv02g0299 . . . . . . .

. . . . . . . .
. . . . .
Vv02g0300 . Gorai.004G128400.1 . . .
. Vv15g0513 . . . . .
. . . . . . . .
Vv02g0301 . . . . . . .
. . . . . . . .
. . . . .
Vv02g0302 . . . . Gorai.008G264900.1
. Vv15g0510 Tc01_g033590 . . .
. . Vv16g0545 . . . .
Gorai.008G260600.1 .
Vv02g0303 . . . . Gorai.008G260800.1
. Vv15g0506 Tc01_g033580 . . .
. . Vv16g0544 Tc03_g021850 . .
. Gorai.008G260800.1 .
Vv02g0304 Tc03_g026420 . . . . .
. . . . . . . .
. . . . . .
Vv02g0305 . . . . . . .
. . . . . . . .
. . . . .
Vv02g0306 . . . . . .
Vv15g0499 Tc01_g033530 . . . . .
. . . . . . .
Vv02g0307 . . . . . . .
. . . . . . . .
. . . . .
Vv02g0308 . Gorai.004G128200.1 . Gorai.
007G114200.1 Gorai.008G261100.1 . . . .
. . . . . . . .
. . .
Vv02g0309 Tc03_g015160 Gorai.004G128100.1 . .
Gorai.008G261300.1 Gorai.006G204300.1 Vv16g0538
Tc03_g021910 . . . Gorai.008G264000.1
. . . . . . . .
Vv02g0310 . . . . . . .
. . . . . . . .
. . . . .
Vv02g0311 Tc03_g010850 . . . . .
. . . . . . . .
. . . . . .
Vv02g0312 . . . . Gorai.008G261500.1
. . . . . . . .
. . . . . . .
Vv02g0313 . . . . . . .
. . . . . . . .
. . . . .
Vv02g0314 . . . . . . .
. . . . . . . .

```
          .       .       .       .       .
Vv02g0315         Tc03_g017330    .       .       .       .
Gorai.006G204000.1        .       .       .       .       .       .
          .       .       .       .       .       .       .       .
Vv02g0316         .       .       .       .       .       .       .
          .       .       .       .       .       .       .       .
          .       .       .       .       .
Vv02g0317         .       .       .       .       Gorai.008G262200.1
          .       .       .       .       .       .       .       .
          .       .       .       .       .       .       .
Vv02g0318         .       .       .       .       Gorai.008G262100.1
          .       Vv04g1420       Tc01_g010700    .       .       .
          .       .       .       .       .       .       .       .
          .
Vv02g0319         .       .       .       .       .       .       .
          .       .       .       .       .       .       .       .
          .       .       .       .       .
Vv02g0320         .       .       .       .       .       .       .
          .       .       .       .       .       .       .       .
          .       .       .       .       .
Vv02g0321         .       .       .       .       .       .
Vv04g1446         .       .       .       .       .       .       .
          .       .       .       .       .       .
Vv02g0322         .       .       .       .       .       .
Vv04g1448         .       .       .       .       .       .       .
          .       .       .       .       .       .
Vv02g0323         .       .       .       .       .       .       .
          .       .       .       .       .       .       .       .
          .       .       .       .       .
Vv02g0324         .       Gorai.004G127800.1      .       .
Gorai.008G261900.1        .       .       .       .       .       .
          .       .       .       .       .       .       .       .
          .
Vv02g0325         .       .       .       .       .       .       .
          .       .       .       .       .       .       .       .
          .       .       .       .       .
Vv02g0326         Tc03_g026700    .       .       .       Gorai.
008G261800.1      .       .       .       .       .       .       .
          .       .       .       .       .       .       .       .
Vv02g0327         .       .       .       .       .       .
Vv04g1449         .       .       .       .       .       .       .
          .       .       .       .       .       .
Vv02g0328         .       .       .       .       Gorai.008G261600.1
          .       .       .       .       .       .       .       .
          .       .       .       .       .       .       .
Vv02g0329         .       Gorai.004G156600.1      .       .       .
          .       .       .       .       .       .       .       .
          .       .       .       .       .       .       .
Vv02g0330         .       .       .       .       Gorai.008G262300.1
          .       .       .       .       .       .       .       .
```

. . . . . . .
Vv02g0331 . . . . Gorai.008G262400.1
. Vv08g1307 Tc05_g008470 . Gorai.
002G242000.1 Gorai.002G159600.1 . . Vv04g1456
Tc01_g010810 . . . . .
Vv02g0332 Tc03_g027840 . . Gorai.007G102100.1
Gorai.008G249300.1 Gorai.006G201000.1 . .
. . . . . . . .
. . . .
Vv02g0333 Tc03_g010630 . . . . .
. . . . . . . .
. . . . . .
Vv02g0334 . . . . . . .
. . . . . . . .
. . . . .
Vv02g0335 Tc03_g017180 Gorai.004G126900.1 . .
. . . . . . . .
. . . . . . . .
Vv02g0336 . . . . . . .
. . . . . . . .
. . . . .
Vv02g0337 Tc03_g027640 Gorai.004G213400.1 Gorai.
003G157400.1 . . . . . . .
. . . . . . . .
. .
Vv02g0338 Tc03_g027630 . Gorai.003G157500.1 .
Gorai.008G275500.1 . . . . .
. . . . . . . .
. .
Vv02g0339 Tc03_g027620 . Gorai.003G157600.1 .
Gorai.008G247800.1 . . . . .
. . . . . . . .
. .
Vv02g0340 . . . Gorai.007G011700.1 .
. . . . . . . .
. . . . . . .
Vv02g0341 Tc03_g027610 Gorai.004G213300.1 . .
. . . . . . . .
. . . . . . . .
Vv02g0342 . . . . . . .
. . . . . . . .
. . . . .
Vv02g0343 . . . . . . .
. . . . . . . .
. . . . .
Vv02g0344 . . . . Gorai.008G261500.1
. . . . . . . .
. . . . . . .
Vv02g0345 . . . . Gorai.008G258500.1
. . . . . . . .

. . . . . . .
Vv02g0346 Tc03_g027590 Gorai.004G213200.1 . .
. . . . . . . .
. . . . . . . .
Vv02g0347 Tc03_g027580 Gorai.004G213100.1 Gorai.
003G158000.1 . . . . . . .
. . . . . . . .
. .
Vv02g0348 Tc03_g027570 Gorai.004G212800.1 Gorai.
003G158100.1 Gorai.007G100000.1 . . . .
. . . . . . . .
. . . .
Vv02g0349 Tc03_g027560 Gorai.004G158800.1 Gorai.
003G158200.1 Gorai.007G011300.1 Gorai.008G276400.1 .
. . . . . . . .
. . . . . .
Vv02g0350 Tc03_g027540 . Gorai.003G158300.1 .
Gorai.008G247200.1 . . . . .
. . . . . . . .
. .
Vv02g0351 Tc03_g027530 . . . Gorai.
008G247100.1 . . . . . . .
. . . . . . . .
Vv02g0352 Tc03_g027520 Gorai.004G212300.1 Gorai.
003G158400.1 . . . . . . .
. . . Vv04g1461 Tc01_g010880
Gorai.007G063500.1 . . . .
Vv02g0353 Tc03_g027510 . . . . .
. . . . . . . .
. . . . . .
Vv02g0354 Tc03_g027500 Gorai.004G212200.1 .
Gorai.007G023500.1 . . . . . .
. . . . . . . .
. .
Vv02g0355 Tc03_g027490 . Gorai.003G158500.1 .
. . . . . . . .
. . . . . . . .
Vv02g0356 . . . . . . .
. . . . . . . .
. . . . .
Vv02g0357 Tc03_g027470 . Gorai.003G158800.1 .
Gorai.008G247000.1 . . . . .
. . . . . . . .
. .
Vv02g0358 Tc03_g027450 . . . .
Gorai.006G209300.1 . . . . . .
. . . . . . . .
Vv02g0359 Tc03_g027440 Gorai.004G212100.1 Gorai.
003G158900.1 . . . . . . .
. . . . . . . .

. .
Vv02g0360 Tc03_g027430 Gorai.004G212000.1 .
Gorai.007G023400.1 . . . . . .
. . . . . . . .
. .
Vv02g0361 Tc03_g027400 . Gorai.003G159000.1 .
. . . . . . . .
. . . . . . . .
Vv02g0362 . . . . . . .
. . . . . . . .
. . . . .
Vv02g0363 Tc03_g027390 . . . .
Gorai.006G209400.1 . . . . . .
. . . . . . . .
Vv02g0364 Tc03_g027380 Gorai.004G211800.1 Gorai.
003G159100.1 . . . . . . .
. . . . . . . .
. .
Vv02g0365 Tc03_g027370 . . . Gorai.
008G246800.1 Gorai.006G209500.1 . . . .
. . . . . . . .
. .
Vv02g0366 . . . . . . .
. . . . . . . .
. . . . .
Vv02g0367 Tc03_g027360 Gorai.004G211700.1 . .
. . . . . . . .
. . . . . . . .
Vv02g0368 . . . . . . .
. . . . . . . .
. . . . .
Vv02g0369 Tc03_g027350 Gorai.004G211600.1 . .
. . . . . . . .
. . . . . . . .
Vv02g0370 Tc03_g027340 Gorai.004G161300.1 .
Gorai.007G098600.1 Gorai.008G246700.1 . Vv08g1334
Tc05_g008170 Gorai.006G158700.1 . Gorai.
002G160000.1 Gorai.009G408800.1 . . . .
. . . .
Vv02g0371 Tc03_g027330 . . . Gorai.
008G246600.1 . . . . . . .
. . . . . . . .
Vv02g0372 Tc03_g027320 Gorai.004G211400.1 . .
Gorai.008G246400.1 . . . . .
. . . . . . . .
. .
Vv02g0373 Tc03_g027310 Gorai.004G211300.1 .
Gorai.007G007000.1 . . Vv08g1362
Tc05_g007810 Gorai.006G159600.1 Gorai.002G244600.1 .
. . . . . . . .

.
Vv02g0374 . Gorai.004G157900.1 . Gorai.
007G098200.1 . . . . . . .
. . . . . . . .
.
Vv02g0375 Tc03_g027300 Gorai.004G211100.1 .
Gorai.007G023300.1 Gorai.008G246300.1 . . .
. . . . . . . .
. . . .
Vv02g0376 . . . . . . .
. . . . . . . .
. . . . .
Vv02g0377 . . . . . . .
. . . . . . . .
. . . . .
Vv02g0378 . . . . . . .
. . . . . . . .
. . . . .
Vv02g0379 . . . . . . .
. . . . . . . .
. . . . .
Vv02g0380 . . . . . . .
. . . . . . . .
. . . . .
Vv02g0381 . . . . . . .
. . . . . . . .
. . . . .
Vv02g0382 . . . . . . .
. . . . . . . .
. . . . .
Vv02g0383 Tc03_g027280 . . . . .
. . . . . . . .
. . . . . .
Vv02g0384 . Gorai.004G210800.1 . . .
. . . . . . . .
. . . . . . .
Vv02g0385 Tc03_g027270 Gorai.004G210700.1 . .
. . . . . . . .
. . . . . . . .
Vv02g0386 Tc03_g027260 . . . Gorai.
008G246100.1 . Vv08g1368 . . . .
Gorai.009G410300.1 . . . . .
. . .
Vv02g0387 Tc03_g027250 Gorai.004G210600.1 Gorai.
003G159400.1 . . . . . . .
. . . . . . . .
. .
Vv02g0388 Tc03_g027230 . . . .
Gorai.006G209700.1 . . . . . .
. . . . . . . .

Vv02g0389 Tc03_g027220 Gorai.004G210200.1 Gorai.
003G159600.1 . . Gorai.006G209800.1 . .
. . . . . . . .
. . . .
Vv02g0390 Tc03_g027210 Gorai.004G157700.1 .
Gorai.007G097900.1 Gorai.008G277100.1 . . .
. . . . . . . .
. . . .
Vv02g0391 . Gorai.004G210100.1 . . .
. . . . . . . .
. . . . . . .
Vv02g0392 Tc03_g027190 . . . Gorai.
008G245600.1 . . . . . . .
. . . . . . . .
Vv02g0393 Tc03_g027180 . . . . .
. . . . . . . .
. . . . . .
Vv02g0394 Tc03_g027170 Gorai.004G210000.1 . .
Gorai.008G277700.1 . . . . .
. . . . . . . .
. .
Vv02g0395 . . Gorai.003G160000.1 . .
. . . . . . . .
. . . . . . .
Vv02g0396 Tc03_g027140 Gorai.004G209900.1 . .
. . . . . . . .
. . . . . . . .
Vv02g0397 . . . . . . .
. . . . . . . .
. . . . .
Vv02g0398 Tc03_g027120 . . Gorai.007G097700.1
Gorai.008G245400.1 . . . . .
. . . . . . . .
. .
Vv02g0399 Tc03_g027080 . . . . .
Vv08g1377 . . . . . .
. . . . . . .
Vv02g0400 . . . . . . .
. . . . . . . .
. . . . .
Vv02g0401 . . . . . . .
. . . . . . . .
. . . . .
Vv02g0402 . . . . . . .
. . . . . . . .
. . . . .
Vv02g0403 Tc03_g013540 . . . . .
. . . . . . . .
. . . . . .
Vv02g0404 . . . . . . .

. . . . . . . .
. . . . .
Vv02g0405 . . . . . . .
. . . . . . . .
. . . . .
Vv02g0406 . . . . . . .
. . . . . . . .
. . . . .
Vv02g0407 Tc03_g027060 Gorai.004G209400.1 . .
. . . . . . . .
. . . . . . . .
Vv02g0408 . . . . . . .
. . . . . . . .
. . . . .
Vv02g0409 . . . . . .
Vv08g1378 Tc05_g030100 . . . Gorai.
009G410800.1 . . . . . . .
.
Vv02g0410 . . . . . . .
. . . . . . . .
. . . . .
Vv02g0411 . Gorai.004G164100.1 . . .
. . . . . . . .
. . . . . . .
Vv02g0412 . . . . . . .
. . . . . . . .
. . . . .
Vv02g0413 . Gorai.004G157300.1 Gorai.003G160200.1
Gorai.007G023100.1 Gorai.008G245300.1 . .
. . . . . . . .
. . . . .
Vv02g0414 . . . . . . .
. . . . . . . .
. . . . .
Vv02g0415 . . . . . . .
. . . . . . . .
. . . . .
Vv02g0416 Tc03_g013620 . . Gorai.007G041400.1
. . . . . . . .
. . . . . . . .
Vv02g0417 . . . . . . .
. . . . . . . .
. . . . .
Vv02g0418 Tc03_g013660 . . . . .
. . . . . . . .
. . . . . .
Vv02g0419 . . . . . . .
. . . . . . . .
. . . . .
Vv02g0420 Tc03_g027050 . . . . .

. . . . . . . .
. . . . . .
Vv02g0421 . . . . . Gorai.
006G210000.1 . . . . . . .
. . . . . . .
Vv02g0422 . . . . . . .
. . . . . . . .
. . . . .
Vv02g0423 . . . . . . .
. . . . . . . .
. . . . .
Vv02g0424 . . . . . . .
. . . . . . . .
. . . . .
Vv02g0425 . . . . . . .
. . . . . . . .
. . . . .
Vv02g0426 . Gorai.004G154600.1 . . .
. . . . . . . .
. . . . . . .
Vv02g0427 . . . . . . .
. . . . . . . .
. . . . .
Vv02g0428 . . . . . . .
. . . . . . . .
. . . . .
Vv02g0429 . . . . . . .
. . . . . . . .
. . . . .
Vv02g0430 . . . . Gorai.008G279300.1
. . . . . . . .
. . . . . . .
Vv02g0431 . . . . . . .
. . . . . . . .
. . . . .
Vv02g0432 . . . . . . .
. . . . . . . .
. . . . .
Vv02g0433 . . . Gorai.007G096300.1 .
. . . . . . . .
. . . . . . .
Vv02g0434 . . Gorai.003G160900.1 . .
. . . . . . . .
. . . . . . .
Vv02g0435 . Gorai.004G208800.1 . Gorai.
007G010900.1 . . . . . . .
. . . . . . . .
.
Vv02g0436 . . . . . . .
. . . . . . . .

. . . . .
Vv02g0437 . . . . . .
Vv08g1380 Tc05_g007620 . . . . .
. . . . . . .
Vv02g0438 . . . . Gorai.008G244600.1
. . . . . . . .
. . . . . . .
Vv02g0439 . . . . . . .
. . . . . . . .
. . . . .
Vv02g0440 Tc03_g027030 . . . . .
. . . . . . . .
. . . . . .
Vv02g0441 . . . . . . .
. . . . . . . .
. . . . .
Vv02g0442 . . . . . . .
. . . . . . . .
. . . . .
Vv02g0443 . . . . . . .
. . . . . . . .
. . . . .
Vv02g0444 Tc03_g027020 . . . Gorai.
008G245000.1 . . . . . . .
. . . . . . . .
Vv02g0445 Tc03_g027010 . . . . .
. . . . . . . .
. . . . . .
Vv02g0446 Tc03_g027000 . . . Gorai.
008G244900.1 . . . . . . .
. . . . . . . .
Vv02g0447 . . . . . . .
. . . . . . . .
. . . . .
Vv02g0448 . . . . . . .
. . . . . . . .
. . . . .
Vv02g0449 Tc03_g026990 . . . . .
. . . . . . . .
. . . . . .
Vv02g0450 Tc03_g026980 . . . Gorai.
008G244800.1 . . . . . . .
. . . . . . . .
Vv02g0451 Tc03_g026960 . . . Gorai.
008G244700.1 . . . . . . .
. . . . . . . .
Vv02g0452 . . . . . .
Vv08g1409 . . . . . . .
. . . . . .
Vv02g0453 . . . Gorai.007G022600.1 .

. . . . . . . .
. . . . . . .
Vv02g0454 Tc03_g013700 . . . . .
. . . . . . . .
. . . . . .
Vv02g0455 Tc03_g013710 . . . . .
. . . . . . . .
. . . . . .
Vv02g0456 Tc03_g026760 Gorai.004G208300.1 .
Gorai.007G095600.1 Gorai.008G279800.1 Gorai.006G210100.1
. . . . . . . .
. . . . . .
Vv02g0457 Tc03_g026750 . . Gorai.007G010500.1
Gorai.008G280000.1 . . . . .
. . . . . . . .
. .
Vv02g0458 Tc03_g026740 . . Gorai.007G022300.1
. . . . . . . .
. . . . . . . .
Vv02g0459 . . . . . . .
. . . . . . . .
. . . . .
Vv02g0460 Tc03_g026720 . . . Gorai.
008G243800.1 . . . . . . .
. . . . . . . .
Vv02g0461 Tc03_g026700 Gorai.004G208000.1 Gorai.
003G162600.1 Gorai.007G010200.1 Gorai.008G243700.1
Gorai.006G197200.1 . . . . . .
. . . . . . . .
Vv02g0462 Tc03_g026690 . Gorai.003G162700.1
Gorai.007G010100.1 Gorai.008G280600.1 . . .
. . . . . . . .
. . . .
Vv02g0463 . . . . . . .
. . . . . . . .
. . . . .
Vv02g0464 . . . . . . .
. . . . . . . .
. . . . .
Vv02g0465 Tc03_g026680 Gorai.004G207700.1 . .
Gorai.008G243500.1 . Vv15g0351 . .
. . . . . . . .
. . .
Vv02g0466 . . . . . . .
. . . . . . . .
. . . . .
Vv02g0467 Tc03_g026670 . . . .
Gorai.006G210200.1 Vv15g0352 . . Gorai.
008G116300.1 . . Gorai.004G156100.1 . .
. . . . .

Vv02g0468 Tc03_g026660 Gorai.004G207600.1 Gorai.
003G162800.1 . . . . . . .
. . . . . . . .
. .
Vv02g0469 . . . . . . .
. . . . . . . .
. . . . .
Vv02g0470 Tc03_g026650 . . . . .
. . . . . . . .
. . . . .
Vv02g0471 Tc03_g030620 . . . . .
. . . . . . . .
. . . . . .
Vv02g0472 Tc03_g026630 Gorai.004G207300.1 .
Gorai.007G022200.1 . . . . . .
. . . . . . . .
. .
Vv02g0473 Tc03_g026620 . Gorai.003G162900.1 .
Gorai.008G243400.1 . Vv15g0355 . .
Gorai.008G115900.1 . . . . .
. . . . .
Vv02g0474 Tc03_g026610 Gorai.004G207200.1 .
Gorai.007G009900.1 Gorai.008G281500.1 . . .
. . . . . . . .
. . . .
Vv02g0475 . . . . . . .
. . . . . . . .
. . . . .
Vv02g0476 Tc03_g026600 . Gorai.003G163000.1 .
Gorai.008G243200.1 . . . . .
. . . . . . . .
. .
Vv02g0477 Tc03_g026590 Gorai.004G207000.1 Gorai.
003G163200.1 . Gorai.008G243100.1 . . .
. . . . . . . .
. . . .
Vv02g0478 Tc03_g026580 Gorai.004G206900.1 .
Gorai.007G094200.1 Gorai.008G243000.1 Gorai.006G197000.1
Vv15g0363 . . . . . .
. . . . . . .
Vv02g0479 Tc03_g026560 Gorai.004G206800.1 . .
. . . . . . . .
. . . . . . . .
Vv02g0480 Tc03_g026530 . Gorai.003G163300.1 .
. . . . . . . .
. . . . . . . .
Vv02g0481 Tc03_g026510 Gorai.004G206700.1 .
Gorai.007G094100.1 . . Vv15g0364 . .
Gorai.008G115700.1 . . . . .
. . . . .

Vv02g0482 Tc03_g026500 Gorai.004G206600.1 Gorai.
003G163800.1 . Gorai.008G242800.1 . . .
. . . . . . . .
. . . .
Vv02g0483 Tc03_g026490 Gorai.004G206500.1 .
Gorai.007G022100.1 . Gorai.006G210500.1 . .
. . . . . . . .
. . . .
Vv02g0484 Tc03_g026480 . Gorai.003G163900.1
Gorai.007G009800.1 Gorai.008G242500.1 . Vv15g0375
. . . . . . Vv016rg0176
. . . . . .
Vv02g0485 Tc03_g026470 Gorai.004G206400.1 . .
. . . . . . . .
. . . . . . . .
Vv02g0486 . . Gorai.003G164000.1 . .
. . . . . . . .
. . . . . . .
Vv02g0487 Tc03_g026460 Gorai.004G206300.1 .
Gorai.007G007000.1 Gorai.008G242400.1 Gorai.006G210600.1
. . . . . . . .
. . . . . .
Vv02g0488 Tc03_g026450 Gorai.004G206200.1 . .
Gorai.008G242300.1 Gorai.006G210700.1 . .
. . . . . . . .
. . . .
Vv02g0489 Tc03_g026440 Gorai.004G206100.1 .
Gorai.007G022000.1 . Gorai.006G210800.1 . .
. . . . . . . .
. . . .
Vv02g0490 Tc03_g026430 Gorai.004G206000.1 . .
Gorai.008G242200.1 . . . . .
. . . . . . . .
. .
Vv02g0491 Tc03_g026420 Gorai.004G205900.1 . .
Gorai.008G242100.1 . . . . .
. . . . . . . .
. .
Vv02g0492 Tc03_g026410 . Gorai.003G164300.1 .
. . Vv15g0383 . . . .
. . . . . . . .
.
Vv02g0493 . . . . . . .
. . . . . . . .
. . . . .
Vv02g0494 . . . . . . .
. . . . . . . .
. . . . .
Vv02g0495 Tc03_g026400 . Gorai.003G164400.1 .
Gorai.008G242000.1 . . . . .

. . . . . . . .
. .
Vv02g0496 Tc03_g026360 Gorai.004G205800.1 . .
. . . . . . . .
. . . . . . . .
Vv02g0497 Tc03_g026350 . . . Gorai.
008G241900.1 . . . . . . .
. . . . . . . .
Vv02g0498 Tc03_g026340 Gorai.004G205700.1 . .
. . . . . . . .
. . . . . . . .
Vv02g0499 Tc03_g026330 Gorai.004G205600.1 . .
. . . . . . . .
. . . . . . . .
Vv02g0500 . . . . . . .
. . . . . . . .
. . . . .
Vv02g0501 Tc03_g026320 . . . . .
. . . . . . . .
. . . . . .
Vv02g0502 . . . . . . .
. . . . . . . .
. . . . .
Vv02g0503 . . . . . . .
. . . . . . . .
. . . . .
Vv02g0504 Tc03_g026310 Gorai.004G205500.1 . .
. . . . . . . .
. . . . . . . .
Vv02g0505 Tc03_g026300 . . . .
Gorai.006G210900.1 . . . . . .
. . . . . . . .
Vv02g0506 Tc03_g026290 Gorai.004G205400.1 . .
. . . . . . . .
. . . . . . . .
Vv02g0507 . . . . . . .
. . . . . . . .
. . . . .
Vv02g0508 Tc03_g026280 Gorai.004G205000.1 . .
. . . . . . . .
. . . . . . . .
Vv02g0509 . . . . . . .
. . . . . . . .
. . . . .
Vv02g0510 Tc03_g026270 Gorai.004G204800.1 . .
. . . . . . . .
. . . . . . . .
Vv02g0511 Tc03_g030330 . . . . .
. . . . . . . .
. . . . . .

Vv02g0512	.	.	.	.	.	.
Vv15g0413	.	.	.	.	.	.	.
	.	.	.	.	.	.
Vv02g0513	.	.	.	.	.	.	.
	.	.	.	.	.	.	.	.
	.	.	.	.	.
Vv02g0514	Tc03_g026260	Gorai.004G204600.1	.
Gorai.007G092400.1	Gorai.008G241800.1	.	Vv15g0429
	.	.	.	.	.	.	.	.
	.	.	.	.	.
Vv02g0515	Tc03_g026250	Gorai.004G204500.1	Gorai.
003G164600.1	.	Gorai.008G241700.1	.	.	.
	.	.	.	.	.	.	.	.
	.	.	.	.
Vv02g0516	Tc03_g026240	Gorai.004G204400.1	.	.
	Gorai.008G241600.1	.	.	.	.	.
	.	.	.	.	.	.	.	.
	.	.
Vv02g0517	Tc03_g026230	Gorai.004G204300.1	.	.
	.	.	.	.	.	.	.	.
	.	.	.	.	.	.	.	.
Vv02g0518	.	.	.	.	.	.	.
	.	.	.	.	.	.	.	.
	.	.	.	.	.
Vv02g0519	Tc03_g026220	.	Gorai.003G164700.1	.
	Gorai.008G241500.1	Gorai.006G196100.1	.	.
	.	.	.	.	.	Vv016rg0140	.
	.	.	.	.	.
Vv02g0520	.	.	.	.	.	.	.
	.	.	.	.	.	.	.	.
	.	.	.	.	.
Vv02g0521	.	.	.	.	.	.	.
	.	.	.	.	.	.	.	.
	.	.	.	.	.
Vv02g0522	Tc03_g026210	Gorai.004G204200.1	.	.
	.	.	.	.	.	.	.	.
	.	Vv016rg0138	.	.	.	.	.
	.
Vv02g0523	.	.	.	.	Gorai.008G283200.1
	.	.	.	.	.	.	.	.
	.	.	.	.	.	.	.
Vv02g0524	Tc03_g026200	Gorai.004G204100.1	.	.
	Gorai.008G241300.1	.	.	.	.	.
	.	.	.	.	.	.	.	.
	.	.
Vv02g0525	Tc03_g026190	Gorai.004G204000.1	Gorai.
003G164800.1	.	.	.	.	.	.	.
	.	.	.	.	.	.	.	.
	.	.
Vv02g0526	Tc03_g026170	.	.	.	Gorai.

008G241200.1 . . . . . . .
. . . . . . . .
Vv02g0527 . . . . . . .
. . . . . . . .
. . . . .
Vv02g0528 Tc03_g026150 . . Gorai.007G092100.1
Gorai.008G241000.1 . Vv15g0451
Tc01_g036690 . . Gorai.001G171000.1 . .
. . . . . . .
Vv02g0529 Tc03_g026140 . . . Gorai.
008G240900.1 Gorai.006G211300.1 Vv016rg0136 . .
. . . . . . . .
. . .
Vv02g0530 Tc03_g026130 . . Gorai.007G091900.1
Gorai.008G240800.1 Gorai.006G211400.1 . .
. . . . . . . .
. . . .
Vv02g0531 Tc03_g026120 Gorai.004G160700.1 .
Gorai.007G091800.1 Gorai.008G240700.1 Gorai.006G211500.1
Vv15g0335 Tc01_g037430 . . . .
Gorai.004G158000.1 . . . . .
. .
Vv02g0532 Tc03_g026100 . . . . .
. . . . . . . .
. . . . . .
Vv02g0533 Tc03_g026080 Gorai.004G203900.1 .
Gorai.007G021800.1 . Gorai.006G211600.1 . .
. . . . . . . .
. . . .
Vv02g0534 Tc03_g026070 Gorai.004G161300.1 .
Gorai.007G021700.1 Gorai.008G283600.1 . Vv15g0324
Tc01_g037530 . . . . .
Vv016rg0120 . . . . . .
Vv02g0535 Tc03_g025930 . . Gorai.007G021600.1
. . . . . . . .
. . . . . . . .
Vv02g0536 Tc03_g025920 . Gorai.003G166600.1 .
. . . . . . . .
. . . . . . . .
Vv02g0537 Tc03_g025910 Gorai.004G162000.1 . .
Gorai.008G239700.1 . Vv15g0317
Tc01_g037740 . Gorai.008G113900.1 . .
Gorai.004G162000.1 Vv016rg0099 . . . .
. .
Vv02g0538 Tc03_g025900 . . . Gorai.
008G239600.1 . . . . . . .
. . . . . . . .
Vv02g0539 Tc03_g025890 . . . Gorai.
008G239500.1 . . . . . . .
. . . . . . . .

Vv02g0540 . . . . . . .
. . . . . . . .
. . . . .
Vv02g0541 Tc03_g025880 . . . Gorai.
008G239400.1 Gorai.006G211900.1 . . . .
. . . . . . . .
. .
Vv02g0542 Tc03_g025870 Gorai.004G162400.1 .
Gorai.007G090600.1 Gorai.008G284100.1 Gorai.006G195900.1
Vv15g0314 Tc01_g037770 . . Gorai.
001G174200.1 Gorai.004G040700.1 Gorai.004G162400.1
Vv016rg0094 . . . . . .
Vv02g0543 Tc03_g025860 . . Gorai.007G021400.1
Gorai.008G239300.1 . . . . .
. . . . . . . .
. .
Vv02g0544 . . . . . . .
. . . . . . . .
. . . . .
Vv02g0545 Tc03_g025850 . . . . .
. . . . . . . .
. . . . . .
Vv02g0546 Tc03_g025840 Gorai.004G162700.1 .
Gorai.007G090500.1 . . . . . .
. . . Vv016rg0079 . . .
. . .
Vv02g0547 . . . . . . .
. . . . . . . .
. . . . .
Vv02g0548 . . . . . . .
. . . . . . . .
. . . . .
Vv02g0549 Tc03_g025830 Gorai.004G202100.1 Gorai.
003G116000.1 . . Gorai.006G195100.1 . .
. . . . . . . .
. . . .
Vv02g0550 Tc03_g025810 . Gorai.003G165000.1 .
. . . . . . . .
. . . . . . . .
Vv02g0551 Tc03_g025790 . Gorai.003G165100.1 .
. . . . . . . .
. . . . . . . .
Vv02g0552 . . . . . . .
. . . . . . . .
. . . . .
Vv02g0553 Tc03_g025780 . Gorai.003G165300.1 .
. . . . . . . .
. . . . . . . .
Vv02g0554 . . . . . . .
. . . . . . . .

. . . . .
Vv02g0555 Tc03_g025770 . Gorai.003G165400.1 .
. . . . . . . .
. . . . . . . .
Vv02g0556 Tc03_g025760 . Gorai.003G165500.1 .
. . . . . . . .
. . . . . . . .
Vv02g0557 Tc03_g025740 Gorai.004G202000.1 Gorai.
003G165600.1 . Gorai.008G239200.1 . . .
. . . . . . . .
. . . .
Vv02g0558 . . . . . . .
. . . . . . . .
. . . . .
Vv02g0559 Tc03_g025720 . Gorai.003G168000.1 .
Gorai.008G238900.1 . . . . .
. . . . . . . .
. .
Vv02g0560 Tc03_g025710 Gorai.004G201900.1 . .
Gorai.008G238800.1 . . . . .
. . . . . . . .
. .
Vv02g0561 Tc03_g025700 Gorai.004G201800.1 .
Gorai.007G021300.1 . . . . . .
. . . . . . . .
. .
Vv02g0562 Tc03_g025690 Gorai.004G201700.1 .
Gorai.007G021100.1 Gorai.008G238700.1 . . .
. . . . . . . .
. . . .
Vv02g0563 Tc03_g025680 . . Gorai.007G021000.1
. . . . . . . .
. . . . . . . .
Vv02g0564 Tc03_g025620 Gorai.004G201500.1 Gorai.
003G114800.1 Gorai.007G090200.1 Gorai.008G238600.1
Gorai.006G212000.1 Vv15g0299 Tc01_g037920 .
Gorai.008G113200.1 . . . . . .
. . . .
Vv02g0565 . . . . . . .
. . . . . . . .
. . . . .
Vv02g0566 Tc03_g025600 Gorai.004G201400.1 Gorai.
003G114700.1 . Gorai.008G238500.1 Gorai.006G212100.1
Vv15g0297 Tc01_g037930 . . . .
. . . . . . . .
Vv02g0567 Tc03_g025580 . . Gorai.007G020900.1
. . . . . . . .
. . . . . . . .
Vv02g0568 Tc03_g025570 . . Gorai.007G020800.1
. Gorai.006G212300.1 . . . .

. . . . . . . .
. .
Vv02g0569 Tc03_g025550 Gorai.004G201300.1 .
Gorai.007G018000.1 . . . . . .
. . . . . . . .
. .
Vv02g0570 Tc03_g025540 Gorai.004G163300.1 Gorai.
003G168900.1 Gorai.007G089900.1 . Gorai.006G212400.1
. . . . . . . .
. . . . . .
Vv02g0571 . . . . . . .
. . . . . . . .
. . . . .
Vv02g0572 Tc03_g025530 . Gorai.003G169000.1 .
. . . . . . . .
. . . . . . . .
Vv02g0573 Tc03_g025520 . . . . .
. . . . . . . .
. . . . . .
Vv02g0574 . Gorai.004G200900.1 . . .
. . . . . . . .
. . . . . . .
Vv02g0575 Tc03_g025510 . . Gorai.007G089700.1
. . . . . . . .
. . . . . . . .
Vv02g0576 Tc03_g025500 . . . .
Gorai.006G212500.1 . . . . . .
. . . . . . . .
Vv02g0577 . . . . . Gorai.
006G212700.1 . . . . . . .
. . . . . . .
Vv02g0578 Tc03_g025480 . . . . .
. . . . . . . .
. . . . . .
Vv02g0579 . . Gorai.003G169200.1 . .
. . . . . . . .
. . . . . . .
Vv02g0580 Tc03_g025470 . Gorai.003G111200.1 .
. . . . . . . .
. . . . . . . .
Vv02g0581 Tc03_g025460 . Gorai.003G169400.1 .
. . . . . . . .
. . . . . . . .
Vv02g0582 Tc03_g025450 . Gorai.003G169500.1 .
. . . . . . . .
. . . . . . . .
Vv02g0583 Tc03_g025440 . Gorai.003G169600.1 .
. Gorai.006G212800.1 . . . .
. . . . . . . .
. .

Vv02g0584 . . . . . . .
. . . . . . . .
. . . . .
Vv02g0585 . . . . . . .
. . . . . . . .
. . . . .
Vv02g0586 Tc03_g025430 Gorai.004G163500.1 Gorai.
003G169800.1 Gorai.007G089600.1 . . . .
. . . . . . . .
. . . .
Vv02g0587 Tc03_g025420 . . . . .
. . . . . . . .
. . . . . .
Vv02g0588 . . . . . . .
. . . . . . . .
. . . . .
Vv02g0589 Tc03_g025410 . Gorai.003G170000.1
Gorai.007G089500.1 . . . . . .
. . . . . . . .
. .
Vv02g0590 . . . Gorai.007G089300.1 .
. . . . . . . .
. . . . . . .
Vv02g0591 Tc03_g025400 . . . . .
. . . . . . . .
. . . . . .
Vv02g0592 . . . . . . .
. . . . . . . .
. . . . .
Vv02g0593 Tc03_g025390 . . . . .
. . . . . . . .
. . . . . .
Vv02g0594 Tc03_g025380 . . . . .
. . . . . . . .
. . . . . .
Vv02g0595 Tc03_g025370 . Gorai.003G170100.1 .
. . . . . . . .
. Vv18g0366 Tc08_g010160 . .
Gorai.009G243200.1 . Gorai.010G048000.1
Vv02g0596 Tc03_g025350 . . . . .
. . . . . . . .
. . . . . .
Vv02g0597 . . . . . . .
. . . . . . . .
. . . . .
Vv02g0598 Tc03_g025340 . . . .
Gorai.006G212900.1 . . . . . .
. . . . . . . .
Vv02g0599 Tc03_g025330 . Gorai.003G170200.1 .
. . . . . . . .

. Vv18g0372 Tc08_g010030 . Gorai.
005G091400.1 Gorai.009G242900.1 Gorai.009G283100.1
Gorai.010G047800.1
Vv02g0600 . . . . . . .
. . . . . . . .
. . . . .
Vv02g0601 Tc03_g025320 . Gorai.003G170400.1 .
. . Vv15g0287 . . . .
. . . . . . . .
.
Vv02g0602 . . . . . . .
. . . . . . . .
. . . . .
Vv02g0603 Tc03_g025310 Gorai.004G200000.1 .
Gorai.007G027400.1 . . . . . .
. . . . . . . .
. .
Vv02g0604 Tc03_g025300 Gorai.004G199900.1 .
Gorai.007G027500.1 . . . . . .
. . . . . . . .
. .
Vv02g0605 . . . . . . .
. . . . . . . .
. . . . .
Vv02g0606 . . . . . . .
. . . . . . . .
. . . . .
Vv02g0607 Tc03_g025290 . . . Gorai.
008G237700.1 Gorai.006G213000.1 . . . .
. . . . . . . .
. .
Vv02g0608 Tc03_g025280 . . Gorai.007G027700.1
Gorai.008G237600.1 . . . . .
. . . . . . . .
. .
Vv02g0609 Tc03_g025270 . . . . .
. . . . . . . .
. . . . . .
Vv02g0610 . . . . . . .
. . . . . . . .
. . . . .
Vv02g0611 Tc03_g025210 . Gorai.003G170500.1 .
. . . . . . . .
. . . . . . . .
Vv02g0612 Tc03_g025200 . . . . .
. . . . . . . .
. . . . . .
Vv02g0613 . . . . . . .
. . . . . . . .
. . . . .

Vv02g0614 Tc03_g025180 Gorai.004G163700.1 Gorai.
003G170600.1 . . Gorai.006G213100.1 . .
. . . . . . . .
. . . .
Vv02g0615 . . . . . . .
. . . . . . . .
. . . . .
Vv02g0616 Tc03_g025170 . Gorai.003G170700.1 .
. . Vv15g0269 Tc01_g038130 .
Gorai.008G106100.1 . . . . . .
. . . .
Vv02g0617 Tc03_g025150 . . . . .
. . . . . . . .
. . . . . .
Vv02g0618 . . . . Gorai.008G236000.1
. . . . . . . .
. . . . . . .
Vv02g0619 . . . . . Gorai.
006G213300.1 . . . . . . .
. . . . . . .
Vv02g0620 . . . . . . .
. . . . . . . .
. . . . .
Vv02g0621 Tc03_g025120 . . . Gorai.
008G216100.1 . . . . . . .
. . . . . . . .
Vv02g0622 Tc03_g025110 Gorai.004G199300.1 . .
. . . . . . . .
. . . . . . . .
Vv02g0623 Tc03_g025100 Gorai.004G163800.1 Gorai.
003G170900.1 Gorai.007G028100.1 Gorai.008G235700.1
Gorai.006G213500.1 Vv15g0266 Tc01_g038160 .
Gorai.008G113000.1 . Gorai.004G040100.1 Gorai.
004G163800.1 . . . . . . .
Vv02g0624 Tc03_g025090 . . . Gorai.
008G235600.1 . . . . . . .
. . . . . . . .
Vv02g0625 Tc03_g025080 Gorai.004G199100.1 Gorai.
003G171000.1 . Gorai.008G217800.1 . . .
. . . . . . . .
. . . .
Vv02g0626 Tc03_g025070 Gorai.004G199000.1 . .
. . . . . . . .
. . . . . . . .
Vv02g0627 Tc03_g025060 . Gorai.003G171100.1 .
. . . . . . . .
. . . . . . . .
Vv02g0628 Tc03_g025050 . Gorai.003G171200.1 .
. . Vv18g0401 Tc08_g009750 . .
. . . . . . . .

. .
Vv02g0629 . . . . . . .
. . . . . . . .
. . . . .
Vv02g0630 Tc03_g025030 Gorai.004G198800.1 . .
Gorai.008G235400.1 . . . . .
. . . . . . . .
. .
Vv02g0631 Tc03_g025020 . Gorai.003G171400.1 .
Gorai.008G235300.1 . . . . .
. . . . . . . .
. .
Vv02g0632 Tc03_g025010 Gorai.004G198700.1 Gorai.
003G171500.1 . Gorai.008G235200.1 . . .
. . . . . . . .
. . . .
Vv02g0633 Tc03_g025000 . . . . .
. . . . . . . .
. . . . . .
Vv02g0634 Tc03_g024980 . . . .
Gorai.006G213600.1 . . . . . .
. . . . . . . .
Vv02g0635 Tc03_g024940 Gorai.004G198200.1 . .
Gorai.008G235000.1 Gorai.006G213800.1 . .
. . . . . . . .
. . . .
Vv02g0636 Tc03_g024930 . . Gorai.007G028200.1
. . . . . . . .
. . . . . . . .
Vv02g0637 . . . . . . .
. . . . . . . .
. . . . .
Vv02g0638 Tc03_g024900 . . Gorai.007G028300.1
Gorai.008G234800.1 . . . . .
. . . . . . . .
. .
Vv02g0639 . Gorai.004G197800.1 . Gorai.
007G028500.1 Gorai.008G232800.1 . . . .
. . . . . . . .
. . .
Vv02g0640 . Gorai.004G191200.1 . . .
. . . . . . . .
. . . . . . .
Vv02g0641 . Gorai.004G191100.1 . . .
. . . . . . . .
. . . . . . .
Vv02g0642 . . . . . .
Vv15g0079 Tc01_g039820 . Gorai.008G110700.1 .
Gorai.004G038500.1 Gorai.004G167300.1 . .
. . . . .

Vv02g0643 . . . . . . .
. . . . . . .
. . . . .
Vv02g0644 . Gorai.004G191000.1 . Gorai.
007G030400.1 . . . . . . .
. . . . . . .
.
Vv02g0645 . . . . . . .
. . . . . . .
. . . . .
Vv02g0646 . Gorai.004G190900.1 . . .
Gorai.006G214800.1 Vv15g0078 Tc01_g039830 .
Gorai.008G110800.1 . . Gorai.004G167400.1
. . . . . . .
Vv02g0647 . Gorai.004G190800.1 . . .
Gorai.006G214900.1 Vv15g0077 Tc01_g039840 .
. . . Gorai.004G167500.1 . .
. . . . .
Vv02g0648 . . . . . . .
. . . . . . .
. . . . .
Vv02g0649 . . . . . . .
. . . . . . .
. . . . .
Vv02g0650 . Gorai.004G190700.1 . . .
. . . . . . .
. . . . . . .
Vv02g0651 . . . . . . .
. . . . . . .
. . . . .
Vv02g0652 . . . . . . .
. . . . . . .
. . . . .
Vv02g0653 . . . . . . .
. . . . . . .
. . . . .
Vv02g0654 . . . Gorai.007G030600.1
Gorai.008G218300.1 Gorai.006G215100.1 . . .
. . . . . . .
. . .
Vv02g0655 . . . . . . .
. . . . . . .
. . . . .
Vv02g0656 . . . . . . .
. . . . . . .
. . . . .
Vv02g0657 . Gorai.004G190500.1 . . .
. . . . . . .
. . . . . . .
Vv02g0658 . . . . . . .

. . . . . . . .
. . . . .
Vv02g0659 . . . . . . .
. . . . . . . .
. . . . .
Vv02g0660 . Gorai.004G190400.1 Gorai.003G179300.1
. . Gorai.006G215200.1 Vv15g0066
Tc01_g039890 . . . . . Vv18g0430
Tc08_g009430 . . Gorai.009G239700.1 .
Gorai.010G044000.1
Vv02g0661 . Gorai.004G190300.1 Gorai.003G179400.1
Gorai.007G030800.1 . . . . .
. . . . . . . .
. . .
Vv02g0662 . . . . . . .
. . . . . . . .
. . . . .
Vv02g0663 . . . . . . .
. . . . . . . .
. . . . .
Vv02g0664 . Gorai.004G190200.1 . Gorai.
007G030900.1 . . . . . . .
. . . . . . . .
.
Vv02g0665 . . . . . . .
. . . . . . . .
. . . . .
Vv02g0666 . . . . . . .
. . . . . . . .
. . . . .
Vv02g0667 . . . . . . .
. . . . . . . .
. . . . .
Vv02g0668 . . . . . . .
. . . . . . . .
. . . . .
Vv02g0669 . . . . . . .
. . . . . . . .
. . . . .
Vv02g0670 . . . . . . .
. . . . . . . .
. . . . .
Vv02g0671 . . . . . . .
. . . . . . . .
. . . . .
Vv02g0672 Tc03_g026420 . . . . .
. . . . . . . .
. . . . . .
Vv02g0673 . . . . . . .
. . . . . . . .

. . . . .
Vv02g0674 . . . . . . .
. . . . . . . .
. . . . .
Vv02g0675 . . . . . . .
. . . . . . . .
. . . . .
Vv02g0676 . . . . . . .
. . . . . . . .
. . . . .
Vv02g0677 . . . . . . .
. . . . . . . .
. . . . .
Vv02g0678 . . . . . . .
. . . . . . . .
. . . . .
Vv02g0679 . Gorai.004G190100.1 . . .
. . . . . . . .
. . . . . . .
Vv02g0680 . . . . . . .
. . . . . . . .
. . . . .
Vv02g0681 . Gorai.004G189700.1 Gorai.003G179500.1
. . Gorai.006G215300.1 . . .
. . . . Vv18g0438
Tc08_g009380 . . Gorai.009G239200.1 Gorai.
009G282100.1 .
Vv02g0682 . . . . . . .
. . . . . . . .
. . . . .
Vv02g0683 . . Gorai.003G179700.1 . .
Gorai.006G215400.1 Vv15g0037 . . .
. . Gorai.004G168200.1 . . .
. . . .
Vv02g0684 . . . . . . .
. . . . . . . .
. . . . .
Vv02g0685 . . . . . . .
. . . . . . . .
. . . . .
Vv02g0686 . . . . . . .
. . . . . . . .
. . . . .
Vv02g0687 . . . . . . .
. . . . . . . .
. . . . .
Vv02g0688 Tc03_g021880 . . . . .
. . . . . . . .
. . . . . .
Vv02g0689 . . . . . . .

. . . . . . . .
. . . . .
Vv02g0690 . . . . . Gorai.
006G215600.1 . . . . . . .
. . . . . . .
Vv02g0691 . . . . . . .
. . . . . . . .
. . . . .
Vv02g0692 . . . . . . .
. . . . . . . .
. . . . .
Vv02g0693 Tc03_g022270 . . . Gorai.
008G220500.1 . . . . . . .
. Vv18g0452 Tc08_g009210 . . .
Gorai.009G281700.1 .
Vv02g0694 . . . . . . .
. . . . . . . .
. . . . .
Vv02g0695 . . . . . . .
. . . . . . . .
. . . . .
Vv02g0696 . . . . . . .
. . . . . . . .
. . . . .
Vv02g0697 . . . . . . .
. . . . . . . .
. . . . .
Vv02g0698 . . Gorai.003G179800.1 . .
. . . . . . . .
. . . . . . .
Vv02g0699 . . . . . . .
. . . . . . . .
. . . . .
Vv02g0700 . . . . . . .
. . . . . . . .
. . . . .
Vv02g0701 . . . . . . .
. . . . . . . .
. . . . .
Vv02g0702 . . . . . . .
. . . . . . . .
. . . . .
Vv02g0703 . . . . Gorai.008G229500.1
. Vv15g0027 . . . . .
. . . . . . . .
Vv02g0704 . Gorai.004G189000.1 . . .
. Vv15g0026 Tc01_g008680 . . .
. . . . . . . .
.
Vv02g0705 . . . . Gorai.008G229400.1

. . . . . . . .
. . . . . . .
Vv02g0706 . . . . . . .
. . . . . . . .
. . . . .
Vv02g0707 . Gorai.004G188800.1 . .
Gorai.008G229300.1 . . . . . .
. . . . . . . .
.
Vv02g0708 . . . . . . .
. . . . . . . .
. . . . .
Vv02g0709 . . . . . . .
. . . . . . . .
. . . . .
Vv02g0710 . . . . . . .
. . . . . . . .
. . . . .
Vv02g0711 Tc03_g026120 Gorai.004G160700.1 . .
. Gorai.006G216300.1 . . . .
. . . . . . . .
. .
Vv02g0712 . . . . . . .
. . . . . . . .
. . . . .
Vv02g0713 . Gorai.004G161300.1 . . .
. . . . . . . .
. . . . . . .
Vv02g0714 . . . Gorai.007G031400.1 .
. . . . . . . .
. . . . . . .
Vv02g0715 . . . . . . .
. . . . . . . .
. . . . .
Vv02g0716 . . . . . . .
. . . . . . . .
. . . . .
Vv02g0717 . . . . . . .
. . . . . . . .
. . . . .
Vv02g0718 . . . . . . .
. . . . . . . .
. . . . .
Vv02g0719 . . . . . . .
. . . . . . . .
. . . . .
Vv02g0720 . . . . . . .
. . . . . . . .
. . . . .
Vv02g0721 . . . . . . .

. . . . . . . .
. . . . .
Vv02g0722 . . . . . . .
. . . . . . . .
. . . . .
Vv02g0723 . . . . . . .
. . . . . . . .
. . . . .
Vv02g0724 . . . . . . .
. . . . . . . .
. . . . .
Vv02g0725 Tc03_g002180 . . . . .
. . . . . . . .
. . . . . .
Vv02g0726 Tc03_g002440 . Gorai.003G180700.1 .
Gorai.008G227600.1 . . . . .
. . . . . . . .
. .
Vv02g0727 . Gorai.004G186500.1 . . .
. . . . . . . .
. . . . . . .
Vv02g0728 . . . . . . .
. . . . . . . .
. . . . .
Vv02g0729 Tc03_g022430 . . . . .
. . . . . . . .
. . . . . .
Vv02g0730 . . . . . . .
. . . . . . . .
. . . . .
Vv02g0731 . . . . . . .
. . . . . . . .
. . . . .
Vv02g0732 . . . . . . .
. . . . . . . .
. . . . .
Vv02g0733 . . . . . . .
. . . . . . Vv18g0466
Tc08_g008980 . . Gorai.009G236500.1 .
Gorai.010G040100.1
Vv02g0734 . . . . . . .
. . . . . . . .
. . . . .
Vv02g0735 . . . . . . .
. . . . . . . .
. . . . .
Vv02g0736 . . . . . . .
. . . . . . . .
. . . . .
Vv02g0737 . . . . . . .

```
        .        .       .        .       .        .       .        .
        .        .       .        .       .
Vv02g0738        Tc03_g031180     .       .        .       .        .
        .        .       .        .       .        .       .        .
        .        .       .        .       .        .
Vv02g0739        .       .        .       .        .       .        .
        .        .       .        .       .        .       .        .
        .        .       .        .       .
Vv02g0740        .       .        .       .        .       .
Vv15g0015        .       .        .       .        .       .        .
        .        .       .        .       .        .
Vv02g0741        .       .        .       .        .       .        .
        .        .       .        .       .        .       .        .
        .        .       .        .       .
Vv02g0742        .       .        .       .        .       .        .
        .        .       .        .       .        .       .        .
        .        .       .        .       .
Vv02g0743        .       .        .       .        .       .        .
        .        .       .        .       .        .       .        .
        .        .       .        .       .
Vv02g0744        .       Gorai.004G185800.1        Gorai.003G181300.1
        .        Gorai.008G226800.1       Gorai.006G216400.1        .
        .        .       .        .       .        .       .        .
        .        .       .        .       .
Vv02g0745        .       .        Gorai.003G181400.1       .        .
        .        .       .        .       .        .       .        .
        .        .       .        .       .        .       .
Vv02g0746        .       Gorai.004G185700.1        .       .        .
        .        .       .        .       .        .       .        .
        .        .       .        .       .        .       .
Vv02g0747        Tc03_g012040     .       .        .       .        .
        Vv16g0119        .        .       .        .       Gorai.
008G226700.1     .       .        .       .        .       .        .
        .
Vv02g0748        .       .        .       .        Gorai.008G226700.1
        .        .       .        .       .        .       .        .
        .        .       .        .       .        .       .
Vv02g0749        .       .        .       .        .       .        .
        .        .       .        .       .        .       .        .
        .        .       .        .       .
Vv02g0750        .       .        Gorai.003G181600.1       Gorai.
007G031800.1     Gorai.008G226500.1       .        .       .        .
        .        .       .        .       .        .       .        .
        .        .       .
Vv02g0751        .       Gorai.004G185400.1        Gorai.003G181800.1
        .        .       Gorai.006G216500.1        .       .        .
        .        .       .        .       .        .       .        .
        .        .       .
Vv02g0752        .       .        .       .        .       .        .
        .        .       .        .       .        .       .        .
```

. . . . .
Vv02g0753 Tc03_g022840 Gorai.004G184800.1 Gorai.
003G182100.1 . Gorai.008G226300.1 Gorai.006G216700.1
. . . . . . . .
. . . . . .
Vv02g0754 . . . . . . .
. . . . . . . .
. . . . .
Vv02g0755 Tc03_g023090 . Gorai.003G182200.1
Gorai.007G031900.1 Gorai.008G226200.1 Gorai.006G216800.1
. . . . . . . .
. . . . . .
Vv02g0756 . . Gorai.003G182400.1 Gorai.
007G032000.1 . . . . . . .
. . . . . . . .
.
Vv02g0757 . . . . . . .
. . . . . . . .
. . . . .
Vv02g0758 . . . . . . .
. . . . . . . .
. . . . .
Vv02g0759 . Gorai.004G184600.1 . Gorai.
007G032300.1 . Gorai.006G216900.1 . . .
. . . . . . . .
. . .
Vv02g0760 . . . . . . .
. . . . . . . .
. . . . .
Vv02g0761 . Gorai.004G163500.1 Gorai.003G183000.1
Gorai.007G032400.1 Gorai.008G226100.1 Gorai.
006G217100.1 Vv16g0127 . . . Gorai.
007G032400.1 Gorai.008G226100.1 . . . .
. . . .
Vv02g0762 . Gorai.004G164100.1 Gorai.003G183100.1
. . . . . . . .
. . . . . . . .
.
Vv02g0763 . . . . . . .
. . . . . . . .
. . . . .
Vv02g0764 Tc03_g012150 . Gorai.003G183300.1
Gorai.007G032500.1 . . . . . .
. . . . . . . .
. .
Vv02g0765 . . . . . . .
. . . . . . . .
. . . . .
Vv02g0766 . . . Gorai.007G032600.1
Gorai.008G225900.1 . . . . . .

. . . . . . . .
.
Vv02g0767 . . . . Gorai.008G225800.1
Gorai.006G217200.1 . . . . .
. . . . . . . .
.
Vv02g0768 . . Gorai.003G183400.1 . .
. . . . . . . .
. . . . . . .
Vv02g0769 . . . . . . .
. . . . . . . .
. . . . .
Vv02g0770 . . . . . . .
. . . . . . . .
. . . . .
Vv02g0771 . . . . . . .
. . . . . . . .
. . . . .
Vv02g0772 . . . . . . .
. . . . . . . .
. . . . .
Vv02g0773 Tc03_g031070 Gorai.004G184200.1 .
Gorai.007G032700.1 Gorai.008G225700.1 Gorai.006G217300.1
. . . . . . . .
. . . . . .
Vv02g0774 . . . Gorai.007G032800.1
Gorai.008G225600.1 . Vv16g0130 . . .
Gorai.007G032800.1 Gorai.008G225600.1 . .
. . . . . .
Vv02g0775 . . . . . . .
. . . . . . . .
. . . . .
Vv02g0776 . . . . . . .
. . . . . . . .
. . . . .
Vv02g0777 . . . . . . .
. . . . . . . .
. . . . .
Vv02g0778 . Gorai.004G184100.1 . . .
. Vv16g0141 . . . . .
. . . . . . . .
Vv02g0779 . Gorai.004G184000.1 . . .
. . . . . . . .
. . . . . . .
Vv02g0780 . Gorai.004G183900.1 . Gorai.
007G033000.1 . . . . . . .
. . . . . . . .
.
Vv02g0781 . . . . . . .
. . . . . . . .

. . . . .
Vv02g0782 . . . . . . .
. . . . . . . .
. . . . .
Vv02g0783 Tc03_g029640 . . . Gorai.
008G225500.1 . . . . . . .
. . . . . . . .
Vv02g0784 Tc03_g029650 Gorai.004G183800.1 Gorai.
003G183600.1 . . Gorai.006G217400.1 . .
. . . . . . . .
. . . .
Vv02g0785 Tc03_g029660 . . Gorai.007G033100.1
. Gorai.006G217500.1 Vv16g0145 . .
. Gorai.007G033100.1 . . . .
. . . . .
Vv02g0786 . . . . . . .
. . . . . . . .
. . . . .
Vv02g0787 Tc03_g029670 . Gorai.003G183700.1 .
. . . . . . . .
. . . . . . . .
Vv02g0788 . . . . . . .
. . . . . . . .
. . . . .
Vv02g0789 Tc03_g012420 . . . . .
. . . . . . . .
. . . . . .
Vv02g0790 Tc03_g030790 . . . . .
. . . . . . . .
. . . . . .
Vv02g0791 Tc03_g029700 . Gorai.003G183800.1 .
Gorai.008G225400.1 . . . . .
. . . . . . . .
. .
Vv02g0792 . . . . . . .
. . . . . . . .
. . . . .
Vv02g0793 . . . . . . .
. . . . . . . .
. . . . .
Vv02g0794 Tc03_g029710 Gorai.004G183400.1 .
Gorai.007G033200.1 . . . . . .
. . . . . . . .
. .
Vv02g0795 . . . . . . .
. . . . . . . .
. . . . .
Vv02g0796 . . . . . . .
. . . . . . . .
. . . . .

Vv02g0797 . . . . . . .
. . . . . . . .
. . . . .
Vv02g0798 . . . . . . .
. . . . . . . .
. . . . .
Vv02g0799 . . . . . . .
. . . . . . . .
. . . . .
Vv02g0800 Tc03_g029720 . . . Gorai.
008G225300.1 . . . . . . .
. . . . . . . .
Vv02g0801 Tc03_g029740 . . . . .
. . . . . . . .
. . . . . .
Vv02g0802 Tc03_g029750 Gorai.004G183200.1 . .
. . . . . . . .
. . . . . . . .
Vv02g0803 . . . . . . .
. . . . . . . .
. . . . .
Vv02g0804 . . . . . . .
. . . . . . . .
. . . . .
Vv02g0805 . . . . . . .
. . . . . . . .
. . . . .
Vv02g0806 Tc03_g029760 . Gorai.003G183900.1
Gorai.007G033300.1 Gorai.008G225200.1 . Vv16g0162
. . . Gorai.007G033300.1 . .
. . . . . . .
Vv02g0807 . . . . . . .
. . . . . . . .
. . . . .
Vv02g0808 . . . . . . .
. . . . . . . .
. . . . .
Vv02g0809 Tc03_g029790 . . . Gorai.
008G224900.1 . . . . . . .
. . . . . . . .
Vv02g0810 Tc03_g029800 Gorai.004G183100.1 Gorai.
003G184100.1 . Gorai.008G224800.1 Gorai.006G217600.1
Vv16g0163 Tc03_g029800 . . .
Gorai.008G224800.1 . . . . . .
. .
Vv02g0811 . . . . . . .
. . . . . . . .
. . . . .
Vv02g0812 Tc03_g029810 . Gorai.003G184200.1 .
Gorai.008G224700.1 . Vv16g0164

Tc03_g029810 . . . Gorai.008G224700.1 .
. . . . . . .
Vv02g0813 . . . . . . .
. . . . . . . .
. . . . .
Vv02g0814 . . . . . . .
. . . . . . . .
. . . . .
Vv02g0815 . . . . . . .
. . . . . . . .
. . . . .
Vv02g0816 . . . . . . .
. . . . . . . .
. . . . .
Vv02g0817 . . . . . . .
. . . . . . . .
. . . . .
Vv02g0818 . . . . . . .
. . . . . . . .
. . . . .
Vv02g0819 . . . . . . .
. . . . . . . .
. . . . .
Vv02g0820 . . . . . . .
. . . . . . . .
. . . . .
Vv02g0821 . . . . . . .
. . . . . . . .
. . . . .
Vv02g0822 . . . . . . .
. . . . . . . .
. . . . .
Vv02g0823 . . . . . . .
. . . . . . . .
. . . . .
Vv02g0824 Tc03_g030440 . . . Gorai.
008G224400.1 . Vv16g0168 . . . .
Gorai.008G224400.1 . . . . .
. . .
Vv02g0825 . . . . . . .
. . . . . . . .
. . . . .
Vv02g0826 Tc03_g029910 Gorai.004G182600.1 . .
. . . . . . . .
. . . . . . . .
Vv02g0827 . . . . . . .
. . . . . . . .
. . . . .
Vv02g0828 . . . . . . .
. . . . . . . .

. . . . .
Vv02g0829 . . . . . . .
. . . . . . . .
. . . . .
Vv02g0830 . . . . . . .
. . . . . . . .
. . . . .
Vv02g0831 . . . . . . .
. . . . . . . .
. . . . .
Vv02g0832 . . . . . . .
. . . . . . . .
. . . . .
Vv02g0833 . . Gorai.003G184900.1 . .
. . . . . . . .
. . . . . . .
Vv02g0834 . . . . . Gorai.
006G217700.1 . . . . . . .
. . . . . . .
Vv02g0835 . . . . . . .
. . . . . . . .
. . . . .
Vv02g0836 . . . . . . .
. . . . . . . .
. . . . .
Vv02g0837 . . . . . . .
. . . . . . . .
. . . . .
Vv02g0838 . . . . . . .
. . . . . . . .
. . . . .
Vv02g0839 . . . . . . .
. . . . . . . .
. . . . .
Vv02g0840 Tc03_g029980 . Gorai.003G185400.1 .
Gorai.008G224000.1 . . . . .
. . . . . . . .
. .
Vv02g0841 . . . . . . .
. . . . . . . .
. . . . .
Vv02g0842 . . . . . . .
. . . . . . . .
. . . . .
Vv02g0843 Tc03_g029990 Gorai.004G182200.1 Gorai.
003G185500.1 Gorai.007G033500.1 . . . .
. . . . . . . .
. . . .
Vv02g0844 Tc03_g030000 . . . Gorai.
008G223800.1 . . . . . . .

. . . . . . . .
Vv02g0845 Tc03_g030010 . . . Gorai.
008G223700.1 . . . . . . .
. . . . . . . .
Vv02g0846 Tc03_g030020 Gorai.004G182100.1 . .
Gorai.008G223600.1 . . . . .
. . . . . . . .
. .
Vv02g0847 . . . . . . .
. . . . . . . .
. . . . .
Vv02g0848 . . . . . . .
. . . . . . . .
. . . . .
Vv02g0849 Tc03_g030030 . Gorai.003G185600.1 .
. . . . . . . .
. . . . . . . .
Vv02g0850 Tc03_g030050 Gorai.004G181900.1 Gorai.
003G185700.1 . . . . . . .
. . . . . . . .
. .
Vv02g0851 . . . . . . .
. . . . . . . .
. . . . .
Vv02g0852 . . . . . . .
. . . . . . . .
. . . . .
Vv02g0853 Tc03_g030060 Gorai.004G181800.1 . .
. . . . . . . .
. . . . . . . .
Vv02g0854 . . . . . . .
. . . . . . . .
. . . . .
Vv02g0855 . . . . . . .
. . . . . . . .
. . . . .
Vv02g0856 Tc03_g030330 . . . . .
. . . . . . . .
. . . . . .
Vv02g0857 Tc03_g030320 . . . . .
. . . . . . . .
. . . . . .
Vv02g0858 . . . . . . .
. . . . . . . .
. . . . .
Vv02g0859 . . . . . . .
. . . . . . . .
. . . . .
Vv02g0860 . . . . . . .
. . . . . . . .

. . . . .
Vv02g0861 . . . . . . .
. . . . . . . .
. . . . .
Vv02g0862 . . . . . . .
. . . . . . . .
. . . . .
Vv02g0863 . . . . . . .
. . . . . . . .
. . . . .
Vv02g0864 . . . . . . .
. . . . . . . .
. . . . .
Vv02g0865 . . . . . . .
. . . . . . . .
. . . . .
Vv02g0866 . . . . . . .
. . . . . . . .
. . . . .
Vv02g0867 . . . . . . .
. . . . . . . .
. . . . .
Vv02g0868 . . . . . . .
. . . . . . . .
. . . . .
Vv02g0869 . . . . . . .
. . . . . . . .
. . . . .
Vv02g0870 . . . . . . .
. . . . . . . .
. . . . .
Vv02g0871 . . . . . . .
. . . . . . . .
. . . . .
Vv02g0872 . . . . . . .
. . . . . . . .
. . . . .
Vv02g0873 Tc03_g030090 . Gorai.003G186000.1 .
. . . . . . . .
. . . . . . . .
Vv02g0874 . . . . . . .
. . . . . . . .
. . . . .
Vv02g0875 Tc03_g030110 . . . Gorai.
008G223300.1 . . . . . . .
. . . . . . . .
Vv02g0876 Tc03_g030120 Gorai.004G181600.1 . .
. . . . . . . .
. . . . . . . .
Vv02g0877 . . . . . . .

. . . . . . . .
. . . . .
Vv02g0878 Tc03_g030130 Gorai.004G181500.1 . .
Gorai.008G223200.1 . . . . .
. . . . . . . .
. .
Vv02g0879 . . . . . . .
. . . . . . . .
. . . . .
Vv02g0880 . . . . Gorai.008G220300.1
. . . . . . . .
. . . . . . .
Vv02g0881 . . . . . . .
. . . . . . . .
. . . . .
Vv02g0882 Tc03_g030140 Gorai.004G180900.1 Gorai.
003G186200.1 Gorai.007G033600.1 . . . .
. . . . . . . .
. . . .
Vv02g0883 . . . . . . .
. . . . . . . .
. . . . .
Vv02g0884 . . . . . . .
. . . . . . . .
. . . . .
Vv02g0885 . . . . . . .
. . . . . . . .
. . . . .
Vv02g0886 Tc03_g030160 . . Gorai.007G033700.1
. . . . . . . .
. . . . . . . .
Vv02g0887 . . . . . . .
. . . . . . . .
. . . . .
Vv02g0888 Tc03_g030250 . . . . .
. . . . . . . .
. . . . . .
Vv02g0889 . . . . . . .
. . . . . . . .
. . . . .
Vv02g0890 . . . . . . .
. . . . . . . .
. . . . .
Vv02g0891 . . . . . . .
. . . . . . . .
. . . . .
Vv02g0892 . . . . . . .
. . . . . . . .
. . . . .
Vv02g0893 . . . . . . .

. . . . . . . .
. . . . .
Vv02g0894 . . . . . . .
. . . . . . . .
. . . . .
Vv02g0895 . . . . . . .
. . . . . . . .
. . . . .
Vv02g0896 . . . . . . .
. . . . . . . .
. . . . .
Vv02g0897 . . Gorai.003G186500.1 . .
. . . . . . . .
. . . . . . .
Vv02g0898 . . . . . . .
. . . . . . . .
. . . . .
Vv02g0899 . . . . . . .
. . . . . . . .
. . . . .
Vv02g0900 Tc03_g030320 . . . . .
. . . . . . . .
. . . . . .
Vv02g0901 . . . . . . .
. . . . . . . .
. . . . .
Vv02g0902 . . . . . . .
. . . . . . . .
. . . . .
Vv02g0903 Tc03_g030330 . . . . .
. . . . . . . .
. . . . . .
Vv02g0904 . . . . . . .
. . . . . . . .
. . . . .
Vv02g0905 . . . . . . .
. . . . . . . .
. . . . .
Vv02g0906 . . . . . . .
. . . . . . . .
. . . . .
Vv02g0907 . . . . . . .
. . . . . . . .
. . . . .
Vv02g0908 . . . . . . .
. . . . . . . .
. . . . .
Vv02g0909 . . . . . . .
. . . . . . . .
. . . . .

| | | | | | | | | | | | | | | | | | | | | |
|---|---|---|---|---|---|---|---|---|---|---|---|---|---|---|---|---|---|---|---|---|
| Vv02g0910 | . | . | . | . | . | . | . | . | . | . | . | . | . | . | . | . | . | . | . | . |
| Vv02g0911 | . | . | . | . | . | . | . | . | . | . | . | . | . | . | . | . | . | . | . | . |
| Vv02g0912 | . | . | . | . | . | . | . | . | . | . | . | . | . | . | . | . | . | . | . | . |
| Vv02g0913 | . | . | . | . | . | . | . | . | . | . | . | . | . | . | . | . | . | . | . | . |
| Vv02g0914 | . | . | . | . | . | . | . | . | . | . | . | . | . | . | . | . | . | . | . | . |
| Vv02g0915 | . | . | . | . | . | . | . | . | . | . | . | . | . | . | . | . | . | . | . | . |
| Vv02g0916 | . | . | . | . | . | . | . | . | . | . | . | . | . | . | . | . | . | . | . | . |
| Vv02g0917 | . | . | . | . | . | . | . | . | . | . | . | . | . | . | . | . | . | . | . | . |
| Vv02g0918 | . | . | . | . | . | . | . | . | . | . | . | . | . | . | . | . | . | . | . | . |
| Vv02g0919 | . | . | . | . | . | . | . | . | . | . | . | . | . | . | . | . | . | . | . | . |
| Vv02g0920 | . | . | . | . | . | . | . | . | . | . | . | . | . | . | . | . | . | . | . | . |
| Vv02g0921 | . | . | . | . | . | . | . | . | . | . | . | . | . | . | . | . | . | . | . | . |
| Vv02g0922 | . | . | . | . | . | . | . | . | . | . | . | . | . | . | . | . | . | . | . | . |
| Vv02g0923 | . | . | . | . | . | . | . | . | . | . | . | . | . | . | . | . | . | . | . | . |
| Vv02g0924 | . | . | . | . | . | . | . | . | . | . | . | . | . | . | . | . | . | . | . | . |
| Vv02g0925 | . | . | . | . | . | . | . | . | . | . | . | . | . | . | . | . | . | . | . | . |
| Vv02g0926 | . | . | . | . | . | . | . | . | . | . | . | . | . | . | . | | | | | |

. . . . .
Vv02g0927 . . . . . . .
. . . . . . . .
. . . . .
Vv02g0928 . . . . . . .
. . . . . . . .
. . . . .
Vv02g0929 . . . . . . .
. . . . . . . .
. . . . .
Vv02g0930 . . . . . . .
. . . . . . . .
. . . . .
Vv02g0931 . . . . . . .
. . . . . . . .
. . . . .
Vv02g0932 . . . . . . .
. . . . . . . .
. . . . .
Vv02g0933 . . . . . . .
. . . . . . . .
. . . . .
Vv02g0934 . . . . . . .
. . . . . . . .
. . . . .
Vv02g0935 . . . . . . .
. . . . . . . .
. . . . .
Vv02g0936 . . . . . . .
. . . . . . . .
. . . . .
Vv02g0937 . . . . . . .
. . . . . . . .
. . . . .
Vv02g0938 . . . . . . .
. . . . . . . .
. . . . .
Vv02g0939 . . . . . . .
. . . . . . . .
. . . . .
Vv02g0940 . . . . . . .
. . . . . . . .
. . . . .
Vv02g0941 . . . . . . .
. . . . . . . .
. . . . .
Vv02g0942 . . . . . . .
. . . . . . . .
. . . . .
Vv02g0943 . . . . . . .

| | | | | | | | |
|---|---|---|---|---|---|---|---|
| Vv02g0944 | Tc03_g030510 | | Gorai.004G179300.1 | | | . | . |
| Vv02g0945 | . | . | . | . | . | . | . |
| Vv02g0946 | . | . | . | . | . | . | . |
| Vv02g0947 | . | . | . | . | . | . | . |
| Vv02g0948 | . | . | . | . | . | . | . |
| Vv02g0949 | . | . | . | . | . | . | . |
| Vv02g0950 | . | . | . | . | . | . | . |
| Vv02g0951 | . | . | . | . | . | . | . |
| Vv02g0952 | . | Gorai.004G179600.1 | | | . | . | . |
| Vv02g0953 | . | . | . | . | . | . | . |
| Vv02g0954 | . | . | . | . | . | . | . |
| Vv02g0955 | . | . | . | . | . | . | . |
| Vv02g0956 | . | . | . | . | . | . | . |
| Vv02g0957 | . | . | . | . | . | . | . |
| Vv02g0958 | . | . | . | . | . | . | . |
| Vv02g0959 | . | . | . | . | . | . | . |

Vv02g0960 . . . . . . .
. . . . . . . .
. . . . .
Vv02g0961 Tc03_g030490 . . . . .
. . . . . . . .
. . . . . .
Vv02g0962 . . . . . . .
. . . . . . . .
. . . . .
Vv02g0963 . . . . . . .
. . . . . . . .
. . . . .
Vv02g0964 . . . . . . .
. . . . . . . .
. . . . .
Vv02g0965 Tc03_g030480 . . . . .
. . . . . . . .
. . . . . .
Vv02g0966 . . . . . . .
. . . . . . . .
. . . . .
Vv02g0967 . . . . . . .
. . . . . . . .
. . . . .
Vv02g0968 . . . . . . .
. . . . . . . .
. . . . .
Vv02g0969 . . . . . . .
. . . . . . . .
. . . . .
Vv02g0970 . . . . . . .
. . . . . . . .
. . . . .
Vv02g0971 . . . . . . .
. . . . . . . .
. . . . .
Vv02g0972 . . . . . . .
. . . . . . . .
. . . . .
Vv02g0973 Tc03_g030470 Gorai.004G179700.1 . .
. . . . . . . .
. . . . . . . .
Vv02g0974 Tc03_g018020 . . . . .
. . . . . . . .
. . . . . .
Vv02g0975 Tc03_g018220 . Gorai.006G060900.1 .
. Gorai.006G189600.1 . . . .
. . . . . . . .
. .
Vv02g0976 Tc03_g030460 . . . . .

. . . . . . . .
. . . . . .
Vv02g0977 . . . . . . .
. . . . . . . .
. . . . .
Vv02g0978 Tc03_g030450 Gorai.004G179800.1 . .
. . . . . . . .
. . . . . . . .
Vv02g0979 . . . . . . .
. . . . . . . .
. . . .
Vv02g0980 . Gorai.004G179900.1 . . .
. . . . . . . .
. . . . . . .
Vv02g0981 Tc03_g030440 Gorai.004G180000.1 . .
. . . . . . . .
. . . . . . . .
Vv02g0982 Tc03_g030430 Gorai.004G180100.1 . .
. . . . . . . .
. . . . . . . .
Vv02g0983 . . . . . . .
. . . . . . . .
. . . . .
Vv02g0984 . . . . . . .
. . . . . . . .
. . . . .
Vv02g0985 Tc03_g018360 . Gorai.006G064000.1 .
. Gorai.006G192400.1 . . . .
. . . . . . . .
. .
Vv02g0986 . . . . . . .
. . . . . . . .
. . . . .
Vv02g0987 . . . . . . .
. . . . . . . .
. . . . .
Vv02g0988 . . . . . . .
. . . . . . . .
. . . . .
Vv02g0989 . . . . . . .
. . . . . . . .
. . . . .
Vv02g0990 . . . . . . .
. . . . . . . .
. . . . .
Vv02g0991 . . . . . . .
. . . . . . . .
. . . . .
Vv02g0992 . . . . . . .
. . . . . . . .

| | | | | | | | |
|---|---|---|---|---|---|---|---|
| Vv02g0993 | . | . | . | . | . | . | . |
| Vv02g0994 | . | . | . | . | . | . | . |
| Vv02g0995 | . | . | . | . | . | . | . |
| Vv02g0996 | . | . | . | . | . | . | . |
| Vv02g0997 | . | . | . | . | . | . | . |
| Vv02g0998 | . | . | . | . | . | . | . |
| Vv02g0999 | . | . | . | . | . | . | . |
| Vv02g1000 | . | . | . | . | . | . | . |
| Vv02g1001 | . | . | . | . | . | . | . |
| Vv02g1002 | . | . | . | . | . | . | . |
| Vv02g1003 | . | . | . | . | . | . | . |
| Vv02g1004 | . | . | . | . | . | . | . |
| Vv02g1005 | . | . | . | . | . | . | . |
| Vv02g1006 | . | . | . | . | . | . | . |
| Vv02g1007 | . | . | . | . | . | . | . |
| Vv02g1008 | . | . | . | . | . | . | . |
| Vv02g1009 | . | . | . | . | . | . | . |

| | | | | | | | |
|---|---|---|---|---|---|---|---|
| . | . | . | . | . | . | . | . |
| . | . | . | . | . | | | |
| Vv02g1010 | . | . | . | . | . | . | . |
| . | . | . | . | . | . | . | . |
| . | . | . | . | . | | | |
| Vv02g1011 | . | . | . | . | . | . | . |
| . | . | . | . | . | . | . | . |
| . | . | . | . | . | | | |
| Vv02g1012 | . | . | . | . | . | . | . |
| . | . | . | . | . | . | . | . |
| . | . | . | . | . | | | |
| Vv02g1013 | . | . | . | . | . | . | . |
| . | . | . | . | . | . | . | . |
| . | . | . | . | . | | | |
| Vv02g1014 | . | Gorai.004G180200.1 | | | . | . | . |
| . | . | . | . | . | . | . | . |
| . | . | . | . | . | . | . | |
| Vv02g1015 | . | . | . | . | . | . | . |
| . | . | . | . | . | . | . | . |
| . | . | . | . | . | | | |
| Vv02g1016 | . | . | . | . | . | . | . |
| . | . | . | . | . | . | . | . |
| . | . | . | . | . | | | |
| Vv02g1017 | . | . | . | . | . | . | . |
| . | . | . | . | . | . | . | . |
| . | . | . | . | . | | | |
| Vv02g1018 | . | . | . | . | . | . | . |
| . | . | . | . | . | . | . | . |
| . | . | . | . | . | | | |
| Vv02g1019 | . | . | . | . | . | . | . |
| . | . | . | . | . | . | . | . |
| . | . | . | . | . | | | |
| Vv02g1020 | . | . | . | . | . | . | . |
| . | . | . | . | . | . | . | . |
| . | . | . | . | . | | | |
| Vv02g1021 | . | . | . | . | . | . | . |
| . | . | . | . | . | . | . | . |
| . | . | . | . | . | | | |
| Vv02g1022 | . | . | . | . | . | . | . |
| . | . | . | . | . | . | . | . |
| . | . | . | . | . | | | |
| Vv02g1023 | . | . | . | . | . | . | . |
| . | . | . | . | . | . | . | . |
| . | . | . | . | . | | | |
| Vv02g1024 | . | . | . | . | . | . | . |
| . | . | . | . | . | . | . | . |
| . | . | . | . | . | | | |
| Vv02g1025 | . | . | . | . | . | . | . |
| . | . | . | . | . | . | . | . |
| . | . | . | . | . | | | |

Vv02g1026 . . . . . . .
. . . . . . . .
. . . . .
Vv02g1027 . . . . . . .
. . . . . . . .
. . . . .
Vv02g1028 . . . . . . .
. . . . . . . .
. . . . .
Vv02g1029 . . . . . . .
. . . . . . . .
. . . . .
Vv02g1030 . . . . . . .
. . . . . . . .
. . . . .
Vv02g1031 . . . . . . .
. . . . . . . .
. . . . .
Vv02g1032 . . . . . . .
. . . . . . . .
. . . . .
Vv02g1033 . . . . . . .
. . . . . . . .
. . . . .
Vv02g1034 . . . . . . .
. . . . . . . .
. . . . .
Vv02g1035 . . . . . . .
. . . . . . . .
. . . . .
Vv02g1036 . . . . . . .
. . . . . . . .
. . . . .
Vv02g1037 . . . . . . .
. . . . . . . .
. . . . .
Vv02g1038 . . . . . . .
. . . . . . . .
. . . . .
Vv02g1039 . . . . . . .
. . . . . . . .
. . . . .
Vv02g1040 . . . . . . .
. . . . . . . .
. . . . .
Vv02g1041 . . . . . . .
. . . . . . . .
. . . . .
Vv02g1042 . . . . . . .
. . . . . . . .

| | | | | | | | |
|---|---|---|---|---|---|---|---|
| . | . | . | . | . | | | |
| Vv02g1043 | . | . | . | . | . | . | . |
| . | . | . | . | . | . | . | . |
| . | . | . | . | . | | | |
| Vv02g1044 | . | . | . | . | . | . | . |
| . | . | . | . | . | . | . | . |
| . | . | . | . | . | | | |
| Vv02g1045 | . | . | . | . | . | . | . |
| . | . | . | . | . | . | . | . |
| . | . | . | . | . | | | |
| Vv02g1046 | . | . | . | . | . | . | . |
| . | . | . | . | . | . | . | . |
| . | . | . | . | . | | | |
| Vv02g1047 | . | . | . | . | . | . | . |
| . | . | . | . | . | . | . | . |
| . | . | . | . | . | | | |
| Vv02g1048 | . | . | . | . | . | . | . |
| . | . | . | . | . | . | . | . |
| . | . | . | . | . | | | |
| Vv02g1049 | . | . | . | . | . | . | . |
| . | . | . | . | . | . | . | . |
| . | . | . | . | . | | | |
| Vv02g1050 | . | . | . | . | . | . | . |
| . | . | . | . | . | . | . | . |
| . | . | . | . | . | | | |
| Vv02g1051 | . | . | . | . | . | . | . |
| . | . | . | . | . | . | . | . |
| . | . | . | . | . | | | |
| Vv02g1052 | . | . | . | . | . | . | . |
| . | . | . | . | . | . | . | . |
| . | . | . | . | . | | | |
| Vv02g1053 | . | . | . | . | . | . | . |
| . | . | . | . | . | . | . | . |
| . | . | . | . | . | | | |
| Vv02g1054 | . | . | . | . | . | . | . |
| . | . | . | . | . | . | . | . |
| . | . | . | . | . | | | |
| Vv02g1055 | . | . | . | . | . | . | . |
| . | . | . | . | . | . | . | . |
| . | . | . | . | . | | | |
| Vv02g1056 | . | . | . | . | . | . | . |
| . | . | . | . | . | . | . | . |
| . | . | . | . | . | | | |
| Vv02g1057 | . | . | . | . | . | . | . |
| . | . | . | . | . | . | . | . |
| . | . | . | . | . | | | |
| Vv02g1058 | . | . | . | . | . | . | . |
| . | . | . | . | . | . | . | . |
| . | . | . | . | . | | | |
| Vv02g1059 | . | . | . | . | . | . | . |

```
          .      .      .      .      .      .      .      .
          .      .      .      .      .
Vv02g1060        .      .      .      .      .      .      .
          .      .      .      .      .      .      .      .
          .      .      .      .      .
Vv02g1061        .      .      .      .      .      .      .
          .      .      .      .      .      .      .      .
          .      .      .      .      .
Vv02g1062        .      .      .      .      .      .      .
          .      .      .      .      .      .      .      .
          .      .      .      .      .
Vv02g1063        .      .      .      .      .      .      .
          .      .      .      .      .      .      .      .
          .      .      .      .      .
Vv02g1064        .      .      .      .      .      .      .
          .      .      .      .      .      .      .      .
          .      .      .      .      .
Vv02g1065        .      .      .      .      .      .      .
          .      .      .      .      .      .      .      .
          .      .      .      .      .
Vv02g1066        .      .      .      .      .      .      .
          .      .      .      .      .      .      .      .
          .      .      .      .      .
Vv02g1067        .      .      .      .      .      .      .
          .      .      .      .      .      .      .      .
          .      .      .      .      .
Vv02g1068        .      .      .      .      .      .      .
          .      .      .      .      .      .      .      .
          .      .      .      .      .
Vv02g1069        .      .      .      .      .      .      .
          .      .      .      .      .      .      .      .
          .      .      .      .      .
Vv02g1070        .      .      .      .      .      .      .
          .      .      .      .      .      .      .      .
          .      .      .      .      .
Vv02g1071        .      .      .      .      .      .      .
          .      .      .      .      .      .      .      .
          .      .      .      .      .
Vv02g1072        .      .      .      .      .      .      .
          .      .      .      .      .      .      .      .
          .      .      .      .      .
Vv02g1073        .      .      .      .      .      .      .
          .      .      .      .      .      .      .      .
          .      .      .      .      .
Vv02g1074        .      .      .      .      .      .      .
          .      .      .      .      .      .      .      .
          .      .      .      .      .
Vv02g1075        .      .      .      .      .      .      .
          .      .      .      .      .      .      .      .
          .      .      .      .      .
```

Vv02g1076 . . . . . . .
. . . . . . . .
. . . . .
Vv02g1077 . . . . . .
Vv0Unrg2494 . . . . . . .
. . . . . .
Vv02g1078 . . . . . . .
. . . . . . . .
. . . . .
Vv02g1079 . . . . . . .
. . . . . . . .
. . . . .
Vv02g1080 . . . . . . .
. . . . . . . .
. . . . .
Vv02g1081 . . . . . . .
. . . . . . . .
. . . . .
Vv02g1082 . . . . . . .
. . . . . . . .
. . . . .
Vv02g1083 . . . . . . .
. . . . . . . .
. . . . .
Vv02g1084 . . . . . . .
. . . . . . . .
. . . . .
Vv02g1085 Tc03_g014330 . . . . .
Vv0Unrg2482 . . . . . .
. . . . . . .
Vv02g1086 . . . . . . .
. . . . . . . .
. . . . .
Vv02g1087 . . . . . . .
. . . . . . . .
. . . . .
Vv02g1088 Tc03_g014340 . . . . .
Vv0Unrg2481 . . . . . .
. . . . . . .
Vv02g1089 . . . . . . .
. . . . . . . .
. . . . .
Vv02g1090 Tc03_g014350 . . . . .
Vv0Unrg2478 . . . . . .
. . . . . . .
Vv02g1091 . . . . . . .
. . . . . . . .
. . . . .
Vv02g1092 . . . . . .
Vv0Unrg2474 . . . . . . .

| | | | | | | | | |
|---|---|---|---|---|---|---|---|---|
| | . | . | . | . | . | . | | |
| Vv02g1093 | | Tc03_g014360 | | . | . | . | . | . |
| | Vv0Unrg2473 | | . | . | . | . | . | . |
| | . | . | . | . | . | . | . | |
| Vv02g1094 | | . | . | . | . | . | . | |
| Vv0Unrg2471 | | . | . | . | . | . | . | . |
| | . | . | . | . | . | . | | |
| Vv02g1095 | | . | . | . | . | . | . | . |
| | . | . | . | . | . | . | . | . |
| | . | . | . | . | . | | | |
| Vv02g1096 | | . | . | . | . | . | . | . |
| | . | . | . | . | . | . | . | . |
| | . | . | . | . | . | | | |
| Vv02g1097 | | . | . | . | . | . | . | . |
| | . | . | . | . | . | . | . | . |
| | . | . | . | . | . | | | |
| Vv02g1098 | | . | . | . | . | . | . | . |
| | . | . | . | . | . | . | . | . |
| | . | . | . | . | . | | | |
| Vv02g1099 | | . | . | . | . | . | . | . |
| | . | . | . | . | . | . | . | . |
| | . | . | . | . | . | | | |
| Vv02g1100 | | . | . | . | . | . | . | . |
| | . | . | . | . | . | . | . | . |
| | . | . | . | . | . | | | |
| Vv02g1101 | | Tc03_g014470 | | . | . | . | . | . |
| | . | . | . | . | . | . | . | . |
| | . | . | . | . | . | . | | |
| Vv02g1102 | | . | . | . | . | . | . | . |
| | . | . | . | . | . | . | . | . |
| | . | . | . | . | . | | | |
| Vv02g1103 | | . | . | . | . | . | . | . |
| | . | . | . | . | . | . | . | . |
| | . | . | . | . | . | | | |
| Vv02g1104 | | . | . | . | . | . | . | . |
| | . | . | . | . | . | . | . | . |
| | . | . | . | . | . | | | |
| Vv02g1105 | | . | . | . | . | . | . | . |
| | . | . | . | . | . | . | . | . |
| | . | . | . | . | . | | | |
| Vv02g1106 | | . | . | . | . | . | . | . |
| | . | . | . | . | . | . | . | . |
| | . | . | . | . | . | | | |
| Vv02g1107 | | . | . | . | . | . | . | . |
| | . | . | . | . | . | . | . | . |
| | . | . | . | . | . | | | |
| Vv02g1108 | | . | . | . | . | . | . | |
| Vv0Unrg2469 | | . | . | . | . | . | . | . |
| | . | . | . | . | . | . | | |
| Vv02g1109 | | . | . | . | . | . | . | . |

```
          .         .         .         .         .         .         .         .
          .         .         .         .         .
Vv02g1110           .         .         .         .         .         .         .
          .         .         .         .         .         .         .         .
          .         .         .         .         .
Vv02g1111           .         .         .         .         .         .         .
          .         .         .         .         .         .         .         .
          .         .         .         .         .
Vv02g1112           .         .         .         .         .         .         .
          .         .         .         .         .         .         .         .
          .         .         .         .         .
Vv02g1113           Tc03_g010380        .         .         .         .         .
          .         .         .         .         .         .         .         .
          .         .         .         .         .         .
Vv02g1114           .         .         .         .         .         .         .
          .         .         .         .         .         .         .         .
          .         .         .         .         .
Vv02g1115           .         .         .         .         .         .         .
          .         .         .         .         .         .         .         .
          .         .         .         .         .
Vv02g1116           .         .         .         .         .         .         .
          .         .         .         .         .         .         .         .
          .         .         .         .         .
Vv02g1117           .         .         .         .         .         .         .
          .         .         .         .         .         .         .         .
          .         .         .         .         .
Vv02g1118           .         .         .         .         .         .         .
          .         .         .         .         .         .         .         .
          .         .         .         .         .
Vv02g1119           .         .         .         .         .         .         .
          .         .         .         .         .         .         .         .
          .         .         .         .         .
Vv02g1120           .         .         .         .         .         .         .
          .         .         .         .         .         .         .         .
          .         .         .         .         .
Vv02g1121           .         .         .         .         .         .         .
          .         .         .         .         .         .         .         .
          .         .         .         .         .
Vv02g1122           .         .         .         .         .         .         .
          .         .         .         .         .         .         .         .
          .         .         .         .         .
Vv02g1123           .         .         .         .         .         .         .
          .         .         .         .         .         .         .         .
          .         .         .         .         .
Vv02g1124           Tc03_g031320        .         .         .         .         .
          .         .         .         .         .         .         .         .
          .         .         .         .         .         .
Vv02g1125           .         .         .         .         .         .         .
          .         .         .         .         .         .         .         .
          .         .         .         .         .
```

Vv02g1126 Tc03_g031310 . . . Gorai.
008G217700.1 . . . . . . .
. . . . . . . .
Vv02g1127 . . . . . . .
. . . . . . . .
. . . . .
Vv02g1128 . . . . . . .
. . . . . . . .
. . . . .
Vv02g1129 Tc03_g010600 . . . . .
. . . . . . . .
. . . . . .
Vv02g1130 Tc03_g031290 . . . . .
. . . . . . . .
. . . . . .
Vv02g1131 Tc03_g010710 . . . . .
. . . . . . . .
. . . . . .
Vv02g1132 . . . . . . .
. . . . . . . .
. . . . .
Vv02g1133 . . . . . . .
. . . . . . . .
. . . . .
Vv02g1134 . . . . . . .
. . . . . . . .
. . . . .
Vv02g1135 . . . . . . .
. . . . . . . .
. . . . .
Vv02g1136 . . . . . . .
. . . . . . . .
. . . . .
Vv02g1137 . . . . . . .
. . . . . . . .
. . . . .
Vv02g1138 . . . . . . .
. . . . . . . .
. . . . .
Vv02g1139 . . . . . . .
. . . . . . . .
. . . . .
Vv02g1140 . . . . . . .
. . . . . . . .
. . . . .
Vv02g1141 . . . . . . .
. . . . . . . .
. . . . .
Vv02g1142 . . . . . . .
. . . . . . . .

| | | | | | | | |
|---|---|---|---|---|---|---|---|
| . | . | . | . | . | | | |
| Vv02g1143 | . | . | . | . | . | . | . |
| . | . | . | . | . | . | . | . |
| . | . | . | . | . | | | |
| Vv02g1144 | . | . | . | . | . | . | . |
| . | . | . | . | . | . | . | . |
| . | . | . | . | . | | | |
| Vv02g1145 | . | . | . | . | . | . | . |
| . | . | . | . | . | . | . | . |
| . | . | . | . | . | | | |
| Vv02g1146 | Tc03_g011010 | | . | . | . | . | . |
| . | . | . | . | . | . | . | . |
| . | . | . | . | . | . | | |
| Vv02g1147 | Tc03_g031250 | | . | . | . | . | . |
| . | . | . | . | . | . | . | . |
| . | . | . | . | . | . | | |
| Vv02g1148 | . | . | . | . | . | . | . |
| . | . | . | . | . | . | . | . |
| . | . | . | . | . | | | |
| Vv02g1149 | . | . | . | . | . | . | . |
| . | . | . | . | . | . | . | . |
| . | . | . | . | . | | | |
| Vv02g1150 | . | . | . | . | . | . | . |
| . | . | . | . | . | . | . | . |
| . | . | . | . | . | | | |
| Vv02g1151 | . | . | . | . | . | . | . |
| . | . | . | . | . | . | . | . |
| . | . | . | . | . | | | |
| Vv02g1152 | . | . | . | . | . | . | . |
| . | . | . | . | . | . | . | . |
| . | . | . | . | . | | | |
| Vv02g1153 | . | Gorai.004G174900.1 | | | . | . | . |
| . | . | . | . | . | . | . | . |
| . | . | . | . | . | . | . | |
| Vv02g1154 | . | . | . | . | . | . | . |
| . | . | . | . | . | . | . | . |
| . | . | . | . | . | | | |
| Vv02g1155 | Tc03_g031240 | | Gorai.004G175200.1 | | | . | . |
| . | . | . | . | . | . | . | . |
| . | . | . | . | . | . | . | . |
| Vv02g1156 | Tc03_g031220 | | Gorai.004G175500.1 | | | . | . |
| . | . | . | . | . | . | . | . |
| . | . | . | . | . | . | . | . |
| Vv02g1157 | . | Gorai.004G175600.1 | | | . | . | . |
| . | . | . | . | . | . | . | . |
| . | . | . | . | . | . | . | |
| Vv02g1158 | Tc03_g031210 | | . | . | . | . | . |
| . | . | . | . | . | . | . | . |
| . | . | . | . | . | . | | |
| Vv02g1159 | . | . | . | . | . | . | . |

. . . . . . . .
. . . . .
Vv02g1160 . . . . . . .
. . . . . . . .
. . . . .
Vv02g1161 . Gorai.004G254300.1 . .
Gorai.008G217800.1 . . . . . .
. . . . . . . .
.
Vv02g1162 . Gorai.004G254200.1 . . .
. . . . . . . .
. . . . . . .
Vv02g1163 Tc03_g031200 . . . . .
. . . . . . . .
. . . . . .
Vv02g1164 . . . . . . .
. . . . . . . .
. . . . .
Vv02g1165 Tc03_g031190 . . . . .
. . . . . . . .
. . . . . .
Vv02g1166 . . . . . . .
. . . . . . . .
. . . . .
Vv02g1167 Tc03_g031180 . . . . .
. . . . . . . .
. . . . . .
Vv02g1168 Tc03_g031170 . . . Gorai.
008G217900.1 . . . . . . .
. . . . . . . .
Vv02g1169 . . . . . . .
. . . . . . . .
. . . . .
Vv02g1170 . . . . . . .
. . . . . . . .
. . . . .
Vv02g1171 . . . . . . .
. . . . . . . .
. . . . .
Vv02g1172 Tc03_g031140 Gorai.004G254000.1 . .
. . . . . . . .
. . . . . . . .
Vv02g1173 Tc03_g031130 . . . . .
. . . . . . . .
. . . . . .
Vv02g1174 Tc03_g031120 . . . Gorai.
008G218100.1 . . . . . . .
. . . . . . . .
Vv02g1175 Tc03_g031110 . . . Gorai.
008G218200.1 . . . . . . .

. . . . . . . .
Vv02g1176 . . . . . . .
. . . . . . . .
. . . . .
Vv02g1177 Tc03_g031100 Gorai.004G176200.1 . .
. . . . . . . .
. . . . . . . .
Vv02g1178 Tc03_g031080 Gorai.004G176300.1 . .
. . . . . . . .
. . . . . . . .
Vv02g1179 Tc03_g031070 Gorai.004G176400.1 . .
. . . . . . . .
. . . . . . . .
Vv02g1180 Tc03_g031060 . . . Gorai.
008G218300.1 . . . . . . .
. . . . . . . .
Vv02g1181 . . . . . . .
. . . . . . . .
. . . . .
Vv02g1182 Tc03_g031030 . . . Gorai.
008G218400.1 . . . . . . .
. . . . . . . .
Vv02g1183 . Gorai.004G176500.1 . . .
. . . . . . . .
. . . . . . .
Vv02g1184 Tc03_g031020 . . . . .
. . . . . . . .
. . . . . .
Vv02g1185 . . . . . . .
. . . . . . . .
. . . . .
Vv02g1186 . . . . . . .
. . . . . . . .
. . . . .
Vv02g1187 . . . . . . .
. . . . . . . .
. . . . .
Vv02g1188 . . . . . . .
. . . . . . . .
. . . . .
Vv02g1189 . . . . . . .
. . . . . . . .
. . . . .
Vv02g1190 . . . . . . .
. . . . . . . .
. . . . .
Vv02g1191 Tc03_g031000 Gorai.004G176600.1 . .
. . . . . . . .
. . . . . . . .
Vv02g1192 . . . . . . .

. . . . . . . .
. . . . .
Vv02g1193 . . . . . . .
. . . . . . . .
. . . . .
Vv02g1194 . . . . . . .
. . . . . . . .
. . . . .
Vv02g1195 . . . . . . .
. . . . . . . .
. . . . .
Vv02g1196 Tc03_g030980 . . . . .
. . . . . . . .
. . . . . .
Vv02g1197 . . . . . . .
. . . . . . . .
. . . . .
Vv02g1198 . . . . . . .
. . . . . . . .
. . . . .
Vv02g1199 Tc03_g030970 . . . . .
. . . . . . . .
. . . . . .
Vv02g1200 Tc03_g030960 . . . . .
. . . . . . . .
. . . . . .
Vv02g1201 . . . . . . .
. . . . . . . .
. . . . .
Vv02g1202 Tc03_g030940 . . . . .
. . . . . . . .
. . . . . .
Vv02g1203 . . . . . . .
. . . . . . . .
. . . . .
Vv02g1204 Tc03_g030930 Gorai.004G177000.1 . .
Gorai.008G218700.1 . . . . .
. . . . . . . .
. .
Vv02g1205 Tc03_g030920 Gorai.004G177100.1 . .
Gorai.008G218800.1 . . . . .
. . . . . . . .
. .
Vv02g1206 Tc03_g030910 Gorai.004G177200.1 . .
Gorai.008G218900.1 . . . . .
. . . . . . . .
. .
Vv02g1207 Tc03_g030890 . . . . .
. . . . . . . .
. . . . . .

Vv02g1208 . . . . . . .
. . . . . . . .
. . . . .
Vv02g1209 . . . . . . .
. . . . . . . .
. . . . .
Vv02g1210 Tc03_g030880 Gorai.004G177500.1 . .
Gorai.008G219000.1 . . . . .
. . . . . . . .
. .
Vv02g1211 . . . . . . .
. . . . . . . .
. . . . .
Vv02g1212 . . . . . . .
. . . . . . . .
. . . . .
Vv02g1213 Tc03_g030870 . . . Gorai.
008G219100.1 . . . . . . .
. . . . . . . .
Vv02g1214 . Gorai.004G177600.1 . . .
. . . . . . . .
. . . . . . .
Vv02g1215 . . . . . . .
. . . . . . . .
. . . . .
Vv02g1216 . . . . . . .
. . . . . . . .
. . . . .
Vv02g1217 Tc03_g030850 . . . Gorai.
008G219400.1 . . . . . . .
. . . . . . . .
Vv02g1218 . . . . . . .
. . . . . . . .
. . . . .
Vv02g1219 . . . . . . .
. . . . . . . .
. . . . .
Vv02g1220 Tc03_g030820 Gorai.004G253900.1 . .
. . . . . . . .
. . . . . . . .
Vv02g1221 . . . . . . .
. . . . . . . .
. . . . .
Vv02g1222 . Gorai.004G251200.1 . . .
. . . . . . . .
. . . . . . .
Vv02g1223 Tc03_g030790 Gorai.004G178100.1 . .
Gorai.008G219500.1 . . . . .
. . . . . . . .
. .

Vv02g1224 . . . . . . .
. . . . . . . .
. . . . .
Vv02g1225 . . . . . . .
. . . . . . . .
. . . . .
Vv02g1226 Tc03_g030770 Gorai.004G178300.1 . .
Gorai.008G219600.1 . . . . .
. . . . . . . .
. .
Vv02g1227 Tc03_g030750 . . . Gorai.
008G219800.1 . . . . . . .
. . . . . . . .
Vv02g1228 . . . . . . .
. . . . . . . .
. . . . .
Vv02g1229 . . . . . . .
. . . . . . . .
. . . . .
Vv02g1230 . . . . . . .
. . . . . . . .
. . . . .
Vv02g1231 . . . . . . .
. . . . . . . .
. . . . .
Vv02g1232 . . . . . . .
. . . . . . . .
. . . . .
Vv02g1233 . . . . . . .
. . . . . . . .
. . . . .
Vv02g1234 . . . . . . .
. . . . . . . .
. . . . .
Vv02g1235 . . . . . . .
. . . . . . . .
. . . . .
Vv02g1236 . . . . . . .
. . . . . . . .
. . . . .
Vv02g1237 . . . . . . .
. . . . . . . .
. . . . .
Vv03g0001 . . . . . . .
. . . . . . . .
. . . . .
Vv03g0002 Tc02_g007890 . . . . .
. . . . . . . .
. . . . . .
Vv03g0003 . . . . . . .

. . . . . . . .
. . . . .
Vv03g0004 . . . . . . .
. . . . . . . .
. . . . .
Vv03g0005 Tc02_g007620 Gorai.013G034700.1 . .
Gorai.008G031000.1 . . . . .
. . . . . . . .
. .
Vv03g0006 Tc02_g007610 Gorai.013G034600.1 Gorai.
005G246400.1 . Gorai.008G031100.1 . Vv18g1150
Tc08_g002700 . . . . Gorai.
010G007800.1 . . . . . . .
Vv03g0007 . . . . . . .
. . . . . . . .
. . . . .
Vv03g0008 Tc02_g007600 . . . . .
. . . . . . .
Vv04g1216 Tc01_g002450 . . Gorai.007G199400.1
. .
Vv03g0009 Tc02_g007590 Gorai.013G034200.1 Gorai.
005G246500.1 . . . . . . .
. . . . . . . .
. .
Vv03g0010 . . . . . . .
. . . . . . . .
. . . . .
Vv03g0011 Tc02_g007580 . Gorai.005G246600.1 .
. . Vv18g1164 . . . .
. . Vv04g1226 Tc01_g002380 . .
Gorai.007G198900.1 Gorai.003G011900.1 .
Vv03g0012 . . . . . . .
. . . . . . . .
. . . . .
Vv03g0013 Tc02_g007560 . . Gorai.002G004900.1
. Gorai.012G154700.1 . . . .
. . . . . . . .
. .
Vv03g0014 . . . . . . .
. . . . . . . .
. . . . .
Vv03g0015 . Gorai.013G034000.1 Gorai.005G246800.1
. . . Vv18g1174 Tc08_g002840 .
. Gorai.009G197700.1 . . . .
. . . . .
Vv03g0016 Tc02_g007550 . . . . .
Vv18g1175 Tc08_g002850 . . Gorai.
009G197600.1 . Gorai.010G007100.1 . . .
. . . .
Vv03g0017 . . . . . . .

. . . . . . . .
. . . . .
Vv03g0018 . . Gorai.005G246900.1 . .
. . . . . . . .
. . . . . . .
Vv03g0019 . . . . . . .
. . . . . . . .
. . . . .
Vv03g0020 Tc02_g007540 Gorai.013G033800.1 Gorai.
005G247000.1 . . . Vv18g1185 . .
. . . . . . . .
. . .
Vv03g0021 Tc02_g007530 Gorai.013G033700.1 .
Gorai.002G004800.1 . . Vv18g1186
Tc08_g002900 Gorai.002G125700.1 . . . .
. . . . . . .
Vv03g0022 Tc02_g007520 Gorai.013G033600.1 . .
. . . . . . . .
. . . . . . . .
Vv03g0023 Tc02_g007510 Gorai.013G033500.1 Gorai.
005G247100.1 . . . Vv18g1188
Tc08_g002910 Gorai.002G125600.1 Gorai.005G110600.1
Gorai.009G197300.1 . Gorai.010G006800.1 Vv04g1235
Tc01_g002320 . . Gorai.007G198100.1
Gorai.003G011800.1 .
Vv03g0024 . . . . . . .
. . . . . . . .
. . . . .
Vv03g0025 Tc02_g007440 Gorai.013G033400.1 .
Gorai.002G004700.1 . Gorai.012G154400.1 Vv18g1195
Tc08_g002980 . Gorai.005G110700.1 Gorai.
009G197000.1 . Gorai.010G006600.1 . . .
. . . .
Vv03g0026 . . . . . . .
. . . . . . . .
. . . . .
Vv03g0027 Tc02_g007430 Gorai.013G033200.1 .
Gorai.002G004600.1 . Gorai.012G154300.1 . .
. . . . . . . .
. . . .
Vv03g0028 Tc02_g007420 Gorai.013G033100.1 . .
. . . . . . . .
. . . . . . . .
Vv03g0029 Tc02_g007410 . . Gorai.002G004500.1
Gorai.008G031600.1 . . . . .
. . . . . . . .
. .
Vv03g0030 Tc02_g007400 . . . Gorai.
008G031700.1 . . . . . . .
. . . . . . . .

Vv03g0031 Tc02_g007390 . Gorai.005G247500.1 .
. . . . . . . .
. . . . . . . .
Vv03g0032 Tc02_g007350 . Gorai.005G247600.1 .
. . . . . . . .
. . . . . . . .
Vv03g0033 Tc02_g007330 . Gorai.005G247700.1
Gorai.002G004400.1 . . . . . .
. . . . . . . .
. .
Vv03g0034 Tc02_g007310 . Gorai.005G247800.1
Gorai.002G004300.1 . . Vv18g1196 . .
. . . . . . . .
. . .
Vv03g0035 Tc02_g007300 Gorai.013G033000.1 . .
. . . . . . . .
. . . . . . . .
Vv03g0036 . Gorai.013G032900.1 Gorai.005G247900.1
. . . . . . . .
. . Vv04g1244 Tc01_g002210 . .
Gorai.007G197900.1 . .
Vv03g0037 . . . . . . .
. . . . . . . .
. . . . .
Vv03g0038 . . . . . . .
. . . . . . Vv04g1247
Tc01_g002180 . . . . .
Vv03g0039 . . . . . . .
. . . . . . . .
. . . . .
Vv03g0040 Tc02_g006470 Gorai.013G032800.1 . .
. . . . . . . .
. . . . . . . .
Vv03g0041 Tc02_g006450 Gorai.013G032700.1 Gorai.
005G248000.1 . . . . . . .
. . . Vv04g1249 . .
Gorai.008G064500.1 Gorai.007G197600.1 . .
Vv03g0042 . . . . . . .
. . . . . . . .
. . . . .
Vv03g0043 Tc02_g006440 . Gorai.005G248100.1 .
. . . . . . . .
. . . . . . . .
Vv03g0044 Tc02_g006430 . Gorai.005G248200.1 .
. . . . . . . .
. Vv04g1251 Tc01_g002130 . . .
Gorai.003G011400.1 Gorai.011G099700.1
Vv03g0045 . . Gorai.005G248300.1 . .
. . . . . . . .
. . . . . . .

Vv03g0046	Tc02_g006420	Gorai.013G032600.1	.	.
	Gorai.008G031800.1	.	.	.	.	.
	.	.	.	.	.	.	.	.
	.	.
Vv03g0047	Tc02_g006410	.	.	.	Gorai.
008G031900.1	.	.	.	.	.	.	.
	.	.	.	.	.	.	.	.
Vv03g0048	.	.	.	.	.	.	.
	.	.	.	.	.	.	.	.
	.	.	.	.	.
Vv03g0049	.	.	.	.	.	.	.
	.	.	.	.	.	.	.	.
	.	.	.	.	.
Vv03g0050	Tc02_g006400	Gorai.013G032400.1	.	.
	.	.	.	.	.	.	.	.
	.	Vv04g1260	Tc01_g002090	.	Gorai.
008G064200.1	.	.	.
Vv03g0051	Tc02_g006390	Gorai.013G032300.1	.
Gorai.002G004700.1	.	.	.	.	.	.
	.	.	.	Vv04g1263	Tc01_g002070	.
	Gorai.008G064100.1	Gorai.007G196900.1	Gorai.
003G011200.1	Gorai.011G099600.1
Vv03g0052	Tc02_g006380	.	Gorai.005G248400.1
Gorai.002G004600.1	.	Gorai.012G154300.1	Vv18g1202
	Tc08_g003030	.	.	Gorai.009G196700.1	.
	Gorai.010G006400.1	Vv04g1265	Tc01_g002040	.
	Gorai.008G063800.1	Gorai.007G196800.1	.	.
Vv03g0053	.	.	.	.	.	.	.
	.	.	.	.	.	.	.	.
	.	.	.	.	.
Vv03g0054	.	.	.	.	.	.	.
	.	.	.	.	.	.	.	.
	.	.	.	.	.
Vv03g0055	Tc02_g006360	.	.	.	.	.
	.	.	.	.	.	.	.	.
	.	.	.	.	.	.
Vv03g0056	Tc02_g006350	.	Gorai.005G248600.1	.
	.	.	.	.	.	.	.	.
	.	.	.	.	.	.	.	.
Vv03g0057	Tc02_g006340	.	.	.	Gorai.
008G032200.1	Gorai.012G154200.1	.	.	.	.
	.	.	.	.	.	.	.	.
	.	.
Vv03g0058	.	.	.	.	.	.	.
	.	.	.	.	.	.	.	.
	.	.	.	.	.
Vv03g0059	Tc02_g006330	.	Gorai.005G248700.1	.
	.	Gorai.012G154100.1	.	.	.	.
	.	.	.	.	.	.	.	.
	.	.

Vv03g0060 Tc02_g006320 . Gorai.005G248800.1
Gorai.002G004000.1 . Gorai.012G154000.1 Vv18g1206
Tc08_g003070 . . . . Gorai.
010G006100.1 . . . . . . .
Vv03g0061 Tc02_g006310 Gorai.013G029300.1 .
Gorai.002G003900.1 . Gorai.012G153900.1 . .
. . . . . . . .
. . . .
Vv03g0062 Tc02_g006300 . . . . .
. . . . . . .
Vv04g1271 Tc01_g002000 . . . Gorai.
003G010800.1 .
Vv03g0063 . . . . . . .
. . . . . . . .
. . . . .
Vv03g0064 Tc02_g006270 . Gorai.005G248900.1 .
. . Vv18g1217 Tc08_g003160 . .
. . . . . . . .
. .
Vv03g0065 Tc02_g006260 . . . . .
. . . . . . . .
. . . . . .
Vv03g0066 Tc02_g006250 . Gorai.005G249000.1 .
Gorai.008G032300.1 . . . . .
. . . . . . . .
. .
Vv03g0067 Tc02_g006240 . . . . .
. . . . . . . .
. . . . . .
Vv03g0068 Tc02_g006230 . . . . .
. . . . . . . .
. . . . . .
Vv03g0069 . . . . . . .
. . . . . . . .
. . . . .
Vv03g0070 Tc02_g006210 . . . Gorai.
008G032500.1 . . . . . . .
. . . . . . . .
Vv03g0071 Tc02_g006200 . Gorai.005G249200.1 .
. . . . . . . .
. . . . . . . .
Vv03g0072 . . Gorai.005G249300.1 .
Gorai.008G032600.1 Gorai.012G153800.1 . . .
. . . . . . . .
. . .
Vv03g0073 Tc02_g006190 . . . Gorai.
008G032700.1 . . . . . . .
. Vv04g1287 Tc01_g001860 Gorai.007G048500.1
. . . .
Vv03g0074 . . . . . .

```
Vv18g1248          .        .        .        .        .        .        .
         .         .        .        .        .        .
Vv03g0075          .        .        .        .        .        .        .
         .         .        .        .        .        .        .        .
         .         .        .        .        .
Vv03g0076          .        .        .        .        .        .        .
         .         .        .        .        .        .        .        .
         .         .        .        .        .
Vv03g0077          Tc02_g006180      .        .        .        .        .
         .         .        .        .        .        .        .        .
         .         .        .        .        .        .
Vv03g0078          Tc02_g006170      .        Gorai.005G249400.1        .
         .         .        .        .        .        .        .        .
         .         .        .        .        .        .        .        .
Vv03g0079          Tc02_g006160      .        .        .        .        .
         .         .        .        .        .        .        .        .
         .         .        .        .        .        .
Vv03g0080          Tc02_g006150      .        .        .        .        .
         .         .        .        .        .        .        .        .
         .         .        .        .        .        .
Vv03g0081          Tc02_g006140      .        Gorai.005G249700.1        .
         .         .        .        .        .        .        .        .
         .         .        .        .        .        .        .        .
Vv03g0082          Tc02_g006130      .        .        .        .
Gorai.012G153700.1          .        .        .        .        .        .
         .         .        .        .        .        .        .        .
Vv03g0083          Tc02_g006120      .        Gorai.005G249900.1        .
         .         .        .        .        .        .        .        .
         .         .        .        .        .        .        .        .
Vv03g0084          Tc02_g006110      .        .        .        .
Gorai.012G150800.1          .        .        .        .        .        .
         .         .        .        .        .        .        .        .
Vv03g0085          .        .        .        .        .        .        .
         .         .        .        .        .        .        .        .
         .         .        .        .        .
Vv03g0086          Tc02_g006100      .        .        .        .        .
         .         .        .        .        .        .        .        .
         .         .        .        .        .        .
Vv03g0087          Tc02_g006090      .        .        .        .        .
         .         .        .        .        .        .        .        .
         .         .        .        .        .        .
Vv03g0088          Tc02_g006080      .        .        .        .        .
         .         .        .        .        .        .        .        .
         .         .        .        .        .        .
Vv03g0089          .        .        .        .        .        .        .
         .         .        .        .        .        .        .        .
         .         .        .        .        .
Vv03g0090          Tc02_g006070      .        Gorai.005G250200.1        .
         .         .        .        .        .        .        .        .
         .         .        .        .        .        .        .        .
```

Vv03g0091 Tc02_g006060 . . . . .
. . . . . . . .
. . . . . .
Vv03g0092 Tc02_g006050 . . . . .
. . . . . . . .
. . . . . .
Vv03g0093 Tc02_g006030 . Gorai.005G250700.1 .
. . . . . . . .
. . . . . . . .
Vv03g0094 Tc02_g006010 . . . . .
. . . . . . . .
. . . . . .
Vv03g0095 Tc02_g005990 . Gorai.005G250900.1 .
. . . . . . . .
. . . . . . . .
Vv03g0096 Tc02_g005970 . Gorai.005G251000.1 .
. . . . . . . .
. . . . . . . .
Vv03g0097 Tc02_g005950 . Gorai.005G251100.1 .
. . . . . . . .
. . . . . . . .
Vv03g0098 Tc02_g005930 . . . . .
. . . . . . . .
. . . . . .
Vv03g0099 Tc02_g005910 . Gorai.005G251300.1 .
. . . . . . . .
. . . . . . . .
Vv03g0100 Tc02_g005880 . Gorai.005G251400.1 .
. . . . . . . .
. . . . . . . .
Vv03g0101 Tc02_g005860 . Gorai.005G251500.1 .
. . . . . . . .
. . . . . . . .
Vv03g0102 Tc02_g005840 . Gorai.005G251600.1 .
. . . . . . . .
. . . . . . . .
Vv03g0103 . . . . . . .
. . . . . . . .
. . . . .
Vv03g0104 Tc02_g005820 . Gorai.005G251700.1 .
. . . . . . . .
. . . . . . . .
Vv03g0105 Tc02_g005800 . . . . .
. . . . . . . .
. . . . . .
Vv03g0106 . . . . . .
Vv18g1256 Tc08_g003470 . . . . .
. . . . . . .
Vv03g0107 . . . . . .
Vv18g1260 . . . Gorai.009G195300.1 .

Gorai.010G005100.1 . . . . .
. .
Vv03g0108 . . Gorai.005G251800.1 .
Gorai.008G032900.1 . . . . . .
. . . . . . . .
.
Vv03g0109 Tc02_g005740 . . . . .
. . . . . . . .
. . . . . .
Vv03g0110 Tc02_g005730 . . . . .
. . . . . . . .
. . . . . .
Vv03g0111 . . . . . . .
. . . . . . . .
. . . . .
Vv03g0112 Tc02_g005660 . . Gorai.002G006300.1
Gorai.008G033200.1 . . . . .
. . . . . . . .
. .
Vv03g0113 Tc02_g005650 . Gorai.005G251900.1 .
. . . . . . . .
. . . . . . . .
Vv03g0114 Tc02_g005640 . . Gorai.002G005700.1
Gorai.008G033300.1 . . . . .
. . . . . . . .
. .
Vv03g0115 Tc02_g005630 Gorai.002G192600.1 Gorai.
005G252000.1 . Gorai.008G033400.1 . Vv18g1275
Tc08_g003580 . . . . Gorai.
010G004300.1 . . . . . . .
Vv03g0116 Tc02_g005620 . Gorai.005G252100.1 .
. . Vv18g1276 Tc08_g003590 Gorai.
002G123500.1 . . . . . . .
. . . .
Vv03g0117 Tc02_g005610 Gorai.002G192700.1 . .
. . . . . . . .
. . . . . . . .
Vv03g0118 . . . . . . .
. . . . . . . .
. . . . .
Vv03g0119 . . . . . . .
. . . . . . . .
. . . . .
Vv03g0120 . . . . . . .
. . . . . . . .
. . . . .
Vv03g0121 . . . . . . .
. . . . . . . .
. . . . .
Vv03g0122 . . . . . . .

. . . . . . . .
. . . . .
Vv03g0123 . . . . . . .
. . . . . . . .
. . . . .
Vv03g0124 . . . . . . .
. . . . . . . .
. . . . .
Vv03g0125 . . . . . . .
. . . . . . . .
. . . . .
Vv03g0126 . . . . . . .
. . . . . . . .
. . . . .
Vv03g0127 . . . . . . .
. . . . . . . .
. . . . .
Vv03g0128 . . . . . . .
. . . . . . . .
. . . . .
Vv03g0129 . . . . . . .
. . . . . . . .
. . . . .
Vv03g0130 . . . . . . .
. . . . . . . .
. . . . .
Vv03g0131 . . . . . . .
. . . . . . . .
. . . . .
Vv03g0132 . . . . . . .
. . . . . . . .
. . . . .
Vv03g0133 . . . . . . .
. . . . . . . .
. . . . .
Vv03g0134 . . . . . . .
. . . . . . . .
. . . . .
Vv03g0135 . . . . . . .
. . . . . . . .
. . . . .
Vv03g0136 . . . . . . .
. . . . . . . .
. . . . .
Vv03g0137 . . . . . . .
. . . . . . . .
. . . . .
Vv03g0138 . . . . . . .
. . . . . . . .
. . . . .

Vv03g0139 . . . . . . .
. . . . . . . .
. . . . .
Vv03g0140 . . . . . . .
. . . . . . . .
. . . . .
Vv03g0141 . . . . . . .
. . . . . . . .
. . . . .
Vv03g0142 Tc02_g005560 Gorai.002G192900.1 . .
. . . . . . . .
. . . . . . . .
Vv03g0143 Tc02_g005550 . Gorai.005G252200.1 .
. . . . . . . .
. Vv04g1307 Tc01_g001660 . .
Gorai.007G194400.1 Gorai.003G009400.1 .
Vv03g0144 . . . . . . .
. . . . . . . .
. . . . .
Vv03g0145 Tc02_g005540 . Gorai.005G252300.1 .
. . . . . . . .
. . . . . . . .
Vv03g0146 Tc02_g005530 Gorai.002G193400.1 Gorai.
005G252400.1 . Gorai.008G033900.1 . . .
. . . . . . . .
. . . .
Vv03g0147 . . . . . . .
. . . . . . . .
. . . . .
Vv03g0148 . . . . . . .
. . . . . . . .
. . . . .
Vv03g0149 . Gorai.002G193500.1 . . .
. . . . . . . .
. . . . . . .
Vv03g0150 . . . . . . .
. . . . . . . .
. . . . .
Vv03g0151 . . . . . . .
. . . . . . . .
. . . . .
Vv03g0152 Tc02_g005520 . . . . .
. . . . . . . .
. . . . . .
Vv03g0153 . . . . . . .
. . . . . . . .
. . . . .
Vv03g0154 . . . . . . .
. . . . . . . .
. . . . .

Vv03g0155 Tc02_g005500 . . . Gorai.
008G034000.1 . . . . . . .
. . . . . . . .
Vv03g0156 Tc02_g005490 Gorai.002G193600.1 .
Gorai.002G003800.1 Gorai.008G034100.1 . Vv18g1285
. . . . . . . .
. . . . .
Vv03g0157 Tc02_g005480 . Gorai.005G252500.1 .
Gorai.008G034300.1 . . . . .
. . . . . . . .
. .
Vv03g0158 . . . . . . .
. . . . . . . .
. . . . .
Vv03g0159 Tc02_g005470 . Gorai.005G252600.1 .
Gorai.008G034500.1 Gorai.012G153000.1 . .
. . . . . . . .
. . . .
Vv03g0160 Tc02_g005460 Gorai.002G193700.1 . .
. . . . . . . .
. . . . . . . .
Vv03g0161 Tc02_g005450 . . . . .
. . . . . . . .
. . . . . .
Vv03g0162 Tc02_g005440 Gorai.002G193800.1 . .
. . . . . . . .
. . . . . . . .
Vv03g0163 Tc02_g005430 . . . . .
. . . . . . . .
. . . . . .
Vv03g0164 Tc02_g005420 Gorai.002G193900.1 . .
. . . . . . . .
. . . . . . . .
Vv03g0165 . . . . . . .
. . . . . . . .
. . . . .
Vv03g0166 . . . . . . .
. . . . . . . .
. . . . .
Vv03g0167 . Gorai.002G194000.1 . . .
. . . . . . . .
. . . . . . .
Vv03g0168 . Gorai.002G193900.1 . . .
. . . . . . . .
. . . . . . .
Vv03g0169 . Gorai.002G193800.1 . . .
. . . . . . . .
. . . . . . .
Vv03g0170 Tc02_g005320 Gorai.002G194900.1 . .
. . . . . . . .

. . . . . . . .
Vv03g0171 . . . . . . .
. . . . . . . .
. . . . .
Vv03g0172 Tc02_g005290 . . . Gorai.
008G034600.1 . . . . . . .
. . . . . . . .
Vv03g0173 Tc02_g005280 . Gorai.005G252900.1 .
. . . . . . . .
. . . . . . . .
Vv03g0174 Tc02_g005270 . Gorai.005G253000.1 .
Gorai.008G034800.1 . . . . .
. . . . . . . .
. .
Vv03g0175 Tc02_g005260 . . . Gorai.
008G034900.1 . . . . . . .
. . . . . . . .
Vv03g0176 . . . . . . .
. . . . . . . .
. . . . .
Vv03g0177 Tc02_g005250 . Gorai.005G253100.1 .
. . . . . . . .
. . . . . . . .
Vv03g0178 Tc02_g005240 . . Gorai.002G003600.1
Gorai.008G035100.1 . Vv04g1310
Tc01_g001620 Gorai.007G048700.1 Gorai.008G062700.1
Gorai.007G193900.1 . . . . . .
. . .
Vv03g0179 . Gorai.002G195100.1 Gorai.005G253200.1
Gorai.002G003500.1 . . Vv04g1313 .
. Gorai.008G062500.1 Gorai.007G193700.1 .
. . . . . . . .
Vv03g0180 Tc02_g005220 Gorai.002G195200.1 Gorai.
005G253300.1 . . . . . . .
. . . . . . . .
. .
Vv03g0181 Tc02_g005210 Gorai.002G195300.1 . .
. . Vv18g1256 Tc08_g003470 . .
. . . . . . . .
. .
Vv03g0182 . . . . . . .
. . . . . . . .
. . . . .
Vv03g0183 . . . . . . .
. . . . . . . .
. . . . .
Vv03g0184 . . . . . . .
. . . . . . . .
. . . . .
Vv03g0185 . . . . . . .

. . . . . . . .
. . . . .
Vv03g0186 Tc02_g005180 Gorai.002G195600.1 . .
. . . . . . . .
. . . . . . . .
Vv03g0187 Tc02_g005170 Gorai.002G195700.1 Gorai.
005G253600.1 . . . . . . .
. . . . . . . .
. .
Vv03g0188 . Gorai.002G193600.1 . Gorai.
002G003400.1 . Gorai.012G153200.1 Vv18g1285 .
. . . . . . . .
. . . .
Vv03g0189 . Gorai.002G195800.1 . . .
. . . . . . . .
. . . . . . .
Vv03g0190 Tc02_g005140 Gorai.002G195900.1 Gorai.
005G253800.1 . Gorai.008G035300.1 . . .
. . . . . . . .
. . . .
Vv03g0191 Tc02_g005130 . . . . .
. . . . . . . .
. . . . . .
Vv03g0192 . . . . . . .
. . . . . . . .
. . . . .
Vv03g0193 Tc02_g005120 . . . . .
. . . . . . . .
. . . . . .
Vv03g0194 Tc02_g005110 . . . Gorai.
008G035600.1 . . . . . . .
. Vv04g1329 Tc01_g001480 . . .
Gorai.003G008500.1 .
Vv03g0195 Tc02_g005100 Gorai.002G196200.1 . .
. . . . . . . .
. . . . . . . .
Vv03g0196 Tc02_g005090 . Gorai.005G253900.1 .
. . . . . . . .
. Vv04g1331 Tc01_g001460 . .
Gorai.007G193100.1 Gorai.003G008300.1 .
Vv03g0197 Tc02_g005080 Gorai.002G196700.1 Gorai.
005G254000.1 . Gorai.008G035700.1 . . .
. . . . . . . .
. . . .
Vv03g0198 Tc02_g005070 Gorai.002G196800.1 Gorai.
005G254100.1 . . . . . . .
. . . . . . . .
. .
Vv03g0199 Tc02_g005060 Gorai.002G191600.1 .
Gorai.002G003300.1 . . . . . .

. . . . . . . .
. .
Vv03g0200 Tc02_g005050 Gorai.002G188800.1 . .
Gorai.008G035900.1 . . . . .
. . . Vv04g1348 . . .
. . .
Vv03g0201 . . . Gorai.002G003200.1 .
. . . . . . . .
Vv04g1349 . . . . . .
Vv03g0202 Tc02_g005040 Gorai.002G197200.1 . .
Gorai.008G036000.1 . . . . .
. . . Vv04g1351 . . .
. . .
Vv03g0203 Tc02_g005030 Gorai.002G197400.1 . .
. . . . . . . .
. . . . . . . .
Vv03g0204 Tc02_g005020 Gorai.002G197500.1 . .
Gorai.008G036100.1 . Vv18g1312
Tc08_g003890 . Gorai.005G112500.1 Gorai.009G193300.1
Gorai.009G269600.1 Gorai.010G002600.1 Vv04g1352
Tc01_g001350 . . Gorai.007G192300.1
Gorai.003G008000.1 .
Vv03g0205 Tc02_g005010 Gorai.002G197600.1 . .
Gorai.008G036200.1 . . . . .
. . . . . . . .
. .
Vv03g0206 Tc02_g005000 Gorai.002G197700.1 . .
Gorai.008G036300.1 . . . . .
. . . . . . . .
. .
Vv03g0207 Tc02_g004980 Gorai.002G197800.1 Gorai.
005G254200.1 Gorai.002G003000.1 . . . .
. . . . . . . .
. . . .
Vv03g0208 Tc02_g004970 . . Gorai.002G002900.1
. . . . . . . .
. . . . . . . .
Vv03g0209 Tc02_g004960 Gorai.002G198000.1 . .
. . . . . . . .
. Vv04g1356 Tc01_g001320 . . .
Gorai.003G007800.1 .
Vv03g0210 Tc02_g004940 Gorai.002G198200.1 Gorai.
005G254300.1 Gorai.002G002800.1 . . Vv18g1319
Tc08_g003950 Gorai.002G122300.1 Gorai.005G112600.1
Gorai.009G192800.1 Gorai.009G269400.1 Gorai.
010G002400.1 . . . . . . .
Vv03g0211 Tc02_g004930 Gorai.002G198300.1 . .
. . . . . . . .
. . . . . . . .
Vv03g0212 . Gorai.002G198400.1 . . .

. Vv18g1332 . . . . .
. . . . . . . .
Vv03g0213 . . . Gorai.002G002700.1
Gorai.008G036400.1 . . . . . .
. . . . . . . .
.
Vv03g0214 . . . . . .
Vv18g1335 Tc08_g003990 . . . .
Gorai.010G002100.1 . . . . . .
.
Vv03g0215 . . . . . . .
. . . . . . . .
. . . . .
Vv03g0216 . . . . . . .
. . . . . . . .
. . . . .
Vv03g0217 Tc02_g004900 . . . Gorai.
008G036500.1 . . . . . . .
. . . . . . . .
Vv03g0218 . . . Gorai.002G002600.1
Gorai.008G036700.1 . . . . . .
. . . . . . . .
.
Vv03g0219 Tc02_g004890 Gorai.002G198600.1 . .
. . . . . . . .
. . . . . . . .
Vv03g0220 Tc02_g004830 Gorai.002G198900.1 Gorai.
005G254400.1 Gorai.002G002500.1 . . . .
. . . . . . . .
. . . .
Vv03g0221 Tc02_g004810 Gorai.002G199000.1 . .
. Gorai.012G156000.1 . . . .
. . . . . . . .
. .
Vv03g0222 Tc02_g004790 . . . Gorai.
008G036900.1 . . . . . . .
. . . . . . . .
Vv03g0223 Tc02_g004780 Gorai.002G199100.1 . .
. . . . . . . .
. . . . . . . .
Vv03g0224 Tc02_g004770 . Gorai.005G254600.1
Gorai.002G002400.1 . . . . . .
. . . . . . . .
. .
Vv03g0225 . . . . . . .
. . . . . . . .
. . . . .
Vv03g0226 Tc02_g004760 . . . .
Gorai.012G156100.1 . . . . . .
. . . . . . . .

Vv03g0227 Tc02_g004750 . Gorai.005G254700.1 .
. . . . . . . .
. . . . . . . .
Vv03g0228 Tc02_g004740 Gorai.002G199200.1 . .
Gorai.008G037000.1 . Vv18g0209
Tc08_g004080 . . . . . . .
. . . . .
Vv03g0229 . Gorai.002G199300.1 . . .
. Vv18g0208 . . . . .
. . . . . . . .
Vv03g0230 Tc02_g004730 Gorai.002G199400.1 . .
. Gorai.012G156400.1 . . . .
. . . . . . . .
. .
Vv03g0231 . Gorai.002G199500.1 . . .
Gorai.012G156500.1 . . . . .
. . . . . . . .
.
Vv03g0232 Tc02_g004720 . Gorai.005G254800.1
Gorai.002G002300.1 . . . . . .
. . . . . . . .
. .
Vv03g0233 Tc02_g004670 . Gorai.005G254900.1 .
. . . . . . . .
. . . . . . . .
Vv03g0234 Tc02_g004640 Gorai.002G199700.1 Gorai.
005G255000.1 . Gorai.008G037200.1 Gorai.012G156600.1
. . . . . . . .
. . . . . .
Vv03g0235 Tc02_g004630 Gorai.002G199800.1 . .
. . . . . . . .
. . . . . . . .
Vv03g0236 Tc02_g004620 Gorai.002G199900.1 . .
Gorai.008G037300.1 . . . . .
. . . . . . . .
. .
Vv03g0237 Tc02_g004610 . . . Gorai.
008G037400.1 . . . . . . .
. . . . . . . .
Vv03g0238 . . . . . . .
. . . . . . . .
. . . . .
Vv03g0239 . . . . . . .
. . . . . . . .
. . . . .
Vv03g0240 . . . . . . .
. . . . . . . .
. . . . .
Vv03g0241 . . . . . . .
. . . . . . . .

. . . . .
Vv03g0242 . . . . . . .
. . . . . . . .
. . . . .
Vv03g0243 . . . . . . .
. . . . . . . .
. . . . .
Vv03g0244 . . . . . . .
. . . . . . . .
. . . . .
Vv03g0245 . . . . . .
Vv18g0199 Tc08_g004150 Gorai.002G121100.1 . .
Gorai.009G269100.1 . . . . .
. . .
Vv03g0246 . . . . . . .
. . . . . . . .
. . . . .
Vv03g0247 . . . . . . .
. . . . . . . .
. . . . .
Vv03g0248 . . . . . . .
. . . . . . . .
. . . . .
Vv03g0249 . . . . . . .
. . . . . . . .
. . . . .
Vv03g0250 . . . . . . .
. . . . . . . .
. . . . .
Vv03g0251 Tc02_g004600 Gorai.002G200000.1 . .
Gorai.008G037500.1 . . . . .
. . . . . . . .
. .
Vv03g0252 Tc02_g004550 Gorai.002G200500.1 Gorai.
005G255100.1 Gorai.002G002200.1 . . Vv18g0194
Tc08_g004200 . . . . . .
. . . . . .
Vv03g0253 Tc02_g004540 . Gorai.005G255200.1 .
. . . . . . . .
. . . . . . . .
Vv03g0254 . . . . . . .
. . . . . . . .
. . . . .
Vv03g0255 Tc02_g004530 . . . .
Gorai.012G156700.1 . . . . . .
. . . . . . . .
Vv03g0256 Tc02_g004510 . . . . . .
. . . . . . . .
. . . . . .
Vv03g0257 . . . . . . .

. . . . . . . .
. . . . .
Vv03g0258 . . . . . . .
. . . . . . . .
. . . . .
Vv03g0259 . . . . . .
Vv18g0184 Tc08_g004260 . . Gorai.009G191000.1
Gorai.009G268600.1 . . . . .
. . .
Vv03g0260 Tc02_g004390 Gorai.002G200900.1 Gorai.
005G255500.1 . Gorai.008G037600.1 . Vv18g0183
Tc08_g004270 Gorai.002G120900.1 . Gorai.
009G190900.1 . . . . . . .
. .
Vv03g0261 . . . . . Gorai.
012G156900.1 . . . . . . .
. . . . . . .
Vv03g0262 . . . . . . .
. . . . . . . .
. . . . .
Vv03g0263 . . . . . . .
. . . . . . . .
. . . . .
Vv03g0264 Tc02_g004370 . . . Gorai.
008G037700.1 . . . . . . .
. . . . . . . .
Vv03g0265 Tc02_g004360 . . . Gorai.
008G037800.1 . . . . . . .
. . . . . . . .
Vv03g0266 Tc02_g004350 . . . . .
. . . . . . . .
. . . . . .
Vv03g0267 . . Gorai.005G255600.1 . .
. . . . . . . .
. . . . . . .
Vv03g0268 Tc02_g004340 . . . Gorai.
008G037900.1 . . . . . . .
. . . . . . . .
Vv03g0269 . Gorai.002G201100.1 . . .
. . . . . . . .
. . . . . . .
Vv03g0270 . . . . . . .
. . . . . . . .
. . . . .
Vv03g0271 . . . . . . .
. . . . . . . .
. . . . .
Vv03g0272 Tc02_g004330 . . . . .
Vv18g0174 Tc08_g004330 . . Gorai.
009G190500.1 . . . . . . .

. .
Vv03g0273 Tc02_g004320 Gorai.002G201200.1 .
Gorai.002G002100.1 . Gorai.012G157000.1 . .
. . . . . . . .
. . . .
Vv03g0274 Tc02_g004300 . . . Gorai.
008G038000.1 . . . . . . .
. . . . . . . .
Vv03g0275 Tc02_g004290 Gorai.002G201300.1 . .
. . . . . . . .
. . . . . . . .
Vv03g0276 . . . . . . .
. . . . . . . .
. . . . .
Vv03g0277 Tc02_g004280 . . . Gorai.
008G038100.1 Gorai.012G157100.1 . . . .
. . . . . . . .
. .
Vv03g0278 . . . . Gorai.008G038200.1
Gorai.012G157300.1 . . . . .
. . . . . . . .
.
Vv03g0279 Tc02_g004270 Gorai.002G201600.1 . .
. . . . . . . .
. . . . . . . .
Vv03g0280 . . . . . . .
. . . . . . . .
. . . . .
Vv03g0281 . . . . . . .
. . . . . . . .
. . . . .
Vv03g0282 . . . . . . .
. . . . . . . .
. . . . .
Vv03g0283 . . . . . . .
. . . . . . . .
. . . . .
Vv03g0284 . . . . . . .
. . . . . . . .
. . . . .
Vv03g0285 . . . . . . .
. . . . . . . .
. . . . .
Vv03g0286 . . . . . . .
. . . . . . . .
. . . . .
Vv03g0287 . . . . . . .
. . . . . . . .
. . . . .
Vv03g0288 . . . . . . .

. . . . . . . .
. . . . .
Vv03g0289 . . . . . . .
. . . . . . . .
. . . . .
Vv03g0290 . . . . . . .
. . . . . . . .
. . . . .
Vv03g0291 . . . . . . .
. . . . . . . .
. . . . .
Vv03g0292 . . . . . . .
. . . . . . . .
. . . . .
Vv03g0293 . . . . . . .
. . . . . . . .
. . . . .
Vv03g0294 . . . . . . .
. . . . . . . .
. . . . .
Vv03g0295 . . . . . . .
. . . . . . . .
. . . . .
Vv03g0296 . . . . . . .
. . . . . . . .
. . . . .
Vv03g0297 . . . . . . .
. . . . . . . .
. . . . .
Vv03g0298 . . . . . . .
. . . . . . . .
. . . . .
Vv03g0299 . . . . . . .
. . . . . . . .
. . . . .
Vv03g0300 . . . . . . .
. . . . . . . .
. . . . .
Vv03g0301 Tc02_g012340 . . . . .
. . . . . . . .
. . . . . .
Vv03g0302 . Gorai.002G203900.1 . Gorai.
002G001800.1 Gorai.008G039200.1 . . . .
. . . . . . . .
. . .
Vv03g0303 . Gorai.002G204000.1 . .
Gorai.008G039300.1 . . . . . .
. . . . . . . .
.
Vv03g0304 . Gorai.002G204100.1 . .

```
Gorai.008G039400.1        .        .        .        .        .        .
         .        .       .        .        .        .        .        .
         .
Vv03g0305         .       Gorai.002G204200.1         .        .
Gorai.008G039500.1        .        .        .        .        .        .
         .        .       .        .        .        .        .        .
         .
Vv03g0306         .       Gorai.002G204400.1         .        .
Gorai.008G039600.1        .        .        .        .        .        .
         .        .       .        .        .        .        .        .
         .
Vv03g0307         .       Gorai.002G204500.1         .        .
Gorai.008G039700.1        Gorai.012G157900.1         .        .        .
         .        .       .        .        .        .        .        .
         .        .       .
Vv03g0308         .       Gorai.002G204700.1         .        .
Gorai.008G039800.1        Gorai.012G158100.1         .        .        .
         .        .       .        .        .        .        .        .
         .        .       .
Vv03g0309         .       .        .        .        .        .        .
         .        .       .        .        .        .        .        .
         .        .       .        .        .
Vv03g0310         Tc02_g012230     .        .        .        .        .
         .        .       .        .        .        .        .        .
         .        .       .        .        .        .
Vv03g0311         .       .        .        .        .        .
Vv18g0139         Tc08_g004660     Gorai.002G119100.1         .
Gorai.009G188900.1        .        .        .        .        .        .
         .        .       .
Vv03g0312         .       .        .        .        .        .        .
         .        .       .        .        .        .        .        .
         .        .       .        .        .
Vv03g0313         .       .        .        .        .        .        .
         .        .       .        .        .        .        .        .
         .        .       .        .        .
Vv03g0314         Tc02_g004110     .        .        .        .        .
         .        .       .        .        .        .        .        .
         .        .       .        .        .        .
Vv03g0315         Tc02_g004100     .        Gorai.005G256400.1        .
         .        .       .        .        .        .        .        .
         .        .       .        .        .        .        .        .
Vv03g0316         Tc02_g004090     .        Gorai.005G256500.1        .
         .        .       .        .        .        .        .        .
         .        .       .        .        .        .        .        .
Vv03g0317         Tc02_g004080     .        Gorai.005G256600.1        .
         .        .       .        .        .        .        .        .
         .        .       .        .        .        .        .        .
Vv03g0318         Tc02_g004070     .        Gorai.005G256700.1        .
         .        .       .        .        .        .        .        .
         .        .       .        .        .        .        .        .
```

| | | | | | | | |
|---|---|---|---|---|---|---|---|
| Vv03g0319 | Tc02_g004060 | | . | Gorai.005G256800.1 | | | . |
| . | . | . | . | . | . | . | . |
| . | . | . | . | . | . | . | . |
| Vv03g0320 | Tc02_g004050 | | . | Gorai.005G256900.1 | | | . |
| . | . | . | . | . | . | . | . |
| . | . | . | . | . | . | . | . |
| Vv03g0321 | Tc02_g004040 | | . | Gorai.005G257000.1 | | | . |
| . | . | . | . | . | . | . | . |
| . | . | . | . | . | . | . | . |
| Vv03g0322 | Tc02_g004030 | | . | . | . | . | . |
| . | . | . | . | . | . | . | . |
| . | . | . | . | . | . | | |
| Vv03g0323 | Tc02_g004020 | | . | Gorai.005G257100.1 | | | . |
| . | . | . | . | . | . | . | . |
| . | . | . | . | . | . | . | . |
| Vv03g0324 | Tc02_g004010 | | . | Gorai.005G257200.1 | | | . |
| . | . | . | . | . | . | . | . |
| . | . | . | . | . | . | . | . |
| Vv03g0325 | Tc02_g004000 | | . | . | . | . | . |
| . | . | . | . | . | . | . | . |
| . | . | . | . | . | . | | |
| Vv03g0326 | . | . | . | . | . | . | . |
| . | . | . | . | . | . | . | . |
| . | . | . | . | . | | | |
| Vv03g0327 | . | . | . | . | . | . | . |
| . | . | . | . | . | . | . | . |
| . | . | . | . | . | | | |
| Vv03g0328 | . | . | . | . | . | . | . |
| . | . | . | . | . | . | . | . |
| . | . | . | . | . | | | |
| Vv03g0329 | . | . | . | . | . | . | . |
| . | . | . | . | . | . | . | . |
| . | . | . | . | . | | | |
| Vv03g0330 | . | . | . | . | . | . | . |
| . | . | . | . | . | . | . | . |
| . | . | . | . | . | | | |
| Vv03g0331 | . | . | . | . | . | . | . |
| . | . | . | . | . | . | . | . |
| . | . | . | . | . | | | |
| Vv03g0332 | . | . | . | . | . | . | . |
| . | . | . | . | . | . | . | . |
| . | . | . | . | . | | | |
| Vv03g0333 | Tc02_g012160 | | . | . | . | . | . |
| . | . | . | . | . | . | . | . |
| . | . | . | . | . | . | | |
| Vv03g0334 | Tc02_g011820 | | . | . | . | . | . |
| . | . | . | . | . | . | . | . |
| . | . | . | . | . | . | | |
| Vv03g0335 | Tc02_g011820 | | . | . | . | . | . |
| . | . | . | . | . | . | . | . |

. . . . . .
Vv03g0336 Tc02_g012140 . . . . .
. . . . . . . .
. . . . . .
Vv03g0337 . . . . . . .
. . . . . . . .
. . . . .
Vv03g0338 . . . . . . .
. . . . . . . .
. . . . .
Vv03g0339 . . . . . . .
. . . . . . . .
. . . . .
Vv03g0340 Tc02_g003620 . . . Gorai.
008G040700.1 . . . . . . .
. . . . . . . .
Vv03g0341 . . . . . .
Vv18g0108 Tc08_g004950 Gorai.002G116800.1 Gorai.
005G113900.1 Gorai.009G187800.1 . . . .
. . . . .
Vv03g0342 . . . . . . .
. . . . . . . .
. . . . .
Vv03g0343 . . . . . . .
. . . . . . . .
. . . . .
Vv03g0344 Tc02_g003540 . . . . .
. . . . . . . .
. . . . . .
Vv03g0345 . Gorai.002G205800.1 Gorai.005G258600.1
Gorai.002G000800.1 . . . . .
. . . . . . . .
. . .
Vv03g0346 Tc02_g003530 . . . .
Gorai.012G158800.1 . . . . . .
. . . . . . . .
Vv03g0347 . Gorai.002G206400.1 Gorai.005G258800.1
. Gorai.008G041000.1 . . . .
. . . . . . . .
. . .
Vv03g0348 . . . . . . .
. . . . . . . .
. . . . .
Vv03g0349 Tc02_g003520 Gorai.002G206500.1 Gorai.
005G258900.1 . . . . . . .
. . . . . . . .
. .
Vv03g0350 Tc02_g003510 Gorai.002G206600.1 Gorai.
005G259000.1 . . . . . . .
. . . . . . . .

. .
Vv03g0351 Tc02_g003500 Gorai.002G206700.1 Gorai.
005G259100.1 . Gorai.008G041100.1 . Vv18g0104
Tc08_g005000 . . . . . .
. . . . . .
Vv03g0352 Tc02_g003490 . . . . .
. . . . . . . .
. . . . . .
Vv03g0353 . Gorai.002G206800.1 . . .
. Vv18g0103 Tc08_g005020 Gorai.002G115700.1
. . . . . . . .
. . .
Vv03g0354 Tc02_g012280 . . . Gorai.
008G014300.1 . . . . . . .
. . . . . . . .
Vv03g0355 Tc02_g012290 Gorai.013G063700.1 .
Gorai.002G013600.1 . . . . . .
. . . . . . . .
. .
Vv03g0356 . . . . . . .
. . . . . . . .
. . . . .
Vv03g0357 . . . . . . .
. . . . . . . .
. . . . .
Vv03g0358 . . . . . . .
. . . . . . . .
. . . . .
Vv03g0359 Tc02_g012340 Gorai.013G063800.1 Gorai.
005G217300.1 . . . . . . .
. . . . . . . .
. .
Vv03g0360 . . . . . . .
. . . . . . . .
. . . . .
Vv03g0361 . . . . Gorai.008G014100.1
. Vv18g0097 Tc08_g005080 . Gorai.
005G114300.1 Gorai.009G187000.1 . . . .
. . . . .
Vv03g0362 Tc02_g012360 . . . .
Gorai.012G139400.1 Vv18g0096 Tc08_g005090 Gorai.
002G115300.1 . . . . . . .
. . . .
Vv03g0363 . . . . . . .
. . . . . . . .
. . . . .
Vv03g0364 Tc02_g012390 . Gorai.005G217200.1 .
. Gorai.012G139200.1 . . . .
. . . . . . . .
. .

Vv03g0365 . . . . . .
Vv18g0087 Tc08_g005170 Gorai.002G114800.1 . .
. . . . . . . .
.
Vv03g0366 . . . . . . .
. . . . . . . .
. . . . .
Vv03g0367 Tc02_g012400 Gorai.013G063900.1 Gorai.
005G217100.1 . . . . . . .
. . . . . . . .
. .
Vv03g0368 Tc02_g012430 . Gorai.005G216800.1 .
. Gorai.012G139100.1 . . . .
. . . . . . . .
. .
Vv03g0369 Tc02_g012440 Gorai.013G064100.1 . .
Gorai.008G013900.1 . Vv18g0080
Tc08_g005230 . . Gorai.009G186500.1 . .
. . . . . . .
Vv03g0370 Tc02_g012460 Gorai.013G064200.1 Gorai.
005G216600.1 Gorai.002G014400.1 . Gorai.012G139000.1
. . . . . . . .
. . . . . .
Vv03g0371 Tc02_g012470 . . . Gorai.
008G013800.1 . . . . . . .
. . . . . . . .
Vv03g0372 Tc02_g012480 Gorai.013G064500.1 Gorai.
005G216500.1 . Gorai.008G013700.1 Gorai.012G138900.1
. . . . . . . .
. . . . . .
Vv03g0373 Tc02_g012490 . . Gorai.002G014500.1
. . . . . . . .
. . . . . . . .
Vv03g0374 . . . . . . .
. . . . . . . .
. . . . .
Vv03g0375 Tc02_g012520 Gorai.013G064700.1 . .
. Gorai.012G138800.1 . . . .
. . . . . . . .
. .
Vv03g0376 Tc02_g012530 . . . Gorai.
008G013500.1 Gorai.012G138700.1 Vv18g0060 . .
. . . . . . . .
. . .
Vv03g0377 Tc02_g012540 Gorai.013G064800.1 . .
. . . . . . . .
. . . . . . . .
Vv03g0378 Tc02_g012550 . . . Gorai.
008G013400.1 . . . . . . .
. . . . . . . .

Vv03g0379 Tc02_g012570 . . . .
Gorai.012G138600.1 . . . . . .
. . . . . . . .
Vv03g0380 . . . . . . .
. . . . . . . .
. . . . .
Vv03g0381 Tc02_g012580 Gorai.013G064900.1 Gorai.
005G216200.1 . . Gorai.012G138500.1 . .
. . . . . . . .
. . . .
Vv03g0382 Tc02_g012590 Gorai.013G065000.1 Gorai.
005G216100.1 . . Gorai.012G138300.1 . .
. . . . . . . .
. . . .
Vv03g0383 Tc02_g012600 . . . Gorai.
008G013200.1 . . . . . . .
. . . . . . . .
Vv03g0384 Tc02_g012610 . Gorai.005G216000.1 .
. . . . . . . .
. . . . . . . .
Vv03g0385 Tc02_g012620 . . Gorai.002G014600.1
. . . . . . . .
. . . . . . . .
Vv03g0386 Tc02_g012630 Gorai.013G065100.1 Gorai.
005G215900.1 . Gorai.008G013100.1 Gorai.012G138200.1
. . . . . . . .
. . . . . .
Vv03g0387 . . . . . . .
. . . . . . . .
. . . . .
Vv03g0388 Tc02_g012640 Gorai.013G065200.1 Gorai.
005G215700.1 Gorai.002G014700.1 Gorai.008G013000.1
Gorai.012G138100.1 Vv18g0044 . . . .
. . . . . . . .
.
Vv03g0389 Tc02_g012650 . Gorai.005G215400.1 .
. Gorai.012G138000.1 Vv18g0042 . .
. . . . . . . .
. . .
Vv03g0390 Tc02_g012660 . . . .
Gorai.012G137800.1 . . . . . .
. . . . . . . .
Vv03g0391 . . . . . . .
. . . . . . . .
. . . . .
Vv03g0392 Tc02_g014050 . . . . .
. . . . . . . .
. . . . . .
Vv03g0393 Tc02_g027460 Gorai.013G065300.1 . .
. Gorai.012G137600.1 Vv18g0039 . .

. . . . . . . .
. . .
Vv03g0394 Tc02_g027450 . . . . .
. . . . . . . .
. . . . . .
Vv03g0395 Tc02_g012710 . . . Gorai.
008G012900.1 . Vv18g0025 . . . .
. . . . . . . .
.
Vv03g0396 Tc02_g012720 . . . Gorai.
008G012800.1 . . . . . . .
. . . . . . . .
Vv03g0397 Tc02_g012730 Gorai.013G065400.1 . .
. . . . . . . .
. . . . . . . .
Vv03g0398 Tc02_g012740 Gorai.013G065500.1 . .
. . . . . . . .
. . . . . . . .
Vv03g0399 Tc02_g012770 Gorai.013G065600.1 . .
Gorai.008G012600.1 . . . . .
. . . . . . . .
. .
Vv03g0400 . . . . Gorai.008G012500.1
. . . . . . . .
. . . . . . .
Vv03g0401 . . . . . . .
. . . . . . . .
. . . . .
Vv03g0402 Tc02_g012780 . . . Gorai.
008G012400.1 . . . . . . .
. . . . . . . .
Vv03g0403 . . Gorai.005G215000.1 Gorai.
002G014800.1 . . . . . . .
. . . . . . . .
.
Vv03g0404 Tc02_g012810 . . . . .
. . . . . . . .
. . . . . .
Vv03g0405 . . . . . . .
. . . . . . . .
. . . . .
Vv03g0406 . . . . . . .
. . . . . . . .
. . . . .
Vv03g0407 Tc02_g012820 . . . Gorai.
008G012300.1 . . . . . . .
. . . . . . . .
Vv03g0408 Tc02_g012840 Gorai.013G065700.1 Gorai.
005G214800.1 . Gorai.008G009400.1 Gorai.012G135700.1
. . . . . . . .

. . . . . .
Vv03g0409 . . . . . . .
. . . . . . . .
. . . . .
Vv03g0410 . . . . . . .
. . . . . . . .
. . . . .
Vv03g0411 . . . . . . .
. . . . . . . .
. . . . .
Vv03g0412 . . . . . . .
. . . . . . . .
. . . . .
Vv03g0413 . Gorai.013G034700.1 . .
Gorai.008G031000.1 . . . . . .
. . . . . . . .
.
Vv03g0414 . . Gorai.005G212300.1 .
Gorai.008G030900.1 . . . . . .
. . . . . . . .
.
Vv03g0415 . . . . . . .
. . . . . . . .
. . . . .
Vv03g0416 . . . . . . .
. . . . . . . .
. . . . .
Vv03g0417 Tc02_g014030 . . . . .
. . . . . . . .
. . . . . .
Vv03g0418 . . . . . . .
. . . . . . . .
. . . . .
Vv03g0419 . . Gorai.005G246000.1 . .
. . . . . . . .
. . . . . . .
Vv03g0420 . . . . . . .
. . . . . . . .
. . . . .
Vv03g0421 . Gorai.013G034900.1 . .
Gorai.008G030700.1 . . . . . .
. . . . . . . .
.
Vv03g0422 . . . . . . .
. . . . . . . .
. . . . .
Vv03g0423 Tc02_g006510 Gorai.013G035100.1 . .
Gorai.008G030600.1 Gorai.012G154900.1 Vv18g0004
. . . . . . . .
. . . . .

Vv03g0424 . . . . . . .
. . . . . . . .
. . . . .
Vv03g0425 Tc02_g006520 Gorai.013G035200.1 Gorai.
005G245700.1 . . Gorai.012G155000.1 Vv18g1139
Tc08_g002600 Gorai.002G126500.1 Gorai.005G109600.1
Gorai.009G199300.1 . Gorai.010G008000.1 .
. . . . . .
Vv03g0426 . . . . . . .
. . . . . . . .
. . . . .
Vv03g0427 Tc02_g006530 Gorai.013G035300.1 Gorai.
005G245600.1 . Gorai.008G030400.1 . . .
. . . . . . . .
. . . .
Vv03g0428 Tc02_g006540 Gorai.013G035400.1 Gorai.
005G245500.1 . Gorai.008G030300.1 Gorai.012G155100.1
Vv18g1131 Tc08_g002530 . Gorai.005G109200.1
Gorai.009G199800.1 . . . . .
. . . .
Vv03g0429 Tc02_g006550 Gorai.013G035500.1 . .
Gorai.008G030100.1 . . . . .
. . . . . . . .
. .
Vv03g0430 . . . . . .
Vv18g1130 . . . . . . .
. . . . . .
Vv03g0431 Tc02_g006560 . Gorai.005G245400.1 .
. . Vv18g1129 . . . .
. . . . . . . .
.
Vv03g0432 Tc02_g006600 . Gorai.005G245300.1 .
Gorai.008G030000.1 . . . . .
. . . . . . . .
. .
Vv03g0433 Tc02_g006610 . Gorai.005G245200.1 .
. . Vv18g1124 Tc08_g002470 . .
Gorai.009G200100.1 . Gorai.010G008500.1 .
. . . . . .
Vv03g0434 . . . . . . .
. . . . . . . .
. . . . .
Vv03g0435 Tc02_g006680 Gorai.013G035700.1 Gorai.
005G245000.1 . Gorai.008G029900.1 Gorai.012G155200.1
. . . . . . . .
. . . . . .
Vv03g0436 Tc02_g006690 Gorai.013G035800.1 Gorai.
005G244900.1 . . . Vv18g1118
Tc08_g002410 . . Gorai.009G200400.1 .
Gorai.010G008700.1 . . . . . .

.
Vv03g0437 Tc02_g006700 . Gorai.005G244800.1 .
. . . . . . . .
. . . . . . . .
Vv03g0438 . . . . Gorai.008G029800.1
. . . . . . . .
. . . . . . .
Vv03g0439 Tc02_g006710 . . . . .
. . . . . . . .
. . . . . .
Vv03g0440 . . . . . . .
. . . . . . . .
. . . . .
Vv03g0441 Tc02_g006720 Gorai.013G035900.1 Gorai.
005G244700.1 . Gorai.008G029700.1 . Vv18g1104
Tc08_g002320 . . . . Gorai.
010G009200.1 . . . . . . .
Vv03g0442 Tc02_g006730 . Gorai.005G244600.1 .
. . . . . . . .
. . . . . . . .
Vv03g0443 . . Gorai.005G244500.1 . .
. . . . . . . .
. . . . . . .
Vv03g0444 Tc02_g006740 . . . . .
. . . . . . . .
. . . . . .
Vv03g0445 Tc02_g006750 . . . . .
. . . . . . . .
. . . . . .
Vv03g0446 . . . . . . .
. . . . . . . .
. . . . .
Vv03g0447 . . . . . . .
. . . . . . . .
. . . . .
Vv03g0448 . . . . . . .
. . . . . . . .
. . . . .
Vv03g0449 . . . . . . .
. . . . . . . .
. . . . .
Vv03g0450 . . . . . .
Vv18g1103 Tc08_g002300 Gorai.002G127000.1 . .
. . . . . . . .
.
Vv03g0451 . . . . . . .
. . . . . . . .
. . . . .
Vv03g0452 Tc02_g006770 . . . . .
. . . . . . . .

. . . . . .
Vv03g0453 Tc02_g006800 . Gorai.005G244400.1 .
Gorai.008G029600.1 . . . . .
. . . . . . . .
. .
Vv03g0454 Tc02_g006830 . Gorai.005G244300.1 .
. . Vv18g1101 Tc08_g002280 . .
Gorai.009G201000.1 Gorai.009G272700.1 . .
. . . . . .
Vv03g0455 Tc02_g006850 . . . Gorai.
008G029500.1 Gorai.012G155300.1 . . . .
. . . . . . . .
. .
Vv03g0456 Tc02_g006860 Gorai.013G036400.1 Gorai.
005G244200.1 . Gorai.008G029300.1 . . .
. . . . . . . .
. . . .
Vv03g0457 . . . . . . .
. . . . . . . .
. . . . .
Vv03g0458 . . . . . . .
. . . . . . . .
. . . . .
Vv03g0459 . . . . . . .
. . . . . . . .
. . . . .
Vv03g0460 . . . . . . .
. . . . . . . .
. . . . .
Vv03g0461 Tc02_g006880 Gorai.013G036700.1 . .
. Gorai.012G155400.1 Vv18g1095
Tc08_g002220 Gorai.002G127300.1 . Gorai.009G201200.1
Gorai.009G273000.1 . . . . .
. . .
Vv03g0462 . . . . . . .
. . . . . . . .
. . . . .
Vv03g0463 Tc02_g006920 . . . Gorai.
008G015800.1 . . . . . . .
. . . . . . . .
Vv03g0464 Tc02_g006950 . Gorai.005G244100.1 .
. . Vv18g1090 Tc08_g002160 .
Gorai.005G108000.1 Gorai.009G201600.1 . . .
. . . . . .
Vv03g0465 . . . . . . .
. . . . . . . .
. . . . .
Vv03g0466 . . . . . . .
. . . . . . . .
. . . . .

Vv03g0467 Tc02_g006960 . Gorai.005G244000.1 .
. . . . . . . .
. . . . . . . .
Vv03g0468 Tc02_g006970 Gorai.013G036800.1 Gorai.
005G243900.1 . . . . . . .
. . . . . . . .
. .
Vv03g0469 Tc02_g006980 Gorai.013G036900.1 Gorai.
005G243800.1 . . . . . . .
. . . . . . . .
. .
Vv03g0470 Tc02_g006990 Gorai.013G037000.1 . .
. . . . . . . .
. . . . . . . .
Vv03g0471 . . . . . . .
. . . . . . . .
. . . . .
Vv03g0472 Tc02_g007000 . . . .
Gorai.012G155500.1 . . . . . .
. . . . . . . .
Vv03g0473 Tc02_g007010 Gorai.013G037100.1 Gorai.
005G243700.1 . Gorai.008G028900.1 . . .
. . . . . . . .
. . . .
Vv03g0474 Tc02_g007020 . Gorai.005G243600.1 .
Gorai.008G028800.1 . . . . .
. . . . . . . .
. .
Vv03g0475 Tc02_g007030 Gorai.013G037200.1 . .
. . Vv18g1079 Tc08_g002030 . .
Gorai.009G202400.1 . . . . .
. . . .
Vv03g0476 Tc02_g007050 Gorai.013G037300.1 . .
Gorai.008G028700.1 . . . . .
. . . . . . . .
. .
Vv03g0477 . . . . . . .
. . . . . . . .
. . . . .
Vv03g0478 Tc02_g007060 . Gorai.005G243300.1 .
Gorai.008G028600.1 . . . . .
. . . . . . . .
. .
Vv03g0479 Tc02_g007070 Gorai.013G037400.1 Gorai.
005G243200.1 . . . . . . .
. . . . . . . .
. .
Vv03g0480 Tc02_g007080 . Gorai.005G243100.1 .
. . Vv18g1075 Tc08_g001980 . .
Gorai.009G202900.1 . . . . .

. . . .
Vv03g0481 . . . . . . .
. . . . . . . .
. . . . .
Vv03g0482 Tc02_g007090 Gorai.013G037500.1 Gorai.
005G243000.1 . . . . . . .
. . . . . . . .
. .
Vv03g0483 . . . . . . .
. . . . . . . .
. . . . .
Vv03g0484 . . . . . . .
. . . . . . . .
. . . . .
Vv03g0485 Tc02_g007100 Gorai.013G037600.1 Gorai.
005G240000.1 . Gorai.008G028500.1 . . .
. . . . . . . .
. . . .
Vv03g0486 Tc02_g007110 Gorai.013G037700.1 . .
. . . . . . . .
. . . . . . . .
Vv03g0487 . . . . . . .
. . . . . . . .
. . . . .
Vv03g0488 Tc02_g007120 Gorai.013G037800.1 Gorai.
005G242800.1 . Gorai.008G028300.1 . . .
. . . . . . . .
. . . .
Vv03g0489 . . . . Gorai.008G025600.1
Gorai.012G152400.1 . . . . .
. . . . . . . .
.
Vv03g0490 Tc02_g007130 . Gorai.005G242600.1 .
. . . . . . . .
. . . . . . . .
Vv03g0491 . . . . . . .
. . . . . . . .
. . . . .
Vv03g0492 . . . . . . .
. . . . . . . .
. . . . .
Vv03g0493 . . . . . . .
. . . . . . . .
. . . . .
Vv03g0494 . . . . . . .
. . . . . . . .
. . . . .
Vv03g0495 . . . . . . .
. . . . . . . .
. . . . .

Vv03g0496	Tc02_g007140	.	.	.	.
Gorai.012G152300.1	.	.	.	.	.	.
.	.	.	.	.	.	.	.
Vv03g0497	.	.	.	.	.	.	.
.	.	.	.	.	.	.	.
.	.	.	.	.
Vv03g0498	Tc02_g007160	.	Gorai.005G261700.1	.
.	Gorai.012G152100.1	.	.	.	.
.	.	.	.	.	.	.	.
.	.
Vv03g0499	.	Gorai.013G053000.1	Gorai.005G261800.1
.	.	Gorai.012G149100.1	.	.	.
.	.	.	.	.	.	.	.
.	.	.
Vv03g0500	.	.	.	.	.	.	.
.	.	.	.	.	.	.	.
.	.	.	.	.
Vv03g0501	.	.	.	.	.	.	.
.	.	.	.	.	.	.	.
.	.	.	.	.
Vv03g0502	.	Gorai.013G052900.1	.	.
Gorai.008G018600.1	.	.	.	.	.	.
.	.	.	.	.	.	.	.
.
Vv03g0503	.	.	.	.	.	.	.
.	.	.	.	.	.	.	.
.	.	.	.	.
Vv03g0504	.	.	Gorai.005G228300.1	.	.
Gorai.012G146000.1	.	.	.	.	.
.	.	.	.	.	.	.	.
.
Vv03g0505	.	.	Gorai.005G228400.1	.	.
Gorai.012G146100.1	.	.	.	.	.
.	.	.	.	.	.	.	.
.
Vv03g0506	.	.	.	.	.	.	.
.	.	.	.	.	.	.	.
.	.	.	.	.
Vv03g0507	.	.	.	.	.	.	.
.	.	.	.	.	.	.	.
.	.	.	.	.
Vv03g0508	Tc02_g007240	.	.	.	.	.
.	.	.	.	.	.	.	.
.	.	.	.	.	.
Vv03g0509	.	.	Gorai.005G228600.1	.	.
Gorai.012G146300.1	.	.	.	.	.
.	.	.	.	.	.	.	.
.
Vv03g0510	.	.	.	.	.	Gorai.
012G146400.1	Vv18g1041	Tc08_g001790	.	.	.

```
        .       Gorai.010G011500.1      .       .       .       .
        .       .       .
Vv03g0511       .       Gorai.013G052800.1      .       .
Gorai.008G018900.1      .       Vv18g1036       Tc08_g001770
Gorai.002G129500.1      .       Gorai.009G204300.1      .
Gorai.010G011700.1      .       .       .       .       .       .
        .
Vv03g0512       .       .       Gorai.005G228800.1      .       .
        .       .       .       .       .       .       .       .
        .       .       .       .       .       .       .
Vv03g0513       .       .       .       .       .       .       .
        .       .       .       .       .       .       .       .
        .       .       .       .       .
Vv03g0514       .       .       .       .       .       .       .
        .       .       .       .       .       .       .       .
        .       .       .       .       .
Vv03g0515       .       .       .       .       .       .       .
        .       .       .       .       .       .       .       .
        .       .       .       .       .
Vv03g0516       .       .       .       .       .       .       .
        .       .       .       .       .       .       .       .
        .       .       .       .       .
Vv03g0517       .       Gorai.013G052700.1      .       .       .
        Gorai.012G146500.1      .       .       .       .       .
        .       .       .       .       .       .       .       .
        .
Vv03g0518       .       Gorai.013G052500.1      Gorai.005G228900.1
        .       .       .       .       .       .       .       .
        .       .       .       .       .       .       .       .
        .
Vv03g0519       .       Gorai.013G052400.1      .       .       .
        .       Vv18g1015       Tc08_g001660    .       .       .
        .       Gorai.010G012300.1      .       .       .       .
        .       .       .
Vv03g0520       Tc02_g007480    .       .       .       .
Gorai.012G146700.1      .       .       .       .       .       .
        .       .       .       .       .       .       .       .
Vv03g0521       .       .       .       .       .       .       .
        .       .       .       .       .       .       .       .
        .       .       .       .       .
Vv03g0522       .       .       Gorai.005G229000.1      .       .
        .       .       .       .       .       .       .       .
        .       .       .       .       .       .       .
Vv03g0523       .       Gorai.013G052300.1      Gorai.005G229100.1
        .       Gorai.008G019000.1      .       .       .       .
        .       .       .       .       .       .       .       .
        .       .       .
Vv03g0524       .       .       Gorai.005G229200.1      .       .
        .       .       .       .       .       .       .       .
        .       .       .       .       .       .       .
```

Vv03g0525 . . . . . Gorai.
012G146900.1 . . . . . . .
. . . . . .
Vv03g0526 . Gorai.013G049300.1 Gorai.005G263200.1
. . Gorai.012G163300.1 Vv18g0998
Tc08_g001540 . . . . . . .
. . . . .
Vv03g0527 . Gorai.002G213700.1 . . .
Gorai.012G163400.1 . . . . .
. . . . . . . .
.
Vv03g0528 . Gorai.002G213800.1 . . .
. . . . . . . .
. . . . . . .
Vv03g0529 . . . . . Gorai.
012G163500.1 . . . . . . .
. . . . . . .
Vv03g0530 . . . . . Gorai.
012G163700.1 . . . . . . .
. . . . . . .
Vv03g0531 . . . . Gorai.008G043600.1
. . . . . . . .
. . . . . . .
Vv03g0532 . . Gorai.005G263400.1 .
Gorai.008G043700.1 . . . . . .
. . . . . . . .
.
Vv03g0533 . . . . . . .
. . . . . . . .
. . . . .
Vv03g0534 . Gorai.002G214000.1 . . .
. . . . . . . .
. . . . . . .
Vv03g0535 . . . . . . .
. . . . . . . .
. . . . .
Vv03g0536 . . . . . . .
. . . . . . . .
. . . . .
Vv03g0537 . . . . . . .
. . . . . . . .
. . . . .
Vv03g0538 . . . . . . .
. . . . . . . .
. . . . .
Vv03g0539 . Gorai.002G214200.1 . . .
. . . . . . . .
. . . . . . .
Vv03g0540 . . . . Gorai.008G043800.1
Gorai.012G164000.1 . . . . .

. . . . . . . .
.
Vv03g0541 . . . . . . .
. . . . . . . .
. . . . .
Vv03g0542 . . . . Gorai.008G043900.1
. . . . . . . .
. . . . . . .
Vv03g0543 . . . . . . .
. . . . . . . .
. . . . .
Vv03g0544 . . . . Gorai.008G044100.1
. . . . . . . .
. . . . . . .
Vv03g0545 . . . . . . .
. . . . . . . .
. . . . .
Vv03g0546 Tc02_g007490 . Gorai.005G263800.1 .
Gorai.008G031300.1 Gorai.012G164100.1 . .
. . . . . . . .
. . . .
Vv03g0547 . Gorai.002G214300.1 . . .
Gorai.012G164200.1 . . . . .
. . . . . . . .
.
Vv03g0548 . Gorai.002G214500.1 . .
Gorai.008G019100.1 . . . . . .
. . . . . . . .
.
Vv03g0549 . . . . Gorai.008G044200.1
. . . . . . . .
. . . . . . .
Vv03g0550 . . . . . . .
. . . . . . . .
. . . . .
Vv03g0551 . . Gorai.005G264000.1 . .
. . . . . . . .
. . . . . . .
Vv03g0552 . . . . . . .
. . . . . . . .
. . . . .
Vv03g0553 . . . . . . .
. . . . . . . .
. . . . .
Vv03g0554 . . . . . . .
. . . . . . . .
. . . . .
Vv03g0555 . . . . . . .
. . . . . . . .
. . . . .

Vv03g0556 . . . . . . .
. . . . . . . .
. . . . .
Vv03g0557 . . . . . . .
. . . . . . . .
. . . . .
Vv03g0558 . . . . . . .
. . . . . . . .
. . . . .
Vv03g0559 . . . . . . .
. . . . . . . .
. . . . .
Vv03g0560 . . . . . . .
. . . . . . . .
. . . . .
Vv03g0561 . . . . . . .
. . . . . . . .
. . . . .
Vv03g0562 . . . . . . .
. . . . . . . .
. . . . .
Vv03g0563 . . . . . . .
. . . . . . . .
. . . . .
Vv03g0564 . . . . . . .
. . . . . . . .
. . . . .
Vv03g0565 . . . . . . .
. . . . . . . .
. . . . .
Vv03g0566 . . . . . . .
. . . . . . . .
. . . . .
Vv03g0567 . . . . . . .
. . . . . . . .
. . . . .
Vv03g0568 . . . . . . .
. . . . . . . .
. . . . .
Vv03g0569 . . . . . . .
. . . . . . . .
. . . . .
Vv03g0570 . . . . . . .
. . . . . . . .
. . . . .
Vv03g0571 . . . . . . .
. . . . . . . .
. . . . .
Vv03g0572 . . . . . . .
. . . . . . . .

. . . . .
Vv03g0573 . . . . . . .
. . . . . . . .
. . . . .
Vv03g0574 . Gorai.002G214600.1 Gorai.005G264100.1
. . . Vv18g0962 Tc08_g001260 .
. . . Gorai.010G015700.1 . .
. . . . .
Vv03g0575 . . Gorai.005G240900.1 . .
. . . . . . . .
. . . . . . .
Vv03g0576 Tc02_g008160 Gorai.013G040000.1 .
Gorai.002G006500.1 . Gorai.012G150900.1 . .
. . . . . . . .
. . . .
Vv03g0577 . . . . . . .
. . . . . . . .
. . . . .
Vv03g0578 Tc02_g008170 . . . . .
Vv18g0961 Tc08_g001250 . Gorai.005G105400.1
Gorai.009G207400.1 . . . . .
. . . .
Vv03g0579 . . . . . . .
. . . . . . . .
. . . . .
Vv03g0580 Tc02_g008190 Gorai.013G040100.1 . .
. . . . . . . .
. . . . . . . .
Vv03g0581 . . . . . . .
. . . . . . . .
. . . . .
Vv03g0582 . . . . . . .
. . . . . . . .
. . . . .
Vv03g0583 . . . . . . .
. . . . . . . .
. . . . .
Vv03g0584 Tc02_g008200 Gorai.013G040200.1 . .
Gorai.008G026900.1 Gorai.012G150800.1 . .
. . . . . . . .
. . . .
Vv03g0585 Tc02_g008210 . . . Gorai.
008G026800.1 . . . . . . .
. . . . . . . .
Vv03g0586 Tc02_g008220 Gorai.013G040300.1 . .
Gorai.008G026700.1 Gorai.012G150700.1 . .
. . . . . . . .
. . . .
Vv03g0587 . . . . . .
Vv18g0956 Tc08_g001210 . Gorai.005G105200.1

Gorai.009G207800.1 . . . . . .
. . .
Vv03g0588 . . . . . . .
. . . . . . . .
. . . . .
Vv03g0589 . . . . . . .
. . . . . . . .
. . . . .
Vv03g0590 . . . . . . .
. . . . . . . .
. . . . .
Vv03g0591 . . . . Gorai.008G026500.1
. . . . . . . .
. . . . . . .
Vv03g0592 . . . . . . .
. . . . . . . .
. . . . .
Vv03g0593 . . . . . . .
. . . . . . . .
. . . . .
Vv03g0594 . . . . . . .
. . . . . . . .
. . . . .
Vv03g0595 . . . . . . .
. . . . . . . .
. . . . .
Vv03g0596 . . . . . . .
. . . . . . . .
. . . . .
Vv03g0597 . . . . . . .
. . . . . . . .
. . . . .
Vv03g0598 . . . . . . .
. . . . . . . .
. . . . .
Vv03g0599 . . . . . . .
. . . . . . . .
. . . . .
Vv03g0600 . . . . . . .
. . . . . . . .
. . . . .
Vv03g0601 . . . . . . .
. . . . . . . .
. . . . .
Vv03g0602 . . . . . . .
. . . . . . . .
. . . . .
Vv03g0603 . . . . . . .
. . . . . . . .
. . . . .

Vv03g0604 . . . . . . .
. . . . . . . .
. . . . .
Vv03g0605 . . . . . . .
. . . . . . . .
. . . . .
Vv03g0606 . . . . . . .
. . . . . . . .
. . . . .
Vv03g0607 Tc02_g008250 . . . . .
. . . . . . . .
. . . . . .
Vv03g0608 . . . . . . .
. . . . . . . .
. . . . .
Vv03g0609 Tc02_g008260 . . Gorai.002G006700.1
. . . . . . . .
. . . . . . . .
Vv03g0610 Tc02_g008270 . . Gorai.002G006800.1
. . . . . . . .
. . . . . . . .
Vv03g0611 Tc02_g008280 . . . . .
. . . . . . . .
. . . . . .
Vv03g0612 . . . . . . .
. . . . . . . .
. . . . .
Vv03g0613 Tc02_g008310 Gorai.013G040500.1 . .
. . . . . . . .
. . . . . . . .
Vv03g0614 Tc02_g008320 . . . Gorai.
008G026400.1 . . . . . . .
. . . . . . . .
Vv03g0615 . . . . . . .
. . . . . . . .
. . . . .
Vv03g0616 Tc02_g008330 . . Gorai.002G006900.1
. . . . . . . .
. . . . . . . .
Vv03g0617 Tc02_g008350 . . . . .
. . . . . . . .
. . . . . .
Vv03g0618 Tc02_g008360 Gorai.013G040600.1 . .
Gorai.008G026300.1 Gorai.012G150500.1 . .
. . . . . . . .
. . . .
Vv03g0619 Tc02_g008370 Gorai.013G040700.1 . .
Gorai.008G026200.1 . . . . .
. . . . . . . .
. .

```
Vv03g0620       Tc02_g008380    .       .       .       .
Gorai.012G150400.1      .       .       .       .       .       .
        .       .       .       .       .       .       .       .
Vv03g0621       Tc02_g008400    Gorai.013G041000.1      Gorai.
005G240200.1    .       .       .       .       .       .       .
        .       .       .       .       .       .       .       .
        .       .
Vv03g0622       Tc02_g008410    .       .       Gorai.002G007100.1
        .       .       .       .       .       .       .       .
        .       .       .       .       .       .       .       .
Vv03g0623       .       .       .       .       Gorai.008G025800.1
        .       .       .       .       .       .       .       .
        .       .       .       .       .       .       .
Vv03g0624       Tc02_g008450    Gorai.013G041200.1      .       .
        .       .       .       .       .       .       .       .
        .       .       .       .       .       .       .       .
Vv03g0625       .       .       .       .       .       .       .
        .       .       .       .       .       .       .       .
        .       .       .       .       .
Vv03g0626       .       .       .       .       Gorai.008G025700.1
        .       .       .       .       .       .       .       .
        .       .       .       .       .       .       .
Vv03g0627       Tc02_g008460    Gorai.013G041800.1      .       .
        .       .       Vv18g0944       Tc08_g001050    .       .
        .       .       Gorai.010G016700.1      .       .       .
        .       .       .       .
Vv03g0628       .       Gorai.013G042000.1      .       .       .
        .       .       .       .       .       .       .       .
        .       .       .       .       .       .       .
Vv03g0629       Tc02_g008480    .       .       .       .       .
        .       .       .       .       .       .       .       .
        .       .       .       .       .       .
Vv03g0630       .       .       .       .       .       .       .
        .       .       .       .       .       .       .       .
        .       .       .       .       .
Vv03g0631       Tc02_g008490    Gorai.013G042100.1      .       .
        .       .       .       .       .       .       .       .
        .       .       .       .       .       .       .       .
Vv03g0632       .       .       .       .       .       .       .
        .       .       .       .       .       .       .       .
        .       .       .       .       .
Vv03g0633       .       .       .       .       .       .       .
        .       .       .       .       .       .       .       .
        .       .       .       .       .
Vv03g0634       Tc02_g008500    .       .       .       .       .
        .       .       .       .       .       .       .       .
        .       .       .       .       .       .
Vv03g0635       .       .       .       .       .       .       .
        .       .       .       .       .       .       .       .
        .       .       .       .       .
```

Vv03g0636 Tc02_g008530 Gorai.013G042200.1 .
Gorai.002G007300.1 Gorai.008G025600.1 . Vv18g0939
Tc08_g001010 . Gorai.005G104600.1 Gorai.
009G209200.1 . . . . . . .
. .
Vv03g0637 Tc02_g008550 . . . . .
. . . . . . . .
. . . . . .
Vv03g0638 Tc02_g008560 . . . . .
. . . . . . . .
. . . . . .
Vv03g0639 Tc02_g008580 Gorai.013G042300.1 . .
Gorai.008G025400.1 . . . . .
. . . . . . . .
. .
Vv03g0640 Tc02_g008590 Gorai.013G042400.1 . .
Gorai.008G025300.1 . Vv18g0932
Tc08_g000920 . . . . . . .
. . . . .
Vv03g0641 . . . . . . .
. . . . . . . .
. . . . .
Vv03g0642 . . . . . . .
. . . . . . Vv04g0564
. . . . . .
Vv03g0643 Tc02_g008600 Gorai.013G042500.1 . .
Gorai.008G025200.1 . . . . .
. . . Vv04g0563 . . .
. . .
Vv03g0644 . . . . . . .
. . . . . . Vv04g0562
. . . . . .
Vv03g0645 Tc02_g008610 . . . . .
. . . . . . .
Vv04g0561 . . . . . .
Vv03g0646 . . . . . . .
. . . . . . Vv04g0560
. . . . . .
Vv03g0647 Tc02_g008620 . Gorai.005G239400.1 .
. . . . . . . .
. . . . . . . .
Vv03g0648 . . Gorai.005G239200.1 . .
. . . . . . . .
Vv04g0558 . . . . .
Gorai.010G114500.1
Vv03g0649 Tc02_g008630 . Gorai.005G239100.1 .
. . Vv18g0905 Tc08_g000810 .
Gorai.005G103700.1 Gorai.009G210700.1 . . .
. . . . . .
Vv03g0650 . . . . . . .

. . . . . . . .
. . . . .
Vv03g0651 . . Gorai.005G239000.1 . .
. . . . . . . .
Vv04g0556 . . . . . .
Vv03g0652 . . . . . . .
. . . . . . . .
. . . . .
Vv03g0653 . . . . . Gorai.
012G150300.1 . . . . . . .
. . . . . . .
Vv03g0654 . . . . . . .
. . . . . . . .
. . . . .
Vv03g0655 . . . . . . .
. . . . . . . .
. . . . .
Vv03g0656 . . Gorai.005G238800.1 Gorai.
002G007500.1 . . Vv18g0900 Tc08_g000740 .
. Gorai.009G210900.1 . . . .
. . . . .
Vv03g0657 . Gorai.013G042700.1 Gorai.005G238700.1
. . . . . . . .
. . . . . . . .
.
Vv03g0658 . . Gorai.005G238600.1 Gorai.
002G007600.1 . . Vv18g0898 Tc08_g000720
Gorai.002G128700.1 . . . . . .
. . . . .
Vv03g0659 . . . . . . .
. . . . . . . .
. . . . .
Vv03g0660 . Gorai.013G042800.1 . Gorai.
002G007700.1 . . . . . . .
. . . . . . . .
.
Vv03g0661 . . Gorai.005G238500.1 . .
. . . . . . . .
. . . . . . .
Vv03g0662 . . . . . . .
. . . . . . . .
. . . . .
Vv03g0663 . . . . . . .
. . . . . . . .
. . . . .
Vv03g0664 . Gorai.013G043100.1 . . .
. . . . . . . .
. . . . . . .
Vv03g0665 . . . . . . .
. . . . . . . .

. . . . .
Vv03g0666 Tc02_g008660 Gorai.002G174700.1 . .
. . . . . . . .
. . . . . . . .
Vv03g0667 . . . . . . .
. . . . . . . .
. . . . .
Vv03g0668 . Gorai.002G174600.1 . . .
Gorai.012G150200.1 . . . . .
. . . . . . . .
.
Vv03g0669 . Gorai.002G174500.1 . . .
. . . . . . . .
. . . . . . .
Vv03g0670 Tc02_g008700 . . . . .
. . . . . . . .
. . . . . .
Vv03g0671 Tc02_g008710 Gorai.002G172400.1 . .
. . . . . . . .
. . . . . . . .
Vv03g0672 Tc02_g008720 Gorai.013G043500.1 . .
. . . . . . . .
. . . . . . . .
Vv03g0673 Tc02_g008730 Gorai.002G170600.1 Gorai.
005G238300.1 Gorai.002G007900.1 . . . .
. . . . . . . .
. . . .
Vv03g0674 Tc02_g008740 . Gorai.005G238200.1
Gorai.002G008000.1 . . . . . .
. . . . . . . .
. .
Vv03g0675 Tc02_g008750 . Gorai.005G238100.1
Gorai.002G008100.1 . . Vv18g0883
Tc08_g000520 . . . . Gorai.010G019700.1
. . . . . . .
Vv03g0676 . Gorai.013G043700.1 Gorai.005G238000.1
. Gorai.008G025100.1 . . . .
. . . . . . . .
. . .
Vv03g0677 . . . . . . .
. . . . . . . .
. . . . .
Vv03g0678 . . . . . . .
. . . . . . . .
. . . . .
Vv03g0679 . . . . . . .
. . . . . . . .
. . . . .
Vv03g0680 . . . . . . .
. . . . . . . .

. . . . .
Vv03g0681 . . . . . . .
. . . . . . . .
. . . . .
Vv03g0682 . . . . . . .
. . . . . . . .
. . . . .
Vv03g0683 . . . . . . .
. . . . . . . .
. . . . .
Vv03g0684 . . . . . . .
. . . . . . . .
. . . . .
Vv03g0685 Tc02_g008760 Gorai.013G043800.1 . .
Gorai.008G025000.1 . . . . .
. . . . . . . .
. .
Vv03g0686 Tc02_g008770 Gorai.013G044100.1 Gorai.
005G237800.1 . . . . . . .
. . . . . . . .
. .
Vv03g0687 Tc02_g008790 Gorai.013G044200.1 . .
. . . . . . . .
. . . . . . . .
Vv03g0688 Tc02_g008800 Gorai.013G044300.1 .
Gorai.002G008200.1 Gorai.008G024900.1 . . .
. . . . . . . .
. . . .
Vv03g0689 . . . . . . .
. . . . . . . .
. . . . .
Vv03g0690 Tc02_g008830 . Gorai.005G237500.1 .
. . . . . . . .
. . . . . . . .
Vv03g0691 Tc02_g008840 Gorai.013G044500.1 Gorai.
005G237400.1 . Gorai.008G024700.1 . Vv18g0869
Tc08_g000390 . Gorai.005G102300.1 Gorai.
009G212600.1 . . Vv04g0536 . . .
Gorai.004G274100.1 . .
Vv03g0692 Tc02_g008860 Gorai.013G044600.1 . .
. . . . . . . .
. . . . . . . .
Vv03g0693 Tc02_g008870 . . . Gorai.
008G024600.1 . . . . . . .
. . . . . . . .
Vv03g0694 Tc02_g008880 . Gorai.005G237300.1 .
Gorai.008G024500.1 . . . . .
. . . . . . . .
. .
Vv03g0695 . . . . . . .

. . . . . . . .
. . . . .
Vv03g0696 Tc02_g008890 Gorai.013G044700.1 . .
. Gorai.012G150000.1 . . . .
. . . . . . . .
. .
Vv03g0697 . . . . . . .
. . . . . . . .
. . . . .
Vv03g0698 Tc02_g008900 . . . Gorai.
008G024400.1 . . . . . . .
. . . . . . . .
Vv03g0699 Tc02_g008910 . . . . .
. . . . . . . .
. . . . . .
Vv03g0700 Tc02_g008920 Gorai.013G044800.1 . .
Gorai.008G024300.1 . . . . .
. . . . . . . .
. .
Vv03g0701 Tc02_g008940 Gorai.013G044900.1 . .
Gorai.008G024200.1 . Vv07g1118
Tc01_g005230 . . Gorai.007G214400.1 . .
. . . . . . .
Vv03g0702 Tc02_g008950 Gorai.013G045000.1 Gorai.
005G237200.1 . . . . . . .
. . . . . . . .
. .
Vv03g0703 Tc02_g008960 Gorai.013G045100.1 . .
. . . . . . . .
. . . . . . . .
Vv03g0704 Tc02_g008970 Gorai.013G045200.1 . .
Gorai.008G024100.1 Gorai.012G149900.1 . .
. . . . . . . .
. . . .
Vv03g0705 . . . . . . .
. . . . . . . .
. . . . .
Vv03g0706 Tc02_g008990 Gorai.013G045300.1 Gorai.
005G237100.1 . Gorai.008G024000.1 Gorai.012G149800.1
Vv07g1105 Tc01_g005290 . . . .
. . . . . . . .
Vv03g0707 . . . . . . .
. . . . . . . .
. . . . .
Vv03g0708 Tc02_g009030 Gorai.013G045400.1 Gorai.
005G236900.1 Gorai.002G008400.1 . Gorai.012G149700.1
. . . . . . . .
. . . . . .
Vv03g0709 . . . . . . .
. . . . . . . .

. . . . .
Vv03g0710 Tc02_g009040 Gorai.013G045500.1 Gorai.
005G236800.1 . . . . . . .
. . . . . . . .
. .
Vv03g0711 Tc02_g009050 Gorai.013G045700.1 . .
. . Vv07g1095 Tc01_g005390 . .
Gorai.007G215700.1 . . . . .
. . . .
Vv03g0712 Tc02_g009060 Gorai.013G045800.1 Gorai.
005G236700.1 . . . Vv07g1094 . .
. Gorai.007G215800.1 . . . .
. . . . .
Vv03g0713 Tc02_g009070 Gorai.013G045900.1 Gorai.
005G236600.1 . . . Vv07g1088 . .
. . . . . . . .
. . .
Vv03g0714 Tc02_g009080 Gorai.013G046000.1 Gorai.
005G236500.1 . . . . . . .
. . . . . . . .
. .
Vv03g0715 . . . . . . .
. . . . . . . .
. . . . .
Vv03g0716 . . . . . . .
. . . . . . . .
. . . . .
Vv03g0717 Tc02_g009130 Gorai.013G046100.1 . .
Gorai.008G023800.1 . . . . .
. . . . . . . .
. .
Vv03g0718 Tc02_g009160 Gorai.013G046200.1 . .
. . . . . . . .
. . . . . . . .
Vv03g0719 Tc02_g009190 . Gorai.005G236300.1 .
. . Vv07g1079 Tc01_g005530 Gorai.
007G055100.1 . Gorai.007G216600.1 . . .
. . . . . .
Vv03g0720 Tc02_g009220 . Gorai.005G236100.1 .
. . . . . . . .
. . . . . . . .
Vv03g0721 . . . . . .
Vv07g1073 . . . Gorai.007G216900.1 .
. . . . . . . .
Vv03g0722 Tc02_g009260 . Gorai.005G235900.1 .
. . Vv18g0822 Tc08_g000020 Gorai.
002G133800.1 . Gorai.009G214900.1 . Gorai.
010G022700.1 Vv07g1072 Tc01_g005600 . . .
. .
Vv03g0723 Tc02_g009270 . Gorai.005G235800.1

Gorai.002G008700.1	Gorai.008G023600.1	.	.	.
	.	.	.	.	.	Vv07g1069
Tc01_g005630	Gorai.007G055500.1	.	.	.	.
Vv03g0724	Tc02_g009280	Gorai.013G046300.1	Gorai.
005G235700.1	Gorai.002G008800.1	.	.	Vv18g0820
	.	.	.	Gorai.009G215200.1	.
Gorai.010G022800.1	.	.	.	.	.	.
	.
Vv03g0725	Tc02_g009300	Gorai.013G046400.1	Gorai.
005G235600.1	.	.	Gorai.012G149300.1	Vv18g0788
	.	.	.	Gorai.009G216800.1	.
Gorai.010G024100.1	Vv07g1065	Tc01_g005650	.	.
	.	Gorai.003G027100.1	Gorai.011G106700.1
Vv03g0726	Tc02_g009310	.	.	.	Gorai.
008G023500.1	Gorai.012G149200.1	.	.	.	.
	.	.	.	.	.	.	.	.
	.	.
Vv03g0727	Tc02_g009320	.	.	.	.	.
	.	.	.	.	.	.	.	.
	.	.	.	.	.	.
Vv03g0728	Tc02_g009330	Gorai.013G046500.1	.
Gorai.002G008900.1	.	.	.	.	.	.
	.	.	.	.	.	.	.	.
	.	.
Vv03g0729	Tc02_g009340	.	.	.	.	.
	.	.	.	.	.	.	.	.
	.	.	.	.	.	.
Vv03g0730	.	.	.	.	.	.	.
	.	.	.	.	.	.	.	.
	.	.	.	.	.
Vv03g0731	Tc02_g009350	Gorai.013G046600.1	.
Gorai.002G009000.1	Gorai.008G023400.1	.	.	.
	.	.	.	.	.	.	.	.
	.	.	.	.
Vv03g0732	Tc02_g009360	Gorai.013G046700.1	.
Gorai.002G009100.1	.	.	.	.	.	.
	.	.	.	.	.	.	.	.
	.	.
Vv03g0733	Tc02_g009370	.	Gorai.005G235500.1	.
	.	.	.	.	.	.	.	.
	.	.	.	.	.	.	.	.
Vv03g0734	Tc02_g009390	.	Gorai.005G235400.1	.
	.	.	.	.	.	.	.	.
	.	.	.	.	.	.	.	.
Vv03g0735	Tc02_g009400	.	Gorai.005G235300.1	.
	.	.	.	.	.	.	.	.
	.	.	.	.	.	.	.	.
Vv03g0736	Tc02_g009410	Gorai.013G046900.1	.	.
	Gorai.008G023300.1	Gorai.012G149100.1	.	.
	.	.	.	.	.	.	.	.

. . . .
Vv03g0737 Tc02_g009420 . . Gorai.002G009200.1
. . . . . . . .
. . . . . . . .
Vv03g0738 . . . . . . .
. . . . . . . .
. . . . .
Vv03g0739 Tc02_g009430 Gorai.013G047000.1 . .
Gorai.008G023200.1 . . . . .
. . . . . . . .
. .
Vv03g0740 Tc02_g009440 Gorai.013G047100.1 Gorai.
005G235200.1 . . . . . . .
. . . . . . . .
. .
Vv03g0741 . . . . . . .
. . . . . . . .
. . . . .
Vv03g0742 Tc02_g009460 . Gorai.005G235000.1 .
. . . . . . . .
. . . . . . . .
Vv03g0743 . . . . . . .
. . . . . . . .
. . . . .
Vv03g0744 Tc02_g009470 . Gorai.005G234900.1
Gorai.002G009300.1 . Gorai.012G149000.1 . .
. . . . . . . .
. . . .
Vv03g0745 . . . . . . .
. . . . . . . .
. . . . .
Vv03g0746 . . . . Gorai.008G022900.1
. . . . . . . .
. . . . . . .
Vv03g0747 Tc02_g009480 . . Gorai.002G009400.1
. . . . . . . .
. . . . . . . .
Vv03g0748 Tc02_g009490 . . . . .
Vv07g1057 Tc01_g005730 . . Gorai.
007G218300.1 . . . . . . .
. .
Vv03g0749 . . . . . . .
. . . . . . . .
. . . . .
Vv03g0750 . . . . . . .
. . . . . . . .
. . . . .
Vv03g0751 Tc02_g009500 . Gorai.005G234800.1 .
Gorai.008G022800.1 . . . . .
. . . . . . . .

. .
Vv03g0752 Tc02_g009510 . . Gorai.002G009500.1
. . . . . . . .
. . . . . . . .
Vv03g0753 . . . . . . .
. . . . . . . .
. . . . .
Vv03g0754 . . . . . . .
. . . . . . . .
. . . . .
Vv03g0755 Tc02_g009530 . Gorai.005G234700.1 .
. . . . . . . .
. . . . . . . .
Vv03g0756 . . . . . . .
. . . . . . . .
. . . . .
Vv03g0757 Tc02_g009540 . Gorai.005G234600.1 .
. . . . . . . .
. . . . . . . .
Vv03g0758 . . . . . . .
. . . . . . . .
. . . . .
Vv03g0759 Tc02_g009910 Gorai.013G049000.1 Gorai.
005G233000.1 . . . Vv18g0765 . .
. . . . Vv07g1000
Tc01_g006140 . . Gorai.007G190900.1 . .
Vv03g0760 Tc02_g009920 Gorai.013G049100.1 . .
Gorai.008G021100.1 . . . . .
. . . . . . . .
. .
Vv03g0761 . Gorai.013G049200.1 . . .
. . . . . . . .
. . . . . . .
Vv03g0762 Tc02_g009930 . . Gorai.002G009800.1
. . . . . . . .
. . . . . . . .
Vv03g0763 . . . Gorai.002G009900.1 .
. . . . . . . .
Vv07g0994 Tc01_g006200 . . Gorai.
007G190600.1 . .
Vv03g0764 . . . . . . .
. . . . . . . .
. . . . .
Vv03g0765 . . . . . . .
. . . . . . . .
. . . . .
Vv03g0766 . . . . . . .
. . . . . . . .
. . . . .
Vv03g0767 Tc02_g009960 Gorai.013G049300.1 . .

. . . . . . . .
. . . . . . . .
Vv03g0768 Tc02_g009970 . . . Gorai.
008G021000.1 . . . . . . .
. . . . . . . .
Vv03g0769 Tc02_g009990 . . . Gorai.
008G020900.1 . . . . . . .
. . . . . . . .
Vv03g0770 . . . . . . .
. . . . . . . .
. . . . .
Vv03g0771 Tc02_g010000 Gorai.013G049500.1 . .
. . Vv18g0755 . . .
Gorai.009G218300.1 . . . . . .
. . .
Vv03g0772 Tc02_g010010 . . Gorai.002G010000.1
. . . . . . . .
. Vv07g0985 . . Gorai.008G080900.1
Gorai.007G221800.1 Gorai.003G029100.1 .
Vv03g0773 Tc02_g010020 . . . .
Gorai.012G148400.1 . . . . . .
. . . . . . . .
Vv03g0774 Tc02_g010050 Gorai.013G049600.1 . .
. Gorai.012G148200.1 . . . .
. . . . . . . .
. .
Vv03g0775 Tc02_g010060 . Gorai.005G232800.1 .
. . . . . . . .
. . . . . . . .
Vv03g0776 . . . . . . .
. . . . . . . .
. . . . .
Vv03g0777 Tc02_g010090 Gorai.013G049900.1 Gorai.
005G232600.1 . . . Vv18g0752 . .
Gorai.005G099700.1 Gorai.009G218600.1 .
Gorai.010G025000.1 . . . . . .
.
Vv03g0778 Tc02_g010100 . Gorai.005G232500.1 .
. . Vv18g0751 . . .
Gorai.009G218800.1 . Gorai.010G025100.1 Vv07g0982
Tc01_g006300 Gorai.007G056300.1 Gorai.008G081100.1
Gorai.007G222000.1 . .
Vv03g0779 Tc02_g010110 Gorai.013G050000.1 Gorai.
005G232400.1 . . . . . . .
. . . Vv07g0973 Tc01_g006380 .
. Gorai.007G222400.1 Gorai.003G039100.1 .
Vv03g0780 Tc02_g010120 Gorai.013G050100.1 Gorai.
005G232300.1 . Gorai.008G020700.1 . . .
. . . . . . . .
. . . .

Vv03g0781 . . . . . . .
. . . . . . . .
. . . . .
Vv03g0782 Tc02_g010130 . . . .
Gorai.012G147700.1 . . . . . .
. . . . . . . .
Vv03g0783 . . . . . . .
. . . . . . . .
. . . . .
Vv03g0784 Tc02_g010140 Gorai.013G050200.1 Gorai.
005G232100.1 . . . . . . .
. . . . . . . .
. .
Vv03g0785 . . . . . . .
. . . . . . . .
. . . . .
Vv03g0786 Tc02_g010150 . Gorai.005G232000.1
Gorai.002G010200.1 Gorai.008G020600.1 . . .
. . . . . . . .
. . . .
Vv03g0787 Tc02_g010160 . Gorai.005G231900.1 .
. . . . . . . .
. . . . . . . .
Vv03g0788 Tc02_g010170 Gorai.013G050300.1 Gorai.
005G231800.1 Gorai.002G010300.1 . . . .
. . . . . . . .
. . . .
Vv03g0789 Tc02_g010190 . . . .
Gorai.012G147500.1 . . . . . .
. . . . . . . .
Vv03g0790 Tc02_g010210 Gorai.013G050400.1 . .
. . Vv18g0745 . . Gorai.
005G099600.1 Gorai.009G219300.1 Gorai.009G277100.1 .
. . . . . . .
Vv03g0791 Tc02_g010220 Gorai.013G050500.1 . .
Gorai.008G020500.1 . . . . .
. . . . . . . .
. .
Vv03g0792 . . . . . . .
. . . . . . . .
. . . . .
Vv03g0793 Tc02_g010230 . Gorai.005G231500.1 .
. . . . . . . .
. . . . . . . .
Vv03g0794 Tc02_g010240 . . . Gorai.
008G020400.1 . . . . . . .
. . . . . . . .
Vv03g0795 . . . . . . .
. . . . . . Vv07g0965
Tc01_g006480 . Gorai.008G082900.1 Gorai.

007G223000.1 . .
Vv03g0796 Tc02_g010260 . . . Gorai.
008G020300.1 . . . . . . .
. . . . . . . .
Vv03g0797 Tc02_g010270 . Gorai.005G231400.1 .
. . . . . . . .
. . . . . . . .
Vv03g0798 . . . . . . .
. . . . . . . .
. . . . .
Vv03g0799 Tc02_g010280 . . . . .
. . . . . . . .
. . . . . .
Vv03g0800 Tc02_g010290 . Gorai.005G231200.1 .
Gorai.008G020100.1 . . . . .
. . . . . . . .
. .
Vv03g0801 . . . . . . .
. . . . . . . .
. . . . .
Vv03g0802 . . . . . . .
. . . . . . . .
. . . . .
Vv03g0803 . . . . . . .
. . . . . . . .
. . . . .
Vv03g0804 . . . . . . .
. . . . . . . .
. . . . .
Vv03g0805 . . . . . . .
. . . . . . . .
. . . . .
Vv03g0806 Tc02_g010300 . Gorai.005G231100.1 .
. . . . . . . .
. . . . . . . .
Vv03g0807 . . . . . . .
. . . . . . . .
. . . . .
Vv03g0808 . . . . . . .
. . . . . . . .
. . . . .
Vv03g0809 . . . . . . .
. . . . . . . .
. . . . .
Vv03g0810 Tc02_g010330 . Gorai.005G230900.1 .
. Gorai.012G147400.1 Vv18g0733 . .
. . . Gorai.010G026100.1 . .
. . . . .
Vv03g0811 Tc02_g010340 . . . .
Gorai.012G147300.1 . . . . . .

```
        .        .        .       .        .       .        .       .
Vv03g0812        Tc02_g010350     .        Gorai.005G230800.1       .
        .        .        .       .        .       .        .       .
        .        .        .       .        .       .        .       .
Vv03g0813        .        .       .        .       .        .       .
        .        .        .       .        .       .        .       .
        .        .        .       .        .
Vv03g0814        .        .       Gorai.005G230600.1        .
Gorai.008G019900.1        .       Vv18g0727        .        .       .
        .        .        .       .        .       .        .       .
        .        .
Vv03g0815        .        Gorai.013G050900.1       Gorai.005G230300.1
        .        .        .       .        .       .        .       .
        .        .        Vv07g0946        .       .        .       .
        .        .
Vv03g0816        .        .       .        .       .        .       .
        .        .        .       .        .       .        .       .
        .        .        .       .        .
Vv03g0817        .        .       .        .       .        .       .
        .        .        .       .        .       .        .       .
        .        .        .       .        .
Vv03g0818        .        .       .        .       .        .       .
        .        .        .       .        .       .        .       .
        .        .        .       .        .
Vv03g0819        .        .       Gorai.005G227300.1        .       .
        .        .        .       .        .       .        .       .
        .        .        .       .        .       .        .
Vv03g0820        .        .       .        .       .        .       .
        .        .        .       .        .       .        .       .
        .        .        .       .        .
Vv03g0821        .        .       .        .       .        .       .
        .        .        .       .        .       .        .       .
        .        .        .       .        .
Vv03g0822        .        .       .        .       .        .       .
        .        .        .       .        .       .        .       .
        .        .        .       .        .
Vv03g0823        .        .       .        .       .        .       .
        .        .        .       .        .       .        .       .
        .        .        .       .        .
Vv03g0824        .        .       .        .       .        .       .
        .        .        .       .        .       .        .       .
        .        .        .       .        .
Vv03g0825        .        .       .        .       .        .       .
        .        .        .       .        .       .        .       .
        .        .        .       .        .
Vv03g0826        Tc02_g032020     Gorai.013G051100.1        .
Gorai.002G010900.1        .       .        .       .        .       .
        .        .        .       .        .       .        .       .
        .        .
Vv03g0827        .        .       .        .       .        .
```

Vv18g0721 . . . . . .
Vv08g0700 . . . . . .
Vv03g0828 . . . . . . .
. . . . . . . .
. . . . .
Vv03g0829 . . . . . . .
. . . . . . . .
. . . . .
Vv03g0830 . . . . . . .
. . . . . . Vv08g0701
. . . . . .
Vv03g0831 . . . . . . .
. . . . . . . .
. . . . .
Vv03g0832 . . . . . . .
. . . . . . Vv08g0702
. . . . . .
Vv03g0833 . . . . . . .
. . . . . . . .
. . . . .
Vv03g0834 . . . . . . .
. . . . . . Vv08g0704
. . . . . .
Vv03g0835 . . . . . . .
. . . . . . . .
. . . . .
Vv03g0836 Tc02_g010380 . . . .
Gorai.012G144300.1 . . . . . .
. . . . . . . .
Vv03g0837 . . . . . . .
. . . . . . . .
. . . . .
Vv03g0838 . . . . . . .
. . . . . . . .
. . . . .
Vv03g0839 Tc02_g010400 . . . . .
. . . . . . . .
. . . . . .
Vv03g0840 Tc02_g013860 . . . . .
. . . . . . .
Vv08g0706 . . . . . .
Vv03g0841 . . . . . . .
. . . . . . . .
. . . . .
Vv03g0842 . . . . . Gorai.
012G142700.1 . . . . . . .
. . . . . . .
Vv03g0843 . . . . . . .
. . . . . . . .
. . . . .

Vv03g0844 . . . . . . .
. . . . . . Vv08g0708
. . . . . .
Vv03g0845 . . . . . . .
. . . . . . . .
. . . . .
Vv03g0846 . . . . . . .
. . . . . . . .
. . . . .
Vv03g0847 . . . . . . .
. . . . . . . .
. . . . .
Vv03g0848 Tc02_g010410 . . . .
Gorai.012G145000.1 . . . . . .
. . . . . . . .
Vv03g0849 Tc02_g013840 . . . . .
. . . . . . . .
. . . . . .
Vv03g0850 . . . . . . .
. . . . . . . .
. . . . .
Vv03g0851 . . . . . . .
. . . . . . . .
. . . . .
Vv03g0852 Tc02_g013830 . . . . .
. . . . . . . .
. . . . . .
Vv03g0853 . . . . . . .
. . . . . . . .
. . . . .
Vv03g0854 . . . . . . .
. . . . . . . .
. . . . .
Vv03g0855 . . . . . . .
. . . . . . . .
. . . . .
Vv03g0856 . . . . . . .
. . . . . . . .
. . . . .
Vv03g0857 Tc02_g031750 . . . . .
. . . . . . . .
. . . . . .
Vv03g0858 . . . . . . .
. . . . . . . .
. . . . .
Vv03g0859 Tc02_g013810 . . . . .
. . . . . . . .
. . . . . .
Vv03g0860 . . . . . . .
. . . . . . . .

.	.	.	.	.
Vv03g0861	.	.	.	.	Gorai.008G019500.1
.	.	.	.	.	.	.	.
.	.	.	.	.	.	.
Vv03g0862	.	Gorai.013G051500.1	.	.	.
.	.	.	.	.	.	.	.
.	.	.	.	.	.	.
Vv03g0863	.	.	.	.	.	.
Vv18g0712	.	.	.	.	.	.	.
.	.	.	.	.	.
Vv03g0864	.	.	.	Gorai.002G011000.1	.
.	.	.	.	.	.	.	.
.	.	.	.	.	.	.
Vv03g0865	.	.	.	.	.	.	.
.	.	.	.	.	.	.	.
.	.	.	.	.
Vv03g0866	.	Gorai.013G052000.1	.	.	.
.	.	.	.	.	.	.	.
.	.	.	.	.	.	.
Vv03g0867	.	.	.	.	.	.	.
.	.	.	.	.	.	.	.
.	.	.	.	.
Vv03g0868	.	.	.	.	.	.	.
.	.	.	.	.	.	.	.
.	.	.	.	.
Vv03g0869	.	.	.	.	.	.	.
.	.	.	.	.	.	.	.
.	.	.	.	.
Vv03g0870	.	.	.	.	.	.	.
.	.	.	.	.	.	Vv07g0912
Tc01_g007180	.	Gorai.008G087400.1	.	.
.
Vv03g0871	.	.	.	.	.	.
Vv18g0694	.	.	.	Gorai.009G222500.1	.
Gorai.010G028600.1	.	.	.	.	.
.	.
Vv03g0872	.	.	.	.	.	.	.
.	.	.	.	.	.	.	.
.	.	.	.	.
Vv03g0873	.	.	.	.	Gorai.008G019300.1
.	Vv18g0672	.	.	.	.	.
.	.	.	.	.	.	.	.
Vv03g0874	.	.	.	.	Gorai.008G019200.1
.	.	.	.	.	.	.	.
.	.	.	.	.	.	.
Vv03g0875	.	Gorai.002G214500.1	.	Gorai.
002G011200.1	Gorai.008G019100.1	.	.	.	.
.	.	.	.	.	.	.	.
.	.	.
Vv03g0876	.	.	.	.	.	.	.

. . . . . . . .
. . . . .
Vv03g0877 . . . . . . .
. . . . . . . .
. . . . .
Vv03g0878 . Gorai.013G052200.1 . . .
. . . . . . . .
. . . . . . .
Vv03g0879 . . . . . . .
. . . . . . . .
. . . . .
Vv03g0880 Tc02_g002290 Gorai.002G213600.1 . .
. . . . . . . .
. . . . . . . .
Vv03g0881 . . . . . . .
. . . . . . . .
. . . . .
Vv03g0882 Tc02_g010670 . . . Gorai.
008G017200.1 . . . . . . .
. Vv07g0913 Tc01_g007160 . .
Gorai.007G228500.1 Gorai.003G034100.1 .
Vv03g0883 . . . . . . .
. . . . . . . .
. . . . .
Vv03g0884 . . . . . . .
. . . . . . . .
. . . . .
Vv03g0885 Tc02_g002300 Gorai.002G213500.1 . .
. . . . . . . .
. . . . . . . .
Vv03g0886 . . . . . . .
. . . . . . . .
. . . . .
Vv03g0887 . . . . . . .
. . . . . . . .
. . . . .
Vv03g0888 . . . . . . .
. . . . . . . .
. . . . .
Vv03g0889 . . . . . . .
. . . . . . . .
. . . . .
Vv03g0890 Tc02_g002310 . . . . .
. . . . . . . .
. . . . . .
Vv03g0891 . . . . . . .
. . . . . . . .
. . . . .
Vv03g0892 Tc02_g002320 . Gorai.005G263100.1 .
. Gorai.012G163200.1 Vv18g0632

Tc08_g002950 . . . . . . .
. . . . .
Vv03g0893 Tc02_g002330 Gorai.002G213400.1 Gorai.
005G263000.1 . . . . . . .
. . . . . . . .
. .
Vv03g0894 Tc02_g002350 . Gorai.005G262800.1 .
Gorai.008G016500.1 . Vv07g0915
Tc01_g007140 . Gorai.008G087300.1 . Gorai.
003G033900.1 . . . . . . .
.
Vv03g0895 . . . . . . .
. . . . . . . .
. . . . .
Vv03g0896 Tc02_g002360 Gorai.002G213300.1 . .
. . . . . . . .
. . . . . . . .
Vv03g0897 . . . . . . .
. . . . . . . .
. . . . .
Vv03g0898 . . . . . . .
. . . . . . . .
. . . . .
Vv03g0899 Tc02_g002370 Gorai.002G213200.1 . .
. . . . . . . .
. . . . . . . .
Vv03g0900 . . . . . . .
. . . . . . . .
. . . . .
Vv03g0901 . Gorai.013G054100.1 . . .
. Vv07g0919 . . . . .
. . . . . . . .
Vv03g0902 . . . . . . .
. . . . . . . .
. . . . .
Vv03g0903 Tc02_g002380 Gorai.002G212900.1 . .
. . . . . . . .
. . . . . . . .
Vv03g0904 . . . . . . .
. . . . . . . .
. . . . .
Vv03g0905 Tc02_g002390 . . . . .
. . . . . . . .
. . . . . .
Vv03g0906 . . . . . . .
. . . . . . . .
. . . . .
Vv03g0907 . Gorai.002G212800.1 . .
Gorai.008G044000.1 . . . . . .
. . . . . . . .

.
Vv03g0908 . . . . . . .
. . . . . . . .
. . . . .
Vv03g0909 Tc02_g002400 . . . . .
. . . . . . . .
. . . . . .
Vv03g0910 Tc02_g002410 Gorai.002G212700.1 . .
. . . . . . . .
. . . . . . . .
Vv03g0911 . . . . . . .
. . . . . . . .
. . . . .
Vv03g0912 . . . . . . .
. . . . . . . .
. . . . .
Vv03g0913 . . . . . . .
. . . . . . . .
. . . . .
Vv03g0914 . . . . . . .
. . . . . . . .
. . . . .
Vv03g0915 . . . . . . .
. . . . . . . .
. . . . .
Vv03g0916 Tc02_g002420 . . . . .
. . . . . . . .
. . . . . .
Vv03g0917 . . . . . . .
. . . . . . . .
. . . . .
Vv03g0918 . . . . . . .
. . . . . . . .
. . . . .
Vv03g0919 . . . . . . .
. . . . . . . .
. . . . .
Vv03g0920 Tc02_g002430 . . . Gorai.
008G043500.1 . . . . . . .
. . . . . . . .
Vv03g0921 . Gorai.002G212500.1 . . .
Gorai.012G163100.1 . . . . .
. . . . . . . .
.
Vv03g0922 Tc02_g002460 . . . . .
. . . . . . . .
. . . . . .
Vv03g0923 . . . . . . .
. . . . . . . .
. . . . .

Vv03g0924	Tc02_g002470	Gorai.002G212400.1	.	.
	.	.	Vv07g0926	Tc01_g007010	.	.
	Gorai.007G227300.1	Gorai.003G032800.1	.	.
	.	.	.	.	.	.
Vv03g0925	.	.	.	.	.	.	.
	.	.	.	.	.	.	.	.
	.	.	.	.	.
Vv03g0926	Tc02_g002480	Gorai.002G212300.1	Gorai.
005G262700.1	.	Gorai.008G043300.1	.	Vv07g0930
	Tc01_g006990	.	.	.	Gorai.003G032700.1
	.	.	.	.	.	.	.	.
Vv03g0927	Tc02_g002490	Gorai.002G212100.1	Gorai.
005G262600.1	.	.	.	.	.	.	.
	.	.	.	.	.	.	.	.
	.	.
Vv03g0928	.	.	.	.	.	.	.
	.	.	.	.	.	.	.	.
	.	.	.	.	.
Vv03g0929	.	.	.	.	.	.	.
	.	.	.	.	.	.	.	.
	.	.	.	.	.
Vv03g0930	Tc02_g002500	.	Gorai.005G262500.1	.
	.	.	.	.	.	.	.	.
	.	.	.	.	.	.	.	.
Vv03g0931	.	.	.	.	.	.	.
	.	.	.	.	.	.	.	.
	.	.	.	.	.
Vv03g0932	.	.	.	.	.	.	.
	.	.	.	.	.	.	.	.
	.	.	.	.	.
Vv03g0933	.	.	.	.	.	.	.
	.	.	.	.	.	.	.	.
	.	.	.	.	.
Vv03g0934	.	.	.	.	.	.	.
	.	.	.	.	.	.	.	.
	.	.	.	.	.
Vv03g0935	.	.	.	.	.	.	.
	.	.	.	.	.	.	.	.
	.	.	.	.	.
Vv03g0936	.	.	.	.	.	.	.
	.	.	.	.	.	.	.	.
	.	.	.	.	.
Vv03g0937	.	.	.	.	.	.	.
	.	.	.	.	.	.	.	.
	.	.	.	.	.
Vv03g0938	Tc02_g002530	.	Gorai.005G262400.1	.
	.	Gorai.012G162200.1	Vv07g0933
Tc01_g006960	.	.	Gorai.007G226900.1	Gorai.
003G032500.1	.	.	.	.	.	.	.
	.

Vv03g0939 . . . . . . .
. . . . . . . .
. . . . .
Vv03g0940 Tc02_g002540 . . . .
Gorai.012G162100.1 . . . . . .
. . . . . . . .
Vv03g0941 . . . . . . .
. . . . . . . .
. . . . .
Vv03g0942 Tc02_g002550 . Gorai.005G262300.1 .
. . . . . . . .
. . . . . . . .
Vv03g0943 Tc02_g002560 . . . Gorai.
008G043200.1 . . . . . . .
. . . . . . . .
Vv03g0944 . . . . . . .
. . . . . . . .
. . . . .
Vv03g0945 Tc02_g002570 Gorai.002G211900.1 . .
. Gorai.012G162000.1 . . . .
. . . . . . . .
. .
Vv03g0946 . . . . . . .
. . . . . . . .
. . . . .
Vv03g0947 . Gorai.002G211700.1 . . .
. . . . . . . .
. . . . . . .
Vv03g0948 Tc02_g002590 . . . . .
. . . . . . . .
. . . . . .
Vv03g0949 Tc02_g002600 Gorai.002G211600.1 . .
Gorai.008G043000.1 . . . . .
. . . . . . . .
. .
Vv03g0950 Tc02_g002610 Gorai.002G211500.1 . .
. Gorai.012G161700.1 . . . .
. . . . . . . .
. .
Vv03g0951 . . . . . . .
. . . . . . . .
. . . . .
Vv03g0952 Tc02_g002620 . . . Gorai.
008G042900.1 . . . . . . .
. . . . . . . .
Vv03g0953 . . . . . . .
. . . . . . . .
. . . . .
Vv03g0954 . . . . . . .
. . . . . . . .

. . . . .
Vv03g0955 Tc02_g002730 Gorai.002G211400.1 . .
. . . . . . . .
. . . . . . . .
Vv03g0956 . . . . . . .
. . . . . . . .
. . . . .
Vv03g0957 Tc02_g002770 Gorai.002G211200.1 . .
. . . . . . . .
. . . . . . . .
Vv03g0958 . . . . . . .
. . . . . . . .
. . . . .
Vv03g0959 . . . . . . .
. . . . . . . .
. . . . .
Vv03g0960 . . . . . . .
. . . . . . . .
. . . . .
Vv03g0961 Tc02_g002780 . Gorai.005G262000.1 .
. . . . . . . .
. . . . . . . .
Vv03g0962 Tc02_g002790 . Gorai.005G261900.1 .
Gorai.008G042700.1 . . . . .
. . . . . . . .
. .
Vv03g0963 . . . . . . .
. . . . . . . .
. . . . .
Vv03g0964 . . . . . . .
. . . . . . . .
. . . . .
Vv03g0965 . . . . . Gorai.
012G158700.1 . . . . . . .
. . . . . . .
Vv03g0966 . . . . . . .
. . . . . . . .
. . . . .
Vv03g0967 . . . . . . .
. . . . . . . .
. . . . .
Vv03g0968 . . . . . . .
. . . . . . . .
. . . . .
Vv03g0969 Tc02_g002800 . . . Gorai.
008G042600.1 . . . . . . .
. . . . . . . .
Vv03g0970 . . . . . . .
. . . . . . . .
. . . . .

Vv03g0971 . . . . . . .
. . . . . . . .
. . . . .
Vv03g0972 . . . . . . .
. . . . . . . .
. . . . .
Vv03g0973 . . Gorai.005G258900.1 .
Gorai.008G039600.1 . . . . . .
. . . . . . . .
.
Vv03g0974 . . . . . . .
. . . . . . . .
. . . . .
Vv03g0975 . . Gorai.005G227800.1 . .
. . . . . . . .
. . . . . . .
Vv03g0976 . . . . . . .
. . . . . . . .
. . . . .
Vv03g0977 . . . . . . .
. . . . . . . .
. . . . .
Vv03g0978 . . . . . . .
. . . . . . . .
. . . . .
Vv03g0979 . . . . . . .
. . . . . . . .
. . . . .
Vv03g0980 . . Gorai.005G227500.1 . .
. . . . . . . .
. . . . . . .
Vv03g0981 . . . . . . .
. . . . . . . .
. . . . .
Vv03g0982 . . . . . . .
. . . . . . . .
. . . . .
Vv03g0983 . . . . . . .
. . . . . . . .
. . . . .
Vv03g0984 . . . . . . .
. . . . . . . .
. . . . .
Vv03g0985 . . Gorai.005G227400.1 . .
. . . . . . . .
. . . . . . .
Vv03g0986 . . . . . . .
. . . . . . . .
. . . . .
Vv03g0987 . . Gorai.005G227300.1 . .

| | | | | | | | |
|---|---|---|---|---|---|---|---|
| Vv03g0988 | . | . | . | . | . | . | . |
| Vv03g0989 | . | . | . | . | . | . | . |
| Vv03g0990 | . | . | . | . | . | . | . |
| Vv03g0991 | . | . | . | . | . | . | . |
| Vv03g0992 | . | . | Gorai.005G226700.1 | | | . | . |
| Vv03g0993 | . | . | . | . | . | . | . |
| Vv03g0994 | . | . | Gorai.005G224200.1 | | | . | . |
| Vv03g0995 | . | . | . | . | . | . | . |
| Vv03g0996 | . | . | . | . | . | . | . |
| Vv03g0997 | . | . | . | . | . | . | . |
| Vv03g0998 | . | . | . | . | . | . | . |
| Vv03g0999 | . | . | . | . | . | . | . |
| Vv03g1000 | . | . | . | . | . | . | . |
| Vv04g0001 | . | . | . | . | . | . | . |
| Vv04g0002 | . | . | . | . | . | . | . |
| Vv04g0003 | . | . | . | . | . | . | . |

Vv04g0004 . . Gorai.001G008700.1 .
Gorai.003G062600.1 Gorai.011G072200.1 Vv11g0333
Tc09_g009070 . Gorai.001G008700.1 . . .
. . . . . . .
Vv04g0005 Tc09_g009300 Gorai.013G219500.1 Gorai.
001G026300.1 Gorai.004G279600.1 . Gorai.011G072100.1
. . . . . . . .
. . . . . .
Vv04g0006 . Gorai.013G219600.1 . . .
Gorai.011G072000.1 . . . . .
. . . . . . . .
.
Vv04g0007 . . . . . . .
. . . . . . . .
. . . . .
Vv04g0008 . . Gorai.001G008900.1 .
Gorai.003G061800.1 Gorai.011G071400.1 Vv11g0329
Tc09_g009030 . Gorai.001G026200.1 . Gorai.
009G048600.1 . . . . . . .
.
Vv04g0009 . . Gorai.001G026100.1 . .
. . . . . . . .
. . . . . . .
Vv04g0010 . . . . . . .
. . . . . . . .
. . . . .
Vv04g0011 . . . . . . .
. . . . . . . .
. . . . .
Vv04g0012 . . . . . Gorai.
011G068900.1 Vv11g0326 Tc09_g008970 . . .
Gorai.009G048500.1 . . . . .
. . .
Vv04g0013 Tc09_g009400 Gorai.013G219700.1 . .
. . . . . . . .
. . . . . . . .
Vv04g0014 . . . . . . .
. . . . . . . .
. . . . .
Vv04g0015 . . . . . . .
. . . . . . . .
. . . . .
Vv04g0016 . . . . . . .
. . . . . . . .
. . . . .
Vv04g0017 . . . . . . .
. . . . . . . .
. . . . .
Vv04g0018 . . . . . . .
. . . . . . . .

| | | | | | | | |
|---|---|---|---|---|---|---|---|
| . | . | . | . | . | | | |
| Vv04g0019 | . | . | . | . | . | . | . |
| . | . | . | . | . | . | . | . |
| . | . | . | . | . | | | |
| Vv04g0020 | . | . | . | . | . | . | . |
| . | . | . | . | . | . | . | . |
| . | . | . | . | . | | | |
| Vv04g0021 | . | . | . | . | . | . | . |
| . | . | . | . | . | . | . | . |
| . | . | . | . | . | | | |
| Vv04g0022 | . | . | . | . | . | . | . |
| . | . | . | . | . | . | . | . |
| . | . | . | . | . | | | |
| Vv04g0023 | . | . | . | . | . | . | . |
| . | . | . | . | . | . | . | . |
| . | . | . | . | . | | | |
| Vv04g0024 | Tc09_g009720 | | . | . | Gorai.004G280200.1 | | |
| . | . | . | . | . | . | . | . |
| . | . | . | . | . | . | . | . |
| Vv04g0025 | . | . | . | . | . | . | . |
| . | . | . | . | . | . | . | . |
| . | . | . | . | . | | | |
| Vv04g0026 | . | . | . | . | . | . | . |
| . | . | . | . | . | . | . | . |
| . | . | . | . | . | | | |
| Vv04g0027 | . | . | . | . | . | . | . |
| . | . | . | . | . | . | . | . |
| . | . | . | . | . | | | |
| Vv04g0028 | Tc01_g025700 | | . | . | . | . | . |
| . | . | . | . | . | . | . | . |
| . | . | . | . | . | . | | |
| Vv04g0029 | . | . | . | . | . | . | . |
| . | . | . | . | . | . | . | . |
| . | . | . | . | . | | | |
| Vv04g0030 | . | . | . | . | . | . | . |
| . | . | . | . | . | . | . | . |
| . | . | . | . | . | | | |
| Vv04g0031 | Tc01_g025760 | | . | . | . | . | . |
| . | . | . | . | . | . | . | . |
| . | . | . | . | . | . | | |
| Vv04g0032 | Tc01_g007670 | | . | . | . | . | . |
| . | . | . | . | . | . | . | . |
| . | . | . | . | . | . | | |
| Vv04g0033 | Tc01_g026140 | | . | . | . | . | . |
| . | . | . | . | . | . | . | . |
| . | . | . | . | . | . | | |
| Vv04g0034 | Tc01_g007620 | | . | . | . | . | . |
| . | . | . | . | . | . | . | . |
| . | . | . | . | . | . | | |
| Vv04g0035 | . | . | . | . | . | . | . |

. . . . . . . .
. . . . .
Vv04g0036 Tc01_g007590 . . . . .
. . . . . . . .
. . . . . .
Vv04g0037 . . . . . . .
. . . . . . . .
. . . . .
Vv04g0038 . . . . . . .
. . . . . . . .
. . . . .
Vv04g0039 Tc01_g007500 . . . . .
. . . . . . . .
. . . . . .
Vv04g0040 . . . . . . .
. . . . . . . .
. . . . .
Vv04g0041 Tc01_g007220 . . . . .
. . . . . . . .
. . . . . .
Vv04g0042 . . . . . . .
. . . . . . . .
. . . . .
Vv04g0043 . . Gorai.001G009600.1 .
Gorai.003G061300.1 . . . . . .
. . . . . . . .
.
Vv04g0044 . . . . . . .
. . . . . . . .
. . . . .
Vv04g0045 Tc01_g026460 . . . . .
. . . . . . . .
. . . . . .
Vv04g0046 . Gorai.013G229500.1 Gorai.001G025300.1
Gorai.004G282500.1 Gorai.003G061200.1 .
Vv11g0300 Tc09_g008680 Gorai.013G229500.1 Gorai.
001G025300.1 . Gorai.009G028500.1 Gorai.010G078500.1
. . . . . . .
Vv04g0047 . . . . . . .
. . . . . . . .
. . . . .
Vv04g0048 . . . Gorai.004G282600.1 .
. . . . . . . .
. . . . . . .
Vv04g0049 . Gorai.013G229200.1 Gorai.001G025000.1
. Gorai.003G061100.1 . Vv11g0291
Tc09_g008570 Gorai.013G229200.1 Gorai.001G025000.1 .
Gorai.009G029100.1 Gorai.010G079100.1 . .
. . . . .
Vv04g0050 Tc01_g026890 . . . . .

. . . . . . . .
. . . . . .
Vv04g0051 . . Gorai.001G024800.1 . .
. . . . . . . .
. . . . . . .
Vv04g0052 . . Gorai.001G024700.1 . .
. . . . . . . .
. . . . . . .
Vv04g0053 . . Gorai.001G024600.1 . .
. . . . . . . .
. . . . . . .
Vv04g0054 . . Gorai.001G024500.1 . .
. . . . . . . .
. . . . . . .
Vv04g0055 . Gorai.013G219900.1 . . .
. Vv11g0285 . . . . .
Gorai.010G079600.1 . . . . .
. .
Vv04g0056 . . . . . . .
. . . . . . . .
. . . . .
Vv04g0057 . Gorai.013G229000.1 Gorai.001G009900.1
Gorai.004G283000.1 . . Vv11g0281
Tc09_g008500 Gorai.013G229000.1 Gorai.001G024400.1
Gorai.004G283000.1 Gorai.009G047600.1 . . .
. . . . .
Vv04g0058 Tc01_g007040 . . . . .
. . . . . . . .
. . . . . .
Vv04g0059 . . . . . . .
. . . . . . . .
. . . . .
Vv04g0060 . . . . . . .
. . . . . . . .
. . . . .
Vv04g0061 . . . . . .
Vv09g0256 . . . . . .
Vv11g0276 Tc09_g008450 Gorai.013G228900.1 Gorai.
001G010300.1 Gorai.004G283100.1 Gorai.009G029400.1
Gorai.010G080200.1
Vv04g0062 . . . . . . .
. . . . . . . .
. . . . .
Vv04g0063 . . . Gorai.004G288200.1 .
. . . . . . . .
. . . . . . .
Vv04g0064 . . . . Gorai.003G060400.1
. Vv09g0250 . . . . .
. . . . . . . .
Vv04g0065 . . Gorai.001G024100.1 . .

. Vv09g0247 . . . . .
. . . . . . . .
Vv04g0066 . . . Gorai.004G288100.1 .
. . . . . . . .
. . . . . . .
Vv04g0067 . . . Gorai.004G288000.1 .
. . . . . . . .
. . . . . . .
Vv04g0068 . . . . . . .
. . . . . . . .
. . . . .
Vv04g0069 . . . . Gorai.003G060300.1
Gorai.011G068900.1 Vv09g0245 . . .
. Gorai.011G068900.1 . . . .
. . . .
Vv04g0070 . . . . . . .
. . . . . . . .
. . . . .
Vv04g0071 . . Gorai.001G023700.1 . .
. . . . . . . .
. . . . . . .
Vv04g0072 . . Gorai.001G023600.1 . .
. . . . . . . .
. . . . . . .
Vv04g0073 . . . . . . .
. . . . . . . .
. . . . .
Vv04g0074 . . . . . . .
. . . . . . . .
. . . . .
Vv04g0075 . . . . . . .
. . . . . . . .
. . . . .
Vv04g0076 . . . . . . .
. . . . . . . .
. . . . .
Vv04g0077 . . . . . . .
. . . . . . . .
. . . . .
Vv04g0078 . . . . . Gorai.
011G067900.1 Vv09g0234 . . Gorai.010G231600.1
. Gorai.011G067900.1 . . . .
. . . .
Vv04g0079 . . . Gorai.004G287500.1 .
. . . . . . . .
. . . . . . .
Vv04g0080 . . . . . . .
. . . . . . . .
. . . . .
Vv04g0081 . . . . . . .

. . . . . . . .
. . . . .
Vv04g0082 . . Gorai.001G023500.1 . .
. . . . . . . .
. . . . . . .
Vv04g0083 . . Gorai.001G023300.1 .
Gorai.003G060000.1 Gorai.011G067400.1 Vv09g0225 .
. . . Gorai.011G067400.1 .
Vv11g0258 . . Gorai.001G023300.1 .
Gorai.009G046000.1 .
Vv04g0084 . . . . . . .
. . . . . . . .
. . . . .
Vv04g0085 . . Gorai.001G011000.1 . .
. . . . . . . .
Vv11g0251 Tc09_g008200 . Gorai.001G023200.1
. Gorai.009G045800.1 Gorai.010G081500.1
Vv04g0086 . . . . . . .
. . . . . . . .
. . . . .
Vv04g0087 . . . . . Gorai.
011G067300.1 Vv09g0223 . . . .
Gorai.011G067300.1 . Vv11g0250 Tc09_g008190 .
. . Gorai.009G030700.1 .
Vv04g0088 Tc09_g001780 . Gorai.001G023100.1 .
. . . . . . . .
. . . . . . . .
Vv04g0089 . . Gorai.001G023000.1 Gorai.
004G287300.1 . . . . . . .
. . . . . . . .
.
Vv04g0090 . . . . . . .
. . . . . . Vv11g0243
. . . . Gorai.009G030900.1
Gorai.010G081800.1
Vv04g0091 . Gorai.013G220000.1 . . .
. . . . . . . .
. . . . . . .
Vv04g0092 Tc01_g022970 . . . . .
. . . . . . . .
. . . . . .
Vv04g0093 Tc01_g034390 . . . . .
. . . . . . . .
. . . . . .
Vv04g0094 . . . . . . .
. . . . . . . .
. . . . .
Vv04g0095 Tc01_g006940 . . . . .
. . . . . . . .
. . . . . .

Vv04g0096 Tc01_g034580 . . . . .
. . . . . . . .
. . . . . .
Vv04g0097 . . . . . . .
. . . . . . . .
. . . . .
Vv04g0098 . . . . . . .
. . . . . . . .
. . . . .
Vv04g0099 . . Gorai.001G022500.1 . .
. . . . . . . .
. . . . . . .
Vv04g0100 . . . . . . .
. . . . . . . .
. . . . .
Vv04g0101 . . . . . . .
. . . . . . . .
. . . . .
Vv04g0102 . . . . . . .
. . . . . . . .
. . . . .
Vv04g0103 Tc01_g034790 Gorai.013G228500.1 . .
. . . . . . . .
. . . . . . . .
Vv04g0104 . Gorai.013G227200.1 Gorai.001G022400.1
. . . . . . . .
. . . . . . . .
.
Vv04g0105 . . Gorai.001G022300.1 . .
. . . . . . . .
. . . . . . .
Vv04g0106 . . . . . . .
. . . . . . . .
. . . . .
Vv04g0107 Tc01_g021630 . . . . .
. . . . . . . .
. . . . . .
Vv04g0108 . . . . . . .
. . . . . . . .
. . . . .
Vv04g0109 Tc01_g029760 . . . . .
. . . . . . . .
. . . . . .
Vv04g0110 Tc01_g034860 . . . . .
. . . . . . . .
. . . . . .
Vv04g0111 . . . . Gorai.003G057000.1
. Vv09g0209 . . . .
Gorai.011G064900.1 . . . . . .
. .

Vv04g0112 . . . . . Gorai.
011G064900.1 . . . . . . .
. . . . . . .
Vv04g0113 Tc01_g029920 . . . .
Gorai.011G064700.1 Vv09g0207 Tc06_g008840 . .
. Gorai.011G064700.1 . . . .
. . . .
Vv04g0114 . Gorai.013G220100.1 Gorai.001G022200.1
. . . . . . . .
. . . . . . . .
.
Vv04g0115 Tc01_g030320 . . . . .
. . . . . . . .
. . . . . .
Vv04g0116 . . . . . Gorai.
011G061800.1 . . . . . . .
Vv11g0206 Tc09_g007750 . . .
Gorai.009G044400.1 Gorai.010G083600.1
Vv04g0117 . . . . . . .
. . . . . . . .
. . . . .
Vv04g0118 . . Gorai.001G012200.1 . .
. . . . . . . .
. . . . . . .
Vv04g0119 Tc01_g035120 . . Gorai.004G286900.1
. . . . . . . .
. . . . . . . .
Vv04g0120 Tc01_g022920 . . . . .
. . . . . . . .
. . . . . .
Vv04g0121 . . . . . . .
. . . . . . . .
. . . . .
Vv04g0122 . . . . . . .
. . . . . . . .
. . . . .
Vv04g0123 . . . . . . .
. . . . . . . .
. . . . .
Vv04g0124 . . . . . . .
. . . . . . . .
. . . . .
Vv04g0125 Tc09_g001960 . . . . .
. . . . . . . .
. . . . . .
Vv04g0126 . . . . . . .
. . . . . . . .
. . . . .
Vv04g0127 . . . . . . .
. . . . . . . .

```
        .       .       .       .       .
Vv04g0128       Tc01_g022480    .       .       .       .
Gorai.001G107000.1      .       .       .       .       .       .
        .       .       .       .       .       .       .       .
Vv04g0129       .       .       .       .       .       .       .
        .       .       .       .       .       .       .       .
        .       .       .       .       .
Vv04g0130       .       .       .       .       .       .       .
        .       .       .       .       .       .       .       .
        .       .       .       .       .
Vv04g0131       .       .       .       .       .       .       .
        .       .       .       .       .       .       .       .
        .       .       .       .       .
Vv04g0132       .       .       .       .       .       .       .
        .       .       .       .       .       .       .       .
        .       .       .       .       .
Vv04g0133       .       .       .       Gorai.004G286500.1      .
        .       .       .       .       .       .       .       .
        .       .       .       .       .       .       .
Vv04g0134       Tc01_g022260    .       .       .       .       .
        .       .       .       .       .       .       .       .
        .       .       .       .       .       .
Vv04g0135       Tc01_g006540    .       .       .       .       .
        .       .       .       .       .       .       .       .
        .       .       .       .       .       .
Vv04g0136       .       Gorai.013G226800.1      .       .       .
        .       .       .       .       .       .       .       .
        Vv11g0176       .       Gorai.013G226800.1      .       .
        .       .
Vv04g0137       Tc01_g030700    .       .       Gorai.004G286400.1
        .       .       .       .       .       .       .       .
        .       .       .       .       .       .       .       .
Vv04g0138       .       .       .       .       .       .       .
        .       .       .       .       .       .       .       .
        .       .       .       .       .
Vv04g0139       .       .       .       Gorai.004G284200.1      .
        .       .       .       .       .       .       .       .
        .       .       .       .       .       .       .
Vv04g0140       .       .       .       .       .       .       .
        .       .       .       .       .       .       Vv11g0173
        Tc09_g007450    .       Gorai.001G013500.1      .       .
        .
Vv04g0141       .       Gorai.013G220700.1      Gorai.001G021800.1
        .       .       .       Vv09g0170       .       .       .
        .       .       .       .       .       .       .       .
        .       .
Vv04g0142       .       .       .       .       .       .       .
        .       .       .       .       .       .       .       .
        .       .       .       .       .
Vv04g0143       .       .       .       .       .       .       .
```

| | | | | | | | |
|---|---|---|---|---|---|---|---|
| . | . | . | . | . | . | . | . |
| . | . | . | . | . | | | |
| Vv04g0144 | . | . | . | . | . | . | . |
| . | . | . | . | . | . | . | . |
| . | . | . | . | . | | | |
| Vv04g0145 | . | Gorai.013G226500.1 | | | Gorai.001G021600.1 | | |
| . | . | . | . | . | . | . | . |
| . | . | Vv11g0159 | | . | . | . | . |
| . | . | | | | | | |
| Vv04g0146 | Tc01_g035290 | | . | . | . | . | . |
| . | . | . | . | . | . | . | . |
| . | . | . | . | . | . | | |
| Vv04g0147 | Tc01_g016710 | | . | . | . | . | . |
| . | . | . | . | . | . | . | . |
| . | . | . | . | . | . | | |
| Vv04g0148 | . | . | . | . | . | . | |
| Vv13g0555 | . | . | . | . | . | . | . |
| . | . | . | . | . | . | | |
| Vv04g0149 | . | . | Gorai.001G021400.1 | | | . | . |
| . | Vv13g0535 | | . | . | Gorai.011G275900.1 | | |
| . | . | . | . | . | . | . | . |
| . | . | | | | | | |
| Vv04g0150 | . | . | . | . | . | . | |
| Vv13g0532 | . | . | . | . | . | . | . |
| . | . | . | . | . | . | | |
| Vv04g0151 | . | . | . | . | . | . | |
| Vv13g0514 | . | . | . | . | . | . | . |
| . | . | . | . | . | . | | |
| Vv04g0152 | . | . | . | . | . | . | . |
| . | . | . | . | . | . | . | . |
| . | . | . | . | . | | | |
| Vv04g0153 | . | . | . | . | . | . | . |
| . | . | . | . | . | . | . | . |
| . | . | . | . | . | | | |
| Vv04g0154 | . | . | . | . | . | . | . |
| . | . | . | . | . | . | . | . |
| . | . | . | . | . | | | |
| Vv04g0155 | . | . | . | . | . | . | |
| Vv13g0508 | . | . | . | . | . | . | . |
| . | . | . | . | . | . | | |
| Vv04g0156 | . | . | . | . | . | . | |
| Vv13g0506 | . | . | . | . | . | . | . |
| . | . | . | . | . | . | | |
| Vv04g0157 | . | . | . | . | . | . | |
| Vv13g0501 | . | . | . | . | . | . | . |
| . | . | . | . | . | . | | |
| Vv04g0158 | . | . | . | . | . | . | |
| Vv13g0500 | . | . | . | . | . | . | . |
| . | . | . | . | . | . | | |
| Vv04g0159 | . | . | . | . | . | . | |

Vv13g0499 . . . . . . .
. . . . . .
Vv04g0160 . . . . . .
Vv13g0498 . . . . . . .
. . . . . .
Vv04g0161 . . . . . . .
. . . . . . . .
. . . . .
Vv04g0162 . . . . . . .
. . . . . . . .
. . . . .
Vv04g0163 . . . . . . .
. . . . . . . .
. . . . .
Vv04g0164 Tc09_g002350 . . . . .
. . . . . . . .
. . . . . .
Vv04g0165 Tc01_g031130 Gorai.013G220800.1 . .
. . . . . . . .
. . . . . . . .
Vv04g0166 . Gorai.013G226400.1 Gorai.001G021300.1
. . . Vv09g0140 . . .
. . . Vv11g0146 Tc09_g007320
Gorai.013G226400.1 . . Gorai.009G041700.1
Gorai.010G086100.1
Vv04g0167 Tc01_g035320 . . . . .
. . . . . . . .
. . . . . .
Vv04g0168 . . Gorai.001G021100.1 . .
. . . . . . . .
. . . . . . .
Vv04g0169 . . Gorai.001G021000.1 . .
. Vv09g0132 . . . . .
. Vv11g0141 Tc09_g007270 . . .
Gorai.009G041500.1 Gorai.010G086600.1
Vv04g0170 Tc01_g035500 . . . . .
. . . . . . . .
. . . . . .
Vv04g0171 . . Gorai.001G020900.1 . .
. . . . . . . .
Vv11g0138 Tc09_g007230 . . .
Gorai.009G041300.1 Gorai.010G086800.1
Vv04g0172 . . . . . . .
. . . . . . Vv11g0136
Tc09_g007220 Gorai.013G225800.1 Gorai.001G014500.1
. . .
Vv04g0173 . . . . . . .
. . . . . . . .
. . . . .
Vv04g0174 . . Gorai.001G020800.1 . .

. Vv09g0128 . . . . .
. Vv11g0134 Tc09_g007200 . Gorai.
001G014700.1 . Gorai.009G041200.1 .
Vv04g0175 . . . . . . .
. . . . . . . .
. . . . .
Vv04g0176 . . . . . . .
. . . . . . . .
. . . . .
Vv04g0177 Tc01_g035730 . Gorai.001G020700.1 .
. . . . . . . .
. . . . . . . .
Vv04g0178 . . . . . . .
. . . . . . . .
. . . . .
Vv04g0179 . . . . . . .
. . . . . . . .
. . . . .
Vv04g0180 . . . . . . .
. . . . . . . .
. . . . .
Vv04g0181 . . . . . . .
. . . . . . . .
. . . . .
Vv04g0182 . . . . . . .
. . . . . . . .
. . . . .
Vv04g0183 . . . . . . .
. . . . . . . .
. . . . .
Vv04g0184 . . . . . . .
. . . . . . . .
. . . . .
Vv04g0185 . . . . . . .
. . . . . . . .
. . . . .
Vv04g0186 . . Gorai.001G020300.1 . .
. . . . . . . .
. . . . . . .
Vv04g0187 Tc01_g017140 . . . . .
. . . . . . . .
. . . . . .
Vv04g0188 Tc01_g031460 . . . . .
. . . . . . . .
. . . . . .
Vv04g0189 . Gorai.013G221000.1 . . .
. . . . . . . .
. . . . . . .
Vv04g0190 Tc09_g002550 . Gorai.001G020200.1 .
. . . . . . . .

```
         .        .        .        .        .        .        .        .
Vv04g0191         .        .        Gorai.001G020100.1        .        .
         .        .        .        .        .        .        .        .
         .        .        .        .        .        .        .
Vv04g0192         .        .        .        .        .        .        .
         .        .        .        .        .        .        .        .
         .        .        .        .        .
Vv04g0193         .        .        Gorai.001G020000.1        .        .
         .        .        .        .        .        .        .        .
         .        .        .        .        .        .        .
Vv04g0194         .        .        .        .        .        .        .
         .        .        .        .        .        .        .        .
         .        .        .        .        .
Vv04g0195         .        .        Gorai.001G019900.1        .        .
         .        .        .        .        .        .        .        .
         .        .        .        .        .        .        .
Vv04g0196         Tc01_g028510      .        .        .        .        .
         .        .        .        .        .        .        .        .
         .        .        .        .        .        .
Vv04g0197         .        .        .        .        .        .        .
         .        .        .        .        .        .        .        .
         .        .        .        .        .
Vv04g0198         Tc09_g002700      .        .        .        .        .
         .        .        .        .        .        .        .        .
         .        .        .        .        .        .
Vv04g0199         .        .        .        .        .        .        .
         .        .        .        .        .        .        .        .
         .        .        .        .        .
Vv04g0200         .        .        .        .        .        .        .
         .        .        .        .        .        .        .        .
         .        .        .        .        .
Vv04g0201         .        .        .        .        .        .        .
         .        .        .        .        .        .        .        .
         .        .        .        .        .
Vv04g0202         .        .        Gorai.001G019700.1        .        .
         .        .        .        .        .        .        .        .
         Vv11g0105         Tc09_g006940      .        Gorai.001G015500.1
         .        Gorai.009G040300.1        .
Vv04g0203         Tc01_g016370      .        .        .        .        .
         .        .        .        .        .        .        .        .
         .        .        .        .        .        .
Vv04g0204         .        .        .        .        .        .        .
         .        .        .        .        .        .        .        .
         .        .        .        .        .
Vv04g0205         .        .        Gorai.001G019600.1        .        .
         .        .        .        .        .        .        .        .
         .        .        .        .        .        .        .
Vv04g0206         .        .        .        .        .        .        .
         .        .        .        .        .        .        .        .
         .        .        .        .        .
```

```
Vv04g0207        .        .        .        .        .        .
Vv09g0102        .        .        Gorai.010G235200.1         .        .
        .        .        .        .        .        .        .        .
Vv04g0208        .        .        .        .        .        .        .
        .        .        .        .        .        .        .        .
        .        .        .        .        .
Vv04g0209        Tc01_g031820      .        .        .        .        .
        .        .        .        .        .        .        .        .
        .        .        .        .        .        .
Vv04g0210        .        .        .        .        .        .        .
        .        .        .        .        .        .        .        .
        .        .        .        .        .
Vv04g0211        .        Gorai.013G223700.1         Gorai.001G019400.1
        .        .        .        .        .        .        .        .
        .        .        .        .        .        .        .        .
        .
Vv04g0212        .        Gorai.013G221200.1         Gorai.001G019300.1
        .        .        .        .        .        .        .        .
        .        .        Vv11g0081         Tc09_g006770      .        .
        .        Gorai.009G036300.1         Gorai.010G089300.1
Vv04g0213        .        Gorai.013G221300.1         .        .        .
        .        .        .        .        .        .        .        .
        .        .        .        .        .        .        .
Vv04g0214        Tc01_g028480      .        .        .        .        .
        .        .        .        .        .        .        .        .
        .        .        .        .        .        .
Vv04g0215        .        .        .        .        .        .        .
        .        .        .        .        .        .        .        .
        .        .        .        .        .
Vv04g0216        .        .        .        .        .        .        .
        .        .        .        .        .        .        .        .
        .        .        .        .        .
Vv04g0217        .        .        .        .        .        .        .
        .        .        .        .        .        .        .        .
        .        .        .        .        .
Vv04g0218        Tc01_g016100      Gorai.013G221400.1         .        .
        .        .        .        .        .        .        .        .
        .        Vv11g0053         .        .        .        .        .
        .
Vv04g0219        .        .        Gorai.001G019200.1         .        .
        .        .        .        .        .        .        .        .
        .        .        .        .        .        .        .
Vv04g0220        .        .        .        .        .        .        .
        .        .        .        .        .        .        .        .
        .        .        .        .        .
Vv04g0221        .        .        .        .        .        .        .
        .        .        .        .        .        .        .        .
        .        .        .        .        .
Vv04g0222        .        .        .        .        .        .        .
        .        .        .        .        .        .        .        .
```

. . . . .
Vv04g0223 . . . . . . .
. . . . . . . .
. . . . .
Vv04g0224 . Gorai.013G222700.1 Gorai.001G016800.1
. . . . . . . .
. . . . . . . .
.
Vv04g0225 . Gorai.013G221500.1 Gorai.001G014100.1
. . . . . . . .
. . . . . . . .
.
Vv04g0226 . . . . . . .
. . . . . . Vv11g0047
Tc09_g006430 . . . Gorai.009G037500.1
.
Vv04g0227 . . . . . . .
. . . . . . . .
. . . . .
Vv04g0228 . . . . . . .
. . . . . . . .
. . . . .
Vv04g0229 . . . . . . .
. . . . . . . .
. . . . .
Vv04g0230 . . . . . . .
. . . . . . . .
. . . . .
Vv04g0231 . . . . . . .
. . . . . . . .
. . . . .
Vv04g0232 . . . . . . .
. . . . . . . .
. . . . .
Vv04g0233 . . . . . Gorai.
010G122200.1 . . . . . . .
. . . . . . .
Vv04g0234 . . . . . Gorai.
010G122100.1 . . . . . . .
. . . . . . .
Vv04g0235 . . . . Gorai.009G067000.1
Gorai.010G122000.1 . . . . .
. . . . . . . .
.
Vv04g0236 . . . . . .
Vv09g0075 Tc06_g010170 . . . . .
. . . . . . .
Vv04g0237 . . . . . . .
. . . . . . . .
. . . . .

Vv04g0238 Tc01_g031900 . . . . .
. . . . . . . .
. . . . . .
Vv04g0239 . Gorai.013G218600.1 . .
Gorai.009G067500.1 . . . . . .
. . . . . . . .
.
Vv04g0240 . . . . . . .
. . . . . . . .
. . . . .
Vv04g0241 . Gorai.013G212300.1 Gorai.001G002000.1
. Gorai.009G067800.1 . . . .
. . . . . . . .
. . .
Vv04g0242 . . Gorai.001G001900.1 .
Gorai.009G067900.1 . . . . . .
. . . . . . . .
.
Vv04g0243 . . . . Gorai.009G068000.1
. . . . . . . .
. . . . . . .
Vv04g0244 . . . . . . .
. . . . . . . .
. . . . .
Vv04g0245 . . . . . . .
. . . . . . . .
. . . . .
Vv04g0246 Tc01_g006470 Gorai.013G212200.1 . .
Gorai.009G068100.1 . . . . .
. . . . . . . .
. .
Vv04g0247 . . . . . . .
. . . . . . . .
. . . . .
Vv04g0248 . . Gorai.001G001600.1 .
Gorai.009G068300.1 . Vv09g0068 . . .
. . . . . . . .
. .
Vv04g0249 . . . . . . .
. . . . . . . .
. . . . .
Vv04g0250 . . . . . . .
. . . . . . . .
. . . . .
Vv04g0251 Tc01_g015660 . . . . .
. . . . . . . .
. . . . . .
Vv04g0252 Tc01_g015650 . . . . .
. . . . . . . .
. . . . . .

Vv04g0253 . . Gorai.001G001500.1 .
Gorai.009G068400.1 . . . . . .
. . . . . . . .
.
Vv04g0254 . . Gorai.001G001400.1 . .
. . . . . . . .
. . . . . . .
Vv04g0255 . . . . Gorai.009G068700.1
Gorai.010G120400.1 . . . . .
. . . . . . . .
.
Vv04g0256 . Gorai.013G212100.1 . .
Gorai.009G068800.1 . . . . . .
. . . . . . . .
.
Vv04g0257 . . . . . Gorai.
010G117400.1 . . . . . . .
. . . . . . .
Vv04g0258 . . . . . . .
. . . . . . . .
. . . . .
Vv04g0259 . . . . . . .
. . . . . . . .
. . . . .
Vv04g0260 . . . . Gorai.009G068900.1
. . . . . . . .
. . . . . . .
Vv04g0261 . . . . . . .
. . . . . . . .
. . . . .
Vv04g0262 . Gorai.013G212000.1 . .
Gorai.009G069100.1 . . . . . .
. . . . . . . .
.
Vv04g0263 . . . . . . .
. . . . . . . .
. . . . .
Vv04g0264 . . . . . . .
. . . . . . . .
. . . . .
Vv04g0265 . . . . . . .
. . . . . . . .
. . . . .
Vv04g0266 . . . . . . .
. . . . . . . .
. . . . .
Vv04g0267 . . . . . . .
. . . . . . . .
. . . . .
Vv04g0268 . . . . . . .

```
.  .  .  .  .  .  .  .
.  .  .  .  .
Vv04g0269  .  .  .  .  .  .  .
.  .  .  .  .  .  .  .
.  .  .  .  .
Vv04g0270  .  .  .  .  .  .  .
.  .  .  .  .  .  .  .
.  .  .  .  .
Vv04g0271  Tc01_g015160  .  .  .  .  .
.  .  .  .  .  .  .  .
.  .  .  .  .  .
Vv04g0272  .  .  .  .  Gorai.009G069200.1
.  .  .  .  .  .  .  .
.  .  .  .  .  .  .
Vv04g0273  .  .  .  .  Gorai.009G069300.1
.  .  .  .  .  .  .  .
.  .  .  .  .  .  .
Vv04g0274  Tc01_g006840  .  .  .  .  .
.  .  .  .  .  .  .  .
.  .  .  .  .  .
Vv04g0275  .  Gorai.013G211900.1  .  .  .
.  .  .  .  .  .  .  .
.  .  .  .  .  .  .
Vv04g0276  .  .  .  .  .  .  .
.  .  .  .  .  .  .  .
.  .  .  .  .
Vv04g0277  .  .  .  .  .  .  .
.  .  .  .  .  .  .  .
.  .  .  .  .
Vv04g0278  Tc01_g036480  .  .  .  .  .
.  .  .  .  .  .  .  .
.  .  .  .  .  .
Vv04g0279  .  .  .  .  .  .  .
.  .  .  .  .  .  .  .
.  .  .  .  .
Vv04g0280  .  .  .  .  .  .  .
.  .  .  .  .  .  .  .
.  .  .  .  .
Vv04g0281  .  .  .  .  .  .  .
.  .  .  .  .  .  .  .
.  .  .  .  .
Vv04g0282  .  .  .  .  Gorai.009G069500.1
.  .  .  .  .  .  .  .
.  .  .  .  .  .  .
Vv04g0283  .  Gorai.013G211700.1  .  .
Gorai.009G069600.1  .  .  .  .  .  .
.  .  .  .  .  .  .  .
.
Vv04g0284  .  .  .  .  .  .  .
.  .  .  .  .  .  .  .
```

| | | | | | | | |
|---|---|---|---|---|---|---|---|
| Vv04g0285 | . | . | . | . | . | . | . |
| Vv04g0286 | . | . | . | . | . | . | . |
| Vv04g0287 | . | Gorai.013G211600.1 | | | . | . | . |
| Vv04g0288 | . | . | . | . | . | . | . |
| Vv04g0289 | . | . | . | . | . | . | . |
| Vv04g0290 | . | . | . | . | . | . | . |
| Vv04g0291 | . | . | . | . | . | . | . |
| Vv04g0292 | . | . | . | . | . | . | . |
| Vv04g0293 | Tc01_g006900 | | . | . | . | . | . |
| Vv04g0294 | . | . | . | . | . | . | . |
| Vv04g0295 | . | . | . | . | . | . | . |
| Vv04g0296 | . | . | . | . | . | . | . |
| Vv04g0297 | . | . | . | . | . | . | . |
| Vv04g0298 | Tc01_g036730 | | . | . | . | . | . |
| Vv04g0299 | Tc01_g014890 | | . | . | . | . | . |
| Vv04g0300 | . | . | . | . | . | . | . |
| Vv04g0301 | . | . | . | . | . | . | . |

```
         .       .       .       .       .       .       .       .
         .       .       .       .       .
Vv04g0302        .       .       Gorai.001G001300.1      .       .
         .       .       .       .       .       .       .       .
         .       .       .       .       .       .       .
Vv04g0303        Tc01_g036080    .       .       .       .       .
         .       .       .       .       .       .       .       .
         .       .       .       .       .       .
Vv04g0304        .       .       .       .       .       .       .
         .       .       .       .       .       .       .       .
         .       .       .       .       .
Vv04g0305        .       .       .       .       .       .       .
         .       .       .       .       .       .       .       .
         .       .       .       .       .
Vv04g0306        .       .       .       .       .       .       .
         .       .       .       .       .       .       .       .
         .       .       .       .       .
Vv04g0307        .       .       .       .       Gorai.009G069800.1
         .       .       .       .       .       .       .       .
         .       .       .       .       .       .       .
Vv04g0308        .       .       .       .       .       .       .
         .       .       .       .       .       .       .       .
         .       .       .       .       .
Vv04g0309        .       .       .       .       .       .       .
         .       .       .       .       .       .       .       .
         .       .       .       .       .
Vv04g0310        .       .       .       .       .       .       .
         .       .       .       .       .       .       .       .
         .       .       .       .       .
Vv04g0311        .       .       .       .       .       .       .
         .       .       .       .       .       .       .       .
         .       .       .       .       .
Vv04g0312        .       .       Gorai.001G001100.1      .       .
         .       .       .       .       .       .       .       .
         .       .       .       .       .       .       .
Vv04g0313        .       .       .       .       .       .       .
         .       .       .       .       .       .       .       .
         .       .       .       .       .
Vv04g0314        .       .       .       .       .       .       .
         .       .       .       .       .       .       .       .
         .       .       .       .       .
Vv04g0315        .       .       Gorai.001G001000.1      .       .
         .       .       .       .       .       .       .       .
         .       .       .       .       .       .       .
Vv04g0316        .       .       .       .       .       .       .
         .       .       .       .       .       .       .       .
         .       .       .       .       .
Vv04g0317        .       .       .       .       .       .       .
         .       .       .       .       .       .       .       .
         .       .       .       .       .
```

Vv04g0318 Tc09_g027780 Gorai.013G211400.1 Gorai.
001G000400.1 . . . . . . .
. . . . . . . .
. .
Vv04g0319 . . . . . . .
. . . . . . . .
. . . . .
Vv04g0320 Tc01_g035020 Gorai.013G208600.1 . .
. . . . . . . .
. . . . . . . .
Vv04g0321 . Gorai.013G219400.1 . . .
. . . . . . . .
. . . . . . .
Vv04g0322 . Gorai.013G219300.1 Gorai.001G008600.1
. . . Vv11g0334 Tc09_g009090
Gorai.013G219400.1 Gorai.001G008600.1 . . .
. . . . . . .
Vv04g0323 . . Gorai.001G026500.1 .
Gorai.009G048900.1 Gorai.010G105600.1 . . .
. . . . . . . .
. . .
Vv04g0324 . . . . Gorai.003G062800.1
Gorai.011G072300.1 Vv09g0350 . . .
. Gorai.011G072300.1 . . . .
. . . .
Vv04g0325 Tc01_g007200 . . . . .
. . . . . . . .
. . . . . .
Vv04g0326 . . . . . . .
. . . . . . . .
. . . . .
Vv04g0327 . . . . Gorai.009G049100.1
. . . . . . . .
. . . . . . .
Vv04g0328 Tc01_g036690 . Gorai.001G026800.1 .
. Gorai.010G105900.1 Vv09g0352
Tc06_g007710 . . . . . . .
. . . . .
Vv04g0329 . . . . Gorai.009G049200.1
. . . . . . . .
. . . . . . .
Vv04g0330 Tc01_g015300 . . Gorai.004G288400.1
Gorai.009G049300.1 Gorai.011G072500.1 Vv09g0353
Tc06_g007740 . Gorai.010G230500.1 .
Gorai.011G072500.1 . . . . . .
. .
Vv04g0331 . . . . . . .
. . . . . . . .
. . . . .
Vv04g0332 . . . . Gorai.009G049500.1

. . . . . . . .
. . . . . . .
Vv04g0333 . . . . . Gorai.
010G106200.1 . . . . . . .
. . . . . . .
Vv04g0334 . . . . . . .
. . . . . . . .
. . . . .
Vv04g0335 . . Gorai.001G026900.1 . .
. . . . . . . .
. . . . . . .
Vv04g0336 . . . . . Gorai.
010G106300.1 . . . . . . .
. . . . . . .
Vv04g0337 . Gorai.013G219200.1 Gorai.001G008200.1
Gorai.004G288600.1 Gorai.009G049700.1 . .
. . . . . . Vv11g0340
Tc09_g009150 Gorai.013G219200.1 Gorai.001G008200.1
. Gorai.009G027200.1 Gorai.010G075600.1
Vv04g0338 . . . . Gorai.009G049900.1
. . . . . . . .
. . . . . . .
Vv04g0339 Tc01_g038590 . Gorai.001G027000.1 .
. Gorai.010G106400.1 . . . .
. . . . . . . .
. .
Vv04g0340 Tc01_g038640 . Gorai.001G008100.1
Gorai.004G281600.1 Gorai.009G050200.1 Gorai.010G106600.1
. . . . . . . .
. . . . . .
Vv04g0341 . . . Gorai.004G289900.1 .
Gorai.010G107100.1 . . . . .
. . . . . . . .
.
Vv04g0342 . . . . . . .
. . . . . . Vv11g0344
Tc09_g009190 Gorai.013G219000.1 . .
Gorai.009G059200.1 .
Vv04g0343 . Gorai.013G219000.1 Gorai.001G007900.1
. . Gorai.010G107300.1 . . .
. . . . Vv11g0345
Tc09_g009200 Gorai.013G216000.1 Gorai.001G007900.1 .
Gorai.009G059300.1 .
Vv04g0344 . . . . . . .
. . . . . . . .
. . . . .
Vv04g0345 Tc01_g034910 Gorai.013G218900.1 . .
. Gorai.010G107600.1 . . . .
. . . . . . . .
. .

Vv04g0346 . . . . . . .
. . . . . . .
. . . . .
Vv04g0347 . . . . . . .
. . . . . . .
. . . . .
Vv04g0348 . . . . Gorai.009G050300.1
. . . . . . .
. . . . . . .
Vv04g0349 Tc01_g001470 . . . . .
. . . . . . .
. . . . . .
Vv04g0350 Tc09_g027340 . . . .
Gorai.010G107700.1 . . . . . .
. . . . . . .
Vv04g0351 Tc01_g039570 . . . . .
. . . . . . .
. . . . . .
Vv04g0352 . . . . . . .
. . . . . . .
. . . . .
Vv04g0353 . . . . . . .
. . . . . . .
. . . . .
Vv04g0354 Tc01_g039590 . . Gorai.004G281500.1
. . . . . . .
. . . . . . .
Vv04g0355 Tc01_g007270 Gorai.013G218800.1 . .
. Gorai.010G107800.1 . . . .
. . . . . . .
. .
Vv04g0356 . . . . . . .
. . . . . . .
. . . . .
Vv04g0357 Tc01_g001220 . . . Gorai.
003G064000.1 Gorai.010G107900.1 Vv09g0374
Tc06_g007990 . . . . . . .
. . . . .
Vv04g0358 . . . . . . .
. . . . . . .
. . . . .
Vv04g0359 . . . Gorai.004G281400.1 .
Gorai.010G108000.1 . . . . .
. . Vv11g0352 Tc09_g009270 . .
Gorai.004G281400.1 Gorai.009G026900.1 Gorai.
010G074500.1
Vv04g0360 . . . . Gorai.009G050400.1
. . . . . . .
. . . . . . .
Vv04g0361 . . . Gorai.004G281000.1 .

. . . . . . . .
Vv11g0358 Tc09_g009350 . . Gorai.
004G281000.1 . .
Vv04g0362 Tc01_g015360 . . . . .
. . . . . . .
Vv11g0369 . . . . . .
Vv04g0363 . . . . . .
Vv09g0385 Tc06_g008150 . . . . .
Vv11g0370 . . . . .
Vv04g0364 . . Gorai.001G006700.1 . .
. . . . . . . .
. . . . . . .
Vv04g0365 . . Gorai.001G027200.1 .
Gorai.009G050600.1 . . . . . .
. . . . . . . .
.
Vv04g0366 Tc01_g005590 . . Gorai.004G278000.1
. . . . . . . .
. . . . . . . .
Vv04g0367 . Gorai.013G218700.1 . Gorai.
004G290400.1 . Gorai.010G108200.1 . . .
. . . . . . . .
. . .
Vv04g0368 . . . . . . .
. . . . . . . .
. . . . .
Vv04g0369 . . Gorai.001G027500.1 . .
. . . . . . . .
. . . . . . .
Vv04g0370 . . Gorai.001G027600.1 . .
. Vv09g0391 . . . . .
. . . . . . . .
Vv04g0371 . . Gorai.001G006500.1 . .
Gorai.011G074200.1 . . . . .
. . Vv11g0376 Tc09_g009530 .
Gorai.001G006500.1 . Gorai.009G025900.1 Gorai.
010G073200.1
Vv04g0372 . . . . . Gorai.
010G108500.1 . . . . . . .
. . . . . . .
Vv04g0373 . . Gorai.001G003700.1 . .
Gorai.010G108600.1 . . . . .
. . . . . . . .
.
Vv04g0374 . . . . . . .
. . . . . . . .
. . . . .
Vv04g0375 . . Gorai.001G027900.1 Gorai.
004G290500.1 . Gorai.010G108800.1 . . .
. . . . . . . .

.	.	.
Vv04g0376	.	.	.	.	.	Gorai.
010G108900.1	.	.	.	.	.	.	.
.	.	.	.	.	.	.
Vv04g0377	.	.	.	.	Gorai.009G051200.1
.	.	.	.	.	.	.	.
.	.	.	.	.	.	.
Vv04g0378	.	.	.	.	.	.	.
.	.	.	.	.	.	.	.
.	.	.	.	.
Vv04g0379	.	.	Gorai.001G028100.1	.	.
.	.	.	.	.	.	.	.
Vv11g0381	Tc09_g009580	.	Gorai.001G028000.1
.	Gorai.009G060500.1	Gorai.010G072900.1
Vv04g0380	.	.	Gorai.001G028200.1	.	.
.	.	.	.	.	.	.	.
.	.	.	.	.	.	.
Vv04g0381	Tc01_g005710	.	.	.	.	.
.	.	.	.	.	.	.	.
.	.	.	.	.	.
Vv04g0382	.	.	.	.	.	.	.
.	.	.	.	.	.	Vv11g0383
.	.	Gorai.001G028200.1	.	.	.
Vv04g0383	.	.	.	.	.	.	.
.	.	.	.	.	.	.	.
.	.	.	.	.
Vv04g0384	.	.	Gorai.001G028400.1	.	.
.	.	.	.	.	.	.	.
.	.	.	.	.	.	.
Vv04g0385	.	.	Gorai.001G028600.1	Gorai.
004G290600.1	.	.	.	.	.	.	.
.	.	.	.	.	.	.	.
.
Vv04g0386	.	.	Gorai.001G028700.1	.
Gorai.009G051700.1	.	Vv09g0398	.	.	.
.	Gorai.011G018500.1	.	.	.	.
.	.	.	.
Vv04g0387	.	.	.	.	Gorai.009G051800.1
.	.	.	.	.	.	.	.
.	.	.	.	.	.	.
Vv04g0388	Tc01_g039720	.	.	.	.	.
.	.	.	.	.	.	.	.
.	.	.	.	.	.
Vv04g0389	Tc01_g039880	.	.	.	.	.
.	.	.	.	.	.	.	.
.	.	.	.	.	.
Vv04g0390	.	.	.	Gorai.004G290800.1	.
.	.	.	.	.	.	.	.
Vv11g0393	Tc09_g009680	.	.	.
Gorai.009G025400.1	Gorai.010G072100.1

Vv04g0391 . . . . . . .
. . . . . . . .
. . . . .
Vv04g0392 . . . Gorai.004G290900.1 .
. . . . . . . .
. . . . . . .
Vv04g0393 . . . . . . .
. . . . . . . .
. . . . .
Vv04g0394 . . Gorai.001G028800.1 .
Gorai.009G051900.1 Gorai.011G074900.1 Vv09g0416 .
. . . Gorai.011G074900.1 . .
. . . . . .
Vv04g0395 . . . . . . .
. . . . . . . .
. . . . .
Vv04g0396 . . Gorai.001G028900.1 .
Gorai.009G052000.1 Gorai.010G109400.1 . . .
. . . . Vv11g0403
Tc09_g009820 . Gorai.001G028900.1 . Gorai.
009G024800.1 Gorai.010G071100.1
Vv04g0397 . . . . . . .
. . . . . . . .
. . . . .
Vv04g0398 . . . . . . .
. . . . . . . .
. . . . .
Vv04g0399 . . . . . . .
. . . . . . . .
. . . . .
Vv04g0400 . . . . . . .
. . . . . . . .
. . . . .
Vv04g0401 . . . . Gorai.009G052100.1
. . . . . . . .
. . . . . . .
Vv04g0402 . . . . . . .
. . . . . . . .
. . . . .
Vv04g0403 . Gorai.013G218600.1 . . .
. . . . . . . .
. . . . . . .
Vv04g0404 . . . . . . .
. . . . . . . .
. . . . .
Vv04g0405 . . . . . . .
. . . . . . . .
. . . . .
Vv04g0406 . . . . . . .
. . . . . . . .

Vv04g0407 Gorai.009G052200.1
Vv04g0408
Vv04g0409
Vv04g0410
Vv04g0411
Vv04g0412
Vv04g0413
Vv04g0414
Vv04g0415
Vv04g0416
Vv04g0417
Vv04g0418
Vv04g0419
Vv04g0420
Vv04g0421
Vv04g0422
Vv04g0423

```
        .       .       .       .       .       .       .       .
        .       .       .       .       .
Vv04g0424       .       .       .       .       .       .       .
        .       .       .       .       .       .       .       .
        .       .       .       .       .
Vv04g0425       .       .       .       .       .       .       .
        .       .       .       .       .       .       .       .
        .       .       .       .       .
Vv04g0426       .       .       .       .       .       .       .
        .       .       .       .       .       .       .       .
        .       .       .       .       .
Vv04g0427       .       .       .       .       .       .       .
        .       .       .       .       .       .       Vv11g0405
        .       .       .       .       .       .
Vv04g0428       .       .       .       .       .       .       .
        .       .       .       .       .       .       .       .
        .       .       .       .       .
Vv04g0429       .       .       .       .       .       .       .
        .       .       .       .       .       .       .       .
        .       .       .       .       .
Vv04g0430       .       .       .       .       .       .       .
        .       .       .       .       .       .       .       .
        .       .       .       .       .
Vv04g0431       .       .       Gorai.001G029000.1      .
Gorai.009G052400.1      .       .       .       .       .       .
        .       .       .       .       .       .       .       .
        .
Vv04g0432       .       .       .       .       Gorai.009G052500.1
        .       Vv09g0437       Tc06_g008660    .       .       .
        .       .       .       .       .       .       .       .
        .
Vv04g0433       .       .       .       .       .       .       .
        .       .       .       .       .       .       .       .
        .       .       .       .       .
Vv04g0434       .       Gorai.013G218300.1      .       .
Gorai.009G052700.1      .       .       .       .       .       .
        .       .       .       .       .       .       .       .
        .
Vv04g0435       .       .       .       .       .       .       .
        .       .       .       .       .       .       .       .
        .       .       .       .       .
Vv04g0436       Tc01_g035520    .       .       .       Gorai.
009G052800.1    .       .       .       .       .       .       .
        .       .       .       .       .       .       .       .
Vv04g0437       .       .       .       .       .       .       .
        .       .       .       .       .       .       .       .
        .       .       .       .       .
Vv04g0438       .       .       .       .       .       .       .
        .       .       .       .       .       .       .       .
        .       .       .       .       .
```

Vv04g0439 . . . . . . .
. . . . . . . .
. . . . .
Vv04g0440 . . . . . . .
. . . . . . . .
. . . . .
Vv04g0441 Tc01_g035360 . . . . .
. . . . . . . .
. . . . . .
Vv04g0442 . . . . . . .
. . . . . . . .
. . . . .
Vv04g0443 . Gorai.013G231300.1 . . .
. . . . . . . .
. . . . . . .
Vv04g0444 . . . . . . .
. . . . . . . .
. . . . .
Vv04g0445 Tc01_g034490 . . . . .
. . . . . . . .
. . . . . .
Vv04g0446 . . . . . . .
. . . . . . . .
. . . . .
Vv04g0447 Tc01_g031480 . . . . .
. . . . . . . .
. . . . . .
Vv04g0448 . . . . . . .
. . . . . . . .
. . . . .
Vv04g0449 . . . . . . .
. . . . . . . .
. . . . .
Vv04g0450 . . . . . . .
. . . . . . . .
. . . . .
Vv04g0451 . . . . . . .
. . . . . . . .
. . . . .
Vv04g0452 . . . . . . .
. . . . . . . .
. . . . .
Vv04g0453 Tc01_g031600 . . . . .
. . . . . . . .
. . . . . .
Vv04g0454 . . . . . . .
. . . . . . . .
. . . . .
Vv04g0455 . . . . . . .
. . . . . . Vv11g0436

```
        Tc09_g010160     .        .       .        Gorai.009G023200.1
        .
Vv04g0456        .       .        .       .        .       .        .
        .        .       .        .       .        .       .        .
        .        .       .        .       .
Vv04g0457        .       .        .       .        .       .        .
        .        .       .        .       .        .       .        .
        .        .       .        .       .
Vv04g0458        .       .        .       .        .       .
Vv09g0445        .       .        .       .        .       .        .
        .        .       .        .       .        .
Vv04g0459        .       .        .       Gorai.004G279500.1        .
        .        Vv09g0461        Tc06_g000240     .       .
Gorai.009G115200.1       .        .       .        .       .        .
        .        .       .
Vv04g0460        .       .        .       .        .       .        .
        .        .       .        .       .        .       .        .
        .        .       .        .       .
Vv04g0461        .       .        .       .        .       .
Vv09g0552        .       .        .       .        Gorai.011G079400.1
        .        .       .        .       .        .       .        .
Vv04g0462        .       .        .       .        .       .        .
        .        .       .        .       .        .       .        .
        .        .       .        .       .
Vv04g0463        .       .        .       .        .       .        .
        .        .       .        .       .        .       .        .
        .        .       .        .       .
Vv04g0464        .       .        .       .        .       .        .
        .        .       .        .       .        .       .        .
        .        .       .        .       .
Vv04g0465        .       .        .       .        .       .        .
        .        .       .        .       .        .       .        .
        .        .       .        .       .
Vv04g0466        .       Gorai.013G218200.1        .       .        .
        .        .       .        .       .        .       .        .
        .        .       .        .       .        .       .
Vv04g0467        .       .        .       .        .       .        .
        .        .       .        .       .        .       .        .
        .        .       .        .       .
Vv04g0468        .       .        .       .        Gorai.009G052900.1
        .        .       .        .       .        .       .        .
        .        .       .        .       .        .       .
Vv04g0469        .       .        Gorai.001G029300.1       .        .
        .        .       .        .       .        .       .        .
        .        .       .        .       .        .       .
Vv04g0470        Tc01_g026460     .       .        .       .        .
        .        .       .        .       .        .       .        .
        .        .       .        .       .        .
Vv04g0471        Tc01_g013210     .       .        .       .        .
        .        .       .        .       .        .       .        .
```

```
        .      .      .      .      .      .
Vv04g0472      .      .      .      .      .      .      .
        .      .      .      .      .      .      .      .
        .      .      .      .      .
Vv04g0473      .      .      .      .      .      .      .
        .      .      .      .      .      .      .      .
        .      .      .      .      .
Vv04g0474      .      .      .      .      .      .      .
        .      .      .      .      .      .      .      .
        .      .      .      .      .
Vv04g0475      .      .      .      .      .      .      .
        .      .      .      .      .      .      .      .
        .      .      .      .      .
Vv04g0476      Tc01_g034110  .      .      .      .      .
        .      .      .      .      .      .      .      .
        .      .      .      .      .      .
Vv04g0477      .      .      .      .      .      .      .
        .      .      .      .      .      .      .      .
        .      .      .      .      .
Vv04g0478      .      .      .      .      .      .      .
        .      .      .      .      .      .      .      .
        .      .      .      .      .
Vv04g0479      Tc01_g034060  .      .      .      .      .
        .      .      .      .      .      .      .      .
        .      .      .      .      .      .
Vv04g0480      Tc01_g029250  Gorai.013G232500.1   .      .
        .      .      .      .      .      .      .      .
        .      .      .      .      .      .      .      .
Vv04g0481      Tc01_g033850  .      .      Gorai.004G278700.1
        .      .      .      .      .      .      .      .
        .      .      .      .      .      .      .      .
Vv04g0482      .      .      .      .      .      .      .
        .      .      .      .      .      .      .      .
        .      .      .      .      .
Vv04g0483      .      .      .      .      .      .      .
        .      .      .      .      .      .      .      .
        .      .      .      .      .
Vv04g0484      .      .      .      .      .      .      .
        .      .      .      .      .      .      .      .
        .      .      .      .      .
Vv04g0485      .      .      .      .      .      .      .
        .      .      .      .      .      .      .      .
        .      .      .      .      .
Vv04g0486      .      .      .      .      .      .      .
        .      .      .      .      .      .      .      .
        .      .      .      .      .
Vv04g0487      .      .      .      .      .      .
Vv09g0559      .      .      .      .      .      .      .
        .      .      .      .      .      .
Vv04g0488      .      .      .      .      .      .      .
```

. . . . . . . .
. . . . .
Vv04g0489 . . . . . . .
. . . . . . . .
. . . . .
Vv04g0490 Tc01_g028380 . . . . .
. . . . . . . .
. . . . . .
Vv04g0491 Tc01_g013540 . . . . .
. . . . . . . .
. . . . . .
Vv04g0492 . . . . . . .
. . . . . . Vv11g0457
Tc09_g010300 . . . . Gorai.
010G068700.1
Vv04g0493 . . . . . . .
. . . . . . . .
. . . . .
Vv04g0494 . . . . . . .
. . . . . . . .
. . . . .
Vv04g0495 . . . . . . .
. . . . . . . .
. . . . .
Vv04g0496 . . . . . . .
. . . . . . . .
. . . . .
Vv04g0497 . . . . . . .
. . . . . . . .
. . . . .
Vv04g0498 . . . . . . .
. . . . . . . .
. . . . .
Vv04g0499 . . . . . . .
. . . . . . . .
. . . . .
Vv04g0500 Tc01_g026220 . . . . .
. . . . . . . .
. . . . . .
Vv04g0501 . . . . . . .
. . . . . . . .
. . . . .
Vv04g0502 . . . . . . .
. . . . . . . .
. . . . .
Vv04g0503 . . . . . . .
. . . . . . . .
. . . . .
Vv04g0504 Tc01_g029300 . . . . .
. . . . . . . .

.	.	.	.	.	.
Vv04g0505	Tc09_g018160	.	.	.	.	.
.	.	.	.	.	.	.	.
.	.	.	.	.	.
Vv04g0506	Tc01_g033570	.	.	.	.	.
.	.	.	.	.	.	.	.
.	.	.	.	.	.
Vv04g0507	.	.	.	.	.	.	.
.	.	.	.	.	.	.	.
.	.	.	.	.
Vv04g0508	.	.	.	.	.	.	.
.	.	.	.	.	.	.	.
.	.	.	.	.
Vv04g0509	.	.	.	.	.	.	.
.	.	.	.	.	.	.	.
.	.	.	.	.
Vv04g0510	Tc01_g007860	.	.	.	.	.
.	.	.	.	.	.	.	.
.	.	.	.	.	.
Vv04g0511	Tc01_g007840	.	.	.	.	.
.	.	.	.	.	.	.	.
.	.	.	.	.	.
Vv04g0512	.	.	.	Gorai.004G274500.1	.
.	Vv09g0567	Tc06_g006590	.	Gorai.
010G227500.1	.	.	.	Vv11g0493
Tc09_g010730	.	.	.	Gorai.009G010600.1	.
Vv04g0513	Tc09_g003920	Gorai.013G232800.1	Gorai.
001G029400.1	Gorai.004G278500.1	.	.	.	.
.	.	.	.	.	.	.	.
.	.	.	.
Vv04g0514	.	.	.	.	.	.	.
.	.	.	.	.	.	.	.
.	.	.	.	.
Vv04g0515	Tc09_g017500	.	.	.	.
Gorai.011G080200.1	Vv09g0574	.	.	Gorai.
010G227400.1	.	Gorai.011G080100.1	.	.	.
.	.	.	.	.
Vv04g0516	Tc01_g039340	.	.	Gorai.004G278300.1
.	.	.	.	.	.	.	.
.	.	.	.	.	.	.	.
Vv04g0517	.	.	.	Gorai.004G276800.1	.
.	.	.	.	.	.	.	.
.	.	.	.	.	.	.
Vv04g0518	.	.	.	.	.	Gorai.
011G080400.1	.	.	.	.	.	.	.
Vv11g0500	Tc09_g010790	.	.	.	.
Gorai.010G065700.1
Vv04g0519	Tc01_g033410	.	.	.	.	.
.	.	.	.	.	.	.	.
.	.	.	.	.	.

Vv04g0520 . . . Gorai.004G274400.1 .
. . . . . . . .
. . . . . . .
Vv04g0521 Tc01_g028480 . . . . .
. . . . . . . .
. . . . . .
Vv04g0522 . Gorai.013G201000.1 . . .
. . . . . . . .
. . . . . . .
Vv04g0523 Tc01_g033120 . . . . .
. . . . . . . .
. . . . . .
Vv04g0524 . . . . . Gorai.
011G080500.1 Vv09g0580 . . Gorai.010G227300.1
Gorai.009G129600.1 Gorai.011G080500.1 . .
. . . . . .
Vv04g0525 . . . . . . .
. . . . . . . .
. . . . .
Vv04g0526 . Gorai.013G233000.1 . . .
Gorai.011G080600.1 Vv09g0581 . .
Gorai.010G227200.1 Gorai.009G129700.1 Gorai.011G080600.1
. Vv11g0506 Tc09_g010830 Gorai.013G233000.1
. . Gorai.009G020100.1 Gorai.010G065500.1
Vv04g0527 Tc09_g003770 . . . . .
Vv09g0582 . . . . . .
. . . . . . .
Vv04g0528 . . . . . . .
. . . . . . . .
. . . . .
Vv04g0529 . . . . . . .
. . . . . . . .
. . . . .
Vv04g0530 . Gorai.013G233200.1 . . .
. Vv09g0593 . . Gorai.010G227000.1
Gorai.009G130100.1 . . . . .
. . . .
Vv04g0531 Tc01_g039010 Gorai.013G182100.1 . .
. . . . . . . .
. . . . . . . .
Vv04g0532 Tc01_g039000 Gorai.013G182300.1 . .
. . Vv09g0595 . . . .
. . . . . . . .
.
Vv04g0533 Tc01_g038970 Gorai.013G182900.1 . .
. . . . . . . .
. . . . . . . .
Vv04g0534 . . . Gorai.004G274300.1 .
Gorai.011G081400.1 Vv09g0610 . . .
. Gorai.011G081400.1 . . . .

. . . .
Vv04g0535 . . . Gorai.004G274200.1 .
. . . . . . . .
. . . . . . .
Vv04g0536 . . . Gorai.004G274100.1 .
. . . . . . . .
. . . . . . .
Vv04g0537 . . . . . . .
. . . . . . . .
. . . . .
Vv04g0538 . Gorai.013G195900.1 . . .
. . . . . . . .
. . . . . . .
Vv04g0539 . . . . . . .
. . . . . . . .
. . . . .
Vv04g0540 . . . . . . .
. . . . . . . .
. . . . .
Vv04g0541 . . . . . . .
. . . . . . . .
. . . . .
Vv04g0542 . Gorai.013G233800.1 . Gorai.
004G274000.1 . . Vv09g0626 . . .
. . . Vv11g0531 Tc09_g011130
Gorai.013G233800.1 . . Gorai.009G010100.1
Gorai.010G064500.1
Vv04g0543 . . . . . . .
. . . . . . . .
. . . . .
Vv04g0544 Tc01_g029390 . Gorai.001G041500.1
Gorai.004G271400.1 . . . . . .
. . . . . . . .
. .
Vv04g0545 . Gorai.013G215500.1 . . .
Gorai.011G082000.1 Vv09g0631 . . .
. Gorai.011G082000.1 . . . .
. . . .
Vv04g0546 . . Gorai.001G033000.1 . .
. . . . . . . .
. . . . . . .
Vv04g0547 . . . . . . .
. . . . . . . .
. . . . .
Vv04g0548 . Gorai.013G215400.1 . . .
. . . . . . . .
Vv11g0540 Tc09_g011240 . . . .
Gorai.010G064200.1
Vv04g0549 . Gorai.013G215300.1 . .
Gorai.009G057400.1 Gorai.010G114100.1 . . .

. . . . Vv11g0541
Tc09_g011250 Gorai.013G215300.1 . . Gorai.
009G057400.1 .
Vv04g0550 Tc09_g003530 . . . . .
. . . . . . . .
. . . . . .
Vv04g0551 Tc01_g007690 . . . . .
. . . . . . . .
. . . . . .
Vv04g0552 . . . . . . .
. . . . . . . .
. . . . .
Vv04g0553 . . . . . . .
. . . . . . . .
. . . . .
Vv04g0554 . Gorai.013G215200.1 . .
Gorai.009G057600.1 Gorai.010G114300.1 . . .
. . . . . . . .
. . .
Vv04g0555 . Gorai.013G234000.1 . . .
Gorai.010G114400.1 . . . . .
. . Vv11g0554 . . . .
. Gorai.010G063900.1
Vv04g0556 . . . . . .
Vv03g0651 . . Gorai.005G239000.1 . .
. . . . . . . .
Vv04g0557 . . . . . . .
. . . . . . . .
. . . . .
Vv04g0558 . . . . . Gorai.
010G114500.1 Vv03g0648 . . Gorai.005G239200.1
. . . . . . . .
. .
Vv04g0559 . . . . . . .
. . . . . . . .
. . . . .
Vv04g0560 . . . . . .
Vv03g0646 . . . . . . .
. . . . . .
Vv04g0561 . . . . . .
Vv03g0645 Tc02_g008610 . . . . .
. . . . . . .
Vv04g0562 . . . . . .
Vv03g0644 . . . . . . .
. . . . . .
Vv04g0563 . . . . . .
Vv03g0643 Tc02_g008600 Gorai.013G042500.1 . .
Gorai.008G025200.1 . . . . .
. . .
Vv04g0564 . . . . . .

Vv03g0642 . . . . . . .
. . . . .
Vv04g0565 . . Gorai.001G042100.1 . .
Gorai.011G082600.1 Vv09g0648 Tc06_g001600 .
. Gorai.009G130800.1 Gorai.011G082600.1 .
Vv11g0558 Tc09_g011370 . Gorai.001G042100.1
. Gorai.009G017600.1 .
Vv04g0566 . . . . . . .
. . . . . . . .
. . . . .
Vv04g0567 . . . . Gorai.009G057700.1
. . . . . . . .
. . . . . . .
Vv04g0568 Tc01_g038640 . . . . .
. . . . . . . .
. . . . . .
Vv04g0569 . . Gorai.001G042400.1 .
Gorai.009G057800.1 Gorai.011G083000.1 Vv09g0652
Tc06_g001640 . Gorai.010G225600.1 . Gorai.
011G083000.1 . Vv11g0567 Tc09_g011400 .
Gorai.001G033300.1 . Gorai.009G057800.1 .
Vv04g0570 . . . . . . .
. . . . . . . .
. . . . .
Vv04g0571 . . . . Gorai.009G057900.1
Gorai.011G083400.1 . . . . .
. . . . . . . .
.
Vv04g0572 Tc01_g038380 Gorai.013G215000.1 Gorai.
001G042600.1 . Gorai.009G058000.1 Gorai.010G114900.1
. . . . . . . .
. . . . . .
Vv04g0573 . Gorai.013G185900.1 Gorai.001G033600.1
. Gorai.009G058200.1 Gorai.010G115100.1 .
. . . . . . Vv11g0576
. Gorai.013G214800.1 Gorai.001G033600.1 .
Gorai.009G058200.1 .
Vv04g0574 . . . . . . .
. . . . . . . .
. . . . .
Vv04g0575 Tc01_g007680 . . . . .
. . . . . . . .
. . . . . .
Vv04g0576 . . Gorai.001G033700.1 . .
. . . . . . . .
. . . . . . .
Vv04g0577 . Gorai.013G234700.1 Gorai.001G033800.1
. Gorai.009G058400.1 Gorai.010G115300.1 .
. . . . . . Vv11g0583
. . Gorai.001G033800.1 . Gorai.

009G058400.1 .
Vv04g0578 Tc09_g027450 Gorai.013G214700.1 . .
. Gorai.010G115500.1 . . . .
. . . . . . . .
. .
Vv04g0579 Tc01_g017660 . Gorai.001G043600.1 .
Gorai.009G058500.1 . . . . .
. . . . . . . .
. .
Vv04g0580 Tc09_g003290 . . . . .
. . . . . . . .
. . . . . .
Vv04g0581 . . . . . . .
. . . . . . . .
. . . . .
Vv04g0582 Tc01_g007380 . Gorai.001G043900.1 .
Gorai.009G058600.1 . . . . .
. . . . . . . .
. .
Vv04g0583 . . Gorai.001G034200.1 . .
. . . . . . . .
Vv11g0608 Tc09_g011820 . Gorai.001G034200.1
. Gorai.009G016000.1 .
Vv04g0584 . . Gorai.001G034300.1 . .
Gorai.010G115600.1 Vv09g0691 . . .
Gorai.009G127600.1 . . . . .
. . . .
Vv04g0585 . . . . . . .
. . . . . . . .
. . . . .
Vv04g0586 Tc01_g004580 . . . Gorai.
009G058700.1 . . . . . . .
. . . . . . . .
Vv04g0587 . . Gorai.001G034500.1 . .
. . . . . . . .
. . . . . . .
Vv04g0588 . . . . Gorai.009G058800.1
Gorai.010G115800.1 . . . . .
. . . . . . . .
.
Vv04g0589 . . Gorai.001G034600.1 . .
Gorai.011G084800.1 Vv09g0704 Tc06_g003130 .
. . Gorai.011G084800.1 . . .
. . . . .
Vv04g0590 Tc01_g038070 Gorai.013G214600.1 Gorai.
001G034700.1 . . . . . . .
. . . Vv11g0620 Tc09_g011940 .
Gorai.004G107700.1 Gorai.004G275600.1 Gorai.
009G015500.1 .
Vv04g0591 . . . . . . .

. . . . . . . .
. . . . .
Vv04g0592 Tc09_g003010 Gorai.013G214400.1 Gorai.
001G034800.1 . Gorai.009G058900.1 . . .
. . . . . Vv11g0621
Tc09_g011950 . Gorai.001G034800.1 . Gorai.
009G058900.1 Gorai.010G061200.1
Vv04g0593 . . . . . Gorai.
011G085000.1 Vv09g0708 . Gorai.006G101600.1 .
Gorai.009G127000.1 . . . . .
. . . .
Vv04g0594 . . . . . . .
. . . . . . . .
. . . . .
Vv04g0595 . . . . Gorai.009G059000.1
. . . . . . . .
Vv11g0623 Tc09_g012000 . . .
Gorai.009G059000.1 Gorai.010G061000.1
Vv04g0596 . . Gorai.001G034900.1 . .
. . . . . . . .
. . . . . . .
Vv04g0597 . . . . Gorai.009G059200.1
. Vv011rg0052 . . . . .
. . . . . . . .
Vv04g0598 . Gorai.013G235500.1 . .
Gorai.009G059300.1 . Vv011rg0048 . . .
. . . . . . . .
. .
Vv04g0599 . . . . . .
Vv011rg0040 . . . . . . .
. . . . . .
Vv04g0600 . Gorai.013G235900.1 . . .
Gorai.010G116100.1 Vv011rg0038 . . .
. . . . . . . .
. .
Vv04g0601 . . . . . .
Vv011rg0037 . . . . . . .
. . . . . .
Vv04g0602 . Gorai.013G214300.1 . .
Gorai.009G059400.1 . . . . . .
. . . . . . . .
.
Vv04g0603 Tc01_g017430 . Gorai.001G037800.1 .
Gorai.009G062400.1 . . . . .
. . . . . . . .
. .
Vv04g0604 . . . . . . .
. . . . . . . .
. . . . .
Vv04g0605 . . Gorai.001G037700.1 .

Gorai.009G062300.1	Gorai.010G118300.1	.	.	.
	.	.	.	.	.	.	.	.
	.	.	.
Vv04g0606	Tc01_g004660	.	.	.	.	.
	.	.	.	.	.	.	.	.
	.	.	.	.	.	.
Vv04g0607	Tc01_g007270	.	Gorai.001G037500.1	.
	.	.	.	.	.	.	.	.
	.	.	.	.	.	.	.	.
Vv04g0608	.	.	.	.	.	.	.
	.	.	.	.	.	.	.	.
	.	.	.	.	.
Vv04g0609	.	.	Gorai.001G037200.1	.
Gorai.009G061900.1	.	.	.	.	.	.
	.	.	Vv11g0635	.	.	.	.
	.	.
Vv04g0610	.	.	.	.	.	.	.
	.	.	.	.	.	.	.	.
	.	.	.	.	.
Vv04g0611	Tc01_g002260	.	Gorai.001G031700.1	.
	Gorai.009G061700.1	.	.	.	.	.
	.	.	.	Vv11g0637	.	.	.
	.	.	.
Vv04g0612	.	.	Gorai.001G037100.1	.
Gorai.009G061600.1	.	.	.	.	.	.
	.	.	.	.	.	.	.	.
	.
Vv04g0613	.	.	.	.	Gorai.009G061400.1
	.	.	.	.	.	.	.	.
	.	.	.	.	.	.	.
Vv04g0614	.	.	.	.	.	.	.
	.	.	.	.	.	.	.	.
	.	.	.	.	.
Vv04g0615	Tc01_g019550	.	.	.	.	.
	.	.	.	.	.	.	.	.
	.	.	.	.	.	.
Vv04g0616	.	.	.	.	.	.	.
	.	.	.	.	.	.	.	.
	.	.	.	.	.
Vv04g0617	Tc09_g027670	.	Gorai.001G031500.1	.
	Gorai.009G061200.1	.	.	.	.	.
	.	.	.	Vv11g0642	.	.
Gorai.001G031500.1	.	Gorai.009G061200.1	.
Vv04g0618	.	.	.	.	.	Gorai.
010G118200.1	.	.	.	.	.	.	.
	.	.	.	.	.	.	.
Vv04g0619	Tc09_g029010	.	Gorai.001G036900.1	.
	.	.	.	.	.	.	.	.
	.	.	.	.	.	.	.	.
Vv04g0620	.	.	.	.	.	.	.

. . . . . . . .
. . . . .
Vv04g0621 Tc09_g013420 . . . Gorai.
009G061100.1 Gorai.011G088000.1 . . . .
. . . . . . . .
. .
Vv04g0622 Tc01_g019720 . Gorai.001G030600.1 .
Gorai.009G061000.1 . . . . .
. . . . . . . .
. .
Vv04g0623 Tc01_g037970 . . . . .
. . . . . . . .
. . . . . .
Vv04g0624 . . . . . . .
. . . . . . . .
. . . . .
Vv04g0625 . . . . . . .
. . . . . . . .
. . . . .
Vv04g0626 . . . . Gorai.009G060800.1
. . . . . . . .
. . . . . . .
Vv04g0627 . . . . . . .
. . . . . . . .
. . . . .
Vv04g0628 Tc09_g013830 . . . .
Gorai.010G117900.1 . . . . . .
. . . . . . . .
Vv04g0629 Tc09_g028790 . Gorai.001G036600.1 .
Gorai.009G060700.1 Gorai.010G117700.1 . .
. . . . . . . .
. . . .
Vv04g0630 . . . . . .
Vv011rg0028 . . . . . . .
. . . . . .
Vv04g0631 . . . . . . .
. . . . . . . .
. . . . .
Vv04g0632 . . . . . . .
. . . . . . . .
. . . . .
Vv04g0633 Tc01_g004930 . Gorai.001G036500.1 .
Gorai.009G060600.1 Gorai.010G117500.1 . .
. . . . . . . .
. . . .
Vv04g0634 . . . . . . .
. . . . . . . .
. . . . .
Vv04g0635 . . . . . . .
. . . . . . . .

.       .       .       .       .
Vv04g0636       .       .       .       .       .       .       .
        .       .       .       .       .       .       .       .
        .       .       .       .       .
Vv04g0637       .       .       .       .       .       .       .
        .       .       .       .       .       .       .       .
        .       .       .       .       .
Vv04g0638       .       .       .       .       .       .       .
        .       .       .       .       .       .       .       .
        .       .       .       .       .
Vv04g0639       .       .       Gorai.001G028000.1      .
Gorai.009G060500.1      Gorai.010G117400.1      .       .       .
        .       .       .       .       .       .       .       .
        .       .       .
Vv04g0640       Tc01_g005240    .       .       .       .       .
        .       .       .       .       .       .       .       .
        .       .       .       .       .       .
Vv04g0641       .       .       Gorai.001G025000.1      .       .
        .       .       .       .       .       .       .       .
        .       .       .       .       .       .       .
Vv04g0642       .       .       Gorai.001G036000.1      .       .
        .       .       .       .       .       .       .       .
        .       .       .       .       .       .       .
Vv04g0643       .       .       Gorai.001G035900.1      .       .
        Gorai.010G117200.1      .       .       .       .       .
        .       .       .       .       .       .       .       .
        .
Vv04g0644       Tc01_g002530    .       .       .       .       .
        .       .       .       .       .       .       .       .
        .       .       .       .       .       .
Vv04g0645       .       Gorai.013G213900.1      Gorai.001G035700.1
        .       Gorai.009G060200.1      Gorai.010G117100.1      .
        .       .       .       .       .       .       .       .
        .       .       .       .       .
Vv04g0646       .       .       .       .       .       Gorai.
010G116900.1    .       .       .       .       .       .       .
        .       .       .       .       .       .       .
Vv04g0647       .       .       Gorai.001G035500.1      .       .
        .       .       .       .       .       .       .       .
        .       .       .       .       .       .       .
Vv04g0648       Tc01_g005320    .       Gorai.001G035300.1      .
        .       .       .       .       .       .       .       .
        .       .       .       .       .       .       .       .
Vv04g0649       .       .       .       .       .       .       .
        .       .       .       .       .       .       .       .
        .       .       .       .       .
Vv04g0650       .       .       .       .       .       .       .
        .       .       .       .       .       .       .       .
        .       .       .       .       .
Vv04g0651       .       .       .       .       .       .       .

. . . . . . . .
. . . . .
Vv04g0652 . Gorai.013G237400.1 . . .
. . . . . . . .
. . . . . . .
Vv04g0653 . Gorai.013G237300.1 . . .
. . . . . . . .
. . . . . . .
Vv04g0654 . . . . Gorai.009G060000.1
. . . . . . . .
. . . . . . .
Vv04g0655 . . . . . . .
. . . . . . . .
. . . . .
Vv04g0656 . . . . . . .
. . . . . . . .
. . . . .
Vv04g0657 . . . . . . .
. . . . . . . .
. . . . .
Vv04g0658 . . . . . . .
. . . . . . . .
. . . . .
Vv04g0659 . . . . . Gorai.
010G116800.1 . . . . . . .
. . . . . . .
Vv04g0660 . . . . . . .
. . . . . . . .
. . . . .
Vv04g0661 Tc01_g010390 . . . . .
. . . . . . . .
. . . . . .
Vv04g0662 . . . . . . .
. . . . . . . .
. . . . .
Vv04g0663 . . . . . . .
. . . . . . . .
. . . . .
Vv04g0664 . . . . . . .
. . . . . . . .
. . . . .
Vv04g0665 . Gorai.013G236600.1 . .
Gorai.009G059900.1 . Vv011rg0002 . . .
. . . . . . . .
. .
Vv04g0666 . . . . . . .
. . . . . . . .
. . . . .
Vv04g0667 . . . . Gorai.009G059800.1
Gorai.010G116600.1 . . . . .

. . . . . . . .
.
Vv04g0668 . . . . . . .
. . . . . . . .
. . . . .
Vv04g0669 . . . . Gorai.009G059700.1
Gorai.010G116500.1 . . . . .
. . . . . . . .
.
Vv04g0670 . . . . . . .
. . . . . . . .
. . . . .
Vv04g0671 . . . . . . .
. . . . . . . .
. . . . .
Vv04g0672 . . . . . . .
. . . . . . . .
. . . . .
Vv04g0673 . . . . . . .
. . . . . . . .
. . . . .
Vv04g0674 . . . . . . .
. . . . . . . .
. . . . .
Vv04g0675 . . . . . . .
. . . . . . . .
. . . . .
Vv04g0676 . . . . . .
Vv09g0702 . . . . . . .
. . . . . .
Vv04g0677 . . . . . . .
. . . . . . . .
. . . . .
Vv04g0678 . . . . . . .
. . . . . . . .
. . . . .
Vv04g0679 . . . . . . .
. . . . . . . .
. . . . .
Vv04g0680 . . . . . Gorai.
010G116400.1 . . . . . . .
. . . . . . .
Vv04g0681 . . Gorai.001G035100.1 . .
Gorai.010G116300.1 . . . . .
. . . . . . . .
.
Vv04g0682 . . Gorai.001G035000.1 . .
. . . . . . . .
. . . . . . .
Vv04g0683 . . . . Gorai.009G059500.1

. . . . . . . .
. . . . . . .
Vv04g0684 Tc01_g022640 Gorai.013G236000.1 . .
. Gorai.010G116200.1 . . . .
. . . . . . . .
. .
Vv04g0685 . Gorai.013G233000.1 . . .
. . . . . . . .
. . . . . . .
Vv04g0686 Tc01_g005630 . . . Gorai.
009G062500.1 . . . . . . .
. . . . . . . .
Vv04g0687 . . . . . . .
. . . . . . . .
. . . . .
Vv04g0688 . . . . . . .
. . . . . . . .
. . . . .
Vv04g0689 . . . . . . .
. . . . . . . .
. . . . .
Vv04g0690 . . . . Gorai.009G062700.1
Gorai.010G118700.1 . . . . .
. . . . . . . .
.
Vv04g0691 . . Gorai.001G037900.1 . .
. . . . . . . .
. . . . . . .
Vv04g0692 . . . . . . .
. . . . . . . .
. . . . .
Vv04g0693 Tc01_g010220 . Gorai.001G032200.1 .
. . . . . . . .
. . . . . . . .
Vv04g0694 . . Gorai.001G029300.1 .
Gorai.009G062800.1 . Vv09g0699 . . .
. . . . . . . .
. .
Vv04g0695 . . Gorai.001G038100.1 . .
Gorai.010G118800.1 . . . . .
. . . . . . . .
.
Vv04g0696 Tc01_g022390 . Gorai.001G038200.1 .
Gorai.009G062900.1 Gorai.010G118900.1 . .
. . . . . . . .
. . . .
Vv04g0697 . . . . Gorai.009G056900.1
Gorai.010G113300.1 . . . . .
. . . . . . . .
.

Vv04g0698 . . . . Gorai.009G063100.1
. . . . . . . .
. . . . . . .
Vv04g0699 . . Gorai.001G038300.1 .
Gorai.009G056400.1 . Vv09g0670 Tc06_g001740 .
. . . . . . . .
. . .
Vv04g0700 . . . . . . .
. . . . . . . .
. . . . .
Vv04g0701 . . . . Gorai.009G063300.1
. . . . . . . .
. . . . . . .
Vv04g0702 . . . . . . .
. . . . . . . .
. . . . .
Vv04g0703 . . . . . . .
. . . . . . . .
. . . . .
Vv04g0704 . . . . . . .
. . . . . . . .
. . . . .
Vv04g0705 Tc01_g005950 . . . . .
. . . . . . . .
. . . . . .
Vv04g0706 . . . . . . .
. . . . . . . .
. . . . .
Vv04g0707 . . Gorai.001G038600.1 . .
. . . . . . . .
. . . . . . .
Vv04g0708 . . . . Gorai.009G063700.1
. . . . . . . .
. . . . . . .
Vv04g0709 . . . . . . .
. . . . . . . .
. . . . .
Vv04g0710 . . . . . Gorai.
010G110300.1 . . . . . . .
. . . . . . .
Vv04g0711 Tc01_g001400 . . . . .
. . . . . . . .
. . . . . .
Vv04g0712 . . . . Gorai.009G063800.1
. . . . . . . .
. . . . . . .
Vv04g0713 . . . . Gorai.009G063900.1
. . . . . . . .
. . . . . . .
Vv04g0714 . . Gorai.001G038700.1 .

Gorai.009G055700.1 . . . . . .
. . . . . . . .
.
Vv04g0715 . . . . . . .
. . . . . . . .
. . . . .
Vv04g0716 . . . . . . .
. . . . . . . .
. . . . .
Vv04g0717 . . . . . . .
. . . . . . . .
. . . . .
Vv04g0718 Tc01_g022330 . . . . .
. . . . . . . .
. . . . . .
Vv04g0719 . . . . . . .
. . . . . . . .
. . . . .
Vv04g0720 . . . . . . .
. . . . . . . .
. . . . .
Vv04g0721 . . . . . Gorai.
010G119600.1 . . . . . . .
. . . . . . .
Vv04g0722 . . . . Gorai.009G064300.1
. . . . . . . .
. . . . . . .
Vv04g0723 . . Gorai.001G038900.1 . .
. . . . . . . .
. . . . . . .
Vv04g0724 . . . . . . .
. . . . . . . .
. . . . .
Vv04g0725 . . . . . . .
. . . . . . . .
. . . . .
Vv04g0726 . . . . . . .
. . . . . . . .
. . . . .
Vv04g0727 . . . . . . .
. . . . . . . .
. . . . .
Vv04g0728 . . . . . . .
. . . . . . . .
. . . . .
Vv04g0729 . . . . . . .
. . . . . . . .
. . . . .
Vv04g0730 Tc01_g001190 . . . . .
. . . . . . . .

. . . . . .
Vv04g0731 Tc01_g010100 . . . . .
. . . . . . . .
. . . . . .
Vv04g0732 . . . . . . .
. . . . . . . .
. . . . .
Vv04g0733 . . . . . . .
. . . . . . . .
. . . . .
Vv04g0734 . . . . . . .
. . . . . . . .
. . . . .
Vv04g0735 Tc01_g001170 . . . Gorai.
009G065000.1 . . . . . . .
. . . . . . . .
Vv04g0736 . . . . . . .
. . . . . . . .
. . . . .
Vv04g0737 . . . . . . .
. . . . . . . .
. . . . .
Vv04g0738 . . . . . . .
. . . . . . . .
. . . . .
Vv04g0739 . . . . . . .
. . . . . . . .
. . . . .
Vv04g0740 Tc01_g001160 . . . . .
. . . . . . . .
. . . . . .
Vv04g0741 Tc01_g006030 . . . . .
. . . . . . . .
. . . . . .
Vv04g0742 . . . . . . .
. . . . . . . .
. . . . .
Vv04g0743 . . . . Gorai.009G065100.1
Gorai.010G120300.1 . . . . .
. . . . . . . .
.
Vv04g0744 . . . . . . .
. . . . . . . .
. . . . .
Vv04g0745 . . Gorai.001G039400.1 .
Gorai.009G053100.1 . . . . . .
. . . . . . . .
.
Vv04g0746 . . . . . . .
. . . . . . . .

. . . . .
Vv04g0747 . . . . . . .
. . . . . . . .
. . . . .
Vv04g0748 . . . . . . .
. . . . . . . .
. . . . .
Vv04g0749 . . . . . . .
. . . . . . . .
. . . . .
Vv04g0750 . . . . Gorai.009G050200.1
Gorai.010G120400.1 . . . . .
. . . . . . . .
.
Vv04g0751 Tc01_g006140 . . . . .
. . . . . . . .
. . . . . .
Vv04g0752 . . . . . . .
. . . . . . . .
. . . . .
Vv04g0753 . . Gorai.001G039500.1 .
Gorai.009G065600.1 . . . . . .
. . . . . . . .
.
Vv04g0754 . . . . . . .
. . . . . . . .
. . . . .
Vv04g0755 . . . . Gorai.009G065700.1
. . . . . . . .
. . . . . . .
Vv04g0756 . . . . . . .
. . . . . . . .
. . . . .
Vv04g0757 . . . . . . .
. . . . . . . .
. . . . .
Vv04g0758 . . . . . . .
. . . . . . . .
. . . . .
Vv04g0759 . . . . Gorai.009G066100.1
. . . . . . . .
. . . . . . .
Vv04g0760 . . . . . . .
. . . . . . . .
. . . . .
Vv04g0761 . . . . . . .
. . . . . . . .
. . . . .
Vv04g0762 . . . . . . .
. . . . . . . .

| | | | | | | | |
|---|---|---|---|---|---|---|---|
| Vv04g0763 | . | . | . | . | . | . | . |
| Vv04g0764 | . | . | . | . | . | . | . |
| Vv04g0765 | . | . | . | . | . | . | . |
| Vv04g0766 | . | . | . | . | . | . | . |
| Vv04g0767 | . | . | . | . | . | . | . |
| Vv04g0768 | . | . | . | . | . | . | . |
| Vv04g0769 | . | . | . | . | . | . | . |
| Vv04g0770 | . | . | . | . | . | . | . |
| Vv04g0771 | . | . | . | . | . | . | . |
| Vv04g0772 | . | . | . | . | . | . | . |
| Vv04g0773 | . | . | . | . | . | . | . |
| Vv04g0774 | . | . | . | . | . | . | . |
| Vv04g0775 | . | . | . | . | . | . | . |
| Vv04g0776 | . | . | . | . | . | . | . |
| Vv04g0777 | . | . | . | . | . | . | . |
| Vv04g0778 | . | . | . | . | . | . | . |
| Vv04g0779 | . | . | . | . | . | . | . |

| | | | | | | | |
|---|---|---|---|---|---|---|---|
| . | . | . | . | . | . | . | . |
| . | . | . | . | . | | | |
| Vv04g0780 | . | . | . | . | . | . | . |
| . | . | . | . | . | . | . | . |
| . | . | . | . | . | | | |
| Vv04g0781 | . | . | . | . | . | . | . |
| . | . | . | . | . | . | . | . |
| . | . | . | . | . | | | |
| Vv04g0782 | . | . | . | . | . | . | . |
| . | . | . | . | . | . | . | . |
| . | . | . | . | . | | | |
| Vv04g0783 | . | . | . | . | . | . | . |
| . | . | . | . | . | . | . | . |
| . | . | . | . | . | | | |
| Vv04g0784 | . | . | . | . | . | . | . |
| . | . | . | . | . | . | . | . |
| . | . | . | . | . | | | |
| Vv04g0785 | . | . | . | . | . | . | . |
| . | . | . | . | . | . | . | . |
| . | . | . | . | . | | | |
| Vv04g0786 | . | . | . | . | . | . | . |
| . | . | . | . | . | . | . | . |
| . | . | . | . | . | | | |
| Vv04g0787 | . | . | . | . | . | . | . |
| . | . | . | . | . | . | . | . |
| . | . | . | . | . | | | |
| Vv04g0788 | . | . | . | . | . | . | . |
| . | . | . | . | . | . | . | . |
| . | . | . | . | . | | | |
| Vv04g0789 | . | . | . | . | . | . | . |
| . | . | . | . | . | . | . | . |
| . | . | . | . | . | | | |
| Vv04g0790 | . | . | . | . | . | . | . |
| . | . | . | . | . | . | . | . |
| . | . | . | . | . | | | |
| Vv04g0791 | . | . | . | . | . | . | . |
| . | . | . | . | . | . | . | . |
| . | . | . | . | . | | | |
| Vv04g0792 | . | . | . | . | . | . | . |
| . | . | . | . | . | . | . | . |
| . | . | . | . | . | | | |
| Vv04g0793 | . | . | . | . | . | . | . |
| . | . | . | . | . | . | . | . |
| . | . | . | . | . | | | |
| Vv04g0794 | . | . | . | . | . | . | . |
| . | . | . | . | . | . | . | . |
| . | . | . | . | . | | | |
| Vv04g0795 | Tc01_g031820 | | . | . | . | . | . |
| . | . | . | . | . | . | . | . |
| . | . | . | . | . | . | | |

| | | | | | | | |
|---|---|---|---|---|---|---|---|
| Vv04g0796 | . | . | . | . | . | . | . |
| . | . | . | . | . | . | . | . |
| . | . | . | . | . | | | |
| Vv04g0797 | . | . | . | . | . | . | . |
| . | . | . | . | . | . | . | . |
| . | . | . | . | . | | | |
| Vv04g0798 | . | . | . | . | . | . | . |
| . | . | . | . | . | . | . | . |
| . | . | . | . | . | | | |
| Vv04g0799 | . | . | . | . | . | . | . |
| . | . | . | . | . | . | . | . |
| . | . | . | . | . | | | |
| Vv04g0800 | Tc01_g014950 | | . | . | . | . | . |
| . | . | . | . | . | . | . | . |
| . | . | . | . | . | . | | |
| Vv04g0801 | . | . | . | . | . | . | . |
| . | . | . | . | . | . | . | . |
| . | . | . | . | . | | | |
| Vv04g0802 | . | . | . | . | . | . | . |
| . | . | . | . | . | . | . | . |
| . | . | . | . | . | | | |
| Vv04g0803 | . | . | . | . | . | . | . |
| . | . | . | . | . | . | . | . |
| . | . | . | . | . | | | |
| Vv04g0804 | . | . | . | . | . | . | . |
| . | . | . | . | . | . | . | . |
| . | . | . | . | . | | | |
| Vv04g0805 | . | . | . | . | . | . | . |
| . | . | . | . | . | . | . | . |
| . | . | . | . | . | | | |
| Vv04g0806 | . | . | . | . | . | . | . |
| . | . | . | . | . | . | . | . |
| . | . | . | . | . | | | |
| Vv04g0807 | . | . | . | . | . | . | . |
| . | . | . | . | . | . | . | . |
| . | . | . | . | . | | | |
| Vv04g0808 | . | . | . | . | . | . | . |
| . | . | . | . | . | . | . | . |
| . | . | . | . | . | | | |
| Vv04g0809 | . | . | . | . | . | . | . |
| . | . | . | . | . | . | . | . |
| . | . | . | . | . | | | |
| Vv04g0810 | . | . | . | . | . | . | . |
| . | . | . | . | . | . | . | . |
| . | . | . | . | . | | | |
| Vv04g0811 | . | . | . | . | . | . | . |
| . | . | . | . | . | . | . | . |
| . | . | . | . | . | | | |
| Vv04g0812 | . | . | . | . | . | . | . |
| . | . | . | . | . | . | . | . |

| | | | | | | | |
|---|---|---|---|---|---|---|---|
| . | . | . | . | . | | | |
| Vv04g0813 | . | . | . | . | . | . | . |
| . | . | . | . | . | . | . | . |
| . | . | . | . | . | | | |
| Vv04g0814 | . | . | . | . | . | . | . |
| . | . | . | . | . | . | . | . |
| . | . | . | . | . | | | |
| Vv04g0815 | . | . | . | . | . | . | . |
| . | . | . | . | . | . | . | . |
| . | . | . | . | . | | | |
| Vv04g0816 | . | . | . | . | . | . | . |
| . | . | . | . | . | . | . | . |
| . | . | . | . | . | | | |
| Vv04g0817 | . | . | . | . | . | . | . |
| . | . | . | . | . | . | . | . |
| . | . | . | . | . | | | |
| Vv04g0818 | . | . | . | . | . | . | . |
| . | . | . | . | . | . | . | . |
| . | . | . | . | . | | | |
| Vv04g0819 | Tc01_g015070 | | . | . | . | . | . |
| . | . | . | . | . | . | . | . |
| . | . | . | . | . | . | | |
| Vv04g0820 | . | . | . | . | . | . | . |
| . | . | . | . | . | . | . | . |
| . | . | . | . | . | | | |
| Vv04g0821 | Tc01_g032090 | | . | . | . | . | . |
| . | . | . | . | . | . | . | . |
| . | . | . | . | . | . | | |
| Vv04g0822 | . | . | . | . | . | . | . |
| . | . | . | . | . | . | . | . |
| . | . | . | . | . | | | |
| Vv04g0823 | . | . | . | . | . | . | . |
| . | . | . | . | . | . | . | . |
| . | . | . | . | . | | | |
| Vv04g0824 | . | . | . | . | . | . | . |
| . | . | . | . | . | . | . | . |
| . | . | . | . | . | | | |
| Vv04g0825 | . | . | . | . | . | . | . |
| . | . | . | . | . | . | . | . |
| . | . | . | . | . | | | |
| Vv04g0826 | . | . | . | . | . | . | . |
| . | . | . | . | . | . | . | . |
| . | . | . | . | . | | | |
| Vv04g0827 | . | . | . | . | . | . | . |
| . | . | . | . | . | . | . | . |
| . | . | . | . | . | | | |
| Vv04g0828 | . | . | . | . | . | . | . |
| . | . | . | . | . | . | . | . |
| . | . | . | . | . | | | |
| Vv04g0829 | . | Gorai.013G188500.1 | | | . | . | . |

. . . . . . . .
. . . . . . .
Vv04g0830 . Gorai.013G188600.1 . . .
. . . . . . . .
. . . . . . .
Vv04g0831 . . . . . . .
. . . . . . . .
. . . . .
Vv04g0832 . . . . . . .
. . . . . . . .
. . . . .
Vv04g0833 . . . . . . .
. . . . . . . .
. . . . .
Vv04g0834 . . . . . . .
. . . . . . . .
. . . . .
Vv04g0835 . . . . . . .
. . . . . . . .
. . . . .
Vv04g0836 . . . . . . .
. . . . . . . .
. . . . .
Vv04g0837 . Gorai.013G188800.1 . Gorai.
004G288100.1 . . . . . . .
. . . . . . . .
.
Vv04g0838 . . . . . . .
. . . . . . . .
. . . . .
Vv04g0839 . . . . . . .
. . . . . . . .
. . . . .
Vv04g0840 . . . . . . .
. . . . . . . .
. . . . .
Vv04g0841 Tc01_g004930 . . . Gorai.
003G023200.1 . . . . . . .
. . . . . . . .
Vv04g0842 Tc01_g021650 . . . . .
. . . . . . . .
. . . . . .
Vv04g0843 . . . . . . .
. . . . . . . .
. . . . .
Vv04g0844 . . . . . . .
. . . . . . . .
. . . . .
Vv04g0845 . . . Gorai.004G288000.1 .
. . . . . . . .

| | | | | | | | |
|---|---|---|---|---|---|---|---|
| . | . | . | . | . | . | . | |
| Vv04g0846 | . | . | . | . | . | . | . |
| . | . | . | . | . | . | . | . |
| . | . | . | . | . | | | |
| Vv04g0847 | . | . | . | Gorai.004G287900.1 | | | . |
| . | . | . | . | . | . | . | . |
| . | . | . | . | . | . | . | |
| Vv04g0848 | Tc01_g010970 | | . | . | Gorai.004G287700.1 | | |
| . | . | . | . | . | . | . | . |
| . | . | . | . | . | . | . | . |
| Vv04g0849 | . | . | . | . | . | . | . |
| . | . | . | . | . | . | . | . |
| . | . | . | . | . | | | |
| Vv04g0850 | . | . | . | . | . | . | . |
| . | . | . | . | . | . | . | . |
| . | . | . | . | . | | | |
| Vv04g0851 | Tc01_g032420 | | . | . | . | . | . |
| . | . | . | . | . | . | . | . |
| . | . | . | . | . | . | | |
| Vv04g0852 | Tc01_g032430 | | . | . | . | . | . |
| . | . | . | . | . | . | . | . |
| . | . | . | . | . | . | | |
| Vv04g0853 | . | . | . | . | . | . | . |
| . | . | . | . | . | . | . | . |
| . | . | . | . | . | | | |
| Vv04g0854 | . | . | . | . | . | . | . |
| . | . | . | . | . | . | . | . |
| . | . | . | . | . | | | |
| Vv04g0855 | . | . | . | . | . | . | . |
| . | . | . | . | . | . | . | . |
| . | . | . | . | . | | | |
| Vv04g0856 | . | . | . | . | . | . | . |
| . | . | . | . | . | . | . | . |
| . | . | . | . | . | | | |
| Vv04g0857 | . | . | . | . | . | . | . |
| . | . | . | . | . | . | . | . |
| . | . | . | . | . | | | |
| Vv04g0858 | . | . | . | . | . | . | . |
| . | . | . | . | . | . | . | . |
| . | . | . | . | . | | | |
| Vv04g0859 | . | . | . | . | . | . | . |
| . | . | . | . | . | . | . | . |
| . | . | . | . | . | | | |
| Vv04g0860 | . | . | . | . | . | . | . |
| . | . | . | . | . | . | . | . |
| . | . | . | . | . | | | |
| Vv04g0861 | . | . | . | Gorai.004G285200.1 | | | . |
| . | . | . | . | . | . | . | . |
| . | . | . | . | . | . | . | |
| Vv04g0862 | Tc01_g021130 | | . | . | . | . | . |

| | | | | | | | |
|---|---|---|---|---|---|---|---|
| . | . | . | . | . | . | . | . |
| . | . | . | . | . | . | | |
| Vv04g0863 | . | . | . | . | . | . | . |
| . | . | . | . | . | . | . | . |
| . | . | . | . | . | | | |
| Vv04g0864 | . | . | . | . | . | . | . |
| . | . | . | . | . | . | . | . |
| . | . | . | . | . | | | |
| Vv04g0865 | . | . | . | . | . | . | . |
| . | . | . | . | . | . | . | . |
| . | . | . | . | . | | | |
| Vv04g0866 | . | . | . | . | . | . | . |
| . | . | . | . | . | . | . | . |
| . | . | . | . | . | | | |
| Vv04g0867 | . | . | . | . | . | . | . |
| . | . | . | . | . | . | . | . |
| . | . | . | . | . | | | |
| Vv04g0868 | . | . | . | . | . | . | . |
| . | . | . | . | . | . | . | . |
| . | . | . | . | . | | | |
| Vv04g0869 | . | . | . | . | . | . | . |
| . | . | . | . | . | . | . | . |
| . | . | . | . | . | | | |
| Vv04g0870 | . | . | . | . | . | . | . |
| . | . | . | . | . | . | . | . |
| . | . | . | . | . | | | |
| Vv04g0871 | . | Gorai.013G189100.1 | | | . | . | . |
| . | . | . | . | . | . | . | . |
| . | . | . | . | . | . | . | |
| Vv04g0872 | . | . | . | . | . | . | . |
| . | . | . | . | . | . | . | . |
| . | . | . | . | . | | | |
| Vv04g0873 | . | . | . | . | . | . | . |
| . | . | . | . | . | . | . | . |
| . | . | . | . | . | | | |
| Vv04g0874 | . | . | . | . | . | . | . |
| . | . | . | . | . | . | . | . |
| . | . | . | . | . | | | |
| Vv04g0875 | . | . | . | . | . | . | . |
| . | . | . | . | . | . | . | . |
| . | . | . | . | . | | | |
| Vv04g0876 | . | . | . | . | . | . | . |
| . | . | . | . | . | . | . | . |
| . | . | . | . | . | | | |
| Vv04g0877 | Tc01_g004990 | | . | . | . | . | . |
| . | . | . | . | . | . | . | . |
| . | . | . | . | . | . | | |
| Vv04g0878 | . | . | . | . | . | . | . |
| . | . | . | . | . | . | . | . |
| . | . | . | . | . | | | |

Vv04g0879 . . . . . . .
. . . . . . . .
. . . . .
Vv04g0880 . . . . . . .
. . . . . . . .
. . . . .
Vv04g0881 Tc01_g005560 . . . . .
. . . . . . . .
. . . . . .
Vv04g0882 . Gorai.013G191400.1 . . .
. . . . . . . .
. . . . . . .
Vv04g0883 . . . . . . .
. . . . . . . .
. . . . .
Vv04g0884 . . . . . . .
. . . . . . . .
. . . . .
Vv04g0885 . . . . . . .
. . . . . . . .
. . . . .
Vv04g0886 . . . . . . .
. . . . . . . .
. . . . .
Vv04g0887 Tc01_g011170 . . . . .
. . . . . . . .
. . . . . .
Vv04g0888 . . . . . . .
. . . . . . . .
. . . . .
Vv04g0889 . . . . . . .
. . . . . . . .
. . . . .
Vv04g0890 . . . . . . .
. . . . . . . .
. . . . .
Vv04g0891 . . . . . . .
. . . . . . . .
. . . . .
Vv04g0892 . . . . . . .
. . . . . . . .
. . . . .
Vv04g0893 . . . . . . .
. . . . . . . .
. . . . .
Vv04g0894 . . . . . . .
. . . . . . . .
. . . . .
Vv04g0895 . . . . . . .
. . . . . . . .

. . . . .
Vv04g0896 . . . . . . .
. . . . . . . .
. . . . .
Vv04g0897 . . . . . . .
. . . . . . . .
. . . . .
Vv04g0898 . . . . . . .
. . . . . . . .
. . . . .
Vv04g0899 . . . . . . .
. . . . . . . .
. . . . .
Vv04g0900 . . . . . . .
. . . . . . . .
. . . . .
Vv04g0901 . . . . . . .
. . . . . . . .
. . . . .
Vv04g0902 Tc01_g022870 . . . Gorai.
004G058300.1 . . . . . . .
. . . . . . . .
Vv04g0903 . . . . . . .
. . . . . . . .
. . . . .
Vv04g0904 . . . . . . .
. . . . . . . .
. . . . .
Vv04g0905 . . . . . . .
. . . . . . . .
. . . . .
Vv04g0906 Tc01_g005910 . . . . .
. . . . . . . .
. . . . . .
Vv04g0907 . . . . . . .
. . . . . . . .
. . . . .
Vv04g0908 Tc01_g005930 . . . . .
. . . . . . . .
. . . . . .
Vv04g0909 Tc01_g011280 . . . . .
. . . . . . . .
. . . . . .
Vv04g0910 Tc01_g011310 . . . . .
. . . . . . . .
. . . . . .
Vv04g0911 . . . . . . .
. . . . . . . .
. . . . .
Vv04g0912 . . . . . . .

. . . . . . . .
. . . . .
Vv04g0913 . . . . . . .
. . . . . . . .
. . . . .
Vv04g0914 . . . . . . .
. . . . . . . .
. . . . .
Vv04g0915 . Gorai.013G180500.1 . . .
. . . . . . . .
. . . . . . .
Vv04g0916 . . . . . . .
. . . . . . . .
. . . . .
Vv04g0917 . . . . . . .
. . . . . . . .
. . . . .
Vv04g0918 . . . . . . .
. . . . . . . .
. . . . .
Vv04g0919 . . . . . . .
. . . . . . . .
. . . . .
Vv04g0920 . . . . . . .
. . . . . . . .
. . . . .
Vv04g0921 . . . . . . .
. . . . . . . .
. . . . .
Vv04g0922 . . . . . . .
. . . . . . . .
. . . . .
Vv04g0923 . . . Gorai.004G260400.1 .
. . . . . . . .
. . . . . . .
Vv04g0924 . . . . . . .
. . . . . . . .
. . . . .
Vv04g0925 . . . . . . .
. . . . . . . .
. . . . .
Vv04g0926 . . . . . . .
. . . . . . . .
. . . . .
Vv04g0927 . . . . . . .
. . . . . . . .
. . . . .
Vv04g0928 . . . . . . .
. . . . . . . .
. . . . .

Vv04g0929 . . . . . . .
. . . . . . . .
. . . . .
Vv04g0930 . . . . . . .
. . . . . . . .
. . . . .
Vv04g0931 . . . Gorai.004G260500.1 .
. . . . . . . .
. . . . . . .
Vv04g0932 Tc01_g010150 . . Gorai.004G261400.1
. . . . . . . .
. . . . . . . .
Vv04g0933 Tc01_g022970 . . . . .
. . . . . . . .
. . . . . .
Vv04g0934 . Gorai.013G180000.1 . . .
. . . . . . . .
. . . . . . .
Vv04g0935 . . . . . . .
. . . . . . . .
. . . . .
Vv04g0936 . . . . . . .
. . . . . . . .
. . . . .
Vv04g0937 . . . . . . .
. . . . . . . .
. . . . .
Vv04g0938 . . . . . . .
. . . . . . . .
. . . . .
Vv04g0939 . . . . . . .
. . . . . . . .
. . . . .
Vv04g0940 . Gorai.013G179900.1 . . .
. . . . . . . .
. . . . . . .
Vv04g0941 . . . . . . .
. . . . . . . .
. . . . .
Vv04g0942 Tc01_g017770 Gorai.013G179800.1 .
Gorai.004G261700.1 . . . . . .
. . . . . . . .
. .
Vv04g0943 . . . . . . .
. . . . . . . .
. . . . .
Vv04g0944 Tc01_g005950 . . . . .
. . . . . . . .
. . . . . .
Vv04g0945 . . . . . . .

| | | | | | | | |
|---|---|---|---|---|---|---|---|
| Vv04g0946 | . | . | . | . | . | . | . |
| Vv04g0947 | . | . | . | . | . | . | . |
| Vv04g0948 | Tc01_g017720 | | Gorai.013G179700.1 | | | . | . |
| Vv04g0949 | . | . | . | . | . | . | . |
| Vv04g0950 | . | . | . | . | . | . | . |
| Vv04g0951 | Tc01_g010220 | | Gorai.013G179400.1 | | | . | . |
| Vv04g0952 | . | . | . | . | . | . | . |
| Vv04g0953 | . | . | . | . | . | . | . |
| Vv04g0954 | . | . | . | . | . | . | . |
| Vv04g0955 | . | . | . | . | . | . | . |
| Vv04g0956 | . | . | . | . | . | . | . |
| Vv04g0957 | . | . | . | . | . | . | . |
| Vv04g0958 | . | . | . | . | . | . | . |
| Vv04g0959 | . | . | . | . | . | . | . |
| Vv04g0960 | . | . | . | . | . | . | . |
| Vv04g0961 | . | . | . | . | . | . | . |

Vv04g0962 . . . . . . .
. . . . . . . .
. . . . .
Vv04g0963 . . . . . . .
. . . . . . . .
. . . . .
Vv04g0964 . . . . . . .
. . . . . . . .
. . . . .
Vv04g0965 . . . . . . .
. . . . . . . .
. . . . .
Vv04g0966 . . . . . . .
. . . . . . . .
. . . . .
Vv04g0967 . . . . . . .
. . . . . . . .
. . . . .
Vv04g0968 . . . . . . .
. . . . . . . .
. . . . .
Vv04g0969 . . . . . . .
. . . . . . . .
. . . . .
Vv04g0970 . . . . . . .
. . . . . . . .
. . . . .
Vv04g0971 . . . . . . .
. . . . . . . .
. . . . .
Vv04g0972 . . . Gorai.004G262800.1 .
. . . . . . . .
. . . . . . .
Vv04g0973 . . . . . . .
. . . . . . . .
. . . . .
Vv04g0974 Tc01_g010440 . . . . .
. . . . . . . .
. . . . . .
Vv04g0975 . . . . . . .
. . . . . . . .
. . . . .
Vv04g0976 . . . . . . .
. . . . . . . .
. . . . .
Vv04g0977 . . . . . . .
. . . . . . . .
. . . . .
Vv04g0978 . . . Gorai.004G262900.1 .
. . . . . . . .

. . . . . . .
Vv04g0979 . . . Gorai.004G263000.1 .
. . . . . . . .
. . . . . . .
Vv04g0980 Tc09_g034120 Gorai.013G177100.1 .
Gorai.004G263300.1 . . . . . .
. . . . . . . .
. .
Vv04g0981 . . . . . . .
. . . . . . . .
. . . . .
Vv04g0982 Tc01_g040140 . . . . .
. . . . . . . .
. . . . . .
Vv04g0983 . . . . . . .
. . . . . . . .
. . . . .
Vv04g0984 . . . . . . .
. . . . . . . .
. . . . .
Vv04g0985 . . . . . . .
. . . . . . . .
. . . . .
Vv04g0986 Tc09_g034000 . . . . .
. . . . . . . .
. . . . . .
Vv04g0987 . . . . . . .
. . . . . . . .
. . . . .
Vv04g0988 . . . . . . .
. . . . . . . .
. . . . .
Vv04g0989 . . . . . . .
. . . . . . . .
. . . . .
Vv04g0990 . . . . . . .
. . . . . . . .
. . . . .
Vv04g0991 . . . Gorai.004G263500.1 .
. . . . . . . .
. . . . . . .
Vv04g0992 . . . Gorai.004G263600.1 .
. . . . . . . .
. . . . . . .
Vv04g0993 . . . . . . .
. . . . . . . .
. . . . .
Vv04g0994 . . . Gorai.004G263700.1 .
. . . . . . . .
. . . . . . .

| | | | | | | | |
|---|---|---|---|---|---|---|---|
| Vv04g0995 | . | . | . | . | . | . | . |
| . | . | . | . | . | . | . | . |
| . | . | . | . | . | | | |
| Vv04g0996 | . | . | . | . | . | . | . |
| . | . | . | . | . | . | . | . |
| . | . | . | . | . | | | |
| Vv04g0997 | . | . | . | . | . | . | . |
| . | . | . | . | . | . | . | . |
| . | . | . | . | . | | | |
| Vv04g0998 | . | . | . | . | . | . | . |
| . | . | . | . | . | . | . | . |
| . | . | . | . | . | | | |
| Vv04g0999 | . | . | . | . | . | . | . |
| . | . | . | . | . | . | . | . |
| . | . | . | . | . | | | |
| Vv04g1000 | . | . | . | . | . | . | . |
| . | . | . | . | . | . | . | . |
| . | . | . | . | . | | | |
| Vv04g1001 | . | . | . | . | . | . | . |
| . | . | . | . | . | . | . | . |
| . | . | . | . | . | | | |
| Vv04g1002 | . | . | . | . | . | . | . |
| . | . | . | . | . | . | . | . |
| . | . | . | . | . | | | |
| Vv04g1003 | . | . | . | . | . | . | . |
| . | . | . | . | . | . | . | . |
| . | . | . | . | . | | | |
| Vv04g1004 | . | . | . | . | . | . | . |
| . | . | . | . | . | . | . | . |
| . | . | . | . | . | | | |
| Vv04g1005 | Tc01_g039970 | | . | . | . | . | . |
| . | . | . | . | . | . | . | . |
| . | . | . | . | . | . | | |
| Vv04g1006 | . | . | . | . | . | . | . |
| . | . | . | . | . | . | . | . |
| . | . | . | . | . | | | |
| Vv04g1007 | . | . | . | . | . | . | . |
| . | . | . | . | . | . | . | . |
| . | . | . | . | . | | | |
| Vv04g1008 | . | . | . | . | . | . | . |
| . | . | . | . | . | . | . | . |
| . | . | . | . | . | | | |
| Vv04g1009 | . | . | . | . | . | . | . |
| . | . | . | . | . | . | . | . |
| . | . | . | . | . | | | |
| Vv04g1010 | . | . | . | . | . | . | . |
| . | . | . | . | . | . | . | . |
| . | . | . | . | . | | | |
| Vv04g1011 | . | . | . | . | . | . | . |
| . | . | . | . | . | . | . | . |

. . . . .
Vv04g1012 . . . . . . .
. . . . . . . .
. . . . .
Vv04g1013 . . . . . . .
. . . . . . . .
. . . . .
Vv04g1014 . . . Gorai.004G263900.1 .
. . . . . . . .
. . . . . . .
Vv04g1015 . . . . . . .
. . . . . . . .
. . . . .
Vv04g1016 . . . . . . .
. . . . . . . .
. . . . .
Vv04g1017 . . . . . . .
. . . . . . . .
. . . . .
Vv04g1018 Tc01_g003910 . . . Gorai.
003G018400.1 . . . . . . .
. . . . . . . .
Vv04g1019 Tc01_g003900 . . . Gorai.
003G018300.1 . . . . . . .
. . . . . . . .
Vv04g1020 Tc01_g003890 . . . Gorai.
003G018200.1 . . . . . . .
. . . . . . . .
Vv04g1021 Tc01_g003880 . Gorai.008G070800.1
Gorai.007G207200.1 Gorai.003G018100.1 . Vv18g1003
. . . . . . . .
. . . . .
Vv04g1022 Tc01_g003870 . . . . .
. . . . . . . .
. . . . . .
Vv04g1023 . . Gorai.008G070600.1 . .
. Vv18g1005 Tc08_g001590 Gorai.002G129900.1
. Gorai.009G205600.1 . . . .
. . . . .
Vv04g1024 Tc01_g003860 Gorai.007G051800.1 Gorai.
008G070500.1 . . . . . . .
. . . . . . . .
. .
Vv04g1025 . . . . . . .
. . . . . . . .
. . . . .
Vv04g1026 Tc01_g003850 Gorai.007G051700.1 Gorai.
008G070400.1 . . . . . . .
. . . . . . . .
. .

Vv04g1027 . . . . . . .
. . . . . . . .
. . . . .
Vv04g1028 Tc01_g003840 Gorai.007G051600.1 . .
. . . . . . . .
. . . . . . . .
Vv04g1029 . . . . . . .
. . . . . . . .
. . . . .
Vv04g1030 . . . . . . .
. . . . . . . .
. . . . .
Vv04g1031 . . . . . . .
. . . . . . . .
. . . . .
Vv04g1032 Tc01_g003830 . Gorai.008G070200.1
Gorai.007G207100.1 . . . . . .
. . . . . . . .
. .
Vv04g1033 . . . . . . .
. . . . . . . .
. . . . .
Vv04g1034 Tc01_g003820 . Gorai.008G070100.1 .
Gorai.003G018000.1 . . . . .
. . . . . . . .
. .
Vv04g1035 Tc01_g003810 . Gorai.008G070000.1
Gorai.007G207000.1 Gorai.003G017900.1 . Vv18g1008
Tc08_g001630 Gorai.002G129800.1 Gorai.005G106100.1
Gorai.009G205300.1 Gorai.009G274300.1 Gorai.
010G012700.1 . . . . . . .
Vv04g1036 Tc01_g003800 . Gorai.008G069900.1 .
Gorai.003G017800.1 . . . . .
. . . . . . . .
. .
Vv04g1037 Tc01_g003790 . . Gorai.007G206900.1
Gorai.003G017700.1 . Vv18g1014
Tc08_g001650 . Gorai.005G106300.1 . .
Gorai.010G012400.1 . . . . . .
.
Vv04g1038 Tc01_g003770 . . Gorai.007G206800.1
Gorai.003G017600.1 Gorai.011G103100.1 Vv18g1019
Tc08_g001690 . . Gorai.009G205000.1 .
. . . . . . . .
Vv04g1039 Tc01_g003760 Gorai.007G051500.1 . .
. . . . . . . .
. . . . . . . .
Vv04g1040 . . . . . .
Vv18g1020 Tc08_g001700 . Gorai.005G106500.1
Gorai.009G204900.1 . Gorai.010G012100.1 . .

. . . . .
Vv04g1041 Tc01_g003750 . Gorai.008G069800.1 .
Gorai.003G016900.1 . . . . .
. . . . . . . .
. .
Vv04g1042 . . . . . . .
. . . . . . . .
. . . . .
Vv04g1043 Tc01_g003740 Gorai.007G051300.1 .
Gorai.007G206700.1 . Gorai.011G102800.1 Vv18g1023
. . . . . . . .
. . . . .
Vv04g1044 Tc01_g003730 . . . . .
. . . . . . . .
. . . . . .
Vv04g1045 . . . . . . .
. . . . . . . .
. . . . .
Vv04g1046 . . . . . . .
. . . . . . . .
. . . . .
Vv04g1047 . . . . . .
Vv18g1025 Tc08_g001730 . Gorai.005G107000.1
Gorai.009G204600.1 Gorai.009G274000.1 . . .
. . . . .
Vv04g1048 Tc01_g003710 . . . . .
. . . . . . . .
. . . . . .
Vv04g1049 . Gorai.007G051200.1 Gorai.008G069700.1
Gorai.007G206300.1 . . . . .
. . . . . . . .
. . .
Vv04g1050 Tc01_g003640 . Gorai.008G069400.1 .
Gorai.003G016800.1 . . . . .
. . . . . . . .
. .
Vv04g1051 Tc01_g003620 . Gorai.008G069200.1 .
. Gorai.011G102600.1 . . . .
. . . . . . . .
. .
Vv04g1052 . . . . . . .
. . . . . . . .
. . . . .
Vv04g1053 . . . . . . .
. . . . . . . .
. . . . .
Vv04g1054 . . . . . . .
. . . . . . . .
. . . . .
Vv04g1055 . . . . . . .

. . . . . . . .
. . . . .
Vv04g1056 Tc01_g003610 . Gorai.008G069000.1
Gorai.007G206100.1 Gorai.003G016700.1 . . .
. . . . . . . .
. . . .
Vv04g1057 Tc01_g003600 Gorai.007G051100.1 .
Gorai.007G206000.1 Gorai.003G016600.1 . . .
. . . . . . . .
. . . .
Vv04g1058 Tc01_g003590 . . Gorai.007G205800.1
. . . . . . . .
. . . . . . . .
Vv04g1059 Tc01_g003580 . Gorai.008G068900.1
Gorai.007G205700.1 . . Vv18g1040 . .
. . . . . . . .
. . .
Vv04g1060 Tc01_g003570 . . . .
Gorai.011G102500.1 . . . . . .
. . . . . . . .
Vv04g1061 Tc01_g003560 . . . Gorai.
003G016500.1 Gorai.011G102400.1 . . . .
. . . . . . . .
. .
Vv04g1062 Tc01_g003550 . . Gorai.007G205600.1
. . . . . . . .
. . . . . . . .
Vv04g1063 Tc01_g003540 . . . Gorai.
003G016400.1 . Vv18g1042 Tc08_g001800 Gorai.
002G129300.1 . Gorai.009G204100.1 . . .
. . . . . .
Vv04g1064 . . . . . . .
. . . . . . . .
. . . . .
Vv04g1065 . . . Gorai.007G205500.1 .
. . . . . . . .
. . . . . . .
Vv04g1066 . . . . . . .
. . . . . . . .
. . . . .
Vv04g1067 Tc01_g003520 . . Gorai.007G205300.1
Gorai.003G016200.1 . Vv18g1053 . .
. Gorai.009G203800.1 . Gorai.010G011200.1
. . . . . . .
Vv04g1068 . . . . . . .
. . . . . . . .
. . . . .
Vv04g1069 . . . . . . .
. . . . . . . .
. . . . .

Vv04g1070 . . . . . . .
. . . . . . . .
. . . . .
Vv04g1071 . . . . . . .
. . . . . . . .
. . . . .
Vv04g1072 Tc01_g003510 . . Gorai.007G205200.1
. . . . . . . .
. . . . . . . .
Vv04g1073 Tc01_g003500 . Gorai.008G068800.1 .
. . . . . . . .
. . . . . . . .
Vv04g1074 Tc01_g003490 . Gorai.008G068700.1 .
. . . . . . . .
. . . . . . . .
Vv04g1075 Tc01_g003480 . . . .
Gorai.011G102300.1 . . . . . .
. . . . . . . .
Vv04g1076 . . . . . . .
. . . . . . . .
. . . . .
Vv04g1077 Tc01_g003470 . . Gorai.007G205100.1
Gorai.003G016100.1 . Vv18g1063
Tc08_g001910 . . Gorai.009G203400.1 Gorai.
009G273600.1 . . . . . . .
.
Vv04g1078 . . . . . . .
. . . . . . . .
. . . . .
Vv04g1079 Tc01_g003460 . . . . .
. . . . . . . .
. . . . . .
Vv04g1080 Tc01_g003450 . Gorai.008G068600.1 .
. . Vv18g1064 Tc08_g001920 Gorai.
002G128700.1 . Gorai.009G203300.1 . . .
. . . . . .
Vv04g1081 Tc01_g003430 . . Gorai.007G204600.1
Gorai.003G015900.1 . Vv18g1066
Tc08_g001930 . . Gorai.009G203200.1 .
Gorai.010G010700.1 . . . . . .
.
Vv04g1082 Tc01_g003420 . . Gorai.007G204500.1
. . . . . . . .
. . . . . . . .
Vv04g1083 Tc01_g003410 . . Gorai.007G204400.1
Gorai.003G015800.1 . . . . .
. . . . . . . .
. .
Vv04g1084 . . . . . . .
. . . . . . . .

. . . . .
Vv04g1085 Tc01_g003390 . Gorai.008G068500.1
Gorai.007G204100.1 . . . . . .
. . . . . . . .
. .
Vv04g1086 Tc01_g003370 . . Gorai.007G204000.1
Gorai.003G015600.1 . . . . .
. . . . . . . .
. .
Vv04g1087 Tc01_g003360 . . . .
Gorai.011G101900.1 . . . . . .
. . . . . . . .
Vv04g1088 Tc01_g003350 Gorai.007G050900.1 .
Gorai.007G203800.1 . . . . . .
. . . . . . . .
. .
Vv04g1089 Tc01_g003340 . Gorai.008G068400.1 .
. . . . . . . .
. . . . . . . .
Vv04g1090 . . . . . . .
. . . . . . . .
. . . . .
Vv04g1091 Tc01_g003310 . . Gorai.007G203700.1
. . . . . . . .
. . . . . . . .
Vv04g1092 . . . . . . .
. . . . . . . .
. . . . .
Vv04g1093 . . . . . . .
. . . . . . . .
. . . . .
Vv04g1094 . . . . . . .
. . . . . . . .
. . . . .
Vv04g1095 . . . . . . .
. . . . . . . .
. . . . .
Vv04g1096 . . . . . . .
. . . . . . . .
. . . . .
Vv04g1097 Tc01_g003300 . Gorai.008G068200.1 .
Gorai.003G015500.1 . . . . .
. . . . . . . .
. .
Vv04g1098 Tc01_g003290 . Gorai.008G068100.1 .
Gorai.003G015400.1 . . . . .
. . . . . . . .
. .
Vv04g1099 Tc01_g003280 . . . . .
. . . . . . . .

. . . . . .
Vv04g1100 . . . . . . .
. . . . . . . .
. . . . .
Vv04g1101 . . . . . . .
. . . . . . . .
. . . . .
Vv04g1102 . . . . . . .
. . . . . . . .
. . . . .
Vv04g1103 . . . . . . .
. . . . . . . .
. . . . .
Vv04g1104 . . . . . . .
. . . . . . . .
. . . . .
Vv04g1105 . . . . . . .
. . . . . . . .
. . . . .
Vv04g1106 . . . . . . .
. . . . . . . .
. . . . .
Vv04g1107 . . . . . . .
. . . . . . . .
. . . . .
Vv04g1108 Tc01_g003270 Gorai.007G050700.1 . .
Gorai.003G015300.1 . . . . .
. . . . . . . .
. .
Vv04g1109 Tc01_g003260 . . . Gorai.
003G015200.1 . . . . . . .
. . . . . . . .
Vv04g1110 . . . . . . .
. . . . . . . .
. . . . .
Vv04g1111 . . . . . . .
. . . . . . . .
. . . . .
Vv04g1112 Tc01_g003250 . . Gorai.007G203400.1
Gorai.003G015100.1 . . . . .
. . . . . . . .
. .
Vv04g1113 . . . . . . .
. . . . . . . .
. . . . .
Vv04g1114 Tc01_g003230 . Gorai.008G068000.1 .
Gorai.003G014900.1 . . . . .
. . . . . . . .
. .
Vv04g1115 Tc01_g003220 . . . Gorai.

003G014800.1 . . . . . . .
. . . . . . . .
Vv04g1116 Tc01_g003210 . . . Gorai.
003G014700.1 . . . . . . .
. . . . . . . .
Vv04g1117 Tc01_g003180 . . . Gorai.
003G014500.1 . Vv18g1089 Tc08_g002150 .
Gorai.005G107900.1 . Gorai.009G273200.1 . .
. . . . . .
Vv04g1118 . . . . . . .
. . . . . . . .
. . . . .
Vv04g1119 Tc01_g003170 . . Gorai.007G203200.1
Gorai.003G014400.1 . . . . .
. . . . . . . .
. .
Vv04g1120 Tc01_g003160 . . Gorai.007G203100.1
. . . . . . . .
. . . . . . . .
Vv04g1121 Tc01_g003150 . Gorai.008G067800.1
Gorai.007G202900.1 Gorai.003G014300.1 . . .
. . . . . . . .
. . . .
Vv04g1122 Tc01_g003140 . . Gorai.007G202800.1
. . . . . . . .
. . . . . . . .
Vv04g1123 Tc01_g003130 Gorai.007G050600.1 .
Gorai.007G202700.1 . . Vv18g1092
Tc08_g002170 . Gorai.005G108100.1 . .
Gorai.010G009900.1 . . . . . .
.
Vv04g1124 . . . . . . .
. . . . . . . .
. . . . .
Vv04g1125 Tc01_g003110 Gorai.007G050500.1 .
Gorai.007G202500.1 Gorai.003G014100.1 . Vv18g1094
Tc08_g002200 Gorai.002G127500.1 . Gorai.
009G201300.1 . . . . . . .
. .
Vv04g1126 Tc01_g003100 . Gorai.008G067600.1 .
. . . . . . . .
. . . . . . . .
Vv04g1127 Tc01_g003090 . . Gorai.007G202400.1
. . . . . . . .
. . . . . . . .
Vv04g1128 . . . . . . .
. . . . . . . .
. . . . .
Vv04g1129 Tc01_g003080 . . Gorai.007G202100.1
Gorai.003G014000.1 Gorai.011G101500.1 Vv18g1096

Tc08_g002230 Gorai.002G127200.1 . . .
. Vv01g0637 . Gorai.013G030100.1 .
. Gorai.008G294400.1 .
Vv04g1130 Tc01_g003070 . Gorai.008G067500.1 .
. . . . . . . .
. . . . . . . .
Vv04g1131 Tc01_g003060 . . . Gorai.
003G013900.1 . . . . . . .
. . . . . . . .
Vv04g1132 Tc01_g003050 Gorai.007G050300.1 Gorai.
008G067400.1 . . Gorai.011G101400.1 Vv18g1100
Tc08_g002270 . . Gorai.009G201100.1
Gorai.009G272900.1 Gorai.010G009400.1 . . .
. . . .
Vv04g1133 Tc01_g003040 . . . .
Gorai.011G101300.1 . . . . . .
. . . . . . . .
Vv04g1134 Tc01_g003030 . . Gorai.007G202000.1
. . . . . . . .
. . . . . . . .
Vv04g1135 Tc01_g003020 Gorai.007G050200.1 Gorai.
008G067300.1 . . . . . . .
. . . . . . . .
. .
Vv04g1136 . . . . . . .
. . . . . . . .
. . . . .
Vv04g1137 Tc01_g003000 Gorai.007G050100.1 . .
. Gorai.011G101200.1 . . . .
. . . . . . . .
. .
Vv04g1138 Tc01_g002990 . . . Gorai.
003G013800.1 . . . . . . .
. . . . . . . .
Vv04g1139 Tc01_g002970 Gorai.007G047100.1 Gorai.
008G067000.1 . Gorai.003G013700.1 . . .
. . . . . . . .
. . . .
Vv04g1140 Tc01_g002960 . . . Gorai.
003G013600.1 . Vv18g1104 Tc08_g002320 . .
. . Gorai.010G009200.1 . . .
. . . .
Vv04g1141 Tc01_g002950 . Gorai.008G066900.1 .
. . . . . . . .
. . . . . . . .
Vv04g1142 Tc01_g002940 . . Gorai.007G201900.1
Gorai.003G013500.1 . Vv18g1106
Tc08_g002330 . . . . . . .
. . . . .
Vv04g1143 Tc01_g002930 . . . . .

```
        .       .       .       .       .       .       .       .
        .       .       .       .       .       .
Vv04g1144       .       .       .       Gorai.007G201800.1
Gorai.003G013400.1      .       Vv18g1109       Tc08_g002360    .
        .       .       .       Gorai.010G009000.1      .       .
        .       .       .       .       .
Vv04g1145       .       .       .       .       .       .
Vv18g1111       .       .       .       .       .       .       .
        .       .       .       .       .       .
Vv04g1146       .       .       .       .       .       .       .
        .       .       .       .       .       .       .       .
        .       .       .       .       .
Vv04g1147       Tc01_g002900    .       Gorai.008G066700.1      .
        Gorai.003G013300.1      Gorai.011G101100.1      .       .
        .       .       .       .       .       .       .       .
        .       .       .       .
Vv04g1148       Tc01_g002890    .       .       .       .
Gorai.011G101000.1      .       .       .       .       .       .
        .       .       .       .       .       .       .       .
Vv04g1149       Tc01_g002880    .       .       Gorai.007G201600.1
        .       .       Vv18g1117       Tc08_g002400    .       .
        Gorai.009G200500.1      .       .       .       .       .
        .       .       .       .
Vv04g1150       .       .       .       .       .       .       .
        .       .       .       .       .       .       .       .
        .       .       .       .       .
Vv04g1151       Tc01_g002870    .       Gorai.008G066600.1      .
        .       .       .       .       .       .       .       .
        .       .       .       .       .       .       .       .
Vv04g1152       Tc01_g002860    .       Gorai.008G066400.1
Gorai.007G201500.1      .       Gorai.011G100900.1      Vv18g1121
        Tc08_g002450    Gorai.002G126600.1      Gorai.005G109100.1
        Gorai.009G200200.1      Gorai.009G271900.1      .       .
        .       .       .       .       .       .
Vv04g1153       Tc01_g002850    .       .       .       .
Gorai.011G100800.1      .       .       .       .       .       .
        .       .       .       .       .       .       .       .
Vv04g1154       Tc01_g002800    .       Gorai.008G066300.1      .
        .       .       Vv18g1123       .       .       .       .
        .       .       .       .       .       .       .       .
        .
Vv04g1155       .       .       .       .       .       .       .
        .       .       .       .       .       .       .       .
        .       .       .       .       .
Vv04g1156       .       .       .       .       .       .       .
        .       .       .       .       .       .       .       .
        .       .       .       .       .
Vv04g1157       .       .       .       .       .       .       .
        .       .       .       .       .       .       .       .
        .       .       .       .       .
```

Vv04g1158 . . . . . . .
. . . . . . . .
. . . . .
Vv04g1159 . . Gorai.008G066100.1 . .
. Vv18g1124 Tc08_g002470 . .
Gorai.009G200100.1 . Gorai.010G008500.1 . .
. . . . .
Vv04g1160 . . . . . . .
. . . . . . . .
. . . . .
Vv04g1161 . . . . . .
Vv18g1125 . . . . . . .
. . . . . .
Vv04g1162 . . Gorai.008G065900.1 . .
. . . . . . . .
. . . . . . .
Vv04g1163 Tc01_g002770 . . . . .
Vv18g1126 Tc08_g002490 . . . .
. . . . . . . .
Vv04g1164 . . . . . . .
. . . . . . Vv01g0617
. . . . . .
Vv04g1165 . . . . . . .
. . . . . . . .
. . . . .
Vv04g1166 Tc01_g002750 . . . . .
. . . . . . .
Vv01g0616 . Gorai.013G029300.1 . . .
.
Vv04g1167 . . . . . . .
. . . . . . . .
. . . . .
Vv04g1168 . . . . . . .
. . . . . . Vv01g0614
. . . . . .
Vv04g1169 Tc01_g002740 . . . . .
. . . . . . .
Vv01g0613 . Gorai.013G029200.1 . . .
.
Vv04g1170 . . . . . . .
. . . . . . Vv01g0612
. . . . . .
Vv04g1171 . . . . . . .
. . . . . . . .
. . . . .
Vv04g1172 . . . Gorai.007G201400.1 .
. . . . . . . .
. . . . . . .
Vv04g1173 . . . . . . .
. . . . . . . .

. . . . .
Vv04g1174 . . . . . . .
. . . . . . . .
. . . . .
Vv04g1175 Tc01_g002730 . . . . .
. . . . . . . .
. . . . . .
Vv04g1176 Tc01_g002710 . . Gorai.007G201200.1
. . . . . . . .
. . . . . . . .
Vv04g1177 Tc01_g002700 . Gorai.008G065700.1 .
. . . . . . . .
. . . . . . . .
Vv04g1178 Tc01_g002690 . . . Gorai.
003G013200.1 . . . . . . .
. . . . . . . .
Vv04g1179 . . . . . . .
. . . . . . . .
. . . . .
Vv04g1180 . . . . . . .
. . . . . . . .
. . . . .
Vv04g1181 Tc01_g002680 . . Gorai.007G201000.1
Gorai.003G013100.1 . . . . .
. . . . . . . .
. .
Vv04g1182 . . . . . . .
. . . . . . . .
. . . . .
Vv04g1183 Tc01_g002620 . Gorai.008G065600.1
Gorai.007G200900.1 . . . . . .
. . . . . . . .
. .
Vv04g1184 Tc01_g002610 . . Gorai.007G200700.1
Gorai.003G013000.1 Gorai.011G100700.1 Vv18g1131
Tc08_g002530 . Gorai.005G109200.1 Gorai.
009G199800.1 . . Vv01g0607 . . .
. . .
Vv04g1185 Tc01_g002600 . Gorai.008G065500.1 .
Gorai.003G012900.1 . . . . .
. . . . . . . .
. .
Vv04g1186 Tc01_g002590 . Gorai.008G065400.1 .
Gorai.003G012800.1 Gorai.011G100600.1 . .
. . . . . . . .
. . . .
Vv04g1187 Tc01_g002580 . Gorai.008G065300.1
Gorai.007G200600.1 . . Vv18g1139
Tc08_g002600 Gorai.002G126500.1 Gorai.005G109600.1
Gorai.009G199300.1 . Gorai.010G008000.1 . .

. . . . .
Vv04g1188 Tc01_g002570 . . Gorai.007G200500.1
. . . . . . . .
. . . . . . . .
Vv04g1189 . . . . . . .
. . . . . . . .
. . . . .
Vv04g1190 . . . . . . .
. . . . . . . .
. . . . .
Vv04g1191 . . . . . . .
. . . . . . . .
. . . . .
Vv04g1192 . . . . . . .
. . . . . . . .
. . . . .
Vv04g1193 . . . . . . .
. . . . . . . .
. . . . .
Vv04g1194 . . . . . . .
. . . . . . . .
. . . . .
Vv04g1195 Tc01_g002530 . . . Gorai.
003G012700.1 . . . . . . .
. . . . . . . .
Vv04g1196 . . . . . . .
. . . . . . . .
. . . . .
Vv04g1197 Tc01_g002520 . . Gorai.007G199800.1
Gorai.003G012600.1 . Vv18g1146
Tc08_g002670 . . Gorai.009G198800.1 . .
. . . . . . .
Vv04g1198 . . . . . . .
. . . . . . . .
. . . . .
Vv04g1199 . . . . . . .
. . . . . . . .
. . . . .
Vv04g1200 . . . . . . .
. . . . . . . .
. . . . .
Vv04g1201 . . . . . . .
. . . . . . . .
. . . . .
Vv04g1202 . . . . . . .
. . . . . . . .
. . . . .
Vv04g1203 . . . . . . .
. . . . . . . .
. . . . .

Vv04g1204 . . . . . . .
. . . . . . . .
. . . . .
Vv04g1205 . . . . . . .
. . . . . . . .
. . . . .
Vv04g1206 . . . . . . .
. . . . . . . .
. . . . .
Vv04g1207 . . . . . . .
. . . . . . . .
. . . . .
Vv04g1208 . . . . . . .
. . . . . . . .
. . . . .
Vv04g1209 . . . . . . .
. . . . . . . .
. . . . .
Vv04g1210 . . . . . . .
. . . . . . . .
. . . . .
Vv04g1211 Tc01_g002500 . . . Gorai.
003G012500.1 . . . . . . .
. . . . . . . .
Vv04g1212 Tc01_g002490 . . Gorai.007G199700.1
. . . . . . . .
. . . . . . . .
Vv04g1213 Tc01_g002480 . . Gorai.007G199600.1
. . . . . . . .
. . . . . . . .
Vv04g1214 . . . . . . .
. . . . . . . .
. . . . .
Vv04g1215 Tc01_g002470 . . . .
Gorai.011G100500.1 . . . . . .
. . . . . . . .
Vv04g1216 Tc01_g002450 . . Gorai.007G199400.1
. . . . . . . .
. Vv03g0008 Tc02_g007600 . . .
. .
Vv04g1217 . . . . Gorai.003G012200.1
. Vv18g1154 . Gorai.002G126400.1 .
Gorai.009G198400.1 . . . . .
. . . .
Vv04g1218 . . . . . . .
. . . . . . . .
. . . . .
Vv04g1219 Tc01_g002430 . . . .
Gorai.011G100400.1 . . . . . .
. . . . . . . .

Vv04g1220	Tc01_g002420	.	.	Gorai.007G199300.1
	.	Gorai.011G100300.1	.	.	.	.
	.	.	.	.	.	.	.	.
	.	.
Vv04g1221	Tc01_g002410	.	.	Gorai.007G199100.1
	Gorai.003G012100.1	.	.	.	.	.
	.	.	.	.	.	.	.	.
	.	.
Vv04g1222	Tc01_g002400	.	.	Gorai.007G199000.1
	.	.	.	.	.	.	.	.
	.	.	.	.	.	.	.	.
Vv04g1223	.	.	.	.	.	.	.
	.	.	.	.	.	.	.	.
	.	.	.	.	.
Vv04g1224	.	.	.	.	.	.	.
	.	.	.	.	.	.	.	.
	.	.	.	.	.
Vv04g1225	Tc01_g002390	.	.	.	Gorai.
003G012000.1	.	.	.	.	.	.	.
	.	.	.	.	.	.	.	.
Vv04g1226	Tc01_g002380	.	.	Gorai.007G198900.1
	Gorai.003G011900.1	.	Vv18g1164	.	.
	.	.	.	.	Vv03g0011
Tc02_g007580	.	Gorai.005G246600.1	.	.	.
Vv04g1227	Tc01_g002370	.	.	Gorai.007G198800.1
	.	Gorai.011G100200.1	Vv18g1165
Tc08_g002780	Gorai.002G126200.1	Gorai.005G110000.1	.
	.	.	.	.	.	.	.	.
	.
Vv04g1228	.	.	.	.	.	.	.
	.	.	.	.	.	.	.	.
	.	.	.	.	.
Vv04g1229	.	.	.	.	.	.	.
	.	.	.	.	.	.	.	.
	.	.	.	.	.
Vv04g1230	Tc01_g002360	.	.	Gorai.007G198700.1
	.	Gorai.011G100100.1	Vv18g1179
Tc08_g002870	Gorai.002G125900.1	.	Gorai.009G197500.1
	.	Gorai.010G006900.1	.	.	.	.
	.	.	.
Vv04g1231	Tc01_g002350	.	.	Gorai.007G198400.1
	.	.	.	.	.	.	.	.
	.	.	.	.	.	.	.	.
Vv04g1232	.	.	.	.	.	.	.
	.	.	.	.	.	.	.	.
	.	.	.	.	.
Vv04g1233	Tc01_g002340	.	Gorai.008G065200.1
Gorai.007G198300.1	.	.	.	.	.	.
	.	.	.	.	.	.	.	.
	.	.

Vv04g1234	.	.	.	.	.	.	.
	.	.	.	.	.	.	.	.
	.	.	.	.	.
Vv04g1235	Tc01_g002320	.	.	Gorai.007G198100.1
	Gorai.003G011800.1	.	Vv18g1188
Tc08_g002910	Gorai.002G125600.1	Gorai.005G110600.1
Gorai.009G197300.1	.	Gorai.010G006800.1	Vv03g0023
	Tc02_g007510	Gorai.013G033500.1	Gorai.005G247100.1
	.	.	.
Vv04g1236	Tc01_g002310	.	.	.	.	.
	.	.	.	.	.	.	.	.
	.	.	.	.	.	.
Vv04g1237	Tc01_g002280	.	.	.	.
Gorai.011G099900.1	.	.	.	.	.	.
	.	.	.	.	.	.	.	.
Vv04g1238	.	.	.	.	.	.	.
	.	.	.	.	.	.	.	.
	.	.	.	.	.
Vv04g1239	Tc01_g002260	Gorai.007G047400.1	.	.
	Gorai.003G011600.1	.	.	.	.	.
	.	.	.	.	.	.	.	.
	.	.
Vv04g1240	Tc01_g002250	.	Gorai.008G064900.1	.
	Gorai.003G011500.1	.	Vv18g1191
Tc08_g002950	.	.	.	Gorai.009G271100.1	.
	.	.	.	.	.	.	.
Vv04g1241	Tc01_g002240	.	.	Gorai.007G198000.1
	.	.	Vv18g1192	.	Gorai.002G125300.1
	.	.	.	.	.	.	.	.
	.	.	.
Vv04g1242	Tc01_g002230	.	Gorai.008G064800.1	.
	.	.	.	.	.	.	.	.
	.	.	.	.	.	.	.	.
Vv04g1243	Tc01_g002220	.	Gorai.008G064700.1	.
	.	.	.	.	.	.	.	.
	.	.	.	.	.	.	.	.
Vv04g1244	Tc01_g002210	.	.	Gorai.007G197900.1
	.	.	.	.	.	.	.	.
	.	Vv03g0036	.	Gorai.013G032900.1
Gorai.005G247900.1	.	.	.
Vv04g1245	.	.	.	.	.	.	.
	.	.	.	.	.	.	.	.
	.	.	.	.	.
Vv04g1246	Tc01_g002190	.	.	Gorai.007G197700.1
	.	Gorai.011G099800.1	.	.	.	.
	.	.	.	.	.	.	.	.
	.	.
Vv04g1247	Tc01_g002180	.	.	.	.	.
	.	.	.	.	.	.	.
Vv03g0038	.	.	.	.	.	.

Vv04g1248 . . . . . . .
. . . . . . . .
. . . . .
Vv04g1249 . . Gorai.008G064500.1 Gorai.
007G197600.1 . . . . . . .
. . Vv03g0041 Tc02_g006450 Gorai.
013G032700.1 Gorai.005G248000.1 . . .
Vv04g1250 Tc01_g002140 . . Gorai.007G197400.1
. . . . . . . .
. . . . . . . .
Vv04g1251 Tc01_g002130 . . . Gorai.
003G011400.1 Gorai.011G099700.1 . . . .
. . . Vv03g0044 Tc02_g006430 .
Gorai.005G248200.1 . .
Vv04g1252 . . . Gorai.007G197200.1 .
. . . . . . . .
. . . . . . .
Vv04g1253 . . . . . . .
. . . . . . . .
. . . . .
Vv04g1254 . . . . . . .
. . . . . . . .
. . . . .
Vv04g1255 . . . . . . .
. . . . . . . .
. . . . .
Vv04g1256 Tc01_g002120 . . . Gorai.
003G011300.1 . . . . . . .
. . . . . . . .
Vv04g1257 Tc01_g002110 . Gorai.008G064400.1 .
. . . . . . . .
. . . . . . . .
Vv04g1258 . . . . . . .
. . . . . . . .
. . . . .
Vv04g1259 . . . . . . .
. . . . . . . .
. . . . .
Vv04g1260 Tc01_g002090 . Gorai.008G064200.1 .
. . . . . . . .
. Vv03g0050 Tc02_g006400 Gorai.013G032400.1
. . . .
Vv04g1261 Tc01_g002080 . . Gorai.007G197100.1
. . . . . . . .
. . . . . . . .
Vv04g1262 . . . Gorai.007G197000.1 .
. . . . . . . .
. . . . . . .
Vv04g1263 Tc01_g002070 . Gorai.008G064100.1
Gorai.007G196900.1 Gorai.003G011200.1 Gorai.011G099600.1

Vv18g1195 Tc08_g002980 . Gorai.005G110700.1
Gorai.009G197000.1 . Gorai.010G006600.1
Vv03g0051 Tc02_g006390 Gorai.013G032300.1 .
Gorai.002G004700.1 . .
Vv04g1264 Tc01_g002060 . Gorai.008G064000.1 .
Gorai.003G011100.1 Gorai.011G099500.1 . .
. . . . . . . .
. . . .
Vv04g1265 Tc01_g002040 . Gorai.008G063800.1
Gorai.007G196800.1 . . Vv18g1201 . .
. . . . Vv03g0052
Tc02_g006380 . Gorai.005G248400.1 Gorai.002G004600.1
. Gorai.012G154300.1
Vv04g1266 Tc01_g002030 . . Gorai.007G196700.1
Gorai.003G011000.1 . . . . .
. . . . . . . .
. .
Vv04g1267 . . . . . . .
. . . . . . . .
. . . . .
Vv04g1268 . . . . . . .
. . . . . . . .
. . . . .
Vv04g1269 . . . . . . .
. . . . . . . .
. . . . .
Vv04g1270 . . . . . . .
. . . . . . . .
. . . . .
Vv04g1271 Tc01_g002000 . . . Gorai.
003G010800.1 . . . . . . .
. Vv03g0062 Tc02_g006300 . . .
. .
Vv04g1272 Tc01_g001980 Gorai.007G048000.1 Gorai.
008G063600.1 Gorai.007G196400.1 Gorai.003G010700.1 .
. . . . . . . .
. . . . . .
Vv04g1273 Tc01_g001970 . . . . .
Vv18g1212 . . Gorai.005G111200.1 .
. . . . . . . .
.
Vv04g1274 . . . . . . .
. . . . . . . .
. . . . .
Vv04g1275 Tc01_g001960 . . Gorai.007G196300.1
. . . . . . . .
. . . . . . . .
Vv04g1276 . . . . . . .
. . . . . . . .
. . . . .

Vv04g1277	Tc01_g001950	.	.	Gorai.007G196200.1
	.	.	.	.	.	.	.	.
	.	.	.	.	.	.	.	.
Vv04g1278	Tc01_g001940	.	Gorai.008G063500.1	.
	Gorai.003G010600.1	.	.	.	.	.
	.	.	.	.	.	.	.	.
	.	.
Vv04g1279	Tc01_g001930	.	.	Gorai.007G196100.1
	.	.	.	.	.	.	.	.
	.	.	.	.	.	.	.	.
Vv04g1280	.	.	.	.	.	.	.
	.	.	.	.	.	.	.	.
	.	.	.	.	.
Vv04g1281	Tc01_g001920	.	.	Gorai.007G196000.1
	.	.	Vv18g1233	Tc08_g003320	Gorai.
002G124700.1	Gorai.005G111400.1	.	.	Gorai.
010G005700.1	.	.	.	.	.	.	.
Vv04g1282	Tc01_g001910	Gorai.007G048100.1	.	.
	.	Gorai.011G099300.1	.	.	.	.
	.	.	.	.	.	.	.	.
	.	.
Vv04g1283	Tc01_g001900	.	Gorai.008G063300.1	.
	.	.	.	.	.	.	.	.
	.	.	.	.	.	.	.	.
Vv04g1284	Tc01_g001890	Gorai.007G048200.1	.	.
	Gorai.003G010500.1	Gorai.011G099200.1	.	.
	.	.	.	.	.	.	.	.
	.	.	.	.
Vv04g1285	Tc01_g001880	Gorai.007G048300.1	.	.
	.	.	Vv18g1242	Tc08_g003390	Gorai.
002G124100.1	.	Gorai.009G195900.1	.	Gorai.
010G005400.1	.	.	.	.	.	.	.
Vv04g1286	Tc01_g001870	.	.	Gorai.007G195900.1
	.	Gorai.011G099000.1	.	.	.	.
	.	.	.	.	.	.	.	.
	.	.
Vv04g1287	Tc01_g001860	Gorai.007G048500.1	.	.
	.	.	.	.	.	.	.	.
	.	Vv03g0073	Tc02_g006190	.	.	.
	Gorai.008G032700.1	.
Vv04g1288	Tc01_g001850	.	.	Gorai.007G195800.1
	Gorai.003G010300.1	.	.	.	.	.
	.	.	.	.	.	.	.	.
	.	.
Vv04g1289	.	.	.	.	.	.	.
	.	.	.	.	.	.	.	.
	.	.	.	.	.
Vv04g1290	.	.	.	Gorai.007G195700.1	.
	.	Vv18g1252	Tc08_g003440	Gorai.002G123700.1
	.	Gorai.009G195400.1	.	Gorai.010G005200.1

        .       .       .       .       .       .       .
Vv04g1291       .       .       .       .       .       .       .
        .       .       .       .       .       .       .       .
        .       .       .       .       .
Vv04g1292       Tc01_g001810    .       .       Gorai.007G195500.1
        .       Gorai.011G098800.1      .       .       .       .
        .       .       .       .       .       .       .       .
        .       .
Vv04g1293       Tc01_g001800    .       .       .       Gorai.
003G010100.1    .       .       .       .       .       .       .
        .       .       .       .       .       .       .       .
Vv04g1294       Tc01_g001790    .       .       Gorai.007G195400.1
        .       .       .       .       .       .       .       .
        .       .       .       .       .       .       .       .
Vv04g1295       Tc01_g001780    .       Gorai.008G063200.1      .
        .       Gorai.011G098600.1      .       .       .       .
        .       .       .       .       .       .       .       .
        .       .
Vv04g1296       Tc01_g001770    .       .       .       Gorai.
003G010000.1    .       .       .       .       .       .       .
        .       .       .       .       .       .       .       .
Vv04g1297       Tc01_g001760    .       .       Gorai.007G195300.1
        Gorai.003G009900.1      Gorai.011G098500.1      .       .
        .       .       .       .       .       .       .       .
        .       .       .       .
Vv04g1298       Tc01_g001750    .       .       Gorai.007G195200.1
        Gorai.003G009800.1      .       Vv18g1269
Tc08_g003520    .       .       .       Gorai.009G270100.1
Gorai.010G004800.1      .       .       .       .       .       .
        .
Vv04g1299       .       .       .       .       .       .
Vv18g1272       Tc08_g003540    .       .       .       .
Gorai.010G004600.1      .       .       .       .       .       .
        .
Vv04g1300       Tc01_g001740    .       .       Gorai.007G195100.1
        Gorai.003G009700.1      .       .       .       .       .
        .       .       .       .       .       .       .       .
        .       .
Vv04g1301       Tc01_g001730    .       Gorai.008G063100.1
Gorai.007G194900.1      Gorai.003G009600.1      .       .       .
        .       .       .       .       .       .       .       .
        .       .       .       .
Vv04g1302       Tc01_g001700    .       .       .       .       .
        .       .       .       .       .       .       .       .
        .       .       .       .       .       .
Vv04g1303       .       .       .       .       .       .       .
        .       .       .       .       .       .       .       .
        .       .       .       .       .
Vv04g1304       Tc01_g001680    .       Gorai.008G062900.1
Gorai.007G194700.1      .       .       .       .       .       .

. . . . . . . .
. .
Vv04g1305 . . . . . . .
. . . . . . . .
. . . . .
Vv04g1306 Tc01_g001670 . . Gorai.007G194500.1
Gorai.003G009500.1 Gorai.011G098300.1 . .
. . . . . . . .
. . . .
Vv04g1307 Tc01_g001660 . . Gorai.007G194400.1
Gorai.003G009400.1 . . . . .
. . . Vv03g0143 Tc02_g005550 .
Gorai.005G252200.1 . . .
Vv04g1308 Tc01_g001650 . . Gorai.007G194300.1
. . . . . . . .
. . . . . . . .
Vv04g1309 . . . . . . .
. . . . . . . .
. . . . .
Vv04g1310 Tc01_g001620 Gorai.007G048700.1 Gorai.
008G062700.1 Gorai.007G193900.1 . . . .
. . . . . Vv03g0178
Tc02_g005240 . . Gorai.002G003600.1 Gorai.
008G035100.1 .
Vv04g1311 . . . . . . .
. . . . . . . .
. . . . .
Vv04g1312 Tc01_g001610 . Gorai.008G062600.1
Gorai.007G193800.1 . . Vv18g1291 . .
. Gorai.009G194200.1 . . . .
. . . . .
Vv04g1313 . . Gorai.008G062500.1 Gorai.
007G193700.1 . . . . . . .
. . Vv03g0179 . Gorai.002G195100.1
Gorai.005G253200.1 Gorai.002G003500.1 . .
Vv04g1314 Tc01_g001590 Gorai.007G048900.1 Gorai.
008G062300.1 Gorai.007G193600.1 Gorai.003G009200.1 .
Vv18g1294 Tc08_g003730 Gorai.002G122900.1
Gorai.005G112000.1 Gorai.009G194100.1 . Gorai.
010G003100.1 . . . . . . .
Vv04g1315 Tc01_g001580 . . Gorai.007G193400.1
. . Vv18g1295 Tc08_g003740 . .
Gorai.009G194000.1 . . . . .
. . . .
Vv04g1316 Tc01_g001570 . . . Gorai.
003G009100.1 . . . . . . .
. . . . . . . .
Vv04g1317 Tc01_g001550 . . . . .
. . . . . . . .
. . . . . .

Vv04g1318 Tc01_g001540 . . . Gorai.
003G008900.1 . . . . . . .
. . . . . . . .
Vv04g1319 Tc01_g001530 . . Gorai.007G193300.1
Gorai.003G008800.1 . . . . .
. . . . . . . .
. .
Vv04g1320 . . . . . . .
. . . . . . . .
. . . . .
Vv04g1321 . . . . . . .
. . . . . . . .
. . . . .
Vv04g1322 Tc01_g001520 Gorai.007G049000.1 . .
. . . . . . . .
. . . . . . . .
Vv04g1323 Tc01_g001510 . . Gorai.007G193200.1
. . . . . . . .
. . . . . . . .
Vv04g1324 Tc01_g001500 . . . Gorai.
003G008700.1 . . . . . . .
. . . . . . . .
Vv04g1325 . . . . . . .
. . . . . . . .
. . . . .
Vv04g1326 . . . . . . .
. . . . . . . .
. . . . .
Vv04g1327 Tc01_g001490 . . . . .
. . . . . . . .
. . . . . .
Vv04g1328 . . . . Gorai.003G008600.1
. . . . . . . .
. . . . . . .
Vv04g1329 Tc01_g001480 . . . Gorai.
003G008500.1 . . . . . . .
. Vv03g0194 Tc02_g005110 . . .
Gorai.008G035600.1 .
Vv04g1330 Tc01_g001470 Gorai.007G049200.1 . .
Gorai.003G008400.1 . . . . .
. . . . . . . .
. .
Vv04g1331 Tc01_g001460 . . Gorai.007G193100.1
Gorai.003G008300.1 . . . . .
. . . Vv03g0196 Tc02_g005090 .
Gorai.005G253900.1 . . .
Vv04g1332 Tc01_g001450 . . Gorai.007G193000.1
. . . . . . . .
. . . . . . . .
Vv04g1333 Tc01_g001440 . . Gorai.007G192900.1

. . . . . . . .
. . . . . . . .
Vv04g1334 . . . . . . .
. . . . . . . .
. . . . .
Vv04g1335 . . . Gorai.007G192700.1 .
. . . . . . . .
. . . . . . .
Vv04g1336 . . . . . . .
. . . . . . . .
. . . . .
Vv04g1337 . . . . . . .
. . . . . . . .
. . . . .
Vv04g1338 Tc01_g001430 . . Gorai.007G192600.1
. . . . . . . .
. . . . . . . .
Vv04g1339 Tc01_g001420 . . . Gorai.
003G008200.1 . . . . . . .
. . . . . . . .
Vv04g1340 Tc01_g001410 Gorai.007G049300.1 . .
Gorai.003G008100.1 . . . . .
. . . . . . . .
. .
Vv04g1341 Tc01_g001400 . . Gorai.007G192500.1
. . . . . . . .
. . . . . . . .
Vv04g1342 Tc01_g001390 . . . .
Gorai.011G098200.1 . . . . . .
. . . . . . . .
Vv04g1343 Tc01_g001380 . . . .
Gorai.011G096500.1 . . . . . .
. . . . . . . .
Vv04g1344 . . . . . . .
. . . . . . . .
. . . . .
Vv04g1345 Tc01_g001370 . . . . .
. . . . . . . .
. . . . . .
Vv04g1346 . . Gorai.008G062200.1 . .
. . . . . . . .
. . . . . . .
Vv04g1347 Tc01_g001360 . . Gorai.007G192400.1
. . . . . . . .
. . . . . . . .
Vv04g1348 . . . . . . .
. . . . . . Vv03g0200
Tc02_g005050 Gorai.002G188800.1 . .
Gorai.008G035900.1 .
Vv04g1349 . . . . . . .

. . . . . . Vv03g0201
. . . Gorai.002G003200.1 . .
Vv04g1350 . . . . . . .
. . . . . . . .
. . . . .
Vv04g1351 . . . . . . .
. . . . . . Vv03g0202
Tc02_g005040 Gorai.002G197200.1 . .
Gorai.008G036000.1 .
Vv04g1352 Tc01_g001350 . . Gorai.007G192300.1
Gorai.003G008000.1 . Vv18g1312
Tc08_g003890 . Gorai.005G112500.1 Gorai.009G193300.1
Gorai.009G269600.1 Gorai.010G002600.1 Vv03g0204
Tc02_g005020 Gorai.002G197500.1 . .
Gorai.008G036100.1 .
Vv04g1353 Tc01_g001330 Gorai.007G049400.1 . .
. . Vv18g1313 Tc08_g003910 Gorai.
002G122600.1 . Gorai.009G193200.1 . . .
. . . . . .
Vv04g1354 . . . . . . .
. . . . . . . .
. . . . .
Vv04g1355 . . . . . . .
. . . . . . . .
. . . . .
Vv04g1356 Tc01_g001320 . . . Gorai.
003G007800.1 . . . . . . .
. Vv03g0209 Tc02_g004960 Gorai.002G198000.1
. . . .
Vv04g1357 Tc01_g001300 . . Gorai.007G192100.1
Gorai.003G007500.1 . . . . .
. . . . . . . .
. .
Vv04g1358 . . . . . . .
. . . . . . . .
. . . . .
Vv04g1359 Tc01_g001240 . . Gorai.007G191800.1
Gorai.003G007000.1 . Vv18g1339
Tc08_g004010 Gorai.002G122200.1 . Gorai.009G192500.1
. . . . . . . .
.
Vv04g1360 Tc01_g001230 . . Gorai.007G191700.1
Gorai.003G006800.1 . Vv18g1340
Tc08_g004020 Gorai.002G121900.1 . . . .
. . . . . . .
Vv04g1361 . . . . . . .
. . . . . . . .
. . . . .
Vv04g1362 . . . Gorai.007G191500.1
Gorai.003G006700.1 . . . . . .

```
. . . . . . . .
.
Vv04g1363 Tc01_g001200 . . . . .
. . . . . . . .
. . . . . .
Vv04g1364 Tc01_g001190 Gorai.007G049900.1 . .
Gorai.003G006600.1 . . . . .
. . . . . . . .
. .
Vv04g1365 . . . . . . .
. . . . . . . .
. . . . .
Vv04g1366 Tc01_g001170 . . Gorai.007G191200.1
Gorai.003G006400.1 . . . . .
. . . . . . . .
. .
Vv04g1367 Tc01_g001150 . . Gorai.007G191000.1
. . . . . . . .
. . . . . . . .
Vv04g1368 . . . . . . .
. . . . . . . .
. . . . .
Vv04g1369 Tc01_g001100 . Gorai.008G061900.1 .
Gorai.003G006000.1 . . . . .
. . . . . . . .
. .
Vv04g1370 . . . . . . .
. . . . . . . .
. . . . .
Vv04g1371 Tc01_g001090 . Gorai.008G061800.1
Gorai.007G190600.1 . . Vv18g0195
Tc08_g004190 . Gorai.005G112700.1 Gorai.009G191400.1
Gorai.009G269000.1 . . . . .
. . .
Vv04g1372 Tc01_g001070 . . . Gorai.
003G005800.1 . . . . . . .
. . . . . . . .
Vv04g1373 . . . . . .
Vv18g0191 . . . . . . .
. . . . . .
Vv04g1374 . . . Gorai.007G190500.1
Gorai.003G005700.1 . Vv18g0190 . . .
. . . . . . . .
. .
Vv04g1375 Tc01_g001060 . . . . .
. . . . . . . .
. . . . . .
Vv04g1376 . . . . Gorai.003G005500.1
. Vv18g0181 Tc08_g004280 Gorai.002G120800.1
. . . . . . . .
```

. . .
Vv04g1377 . . . . . . .
. . . . . . . .
. . . . .
Vv04g1378 Tc01_g001040 . Gorai.008G061600.1 .
. . Vv18g0180 . . . .
Gorai.009G268500.1 . . . . .
. . .
Vv04g1379 . . . . . . .
. . . . . . . .
. . . . .
Vv04g1380 . . . . . . .
. . . . . . . .
. . . . .
Vv04g1381 Tc01_g001030 . . . Gorai.
003G005400.1 . . . . . . .
. . . . . . . .
Vv04g1382 Tc01_g001020 . . . Gorai.
003G005300.1 . . . . . . .
. . . . . . . .
Vv04g1383 . . . . . . .
. . . . . . . .
. . . . .
Vv04g1384 Tc01_g001010 . . Gorai.007G190300.1
Gorai.003G005200.1 . . . . .
. . . . . . . .
. .
Vv04g1385 Tc01_g001000 . Gorai.008G061500.1 .
. . . . . . . .
. . . . . . . .
Vv04g1386 Tc01_g000990 . . Gorai.007G190200.1
Gorai.003G005100.1 . . . . .
. . . . . . . .
. .
Vv04g1387 Tc01_g000980 . . . Gorai.
003G005000.1 . . . . . . .
. . . . . . . .
Vv04g1388 Tc01_g000970 . Gorai.008G061400.1 .
. . . . . . . .
. . . . . . . .
Vv04g1389 Tc01_g000960 . . . Gorai.
003G004900.1 . . . . . . .
. . . . . . . .
Vv04g1390 . . . . . . .
. . . . . . . .
. . . . .
Vv04g1391 . . . . . . .
. . . . . . . .
. . . . .
Vv04g1392 . . . . . . .

. . . . . . . .
. . . . .
Vv04g1393 Tc01_g000950 . Gorai.008G059900.1 .
Gorai.003G004800.1 . . . . .
. . . . . . . .
. .
Vv04g1394 . . . . . . .
. . . . . . . .
. . . . .
Vv04g1395 Tc01_g000930 . . . Gorai.
003G004700.1 . . . . . . .
. . . . . . . .
Vv04g1396 . . . . Gorai.003G004600.1
. . . . . . . .
. . . . . . .
Vv04g1397 . . . . . . .
. . . . . . . .
. . . . .
Vv04g1398 Tc01_g000550 . . . Gorai.
003G002700.1 . . . . . . .
. . . . . . . .
Vv04g1399 . . . . . . .
. . . . . . . .
. . . . .
Vv04g1400 Tc01_g000540 . . . Gorai.
003G002600.1 . . . . . . .
. . . . . . . .
Vv04g1401 . . . . . . .
. . . . . . . .
. . . . .
Vv04g1402 Tc01_g000530 . . . Gorai.
003G002500.1 . . . . . . .
. . . . . . . .
Vv04g1403 . . . . . . .
. . . . . . . .
. . . . .
Vv04g1404 Tc01_g000520 . . . . .
. . . . . . . .
. . . . . .
Vv04g1405 . . . . . . .
. . . . . . . .
. . . . .
Vv04g1406 . . . . . . .
. . . . . . . .
. . . . .
Vv04g1407 Tc01_g000510 . . . . .
. . . . . . . .
. . . . . .
Vv04g1408 . . . . . . .
. . . . . . . .

. . . . .
Vv04g1409 Tc01_g000500 . . . . .
Vv18g0171 Tc08_g004370 . . Gorai.
009G190400.1 . . . . . . .
. .
Vv04g1410 Tc01_g000490 . . . . .
. . . . . . . .
. . . . . .
Vv04g1411 Tc01_g000480 . . . Gorai.
003G002400.1 . . . . . . .
. . . . . . . .
Vv04g1412 Tc01_g000470 . . . Gorai.
003G002300.1 . . . . . . .
. . . . . . . .
Vv04g1413 Tc01_g000460 . . . . .
. . . . . . .
Vv02g0264 Tc03_g029890 . . Gorai.007G111400.1
Gorai.008G258900.1 .
Vv04g1414 . . . . . . .
. . . . . . . .
. . . . .
Vv04g1415 . . . . Gorai.003G002200.1
. . . . . . . .
. . . . . . .
Vv04g1416 . . . . Gorai.003G002000.1
. . . . . . . .
. . . . . . .
Vv04g1417 . . . . Gorai.003G000600.1
. . . . . . . .
. . . . . . .
Vv04g1418 . . . . . . .
. . . . . . . .
. . . . .
Vv04g1419 . . . . . .
Vv18g0154 . . . . . .
Vv02g0280 . Gorai.004G129000.1 . . .
.
Vv04g1420 Tc01_g010700 . . . . .
Vv18g0153 Tc08_g004530 Gorai.002G119700.1 .
. . . Vv02g0318 . . .
. Gorai.008G262100.1 .
Vv04g1421 . . . Gorai.007G185500.1 .
. . . . . . . .
. . . . . . .
Vv04g1422 . . . . . . .
. . . . . . . .
. . . . .
Vv04g1423 . . . . . . .
. . . . . . . .
. . . . .

Vv04g1424 . . . . . . .
. . . . . . . .
. . . . .
Vv04g1425 . . . . . . .
. . . . . . . .
. . . . .
Vv04g1426 . . . . . . .
. . . . . . . .
. . . . .
Vv04g1427 . . . . . . .
. . . . . . . .
. . . . .
Vv04g1428 . . . . . . .
. . . . . . . .
. . . . .
Vv04g1429 . . . . . . .
. . . . . . . .
. . . . .
Vv04g1430 . . . . . . .
. . . . . . . .
. . . . .
Vv04g1431 . . . . . . .
. . . . . . . .
. . . . .
Vv04g1432 . . . . . . .
. . . . . . . .
. . . . .
Vv04g1433 . . . . . . .
. . . . . . . .
. . . . .
Vv04g1434 Tc01_g010740 . . . . .
. . . . . . . .
. . . . . .
Vv04g1435 . . . . . . .
. . . . . . . .
. . . . .
Vv04g1436 Tc01_g010750 . . . . .
. . . . . . . .
. . . . . .
Vv04g1437 . . . . . . .
. . . . . . . .
. . . . .
Vv04g1438 . . . . . . .
. . . . . . . .
. . . . .
Vv04g1439 . . . . . . .
. . . . . . . .
. . . . .
Vv04g1440 . . . . . . .
. . . . . . . .

```
         .       .       .       .       .
Vv04g1441        .       .       .       .       .       .
Vv01g0836        .       Gorai.002G164300.1      Gorai.005G184500.1
         Gorai.009G344100.1      .       .       .       .       .
         .       .       .       .
Vv04g1442        .       .       .       .       .       .       .
         .       .       .       .       .       .       .       .
         .       .       .       .       .
Vv04g1443        Tc01_g010770    Gorai.007G063300.1      .       .
         .       .       Vv01g0835       .       .       .       .
         .       .       .       .       .       .       .       .
         .
Vv04g1444        .       .       .       .       .       .
Vv01g0834        .       .       .       .       .       .       .
         .       .       .       .       .       .
Vv04g1445        .       .       .       .       .       .       .
         .       .       .       .       .       .       .       .
         .       .       .       .       .
Vv04g1446        .       .       .       .       .       .
Vv01g0833        .       .       .       .       .       .
Vv02g0321        .       .       .       .       .       .
Vv04g1447        .       .       .       .       .       .       .
         .       .       .       .       .       .       .       .
         .       .       .       .       .
Vv04g1448        .       .       .       .       .       .
Vv01g0832        Tc02_g023290    .       Gorai.005G184400.1      .
         .       .       Vv02g0322       .       .       .       .
         .       .
Vv04g1449        .       .       .       .       .       .       .
         .       .       .       .       .       .       Vv02g0327
         .       .       .       .       .       .
Vv04g1450        .       .       .       .       .       .
Vv01g0821        Tc02_g023300    .       Gorai.005G184300.1      .
         .       .       .       .       .       .       .       .
         .
Vv04g1451        .       .       .       .       .       .       .
         .       .       .       .       .       .       .       .
         .       .       .       .       .
Vv04g1452        .       .       .       .       .       .       .
         .       .       .       .       .       .       .       .
         .       .       .       .       .
Vv04g1453        Tc01_g010790    .       .       .       .       .
         .       .       .       .       .       .       .       .
         .       .       .       .       .       .
Vv04g1454        .       .       .       .       .       .       .
         .       .       .       .       .       .       .       .
         .       .       .       .       .
Vv04g1455        Tc01_g010800    .       .       .       .
Gorai.011G115200.1      .       .       .       .       .       .
         .       .       .       .       .       .       .       .
```

Vv04g1456 Tc01_g010810 . . . . .
. . . . . . .
Vv02g0331 . . . . Gorai.008G262400.1
.
Vv04g1457 . . . . . . .
. . . . . . . .
. . . . .
Vv04g1458 Tc01_g010820 . . . . .
. . . . . . . .
. . . . . .
Vv04g1459 Tc01_g010830 . . . . .
. . . . . . . .
. . . . . .
Vv04g1460 . . . . . . .
. . . . . . . .
. . . . .
Vv04g1461 Tc01_g010880 Gorai.007G063500.1 . .
. . . . . . . .
. Vv02g0352 Tc03_g027520 Gorai.004G212300.1
Gorai.003G158400.1 . . .
Vv04g1462 . . . . . . .
. . . . . . . .
. . . . .
Vv04g1463 . . . . . . .
. . . . . . . .
. . . . .
Vv04g1464 Tc01_g010900 Gorai.007G063600.1 . .
. . . . . . . .
. . . . . . . .
Vv04g1465 Tc01_g010920 . . . .
Gorai.011G115300.1 . . . . . .
. . . . . . . .
Vv04g1466 Tc01_g010940 . . . . .
. . . . . . . .
. . . . . .
Vv04g1467 Tc01_g010950 . . . . .
Vv01g0816 . . . . . .
. . . . . . .
Vv04g1468 . . . . . . .
. . . . . . . .
. . . . .
Vv04g1469 . . . . . . .
. . . . . . . .
. . . . .
Vv04g1470 Tc01_g011020 Gorai.007G063800.1 . .
. . . . . . . .
. . . . . . . .
Vv04g1471 Tc09_g009880 . . . . .
. . . . . . . .
. . . . . .

Vv04g1472 Tc01_g011030 . . . . .
. . . . . . . .
. . . . . .
Vv04g1473 . . . . . . .
. . . . . . . .
. . . . .
Vv04g1474 . . . . . . .
. . . . . . . .
. . . . .
Vv04g1475 Tc01_g011040 . . . . .
. . . . . . . .
. . . . . .
Vv04g1476 . . . . . . .
. . . . . . . .
. . . . .
Vv04g1477 . . . . . . .
. . . . . . . .
. . . . .
Vv04g1478 . . . . . . .
. . . . . . . .
. . . . .
Vv04g1479 . . . . . . .
. . . . . . . .
. . . . .
Vv04g1480 . . . . . . .
. . . . . . . .
. . . . .
Vv04g1481 . . . . . . .
. . . . . . . .
. . . . .
Vv04g1482 . . . . . . .
. . . . . . . .
. . . . .
Vv04g1483 . . . . . . .
. . . . . . . .
. . . . .
Vv04g1484 . . . . . . .
. . . . . . . .
. . . . .
Vv04g1485 . . . . . . .
. . . . . . . .
. . . . .
Vv04g1486 Tc01_g011050 . . . . .
. . . . . . . .
. . . . . .
Vv04g1487 Tc01_g011060 . . . . .
. . . . . . . .
. . . . . .
Vv04g1488 . . . . . . .
. . . . . . . .

. . . . .
Vv04g1489 . . . . . . .
. . . . . . . .
. . . . .
Vv04g1490 . . . . . . .
. . . . . . . .
. . . . .
Vv04g1491 . . . . . . .
. . . . . . . .
. . . . .
Vv04g1492 Tc01_g011070 . . . . .
. . . . . . . .
. . . . . .
Vv04g1493 . . . . . . .
. . . . . . . .
. . . . .
Vv04g1494 Tc01_g011090 . . . . .
. . . . . . . .
. . . . . .
Vv04g1495 . . . . . . .
. . . . . . . .
. . . . .
Vv04g1496 Tc01_g011140 . . . . .
. . . . . . . .
. . . . . .
Vv04g1497 Tc01_g011150 . . . . .
. . . . . . . .
. . . . . .
Vv04g1498 Tc01_g011160 Gorai.007G064600.1 . .
. Gorai.011G115500.1 . . . .
. . . . . . . .
. .
Vv04g1499 Tc01_g011170 Gorai.007G064700.1 . .
. Gorai.011G115700.1 . . . .
. . . . . . . .
. .
Vv04g1500 . . . . . . .
. . . . . . . .
. . . . .
Vv04g1501 Tc01_g011180 . . . .
Gorai.011G115900.1 . . . . . .
. . . . . . . .
Vv04g1502 . . . . . . .
. . . . . . . .
. . . . .
Vv04g1503 . . . . . . .
. . . . . . . .
. . . . .
Vv04g1504 Tc01_g011220 . . . . .
. . . . . . . .

. . . . . .
Vv04g1505 Tc01_g011230 . . . .
Gorai.011G116000.1 . . . . . .
. . . . . . . .
Vv04g1506 Tc01_g011280 . . . . .
. . . . . . . .
. . . . . .
Vv04g1507 Tc01_g011320 . . . . .
. . . . . . . .
. . . . . .
Vv04g1508 Tc01_g021630 . . . . .
. . . . . . . .
. . . . . .
Vv04g1509 Tc09_g009460 . . . . .
. . . . . . . .
. . . . . .
Vv04g1510 Tc01_g011380 . . . . .
. . . . . . . .
. . . . . .
Vv04g1511 Tc01_g011390 . . . . .
Vv18g1411 . . . . . .
. . . . . . .
Vv04g1512 Tc01_g011410 . . . . .
. . . . . . . .
. . . . . .
Vv04g1513 Tc01_g011420 Gorai.007G064800.1 . .
. Gorai.011G116600.1 Vv18g1409 . .
. . . . . . . .
. . .
Vv04g1514 Tc01_g011430 Gorai.007G064900.1 . .
. . . . . . . .
. . . . . . . .
Vv04g1515 . . . . . . .
. . . . . . . .
. . . . .
Vv04g1516 Tc01_g037920 . . . . .
. . . . . . . .
. . . . . .
Vv04g1517 . . . . . . .
. . . . . . . .
. . . . .
Vv04g1518 Tc01_g037930 . . . . .
. . . . . . . .
. . . . . .
Vv04g1519 . . . . . . .
. . . . . . . .
. . . . .
Vv04g1520 Tc01_g011470 . . . . .
Vv18g1402 . . . . . .
. . . . . . .

Vv04g1521 . . . . . . .
. . . . . . . .
. . . . .
Vv04g1522 Tc01_g011480 . . . . .
. . . . . . . .
. . . . . .
Vv04g1523 . . . . . .
Vv18g1397 . . Gorai.005G082400.1 . .
. . . . . . . .
Vv04g1524 . Gorai.007G065000.1 . . .
Gorai.011G122400.1 . . . . .
. . . . . . . .
.
Vv04g1525 Tc01_g011540 . . . . .
Vv18g1394 Tc08_g004030 . . . .
. . . . . . . .
Vv04g1526 . . . . . . .
. . . . . . . .
. . . . .
Vv04g1527 Tc01_g011550 Gorai.007G065100.1 . .
. . . . . . . .
. . . . . . . .
Vv04g1528 . . . . . . .
. . . . . . . .
. . . . .
Vv04g1529 . . . . . . .
. . . . . . . .
. . . . .
Vv04g1530 . . . . . . .
. . . . . . . .
. . . . .
Vv04g1531 . Gorai.007G065500.1 . . .
. . . . . . . .
. . . . . . .
Vv04g1532 . . . . . . .
. . . . . . . .
. . . . .
Vv04g1533 Tc01_g011600 . . . . .
. . . . . . . .
. . . . . .
Vv04g1534 Tc01_g011610 Gorai.007G065600.1 . .
. . . . . . . .
. . . . . . . .
Vv04g1535 . . . . . . .
. . . . . . . .
. . . . .
Vv04g1536 Tc01_g011620 . . . . .
. . . . . . . .
. . . . . .
Vv04g1537 Tc01_g011650 . . . .

Gorai.011G120000.1 . . . . . .
. . . . . . . .
Vv04g1538 Tc01_g011710 . . . . .
. . . . . . . .
. . . . . .
Vv04g1539 . . . . . . .
. . . . . . . .
. . . . .
Vv04g1540 Tc01_g022000 . . . . .
. . . . . . . .
. . . . . .
Vv04g1541 . . . . . . .
. . . . . . . .
. . . . .
Vv04g1542 . . . . . . .
. . . . . . . .
. . . . .
Vv04g1543 Tc01_g011740 . . . . .
. . . . . . . .
. . . . . .
Vv04g1544 . . . . . . .
. . . . . . . .
. . . . .
Vv04g1545 Tc01_g011750 Gorai.007G065800.1 . .
. . Vv18g1386 . . Gorai.
005G083200.1 . . . . . . .
. . .
Vv04g1546 . . . . . . .
. . . . . . . .
. . . . .
Vv04g1547 Tc01_g011760 . . . .
Gorai.011G119900.1 . . . . . .
. . . . . . . .
Vv04g1548 . . . . . . .
. . . . . . . .
. . . . .
Vv04g1549 Tc01_g011790 . . . . .
. . . . . . . .
. . . . . .
Vv04g1550 Tc01_g011800 . . . . .
. . . . . . . .
. . . . . .
Vv04g1551 Tc01_g011810 . . . .
Gorai.011G119800.1 . . . . . .
. . . . . . . .
Vv04g1552 Tc01_g011820 . . . . .
. . . . . . . .
. . . . . .
Vv04g1553 Tc01_g011830 . . . . .
. . . . . . . .

. . . . . .
Vv04g1554 . . . . . . .
. . . . . . . .
. . . . .
Vv04g1555 . . . . . . .
. . . . . . . .
. . . . .
Vv04g1556 Tc01_g038130 . . . . .
Vv18g1382 . . . . . .
. . . . . . .
Vv04g1557 Tc01_g011910 . . . . .
. . . . . . . .
. . . . . .
Vv04g1558 . . . . . . .
. . . . . . . .
. . . . .
Vv04g1559 . . . . . . .
. . . . . . . .
. . . . .
Vv04g1560 Tc01_g011930 . . . . .
. . . . . . . .
. . . . . .
Vv04g1561 Tc01_g011940 . . . . .
. . . . . . . .
. . . . . .
Vv04g1562 . . . . . . .
. . . . . . . .
. . . . .
Vv04g1563 . . . . . . .
. . . . . . . .
. . . . .
Vv04g1564 Tc01_g011950 . . . . .
Vv18g1376 . . Gorai.005G084100.1 .
. . . . . . . .
.
Vv04g1565 . . . . . . .
. . . . . . . .
. . . . .
Vv04g1566 Tc01_g011990 . . . . .
. . . . . . . .
. . . . . .
Vv04g1567 Tc01_g012000 . . . .
Gorai.011G119300.1 . . . . . .
. . . . . . . .
Vv04g1568 . . . . . . .
. . . . . . . .
. . . . .
Vv04g1569 Tc01_g012040 . . . .
Gorai.011G119100.1 . . . . . .
. . . . . . . .

Vv04g1570 Tc01_g012050 . . . . .
Vv18g1348 . . Gorai.005G085000.1
Gorai.009G251000.1 Gorai.009G251000.1 . . .
. . . . .
Vv04g1571 . . . . . . .
. . . . . . . .
. . . . .
Vv04g1572 Tc01_g012060 . . . . .
. . . . . . . .
. . . . . .
Vv04g1573 Tc01_g012070 . . . . .
. . . . . . . .
. . . . . .
Vv04g1574 . . . . . . .
. . . . . . . .
. . . . .
Vv04g1575 Tc01_g012090 . . . . .
. . . . . . . .
. . . . . .
Vv04g1576 . . . . . . .
. . . . . . . .
. . . . .
Vv04g1577 Tc01_g012220 . . . . .
. . . . . . . .
. . . . . .
Vv04g1578 . . . . . . .
. . . . . . . .
. . . . .
Vv04g1579 . . . . . . .
. . . . . . . .
. . . . .
Vv04g1580 . . . . . . .
. . . . . . . .
. . . . .
Vv04g1581 Tc01_g018550 . . . . .
. . . . . . . .
. . . . . .
Vv04g1582 . . . . . . .
. . . . . . . .
. . . . .
Vv04g1583 . . . . . . .
. . . . . . . .
. . . . .
Vv04g1584 . . . . . . .
. . . . . . . .
. . . . .
Vv04g1585 Tc01_g018680 . . . . .
. . . . . . . .
. . . . . .
Vv04g1586 Tc01_g012260 . . . . .

. . . . . . . .
. . . . . .
Vv04g1587 . . . . . . .
. . . . . . . .
. . . . .
Vv04g1588 . . . . . . .
. . . . . . . .
. . . . .
Vv04g1589 . . . . . . .
. . . . . . . .
. . . . .
Vv04g1590 . . . . . . .
. . . . . . . .
. . . . .
Vv04g1591 . . . . . . .
. . . . . . . .
. . . . .
Vv04g1592 . . . . . . .
. . . . . . . .
. . . . .
Vv04g1593 . . . . . . .
. . . . . . . .
. . . . .
Vv04g1594 . . . . . . .
. . . . . . . .
. . . . .
Vv04g1595 Tc01_g025360 Gorai.007G066000.1 . .
. Gorai.011G118900.1 . . . .
. . . . . . . .
. .
Vv04g1596 Tc01_g025400 . . . .
Gorai.011G115900.1 . . . . . .
. . . . . . . .
Vv04g1597 Tc01_g012290 . . . . .
. . . . . . . .
. . . . . .
Vv04g1598 . . . . . . .
. . . . . . . .
. . . . .
Vv04g1599 Tc01_g000230 . . . . .
. . . . . . . .
. . . . . .
Vv04g1600 . . . . . . .
. . . . . . . .
. . . . .
Vv04g1601 Tc01_g000220 . . . . .
. . . . . . . .
. . . . . .
Vv04g1602 Tc01_g000210 . . . . .
. . . . . . . .

. . . . . .
Vv04g1603 Tc01_g000190 . . . . .
. . . . . . . .
. . . . . .
Vv04g1604 . . . . . . .
. . . . . . . .
. . . . .
Vv04g1605 Tc01_g000170 . . . . .
. . . . . . . .
. . . . . .
Vv04g1606 . . . . . . .
. . . . . . . .
. . . . .
Vv04g1607 . . . . . . .
. . . . . . . .
. . . . .
Vv04g1608 . . . . . . .
. . . . . . . .
. . . . .
Vv04g1609 . . . . . . .
. . . . . . . .
. . . . .
Vv04g1610 Tc01_g000160 . . . . .
. . . . . . . .
. . . . . .
Vv04g1611 . . . . . . .
. . . . . . . .
. . . . .
Vv04g1612 . . . . . . .
. . . . . . . .
. . . . .
Vv04g1613 . . . . . . .
. . . . . . . .
. . . . .
Vv04g1614 . . . . . . .
. . . . . . . .
. . . . .
Vv04g1615 . . . . . . .
. . . . . . . .
. . . . .
Vv04g1616 . . . . . . .
. . . . . . . .
. . . . .
Vv04g1617 . . . . . . .
. . . . . . . .
. . . . .
Vv04g1618 . . . . . . .
. . . . . . . .
. . . . .
Vv04g1619 . . . . . . .

| | | | | | | | |
|---|---|---|---|---|---|---|---|
| Vv04g1620 | Tc01_g000150 | | . | . | . | . | . |
| Vv04g1621 | . | . | . | . | . | . | . |
| Vv04g1622 | . | . | . | . | . | . | . |
| Vv04g1623 | . | . | . | . | . | . | . |
| Vv04g1624 | Tc01_g000140 | | . | . | . | . | . |
| Vv04g1625 | . | . | . | . | . | . | . |
| Vv04g1626 | Tc01_g000130 | | . | . | . | . | . |
| Vv04g1627 | Tc01_g000090 | | . | . | . | . | . |
| Vv04g1628 | . | . | . | . | . | . | . |
| Vv04g1629 | . | . | . | . | . | . | . |
| Vv04g1630 | . | . | . | . | . | . | . |
| Vv04g1631 | . | . | . | . | . | . | . |
| Vv04g1632 | . | . | . | . | . | . | . |
| Vv04g1633 | Tc01_g001700 | | . | . | . | . | . |
| Vv04g1634 | . | . | . | . | . | . | . |
| Vv04g1635 | Tc01_g002040 | | . | . | . | . | . |

Vv04g1636 . . . . . . .
. . . . . . . .
. . . . .
Vv04g1637 . . . . . . .
. . . . . . . .
. . . . .
Vv04g1638 . . . . . . .
. . . . . . . .
. . . . .
Vv05g0001 . . Gorai.005G060800.1 . .
. . . . . . . .
. . . . . . .
Vv05g0002 . . . . . . .
. . . . . . . .
. . . . .
Vv05g0003 . . . . . . .
. . . . . . . .
. . . . .
Vv05g0004 . . . . . . .
. . . . . . . .
. . . . .
Vv05g0005 Tc04_g028280 Gorai.006G261800.1 . .
. . . . . . . .
. . . . . . . .
Vv05g0006 . . . . . .
Vv14g0248 . . . . . . .
. . . . . .
Vv05g0007 . . . . . . .
. . . . . . . .
. . . . .
Vv05g0008 Tc04_g028290 Gorai.006G261700.1 Gorai.
005G060900.1 . . . . . . .
. . . . . . . .
. .
Vv05g0009 Tc04_g028300 . Gorai.005G061000.1 .
. . Vv07g0406 Tc01_g030580 Gorai.
007G128200.1 Gorai.008G145200.1 . Gorai.004G090000.1
. . . . . . . .
Vv05g0010 Tc04_g028320 . . . . .
Vv07g0413 Tc01_g030610 Gorai.007G128000.1 .
. Gorai.004G090300.1 . Vv14g0219
Tc04_g028320 . Gorai.007G300700.1 Gorai.007G128000.1
. .
Vv05g0011 Tc04_g028330 . . . Gorai.
011G191400.1 . . . . . . .
. . . . . . . .
Vv05g0012 . Gorai.006G261600.1 . .
Gorai.011G191600.1 . . . . . .
. . . . . . . .
.

Vv05g0013 Tc04_g028350 . Gorai.005G061100.1 .
Gorai.011G191700.1 . . . . .
. . . . . . . .
. .
Vv05g0014 . . . . . . .
. . . . . . . .
. . . . .
Vv05g0015 . . . . . . .
. . . . . . . .
. . . . .
Vv05g0016 . . . . . . .
. . . . . . . .
. . . . .
Vv05g0017 . . . . . . .
. . . . . . . .
. . . . .
Vv05g0018 . . . . . . .
. . . . . . . .
. . . . .
Vv05g0019 . . . . . . .
. . . . . . . .
. . . . .
Vv05g0020 . . . . . . .
. . . . . . . .
. . . . .
Vv05g0021 . . . . . . .
. . . . . . . .
. . . . .
Vv05g0022 Tc04_g028360 . . Gorai.006G076600.1
Gorai.011G191800.1 . . . . .
. . . . . . . .
. .
Vv05g0023 Tc04_g028370 . . Gorai.006G076700.1
. . . . . . . .
. . . . . . . .
Vv05g0024 . . . . . . .
. . . . . . . .
. . . . .
Vv05g0025 Tc04_g028390 Gorai.006G261400.1 Gorai.
005G061300.1 . Gorai.011G192100.1 . . .
. . . . . . . .
. . . .
Vv05g0026 Tc04_g028400 . Gorai.005G061400.1 .
Gorai.011G192200.1 . . . . .
. . . . . . . .
. .
Vv05g0027 Tc04_g028410 Gorai.006G261300.1 . .
. . . . . . . .
. . . . . . . .
Vv05g0028 . . . . . .

Vv07g0428 Tc01_g030790 Gorai.007G127300.1 . .
Gorai.004G052100.1 Gorai.004G239000.1 . .
. . . . .
Vv05g0029 Tc04_g028420 . Gorai.005G061500.1 .
. . . . . . . .
. . . . . . . .
Vv05g0030 Tc04_g028430 . . . .
Gorai.002G028600.1 . . . . . .
. . . . . . . .
Vv05g0031 . . . . Gorai.011G192300.1
. . . . . . . .
Vv14g0209 . . . . . .
Vv05g0032 Tc04_g028440 . . . . .
Vv07g0429 Tc01_g030800 . . .
Gorai.004G091300.1 . . . . . .
. .
Vv05g0033 . . . . . . .
. . . . . . . .
. . . . .
Vv05g0034 Tc04_g028450 . . . . .
. . . . . . . .
. . . . . .
Vv05g0035 Tc04_g028460 Gorai.006G261200.1 Gorai.
005G061600.1 . . . . . . .
. . . . . . . .
. .
Vv05g0036 Tc04_g028470 Gorai.006G261100.1 . .
. Gorai.002G028700.1 . . . .
. . . . . . . .
. .
Vv05g0037 Tc04_g028480 . Gorai.005G061700.1 .
. . . . . . . .
. . . . . . . .
Vv05g0038 Tc04_g028490 Gorai.006G260900.1 . .
. . Vv07g0432 Tc01_g030870 . .
. . . . . . . .
. .
Vv05g0039 . . . . . . .
. . . . . . . .
. . . . .
Vv05g0040 Tc04_g028510 . Gorai.005G061800.1 .
. . . . . . . .
. . . . . . . .
Vv05g0041 Tc04_g028520 . . . . .
Vv07g0434 Tc01_g030890 . . . .
Gorai.004G142700.1 . . . . .
. .
Vv05g0042 . . . . . . .
. . . . . . . .
. . . . .

Vv05g0043 . . . . . . .
. . . . . . .
. . . . .
Vv05g0044 Tc04_g028570 . . Gorai.006G076800.1
. . . . . . .
. . . . . . .
Vv05g0045 Tc04_g028580 Gorai.006G260500.1 Gorai.
005G061900.1 . Gorai.011G193300.1 . Vv07g0435
Tc01_g030910 . . . . . .
. . . . . .
Vv05g0046 Tc04_g028590 Gorai.006G260400.1 . .
. . . . . . .
. . . . . . .
Vv05g0047 Tc04_g028600 . . . Gorai.
011G193400.1 . . . . . . .
. . . . . . .
Vv05g0048 . . . . . . .
. . . . . . .
. . . . .
Vv05g0049 Tc04_g028610 Gorai.006G260300.1 . .
Gorai.011G193600.1 . . . . .
. . . . . . .
. .
Vv05g0050 . . . . . . .
. . . . . . .
. . . . .
Vv05g0051 . . . . . . .
. . . . . . .
. . . . .
Vv05g0052 Tc04_g028630 Gorai.006G260200.1 Gorai.
005G062000.1 . Gorai.011G193800.1 . Vv07g0441
Tc01_g031050 Gorai.007G126400.1 . . .
. . . . . . .
Vv05g0053 Tc04_g028640 . Gorai.005G062100.1 .
Gorai.011G193900.1 Gorai.002G028800.1 . .
. . . . . . .
. . . .
Vv05g0054 Tc04_g028650 . Gorai.005G062200.1 .
Gorai.011G194000.1 . . . . .
. . . Vv14g0183 . .
Gorai.007G303500.1 . . .
Vv05g0055 . . . . . . .
. . . . . . .
. . . . .
Vv05g0056 Tc04_g028660 . . . .
Gorai.002G028900.1 . . . . . .
. . . . . . .
Vv05g0057 Tc04_g028670 . . Gorai.006G077000.1
. Gorai.002G029000.1 . . . .
. . . . . . .

.        .
Vv05g0058        Tc04_g028690     .        .        Gorai.006G077200.1
.        .        .        .        .        .        .        .
.        .        .        .        .        .        .        .
Vv05g0059        Tc04_g028710     Gorai.006G260100.1        .
Gorai.006G077400.1        Gorai.011G194200.1        .        Vv07g0448
.        .        .        .        .        .        .        .
.        .        .        .        .
Vv05g0060        .        .        .        .        .        .        .
.        .        .        .        .        .        .        .
.        .        .        .        .
Vv05g0061        .        .        .        .        .        .        .
.        .        .        .        .        .        .        .
.        .        .        .        .
Vv05g0062        .        .        .        .        .        .        .
.        .        .        .        .        .        .        .
.        .        .        .        .
Vv05g0063        Tc04_g028720     .        .        Gorai.006G077500.1
.        .        .        .        .        .        .        .
.        .        .        .        .        .        .        .
Vv05g0064        Tc04_g028730     .        Gorai.005G062300.1        .
Gorai.011G194300.1        .        .        .        .        .
.        .        .        .        .        .        .        .
.        .
Vv05g0065        .        .        Gorai.005G062500.1        .
Gorai.011G194900.1        .        .        .        .        .        .
.        .        .        .        .        .        .        .
.
Vv05g0066        Tc04_g028740     .        Gorai.005G062600.1        .
Gorai.011G196500.1        .        .        .        .        .
.        .        .        .        .        .        .        .
.        .
Vv05g0067        .        .        .        .        .        .        .
.        .        .        .        .        .        .        .
.        .        .        .        .
Vv05g0068        .        .        Gorai.005G062700.1        .
Gorai.011G196600.1        .        .        .        .        .        .
.        .        .        .        .        .        .        .
.
Vv05g0069        Tc04_g028750     .        Gorai.005G062800.1        .
Gorai.011G196800.1        .        Vv01g0538
Tc02_g028590     Gorai.002G177600.1        .        .        .        .
.        .        .        .        .        .        .
Vv05g0070        .        .        .        .        .        .        .
.        .        .        .        .        .        .        .
.        .        .        .        .
Vv05g0071        .        .        Gorai.005G063200.1        .
Gorai.011G196900.1        .        Vv01g0535        .        Gorai.
002G178000.1        .        .        .        .        .        .        .
.        .        .        .

Vv05g0072 Tc04_g028760 . . . . .
. . . . . . . .
. . . . . .
Vv05g0073 . . . . . . .
. . . . . . . .
. . . . .
Vv05g0074 . . Gorai.005G063300.1 .
Gorai.011G197000.1 . Vv01g0533 Tc02_g028610 .
. . . . . . . .
. . .
Vv05g0075 Tc04_g028780 . . . . .
. . . . . . . .
. . . . . .
Vv05g0076 . . Gorai.005G063400.1 .
Gorai.011G197200.1 . Vv01g0531 . .
Gorai.005G160700.1 . . . . . .
. . . .
Vv05g0077 . . . . . . .
. . . . . . . .
. . . . .
Vv05g0078 Tc04_g028790 . Gorai.005G063900.1 .
Gorai.011G197300.1 . Vv01g0529
Tc02_g028620 . . . . . . .
. . . . .
Vv05g0079 . . . . . . .
. . . . . . . .
. . . . .
Vv05g0080 Tc04_g028820 . . . . .
Vv01g0527 . . . . . .
. . . . . . .
Vv05g0081 . . . . . . .
. . . . . . . .
. . . . .
Vv05g0082 . . . . . . .
. . . . . . . .
. . . . .
Vv05g0083 . . . . . .
Vv01g0526 Tc02_g028660 . . . . .
. . . . . . .
Vv05g0084 Tc04_g028860 . Gorai.005G064100.1 .
. . . . . . . .
. . . . . . . .
Vv05g0085 Tc04_g028880 . Gorai.005G064300.1 .
. . . . . . . .
. . . . . . . .
Vv05g0086 . . . . . . .
. . . . . . . .
. . . . .
Vv05g0087 . . Gorai.005G064500.1 .
Gorai.011G197400.1 . Vv01g0525 Tc02_g027000 .

Gorai.005G160800.1 . . . . .
. . . . .
Vv05g0088 . . . . . . .
. . . . . . . .
. . . . .
Vv05g0089 Tc04_g028900 . Gorai.005G064700.1 .
Gorai.011G197500.1 . Vv01g0524 . .
. . . . . . . .
. . .
Vv05g0090 Tc04_g028940 . Gorai.005G064800.1 .
Gorai.011G197600.1 . . . . .
. . . . . . . .
. .
Vv05g0091 Tc04_g028960 Gorai.006G259900.1 .
Gorai.006G077900.1 . . Vv07g0454
Tc01_g031140 Gorai.007G126100.1 Gorai.008G053400.1 .
. Gorai.004G143000.1 . . . .
. . .
Vv05g0092 Tc04_g028970 Gorai.006G259800.1 Gorai.
005G065100.1 . . . Vv07g0456
Tc01_g031160 Gorai.007G126000.1 . . .
Gorai.004G237800.1 Vv14g0179 Tc04_g028970 . .
. . .
Vv05g0093 Tc04_g028990 Gorai.006G259700.1 . .
. Gorai.002G029200.1 . . . .
. . . . . . . .
. .
Vv05g0094 Tc04_g029000 . Gorai.005G065200.1 .
. . Vv07g0459 Tc01_g031190 Gorai.
007G125700.1 . . Gorai.004G063600.1 . .
. . . . . .
Vv05g0095 . . . . . . .
. . . . . . . .
. . . . .
Vv05g0096 Tc04_g029020 . Gorai.005G065300.1 .
. Gorai.002G029400.1 Vv07g0465
Tc01_g031290 . . . . . Vv14g0172
Tc04_g029020 . . . . .
Vv05g0097 Tc04_g029030 . Gorai.005G065400.1 .
. Gorai.002G029800.1 . . . .
. . . Vv14g0171 Tc04_g029030
Gorai.013G128000.1 . Gorai.007G124900.1 . .
Vv05g0098 Tc04_g029040 . . . . .
. . . . . . . .
. . . . . .
Vv05g0099 . . . . . .
Vv07g0471 . . . . . . .
. . . . . .
Vv05g0100 Tc04_g029070 . . Gorai.006G078000.1
. . . . . . . .

.	.	.	.	.	.	.	.
Vv05g0101	Tc04_g029090	.	.	.	.	.
.	.	.	.	.	.
Vv14g0159	Tc04_g029090	Gorai.013G128700.1	.	.
.	.
Vv05g0102	.	.	.	Gorai.006G078400.1	.
.	.	.	.	.	.	.	.
.	.	.	.	.	.	.
Vv05g0103	Tc04_g029110	.	.	.	.	.
.	.	.	.	.	.	.	.
.	.	.	.	.	.
Vv05g0104	Tc04_g029120	.	.	Gorai.006G078500.1
.	.	.	.	.	.	.	.
.	.	.	.	.	.	.	.
Vv05g0105	Tc04_g029130	.	.	.	.	.
.	.	.	.	.	.	.	.
.	.	.	.	.	.
Vv05g0106	Tc04_g029140	.	.	Gorai.006G078600.1
.	.	.	.	.	.	.	.
.	.	.	.	.	.	.	.
Vv05g0107	.	.	.	Gorai.006G078700.1	.
.	.	.	.	.	.	.	.
.	.	.	.	.	.	.
Vv05g0108	Tc04_g029150	.	.	.	Gorai.
011G198100.1	.	.	.	.	.	.	.
.	.	.	.	.	.	.	.
Vv05g0109	Tc04_g029160	Gorai.006G259500.1	.	.
Gorai.011G198200.1	.	Vv07g0492
Tc01_g031530	.	Gorai.008G140600.1	.	.	.
.	.	.	.	.	.	.
Vv05g0110	.	.	Gorai.005G065500.1	Gorai.
006G078800.1	.	.	.	.	.	.	.
.	.	.	.	.	.	.	.
.
Vv05g0111	.	.	.	.	.	.	.
.	.	.	.	.	.	.	.
.	.	.	.	.
Vv05g0112	Tc04_g029170	.	Gorai.005G065600.1	.
Gorai.011G198400.1	.	.	.	.	.
.	.	.	.	.	.	.	.
.	.
Vv05g0113	Tc04_g029180	.	.	Gorai.006G078900.1
Gorai.011G198500.1	.	.	.	.	.
.	.	.	.	.	.	.	.
.	.
Vv05g0114	.	.	.	.	.	.	.
.	.	.	.	.	.	.	.
.	.	.	.	.
Vv05g0115	Tc04_g029190	.	Gorai.005G065700.1
Gorai.006G079000.1	Gorai.011G198600.1	.	.	.

. . . . . . . .
. . . .
Vv05g0116 Tc04_g029210 . Gorai.005G065800.1 .
Gorai.011G198700.1 . . . . .
. . . Vv14g0148 Tc04_g029200
Gorai.013G129100.1 . . . .
Vv05g0117 Tc04_g029220 Gorai.006G259400.1 Gorai.
005G066000.1 . Gorai.011G198800.1 . Vv07g0496
Tc01_g031570 . . . . . .
. . . . . .
Vv05g0118 . . . . . . .
. . . . . . . .
. . . . .
Vv05g0119 Tc04_g029230 Gorai.006G259300.1 .
Gorai.006G079100.1 . . . . . .
. . . Vv14g0147 Tc04_g029230 .
. . . .
Vv05g0120 Tc04_g029240 Gorai.006G259200.1 Gorai.
005G066100.1 . Gorai.011G199200.1 . . .
. . . . . . . .
. . . .
Vv05g0121 Tc04_g029250 Gorai.006G259100.1 . .
Gorai.011G199300.1 . . . . .
. . . . . . . .
. .
Vv05g0122 . . . Gorai.006G079200.1
Gorai.011G199400.1 . . . . . .
. . . . . . . .
.
Vv05g0123 Tc04_g029280 . . . .
Gorai.002G030000.1 . . . . . .
. . . . . . . .
Vv05g0124 Tc04_g029290 . . Gorai.006G079300.1
. . . . . . . .
. . . . . . . .
Vv05g0125 Tc04_g029300 . . Gorai.006G079400.1
. . . . . . . .
. . . . . . . .
Vv05g0126 Tc04_g029350 . . Gorai.006G079800.1
. . Vv07g0509 Tc01_g031670 Gorai.
007G122800.1 Gorai.008G139700.1 . Gorai.004G050400.1
. Vv14g0131 Tc04_g029350 . .
Gorai.007G122800.1 . .
Vv05g0127 Tc04_g029360 Gorai.006G258900.1 . .
. . . . . . . .
. . . . . . . .
Vv05g0128 Tc04_g029390 Gorai.006G258800.1 Gorai.
005G066300.1 . . . Vv07g0514
Tc01_g031760 . Gorai.008G139000.1 . . .
. . . . . . .

Vv05g0129 Tc04_g029400 Gorai.006G258700.1 . .
Gorai.011G199600.1 . Vv07g0515 . .
. . . . . . . .
. . .
Vv05g0130 . . . . . . .
. . . . . . . .
. . . . .
Vv05g0131 Tc04_g029410 . Gorai.005G066400.1 .
. . Vv07g0522 Tc01_g031800 . .
. . . Vv14g0122 Tc04_g029410 .
Gorai.007G306800.1 . . .
Vv05g0132 Tc04_g029430 . Gorai.005G066600.1
Gorai.006G079900.1 . . . . . .
. . . . . . . .
. .
Vv05g0133 . . . . . . .
. . . . . . . .
. . . . .
Vv05g0134 Tc04_g029450 . Gorai.005G066700.1 .
. . . . . . . .
. . . . . . . .
Vv05g0135 Tc04_g029460 Gorai.006G258500.1 . .
. . . . . . . .
. . . . . . . .
Vv05g0136 Tc04_g029470 . Gorai.005G066800.1 .
. . . . . . . .
. . . . . . . .
Vv05g0137 . . . . . . .
. . . . . . . .
. . . . .
Vv05g0138 Tc04_g029490 . Gorai.005G066900.1 .
Gorai.011G199700.1 . . . . .
. . . . . . . .
. .
Vv05g0139 Tc04_g029500 . . . Gorai.
011G199800.1 . . . . . . .
. . . . . . . .
Vv05g0140 . . . . . . .
. . . . . . . .
. . . . .
Vv05g0141 Tc04_g029540 . Gorai.005G067100.1 .
. . . . . . . .
. . . . . . . .
Vv05g0142 Tc04_g029550 . . . .
Gorai.002G030100.1 . . . . . .
. . . . . . . .
Vv05g0143 Tc04_g029570 . . . Gorai.
011G200100.1 Gorai.002G030200.1 . . . .
. . . . . . . .
. .

Vv05g0144	Tc04_g029580	.	.	.	Gorai.
011G200200.1	.	.	.	.	.	.	.
	.	.	.	.	.	.	.	.
Vv05g0145	.	.	.	.	.	.	.
	.	.	.	.	.	.	.	.
	.	.	.	.	.
Vv05g0146	Tc04_g029590	Gorai.006G258400.1	Gorai.
005G067400.1	Gorai.006G080100.1	Gorai.011G200300.1	.
	.	.	.	.	.	.	.	.
	.	.	.	.	.	.
Vv05g0147	Tc04_g029600	.	Gorai.005G067500.1	.
	.	Gorai.002G030300.1	.	.	.	.
	.	.	.	.	.	.	.	.
	.	.
Vv05g0148	Tc04_g029610	.	.	.	.
Gorai.002G030400.1	.	.	.	.	.	.
	.	Vv14g0115	Tc04_g029610	.	.	.
	.	Gorai.002G030400.1
Vv05g0149	.	.	.	.	.	.	.
	.	.	.	.	.	.	.	.
	.	.	.	.	.
Vv05g0150	Tc04_g029620	.	.	.	Gorai.
011G200600.1	.	.	.	.	.	.	.
	.	.	.	.	.	.	.	.
Vv05g0151	Tc04_g029640	Gorai.006G258100.1	.	.
	Gorai.011G200700.1	.	.	.	.	.
	.	.	.	.	.	.	.	.
	.	.
Vv05g0152	Tc04_g029650	.	.	.	.
Gorai.002G030500.1	.	.	.	.	.	.
	.	.	.	.	.	.	.	.
Vv05g0153	Tc04_g029660	Gorai.006G258000.1	.	.
	.	.	.	.	.	.	.	.
	.	.	.	.	.	.	.	.
Vv05g0154	.	.	.	.	.	.
Vv07g0533	Tc01_g031940	.	Gorai.008G138000.1	.
	.	.	.	.	.	.	.	.
	.
Vv05g0155	Tc04_g029680	Gorai.006G263300.1	Gorai.
005G067600.1	.	Gorai.011G200800.1	.	.	.
	.	.	.	.	.	.	.	.
	.	.	.	.
Vv05g0156	Tc04_g029690	.	.	.	Gorai.
011G200900.1	.	.	.	.	.	.	.
	.	.	.	.	.	.	.	.
Vv05g0157	.	.	.	.	.	.	.
	.	.	.	.	.	.	.	.
	.	.	.	.	.
Vv05g0158	Tc04_g029700	Gorai.006G257800.1	.	.
	Gorai.011G201000.1	.	.	.	.	.

. . . . . . . .
. .
Vv05g0159 Tc04_g029710 . . . Gorai.
011G201500.1 . . . . . . .
. . . . . . . .
Vv05g0160 . . . . . . .
. . . . . . . .
. . . . .
Vv05g0161 . . . . . . .
. . . . . . . .
. . . . .
Vv05g0162 . . . . . . .
. . . . . . . .
. . . . .
Vv05g0163 Tc04_g029780 Gorai.006G265000.1 Gorai.
005G067900.1 . Gorai.011G201700.1 Gorai.002G030700.1
. . . . . . .
Vv14g0112 Tc04_g029780 . . . . .
Vv05g0164 Tc04_g029790 . . . .
Gorai.002G030800.1 . . . . . .
. . . . . . . .
Vv05g0165 Tc04_g029800 Gorai.006G265200.1 Gorai.
005G068100.1 . Gorai.011G201800.1 . Vv07g0536
Tc01_g031960 Gorai.007G121200.1 Gorai.008G137900.1
. Gorai.004G069500.1 Gorai.004G144900.1 .
. . . . . .
Vv05g0166 Tc04_g029820 . Gorai.005G068200.1 .
. . . . . . . .
. . . . . . . .
Vv05g0167 . . . . . . .
. . . . . . . .
. . . . .
Vv05g0168 Tc04_g029830 . . . Gorai.
011G201900.1 . . . . . . .
. . . . . . . .
Vv05g0169 . . . . . . .
. . . . . . . .
. . . . .
Vv05g0170 Tc04_g029850 Gorai.006G265300.1 . .
. Gorai.002G030900.1 . . . .
. . . . . . . .
. .
Vv05g0171 Tc04_g029860 Gorai.006G265400.1 .
Gorai.006G080200.1 Gorai.011G202000.1 Gorai.002G031000.1
Vv07g0540 Tc01_g032010 . . . .
. Vv14g0111 Tc04_g029860 . . .
Gorai.011G202000.1 .
Vv05g0172 Tc04_g029890 . . . Gorai.
011G202200.1 Gorai.002G031100.1 . . . .
. . . . . . . .

. .
Vv05g0173 . . . . . . .
. . . . . . . .
. . . . .
Vv05g0174 Tc04_g029910 . Gorai.005G068400.1 .
. . . . . . . .
. . . . . . . .
Vv05g0175 Tc04_g029920 . . Gorai.006G080300.1
. . . . . . . .
. . . . . . . .
Vv05g0176 . . . . . . .
. . . . . . . .
. . . . .
Vv05g0177 Tc04_g029950 . . Gorai.006G080500.1
Gorai.011G202400.1 . . . . .
. . . . . . . .
. .
Vv05g0178 . . . . . . .
. . . . . . . .
. . . . .
Vv05g0179 Tc04_g029980 Gorai.006G265500.1 . .
. . . . . . . .
. . . . . . . .
Vv05g0180 . . . . . . .
. . . . . . . .
. . . . .
Vv05g0181 Tc04_g029990 . . . Gorai.
011G202600.1 . . . . . . .
. . . . . . . .
Vv05g0182 . . . . Gorai.011G202700.1
. . . . . . . .
. . . . . . .
Vv05g0183 Tc04_g030000 . . . Gorai.
011G202800.1 Gorai.002G031500.1 . . . .
. . . . . . . .
. .
Vv05g0184 Tc04_g030030 . . Gorai.006G080600.1
. . . . . . . .
. . . . . . . .
Vv05g0185 Tc04_g030040 . . Gorai.006G080700.1
. . . . . . . .
. . . . . . . .
Vv05g0186 . . . . . . .
. . . . . . . .
. . . . .
Vv05g0187 Tc04_g030060 . . . . .
. . . . . . . .
. . . . . .
Vv05g0188 Tc04_g030070 . . . Gorai.
011G203600.1 . . . . . . .

. Vv14g0099 Tc04_g030070 Gorai.013G130600.1
Gorai.007G308100.1 Gorai.013G208200.1 . .
Vv05g0189 Tc04_g030080 . Gorai.005G068900.1 .
Gorai.011G203900.1 . . . . .
. . . . . . . .
. .
Vv05g0190 Tc04_g030090 . . . Gorai.
011G204200.1 . . . . . . .
. . . . . . . .
Vv05g0191 . . Gorai.005G069100.1 . .
. . . . . . . .
. . . . . . .
Vv05g0192 Tc04_g030110 . Gorai.005G069200.1 .
. . . . . . . .
. . . . . . . .
Vv05g0193 Tc04_g030120 Gorai.006G265600.1 . .
. . Vv07g0563 . Gorai.007G120900.1
. . . . Vv14g0094 . .
. . . .
Vv05g0194 . . . . . . .
. . . . . . . .
. . . . .
Vv05g0195 Tc04_g030130 . . . . .
. . . . . . . .
. . . . . .
Vv05g0196 . . . . . . .
. . . . . . . .
. . . . .
Vv05g0197 . . . . . . .
. . . . . . . .
. . . . .
Vv05g0198 . . . . . . .
. . . . . . . .
. . . . .
Vv05g0199 . . . . . . .
. . . . . . . .
. . . . .
Vv05g0200 . . . . . . .
. . . . . . . .
. . . . .
Vv05g0201 . . . . . . .
. . . . . . . .
. . . . .
Vv05g0202 . . . . . . .
. . . . . . . .
. . . . .
Vv05g0203 . Gorai.006G257900.1 . . .
. . . . . . . .
. . . . . . .
Vv05g0204 . . . . . . .

| | | | | | | | | |
|---|---|---|---|---|---|---|---|---|
| | . | . | . | . | . | . | . | . |
| Vv05g0205 | . | . | . | . | . | . | . | . |
| Vv05g0206 | . | . | . | . | . | . | . | . |
| Vv05g0207 | . | . | . | . | . | . | . | . |
| Vv05g0208 | . | . | . | . | . | . | . | . |
| Vv05g0209 | . | . | . | . | . | . | . | . |
| Vv05g0210 | . | . | . | . | . | . | . | . |
| Vv05g0211 | . | . | . | . | . | . | . | . |
| Vv05g0212 | . | . | . | . | . | . | . | . |
| Vv05g0213 | . | . | . | . | . | . | . | . |
| Vv05g0214 | . | . | . | . | . | . | . | . |
| Vv05g0215 | . | . | . | . | . | . | . | . |
| Vv05g0216 | . | . | . | . | . | . | . | . |
| Vv05g0217 | . | . | . | . | . | . | . | . |
| Vv05g0218 | . | . | . | . | . | . | . | . |
| Vv05g0219 | . | . | . | . | . | . | . | . |
| Vv05g0220 | . | . | . | . | . | . | . | . |
| | . | . | . | . | . | | | |

Vv05g0221 . . . . . . .
. . . . . . . .
. . . . .
Vv05g0222 . . . . . . .
. . . . . . . .
. . . . .
Vv05g0223 . . . . . . .
. . . . . . . .
. . . . .
Vv05g0224 . . . . . . .
. . . . . . . .
. . . . .
Vv05g0225 . . . . . . .
. . . . . . . .
. . . . .
Vv05g0226 . . . . . . .
. . . . . . . .
. . . . .
Vv05g0227 . Gorai.006G265700.1 . . .
. . . . . . . .
. . . . . . .
Vv05g0228 . . . . . .
Vv07g0565 Tc01_g032360 . . . . .
Vv14g0082 Tc04_g030130 Gorai.013G130900.1 .
Gorai.007G120900.1 . .
Vv05g0229 . . . . . . .
. . . . . . . .
. . . . .
Vv05g0230 Tc04_g030140 . Gorai.005G069300.1 .
. . . . . . . .
. . . . . . . .
Vv05g0231 Tc04_g030150 Gorai.006G259300.1 Gorai.
005G069400.1 . . . . . . .
. . . . . . . .
. .
Vv05g0232 . . . . . . .
. . . . . . . .
. . . . .
Vv05g0233 Tc04_g030170 . Gorai.005G069600.1 .
. . . . . . . .
. . . . . . . .
Vv05g0234 Tc04_g030190 . Gorai.005G069700.1 .
Gorai.011G204400.1 . . . . .
. . . . . . . .
. .
Vv05g0235 . . . . . . .
. . . . . . . .
. . . . .
Vv05g0236 . . . . . . .
. . . . . . . .

. . . . .
Vv05g0237 Tc04_g030230 Gorai.006G266100.1 Gorai.
005G069900.1 . . . Vv07g0520 . .
. . Gorai.004G066400.1 . Vv14g0072
Tc04_g030230 . . . . .
Vv05g0238 Tc04_g030240 . Gorai.005G070100.1 .
. . . . . . . .
. . . . . . . .
Vv05g0239 Tc04_g030250 Gorai.006G266200.1 Gorai.
005G070200.1 . . . . . . .
. . . . . . . .
. .
Vv05g0240 Tc04_g030260 . Gorai.005G070400.1 .
. . . . . . . .
. . . . . . . .
Vv05g0241 . . . . . . .
. . . . . . . .
. . . . .
Vv05g0242 Tc04_g030270 . Gorai.005G070500.1 .
. . . . . . . .
. . . . . . . .
Vv05g0243 Tc04_g030280 . Gorai.005G070600.1 .
. . . . . . . .
. . . . . . . .
Vv05g0244 . . . . . . .
. . . . . . . .
. . . . .
Vv05g0245 . . . . . . .
. . . . . . . .
. . . . .
Vv05g0246 . . . . . . .
. . . . . . . .
. . . . .
Vv05g0247 . Gorai.006G259500.1 . . .
. . . . . . . .
. . . . . . .
Vv05g0248 . . . . . . .
. . . . . . . .
. . . . .
Vv05g0249 . . . . . . .
. . . . . . . .
. . . . .
Vv05g0250 . . . . . . .
. . . . . . . .
. . . . .
Vv05g0251 . . . . . .
Vv07g0492 Tc01_g031530 . Gorai.008G140600.1 .
. . . . . . . .
.
Vv05g0252 . Gorai.006G266400.1 . . .

.	.	.	.	.	.	.	.
.	.	.	.	.	.	.
Vv05g0253	.	.	.	.	.	.	.
.	.	.	.	.	.	.	.
.	.	.	.	.
Vv05g0254	.	.	.	.	.	.	.
.	.	.	.	.	.	.	.
.	.	.	.	.
Vv05g0255	.	.	.	.	.	.	.
.	.	.	.	.	.	.	.
.	.	.	.	.
Vv05g0256	.	.	.	.	.	.	.
.	.	.	.	.	.	.	.
.	.	.	.	.
Vv05g0257	Tc04_g030330	.	Gorai.005G070900.1	.
.	.	.	.	.	.	.	.
.	.	.	.	.	.	.	.
Vv05g0258	.	.	.	.	Gorai.011G204600.1
.	.	.	.	.	.	.	.
.	.	.	.	.	.	.
Vv05g0259	Tc04_g030340	.	.	.	.	.
.	.	.	.	.	.	.	.
.	.	.	.	.	.
Vv05g0260	Tc04_g030350	Gorai.006G266300.1	.	.
.	.	.	.	.	.	.	.
.	.	.	.	.	.	.	.
Vv05g0261	Tc04_g030360	.	.	.	.	.
Vv07g0452	Tc01_g031120	.	.	.	.
.	.	.	.	.	.	.	.
Vv05g0262	Tc04_g030370	.	.	.	Gorai.
011G204700.1	.	.	.	.	.	.	.
.	.	.	.	.	.	.	.
Vv05g0263	Tc04_g030380	.	Gorai.005G071000.1	.
.	.	.	.	.	.	.	.
.	.	.	.	.	.	.	.
Vv05g0264	.	.	.	.	.	.	.
.	.	.	.	.	.	.	.
.	.	.	.	.
Vv05g0265	.	.	.	.	.	.	.
.	.	.	.	.	.	.	.
.	.	.	.	.
Vv05g0266	Tc04_g030460	.	Gorai.005G071300.1	.
.	.	.	.	.	.	.	.
.	Vv14g0055	Tc04_g030460	.	.
Gorai.007G119200.1	.	.
Vv05g0267	Tc04_g030470	.	.	.	Gorai.
011G204900.1	.	.	.	.	.	.	.
.	.	.	.	.	.	.	.
Vv05g0268	Tc04_g030480	.	.	.	Gorai.
011G205000.1	.	.	.	.	.	.	.

. . . . . . . .
Vv05g0269 Tc04_g030490 . . . Gorai.
011G205100.1 . . . . . . .
. . . . . . . .
Vv05g0270 Tc04_g030500 . Gorai.005G071400.1 .
. . . . . . . .
. . . . . . . .
Vv05g0271 Tc04_g030510 . Gorai.005G071500.1 .
. . . . . . . .
. . . . . . . .
Vv05g0272 Tc04_g030520 . Gorai.005G071600.1 .
. . . . . . . .
. . . . . . . .
Vv05g0273 Tc04_g030540 . . . . .
. . . . . . . .
. . . . . .
Vv05g0274 Tc04_g030550 . Gorai.005G071800.1 .
. . . . . . . .
. . . . . . . .
Vv05g0275 Tc04_g030560 . . . Gorai.
011G205200.1 . . . . . . .
. Vv14g0047 Tc04_g030560 . . .
. Gorai.002G032100.1
Vv05g0276 Tc04_g030570 . Gorai.005G071900.1 .
. . . . . . . .
. . . . . . . .
Vv05g0277 Tc04_g030580 . . . . .
. . . . . . . .
. . . . . .
Vv05g0278 Tc04_g030600 . . . . .
. . . . . . .
Vv14g0044 Tc04_g030600 . . . . .
Vv05g0279 Tc04_g030620 . Gorai.005G072600.1 .
. . . . . . . .
. . . . . . . .
Vv05g0280 Tc04_g030630 . . . Gorai.
011G205600.1 . Vv07g0437 Tc01_g030930 . .
. Gorai.004G051900.1 . . . .
. . . .
Vv05g0281 Tc04_g030640 . . . . .
. . . . . . . .
. . . . . .
Vv05g0282 . . . . . . .
. . . . . . . .
. . . . .
Vv05g0283 Tc04_g030660 . Gorai.005G073000.1 .
. . . . . . . .
. . . . . . . .
Vv05g0284 Tc04_g030670 . . . . .
. . . . . . . .

| | | | | | | | |
|---|---|---|---|---|---|---|---|
| . | . | . | . | . | . | | |
| Vv05g0285 | Tc04_g030700 | | . | Gorai.005G073300.1 | | | . |
| . | . | . | . | . | . | . | . |
| . | . | . | . | . | . | . | . |
| Vv05g0286 | . | . | . | . | . | . | . |
| . | . | . | . | . | . | . | . |
| . | . | . | . | . | | | |
| Vv05g0287 | . | . | . | . | . | . | . |
| . | . | . | . | . | . | . | . |
| . | . | . | . | . | | | |
| Vv05g0288 | . | . | . | . | . | . | . |
| . | . | . | . | . | . | . | . |
| . | . | . | . | . | | | |
| Vv05g0289 | Tc04_g030720 | | . | Gorai.005G073400.1 | | | . |
| . | . | . | . | . | . | . | . |
| . | . | . | . | . | . | . | . |
| Vv05g0290 | . | . | . | . | . | . | . |
| . | . | . | . | . | . | . | . |
| . | . | . | . | . | | | |
| Vv05g0291 | . | Gorai.006G264900.1 | | | . | . | . |
| . | . | . | . | . | . | . | . |
| . | . | . | . | . | . | . | |
| Vv05g0292 | Tc04_g030740 | | . | Gorai.005G073900.1 | | | . |
| . | . | . | . | . | . | . | . |
| . | Vv14g0040 | | . | . | . | . | . |
| . | | | | | | | |
| Vv05g0293 | . | . | . | . | . | . | . |
| . | . | . | . | . | . | . | . |
| . | . | . | . | . | | | |
| Vv05g0294 | Tc04_g030750 | | . | Gorai.005G074000.1 | | | . |
| . | . | . | . | . | . | . | . |
| . | . | . | . | . | . | . | . |
| Vv05g0295 | . | . | . | . | . | . | . |
| . | . | . | . | . | . | . | . |
| . | . | . | . | . | | | |
| Vv05g0296 | Tc04_g030770 | | . | . | . | . | . |
| . | . | . | . | . | . | . | . |
| . | . | . | . | . | . | | |
| Vv05g0297 | . | . | . | . | . | . | . |
| . | . | . | . | . | . | Vv14g0034 | |
| . | . | . | Gorai.013G210200.1 | | | . | . |
| Vv05g0298 | . | . | . | . | . | . | . |
| . | . | . | . | . | . | . | . |
| . | . | . | . | . | | | |
| Vv05g0299 | . | . | . | . | . | . | . |
| . | . | . | . | . | . | . | . |
| . | . | . | . | . | | | |
| Vv05g0300 | Tc04_g024600 | | . | . | . | . | . |
| . | . | . | . | . | . | . | . |
| . | . | . | . | . | . | | |

Vv05g0301 . . . . . . .
. . . . . . . .
. . . . .
Vv05g0302 . . . . . . .
. . . . . . . .
. . . . .
Vv05g0303 Tc04_g029100 . . . . .
. . . . . . . .
. . . . . .
Vv05g0304 . . . . . . .
. . . . . . Vv14g0027
Tc05_g004610 Gorai.013G131600.1 . Gorai.
013G211000.1 . .
Vv05g0305 . . . . . . .
. . . . . . . .
. . . . .
Vv05g0306 . . . . . . .
. . . . . . . .
. . . . .
Vv05g0307 . . . . . . .
. . . . . . . .
. . . . .
Vv05g0308 . . . . . . .
. . . . . . . .
. . . . .
Vv05g0309 . . . . . . .
. . . . . . . .
. . . . .
Vv05g0310 . . . . Gorai.011G191000.1
. Vv07g0400 . . . . .
Gorai.004G142300.1 . . . . .
. .
Vv05g0311 . . Gorai.005G060700.1 . .
. . . . . . . .
. . . . . . .
Vv05g0312 . . . . . . .
. . . . . . . .
. . . . .
Vv05g0313 Tc04_g001060 . . . . .
Vv07g0386 Tc01_g030440 . . .
Gorai.004G089400.1 . . . . . .
. .
Vv05g0314 . . . . . . .
. . . . . . . .
. . . . .
Vv05g0315 . . . . . . .
. . . . . . . .
. . . . .
Vv05g0316 . . . . Gorai.011G190600.1
Gorai.002G028400.1 . . . . .

. . . . . . . .
.
Vv05g0317 . . . . . . .
. . . . . . . .
. . . . .
Vv05g0318 . . Gorai.005G060600.1 . .
. . . . . . . .
. . . . . . .
Vv05g0319 Tc04_g024850 . . . . .
. . . . . . . .
. . . . . .
Vv05g0320 . . . . Gorai.011G190500.1
. Vv07g0371 . . . . .
. . . . . . . .
Vv05g0321 Tc04_g025170 . . . . .
. . . . . . . .
. . . . . .
Vv05g0322 . . . . . Gorai.
002G027800.1 . . . . . . .
. . . . . . .
Vv05g0323 . Gorai.006G263900.1 . . .
. . . . . . . .
. . . . . . .
Vv05g0324 . . . . . . .
. . . . . . . .
. . . . .
Vv05g0325 . . . . . . .
. . . . . . . .
. . . . .
Vv05g0326 Tc04_g005400 . . . . .
. . . . . . . .
. . . . . .
Vv05g0327 Tc04_g001070 . . . . .
. . . . . . . .
. . . . . .
Vv05g0328 . Gorai.006G263800.1 . . .
. . . . . . . .
. . . . . . .
Vv05g0329 . . . . . Gorai.
002G028000.1 . . . . . . .
. . . . . . .
Vv05g0330 . Gorai.006G263600.1 Gorai.005G059800.1
. . Gorai.002G028100.1 . . .
. . . . . . . .
. . .
Vv05g0331 . Gorai.006G263500.1 . . .
Gorai.002G028200.1 . . . . . .
. . Vv14g0008 . . . .
. .
Vv05g0332 . Gorai.006G263400.1 . .

Gorai.011G189600.1 . . . . . .
. . . . . . . .
.
Vv05g0333 . . . . . . .
. . . . . . . .
. . . . .
Vv05g0334 Tc04_g028650 Gorai.006G263300.1 . .
. . . . . . . .
. . . . . . . .
Vv05g0335 . Gorai.006G263200.1 . . .
. . . . . . . .
. . . . . . .
Vv05g0336 . . . . . . .
. . . . . . . .
. . . . .
Vv05g0337 . . . . . Gorai.
002G028300.1 Vv07g0364 Tc01_g030240 Gorai.007G129000.1
. . Gorai.004G052800.1 Gorai.004G142100.1
. . . . . . .
Vv05g0338 . . . . . . .
. . . . . . . .
. . . . .
Vv05g0339 . . . . . . .
. . . . . . . .
. . . . .
Vv05g0340 . . . . . . .
. . . . . . . .
. . . . .
Vv05g0341 . . . . . . .
. . . . . . . .
. . . . .
Vv05g0342 . Gorai.006G262700.1 . Gorai.
006G076000.1 . . . . . . .
. . . . . . . .
.
Vv05g0343 . Gorai.006G259700.1 . . .
. . . . . . . .
. . . . . . .
Vv05g0344 . . . . . . .
. . . . . . . .
. . . . .
Vv05g0345 Tc04_g005020 . . . . .
. . . . . . . .
. . . . . .
Vv05g0346 . . . . . . .
. . . . . . . .
. . . . .
Vv05g0347 . . Gorai.005G059300.1 .
Gorai.011G188700.1 . . . . . .
. . . . . . . .

.
Vv05g0348 . . . . . . .
. . . . . . . .
. . . . .
Vv05g0349 . . Gorai.005G059200.1 .
Gorai.011G188600.1 . . . . . .
. . . . . . . .
.
Vv05g0350 . . . . . .
Vv07g0337 . . . . . . .
. . . . .
Vv05g0351 . . Gorai.005G059100.1 . .
. . . . . . . .
. . . . . . .
Vv05g0352 . . . . . . .
. . . . . . . .
. . . . .
Vv05g0353 . . Gorai.005G058900.1 . .
. . . . . . . .
. . . . . . .
Vv05g0354 Tc04_g019190 . . . . .
. . . . . . . .
. . . . . .
Vv05g0355 . . Gorai.005G058800.1 .
Gorai.011G188400.1 Gorai.002G027000.1 Vv07g0312
Tc01_g029810 Gorai.007G130500.1 . . Gorai.
004G053200.1 . . . . . . .
.
Vv05g0356 Tc04_g025400 . Gorai.005G058700.1 .
Gorai.011G188200.1 . . . . .
. . . . . . . .
. .
Vv05g0357 . . . . . . .
. . . . . . . .
. . . . .
Vv05g0358 . . . . Gorai.011G188100.1
. . . . . . . .
. . . . . . .
Vv05g0359 . . . . . . .
. . . . . . . .
. . . . .
Vv05g0360 Tc04_g019130 . . . . .
. . . . . . . .
. . . . . .
Vv05g0361 Tc04_g025660 . . . Gorai.
011G187900.1 . . . . . . .
. . . . . . . .
Vv05g0362 . . . . Gorai.011G187800.1
. . . . . . . .
. . . . . . .

Vv05g0363 . . . . Gorai.011G187600.1
. . . . . . . .
. . . . . . .
Vv05g0364 . . . . Gorai.011G187500.1
. . . . . . . .
Vv14g0265 . Gorai.013G125100.1 Gorai.
007G298800.1 Gorai.013G205300.1 . .
Vv05g0365 . . Gorai.005G058600.1 .
Gorai.011G187300.1 . Vv07g0307 Tc01_g029730
Gorai.007G130800.1 . . Gorai.004G086400.1
Gorai.004G140900.1 . . . . . .
.
Vv05g0366 Tc04_g001350 . Gorai.005G058500.1 .
Gorai.011G187200.1 . Vv07g0306
Tc01_g029720 Gorai.007G130900.1 . . .
Gorai.004G140800.1 . . . . . .
.
Vv05g0367 Tc04_g004590 . . . . .
. . . . . . . .
. . . . . .
Vv05g0368 . . Gorai.005G058400.1 .
Gorai.011G187000.1 . . . . . .
. . Vv14g0285 . . . .
. .
Vv05g0369 Tc04_g018910 . Gorai.005G057700.1
Gorai.006G075600.1 . . . . . .
. . . . . . . .
. .
Vv05g0370 . . Gorai.005G057600.1 .
Gorai.011G186700.1 . Vv07g0303 Tc01_g029700 .
. . Gorai.004G086200.1 . . .
. . . . .
Vv05g0371 . Gorai.006G257600.1 . . .
. . . . . . . .
. . . . . . .
Vv05g0372 . . Gorai.005G057500.1 .
Gorai.011G186300.1 . . . . . .
. . . . . . . .
.
Vv05g0373 . Gorai.006G257500.1 Gorai.005G057400.1
. . . Vv07g0301 Tc01_g029680
Gorai.007G131100.1 . . . . . .
. . . . .
Vv05g0374 . . . . Gorai.011G186200.1
. . . . . . . .
. . . . . . .
Vv05g0375 . . . . . . .
. . . . . . . .
. . . . .
Vv05g0376 . Gorai.006G257400.1 . .

Gorai.011G186100.1 . . . . . .
. . . . . . . .
.
Vv05g0377 . . . . . . .
. . . . . . . .
. . . . .
Vv05g0378 . . . . . . .
. . . . . . . .
. . . . .
Vv05g0379 . . . . . . .
. . . . . . . .
. . . . .
Vv05g0380 . . . . . . .
. . . . . . . .
. . . . .
Vv05g0381 . . . . Gorai.011G185900.1
. . . . . . . .
. . . . . . .
Vv05g0382 . . . . . . .
. . . . . . . .
. . . . .
Vv05g0383 . . . . . . .
. . . . . . . .
. . . . .
Vv05g0384 . . Gorai.005G057300.1 .
Gorai.011G185700.1 . . . . . .
. . . . . . . .
.
Vv05g0385 . . Gorai.005G057200.1 Gorai.
006G075400.1 Gorai.011G185600.1 . Vv07g0295
Tc01_g029580 Gorai.007G131500.1 . . Gorai.
004G085800.1 . . . . . . .
.
Vv05g0386 . . . . . . .
. . . . . . . .
. . . . .
Vv05g0387 . . . Gorai.006G075300.1 .
. . . . . . . .
. . . . . . .
Vv05g0388 . . . . . . .
. . . . . . . .
. . . . .
Vv05g0389 . Gorai.006G257200.1 Gorai.005G057100.1
Gorai.006G075200.1 . . Vv07g0294
Tc01_g029560 . . . . . . .
. . . . .
Vv05g0390 . . . . Gorai.011G185500.1
. . . . . . . .
. . . . . . .
Vv05g0391 . . . . . . .

. . . . . . . .
. . . . .
Vv05g0392 Tc04_g024980 . . . . .
. . . . . . . .
. . . . . .
Vv05g0393 Tc04_g025070 Gorai.006G257000.1 . .
Gorai.011G166100.1 . . . . .
. . . . . . . .
. .
Vv05g0394 . . . Gorai.006G075100.1 .
. . . . . . . .
. . . . . . .
Vv05g0395 Tc04_g001430 . . . . .
. . . . . . . .
. . . . . .
Vv05g0396 . . . . . . .
. . . . . . . .
. . . . .
Vv05g0397 . . Gorai.005G056900.1 .
Gorai.011G184900.1 . . . . . .
. . . . . . . .
.
Vv05g0398 . . Gorai.005G056800.1 .
Gorai.011G184800.1 . . . . . .
. . . . . . . .
.
Vv05g0399 . . . . Gorai.011G184700.1
Gorai.002G026800.1 . . . . .
. . . . . . . .
.
Vv05g0400 . . . . . . .
. . . . . . . .
. . . . .
Vv05g0401 Tc04_g004360 . . . . .
. . . . . . . .
. . . . . .
Vv05g0402 Tc04_g004350 . . . . .
. . . . . . . .
. . . . . .
Vv05g0403 . . . . . . .
. . . . . . . .
. . . . .
Vv05g0404 . . . . Gorai.011G184200.1
. . . . . . . .
. . . . . . .
Vv05g0405 . . . . . . .
. . . . . . . .
. . . . .
Vv05g0406 . . . . . . .
. . . . . . . .

```
        .       .       .       .       .
Vv05g0407       .       .       .       .       .       .       .
        .       .       .       .       .       .       .       .
        .       .       .       .       .
Vv05g0408       .       .       .       .       .       .       .
        .       .       .       .       .       .       .       .
        .       .       .       .       .
Vv05g0409       .       .       .       .       .       .       .
        .       .       .       .       .       .       .       .
        .       .       .       .       .
Vv05g0410       .       .       Gorai.005G056500.1      .
Gorai.011G183800.1      Gorai.002G023800.1      .       .       .
        .       .       .       .       .       .       .       .
        .       .       .
Vv05g0411       .       .       .       .       .       .       .
        .       .       .       .       .       .       .       .
        .       .       .       .       .
Vv05g0412       .       .       .       Gorai.006G074800.1
Gorai.011G183300.1      .       .       .       .       .       .
        .       .       .       .       .       .       .       .
        .
Vv05g0413       .       Gorai.006G256700.1      Gorai.005G056300.1
        .       Gorai.011G183100.1      .       .       .       .
        .       .       .       .       .       .       .       .
        .       .       .
Vv05g0414       .       Gorai.006G256600.1      .       .
Gorai.011G182900.1      .       Vv07g0259       Tc01_g029180
Gorai.007G132900.1      .       .       .       .       .       .
        .       .       .       .       .
Vv05g0415       .       Gorai.006G256500.1      .       .
Gorai.011G182800.1      .       .       .       .       .       .
        .       .       .       .       .       .       .       .
        .
Vv05g0416       .       .       .       .       .       .       .
        .       .       .       .       .       .       .       .
        .       .       .       .       .
Vv05g0417       .       .       Gorai.005G056200.1      .
Gorai.011G182600.1      .       .       .       .       .       .
        .       .       .       .       .       .       .       .
        .
Vv05g0418       Tc04_g025470    .       .       .       .       .
        .       .       .       .       .       .       .       .
        .       .       .       .       .       .
Vv05g0419       .       .       .       .       .       .       .
        .       .       .       .       .       .       .       .
        .       .       .       .       .
Vv05g0420       .       .       .       Gorai.006G074600.1
Gorai.011G182400.1      .       .       .       .       .       .
        .       .       .       .       .       .       .       .
        .
```

Vv05g0421 . Gorai.006G256300.1 . .
Gorai.011G182100.1 . . . . . .
. . . . . . . .
.
Vv05g0422 . . . . Gorai.011G182000.1
. Vv07g0250 Tc01_g029110 Gorai.007G133200.1
. . . Gorai.004G215000.1 . .
. . . . .
Vv05g0423 . . . . Gorai.011G181900.1
. . . . . . . .
. . . . . . .
Vv05g0424 . . . . Gorai.011G181800.1
. . . . . . . .
. . . . . . .
Vv05g0425 . Gorai.006G256200.1 Gorai.005G056100.1
. . . . . . . .
. . . . . . . .
.
Vv05g0426 . . . . . . .
. . . . . . . .
. . . . .
Vv05g0427 . Gorai.006G256100.1 . . .
. . . . . . . .
. . . . . . .
Vv05g0428 . Gorai.006G256000.1 . . .
. . . . . . . .
. . . . . . .
Vv05g0429 . Gorai.006G255900.1 Gorai.005G055900.1
. Gorai.011G181400.1 . . . .
. . . . . . . .
. . .
Vv05g0430 . Gorai.006G255800.1 Gorai.005G055800.1
. Gorai.011G181300.1 . . . .
. . . . . . . .
. . .
Vv05g0431 . . . . Gorai.011G181200.1
. Vv07g0239 Tc01_g029000 Gorai.007G133400.1
. . . . . . . .
. . .
Vv05g0432 . Gorai.006G255700.1 . . .
. . . . . . . .
. . . . . . .
Vv05g0433 Tc04_g001660 Gorai.006G255600.1 . .
. . . . . . . .
. . . . . . . .
Vv05g0434 . . . Gorai.006G074300.1 .
. . . . . . . .
. . . . . . .
Vv05g0435 . . . . . .
Vv07g0238 Tc01_g028950 . . . Gorai.

004G083900.1 . . . . . . .
.
Vv05g0436 . . Gorai.005G055600.1 .
Gorai.011G181100.1 . . . . . .
. . . . . . .
.
Vv05g0437 . Gorai.006G255400.1 Gorai.005G052600.1
Gorai.006G072000.1 Gorai.011G178100.1 . .
. . . . . . .
. . . . .
Vv05g0438 . Gorai.006G218300.1 . . .
. . . . . . .
. . . . . . .
Vv05g0439 Tc04_g022620 Gorai.006G218200.1 . .
. . . . . . .
. . . . . . .
Vv05g0440 Tc04_g022630 Gorai.006G218100.1 . .
. . Vv07g0234 . . . .
. . . . . . .
.
Vv05g0441 Tc04_g022640 . . . Gorai.
011G168000.1 . . . . . . .
. . . . . . .
Vv05g0442 Tc04_g022650 . . . Gorai.
011G168100.1 . . . . . . .
. . . . . . .
Vv05g0443 Tc04_g022660 Gorai.006G218000.1 . .
Gorai.011G168200.1 . . . . .
. . . . . . .
. .
Vv05g0444 Tc04_g022670 . . . . .
Vv07g0227 Tc01_g028870 Gorai.007G134100.1 .
. Gorai.004G055100.1 Gorai.004G215300.1 .
. . . . . .
Vv05g0445 Tc04_g022680 . . . . .
. . . . . . .
. . . . . .
Vv05g0446 . . . . . . .
. . . . . . .
. . . . .
Vv05g0447 Tc04_g022720 . Gorai.005G040100.1 .
. . Vv07g0216 . . . .
. . . . . . .
.
Vv05g0448 Tc04_g022730 . Gorai.005G040200.1 .
. . Vv07g0215 . . . .
. . . . . . .
.
Vv05g0449 Tc04_g022740 . Gorai.005G040300.1 .
. . . . . . .

. . . . . . . .
Vv05g0450 Tc04_g022760 . Gorai.005G040400.1 .
. . Vv07g0210 Tc01_g028830 Gorai.
007G134500.1 . . Gorai.004G055200.1 . .
. . . . . .
Vv05g0451 Tc04_g022770 . . . . .
. . . . . . . .
. . . . . .
Vv05g0452 Tc04_g022780 . . . Gorai.
011G170000.1 . . . . . . .
. . . . . . . .
Vv05g0453 Tc04_g022790 . Gorai.005G040500.1 .
. . . . . . . .
. . . . . . . .
Vv05g0454 Tc04_g022810 . Gorai.005G040600.1 .
Gorai.011G170300.1 . Vv07g0199
Tc01_g028730 . . . Gorai.004G055400.1 .
. . . . . . .
Vv05g0455 . . . . . . .
. . . . . . . .
. . . . .
Vv05g0456 . . . . . .
Vv07g0197 Tc01_g028710 Gorai.007G135400.1 . .
Gorai.004G082600.1 . . . . .
. . .
Vv05g0457 . . . . . . .
. . . . . . . .
. . . . .
Vv05g0458 Tc04_g022850 . . . . .
. . . . . . . .
. . . . . .
Vv05g0459 Tc04_g022860 . . Gorai.006G053500.1
. . . . . . . .
. . . . . . . .
Vv05g0460 . . . . . . .
. . . . . . . .
. . . . .
Vv05g0461 Tc04_g022870 Gorai.006G217400.1 .
Gorai.006G053600.1 . . Vv07g0196 .
Gorai.007G135500.1 . . . . . .
. . . . .
Vv05g0462 Tc04_g022880 . . . . .
. . . . . . . .
. . . . . .
Vv05g0463 Tc04_g022910 . . Gorai.006G053800.1
. . . . . . . .
. Vv14g0389 Tc04_g025620 . . .
Gorai.006G053800.1 .
Vv05g0464 Tc04_g022920 . . Gorai.006G054000.1
. . . . . . . .

. Vv14g0388 Tc04_g022920 . Gorai.
007G295300.1 . . .
Vv05g0465 Tc04_g022930 Gorai.006G217200.1 .
Gorai.006G054300.1 . . . . . .
. . . . . . . .
. .
Vv05g0466 Tc04_g022950 Gorai.006G214300.1 . .
. . . . . . . .
. . . . . . . .
Vv05g0467 Tc04_g022970 . . . . .
Vv07g0187 . . . . Gorai.
004G081800.1 Gorai.004G242400.1 Vv14g0387
Tc04_g022960 . Gorai.007G295400.1 . .
Gorai.004G215700.1
Vv05g0468 Tc04_g022990 . . . . .
Vv07g0186 . . . . .
Gorai.004G242500.1 Vv14g0386 Tc04_g022980 . .
. . .
Vv05g0469 Tc04_g023030 Gorai.006G236300.1 . .
. . . . . . . .
. . . . . . . .
Vv05g0470 . . . . . . .
. . . . . . Vv14g0385
. . . . . .
Vv05g0471 . . . . . . .
. . . . . . . .
. . . . .
Vv05g0472 . . . . . . .
. . . . . . . .
. . . . .
Vv05g0473 . . . . . . .
. . . . . . . .
. . . . .
Vv05g0474 . . . . . . .
. . . . . . . .
. . . . .
Vv05g0475 Tc04_g023050 . Gorai.005G040900.1 .
. . . . . . . .
. . . . . . . .
Vv05g0476 Tc04_g023060 . Gorai.005G041000.1 .
. . . . . . . .
. . . . . . . .
Vv05g0477 . . . . . . .
. . . . . . . .
. . . . .
Vv05g0478 Tc04_g023070 . Gorai.005G041100.1 .
. . Vv07g0183 . . . .
. . . . . . . .
.
Vv05g0479 Tc04_g023080 Gorai.006G236400.1 . .

. . . . . . . .
. . . . . . . .
Vv05g0480 Tc04_g023090 . . . . .
. . . . . . . .
. . . . . .
Vv05g0481 Tc04_g023110 . . . . .
. . . . . . . .
. . . . . .
Vv05g0482 Tc04_g023140 . . . . .
. . . . . . . .
. . . . . .
Vv05g0483 Tc04_g023180 Gorai.006G236500.1 . .
. . . . . . . .
. . . . . . . .
Vv05g0484 Tc04_g023190 . . . Gorai.
011G165900.1 . . . . . . .
. . . . . . . .
Vv05g0485 Tc04_g023200 Gorai.006G236600.1 . .
. . . . . . . .
. . . . . . . .
Vv05g0486 . . . . . . .
. . . . . . . .
. . . . .
Vv05g0487 . . . . . . .
. . . . . . . .
. . . . .
Vv05g0488 . . . . . . .
. . . . . . . .
. . . . .
Vv05g0489 Tc04_g023210 . Gorai.005G041200.1 .
. . . . . . . .
. Vv14g0370 Tc04_g023210 Gorai.013G123800.1
. . Gorai.006G052800.1 .
Vv05g0490 Tc04_g023230 . Gorai.005G041300.1 .
. . . . . . . .
. Vv14g0365 Tc04_g023230 . Gorai.
007G296500.1 . . .
Vv05g0491 Tc04_g023240 . . . . .
. . . . . . . .
. . . . . .
Vv05g0492 . . . . . . .
. . . . . . . .
. . . . .
Vv05g0493 . . . . . . .
. . . . . . . .
. . . . .
Vv05g0494 Tc04_g023260 . Gorai.005G041400.1
Gorai.006G053000.1 . . . . . .
. . . . . . . .
. .

Vv05g0495 Tc04_g023270 . . . . .
. . . . . . . .
. . . . . .
Vv05g0496 Tc04_g023290 . . . . .
. . . . . . . .
. . . . . .
Vv05g0497 Tc04_g023300 . . . Gorai.
011G166200.1 . . . . . . .
. . . . . . . .
Vv05g0498 . . . . . . .
. . . . . . . .
. . . . .
Vv05g0499 Tc04_g023310 . . . . .
. . . . . . . .
. . . . . .
Vv05g0500 Tc04_g023320 Gorai.006G237000.1 . .
. . . . . . . .
. . . . . . . .
Vv05g0501 Tc04_g023330 . . . . .
. . . . . . . .
. . . . . .
Vv05g0502 . Gorai.006G237100.1 Gorai.005G041500.1
. . . . . . . .
. . . . . . . .
.
Vv05g0503 Tc04_g023340 . . . . .
. . . . . . . .
. . . . . .
Vv05g0504 Tc04_g023350 Gorai.006G237200.1 . .
. . . . . . . .
. . . . . . . .
Vv05g0505 . . . . . . .
. . . . . . . .
. . . . .
Vv05g0506 . . . . . . .
. . . . . . . .
. . . . .
Vv05g0507 Tc04_g023390 . . . . .
. . . . . . . .
. . . . . .
Vv05g0508 Tc04_g023400 . . Gorai.006G052200.1
. Gorai.002G023900.1 . . . .
. . . Vv14g0331 Tc04_g023400 .
. . Gorai.007G342400.1 Gorai.002G023900.1
Vv05g0509 Tc04_g023410 . . . Gorai.
011G168600.1 Gorai.002G024000.1 . . . .
. . . . . . . .
. .
Vv05g0510 . . . . . . .
. . . . . . . .

. . . . .
Vv05g0511 . . . . . . .
. . . . . . . .
. . . . .
Vv05g0512 Tc04_g023430 . . Gorai.006G051900.1
. . . . . . . .
. . . . . . . .
Vv05g0513 . . . . . . .
. . . . . . . .
. . . . .
Vv05g0514 Tc04_g023440 Gorai.006G237500.1 . .
Gorai.011G169100.1 . Vv07g0172
Tc01_g027640 Gorai.007G137200.1 . . Gorai.
004G079700.1 . . . . . . .
.
Vv05g0515 Tc04_g023450 . . Gorai.006G051800.1
. . . . . . . .
. . . . . . . .
Vv05g0516 Tc04_g023460 Gorai.006G237700.1 . .
. . Vv07g0170 Tc01_g027740 . .
. . . . . . . .
. .
Vv05g0517 . . . . . . .
. . . . . . . .
. . . . .
Vv05g0518 Tc04_g023470 Gorai.006G238000.1 Gorai.
005G041600.1 . Gorai.011G169300.1 . . .
. . . . . . . .
. . . .
Vv05g0519 Tc04_g023490 Gorai.006G238200.1 Gorai.
005G041900.1 . . . . . . .
. . . Vv14g0323 . . .
. . .
Vv05g0520 Tc04_g023500 . Gorai.005G042000.1 .
. . Vv07g0163 Tc01_g027810 . .
. Gorai.004G079200.1 . . . .
. . . .
Vv05g0521 . . . . . . .
. . . . . . . .
. . . . .
Vv05g0522 Tc04_g023510 . . . . .
. . . . . . . .
. . . . . .
Vv05g0523 . . . . . . .
. . . . . . . .
. . . . .
Vv05g0524 Tc04_g023520 . . . . .
. . . . . . . .
. . . . . .
Vv05g0525 . . . . . . .

. . . . . . . .
. . . . .
Vv05g0526 Tc04_g023580 . . . . .
. . . . . . . .
. . . . . .
Vv05g0527 . . . . . . .
. . . . . . . .
. . . . .
Vv05g0528 . . . . . . .
. . . . . . . .
. . . . .
Vv05g0529 . . Gorai.005G042100.1 Gorai.
006G051500.1 . . . . . . .
. . . . . . . .
.
Vv05g0530 Tc04_g023590 . . . . .
. . . . . . . .
. . . . . .
Vv05g0531 . . . . . . .
. . . . . . . .
. . . . .
Vv05g0532 Tc04_g023600 Gorai.006G238300.1 .
Gorai.006G048800.1 . . . . . .
. . . . . . . .
. .
Vv05g0533 . . . . . . .
. . . . . . . .
. . . . .
Vv05g0534 . . . . . . .
. . . . . . . .
. . . . .
Vv05g0535 Tc04_g023610 . . . . .
. . . . . . . .
. . . . . .
Vv05g0536 . . . . . . .
. . . . . . . .
. . . . .
Vv05g0537 Tc04_g023620 Gorai.006G238500.1 . .
. . . . . . . .
. . . . . . . .
Vv05g0538 . . . . . .
Vv07g0159 Tc01_g027870 Gorai.007G137800.1 . .
Gorai.004G079100.1 . . . . .
. . .
Vv05g0539 Tc04_g023630 . Gorai.005G042200.1 .
. . . . . . . .
. . . . . . . .
Vv05g0540 Tc04_g023640 Gorai.006G238700.1 Gorai.
005G042300.1 . . . Vv07g0156 . .
. . . . . . . .

. . .
Vv05g0541 Tc04_g023660 . . . . .
. . . . . . . .
. . . . . .
Vv05g0542 . . . . . . .
. . . . . . . .
. . . . .
Vv05g0543 Tc04_g023670 . Gorai.005G042400.1
Gorai.006G049500.1 . . . . . .
. . . . . . . .
. .
Vv05g0544 . . . . . . .
. . . . . . . .
. . . . .
Vv05g0545 . . . . . . .
. . . . . . . .
. . . . .
Vv05g0546 Tc04_g023730 Gorai.006G239000.1 Gorai.
005G042800.1 . . . Vv07g0154
Tc01_g027910 . . . Gorai.004G078800.1 .
. . . . . . .
Vv05g0547 Tc04_g023740 Gorai.006G239100.1 . .
. . . . . . . .
. . . . . . . .
Vv05g0548 Tc04_g023750 . . . . .
. . . . . . . .
. . . . . .
Vv05g0549 . . . . . .
Vv07g0152 . . . . . . .
. . . . . .
Vv05g0550 Tc04_g023760 . . . . .
. . . . . . . .
. . . . . .
Vv05g0551 . Gorai.006G239300.1 Gorai.005G042900.1
. . . . . . . .
. . . . . . . .
.
Vv05g0552 Tc04_g023770 . . . . .
. . . . . . . .
. . . . . .
Vv05g0553 Tc04_g023780 Gorai.006G239400.1 . .
. . . . . . . .
. . . . . . . .
Vv05g0554 . . . . . . .
. . . . . . . .
. . . . .
Vv05g0555 . . . . . . .
. . . . . . . .
. . . . .
Vv05g0556 Tc04_g023800 Gorai.006G239600.1 . .

. . . . . . . .
. . . . . . . .
Vv05g0557 . . . . . . .
. . . . . . . .
. . . . .
Vv05g0558 Tc04_g023820 Gorai.006G239700.1 . .
. . . . . . . .
. . . . . . . .
Vv05g0559 Tc04_g023830 . Gorai.005G043200.1 .
. . Vv07g0150 Tc01_g027970 Gorai.
007G138200.1 . . Gorai.004G078600.1 . .
. . . . . .
Vv05g0560 Tc04_g023840 . . . .
Gorai.002G024200.1 . . . . . .
. . . . . . . .
Vv05g0561 . . . . . . .
. . . . . . . .
. . . . .
Vv05g0562 Tc04_g023850 . . . . .
. . . . . . . .
. . . . . .
Vv05g0563 Tc04_g023880 . . . . .
. . . . . . . .
. . . . . .
Vv05g0564 Tc04_g023890 Gorai.006G239800.1 Gorai.
005G043500.1 . . . . . . .
. . . . . . . .
. .
Vv05g0565 . . . . . . .
. . . . . . . .
. . . . .
Vv05g0566 Tc04_g023910 . . . . .
. . . . . . . .
. . . . . .
Vv05g0567 Tc04_g023920 Gorai.006G239900.1 . .
. Gorai.002G024300.1 Vv07g0149 . .
. . . . . . . .
. . .
Vv05g0568 Tc04_g023930 . . . . .
. . . . . . . .
. . . . . .
Vv05g0569 Tc04_g023940 . . . . .
. . . . . . . .
. . . . . .
Vv05g0570 . . . . . . .
. . . . . . . .
. . . . .
Vv05g0571 Tc04_g023950 Gorai.006G240000.1 . .
. . . . . . . .
. . . . . . . .

Vv05g0572 . . . . . . .
. . . . . . . .
. . . . .
Vv05g0573 Tc04_g023960 Gorai.006G240100.1 . .
. . . . . . . .
. . . . . . . .
Vv05g0574 Tc04_g023970 Gorai.006G240200.1 . .
. . . . . . . .
. . . . . . . .
Vv05g0575 Tc04_g023980 . . . . .
. . . . . . . .
. . . . . .
Vv05g0576 Tc04_g023990 . Gorai.005G044300.1 .
. . . . . . . .
. . . . . . . .
Vv05g0577 Tc04_g024000 . Gorai.005G044500.1 .
. . . . . . . .
. . . . . . . .
Vv05g0578 . . . . . . .
. . . . . . . .
. . . . .
Vv05g0579 . . . . . . .
. . . . . . . .
. . . . .
Vv05g0580 Tc04_g024010 . . . . .
. . . . . . . .
. . . . . .
Vv05g0581 Tc04_g024020 Gorai.006G240400.1 . .
. . Vv07g0144 Tc01_g028030 Gorai.
007G138600.1 . . . Gorai.004G216700.1 .
. . . . . .
Vv05g0582 Tc04_g024030 Gorai.006G240500.1 . .
. . Vv07g0142 Tc01_g028070 . .
. . . . . . . .
. .
Vv05g0583 Tc04_g024040 Gorai.006G240600.1 . .
. . Vv07g0138 Tc01_g028080 . .
. . . . . . . .
. .
Vv05g0584 Tc04_g024060 Gorai.006G240800.1 . .
. . . . . . . .
. . . . . . . .
Vv05g0585 Tc04_g024100 Gorai.006G241200.1 . .
Gorai.011G165500.1 . Vv07g0130
Tc01_g028180 Gorai.007G139100.1 . . Gorai.
004G078100.1 Gorai.004G217200.1 . . . .
. . .
Vv05g0586 . . . . . . .
. . . . . . . .
. . . . .

Vv05g0587 Tc04_g024110 Gorai.006G241400.1 . .
. . . . . . . .
. . . . . . . .
Vv05g0588 Tc04_g024120 . . . . .
. . . . . . . .
. . . . . .
Vv05g0589 . . . . . . .
. . . . . . . .
. . . . .
Vv05g0590 . . . . . . .
. . . . . . . .
. . . . .
Vv05g0591 Tc04_g024130 . . . Gorai.
011G165600.1 . . . . . . .
. . . . . . . .
Vv05g0592 Tc04_g024140 . . . Gorai.
011G165700.1 . . . . . . .
. . . . . . . .
Vv05g0593 Tc04_g024150 . Gorai.005G044600.1
Gorai.006G067900.1 . . Vv07g0126
Tc01_g028210 . . . Gorai.004G078000.1 .
. . . . . . .
Vv05g0594 Tc04_g024160 Gorai.006G241500.1 Gorai.
005G044700.1 . Gorai.011G167400.1 . . .
. . . . . . . .
. . . .
Vv05g0595 Tc04_g024170 . Gorai.005G044800.1 .
. . . . . . . .
. Vv14g0592 Tc04_g024170 Gorai.013G121300.1
Gorai.007G284200.1 . . .
Vv05g0596 Tc04_g024180 Gorai.006G241600.1 . .
. . Vv07g0122 Tc01_g028240 Gorai.
007G139300.1 . . Gorai.004G077700.1 Gorai.
004G217300.1 . . . . . . .
Vv05g0597 Tc04_g024200 . . . . .
. . . . . . . .
. . . . . .
Vv05g0598 Tc04_g024210 . . . .
Gorai.002G024400.1 . . . . . .
. . . . . . . .
Vv05g0599 . . . . . .
Vv07g0121 Tc01_g028250 Gorai.007G139400.1 . .
. . . . . . . .
.
Vv05g0600 . . . . . . .
. . . . . . . .
. . . . .
Vv05g0601 . . . . . . .
. . . . . . . .
. . . . .

Vv05g0602 Tc04_g024220 . . . Gorai.
011G167600.1 . Vv07g0118 . . . .
. . . . . . . .
.
Vv05g0603 Tc04_g024240 Gorai.006G241800.1 . .
Gorai.011G167700.1 . . . . .
. . . Vv14g0601 Tc04_g024240 .
. Gorai.013G201400.1 Gorai.011G167700.1 .
Vv05g0604 . . . . . . .
. . . . . . . .
. . . . .
Vv05g0605 Tc04_g024250 Gorai.006G241900.1 Gorai.
005G045100.1 . . . . . . .
. . . . . . . .
. .
Vv05g0606 Tc04_g024260 Gorai.006G242000.1 Gorai.
005G045200.1 . . . Vv07g0115
Tc01_g028280 . . . Gorai.004G077500.1 .
. . . . . . .
Vv05g0607 . . . . . .
Vv07g0114 Tc01_g028290 . . . Gorai.
004G056000.1 Gorai.004G217400.1 . . . .
. . .
Vv05g0608 Tc04_g024280 . . . Gorai.
011G170100.1 . Vv07g0113 . . . .
. . . . . . . .
.
Vv05g0609 Tc04_g024300 Gorai.006G242100.1 . .
Gorai.011G168700.1 . . . . .
. . . . . . . .
. .
Vv05g0610 Tc04_g024310 . . . . .
Vv07g0094 Tc01_g028330 . . .
Gorai.004G077100.1 . . . . . .
. .
Vv05g0611 . . . . . . .
. . . . . . . .
. . . . .
Vv05g0612 . . . . . . .
. . . . . . . .
. . . . .
Vv05g0613 . . . . . . .
. . . . . . . .
. . . . .
Vv05g0614 Tc04_g024340 Gorai.006G242400.1 . .
Gorai.011G168400.1 . Vv07g0088
Tc01_g028390 Gorai.007G139600.1 . . Gorai.
004G076900.1 . . . . . . .
.
Vv05g0615 Tc04_g024350 Gorai.006G242500.1 . .

. . . . . . . .
. . . . . . . .
Vv05g0616 Tc04_g024370 Gorai.006G242700.1 . .
. . . . . . . .
. . . . . . . .
Vv05g0617 . . . . . . .
. . . . . . . .
. . . . .
Vv05g0618 . . . . . . .
. . . . . . . .
. . . . .
Vv05g0619 Tc04_g024410 Gorai.006G242800.1 Gorai.
005G045500.1 . . . . . . .
. . . Vv14g0622 . Gorai.
013G121000.1 Gorai.007G282000.1 . . .
Vv05g0620 . . . . . . .
. . . . . . . .
. . . . .
Vv05g0621 Tc04_g024420 Gorai.006G242900.1 . .
Gorai.011G168300.1 Gorai.002G024500.1 Vv07g0085
Tc01_g028460 . . . . . .
. . . . . .
Vv05g0622 Tc04_g024430 . . Gorai.006G069700.1
Gorai.011G166300.1 Gorai.002G024600.1 . .
. . . . . . . .
. . . .
Vv05g0623 . . . . . . .
. . . . . . . .
. . . . .
Vv05g0624 Tc04_g024440 Gorai.006G243000.1 Gorai.
005G045700.1 . . . . . . .
. . . . . . . .
. .
Vv05g0625 Tc04_g024450 . . . Gorai.
011G166400.1 . . . . . . .
. . . . . . . .
Vv05g0626 Tc04_g003600 Gorai.006G243200.1 . .
Gorai.011G166600.1 . . . . .
. . . . . . . .
. .
Vv05g0627 . . . . . . .
. . . . . . . .
. . . . .
Vv05g0628 . . . . . . .
. . . . . . . .
. . . . .
Vv05g0629 . Gorai.006G243400.1 Gorai.005G045800.1
. . . . . . . .
. . . . . . . .
.

Vv05g0630 . . . . . . .
. . . . . . . .
. . . . .
Vv05g0631 . . . . . . .
. . . . . . . .
. . . . .
Vv05g0632 . . . . . . .
. . . . . . Vv14g0626
Tc05_g017380 . Gorai.007G279900.1 . .
.
Vv05g0633 . . . . . . .
. . . . . . . .
. . . . .
Vv05g0634 . . . . . . .
. . . . . . . .
. . . . .
Vv05g0635 . . . . . . .
. . . . . . . .
. . . . .
Vv05g0636 . . . . . . .
. . . . . . . .
. . . . .
Vv05g0637 . . . . . . .
. . . . . . . .
. . . . .
Vv05g0638 . . . . . . .
. . . . . . . .
. . . . .
Vv05g0639 . . . . . . .
. . . . . . . .
. . . . .
Vv05g0640 . . . . . . .
. . . . . . . .
. . . . .
Vv05g0641 . Gorai.006G243500.1 . . .
. . . . . . . .
Vv14g0627 . . . . . .
Vv05g0642 . . . . . . .
. . . . . . Vv14g0628
. . . . . .
Vv05g0643 . . . . . . .
. . . . . . . .
. . . . .
Vv05g0644 . . . . . . .
. . . . . . . .
. . . . .
Vv05g0645 . . . . . . .
. . . . . . . .
. . . . .
Vv05g0646 . . . . . . .

. . . . . . . .
. . . . .
Vv05g0647 . . . . . . .
. . . . . . . .
. . . . .
Vv05g0648 . . . . . . .
. . . . . . . .
. . . . .
Vv05g0649 . . . . . . .
. . . . . . . .
. . . . .
Vv05g0650 . . . . . . .
. . . . . . . .
. . . . .
Vv05g0651 . . . . . . .
. . . . . . . .
. . . . .
Vv05g0652 . . . . . . .
. . . . . . . .
. . . . .
Vv05g0653 Tc04_g003530 Gorai.006G243600.1 Gorai.
005G046000.1 . Gorai.011G166700.1 . . .
. . . . . . . .
. . . .
Vv05g0654 . . Gorai.005G046100.1 .
Gorai.011G166800.1 . Vv07g0076 . Gorai.
007G140400.1 . . Gorai.004G076300.1 . .
. . . . . .
Vv05g0655 . . . . . . .
. . . . . . . .
. . . . .
Vv05g0656 . Gorai.006G243700.1 . . .
. . . . . . . .
. . . . . . .
Vv05g0657 . . . . . . .
. . . . . . . .
. . . . .
Vv05g0658 . Gorai.006G244200.1 . . .
. . . . . . . .
. . . . . . .
Vv05g0659 Tc04_g001110 Gorai.006G244300.1 . .
Gorai.011G167100.1 . . . . .
. . . . . . . .
. .
Vv05g0660 . . . . . . .
. . . . . . . .
. . . . .
Vv05g0661 . Gorai.006G244400.1 . .
Gorai.011G167200.1 . Vv07g0071 . . .
. . Gorai.004G218100.1 . . .

```
        .       .       .       .
Vv05g0662       .       Gorai.006G244800.1      .       .       .
        .       Vv07g0070       .       .       Gorai.001G242200.1
        .       Gorai.004G075500.1      Gorai.004G218200.1      .
        .       .       .       .       .       .
Vv05g0663       .       .       .       .       .       .       .
        .       .       .       .       .       .       Vv14g0636
        Tc04_g020510    Gorai.013G120800.1      .       .       .
        .
Vv05g0664       .       .       .       .       Gorai.011G167300.1
        .       .       .       .       .       .       .       .
        .       .       .       .       .       .       .
Vv05g0665       Tc04_g024460    Gorai.006G245000.1      .
Gorai.006G069800.1      Gorai.011G165200.1      .       Vv07g0068
        .       .       .       .       .       .       .       .
        .       .       .       .       .
Vv05g0666       .       .       .       .       .       .       .
        .       .       .       .       .       .       .       .
        .       .       .       .       .
Vv05g0667       Tc04_g024480    .       .       .       .       .
        .       .       .       .       .       .       .       .
        .       .       .       .       .       .
Vv05g0668       .       Gorai.006G245100.1      .       .       .
        .       .       .       .       .       .       .       .
        .       .       .       .       .       .       .
Vv05g0669       Tc04_g024510    Gorai.006G245200.1      Gorai.
005G046300.1    .       .       .       .       .       .       .
        .       .       .       .       .       .       .       .
        .       .
Vv05g0670       Tc04_g024520    Gorai.006G245300.1      .       .
        .       .       .       .       .       .       .       .
        .       .       .       .       .       .       .       .
Vv05g0671       Tc04_g024530    Gorai.006G245500.1      .       .
        .       .       .       .       .       .       .       .
        .       .       .       .       .       .       .       .
Vv05g0672       Tc04_g024540    .       .       .       Gorai.
011G164700.1    .       .       .       .       .       .       .
        .       .       .       .       .       .       .       .
Vv05g0673       Tc04_g024550    Gorai.006G245600.1      .       .
        .       .       .       .       .       .       .       .
        .       .       .       .       .       .       .       .
Vv05g0674       .       .       .       .       .       .       .
        .       .       .       .       .       .       .       .
        .       .       .       .       .
Vv05g0675       Tc04_g024560    Gorai.006G245700.1      .
Gorai.006G070000.1      Gorai.011G161700.1      .       .       .
        .       .       .       .       .       .       .       .
        .       .       .       .
Vv05g0676       Tc04_g024570    .       .       Gorai.006G070100.1
        Gorai.011G170500.1      .       .       .       .       .
```

. . . . . . . .
. .
Vv05g0677 . Gorai.006G245800.1 . . .
. . . . . . . .
. . . . . . .
Vv05g0678 Tc04_g024580 . . Gorai.006G070200.1
. . . . . . . .
. . . . . . . .
Vv05g0679 Tc04_g024590 Gorai.006G245900.1 Gorai.
005G046400.1 . . . Vv07g0049 .
Gorai.007G141900.1 Gorai.001G242800.1 . Gorai.
004G075000.1 . Vv14g0651 Tc04_g024590 . .
Gorai.007G141900.1 . .
Vv05g0680 Tc04_g024600 Gorai.006G246000.1 Gorai.
005G046500.1 . Gorai.011G170700.1 . Vv07g0048
. . Gorai.001G242900.1 . . .
. . . . . . .
Vv05g0681 Tc04_g024620 . Gorai.005G046600.1 .
. . . . . . . .
. . . . . . . .
Vv05g0682 Tc04_g024640 Gorai.006G246100.1 . .
. . . . . . . .
. . . . . . . .
Vv05g0683 . . . . . . .
. . . . . . . .
. . . . .
Vv05g0684 Tc04_g024650 . . . Gorai.
011G170800.1 . Vv07g0030 . . . .
Gorai.004G074400.1 . . . . .
. . .
Vv05g0685 Tc04_g024660 . . . . .
. . . . . . . .
. . . . . .
Vv05g0686 . . . . . .
Vv07g0028 . Gorai.007G142500.1 . .
Gorai.004G074300.1 . . . . . .
. .
Vv05g0687 Tc04_g024720 Gorai.006G246300.1 . .
. . . . . . . .
. . . . . . . .
Vv05g0688 Tc04_g024730 . . . Gorai.
011G170900.1 . . . . . . .
. . . . . . . .
Vv05g0689 . . . . . . .
. . . . . . . .
. . . . .
Vv05g0690 Tc04_g024740 Gorai.006G246400.1 Gorai.
005G046700.1 . . . . . . .
. . . . . . . .
. .

Vv05g0691 . . . . . . .
. . . . . . . .
. . . . .
Vv05g0692 Tc04_g024750 Gorai.006G246500.1 Gorai.
005G046800.1 . Gorai.011G171000.1 . Vv07g0022
. . . . . . . .
. . . . .
Vv05g0693 . . . . . .
Vv07g0019 . . . . . . .
. . . . . .
Vv05g0694 Tc04_g000930 . . . . .
. . . . . . . .
. . . . . .
Vv05g0695 . . . . . . .
. . . . . . . .
. . . . .
Vv05g0696 Tc04_g024760 . . . Gorai.
011G171100.1 . Vv07g0018 . . . .
Gorai.004G074100.1 . . . . .
. . .
Vv05g0697 . Gorai.006G246600.1 . . .
. . . . . . . .
. . . . . . .
Vv05g0698 . . . . . . .
. . . . . . . .
. . . . .
Vv05g0699 . Gorai.006G246800.1 . . .
. . . . . . . .
. . . . . . .
Vv05g0700 Tc04_g024790 . . Gorai.006G070300.1
. Gorai.002G024700.1 . . . .
. . . . . . . .
. .
Vv05g0701 . . . . . . .
. . . . . . . .
. . . . .
Vv05g0702 . . . . . . .
. . . . . . . .
. . . . .
Vv05g0703 Tc04_g024810 . Gorai.005G046900.1 .
. . . . . . . .
. . . . . . . .
Vv05g0704 Tc04_g024820 . . Gorai.006G070400.1
. . . . . . . .
. . . . . . . .
Vv05g0705 . . . . . . .
. . . . . . . .
. . . . .
Vv05g0706 Tc04_g024830 . . . Gorai.
011G171700.1 . . . . . . .

. . . . . . . .
Vv05g0707 Tc04_g024840 . . . . .
. . . . . . . .
. . . . . .
Vv05g0708 . . . . . . .
. . . . . . . .
. . . . .
Vv05g0709 . . . . . . .
. . . . . . . .
. . . . .
Vv05g0710 . . . . . . .
. . . . . . . .
. . . . .
Vv05g0711 Tc04_g024860 . . . . .
. . . . . . . .
. . . . . .
Vv05g0712 . . . . . . .
. . . . . . . .
. . . . .
Vv05g0713 . . . . . . .
. . . . . . . .
. . . . .
Vv05g0714 . . Gorai.005G047200.1 . .
. . . . . . . .
. . . . . . .
Vv05g0715 Tc04_g024910 . . . . .
. . . . . . . .
. . . . . .
Vv05g0716 . . . . . . .
. . . . . . . .
. . . . .
Vv05g0717 . Gorai.006G247200.1 Gorai.005G047400.1
. Gorai.011G171900.1 . . . .
. . . . . . . .
. . .
Vv05g0718 . . . . . . .
. . . . . . . .
. . . . .
Vv05g0719 . . . . . . .
. . . . . . . .
. . . . .
Vv05g0720 . . . . . . .
. . . . . . . .
. . . . .
Vv05g0721 . . . . . . .
. . . . . . . .
. . . . .
Vv05g0722 . . . . . . .
. . . . . . . .
. . . . .

Vv05g0723 Tc04_g024920 . . . . .
. . . . . . . .
. . . . . .
Vv05g0724 Tc04_g024930 Gorai.006G247300.1 . .
. . . . . . . .
. . . . . . . .
Vv05g0725 Tc04_g024950 Gorai.006G247400.1 . .
. . . . . . . .
. . . . . . . .
Vv05g0726 . . . . . . .
. . . . . . . .
. . . . .
Vv05g0727 Tc04_g024960 . . . . .
. . . . . . . .
. . . . . .
Vv05g0728 Tc04_g024970 Gorai.006G247500.1 Gorai.
005G047700.1 . . . . . . .
. . . . . . . .
. .
Vv05g0729 Tc04_g024980 Gorai.006G247600.1 . .
. . . . . . . .
. . . . . . . .
Vv05g0730 Tc04_g024990 . Gorai.005G047800.1 .
Gorai.011G172000.1 . . . . .
. . . . . . . .
. .
Vv05g0731 Tc04_g025000 Gorai.006G247700.1 . .
. . . . . . . .
. . . . . . . .
Vv05g0732 . . . . . . .
. . . . . . . .
. . . . .
Vv05g0733 Tc04_g025010 Gorai.006G247800.1 Gorai.
005G047900.1 . . . . . . .
. . . . . . . .
. .
Vv05g0734 Tc04_g025020 Gorai.006G247900.1 Gorai.
005G048000.1 . . . . . . .
. . . . . . . .
. .
Vv05g0735 Tc04_g025040 Gorai.006G248400.1 Gorai.
005G048100.1 . . . . . . .
. . . . . . . .
. .
Vv05g0736 Tc04_g025060 Gorai.006G248700.1 Gorai.
005G048200.1 Gorai.006G070500.1 . . . .
. . . . . . . .
. . . .
Vv05g0737 Tc04_g025070 Gorai.006G248800.1 . .
. . . . . . . .

. . . . . . . .
Vv05g0738 . . . . . . .
. . . . . . . .
. . . . .
Vv05g0739 . . . . . . .
. . . . . . . .
. . . . .
Vv05g0740 Tc04_g025140 Gorai.006G249000.1 . .
. . . . . . . .
. . . . . . . .
Vv05g0741 Tc04_g025150 . . Gorai.006G070600.1
Gorai.011G172100.1 . . . . .
. . . . . . . .
. .
Vv05g0742 Tc04_g025160 . . . Gorai.
011G172200.1 . . . . . . .
. . . . . . . .
Vv05g0743 . . . . . . .
. . . . . . . .
. . . . .
Vv05g0744 Tc04_g025170 Gorai.006G249100.1 Gorai.
005G048600.1 Gorai.006G070900.1 . . . .
. . . . . . . .
. . . .
Vv05g0745 . . . . . . .
. . . . . . . .
. . . . .
Vv05g0746 . . . . . . .
. . . . . . . .
. . . . .
Vv05g0747 Tc04_g025180 . . . . .
. . . . . . . .
. . . . . .
Vv05g0748 . . . . . . .
. . . . . . . .
. . . . .
Vv05g0749 Tc04_g025190 Gorai.006G249200.1 . .
. . . . . . . .
. . . . . . . .
Vv05g0750 . . . . . . .
. . . . . . . .
. . . . .
Vv05g0751 . . . . . . .
. . . . . . . .
. . . . .
Vv05g0752 . . . . . . .
. . . . . . . .
. . . . .
Vv05g0753 Tc04_g025200 . . . . .
. . . . . . . .

. . . . . .
Vv05g0754 . . . . . . .
. . . . . . . .
. . . . .
Vv05g0755 Tc04_g025210 Gorai.006G249300.1 .
Gorai.006G071000.1 . . . . . .
. . . . . . . .
. .
Vv05g0756 Tc04_g025220 . . . . .
. . . . . . . .
. . . . . .
Vv05g0757 . . . . . . .
. . . . . . . .
. . . . .
Vv05g0758 Tc04_g025230 Gorai.006G249400.1 .
Gorai.006G071200.1 . . . . . .
. . . . . . . .
. .
Vv05g0759 Tc04_g025240 . . . . .
. . . . . . . .
. . . . . .
Vv05g0760 . . . . . . .
. . . . . . . .
. . . . .
Vv05g0761 Tc04_g025250 Gorai.006G249500.1 . .
. . . . . . . .
. . . . . . . .
Vv05g0762 Tc04_g025260 . . . . .
. . . . . . . .
. . . . . .
Vv05g0763 . . . . . . .
. . . . . . . .
. . . . .
Vv05g0764 Tc04_g025270 Gorai.006G249600.1 .
Gorai.006G071300.1 . . . . . .
. . . . . . . .
. .
Vv05g0765 . . . . . . .
. . . . . . . .
. . . . .
Vv05g0766 Tc04_g025290 . . Gorai.006G071500.1
. . . . . . . .
. . . . . . . .
Vv05g0767 Tc04_g025300 . . Gorai.006G071600.1
. . . . . . . .
. . . . . . . .
Vv05g0768 Tc04_g025310 . . Gorai.006G071700.1
. . . . . . . .
. . . . . . . .
Vv05g0769 Tc04_g025320 . Gorai.005G048800.1

Gorai.006G071900.1 . . . . . .
. . . . . . . .
. .
Vv05g0770 . . . . . . .
. . . . . . . .
. . . . .
Vv05g0771 Tc04_g025330 . Gorai.005G048900.1 .
. . . . . . . .
. . . . . . . .
Vv05g0772 . . . . . . .
. . . . . . . .
. . . . .
Vv05g0773 Tc04_g025340 . Gorai.005G049000.1 .
. . Vv07g0695 Tc01_g033420 .
Gorai.008G132500.1 . . . . . .
. . . .
Vv05g0774 . . . . . . .
. . . . . . . .
. . . . .
Vv05g0775 Tc04_g025360 . . Gorai.006G072000.1
. . . . . . . .
. . . . . . . .
Vv05g0776 Tc04_g025370 Gorai.006G249700.1 Gorai.
005G049100.1 . . . . . . .
. . . . . . . .
. .
Vv05g0777 Tc04_g025390 Gorai.006G249900.1 . .
. . . . . . . .
. . . . . . . .
Vv05g0778 Tc04_g025400 Gorai.006G250000.1 Gorai.
005G049200.1 . Gorai.011G172800.1 . . .
. . . . . . . .
. . . .
Vv05g0779 . . . . . . .
. . . . . . . .
. . . . .
Vv05g0780 Tc04_g025410 Gorai.006G250100.1 Gorai.
005G049300.1 . Gorai.011G173000.1 . . .
. . . . . . . .
. . . .
Vv05g0781 . . . . . . .
. . . . . . . .
. . . . .
Vv05g0782 . . . . . . .
. . . . . . . .
. . . . .
Vv05g0783 . . . . . . .
. . . . . . . .
. . . . .
Vv05g0784 . . . . . . .

. . . . . . . .
. . . . .
Vv05g0785 Tc04_g025420 . . . . .
. . . . . . . .
. . . . . .
Vv05g0786 . . . . . . .
. . . . . . . .
. . . . .
Vv05g0787 . . . . . . .
. . . . . . . .
. . . . .
Vv05g0788 . . . . . . .
. . . . . . . .
. . . . .
Vv05g0789 . . . . . . .
. . . . . . . .
. . . . .
Vv05g0790 . . . . . . .
. . . . . . . .
. . . . .
Vv05g0791 . . . . . . .
. . . . . . . .
. . . . .
Vv05g0792 . . . . . . .
. . . . . . . .
. . . . .
Vv05g0793 . . . . . . .
. . . . . . . .
. . . . .
Vv05g0794 . . . . . . .
. . . . . . . .
. . . . .
Vv05g0795 . . . . . . .
. . . . . . . .
. . . . .
Vv05g0796 . . . . . . .
. . . . . . . .
. . . . .
Vv05g0797 . . . . . . .
. . . . . . . .
. . . . .
Vv05g0798 . . . . . . .
. . . . . . . .
. . . . .
Vv05g0799 . . . . . . .
. . . . . . . .
. . . . .
Vv05g0800 . . . . . . .
. . . . . . . .
. . . . .

Vv05g0801 . . . . . . .
. . . . . . . .
. . . . .
Vv05g0802 . . . . . . .
. . . . . . . .
. . . . .
Vv05g0803 . . . . . . .
. . . . . . . .
. . . . .
Vv05g0804 . . . . . . .
. . . . . . . .
. . . . .
Vv05g0805 . . . . . . .
. . . . . . . .
. . . . .
Vv05g0806 . . . . . . .
. . . . . . . .
. . . . .
Vv05g0807 . . . . . . .
. . . . . . . .
. . . . .
Vv05g0808 . . . . . . .
. . . . . . . .
. . . . .
Vv05g0809 . . . . . . .
. . . . . . . .
. . . . .
Vv05g0810 . . . . . . .
. . . . . . . .
. . . . .
Vv05g0811 Tc04_g025460 Gorai.006G250300.1 Gorai.
005G049800.1 . . . . . . .
. . . . . . . .
. .
Vv05g0812 Tc04_g001190 . . . . .
. . . . . . . .
. . . . . .
Vv05g0813 Tc04_g025470 . . Gorai.006G072100.1
. . Vv07g0717 Tc01_g003460 Gorai.
007G146000.1 . . . . . . .
. . . .
Vv05g0814 . . Gorai.005G049900.1 . .
. . . . . . . .
. . . . . . .
Vv05g0815 . . . . . . .
. . . . . . . .
. . . . .
Vv05g0816 Tc04_g025480 . . . . .
Vv07g0719 . . . . . .
. . . . . . .

Vv05g0817 . . . . . . .
. . . . . . . .
. . . . .
Vv05g0818 Tc04_g025500 . . . Gorai.
011G173700.1 . . . . . . .
. . . . . . . .
Vv05g0819 Tc04_g025510 Gorai.006G253700.1 Gorai.
005G050000.1 . . . . . . .
. . . . . . . .
. .
Vv05g0820 . . . . . . .
. . . . . . . .
. . . . .
Vv05g0821 . . . . . . .
. . . . . . . .
. . . . .
Vv05g0822 . . . . . . .
. . . . . . . .
. . . . .
Vv05g0823 . . Gorai.005G050100.1 . .
. Vv07g0721 . . . . .
Gorai.004G220900.1 . . . . .
. .
Vv05g0824 Tc04_g001100 . . . . .
. . . . . . . .
. . . . . .
Vv05g0825 . . . . . . .
. . . . . . . .
. . . . .
Vv05g0826 . . . . . . .
. . . . . . . .
. . . . .
Vv05g0827 . . Gorai.005G050300.1 .
Gorai.011G174300.1 . . . . . .
. . . . . . . .
.
Vv05g0828 . . . . . . .
. . . . . . . .
. . . . .
Vv05g0829 Tc04_g025560 . Gorai.005G037900.1 .
. . . . . . . .
. . . . . . . .
Vv05g0830 . . . . . . .
. . . . . . . .
. . . . .
Vv05g0831 . . Gorai.005G037800.1 . .
. . . . . . . .
. . . . . . .
Vv05g0832 Tc04_g025570 . Gorai.005G037700.1 .
. . . . . . . .

. . . . . . . .
Vv05g0833 Tc04_g025600 . . . .
Gorai.002G025400.1 . . . . . .
. . . . . . . .
Vv05g0834 . . Gorai.005G036300.1 . .
. . . . . . . .
. . . . . . .
Vv05g0835 . . . . . Gorai.
002G025500.1 . . . . . . .
. . . . . . .
Vv05g0836 Tc04_g025610 Gorai.006G253400.1 Gorai.
005G033800.1 . . Gorai.002G025600.1 . .
. . . . . . . .
. . . .
Vv05g0837 . . . . . . .
. . . . . . . .
. . . . .
Vv05g0838 Tc04_g025620 . . . Gorai.
011G174600.1 . . . . . . .
. . . . . . . .
Vv05g0839 Tc04_g025630 Gorai.006G253300.1 Gorai.
005G050400.1 . Gorai.011G174900.1 . Vv07g0744
. Gorai.007G147000.1 . . .
Gorai.004G221200.1 . . . . . .
.
Vv05g0840 . . . . . . .
. . . . . . . .
. . . . .
Vv05g0841 Tc04_g025640 . Gorai.005G050500.1 .
Gorai.011G175000.1 . Vv07g0751
Tc01_g025360 . . . . . . .
. . . . .
Vv05g0842 . . . . . . .
. . . . . . . .
. . . . .
Vv05g0843 . . . . . . .
. . . . . . . .
. . . . .
Vv05g0844 Tc04_g025650 Gorai.006G253200.1 Gorai.
005G050600.1 . Gorai.011G175100.1 . . .
. . . . . . . .
. . . .
Vv05g0845 Tc04_g025680 . . . . .
. . . . . . . .
. . . . . .
Vv05g0846 . . . . . . .
. . . . . . . .
. . . . .
Vv05g0847 . Gorai.006G253000.1 Gorai.005G050700.1
. Gorai.011G175500.1 . . . .

. . . . . . . .
. . .
Vv05g0848 . . . . . . .
. . . . . . . .
. . . . .
Vv05g0849 . . . . . . .
. . . . . . . .
. . . . .
Vv05g0850 . . . . . . .
. . . . . . . .
. . . . .
Vv05g0851 . . . . . . .
. . . . . . . .
. . . . .
Vv05g0852 Tc04_g025740 Gorai.006G252900.1 Gorai.
005G050900.1 . . . . . . .
. . . . . . . .
. .
Vv05g0853 Tc04_g025750 Gorai.006G252800.1 . .
. . . . . . . .
. Vv11g0327 Tc09_g009000 . . .
. Gorai.010G076700.1
Vv05g0854 Tc04_g025770 Gorai.006G252700.1 Gorai.
005G051000.1 Gorai.006G072600.1 . Gorai.002G025700.1
. . . . . . . .
. . . . . .
Vv05g0855 Tc04_g025790 . . . Gorai.
011G176000.1 . . . . . . .
. . . . . . . .
Vv05g0856 . . . . . . .
. . . . . . . .
. . . . .
Vv05g0857 Tc04_g025800 . Gorai.005G051100.1 .
Gorai.011G176400.1 . . . . .
. . . . . . . .
. .
Vv05g0858 Tc04_g025810 . . . Gorai.
011G176800.1 . Vv07g0762 Tc01_g025530 Gorai.
007G148100.1 . . . . . . .
. . . .
Vv05g0859 Tc04_g025820 . Gorai.005G051200.1 .
Gorai.011G176900.1 . . . . .
. . . . . . . .
. .
Vv05g0860 Tc04_g025830 Gorai.006G252600.1 . .
. . . . . . . .
. . . . . . . .
Vv05g0861 . . . . . . .
. . . . . . . .
. . . . .

Vv05g0862 . . . . . . .
. . . . . . . .
. . . . .
Vv05g0863 Tc04_g025840 . Gorai.005G051300.1 .
. . . . . . . .
. . . . . . . .
Vv05g0864 Tc04_g025850 Gorai.006G252500.1 . .
Gorai.011G177100.1 . Vv07g0766
Tc01_g025580 . . . . . . .
. . . . .
Vv05g0865 Tc04_g025860 . Gorai.005G051400.1 .
Gorai.011G177200.1 . . . . .
. . . . . . . .
. .
Vv05g0866 . . . . . . .
. . . . . . . .
. . . . .
Vv05g0867 Tc04_g025890 Gorai.006G252300.1 . .
. . . . . . . .
. . . . . . . .
Vv05g0868 Tc04_g025900 Gorai.006G252200.1 Gorai.
005G051600.1 . . . Vv07g0770
Tc01_g025610 Gorai.007G148300.1 . . .
Gorai.004G221900.1 . . . . . .
.
Vv05g0869 . . . . . . .
. . . . . . . .
. . . . .
Vv05g0870 Tc04_g025920 . Gorai.005G051900.1 .
. . Vv07g0772 Tc01_g025620 Gorai.
007G148500.1 . . . Gorai.004G222000.1
Vv11g0335 Tc09_g009100 . . . Gorai.
009G027300.1 .
Vv05g0871 . . . . . . .
. . . . . . . .
. . . . .
Vv05g0872 . . . . . . .
. . . . . . . .
. . . . .
Vv05g0873 Tc04_g025930 . Gorai.005G052000.1 .
Gorai.011G177300.1 . . . . .
. . . . . . . .
. .
Vv05g0874 Tc04_g025940 . . . Gorai.
011G177400.1 . . . . . . .
. . . . . . . .
Vv05g0875 . . . . . . .
. . . . . . . .
. . . . .
Vv05g0876 Tc04_g025950 . Gorai.005G052100.1 .

. . Vv07g0779 . . . .
. . . . . . . .
.
Vv05g0877 Tc04_g025960 Gorai.006G252000.1 . .
. . . . . . . .
. . . . . . . .
Vv05g0878 Tc04_g025970 . Gorai.005G052500.1 .
. . Vv07g0783 Tc01_g025680 .
Gorai.001G238300.1 . . . . . .
. . . .
Vv05g0879 Tc04_g025980 Gorai.006G251900.1 Gorai.
005G052600.1 . . . . . . .
. . . Vv11g0340 Tc09_g009150
Gorai.013G219200.1 Gorai.001G008200.1 . Gorai.
009G027200.1 Gorai.010G075600.1
Vv05g0880 . . . . . . .
. . . . . . . .
. . . . .
Vv05g0881 . . . . . . .
. . . . . . . .
. . . . .
Vv05g0882 Tc04_g026000 . . . .
Gorai.002G025800.1 . . . . . .
. . . . . . . .
Vv05g0883 Tc04_g026020 Gorai.006G251800.1 . .
. . . . . . . .
. . . . . . . .
Vv05g0884 Tc04_g026050 . Gorai.005G052800.1 .
. . . . . . . .
. . . . . . . .
Vv05g0885 . . . . . . .
. . . . . . . .
. . . . .
Vv05g0886 Tc04_g026070 . Gorai.005G052900.1 .
. Gorai.002G026100.1 . . . .
. . . . . . . .
. .
Vv05g0887 Tc04_g026090 . Gorai.005G053000.1 .
. . . . . . . .
. . . . . . . .
Vv05g0888 . . . . . . .
. . . . . . . .
. . . . .
Vv05g0889 . . . . . . .
. . . . . . . .
. . . . .
Vv05g0890 Tc04_g026110 Gorai.006G251700.1 . .
. . . . . . . .
. . . . . . . .
Vv05g0891 Tc04_g026130 Gorai.006G251600.1 . .

. Gorai.002G026400.1 . . . .
. . . . . . . .
. .
Vv05g0892 Tc04_g026140 . Gorai.005G053200.1 .
. . . . . . . .
. . . . . . . .
Vv05g0893 Tc04_g026170 . . Gorai.006G072700.1
Gorai.011G178600.1 . . . . .
. . . . . . . .
. .
Vv05g0894 Tc04_g026210 Gorai.006G251500.1 Gorai.
005G053300.1 . . . Vv07g0801
Tc01_g025870 Gorai.007G149400.1 . . . .
. . . . . . .
Vv05g0895 . . . . . .
Vv07g0804 Tc01_g025890 Gorai.007G149600.1 . .
. Gorai.004G222200.1 . . . .
. . .
Vv05g0896 . . . . . . .
. . . . . . . .
. . . . .
Vv05g0897 Tc04_g026220 Gorai.006G251400.1 Gorai.
005G053400.1 . . . . . . .
. . . . . . . .
. .
Vv05g0898 Tc04_g026230 . Gorai.005G053500.1
Gorai.006G073000.1 . . . . . .
. . . . . . . .
. .
Vv05g0899 . . . . . . .
. . . . . . . .
. . . . .
Vv05g0900 . . . . . . .
. . . . . . . .
. . . . .
Vv05g0901 Tc04_g026240 Gorai.006G251300.1 Gorai.
005G053600.1 . . Gorai.002G026500.1 . .
. . . . . . . .
. . . .
Vv05g0902 Tc04_g026250 Gorai.006G251200.1 Gorai.
005G053700.1 Gorai.006G073200.1 . . . .
. . . . . . . .
. . . .
Vv05g0903 Tc04_g026260 Gorai.006G251100.1 . .
. . . . . . . .
. . . . . . . .
Vv05g0904 Tc04_g026280 . . . . .
Vv07g0806 Tc01_g025950 . . . .
Gorai.004G222400.1 . . . . .
. .

Vv05g0905 Tc04_g026290 . . . . .
. . . . . . . .
. . . . . .
Vv05g0906 Tc04_g026300 . Gorai.005G053900.1 .
. . . . . . . .
. . . . . . . .
Vv05g0907 Tc04_g026310 . . . . .
. . . . . . . .
. . . . . .
Vv05g0908 Tc04_g026390 . Gorai.005G054000.1 .
. . . . . . . .
. . . . . . . .
Vv05g0909 Tc04_g026400 . Gorai.005G054100.1 .
Gorai.011G179700.1 . Vv07g0810
Tc01_g026020 Gorai.007G149800.1 . . Gorai.
004G059100.1 . . . . . . .
.
Vv05g0910 Tc04_g026410 . . . Gorai.
011G179800.1 . . . . . . .
. . . . . . . .
Vv05g0911 Tc04_g026420 Gorai.006G251000.1 Gorai.
005G054200.1 . Gorai.011G179900.1 . . .
. . . . . . . .
. . . .
Vv05g0912 . . . . . . .
. . . . . . . .
. . . . .
Vv05g0913 Tc04_g026430 Gorai.006G250900.1 . .
Gorai.011G180100.1 . Vv07g0816
Tc01_g026130 Gorai.007G150000.1 . . .
Gorai.004G222500.1 Vv11g0364 Tc09_g009400 . .
. Gorai.009G026700.1 .
Vv05g0914 Tc04_g026440 . . . . .
. . . . . . . .
. . . . . .
Vv05g0915 . . . . . . .
. . . . . . . .
. . . . .
Vv05g0916 Tc04_g026450 Gorai.006G250800.1 .
Gorai.006G073300.1 . . . . . .
. . . . . . . .
. .
Vv05g0917 . . . . . . .
. . . . . . . .
. . . . .
Vv05g0918 . . . . . . .
. . . . . . . .
. . . . .
Vv05g0919 . . . . . . .
. . . . . . . .

. . . . .
Vv05g0920 . . . . . . .
. . . . . . . .
. . . . .
Vv05g0921 . . . . . . .
. . . . . . . .
. . . . .
Vv05g0922 . . . . . . .
. . . . . . . .
. . . . .
Vv05g0923 . . . . . . .
. . . . . . . .
. . . . .
Vv05g0924 . Gorai.006G247800.1 . . .
. . . . . . . .
. . . . . . .
Vv05g0925 . . . . . . .
. . . . . . . .
. . . . .
Vv05g0926 Tc04_g026520 . . . . .
. . . . . . . .
. . . . . .
Vv05g0927 . . Gorai.005G054300.1 . .
. . . . . . . .
. . . . . . .
Vv05g0928 . . . . . .
Vv11g0388 . . . . . . .
. . . . . .
Vv05g0929 . . . . . . .
. . . . . . . .
. . . . .
Vv05g0930 . . . . . . .
. . . . . . . .
. . . . .
Vv05g0931 Tc04_g026580 . . . . .
. . . . . . . .
. . . . . .
Vv05g0932 Tc04_g026590 . . . . .
. . . . . . . .
. . . . . .
Vv05g0933 Tc04_g026600 . . . Gorai.
011G180300.1 . . . . . . .
. . . . . . . .
Vv05g0934 Tc04_g026610 . . . . .
. . . . . . . .
. . . . . .
Vv05g0935 . . . . . . .
. . . . . . . .
. . . . .
Vv05g0936 Tc04_g026620 . Gorai.005G054900.1

Gorai.006G073500.1 . . . . . .
. . . . . . . .
. .
Vv05g0937 . . . . . . .
. . . . . . . .
. . . . .
Vv05g0938 Tc04_g026630 . Gorai.005G055000.1 .
. . . . . . . .
. . . . . . . .
Vv05g0939 Tc04_g026640 . Gorai.005G055100.1 .
Gorai.011G165800.1 . . . . .
. . . . . . . .
. .
Vv05g0940 Tc04_g026660 . . . Gorai.
011G180400.1 . . . . . . .
. . . . . . . .
Vv05g0941 . . . . . . .
. . . . . . . .
. . . . .
Vv05g0942 Tc04_g026670 . . . . .
. . . . . . . .
. . . . . .
Vv05g0943 . . . . . . .
. . . . . . . .
. . . . .
Vv05g0944 . . . . . . .
. . . . . . . .
. . . . .
Vv05g0945 . . . . . . .
. . . . . . . .
. . . . .
Vv05g0946 Tc04_g026690 . Gorai.005G055200.1 .
Gorai.011G180800.1 . . . . .
. . . . . . . .
. .
Vv05g0947 . . . . . . .
. . . . . . . .
. . . . .
Vv05g0948 Tc04_g026700 . . . . .
. . . . . . . .
. . . . . .
Vv05g0949 Tc04_g026720 . . . . .
. . . . . . . .
. . . . . .
Vv05g0950 Tc04_g026730 . . . . .
. . . . . . . .
. . . . . .
Vv05g0951 Tc04_g026770 . . . . .
. . . . . . . .
. . . . . .

Vv05g0952 . . . . . . .
. . . . . . . .
. . . . .
Vv05g0953 Tc04_g026780 . Gorai.005G057300.1
Gorai.006G073600.1 Gorai.011G180900.1 . . .
. . . . . . . .
. . . .
Vv05g0954 . . . . . . .
. . . . . . . .
. . . . .
Vv05g0955 . . . . . . .
. . . . . . . .
. . . . .
Vv05g0956 Tc04_g022600 . Gorai.005G036200.1 .
. . . . . . . .
. . . . . . . .
Vv05g0957 Tc04_g022590 Gorai.006G218600.1 . .
. . . . . . . .
. . . . . . . .
Vv05g0958 . . . . Gorai.011G181000.1
. . . . . . . .
. . . . . . .
Vv05g0959 Tc04_g022580 Gorai.006G218700.1 .
Gorai.006G053100.1 Gorai.011G164300.1 . . .
. . . . . . . .
. . . .
Vv05g0960 Tc04_g022570 . . . Gorai.
011G164200.1 . Vv11g0403 Tc09_g009820 .
Gorai.001G028900.1 . Gorai.009G024800.1 Gorai.
010G071100.1 . . . . . . .
Vv05g0961 Tc04_g022560 . . . Gorai.
011G164100.1 . . . . . . .
. . . . . . . .
Vv05g0962 . . . . . . .
. . . . . . . .
. . . . .
Vv05g0963 . . . . . . .
. . . . . . . .
. . . . .
Vv05g0964 . . . . . . .
. . . . . . . .
. . . . .
Vv05g0965 . . . . . . .
. . . . . . . .
. . . . .
Vv05g0966 Tc04_g022320 Gorai.006G218800.1 . .
Gorai.011G164000.1 . . . . .
. . . . . . . .
. .
Vv05g0967 Tc04_g022310 . Gorai.005G038200.1 .

. . . . . . . .
. . . . . . . .
Vv05g0968 . . . . . . .
. . . . . . . .
. . . . .
Vv05g0969 Tc04_g022290 . Gorai.005G038400.1 .
Gorai.011G163600.1 . . . . .
. . . . . . . .
. .
Vv05g0970 . . . . . . .
. . . . . . . .
. . . . .
Vv05g0971 . . . . . . .
. . . . . . . .
. . . . .
Vv05g0972 . . . . . . .
. . . . . . . .
. . . . .
Vv05g0973 . . . . . . .
. . . . . . . .
. . . . .
Vv05g0974 Tc04_g016890 . . . . .
. . . . . . . .
. . . . . .
Vv05g0975 . . . . . . .
. . . . . . . .
. . . . .
Vv05g0976 . . . . . . .
. . . . . . . .
. . . . .
Vv05g0977 . . . . . . .
. . . . . . . .
. . . . .
Vv05g0978 Tc04_g022240 . Gorai.005G038500.1 .
. . . . . . . .
. . . . . . . .
Vv05g0979 . . . . . . .
. . . . . . . .
. . . . .
Vv05g0980 Tc04_g016870 . . . . .
. . . . . . . .
. . . . . .
Vv05g0981 . . . . . . .
. . . . . . . .
. . . . .
Vv05g0982 . . . . . . .
. . . . . . . .
. . . . .
Vv05g0983 . . . . . . .
. . . . . . . .

. . . . .
Vv05g0984 . . . . . . .
. . . . . . . .
. . . . .
Vv05g0985 . . . . . . .
. . . . . . . .
. . . . .
Vv05g0986 Tc04_g016850 . . . . .
. . . . . . . .
. . . . . .
Vv05g0987 . . . . . . .
. . . . . . . .
. . . . .
Vv05g0988 . . . . . . .
. . . . . . . .
. . . . .
Vv05g0989 . . . . . . .
. . . . . . . .
. . . . .
Vv05g0990 Tc04_g022230 . . . . .
. . . . . . . .
. . . . . .
Vv05g0991 Tc04_g022200 . . . . .
. . . . . . . .
. . . . . .
Vv05g0992 . . . . . . .
. . . . . . . .
. . . . .
Vv05g0993 . Gorai.006G219100.1 . . .
. . . . . . . .
. . . . . . .
Vv05g0994 Tc04_g022140 Gorai.006G219200.1 . .
. . . . . . . .
. . . . . . . .
Vv05g0995 . . . . . . .
. . . . . . . .
. . . . .
Vv05g0996 Tc04_g022120 . Gorai.005G039000.1
Gorai.006G054900.1 . . . . . .
. . . . . . . .
. .
Vv05g0997 . . . . . . .
. . . . . . . .
. . . . .
Vv05g0998 Tc04_g022110 Gorai.006G219300.1 . .
Gorai.011G163200.1 . Vv11g0431
Tc09_g010110 . Gorai.001G003900.1 . Gorai.
009G023600.1 . . . . . . .
.
Vv05g0999 Tc04_g022100 . . . . .

. . . . . . . .
. . . . . .
Vv05g1000 Tc04_g022090 . . . Gorai.
011G163100.1 . . . . . . .
. . . . . . . .
Vv05g1001 . . . . . . .
. . . . . . . .
. . . . .
Vv05g1002 Tc04_g022080 . . . Gorai.
011G163000.1 . . . . . . .
. . . . . . . .
Vv05g1003 . . . . . . .
. . . . . . . .
. . . . .
Vv05g1004 Tc04_g022070 . Gorai.005G039100.1 .
Gorai.011G162900.1 . . . . .
. . . . . . . .
. .
Vv05g1005 . . . . . . .
. . . . . . . .
. . . . .
Vv05g1006 Tc04_g022050 . Gorai.005G039200.1 .
. . . . . . . .
. . . . . . . .
Vv05g1007 . . . . . . .
. . . . . . . .
. . . . .
Vv05g1008 . . . . . . .
. . . . . . . .
. . . . .
Vv05g1009 Tc04_g021930 . . . . .
. . . . . . . .
. . . . . .
Vv05g1010 . . . . . . .
. . . . . . . .
. . . . .
Vv05g1011 . . . . . . .
. . . . . . . .
. . . . .
Vv05g1012 Tc04_g021890 . . Gorai.006G055400.1
Gorai.011G162600.1 . . . . .
. . . . . . . .
. .
Vv05g1013 Tc04_g021850 . . Gorai.006G055600.1
. . . . . . . .
. . . . . . . .
Vv05g1014 . . . . . . .
. . . . . . . .
. . . . .
Vv05g1015 Tc04_g021800 . . . Gorai.

011G162100.1 . . . . . . .
. . . . . . . .
Vv05g1016 Tc04_g021790 Gorai.006G219600.1 . .
. . . . . . . .
. . . . . . . .
Vv05g1017 . . . . . . .
. . . . . . . .
. . . . .
Vv05g1018 Tc04_g021780 Gorai.006G219800.1 . .
Gorai.011G161900.1 . . . . .
. . . . . . . .
. .
Vv05g1019 . . . . . . .
. . . . . . . .
. . . . .
Vv05g1020 . . . . . . .
. . . . . . . .
. . . . .
Vv05g1021 . . . . . . .
. . . . . . . .
. . . . .
Vv05g1022 Tc04_g021760 . . . Gorai.
011G161800.1 . . . . . . .
. . . . . . . .
Vv05g1023 Tc04_g021750 . . Gorai.006G055700.1
. . . . . . . .
. . . . . . . .
Vv05g1024 Tc04_g021720 . Gorai.005G035500.1 .
. . . . . . . .
. . . . . . . .
Vv05g1025 . . . . . . .
. . . . . . . .
. . . . .
Vv05g1026 Tc04_g021670 Gorai.006G219900.1 . .
. . . . . . . .
. . . . . . . .
Vv05g1027 . . . . . . .
. . . . . . . .
. . . . .
Vv05g1028 . . . . . . .
. . . . . . . .
. . . . .
Vv05g1029 . . . . . . .
. . . . . . . .
. . . . .
Vv05g1030 . . . . . . .
. . . . . . . .
. . . . .
Vv05g1031 Tc04_g021660 . . . Gorai.
011G161700.1 . . . . . . .

. . . . . . . .
Vv05g1032 . . . . . . .
. . . . . . . .
. . . . .
Vv05g1033 . . . . . . .
. . . . . . . .
. . . . .
Vv05g1034 . . . . . . .
. . . . . . . .
. . . . .
Vv05g1035 . . . . . . .
. . . . . . . .
. . . . .
Vv05g1036 . . . . . . .
. . . . . . . .
. . . . .
Vv05g1037 . . . . . . .
. . . . . . . .
. . . . .
Vv05g1038 . . . . . . .
. . . . . . . .
. . . . .
Vv05g1039 . . . . . . .
. . . . . . . .
. . . . .
Vv05g1040 . . . . . . .
. . . . . . . .
. . . . .
Vv05g1041 . . . . . . .
. . . . . . . .
. . . . .
Vv05g1042 . . . . . . .
. . . . . . . .
. . . . .
Vv05g1043 . . . . . . .
. . . . . . . .
. . . . .
Vv05g1044 . . . . . . .
. . . . . . . .
. . . . .
Vv05g1045 Tc04_g021640 . . . Gorai.
011G161400.1 . . . . . . .
. . . . . . . .
Vv05g1046 . . Gorai.005G035200.1 . .
. . . . . . . .
. . . . . . .
Vv05g1047 . . . . . . .
. . . . . . . .
. . . . .
Vv05g1048 . . . . . . .

| | | | | | | | |
|---|---|---|---|---|---|---|---|
| Vv05g1049 | . | . | . | . | . | . | . |
| Vv05g1050 | . | . | . | . | Gorai.011G160200.1 | | |
| Vv05g1051 | . | . | . | . | . | . | . |
| Vv05g1052 | . | . | . | . | . | . | . |
| Vv05g1053 | . | Gorai.006G221200.1 | | | . | . | . |
| Vv05g1054 | . | . | . | . | . | . | . |
| Vv05g1055 | Tc04_g021390 | | . | . | . | . | . |
| Vv05g1056 | . | . | . | . | . | . | . |
| Vv05g1057 | . | . | . | . | . | . | . |
| Vv05g1058 | . | . | . | . | . | . | . |
| Vv05g1059 | . | . | . | Gorai.006G056100.1 | | | . |
| Vv05g1060 | . | . | . | . | . | . | . |
| Vv05g1061 | . | . | . | . | . | . | . |
| Vv05g1062 | . | . | . | . | . | . | . |
| Vv05g1063 | . | . | . | . | . | . | . |
| Vv05g1064 | . | . | . | . | . | . | . |

Vv05g1065 . . . . . . .
. . . . . . . .
. . . . .
Vv05g1066 . . . . . . .
. . . . . . . .
. . . . .
Vv05g1067 . . . . . . .
. . . . . . . .
. . . . .
Vv05g1068 . . . . . . .
. . . . . . . .
. . . . .
Vv05g1069 . . . . . . .
. . . . . . . .
. . . . .
Vv05g1070 . . . . . . .
. . . . . . . .
. . . . .
Vv05g1071 . . . . . . .
. . . . . . . .
. . . . .
Vv05g1072 . . . . . . .
. . . . . . . .
. . . . .
Vv05g1073 . . . . . . .
. . . . . . . .
. . . . .
Vv05g1074 . . . . . . .
. . . . . . . .
. . . . .
Vv05g1075 . . . . . . .
. . . . . . . .
. . . . .
Vv05g1076 . . . . . . .
. . . . . . . .
. . . . .
Vv05g1077 . . . . . . .
. . . . . . . .
. . . . .
Vv05g1078 . . . . . . .
. . . . . . . .
. . . . .
Vv05g1079 . . . . . . .
. . . . . . . .
. . . . .
Vv05g1080 . . . . . . .
. . . . . . . .
. . . . .
Vv05g1081 . . . . . . .
. . . . . . . .

. . . . .
Vv05g1082 . . . . . . .
. . . . . . . .
. . . . .
Vv05g1083 . . . . . . .
. . . . . . . .
. . . . .
Vv05g1084 . . . . . . .
. . . . . . . .
. . . . .
Vv05g1085 . . . . . . .
. . . . . . . .
. . . . .
Vv05g1086 . . . . . . .
. . . . . . . .
. . . . .
Vv05g1087 . . . . . . .
. . . . . . . .
. . . . .
Vv05g1088 . . . . . . .
. . . . . . . .
. . . . .
Vv05g1089 . . . . . . .
. . . . . . . .
. . . . .
Vv05g1090 Tc04_g021340 Gorai.006G221300.1 Gorai.
005G034400.1 . Gorai.011G160000.1 . . .
. . . . . . . .
. . . .
Vv05g1091 . . . . . . .
. . . . . . . .
. . . . .
Vv05g1092 . . . . . . .
. . . . . . . .
. . . . .
Vv05g1093 . . . . . . .
. . . . . . . .
. . . . .
Vv05g1094 . . . . . . .
. . . . . . . .
. . . . .
Vv05g1095 . . . . . . .
. . . . . . . .
. . . . .
Vv05g1096 . . . . . . .
. . . . . . . .
. . . . .
Vv05g1097 . . . . . . .
. . . . . . . .
. . . . .

Vv05g1098 . Gorai.006G221900.1 . . .
. . . . . . . .
. . . . . . .
Vv05g1099 . . . . . . .
. . . . . . . .
. . . . .
Vv05g1100 . . . . Gorai.011G159100.1
. . . . . . . .
. . . . . . .
Vv05g1101 . . . . . . .
. . . . . . . .
. . . . .
Vv05g1102 . Gorai.006G222400.1 Gorai.005G033900.1
. Gorai.011G158500.1 . . . .
. . . . . . . .
. . .
Vv05g1103 . . . . Gorai.011G158400.1
. . . . . . . .
. . . . . . .
Vv05g1104 . . . . . . .
. . . . . . . .
. . . . .
Vv05g1105 . . . . . . .
. . . . . . . .
. . . . .
Vv05g1106 . . . . . . .
. . . . . . . .
. . . . .
Vv05g1107 . . . . . . .
. . . . . . . .
. . . . .
Vv05g1108 . . . . . . .
. . . . . . . .
. . . . .
Vv05g1109 . . . . . . .
. . . . . . . .
. . . . .
Vv05g1110 Tc04_g021250 Gorai.006G222500.1 Gorai.
005G033800.1 . . . . . . .
. . . . . . . .
. .
Vv05g1111 . . . . . . .
. . . . . . . .
. . . . .
Vv05g1112 . . . . . . .
. . . . . . . .
. . . . .
Vv05g1113 Tc04_g021210 . . . Gorai.
011G157600.1 . . . . . . .
. . . . . . . .

Vv05g1114 Tc04_g021200 Gorai.006G222600.1 . .
Gorai.011G157500.1 . . . . .
. . . . . . . .
. .
Vv05g1115 . . . . . . .
. . . . . . . .
. . . . .
Vv05g1116 . . . . Gorai.011G156700.1
. . . . . . . .
. . . . . . .
Vv05g1117 . . . . . . .
. . . . . . . .
. . . . .
Vv05g1118 . . . . . . .
. . . . . . . .
. . . . .
Vv05g1119 . . . . . . .
. . . . . . . .
. . . . .
Vv05g1120 . . . . . . .
. . . . . . . .
. . . . .
Vv05g1121 . . . . . . .
. . . . . . . .
. . . . .
Vv05g1122 . . . . . . .
. . . . . . . .
. . . . .
Vv05g1123 . . . . . . .
. . . . . . . .
. . . . .
Vv05g1124 . . . . . . .
. . . . . . . .
. . . . .
Vv05g1125 . . . . . . .
. . . . . . . .
. . . . .
Vv05g1126 Tc04_g021150 . Gorai.005G032500.1 .
. . . . . . . .
. . . . . . . .
Vv05g1127 Tc04_g021140 Gorai.006G222700.1 Gorai.
005G032400.1 . . . . . . .
. . . . . . . .
. .
Vv05g1128 . . Gorai.005G029800.1 . .
. . . . . . . .
. . . . . . .
Vv05g1129 . . . . . . .
. . . . . . . .
. . . . .

Vv05g1130 . . . . . . .
. . . . . . . .
. . . . .
Vv05g1131 . . . . . . .
. . . . . . . .
. . . . .
Vv05g1132 . . Gorai.005G027000.1 . .
. . . . . . . .
. . . . . . .
Vv05g1133 . . . . . . .
. . . . . . . .
. . . . .
Vv05g1134 . . . . . . .
. . . . . . . .
. . . . .
Vv05g1135 . . . . . . .
. . . . . . . .
. . . . .
Vv05g1136 . . . . . . .
. . . . . . . .
. . . . .
Vv05g1137 . Gorai.006G223800.1 . . .
. . . . . . . .
. . . . . . .
Vv05g1138 . . . . . . .
. . . . . . . .
. . . . .
Vv05g1139 . . . . . . .
. . . . . . . .
. . . . .
Vv05g1140 . . . . . . .
. . . . . . . .
. . . . .
Vv05g1141 . . . . . . .
. . . . . . . .
. . . . .
Vv05g1142 . . . . . . .
. . . . . . . .
. . . . .
Vv05g1143 . Gorai.006G223900.1 . .
Gorai.011G154000.1 . . . . . .
. . . . . . . .
.
Vv05g1144 . . . . . . .
. . . . . . . .
. . . . .
Vv05g1145 . . . . . . .
. . . . . . . .
. . . . .
Vv05g1146 . . . . . . .

. . . . . . . .
. . . . .
Vv05g1147 . . . . . . .
. . . . . . . .
. . . . .
Vv05g1148 . Gorai.006G224000.1 Gorai.005G027500.1
. . . . . . . .
. . . . . . . .
.
Vv05g1149 Tc04_g028080 . Gorai.005G027600.1 .
. . . . . . . .
. . . . . . . .
Vv05g1150 . . . . . . .
. . . . . . . .
. . . . .
Vv05g1151 . . . . . . .
. . . . . . . .
. . . . .
Vv05g1152 . . . . . . .
. . . . . . . .
. . . . .
Vv05g1153 . Gorai.006G225700.1 . . .
. . . . . . . .
. . . . . . .
Vv05g1154 Tc04_g028190 . . Gorai.006G076100.1
. . . . . . . .
. . . . . . . .
Vv05g1155 . . . . . . .
. . . . . . . .
. . . . .
Vv05g1156 . . . . . . .
. . . . . . . .
. . . . .
Vv05g1157 Tc04_g028300 . Gorai.005G028000.1 .
Gorai.011G153500.1 . . . . .
. . . . . . . .
. .
Vv05g1158 . . . . . . .
. . . . . . . .
. . . . .
Vv05g1159 Tc04_g020940 . . . . .
. . . . . . . .
. . . . . .
Vv05g1160 . . . . . . .
. . . . . . . .
. . . . .
Vv05g1161 . . Gorai.005G028200.1 .
Gorai.011G153200.1 . . . . . . .
. . . . . . . .
.

| | | | | | | | | | | | | | | | | | | | | |
|---|---|---|---|---|---|---|---|---|---|---|---|---|---|---|---|---|---|---|---|---|
| Vv05g1162 | . | . | . | . | . | . | . | . | . | . | . | . | . | . | . | . | . | . | . | . |
| Vv05g1163 | . | . | . | . | . | . | . | . | . | . | . | . | . | . | . | . | . | . | . | . |
| Vv05g1164 | . | . | Gorai.005G028300.1 | . | . | . | . | . | . | . | . | . | . | . | . | . | . | . | . | . |
| Vv05g1165 | . | . | . | . | . | . | . | . | . | . | . | . | . | . | . | . | . | . | . | . |
| Vv05g1166 | . | . | . | . | Gorai.011G152900.1 | . | . | . | . | . | . | . | . | . | . | . | . | . | . | . |
| Vv05g1167 | . | . | . | . | . | . | . | . | . | . | . | . | . | . | . | . | . | . | . | . |
| Vv05g1168 | . | . | . | . | . | . | . | . | . | . | . | . | . | . | . | . | . | . | . | . |
| Vv05g1169 | . | . | . | . | . | . | . | . | . | . | . | . | . | . | . | . | . | . | . | . |
| Vv05g1170 | . | . | . | . | . | . | . | . | . | . | . | . | . | . | . | . | . | . | . | . |
| Vv05g1171 | . | . | . | . | . | . | . | . | . | . | . | . | . | . | . | . | . | . | . | . |
| Vv05g1172 | . | . | . | . | . | . | . | . | . | . | . | . | . | . | . | . | . | . | . | . |
| Vv05g1173 | . | . | . | . | . | . | . | . | . | . | . | . | . | . | . | . | . | . | . | . |
| Vv05g1174 | . | . | . | . | . | . | . | . | . | . | . | . | . | . | . | . | . | . | . | . |
| Vv05g1175 | . | . | . | . | . | . | . | . | . | . | . | . | . | . | . | . | . | . | . | . |
| Vv05g1176 | . | . | Gorai.005G028500.1 | . | . | . | . | . | . | . | . | . | . | . | . | . | . | . | . | . |
| Vv05g1177 | . | . | . | . | . | . | . | . | . | . | . | . | . | . | . | . | . | . | . | . |
| Vv05g1178 | . | . | . | . | . | . | . | . | . | . | . | . | . | . | . | | | | | |

| | | | | | | | |
|---|---|---|---|---|---|---|---|
| . | . | . | . | . | | | |
| Vv05g1179 | . | . | . | . | Gorai.011G150100.1 | | |
| . | . | . | . | . | . | . | . |
| . | . | . | . | . | . | . | |
| Vv05g1180 | . | . | . | . | . | . | . |
| . | . | . | . | . | . | . | . |
| . | . | . | . | . | | | |
| Vv05g1181 | . | . | . | . | . | . | . |
| . | . | . | . | . | . | . | . |
| . | . | . | . | . | | | |
| Vv05g1182 | . | . | . | . | . | . | . |
| . | . | . | . | . | . | . | . |
| . | . | . | . | . | | | |
| Vv05g1183 | Tc04_g020750 | | . | Gorai.005G030200.1 | | | . |
| . | . | . | . | . | . | . | . |
| . | . | . | . | . | . | . | . |
| Vv05g1184 | . | . | . | . | . | . | . |
| . | . | . | . | . | . | . | . |
| . | . | . | . | . | | | |
| Vv05g1185 | . | . | . | . | . | . | . |
| . | . | . | . | . | . | . | . |
| . | . | . | . | . | | | |
| Vv05g1186 | . | . | . | . | . | . | . |
| . | . | . | . | . | . | . | . |
| . | . | . | . | . | | | |
| Vv05g1187 | Tc04_g012170 | | . | . | . | . | . |
| . | . | . | . | . | . | . | . |
| . | . | . | . | . | . | | |
| Vv05g1188 | Tc04_g012120 | | . | . | . | . | . |
| . | . | . | . | . | . | . | . |
| . | . | . | . | . | . | | |
| Vv05g1189 | Tc04_g012080 | | . | . | . | . | . |
| . | . | . | . | . | . | . | . |
| . | . | . | . | . | . | | |
| Vv05g1190 | . | . | . | . | . | . | . |
| . | . | . | . | . | . | . | . |
| . | . | . | . | . | | | |
| Vv05g1191 | . | . | . | . | . | . | . |
| . | . | . | . | . | . | . | . |
| . | . | . | . | . | | | |
| Vv05g1192 | Tc04_g017220 | | . | . | . | . | . |
| . | . | . | . | . | . | . | . |
| . | . | . | . | . | . | | |
| Vv05g1193 | . | . | . | . | . | . | . |
| . | . | . | . | . | . | . | . |
| . | . | . | . | . | | | |
| Vv05g1194 | Tc04_g017230 | | . | . | . | . | . |
| . | . | . | . | . | . | . | . |
| . | . | . | . | . | . | | |
| Vv05g1195 | . | . | . | . | . | . | . |

| | | | | | | | |
|---|---|---|---|---|---|---|---|
| . | . | . | . | . | . | . | . |
| . | . | . | . | . | | | |
| Vv05g1196 | . | . | . | . | . | . | . |
| . | . | . | . | . | . | . | . |
| . | . | . | . | . | | | |
| Vv05g1197 | Tc04_g020430 | | . | . | . | . | . |
| . | . | . | . | . | . | . | . |
| . | . | . | . | . | . | | |
| Vv05g1198 | . | . | . | . | . | . | . |
| . | . | . | . | . | . | . | . |
| . | . | . | . | . | | | |
| Vv05g1199 | . | . | . | . | . | . | . |
| . | . | . | . | . | . | . | . |
| . | . | . | . | . | | | |
| Vv05g1200 | . | . | . | . | . | . | . |
| . | . | . | . | . | . | . | . |
| . | . | . | . | . | | | |
| Vv05g1201 | . | . | . | . | . | . | . |
| . | . | . | . | . | . | . | . |
| . | . | . | . | . | | | |
| Vv05g1202 | . | . | . | . | . | . | . |
| . | . | . | . | . | . | . | . |
| . | . | . | . | . | | | |
| Vv05g1203 | . | . | . | . | . | . | . |
| . | . | . | . | . | . | . | . |
| . | . | . | . | . | | | |
| Vv05g1204 | . | . | . | . | . | . | . |
| . | . | . | . | . | . | . | . |
| . | . | . | . | . | | | |
| Vv05g1205 | . | . | . | . | . | . | . |
| . | . | . | . | . | . | . | . |
| . | . | . | . | . | | | |
| Vv05g1206 | . | . | . | . | . | . | . |
| . | . | . | . | . | . | . | . |
| . | . | . | . | . | | | |
| Vv05g1207 | . | . | . | . | . | . | . |
| . | . | . | . | . | . | . | . |
| . | . | . | . | . | | | |
| Vv05g1208 | . | . | . | . | . | . | . |
| . | . | . | . | . | . | . | . |
| . | . | . | . | . | | | |
| Vv05g1209 | . | . | . | . | . | . | . |
| . | . | . | . | . | . | . | . |
| . | . | . | . | . | | | |
| Vv05g1210 | . | . | . | . | . | . | . |
| . | . | . | . | . | . | . | . |
| . | . | . | . | . | | | |
| Vv05g1211 | . | . | . | . | . | . | . |
| . | . | . | . | . | . | . | . |
| . | . | . | . | . | | | |

Vv05g1212 . . . . . . .
. . . . . . . .
. . . . .
Vv05g1213 . . . . . . .
. . . . . . . .
. . . . .
Vv05g1214 . . . . . . .
. . . . . . . .
. . . . .
Vv05g1215 . . . . . . .
. . . . . . . .
. . . . .
Vv05g1216 . . . . . . .
. . . . . . . .
. . . . .
Vv05g1217 . . . . . . .
. . . . . . . .
. . . . .
Vv05g1218 . . . . . . .
. . . . . . . .
. . . . .
Vv05g1219 . . . . . . .
. . . . . . . .
. . . . .
Vv05g1220 . . . . . . .
. . . . . . . .
. . . . .
Vv05g1221 . . . . . . .
. . . . . . . .
. . . . .
Vv05g1222 . . . . . . .
. . . . . . . .
. . . . .
Vv05g1223 . . . . . . .
. . . . . . . .
. . . . .
Vv05g1224 . . . . . . .
. . . . . . . .
. . . . .
Vv05g1225 . . . . . . .
. . . . . . . .
. . . . .
Vv05g1226 . . . . . . .
. . . . . . . .
. . . . .
Vv05g1227 . . . . . . .
. . . . . . . .
. . . . .
Vv05g1228 . . . . . . .
. . . . . . . .

| | | | | | | | |
|---|---|---|---|---|---|---|---|
| . | . | . | . | . | | | |
| Vv05g1229 | . | . | . | . | . | . | . |
| . | . | . | . | . | . | . | . |
| . | . | . | . | . | | | |
| Vv05g1230 | . | . | . | . | . | . | . |
| . | . | . | . | . | . | . | . |
| . | . | . | . | . | | | |
| Vv05g1231 | Tc04_g017430 | | . | . | . | . | . |
| . | . | . | . | . | . | . | . |
| . | . | . | . | . | . | | |
| Vv05g1232 | Tc04_g020330 | | . | Gorai.005G026900.1 | | | . |
| . | . | . | . | . | . | . | . |
| . | . | . | . | . | . | . | . |
| Vv05g1233 | . | . | . | . | . | . | . |
| . | . | . | . | . | . | . | . |
| . | . | . | . | . | | | |
| Vv05g1234 | . | . | . | . | . | . | . |
| . | . | . | . | . | . | . | . |
| . | . | . | . | . | | | |
| Vv05g1235 | . | . | . | . | . | . | . |
| . | . | . | . | . | . | . | . |
| . | . | . | . | . | | | |
| Vv05g1236 | . | . | . | . | . | . | . |
| . | . | . | . | . | . | . | . |
| . | . | . | . | . | | | |
| Vv05g1237 | . | . | . | . | . | . | . |
| . | . | . | . | . | . | . | . |
| . | . | . | . | . | | | |
| Vv05g1238 | . | . | . | . | . | . | . |
| . | . | . | . | . | . | . | . |
| . | . | . | . | . | | | |
| Vv05g1239 | . | . | . | . | . | . | . |
| . | . | . | . | . | . | . | . |
| . | . | . | . | . | | | |
| Vv05g1240 | . | . | . | . | . | . | . |
| . | . | . | . | . | . | . | . |
| . | . | . | . | . | | | |
| Vv05g1241 | . | . | . | . | . | . | . |
| . | . | . | . | . | . | . | . |
| . | . | . | . | . | | | |
| Vv05g1242 | . | . | . | . | . | . | . |
| . | . | . | . | . | . | . | . |
| . | . | . | . | . | | | |
| Vv05g1243 | . | . | . | . | . | . | . |
| . | . | . | . | . | . | . | . |
| . | . | . | . | . | | | |
| Vv05g1244 | . | . | . | . | . | . | . |
| . | . | . | . | . | . | . | . |
| . | . | . | . | . | | | |
| Vv05g1245 | . | . | . | . | . | . | . |

. . . . . . . .
. . . . .
Vv05g1246 . . . . . . .
. . . . . . . .
. . . . .
Vv05g1247 . . . . . . .
. . . . . . . .
. . . . .
Vv05g1248 . . . . . . .
. . . . . . . .
. . . . .
Vv05g1249 . . . . . . .
. . . . . . . .
. . . . .
Vv05g1250 Tc04_g015630 . . . . .
. . . . . . . .
. . . . . .
Vv05g1251 . . . . . . .
. . . . . . . .
. . . . .
Vv05g1252 . . . . . . .
. . . . . . . .
. . . . .
Vv05g1253 . . . . . . .
. . . . . . . .
. . . . .
Vv05g1254 . . . . . . .
. . . . . . . .
. . . . .
Vv05g1255 . . . . . . .
. . . . . . . .
. . . . .
Vv05g1256 . . . . . . .
. . . . . . . .
. . . . .
Vv05g1257 . . . . . . .
. . . . . . . .
. . . . .
Vv05g1258 . . . . . . .
. . . . . . . .
. . . . .
Vv05g1259 . . . . . . .
. . . . . . . .
. . . . .
Vv05g1260 . . . . . . .
. . . . . . . .
. . . . .
Vv05g1261 Tc04_g020280 . . . . .
. . . . . . . .
. . . . . .

Vv05g1262 . . . . . . .
. . . . . . . .
. . . . .
Vv05g1263 Tc04_g020270 . . . . .
. . . . . . . .
. . . . . .
Vv05g1264 . . . . . . .
. . . . . . . .
. . . . .
Vv05g1265 Tc04_g020200 . Gorai.005G025000.1 .
. . . . . . . .
. . . . . . . .
Vv05g1266 . . . . . . .
. . . . . . . .
. . . . .
Vv05g1267 . . . . . . .
. . . . . . . .
. . . . .
Vv05g1268 . . . . . . .
. . . . . . . .
. . . . .
Vv05g1269 . . . . . . .
. . . . . . . .
. . . . .
Vv05g1270 . . . . . . .
. . . . . . . .
. . . . .
Vv05g1271 . . . . . . .
. . . . . . . .
. . . . .
Vv05g1272 . . . . . . .
. . . . . . . .
. . . . .
Vv05g1273 . . . . . . .
. . . . . . . .
. . . . .
Vv05g1274 . . . . . . .
. . . . . . . .
. . . . .
Vv05g1275 . . . . . . .
. . . . . . . .
. . . . .
Vv05g1276 . . . . . . .
. . . . . . . .
. . . . .
Vv05g1277 . . . . . . .
. . . . . . . .
. . . . .
Vv05g1278 . . . . . . .
. . . . . . . .

. . . . .
Vv05g1279 . . . . . . .
. . . . . . . .
. . . . .
Vv05g1280 . . . . . . .
. . . . . . . .
. . . . .
Vv05g1281 . . . . . . .
. . . . . . . .
. . . . .
Vv05g1282 . . . . . . .
. . . . . . . .
. . . . .
Vv05g1283 Tc04_g016050 . . . . .
. . . . . . . .
. . . . . .
Vv05g1284 . . . . . . .
. . . . . . . .
. . . . .
Vv05g1285 . Gorai.006G226200.1 Gorai.005G021000.1
. . . . . . . .
. . . . . . . .
.
Vv05g1286 Tc04_g016470 Gorai.006G226100.1 Gorai.
005G021100.1 . . . . . . .
. . . . . . . .
. .
Vv05g1287 . . . . . . .
. . . . . . . .
. . . . .
Vv05g1288 . . . . . . .
. . . . . . . .
. . . . .
Vv05g1289 . . . . . . .
. . . . . . . .
. . . . .
Vv05g1290 Tc04_g018240 Gorai.006G225900.1 . .
. . . . . . . .
. . . . . . . .
Vv05g1291 . . . . . . .
. . . . . . . .
. . . . .
Vv05g1292 . . . . . . .
. . . . . . . .
. . . . .
Vv05g1293 . . Gorai.005G021300.1 . .
. . . . . . . .
. . . . . . .
Vv05g1294 . . . . . . .
. . . . . . . .

. . . . .
Vv05g1295 . . . . . . .
. . . . . . . .
. . . . .
Vv05g1296 . . Gorai.005G022200.1 . .
. . . . . . . .
. . . . . . .
Vv05g1297 . . . . . . .
. . . . . . . .
. . . . .
Vv05g1298 . . . . . . .
. . . . . . . .
. . . . .
Vv05g1299 . . . . . . .
. . . . . . . .
. . . . .
Vv05g1300 . . . . . . .
. . . . . . . .
. . . . .
Vv05g1301 . . . . . . .
. . . . . . . .
. . . . .
Vv05g1302 . . . . . . .
. . . . . . . .
. . . . .
Vv05g1303 . . . . . . .
. . . . . . . .
. . . . .
Vv05g1304 Tc04_g018280 . . . Gorai.
011G147600.1 . . . . . . .
. . . . . . . .
Vv05g1305 . . . . . . .
. . . . . . . .
. . . . .
Vv05g1306 . . . . . . .
. . . . . . . .
. . . . .
Vv05g1307 . . . . . . .
. . . . . . . .
. . . . .
Vv05g1308 Tc04_g018290 . . . Gorai.
011G147400.1 . . . . . . .
. . . . . . . .
Vv05g1309 Tc04_g018330 . . . . .
. . . . . . . .
. . . . . .
Vv05g1310 . Gorai.006G224200.1 . . .
. . . . . . . .
. . . . . . .
Vv05g1311 . . . . . . .

. . . . . . . .
. . . . .
Vv05g1312 . . . . . . .
. . . . . . . .
. . . . .
Vv05g1313 . . . . . . .
. . . . . . . .
. . . . .
Vv05g1314 . . . . . . .
. . . . . . . .
. . . . .
Vv05g1315 Tc04_g018340 Gorai.006G221200.1 . .
. . . . . . . .
. . . . . . . .
Vv05g1316 . . . . . . .
. . . . . . . .
. . . . .
Vv05g1317 . . . . . . .
. . . . . . . .
. . . . .
Vv05g1318 Tc04_g018370 . . . . .
. . . . . . . .
. . . . . .
Vv05g1319 Tc04_g018390 . . . . .
. . . . . . . .
. . . . . .
Vv05g1320 Tc04_g018400 . . . Gorai.
011G147300.1 . . . . . . .
. . . . . . . .
Vv05g1321 Tc04_g018430 . Gorai.005G020900.1
Gorai.005G127200.1 . . . . . .
. . . . . . . .
. .
Vv05g1322 Tc04_g018440 . . . . .
. . . . . . . .
. . . . . .
Vv05g1323 Tc04_g018450 . . Gorai.005G127800.1
Gorai.011G147000.1 . . . . .
. . . . . . . .
. .
Vv05g1324 Tc04_g018460 . . . . .
. . . . . . . .
. . . . . .
Vv05g1325 Tc04_g018470 . . . . .
. . . . . . . .
. . . . . .
Vv05g1326 . . . . . . .
. . . . . . . .
. . . . .
Vv05g1327 . . . . . . .

. . . . . . . .
. . . . .
Vv05g1328 . . . . . . .
. . . . . . . .
. . . . .
Vv05g1329 . . . . . . .
. . . . . . . .
. . . . .
Vv05g1330 . . . . . . .
. . . . . . . .
. . . . .
Vv05g1331 . . . . . . .
. . . . . . . .
. . . . .
Vv05g1332 . . . . . . .
. . . . . . . .
. . . . .
Vv05g1333 . . . . . . .
. . . . . . . .
. . . . .
Vv05g1334 . . . . . . .
. . . . . . . .
. . . . .
Vv05g1335 . . . . . . .
. . . . . . . .
. . . . .
Vv05g1336 . . . . . . .
. . . . . . . .
. . . . .
Vv05g1337 . . . . . . .
. . . . . . . .
. . . . .
Vv05g1338 . . . . Gorai.011G143900.1
. . . . . . . .
. . . . . . .
Vv05g1339 . . . . Gorai.011G144400.1
. . . . . . . .
. . . . . . .
Vv05g1340 . . . . Gorai.011G144300.1
. . . . . . . .
. . . . . . .
Vv05g1341 Tc04_g018700 . . . . .
. . . . . . . .
. . . . . .
Vv05g1342 . . . Gorai.005G128400.1
Gorai.011G144200.1 . . . . . .
. . . . . . . .
.
Vv05g1343 . . . Gorai.005G128500.1
Gorai.011G141500.1 . . . . . .

| | | | | | | | |
|---|---|---|---|---|---|---|---|
| . | . | . | . | . | . | . | . |
| . | | | | | | | |
| Vv05g1344 | . | . | . | . | . | . | . |
| . | . | . | . | . | . | . | . |
| . | . | . | . | . | | | |
| Vv05g1345 | . | . | . | . | . | . | . |
| . | . | . | . | . | . | . | . |
| . | . | . | . | . | | | |
| Vv05g1346 | . | . | . | . | . | . | . |
| . | . | . | . | . | . | . | . |
| . | . | . | . | . | | | |
| Vv05g1347 | Tc04_g020280 | | . | . | . | . | . |
| . | . | . | . | . | . | . | . |
| . | . | . | . | . | . | | |
| Vv05g1348 | . | . | . | . | . | . | . |
| . | . | . | . | . | . | . | . |
| . | . | . | . | . | | | |
| Vv05g1349 | Tc04_g020650 | | . | . | . | . | . |
| . | . | . | . | . | . | . | . |
| . | . | . | . | . | . | | |
| Vv05g1350 | . | . | . | . | . | . | . |
| . | . | . | . | . | . | . | . |
| . | . | . | . | . | | | |
| Vv05g1351 | . | . | . | . | . | . | . |
| . | . | . | . | . | . | . | . |
| . | . | . | . | . | | | |
| Vv05g1352 | . | . | . | . | . | . | . |
| . | . | . | . | . | . | . | . |
| . | . | . | . | . | | | |
| Vv05g1353 | . | . | . | . | . | . | . |
| . | . | . | . | . | . | . | . |
| . | . | . | . | . | | | |
| Vv05g1354 | . | . | . | . | . | . | . |
| . | . | . | . | . | . | . | . |
| . | . | . | . | . | | | |
| Vv05g1355 | . | . | . | . | . | . | . |
| . | . | . | . | . | . | . | . |
| . | . | . | . | . | | | |
| Vv05g1356 | . | . | . | . | . | . | . |
| . | . | . | . | . | . | . | . |
| . | . | . | . | . | | | |
| Vv05g1357 | . | . | . | . | . | . | . |
| . | . | . | . | . | . | . | . |
| . | . | . | . | . | | | |
| Vv05g1358 | . | . | . | . | . | . | . |
| . | . | . | . | . | . | . | . |
| . | . | . | . | . | | | |
| Vv05g1359 | Tc04_g018740 | | . | . | . | . | . |
| . | . | . | . | . | . | . | . |
| . | . | . | . | . | . | | |

Vv05g1360 Tc04_g020680 . . . . .
. . . . . . . .
. . . . . .
Vv05g1361 . . . . . . .
. . . . . . . .
. . . . .
Vv05g1362 . . . . . . .
. . . . . . . .
. . . . .
Vv05g1363 . . . . . . .
. . . . . . . .
. . . . .
Vv05g1364 Tc04_g018820 . . . . .
. . . . . . . .
. . . . . .
Vv05g1365 . . . . . . .
. . . . . . . .
. . . . .
Vv05g1366 Tc04_g018830 . . . . .
. . . . . . . .
. . . . . .
Vv05g1367 Tc04_g018840 . . . . .
. . . . . . . .
. . . . . .
Vv05g1368 Tc04_g020940 . . . . .
. . . . . . . .
. . . . . .
Vv05g1369 . . . . . . .
. . . . . . . .
. . . . .
Vv05g1370 . . . . . . .
. . . . . . . .
. . . . .
Vv05g1371 Tc04_g018880 . . . . .
. . . . . . . .
. . . . . .
Vv05g1372 Tc04_g018890 . . . . .
. . . . . . . .
. . . . . .
Vv05g1373 . . . . . . .
. . . . . . . .
. . . . .
Vv05g1374 . . . . . . .
. . . . . . . .
. . . . .
Vv05g1375 . . . . . . .
. . . . . . . .
. . . . .
Vv05g1376 Tc04_g018900 . . . . .
. . . . . . . .

. . . . . .
Vv05g1377 Tc04_g018910 . . . . .
. . . . . . . .
. . . . . .
Vv05g1378 . . . . . . .
. . . . . . . .
. . . . .
Vv05g1379 . . . . . . .
. . . . . . . .
. . . . .
Vv05g1380 Tc04_g018930 . . . Gorai.
011G144600.1 . . . . . . .
. . . . . . . .
Vv05g1381 Tc04_g019000 . . . . .
. . . . . . . .
. . . . . .
Vv05g1382 Tc04_g014440 . . . . .
. . . . . . . .
. . . . . .
Vv05g1383 Tc04_g014570 . . . . .
. . . . . . . .
. . . . . .
Vv05g1384 . . . . . . .
. . . . . . . .
. . . . .
Vv05g1385 . . . . . . .
. . . . . . . .
. . . . .
Vv05g1386 . . . . . . .
. . . . . . . .
. . . . .
Vv05g1387 . . . . Gorai.011G145300.1
. . . . . . . .
. . . . . . .
Vv05g1388 . . . . . . .
. . . . . . . .
. . . . .
Vv05g1389 . . . . . . .
. . . . . . . .
. . . . .
Vv05g1390 . . . . . . .
. . . . . . . .
. . . . .
Vv05g1391 . . . . . . .
. . . . . . . .
. . . . .
Vv05g1392 . . . . . . .
. . . . . . . .
. . . . .
Vv05g1393 . . . . . . .

| | | | | | | | | |
|---|---|---|---|---|---|---|---|---|
| | . | . | . | . | . | . | . | . |
| | . | . | . | . | . | | | |
| Vv05g1394 | | . | . | . | . | . | . | . |
| | . | . | . | . | . | . | . | . |
| | . | . | . | . | . | | | |
| Vv05g1395 | | . | . | . | . | . | . | . |
| | . | . | . | . | . | . | . | . |
| | . | . | . | . | . | | | |
| Vv05g1396 | | . | . | . | . | Gorai.011G145500.1 | | |
| | . | . | . | . | . | . | . | . |
| | . | . | . | . | . | . | . | |
| Vv05g1397 | | . | . | . | . | . | . | . |
| | . | . | . | . | . | . | . | . |
| | . | . | . | . | . | | | |
| Vv05g1398 | | . | . | . | . | . | . | . |
| | . | . | . | . | . | . | . | . |
| | . | . | . | . | . | | | |
| Vv05g1399 | | . | . | . | . | . | . | . |
| | . | . | . | . | . | . | . | . |
| | . | . | . | . | . | | | |
| Vv05g1400 | | . | . | . | . | . | . | . |
| | . | . | . | . | . | . | . | . |
| | . | . | . | . | . | | | |
| Vv05g1401 | | Tc04_g024840 | | . | . | . | . | . |
| | . | . | . | . | . | . | . | . |
| | . | . | . | . | . | . | | |
| Vv05g1402 | | . | . | . | . | . | . | . |
| | . | . | . | . | . | . | . | . |
| | . | . | . | . | . | | | |
| Vv05g1403 | | . | . | . | . | . | . | . |
| | . | . | . | . | . | . | . | . |
| | . | . | . | . | . | | | |
| Vv05g1404 | | . | . | . | . | . | . | . |
| | . | . | . | . | . | . | . | . |
| | . | . | . | . | . | | | |
| Vv05g1405 | | . | . | . | . | . | . | . |
| | . | . | . | . | . | . | . | . |
| | . | . | . | . | . | | | |
| Vv05g1406 | | . | . | . | . | . | . | . |
| | . | . | . | . | . | . | . | . |
| | . | . | . | . | . | | | |
| Vv05g1407 | | . | . | . | . | . | . | . |
| | . | . | . | . | . | . | . | . |
| | . | . | . | . | . | | | |
| Vv05g1408 | | . | . | . | . | . | . | . |
| | . | . | . | . | . | . | . | . |
| | . | . | . | . | . | | | |
| Vv05g1409 | | . | . | . | . | . | . | . |
| | . | . | . | . | . | . | . | . |
| | . | . | . | . | . | | | |

Vv05g1410 . . . . . . .
. . . . . . . .
. . . . .
Vv05g1411 . . . . . . .
. . . . . . . .
. . . . .
Vv05g1412 . . . . . . .
. . . . . . . .
. . . . .
Vv05g1413 . . . . . . .
. . . . . . . .
. . . . .
Vv05g1414 . Gorai.006G186500.1 . . .
. . . . . . . .
. . . . . . .
Vv05g1415 . . . . . . .
. . . . . . . .
. . . . .
Vv05g1416 . . . . . . .
. . . . . . . .
. . . . .
Vv05g1417 . . . . . . .
. . . . . . . .
. . . . .
Vv05g1418 . . . . . . .
. . . . . . . .
. . . . .
Vv05g1419 Tc04_g014820 . . . . .
. . . . . . . .
. . . . . .
Vv05g1420 Tc04_g014820 . . . . .
. . . . . . . .
. . . . . .
Vv05g1421 . . . . . . .
. . . . . . . .
. . . . .
Vv05g1422 . . . . . . .
. . . . . . . .
. . . . .
Vv05g1423 . . . . . . .
. . . . . . . .
. . . . .
Vv05g1424 . . . . . . .
. . . . . . . .
. . . . .
Vv05g1425 . . . . . . .
. . . . . . . .
. . . . .
Vv05g1426 . . . . . . .
. . . . . . . .

. . . . .
Vv05g1427 . . . . . . .
. . . . . . . .
. . . . .
Vv05g1428 . . . . . . .
. . . . . . . .
. . . . .
Vv05g1429 . . . . . . .
. . . . . . . .
. . . . .
Vv05g1430 . . . . . . .
. . . . . . . .
. . . . .
Vv05g1431 Tc04_g024710 . . . . .
. . . . . . . .
. . . . . .
Vv05g1432 Tc04_g024690 . . . . .
. . . . . . . .
. . . . . .
Vv05g1433 . . . . . . .
. . . . . . . .
. . . . .
Vv05g1434 . . . . . . .
. . . . . . . .
. . . . .
Vv05g1435 . . . . . . .
. . . . . . . .
. . . . .
Vv05g1436 . . . . . . .
. . . . . . . .
. . . . .
Vv05g1437 . . . . . . .
. . . . . . . .
. . . . .
Vv05g1438 . . . . . . .
. . . . . . . .
. . . . .
Vv05g1439 . . . . . . .
. . . . . . . .
. . . . .
Vv05g1440 . Gorai.006G186400.1 . . .
. . . . . . . .
. . . . . . .
Vv05g1441 . Gorai.006G186300.1 . . .
. . . . . . . .
. . . . . . .
Vv05g1442 . . . . . . .
. . . . . . . .
. . . . .
Vv05g1443 Tc04_g014980 Gorai.006G186200.1 . .

| | | | | | | | |
|---|---|---|---|---|---|---|---|
| Vv05g1444 | . | . | . | . | . | . | . |
| Vv05g1445 | . | Gorai.006G185900.1 | | | . | . | . |
| Vv05g1446 | . | . | . | . | . | . | . |
| Vv05g1447 | . | . | . | . | . | . | . |
| Vv05g1448 | Tc04_g014440 | | . | . | . | . | . |
| Vv05g1449 | . | Gorai.006G227400.1 | | | Gorai.005G019700.1 | | |
| Vv05g1450 | . | . | . | . | . | . | . |
| Vv05g1451 | . | . | . | . | . | . | . |
| Vv05g1452 | . | . | . | . | . | . | . |
| Vv05g1453 | . | . | . | . | . | . | . |
| Vv05g1454 | . | . | . | . | . | . | . |
| Vv05g1455 | . | . | . | . | . | . | . |
| Vv05g1456 | . | . | . | . | . | . | . |
| Vv05g1457 | . | . | . | . | . | . | . |
| Vv05g1458 | . | . | . | . | . | . | . |
| Vv05g1459 | . | . | . | . | . | . | . |

. . . . .
Vv05g1460 . Gorai.006G228200.1 . .
Gorai.011G143200.1 . . . . . .
. . . . . . . .
.
Vv05g1461 . . . . Gorai.011G143100.1
. . . . . . . .
. . . . . . .
Vv05g1462 . . . . Gorai.011G143000.1
. . . . . . . .
. . . . . . .
Vv05g1463 . Gorai.006G228300.1 . .
Gorai.011G142800.1 . . . . . .
. . . . . . . .
.
Vv05g1464 . . . . Gorai.011G142600.1
. . . . . . . .
. . . . . . .
Vv05g1465 Tc04_g012740 . . . . .
. . . . . . . .
. . . . . .
Vv05g1466 . . . . . . .
. . . . . . . .
. . . . .
Vv05g1467 . . . . . . .
. . . . . . . .
. . . . .
Vv05g1468 . . . . . . .
. . . . . . . .
. . . . .
Vv05g1469 . . . . . . .
. . . . . . . .
. . . . .
Vv05g1470 . . . . . . .
. . . . . . . .
. . . . .
Vv05g1471 Tc04_g014180 . Gorai.005G016900.1 .
Gorai.011G142200.1 . . . . .
. . . . . . . .
. .
Vv05g1472 . . . . . . .
. . . . . . . .
. . . . .
Vv05g1473 . . . . . . .
. . . . . . . .
. . . . .
Vv05g1474 Tc04_g001390 . . . . .
. . . . . . . .
. . . . . .
Vv05g1475 . . . . . . .

| | | | | | | | |
|---|---|---|---|---|---|---|---|
| Vv05g1476 | . | . | . | . | . | . | . |
| Vv05g1477 | . | . | . | . | . | . | . |
| Vv05g1478 | . | . | . | . | . | . | . |
| Vv05g1479 | . | . | . | . | . | . | . |
| Vv05g1480 | . | . | . | . | . | . | . |
| Vv05g1481 | . | . | . | . | . | . | . |
| Vv05g1482 | . | . | . | . | . | . | . |
| Vv05g1483 | . | . | Gorai.005G016800.1 | | | . | . |
| Vv05g1484 | . | . | . | . | . | . | . |
| Vv05g1485 | . | . | . | . | . | . | . |
| Vv05g1486 | . | . | . | . | . | . | . |
| Vv05g1487 | Tc04_g001590 | | . | . | . | . | . |
| Vv05g1488 | . | . | . | . | . | . | . |
| Vv05g1489 | . | . | . | . | . | . | . |
| Vv05g1490 | . | . | . | . | . | . | . |
| Vv05g1491 | . | . | . | . | . | . | . |

Vv05g1492 . . Gorai.005G016700.1 .
Gorai.011G141900.1 . . . . . .
. . . . . . . .
.
Vv05g1493 . . . . . . .
. . . . . . . .
. . . . .
Vv05g1494 Tc04_g001930 Gorai.006G229000.1 . .
. . . . . . . .
. . . . . . . .
Vv05g1495 . Gorai.006G229100.1 . .
Gorai.011G141600.1 . . . . . .
. . . . . . . .
.
Vv05g1496 . . . . Gorai.011G141500.1
. . . . . . . .
. . . . . . .
Vv05g1497 . . Gorai.005G016400.1 . .
. . . . . . . .
. . . . . . .
Vv05g1498 Tc04_g012450 Gorai.006G229300.1 . .
. . . . . . . .
. . . . . . . .
Vv05g1499 . . Gorai.005G016100.1 . .
. . . . . . . .
. . . . . . .
Vv05g1500 . . . . Gorai.011G140800.1
. . . . . . . .
. . . . . . .
Vv05g1501 . . . . . . .
. . . . . . . .
. . . . .
Vv05g1502 . . . . . . .
. . . . . . . .
. . . . .
Vv05g1503 . . Gorai.005G040000.1 . .
. Vv09g0113 . . . . .
. . . . . . . .
Vv05g1504 . . . . . . .
. . . . . . . .
. . . . .
Vv05g1505 . . Gorai.005G039900.1 . .
. Vv09g0112 . . . . .
. . . . . . . .
Vv05g1506 . . . . . . .
. . . . . . . .
. . . . .
Vv05g1507 . . . . . . .
. . . . . . . .
. . . . .

Vv05g1508 . Gorai.006G229400.1 . . .
. . . . . . . .
. . . . . . .
Vv05g1509 . . . . . . .
. . . . . . . .
. . . . .
Vv05g1510 . . Gorai.005G039800.1 . .
. Vv09g0110 . . . . .
. . . . . . . .
Vv05g1511 . . . . . . .
. . . . . . . .
. . . . .
Vv05g1512 . . . . . . .
. . . . . . . .
. . . . .
Vv05g1513 . . Gorai.005G039700.1 . .
. Vv09g0108 . . . . .
. . . . . . . .
Vv05g1514 . . . . . . .
. . . . . . . .
. . . . .
Vv05g1515 . . . . . . .
. . . . . . . .
. . . . .
Vv05g1516 . . Gorai.005G036700.1 . .
. . . . . . . .
. . . . . . .
Vv05g1517 . . . . . . .
. . . . . . . .
. . . . .
Vv05g1518 . . . . . .
Vv09g0107 . . . . . . .
. . . . . .
Vv05g1519 . . . . . .
Vv09g0106 . . . . . . .
. . . . . .
Vv05g1520 . . . . . . .
. . . . . . . .
. . . . .
Vv05g1521 . . . . . . .
. . . . . . . .
. . . . .
Vv05g1522 . . . . . . .
. . . . . . . .
. . . . .
Vv05g1523 . . . . . . .
. . . . . . . .
. . . . .
Vv05g1524 . . . . . . .
. . . . . . . .

| | | | | | | | |
|---|---|---|---|---|---|---|---|
| . | . | . | . | . | | | |
| Vv05g1525 | . | . | . | . | . | . | . |
| . | . | . | . | . | . | . | . |
| . | . | . | . | . | | | |
| Vv05g1526 | . | . | . | . | . | . | . |
| . | . | . | . | . | . | . | . |
| . | . | . | . | . | | | |
| Vv05g1527 | . | . | . | . | . | . | . |
| . | . | . | . | . | . | . | . |
| . | . | . | . | . | | | |
| Vv05g1528 | . | . | . | . | . | . | . |
| . | . | . | . | . | . | . | . |
| . | . | . | . | . | | | |
| Vv05g1529 | Tc04_g002120 | | . | . | . | . | . |
| . | . | . | . | . | . | . | . |
| . | . | . | . | . | . | | |
| Vv05g1530 | . | . | . | . | Gorai.011G140700.1 | | |
| . | . | . | . | . | . | . | . |
| . | . | . | . | . | . | . | |
| Vv05g1531 | . | Gorai.006G229500.1 | | | Gorai.005G015900.1 | | |
| . | Gorai.011G140400.1 | | | . | . | . | . |
| . | . | . | . | . | . | . | . |
| . | . | . | | | | | |
| Vv05g1532 | . | . | . | . | . | . | . |
| . | . | . | . | . | . | . | . |
| . | . | . | . | . | | | |
| Vv05g1533 | . | . | . | . | . | . | |
| Vv09g0105 | Tc06_g010130 | | . | . | . | . | . |
| . | . | . | . | . | . | . | |
| Vv05g1534 | . | . | . | . | . | . | . |
| . | . | . | . | . | . | . | . |
| . | . | . | . | . | | | |
| Vv05g1535 | . | . | Gorai.005G015800.1 | | | . | . |
| . | . | . | . | . | . | . | . |
| . | . | . | . | . | . | . | |
| Vv05g1536 | . | . | . | . | . | . | . |
| . | . | . | . | . | . | . | . |
| . | . | . | . | . | | | |
| Vv05g1537 | . | . | . | . | . | . | . |
| . | . | . | . | . | . | . | . |
| . | . | . | . | . | | | |
| Vv05g1538 | . | . | . | . | . | . | . |
| . | . | . | . | . | . | . | . |
| . | . | . | . | . | | | |
| Vv05g1539 | . | . | . | . | . | . | . |
| . | . | . | . | . | . | . | . |
| . | . | . | . | . | | | |
| Vv05g1540 | . | . | . | . | . | . | . |
| . | . | . | . | . | . | . | . |
| . | . | . | . | . | | | |

Vv05g1541 . . Gorai.005G015700.1 . .
. . . . . . . .
. . . . . . .
Vv05g1542 . . Gorai.005G015600.1 . .
. . . . . . . .
. . . . . . .
Vv05g1543 . Gorai.006G229600.1 Gorai.005G015500.1
. . . . . . . .
. . . . . . . .
.
Vv05g1544 . . Gorai.005G015400.1 . .
. . . . . . . .
. . . . . . .
Vv05g1545 . . Gorai.005G015300.1 . .
. . . . . . . .
. . . . . . .
Vv05g1546 . . . . Gorai.011G139800.1
. . . . . . . .
. . . . . . .
Vv05g1547 . . . . . . .
. . . . . . . .
. . . . .
Vv05g1548 . . . . . . .
. . . . . . . .
. . . . .
Vv05g1549 . Gorai.006G229800.1 Gorai.005G014500.1
. . . . . . . .
. . . . . . . .
.
Vv05g1550 . . . . . . .
. . . . . . . .
. . . . .
Vv05g1551 . . . . . . .
. . . . . . . .
. . . . .
Vv05g1552 . . . . . . .
. . . . . . . .
. . . . .
Vv05g1553 . Gorai.006G229900.1 . . .
. . . . . . . .
. . . . . . .
Vv05g1554 . . . . . . .
. . . . . . . .
. . . . .
Vv05g1555 . . . . . . .
. . . . . . . .
. . . . .
Vv05g1556 . . . . . . .
. . . . . . . .
. . . . .

Vv05g1557 Tc04_g017880 . . . . .
. . . . . . . .
. . . . . .
Vv05g1558 . . . . . . .
. . . . . . . .
. . . . .
Vv05g1559 . . . . Gorai.011G139000.1
. . . . . . . .
. . . . . . .
Vv05g1560 . . . . Gorai.011G138900.1
. . . . . . . .
. . . . . . .
Vv05g1561 Tc04_g017870 . . . Gorai.
011G138800.1 . . . . . . .
. . . . . . . .
Vv05g1562 . . . . . . .
. . . . . . . .
. . . . .
Vv05g1563 . . Gorai.005G013500.1 .
Gorai.011G138700.1 . . . . . .
. . . . . . . .
.
Vv05g1564 . . Gorai.005G013400.1 . .
. . . . . . . .
. . . . . . .
Vv05g1565 Tc04_g017830 Gorai.006G230000.1 . .
. . . . . . . .
. . . . . . . .
Vv05g1566 . . . . . . .
. . . . . . . .
. . . . .
Vv05g1567 . . . . . . .
. . . . . . . .
. . . . .
Vv05g1568 . . . . Gorai.011G138600.1
. . . . . . . .
. . . . . . .
Vv05g1569 Tc04_g002370 . . . . .
. . . . . . . .
. . . . . .
Vv05g1570 . . . . . . .
. . . . . . . .
. . . . .
Vv05g1571 . . . . . . .
. . . . . . . .
. . . . .
Vv05g1572 . . . . . . .
. . . . . . . .
. . . . .
Vv05g1573 . . . . . . .

. . . . . . . .
. . . . .
Vv05g1574 . . . . . . .
. . . . . . . .
. . . . .
Vv05g1575 . . . . Gorai.011G137800.1
. . . . . . . .
. . . . . . .
Vv05g1576 . . . . . . .
. . . . . . . .
. . . . .
Vv05g1577 . . . . . . .
. . . . . . . .
. . . . .
Vv05g1578 . . . . . . .
. . . . . . . .
. . . . .
Vv05g1579 Tc04_g017710 . . . . .
. . . . . . . .
. . . . . .
Vv05g1580 . Gorai.006G230300.1 . .
Gorai.011G137500.1 . . . . . .
. . . . . . . .
.
Vv05g1581 . . . . . . .
. . . . . . . .
. . . . .
Vv05g1582 . . . . . . .
. . . . . . . .
. . . . .
Vv05g1583 . Gorai.006G230400.1 . .
Gorai.011G137300.1 . . . . . .
. . . . . . . .
.
Vv05g1584 . . . . . . .
. . . . . . . .
. . . . .
Vv05g1585 . . . . . . .
. . . . . . . .
. . . . .
Vv05g1586 . Gorai.006G230600.1 . .
Gorai.011G137200.1 . . . . . .
. . . . . . . .
.
Vv05g1587 . . . . . . .
. . . . . . . .
. . . . .
Vv05g1588 . . . . . . .
. . . . . . . .
. . . . .

Vv05g1589 . . . . . . .
. . . . . . . .
. . . . .
Vv05g1590 . Gorai.006G230700.1 . . .
. . . . . . . .
. . . . . . .
Vv05g1591 . . . . . . .
. . . . . . . .
. . . . .
Vv05g1592 . . . . . . .
. . . . . . . .
. . . . .
Vv05g1593 . . . . . . .
. . . . . . . .
. . . . .
Vv05g1594 . . . . . . .
. . . . . . . .
. . . . .
Vv05g1595 . . . . . . .
. . . . . . . .
. . . . .
Vv05g1596 . . . . . . .
. . . . . . . .
. . . . .
Vv05g1597 . . . . . . .
. . . . . . . .
. . . . .
Vv05g1598 . . . . . . .
. . . . . . . .
. . . . .
Vv05g1599 . . . . . . .
. . . . . . . .
. . . . .
Vv05g1600 . . . . . . .
. . . . . . . .
. . . . .
Vv05g1601 . . . . . . .
. . . . . . . .
. . . . .
Vv05g1602 . . . . . . .
. . . . . . . .
. . . . .
Vv05g1603 . . . . . . .
. . . . . . . .
. . . . .
Vv05g1604 . . . . . . .
. . . . . . . .
. . . . .
Vv05g1605 . . . . . . .
. . . . . . . .

. . . . .
Vv05g1606 . . . . . . .
. . . . . . . .
. . . . .
Vv05g1607 . . . . . . .
. . . . . . . .
. . . . .
Vv05g1608 . . . . . . .
. . . . . . . .
. . . . .
Vv05g1609 Tc04_g017690 . . . . .
. . . . . . . .
. . . . . .
Vv05g1610 . . . . . . .
. . . . . . . .
. . . . .
Vv05g1611 Tc04_g017640 . . . Gorai.
011G136700.1 . . . . . . .
. . . . . . . .
Vv05g1612 . . Gorai.005G012500.1 . .
. . . . . . . .
. . . . . . .
Vv05g1613 Tc04_g017620 . . . Gorai.
011G136400.1 . . . . . . .
. . . . . . . .
Vv05g1614 Tc04_g017610 . Gorai.005G012400.1 .
Gorai.011G135700.1 . . . . .
. . . . . . . .
. .
Vv05g1615 Tc04_g017600 . . . . .
. . . . . . . .
. . . . . .
Vv05g1616 Tc04_g017590 . . . . .
. . . . . . . .
. . . . . .
Vv05g1617 . . Gorai.005G012300.1 .
Gorai.011G135600.1 . . . . . .
. . . . . . . .
.
Vv05g1618 Tc04_g017580 . . . Gorai.
011G135500.1 . . . . . . .
. . . . . . . .
Vv05g1619 Tc04_g017570 . . . Gorai.
011G135400.1 . . . . . . .
. . . . . . . .
Vv05g1620 Tc04_g017560 Gorai.006G231900.1 Gorai.
005G012200.1 . Gorai.011G135300.1 . . .
. . . . . . . .
. . . .
Vv05g1621 Tc04_g017540 . . . . .

. . . . . . . .
. . . . . .
Vv05g1622 Tc04_g017520 . . . Gorai.
011G135200.1 . . . . . . .
. . . . . . . .
Vv05g1623 . . . . . . .
. . . . . . . .
. . . . .
Vv05g1624 Tc04_g017510 . . . Gorai.
011G135100.1 . . . . . . .
. . . . . . . .
Vv05g1625 . . . . . . .
. . . . . . . .
. . . . .
Vv05g1626 . . . . . . .
. . . . . . . .
. . . . .
Vv05g1627 . . . . . . .
. . . . . . . .
. . . . .
Vv05g1628 . . . . . . .
. . . . . . . .
. . . . .
Vv05g1629 . . . . . . .
. . . . . . . .
. . . . .
Vv05g1630 . . . . Gorai.011G134800.1
. . . . . . . .
. . . . . . .
Vv05g1631 . . . . . . .
. . . . . . . .
. . . . .
Vv05g1632 . . . . . . .
. . . . . . . .
. . . . .
Vv05g1633 . . . . . . .
. . . . . . . .
. . . . .
Vv05g1634 . . . . . . .
. . . . . . . .
. . . . .
Vv05g1635 . . . . . . .
. . . . . . . .
. . . . .
Vv05g1636 . . . . . . .
. . . . . . . .
. . . . .
Vv05g1637 . . . . . . .
. . . . . . . .
. . . . .

Vv05g1638 . . . . . . .
. . . . . . . .
. . . . .
Vv05g1639 . . . . . . .
. . . . . . . .
. . . . .
Vv05g1640 . . . . . . .
. . . . . . . .
. . . . .
Vv05g1641 . . . . . . .
. . . . . . . .
. . . . .
Vv05g1642 Tc04_g017480 . . . Gorai.
011G134700.1 . . . . . . .
. . . . . . . .
Vv05g1643 . . . . . . .
. . . . . . . .
. . . . .
Vv05g1644 Tc04_g017470 . . . Gorai.
011G134500.1 . . . . . . .
. . . . . . . .
Vv05g1645 Tc04_g017460 Gorai.006G232200.1 . .
. . . . . . . .
. . . . . . . .
Vv05g1646 . . . . . . .
. . . . . . . .
. . . . .
Vv05g1647 . . . . . . .
. . . . . . . .
. . . . .
Vv05g1648 Tc04_g017430 . . . Gorai.
011G134300.1 . . . . . . .
. . . . . . . .
Vv05g1649 Tc04_g017410 . . . . .
. . . . . . . .
. . . . . .
Vv05g1650 Tc04_g017380 Gorai.006G232500.1 . .
Gorai.011G133900.1 . . . . .
. . . . . . . .
. .
Vv05g1651 Tc04_g017360 Gorai.006G232600.1 Gorai.
005G010100.1 . . . . . . .
. . . . . . . .
. .
Vv05g1652 . . . . . . .
. . . . . . . .
. . . . .
Vv05g1653 Tc04_g017350 . . . Gorai.
011G133700.1 . . . . . . .
. . . . . . . .

Vv05g1654 Tc04_g017340 . . . Gorai.
011G133600.1 . . . . . . .
. . . . . . . .
Vv05g1655 Tc04_g017290 . . . Gorai.
011G132900.1 . . . . . . .
. . . . . . . .
Vv05g1656 Tc04_g017280 . . . Gorai.
011G132800.1 . . . . . . .
. . . . . . . .
Vv05g1657 Tc04_g017270 . . . Gorai.
011G132700.1 . . . . . . .
. . . . . . . .
Vv05g1658 Tc04_g017260 . Gorai.005G008700.1 .
Gorai.011G132600.1 . . . . .
. . . . . . . .
. .
Vv05g1659 Tc04_g017250 . . . Gorai.
011G132500.1 . . . . . . .
. . . . . . . .
Vv05g1660 . . . . . . .
. . . . . . . .
. . . . .
Vv05g1661 Tc04_g017120 . Gorai.005G008300.1 .
. . . . . . . .
. . . . . . . .
Vv05g1662 . . . . . . .
. . . . . . . .
. . . . .
Vv05g1663 Tc04_g017090 . Gorai.005G008100.1 .
. . . . . . . .
. . . . . . . .
Vv05g1664 Tc04_g018940 . . . . .
. . . . . . . .
. . . . . .
Vv05g1665 . . . . . . .
. . . . . . . .
. . . . .
Vv05g1666 . . . . . . .
. . . . . . . .
. . . . .
Vv05g1667 Tc04_g017080 . Gorai.005G008000.1 .
. . . . . . . .
. . . . . . . .
Vv05g1668 Tc04_g017060 . Gorai.005G007900.1 .
. . . . . . . .
. . . . . . . .
Vv05g1669 . . . . . . .
. . . . . . . .
. . . . .
Vv05g1670 Tc04_g017050 . . . . .

. . . . . . . .
. . . . . .
Vv05g1671 . . . . . . .
. . . . . . . .
. . . . .
Vv05g1672 Tc04_g017040 . . . Gorai.
011G131200.1 . . . . . . .
. . . . . . . .
Vv05g1673 . . . . . . .
. . . . . . . .
. . . . .
Vv05g1674 Tc04_g017020 . . . . .
. . . . . . . .
. . . . . .
Vv05g1675 Tc04_g019030 . . . . .
. . . . . . . .
. . . . . .
Vv05g1676 Tc04_g017010 Gorai.006G233000.1 . .
Gorai.011G131100.1 . . . . .
. . . . . . . .
. .
Vv05g1677 . . . . . . .
. . . . . . . .
. . . . .
Vv05g1678 . . . . . . .
. . . . . . . .
. . . . .
Vv05g1679 Tc04_g017000 Gorai.006G233100.1 Gorai.
005G007700.1 . Gorai.011G131000.1 . . .
. . . . . . . .
. . . .
Vv05g1680 . . . . . . .
. . . . . . . .
. . . . .
Vv05g1681 . . . . . . .
. . . . . . . .
. . . . .
Vv05g1682 . . . . . . .
. . . . . . . .
. . . . .
Vv05g1683 Tc04_g016990 Gorai.006G233200.1 Gorai.
005G007600.1 . . . . . . .
. . . . . . . .
. .
Vv05g1684 Tc04_g016980 . Gorai.005G007500.1 .
. . . . . . . .
. . . . . . . .
Vv05g1685 . . . . . . .
. . . . . . . .
. . . . .

```
Vv05g1686 Tc04_g016970 . Gorai.005G007400.1 .
. . . . . . . .
. . . . . . . .
Vv05g1687 Tc04_g016960 . Gorai.005G007100.1 .
. . . . . . . .
. . . . . . . .
Vv05g1688 Tc04_g016950 . Gorai.005G006800.1 .
. . . . . . . .
. . . . . . . .
Vv05g1689 Tc04_g016930 . Gorai.005G004800.1 .
. . . . . . . .
. . . . . . . .
Vv05g1690 Tc04_g016920 . . . . .
. . . . . . . .
. . . . . .
Vv05g1691 . . . . . . .
. . . . . . . .
. . . . .
Vv05g1692 Tc04_g019170 Gorai.006G228400.1 . .
. . . . . . . .
. . . . . . . .
Vv05g1693 . . . . . . .
. . . . . . . .
. . . . .
Vv05g1694 . . . . . . .
. . . . . . . .
. . . . .
Vv05g1695 . . . . . . .
. . . . . . . .
. . . . .
Vv05g1696 . . . . . . .
. . . . . . . .
. . . . .
Vv05g1697 . . . . . . .
. . . . . . . .
. . . . .
Vv05g1698 . . . . . . .
. . . . . . . .
. . . . .
Vv05g1699 . . . . . . .
. . . . . . . .
. . . . .
Vv05g1700 . . . . . . .
. . . . . . . .
. . . . .
Vv05g1701 . . . . . . .
. . . . . . . .
. . . . .
Vv05g1702 . . . . . . .
. . . . . . . .
```

| | | | | | | | |
|---|---|---|---|---|---|---|---|
| . | . | . | . | . | | | |
| Vv05g1703 | . | . | . | . | . | . | . |
| . | . | . | . | . | . | . | . |
| . | . | . | . | . | | | |
| Vv05g1704 | . | . | . | . | . | . | . |
| . | . | . | . | . | . | . | . |
| . | . | . | . | . | | | |
| Vv05g1705 | . | . | . | . | . | . | . |
| . | . | . | . | . | . | . | . |
| . | . | . | . | . | | | |
| Vv05g1706 | Tc04_g019180 | | . | . | . | . | . |
| . | . | . | . | . | . | . | . |
| . | . | . | . | . | . | | |
| Vv05g1707 | . | . | . | . | . | . | . |
| . | . | . | . | . | . | . | . |
| . | . | . | . | . | | | |
| Vv05g1708 | . | . | . | . | . | . | . |
| . | . | . | . | . | . | . | . |
| . | . | . | . | . | | | |
| Vv05g1709 | . | . | . | . | . | . | . |
| . | . | . | . | . | . | . | . |
| . | . | . | . | . | | | |
| Vv05g1710 | . | . | . | . | . | . | . |
| . | . | . | . | . | . | . | . |
| . | . | . | . | . | | | |
| Vv05g1711 | . | . | . | . | . | . | . |
| . | . | . | . | . | . | . | . |
| . | . | . | . | . | | | |
| Vv05g1712 | . | . | . | . | . | . | . |
| . | . | . | . | . | . | . | . |
| . | . | . | . | . | | | |
| Vv05g1713 | . | . | . | . | . | . | . |
| . | . | . | . | . | . | . | . |
| . | . | . | . | . | | | |
| Vv05g1714 | . | . | . | . | . | . | . |
| . | . | . | . | . | . | . | . |
| . | . | . | . | . | | | |
| Vv05g1715 | . | . | . | . | . | . | . |
| . | . | . | . | . | . | . | . |
| . | . | . | . | . | | | |
| Vv05g1716 | . | . | . | . | . | . | . |
| . | . | . | . | . | . | . | . |
| . | . | . | . | . | | | |
| Vv05g1717 | . | . | . | . | . | . | . |
| . | . | . | . | . | . | . | . |
| . | . | . | . | . | | | |
| Vv05g1718 | . | . | . | . | . | . | . |
| . | . | . | . | . | . | . | . |
| . | . | . | . | . | | | |
| Vv05g1719 | . | . | . | . | . | . | . |

. . . . . . . .
. . . . .
Vv05g1720 . . . . . . .
. . . . . . . .
. . . . .
Vv05g1721 . . . . . . .
. . . . . . . .
. . . . .
Vv05g1722 . . . . . . .
. . . . . . . .
. . . . .
Vv05g1723 . . . . . . .
. . . . . . . .
. . . . .
Vv05g1724 . . . . . . .
. . . . . . . .
. . . . .
Vv05g1725 . . . . . . .
. . . . . . . .
. . . . .
Vv05g1726 . . . . . . .
. . . . . . . .
. . . . .
Vv05g1727 . . . . . . .
. . . . . . . .
. . . . .
Vv05g1728 . . . . . . .
. . . . . . . .
. . . . .
Vv05g1729 . . . . . . .
. . . . . . . .
. . . . .
Vv05g1730 . . . . . . .
. . . . . . . .
. . . . .
Vv05g1731 . . . . . . .
. . . . . . . .
. . . . .
Vv05g1732 . . . . . . .
. . . . . . . .
. . . . .
Vv05g1733 . . . . . . .
. . . . . . . .
. . . . .
Vv05g1734 . . . . . . .
. . . . . . . .
. . . . .
Vv05g1735 . . . . . . .
. . . . . . . .
. . . . .

Vv05g1736 . . . . . . .
. . . . . . . .
. . . . .
Vv05g1737 . . . . . . .
. . . . . . . .
. . . . .
Vv05g1738 . . . . . . .
. . . . . . . .
. . . . .
Vv05g1739 Tc04_g020790 Gorai.006G223400.1 . .
. . . . . . . .
. . . . . . . .
Vv05g1740 . . . . . . .
. . . . . . . .
. . . . .
Vv05g1741 . . . . . . .
. . . . . . . .
. . . . .
Vv05g1742 Tc04_g020860 . . . . .
. . . . . . . .
. . . . . .
Vv05g1743 . . . . . . .
. . . . . . . .
. . . . .
Vv05g1744 . . . . . . .
. . . . . . . .
. . . . .
Vv05g1745 . . . . . . .
. . . . . . . .
. . . . .
Vv05g1746 . . . . . . .
. . . . . . . .
. . . . .
Vv05g1747 . . . . . . .
. . . . . . . .
. . . . .
Vv05g1748 . . . . . . .
. . . . . . . .
. . . . .
Vv06g0001 . . . . . . .
. . . . . . . .
. . . . .
Vv06g0002 . . . . . . .
. . . . . . . .
. . . . .
Vv06g0003 . . . . . . .
. . . . . . . .
. . . . .
Vv06g0004 . . . . . . .
. . . . . . . .

. . . . .
Vv06g0005 . . . . . . .
. . . . . . . .
. . . . .
Vv06g0006 Tc09_g032790 . . . . .
. . . . . . . .
. . . . . .
Vv06g0007 . . . . . . .
. . . . . . . .
. . . . .
Vv06g0008 . . . . . . .
. . . . . . . .
. . . . .
Vv06g0009 . . . . . . .
. . . . . . . .
. . . . .
Vv06g0010 . Gorai.013G248500.1 . . .
. . . . . . . .
. . . . . . .
Vv06g0011 . . . . . Gorai.
003G154600.1 . . . . . . .
. . . . . . .
Vv06g0012 Tc09_g018800 . . . Gorai.
009G003200.1 . . . . . . .
. . . . . . . .
Vv06g0013 Tc09_g032740 . . . . .
. . . . . . . .
. . . . . .
Vv06g0014 . . . Gorai.004G019900.1 .
. . . . . . . .
. . . . . . .
Vv06g0015 . . . . . . .
. . . . . . . .
. . . . .
Vv06g0016 . . . . . . .
. . . . . . . .
. . . . .
Vv06g0017 . . . . . . .
. . . . . . . .
. . . . .
Vv06g0018 . . . . . . .
. . . . . . . .
. . . . .
Vv06g0019 . . . . . . .
. . . . . . . .
. . . . .
Vv06g0020 . . . . . . .
. . . . . . . .
. . . . .
Vv06g0021 . . . Gorai.004G020100.1 .

Gorai.003G154400.1 . . . . .
. . . . . . . .
.
Vv06g0022 Tc09_g018480 . . . .
Gorai.003G129300.1 . . . . . .
. . . . . . . .
Vv06g0023 Tc09_g018470 . . . .
Gorai.003G154200.1 Vv08g1698 Tc05_g024670 Gorai.
013G117600.1 . . . . . . .
. . . .
Vv06g0024 Tc09_g018460 Gorai.013G245500.1 .
Gorai.004G020200.1 . . . . . .
. . . . . . . .
. .
Vv06g0025 . . . . . . .
. . . . . . . .
. . . . .
Vv06g0026 . . . . . . .
. . . . . . . .
. . . . .
Vv06g0027 Tc09_g018450 . . Gorai.004G020300.1
. . . . . . . .
. . . . . . . .
Vv06g0028 . . . . . . .
. . . . . . . .
. . . . .
Vv06g0029 Tc09_g018430 . . . . .
. . . . . . . .
. . . . . .
Vv06g0030 Tc09_g020730 . . Gorai.004G020600.1
. . . . . . . .
. . . . . . . .
Vv06g0031 . . . . . . .
. . . . . . . .
. . . . .
Vv06g0032 . . . . . . .
. . . . . . . .
. . . . .
Vv06g0033 . . . . . . .
. . . . . . . .
. . . . .
Vv06g0034 . . . . . . .
. . . . . . . .
. . . . .
Vv06g0035 Tc09_g034740 . . . .
Gorai.003G150700.1 . . . . . .
. . . . . . . .
Vv06g0036 . . . . . .
Vv08g1683 . . . . . Gorai.
007G343600.1 . . . . . . .

Vv06g0037 . . . . . Gorai.
003G129200.1 Vv08g1681 . . . . .
. . . . . . . .
Vv06g0038 . . . . . . .
. . . . . . . .
. . . . .
Vv06g0039 Tc09_g020720 . . . . .
. . . . . . . .
. . . . . .
Vv06g0040 . . . . . . .
. . . . . . . .
. . . . .
Vv06g0041 . . . . . . .
. . . . . . . .
. . . . .
Vv06g0042 Tc09_g004160 . Gorai.001G026700.1 .
Gorai.009G049000.1 . . . . .
. . . . . . . .
. .
Vv06g0043 Tc09_g004330 . Gorai.001G026000.1 .
Gorai.009G048200.1 . . . . .
. . . . . . . .
. .
Vv06g0044 . . . . . . .
. . . . . . . .
. . . . .
Vv06g0045 . . . . . . .
. . . . . . . .
. . . . .
Vv06g0046 . . . . . . .
. . . . . . . .
. . . . .
Vv06g0047 . . . . . . .
. . . . . . . .
. . . . .
Vv06g0048 . . . . . . .
. . . . . . . .
. . . . .
Vv06g0049 . . . . . . .
. . . . . . . .
. . . . .
Vv06g0050 . . . . . . .
. . . . . . . .
. . . . .
Vv06g0051 . . . . . . .
. . . . . . . .
. . . . .
Vv06g0052 . . . Gorai.004G020700.1 .
. . . . . . . .
. . . . . . .

Vv06g0053 . . . . . . .
. . . . . . . .
. . . . .
Vv06g0054 Tc09_g004530 . . . . .
. . . . . . . .
. . . . . .
Vv06g0055 . . . . . Gorai.
003G153800.1 . . . . . . .
. . . . . . .
Vv06g0056 Tc09_g004560 . Gorai.001G023800.1 .
Gorai.009G047000.1 Gorai.003G150800.1 Vv08g1672
Tc05_g024270 Gorai.013G115700.1 . . .
. . . . . . . .
Vv06g0057 . . . . . . .
. . . . . . . .
. . . . .
Vv06g0058 . . . . . . .
. . . . . . . .
. . . . .
Vv06g0059 . . . . . . .
. . . . . . . .
. . . . .
Vv06g0060 . . . . . . .
. . . . . . . .
. . . . .
Vv06g0061 . . . . . . .
. . . . . . . .
. . . . .
Vv06g0062 . . . . . . .
. . . . . . . .
. . . . .
Vv06g0063 . . . . . . .
. . . . . . . .
. . . . .
Vv06g0064 . . . . . . .
. . . . . . . .
. . . . .
Vv06g0065 . . . . . . .
. . . . . . . .
. . . . .
Vv06g0066 . . . . . . .
. . . . . . . .
. . . . .
Vv06g0067 Tc09_g034570 . . Gorai.004G015800.1
. . . . . . . .
. . . . . . . .
Vv06g0068 Tc09_g020300 Gorai.013G244600.1 . .
. Gorai.003G126300.1 Vv08g1665 . .
. Gorai.002G151800.1 . . . .
. . . . .

Vv06g0069 Tc09_g020290 Gorai.013G244500.1 . .
. . . . . . . .
. . . . . . . .
Vv06g0070 Tc09_g020260 Gorai.013G244400.1 . .
. . Vv08g1663 Tc05_g024150 . .
. . . . . . . .
. .
Vv06g0071 . . . . . . .
. . . . . . . .
. . . . .
Vv06g0072 Tc09_g026250 . . . .
Gorai.003G123500.1 . . . . . .
. . . . . . . .
Vv06g0073 . . . . . . .
. . . . . . . .
. . . . .
Vv06g0074 Tc09_g026190 . . . .
Gorai.003G120900.1 . . . . . .
. . . . . . . .
Vv06g0075 . . . . . . .
. . . . . . . .
. . . . .
Vv06g0076 . . . . . . .
. . . . . . . .
. . . . .
Vv06g0077 . . . . . . .
. . . . . . . .
. . . . .
Vv06g0078 Tc09_g021690 Gorai.013G244100.1 . .
. . Vv08g1650 Tc05_g020300 . .
. . . . . . . .
. .
Vv06g0079 . . . . . . .
. . . . . . . .
. . . . .
Vv06g0080 . . . Gorai.004G016100.1 .
. . . . . . . .
. . . . . . .
Vv06g0081 . . . Gorai.004G016200.1 .
. . . . . . . .
. . . . . . .
Vv06g0082 . . . . . . .
. . . . . . . .
. . . . .
Vv06g0083 . . . Gorai.004G016300.1 .
. . . . . . . .
. . . . . . .
Vv06g0084 . . . . . . .
. . . . . . . .
. . . . .

Vv06g0085 . . . . . . .
. . . . . . . .
. . . . .
Vv06g0086 . . . . . . .
. . . . . . . .
. . . . .
Vv06g0087 . . . Gorai.004G016400.1 .
. . . . . . . .
. . . . . . .
Vv06g0088 Tc09_g021650 . . . . .
. . . . . . . .
. . . . . .
Vv06g0089 . . . . . . .
. . . . . . . .
. . . . .
Vv06g0090 . . . . . . .
. . . . . . . .
. . . . .
Vv06g0091 Tc09_g021640 . . . . .
. . . . . . . .
. . . . . .
Vv06g0092 . . . . . . .
. . . . . . . .
. . . . .
Vv06g0093 Tc09_g021630 Gorai.013G241700.1 Gorai.
004G091700.1 Gorai.004G016600.1 . Gorai.010G126100.1
. . . . . . . .
. . . . . .
Vv06g0094 Tc09_g021620 . . Gorai.004G017100.1
. . . . . . . .
. . . . . . . .
Vv06g0095 . . . . . . .
. . . . . . . .
. . . . .
Vv06g0096 Tc09_g021610 . . . .
Gorai.010G126300.1 . . . . . .
. Vv013rg0055 . . . . .
.
Vv06g0097 Tc09_g021600 . . . .
Gorai.010G126400.1 . . . . . .
. . . . . . . .
Vv06g0098 Tc09_g021590 . . . .
Gorai.010G126700.1 . . . . . .
. . . . . . . .
Vv06g0099 Tc09_g021560 Gorai.013G247300.1 . .
. . . . . . . .
. . . . . . . .
Vv06g0100 Tc09_g021520 . . . . .
. . . . . . . .
. . . . . .

Vv06g0101 . . . . . . .
. . . . . . . .
. . . .
Vv06g0102 Tc09_g021470 Gorai.013G247400.1 . .
. . . . . . . .
. . . . . . . .
Vv06g0103 . . . . . . .
. . . . . . . .
. . . . .
Vv06g0104 . . . . . . .
. . . . . . . .
. . . . .
Vv06g0105 Tc09_g021220 . . . . .
. . . . . . . .
. . . . . .
Vv06g0106 . . . . . . .
. . . . . . . .
. . . . .
Vv06g0107 . . . Gorai.004G017200.1 .
. . . . . . . .
. . . . . . .
Vv06g0108 . . . . . . .
. . . . . . . .
. . . . .
Vv06g0109 . . . . . . .
. . . . . . . .
. . . . .
Vv06g0110 . Gorai.013G247500.1 . Gorai.
004G017300.1 . . . . . . .
. . Vv013rg0057 . . . .
. .
Vv06g0111 . . . . . . .
. . . . . . . .
. . . . .
Vv06g0112 . Gorai.013G247700.1 . .
Gorai.009G067500.1 . Vv08g1648 Tc05_g020330 .
. . . . . . . .
. . .
Vv06g0113 . . . . . . .
. . . . . . . .
. . . . .
Vv06g0114 . . . . . . .
. . . . . . . .
. . . . .
Vv06g0115 . . . Gorai.004G017400.1 .
. . . . . . . .
. . . . . . .
Vv06g0116 . . . . . . .
. . . . . . . .
. . . . .

```
Vv06g0117       .       .       .       .       .       .
Vv08g1636       .       .       .       .       .       .       .
        .       .       .       .       .       .
Vv06g0118       .       .       .       .       .       .       .
        .       .       .       .       .       .       .       .
        .       .       .       .       .
Vv06g0119       .       .       .       .       .       .       .
        .       .       .       .       .       .       .       .
        .       .       .       .       .
Vv06g0120       Tc09_g021000    .       Gorai.004G094000.1      .
        .       Gorai.010G127100.1      .       .       .       .
        .       .       .       Vv013rg0060     .       .       .
        .       .       .
Vv06g0121       .       .       .       .       .       .       .
        .       .       .       .       .       .       .       .
        .       .       .       .       .
Vv06g0122       .       .       .       Gorai.004G017500.1      .
        .       .       .       .       .       .       .       .
        .       .       .       .       .       .       .
Vv06g0123       .       Gorai.013G248000.1      .       .       .
        .       .       .       .       .       .       .       .
        .       .       .       .       .       .       .
Vv06g0124       .       .       .       Gorai.004G017600.1      .
        .       .       .       .       .       .       .       .
        .       .       .       .       .       .       .
Vv06g0125       .       .       .       .       .       .
Vv08g1632       .       .       .       .       .       .       .
        .       .       .       .       .       .
Vv06g0126       .       Gorai.013G248100.1      .       Gorai.
004G017700.1    .       .       Vv08g1631       Tc05_g020650    .
        .       .       .       .       .       .       .       .
        .       .       .
Vv06g0127       .       .       .       .       .       .
Vv08g1629       .       .       .       .       .       .       .
        .       .       .       .       .       .
Vv06g0128       .       .       .       .       .       .
Vv08g1627       .       .       .       .       .       .       .
        .       .       .       .       .       .
Vv06g0129       .       .       .       .       .       .
Vv08g1625       .       .       .       .       .       Gorai.
007G362600.1    .       .       .       .       .       .       .
Vv06g0130       .       .       .       .       .       .       .
        .       .       .       .       .       .       .       .
        .       .       .       .       .
Vv06g0131       .       .       .       .       .       .       .
        .       .       .       .       .       .       .       .
        .       .       .       .       .
Vv06g0132       .       .       .       .       .       .
Vv08g1624       Tc05_g020670    .       .       .       .       .
        .       .       .       .       .       .       .
```

Vv06g0133 . . . . . .
Vv08g1623 Tc05_g020680 . . . .
Gorai.007G362500.1 . . . . . .
.
Vv06g0134 . . . . . . .
. . . . . . . .
. . . . .
Vv06g0135 . . . . . . .
. . . . . . . .
. . . . .
Vv06g0136 . . . . . . .
. . . . . . Vv013rg0061
. . . . . .
Vv06g0137 . . . . . . .
. . . . . . . .
. . . . .
Vv06g0138 Tc09_g031010 . . . .
Gorai.003G128500.1 . . . . . .
. . . . . . . .
Vv06g0139 . . . . . .
Vv08g1615 . . . . . . .
. . . . . .
Vv06g0140 Tc09_g009300 . Gorai.001G007400.1
Gorai.004G018000.1 . . . . . .
. . . . . . . .
. .
Vv06g0141 . . . . . . .
. . . . . . . .
. . . . .
Vv06g0142 . Gorai.013G248400.1 . . .
. Vv08g1610 . . . . .
. . . . . . . .
Vv06g0143 . . . . . . .
. . . . . . . .
. . . . .
Vv06g0144 . . . . . .
Vv08g1608 . . . Gorai.002G155600.1 .
. . . . . . . .
Vv06g0145 . . . . . . .
. . . . . . . .
. . . . .
Vv06g0146 Tc09_g024980 . . . .
Gorai.006G124800.1 . . . . . .
. . . . . . . .
Vv06g0147 . . . . . . .
. . . . . . . .
. . . . .
Vv06g0148 . . . . . . .
. . . . . . . .
. . . . .

Vv06g0149 . . . . . . .
. . . . . . . .
. . . . .
Vv06g0150 . . . . . . .
. . . . . . . .
. . . . .
Vv06g0151 . . . . . . .
. . . . . . . .
. . . . .
Vv06g0152 Tc09_g020460 . . . . .
. . . . . . .
Vv013rg0072 . . . . . .
Vv06g0153 . . . . . . .
. . . . . . . .
. . . . .
Vv06g0154 . . . . . .
Vv08g1585 Tc05_g018780 . . . . .
. . . . . . .
Vv06g0155 . . . . . . .
. . . . . . Vv013rg0074
. . . . . .
Vv06g0156 . . . . . . .
. . . . . . . .
. . . . .
Vv06g0157 . Gorai.013G248700.1 . . .
. Vv08g1582 Tc05_g018800 . .
Gorai.002G156600.1 Gorai.007G362300.1 Gorai.007G362300.1
. . . . . . .
Vv06g0158 Tc09_g009440 . . . . .
. . . . . . . .
. . . . . .
Vv06g0159 Tc09_g020380 . . . . .
. . . . . . . .
. . . . . .
Vv06g0160 . . . Gorai.004G018200.1 .
. . . . . . . .
. . . . . . .
Vv06g0161 . . . . . . .
. . . . . . . .
. . . . .
Vv06g0162 . . . . Gorai.009G070300.1
. Vv08g1577 Tc05_g018850 . . .
. . Vv013rg0079 . . . .
. .
Vv06g0163 Tc09_g025270 . . . . .
. . . . . . . .
. . . . . .
Vv06g0164 Tc09_g031150 Gorai.013G249200.1 .
Gorai.004G018400.1 . . Vv08g1576
Tc05_g018860 . . . . . . .

. . . . .
Vv06g0165 . . . . Gorai.009G070400.1
. Vv08g1575 Tc05_g018870 . . .
. . . . . . . .
.
Vv06g0166 Tc09_g031280 . . . . .
. . . . . . . .
. . . . . .
Vv06g0167 . Gorai.013G249300.1 . .
Gorai.009G070500.1 . . . . . .
. . . . . . . .
.
Vv06g0168 . Gorai.013G249400.1 . Gorai.
004G018500.1 Gorai.009G070600.1 . Vv08g1570
Tc05_g018910 . . . . . Vv013rg0082
. . . . . .
Vv06g0169 . . . Gorai.004G018600.1 .
. . . . . . . .
. . . . . . .
Vv06g0170 . . . . Gorai.009G070700.1
. . . . . . . .
. . . . . . .
Vv06g0171 . Gorai.013G249500.1 . . .
. . . . . . . .
. . . . . . .
Vv06g0172 Tc09_g009720 . Gorai.001G005700.1 .
. . . . . . . .
. . . . . . . .
Vv06g0173 . . . . . . .
. . . . . . . .
. . . . .
Vv06g0174 . . . . . . .
. . . . . . . .
. . . . .
Vv06g0175 Tc09_g031220 . . . . .
. . . . . . . .
. . . . . .
Vv06g0176 Tc09_g031220 . . . . .
. . . . . . . .
. . . . . .
Vv06g0177 . . . . . . .
. . . . . . . .
. . . . .
Vv06g0178 . . . . . . .
. . . . . . . .
. . . . .
Vv06g0179 . . . . . . .
. . . . . . . .
. . . . .
Vv06g0180 Tc09_g031290 . . . . .

| | | | | | | | | |
|---|---|---|---|---|---|---|---|---|
| | . | . | . | . | . | . | . | . |
| | . | . | . | . | . | . | | |
| Vv06g0181 | | . | . | . | . | . | . | . |
| | . | . | . | . | . | . | . | . |
| | . | . | . | . | . | | | |
| Vv06g0182 | | . | . | . | . | . | . | |
| Vv013rg0121 | | . | . | . | . | . | . | . |
| | . | . | . | . | . | . | | |
| Vv06g0183 | | . | . | . | . | . | . | . |
| | . | . | . | . | . | . | . | . |
| | . | . | . | . | . | | | |
| Vv06g0184 | | . | . | . | . | . | . | . |
| | . | . | . | . | . | . | . | . |
| | . | . | . | . | . | | | |
| Vv06g0185 | | . | . | . | . | . | . | |
| Vv013rg0122 | | . | . | . | . | . | . | . |
| | . | . | . | . | . | . | | |
| Vv06g0186 | | . | . | . | . | . | . | . |
| | . | . | . | . | . | . | . | . |
| | . | . | . | . | . | | | |
| Vv06g0187 | | . | . | . | . | . | . | |
| Vv013rg0123 | | . | . | . | . | . | . | . |
| | . | . | . | . | . | . | | |
| Vv06g0188 | | . | . | . | . | . | . | . |
| | . | . | . | . | . | . | . | . |
| | . | . | . | . | . | | | |
| Vv06g0189 | | . | . | . | . | . | . | . |
| | . | . | . | . | . | . | . | . |
| | . | . | . | . | . | | | |
| Vv06g0190 | | Tc09_g025570 | | . | . | . | . | . |
| | Vv013rg0124 | | . | . | . | . | . | . |
| | . | . | . | . | . | . | . | |
| Vv06g0191 | | . | . | . | . | Gorai.009G071200.1 | | |
| | . | Vv13g1325 | | . | . | . | . | . |
| | . | . | . | . | . | . | . | . |
| Vv06g0192 | | . | . | . | . | . | . | |
| Vv13g1324 | | . | . | . | . | . | . | . |
| | . | . | . | . | . | . | | |
| Vv06g0193 | | . | . | . | . | . | . | . |
| | . | . | . | . | . | . | . | . |
| | . | . | . | . | . | | | |
| Vv06g0194 | | . | . | . | . | . | . | |
| Vv13g1323 | | Tc10_g006390 | | . | . | . | . | . |
| | . | . | . | . | . | . | . | |
| Vv06g0195 | | . | . | . | . | . | . | . |
| | . | . | . | . | . | . | . | . |
| | . | . | . | . | . | | | |
| Vv06g0196 | | . | . | . | . | . | . | . |
| | . | . | . | . | . | . | . | . |
| | . | . | . | . | . | | | |

Vv06g0197 . . . . . . .
. . . . . . . .
. . . . .
Vv06g0198 . . . . . . .
. . . . . . . .
. . . . .
Vv06g0199 . . . . . . .
. . . . . . . .
. . . . .
Vv06g0200 . . . . . . .
. . . . . . . .
. . . . .
Vv06g0201 . . . . . . .
. . . . . . . .
. . . . .
Vv06g0202 . . . . . . .
. . . . . . . .
. . . . .
Vv06g0203 Tc09_g031010 . . . .
Gorai.003G128500.1 . . . . . .
. . . . . . . .
Vv06g0204 . Gorai.013G250000.1 . . .
. . . . . . . .
. . . . . . .
Vv06g0205 . . . . . . .
. . . . . . . .
. . . . .
Vv06g0206 . Gorai.013G250100.1 . . .
Gorai.006G141200.1 . . . . .
. . . . . . . .
.
Vv06g0207 . . . . Gorai.009G071300.1
. . . . . . . .
. . . . . . .
Vv06g0208 . . . . . . .
. . . . . . . .
. . . . .
Vv06g0209 . . . . . . .
. . . . . . . .
. . . . .
Vv06g0210 . . . . . . .
. . . . . . . .
. . . . .
Vv06g0211 . Gorai.013G250300.1 . .
Gorai.009G071400.1 . . . . . .
. . . . . . . .
.
Vv06g0212 . . . . . . .
. . . . . . . .
. . . . .

Vv06g0213 Tc09_g030790 . Gorai.001G113800.1 .
. . . . . . . .
. . . . . . . .
Vv06g0214 . . . . . . .
. . . . . . . .
. . . . .
Vv06g0215 . . . . . . .
. . . . . . . .
. . . . .
Vv06g0216 . . . . . . .
. . . . . . . .
. . . . .
Vv06g0217 . . . . . . .
. . . . . . . .
. . . . .
Vv06g0218 Tc09_g030470 . . . Gorai.
009G071800.1 . . . . . . .
. . . . . . . .
Vv06g0219 . . . . . .
Vv13g1306 . . . . . . .
. . . . . .
Vv06g0220 . . . . . Gorai.
006G129600.1 . . . . . . .
. . . . . . .
Vv06g0221 . Gorai.013G250500.1 . . .
. . . . . . . .
. . . . . . .
Vv06g0222 . . Gorai.001G140400.1 . .
Gorai.006G129500.1 . . . . .
. . . . . . . .
.
Vv06g0223 . Gorai.013G250600.1 . . .
. . . . . . . .
. . . . . . .
Vv06g0224 . . . Gorai.004G035800.1
Gorai.009G071900.1 Gorai.006G140200.1 . . .
. . . . . . . .
. . .
Vv06g0225 Tc09_g030110 . . . . .
. . . . . . . .
. . . . . .
Vv06g0226 . . . . . . .
. . . . . . . .
. . . . .
Vv06g0227 . . Gorai.001G140300.1 . .
. . . . . . . .
. . . . . . .
Vv06g0228 Tc09_g025900 . . . . .
. . . . . . . .
. . . . . .

Vv06g0229 . . . Gorai.004G036200.1 .
. Vv13g1290 . . . . .
. . . . . . .
Vv06g0230 . . Gorai.001G140000.1 . .
Gorai.006G139300.1 . . . . .
. . . . . . . .
.
Vv06g0231 . . . . Gorai.009G072300.1
. . . . . . . .
. . . . . . .
Vv06g0232 . . . . . . .
. . . . . . . .
. . . . .
Vv06g0233 . . . . . . .
. . . . . . . .
. . . . .
Vv06g0234 . . . . . . .
. . . . . . . .
. . . . .
Vv06g0235 . . . Gorai.004G018900.1 .
. . . . . . . .
. . . . . . .
Vv06g0236 . Gorai.013G250800.1 . Gorai.
004G037000.1 . Gorai.006G138700.1 Vv13g1280 .
. . . . . . . .
. . . .
Vv06g0237 . . . Gorai.004G019000.1
Gorai.009G072400.1 . . . . . .
. . . . . . . .
.
Vv06g0238 . . . . . . .
. . . . . . . .
. . . . .
Vv06g0239 . . . . . . .
. . . . . . . .
. . . . .
Vv06g0240 Tc09_g003230 . . . Gorai.
009G072500.1 . . . . . . .
. . . . . . . .
Vv06g0241 . . Gorai.001G139700.1 . .
. . . . . . . .
. . . . . . .
Vv06g0242 . . . . . . .
. . . . . . . .
. . . . .
Vv06g0243 Tc09_g034570 . Gorai.001G139600.1
Gorai.004G037100.1 Gorai.009G072600.1 . . .
. . . . . . . .
. . . .
Vv06g0244 Tc09_g030080 . . . . .

. . . . . . . .
. . . . . .
Vv06g0245 Tc09_g034210 . . Gorai.004G019200.1
Gorai.009G072700.1 . . . . .
. . . . . . . .
. .
Vv06g0246 . . . . . . .
. . . . . . . .
. . . . .
Vv06g0247 . . . . . . .
. . . . . . . .
. . . . .
Vv06g0248 . . . . . . .
. . . . . . . .
. . . . .
Vv06g0249 . . . . . . .
. . . . . . . .
. . . . .
Vv06g0250 . . . . . . .
. . . . . . . .
. . . . .
Vv06g0251 . . . . . Gorai.
006G129200.1 . . . . . . .
. . . . . . .
Vv06g0252 . . . . . . .
. . . . . . . .
. . . . .
Vv06g0253 Tc09_g026260 . Gorai.001G139100.1
Gorai.004G019500.1 . . . . . .
. . . . . . . .
. .
Vv06g0254 . . . Gorai.004G019600.1 .
. . . . . . . .
. . . . . . .
Vv06g0255 . . . . . . .
. . . . . . . .
. . . . .
Vv06g0256 Tc09_g002910 . . . Gorai.
009G072900.1 . . . . . . .
. . . . . . . .
Vv06g0257 . . . Gorai.004G019700.1 .
. . . . . . . .
. . . . . . .
Vv06g0258 . . . . . . .
. . . . . . . .
. . . . .
Vv06g0259 . . . . Gorai.009G073000.1
. . . . . . . .
. . . . . . .
Vv06g0260 Tc09_g029840 . . . . .

. . . . . . . .
. . . . . .
Vv06g0261 . . . . . . .
. . . . . . . .
. . . . .
Vv06g0262 Tc09_g033950 . . . . .
. . . . . . . .
. . . . . .
Vv06g0263 . . . . . Gorai.
006G129000.1 . . . . . . .
. . . . . . .
Vv06g0264 . . . Gorai.004G037400.1 .
Gorai.006G128800.1 Vv13g1265 . . .
. . . . . . . .
. .
Vv06g0265 . . . . . . .
. . . . . . . .
. . . . .
Vv06g0266 . . Gorai.001G138300.1 Gorai.
004G037500.1 . Gorai.006G138100.1 Vv13g1262 .
. . . . . . . .
. . . .
Vv06g0267 . . . . . . .
. . . . . . . .
. . . . .
Vv06g0268 . . . . . Gorai.
006G128700.1 . . . . . . .
. . . . . . .
Vv06g0269 . . . . Gorai.009G073300.1
Gorai.006G128600.1 Vv13g1261 . . .
. . . . . . . .
. .
Vv06g0270 . . . . . Gorai.
006G128500.1 . . . . . . .
. . . . . . .
Vv06g0271 . . . . . . .
. . . . . . . .
. . . . .
Vv06g0272 . Gorai.013G251200.1 . . .
. Vv13g1257 . . . . . .
. . . . . . . .
Vv06g0273 . . . Gorai.004G019800.1 .
. . . . . . . .
. . . . . . .
Vv06g0274 . . . . . . .
. . . . . . . .
. . . . .
Vv06g0275 . . . . . . .
. . . . . . . .
. . . . .

Vv06g0276 . . . . . . .
. . . . . . . .
. . . . .
Vv06g0277 . Gorai.013G251300.1 Gorai.001G137900.1
. . . . . . . .
. . . . . . . .
.
Vv06g0278 . . . . . . .
. . . . . . . .
. . . . .
Vv06g0279 . . . . . . .
. . . . . . . .
. . . . .
Vv06g0280 . . . . . . .
. . . . . . . .
. . . . .
Vv06g0281 . . . . . . .
. . . . . . . .
. . . . .
Vv06g0282 . . . . . . .
. . . . . . . .
. . . . .
Vv06g0283 . Gorai.013G251400.1 . . .
Gorai.006G127900.1 . . . . .
. . . . . . . .
.
Vv06g0284 . . . . . . .
. . . . . . . .
. . . . .
Vv06g0285 . . Gorai.001G137800.1 .
Gorai.009G073600.1 . . . . . .
. . . . . . . .
.
Vv06g0286 . . Gorai.001G137700.1 . .
. . . . . . . .
. . . . . . .
Vv06g0287 . . . Gorai.004G022400.1 .
Gorai.006G127600.1 . . . . .
. . . . . . . .
.
Vv06g0288 . Gorai.013G251600.1 Gorai.001G137600.1
. . Gorai.006G127400.1 . . .
. . . . . . . .
. . .
Vv06g0289 . . . . Gorai.009G073700.1
Gorai.006G127100.1 . . . . .
. . . . . . . .
.
Vv06g0290 . . . . . Gorai.
006G127000.1 . . . . . . .

. . . . . . .
Vv06g0291 Tc09_g033710 . . . .
Gorai.003G143800.1 . . . . . .
. . . . . . . .
Vv06g0292 Tc09_g033700 . . . . .
Vv08g0753 . Gorai.006G165300.1 . .
. . . . . . . .
.
Vv06g0293 Tc09_g033690 Gorai.013G268100.1 Gorai.
001G134600.1 . . Gorai.006G165400.1 . .
. . . . . Vv13g0704 .
. . . . .
Vv06g0294 Tc09_g033680 . . . . .
. . . . . . . .
. . . . . .
Vv06g0295 . . . . Gorai.009G109800.1
. . . . . . . .
Vv13g0701 . . Gorai.011G238300.1
Gorai.009G312500.1 . .
Vv06g0296 . Gorai.013G267700.1 . .
Gorai.009G109700.1 . . . . . .
. . . . . . . .
.
Vv06g0297 . . . . . .
Vv08g0758 Tc05_g006620 . Gorai.002G247800.1 .
. Gorai.007G373500.1 . . . .
. . .
Vv06g0298 . . . . . . .
. . . . . . . .
. . . . .
Vv06g0299 . . . . Gorai.009G109500.1
. . . . . . . .
. . . . . . .
Vv06g0300 . . . . . . .
. . . . . . . .
. . . . .
Vv06g0301 . . . . . . .
. . . . . . . .
. . . . .
Vv06g0302 . . . . . . .
. . . . . . . .
. . . . .
Vv06g0303 . . . . . . .
. . . . . . . .
. . . . .
Vv06g0304 . . . . Gorai.009G109400.1
. . . . . . . .
. . . . . . .
Vv06g0305 . . . . . . .
. . . . . . . .

.	.	.	.	.
Vv06g0306	.	.	.	.	Gorai.009G109300.1
Gorai.006G115600.1	.	.	.	.	.
.	.	.	.	.	.	.	.
.
Vv06g0307	.	.	.	.	.	.	.
.	.	.	.	.	.	.	.
.	.	.	.	.
Vv06g0308	.	Gorai.013G267400.1	.	.
Gorai.009G109100.1	Gorai.006G135100.1	.	.	.
.	.	.	.	.	.	.	.
.	.	.
Vv06g0309	.	.	.	.	.	.	.
.	.	.	.	.	.	.	.
.	.	.	.	.
Vv06g0310	.	Gorai.013G252300.1	.	.	.
Gorai.006G132200.1	.	.	.	.	.
.	.	.	.	.	.	.	.
.
Vv06g0311	.	.	.	.	Gorai.009G108800.1
.	.	.	.	.	.	.	.
.	.	.	.	.	.	.
Vv06g0312	.	.	.	.	.	Gorai.
006G166000.1	Vv08g0763	Tc05_g006550	Gorai.006G166000.1
Gorai.002G248000.1	.	Gorai.009G416300.1
Gorai.007G373600.1	Vv13g0688	Tc10_g005730	.
Gorai.011G238400.1	.	.	.
Vv06g0313	Tc09_g033630	.	.	.	.	.
.	.	.	.	.	.	.	.
.	.	.	.	.	.
Vv06g0314	Tc09_g033620	.	.	.	Gorai.
009G108700.1	Gorai.003G143400.1	.	.	.	.
.	.	.	.	.	.	.	.
.	.
Vv06g0315	Tc09_g033610	.	.	.	Gorai.
009G108600.1	.	.	.	.	.	.	.
.	.	.	.	.	.	.	.
Vv06g0316	Tc09_g033600	.	.	.	Gorai.
009G108500.1	Gorai.003G143300.1	Vv08g0771
Tc05_g006510	.	Gorai.002G248200.1	.	.	.
.	.	.	.	.	.	.
Vv06g0317	Tc09_g033590	.	.	.	Gorai.
009G108400.1	Gorai.003G143200.1	Vv08g0773
Tc05_g006490	.	Gorai.002G248300.1	.	Gorai.
009G417300.1	.	.	.	.	.	.	.
.
Vv06g0318	.	.	.	.	.	.	.
.	.	.	.	.	.	.	.
.	.	.	.	.
Vv06g0319	Tc09_g033580	.	.	.	Gorai.

009G108300.1 Gorai.003G143100.1 . . . .
. . . . . . . .
. .
Vv06g0320 Tc09_g033560 Gorai.013G267300.1 . .
Gorai.009G108200.1 Gorai.003G143000.1 . .
. . . . . . . .
. . . .
Vv06g0321 Tc09_g033550 . . . Gorai.
009G108100.1 Gorai.003G142900.1 . . . .
. . . . . . . .
. .
Vv06g0322 . . . . . . .
. . . . . . . .
. . . . .
Vv06g0323 Tc09_g033540 . . . Gorai.
009G108000.1 Gorai.003G142800.1 . . . .
. . . Vv13g0682 Tc10_g005630 .
. Gorai.009G313600.1 Gorai.010G205600.1 .
Vv06g0324 Tc09_g033530 . . . .
Gorai.003G142700.1 . . . . . .
. . . . . . . .
Vv06g0325 Tc09_g033520 Gorai.013G267200.1 . .
Gorai.009G107900.1 Gorai.003G142600.1 . .
. . . . . Vv13g0681
Tc10_g005620 . Gorai.011G238800.1 Gorai.009G313700.1
. .
Vv06g0326 Tc09_g033510 Gorai.013G267100.1 . .
Gorai.009G107800.1 Gorai.006G120400.1 Vv08g0776
Tc05_g006450 Gorai.006G166400.1 . . .
. Vv13g0680 Tc10_g005610 . Gorai.
011G238900.1 . . Gorai.012G004800.1
Vv06g0327 . . . . . . .
. . . . . . . .
. . . . .
Vv06g0328 Tc09_g033490 Gorai.013G267000.1 . .
Gorai.009G107700.1 Gorai.003G142400.1 . .
. . . . . . . .
. . . .
Vv06g0329 . . . . . . .
. . . . . . . .
. . . . .
Vv06g0330 Tc09_g033480 . . . Gorai.
009G107600.1 Gorai.003G142300.1 Vv08g0777
Tc05_g006440 . . . Gorai.009G417500.1 .
Vv13g0676 . Gorai.013G027400.1 . .
. .
Vv06g0331 Tc09_g033470 Gorai.013G266900.1 . .
Gorai.009G107500.1 Gorai.003G142200.1 . .
. . . . . Vv13g0675 .
. . . . .

Vv06g0332 Tc09_g033450 Gorai.013G263900.1 . .
. Gorai.003G142000.1 . . . .
. . . . . . . .
. .
Vv06g0333 Tc09_g033440 . . . Gorai.
009G107400.1 Gorai.003G141900.1 . . . .
. . . . . . . .
. .
Vv06g0334 . . . . . . .
. . . . . . . .
. . . . .
Vv06g0335 Tc09_g033430 . . . .
Gorai.006G166600.1 Vv08g0780 Tc05_g006420 Gorai.
006G166600.1 . . Gorai.009G417700.1 . .
. . . . . .
Vv06g0336 . . . . . . .
. . . . . . . .
. . . . .
Vv06g0337 Tc09_g033410 . . . Gorai.
009G107200.1 Gorai.006G166800.1 Vv08g0782
Tc05_g006380 Gorai.006G166800.1 Gorai.002G248400.1 .
Gorai.009G418100.1 . . . . .
. . .
Vv06g0338 . . . . . . .
. . . . . . . .
. . . . .
Vv06g0339 Tc09_g033380 . . . Gorai.
009G106900.1 . . . . . . .
. . . . . . . .
Vv06g0340 Tc09_g033360 . . . Gorai.
009G106600.1 Gorai.003G141300.1 . . . .
. . . Vv13g0668 Tc10_g005420 .
. . . .
Vv06g0341 . . . . . . .
. . . . . . . .
. . . . .
Vv06g0342 Tc09_g033330 . . . . .
. . . . . . . .
. . . . . .
Vv06g0343 . . . . . Gorai.
006G113600.1 . . . . . . .
. . . . . . .
Vv06g0344 Tc09_g033320 . . . . .
. . . . . . . .
. . . . . .
Vv06g0345 . . . . Gorai.009G106300.1
Gorai.003G141100.1 . . . . .
. . . . . . . .
.
Vv06g0346 . . . . . . .

```
        .       .       .       .       .       .       .       .
        .       .       .       .       .
Vv06g0347       .       .       .       .       .       Gorai.
003G141000.1    .       .       .       .       .       .       .
        .       .       .       .       .       .       .
Vv06g0348       .       .       .       .       .       .       .
        .       .       .       .       .       .       .       .
        .       .       .       .       .
Vv06g0349       .       .       .       .       .       .       .
        .       .       .       .       .       .       .       .
        .       .       .       .       .
Vv06g0350       .       .       .       .       Gorai.009G103400.1
        .       .       .       .       .       .       .       .
        Vv13g0664       Tc10_g005370    .       Gorai.011G240100.1
        Gorai.009G314200.1      .       .
Vv06g0351       .       .       .       .       .       Gorai.
006G167300.1    Vv08g0787       Tc05_g006250    Gorai.006G167300.1
        .       .       Gorai.009G418300.1      Gorai.007G374000.1
        .       .       .       .       .       .       .
Vv06g0352       .       .       .       .       .       .       .
        .       .       .       .       .       .       .       .
        .       .       .       .       .
Vv06g0353       .       .       .       .       .       Gorai.
003G138000.1    .       .       .       .       .       .       .
        .       .       .       .       .       .       .
Vv06g0354       Tc09_g025520    .       Gorai.001G132200.1      .
        .       Gorai.006G121600.1      .       .       .       .
        .       .       .       .       .       .       .       .
        .       .
Vv06g0355       .       .       .       .       .       .       .
        .       .       .       .       .       .       .       .
        .       .       .       .       .
Vv06g0356       Tc09_g025610    .       .       .       Gorai.
009G078300.1    .       .       .       .       .       .       .
        .       .       .       .       .       .       .       .
Vv06g0357       Tc09_g025630    .       Gorai.001G131700.1      .
        .       Gorai.006G119700.1      .       .       .       .
        .       .       .       .       .       .       .       .
        .       .
Vv06g0358       Tc09_g025640    .       .       .       Gorai.
009G078400.1    .       .       .       .       .       .       .
        .       .       .       .       .       .       .       .
Vv06g0359       Tc09_g025660    .       Gorai.001G131300.1      .
        .       .       .       .       .       .       .       .
        .       .       .       .       .       .       .       .
Vv06g0360       .       .       .       .       .       Gorai.
006G167400.1    .       .       .       .       .       .       .
        .       .       .       .       .       .       .
Vv06g0361       .       .       .       .       .       .       .
        .       .       .       .       .       .       .       .
```

. . . . .
Vv06g0362 Tc09_g025680 . Gorai.001G131000.1 .
. Gorai.006G167600.1 Vv08g0796
Tc05_g006190 Gorai.006G167600.1 . . Gorai.
009G418600.1 . . . . . . .
.
Vv06g0363 Tc09_g025700 . . . .
Gorai.006G127700.1 . . . . . .
. . . . . . . .
Vv06g0364 Tc09_g025710 . . . .
Gorai.006G120200.1 Vv08g0799 Tc05_g006170 .
Gorai.002G248800.1 . Gorai.009G418800.1 . .
. . . . . .
Vv06g0365 . . . . . . .
. . . . . . Vv13g0647
. . . . . .
Vv06g0366 Tc09_g025760 . . . Gorai.
009G078600.1 . Vv08g0804 Tc05_g006150 . .
. . . Vv13g0646 Tc10_g005220 .
Gorai.011G240700.1 . Gorai.010G205100.1
Gorai.012G004100.1
Vv06g0367 . . Gorai.001G130800.1 .
Gorai.009G078700.1 . Vv08g0805 Tc05_g006140 .
. . . . Vv13g0644 . .
. . Gorai.010G205000.1 .
Vv06g0368 Tc09_g033510 . . . . .
. . . . . . . .
. . . . . .
Vv06g0369 . . . . . Gorai.
006G120500.1 . . . . . . .
. . . . . . .
Vv06g0370 . . . . . . .
. . . . . . . .
. . . . .
Vv06g0371 . . . . . .
Vv08g0809 . . . . . . .
. . . . . .
Vv06g0372 . . . . . Gorai.
006G120700.1 . . . . . . .
. . . . . . .
Vv06g0373 . . . . . . .
. . . . . . . .
. . . . .
Vv06g0374 . . . . . . .
. . . . . . . .
. . . . .
Vv06g0375 . . . . . . .
. . . . . . . .
. . . . .
Vv06g0376 . . . . . . .

. . . . . . . .
. . . . .
Vv06g0377 . . Gorai.001G130700.1 . .
. Vv08g0824 Tc05_g006010 . Gorai.
002G249600.1 . . . . . . .
. . .
Vv06g0378 . . . . . . .
. . . . . . . .
. . . . .
Vv06g0379 . . . . . . .
. . . . . . . .
. . . . .
Vv06g0380 . . Gorai.001G103500.1 Gorai.
004G012900.1 . . . . . . .
. . . . . . . .
.
Vv06g0381 . . . . . . .
. . . . . . . .
. . . . .
Vv06g0382 . . . . . . .
. . . . . . Vv13g0630
Tc10_g005090 . Gorai.011G241200.1 .
Gorai.010G204400.1 .
Vv06g0383 Tc09_g033360 . Gorai.001G130300.1 .
. . . . . . . .
. . . . . . . .
Vv06g0384 . . . . . . .
. . . . . . . .
. . . . .
Vv06g0385 . . Gorai.001G130200.1 . .
. . . . . . . .
. . . . . . .
Vv06g0386 . . . . . . .
. . . . . . . .
. . . . .
Vv06g0387 . . . . . . .
. . . . . . . .
. . . . .
Vv06g0388 . . . . . . .
. . . . . . . .
. . . . .
Vv06g0389 . . . . . . .
. . . . . . . .
. . . . .
Vv06g0390 . . . . Gorai.009G078900.1
. . . . . . . .
. . . . . . .
Vv06g0391 . . Gorai.001G130100.1 . .
Gorai.006G111500.1 Vv08g0830 Tc05_g005970 .
. . . . . . . .

. . .
Vv06g0392 . . Gorai.001G129800.1 . .
Gorai.006G120800.1 . . . . .
. . . . . . . .
.
Vv06g0393 . . . . . . .
. . . . . . Vv13g0618
. . . . . .
Vv06g0394 . . . . . . .
. . . . . . . .
. . . . .
Vv06g0395 . . Gorai.001G129700.1 Gorai.
004G012800.1 . Gorai.006G120900.1 . . .
. . . . Vv13g0616 . .
. . . .
Vv06g0396 . . . . . . .
. . . . . . . .
. . . . .
Vv06g0397 . . . . . . .
. . . . . . . .
. . . . .
Vv06g0398 . . . . Gorai.009G079100.1
Gorai.006G121000.1 . . . . .
. . . . . . . .
.
Vv06g0399 . . . . . . .
. . . . . . . .
. . . . .
Vv06g0400 . . . . Gorai.009G079200.1
Gorai.006G121100.1 . . . . .
. . . . . . . .
.
Vv06g0401 . . . . Gorai.009G079300.1
Gorai.006G168600.1 . . . . .
. . . . . . . .
.
Vv06g0402 . . . . . . .
. . . . . . . .
. . . . .
Vv06g0403 . . . . . . .
. . . . . . . .
. . . . .
Vv06g0404 . . Gorai.001G129400.1 . .
Gorai.006G119500.1 . . . . .
. . . . . . . .
.
Vv06g0405 . . . . Gorai.009G079500.1
. . . . . . . .
. . . . . . .
Vv06g0406 . . . Gorai.004G012600.1 .

Gorai.006G119300.1 . . . . .
. . . . . . . .
.
Vv06g0407 . . . Gorai.004G012500.1 .
. . . . . . . .
. . . . . . .
Vv06g0408 . . Gorai.001G129300.1 . .
. Vv08g0836 Tc05_g005910 Gorai.006G168800.1
Gorai.002G250300.1 . . Gorai.007G374500.1
. . . . . . .
Vv06g0409 . . . . . . .
. . . . . . . .
. . . . .
Vv06g0410 . . Gorai.001G126500.1 Gorai.
004G011500.1 . Gorai.006G116300.1 . . .
. . . . Vv13g0590
Tc10_g004870 . Gorai.011G241600.1 Gorai.009G315600.1
. .
Vv06g0411 . . . . . . .
. . . . . . . .
. . . . .
Vv06g0412 Tc09_g033050 . . . . .
. . . . . . . .
. . . . . .
Vv06g0413 Tc09_g033040 . . . . .
. . . . . . . .
. . . . . .
Vv06g0414 . . . . . . .
. . . . . . . .
. . . . .
Vv06g0415 Tc09_g033030 . . . . .
. . . . . . . .
. . . . . .
Vv06g0416 Tc09_g033020 . . . . .
. . . . . . . .
. . . . . .
Vv06g0417 . . . . . . .
. . . . . . . .
. . . . .
Vv06g0418 Tc09_g033010 Gorai.013G266100.1 . .
Gorai.009G104500.1 . . . . .
. . . . . . . .
. .
Vv06g0419 Tc09_g033000 Gorai.013G266000.1 Gorai.
001G105300.1 Gorai.004G002000.1 Gorai.009G104400.1 .
Vv08g0903 . . . . . .
. . . . . . .
Vv06g0420 Tc09_g032990 . . . . .
. . . . . . . .
. . . . . .

Vv06g0421 . . . Gorai.004G002100.1
Gorai.009G104200.1 Gorai.003G139800.1 . . .
. . . . . . . .
. . .
Vv06g0422 Tc09_g032980 . Gorai.001G105400.1 .
. . . . . . . .
. . . . . . . .
Vv06g0423 . . . . . . .
. . . . . . . .
. . . . .
Vv06g0424 . . . . . . .
. . . . . . . .
. . . . .
Vv06g0425 . . . . . . .
. . . . . . . .
. . . . .
Vv06g0426 Tc09_g032960 . . . Gorai.
009G103800.1 . . . . . . .
. . . . . . . .
Vv06g0427 Tc09_g032950 . . Gorai.004G002200.1
. . . . . . . .
. Vv13g0583 . . . Gorai.
009G315700.1 . .
Vv06g0428 Tc09_g032940 . . . .
Gorai.003G139600.1 . . . . . .
. . . . . . . .
Vv06g0429 Tc09_g032930 . . . Gorai.
009G103700.1 . Vv08g0899 Tc05_g026640 . .
. . . Vv13g0252 Tc10_g004650 .
. . . .
Vv06g0430 Tc09_g032920 Gorai.013G265900.1 .
Gorai.004G002300.1 . . . . . .
. . . . . . . .
. .
Vv06g0431 Tc09_g032900 . Gorai.001G105800.1 .
. Gorai.003G139400.1 . . . .
. . . . . . . .
. .
Vv06g0432 . . . . . .
Vv08g0891 . Gorai.006G177600.1 Gorai.002G252300.1
. . . Vv13g0258 Tc10_g004580
Gorai.013G024200.1 . . . .
Vv06g0433 . . . . . . .
. . . . . . . .
. . . . .
Vv06g0434 Tc09_g032890 . . . .
Gorai.003G139300.1 . . . . . .
. . . . . . . .
Vv06g0435 Tc09_g032880 . Gorai.001G105900.1 .
. Gorai.003G139200.1 . . . .

. . . . . . . .
. .
Vv06g0436 . . . . . . .
. . . . . . . .
. . . . .
Vv06g0437 Tc09_g032870 . . . .
Gorai.003G139100.1 . . . . . .
. . . . . . . .
Vv06g0438 . . Gorai.001G106000.1 . .
. . . . . . . .
. . . . . . .
Vv06g0439 Tc09_g032790 . . . Gorai.
009G103600.1 . . . . . . .
. . . . . . . .
Vv06g0440 Tc09_g032780 . Gorai.001G106200.1 .
. . . . . . . .
. . . . . . . .
Vv06g0441 Tc09_g032770 . . . Gorai.
009G103500.1 Gorai.003G138900.1 . . . .
. . . . . . . .
. .
Vv06g0442 Tc09_g032760 . Gorai.001G106500.1 .
. . . . . . . .
. . . . . . . .
Vv06g0443 Tc09_g032750 . . Gorai.004G002400.1
Gorai.009G103400.1 Gorai.003G138700.1 . .
. . . . . . . .
. . . .
Vv06g0444 Tc09_g032740 . . . Gorai.
009G103300.1 Gorai.003G138600.1 Vv08g0851 . .
. . . . . . . .
. . .
Vv06g0445 . . . . . . .
. . . . . . . .
. . . . .
Vv06g0446 . . . . . . .
. . . . . . . .
. . . . .
Vv06g0447 . . . . . . .
. . . . . . . .
. . . . .
Vv06g0448 Tc09_g032590 . Gorai.001G106600.1 .
Gorai.009G103100.1 Gorai.003G138200.1 . .
. . . . . . . .
. . . .
Vv06g0449 . . . . Gorai.009G103000.1
. . . . . . . .
. . . . . . .
Vv06g0450 Tc09_g032560 Gorai.013G265800.1 .
Gorai.004G002700.1 Gorai.009G102900.1 Gorai.003G138000.1

. . . . . . . .
. . . . . .
Vv06g0451 Tc09_g032550 . Gorai.001G106800.1 .
. Gorai.003G137900.1 . . . .
. . . . . . . .
. .
Vv06g0452 Tc09_g032540 . Gorai.001G106900.1
Gorai.004G003100.1 . Gorai.003G137800.1 Vv08g0874
. . . . . . . .
. . . . .
Vv06g0453 Tc09_g032530 . Gorai.001G107000.1
Gorai.004G003200.1 . Gorai.003G137700.1 . .
. . . . . . . .
. . . .
Vv06g0454 . . . . . . .
. . . . . . . .
. . . . .
Vv06g0455 . . . . . . .
. . . . . . . .
. . . . .
Vv06g0456 Tc09_g032510 . . . Gorai.
009G102800.1 . . . . . . .
. Vv13g0278 . . . . .
.
Vv06g0457 Tc09_g032500 Gorai.013G265700.1 .
Gorai.004G003300.1 Gorai.009G102700.1 . . .
. . . . . . . .
. . . .
Vv06g0458 Tc09_g032490 . . Gorai.004G003400.1
. . . . . . . .
. . . . . . . .
Vv06g0459 Tc09_g032460 . . . Gorai.
009G102500.1 . . . . . . .
. . . . . . . .
Vv06g0460 Tc09_g032440 . Gorai.001G107200.1
Gorai.004G003600.1 . Gorai.003G137600.1 . .
. . . . . . . .
. . . .
Vv06g0461 Tc09_g032430 . . . Gorai.
009G102400.1 . . . . . . .
. . . . . . . .
Vv06g0462 . . . . . . .
. . . . . . . .
. . . . .
Vv06g0463 Tc09_g032420 . Gorai.001G107300.1 .
Gorai.009G102300.1 . . . .
. . . Vv13g0285 . . .
. Gorai.010G202800.1 .
Vv06g0464 . . . . . . .
. . . . . . . .

.	.	.	.	.
Vv06g0465	Tc09_g032410	.	Gorai.001G107400.1	.
Gorai.009G102200.1	Gorai.003G137500.1	Vv08g0865
.	.	.	.	.	.	Vv13g0287
.	.	.	Gorai.009G316600.1	.	.
Vv06g0466	.	.	.	.	.	.	.
.	.	.	.	.	.	.	.
.	.	.	.	.
Vv06g0467	Tc09_g032390	.	Gorai.001G107500.1	.
Gorai.009G102100.1	Gorai.003G137300.1	Vv08g0861
.	.	.	.	.	.	.	.
.	.	.	.	.
Vv06g0468	Tc09_g032380	.	.	.	.
Gorai.003G137200.1	.	.	.	.	.	.
.	Vv13g0292	Tc10_g000500	.	Gorai.
011G246000.1	.	.	.
Vv06g0469	Tc09_g032370	.	.	.	.	.
.	.	.	.	.	.	.	.
.	.	.	.	.	.
Vv06g0470	Tc09_g032350	.	.	.	Gorai.
009G102000.1	.	.	.	.	.	.	.
.	.	.	.	.	.	.	.
Vv06g0471	.	.	.	.	.	.	.
.	.	.	.	.	.	.	.
.	.	.	.	.
Vv06g0472	Tc09_g032340	.	.	.	.
Gorai.003G137100.1	.	.	.	.	.	.
.	.	.	.	.	.	.	.
Vv06g0473	Tc09_g032330	.	.	.	Gorai.
009G101900.1	Gorai.003G137000.1	.	.	.	.
.	.	.	.	.	.	.	.
.	.
Vv06g0474	Tc09_g032320	.	.	.	.
Gorai.003G136900.1	.	.	.	.	.	.
.	.	.	.	.	.	.	.
Vv06g0475	.	.	.	.	.	.	.
.	.	.	.	.	.	.	.
.	.	.	.	.
Vv06g0476	.	.	.	.	.	.	.
.	.	.	.	.	.	.	.
.	.	.	.	.
Vv06g0477	.	.	.	.	.	.	.
.	.	.	.	.	.	.	.
.	.	.	.	.
Vv06g0478	Tc09_g032290	Gorai.013G265500.1	Gorai.
001G107800.1	.	.	Gorai.003G136700.1	.	.
.	.	.	.	.	.	.	.
.	.	.	.
Vv06g0479	Tc09_g032280	Gorai.013G265400.1	.	.
Gorai.009G101700.1	Gorai.003G136600.1	.	.

. . . . . Vv13g0315
Tc10_g004200 . Gorai.011G253100.1 . . .
Vv06g0480 Tc09_g032270 . . . .
Gorai.003G136500.1 . . . . . .
. . . . . . . .
Vv06g0481 Tc09_g032260 Gorai.013G265300.1 Gorai.
001G107900.1 . . Gorai.003G136400.1 . .
. . . . . . . .
. . . .
Vv06g0482 . . . . . . .
. . . . . . . .
. . . . .
Vv06g0483 Tc09_g032240 . Gorai.001G108000.1 .
Gorai.009G101600.1 Gorai.003G136300.1 . .
. . . . . Vv13g0323
Tc10_g003860 . Gorai.011G254000.1 . . .
Vv06g0484 . . . . . . .
. . . . . . Vv13g0324
. . . . . .
Vv06g0485 . . . . . . .
. . . . . . . .
. . . . .
Vv06g0486 Tc09_g032220 Gorai.013G265200.1 . .
. Gorai.006G104800.1 . . . .
. . . . . . . .
. .
Vv06g0487 . . . . . . .
. . . . . . . .
. . . . .
Vv06g0488 Tc09_g032210 . . . Gorai.
009G101500.1 . Vv08g0923 Tc05_g005000 Gorai.
006G175000.1 Gorai.002G254400.1 . . .
Vv13g0327 Tc10_g003840 Gorai.013G023200.1 .
Gorai.009G318200.1 . .
Vv06g0489 Tc09_g032200 Gorai.013G265100.1 . .
Gorai.009G101400.1 . . . . .
. . . . . . . .
. .
Vv06g0490 . . Gorai.001G108300.1 Gorai.
004G003900.1 . Gorai.006G104600.1 . . .
. . . . . . . .
. . .
Vv06g0491 Tc09_g032170 . . . .
Gorai.006G104500.1 . . . . . .
. . . . . . . .
Vv06g0492 . Gorai.013G264900.1 . . .
Gorai.006G104400.1 . . . . .
. . . . . . . .
.
Vv06g0493 Tc09_g032160 . . . . .

. . . . . . . .
. . . . . .
Vv06g0494 Tc09_g032140 . . Gorai.004G004000.1
. . . . . . . .
. . . . . . . .
Vv06g0495 . . . . . . .
. . . . . . . .
. . . . .
Vv06g0496 Tc09_g032130 Gorai.013G264800.1 . .
. Gorai.003G135600.1 Vv08g0925
Tc05_g004990 . Gorai.002G254800.1 . . .
Vv13g0333 Tc10_g003700 Gorai.013G022900.1 .
. . Gorai.012G069600.1
Vv06g0497 Tc09_g032120 . . . .
Gorai.003G135500.1 . . . . . .
. . . . . . . .
Vv06g0498 Tc09_g032110 Gorai.013G264700.1 . .
. Gorai.006G103900.1 . . . .
. . . . . . . .
. .
Vv06g0499 . . . . . . .
. . . . . . . .
. . . . .
Vv06g0500 . . . . . Gorai.
006G101600.1 . . . . . . .
. . . . . . .
Vv06g0501 . . . . . . .
. . . . . . . .
. . . . .
Vv06g0502 . . . . . . .
. . . . . . . .
. . . . .
Vv06g0503 . . . . . . .
. . . . . . . .
. . . . .
Vv06g0504 Tc09_g032080 Gorai.013G264600.1 Gorai.
001G108500.1 Gorai.004G004300.1 . Gorai.003G135200.1
. . . . . . . .
. . . . . .
Vv06g0505 Tc09_g032070 . . Gorai.004G004400.1
. Gorai.003G135100.1 . . . .
. . . . . . . .
. .
Vv06g0506 Tc09_g032060 Gorai.013G264500.1 . .
. Gorai.003G135000.1 . . . .
. . . Vv13g0344 Tc10_g003640
Gorai.013G022700.1 . . . .
Vv06g0507 Tc09_g032050 Gorai.013G264400.1 .
Gorai.004G004600.1 . Gorai.003G134900.1 Vv08g0934
Tc05_g004880 . Gorai.002G255200.1 .

Gorai.012G102800.1 . Vv13g0346 Tc10_g003630 .
. . . .
Vv06g0508 Tc09_g032040 . . . Gorai.
009G101000.1 Gorai.003G134700.1 . . . .
. . . . . . . .
. .
Vv06g0509 Tc09_g032030 . . . Gorai.
009G100900.1 Gorai.003G134600.1 . . . .
. . . Vv13g0350 Tc10_g003610 .
Gorai.011G256100.1 . . Gorai.012G070000.1
Vv06g0510 Tc09_g032020 . Gorai.001G108600.1 .
. . Vv08g0941 Tc05_g004820 .
Gorai.002G258600.1 . . . . . .
. . . .
Vv06g0511 Tc09_g032010 . . . .
Gorai.003G134500.1 . . . . . .
. . . . . . . .
Vv06g0512 Tc09_g032000 . Gorai.001G108700.1 .
Gorai.009G100800.1 . . . . .
. . . . . . . .
. .
Vv06g0513 Tc09_g031990 Gorai.013G264300.1 Gorai.
001G108800.1 . Gorai.009G100700.1 Gorai.003G134400.1
Vv08g0943 . . . . . .
Vv13g0356 Tc10_g003480 . . . .
.
Vv06g0514 Tc09_g031980 . . . Gorai.
009G100600.1 Gorai.003G134300.1 . . . .
. . . . . . . .
. .
Vv06g0515 Tc09_g031970 . . . .
Gorai.003G134000.1 . . . . . .
. . . . . . . .
Vv06g0516 Tc09_g031960 . . . Gorai.
009G100400.1 Gorai.003G133700.1 Vv08g0944
Tc05_g004790 Gorai.006G174000.1 Gorai.002G258500.1 .
. Gorai.007G375600.1 Vv13g0357
Tc10_g003470 . Gorai.011G256600.1 . Gorai.
010G202100.1 .
Vv06g0517 Tc09_g031950 . . . Gorai.
009G100300.1 . Vv08g0945 Tc05_g004780 .
Gorai.002G258400.1 . Gorai.012G103100.1 .
Vv13g0358 Tc10_g003460 . . Gorai.009G319000.1
. .
Vv06g0518 Tc09_g031940 Gorai.013G264200.1 Gorai.
001G108900.1 . . . . . . .
. . . . . . . .
. .
Vv06g0519 Tc09_g031930 . Gorai.001G109100.1 .
Gorai.009G100200.1 Gorai.003G133600.1 . .

. . . . . Vv13g0360
Tc10_g003430 Gorai.013G022600.1 Gorai.011G256800.1
Gorai.009G319200.1 . .
Vv06g0520 . . . . . . .
. . . . . . . .
. . . . .
Vv06g0521 Tc09_g031910 . . . .
Gorai.003G133500.1 . . . . . .
. . . . . . . .
Vv06g0522 Tc09_g031900 . . . .
Gorai.003G133400.1 . . . . . .
. . . . . . . .
Vv06g0523 . . . . . . .
. . . . . . . .
. . . . .
Vv06g0524 Tc09_g031890 . Gorai.001G109300.1
Gorai.004G004800.1 Gorai.009G100100.1 Gorai.003G133200.1
Vv08g0949 Tc05_g004730 Gorai.006G173700.1 .
. Gorai.012G103400.1 . Vv13g0381 .
Gorai.013G022500.1 . Gorai.009G319300.1 .
.
Vv06g0525 Tc09_g031880 . Gorai.001G109500.1 .
Gorai.009G100000.1 Gorai.003G133100.1 . .
. . . . . . . .
. . . .
Vv06g0526 Tc09_g031870 . . . . .
. . . . . . . .
. . . . . .
Vv06g0527 . . . . . Gorai.
003G133000.1 . . . . . . .
. . . . . . .
Vv06g0528 . . . . . . .
. . . . . . . .
. . . . .
Vv06g0529 Tc09_g031840 . Gorai.001G110100.1 .
Gorai.009G099800.1 Gorai.003G132700.1 . .
. . . . . . . .
. . . .
Vv06g0530 Tc09_g031830 . Gorai.001G110200.1 .
Gorai.009G099700.1 . Vv08g0952
Tc05_g004680 . . . . . . .
. . . . .
Vv06g0531 Tc09_g031810 Gorai.013G264100.1 Gorai.
001G110400.1 Gorai.004G004900.1 . Gorai.003G132400.1
. . . . . . . .
. . . . . .
Vv06g0532 Tc09_g031800 Gorai.013G264000.1 . .
. . . . . . . .
. . . . . . . .
Vv06g0533 Tc09_g031790 . . . Gorai.

009G099500.1 Gorai.003G132300.1 . . . .
. . . . . . . .
. .
Vv06g0534 Tc09_g031780 . . . Gorai.
009G099400.1 Gorai.003G132200.1 Vv08g0958
Tc05_g004600 Gorai.006G172900.1 . . Gorai.
009G421600.1 . . . . . . .
.
Vv06g0535 Tc09_g031770 . Gorai.001G110500.1 .
. Gorai.003G132100.1 . . . .
. . . . . . . .
. .
Vv06g0536 Tc09_g031760 . . . Gorai.
009G099300.1 Gorai.003G132000.1 Vv08g0959
Tc05_g004590 . . . . Gorai.007G375900.1
. . . . . . .
Vv06g0537 . . . . . . .
. . . . . . . .
. . . . .
Vv06g0538 . . . . . .
Vv08g0960 Tc05_g004580 . Gorai.002G257700.1 .
. Gorai.007G376000.1 . . . .
. . .
Vv06g0539 . . . . . . .
. . . . . . . .
. . . . .
Vv06g0540 . . . . . . .
. . . . . . . .
. . . . .
Vv06g0541 . . . . . . .
. . . . . . . .
. . . . .
Vv06g0542 . . . . . . .
. . . . . . . .
. . . . .
Vv06g0543 . . . . . . .
. . . . . . . .
. . . . .
Vv06g0544 . . . . . . .
. . . . . . . .
. . . . .
Vv06g0545 . . . . . . .
. . . . . . . .
. . . . .
Vv06g0546 . . . . . . .
. . . . . . . .
. . . . .
Vv06g0547 . . . . . . .
. . . . . . . .
. . . . .

Vv06g0548 . . . . . . .
. . . . . . . .
. . . . .
Vv06g0549 . . Gorai.001G110600.1 . .
. . . . . . . .
. . . . . . .
Vv06g0550 Tc09_g031750 . Gorai.001G110700.1 .
. . . . . . . .
. . . . . . . .
Vv06g0551 . . . . . . .
. . . . . . . .
. . . . .
Vv06g0552 Tc09_g031700 Gorai.013G263900.1 . .
. Gorai.003G131900.1 Vv08g0962 . .
. . . . . . . .
. . .
Vv06g0553 . . . . . . .
. . . . . . . .
. . . . .
Vv06g0554 . . . . . . .
. . . . . . . .
. . . . .
Vv06g0555 Tc09_g031660 Gorai.013G263800.1 . .
. . . . . . . .
. . . . . . . .
Vv06g0556 . . . . . . .
. . . . . . . .
. . . . .
Vv06g0557 Tc09_g031650 Gorai.013G263700.1 Gorai.
001G110900.1 . Gorai.009G099200.1 . . .
. . . . . . . .
. . . .
Vv06g0558 . . . . . . .
. . . . . . . .
. . . . .
Vv06g0559 Tc09_g031630 . . Gorai.004G005000.1
Gorai.009G099100.1 Gorai.003G131500.1 . .
. . . . . . . .
. . . .
Vv06g0560 Tc09_g031620 Gorai.013G263600.1 . .
Gorai.009G099000.1 . . . . .
. . . . . . . .
. .
Vv06g0561 Tc09_g031610 Gorai.013G263500.1 . .
Gorai.009G098900.1 . . . . .
. . . . . . . .
. .
Vv06g0562 . . . . . . .
. . . . . . . .
. . . . .

Vv06g0563 . . . . . . .
. . . . . . . .
. . . . .
Vv06g0564 . Gorai.013G263400.1 . . .
. . . . . . . .
Vv13g0392 Tc10_g003060 . Gorai.011G265400.1
. Gorai.010G201400.1 Gorai.012G071000.1
Vv06g0565 Tc09_g031590 . . . . .
. . . . . . . .
. . . . . .
Vv06g0566 . . . . . . .
. . . . . . Vv13g0394
. . . . . .
Vv06g0567 Tc09_g031580 . Gorai.001G111100.1 .
Gorai.009G098800.1 Gorai.003G131000.1 Vv08g0967
Tc05_g004480 Gorai.006G172400.1 Gorai.002G257400.1
. Gorai.012G104400.1 Gorai.007G376300.1
Vv13g0395 . Gorai.013G022200.1 Gorai.011G266000.1
. . .
Vv06g0568 . . . . . . .
. . . . . . . .
. . . . .
Vv06g0569 . . . . . . .
. . . . . . . .
. . . . .
Vv06g0570 Tc09_g031570 . . . Gorai.
009G098700.1 Gorai.003G130900.1 Vv08g0970
Tc05_g004460 Gorai.006G172200.1 . . . .
. . . . . . .
Vv06g0571 Tc09_g031560 . Gorai.001G111200.1 .
Gorai.009G098600.1 . . . . .
. . . . . . . .
. .
Vv06g0572 Tc09_g031550 . Gorai.001G111300.1 .
. . . . . . . .
. . . . . . . .
Vv06g0573 Tc09_g031540 . Gorai.001G111400.1 .
Gorai.009G098500.1 Gorai.003G130800.1 . .
. . . . . . . .
. . . .
Vv06g0574 Tc09_g031530 . . . . .
Vv08g0971 Tc05_g004450 Gorai.006G172100.1 .
. . . . . . . .
. .
Vv06g0575 . . . . Gorai.009G098400.1
. . . . . . . .
. . . . . . .
Vv06g0576 Tc09_g031520 . . . Gorai.
009G098300.1 Gorai.003G130700.1 Vv08g0973
Tc05_g004440 . . . . . Vv13g0396

. Gorai.013G021300.1 Gorai.011G266100.1 .
. .
Vv06g0577 Tc09_g031510 . Gorai.001G111500.1
Gorai.004G005200.1 . Gorai.003G130600.1 . .
. . . . . . . .
. . . .
Vv06g0578 Tc09_g031500 . . . Gorai.
009G098200.1 Gorai.003G130500.1 . . . .
. . . Vv13g0398 . . .
. . .
Vv06g0579 Tc09_g031490 . Gorai.001G111600.1 .
. . Vv08g0975 Tc05_g004430 . .
. . Gorai.007G376400.1 Vv13g0400 .
. . . . .
Vv06g0580 Tc09_g031480 . . . Gorai.
009G098100.1 Gorai.003G130400.1 . . . .
. . . Vv13g0402 . . .
. . .
Vv06g0581 Tc09_g031460 . . . Gorai.
009G098000.1 Gorai.003G130300.1 . . . .
. . . . . . . .
. .
Vv06g0582 Tc09_g031450 . . . .
Gorai.003G130200.1 . . . . . .
. . . . . . . .
Vv06g0583 Tc09_g031440 . . Gorai.004G005300.1
. Gorai.003G130000.1 Vv08g0978
Tc05_g004400 . Gorai.002G257300.1 . Gorai.
012G104700.1 Gorai.007G376500.1 . . . .
. . .
Vv06g0584 Tc09_g031420 . . . Gorai.
009G097900.1 . . . . . . .
. . . . . . . .
Vv06g0585 Tc09_g031410 . . . Gorai.
009G097800.1 . . . . . . .
. . . . . . . .
Vv06g0586 Tc09_g031400 . Gorai.001G111700.1 .
. Gorai.003G129900.1 . . . .
. . . . . . . .
. .
Vv06g0587 . . . . . . .
. . . . . . . .
. . . . .
Vv06g0588 Tc09_g031350 . . . Gorai.
009G097300.1 . . . . . . .
. . . . . . . .
Vv06g0589 Tc09_g031340 Gorai.013G263100.1 . .
. Gorai.003G129400.1 . . . .
. . . . . . . .
. .

Vv06g0590 . . . . . . .
. . . . . . . .
. . . . .
Vv06g0591 Tc09_g031330 . Gorai.001G111900.1 .
. Gorai.003G129300.1 . . . .
. . . . . . . .
. .
Vv06g0592 Tc09_g031320 . . . . .
. . . . . . .
Vv13g0416 . . . . . .
Vv06g0593 . . Gorai.001G112000.1 Gorai.
004G005400.1 Gorai.009G097200.1 Gorai.003G129200.1 .
. . . . . . Vv13g0417
. . . . . .
Vv06g0594 Tc09_g031310 . Gorai.001G112100.1 .
. . . . . . . .
. . . . . . . .
Vv06g0595 . . . . . . .
. . . . . . . .
. . . . .
Vv06g0596 Tc09_g031300 . Gorai.001G112200.1 .
Gorai.009G097100.1 Gorai.003G129100.1 . .
. . . . . . . .
. . . .
Vv06g0597 Tc09_g031290 Gorai.013G263000.1 . .
. . . . . . . .
. . . . . . . .
Vv06g0598 Tc09_g031280 . . . Gorai.
009G097000.1 . . . . . . .
. . . . . . . .
Vv06g0599 Tc09_g031270 . . Gorai.004G005500.1
. . . . . . . .
. . . . . . . .
Vv06g0600 Tc09_g031260 Gorai.013G262900.1 Gorai.
001G112300.1 . Gorai.009G096900.1 . . .
. . . . . . . .
. . . .
Vv06g0601 Tc09_g031250 . . . . .
. . . . . . . .
. . . . . .
Vv06g0602 Tc09_g031230 . . . . .
. . . . . . . .
. . . . . .
Vv06g0603 . . . . . .
Vv08g1004 Tc05_g004060 . . . Gorai.
012G106000.1 . Vv13g0423 . . Gorai.
011G267600.1 . Gorai.010G200600.1 .
Vv06g0604 Tc09_g031210 . . . .
Gorai.003G129000.1 . . . . . .
. . . . . . . .

Vv06g0605 Tc09_g031190 . Gorai.001G112500.1 .
Gorai.009G096500.1 . . . . .
. . . . . . . .
. .
Vv06g0606 Tc09_g031180 Gorai.013G262700.1 . .
. . . . . . . .
. . . . . . . .
Vv06g0607 . . . . . . .
. . . . . . . .
. . . . .
Vv06g0608 Tc09_g031170 . Gorai.001G112600.1 .
. . . . . . . .
. . . . . . . .
Vv06g0609 . . . . . . .
. . . . . . . .
. . . . .
Vv06g0610 . . . . . .
Vv08g1008 Tc05_g004020 . . . . .
Vv13g0431 . . Gorai.011G268000.1 .
. .
Vv06g0611 Tc09_g031160 . Gorai.001G112700.1 .
. . . . . . . .
. . . . . . . .
Vv06g0612 . Gorai.013G262600.1 . .
Gorai.009G096400.1 . . . . . .
. . . . . . . .
.
Vv06g0613 . . . . . . .
. . . . . . . .
. . . . .
Vv06g0614 . . . . . . .
. . . . . . . .
. . . . .
Vv06g0615 Tc09_g031150 . . . . .
Vv08g1013 Tc05_g003990 . . . .
. . . . . . . .
Vv06g0616 . Gorai.013G262500.1 . .
Gorai.009G096300.1 . . . . . .
. . . . . . . .
.
Vv06g0617 Tc09_g031140 . . . . .
. . . . . . . .
. . . . . .
Vv06g0618 . . . . . . .
. . . . . . . .
. . . . .
Vv06g0619 . Gorai.013G262400.1 . . .
. . . . . . . .
. . . . . . .
Vv06g0620 Tc09_g031130 . . . . .

| | | | | | | | |
|---|---|---|---|---|---|---|---|
| . | . | . | . | . | . | . | . |
| . | . | . | . | . | . | | |
| Vv06g0621 | . | Gorai.013G262300.1 | | | . | . | . |
| . | Vv08g1014 | | . | . | . | . | . |
| . | . | . | . | . | . | . | . |
| Vv06g0622 | Tc09_g031120 | | . | . | . | . | . |
| . | . | . | . | . | . | . | . |
| . | . | . | . | . | . | | |
| Vv06g0623 | . | . | . | . | . | . | . |
| . | . | . | . | . | . | . | . |
| . | . | . | . | . | | | |
| Vv06g0624 | Tc09_g031110 | | Gorai.013G262200.1 | | | . | . |
| . | . | . | . | . | . | . | . |
| . | . | . | . | . | . | . | . |
| Vv06g0625 | . | . | . | . | . | . | . |
| . | . | . | . | . | . | . | . |
| . | . | . | . | . | | | |
| Vv06g0626 | . | . | . | . | . | . | . |
| . | . | . | . | . | . | . | . |
| . | . | . | . | . | | | |
| Vv06g0627 | Tc09_g031100 | | . | . | . | . | . |
| . | . | . | . | . | . | . | . |
| . | . | . | . | . | . | | |
| Vv06g0628 | . | . | . | . | . | . | . |
| . | . | . | . | . | . | . | . |
| . | . | . | . | . | | | |
| Vv06g0629 | Tc09_g031090 | | Gorai.013G262100.1 | | | . | . |
| . | . | . | . | . | . | . | . |
| . | . | . | . | . | . | . | . |
| Vv06g0630 | . | . | . | . | . | . | . |
| . | . | . | . | . | . | . | . |
| . | . | . | . | . | | | |
| Vv06g0631 | . | . | . | . | . | . | . |
| . | . | . | . | . | . | . | . |
| . | . | . | . | . | | | |
| Vv06g0632 | . | . | . | . | . | . | . |
| . | . | . | . | . | . | . | . |
| . | . | . | . | . | | | |
| Vv06g0633 | . | . | . | . | . | . | . |
| . | . | . | . | . | . | . | . |
| . | . | . | . | . | | | |
| Vv06g0634 | Tc09_g031080 | | . | . | . | . | . |
| . | . | . | . | . | . | . | . |
| . | . | . | . | . | . | | |
| Vv06g0635 | Tc09_g031070 | | Gorai.013G262000.1 | | | . | . |
| . | . | . | . | . | . | . | . |
| . | . | . | . | . | . | . | . |
| Vv06g0636 | . | . | . | . | . | . | . |
| . | . | . | . | . | . | . | . |
| . | . | . | . | . | | | |

Vv06g0637 . . . . . . .
. . . . . . . .
. . . . .
Vv06g0638 Tc09_g031060 . . . Gorai.
009G096200.1 Gorai.003G128900.1 Vv13g0440
Tc10_g001270 . . . . . . .
. . . . .
Vv06g0639 Tc09_g031050 Gorai.013G261900.1 . .
. Gorai.003G128800.1 Vv13g0441 . .
. . . . . . . .
. . .
Vv06g0640 . Gorai.013G261800.1 . . .
Gorai.003G128700.1 . . . . .
. . . . . . . .
.
Vv06g0641 Tc09_g031020 . . . .
Gorai.003G128600.1 . . . . . .
. . . . . . . .
Vv06g0642 Tc09_g031010 . . . .
Gorai.003G128500.1 . . . . . .
. . . . . . . .
Vv06g0643 . . . . . . .
. . . . . . . .
. . . . .
Vv06g0644 Tc09_g031000 . . Gorai.004G006200.1
Gorai.009G096000.1 . . . . .
. . . . . . . .
. .
Vv06g0645 . . . . . . .
. . . . . . . .
. . . . .
Vv06g0646 Tc09_g030990 . Gorai.001G112800.1 .
Gorai.009G095800.1 Gorai.003G128300.1 . .
. . . . . . . .
. . . .
Vv06g0647 Tc09_g030980 . Gorai.001G112900.1 .
. Gorai.003G128200.1 . . . .
. . . . . . . .
. .
Vv06g0648 Tc09_g030970 . Gorai.001G113000.1
Gorai.004G006300.1 Gorai.009G095700.1 Gorai.003G128100.1
. . . . . . . .
. . . . . .
Vv06g0649 Tc09_g030960 Gorai.013G261600.1 . .
. Gorai.003G127900.1 . . . .
. . . . . . . .
. .
Vv06g0650 . . . . . . .
. . . . . . . .
. . . . .

Vv06g0651 . . . . . . .
. . . . . . . .
. . . . .
Vv06g0652 . . . . . . .
. . . . . . . .
. . . . .
Vv06g0653 . . . . . . .
. . . . . . . .
. . . . .
Vv06g0654 . . . . . . .
. . . . . . . .
. . . . .
Vv06g0655 . . . . . . .
. . . . . . . .
. . . . .
Vv06g0656 Tc09_g030940 . . . Gorai.
009G095600.1 . . . . . . .
. . . . . . . .
Vv06g0657 . . . . . . .
. . . . . . . .
. . . . .
Vv06g0658 Tc09_g030930 . . . Gorai.
009G095500.1 . . . . . . .
. . . . . . . .
Vv06g0659 . . . . . . .
. . . . . . . .
. . . . .
Vv06g0660 Tc09_g030910 . . . .
Gorai.003G127800.1 . . . . . .
. . . . . . . .
Vv06g0661 . . Gorai.001G113500.1 . .
. . . . . . . .
. . . . . . .
Vv06g0662 Tc09_g030900 Gorai.013G179300.1 . .
. . . . . . . .
. . . . . . . .
Vv06g0663 . . . . . . .
. . . . . . . .
. . . . .
Vv06g0664 Tc09_g030890 Gorai.013G177600.1 . .
. . . . . . . .
. . . . . . . .
Vv06g0665 . Gorai.013G177400.1 . . .
Gorai.003G127600.1 . . . . . .
. . . . . . . .
.
Vv06g0666 . . . . . . .
. . . . . . . .
. . . . .
Vv06g0667 Tc09_g030860 Gorai.013G177300.1 . .

.	Gorai.003G127500.1	.	.	.	.
.	.	.	.	.	.	.	.
.	.
Vv06g0668	.	.	.	.	.	.	.
.	.	.	.	.	.	.	.
.	.	.	.	.
Vv06g0669	.	.	.	.	.	.	.
.	.	.	.	.	.	.	.
.	.	.	.	.
Vv06g0670	.	.	.	.	.	.	.
.	.	.	.	.	.	.	.
.	.	.	.	.
Vv06g0671	Tc09_g030850	.	.	.	.
Gorai.003G127400.1	Vv08g1062	Tc05_g003500	.	.
.	.	.	.	.	.	.	.
.	.
Vv06g0672	.	.	.	.	.	.	.
.	.	.	.	.	.	.	.
.	.	.	.	.
Vv06g0673	Tc09_g030830	.	.	Gorai.004G006400.1
Gorai.009G095200.1	.	.	.	.	.
.	.	.	.	.	.	.	.
.	.
Vv06g0674	Tc09_g030820	.	.	.	.
Gorai.003G127000.1	.	.	.	.	.	.
.	.	.	.	.	.	.	.
Vv06g0675	Tc09_g030810	.	.	.	.
Gorai.003G126900.1	Vv08g1066	Tc05_g003460	.
Gorai.002G259000.1	.	.	.	.	.	.
.	.	.	.
Vv06g0676	Tc09_g030710	.	Gorai.001G114100.1
Gorai.004G006500.1	Gorai.009G095000.1	.	.	.
.	.	.	.	.	.	.	.
.	.	.	.
Vv06g0677	Tc09_g030690	.	.	.	.	.
.	.	.	.	.	.	.	.
.	.	.	.	.	.
Vv06g0678	.	.	.	.	.	.	.
.	.	.	.	.	.	.	.
.	.	.	.	.
Vv06g0679	Tc09_g030660	.	.	.	Gorai.
009G094900.1	Gorai.003G126700.1	Vv08g1068
Tc05_g003360	Gorai.006G179200.1	.	.	.	.
.	.	.	.	.	.	.
Vv06g0680	Tc09_g030650	.	.	.	.	.
Vv08g1070	Tc05_g003330	Gorai.006G179400.1
Gorai.002G259100.1	.	Gorai.012G109300.1	Gorai.
007G377600.1	.	.	.	.	.	.	.
Vv06g0681	Tc09_g030640	.	Gorai.001G114400.1	.
Gorai.009G094800.1	Gorai.003G126600.1	.	.

. . . . . . . .
. . . .
Vv06g0682 . . . . . . .
. . . . . . . .
. . . . .
Vv06g0683 Tc09_g030620 . . . .
Gorai.003G126400.1 . . . . . .
. . . . . . . .
Vv06g0684 . . . . . .
Vv08g1072 Tc05_g003310 Gorai.006G179600.1 . .
Gorai.012G109400.1 . . . . .
. . .
Vv06g0685 . . . . . .
Vv08g1076 Tc05_g003270 . Gorai.002G259300.1
Gorai.002G148300.1 . Gorai.007G377700.1 . .
. . . . .
Vv06g0686 Tc09_g030610 . Gorai.001G114500.1 .
. . . . . . . .
. . . . . . . .
Vv06g0687 Tc09_g030600 Gorai.013G261300.1 . .
Gorai.009G094600.1 Gorai.003G126300.1 Vv08g1086
Tc05_g003210 Gorai.006G179900.1 . . .
. . . . . . . .
Vv06g0688 . . . . . . .
. . . . . . . .
. . . . .
Vv06g0689 Tc09_g030590 . . . .
Gorai.003G125700.1 . . . . . .
. . . . . . . .
Vv06g0690 . . . . . . .
. . . . . . . .
. . . . .
Vv06g0691 . . . . . . .
. . . . . . . .
. . . . .
Vv06g0692 Tc09_g030530 . . . Gorai.
009G094400.1 Gorai.003G125400.1 Vv08g1093
Tc05_g003130 . Gorai.002G260400.1 . . .
. . . . . . .
Vv06g0693 Tc09_g030520 Gorai.013G261200.1 . .
. . . . . . . .
. . . . . . . .
Vv06g0694 Tc09_g030510 . . Gorai.004G006600.1
. . Vv08g1094 Tc05_g003120 .
Gorai.002G260500.1 . . . . . .
. . . .
Vv06g0695 Tc09_g030500 Gorai.013G261100.1 . .
. Gorai.003G125300.1 . . . .
. . . . . . . .
. .

Vv06g0696 . . . . . . .
. . . . . . . .
. . . . .
Vv06g0697 . . . . . . .
. . . . . . . .
. . . . .
Vv06g0698 Tc09_g030480 . Gorai.001G114600.1 .
. . . . . . . .
. . . . . . . .
Vv06g0699 Tc09_g030470 Gorai.013G175100.1 . .
Gorai.009G094300.1 . . . . .
. . . . . . . .
. .
Vv06g0700 Tc09_g030460 . . . .
Gorai.003G125200.1 . . . . . .
. . . . . . . .
Vv06g0701 . . . . . . .
. . . . . . . .
. . . . .
Vv06g0702 . Gorai.013G173100.1 . . .
. . . . . . . .
. . . . . . .
Vv06g0703 . . . . . . .
. . . . . . . .
. . . . .
Vv06g0704 Tc09_g030450 . . . .
Gorai.003G125100.1 . . . . . .
. . . . . . . .
Vv06g0705 . . . . . . .
. . . . . . . .
. . . . .
Vv06g0706 Tc09_g030440 Gorai.013G261000.1 . .
. Gorai.003G125000.1 . . . .
. . . . . . . .
. .
Vv06g0707 Tc09_g030430 Gorai.013G260900.1 . .
. . . . . . . .
. . . . . . . .
Vv06g0708 Tc09_g030420 . Gorai.001G114800.1 .
. . . . . . . .
. . . . . . . .
Vv06g0709 Tc09_g030410 . . . .
Gorai.003G124900.1 Vv08g1107 Tc05_g002990 Gorai.
006G180500.1 . . . . . . .
. . . .
Vv06g0710 . . . . Gorai.009G094100.1
Gorai.003G124800.1 . . . . .
. . . . . . . .
.
Vv06g0711 Tc09_g030390 . Gorai.001G114900.1 .

. . Vv08g1110 Tc05_g002950 .
Gorai.002G261000.1 . Gorai.012G111800.1 Gorai.
007G378100.1 . . . . . . .
Vv06g0712 . . . . . . .
. . . . . . . .
. . . . .
Vv06g0713 . Gorai.013G260800.1 . . .
. . . . . . . .
. . . . . . .
Vv06g0714 Tc09_g030360 . . . Gorai.
009G093900.1 . Vv08g1114 Tc05_g002910 .
Gorai.002G261400.1 . . . . . .
. . . .
Vv06g0715 Tc09_g030350 . . . Gorai.
009G093800.1 Gorai.003G124600.1 . . . .
. . . . . . . .
. .
Vv06g0716 Tc09_g030340 . . . Gorai.
009G093700.1 . Vv08g1116 Tc05_g002890 Gorai.
006G180900.1 Gorai.002G261600.1 . Gorai.012G112000.1
Gorai.007G378300.1 . . . . .
. .
Vv06g0717 Tc09_g030320 Gorai.013G260700.1 . .
Gorai.009G093600.1 . . . . .
. . . . . . . .
. .
Vv06g0718 Tc09_g030300 . . Gorai.004G006800.1
Gorai.009G093400.1 Gorai.003G124500.1 . .
. . . . . . . .
. . . .
Vv06g0719 Tc09_g030290 . . . Gorai.
009G093300.1 Gorai.003G124400.1 . . . .
. . . . . . . .
. .
Vv06g0720 . . . . . . .
. . . . . . . .
. . . . .
Vv06g0721 Tc09_g030280 . . . .
Gorai.003G124300.1 . . . . . .
. . . . . . . .
Vv06g0722 Tc09_g030270 . . Gorai.004G006900.1
. . . . . . . .
. . . . . . . .
Vv06g0723 . . . . . . .
. . . . . . Vv13g0008
Tc10_g002060 . . . . .
Vv06g0724 Tc09_g030250 . . . Gorai.
009G093200.1 . . . . . . .
. . . . . . . .
Vv06g0725 . . . . . . .

. . . . . . . .
. . . . .
Vv06g0726 Tc09_g030220 . Gorai.001G115100.1 .
Gorai.009G092900.1 Gorai.003G123900.1 . .
. . . . . . . .
. . . .
Vv06g0727 Tc09_g030210 . . . .
Gorai.003G123800.1 . . . . . .
. . . . . . . .
Vv06g0728 Tc09_g030200 . Gorai.001G115200.1 .
Gorai.009G092800.1 . . . . .
. . . . . . . .
. .
Vv06g0729 . . . . . . .
. . . . . . . .
. . . . .
Vv06g0730 . . . . . . .
. . . . . . . .
. . . . .
Vv06g0731 . . . . . .
Vv08g1118 Tc05_g002860 . Gorai.002G261700.1 .
Gorai.012G112200.1 . . . . .
. . .
Vv06g0732 . . . . . . .
. . . . . . . .
. . . . .
Vv06g0733 Tc09_g030190 . . . .
Gorai.003G123700.1 . . . . . .
. . . . . . . .
Vv06g0734 Tc09_g030180 . Gorai.001G115300.1 .
. . . . . . . .
. . . . . . . .
Vv06g0735 Tc09_g030170 . Gorai.001G115400.1
Gorai.004G007000.1 . Gorai.003G123600.1 . .
. . . . . Vv13g0017 .
. Gorai.011G283500.1 . Gorai.010G198700.1
.
Vv06g0736 Tc09_g030160 . . . .
Gorai.003G123500.1 . . . . . .
. . . . . . . .
Vv06g0737 Tc09_g030150 . . . .
Gorai.003G123400.1 . . . . . .
. Vv13g0020 . Gorai.013G019000.1
Gorai.011G283700.1 . . Gorai.012G076000.1
Vv06g0738 Tc09_g030140 . Gorai.001G115500.1 .
. . . . . . . .
. Vv13g0025 . . . Gorai.
009G322200.1 . .
Vv06g0739 Tc09_g030130 . . . .
Gorai.003G123300.1 . . . . . .

. . . . . . . .
Vv06g0740 Tc09_g030120 Gorai.013G260600.1 Gorai.
001G115600.1 . . . . . . .
. . . Vv13g0028 Tc10_g001620 .
. Gorai.009G322300.1 . .
Vv06g0741 . . . . . . .
. . . . . . . .
. . . . .
Vv06g0742 . . . . . . .
. . . . . . . .
. . . . .
Vv06g0743 . . . . . . .
. . . . . . . .
. . . . .
Vv06g0744 Tc09_g030100 . . . . .
. . . . . . . .
. . . . . .
Vv06g0745 . . . . . . .
. . . . . . . .
. . . . .
Vv06g0746 Tc09_g030090 Gorai.013G260500.1 . .
. Gorai.003G123200.1 . . . .
. . . . . . . .
. .
Vv06g0747 . . . . . . .
. . . . . . . .
. . . . .
Vv06g0748 Tc09_g030080 . . . Gorai.
009G092600.1 . . . . . . .
. . . . . . . .
Vv06g0749 . . . . . . .
. . . . . . . .
. . . . .
Vv06g0750 Tc09_g030060 . . . Gorai.
009G092500.1 . . . . . . .
. . . . . . . .
Vv06g0751 . . . . . . .
. . . . . . . .
. . . . .
Vv06g0752 . . . . . . .
. . . . . . . .
. . . . .
Vv06g0753 . . . . . . .
. . . . . . . .
. . . . .
Vv06g0754 . . . . . . .
. . . . . . . .
. . . . .
Vv06g0755 . . . . . . .
. . . . . . . .

. . . . .
Vv06g0756 . . . . . . .
. . . . . . . .
. . . . .
Vv06g0757 Tc09_g030040 . . . Gorai.
009G092400.1 . . . . . . .
. . . . . . . .
Vv06g0758 Tc09_g030030 . . . .
Gorai.003G120900.1 . . . . . .
. . . . . . . .
Vv06g0759 . . . . . . .
. . . . . . . .
. . . . .
Vv06g0760 Tc09_g030020 . . . . .
. . . . . . . .
. . . . . .
Vv06g0761 Tc09_g030010 Gorai.013G260400.1 . .
Gorai.009G092300.1 . . . . .
. . . . . . . .
. .
Vv06g0762 Tc09_g030000 . . . .
Gorai.006G106800.1 . . . . . .
. . . . . . . .
Vv06g0763 . Gorai.013G260300.1 . . .
. . . . . . . .
. . . . . . .
Vv06g0764 . . . . . . .
. . . . . . . .
. . . . .
Vv06g0765 Tc09_g029980 . Gorai.001G116000.1 .
. . . . . . . .
. . . . . . . .
Vv06g0766 . . . . . . .
. . . . . . . .
. . . . .
Vv06g0767 Tc09_g029970 . . . . .
. . . . . . . .
. . . . . .
Vv06g0768 Tc09_g029960 . . Gorai.004G007100.1
Gorai.009G092200.1 . Vv08g1148
Tc05_g002540 Gorai.006G182300.1 . . Gorai.
012G113600.1 . . . . . . .
.
Vv06g0769 . . . . . . .
. . . . . . . .
. . . . .
Vv06g0770 Tc09_g029940 . . . .
Gorai.006G106700.1 . . . . . .
. . . . . . . .
Vv06g0771 Tc09_g029930 . Gorai.001G116100.1 .

. . . . . . . .
. . . . . . . .
Vv06g0772 Tc09_g029920 . . . Gorai.
009G092100.1 . . . . . . .
. Vv13g0056 Tc10_g001550 . Gorai.
011G287800.1 . Gorai.010G198100.1 .
Vv06g0773 Tc09_g029910 . . . .
Gorai.006G106600.1 . . . . . .
. Vv13g0057 Tc10_g001540 . Gorai.
011G287900.1 . . .
Vv06g0774 Tc09_g029900 . . . Gorai.
009G092000.1 . Vv08g0344 Tc05_g002480 .
Gorai.002G263500.1 . . . . . .
. . . .
Vv06g0775 Tc09_g029890 . Gorai.001G116200.1 .
. . . . . . . .
. . . . . . . .
Vv06g0776 . . . . . . .
. . . . . . . .
. . . . .
Vv06g0777 . . . . . . .
. . . . . . . .
. . . . .
Vv06g0778 . . . . . . .
. . . . . . . .
. . . . .
Vv06g0779 Tc09_g029880 . . . Gorai.
009G091700.1 Gorai.006G106400.1 . . . .
. . . Vv13g0058 Tc10_g001510 .
. . Gorai.010G198000.1 Gorai.012G076900.1
Vv06g0780 Tc09_g029870 . . . Gorai.
009G091600.1 . Vv08g0345 . . . .
. . . . . . . .
.
Vv06g0781 Tc09_g029860 . . . Gorai.
009G091400.1 . . . . . . .
. Vv13g0059 Tc10_g001480 . Gorai.
011G288000.1 . Gorai.010G197900.1 .
Vv06g0782 Tc09_g029810 . . Gorai.004G007200.1
. . . . . . . .
. . . . . . . .
Vv06g0783 . . . . . . .
. . . . . . . .
. . . . .
Vv06g0784 Tc09_g029790 . . . Gorai.
009G091300.1 . . . . . . .
. Vv13g0065 Tc10_g001410 . Gorai.
011G289100.1 Gorai.009G322600.1 . .
Vv06g0785 Tc09_g029780 . Gorai.001G116400.1
Gorai.004G007300.1 Gorai.009G091200.1 Gorai.006G106300.1

. . . . . . .
Vv13g0068 Tc10_g001380 Gorai.013G017600.1 Gorai.
011G289400.1 . Gorai.010G196500.1 .
Vv06g0786 . . . . . . .
. . . . . . . .
. . . . .
Vv06g0787 Tc09_g029760 . . . . .
. . . . . . . .
. . . . . .
Vv06g0788 Tc09_g029750 . Gorai.001G116500.1 .
. Gorai.006G106100.1 Vv08g0357
Tc05_g002310 . Gorai.002G262800.1 . Gorai.
012G115000.1 . Vv13g0073 Tc10_g001360 .
Gorai.011G289600.1 . . .
Vv06g0789 . . . . . . .
. . . . . . . .
. . . . .
Vv06g0790 Tc09_g029740 . . . Gorai.
009G091000.1 Gorai.006G106000.1 . . . .
. . . . . . . .
. .
Vv06g0791 Tc09_g029720 . Gorai.001G116600.1 .
. Gorai.006G105800.1 . . . .
. . . . . . . .
. .
Vv06g0792 Tc09_g029710 Gorai.013G259800.1 . .
. Gorai.006G105700.1 . . . .
. . . . . . . .
. .
Vv06g0793 . . . . . . .
. . . . . . . .
. . . . .
Vv06g0794 Tc09_g029690 . . . Gorai.
009G090900.1 Gorai.006G105500.1 . . . .
. . . . . . . .
. .
Vv06g0795 . . . . . . .
. . . . . . . .
. . . . .
Vv06g0796 Tc09_g029670 . . . .
Gorai.006G104200.1 . . . . . .
. . . . . . . .
Vv06g0797 . . Gorai.001G116700.1 . .
Gorai.006G101600.1 . . . . .
. . . . . . . .
.
Vv06g0798 Tc09_g029660 . Gorai.001G116900.1 .
. Gorai.006G105300.1 Vv08g0368
Tc05_g002230 . . . Gorai.012G115400.1 .
Vv13g0077 Tc10_g001320 . . . .

Gorai.012G077500.1
Vv06g0799 . Gorai.013G259500.1 Gorai.001G117000.1
. Gorai.009G090500.1 . . . .
. . . . . . . .
. . .
Vv06g0800 . . . . . . .
. . . . . . . .
. . . . .
Vv06g0801 . . . . . . .
. . . . . . Vv13g0084
Tc10_g001270 . . . . Gorai.
012G077700.1
Vv06g0802 . Gorai.013G269600.1 . .
Gorai.009G111600.1 Gorai.003G146500.1 . . .
. . . . . . . .
. . .
Vv06g0803 . . . . . . .
. . . . . . . .
. . . . .
Vv06g0804 . . . . . . .
. . . . . . Vv13g0085
Tc10_g001260 Gorai.013G017300.1 . Gorai.
009G322700.1 Gorai.010G196100.1 Gorai.012G077900.1
Vv06g0805 . . . . . .
Vv08g0381 Tc05_g002150 Gorai.006G183700.1 Gorai.
002G262700.1 . . . . . . .
. . .
Vv06g0806 . Gorai.013G269700.1 . .
Gorai.009G111700.1 . Vv08g0382 Tc05_g002140 .
Gorai.002G262600.1 . . . . .
. . . . .
Vv06g0807 . . . . . . .
. . . . . . . .
. . . . .
Vv06g0808 . . . . . .
Vv08g0386 Tc05_g002100 Gorai.006G183900.1 . .
. . . . . . . .
.
Vv06g0809 . . . . . Gorai.
003G146800.1 . . . . . . .
. . . . . . .
Vv06g0810 . . . . . . .
. . . . . . . .
. . . . .
Vv06g0811 . . . . . Gorai.
003G146900.1 . . . . . . .
. . . . . . .
Vv06g0812 . . . . . . .
. . . . . . . .
. . . . .

Vv06g0813 . . . . . Gorai.
003G147200.1 . . . . . . .
Vv13g0092 . . . . . .
Vv06g0814 . Gorai.013G269900.1 . . .
Gorai.003G147300.1 . . . . .
. . . . . . . .
.
Vv06g0815 . . . . Gorai.009G111900.1
. Vv08g0418 Tc05_g001960 Gorai.006G184200.1
Gorai.002G265100.1 . Gorai.012G117500.1 .
. . . . . . .
Vv06g0816 . . . . . . .
. . . . . . . .
. . . . .
Vv06g0817 . . . . . . .
. . . . . . . .
. . . . .
Vv06g0818 . . . . . . .
. . . . . . . .
. . . . .
Vv06g0819 . Gorai.013G270100.1 . . .
Gorai.003G147500.1 . . . . .
. . . . . . . .
.
Vv06g0820 . . . . . . .
. . . . . . . .
. . . . .
Vv06g0821 . . . . Gorai.009G112300.1
. Vv08g0435 Tc05_g001910 . Gorai.
002G265400.1 . Gorai.012G117800.1 . Vv13g0101
Tc10_g001130 . . . . .
Vv06g0822 . . . . Gorai.009G112400.1
. . . . . . . .
. . . . . . .
Vv06g0823 . Gorai.013G270200.1 . . .
Gorai.003G147900.1 Vv08g0436 Tc05_g001900 .
Gorai.002G265500.1 . . . . .
. . . . .
Vv06g0824 . . . . . . .
. . . . . . . .
. . . . .
Vv06g0825 Tc09_g005700 . . . .
Gorai.003G148100.1 . . . . . .
. . . . . . . .
Vv06g0826 . Gorai.013G270400.1 . . .
Gorai.003G148200.1 Vv08g0448 . . .
. . . . . . . .
. .
Vv06g0827 Tc09_g005470 . . . . .
. . . . . . . .

. . . . . .
Vv06g0828 . . . . . . .
. . . . . . . .
. . . . .
Vv06g0829 . Gorai.013G270500.1 . . .
Gorai.003G148700.1 . . . . .
. . . . . . . .
.
Vv06g0830 . Gorai.013G270700.1 . .
Gorai.009G112600.1 Gorai.003G148800.1 . . .
. . . . . . . .
. . .
Vv06g0831 . . . . . . .
. . . . . . Vv13g0116
. . . . . .
Vv06g0832 . . . . . Gorai.
003G149000.1 . . . . . . .
. . . . . . .
Vv06g0833 . . . . . .
Vv08g0452 Tc05_g001810 Gorai.006G184500.1 . .
Gorai.012G118200.1 . . . . .
. . .
Vv06g0834 Tc09_g015190 . . . . .
. . . . . . . .
. . . . . .
Vv06g0835 . . . . . . .
. . . . . . . .
. . . . .
Vv06g0836 . . . . . . .
. . . . . . . .
. . . . .
Vv06g0837 . . . . . . .
. . . . . . . .
. . . . .
Vv06g0838 . . . . . .
Vv08g0456 Tc05_g001770 Gorai.006G184600.1 Gorai.
002G266000.1 . Gorai.012G118500.1 . . .
. . . . .
Vv06g0839 . . . . . .
Vv08g0457 Tc05_g001750 . . . . .
Vv13g0123 Tc10_g001000 . . Gorai.
009G322800.1 . Gorai.012G079300.1
Vv06g0840 . . . . Gorai.009G112800.1
. . . . . . . .
. . . . . . .
Vv06g0841 Tc09_g014960 . . . . .
. . . . . . . .
. . . . . .
Vv06g0842 . . . . . . .
. . . . . . . .

. . . . .
Vv06g0843 . . . . . Gorai.
003G149500.1 . . . . . . .
. . . . . . .
Vv06g0844 . . . . . . .
. . . . . . Vv13g0125
Tc10_g000990 . Gorai.011G291500.1 .
Gorai.010G195100.1 .
Vv06g0845 . . . . Gorai.009G113000.1
. . . . . . . .
. . . . . . .
Vv06g0846 . . . . . . .
. . . . . . . .
. . . . .
Vv06g0847 . . . . . . .
. . . . . . . .
. . . . .
Vv06g0848 . . . . . . .
. . . . . . . .
. . . . .
Vv06g0849 . . . . . . .
. . . . . . . .
. . . . .
Vv06g0850 . . . . . . .
. . . . . . . .
. . . . .
Vv06g0851 . . . . . . .
. . . . . . . .
. . . . .
Vv06g0852 . . . . . . .
. . . . . . . .
. . . . .
Vv06g0853 . . . . . . .
. . . . . . . .
. . . . .
Vv06g0854 . . . . . . .
. . . . . . . .
. . . . .
Vv06g0855 . . . . . . .
. . . . . . . .
. . . . .
Vv06g0856 . . . . . . .
. . . . . . . .
. . . . .
Vv06g0857 . . . . . . .
. . . . . . . .
. . . . .
Vv06g0858 Tc09_g005360 Gorai.013G271200.1 . .
Gorai.009G113200.1 Gorai.003G149700.1 . .
. . . . . . . .

. . . .
Vv06g0859 . . . . Gorai.009G113300.1
. . . . . . . .
. . . . . . .
Vv06g0860 . . . . . Gorai.
003G149800.1 . . . . . . .
. . . . . . .
Vv06g0861 . . . . . Gorai.
003G150000.1 . . . . . . .
. . . . . . .
Vv06g0862 . Gorai.013G271300.1 . . .
. . . . . . . .
. . . . . . .
Vv06g0863 . . . . Gorai.009G113400.1
. . . . . . . .
. . . . . . .
Vv06g0864 . . . . . . .
. . . . . . . .
. . . . .
Vv06g0865 . . . . . . .
. . . . . . . .
. . . . .
Vv06g0866 . . . . Gorai.009G113500.1
. . . . . . . .
. . . . . . .
Vv06g0867 . . . . . . .
. . . . . . . .
. . . . .
Vv06g0868 . . . . . Gorai.
003G150300.1 . . . . . . .
Vv13g0137 . . . . . .
Vv06g0869 . . . . . . .
. . . . . . . .
. . . . .
Vv06g0870 . . . . . Gorai.
003G150400.1 . . . . . . .
. . . . . . .
Vv06g0871 . . . . . . .
. . . . . . . .
. . . . .
Vv06g0872 . . . . . . .
. . . . . . . .
. . . . .
Vv06g0873 . . . . . . .
. . . . . . . .
. . . . .
Vv06g0874 . . . . Gorai.009G113600.1
Gorai.003G150600.1 . . . . .
. . . . . . . .
.

Vv06g0875 . . . . . . .
. . . . . . . .
. . . . .
Vv06g0876 . . . . . .
Vv08g0487 Tc05_g001540 . . . . .
. . . . . . .
Vv06g0877 . . . Gorai.004G001100.1 .
. . . . . . . .
. . . . . . .
Vv06g0878 . . . . . .
Vv08g0489 Tc05_g001510 . . . Gorai.
012G119400.1 . . . . . . .
.
Vv06g0879 Tc09_g005290 . . . . .
. . . . . . . .
. . . . . .
Vv06g0880 . . . . . Gorai.
003G150800.1 . . . . . . .
. . . . . . .
Vv06g0881 Tc09_g005210 . . . .
Gorai.003G150900.1 . . . . . .
. . . . . . . .
Vv06g0882 . . . . Gorai.009G113700.1
. . . . . . . .
. . . . . . .
Vv06g0883 . . . . . .
Vv08g0506 Tc05_g000270 . Gorai.002G267300.1 .
. . . . . . . .
.
Vv06g0884 . . . . . . .
. . . . . . . .
. . . . .
Vv06g0885 . . . . . . .
. . . . . . . .
. . . . .
Vv06g0886 . . . . . . .
. . . . . . . .
. . . . .
Vv06g0887 . . . . . Gorai.
003G151000.1 . . . . . . .
. . . . . . .
Vv06g0888 . . . . . . .
. . . . . . . .
. . . . .
Vv06g0889 . . . . . . .
. . . . . . . .
. . . . .
Vv06g0890 . . . . . . .
. . . . . . . .
. . . . .

Vv06g0891 . . . . . Gorai.
003G151100.1 Vv08g0514 . . . . .
. . . . . . . .
Vv06g0892 . . . . . . .
. . . . . . . .
. . . . .
Vv06g0893 . . . . . Gorai.
003G151200.1 . . . . . . .
. . . . . . .
Vv06g0894 . . . . . .
Vv08g0524 . . . . . . .
. . . . . .
Vv06g0895 . . . . . Gorai.
003G151300.1 . . . . . . .
. . . . . . .
Vv06g0896 . . . . . . .
. . . . . . . .
. . . . .
Vv06g0897 . . . . Gorai.009G113800.1
. . . . . . . .
. . . . . . .
Vv06g0898 Tc09_g024630 . . . . .
. . . . . . . .
. . . . . .
Vv06g0899 . . . . . .
Vv08g0528 . . . . . .
Vv13g0164 . . . . . .
Vv06g0900 . . . . . Gorai.
003G151400.1 . . . . . . .
. . . . . . .
Vv06g0901 . . . . . . .
. . . . . . . .
. . . . .
Vv06g0902 . . . . . Gorai.
003G151500.1 . . . . . . .
. . . . . . .
Vv06g0903 . . . . . . .
. . . . . . . .
. . . . .
Vv06g0904 . . . . . . .
. . . . . . . .
. . . . .
Vv06g0905 . . . . . Gorai.
003G151600.1 . . . . . . .
. . . . . . .
Vv06g0906 . . . . . . .
. . . . . . . .
. . . . .
Vv06g0907 . . . . . . .
. . . . . . . .

. . . . .
Vv06g0908 Tc09_g004920 . . . . .
. . . . . . .
Vv13g0170 . . . . . .
Vv06g0909 Tc09_g024320 . . . . .
. . . . . . . .
. . . . . .
Vv06g0910 . . . . . . .
. . . . . . . .
. . . . .
Vv06g0911 . . . . . . .
. . . . . . . .
. . . . .
Vv06g0912 . . . . . . .
. . . . . . . .
. . . . .
Vv06g0913 . Gorai.013G272400.1 . . .
. . . . . . . .
. . . . . . .
Vv06g0914 . . . . . . .
. . . . . . . .
. . . . .
Vv06g0915 . . . . . Gorai.
003G152200.1 . . . . . . .
. . . . . . .
Vv06g0916 Tc09_g016540 . . . . .
. . . . . . . .
. . . . . .
Vv06g0917 . . . . . . .
. . . . . . . .
. . . . .
Vv06g0918 . . . . . Gorai.
003G152400.1 . . . . . . .
. . . . . . .
Vv06g0919 . . . . . . .
. . . . . . . .
. . . . .
Vv06g0920 . . . . . .
Vv08g0548 . . . . . . .
. . . . . .
Vv06g0921 . Gorai.013G272200.1 . . .
. . . . . . . .
. . . . . . .
Vv06g0922 Tc09_g016780 . . . . .
. . . . . . . .
. . . . . .
Vv06g0923 . . . . . .
Vv08g0552 . . . . . .
Vv13g0187 . . . . . .
Vv06g0924 . . . . . . .

. . . . . . Vv13g0189
. Gorai.013G007300.1 . . . .
Vv06g0925 . . . . . . .
. . . . . . Vv13g0217
Tc10_g016420 . Gorai.011G207900.1 . .
.
Vv06g0926 . . . Gorai.004G000900.1 .
. . . . . . . .
. . . . . . .
Vv06g0927 . Gorai.013G272000.1 . . .
. Vv08g0557 Tc05_g024960 . Gorai.
007G266900.1 . . . . . . .
. . .
Vv06g0928 . . . . . Gorai.
003G152500.1 . . . . . . .
. . . . . . .
Vv06g0929 . . . . . . .
. . . . . . . .
. . . . .
Vv06g0930 . . . . . . .
. . . . . . . .
. . . . .
Vv06g0931 . . . . . . .
. . . . . . . .
. . . . .
Vv06g0932 . . . . . . .
. . . . . . . .
. . . . .
Vv06g0933 . . . . . . .
. . . . . . . .
. . . . .
Vv06g0934 . . . . . . .
. . . . . . . .
. . . . .
Vv06g0935 . . . . . . .
. . . . . . . .
. . . . .
Vv06g0936 Tc09_g032300 Gorai.013G271800.1 . .
. . . . . . . .
. . . . . . . .
Vv06g0937 . . . . . Gorai.
003G152600.1 . . . . . . .
. . . . . . .
Vv06g0938 . . . . . .
Vv08g0567 . . . . . . .
. . . . . .
Vv06g0939 . . . . . Gorai.
003G152700.1 . . . . . . .
. . . . . . .
Vv06g0940 . . . . . . .

. . . . . . . .
. . . . .
Vv06g0941 Tc09_g024240 . . . . .
. . . . . . . .
. . . . . .
Vv06g0942 . . . . . . .
. . . . . . . .
. . . . .
Vv06g0943 . . . . . . .
. . . . . . Vv13g0242
Tc10_g016660 Gorai.013G008700.1 . . .
.
Vv06g0944 . . . . . . .
. . . . . . . .
. . . . .
Vv06g0945 . . . . . . .
. . . . . . . .
. . . . .
Vv06g0946 . . . . . . .
. . . . . . . .
. . . . .
Vv06g0947 Tc09_g024080 . . . . .
Vv08g0591 Tc05_g023640 . . . .
. . . . . . . .
Vv06g0948 Tc09_g004910 . . . . .
. . . . . . . .
. . . . . .
Vv06g0949 . . . . . . .
. . . . . . . .
. . . . .
Vv06g0950 Tc09_g024050 . . . . .
. . . . . . . .
. . . . . .
Vv06g0951 . . . . . . .
. . . . . . . .
. . . . .
Vv06g0952 . . . . . . .
. . . . . . . .
. . . . .
Vv06g0953 . Gorai.013G271500.1 . . .
. . . . . . . .
. . . . . . .
Vv06g0954 . . . . . . .
. . . . . . . .
. . . . .
Vv06g0955 . . . . . . .
. . . . . . . .
. . . . .
Vv06g0956 . . . . . . .
. . . . . . . .

. . . . .
Vv06g0957 . . . . . . .
. . . . . . . .
. . . . .
Vv06g0958 . . . . . . .
. . . . . . . .
. . . . .
Vv06g0959 . Gorai.013G271400.1 . . .
. . . . . . . .
. . . . . . .
Vv06g0960 . . . . . . .
. . . . . . . .
. . . . .
Vv06g0961 . . . . . . .
. . . . . . . .
. . . . .
Vv06g0962 . . . . . . .
. . . . . . . .
. . . . .
Vv06g0963 . Gorai.013G268400.1 . . .
. . . . . . . .
. . . . . . .
Vv06g0964 . . . . . Gorai.
003G153200.1 . . . . . . .
. . . . . . .
Vv06g0965 . . . . . . .
. . . . . . . .
. . . . .
Vv06g0966 Tc09_g032100 . . . Gorai.
009G101200.1 . . . . . . .
. . . . . . . .
Vv06g0967 . . . . . . .
. . . . . . . .
. . . . .
Vv06g0968 . . . . . . .
. . . . . . . .
. . . . .
Vv06g0969 . . . . . . .
. . . . . . . .
. . . . .
Vv06g0970 . . . . . . .
. . . . . . . .
. . . . .
Vv06g0971 . . . . . . .
. . . . . . . .
. . . . .
Vv06g0972 . . . . . . .
. . . . . . . .
. . . . .
Vv06g0973 . . . Gorai.004G000800.1 .

. . . . . . . .
. . . . . . .
Vv06g0974 . . . Gorai.004G000700.1 .
. . . . . . . .
. . . . . . .
Vv06g0975 . . . . . . .
. . . . . . . .
. . . . .
Vv06g0976 . . . . . . .
. . . . . . . .
. . . . .
Vv06g0977 . . . . . Gorai.
003G156000.1 . . . . . . .
. . . . . . .
Vv06g0978 . . . . . . .
. . . . . . . .
. . . . .
Vv06g0979 . . . . . . .
. . . . . . . .
. . . . .
Vv06g0980 . . . . . . .
. . . . . . . .
. . . . .
Vv06g0981 . . . . . . .
. . . . . . . .
. . . . .
Vv06g0982 . . . . . . .
. . . . . . . .
. . . . .
Vv06g0983 . . . . . . .
. . . . . . . .
. . . . .
Vv06g0984 . . . . . . .
. . . . . . . .
. . . . .
Vv06g0985 . . . . . . .
. . . . . . . .
. . . . .
Vv06g0986 . . . . . . .
. . . . . . . .
. . . . .
Vv06g0987 . . . . . . .
. . . . . . . .
. . . . .
Vv06g0988 . . . . . . .
. . . . . . . .
. . . . .
Vv06g0989 . . . . . . .
. . . . . . . .
. . . . .

Vv06g0990 . . . . . . .
. . . . . . . .
. . . . .
Vv06g0991 . . . . . . .
. . . . . . . .
. . . . .
Vv06g0992 . . . . . . .
. . . . . . . .
. . . . .
Vv06g0993 . . . . . Gorai.
006G116500.1 . . . . . . .
. . . . . . .
Vv06g0994 . . . . . . .
. . . . . . . .
. . . . .
Vv06g0995 . . . . . . .
. . . . . . . .
. . . . .
Vv06g0996 Tc09_g031880 . . . Gorai.
009G100000.1 Gorai.006G116400.1 . . . .
. . . . . . . .
. .
Vv06g0997 . . . . . . .
. . . . . . . .
. . . . .
Vv06g0998 . . . . . . .
. . . . . . . .
. . . . .
Vv06g0999 . . . . . . .
. . . . . . . .
. . . . .
Vv06g1000 . . . . . . .
. . . . . . . .
. . . . .
Vv06g1001 . . . . . . .
. . . . . . . .
. . . . .
Vv06g1002 . . . . . . .
. . . . . . . .
. . . . .
Vv06g1003 . . . . . . .
. . . . . . . .
. . . . .
Vv06g1004 . . . . . . .
. . . . . . . .
. . . . .
Vv06g1005 . . . . . . .
. . . . . . . .
. . . . .
Vv06g1006 . . . . . . .

. . . . . . . .
. . . . .
Vv06g1007 . . . . . . .
. . . . . . . .
. . . . .
Vv06g1008 Tc09_g031880 . . . Gorai.
009G100000.1 Gorai.006G116300.1 . . . .
. . . . . . . .
. .
Vv06g1009 . . . . . Gorai.
006G115600.1 . . . . . . .
. . . . . . .
Vv06g1010 . . . . . . .
. . . . . . . .
. . . . .
Vv06g1011 . . . . . Gorai.
006G115500.1 . . . . . . .
. . . . . . .
Vv06g1012 . . . . . . .
. . . . . . . .
. . . . .
Vv06g1013 . . . . . Gorai.
006G115400.1 . . . . . . .
. . . . . . .
Vv06g1014 . . . . . . .
. . . . . . . .
. . . . .
Vv06g1015 . . . . . Gorai.
006G149200.1 . . . . . . .
. . . . . . .
Vv06g1016 . . . . . . .
. . . . . . . .
. . . . .
Vv06g1017 Tc09_g029980 . . . .
Gorai.006G113100.1 . . . . . .
. . . . . . . .
Vv06g1018 . . . . . . .
. . . . . . . .
. . . . .
Vv06g1019 . . . . . . .
. . . . . . . .
. . . . .
Vv06g1020 Tc09_g030110 . . . Gorai.
009G092700.1 . . . . . . .
. . . . . . . .
Vv06g1021 Tc09_g024620 . . . . .
. . . . . . . .
. . . . . .
Vv06g1022 . . . . . . .
. . . . . . . .

. . . . .
Vv06g1023 . . . . . . .
. . . . . . . .
. . . . .
Vv06g1024 . . . . . . .
. . . . . . . .
. . . . .
Vv06g1025 . . . . . . .
. . . . . . . .
. . . . .
Vv06g1026 . . . . . . .
. . . . . . . .
. . . . .
Vv06g1027 Tc09_g024930 . . . . .
. . . . . . . .
. . . . . .
Vv06g1028 . . . . . . .
. . . . . . . .
. . . . .
Vv06g1029 . . . . . . .
. . . . . . . .
. . . . .
Vv06g1030 . . Gorai.001G125100.1 . .
. . . . . . . .
. . . . . . .
Vv06g1031 . . . . . . .
. . . . . . . .
. . . . .
Vv06g1032 Tc09_g024980 . . . .
Gorai.006G124800.1 . . . . . .
. . . . . . . .
Vv06g1033 . . . . . . .
. . . . . . . .
. . . . .
Vv06g1034 . . . . . . .
. . . . . . . .
. . . . .
Vv06g1035 . . . . . . .
. . . . . . . .
. . . . .
Vv06g1036 . . . . . . .
. . . . . . . .
. . . . .
Vv06g1037 . . . . . . .
. . . . . . . .
. . . . .
Vv06g1038 . . . . . . .
. . . . . . . .
. . . . .
Vv06g1039 . . . . . . .

. . . . . . . .
. . . . .
Vv06g1040 . . . . . . .
. . . . . . . .
. . . . .
Vv06g1041 Tc09_g032200 . . . Gorai.
009G101400.1 . . . . . . .
. . . . . . . .
Vv06g1042 . . . . . . .
. . . . . . . .
. . . . .
Vv06g1043 . . . . Gorai.009G080800.1
. . . . . . . .
. . . . . . .
Vv06g1044 . . . . . . .
. . . . . . . .
. . . . .
Vv06g1045 Tc09_g030320 . . . . . .
Vv08g0211 Tc05_g024050 . . . . .
. . . . . . . .
Vv06g1046 Tc09_g030330 . . . . . .
Vv08g0210 Tc05_g024040 . . . . .
. . . . . . . .
Vv06g1047 . . . . . . .
. . . . . . . .
. . . . .
Vv06g1048 . . . . . . .
. . . . . . . .
. . . . .
Vv06g1049 . . . . . . .
. . . . . . . .
. . . . .
Vv06g1050 . . . . . . .
. . . . . . . .
. . . . .
Vv06g1051 . . . . Gorai.009G081100.1
. . . . . . . .
. . . . . . .
Vv06g1052 . . . . . . .
. . . . . . . .
. . . . .
Vv06g1053 . . . . . . .
. . . . . . . .
. . . . .
Vv06g1054 Tc09_g027360 . . . . . .
. . . . . . . .
. . . . . .
Vv06g1055 . . . . . . .
. . . . . . . .
. . . . .

Vv06g1056 . . . . . . .
. . . . . . . .
. . . . .
Vv06g1057 Tc09_g027210 . Gorai.001G126900.1 .
. Gorai.006G117500.1 . . . .
. . . . . . . .
. .
Vv06g1058 . . . . . . .
. . . . . . . .
. . . . .
Vv06g1059 . . . . . . .
. . . . . . . .
. . . . .
Vv06g1060 . . . . . . .
. . . . . . . .
. . . . .
Vv06g1061 . . . . . .
Vv08g0177 . . . . . . .
. . . . . .
Vv06g1062 . . . . . . .
. . . . . . . .
. . . . .
Vv06g1063 . . . . . . .
. . . . . . . .
. . . . .
Vv06g1064 Tc09_g027180 . Gorai.001G127000.1 .
. Gorai.006G117800.1 . . . .
. . . . . . . .
. .
Vv06g1065 . . . . . Gorai.
006G147900.1 . . . . . . .
. . . . . . .
Vv06g1066 . . . . . . .
. . . . . . . .
. . . . .
Vv06g1067 Tc09_g027170 . . . . .
. . . . . . . .
. . . . . .
Vv06g1068 . . . . . . .
. . . . . . . .
. . . . .
Vv06g1069 . . . . . . .
. . . . . . . .
. . . . .
Vv06g1070 Tc09_g027160 . Gorai.001G127100.1 .
Gorai.009G081400.1 . . . . .
. . . . . . . .
. .
Vv06g1071 . . . . . . .
. . . . . . . .

```
                .       .       .       .       .
Vv06g1072               .       .       .       .       .       .       .
                .       .       .       .       .       .       .       .
                .       .       .       .       .
Vv06g1073               .       .       .       .       .       .       .
                .       .       .       .       .       .       .       .
                .       .       .       .       .
Vv06g1074               .       .       .       .       .       Gorai.
006G146700.1    Vv08g0167       Tc05_g005040            .       .       .
                .       .       .       .       .       .       .       .
                .
Vv06g1075               .       .       .       .       .       .       .
                .       .       .       .       .       .       .       .
                .       .       .       .       .
Vv06g1076       Tc09_g015990    .       .       .       .       .
                .       .       .       .       .       .       .       .
                .       .       .       .       .       .
Vv06g1077               .       .       .       .       .       .       .
                .       .       .       .       .       .       .       .
                .       .       .       .       .
Vv06g1078               .       .       .       .       .       .       .
                .       .       .       .       .       .       .       .
                .       .       .       .       .
Vv06g1079               .       .       .       .       Gorai.009G081800.1
                .       .       .       .       .       .       .       .
                .       .       .       .       .       .       .
Vv06g1080               .       .       .       .       .       .       .
                .       .       .       .       .       .       .       .
                .       .       .       .       .
Vv06g1081               .       .       .       .       .       .
Vv08g0159               .       .       .       .       .       .       .
                .       .       .       .       .       .
Vv06g1082       Tc09_g015670    .       .       .       .       .
                .       .       .       .       .       .       .       .
                .       .       .       .       .       .
Vv06g1083               .       .       .       .       .       .       .
                .       .       .       .       .       .       .       .
                .       .       .       .       .
Vv06g1084               .       .       .       .       .       .       .
                .       .       .       .       .       .       .       .
                .       .       .       .       .
Vv06g1085       Tc09_g027130    .       Gorai.001G127300.1      .
                .       .       .       .       .       .       .       .
                .       .       .       .       .       .       .       .
Vv06g1086       Tc09_g027120    .       .       .       .       .
                .       .       .       .       .       .       .       .
                .       .       .       .       .       .
Vv06g1087       Tc09_g026980    .       Gorai.001G128200.1      .
                .       .       Vv08g0147       .       .       .       .
                .       .       .       .       .       .       .       .
```

.
Vv06g1088 . . . . . . .
. . . . . . . .
. . . . .
Vv06g1089 . . . . . .
Vv08g0137 . . . . . . .
. . . . . .
Vv06g1090 . . . . . . .
. . . . . . . .
. . . . .
Vv06g1091 . . . . . . .
. . . . . . . .
. . . . .
Vv06g1092 Tc09_g026940 . . . . .
. . . . . . . .
. . . . . .
Vv06g1093 . . . . . . .
. . . . . . . .
. . . . .
Vv06g1094 . . . . . . .
. . . . . . . .
. . . . .
Vv06g1095 . . . . . . .
. . . . . . . .
. . . . .
Vv06g1096 . . . . . Gorai.
006G146500.1 . . . . . . .
. . . . . . .
Vv06g1097 . . . . . . .
. . . . . . . .
. . . . .
Vv06g1098 . . . . . . .
. . . . . . . .
. . . . .
Vv06g1099 . . . . Gorai.009G105900.1
Gorai.006G144700.1 . . . . .
. . . . . . . .
.
Vv06g1100 . . . . . . .
. . . . . . . .
. . . . .
Vv06g1101 . . . . . . .
. . . . . . . .
. . . . .
Vv06g1102 . . . . . . .
. . . . . . . .
. . . . .
Vv06g1103 . . . . . . .
. . . . . . . .
. . . . .

Vv06g1104 . . . . Gorai.009G105600.1
. . . . . . . .
. . . . . . .
Vv06g1105 . . . . . . .
. . . . . . . .
. . . . .
Vv06g1106 . . . . Gorai.009G105500.1
Gorai.003G140700.1 . . . . .
. . . . . . . .
.
Vv06g1107 . . . . . . .
. . . . . . . .
. . . . .
Vv06g1108 Tc09_g030940 . . . . .
. . . . . . . .
. . . . . .
Vv06g1109 . . . . . . .
. . . . . . . .
. . . . .
Vv06g1110 . . . . . . .
. . . . . . . .
. . . . .
Vv06g1111 . . . . . . .
. . . . . . . .
. . . . .
Vv06g1112 . . . . . . .
. . . . . . . .
. . . . .
Vv06g1113 . . . . . . .
. . . . . . . .
. . . . .
Vv06g1114 . . . . . . .
. . . . . . . .
. . . . .
Vv06g1115 . . . . . . .
. . . . . . . .
. . . . .
Vv06g1116 . . . . . . .
. . . . . . . .
. . . . .
Vv06g1117 . . . . . . .
. . . . . . . .
. . . . .
Vv06g1118 . . . . . . .
. . . . . . . .
. . . . .
Vv06g1119 . . . . . . .
. . . . . . . .
. . . . .
Vv06g1120 . . . . . . .

. . . . . . . .
. . . . .
Vv06g1121 . . . . Gorai.009G105400.1
Gorai.003G140600.1 . . . . .
. . . . . . . .
.
Vv06g1122 . . . . . . .
. . . . . . . .
. . . . .
Vv06g1123 . . . . . . .
. . . . . . . .
. . . . .
Vv06g1124 Tc09_g026760 . . . . .
. . . . . . . .
. . . . . .
Vv06g1125 . . . . . . .
. . . . . . . .
. . . . .
Vv06g1126 . . . . . . .
. . . . . . . .
. . . . .
Vv06g1127 . . . . . . .
. . . . . . . .
. . . . .
Vv06g1128 . . . . . . .
. . . . . . . .
. . . . .
Vv06g1129 Tc09_g031140 . Gorai.001G129000.1 .
. . . . . . . .
. . . . . . . .
Vv06g1130 . . . . . Gorai.
003G140000.1 . . . . . . .
. . . . . . .
Vv06g1131 . . . . . . .
. . . . . . . .
. . . . .
Vv06g1132 . . . . . . .
. . . . . . . .
. . . . .
Vv06g1133 . . . . . . .
. . . . . . . .
. . . . .
Vv06g1134 . . . . . . .
. . . . . . . .
. . . . .
Vv06g1135 . . . . . . .
. . . . . . . .
. . . . .
Vv06g1136 . . . . . . .
. . . . . . . .

. . . . .
Vv06g1137 . . . . . . .
. . . . . . . .
. . . . .
Vv06g1138 . . . . . . .
. . . . . . . .
. . . . .
Vv06g1139 . . . . . . .
. . . . . . . .
. . . . .
Vv06g1140 . . . . . . .
. . . . . . . .
. . . . .
Vv06g1141 . . . . . . .
. . . . . . . .
. . . . .
Vv06g1142 . . . . . . .
. . . . . . . .
. . . . .
Vv06g1143 . . . . . . .
. . . . . . . .
. . . . .
Vv06g1144 . . . . . . .
. . . . . . . .
. . . . .
Vv06g1145 . . . . . . .
. . . . . . . .
. . . . .
Vv06g1146 . . . . . . .
. . . . . . . .
. . . . .
Vv06g1147 . . . . . . .
. . . . . . . .
. . . . .
Vv06g1148 . . . . . . .
. . . . . . . .
. . . . .
Vv06g1149 . . . . . Gorai.
003G137000.1 . . . . . . .
. . . . . . .
Vv06g1150 . . . . . . .
. . . . . . . .
. . . . .
Vv06g1151 . . . . . . .
. . . . . . . .
. . . . .
Vv06g1152 . Gorai.013G266200.1 . .
Gorai.009G104900.1 . . . . . .
. . . . . . . .
.

Vv06g1153 . . . . Gorai.009G101900.1
. . . . . . . .
. . . . . . .
Vv06g1154 . . . . . . .
. . . . . . . .
. . . . .
Vv06g1155 . . . . . . .
. . . . . . . .
. . . . .
Vv06g1156 . . . . . . .
. . . . . . . .
. . . . .
Vv06g1157 Tc09_g025980 . . . . .
. . . . . . . .
. . . . . .
Vv06g1158 Tc09_g035300 . . . . .
. . . . . . . .
. . . . . .
Vv06g1159 . . . . . . .
. . . . . . . .
. . . . .
Vv06g1160 . . . . . . .
. . . . . . . .
. . . . .
Vv06g1161 . . . . Gorai.009G079700.1
. . . . . . . .
. . . . . . .
Vv06g1162 . . . . . . .
. . . . . . . .
. . . . .
Vv06g1163 Tc09_g035070 . . . . .
. . . . . . . .
. . . . . .
Vv06g1164 . . . . . Gorai.
006G119100.1 . . . . . . .
. . . . . . .
Vv06g1165 . . . . . . .
. . . . . . . .
. . . . .
Vv06g1166 . . . . . . .
. . . . . . . .
. . . . .
Vv06g1167 . . . . . Gorai.
006G119200.1 . . . . . . .
. . . . . . .
Vv06g1168 Tc09_g021760 . . . . .
. . . . . . . .
. . . . . .
Vv06g1169 . . . . Gorai.009G079600.1
. . . . . . . .

. . . . . . .
Vv06g1170 Tc09_g034700 . . . . .
. . . . . . . .
. . . . . .
Vv06g1171 . . . . . . .
. . . . . . . .
. . . . .
Vv06g1172 . Gorai.013G266400.1 . . .
. . . . . . . .
. . . . . . .
Vv06g1173 Tc09_g016540 . . . . .
. . . . . . . .
. . . . . .
Vv06g1174 . . . . . . .
. . . . . . . .
. . . . .
Vv06g1175 Tc09_g025560 . . . . .
. . . . . . . .
. . . . . .
Vv06g1176 . . . . . . .
. . . . . . . .
. . . . .
Vv06g1177 . . . . . . .
. . . . . . . .
. . . . .
Vv06g1178 Tc09_g025520 . . . .
Gorai.006G121600.1 . . . . . .
. . . . . . . .
Vv06g1179 . . . . . . .
. . . . . . . .
. . . . .
Vv06g1180 . . . . . . .
. . . . . . . .
. . . . .
Vv06g1181 . . . . . . .
. . . . . . . .
. . . . .
Vv06g1182 . . . . . . .
. . . . . . . .
. . . . .
Vv06g1183 . . . . . . .
. . . . . . . .
. . . . .
Vv06g1184 . . . . . . .
. . . . . . . .
. . . . .
Vv06g1185 Tc09_g025510 . Gorai.001G132100.1 .
. Gorai.006G121700.1 . . . .
. . . . . . . .
. .

Vv06g1186 . Gorai.013G266500.1 . . .
. . . . . . . .
. . . . . . .
Vv06g1187 . . . . . . .
. . . . . . . .
. . . . .
Vv06g1188 . . . . . . .
. . . . . . . .
. . . . .
Vv06g1189 Tc09_g024980 Gorai.013G256400.1 . .
Gorai.009G078000.1 . . . . .
. . . . . . . .
. .
Vv06g1190 . . . . . . .
. . . . . . . .
. . . . .
Vv06g1191 . Gorai.013G268600.1 . . .
. . . . . . . .
. . . . . . .
Vv06g1192 Tc09_g016030 . . . . .
. . . . . . . .
. . . . . .
Vv06g1193 . . . . . . .
. . . . . . . .
. . . . .
Vv06g1194 Tc09_g034550 . . . . .
. . . . . . . .
. . . . . .
Vv06g1195 . . . . . . .
. . . . . . . .
. . . . .
Vv06g1196 Tc09_g034530 . . . . .
. . . . . . . .
. . . . . .
Vv06g1197 Tc09_g034520 . . . . .
. . . . . . . .
. . . . . .
Vv06g1198 . . . . . . .
. . . . . . . .
. . . . .
Vv06g1199 . . . . . . .
. . . . . . . .
. . . . .
Vv06g1200 . . Gorai.001G132500.1 . .
. . . . . . . .
. . . . . . .
Vv06g1201 . . . . . . .
. . . . . . . .
. . . . .
Vv06g1202 Tc09_g024910 . . . Gorai.

```
009G077000.1     .       .       .       .       .       .       .
        .        .       .       .       .       .       .       .
Vv06g1203        .       Gorai.013G254900.1      .       .       .
        Gorai.006G122700.1       .       .       .       .       .
        .        .       .       .       .       .       .       .
        .
Vv06g1204        Tc09_g021220    .       .       .       .       .
        .        .       .       .       .       .       .       .
        .        .       .       .       .       .
Vv06g1205        Tc09_g024820    .       Gorai.001G132800.1      .
        .        .       .       .       .       .       .       .
        .        .       .       .       .       .       .       .
Vv06g1206        .       .       .       .       .       .       .
        .        .       .       .       .       .       .       .
        .        .       .       .       .
Vv06g1207        Tc09_g024810    .       .       .       .
Gorai.006G123200.1       .       .       .       .       .       .
        .        .       .       .       .       .       .       .
Vv06g1208        .       .       .       .       .       .       .
        .        .       .       .       .       .       .       .
        .        .       .       .       .
Vv06g1209        .       .       .       .       .       .       .
        .        .       .       .       .       .       .       .
        .        .       .       .       .
Vv06g1210        .       .       .       .       .       .       .
        .        .       .       .       .       .       .       .
        .        .       .       .       .
Vv06g1211        Tc09_g024800    .       Gorai.001G132900.1      .
        .        .       .       .       .       .       .       .
        .        .       .       .       .       .       .       .
Vv06g1212        .       .       .       .       .       .       .
        .        .       .       .       .       .       .       .
        .        .       .       .       .
Vv06g1213        .       .       .       .       .       .       .
        .        .       .       .       .       .       .       .
        .        .       .       .       .
Vv06g1214        .       .       .       .       .       .       .
        .        .       .       .       .       .       .       .
        .        .       .       .       .
Vv06g1215        .       .       .       .       .       .       .
        .        .       .       .       .       .       .       .
        .        .       .       .       .
Vv06g1216        .       .       .       .       .       .       .
        .        .       .       .       .       .       .       .
        .        .       .       .       .
Vv06g1217        Tc09_g024760    .       .       .       .       .
        .        .       .       .       .       .       .       .
        .        .       .       .       .       .
Vv06g1218        .       .       .       .       .       .       .
        .        .       .       .       .       .       .       .
```

. . . . .
Vv06g1219 . . . . . . .
. . . . . . . .
. . . . .
Vv06g1220 Tc09_g007330 . . . . .
. . . . . . . .
. . . . . .
Vv06g1221 . . . . . . .
. . . . . . . .
. . . . .
Vv06g1222 . Gorai.013G254300.1 . . .
. . . . . . . .
. . . . . . .
Vv06g1223 . . . . . . .
. . . . . . . .
. . . . .
Vv06g1224 . . . . . . .
. . . . . . . .
. . . . .
Vv06g1225 Tc09_g024710 . . . Gorai.
009G075700.1 . . . . . . .
. . . . . . . .
Vv06g1226 . . . . . . .
. . . . . . . .
. . . . .
Vv06g1227 . . . . . . .
. . . . . . . .
. . . . .
Vv06g1228 Tc09_g024700 . Gorai.001G133000.1 .
. . . . . . . .
. . . . . . . .
Vv06g1229 . . . . . . .
. . . . . . . .
. . . . .
Vv06g1230 . . . . . . .
. . . . . . . .
. . . . .
Vv06g1231 . Gorai.013G254200.1 . .
Gorai.009G072700.1 . . . . . .
. . . . . . . .
.
Vv06g1232 Tc09_g024690 . . . .
Gorai.006G123700.1 . . . . . .
. . . . . . . .
Vv06g1233 Tc09_g007250 . . . . .
. . . . . . . .
. . . . . .
Vv06g1234 . . . . . . .
. . . . . . . .
. . . . .

Vv06g1235 Tc09_g024630 . . . . .
. . . . . . . .
. . . . . .
Vv06g1236 . . . . . . .
. . . . . . . .
. . . . .
Vv06g1237 Tc09_g034350 . . . . .
. . . . . . . .
. . . . . .
Vv06g1238 . . . . . . .
. . . . . . . .
. . . . .
Vv06g1239 . . . . . . .
. . . . . . . .
. . . . .
Vv06g1240 Tc09_g006890 . . . . .
. . . . . . . .
. . . . . .
Vv06g1241 . . . . . . .
. . . . . . . .
. . . . .
Vv06g1242 . . . . . . .
. . . . . . . .
. . . . .
Vv06g1243 . . . . . . .
. . . . . . . .
. . . . .
Vv06g1244 Tc09_g024260 . . . . .
. . . . . . . .
. . . . . .
Vv06g1245 . . . . . . .
. . . . . . . .
. . . . .
Vv06g1246 . . . . . . .
. . . . . . . .
. . . . .
Vv06g1247 . . . . . . .
. . . . . . . .
. . . . .
Vv06g1248 . . . . . . .
. . . . . . . .
. . . . .
Vv06g1249 . . . . . . .
. . . . . . . .
. . . . .
Vv06g1250 . . . . . . .
. . . . . . . .
. . . . .
Vv06g1251 . . . . . . .
. . . . . . . .

| | | | | | | | |
|---|---|---|---|---|---|---|---|
| Vv06g1252 | . | . | . | . | . | . | . |
| Vv06g1253 | . | . | . | . | . | . | . |
| Vv06g1254 | . | . | . | . | . | . | . |
| Vv06g1255 | Tc09_g006510 | | . | . | . | . | . |
| Vv06g1256 | . | . | . | . | . | . | . |
| Vv06g1257 | Tc09_g006460 | | . | . | . | . | . |
| Vv06g1258 | . | . | . | . | . | . | . |
| Vv06g1259 | . | . | . | . | . | . | . |
| Vv06g1260 | Tc09_g006360 | | . | . | . | . | . |
| Vv06g1261 | . | . | . | . | . | . | . |
| Vv06g1262 | Tc09_g006190 | | . | . | . | . | . |
| Vv06g1263 | . | . | . | . | . | . | . |
| Vv06g1264 | . | . | . | . | . | . | . |
| Vv06g1265 | Tc09_g006170 | | . | . | . | . | . |
| Vv06g1266 | . | . | . | . | . | . | . |
| Vv06g1267 | . | . | . | . | . | . | . |
| Vv06g1268 | . | . | . | . | . | . | . |

. . . . . . . .
. . . . .
Vv06g1269 . . Gorai.001G135400.1 . .
. . . . . . . .
. . . . . . .
Vv06g1270 . . . . . . .
. . . . . . . .
. . . . .
Vv06g1271 . . . . . . .
. . . . . . . .
. . . . .
Vv06g1272 . . . . . . .
. . . . . . . .
. . . . .
Vv06g1273 . . . . . . .
. . . . . . . .
. . . . .
Vv06g1274 . . . . . . .
. . . . . . . .
. . . . .
Vv06g1275 . . . . . . .
. . . . . . . .
. . . . .
Vv06g1276 . . . . . . .
. . . . . . . .
. . . . .
Vv06g1277 . . . . . . .
. . . . . . . .
. . . . .
Vv06g1278 . . . . . Gorai.
006G125000.1 . . . . . . .
. . . . . . .
Vv06g1279 . . . . . . .
. . . . . . . .
. . . . .
Vv06g1280 . . . . . . .
. . . . . . . .
. . . . .
Vv06g1281 . Gorai.013G253200.1 . . .
. . . . . . .
. . . . . . .
Vv06g1282 . . . . . . .
. . . . . . . .
. . . . .
Vv06g1283 . . . . . . .
. . . . . . . .
. . . . .
Vv06g1284 . . . . . . .
. . . . . . . .
. . . . .

Vv06g1285 . . . . . . .
. . . . . . . .
. . . . .
Vv06g1286 . . . . . . .
. . . . . . . .
. . . . .
Vv06g1287 . . . . . . .
. . . . . . . .
. . . . .
Vv06g1288 . . . . . . .
. . . . . . . .
. . . . .
Vv06g1289 . . . . . . .
. . . . . . . .
. . . . .
Vv06g1290 . . . . . . .
. . . . . . . .
. . . . .
Vv06g1291 . . . . . . .
. . . . . . . .
. . . . .
Vv06g1292 . . . . . . .
. . . . . . . .
. . . . .
Vv06g1293 . . . . . . .
. . . . . . . .
. . . . .
Vv06g1294 . . . . . . .
. . . . . . . .
. . . . .
Vv06g1295 . . . . . . .
. . . . . . . .
. . . . .
Vv06g1296 . . . . . . .
. . . . . . . .
. . . . .
Vv06g1297 . . . . . . .
. . . . . . . .
. . . . .
Vv06g1298 . . . . . . .
. . . . . . . .
. . . . .
Vv06g1299 . . . . . . .
. . . . . . . .
. . . . .
Vv06g1300 . . . . . . .
. . . . . . . .
. . . . .
Vv06g1301 . . . . . . .
. . . . . . . .

. . . . .
Vv06g1302 . . . . . . .
. . . . . . . .
. . . . .
Vv06g1303 . . . . . . .
. . . . . . . .
. . . . .
Vv06g1304 . . . . . . .
. . . . . . . .
. . . . .
Vv06g1305 . . . . . . .
. . . . . . . .
. . . . .
Vv06g1306 . . . . . . .
. . . . . . . .
. . . . .
Vv06g1307 . . . . . . .
. . . . . . . .
. . . . .
Vv06g1308 . . . . . . .
. . . . . . . .
. . . . .
Vv06g1309 . . . . . . .
. . . . . . . .
. . . . .
Vv06g1310 . . . . . . .
. . . . . . . .
. . . . .
Vv06g1311 . . . . . . .
. . . . . . . .
. . . . .
Vv06g1312 . . . . . . .
. . . . . . . .
. . . . .
Vv06g1313 . . . . . . .
. . . . . . . .
. . . . .
Vv06g1314 . . . . . . .
. . . . . . . .
. . . . .
Vv06g1315 . . . . . . .
. . . . . . . .
. . . . .
Vv06g1316 . . . . . . .
. . . . . . . .
. . . . .
Vv06g1317 . . . . . . .
. . . . . . . .
. . . . .
Vv06g1318 . Gorai.013G252300.1 . . .

Gorai.006G125500.1 . . . . .
. . . . . . . .
.
Vv06g1319 . Gorai.013G249300.1 . . .
. . . . . . . .
. . . . . . .
Vv06g1320 . . . . . . .
. . . . . . . .
. . . . .
Vv06g1321 . . . . . . .
. . . . . . . .
. . . . .
Vv06g1322 . . . . . . .
. . . . . . . .
. . . . .
Vv06g1323 . . . . . . .
. . . . . . . .
. . . . .
Vv06g1324 . . . . . . .
. . . . . . . .
. . . . .
Vv06g1325 . . . . . . .
. . . . . . . .
. . . . .
Vv06g1326 . . . . . . .
. . . . . . . .
. . . . .
Vv06g1327 . . . . . . .
. . . . . . . .
. . . . .
Vv06g1328 . . . . . . .
. . . . . . . .
. . . . .
Vv06g1329 . . . . . . .
. . . . . . . .
. . . . .
Vv06g1330 . . . . . . .
. . . . . . . .
. . . . .
Vv06g1331 . . . . . . .
. . . . . . . .
. . . . .
Vv06g1332 . . . . . . .
. . . . . . . .
. . . . .
Vv06g1333 . . . . . . .
. . . . . . . .
. . . . .
Vv06g1334 . . . . . . .
. . . . . . . .

. . . . .
Vv06g1335 . . . . . . .
. . . . . . . .
. . . . .
Vv06g1336 . . . . . . .
. . . . . . . .
. . . . .
Vv06g1337 . . . . . . .
. . . . . . . .
. . . . .
Vv06g1338 . . . . . . .
. . . . . . . .
. . . . .
Vv06g1339 . . . . . . .
. . . . . . . .
. . . . .
Vv06g1340 . . . . . . .
. . . . . . . .
. . . . .
Vv06g1341 . . . . . . .
. . . . . . . .
. . . . .
Vv06g1342 . . . . . . .
. . . . . . . .
. . . . .
Vv06g1343 . . . . . . .
. . . . . . . .
. . . . .
Vv06g1344 . . . . . . .
. . . . . . . .
. . . . .
Vv06g1345 . . . . . . .
. . . . . . . .
. . . . .
Vv06g1346 . . . . . . .
. . . . . . . .
. . . . .
Vv06g1347 . . . . . . .
. . . . . . . .
. . . . .
Vv06g1348 . . . . . . .
. . . . . . . .
. . . . .
Vv06g1349 . . . . . . .
. . . . . . . .
. . . . .
Vv06g1350 . . . . . . .
. . . . . . . .
. . . . .
Vv06g1351 . . . . . . .

. . . . . . . .
. . . . .
Vv06g1352 . . . . . . .
. . . . . . . .
. . . . .
Vv06g1353 . . . . . . .
. . . . . . . .
. . . . .
Vv06g1354 . . . . . . .
. . . . . . . .
. . . . .
Vv06g1355 . . . . . . .
. . . . . . . .
. . . . .
Vv06g1356 . . . . . . .
. . . . . . . .
. . . . .
Vv06g1357 . . . . . . .
. . . . . . . .
. . . . .
Vv06g1358 . . . . . . .
. . . . . . . .
. . . . .
Vv06g1359 . . . . . . .
. . . . . . . .
. . . . .
Vv06g1360 . . . . . . .
. . . . . . . .
. . . . .
Vv06g1361 . . . . . . .
. . . . . . . .
. . . . .
Vv06g1362 . . . . . . .
. . . . . . . .
. . . . .
Vv06g1363 Tc09_g009260 . . . . .
. . . . . . . .
. . . . . .
Vv06g1364 . . . . . . .
. . . . . . . .
. . . . .
Vv06g1365 . . . . . . .
. . . . . . . .
. . . . .
Vv06g1366 . . . . . . .
. . . . . . . .
. . . . .
Vv06g1367 . . . . . . .
. . . . . . . .
. . . . .

Vv06g1368 . . . . . . .
. . . . . . . .
. . . . .
Vv06g1369 Tc09_g008980 . . . . .
. . . . . . . .
. . . . . .
Vv06g1370 . . . . . . .
. . . . . . . .
. . . . .
Vv06g1371 . . . . . . .
. . . . . . . .
. . . . .
Vv06g1372 . . . . . . .
. . . . . . . .
. . . . .
Vv06g1373 . . . . . . .
. . . . . . . .
. . . . .
Vv06g1374 . . . . . . .
. . . . . . . .
. . . . .
Vv06g1375 . . . . . . .
. . . . . . . .
. . . . .
Vv06g1376 . . . . . . .
. . . . . . . .
. . . . .
Vv06g1377 . . . . . . .
. . . . . . . .
. . . . .
Vv06g1378 . . . . . . .
. . . . . . . .
. . . . .
Vv06g1379 . Gorai.013G176100.1 . . .
. . . . . . . .
. . . . . . .
Vv06g1380 . . . . . . .
. . . . . . . .
. . . . .
Vv06g1381 . . . . . . .
. . . . . . . .
. . . . .
Vv06g1382 . . . . . . .
. . . . . . . .
. . . . .
Vv06g1383 . . . . . . .
. . . . . . . .
. . . . .
Vv06g1384 . . . . . . .
. . . . . . . .

| | | | | | | | |
|---|---|---|---|---|---|---|---|
| Vv06g1385 | . | . | . | . | . | . | . |
| Vv06g1386 | . | . | . | . | . | . | . |
| Vv06g1387 | . | . | . | . | . | . | . |
| Vv06g1388 | . | . | . | . | . | . | . |
| Vv06g1389 | . | . | . | . | . | . | . |
| Vv06g1390 | . | . | . | . | . | . | . |
| Vv06g1391 | . | . | . | . | . | . | . |
| Vv06g1392 | . | . | . | . | . | . | . |
| Vv06g1393 | . | . | . | . | . | . | . |
| Vv06g1394 | . | . | . | . | . | . | . |
| Vv06g1395 | . | . | . | . | . | . | . |
| Vv06g1396 | . | . | . | . | . | . | . |
| Vv06g1397 | . | . | . | . | . | . | . |
| Vv06g1398 | Tc09_g031580 | | . | . | . | . | . |
| Vv06g1399 | . | . | . | . | . | . | . |
| Vv06g1400 | . | . | . | . | . | . | . |
| Vv06g1401 | . | . | . | . | . | . | . |

. . . . . . . .
. . . . .
Vv06g1402 . . . . . . .
. . . . . . . .
. . . . .
Vv06g1403 . . . . . . .
. . . . . . . .
. . . . .
Vv06g1404 . . . . . . .
. . . . . . . .
. . . . .
Vv06g1405 . . . . . . .
. . . . . . . .
. . . . .
Vv06g1406 . . . . . . .
. . . . . . . .
. . . . .
Vv06g1407 . . . . . . .
. . . . . . . .
. . . . .
Vv06g1408 . . . . . . .
. . . . . . . .
. . . . .
Vv06g1409 . . . . . . .
. . . . . . . .
. . . . .
Vv06g1410 . . . . . . .
. . . . . . . .
. . . . .
Vv06g1411 Tc09_g021200 . . . . .
. . . . . . . .
. . . . . .
Vv06g1412 . . . . . . .
. . . . . . . .
. . . . .
Vv06g1413 . . . . . . .
. . . . . . . .
. . . . .
Vv06g1414 . . . . . . .
. . . . . . . .
. . . . .
Vv06g1415 . . . . . . .
. . . . . . . .
. . . . .
Vv06g1416 . . . . . . .
. . . . . . . .
. . . . .
Vv06g1417 . . . . . . .
. . . . . . . .
. . . . .

Vv06g1418 . . . . . . .
. . . . . . . .
. . . . .
Vv06g1419 Tc09_g031420 . . . . .
. . . . . . . .
. . . . . .
Vv06g1420 . . . . . . .
. . . . . . . .
. . . . .
Vv06g1421 . . . . . . .
. . . . . . . .
. . . . .
Vv06g1422 . . . . . . .
. . . . . . . .
. . . . .
Vv06g1423 . . . . . . .
. . . . . . . .
. . . . .
Vv06g1424 . . . . . . .
. . . . . . . .
. . . . .
Vv06g1425 Tc09_g021560 . . . . .
. . . . . . . .
. . . . . .
Vv06g1426 Tc09_g031010 . . . . .
. . . . . . . .
. . . . . .
Vv06g1427 . . . . . . .
. . . . . . . .
. . . . .
Vv06g1428 . . . . . . .
. . . . . . . .
. . . . .
Vv06g1429 Tc09_g030900 Gorai.013G177200.1 . .
. . . . . . . .
. . . . . . . .
Vv06g1430 . . . . . . .
. . . . . . . .
. . . . .
Vv06g1431 . . . . . . .
. . . . . . . .
. . . . .
Vv06g1432 . . . . . . .
. . . . . . . .
. . . . .
Vv06g1433 . . . . . . .
. . . . . . . .
. . . . .
Vv06g1434 . . . . . . .
. . . . . . . .

| | | | | | | | |
|---|---|---|---|---|---|---|---|
| . | . | . | . | . | | | |
| Vv06g1435 | . | . | . | . | . | . | . |
| . | . | . | . | . | . | . | . |
| . | . | . | . | . | | | |
| Vv06g1436 | . | . | . | . | . | . | . |
| . | . | . | . | . | . | . | . |
| . | . | . | . | . | | | |
| Vv06g1437 | Tc09_g030890 | | Gorai.013G177300.1 | | | . | . |
| . | . | . | . | . | . | . | . |
| . | . | . | . | . | . | . | . |
| Vv06g1438 | . | . | . | . | . | . | . |
| . | . | . | . | . | . | . | . |
| . | . | . | . | . | | | |
| Vv06g1439 | . | . | . | . | . | . | . |
| . | . | . | . | . | . | . | . |
| . | . | . | . | . | | | |
| Vv06g1440 | . | . | . | . | . | . | . |
| . | . | . | . | . | . | . | . |
| . | . | . | . | . | | | |
| Vv06g1441 | . | . | . | . | . | . | . |
| . | . | . | . | . | . | . | . |
| . | . | . | . | . | | | |
| Vv06g1442 | Tc09_g003120 | | . | . | . | . | . |
| . | . | . | . | . | . | . | . |
| . | . | . | . | . | . | | |
| Vv06g1443 | . | . | . | . | . | . | . |
| . | . | . | . | . | . | . | . |
| . | . | . | . | . | | | |
| Vv06g1444 | . | . | . | . | . | . | . |
| . | . | . | . | . | . | . | . |
| . | . | . | . | . | | | |
| Vv06g1445 | . | . | . | . | . | . | . |
| . | . | . | . | . | . | . | . |
| . | . | . | . | . | | | |
| Vv06g1446 | . | . | . | . | . | . | . |
| . | . | . | . | . | . | . | . |
| . | . | . | . | . | | | |
| Vv06g1447 | Tc09_g003150 | | . | . | . | . | . |
| . | . | . | . | . | . | . | . |
| . | . | . | . | . | . | | |
| Vv06g1448 | . | . | . | . | . | . | . |
| . | . | . | . | . | . | . | . |
| . | . | . | . | . | | | |
| Vv06g1449 | Tc09_g003180 | | . | . | . | . | . |
| . | . | . | . | . | . | . | . |
| . | . | . | . | . | . | | |
| Vv06g1450 | . | . | . | . | . | . | . |
| . | . | . | . | . | . | . | . |
| . | . | . | . | . | | | |
| Vv06g1451 | . | . | . | . | . | . | . |

| | | | | | | | |
|---|---|---|---|---|---|---|---|
| . | . | . | . | . | . | . | . |
| . | . | . | . | . | | | |
| Vv06g1452 | . | . | . | . | . | . | . |
| . | . | . | . | . | . | . | . |
| . | . | . | . | . | | | |
| Vv06g1453 | . | . | . | . | . | . | . |
| . | . | . | . | . | . | . | . |
| . | . | . | . | . | | | |
| Vv06g1454 | . | . | . | . | . | . | . |
| . | . | . | . | . | . | . | . |
| . | . | . | . | . | | | |
| Vv06g1455 | . | . | . | . | . | . | . |
| . | . | . | . | . | . | . | . |
| . | . | . | . | . | | | |
| Vv06g1456 | . | . | . | . | . | . | . |
| . | . | . | . | . | . | . | . |
| . | . | . | . | . | | | |
| Vv06g1457 | . | . | . | . | . | . | . |
| . | . | . | . | . | . | . | . |
| . | . | . | . | . | | | |
| Vv06g1458 | . | . | . | . | . | . | . |
| . | . | . | . | . | . | . | . |
| . | . | . | . | . | | | |
| Vv06g1459 | . | . | . | . | . | . | . |
| . | . | . | . | . | . | . | . |
| . | . | . | . | . | | | |
| Vv06g1460 | Tc09_g021750 | | . | . | . | . | . |
| . | . | . | . | . | . | . | . |
| . | . | . | . | . | . | | |
| Vv06g1461 | . | . | . | . | . | . | . |
| . | . | . | . | . | . | . | . |
| . | . | . | . | . | | | |
| Vv06g1462 | . | . | . | . | . | . | . |
| . | . | . | . | . | . | . | . |
| . | . | . | . | . | | | |
| Vv06g1463 | . | . | . | . | . | . | . |
| . | . | . | . | . | . | . | . |
| . | . | . | . | . | | | |
| Vv06g1464 | . | . | . | . | . | . | . |
| . | . | . | . | . | . | . | . |
| . | . | . | . | . | | | |
| Vv06g1465 | . | . | . | . | . | . | . |
| . | . | . | . | . | . | . | . |
| . | . | . | . | . | | | |
| Vv06g1466 | . | . | . | . | . | . | . |
| . | . | . | . | . | . | . | . |
| . | . | . | . | . | | | |
| Vv06g1467 | . | . | . | . | . | . | . |
| . | . | . | . | . | . | . | . |
| . | . | . | . | . | | | |

Vv06g1468 . . . . . . .
. . . . . . . .
. . . . .
Vv06g1469 . . . . . . .
. . . . . . . .
. . . . .
Vv06g1470 . . . . . . .
. . . . . . . .
. . . . .
Vv06g1471 . . . . . . .
. . . . . . . .
. . . . .
Vv06g1472 . . . . . . .
. . . . . . . .
. . . . .
Vv06g1473 . . . . . . .
. . . . . . . .
. . . . .
Vv06g1474 . . . . . . .
. . . . . . . .
. . . . .
Vv06g1475 . . . . . . .
. . . . . . . .
. . . . .
Vv06g1476 . . . . . . .
. . . . . . . .
. . . . .
Vv06g1477 . . . . . . .
. . . . . . . .
. . . . .
Vv06g1478 . . . . . . .
. . . . . . . .
. . . . .
Vv06g1479 . . . . . . .
. . . . . . . .
. . . . .
Vv06g1480 Tc09_g003520 . . . . .
. . . . . . . .
. . . . . .
Vv06g1481 . Gorai.013G177400.1 . . .
. . . . . . . .
. . . . . . .
Vv06g1482 . . . . . . .
. . . . . . . .
. . . . .
Vv06g1483 . . . . . . .
. . . . . . . .
. . . . .
Vv06g1484 . . . . . . .
. . . . . . . .

| | | | | | | | |
|---|---|---|---|---|---|---|---|
| . | . | . | . | . | | | |
| Vv06g1485 | . | . | . | . | . | . | . |
| . | . | . | . | . | . | . | . |
| . | . | . | . | . | | | |
| Vv06g1486 | . | . | . | . | . | . | . |
| . | . | . | . | . | . | . | . |
| . | . | . | . | . | | | |
| Vv06g1487 | . | . | . | . | . | . | . |
| . | . | . | . | . | . | . | . |
| . | . | . | . | . | | | |
| Vv06g1488 | . | Gorai.013G177600.1 | | | . | . | . |
| . | . | . | . | . | . | . | . |
| . | . | . | . | . | . | . | |
| Vv06g1489 | . | . | . | . | . | . | . |
| . | . | . | . | . | . | . | . |
| . | . | . | . | . | | | |
| Vv06g1490 | . | . | . | . | . | . | . |
| . | . | . | . | . | . | . | . |
| . | . | . | . | . | | | |
| Vv06g1491 | . | . | . | . | . | . | . |
| . | . | . | . | . | . | . | . |
| . | . | . | . | . | | | |
| Vv06g1492 | . | . | . | . | . | . | . |
| . | . | . | . | . | . | . | . |
| . | . | . | . | . | | | |
| Vv06g1493 | . | . | . | . | . | . | . |
| . | . | . | . | . | . | . | . |
| . | . | . | . | . | | | |
| Vv06g1494 | . | . | . | . | . | . | . |
| . | . | . | . | . | . | . | . |
| . | . | . | . | . | | | |
| Vv06g1495 | . | . | . | . | . | . | . |
| . | . | . | . | . | . | . | . |
| . | . | . | . | . | | | |
| Vv06g1496 | . | . | . | . | . | . | . |
| . | . | . | . | . | . | . | . |
| . | . | . | . | . | | | |
| Vv06g1497 | . | . | . | . | . | . | . |
| . | . | . | . | . | . | . | . |
| . | . | . | . | . | | | |
| Vv06g1498 | . | . | . | . | . | . | . |
| . | . | . | . | . | . | . | . |
| . | . | . | . | . | | | |
| Vv06g1499 | . | . | . | . | . | . | . |
| . | . | . | . | . | . | . | . |
| . | . | . | . | . | | | |
| Vv06g1500 | . | . | . | . | . | . | . |
| . | . | . | . | . | . | . | . |
| . | . | . | . | . | | | |
| Vv06g1501 | Tc09_g035230 | | . | . | . | . | . |

| | | | | | | | |
|---|---|---|---|---|---|---|---|
| . | . | . | . | . | . | . | . |
| . | . | . | . | . | . | | |
| Vv06g1502 | . | . | . | . | . | . | . |
| . | . | . | . | . | . | . | . |
| . | . | . | . | . | | | |
| Vv06g1503 | . | . | . | . | . | . | . |
| . | . | . | . | . | . | . | . |
| . | . | . | . | . | | | |
| Vv06g1504 | . | . | . | . | . | . | . |
| . | . | . | . | . | . | . | . |
| . | . | . | . | . | | | |
| Vv06g1505 | . | . | . | . | . | . | . |
| . | . | . | . | . | . | . | . |
| . | . | . | . | . | | | |
| Vv06g1506 | . | . | . | . | . | . | . |
| . | . | . | . | . | . | . | . |
| . | . | . | . | . | | | |
| Vv06g1507 | . | . | . | . | . | . | . |
| . | . | . | . | . | . | . | . |
| . | . | . | . | . | | | |
| Vv06g1508 | . | . | . | . | . | . | . |
| . | . | . | . | . | . | . | . |
| . | . | . | . | . | | | |
| Vv06g1509 | . | . | . | . | . | . | . |
| . | . | . | . | . | . | . | . |
| . | . | . | . | . | | | |
| Vv06g1510 | . | . | . | . | . | . | . |
| . | . | . | . | . | . | . | . |
| . | . | . | . | . | | | |
| Vv06g1511 | . | . | . | . | . | . | . |
| . | . | . | . | . | . | . | . |
| . | . | . | . | . | | | |
| Vv06g1512 | Tc09_g035200 | | . | . | . | . | . |
| . | . | . | . | . | . | . | . |
| . | . | . | . | . | . | | |
| Vv06g1513 | . | . | . | . | . | . | . |
| . | . | . | . | . | . | . | . |
| . | . | . | . | . | | | |
| Vv06g1514 | . | . | . | . | . | . | . |
| . | . | . | . | . | . | . | . |
| . | . | . | . | . | | | |
| Vv06g1515 | . | . | . | . | . | . | . |
| . | . | . | . | . | . | . | . |
| . | . | . | . | . | | | |
| Vv06g1516 | . | . | . | . | . | . | . |
| . | . | . | . | . | . | . | . |
| . | . | . | . | . | | | |
| Vv06g1517 | . | . | . | . | . | . | . |
| . | . | . | . | . | . | . | . |
| . | . | . | . | . | | | |

Vv06g1518 . . . . . . .
. . . . . . . .
. . . . .
Vv06g1519 . . . . . . .
. . . . . . . .
. . . . .
Vv06g1520 . . . . . . .
. . . . . . . .
. . . . .
Vv06g1521 . . . . . . .
. . . . . . . .
. . . . .
Vv06g1522 Tc09_g024300 Gorai.013G253300.1 . .
. Gorai.006G124500.1 . . . .
. . . . . . . .
. .
Vv06g1523 . . . . . . .
. . . . . . . .
. . . . .
Vv06g1524 Tc09_g024320 Gorai.013G253400.1 . .
. . . . . . . .
. . . . . . . .
Vv06g1525 Tc09_g024330 . . . Gorai.
009G077200.1 . . . . . . .
. . . . . . . .
Vv06g1526 Tc09_g024350 . Gorai.001G133600.1 .
. . . . . . . .
. . . . . . . .
Vv06g1527 . Gorai.013G253200.1 . . .
. . . . . . . .
. . . . . . .
Vv06g1528 . . . . . . .
. . . . . . . .
. . . . .
Vv06g1529 . . . . . . .
. . . . . . . .
. . . . .
Vv06g1530 Tc09_g024370 . . . . .
. . . . . . . .
. . . . . .
Vv06g1531 . . Gorai.001G133700.1 .
Gorai.009G074900.1 Gorai.006G124400.1 . . .
. . . . . . . .
. . .
Vv06g1532 . . . . . . .
. . . . . . . .
. . . . .
Vv06g1533 Tc09_g024380 . Gorai.001G133800.1 .
. . . . . . . .
. . . . . . . .

Vv06g1534 Tc09_g024390 . . . .
Gorai.006G124300.1 . . . . . .
. . . . . . . .
Vv06g1535 . . . . . . .
. . . . . . . .
. . . . .
Vv06g1536 Tc09_g024410 . . . . .
. . . . . . . .
. . . . . .
Vv06g1537 . . . . . . .
. . . . . . . .
. . . . .
Vv06g1538 Tc09_g024510 . . . Gorai.
009G075200.1 . . . . . . .
. . . . . . . .
Vv06g1539 Tc09_g024520 . . . .
Gorai.006G124000.1 . . . . . .
. . . . . . . .
Vv06g1540 Tc09_g024530 . Gorai.001G134000.1 .
. . . . . . . .
. . . . . . . .
Vv06g1541 Tc09_g024540 . Gorai.001G134100.1 .
Gorai.009G075300.1 Gorai.006G123900.1 . .
. . . . . . . .
. . . .
Vv06g1542 . . . . . . .
. . . . . . . .
. . . . .
Vv06g1543 Tc09_g024560 . Gorai.001G134300.1 .
Gorai.009G075400.1 . . . . .
. . . . . . . .
. .
Vv06g1544 Tc09_g024570 . . . . .
. . . . . . . .
. . . . . .
Vv06g1545 . . . . . . .
. . . . . . . .
. . . . .
Vv06g1546 Tc09_g024580 . Gorai.001G134600.1 .
Gorai.009G075500.1 . . . . .
. . . . . . . .
. .
Vv06g1547 Tc09_g024590 . Gorai.001G134800.1 .
. . . . . . . .
. . . . . . . .
Vv06g1548 Tc09_g024610 . . . .
Gorai.006G123800.1 . . . . . .
. . . . . . . .
Vv06g1549 Tc09_g024630 . . . . .
. . . . . . . .

. . . . . .
Vv06g1550 . . . . . . .
. . . . . . . .
. . . . .
Vv06g1551 Tc09_g003120 . . . . .
. . . . . . . .
. . . . . .
Vv06g1552 . . . . Gorai.009G075600.1
. . . . . . . .
. . . . . . .
Vv06g1553 . . . . . . .
. . . . . . . .
. . . . .
Vv06g1554 Tc09_g024840 . . . . .
. . . . . . . .
. . . . . .
Vv06g1555 Tc09_g025170 . . . Gorai.
009G077400.1 Gorai.006G120900.1 . . . .
. . . . . . . .
. .
Vv06g1556 Tc09_g025230 . Gorai.001G135700.1 .
. . . . . . . .
. . . . . . . .
Vv06g1557 . Gorai.013G252800.1 . . .
Gorai.006G133200.1 . . . . .
. . . . . . . .
.
Vv06g1558 . . . . . . .
. . . . . . . .
. . . . .
Vv06g1559 . . . . . Gorai.
006G125400.1 . . . . . . .
. . . . . . .
Vv06g1560 . . . . . . .
. . . . . . . .
. . . . .
Vv06g1561 . . . . . . .
. . . . . . . .
. . . . .
Vv06g1562 . . . . . . .
. . . . . . . .
. . . . .
Vv06g1563 . . . . Gorai.009G074800.1
. . . . . . . .
. . . . . . .
Vv06g1564 . . . . Gorai.009G108800.1
Gorai.006G125500.1 . . . . .
. . . . . . . .
.
Vv06g1565 Tc09_g003380 . . . . .

. . . . . . . .
. . . . . .
Vv06g1566 . Gorai.013G252400.1 . . .
. . . . . . . .
. . . . . . .
Vv06g1567 . . . . . Gorai.
006G125600.1 . . . . . . .
. . . . . . .
Vv06g1568 . . Gorai.001G136400.1 . .
. . . . . . . .
. . . . . . .
Vv06g1569 . . . . . . .
. . . . . . . .
. . . . .
Vv06g1570 . Gorai.013G252000.1 . . .
. . . . . . . .
. . . . . . .
Vv06g1571 . . . . . . .
. . . . . . . .
. . . . .
Vv06g1572 . . . . Gorai.009G074300.1
Gorai.006G125800.1 . . . . .
. . . . . . . .
.
Vv06g1573 . . . . . . .
. . . . . . . .
. . . . .
Vv06g1574 . Gorai.013G251900.1 . . .
. . . . . . . .
. . . . . . .
Vv06g1575 . . . . . . .
. . . . . . . .
. . . . .
Vv06g1576 . . . . . Gorai.
006G126000.1 . . . . . . .
. . . . . . .
Vv06g1577 Tc09_g003720 . Gorai.001G136800.1 .
. . . . . . . .
. . . . . . . .
Vv06g1578 . . Gorai.001G136900.1 . .
. . . . . . . .
. . . . . . .
Vv06g1579 . Gorai.013G251800.1 . .
Gorai.009G074200.1 Gorai.006G135000.1 . . .
. . . . . . . .
. . .
Vv06g1580 . . . . . . .
. . . . . . . .
. . . . .
Vv06g1581 . . . . . . .

. . . . . . . .
. . . . .
Vv06g1582 Tc09_g008700 . . . . .
. . . . . . . .
. . . . . .
Vv06g1583 . . . . . . .
. . . . . . . .
. . . . .
Vv06g1584 . . . . . . .
. . . . . . . .
. . . . .
Vv06g1585 . Gorai.013G251700.1 Gorai.001G137100.1
. . Gorai.006G126400.1 . . .
. . . . . . . .
. . .
Vv06g1586 . . . . . . .
. . . . . . . .
. . . . .
Vv06g1587 . . . . . . .
. . . . . . . .
. . . . .
Vv06g1588 . . . . . . .
. . . . . . . .
. . . . .
Vv06g1589 . . . . Gorai.009G074000.1
Gorai.006G126600.1 . . . . .
. . . . . . . .
.
Vv06g1590 . . . . . Gorai.
006G136100.1 . . . . . . .
. . . . . . .
Vv06g1591 . . . . . . .
. . . . . . . .
. . . . .
Vv06g1592 Tc09_g008960 . . . . .
. . . . . . . .
. . . . . .
Vv06g1593 . . . . . . .
. . . . . . . .
. . . . .
Vv06g1594 . . . . . . .
. . . . . . . .
. . . . .
Vv06g1595 . . . . . . .
. . . . . . . .
. . . . .
Vv06g1596 . . . . . . .
. . . . . . . .
. . . . .
Vv06g1597 . . . . . . .

. . . . . . . .
. . . . .
Vv06g1598 . . . . . . .
. . . . . . . .
. . . . .
Vv06g1599 . Gorai.013G268400.1 . .
Gorai.009G071200.1 . . . . . .
. . . . . . . .
.
Vv06g1600 . Gorai.013G268500.1 . .
Gorai.009G110000.1 Gorai.003G143900.1 . . .
. . . . . . . .
. . .
Vv06g1601 . . . . . . .
. . . . . . . .
. . . . .
Vv06g1602 . Gorai.013G268600.1 . . .
Gorai.003G144000.1 . . . . .
. . . . . . . .
.
Vv06g1603 . . . . . . .
. . . . . . . .
. . . . .
Vv06g1604 . . . . . Gorai.
003G144100.1 . . . . . . .
. . . . . . .
Vv06g1605 . . . . . . .
. . . . . . . .
. . . . .
Vv06g1606 . . . . Gorai.009G110300.1
Gorai.006G165000.1 . . . . .
. . . . . . . .
.
Vv06g1607 . . . . Gorai.009G110400.1
. . . . . . . .
. . . . . . .
Vv06g1608 . . . . . . .
. . . . . . . .
. . . . .
Vv06g1609 . . . . . . .
. . . . . . . .
. . . . .
Vv06g1610 . . . . . . .
. . . . . . . .
. . . . .
Vv06g1611 Tc09_g004980 . . . . .
. . . . . . . .
. . . . . .
Vv06g1612 Tc09_g014260 . . . . .
. . . . . . . .

. . . . . .
Vv06g1613 . . . . . . .
. . . . . . . .
. . . . .
Vv06g1614 . . . . . . .
. . . . . . . .
. . . . .
Vv06g1615 . Gorai.013G268700.1 . . .
Gorai.003G144600.1 . . . . .
. . . . . . . .
.
Vv06g1616 . . . . . . .
. . . . . . . .
. . . . .
Vv06g1617 . Gorai.013G268900.1 . . .
. . . . . . . .
. . . . . .
Vv06g1618 . . . . . . .
. . . . . . . .
. . . . .
Vv06g1619 . . . . . . .
. . . . . . . .
. . . . .
Vv06g1620 . . . . . . .
. . . . . . . .
. . . . .
Vv06g1621 . . . . Gorai.009G110700.1
Gorai.006G163200.1 . . . . .
. . . . . . . .
.
Vv06g1622 . . . . . . .
. . . . . . . .
. . . . .
Vv06g1623 . . . . . . .
. . . . . . . .
. . . . .
Vv06g1624 . Gorai.013G269100.1 . . .
Gorai.006G162900.1 Vv08g1449 . Gorai.
006G162900.1 . . . . . . .
. . . .
Vv06g1625 Tc09_g009270 Gorai.013G269200.1 . .
. Gorai.003G145100.1 . . . .
. . . . . . . .
. .
Vv06g1626 . . . . . . .
. . . . . . . .
. . . . .
Vv06g1627 Tc09_g016760 . . . . .
. . . . . . . .
. . . . . .

Vv06g1628 . . . . . . .
. . . . . . . .
. . . . .
Vv06g1629 Tc09_g005160 . . . . .
. . . . . . . .
. . . . . .
Vv06g1630 . . . . . Gorai.
006G162700.1 Vv08g1442 Tc05_g007120 Gorai.006G162700.1
. . Gorai.009G414500.1 Gorai.007G373100.1
. . . . . . .
Vv06g1631 . . . . . . .
. . . . . . . .
. . . . .
Vv06g1632 Tc09_g014490 . . . . .
. . . . . . . .
. . . . . .
Vv06g1633 . . . . . . .
. . . . . . . .
. . . . .
Vv06g1634 . . . . . . .
. . . . . . . .
. . . . .
Vv06g1635 . . . . . Gorai.
003G145500.1 . . . . . . .
. . . . . . .
Vv06g1636 Tc09_g009340 . . . Gorai.
009G110900.1 Gorai.006G162400.1 Vv08g1437
Tc05_g007170 Gorai.006G162400.1 . . Gorai.
009G414100.1 Gorai.007G373000.1 . . . .
. . .
Vv06g1637 . Gorai.013G269400.1 . .
Gorai.009G111000.1 Gorai.006G138400.1 . . .
. . . . . . . .
. . .
Vv06g1638 . . . . . . .
. . . . . . . .
. . . . .
Vv06g1639 . . . . . . .
. . . . . . . .
. . . . .
Vv06g1640 . . . . . . .
. . . . . . . .
. . . . .
Vv06g1641 . . . . Gorai.009G111200.1
Gorai.006G161600.1 Vv08g1430 Tc05_g007230
Gorai.006G161600.1 . . . . . .
. . . . .
Vv06g1642 . . . . . Gorai.
003G145800.1 . . . . . . .
. . . . . . .

Vv06g1643 . . . . . . .
. . . . . . . .
. . . . .
Vv06g1644 . . . . Gorai.009G111300.1
. . . . . . . .
. . . . . . .
Vv06g1645 . . . . . . .
. . . . . . . .
. . . . .
Vv06g1646 Tc09_g016530 . . . . .
. . . . . . . .
. . . . . .
Vv06g1647 . . . . . . .
. . . . . . . .
. . . . .
Vv06g1648 . . . . . . .
. . . . . . . .
. . . . .
Vv06g1649 Tc09_g009730 . . . . .
. . . . . . . .
. . . . . .
Vv06g1650 . . . . . . .
. . . . . . . .
. . . . .
Vv06g1651 . . . . . . .
. . . . . . . .
. . . . .
Vv06g1652 . . . . Gorai.009G111400.1
Gorai.003G145900.1 . . . . .
. . . . . . . .
.
Vv06g1653 . . . . . Gorai.
003G146000.1 . . . . . . .
. . . . . . .
Vv06g1654 . . . . . Gorai.
003G146100.1 . . . . . . .
. . . . . . .
Vv06g1655 . . . . . Gorai.
003G146200.1 Vv08g1424 . . . .
Gorai.009G413400.1 . . . . . .
. .
Vv06g1656 Tc09_g029640 . . . Gorai.
009G090400.1 Gorai.006G105000.1 . . . .
. . . . . . . .
. .
Vv06g1657 . . . . . .
Vv08g1423 . . Gorai.002G248400.1 .
Gorai.009G413000.1 Gorai.007G372700.1 . . .
. . . .
Vv06g1658 Tc09_g029630 Gorai.013G259300.1 . .

```
       .      .      .      .      .      .      .      .
       .      .      .      .      .      .      .      .
Vv06g1659      .      .      .      .      Gorai.009G090300.1
       Gorai.006G104900.1      .      .      .      .      .
       .      .      .      .      .      .      .      .
       .
Vv06g1660      Tc09_g029610      .      Gorai.001G117100.1      .
       Gorai.009G090200.1      Gorai.006G161200.1      .      .
       .      .      .      .      .      .      .      .
       .      .      .      .
Vv06g1661      Tc09_g029600      .      .      .      Gorai.
009G090100.1      .      .      .      .      .      .      .
       .      .      .      .      .      .      .      .
Vv06g1662      .      .      .      .      .      .      .
       .      .      .      .      .      .      .      .
       .      .      .      .      .
Vv06g1663      Tc09_g029570      .      Gorai.001G117200.1      .
       .      .      .      .      .      .      .      .
       .      .      .      .      .      .      .      .
Vv06g1664      Tc09_g029560      .      .      .      .      .
       .      .      .      .      .      .      .      .
       .      .      .      .      .      .
Vv06g1665      Tc09_g029380      .      Gorai.001G117400.1      .
       Gorai.009G089700.1      .      .      .      .      .
       .      .      .      .      .      .      .      .
       .      .
Vv06g1666      Tc09_g029370      .      .      .      .
Gorai.006G158300.1      Vv08g1389      .      .      Gorai.
002G245500.1      .      .      .      .      .      .      .
       .      .      .
Vv06g1667      .      .      Gorai.001G117500.1      .      .
       Gorai.006G104200.1      Vv08g1388      .      .
Gorai.002G245400.1      .      .      .      .      .      .
       .      .      .      .
Vv06g1668      .      .      .      .      .      .      .
       .      .      .      .      .      .      .      .
       .      .      .      .      .
Vv06g1669      Tc09_g029340      .      Gorai.001G117600.1      .
       Gorai.009G089300.1      Gorai.006G104000.1      .      .
       .      .      .      .      .      .      .      .
       .      .      .      .
Vv06g1670      Tc09_g029300      .      Gorai.001G117900.1      .
       Gorai.009G089200.1      .      .      .      .      .
       .      .      .      .      .      .      .      .
       .      .
Vv06g1671      Tc09_g029270      .      .      .      .
Gorai.006G103700.1      Vv08g1386      .      .      .      .
       .      .      .      .      .      .      .      .
       .
Vv06g1672      .      .      .      .      .      .      .
```

. . . . . . . .
. . . . .
Vv06g1673 Tc09_g029260 . Gorai.001G118200.1 .
Gorai.009G089000.1 . . . . .
. . . . . . . .
. .
Vv06g1674 . . . Gorai.004G007900.1 .
. . . . . . . .
. . . . . . .
Vv06g1675 . . . . . Gorai.
006G103600.1 . . . . . . .
. . . . . . .
Vv06g1676 Tc09_g029250 . Gorai.001G118300.1 .
Gorai.009G088900.1 . . . . .
. . . . . . . .
. .
Vv06g1677 . . . . . . .
. . . . . . . .
. . . . .
Vv06g1678 Tc09_g029240 . . . . .
. . . . . . . .
. . . . . .
Vv06g1679 . . . . . . .
. . . . . . . .
. . . . .
Vv06g1680 Tc09_g029230 . . . . .
. . . . . . . .
. . . . . .
Vv06g1681 . . . . . . .
. . . . . . . .
. . . . .
Vv06g1682 . . . . . . .
. . . . . . . .
. . . . .
Vv06g1683 Tc09_g029220 . . . . .
. . . . . . . .
. . . . . .
Vv06g1684 . . . . . Gorai.
006G103400.1 . . . . . . .
. . . . . . .
Vv06g1685 Tc09_g029210 . Gorai.001G118400.1 .
. . . . . . . .
. . . . . . . .
Vv06g1686 Tc09_g029200 Gorai.013G259200.1 Gorai.
001G118500.1 . . Gorai.006G103200.1 Vv08g1374
Tc05_g007650 . Gorai.002G245300.1 . .
. . . . . . . .
Vv06g1687 Tc09_g029180 . . . .
Gorai.006G103000.1 . . . . . .
. . . . . . . .

Vv06g1688 Tc09_g029160 . . . Gorai.
009G088800.1 Gorai.006G102900.1 . . . .
. . . . . . . .
. .
Vv06g1689 Tc09_g029150 Gorai.013G259100.1 . .
. Gorai.006G102800.1 . . . .
. . . . . . . .
. .
Vv06g1690 . . . . . . .
. . . . . . . .
. . . . .
Vv06g1691 . . . . . . .
. . . . . . . .
. . . . .
Vv06g1692 Tc09_g029140 . Gorai.001G118600.1 .
. . . . . . . .
. . . . . . . .
Vv06g1693 . . . . . Gorai.
006G100200.1 . . . . . . .
. . . . . . .
Vv06g1694 Tc09_g029130 Gorai.013G259000.1 . .
. . . . . . . .
. . . . . . . .
Vv06g1695 Tc09_g029120 . Gorai.001G118700.1 .
. . . . . . . .
. . . . . . . .
Vv06g1696 Tc09_g029110 Gorai.013G258900.1 Gorai.
001G118800.1 . . . . . . .
. . . . . . . .
. .
Vv06g1697 Tc09_g029070 Gorai.013G258800.1 Gorai.
001G118900.1 . Gorai.009G088500.1 Gorai.006G108100.1
. . . . . . . .
. . . . . .
Vv06g1698 Tc09_g029060 . Gorai.001G119000.1 .
. Gorai.006G108300.1 . . . .
. . . . . . . .
. .
Vv06g1699 . . . . . . .
. . . . . . . .
. . . . .
Vv06g1700 Tc09_g029050 Gorai.013G258700.1 . .
. Gorai.006G108400.1 . . . .
. . . . . . . .
. .
Vv06g1701 Tc09_g029040 . . Gorai.004G008000.1
. Gorai.006G108500.1 . . . .
. . . . . . . .
. .
Vv06g1702 . . . . . . .

. . . . . . . .
. . . . .
Vv06g1703 Tc09_g029030 . . . .
Gorai.006G108600.1 . . . . . .
. . . . . . . .
Vv06g1704 Tc09_g029010 Gorai.013G258600.1 . .
. . . . . . . .
. . . . . . . .
Vv06g1705 . . . . . . .
. . . . . . . .
. . . . .
Vv06g1706 Tc09_g028980 . . . .
Gorai.006G109000.1 . . . . . .
. . . . . . . .
Vv06g1707 Tc09_g028950 . . Gorai.004G008100.1
. Gorai.006G109100.1 . . . .
. . . . . . . .
. .
Vv06g1708 Tc09_g028910 . . . .
Gorai.006G109300.1 . . . . . .
. . . . . . . .
Vv06g1709 . . . . . . .
. . . . . . . .
. . . . .
Vv06g1710 . . . . . . .
. . . . . . . .
. . . . .
Vv06g1711 Tc09_g028900 . Gorai.001G119300.1 .
. Gorai.006G109400.1 . . . .
. . . . . . . .
. .
Vv06g1712 Tc09_g028890 . Gorai.001G119400.1 .
. . . . . . . .
. . . . . . . .
Vv06g1713 Tc09_g028880 . . . Gorai.
009G088400.1 . . . . . . .
. . . . . . . .
Vv06g1714 . . . . . Gorai.
006G158300.1 . . . . . . .
. . . . . . .
Vv06g1715 . . . . . . .
. . . . . . . .
. . . . .
Vv06g1716 . . . . . . .
. . . . . . . .
. . . . .
Vv06g1717 Tc09_g028840 . . Gorai.004G008200.1
. Gorai.006G109800.1 . . . .
. . . . . . . .
. .

Vv06g1718 . . . . . . .
. . . . . . . .
. . . . .
Vv06g1719 Tc09_g028820 . Gorai.001G119800.1 .
. . . . . . . .
. . . . . . . .
Vv06g1720 . . . . . . .
. . . . . . . .
. . . . .
Vv06g1721 . . . . . . .
. . . . . . . .
. . . . .
Vv06g1722 Tc09_g028810 . . Gorai.004G008300.1
. . . . . . . .
. . . . . . . .
Vv06g1723 Tc09_g028800 . Gorai.001G119900.1 .
Gorai.009G088100.1 . . . . .
. . . . . . . .
. .
Vv06g1724 Tc09_g028790 . Gorai.001G120000.1 .
Gorai.009G088000.1 Gorai.006G109900.1 . .
. . . . . . . .
. . . .
Vv06g1725 Tc09_g028780 . . . .
Gorai.006G110000.1 . . . . . .
. . . . . . . .
Vv06g1726 Tc09_g028770 . . Gorai.004G008400.1
Gorai.009G087900.1 . . . . .
. . . . . . . .
. .
Vv06g1727 . . . . . . .
. . . . . . . .
. . . . .
Vv06g1728 . . . . . . .
. . . . . . . .
. . . . .
Vv06g1729 Tc09_g028750 . . . Gorai.
009G087700.1 . . . . . . .
. . . . . . . .
Vv06g1730 Tc09_g028740 . . . .
Gorai.006G110100.1 . . . . . .
. . . . . . . .
Vv06g1731 Tc09_g028730 . Gorai.001G120100.1 .
. . . . . . . .
. . . . . . . .
Vv06g1732 Tc09_g028710 . Gorai.001G120300.1 .
. Gorai.006G157400.1 . . . .
. . . . . . . .
. .
Vv06g1733 Tc09_g028700 . Gorai.001G120400.1 .

. . . . . . . .
. . . . . . . .
Vv06g1734 . . . . . . .
. . . . . . . .
. . . . .
Vv06g1735 . . . . . . .
. . . . . . . .
. . . . .
Vv06g1736 . . . . . . .
. . . . . . . .
. . . . .
Vv06g1737 . . . . . . .
. . . . . . . .
. . . . .
Vv06g1738 . . . . . . .
. . . . . . . .
. . . . .
Vv06g1739 Tc09_g028690 . . . .
Gorai.006G157200.1 . . . . . .
. . . . . . . .
Vv06g1740 . . . . . . .
. . . . . . . .
. . . . .
Vv06g1741 . . . . . . .
. . . . . . . .
. . . . .
Vv06g1742 . . . . . . .
. . . . . . . .
. . . . .
Vv06g1743 Tc09_g028670 . . . Gorai.
009G087600.1 . . . . . . .
. . . . . . . .
Vv06g1744 . . . . . . .
. . . . . . . .
. . . . .
Vv06g1745 Tc09_g028660 Gorai.013G256700.1 . .
. Gorai.006G110500.1 . . . .
. . . . . . . .
. .
Vv06g1746 Tc09_g028630 Gorai.013G253900.1 . .
. Gorai.006G110700.1 . . . .
. . . . . . . .
. .
Vv06g1747 . . . . Gorai.009G087400.1
. . . . . . . .
. . . . . . .
Vv06g1748 Tc09_g028620 . . . . .
. . . . . . . .
. . . . . .
Vv06g1749 . . . . . Gorai.

006G156900.1 . . . . . . .
. . . . . . .
Vv06g1750 Tc09_g028610 . Gorai.001G120600.1 .
. . . . . . . .
. . . . . . . .
Vv06g1751 . . . . . . .
. . . . . . . .
. . . . .
Vv06g1752 Tc09_g028600 . Gorai.001G120700.1 .
. . . . . . . .
. . . . . . . .
Vv06g1753 . . . . . . .
. . . . . . . .
. . . . .
Vv06g1754 Tc09_g028580 . Gorai.001G120800.1 .
Gorai.009G087300.1 Gorai.006G156700.1 . .
. . . . . . . .
. . . .
Vv06g1755 Tc09_g028560 . . . Gorai.
009G087200.1 . . . . . . .
. . . . . . . .
Vv06g1756 . . . . . Gorai.
006G153700.1 . . . . . . .
. . . . . . .
Vv06g1757 . . . . . . .
. . . . . . . .
. . . . .
Vv06g1758 Tc09_g028550 . Gorai.001G120900.1 .
. . . . . . . .
. . . . . . . .
Vv06g1759 Tc09_g028540 . Gorai.001G121000.1 .
. . . . . . . .
. . . . . . . .
Vv06g1760 Tc09_g028530 . . . Gorai.
009G087100.1 . . . . . . .
. . . . . . . .
Vv06g1761 Tc09_g028520 . . Gorai.004G008500.1
. . . . . . . .
. . . . . . . .
Vv06g1762 . . . . . . .
. . . . . . . .
. . . . .
Vv06g1763 . . . . . . .
. . . . . . . .
. . . . .
Vv06g1764 Tc09_g028510 . . . Gorai.
009G087000.1 . . . . . . .
. . . . . . . .
Vv06g1765 . . . . . . .
. . . . . . . .

. . . . .
Vv06g1766 . . . . . . .
. . . . . . . .
. . . . .
Vv06g1767 Tc09_g028460 . . Gorai.004G009100.1
Gorai.009G086900.1 . . . . .
. . . . . . . .
. .
Vv06g1768 Tc09_g028430 . . . . .
. . . . . . . .
. . . . . .
Vv06g1769 Tc09_g028090 . Gorai.001G121200.1 .
. Gorai.006G111100.1 . . . .
. . . . . . . .
. .
Vv06g1770 . . . . . . .
. . . . . . . .
. . . . .
Vv06g1771 Tc09_g028080 . . Gorai.004G009200.1
. . . . . . . .
. . . . . . . .
Vv06g1772 Tc09_g028070 . . Gorai.004G009300.1
Gorai.009G083900.1 . . . . .
. . . . . . . .
. .
Vv06g1773 Tc09_g028060 . . . . .
. . . . . . . .
. . . . . .
Vv06g1774 Tc09_g028050 . . . . .
. . . . . . . .
. . . . . .
Vv06g1775 . . . . . . .
. . . . . . . .
. . . . .
Vv06g1776 . . . . . . .
. . . . . . . .
. . . . .
Vv06g1777 . . . . . . .
. . . . . . . .
. . . . .
Vv06g1778 . . . . . . .
. . . . . . . .
. . . . .
Vv06g1779 . . . . . . .
. . . . . . . .
. . . . .
Vv07g0001 . . . . . . .
. . . . . . . .
. . . . .
Vv07g0002 . . . . . . .

. . . . . . . .
. . . . .
Vv07g0003 . . . . . . .
. . . . . . . .
. . . . .
Vv07g0004 . . . . . . .
. . . . . . . .
. . . . .
Vv07g0005 . Gorai.007G143800.1 . . .
. . . . . . . .
. . . . . . .
Vv07g0006 . . . . . . .
. . . . . . . .
. . . . .
Vv07g0007 . . . . . . .
. . . . . . . .
. . . . .
Vv07g0008 . . . . . . .
. . . . . . . .
. . . . .
Vv07g0009 Tc01_g032180 . . . . .
. . . . . . . .
. . . . . .
Vv07g0010 . . . . . . .
. . . . . . . .
. . . . .
Vv07g0011 . . . . . . .
. . . . . . . .
. . . . .
Vv07g0012 . . . . Gorai.004G056900.1
. . . . . . . .
. . . . . . .
Vv07g0013 . . . . Gorai.004G073900.1
. . . . . . . .
. . . . . . .
Vv07g0014 . Gorai.007G143300.1 . .
Gorai.004G074000.1 . . . . . .
. . . . . . . .
.
Vv07g0015 . . . . . . .
. . . . . . . .
. . . . .
Vv07g0016 . Gorai.007G143200.1 . . .
Gorai.004G247700.1 . . . . .
. . . . . . . .
.
Vv07g0017 Tc01_g031900 . Gorai.008G138300.1 .
. . . . . . . .
. . . . . . . .
Vv07g0018 . . . . Gorai.004G074100.1

. Vv05g0696 Tc04_g024760 . . .
Gorai.011G171100.1 . . . . .
. . .
Vv07g0019 . . . . . .
Vv05g0693 . . . . . . .
. . . . . .
Vv07g0020 . . . . . . .
. . . . . . . .
. . . . .
Vv07g0021 . . . . . . .
. . . . . . . .
. . . . .
Vv07g0022 . . . . . .
Vv05g0692 Tc04_g024750 Gorai.006G246500.1 Gorai.
005G046800.1 . Gorai.011G171000.1 . . .
. . . . .
Vv07g0023 Tc01_g005480 . Gorai.008G077900.1 .
. . . . . . . .
. . . . . . . .
Vv07g0024 Tc01_g005580 Gorai.007G142800.1 . .
. Gorai.004G247000.1 . . . .
. . . . . . . .
. .
Vv07g0025 . Gorai.007G142700.1 . . .
Gorai.004G219200.1 . . . . .
. . . . . . . .
.
Vv07g0026 . . . . . . .
. . . . . . . .
. . . . .
Vv07g0027 . Gorai.007G142600.1 . . .
. . . . . . . .
. . . . . . .
Vv07g0028 . Gorai.007G142500.1 . .
Gorai.004G074300.1 . Vv05g0686 . . .
. . . . . . . .
. .
Vv07g0029 . . . . . . .
. . . . . . . .
. . . . .
Vv07g0030 . . . . Gorai.004G074400.1
. Vv05g0684 Tc04_g024650 . . .
Gorai.011G170800.1 . . . . .
. . .
Vv07g0031 Tc01_g029000 Gorai.007G142400.1 Gorai.
001G243400.1 . Gorai.004G074500.1 Gorai.004G219100.1
. . . . . . . .
. . . . . .
Vv07g0032 . Gorai.007G142300.1 Gorai.001G243300.1
. . Gorai.004G219000.1 . . .

```
. . . . . . . .
. . .
Vv07g0033 . . . . . . .
. . . . . . . .
. . . . .
Vv07g0034 . . . . . . .
. . . . . . . .
. . . . .
Vv07g0035 . . . . . . .
. . . . . . . .
. . . . .
Vv07g0036 . . . . . . .
. . . . . . . .
. . . . .
Vv07g0037 . . . . . . .
. . . . . . . .
. . . . .
Vv07g0038 . . . . . . .
. . . . . . . .
. . . . .
Vv07g0039 . . . . . . .
. . . . . . . .
. . . . .
Vv07g0040 . . Gorai.001G243200.1 . .
. . . . . . . .
. . . . . . .
Vv07g0041 . Gorai.007G142200.1 . . .
. . . . . . . .
. . . . . . .
Vv07g0042 . . . . . . .
. . . . . . . .
. . . . .
Vv07g0043 . . . . . . .
. . . . . . . .
. . . . .
Vv07g0044 . Gorai.007G142100.1 . .
Gorai.004G074700.1 . . . . . .
. . . . . . . .
.
Vv07g0045 . . Gorai.001G243000.1 . .
. . . . . . . .
. . . . . . .
Vv07g0046 . Gorai.007G142000.1 . . .
. . . . . . . .
. . . . . . .
Vv07g0047 . . . . . . .
. . . . . . . .
. . . . .
Vv07g0048 . . Gorai.001G242900.1 . .
. Vv05g0680 Tc04_g024600 Gorai.006G246000.1
```

```
        Gorai.005G046500.1      .       Gorai.011G170700.1      .
        .       .       .       .       .       .       .
Vv07g0049       .       Gorai.007G141900.1      Gorai.001G242800.1
        .       Gorai.004G075000.1      .       Vv05g0679
Tc04_g024590    Gorai.006G245900.1      Gorai.005G046400.1      .
        .       .       .       .       .       .       .       .
        .
Vv07g0050       .       .       Gorai.001G242700.1      .       .
        .       .       .       .       .       .       .       .
        .       .       .       .       .       .       .
Vv07g0051       .       .       Gorai.001G242600.1      .
Gorai.004G056700.1      .       .       .       .       .       .
        .       .       .       .       .       .       .       .
        .
Vv07g0052       Tc01_g028690    Gorai.007G141800.1      Gorai.
001G242500.1    .       Gorai.004G075200.1      Gorai.004G218900.1
        .       .       .       .       .       .       .       .
        .       .       .       .       .       .
Vv07g0053       .       .       .       .       .       Gorai.
004G218800.1    .       .       .       .       .       .       .
        .       .       .       .       .       .       .
Vv07g0054       Tc01_g005950    .       .       .       .       .
        .       .       .       .       .       .       .       .
        .       .       .       .       .       .
Vv07g0055       .       .       .       .       .       .       .
        .       .       .       .       .       .       .       .
        .       .       .       .       .
Vv07g0056       .       Gorai.007G141500.1      .       .
Gorai.004G075400.1      Gorai.004G246700.1      .       .       .
        .       .       .       .       .       .       .       .
        .       .       .
Vv07g0057       .       .       .       .       .       .       .
        .       .       .       .       .       .       .       .
        .       .       .       .       .
Vv07g0058       .       .       .       .       .       .       .
        .       .       .       .       .       .       .       .
        .       .       .       .       .
Vv07g0059       .       .       .       .       .       .       .
        .       .       .       .       .       .       .       .
        .       .       .       .       .
Vv07g0060       .       .       .       .       .       .       .
        .       .       .       .       .       .       .       .
        .       .       .       .       .
Vv07g0061       .       Gorai.007G141400.1      .       .       .
        .       .       .       .       .       .       .       .
        .       .       .       .       .       .       .
Vv07g0062       .       .       .       .       .       .       .
        .       .       .       .       .       .       .       .
        .       .       .       .       .
Vv07g0063       .       Gorai.007G141200.1      Gorai.001G242400.1
```

. . . . . . . .
. . . . . . . .
.
Vv07g0064 . . . . . . .
. . . . . . . .
. . . . .
Vv07g0065 . . . . . . .
. . . . . . . .
. . . . .
Vv07g0066 . . . . . . .
. . . . . . . .
. . . . .
Vv07g0067 . . . . . . .
. . . . . . . .
. . . . .
Vv07g0068 . . . . . .
Vv05g0665 Tc04_g024460 Gorai.006G245000.1 .
Gorai.006G069800.1 Gorai.011G165200.1 . . .
. . . . .
Vv07g0069 . . . . . . .
. . . . . . . .
. . . . .
Vv07g0070 . . Gorai.001G242200.1 .
Gorai.004G075500.1 Gorai.004G218200.1 Vv05g0662 .
Gorai.006G244800.1 . . . . .
. . . . . .
Vv07g0071 . . . . . Gorai.
004G218100.1 Vv05g0661 . Gorai.006G244400.1 .
. Gorai.011G167200.1 . . . .
. . . .
Vv07g0072 . Gorai.007G141000.1 Gorai.001G242100.1
. . Gorai.004G244200.1 . . .
. . . . . . . .
. . .
Vv07g0073 . . Gorai.001G242000.1 . .
. . . . . . . .
. . . . . . .
Vv07g0074 . Gorai.007G140900.1 Gorai.001G241900.1
. Gorai.004G075700.1 Gorai.004G241600.1 .
. . . . . . . .
. . . . .
Vv07g0075 . Gorai.007G140600.1 . .
Gorai.004G076100.1 . . . . . .
. . . . . . . .
.
Vv07g0076 . Gorai.007G140400.1 . .
Gorai.004G076300.1 . Vv05g0654 . .
Gorai.005G046100.1 . Gorai.011G166800.1 . .
. . . . .
Vv07g0077 . . Gorai.001G239700.1 . .

Gorai.004G217800.1 . . . . .
. . . . . . . .
.
Vv07g0078 Tc01_g028530 Gorai.007G140300.1 . .
Gorai.004G076400.1 . . . . .
. . . . . . . .
. .
Vv07g0079 Tc01_g028520 . . . .
Gorai.004G217700.1 . . . . . .
. . . . . . . .
Vv07g0080 Tc01_g028510 . . . . .
. . . . . . . .
. . . . . .
Vv07g0081 Tc01_g028480 . . . . .
. . . . . . . .
. . . . . .
Vv07g0082 . Gorai.007G140000.1 . . .
. . . . . . . .
. . . . . . .
Vv07g0083 Tc01_g028470 . . . .
Gorai.004G217600.1 . . . . . .
. . . . . . . .
Vv07g0084 . . . . Gorai.004G056400.1
. . . . . . . .
. . . . . . .
Vv07g0085 Tc01_g028460 . . . . .
Vv05g0621 Tc04_g024420 Gorai.006G242900.1 .
. Gorai.011G168300.1 Gorai.002G024500.1 .
. . . . . .
Vv07g0086 Tc01_g028430 . . . Gorai.
004G076600.1 . . . . . . .
. Vv18g0502 Tc08_g008770 . .
Gorai.009G234900.1 . .
Vv07g0087 Tc01_g028410 Gorai.007G139800.1 . .
Gorai.004G076800.1 . . . . .
. . . Vv18g0477 Tc08_g008870 .
. . . Gorai.010G039600.1
Vv07g0088 Tc01_g028390 Gorai.007G139600.1 . .
Gorai.004G076900.1 . Vv05g0614
Tc04_g024340 Gorai.006G242400.1 . . Gorai.
011G168400.1 . . . . . . .
.
Vv07g0089 Tc01_g028380 . . . Gorai.
004G056100.1 . . . . . . .
. Vv18g0466 Tc08_g008980 . .
Gorai.009G236500.1 . Gorai.010G040100.1
Vv07g0090 . . . . . . .
. . . . . . . .
. . . . .
Vv07g0091 Tc01_g006240 . Gorai.008G080500.1 .

. . . . . . . .
. . . . . . . .
Vv07g0092 . . . . . . .
. . . . . . . .
. . . . .
Vv07g0093 Tc01_g028370 Gorai.007G139500.1 . .
Gorai.004G077000.1 . . . . .
. . . . . . . .
. .
Vv07g0094 Tc01_g028330 . . . Gorai.
004G077100.1 . Vv05g0610 Tc04_g024310 . .
. . . . . . . .
. .
Vv07g0095 . . . . . . .
. . . . . . . .
. . . . .
Vv07g0096 . . . . . . .
. . . . . . . .
. . . . .
Vv07g0097 . . . . Gorai.004G077300.1
. . . . . . . .
. . . . . . .
Vv07g0098 . . . . . . .
. . . . . . . .
. . . . .
Vv07g0099 . . . . . . .
. . . . . . . .
. . . . .
Vv07g0100 . . . . . . .
. . . . . . . .
. . . . .
Vv07g0101 . . . . . . .
. . . . . . . .
. . . . .
Vv07g0102 . . . . . . .
. . . . . . . .
. . . . .
Vv07g0103 . . . . . . .
. . . . . . . .
. . . . .
Vv07g0104 . . . . . . .
. . . . . . . .
. . . . .
Vv07g0105 . . . . . . .
. . . . . . . .
. . . . .
Vv07g0106 . . . . . . .
. . . . . . . .
. . . . .
Vv07g0107 . . . . . . .

```
. . . . . . . .
. . . . .
Vv07g0108 . . . . . . .
. . . . . . . .
. . . . .
Vv07g0109 . . . . . Gorai.
004G217500.1 . . . . . . .
. . . . . . .
Vv07g0110 Tc01_g028320 . . . . .
. . . . . . . .
. . . . . .
Vv07g0111 . . . . . . .
. . . . . . . .
. . . . .
Vv07g0112 . . . . . . .
. . . . . . . .
. . . . .
Vv07g0113 . . . . . .
Vv05g0608 Tc04_g024280 . . . Gorai.
011G170100.1 . . . . . . .
.
Vv07g0114 Tc01_g028290 . . . Gorai.
004G056000.1 Gorai.004G217400.1 Vv05g0607 . .
. . . . . . . .
. . .
Vv07g0115 Tc01_g028280 . . . Gorai.
004G077500.1 . Vv05g0606 Tc04_g024260 Gorai.
006G242000.1 Gorai.005G045200.1 . . . .
. . . . . .
Vv07g0116 Tc01_g028270 . . . . .
. . . . . . . .
. . . . . .
Vv07g0117 Tc01_g028260 . . . Gorai.
004G077600.1 . . . . . . .
. . . . . . . .
Vv07g0118 . . . . . .
Vv05g0602 Tc04_g024220 . . . Gorai.
011G167600.1 . . . . . . .
.
Vv07g0119 . . . . . . .
. . . . . . . .
. . . . .
Vv07g0120 . . . . . . .
. . . . . . . .
. . . . .
Vv07g0121 Tc01_g028250 Gorai.007G139400.1 . .
. . Vv05g0599 . . . .
. . . . . . . .
.
Vv07g0122 Tc01_g028240 Gorai.007G139300.1 . .
```

Gorai.004G077700.1 Gorai.004G217300.1 Vv05g0596
Tc04_g024180 Gorai.006G241600.1 . . .
. . . . . . . .
Vv07g0123 Tc01_g028230 . . . Gorai.
004G077800.1 . . . . . . .
. Vv18g0429 . . . . .
.
Vv07g0124 Tc01_g028220 Gorai.007G139200.1 . .
. . . . . . . .
. . . . . . . .
Vv07g0125 . . . . . . .
. . . . . . . .
. . . . .
Vv07g0126 Tc01_g028210 . . . Gorai.
004G078000.1 . Vv05g0593 Tc04_g024150 .
Gorai.005G044600.1 Gorai.006G067900.1 . . .
. . . . . .
Vv07g0127 . . . . . . .
. . . . . . . .
. . . . .
Vv07g0128 . . . . . . .
. . . . . . . .
. . . . .
Vv07g0129 . . . . . . .
. . . . . . . .
. . . . .
Vv07g0130 Tc01_g028180 Gorai.007G139100.1 . .
Gorai.004G078100.1 Gorai.004G217200.1 Vv05g0585
Tc04_g024100 Gorai.006G241200.1 . .
Gorai.011G165500.1 . . . . . .
. .
Vv07g0131 . . . . . . .
. . . . . . . .
. . . . .
Vv07g0132 . . . . . . .
. . . . . . . .
. . . . .
Vv07g0133 Tc01_g028140 . . . Gorai.
004G055800.1 . . . . . . .
. . . . . . . .
Vv07g0134 Tc01_g028120 . . . . .
. . . . . . . .
. . . . . .
Vv07g0135 . . . . . . .
. . . . . . . .
. . . . .
Vv07g0136 Tc01_g028110 Gorai.007G138900.1 . .
. . . . . . . .
. . . . . . . .
Vv07g0137 Tc01_g028090 . . . . .

```
         .       .       .       .       .       .       .       .
         .       .       .       .       .       .
Vv07g0138        Tc01_g028080    .       .       .       .       .
         Vv05g0583       Tc04_g024040    Gorai.006G240600.1      .
         .       .       .       .       .       .       .       .
         .       .
Vv07g0139        .       .       .       .       .       .       .
         .       .       .       .       .       .       .       .
         .       .       .       .       .
Vv07g0140        .       .       .       .       .       .       .
         .       .       .       .       .       .       .       .
         .       .       .       .       .
Vv07g0141        .       .       .       .       .       .       .
         .       .       .       .       .       .       .       .
         .       .       .       .       .
Vv07g0142        Tc01_g028070    .       .       .       .       .
         Vv05g0582       Tc04_g024030    Gorai.006G240500.1      .
         .       .       .       .       .       .       .       .
         .       .
Vv07g0143        Tc01_g028040    Gorai.007G138700.1      .       .
         .       .       .       .       .       .       .       .
         .       .       .       .       .       .       .       .
Vv07g0144        Tc01_g028030    Gorai.007G138600.1      .       .
         .       Gorai.004G216700.1      Vv05g0581
Tc04_g024020     Gorai.006G240400.1      .       .       .       .
         .       .       .       .       .       .       .
Vv07g0145        .       .       .       .       .       .       .
         .       .       .       .       .       .       .       .
         .       .       .       .       .
Vv07g0146        Tc01_g028010    .       .       .       .       .
         .       .       .       .       .       .       .       .
         .       .       .       .       .       .
Vv07g0147        .       .       .       .       .       .       .
         .       .       .       .       .       .       .       .
         .       .       .       .       .
Vv07g0148        .       .       .       .       .       .       .
         .       .       .       .       .       .       .       .
         .       .       .       .       .
Vv07g0149        .       .       .       .       .       .
Vv05g0567        Tc04_g023920    Gorai.006G239900.1      .       .
         .       Gorai.002G024300.1      .       .       .       .
         .       .       .
Vv07g0150        Tc01_g027970    Gorai.007G138200.1      .       .
         Gorai.004G078600.1      .       Vv05g0559
Tc04_g023830     .       Gorai.005G043200.1      .       .       .
         .       .       .       .       .       .       .
Vv07g0151        .       .       .       .       .       .       .
         .       .       .       .       .       .       .       .
         .       .       .       .       .
Vv07g0152        .       .       .       .       .       .
```

Vv05g0549 . . . . . . .
. . . . . .
Vv07g0153 . . . . . . .
. . . . . . . .
. . . . .
Vv07g0154 Tc01_g027910 . . . Gorai.
004G078800.1 . Vv05g0546 Tc04_g023730 Gorai.
006G239000.1 Gorai.005G042800.1 . . . .
. . . . . .
Vv07g0155 . . . . . . .
. . . . . . . .
. . . . .
Vv07g0156 . . . . . .
Vv05g0540 Tc04_g023640 Gorai.006G238700.1 Gorai.
005G042300.1 . . . . . . .
. . .
Vv07g0157 Tc01_g027890 . . . . .
. . . . . . . .
. . . . . .
Vv07g0158 . . . . . . .
. . . . . . . .
. . . . .
Vv07g0159 Tc01_g027870 Gorai.007G137800.1 . .
Gorai.004G079100.1 . Vv05g0538 . .
. . . . . . . .
. . .
Vv07g0160 Tc01_g027860 Gorai.007G137700.1 . .
. . . . . . . .
. . . . . . . .
Vv07g0161 Tc01_g027850 . . . Gorai.
004G055700.1 . . . . . . .
. . . . . . . .
Vv07g0162 Tc01_g027840 Gorai.007G137600.1 . .
. Gorai.004G216500.1 . . . .
. . . Vv18g0428 Tc08_g009450 .
. . Gorai.009G282300.1 .
Vv07g0163 Tc01_g027810 . . . Gorai.
004G079200.1 . Vv05g0520 Tc04_g023500 .
Gorai.005G042000.1 . . . . . .
. . . .
Vv07g0164 Tc01_g027800 . . . Gorai.
004G079300.1 Gorai.004G216400.1 . . . .
. . . . . . . .
. .
Vv07g0165 Tc01_g027790 . . . Gorai.
004G079400.1 Gorai.004G216300.1 . . . .
. . . . . . . .
. .
Vv07g0166 Tc01_g027780 Gorai.007G137400.1 . .
Gorai.004G079500.1 . . . . .

. . . . . . . .
. .
Vv07g0167 Tc01_g027750 . . . .
Gorai.004G216200.1 . . . . . .
. . . . . . . .
Vv07g0168 . . . . . . .
. . . . . . . .
. . . . .
Vv07g0169 . . . . . . .
. . . . . . . .
. . . . .
Vv07g0170 Tc01_g027740 . . . . .
Vv05g0516 Tc04_g023460 Gorai.006G237700.1 .
. . . . . . . .
. .
Vv07g0171 . . . . . . .
. . . . . . . .
. . . . .
Vv07g0172 Tc01_g027640 Gorai.007G137200.1 . .
Gorai.004G079700.1 . Vv05g0514
Tc04_g023440 Gorai.006G237500.1 . . Gorai.
011G169100.1 . . . . . . .
.
Vv07g0173 Tc01_g027620 Gorai.007G137100.1 . .
. Gorai.004G216100.1 . . . .
. . . . . . . .
. .
Vv07g0174 . . . . . . .
. . . . . . . .
. . . . .
Vv07g0175 . . . . . . .
. . . . . . . .
. . . . .
Vv07g0176 Tc01_g027610 . . . . .
. . . . . . . .
. . . . . .
Vv07g0177 Tc01_g027460 . . . . .
. . . . . . . .
. . . . . .
Vv07g0178 Tc01_g027450 . . . Gorai.
004G081100.1 . . . . . . .
. . . . . . . .
Vv07g0179 Tc01_g027440 Gorai.007G136500.1 . .
Gorai.004G081200.1 . . . . . .
. . . . . . . .
. .
Vv07g0180 . . . . . . .
. . . . . . . .
. . . . .
Vv07g0181 Tc01_g027430 Gorai.007G136400.1 . .

. Gorai.004G215900.1 . . . .
. . . . . . . .
. .
Vv07g0182 Tc01_g027400 Gorai.007G136100.1 . .
Gorai.004G081400.1 . . . . .
. . . . . . . .
. .
Vv07g0183 . . . . . .
Vv05g0478 Tc04_g023070 . Gorai.005G041100.1 .
. . . . . . . .
.
Vv07g0184 . Gorai.007G135800.1 . . .
. . . . . . . .
. . . . . . .
Vv07g0185 . . . . . . .
. . . . . . . .
. . . . .
Vv07g0186 . . . . . Gorai.
004G242500.1 Vv05g0468 Tc04_g022990 . . .
. . Vv18g0419 Tc08_g009580 . .
. . .
Vv07g0187 . . . . Gorai.004G081800.1
Gorai.004G242400.1 Vv05g0467 Tc04_g022970 .
. . . . . . . .
. . .
Vv07g0188 . . . . Gorai.004G081900.1
. . . . . . . .
. . . . . . .
Vv07g0189 . . . . . . .
. . . . . . . .
. . . . .
Vv07g0190 Tc01_g028660 . . . Gorai.
004G082000.1 . . . . . . .
. . . . . . . .
Vv07g0191 . . . . . . .
. . . . . . . .
. . . . .
Vv07g0192 Tc01_g028670 . . . Gorai.
004G082200.1 . . . . . . .
. . . . . . . .
Vv07g0193 . . . . . . .
. . . . . . . .
. . . . .
Vv07g0194 Tc01_g028680 . . . Gorai.
004G082300.1 . . . . . . .
. . . . . . . .
Vv07g0195 . . . . . . .
. . . . . . . .
. . . . .
Vv07g0196 . Gorai.007G135500.1 . . .

. Vv05g0461 Tc04_g022870 Gorai.006G217400.1
. Gorai.006G053600.1 . . . .
. . . . .
Vv07g0197 Tc01_g028710 Gorai.007G135400.1 . .
Gorai.004G082600.1 . Vv05g0456 . .
. . . . . . . .
. . .
Vv07g0198 . . . . . . .
. . . . . . . .
. . . . .
Vv07g0199 Tc01_g028730 . . . Gorai.
004G055400.1 . Vv05g0454 Tc04_g022810 .
Gorai.005G040600.1 . Gorai.011G170300.1 . .
. . . . . .
Vv07g0200 . . . . . . .
. . . . . . . .
. . . . .
Vv07g0201 . . . . . . .
. . . . . . . .
. . . . .
Vv07g0202 Tc01_g028740 . . . .
Gorai.004G215600.1 . . . . . .
. . . . . . . .
Vv07g0203 Tc01_g028750 . . . Gorai.
004G083200.1 Gorai.004G215500.1 . . . .
. . . . . . . .
. .
Vv07g0204 . Gorai.007G135000.1 . . .
. . . . . . . .
. . . . . . .
Vv07g0205 Tc01_g028760 Gorai.007G134800.1 . .
. . . . . . . .
. . . . . . . .
Vv07g0206 Tc01_g028790 . . . .
Gorai.004G215400.1 . . . . . .
. . . . . . . .
Vv07g0207 Tc01_g028800 Gorai.007G134700.1 . .
Gorai.004G055300.1 . . . . .
. . . . . . . .
. .
Vv07g0208 Tc01_g028810 Gorai.007G134600.1 . .
. . . . . . . .
. . . . . . . .
Vv07g0209 Tc01_g028820 . . . Gorai.
004G083400.1 . . . . . . .
. . . . . . . .
Vv07g0210 Tc01_g028830 Gorai.007G134500.1 . .
Gorai.004G055200.1 . Vv05g0450
Tc04_g022760 . Gorai.005G040400.1 . . .
. . . . . . .

Vv07g0211 Tc01_g028840 Gorai.007G134400.1 . .
. . . . . . . .
. . . . . . . .
Vv07g0212 . . . . . . .
. . . . . . . .
. . . . .
Vv07g0213 . . . . . . .
. . . . . . . .
. . . . .
Vv07g0214 . . . . . . .
. . . . . . . .
. . . . .
Vv07g0215 . . . . . .
Vv05g0448 Tc04_g022730 . Gorai.005G040200.1 .
. . . . . . . .
.
Vv07g0216 . . . . . .
Vv05g0447 Tc04_g022720 . Gorai.005G040100.1 .
. . . . . . . .
.
Vv07g0217 . . . . . . .
. . . . . . . .
. . . . .
Vv07g0218 . . . . . Gorai.
004G241600.1 . . . . . . .
Vv14g0364 . . . . Gorai.
007G342600.1 .
Vv07g0219 . . . . . . .
. . . . . . . .
. . . . .
Vv07g0220 . . . . . . .
. . . . . . . .
. . . . .
Vv07g0221 . . . . . . .
. . . . . . . .
. . . . .
Vv07g0222 . . . . . . .
. . . . . . . .
. . . . .
Vv07g0223 . . . . . . .
. . . . . . . .
. . . . .
Vv07g0224 . . . . . . .
. . . . . . . .
. . . . .
Vv07g0225 . . . . . . .
. . . . . . . .
. . . . .
Vv07g0226 Tc01_g028860 Gorai.007G134200.1 . .
Gorai.004G083500.1 . . . . .

. . . . . . . .
. .
Vv07g0227 Tc01_g028870 Gorai.007G134100.1 . .
Gorai.004G055100.1 Gorai.004G215300.1 Vv05g0444
Tc04_g022670 . . . . . .
. . . . . .
Vv07g0228 . . . . . . .
. . . . . . . .
. . . . .
Vv07g0229 . . . . . . .
. . . . . . . .
. . . . .
Vv07g0230 . . . . . . .
. . . . . . . .
. . . . .
Vv07g0231 . . . . . . .
. . . . . . . .
. . . . .
Vv07g0232 Tc01_g028880 Gorai.007G133800.1 . .
. . . . . . . .
. . . . . . . .
Vv07g0233 Tc01_g028890 . . . . .
. . . . . . . .
. . . . . .
Vv07g0234 . . . . . .
Vv05g0440 Tc04_g022630 Gorai.006G218100.1 . .
. . . . . . . .
.
Vv07g0235 Tc01_g028910 . . . . .
. . . . . . . .
. . . . . .
Vv07g0236 Tc01_g028920 Gorai.007G133700.1 . .
Gorai.004G054800.1 . . . . .
. . . Vv14g0389 Tc04_g025620 .
. . Gorai.006G053800.1 .
Vv07g0237 Tc01_g028940 Gorai.007G133500.1 . .
Gorai.004G083800.1 . . . . .
. . . . . . . .
. .
Vv07g0238 Tc01_g028950 . . . Gorai.
004G083900.1 . Vv05g0435 . . . .
. . . . . . . .
.
Vv07g0239 Tc01_g029000 Gorai.007G133400.1 . .
. . Vv05g0431 . . . .
Gorai.011G181200.1 . . . . .
. . .
Vv07g0240 . . . . . . .
. . . . . . . .
. . . . .

Vv07g0241 . . . . . . .
. . . . . . . .
. . . . .
Vv07g0242 Tc01_g029020 . . . . .
. . . . . . . .
. . . . . .
Vv07g0243 . . . . . . .
. . . . . . . .
. . . . .
Vv07g0244 Tc01_g029030 . . . Gorai.
004G084100.1 . . . . . . .
. . . . . . . .
Vv07g0245 Tc01_g029040 . . . Gorai.
004G084200.1 . . . . . . .
. . . . . . . .
Vv07g0246 Tc01_g029050 Gorai.007G133300.1 . .
. Gorai.004G215100.1 . . . .
. . . Vv14g0416 . Gorai.
013G123400.1 . Gorai.007G133300.1 . Gorai.
004G215100.1
Vv07g0247 . . . . . . .
. . . . . . . .
. . . . .
Vv07g0248 . . . . . . .
. . . . . . . .
. . . . .
Vv07g0249 . . . . . . .
. . . . . . . .
. . . . .
Vv07g0250 Tc01_g029110 Gorai.007G133200.1 . .
. Gorai.004G215000.1 Vv05g0422 . .
. . Gorai.011G182000.1 . . .
. . . . .
Vv07g0251 Tc01_g029120 . . . .
Gorai.004G214900.1 . . . . . .
. . . . . . . .
Vv07g0252 . . . . . . .
. . . . . . . .
. . . . .
Vv07g0253 . . . . . . .
. . . . . . . .
. . . . .
Vv07g0254 . . . . . . .
. . . . . . . .
. . . . .
Vv07g0255 Tc01_g029130 . . . Gorai.
004G084300.1 . . . . . . .
. . . . . . . .
Vv07g0256 Tc01_g029150 . . . . .
. . . . . . . .

```
        .       .       .       .       .       .
Vv07g0257       Tc01_g029160    .       .       .       .       .
        .       .       .       .       .       .       .       .
        .       .       .       .       .       .
Vv07g0258       Tc01_g029170    .       .       .       .       .
        .       .       .       .       .       .       .
Vv14g0440       .       .       Gorai.007G291800.1      Gorai.
013G203700.1    .       .
Vv07g0259       Tc01_g029180    Gorai.007G132900.1      .       .
        .       .       Vv05g0414       .       Gorai.006G256600.1
        .       .       Gorai.011G182900.1      .       Vv14g0441
        Tc04_g027120    .       Gorai.007G291700.1      Gorai.
007G132900.1    .       .
Vv07g0260       Tc01_g029190    .       .       .       Gorai.
004G084400.1    .       .       .       .       .       .       .
        .       .       .       .       .       .       .       .
Vv07g0261       Tc01_g029200    .       .       .       Gorai.
004G084500.1    .       .       .       .       .       .       .
        .       .       .       .       .       .       .       .
Vv07g0262       .       .       .       .       .       .       .
        .       .       .       .       .       .       .       .
        .       .       .       .       .
Vv07g0263       Tc01_g029210    .       .       .       Gorai.
004G084600.1    .       .       .       .       .       .       .
        .       .       .       .       .       .       .       .
Vv07g0264       Tc01_g029220    Gorai.007G132800.1      .       .
        .       Gorai.004G214300.1      .       .       .       .
        .       .       .       Vv14g0444       .       .       .
        Gorai.007G132800.1      .       Gorai.004G214300.1
Vv07g0265       Tc01_g029240    .       .       .       .
Gorai.004G214200.1      .       .       .       .       .       .
        .       .       .       .       .       .       .       .
Vv07g0266       .       .       .       .       Gorai.004G084700.1
        Gorai.004G214100.1      .       .       .       .       .
        .       .       .       .       .       .       .       .
        .
Vv07g0267       Tc01_g029250    .       .       .       .
Gorai.004G239900.1      .       .       .       .       .       .
        .       Vv14g0446       .       .       Gorai.007G291200.1
        .       .       Gorai.004G214100.1
Vv07g0268       .       .       .       .       .       .       .
        .       .       .       .       .       .       .       .
        .       .       .       .       .
Vv07g0269       .       .       .       .       .       .       .
        .       .       .       .       .       .       .       .
        .       .       .       .       .
Vv07g0270       .       .       .       .       .       .       .
        .       .       .       .       .       .       .       .
        .       .       .       .       .
Vv07g0271       .       .       .       .       .       .       .
```

. . . . . . . .
. . . . .
Vv07g0272 Tc01_g029280 . . . . .
. . . . . . . .
. . . . . .
Vv07g0273 . Gorai.007G132700.1 . .
Gorai.004G054500.1 Gorai.004G239200.1 . . .
. . . . . . . .
. . .
Vv07g0274 . . . . . . .
. . . . . . . .
. . . . .
Vv07g0275 . . . . . . .
. . . . . . . .
. . . . .
Vv07g0276 Tc01_g029300 . . . Gorai.
004G054300.1 . . . . . . .
. . . . . . . .
Vv07g0277 Tc01_g029310 Gorai.007G132500.1 . .
. . . . . . . .
. . . . . . . .
Vv07g0278 Tc01_g029320 . . . .
Gorai.004G139800.1 . . . . . .
. . . . . . . .
Vv07g0279 Tc01_g029350 . . . . .
. . . . . . . .
. . . . . .
Vv07g0280 Tc01_g029360 Gorai.007G132400.1 . .
Gorai.004G084800.1 Gorai.004G140000.1 . .
. . . . . . . .
. . . .
Vv07g0281 . . . . . . .
. . . . . . . .
. . . . .
Vv07g0282 Tc01_g029380 Gorai.007G132300.1 . .
. . . . . . . .
. . . . . . . .
Vv07g0283 Tc01_g029390 . . . Gorai.
004G054200.1 . . . . . . .
. . . . . . . .
Vv07g0284 . . . . . . .
. . . . . . . .
. . . . .
Vv07g0285 . . . . . . .
. . . . . . Vv14g0471
. Gorai.013G116000.1 . . . .
Vv07g0286 . . . . . . .
. . . . . . . .
. . . . .
Vv07g0287 Tc01_g029430 . . . Gorai.

004G085300.1 . . . . . . .
. . . . . . . .
Vv07g0288 Tc01_g029450 Gorai.007G131900.1 . .
Gorai.004G085400.1 . . . . .
. . . . . . . .
. .
Vv07g0289 Tc01_g029510 . . . . .
. . . . . . . .
. . . . . .
Vv07g0290 . . . . . Gorai.
004G140200.1 . . . . . . .
. . . . . . .
Vv07g0291 Tc01_g029530 . . . Gorai.
004G085500.1 . . . . . . .
. . . . . . . .
Vv07g0292 . . . . . . .
. . . . . . . .
. . . . .
Vv07g0293 Tc01_g029550 Gorai.007G131700.1 . .
Gorai.004G085700.1 Gorai.004G140400.1 . .
. . . . . . . .
. . . .
Vv07g0294 Tc01_g029560 . . . . .
Vv05g0389 . Gorai.006G257200.1 Gorai.
005G057100.1 Gorai.006G075200.1 . . Vv14g0482
. . . . . .
Vv07g0295 Tc01_g029580 Gorai.007G131500.1 . .
Gorai.004G085800.1 . Vv05g0385 . .
Gorai.005G057200.1 Gorai.006G075400.1 Gorai.
011G185600.1 . . . . . . .
.
Vv07g0296 . . . . . . .
. . . . . . . .
. . . . .
Vv07g0297 . . . . . . .
. . . . . . . .
. . . . .
Vv07g0298 Tc01_g029600 Gorai.007G131300.1 . .
Gorai.004G053500.1 Gorai.004G140500.1 . .
. . . . . . . .
. . . .
Vv07g0299 Tc01_g029630 Gorai.007G131200.1 . .
. . . . . . . .
. . . . . . . .
Vv07g0300 Tc01_g029640 . . . Gorai.
004G086000.1 . . . . . . .
. . . . . . . .
Vv07g0301 Tc01_g029680 Gorai.007G131100.1 . .
. . Vv05g0373 . Gorai.006G257500.1
Gorai.005G057400.1 . . . . .

. . . . .
Vv07g0302 Tc01_g029690 . . . .
Gorai.004G140600.1 . . . . . .
. . . . . . . .
Vv07g0303 Tc01_g029700 . . . Gorai.
004G086200.1 . Vv05g0370 . . Gorai.
005G057600.1 . Gorai.011G186700.1 . . .
. . . . .
Vv07g0304 . . . . . . .
. . . . . . . .
. . . . .
Vv07g0305 . . . . . . .
. . . . . . . .
. . . . .
Vv07g0306 Tc01_g029720 Gorai.007G130900.1 . .
. Gorai.004G140800.1 Vv05g0366
Tc04_g001350 . Gorai.005G058500.1 . Gorai.
011G187200.1 . . . . . . .
.
Vv07g0307 Tc01_g029730 Gorai.007G130800.1 . .
Gorai.004G086400.1 Gorai.004G140900.1 Vv05g0365
. . Gorai.005G058600.1 . Gorai.
011G187300.1 . . . . . . .
.
Vv07g0308 Tc01_g029740 . . . .
Gorai.004G141000.1 . . . . . .
. . . . . . . .
Vv07g0309 Tc01_g029770 Gorai.007G130700.1 . .
Gorai.004G086500.1 Gorai.004G141100.1 . .
. . . . . . . .
. . . .
Vv07g0310 . . . . . . .
. . . . . . . .
. . . . .
Vv07g0311 Tc01_g029790 Gorai.007G130600.1 . .
Gorai.004G086600.1 . . . . .
. . . . . . . .
. .
Vv07g0312 Tc01_g029810 Gorai.007G130500.1 . .
Gorai.004G053200.1 . Vv05g0355 . .
Gorai.005G058800.1 . Gorai.011G188400.1
Gorai.002G027000.1 . . . . . .
.
Vv07g0313 Tc01_g029830 . . . .
Gorai.004G236800.1 . . . . . .
. . . . . . . .
Vv07g0314 Tc01_g029840 Gorai.007G130400.1 . .
. . . . . . . .
. . . . . . . .
Vv07g0315 . . . . . Gorai.

004G233900.1 . . . . . . .
. . . . . . .
Vv07g0316 . . . . . . .
. . . . . . . .
. . . . .
Vv07g0317 Tc01_g029850 . . . .
Gorai.004G141300.1 . . . . . .
. . . . . . . .
Vv07g0318 . . . . . . .
. . . . . . . .
. . . . .
Vv07g0319 . . . . . . .
. . . . . . . .
. . . . .
Vv07g0320 . . . . . . .
. . . . . . . .
. . . . .
Vv07g0321 . . . . . . .
. . . . . . . .
. . . . .
Vv07g0322 . . . . . . .
. . . . . . . .
. . . . .
Vv07g0323 . . . . . . .
. . . . . . . .
. . . . .
Vv07g0324 Tc01_g029880 . . . Gorai.
004G088000.1 Gorai.004G141400.1 . . . .
. . . . . . . .
. .
Vv07g0325 . Gorai.007G130300.1 . .
Gorai.004G053100.1 . . . . . .
. . Vv14g0314 . . .
Gorai.007G130300.1 . .
Vv07g0326 . . . . . . .
. . . . . . . .
. . . . .
Vv07g0327 . . . . . . .
. . . . . . . .
. . . . .
Vv07g0328 Tc01_g029920 Gorai.007G130100.1 . .
Gorai.004G088400.1 . . . . .
. . . Vv14g0268 . .
Gorai.007G298600.1 . . .
Vv07g0329 Tc01_g029930 Gorai.007G130000.1 . .
Gorai.004G053000.1 . . . . .
. . . Vv14g0286 Tc04_g027920
Gorai.013G124900.1 . . . .
Vv07g0330 Tc01_g029960 Gorai.007G129900.1 . .
. Gorai.004G141600.1 . . . .

| | | | | | | | |
|---|---|---|---|---|---|---|---|
| . | . | . | Vv14g0288 | | Tc04_g027900 | | . |
| . | Gorai.007G129900.1 | | | Gorai.011G189100.1 | | | . |
| Vv07g0331 | . | . | . | . | . | . | . |
| . | . | . | . | . | . | . | . |
| . | . | . | . | . | | | |
| Vv07g0332 | Tc01_g029970 | | Gorai.007G129800.1 | | | . | . |
| . | . | . | . | . | . | . | . |
| . | . | . | . | . | . | . | . |
| Vv07g0333 | . | . | . | . | . | . | . |
| . | . | . | . | . | . | . | . |
| . | . | . | . | . | | | |
| Vv07g0334 | Tc01_g029980 | | . | . | . | . | . |
| . | . | . | . | . | . | . | . |
| . | . | . | . | . | . | | |
| Vv07g0335 | . | . | . | . | . | . | . |
| . | . | . | . | . | . | . | . |
| . | . | . | . | . | | | |
| Vv07g0336 | . | . | . | . | . | . | . |
| . | . | . | . | . | . | . | . |
| . | . | . | . | . | | | |
| Vv07g0337 | . | . | . | . | . | . | |
| Vv05g0350 | . | . | . | . | . | . | . |
| . | . | . | . | . | . | | |
| Vv07g0338 | . | . | . | . | . | . | . |
| . | . | . | . | . | . | . | . |
| . | . | . | . | . | | | |
| Vv07g0339 | . | Gorai.007G129700.1 | | | . | . | . |
| . | . | . | . | . | . | . | . |
| . | . | . | . | . | . | . | |
| Vv07g0340 | Tc01_g029990 | | . | . | . | . | . |
| . | . | . | . | . | . | . | . |
| . | . | . | . | . | . | | |
| Vv07g0341 | . | . | . | . | . | . | . |
| . | . | . | . | . | . | . | . |
| . | . | . | . | . | | | |
| Vv07g0342 | Tc01_g030000 | | . | . | . | . | . |
| . | . | . | . | . | . | . | . |
| . | . | . | . | . | . | | |
| Vv07g0343 | Tc01_g030030 | | . | . | . | . | . |
| . | . | . | . | . | . | . | . |
| . | . | . | . | . | . | | |
| Vv07g0344 | . | . | . | . | . | . | . |
| . | . | . | . | . | . | . | . |
| . | . | . | . | . | | | |
| Vv07g0345 | Tc01_g030060 | | Gorai.007G129500.1 | | | . | . |
| . | . | . | . | . | . | . | . |
| . | Vv14g0261 | | Tc05_g028810 | | Gorai.013G125200.1 | | |
| . | Gorai.007G129500.1 | | | . | . | | |
| Vv07g0346 | . | . | . | . | . | . | . |
| . | . | . | . | . | . | . | . |

. . . . .
Vv07g0347 . . . . . . .
. . . . . . . .
. . . . .
Vv07g0348 Tc01_g030070 Gorai.007G129400.1 . .
. Gorai.004G141900.1 . . . .
. . . . . . . .
. .
Vv07g0349 Tc01_g030080 . . . . .
. . . . . . . .
. . . . . .
Vv07g0350 Tc01_g030090 . . . . .
. . . . . . . .
. . . . . .
Vv07g0351 Tc01_g030100 . . . . .
. . . . . . . .
. . . . . .
Vv07g0352 Tc01_g030110 . . . . .
. . . . . . . .
. . . . . .
Vv07g0353 Tc01_g030120 . . . Gorai.
004G088700.1 . . . . . . .
. . . . . . . .
Vv07g0354 . . . . . . .
. . . . . . . .
. . . . .
Vv07g0355 . . . . . . .
. . . . . . . .
. . . . .
Vv07g0356 . . . . . . .
. . . . . . . .
. . . . .
Vv07g0357 . . . . . . .
. . . . . . . .
. . . . .
Vv07g0358 Tc01_g030130 . . . . .
. . . . . . . .
. . . . . .
Vv07g0359 Tc01_g030140 . . . Gorai.
004G088800.1 . . . . . . .
. . . . . . . .
Vv07g0360 Tc01_g030170 . . . . .
. . . . . . . .
. . . . . .
Vv07g0361 Tc01_g030190 Gorai.007G129300.1 . .
Gorai.004G052900.1 . . . . .
. . . . . . . .
. .
Vv07g0362 Tc01_g030220 Gorai.007G129200.1 . .
. . . . . . . .

```
        .        .        .       .        .       .        .       .
Vv07g0363        Tc01_g030230     Gorai.007G129100.1        .       .
        .        .        .       .        .       .        .       .
        .        .        .       .        .       .        .       .
Vv07g0364        Tc01_g030240     Gorai.007G129000.1        .       .
        Gorai.004G052800.1        Gorai.004G142100.1        Vv05g0337
        .        .        .       .        .       Gorai.002G028300.1
        .        .        .       .        .       .        .
Vv07g0365        .        .       .        .       .        .       .
        .        .        .       .        .       .        .       .
        .        .        .       .        .
Vv07g0366        Tc01_g030260     .        .       .        .       .
        .        .        .       .        .       .        .       .
        .        .        .       .        .       .
Vv07g0367        Tc01_g030280     .        .       .        .       .
        .        .        .       .        .       .        .       .
        .        .        .       .        .       .
Vv07g0368        Tc01_g030290     Gorai.007G128800.1        .       .
        Gorai.004G052700.1        .        .       .        .       .
        .        .        .       .        .       .        .       .
        .        .
Vv07g0369        Tc01_g030300     .        .       .        .       .
        .        .        .       .        .       .        .       .
        .        .        .       .        .       .
Vv07g0370        Tc01_g030310     .        Gorai.008G146600.1       .
        .        .        .       .        .       .        .       .
        .        .        .       .        .       .        .       .
Vv07g0371        .        .       .        .       .        .
Vv05g0320        .        .       .        .       Gorai.011G190500.1
        .        .        .       .        .       .        .       .
Vv07g0372        Tc01_g030320     .        Gorai.008G146500.1       .
        .        .        .       .        .       .        .       .
        .        .        .       .        .       .        .       .
Vv07g0373        .        .       .        .       .        .       .
        .        .        .       .        .       .        .       .
        .        .        .       .        .
Vv07g0374        Tc01_g030330     .        Gorai.008G146400.1       .
        Gorai.004G052600.1        .        .       .        .       .
        .        .        .       .        .       .        .       .
        .        .
Vv07g0375        Tc01_g030340     .        Gorai.008G146300.1       .
        .        .        .       .        .       .        .       .
        .        .        .       .        .       .        .       .
Vv07g0376        .        .       .        .       .        .       .
        .        .        .       .        .       .        .       .
        .        .        .       .        .
Vv07g0377        Tc01_g030370     .        Gorai.008G146200.1       .
        Gorai.004G052500.1        .        .       .        .       .
        .        .        .       Vv14g0241        .        .       .
        .        .        .
```

Vv07g0378 Tc01_g030380 . Gorai.008G146100.1 .
. . . . . . . .
. . . . . . . .
Vv07g0379 Tc01_g030390 . Gorai.008G146000.1 .
Gorai.004G052400.1 . . . . .
. . . . . . . .
. .
Vv07g0380 . . . . . . .
. . . . . . . .
. . . . .
Vv07g0381 Tc01_g030410 . Gorai.008G145800.1 .
Gorai.004G089200.1 . . . . .
. . . . . . . .
. .
Vv07g0382 Tc01_g030420 . . . Gorai.
004G089300.1 . . . . . . .
. . . . . . . .
Vv07g0383 Tc01_g030430 Gorai.007G128500.1 Gorai.
008G145700.1 . . . . . . .
. . . . . . . .
. .
Vv07g0384 . . . . . . .
. . . . . . . .
. . . . .
Vv07g0385 . . . . . . .
. . . . . . . .
. . . . .
Vv07g0386 Tc01_g030440 . . . Gorai.
004G089400.1 . Vv05g0313 Tc04_g001060 . .
. . . . . . . .
. .
Vv07g0387 . . . . . . .
. . . . . . . .
. . . . .
Vv07g0388 . . . . . . .
. . . . . . . .
. . . . .
Vv07g0389 . . . . . . .
. . . . . . . .
. . . . .
Vv07g0390 . Gorai.007G128300.1 . . .
. . . . . . . .
. . . . . . .
Vv07g0391 . . . . Gorai.004G089600.1
. . . . . . . .
. . . . . . .
Vv07g0392 . . . . . .
Vv013rg0130 . . . . . . .
. . . . . .
Vv07g0393 Tc01_g030460 . . . . .

Vv013rg0131 . . . . . .
. . . . . . .
Vv07g0394 Tc01_g030470 . . . . .
Vv013rg0132 . . . . . .
. . . . . . .
Vv07g0395 Tc01_g030480 . . . . .
Vv013rg0134 . . . . . .
. . . . . . .
Vv07g0396 Tc01_g030490 . . . . .
Vv013rg0135 . . . . . .
. . . . . . .
Vv07g0397 . . . . . . .
. . . . . . . .
. . . . .
Vv07g0398 . . . . . .
Vv013rg0137 . . . . . . .
. . . . . .
Vv07g0399 . . . . . .
Vv013rg0138 . . . . . . .
. . . . . .
Vv07g0400 . . . . . Gorai.
004G142300.1 Vv05g0310 . . . .
Gorai.011G191000.1 . . . . . .
. .
Vv07g0401 . . . . . . .
. . . . . . . .
. . . . .
Vv07g0402 Tc01_g030540 . . . Gorai.
004G089800.1 . . . . . . .
. . . . . . . .
Vv07g0403 . . . . . . .
. . . . . . . .
. . . . .
Vv07g0404 Tc01_g030560 . . . .
Gorai.004G142400.1 . . . . . .
. . . . . . . .
Vv07g0405 Tc01_g030570 . Gorai.008G145300.1 .
. . . . . . . .
. . . . . . . .
Vv07g0406 Tc01_g030580 Gorai.007G128200.1 Gorai.
008G145200.1 . Gorai.004G090000.1 . Vv05g0009
Tc04_g028300 . Gorai.005G061000.1 . .
. . . . . . . .
Vv07g0407 . . . . . . .
. . . . . . . .
. . . . .
Vv07g0408 Tc01_g030590 Gorai.007G128100.1 Gorai.
008G055800.1 . Gorai.004G090200.1 Gorai.004G142500.1
. . . . . . . .
. . . . . .

Vv07g0409 Tc01_g030600 . . . . .
. . . . . . . .
. . . . . .
Vv07g0410 . . . . . . .
. . . . . . . .
. . . . .
Vv07g0411 . . . . . . .
. . . . . . . .
. . . . .
Vv07g0412 . . . . . . .
. . . . . . . .
. . . . .
Vv07g0413 Tc01_g030610 Gorai.007G128000.1 . .
Gorai.004G090300.1 . Vv05g0010
Tc04_g028320 . . . . . Vv14g0219
Tc04_g028320 . Gorai.007G300700.1 Gorai.
007G128000.1 . .
Vv07g0414 Tc01_g030620 . . . . .
. . . . . . . .
. . . . . .
Vv07g0415 Tc01_g030630 Gorai.007G127900.1 . .
. . . . . . . .
. . . . . . . .
Vv07g0416 Tc01_g030640 Gorai.007G127800.1 . .
. . . . . . . .
. . . . . . . .
Vv07g0417 Tc01_g030650 . . . Gorai.
004G090400.1 . . . . . . .
. . . . . . . .
Vv07g0418 Tc01_g030660 Gorai.007G127700.1 . .
Gorai.004G090500.1 . . . . .
. . . . . . . .
. .
Vv07g0419 Tc01_g030670 . . . . .
. . . . . . . .
. . . . . .
Vv07g0420 Tc01_g030680 Gorai.007G127600.1 . .
. . . . . . . .
. . . . . . . .
Vv07g0421 Tc01_g030690 . . . . .
. . . . . . . .
. . . . . .
Vv07g0422 Tc01_g030700 . . . Gorai.
004G090600.1 . . . . . . .
. . . . . . . .
Vv07g0423 . . . . . . .
. . . . . . . .
. . . . .
Vv07g0424 Tc01_g030720 . . . . .
. . . . . . . .

. . . . . .
Vv07g0425 Tc01_g030760 Gorai.007G127500.1 . .
Gorai.004G091000.1 Gorai.004G239100.1 . .
. . . . . . . .
. . . .
Vv07g0426 Tc01_g030770 Gorai.007G127400.1 . .
. . . . . . . .
. . . . . . . .
Vv07g0427 . . . . . . .
. . . . . . . .
. . . . .
Vv07g0428 Tc01_g030790 Gorai.007G127300.1 . .
Gorai.004G052100.1 Gorai.004G239000.1 Vv05g0028
. . . . . . . .
. . . . .
Vv07g0429 Tc01_g030800 . . . Gorai.
004G091300.1 . Vv05g0032 Tc04_g028440 . .
. . . . . . . .
. .
Vv07g0430 Tc01_g030830 Gorai.007G127200.1 . .
Gorai.004G091600.1 . . . . .
. . . . . . . .
. .
Vv07g0431 Tc01_g030860 . . . . .
. . . . . . .
Vv14g0212 . . . . . .
Vv07g0432 Tc01_g030870 . . . . .
Vv05g0038 Tc04_g028490 Gorai.006G260900.1 .
. . . Vv14g0211 Tc04_g028490 .
. . . .
Vv07g0433 Tc01_g030880 Gorai.007G126900.1 . .
. . . . . . . .
. . . . . . . .
Vv07g0434 Tc01_g030890 . . . .
Gorai.004G142700.1 Vv05g0041 Tc04_g028520 . .
. . . . . . . .
. .
Vv07g0435 Tc01_g030910 . . . . .
Vv05g0045 Tc04_g028580 Gorai.006G260500.1
Gorai.005G061900.1 . Gorai.011G193300.1 . .
. . . . . .
Vv07g0436 . . . . . . .
. . . . . . . .
. . . . .
Vv07g0437 Tc01_g030930 . . . Gorai.
004G051900.1 . . . . . . .
. . . . . . . .
Vv07g0438 . . . . Gorai.004G051700.1
. . . . . . . .
. . . . . . .

```
Vv07g0439       Tc01_g031000    .       .       .       Gorai.
004G051600.1    Gorai.004G142800.1      .       .       .       .
        .       .       .       .       .       .       .       .
        .       .
Vv07g0440       .       .       .       .       .       .       .
        .       .       .       .       .       .       .       .
        .       .       .       .       .
Vv07g0441       Tc01_g031050    Gorai.007G126400.1      .       .
        .       .       Vv05g0052       Tc04_g028630    Gorai.
006G260200.1    Gorai.005G062000.1      .       Gorai.011G193800.1
        .       .       .       .       .       .       .       .
Vv07g0442       Tc01_g031060    Gorai.007G126300.1      .       .
        .       .       .       .       .       .       .       .
        .       .       .       .       .       .       .       .
Vv07g0443       Tc01_g031070    .       .       .       .       .
        .       .       .       .       .       .       .       .
        .       .       .       .       .       .
Vv07g0444       .       .       .       .       .       .       .
        .       .       .       .       .       .       .       .
        .       .       .       .       .
Vv07g0445       Tc01_g031080    Gorai.007G126200.1      .       .
        .       .       .       .       .       .       .       .
        .       .       .       .       .       .       .       .
Vv07g0446       Tc01_g031090    .       .       .       Gorai.
004G051500.1    .       .       .       .       .       .       .
        .       Vv14g0194       .       .       .       .       .
        .
Vv07g0447       .       .       .       .       .       .       .
        .       .       .       .       .       .       .       .
        .       .       .       .       .
Vv07g0448       .       .       .       .       .       .
Vv05g0059       Tc04_g028710    Gorai.006G260100.1      .
Gorai.006G077400.1      Gorai.011G194200.1      .       .       .
        .       .       .       .       .
Vv07g0449       .       .       .       .       .       .       .
        .       .       .       .       .       .       .       .
        .       .       .       .       .
Vv07g0450       .       .       .       .       .       .       .
        .       .       .       .       .       .       .       .
        .       .       .       .       .
Vv07g0451       .       .       .       .       .       .       .
        .       .       .       .       .       .       .       .
        .       .       .       .       .
Vv07g0452       Tc01_g031120    .       .       .       .       .
        .       .       .       .       .       .       .
Vv14g0181       Tc04_g020020    .       .       .       Gorai.
011G150300.1    .
Vv07g0453       Tc01_g031130    .       Gorai.008G053500.1      .
        .       .       .       .       .       .       .       .
        .       .       .       .       .       .       .       .
```

Vv07g0454 Tc01_g031140 Gorai.007G126100.1 Gorai.
008G053400.1 . . Gorai.004G143000.1 Vv05g0091
Tc04_g028960 Gorai.006G259900.1 . Gorai.
006G077900.1 . . . . . . .
. .
Vv07g0455 Tc01_g031150 . . . Gorai.
004G051400.1 . . . . . . .
. . . . . . . .
Vv07g0456 Tc01_g031160 Gorai.007G126000.1 . .
. Gorai.004G237800.1 Vv05g0092
Tc04_g028970 Gorai.006G259800.1 Gorai.005G065100.1 .
. . Vv14g0179 Tc04_g028970 . .
. . .
Vv07g0457 Tc01_g031170 Gorai.007G125900.1 . .
. . . . . . . .
. Vv14g0174 . Gorai.013G127800.1
Gorai.007G303900.1 Gorai.007G125900.1 . .
Vv07g0458 Tc01_g031180 Gorai.007G125800.1 . .
. . . . . . . .
. . . . . . . .
Vv07g0459 Tc01_g031190 Gorai.007G125700.1 . .
Gorai.004G063600.1 . Vv05g0094
Tc04_g029000 . Gorai.005G065200.1 . . .
. . . . . . .
Vv07g0460 Tc01_g031200 . . . . .
. . . . . . . .
. . . . . .
Vv07g0461 Tc01_g031210 Gorai.007G125600.1 . .
. . . . . . . .
. . . . . . . .
Vv07g0462 Tc01_g031230 Gorai.007G125400.1 . .
. . . . . . . .
. . . . . . . .
Vv07g0463 Tc01_g031260 Gorai.007G125300.1 . .
. . . . . . . .
. . . . . . . .
Vv07g0464 Tc01_g031280 . . . . .
. . . . . . . .
. . . . . .
Vv07g0465 Tc01_g031290 . . . . .
Vv05g0096 Tc04_g029020 . Gorai.005G065300.1
. . Gorai.002G029400.1 Vv14g0172
Tc04_g029020 . . . . .
Vv07g0466 Tc01_g031300 . . . . .
. . . . . . . .
. . . . . .
Vv07g0467 . . . . . . .
. . . . . . . .
. . . . .
Vv07g0468 Tc01_g031310 Gorai.007G124900.1 . .

.	Gorai.004G235800.1	.	.	.	.
.	.	.	.	.	.	.	.
.	.
Vv07g0469	Tc01_g031320	.	.	.	.	.
.	.	.	.	.	.	.	.
.	.	.	.	.	.
Vv07g0470	.	.	.	.	.	Gorai.
004G232800.1	.	.	.	.	.	.	.
.	.	.	.	.	.	.
Vv07g0471	.	.	.	.	.	.
Vv05g0099	.	.	.	.	.	.	.
.	.	.	.	.	.
Vv07g0472	Tc01_g031380	Gorai.007G124700.1	.	.
.	.	.	.	.	.	.	.
.	.	.	.	.	.	.	.
Vv07g0473	.	.	.	.	.	.	.
.	.	.	.	.	.	.	.
.	.	.	.	.
Vv07g0474	Tc01_g031390	Gorai.007G124600.1	Gorai.
008G049700.1	.	Gorai.004G050800.1	.	.	.
.	.	.	.	.	.	.	.
.	.	.	.
Vv07g0475	.	.	.	.	.	.	.
.	.	.	.	.	.	.	.
.	.	.	.	.
Vv07g0476	Tc01_g031410	Gorai.007G124500.1	.	.
.	.	.	.	.	.	.	.
.	.	.	.	.	.	.	.
Vv07g0477	Tc01_g031420	Gorai.007G124400.1	.	.
.	.	.	.	.	.	.	.
.	Vv14g0158	.	Gorai.013G128900.1
Gorai.007G305200.1	Gorai.007G124400.1	.	.
Vv07g0478	Tc01_g031430	Gorai.007G124300.1	Gorai.
008G141200.1	.	Gorai.004G050700.1	.	.	.
.	.	.	.	.	.	.	.
.	.	.	.
Vv07g0479	.	.	.	.	.	.	.
.	.	.	.	.	.	.	.
.	.	.	.	.
Vv07g0480	Tc01_g031460	.	Gorai.008G141000.1	.
.	.	.	.	.	.	.	.
.	.	.	.	.	.	.	.
Vv07g0481	.	.	.	.	.	.	.
.	.	.	.	.	.	.	.
.	.	.	.	.
Vv07g0482	Tc01_g031480	Gorai.007G123700.1	.	.
Gorai.004G065200.1	Gorai.004G143600.1	.	.
.	.	.	.	.	.	.	.
.	.	.	.
Vv07g0483	.	.	.	.	.	.	.

. . . . . . . .
. . . . .
Vv07g0484 . . . . . . .
. . . . . . . .
. . . . .
Vv07g0485 Tc01_g031490 Gorai.007G123600.1 . .
. Gorai.004G143700.1 . . . .
. . . . . . . .
. .
Vv07g0486 Tc01_g031500 Gorai.007G123500.1 . .
. Gorai.004G143800.1 . . . .
. . . . . . . .
. .
Vv07g0487 . . . . . . .
. . . . . . . .
. . . . .
Vv07g0488 . . . . . . .
. . . . . . . .
. . . . .
Vv07g0489 . . . . . . .
. . . . . . . .
. . . . .
Vv07g0490 Tc01_g031510 . Gorai.008G140900.1 .
. . . . . . . .
. . . . . . . .
Vv07g0491 Tc01_g031520 Gorai.007G123400.1 Gorai.
008G140700.1 . . Gorai.004G143900.1 . .
. . . . . . . .
. . . .
Vv07g0492 Tc01_g031530 . Gorai.008G140600.1 .
. . Vv05g0109 Tc04_g029160 Gorai.
006G259500.1 . . Gorai.011G198200.1 . .
. . . . . .
Vv07g0493 Tc01_g031540 . Gorai.008G140500.1 .
. Gorai.004G144000.1 . . . .
. . . . . . . .
. .
Vv07g0494 . . . . . . .
. . . . . . . .
. . . . .
Vv07g0495 . . . . . . .
. . . . . . . .
. . . . .
Vv07g0496 Tc01_g031570 . . . . .
Vv05g0117 Tc04_g029220 Gorai.006G259400.1
Gorai.005G066000.1 . Gorai.011G198800.1 . .
. . . . . .
Vv07g0497 Tc01_g031580 . Gorai.008G140200.1 .
. Gorai.004G144200.1 . . . .
. . . Vv14g0144 . . .

. . .
Vv07g0498 Tc01_g031590 Gorai.007G123200.1 Gorai.
008G140100.1 . . . . . . .
. . . . . . . .
. .
Vv07g0499 Tc01_g031600 . . . . .
. . . . . . . .
. . . . . .
Vv07g0500 Tc01_g031620 . Gorai.008G139900.1 .
. . . . . . . .
. . . . . . . .
Vv07g0501 . . . . . . .
. . . . . . Vv14g0143
. Gorai.013G129200.1 Gorai.007G305800.1
Gorai.013G206900.1 . .
Vv07g0502 . . . . . . .
. . . . . . . .
. . . . .
Vv07g0503 . . . . . . .
. . . . . . . .
. . . . .
Vv07g0504 Tc01_g031630 . . . . .
. . . . . . . .
. . . . . .
Vv07g0505 . . . . . . .
. . . . . . . .
. . . . .
Vv07g0506 Tc01_g031640 . . . . .
. . . . . . . .
. . . . . .
Vv07g0507 Tc01_g031650 Gorai.007G123000.1 . .
. Gorai.004G144300.1 . . . .
. . . . . . . .
. .
Vv07g0508 Tc01_g031660 Gorai.007G122900.1 Gorai.
008G139800.1 . . . . . . .
. . . . . . . .
. .
Vv07g0509 Tc01_g031670 Gorai.007G122800.1 Gorai.
008G139700.1 . Gorai.004G050400.1 . Vv05g0126
Tc04_g029350 . . Gorai.006G079800.1 .
. Vv14g0131 Tc04_g029350 . .
Gorai.007G122800.1 . .
Vv07g0510 Tc01_g031710 . Gorai.008G139300.1 .
. . . . . . . .
. . . . . . . .
Vv07g0511 Tc01_g031730 Gorai.007G122600.1 . .
. Gorai.004G144500.1 . . . .
. . . . . . . .
. .

Vv07g0512 Tc01_g031740 . Gorai.008G139200.1 .
. . . . . . . .
. . . . . . . .
Vv07g0513 Tc01_g031750 . Gorai.008G139100.1 .
. . . . . . . .
. . . . . . . .
Vv07g0514 Tc01_g031760 . Gorai.008G139000.1 .
. . Vv05g0128 Tc04_g029390 Gorai.
006G258800.1 Gorai.005G066300.1 . . . .
. . . . . .
Vv07g0515 . . . . . .
Vv05g0129 Tc04_g029400 Gorai.006G258700.1 . .
Gorai.011G199600.1 . . . . .
. . .
Vv07g0516 Tc01_g031770 Gorai.007G122400.1 . .
Gorai.004G050300.1 . . . . .
. . . . . . . .
. .
Vv07g0517 Tc01_g031780 Gorai.007G122300.1 Gorai.
008G138900.1 . Gorai.004G048600.1 . . .
. . . . . . . .
. . . .
Vv07g0518 Tc01_g031790 Gorai.007G122200.1 Gorai.
008G138800.1 . . . . . . .
. . . . . . . .
. .
Vv07g0519 . . . . . . .
. . . . . . . .
. . . . .
Vv07g0520 . . . . Gorai.004G066400.1
. . . . . . . .
. . . . . . .
Vv07g0521 . . . . . . .
. . . . . . . .
. . . . .
Vv07g0522 Tc01_g031800 . . . . .
Vv14g0122 Tc04_g029410 . Gorai.007G306800.1
. . . . . . . .
. .
Vv07g0523 Tc01_g031810 . Gorai.008G138700.1 .
. . . . . . . .
. . . . . . . .
Vv07g0524 Tc01_g031820 . Gorai.008G138500.1 .
. . . . . . . .
. . . . . . . .
Vv07g0525 . . . . . . .
. . . . . . . .
. . . . .
Vv07g0526 Tc01_g031830 . . . . .
Vv05g0307 . . . . . .

. . . . . . .
Vv07g0527 Tc01_g031840 Gorai.007G121600.1 . .
. . . . . . . .
. . . . . . . .
Vv07g0528 . . . . . . .
. . . . . . . .
. . . . .
Vv07g0529 Tc01_g031850 . . . Gorai.
004G069400.1 Gorai.004G144700.1 . . . .
. . . . . . . .
. .
Vv07g0530 . . . . . . .
. . . . . . . .
. . . . .
Vv07g0531 Tc01_g031890 . . . .
Gorai.004G144800.1 . . . . . .
. . . . . . . .
Vv07g0532 Tc01_g031900 . Gorai.008G138300.1 .
. . . . . . . .
. . . . . . . .
Vv07g0533 Tc01_g031940 . Gorai.008G138000.1 .
. . . . . . . .
. . . . . . . .
Vv07g0534 . . . . . . .
. . . . . . . .
. . . . .
Vv07g0535 . . . . . . .
. . . . . . . .
. . . . .
Vv07g0536 Tc01_g031960 Gorai.007G121200.1 Gorai.
008G137900.1 . Gorai.004G069500.1 Gorai.004G144900.1
. . . . . . . .
. . . . . .
Vv07g0537 Tc01_g031970 . . . . .
. . . . . . . .
. . . . . .
Vv07g0538 . . . . . . .
. . . . . . . .
. . . . .
Vv07g0539 Tc01_g032000 . . . . .
. . . . . . . .
. . . . . .
Vv07g0540 Tc01_g032010 . . . . .
. . . . . . .
Vv14g0111 Tc04_g029860 . . . Gorai.
011G202000.1 .
Vv07g0541 Tc01_g032040 . . . .
Gorai.004G145100.1 . . . . . .
. . . . . . . .
Vv07g0542 Tc01_g032050 . Gorai.008G137800.1 .

. . . . . . . .
. . . . . . . .
Vv07g0543 Tc01_g008480 . . . . .
. . . . . . . .
. . . . . .
Vv07g0544 Tc01_g032080 . . . . .
. . . . . . . .
. . . . . .
Vv07g0545 . . . . . . .
. . . . . . . .
. . . . .
Vv07g0546 Tc01_g032270 . . . .
Gorai.004G145200.1 . . . . . .
. Vv14g0102 Tc04_g030020 . .
Gorai.013G208100.1 . Gorai.002G031600.1
Vv07g0547 Tc01_g032280 . Gorai.008G137600.1 .
. . . . . . . .
. . . . . . . .
Vv07g0548 Tc01_g032290 . . . . .
. . . . . . . .
. . . . . .
Vv07g0549 . . . . . . .
. . . . . . . .
. . . . .
Vv07g0550 . . . . . . .
. . . . . . . .
. . . . .
Vv07g0551 . . . . . . .
. . . . . . . .
. . . . .
Vv07g0552 Tc01_g032300 . . . . .
. . . . . . . .
. . . . . .
Vv07g0553 . . . . . . .
. . . . . . . .
. . . . .
Vv07g0554 . . . . . . .
. . . . . . . .
. . . . .
Vv07g0555 . . . . . . .
. . . . . . . .
. . . . .
Vv07g0556 . . . . . . .
. . . . . . . .
. . . . .
Vv07g0557 . . . . . Gorai.
004G145300.1 . . . . . . .
. . . . . . .
Vv07g0558 . . . . . . .
. . . . . . . .

. . . . .
Vv07g0559 Tc01_g032310 . Gorai.008G137500.1 .
. . . . . . . .
. . . . . . . .
Vv07g0560 Tc01_g032330 . Gorai.008G137400.1 .
. . Vv05g0278 Tc04_g030600 . .
. . . . . . . .
. .
Vv07g0561 Tc01_g032340 . Gorai.008G137300.1 .
. . . . . . . .
. . . . . . . .
Vv07g0562 Tc01_g032350 . . . .
Gorai.004G145900.1 . . . . . .
. Vv14g0096 . . Gorai.007G308200.1
. . .
Vv07g0563 . Gorai.007G120900.1 . . .
. . . . . . . .
Vv14g0085 . Gorai.013G130800.1 . .
. .
Vv07g0564 . . . . . . .
. . . . . . . .
. . . . .
Vv07g0565 Tc01_g032360 . . . . .
. . . . . . .
Vv14g0082 Tc04_g030130 Gorai.013G130900.1 .
Gorai.007G120900.1 . .
Vv07g0566 Tc01_g032380 . . . . .
. . . . . . . .
. . . . . .
Vv07g0567 Tc01_g032390 Gorai.007G120600.1 Gorai.
008G137100.1 . . Gorai.004G146100.1 . .
. . . . . . . .
. . . .
Vv07g0568 . . . . . . .
. . . . . . . .
. . . . .
Vv07g0569 Tc01_g032400 . . . . .
. . . . . . . .
. . . . . .
Vv07g0570 Tc01_g032410 Gorai.007G120500.1 . .
. . . . . . . .
. . . . . . . .
Vv07g0571 . . . . . . .
. . . . . . . .
. . . . .
Vv07g0572 Tc01_g032420 . . . . .
. . . . . . . .
. . . . . .
Vv07g0573 Tc01_g032430 . . . . .
. . . . . . . .

. . . . . .
Vv07g0574 Tc01_g032440 . Gorai.008G137000.1 .
. Gorai.004G146300.1 Vv05g0239
Tc04_g030250 Gorai.006G266200.1 Gorai.005G070200.1 .
. . . . . . . .
.
Vv07g0575 Tc01_g032480 . Gorai.008G136700.1 .
. . Vv05g0237 Tc04_g030230 Gorai.
006G266100.1 Gorai.005G069900.1 . . .
Vv14g0072 Tc04_g030230 . . . . .
Vv07g0576 Tc01_g032490 . Gorai.008G136600.1 .
. . . . . . . .
. . . . . . . .
Vv07g0577 Tc01_g032500 . Gorai.008G136500.1 .
. . . . . . . .
. . . . . . . .
Vv07g0578 Tc01_g032510 Gorai.007G119900.1 . .
. . . . . . . .
. . . . . . . .
Vv07g0579 . . . . . . .
. . . . . . . .
. . . . .
Vv07g0580 Tc01_g032580 . . . . .
. . . . . . . .
. . . . . .
Vv07g0581 Tc01_g008530 . . . . .
. . . . . . . .
. . . . . .
Vv07g0582 . . . . . . .
. . . . . . . .
. . . . .
Vv07g0583 . . . . . . .
. . . . . . . .
. . . . .
Vv07g0584 . . . . . Gorai.
004G146600.1 . . . . . . .
. . . . . . .
Vv07g0585 . . . . . . .
. . . . . . . .
. . . . .
Vv07g0586 Tc01_g032620 Gorai.007G119400.1 Gorai.
008G136100.1 . . Gorai.004G146700.1 . .
. . . . . . . .
. . . .
Vv07g0587 Tc01_g032630 Gorai.007G119300.1 . .
. . . . . . . .
. . . . . . . .
Vv07g0588 Tc01_g032640 . Gorai.008G136000.1 .
. . . . . . . .
. . . . . . . .

Vv07g0589 Tc01_g032650 . . . . .
. . . . . . . .
. . . . . .
Vv07g0590 Tc01_g032660 Gorai.007G119200.1 Gorai.
008G135900.1 . . Gorai.004G146800.1 Vv05g0266
Tc04_g030460 . Gorai.005G071300.1 . .
. Vv14g0055 Tc04_g030460 . .
Gorai.007G119200.1 . .
Vv07g0591 Tc01_g032690 . . . . .
. . . . . . . .
. . . . . .
Vv07g0592 Tc01_g032710 . . . . .
. . . . . . . .
. . . . . .
Vv07g0593 Tc01_g032720 . Gorai.008G135700.1 .
. . Vv05g0270 Tc04_g030500 .
Gorai.005G071400.1 . . . . . .
. . . .
Vv07g0594 . . . . . . .
. . . . . . . .
. . . . .
Vv07g0595 Tc01_g032730 . Gorai.008G135600.1 .
. . . . . . . .
. . . . . . . .
Vv07g0596 . . . . . .
Vv05g0273 Tc04_g030540 . . . . .
. . . . . . .
Vv07g0597 . . . . . . .
. . . . . . Vv14g0049
. Gorai.013G131200.1 . . . .
Vv07g0598 Tc01_g032740 . Gorai.008G135500.1 .
. . . . . . . .
. Vv14g0046 . . . . .
.
Vv07g0599 . Gorai.007G118900.1 . . .
. . . . . . . .
. . . . . . .
Vv07g0600 . . . . . . .
. . . . . . . .
. . . . .
Vv07g0601 Tc01_g008560 . . . . .
. . . . . . . .
. . . . . .
Vv07g0602 . . . . . . .
. . . . . . . .
. . . . .
Vv07g0603 . Gorai.007G118800.1 . . .
. . . . . . . .
. . . . . . .
Vv07g0604 . . . . . . .

| | | | | | | | |
|---|---|---|---|---|---|---|---|
| . | . | . | . | . | . | . | . |
| . | . | . | . | . | | | |
| Vv07g0605 | . | . | . | . | . | . | . |
| . | . | . | . | . | . | . | . |
| . | . | . | . | . | | | |
| Vv07g0606 | . | . | . | . | . | . | . |
| . | . | . | . | . | . | . | . |
| . | . | . | . | . | | | |
| Vv07g0607 | . | . | . | . | . | . | . |
| . | . | . | . | . | . | . | . |
| . | . | . | . | . | | | |
| Vv07g0608 | . | . | . | . | . | . | . |
| . | . | . | . | . | . | . | . |
| . | . | . | . | . | | | |
| Vv07g0609 | . | . | . | . | . | . | . |
| . | . | . | . | . | . | . | . |
| . | . | . | . | . | | | |
| Vv07g0610 | . | Gorai.007G118600.1 | | | . | . | . |
| . | . | . | . | . | . | . | . |
| . | . | . | . | . | . | . | |
| Vv07g0611 | . | . | . | . | . | . | . |
| . | . | . | . | . | . | . | . |
| . | . | . | . | . | | | |
| Vv07g0612 | . | . | . | . | . | . | . |
| . | . | . | . | . | . | . | . |
| . | . | . | . | . | | | |
| Vv07g0613 | . | . | . | . | . | . | . |
| . | . | . | . | . | . | . | . |
| . | . | . | . | . | | | |
| Vv07g0614 | . | . | . | . | . | . | . |
| . | . | . | . | . | . | . | . |
| . | . | . | . | . | | | |
| Vv07g0615 | . | . | . | . | . | . | . |
| . | . | . | . | . | . | . | . |
| . | . | . | . | . | | | |
| Vv07g0616 | . | Gorai.007G118500.1 | | | . | . | . |
| . | . | . | . | . | . | . | . |
| . | . | . | . | . | . | . | |
| Vv07g0617 | . | . | . | . | . | . | |
| Vv05g0304 | . | . | . | . | . | . | . |
| . | . | . | . | . | . | | |
| Vv07g0618 | . | . | . | . | . | . | . |
| . | . | . | . | . | . | . | . |
| . | . | . | . | . | | | |
| Vv07g0619 | . | Gorai.007G118400.1 | | | . | . | . |
| . | Vv05g0305 | | . | . | . | . | . |
| . | Vv14g0027 | | Tc05_g004610 | | Gorai.013G131600.1 | | |
| . | Gorai.013G211000.1 | | | . | . | | |
| Vv07g0620 | . | Gorai.007G118300.1 | | | . | . | . |
| . | . | . | . | . | . | . | . |

. . . . . . .
Vv07g0621 Tc01_g040640 . . . . .
. . . . . . . .
. . . . . .
Vv07g0622 Tc01_g008850 . . . . .
. . . . . . . .
. . . . . .
Vv07g0623 . . . . . . .
. . . . . . . .
. . . . .
Vv07g0624 . . . . . . .
. . . . . . . .
. . . . .
Vv07g0625 Tc01_g040930 Gorai.007G118100.1 . .
. . . . . . . .
. . . . . . . .
Vv07g0626 . Gorai.007G117900.1 . . .
. . . . . . . .
. . . . . . .
Vv07g0627 . Gorai.007G117800.1 . . .
. . . . . . . .
. . . . . . .
Vv07g0628 . Gorai.007G117700.1 . . .
. . . . . . . .
. . . . . . .
Vv07g0629 . Gorai.007G117600.1 . . .
. . . . . . . .
. . . . . . .
Vv07g0630 Tc01_g032940 Gorai.007G117200.1 Gorai.
008G134300.1 . . . Vv05g0165
Tc04_g029800 Gorai.006G265200.1 Gorai.005G068100.1 .
Gorai.011G201800.1 . . . . .
. . .
Vv07g0631 Tc01_g037710 . . . . .
. . . . . . . .
. . . . . .
Vv07g0632 . . . . . Gorai.
004G147300.1 . . . . . . .
. . . . . . .
Vv07g0633 . Gorai.007G117100.1 . . .
. . . . . . . .
. . . . . . .
Vv07g0634 . . . . . . .
. . . . . . . .
. . . . .
Vv07g0635 . . . . . . .
. . . . . . . .
. . . . .
Vv07g0636 . . . . . . .
. . . . . . . .

. . . . .
Vv07g0637 . Gorai.007G117000.1 . . .
. . . . . . . .
. . . . . . .
Vv07g0638 . Gorai.007G116900.1 . . .
. . . . . . . .
. . . . . . .
Vv07g0639 . . . . . . .
. . . . . . . .
. . . . .
Vv07g0640 . . . . . . .
. . . . . . . .
. . . . .
Vv07g0641 Tc01_g009040 . . . . .
. . . . . . . .
. . . . . .
Vv07g0642 . Gorai.007G116600.1 . . .
. . . . . . . .
. . . . . . .
Vv07g0643 . Gorai.007G116500.1 . . .
. . . . . . . .
. . . . . . .
Vv07g0644 . . . . . . .
. . . . . . . .
. . . . .
Vv07g0645 . Gorai.007G116400.1 . . .
. . . . . . . .
. . . . . . .
Vv07g0646 . Gorai.007G116300.1 . . .
. . . . . . . .
. . . . . . .
Vv07g0647 . . . . . . .
. . . . . . . .
. . . . .
Vv07g0648 Tc01_g037910 . . . . .
Vv05g0148 Tc04_g029610 . . . .
Gorai.002G030400.1 . . . . .
. .
Vv07g0649 . . . . . . .
. . . . . . . .
. . . . .
Vv07g0650 Tc01_g037920 . Gorai.008G113200.1 .
. . . . . . . .
. . . . . . . .
Vv07g0651 . . . . . . .
. . . . . . . .
. . . . .
Vv07g0652 . . . . . . .
. . . . . . . .
. . . . .

Vv07g0653 Tc01_g033200 . Gorai.008G133300.1 .
. . . . . . . .
. . . . . . . .
Vv07g0654 Tc01_g033210 . . . . .
. . . . . . . .
. . . . . .
Vv07g0655 Tc01_g033230 . . . . .
. . . . . . . .
. . . . . .
Vv07g0656 Tc01_g033240 . . . .
Gorai.004G148300.1 . . . . . .
. . . . . . . .
Vv07g0657 . . . . . . .
. . . . . . . .
. . . . .
Vv07g0658 Tc01_g033250 Gorai.007G115700.1 . .
. . . . . . . .
. . . . . . . .
Vv07g0659 Tc01_g033270 Gorai.007G115600.1 . .
. . . . . . . .
. . . . . . . .
Vv07g0660 Tc01_g033290 Gorai.007G115300.1 . .
. . . . . . . .
. . . . . . . .
Vv07g0661 . . . . . . .
. . . . . . . .
. . . . .
Vv07g0662 . . . . . . .
. . . . . . . .
. . . . .
Vv07g0663 . . . . . . .
. . . . . . . .
. . . . .
Vv07g0664 . . . . . . .
. . . . . . . .
. . . . .
Vv07g0665 . . . . . . .
. . . . . . . .
. . . . .
Vv07g0666 . . . . . . .
. . . . . . . .
. . . . .
Vv07g0667 . . . . . . .
. . . . . . . .
. . . . .
Vv07g0668 . . . . . . .
. . . . . . . .
. . . . .
Vv07g0669 . . . . . . .
. . . . . . . .

| | | | | | | | | |
|---|---|---|---|---|---|---|---|---|
| | . | . | . | . | . | | | |
| Vv07g0670 | | . | . | . | . | . | . | . |
| | . | . | . | . | . | . | . | . |
| | . | . | . | . | . | | | |
| Vv07g0671 | | . | . | . | . | . | . | . |
| | . | . | . | . | . | . | . | . |
| | . | . | . | . | . | | | |
| Vv07g0672 | | . | . | . | . | . | . | . |
| | . | . | . | . | . | . | . | . |
| | . | . | . | . | . | | | |
| Vv07g0673 | | . | . | . | . | . | . | . |
| | . | . | . | . | . | . | . | . |
| | . | . | . | . | . | | | |
| Vv07g0674 | | . | . | . | . | . | . | . |
| | . | . | . | . | . | . | . | . |
| | . | . | . | . | . | | | |
| Vv07g0675 | | . | . | . | . | . | . | . |
| | . | . | . | . | . | . | . | . |
| | . | . | . | . | . | | | |
| Vv07g0676 | | . | . | . | . | . | . | . |
| | . | . | . | . | . | . | . | . |
| | . | . | . | . | . | | | |
| Vv07g0677 | | . | . | . | . | . | . | . |
| | . | . | . | . | . | . | . | . |
| | . | . | . | . | . | | | |
| Vv07g0678 | | . | . | . | . | . | . | . |
| | . | . | . | . | . | . | . | . |
| | . | . | . | . | . | | | |
| Vv07g0679 | | . | . | . | . | . | . | . |
| | . | . | . | . | . | . | . | . |
| | . | . | . | . | . | | | |
| Vv07g0680 | | . | . | . | . | . | . | . |
| | . | . | . | . | . | . | . | . |
| | . | . | . | . | . | | | |
| Vv07g0681 | | . | Gorai.007G113800.1 | | | . | . | . |
| | . | . | . | . | . | . | . | . |
| | . | . | . | . | . | . | . | |
| Vv07g0682 | | . | . | . | . | Gorai.004G073600.1 | | |
| | . | . | . | . | . | . | . | . |
| | . | . | . | . | . | . | . | |
| Vv07g0683 | | . | . | . | . | . | . | . |
| | . | . | . | . | . | . | . | . |
| | . | . | . | . | . | | | |
| Vv07g0684 | | . | . | . | . | . | Gorai. | |
| 004G219700.1 | | . | . | . | . | . | . | . |
| | . | . | . | . | . | . | . | |
| Vv07g0685 | | . | . | . | . | . | . | . |
| | . | . | . | . | . | . | . | . |
| | . | . | . | . | . | | | |
| Vv07g0686 | | . | . | . | . | . | . | . |

.	.	.	.	.	.	.	.
.	.	.	.	.
Vv07g0687	.	.	.	.	.	.	.
.	.	.	.	.	.	.	.
.	.	.	.	.
Vv07g0688	Tc01_g037490	Gorai.007G144400.1	Gorai.
008G114300.1	.	Gorai.004G073300.1	Gorai.004G219900.1
.	.	.	.	.	.	.	.
.	.	.	.	.	.
Vv07g0689	Tc01_g037470	Gorai.007G144500.1	Gorai.
008G114400.1	.	Gorai.004G073200.1	Gorai.004G220000.1
.	.	.	.	.	.	.	.
.	.	.	.	.	.
Vv07g0690	.	.	.	.	.	.	.
.	.	.	.	.	.	.	.
.	.	.	.	.
Vv07g0691	.	.	.	.	.	.	.
.	.	.	.	.	.	.	.
.	.	.	.	.
Vv07g0692	.	.	.	.	.	.	.
.	.	.	.	.	.	.	.
.	.	.	.	.
Vv07g0693	.	.	.	.	.	.	.
.	.	.	.	.	.	.	.
.	.	.	.	.
Vv07g0694	.	.	.	.	Gorai.004G073000.1
.	.	.	.	.	.	.	.
.	.	.	.	.	.	.
Vv07g0695	Tc01_g033420	.	Gorai.008G132500.1	.
.	.	Vv05g0773	Tc04_g025340	.
Gorai.005G049000.1	.	.	.	.	.	.
.	.	.	.
Vv07g0696	.	Gorai.007G145100.1	.	.	.
.	.	.	.	.	.	.	.
.	.	.	.	.	.	.
Vv07g0697	.	Gorai.007G145200.1	.	.
Gorai.004G072800.1	Gorai.004G220200.1	.	.	.
.	.	.	.	.	.	.	.
.	.	.
Vv07g0698	.	Gorai.007G145300.1	.	.
Gorai.004G070200.1	Gorai.004G220300.1	.	.	.
.	.	.	.	.	.	.	.
.	.	.
Vv07g0699	.	Gorai.007G145400.1	.	.	.
.	.	.	.	.	.	.	.
.	.	.	.	.	.	.
Vv07g0700	.	.	.	.	.	.	.
.	.	.	.	.	.	.	.
.	.	.	.	.
Vv07g0701	Tc01_g033570	.	.	.	.

Gorai.004G220500.1 . . . . . .
. . . . . . . .
Vv07g0702 . . . . . . .
. . . . . . . .
. . . . .
Vv07g0703 Tc01_g037120 . . . . .
. . . . . . . .
. . . . . .
Vv07g0704 . . . . . . .
. . . . . . . .
. . . . .
Vv07g0705 . Gorai.007G145600.1 . . .
. . . . . . . .
. . . . . . .
Vv07g0706 . . . . . . .
. . . . . . . .
. . . . .
Vv07g0707 Tc01_g004930 . . . . .
. . . . . . . .
. . . . . .
Vv07g0708 . . . . . . .
. . . . . . . .
. . . . .
Vv07g0709 . Gorai.007G145700.1 . . .
. . . . . . . .
. . . . . . .
Vv07g0710 . . . . . . .
. . . . . . . .
. . . . .
Vv07g0711 . . . . . . .
. . . . . . . .
. . . . .
Vv07g0712 Tc01_g004990 . . . . .
. . . . . . . .
. . . . . .
Vv07g0713 Tc01_g036820 Gorai.007G145800.1 Gorai.
008G116800.1 . Gorai.004G057000.1 Gorai.004G220800.1
. . . . . . . .
. . . . . .
Vv07g0714 . . . . Gorai.004G057100.1
. . . . . . . .
. . . . . . .
Vv07g0715 . . . . . . .
. . . . . . . .
. . . . .
Vv07g0716 . . . . . . .
. . . . . . . .
. . . . .
Vv07g0717 Tc01_g003460 Gorai.007G146000.1 . .
. . Vv05g0813 Tc04_g025470 . .

Gorai.006G072100.1 . . . . .
. . . .
Vv07g0718 . . . . . . .
. . . . . . . .
. . . . .
Vv07g0719 . . . . . .
Vv05g0816 Tc04_g025480 . . . . .
. . . . . . .
Vv07g0720 . . . . . . .
. . . . . . . .
. . . . .
Vv07g0721 . . . . . Gorai.
004G220900.1 Vv05g0823 . . Gorai.005G050100.1
. . . . . . . .
. .
Vv07g0722 . . . . . . .
. . . . . . . .
. . . . .
Vv07g0723 . . . . . . .
. . . . . . . .
. . . . .
Vv07g0724 . . . . . . .
. . . . . . . .
. . . . .
Vv07g0725 . . . . . . .
. . . . . . . .
. . . . .
Vv07g0726 . . . . . . .
. . . . . . . .
. . . . .
Vv07g0727 . . . . . Gorai.
004G221000.1 . . . . . . .
. . . . . . .
Vv07g0728 . . . . . . .
. . . . . . . .
. . . . .
Vv07g0729 . . . . Gorai.004G057200.1
. . . . . . . .
. . . . . . .
Vv07g0730 . . . . . . .
. . . . . . . .
. . . . .
Vv07g0731 . . . . . . .
. . . . . . . .
. . . . .
Vv07g0732 . . . . . . .
. . . . . . . .
. . . . .
Vv07g0733 . . . . . . .
. . . . . . . .

. . . . .
Vv07g0734 . . . . . . .
. . . . . . . .
. . . . .
Vv07g0735 Tc01_g003270 Gorai.007G146400.1 . .
. . . . . . . .
. . . . . . . .
Vv07g0736 . . . . . . .
. . . . . . . .
. . . . .
Vv07g0737 . . . . . . .
. . . . . . . .
. . . . .
Vv07g0738 . Gorai.007G146500.1 . . .
. . . . . . . .
. . . . . . .
Vv07g0739 . . . . . . .
. . . . . . . .
. . . . .
Vv07g0740 . . . . . . .
. . . . . . . .
. . . . .
Vv07g0741 . . . . . . .
. . . . . . . .
. . . . .
Vv07g0742 . Gorai.007G146600.1 . . .
Gorai.004G221100.1 . . . . .
. . . . . . . .
.
Vv07g0743 Tc01_g036810 . Gorai.008G116900.1 .
. . . . . . . .
. . . . . . . .
Vv07g0744 . Gorai.007G147000.1 . . .
Gorai.004G221200.1 Vv05g0839 Tc04_g025630
Gorai.006G253300.1 Gorai.005G050400.1 . Gorai.
011G174900.1 . . . . . . .
.
Vv07g0745 . . . . . . .
. . . . . . . .
. . . . .
Vv07g0746 . . . . . . .
. . . . . . . .
. . . . .
Vv07g0747 Tc01_g025290 Gorai.007G147100.1 . .
. . . . . . . .
. . . . . . . .
Vv07g0748 Tc01_g025300 . . . . .
. . . . . . . .
. . . . . .
Vv07g0749 Tc01_g025330 Gorai.007G147300.1 . .

```
        .       .       .       .       .       .       .       .
        .       .       .       .       .       .       .       .
Vv07g0750       Tc01_g025350    Gorai.007G147500.1      .       .
        .       .       .       .       .       .       .       .
        .       .       .       .       .       .       .       .
Vv07g0751       Tc01_g025360    .       .       .       .       .
        Vv05g0841       Tc04_g025640    .       Gorai.005G050500.1
        .       Gorai.011G175000.1      .       .       .       .
        .       .       .       .
Vv07g0752       Tc01_g025370    Gorai.007G147600.1      .       .
        .       .       .       .       .       .       .       .
        .       .       .       .       .       .       .       .
Vv07g0753       Tc01_g025390    .       .       .       .       .
        .       .       .       .       .       .       .       .
        .       .       .       .       .       .
Vv07g0754       Tc01_g025400    .       .       .       .
Gorai.004G221400.1      .       .       .       .       .       .
        .       .       .       .       .       .       .       .
Vv07g0755       Tc01_g025410    .       .       .       .       .
        .       .       .       .       .       .       .       .
        .       .       .       .       .       .
Vv07g0756       Tc01_g025420    Gorai.007G147700.1      .       .
        .       .       .       .       .       .       .       .
        .       .       .       .       .       .       .       .
Vv07g0757       Tc01_g025440    .       .       .       .       .
        .       .       .       .       .       .       .       .
        .       .       .       .       .       .
Vv07g0758       Tc01_g025460    Gorai.007G147800.1      .       .
        .       Gorai.004G221700.1      .       .       .       .
        .       .       .       .       .       .       .       .
        .       .
Vv07g0759       Tc01_g025470    .       .       .       .       .
        .       .       .       .       .       .       .       .
        .       .       .       .       .       .
Vv07g0760       Tc01_g025500    .       .       .       .       .
        .       .       .       .       .       .       .       .
        .       .       .       .       .       .
Vv07g0761       Tc01_g025520    Gorai.007G148000.1      .       .
        .       .       .       .       .       .       .       .
        .       .       .       .       .       .       .       .
Vv07g0762       Tc01_g025530    Gorai.007G148100.1      .       .
        .       .       Vv05g0858       Tc04_g025810    .       .
        .       Gorai.011G176800.1      .       .       .       .
        .       .       .       .
Vv07g0763       Tc01_g025540    .       .       .       .       .
        .       .       .       .       .       .       .       .
        .       .       .       .       .       .
Vv07g0764       Tc01_g025560    Gorai.007G148200.1      .       .
        .       .       .       .       .       .       .       .
        .       .       .       .       .       .       .       .
```

Vv07g0765 . . . . . . .
. . . . . . . .
. . . . .
Vv07g0766 Tc01_g025580 . . . . .
Vv05g0864 Tc04_g025850 Gorai.006G252500.1 .
. Gorai.011G177100.1 . . . .
. . . .
Vv07g0767 Tc01_g025590 . . . . .
. . . . . . . .
. . . . . .
Vv07g0768 . . . . . . .
. . . . . . . .
. . . . .
Vv07g0769 Tc01_g025600 . . . . .
. . . . . . . .
. . . . . .
Vv07g0770 Tc01_g025610 Gorai.007G148300.1 . .
. Gorai.004G221900.1 Vv05g0868
Tc04_g025900 Gorai.006G252200.1 Gorai.005G051600.1 .
. . . . . . . .
.
Vv07g0771 . . . . . . .
. . . . . . . .
. . . . .
Vv07g0772 Tc01_g025620 Gorai.007G148500.1 . .
. Gorai.004G222000.1 Vv05g0870
Tc04_g025920 . Gorai.005G051900.1 . . .
. . . . . . .
Vv07g0773 . . . . . . .
. . . . . . . .
. . . . .
Vv07g0774 . . . . . . .
. . . . . . . .
. . . . .
Vv07g0775 . . . . . . .
. . . . . . . .
. . . . .
Vv07g0776 Tc01_g025640 Gorai.007G148600.1 . .
Gorai.004G061600.1 . . . . .
. . . . . . . .
. .
Vv07g0777 Tc01_g025650 . . . Gorai.
004G057300.1 . . . . . . .
. . . . . . . .
Vv07g0778 . . . . . . .
. . . . . . . .
. . . . .
Vv07g0779 . . . . . .
Vv05g0876 Tc04_g025950 . Gorai.005G052100.1 .
. . . . . . . .

.
Vv07g0780 Tc01_g025670 . Gorai.001G238400.1 .
. . . . . . . .
. . . . . . . .
Vv07g0781 . . . . . . .
. . . . . . . .
. . . . .
Vv07g0782 . . . . . . .
. . . . . . . .
. . . . .
Vv07g0783 Tc01_g025680 . Gorai.001G238300.1 .
. . Vv05g0878 Tc04_g025970 .
Gorai.005G052500.1 . . . . . .
. . . .
Vv07g0784 . . . . . . .
. . . . . . . .
. . . . .
Vv07g0785 . . . . Gorai.004G061400.1
. . . . . . . .
. . . . . . .
Vv07g0786 Tc01_g025690 Gorai.007G148800.1 . .
. . . . . . . .
. . . . . . . .
Vv07g0787 . . . . . . .
. . . . . . . .
. . . . .
Vv07g0788 . . . . . . .
. . . . . . . .
. . . . .
Vv07g0789 . . . . . . .
. . . . . . . .
. . . . .
Vv07g0790 Tc01_g025700 . . . . .
. . . . . . . .
. . . . . .
Vv07g0791 . . . . . . .
. . . . . . . .
. . . . .
Vv07g0792 Tc01_g025760 . . . . .
. . . . . . . .
. . . . . .
Vv07g0793 . . . . . . .
. . . . . . . .
. . . . .
Vv07g0794 . . . . . . .
. . . . . . . .
. . . . .
Vv07g0795 . . . . . . .
. . . . . . . .
. . . . .

Vv07g0796 Tc01_g025770 . Gorai.001G238100.1 .
. . . . . . . .
. . . . . . . .
Vv07g0797 Tc01_g025780 Gorai.007G149100.1 . .
. . . . . . . .
. . . . . . . .
Vv07g0798 Tc01_g025830 . . . . .
. . . . . . . .
. . . . . .
Vv07g0799 . . . . . . .
. . . . . . . .
. . . . .
Vv07g0800 Tc01_g025840 Gorai.007G149200.1 . .
. . . . . . . .
. . . . . . . .
Vv07g0801 Tc01_g025870 Gorai.007G149400.1 . .
. . Vv05g0894 Tc04_g026210 Gorai.
006G251500.1 Gorai.005G053300.1 . . . .
. . . . . .
Vv07g0802 . . . . . . .
. . . . . . . .
. . . . .
Vv07g0803 Tc01_g025880 . . . . .
. . . . . . . .
. . . . . .
Vv07g0804 Tc01_g025890 Gorai.007G149600.1 . .
. Gorai.004G222200.1 Vv05g0895 . .
. . . . . . . .
. . .
Vv07g0805 Tc01_g025940 . . . . .
. . . . . . . .
. . . . . .
Vv07g0806 Tc01_g025950 . . . .
Gorai.004G222400.1 Vv05g0904 Tc04_g026280 . .
. . . . . . . .
. .
Vv07g0807 . . . . . . .
. . . . . . . .
. . . . .
Vv07g0808 . . . . . . .
. . . . . . . .
. . . . .
Vv07g0809 Tc01_g026000 Gorai.007G149700.1 Gorai.
001G237700.1 . . . . . . .
. . . . . . . .
. .
Vv07g0810 Tc01_g026020 Gorai.007G149800.1 . .
Gorai.004G059100.1 . Vv05g0909
Tc04_g026400 . Gorai.005G054100.1 . Gorai.
011G179700.1 . . . . . . .

.
Vv07g0811 . . . . . . .
. . . . . . . .
. . . . .
Vv07g0812 . . . . . . .
. . . . . . . .
. . . . .
Vv07g0813 . . . . . . .
. . . . . . . .
. . . . .
Vv07g0814 Tc01_g026060 Gorai.007G149900.1 . .
Gorai.004G057500.1 . . . . .
. . . . . . . .
. .
Vv07g0815 Tc01_g026070 . . . . .
. . . . . . . .
. . . . . .
Vv07g0816 Tc01_g026130 Gorai.007G150000.1 . .
. Gorai.004G222500.1 Vv05g0913
Tc04_g026430 Gorai.006G250900.1 . . Gorai.
011G180100.1 . . . . . . .
.
Vv07g0817 Tc01_g026140 . Gorai.001G237400.1 .
. . . . . . . .
. . . . . . . .
Vv07g0818 . . . . . . .
. . . . . . . .
. . . . .
Vv07g0819 Tc01_g026150 . . . . .
. . . . . . . .
. . . . . .
Vv07g0820 Tc01_g026160 . . . . .
. . . . . . . .
. . . . . .
Vv07g0821 Tc01_g026170 . . . . .
. . . . . . . .
. . . . . .
Vv07g0822 Tc01_g026180 Gorai.007G150100.1 . .
. . . . . . . .
. . . . . . . .
Vv07g0823 Tc01_g026200 Gorai.007G150200.1 . .
. . . . . . . .
. . . . . . . .
Vv07g0824 Tc01_g028640 . . . . .
. . . . . . . .
. . . . . .
Vv07g0825 Tc01_g026210 Gorai.007G150300.1 . .
. . . . . . . .
. . . . . . . .
Vv07g0826 . . . . . . .

. . . . . . . .
. . . . .
Vv07g0827 Tc01_g026220 . . . . .
. . . . . . . .
. . . . . .
Vv07g0828 . . . . . . .
. . . . . . . .
. . . . .
Vv07g0829 . . . . . . .
. . . . . . . .
. . . . .
Vv07g0830 Tc01_g026290 . Gorai.001G237200.1 .
. . . . . . . .
. . . . . . . .
Vv07g0831 . . . . . . .
. . . . . . . .
. . . . .
Vv07g0832 Tc01_g026300 Gorai.007G150400.1 Gorai.
001G237100.1 . . . . . . .
. . . . . . . .
. .
Vv07g0833 . . . . . . .
. . . . . . . .
. . . . .
Vv07g0834 . . . . . . .
. . . . . . . .
. . . . .
Vv07g0835 . . . . . . .
. . . . . . . .
. . . . .
Vv07g0836 . . . . . . .
. . . . . . . .
. . . . .
Vv07g0837 . . . . . . .
. . . . . . . .
. . . . .
Vv07g0838 . . . . . . .
. . . . . . . .
. . . . .
Vv07g0839 . Gorai.007G150500.1 . .
Gorai.004G057600.1 . . . . . .
. . . . . . . .
.
Vv07g0840 . . . . . . .
. . . . . . . .
. . . . .
Vv07g0841 . Gorai.007G150600.1 . . .
. . . . . . . .
. . . . . . .
Vv07g0842 Tc01_g007730 . Gorai.001G236500.1 .

```
         .        .        .        .        .        .        .        .
         .        .        .        .        .        .        .        .
Vv07g0843         .        .        .        .        .        .        .
         .        .        .        .        .        .        .        .
         .        .        .        .        .
Vv07g0844         .        .        .        .        .        .        .
         .        .        .        .        .        .        .        .
         .        .        .        .        .
Vv07g0845         .        .        .        .        .        .        .
         .        .        .        .        .        .        .        .
         .        .        .        .        .
Vv07g0846         .        .        .        .       Gorai.004G055700.1
         Gorai.004G222700.1         .        .        .        .        .
         .        .        .        .        .        .        .        .
         .
Vv07g0847         .        .        .        .        .        .        .
         .        .        .        .        .        .        .        .
         .        .        .        .        .
Vv07g0848         .        .        .        .        .        .        .
         .        .        .        .        .        .        .        .
         .        .        .        .        .
Vv07g0849         .        .        .        .        .        .        .
         .        .        .        .        .        .        .        .
         .        .        .        .        .
Vv07g0850         Tc01_g028260      .        .        .        .        .
         .        .        .        .        .        .        .        .
         .        .        .        .        .        .
Vv07g0851         .        .        .        .        .        .        .
         .        .        .        .        .        .        .        .
         .        .        .        .        .
Vv07g0852         Tc01_g007500      .        .        .        .        .
         .        .        .        .        .        .        .        .
         .        .        .        .        .        .
Vv07g0853         .        .        .        .       Gorai.004G052700.1
         .        .        .        .        .        .        .        .
         .        .        .        .        .        .        .
Vv07g0854         .        .       Gorai.001G236300.1          .
Gorai.004G057700.1         .        .        .        .        .        .
         .        .        .        .        .        .        .        .
         .
Vv07g0855         .        .        .        .        .        .        .
         .        .        .        .        .        .        .        .
         .        .        .        .        .
Vv07g0856         Tc01_g026670     Gorai.007G150900.1          .        .
         .        Gorai.004G223100.1        .        .        .        .
         .        .        .        .        .        .        .        .
         .        .
Vv07g0857         .        .        .        .        .        .        .
         .        .        .        .        .        .        .        .
         .        .        .        .        .
```

Vv07g0858 . Gorai.007G151100.1 Gorai.001G236200.1
. . Gorai.004G223200.1 . . .
. . . . . . . .
. . .
Vv07g0859 . . . . . . .
. . . . . . . .
. . . . .
Vv07g0860 . . . . . . .
. . . . . . . .
. . . . .
Vv07g0861 . . . . . . .
. . . . . . . .
. . . . .
Vv07g0862 . . . . . . .
. . . . . . . .
. . . . .
Vv07g0863 . . . . . . .
. . . . . . . .
. . . . .
Vv07g0864 . Gorai.007G151300.1 Gorai.001G236100.1
. . . . . . . .
. . . . . . . .
.
Vv07g0865 . . . . . . .
. . . . . . . .
. . . . .
Vv07g0866 . Gorai.007G151400.1 Gorai.001G236000.1
. . . . . . . .
. . . . . . . .
.
Vv07g0867 . . . . . . .
. . . . . . . .
. . . . .
Vv07g0868 . . . . . . .
. . . . . . . .
. . . . .
Vv07g0869 . . Gorai.001G235900.1 . .
. . . . . . . .
. . . . . . .
Vv07g0870 . . . . . . .
. . . . . . . .
. . . . .
Vv07g0871 . . . . . Gorai.
004G223300.1 . . . . . . .
. . . . . . .
Vv07g0872 Tc01_g023400 . Gorai.001G235600.1 .
. . . . . . . .
. . . . . . . .
Vv07g0873 Tc01_g023410 . . . Gorai.
004G057900.1 Gorai.004G223400.1 . . . .

```
         .        .        .        .        .        .        .        .
         .        .
Vv07g0874         .        .        .        .        .        .        .
         .        .        .        .        .        .        .        .
         .        .        .        .        .
Vv07g0875         .        .        .        .        .        .        .
         .        .        .        .        .        .        .        .
         .        .        .        .        .
Vv07g0876         .        .        .        .        .        .        .
         .        .        .        .        .        .        .        .
         .        .        .        .        .
Vv07g0877         .        .        .        .        .        .        .
         .        .        .        .        .        .        .        .
         .        .        .        .        .
Vv07g0878         .        .        .        .        .        .        .
         .        .        .        .        .        .        .        .
         .        .        .        .        .
Vv07g0879         Tc01_g023510      .        .        .        .        .
         .        .        .        .        .        .        .        .
         .        .        .        .        .        .
Vv07g0880         .        Gorai.007G151600.1         .        .        .
         .        .        .        .        .        .        .        .
         .        .        .        .        .        .        .
Vv07g0881         .        .        Gorai.001G235400.1         .        .
         Gorai.004G223500.1         .        .        .        .        .
         .        .        .        .        .        .        .        .
         .
Vv07g0882         .        .        Gorai.001G235100.1         .        .
         Gorai.004G223600.1         .        .        .        .        .
         .        .        .        .        .        .        .        .
         .
Vv07g0883         .        .        .        .        .        .        .
         .        .        .        .        .        .        .        .
         .        .        .        .        .
Vv07g0884         .        .        .        .        .        .        .
         .        .        .        .        .        .        .        .
         .        .        .        .        .
Vv07g0885         .        .        .        .        .        .        .
         .        .        .        .        .        .        .        .
         .        .        .        .        .
Vv07g0886         .        .        .        .        .        .        .
         .        .        .        .        .        .        .        .
         .        .        .        .        .
Vv07g0887         .        .        .        .        .        .        .
         .        .        .        .        .        .        .        .
         .        .        .        .        .
Vv07g0888         .        .        .        .        .        .        .
         .        .        .        .        .        .        .        .
         .        .        .        .        .
Vv07g0889         Tc01_g007370      .        .        .        Gorai.
```

003G035600.1 . Vv18g0650 . . . .
. . . . . . . .
.
Vv07g0890 Tc01_g007360 . . . Gorai.
003G035500.1 . . . . . . .
. . . . . . . .
Vv07g0891 Tc01_g007350 . . . Gorai.
003G035300.1 . . . . . . .
. . . . . . . .
Vv07g0892 . . . . . . .
. . . . . . . .
. . . . .
Vv07g0893 Tc01_g007340 . Gorai.008G087900.1
Gorai.007G229400.1 Gorai.003G035200.1 . . .
. . . . . . . .
. . . .
Vv07g0894 . . . . . . .
. . . . . . . .
. . . . .
Vv07g0895 Tc01_g007300 . . . Gorai.
003G034900.1 . . . . . . .
. . . . . . . .
Vv07g0896 Tc01_g007290 . Gorai.008G087800.1
Gorai.007G229300.1 Gorai.003G034800.1 . . .
. . . . . . . .
. . . .
Vv07g0897 . . . . . . .
. . . . . . . .
. . . . .
Vv07g0898 . . . . . . .
. . . . . . . .
. . . . .
Vv07g0899 Tc01_g036000 . . . . .
. . . . . . . .
. . . . . .
Vv07g0900 . . Gorai.008G087600.1 Gorai.
007G229000.1 Gorai.003G034700.1 . . . .
. . . . . . . .
. . .
Vv07g0901 Tc01_g007250 . . . . .
. . . . . . . .
. . . . . .
Vv07g0902 . . . Gorai.007G228900.1
Gorai.003G034600.1 . . . . . .
. . . . . . . .
.
Vv07g0903 . . . . . . .
. . . . . . . .
. . . . .
Vv07g0904 Tc01_g007240 . . . Gorai.

003G034600.1 . . . . . . .
. . . . . . . .
Vv07g0905 . Gorai.007G057800.1 . .
Gorai.003G034700.1 . . . . . .
. . . . . . . .
.
Vv07g0906 Tc01_g007230 Gorai.007G057600.1 . .
. . . . . . . .
. . . . . . . .
Vv07g0907 Tc01_g007220 . . . Gorai.
003G034500.1 . . . . . . .
. . . . . . . .
Vv07g0908 Tc01_g035730 . Gorai.008G120700.1 .
. . . . . . . .
. . . . . . . .
Vv07g0909 Tc01_g007210 . Gorai.001G234000.1 .
Gorai.003G034300.1 . . . . .
. . . . . . . .
. .
Vv07g0910 Tc01_g007200 . Gorai.008G087500.1
Gorai.007G228700.1 Gorai.003G034200.1 . . .
. . . . . . . .
. . . .
Vv07g0911 . . . . . . .
. . . . . . . .
. . . . .
Vv07g0912 Tc01_g007180 . Gorai.008G087400.1 .
. . Vv03g0870 . . . .
. . Vv18g0665 . . Gorai.
005G097800.1 Gorai.009G224800.1 . .
Vv07g0913 Tc01_g007160 . . Gorai.007G228500.1
Gorai.003G034100.1 . Vv03g0882
Tc02_g010670 . . . Gorai.008G017200.1 .
. . . . . . .
Vv07g0914 Tc01_g007150 . . Gorai.007G228300.1
Gorai.003G034000.1 . . . . .
. . . . . . . .
. .
Vv07g0915 Tc01_g007140 . Gorai.008G087300.1 .
Gorai.003G033900.1 . Vv03g0894
Tc02_g002350 . Gorai.005G262800.1 . Gorai.
008G016500.1 . . . . . . .
.
Vv07g0916 Tc01_g007130 . Gorai.008G087200.1 .
. . . . . . . .
. . . . . . . .
Vv07g0917 Tc01_g007120 Gorai.007G057500.1 Gorai.
008G087000.1 Gorai.007G228200.1 Gorai.003G033800.1 .
. . . . . . .
Vv18g0677 . . . . . .

Vv07g0918 Tc01_g007070 . . . Gorai.
003G033300.1 . . . . . . .
. . . . . . . .
Vv07g0919 . . . . . .
Vv03g0901 . Gorai.013G054100.1 . . .
. . . . . . . .
Vv07g0920 Tc01_g007060 . . Gorai.007G227800.1
Gorai.003G033100.1 . . . . .
. . . . . . . .
. .
Vv07g0921 . . . . . . .
. . . . . . . .
. . . . .
Vv07g0922 . . . . . . .
. . . . . . . .
. . . . .
Vv07g0923 . . . . . . .
. . . . . . . .
. . . . .
Vv07g0924 Tc01_g007050 . Gorai.008G086600.1
Gorai.007G227600.1 . . . . . .
. . . Vv18g0683 . . .
. Gorai.009G277800.1 .
Vv07g0925 . . . . . . .
. . . . . . . .
. . . . .
Vv07g0926 Tc01_g007010 . . Gorai.007G227300.1
Gorai.003G032800.1 . Vv03g0924
Tc02_g002470 Gorai.002G212400.1 . . . .
Vv18g0693 . . . Gorai.009G222600.1
. .
Vv07g0927 Tc01_g007000 . . Gorai.007G227000.1
. . . . . . . .
. . . . . . . .
Vv07g0928 . . . . . . .
. . . . . . . .
. . . . .
Vv07g0929 . . . . . . .
. . . . . . . .
. . . . .
Vv07g0930 Tc01_g006990 . . . Gorai.
003G032700.1 . Vv03g0926 Tc02_g002480 Gorai.
002G212300.1 Gorai.005G262700.1 . Gorai.008G043300.1
. . . . . . . .
Vv07g0931 Tc01_g006980 . . . Gorai.
003G032600.1 . . . . . . .
. . . . . . . .
Vv07g0932 Tc01_g006970 . Gorai.008G086200.1 .
. . . . . . . .
. . . . . . . .

```
Vv07g0933	Tc01_g006960	.	.	Gorai.007G226900.1
	Gorai.003G032500.1	.	Vv03g0938
Tc02_g002530	.	Gorai.005G262400.1	.	.
Gorai.012G162200.1	Vv18g0695	.	.	.
Gorai.009G222400.1	.	Gorai.010G028500.1
Vv07g0934	Tc01_g006940	.	.	Gorai.007G226800.1
	.	.	.	.	.	.	.	.
	.	.	.	.	.	.	.	.
Vv07g0935	Tc01_g006930	.	Gorai.008G086100.1
Gorai.007G226700.1	Gorai.003G032400.1	.	.	.
	.	.	.	.	.	.	.	.
	.	.	.	.
Vv07g0936	.	.	.	.	.	.	.
	.	.	.	.	.	.	.	.
	.	.	.	.	.
Vv07g0937	Tc01_g006920	.	Gorai.008G086000.1
Gorai.007G226500.1	Gorai.003G037100.1	Gorai.011G109500.1
	.	.	.	.	.	.	.	.
	.	.	.	.	.	.
Vv07g0938	.	.	.	.	.	.	.
	.	.	.	.	.	.	.	.
	.	.	.	.	.
Vv07g0939	Tc01_g006800	.	.	Gorai.007G225900.1
	.	.	Vv18g0713	.	.	.	.
	.	Gorai.010G026600.1	.	.	.	.
	.	.	.
Vv07g0940	Tc01_g006790	.	.	Gorai.007G225800.1
	Gorai.003G031800.1	.	.	.	.	.
	.	.	.	.	.	.	.	.
	.	.
Vv07g0941	Tc01_g006780	.	.	Gorai.007G225700.1
	Gorai.003G031700.1	.	Vv18g0714	.	.
	.	Gorai.009G221400.1	.	Gorai.010G026500.1
	.	.	.	.	.	.	.
Vv07g0942	.	.	.	.	.	.	.
	.	.	.	.	.	.	.	.
	.	.	.	.	.
Vv07g0943	Tc01_g006770	.	.	Gorai.007G225600.1
	.	.	.	.	.	.	.	.
	.	.	.	.	.	.	.	.
Vv07g0944	Tc01_g006750	.	Gorai.008G084500.1
Gorai.007G187800.1	.	.	Vv18g0715	.	.
	.	.	Gorai.009G277400.1	Gorai.010G026400.1
	.	.	.	.	.	.	.
Vv07g0945	Tc01_g006710	.	.	Gorai.007G224900.1
	.	.	.	.	.	.	.	.
	.	.	.	.	.	.	.	.
Vv07g0946	.	.	.	.	.	.
Vv03g0815	.	Gorai.013G050900.1	Gorai.005G230300.1
	.	.	.	.	.	.	.	.
```

. .
Vv07g0947 . . . . . . .
. . . . . . . .
. . . . .
Vv07g0948 Tc01_g006700 . Gorai.008G084100.1
Gorai.007G224800.1 Gorai.003G031400.1 . . .
. . . . . . . .
. . . .
Vv07g0949 Tc01_g006690 Gorai.007G056700.1 .
Gorai.007G224700.1 Gorai.003G031300.1 . . .
. . . . . Vv18g0725 .
. . . . .
Vv07g0950 . . . . . . .
. . . . . . . .
. . . . .
Vv07g0951 Tc01_g006680 . . Gorai.007G224600.1
Gorai.003G031200.1 . . . . .
. . . Vv18g0744 . . .
Gorai.009G219400.1 . .
Vv07g0952 Tc01_g006670 . . Gorai.007G224500.1
Gorai.003G031100.1 . . . . .
. . . . . . . .
. .
Vv07g0953 Tc01_g006650 . Gorai.008G083300.1 .
. . . . . . . .
. . . . . . . .
Vv07g0954 . . . . . . .
. . . . . . . .
. . . . .
Vv07g0955 . . . . . . .
. . . . . . . .
. . . . .
Vv07g0956 Tc01_g006630 . . . . .
. . . . . . . .
. . . . . .
Vv07g0957 . . . Gorai.007G224300.1
Gorai.003G030900.1 Gorai.011G108300.1 . . .
. . . . . . . .
. . .
Vv07g0958 Tc01_g006620 Gorai.007G056600.1 . .
. . . . . . . .
. . . . . . . .
Vv07g0959 Tc01_g006600 . . . .
Gorai.011G108200.1 . . . . . .
. . . . . . . .
Vv07g0960 . . . . . . .
. . . . . . . .
. . . . .
Vv07g0961 . . . . . . .
. . . . . . . .

. . . . .
Vv07g0962 Tc01_g006510 . . Gorai.007G223200.1
Gorai.003G030100.1 . . . . .
. . . . . . . .
. .
Vv07g0963 Tc01_g006500 . Gorai.008G083000.1 .
. . . . . . . .
. . . . . . . .
Vv07g0964 Tc01_g006490 Gorai.007G056500.1 .
Gorai.007G223100.1 Gorai.003G030000.1 Gorai.011G108100.1
. . . . . . . .
. . . . . .
Vv07g0965 Tc01_g006480 . Gorai.008G082900.1
Gorai.007G223000.1 . . Vv03g0795 . .
. . . . . . . .
. . .
Vv07g0966 Tc01_g006470 . . . Gorai.
003G029900.1 Gorai.011G108000.1 . . . .
. . . . . . . .
. .
Vv07g0967 Tc01_g006460 . . Gorai.007G222900.1
. . . . . . . .
. . . . . . . .
Vv07g0968 Tc01_g006440 Gorai.007G056400.1 .
Gorai.007G222700.1 Gorai.003G029700.1 . . .
. . . . . . . .
. . . .
Vv07g0969 Tc01_g006430 . Gorai.008G082700.1
Gorai.007G222600.1 . Gorai.011G105000.1 . .
. . . . . . . .
. . . .
Vv07g0970 Tc01_g006420 . Gorai.008G082600.1 .
. . . . . . . .
. . . . . . . .
Vv07g0971 . . . . . . .
. . . . . . . .
. . . . .
Vv07g0972 . . . . . . .
. . . . . . . .
. . . . .
Vv07g0973 Tc01_g006380 . . Gorai.007G222400.1
Gorai.003G039100.1 . Vv03g0779
Tc02_g010110 Gorai.013G050000.1 Gorai.005G232400.1 .
. . . . . . . .
.
Vv07g0974 Tc01_g006370 . Gorai.008G082400.1
Gorai.007G222300.1 Gorai.003G029400.1 . . .
. . . . . . . .
. . . .
Vv07g0975 Tc01_g006360 . . Gorai.007G222200.1

Gorai.003G029300.1 . . . . .
. . . . . . . .
. .
Vv07g0976 Tc01_g006340 . . . . .
. . . . . . . .
. . . . . .
Vv07g0977 Tc01_g006330 . . . . .
. . . . . . . .
. . . . . .
Vv07g0978 Tc01_g006320 . . Gorai.007G222100.1
. . . . . . . .
. . . . . . . .
Vv07g0979 . . . . . . .
. . . . . . . .
. . . . .
Vv07g0980 Tc01_g006310 . . . . .
. . . . . . . .
. . . . . .
Vv07g0981 . . . . . . .
. . . . . . . .
. . . . .
Vv07g0982 Tc01_g006300 Gorai.007G056300.1 Gorai.
008G081100.1 Gorai.007G222000.1 . . Vv03g0778
Tc02_g010100 . Gorai.005G232500.1 . .
. Vv18g0751 . . . Gorai.
009G218800.1 . Gorai.010G025100.1
Vv07g0983 Tc01_g006290 Gorai.007G056200.1 .
Gorai.007G221900.1 . . . . . .
. . . . . . . .
. .
Vv07g0984 . . . . . . .
. . . . . . . .
. . . . .
Vv07g0985 . . Gorai.008G080900.1 Gorai.
007G221800.1 Gorai.003G029100.1 . Vv03g0772
Tc02_g010010 . . Gorai.002G010000.1 . .
. . . . . . .
Vv07g0986 Tc01_g006280 . Gorai.008G080800.1 .
Gorai.003G029000.1 . . . . .
. . . . . . . .
. .
Vv07g0987 Tc01_g006270 . . Gorai.007G221700.1
Gorai.003G028900.1 . . . . .
. . . . . . . .
. .
Vv07g0988 Tc01_g006260 . Gorai.008G080700.1
Gorai.007G221600.1 . . . . . .
. . . . . . . .
. .
Vv07g0989 Tc01_g006250 . Gorai.008G080600.1 .

. . . . . . . .
. . . . . . . .
Vv07g0990 Tc01_g006240 Gorai.007G056100.1 Gorai.
008G080500.1 . . . . . . .
. . . . . . . .
. .
Vv07g0991 Tc01_g006230 . Gorai.008G080400.1 .
Gorai.003G028800.1 . . . . .
. . . Vv18g0759 . . .
Gorai.009G218200.1 Gorai.009G274000.1 Gorai.
010G024800.1
Vv07g0992 Tc01_g006220 . Gorai.008G080300.1
Gorai.007G221500.1 . . . . . .
. . . Vv18g0760 . . .
. . Gorai.010G024700.1
Vv07g0993 Tc01_g006210 . Gorai.008G080200.1
Gorai.007G221400.1 . . . . . .
. . . . . . . .
. .
Vv07g0994 Tc01_g006200 . . Gorai.007G190600.1
. . Vv03g0763 . . .
Gorai.002G009900.1 . . . . . .
. . .
Vv07g0995 Tc01_g006180 . Gorai.008G080100.1 .
. . . . . . . .
. . . . . . . .
Vv07g0996 Tc01_g006170 . . . . .
. . . . . . . .
. . . . . .
Vv07g0997 Tc01_g006160 . . . . .
. . . . . . . .
. . . . . .
Vv07g0998 . . . . Gorai.003G028700.1
. . . . . . . .
. . . . . . .
Vv07g0999 Tc01_g006150 . Gorai.008G080000.1
Gorai.007G220900.1 Gorai.003G028600.1 . . .
. . . . . . . .
. . . .
Vv07g1000 Tc01_g006140 . . Gorai.007G190900.1
. . Vv03g0759 Tc02_g009910 Gorai.
013G049000.1 Gorai.005G233000.1 . . .
Vv18g0765 . . . . . .
Vv07g1001 Tc01_g006130 . . . Gorai.
003G028500.1 . . . . . . .
. . . . . . . .
Vv07g1002 Tc01_g006110 . . Gorai.007G220700.1
. . . . . . . .
. . . . . . . .
Vv07g1003 Tc01_g006100 . . Gorai.007G220600.1

.	.	Vv18g0770	.	.	.	.
.	Gorai.010G024500.1	.	.	.	.
.	.	.
Vv07g1004	.	.	.	.	.	.	.
.	.	.	.	.	.	.	.
.	.	.	.	.
Vv07g1005	.	.	.	.	.	.	.
.	.	.	.	.	.	.	.
.	.	.	.	.
Vv07g1006	Tc01_g006080	.	.	.	Gorai.
003G028400.1	.	.	.	.	.	.	.
.	.	.	.	.	.	.	.
Vv07g1007	Tc01_g006070	.	.	Gorai.007G220200.1
Gorai.003G028300.1	.	.	.	.	.
.	.	.	.	.	.	.	.
.	.
Vv07g1008	Tc01_g006040	.	Gorai.008G079700.1
Gorai.007G219900.1	Gorai.003G028100.1	.	.	.
.	.	.	.	.	.	.	.
.	.	.	.
Vv07g1009	Tc01_g006030	Gorai.007G056000.1	.
Gorai.007G191200.1	Gorai.003G027900.1	.	Vv18g0773
.	Gorai.002G132900.1	.	Gorai.009G217300.1
.	.	.	.	.	.	.	.
.
Vv07g1010	Tc01_g006020	.	.	.	.	.
.	.	.	.	.	.	.	.
.	.	.	.	.	.
Vv07g1011	Tc01_g006010	.	Gorai.008G079600.1
Gorai.007G219700.1	Gorai.003G027800.1	.	Vv18g0775
.	.	.	Gorai.009G217100.1	.	.
.	.	.	.	.	.	.
Vv07g1012	Tc01_g006000	.	.	.	Gorai.
003G027700.1	.	Vv18g0779	.	.	Gorai.
005G100500.1	.	.	.	.	.	.	.
.	.	.
Vv07g1013	Tc01_g005980	.	.	Gorai.007G219600.1
.	.	Vv18g0781	.	.	.	.
.	.	.	.	.	.	.	.
.
Vv07g1014	.	.	.	.	.	.	.
.	.	.	.	.	.	.	.
.	.	.	.	.
Vv07g1015	Tc01_g005970	Gorai.007G055900.1	Gorai.
008G076600.1	Gorai.007G219500.1	.	.	.	.
.	.	.	.	.	.	.	.
.	.	.	.
Vv07g1016	Tc01_g005950	.	.	.	.	.
.	.	.	.	.	.	.	.
.	.	.	.	.	.

Vv07g1017 . . . . Gorai.003G027600.1
. . . . . . . .
. . . . . . .
Vv07g1018 Tc01_g005940 Gorai.007G055800.1 .
Gorai.007G219300.1 . . . . . .
. . . . . . . .
. .
Vv07g1019 . . . . . . .
. . . . . . . .
. . . . .
Vv07g1020 . . . . . . .
. . . . . . . .
. . . . .
Vv07g1021 . . . . . . .
. . . . . . . .
. . . . .
Vv07g1022 . . . . . . .
. . . . . . . .
. . . . .
Vv07g1023 . . . . . . .
. . . . . . . .
. . . . .
Vv07g1024 Tc01_g005930 . . . . .
. . . . . . . .
. . . . . .
Vv07g1025 . . . . . . .
. . . . . . . .
. . . . .
Vv07g1026 . . . . . . .
. . . . . . . .
. . . . .
Vv07g1027 Tc01_g005910 . . . . .
. . . . . . . .
. . . . . .
Vv07g1028 . . . . . . .
. . . . . . . .
. . . . .
Vv07g1029 . . . . . . .
. . . . . . . .
. . . . .
Vv07g1030 Tc01_g005890 . . Gorai.007G219200.1
. . . . . . . .
. . . . . . . .
Vv07g1031 . . . . . . .
. . . . . . . .
. . . . .
Vv07g1032 Tc01_g005880 . . . . .
. . . . . . . .
. . . . . .
Vv07g1033 . . . . . . .

| | | | | | | | |
|---|---|---|---|---|---|---|---|
| . | . | . | . | . | . | . | . |
| . | . | . | . | . | | | |
| Vv07g1034 | . | . | . | . | . | . | . |
| . | . | . | . | . | . | . | . |
| . | . | . | . | . | | | |
| Vv07g1035 | . | . | . | . | . | . | . |
| . | . | . | . | . | . | . | . |
| . | . | . | . | . | | | |
| Vv07g1036 | Tc01_g005870 | | . | . | Gorai.007G219100.1 | | |
| . | . | . | . | . | . | . | . |
| . | . | . | . | . | . | . | . |
| Vv07g1037 | . | . | . | . | . | . | . |
| . | . | . | . | . | . | . | . |
| . | . | . | . | . | | | |
| Vv07g1038 | . | . | . | . | . | . | |
| Vv18g0250 | . | . | . | . | . | . | . |
| . | . | . | . | . | . | | |
| Vv07g1039 | . | . | . | . | . | . | . |
| . | . | . | . | . | . | . | . |
| . | . | . | . | . | | | |
| Vv07g1040 | . | . | . | . | . | . | . |
| . | . | . | . | . | . | . | . |
| . | . | . | . | . | | | |
| Vv07g1041 | . | . | . | . | . | . | . |
| . | . | . | . | . | . | . | . |
| . | . | . | . | . | | | |
| Vv07g1042 | Tc01_g005840 | | . | . | Gorai.007G219000.1 | | |
| . | . | . | . | . | . | . | . |
| . | . | . | . | . | . | . | . |
| Vv07g1043 | . | . | . | . | . | . | . |
| . | . | . | . | . | . | . | . |
| . | . | . | . | . | | | |
| Vv07g1044 | . | . | . | . | . | . | . |
| . | . | . | . | . | . | . | . |
| . | . | . | . | . | | | |
| Vv07g1045 | . | . | . | . | . | . | . |
| . | . | . | . | . | . | . | . |
| . | . | . | . | . | | | |
| Vv07g1046 | . | . | . | . | . | . | . |
| . | . | . | . | . | . | . | . |
| . | . | . | . | . | | | |
| Vv07g1047 | Tc01_g005830 | | . | . | Gorai.007G218700.1 | | |
| . | . | . | . | . | . | . | . |
| . | . | . | . | . | . | . | . |
| Vv07g1048 | . | . | . | . | . | . | . |
| . | . | . | . | . | . | . | . |
| . | . | . | . | . | | | |
| Vv07g1049 | Tc01_g005820 | | . | . | . | . | . |
| . | . | . | . | . | . | . | . |
| . | . | . | . | . | . | | |

Vv07g1050 Tc01_g005810 . . Gorai.007G218600.1
. . . . . . . .
. . . . . . . .
Vv07g1051 . . . . . . .
. . . . . . . .
. . . . .
Vv07g1052 Tc01_g005800 . . Gorai.007G218500.1
Gorai.003G027400.1 . . . . .
. . . . . . . .
. .
Vv07g1053 . . . . . . .
. . . . . . . .
. . . . .
Vv07g1054 Tc01_g005750 . . . . .
Vv18g0797 . . . . . .
. . . . . . .
Vv07g1055 . . . . . . .
. . . . . . . .
. . . . .
Vv07g1056 . Gorai.007G055700.1 . . .
. Vv18g0798 . . . . .
. . . . . . . .
Vv07g1057 Tc01_g005730 . . Gorai.007G218300.1
. . Vv03g0748 Tc02_g009490 . .
. . . . . . . .
. .
Vv07g1058 Tc01_g005720 . . Gorai.007G218200.1
. . . . . . . .
. . . . . . . .
Vv07g1059 Tc01_g005710 Gorai.007G055600.1 .
Gorai.007G218100.1 . . . . . .
. . . . . . . .
. .
Vv07g1060 Tc01_g005690 . . Gorai.007G218000.1
. . . . . . . .
. . . . . . . .
Vv07g1061 Tc01_g005680 . . . Gorai.
003G027300.1 . . . . . . .
. . . . . . . .
Vv07g1062 Tc01_g005670 . . . Gorai.
003G027200.1 . . . . . . .
. . . . . . . .
Vv07g1063 . . . . . . .
. . . . . . . .
. . . . .
Vv07g1064 Tc01_g005660 . . Gorai.007G217400.1
. . . . . . . .
. . . . . . . .
Vv07g1065 Tc01_g005650 . . . Gorai.
003G027100.1 Gorai.011G106700.1 Vv03g0725

Tc02_g009300	Gorai.013G046400.1	Gorai.005G235600.1	.
.	Gorai.012G149300.1	Vv18g0816	.
Gorai.002G133500.1	Gorai.005G101100.1	Gorai.009G215600.1
.	.
Vv07g1066	.	.	.	.	Gorai.003G027000.1
Gorai.011G106600.1	.	.	.	.	.
.	.	.	.	.	.	.	.
.
Vv07g1067	Tc01_g005640	.	.	.	.	.
.	.	.	.	.	.	.	.
.	.	.	.	.	.
Vv07g1068	.	.	.	.	.	.	.
.	.	.	.	.	.	.	.
.	.	.	.	.
Vv07g1069	Tc01_g005630	Gorai.007G055500.1	.	.
.	.	Vv03g0723	Tc02_g009270	.
Gorai.005G235800.1	Gorai.002G008700.1	Gorai.008G023600.1
.	.	.	.	.	.	.	.
Vv07g1070	Tc01_g005620	.	.	Gorai.007G217100.1
Gorai.003G026900.1	.	.	.	.	.
.	.	.	.	.	.	.	.
.	.
Vv07g1071	Tc01_g005610	.	.	Gorai.007G217000.1
.	.	.	.	.	.	.	.
.	.	.	.	.	.	.	.
Vv07g1072	Tc01_g005600	.	.	.	.	.
Vv03g0722	Tc02_g009260	.	Gorai.005G235900.1
.	.	.	Vv18g0822	Tc08_g000020
Gorai.002G133800.1	.	Gorai.009G214900.1	.
Gorai.010G022700.1
Vv07g1073	.	.	.	Gorai.007G216900.1	.
.	Vv03g0721	.	.	.	.	.
.	.	.	.	.	.	.	.
Vv07g1074	.	.	.	.	.	.	.
.	.	.	.	.	.	.	.
.	.	.	.	.
Vv07g1075	Tc01_g005590	.	.	Gorai.007G216800.1
Gorai.003G026800.1	.	.	.	.	.
.	.	.	.	.	.	.	.
.	.
Vv07g1076	Tc01_g005580	.	.	Gorai.007G216700.1
Gorai.003G026600.1	.	.	.	.	.
.	.	.	Vv18g0825	Tc08_g000030	.
.	Gorai.009G214800.1	.	Gorai.010G022600.1
Vv07g1077	Tc01_g005570	.	.	.	Gorai.
003G026500.1	.	.	.	.	.	.	.
.	.	.	.	.	.	.	.
Vv07g1078	Tc01_g005550	.	.	.	.	.
.	.	.	.	.	.	.	.
.	.	.	.	.	.

Vv07g1079 Tc01_g005530 Gorai.007G055100.1 .
Gorai.007G216600.1 . . Vv03g0719
Tc02_g009190 . Gorai.005G236300.1 . . .
. . . . . . .
Vv07g1080 Tc01_g005520 . . Gorai.007G216500.1
Gorai.003G026300.1 . . . . .
. . . . . . . .
. .
Vv07g1081 Tc01_g005510 . . Gorai.007G216400.1
Gorai.003G026200.1 . . . . .
. . . Vv18g0829 Tc08_g000080 .
. Gorai.009G214600.1 . Gorai.010G022400.1
Vv07g1082 Tc01_g005500 . . Gorai.007G216300.1
. . . . . . . .
. Vv18g0831 Tc08_g000090 . .
Gorai.009G214500.1 . .
Vv07g1083 Tc01_g005490 . . Gorai.007G216200.1
. . . . . . . .
. . . . . . . .
Vv07g1084 Tc01_g005480 . . . Gorai.
003G026100.1 . . . . . . .
. . . . . . . .
Vv07g1085 Tc01_g005470 . . . Gorai.
003G026000.1 . . . . . . .
. . . . . . . .
Vv07g1086 Tc01_g005460 Gorai.007G054700.1 . .
. . . . . . . .
. . . . . . . .
Vv07g1087 Tc01_g005440 . . Gorai.007G215900.1
Gorai.003G025900.1 Gorai.011G106200.1 . .
. . . . . . . .
. . . .
Vv07g1088 . . . . . .
Vv03g0713 Tc02_g009070 Gorai.013G045900.1 Gorai.
005G236600.1 . . . . . . .
. . .
Vv07g1089 . . . . . . .
. . . . . . . .
. . . . .
Vv07g1090 . . . . . . .
. . . . . . . .
. . . . .
Vv07g1091 Tc01_g005430 . . . . .
. . . . . . . .
. . . . . .
Vv07g1092 . . . . . . .
. . . . . . . .
. . . . .
Vv07g1093 Tc01_g005400 . . . Gorai.
003G025700.1 . . . . . . .

. . . . . . . .
Vv07g1094 . . . Gorai.007G215800.1 .
. Vv03g0712 Tc02_g009060 Gorai.013G045800.1
Gorai.005G236700.1 . . . . .
. . . . .
Vv07g1095 Tc01_g005390 . . Gorai.007G215700.1
. . Vv03g0711 Tc02_g009050 Gorai.
013G045700.1 . . . . . . .
. . . .
Vv07g1096 . . . . . . .
. . . . . . . .
. . . . .
Vv07g1097 Tc01_g005380 . . Gorai.007G215600.1
. . . . . . . .
. Vv18g0837 Tc08_g000160 . . .
. Gorai.010G021700.1
Vv07g1098 Tc01_g005370 . . Gorai.007G215500.1
. . . . . . . .
. Vv18g0840 Tc08_g000170 . . .
. Gorai.010G021400.1
Vv07g1099 . . . Gorai.007G215400.1 .
. . . . . . . .
Vv18g0843 . . . . .
Gorai.010G021300.1
Vv07g1100 Tc01_g005340 . . Gorai.007G215300.1
. . . . . . . .
. . . . . . . .
Vv07g1101 Tc01_g005330 . . . . .
. . . . . . .
Vv18g0844 . . . . . Gorai.
010G021200.1
Vv07g1102 Tc01_g005320 . . Gorai.007G215200.1
. Gorai.011G106100.1 . . . .
. . . . . . . .
. .
Vv07g1103 Tc01_g005310 . Gorai.008G077600.1 .
Gorai.003G025600.1 . . . . .
. . . . . . . .
. .
Vv07g1104 Tc01_g005300 . Gorai.008G077500.1 .
Gorai.003G025500.1 . . . . .
. . . . . . . .
. .
Vv07g1105 Tc01_g005290 . . . . .
Vv03g0706 Tc02_g008990 Gorai.013G045300.1
Gorai.005G237100.1 . Gorai.008G024000.1 Gorai.
012G149800.1 . . . . . . .
Vv07g1106 . . . . . . .
. . . . . . . .
. . . . .

Vv07g1107 . . Gorai.008G077400.1 Gorai.
007G215000.1 . . . . . . .
. . . . . . . .
.
Vv07g1108 . . . . . . .
. . . . . . . .
. . . . .
Vv07g1109 . . . Gorai.007G214900.1 .
. . . . . . . .
. . . . . . .
Vv07g1110 . . . . . . .
. . . . . . . .
. . . . .
Vv07g1111 . . . . . . .
. . . . . . . .
. . . . .
Vv07g1112 . . . . . . .
. . . . . . . .
. . . . .
Vv07g1113 Tc01_g005280 . . . . .
. . . . . . . .
. . . . . .
Vv07g1114 Tc01_g005270 . Gorai.008G077300.1
Gorai.007G214800.1 . . . . . .
. . . . . . . .
. .
Vv07g1115 Tc01_g005260 . Gorai.008G077200.1
Gorai.007G214700.1 . . . . . .
. . . . . . . .
. .
Vv07g1116 Tc01_g005250 . . Gorai.007G214600.1
. . . . . . . .
. . . . . . . .
Vv07g1117 Tc01_g005240 . . Gorai.007G214500.1
. . . . . . . .
. . . . . . . .
Vv07g1118 Tc01_g005230 . . Gorai.007G214400.1
. . Vv03g0701 Tc02_g008940 Gorai.
013G044900.1 . . Gorai.008G024200.1 . .
. . . . . .
Vv07g1119 Tc01_g005220 . . Gorai.007G214100.1
. . . . . . . .
. . . . . . . .
Vv07g1120 Tc01_g005200 . Gorai.008G077100.1
Gorai.007G214000.1 . . Vv18g0859
Tc08_g000280 . Gorai.005G101900.1 . .
Gorai.010G020900.1 . . . . . .
.
Vv07g1121 Tc01_g005190 . Gorai.008G077000.1
Gorai.007G213900.1 Gorai.003G025400.1 Gorai.011G106000.1

.	.	.	.	.	.	.	.
.	.	.	.	.	.
Vv07g1122	Tc01_g005180	.	Gorai.008G076900.1
Gorai.007G213800.1	.	Gorai.011G105900.1	.	.
.	.	.	.	.	.	.	.
.	.	.	.
Vv07g1123	Tc01_g005170	.	.	Gorai.007G213700.1
Gorai.003G025300.1	.	.	.	.	.
.	.	.	.	.	.	.	.
.	.
Vv07g1124	Tc01_g005160	Gorai.007G054600.1	.	.
.	.	.	.	.	.	.	.
.	.	.	.	.	.	.	.
Vv07g1125	.	.	.	.	.	.	.
.	.	.	.	.	.	.	.
.	.	.	.	.
Vv07g1126	Tc01_g005150	Gorai.007G054500.1	.	.
.	.	.	.	.	.	.	.
.	.	.	.	.	.	.	.
Vv07g1127	Tc01_g005120	Gorai.007G054400.1	.	.
.	.	Vv18g0864	Tc08_g000340	.
Gorai.005G102200.1	.	.	.	.	.	.
.	.	.	.
Vv07g1128	.	.	.	Gorai.007G213600.1	.
.	.	.	.	.	.	.	.
.	.	.	.	.	.	.
Vv07g1129	Tc01_g005080	.	Gorai.008G076700.1	.
.	.	.	.	.	.	.	.
.	.	.	.	.	.	.	.
Vv07g1130	Tc01_g005070	.	Gorai.008G076600.1	.
.	.	Vv18g0865	Tc08_g000350	.	.
Gorai.009G212800.1	.	Gorai.010G020400.1	.
.	.	.	.	.	.
Vv07g1131	Tc01_g005060	.	.	Gorai.007G213500.1
.	.	.	.	.	.	.	.
.	.	.	.	.	.	.	.
Vv07g1132	Tc01_g005050	.	Gorai.008G076000.1
Gorai.007G213400.1	Gorai.003G025200.1	.	.	.
.	.	.	.	.	.	.	.
.	.	.	.
Vv07g1133	.	.	.	.	.	.	.
.	.	.	.	.	.	.	.
.	.	.	.	.
Vv07g1134	Tc01_g005040	.	.	Gorai.007G213300.1
Gorai.003G025100.1	.	.	.	.	.
.	.	.	.	.	.	.	.
.	.
Vv07g1135	.	.	.	.	.	.
Vv18g0873	Tc08_g000400	.	Gorai.005G102400.1	.
.	Gorai.010G020200.1	.	.	.	.

. . .
Vv07g1136 Tc01_g004980 . Gorai.008G075900.1
Gorai.007G213000.1 Gorai.003G024600.1 Gorai.011G105800.1
. . . . . . . .
. . . . . .
Vv07g1137 Tc01_g004970 Gorai.007G053900.1 . .
Gorai.003G023500.1 Gorai.011G105700.1 . .
. . . . . . . .
. . . .
Vv07g1138 Tc01_g004960 . . Gorai.007G212700.1
. . . . . . . .
. . . . . . . .
Vv07g1139 . . . . . . .
. . . . . . . .
. . . . .
Vv07g1140 . . . . . . .
. . . . . . . .
. . . . .
Vv07g1141 Tc01_g004950 . . . Gorai.
003G023300.1 Gorai.011G105500.1 . . . .
. . . . . . . .
. .
Vv07g1142 Tc01_g004940 . . Gorai.007G212600.1
. . . . . . . .
. . . . . . . .
Vv07g1143 Tc01_g004930 . Gorai.008G075600.1 .
Gorai.003G023200.1 . . . . .
. . . . . . . .
. .
Vv07g1144 Tc01_g004910 . Gorai.008G075400.1 .
. . . . . . . .
. . . . . . . .
Vv07g1145 Tc01_g004900 . Gorai.008G075300.1 .
. . . . . . . .
. . . . . . . .
Vv07g1146 Tc01_g004890 . . Gorai.007G212400.1
Gorai.003G023100.1 . . . . .
. . . . . . . .
. .
Vv07g1147 Tc01_g004860 . Gorai.008G074900.1
Gorai.007G212200.1 . . . . . .
. . . . . . . .
. .
Vv07g1148 Tc01_g004850 . . Gorai.007G212100.1
. . Vv18g0898 Tc08_g000720 Gorai.
002G128700.1 . . . . . . .
. . . .
Vv07g1149 Tc01_g004840 . . Gorai.007G211900.1
. . . . . . . .
. . . . . . . .

Vv07g1150 . . . . . . .
. . . . . . . .
. . . . .
Vv07g1151 Tc01_g004820 . . . . .
. . . . . . . .
. . . . . .
Vv07g1152 . . Gorai.008G072100.1 Gorai.
007G211700.1 . . . . . . .
. . . . . . . .
.
Vv07g1153 Tc01_g004810 Gorai.007G051000.1 .
Gorai.007G211600.1 . Gorai.011G105000.1 Vv18g0908
. . . . . Gorai.010G017600.1
. . . . . . .
Vv07g1154 Tc01_g004800 . . . . .
. . . . . . . .
. . . . . .
Vv07g1155 Tc01_g004790 . . Gorai.007G211400.1
Gorai.003G022900.1 . . . . .
. . . . . . . .
. .
Vv07g1156 Tc01_g004780 Gorai.007G053800.1 . .
Gorai.003G021700.1 Gorai.011G102000.1 Vv18g0909
Tc08_g000830 . Gorai.005G103900.1 Gorai.
009G210500.1 . . . . . . .
. .
Vv07g1157 . . . . . . .
. . . . . . . .
. . . . .
Vv07g1158 Tc01_g004630 . . . Gorai.
003G018700.1 . Vv18g0913 Tc08_g000840 . .
. . . . . . . .
. .
Vv07g1159 Tc01_g004620 . . . . .
Vv18g0915 Tc08_g000850 . . . .
. . . . . . . .
Vv07g1160 . . . Gorai.007G210500.1 .
. Vv18g0932 Tc08_g000920 . . .
. . . . . . . .
.
Vv07g1161 . . . . . . .
. . . . . . . .
. . . . .
Vv07g1162 . . . . . . .
. . . . . . . .
. . . . .
Vv07g1163 . . . . . . .
. . . . . . . .
. . . . .
Vv07g1164 . . . . . . .

| | | | | | | | |
|---|---|---|---|---|---|---|---|
| . | . | . | . | . | . | . | . |
| . | . | . | . | . | | | |
| Vv07g1165 | . | . | . | . | . | . | . |
| . | . | . | . | . | . | . | . |
| . | . | . | . | . | | | |
| Vv07g1166 | . | . | . | . | . | . | . |
| . | . | . | . | . | . | . | . |
| . | . | . | . | . | | | |
| Vv07g1167 | . | . | . | . | Gorai.003G044700.1 | | |
| . | . | . | . | . | . | . | . |
| . | . | . | . | . | . | . | |
| Vv07g1168 | . | . | . | . | . | . | |
| Vv18g0425 | Tc08_g009510 | | . | . | Gorai.009G240100.1 | | |
| . | . | . | . | . | . | . | . |
| . | | | | | | | |
| Vv07g1169 | . | . | . | . | Gorai.003G044900.1 | | |
| . | . | . | . | . | . | . | . |
| . | . | . | . | . | . | . | |
| Vv07g1170 | . | . | . | . | . | . | |
| Vv18g0423 | . | . | . | . | . | . | . |
| . | . | . | . | . | . | | |
| Vv07g1171 | . | . | . | . | . | . | . |
| . | . | . | . | . | . | . | . |
| . | . | . | . | . | | | |
| Vv07g1172 | . | . | . | . | . | . | |
| Vv18g0422 | . | . | . | . | . | . | . |
| . | . | . | . | . | . | | |
| Vv07g1173 | . | . | . | . | . | . | . |
| . | . | . | . | . | . | . | . |
| . | . | . | . | . | | | |
| Vv07g1174 | Tc01_g004450 | | . | . | Gorai.007G209900.1 | | |
| . | Gorai.011G104400.1 | | | . | . | . | . |
| . | . | . | . | . | . | . | . |
| . | . | | | | | | |
| Vv07g1175 | . | Gorai.007G061700.1 | | | . | . | . |
| . | . | . | . | . | . | . | . |
| . | . | . | . | . | . | . | |
| Vv07g1176 | . | . | . | . | . | . | . |
| . | . | . | . | . | . | . | . |
| . | . | . | . | . | | | |
| Vv07g1177 | Tc01_g004440 | | . | . | Gorai.007G209700.1 | | |
| . | . | . | . | . | . | . | . |
| . | . | . | . | . | . | . | . |
| Vv07g1178 | . | . | . | . | . | . | . |
| . | . | . | . | . | . | . | . |
| . | . | . | . | . | | | |
| Vv07g1179 | . | . | . | . | . | . | . |
| . | . | . | . | . | . | . | . |
| . | . | . | . | . | | | |
| Vv07g1180 | . | . | . | . | . | . | . |

. . . . . . . .
. . . . .
Vv07g1181 . . . . . . .
. . . . . . . .
. . . . .
Vv07g1182 . . . . Gorai.003G045100.1
. . . . . . . .
. . . . . . .
Vv07g1183 . . . . . . .
. . . . . . . .
. . . . .
Vv07g1184 . . . . . . .
. . . . . . . .
. . . . .
Vv07g1185 . . . . . .
Vv18g0421 Tc08_g009560 . Gorai.005G093000.1 .
. . . . . . . .
.
Vv07g1186 . . . . . . .
. . . . . . . .
. . . . .
Vv07g1187 . . . . Gorai.003G045300.1
. Vv18g0420 Tc08_g009570 . .
Gorai.009G240400.1 . . . . . .
. . .
Vv07g1188 . . . . . .
Vv18g0419 Tc08_g009580 . . . . .
. . . . . . .
Vv07g1189 . . . . . . .
. . . . . . . .
. . . . .
Vv07g1190 . . . . . . .
. . . . . . . .
. . . . .
Vv07g1191 . . . . . . .
. . . . . . . .
. . . . .
Vv07g1192 . . . . Gorai.003G045400.1
. . . . . . . .
. . . . . . .
Vv07g1193 . . . . Gorai.003G045500.1
. Vv18g0417 . . . . .
. . . . . . . .
Vv07g1194 . Gorai.007G061800.1 . . .
. Vv18g0416 . . . . .
. . . . . . . .
Vv07g1195 . . . . . . .
. . . . . . . .
. . . . .
Vv07g1196 . . . . . Gorai.

011G108600.1 . . . . . . .
. . . . . . .
Vv07g1197 . . . . . . .
. . . . . . . .
. . . . .
Vv07g1198 . . . . . . .
. . . . . . . .
. . . . .
Vv07g1199 . . . . . . .
. . . . . . . .
. . . . .
Vv07g1200 . . . . . . .
. . . . . . . .
. . . . .
Vv07g1201 . . . . . .
Vv18g0404 . . . . . . .
. . . . . .
Vv07g1202 . . . . . .
Vv18g0403 Tc08_g009730 . . . .
Gorai.010G045600.1 . . . . . .
.
Vv07g1203 Tc01_g017090 . . . . .
. . . . . . . .
. . . . . .
Vv07g1204 . . . . . . .
. . . . . . . .
. . . . .
Vv07g1205 . . . . . . .
. . . . . . . .
. . . . .
Vv07g1206 . . . . . . .
. . . . . . . .
. . . . .
Vv07g1207 . Gorai.007G062000.1 . . .
. . . . . . . .
. . . . . . .
Vv07g1208 . . . . . . .
. . . . . . . .
. . . . .
Vv07g1209 . . . . . . .
. . . . . . . .
. . . . .
Vv07g1210 Tc01_g004130 . . Gorai.007G208700.1
. . . . . . . .
. . . . . . . .
Vv07g1211 . . . . . . .
. . . . . . . .
. . . . .
Vv07g1212 . . . . . . .
. . . . . . . .

. . . . .
Vv07g1213 . . . . . . .
. . . . . . . .
. . . . .
Vv07g1214 Tc01_g033100 . . . Gorai.
003G046300.1 . . . . . . .
. . . . . . . .
Vv07g1215 . . . . . . .
. . . . . . . .
. . . . .
Vv07g1216 . . . . . . .
. . . . . . . .
. . . . .
Vv07g1217 . . . . Gorai.003G046500.1
. . . . . . . .
. . . . . . .
Vv07g1218 . Gorai.007G062100.1 . . .
. . . . . . . .
. . . . . . .
Vv07g1219 . . . . . . .
. . . . . . . .
. . . . .
Vv07g1220 . . . . . . .
. . . . . . . .
. . . . .
Vv07g1221 . . . . Gorai.003G046700.1
. . . . . . . .
. . . . . . .
Vv07g1222 . . . . . . .
. . . . . . . .
. . . . .
Vv07g1223 . . . . . Gorai.
011G111600.1 . . . . . . .
. . . . . . .
Vv07g1224 . . . . . . .
. . . . . . . .
. . . . .
Vv07g1225 . . . . Gorai.003G047000.1
. . . . . . . .
. . . . . . .
Vv07g1226 . . . . Gorai.003G047100.1
. . . . . . . .
. . . . . . .
Vv07g1227 . . . . Gorai.003G047200.1
. . . . . . . .
. . . . . . .
Vv07g1228 . . . . . . .
. . . . . . . .
. . . . .
Vv07g1229 . Gorai.007G062200.1 . . .

. . . . . . . .
. . . . . . .
Vv07g1230 . . . . . . .
. . . . . . . .
. . . . .
Vv07g1231 Tc01_g033020 . . . . .
. . . . . . . .
. . . . . .
Vv07g1232 . . . . . . .
. . . . . . . .
. . . . .
Vv07g1233 . Gorai.007G062300.1 . . .
. . . . . . . .
. . . . . . .
Vv07g1234 . . . . . . .
. . . . . . . .
. . . . .
Vv07g1235 . . . . . . .
. . . . . . . .
. . . . .
Vv07g1236 . . . . Gorai.003G047600.1
. . . . . . . .
. . . . . . .
Vv07g1237 . . . . . . .
. . . . . . . .
. . . . .
Vv07g1238 Tc01_g017140 . . . . .
. . . . . . . .
. . . . . .
Vv07g1239 . . . . . . .
. . . . . . . .
. . . . .
Vv07g1240 . . . . . . .
. . . . . . . .
. . . . .
Vv07g1241 . . . . . . .
. . . . . . . .
. . . . .
Vv07g1242 . . . . . . .
. . . . . . . .
. . . . .
Vv07g1243 . . . . . . .
. . . . . . . .
. . . . .
Vv07g1244 Tc01_g040940 Gorai.007G062600.1 . .
. . . . . . . .
. . . . . . . .
Vv07g1245 Tc01_g017430 . . . Gorai.
003G047800.1 Gorai.011G114200.1 . . . .
. . . . . . . .

. .
Vv07g1246 Tc01_g011970 . . . . .
. . . . . . . .
. . . . . .
Vv07g1247 . . . . . Gorai.
011G114300.1 . . . . . . .
. . . . . .
Vv07g1248 . Gorai.007G062700.1 . . .
. . . . . . . .
. . . . . . .
Vv07g1249 . . . . . . .
. . . . . . . .
. . . . .
Vv07g1250 . . . . . . .
. . . . . . . .
. . . . .
Vv07g1251 . . . . Gorai.003G048000.1
. . . . . . . .
. . . . . . .
Vv07g1252 . . . . . . .
. . . . . . . .
. . . . .
Vv07g1253 Tc01_g017650 . . . . .
. . . . . . . .
. . . . . .
Vv07g1254 . . . . . . .
. . . . . . . .
. . . . .
Vv07g1255 . . . . . . .
. . . . . . . .
. . . . .
Vv07g1256 . . . . . . .
. . . . . . . .
. . . . .
Vv07g1257 . . . . . . .
. . . . . . . .
. . . . .
Vv07g1258 . . . . . . .
. . . . . . . .
. . . . .
Vv07g1259 Tc01_g040840 . . . . .
. . . . . . . .
. . . . . .
Vv07g1260 Tc01_g032940 . . . . .
. . . . . . . .
. . . . . .
Vv07g1261 . . . . . . .
. . . . . . . .
. . . . .
Vv07g1262 . . . . . . .

. . . . . . . .
. . . . .
Vv07g1263 Tc01_g017770 . . . . .
. . . . . . . .
. . . . . .
Vv07g1264 . . . . . . .
. . . . . . . .
. . . . .
Vv07g1265 . . . . . Gorai.
011G114700.1 . . . . . . .
. . . . . . .
Vv07g1266 . . . . . .
Vv018rg0071 . . . . . . .
. . . . . .
Vv07g1267 . . . . . . .
. . . . . . . .
. . . . .
Vv07g1268 . . . . . . .
. . . . . . . .
. . . . .
Vv07g1269 . Gorai.007G063000.1 . .
Gorai.003G048400.1 . . . . . .
. . . . . . . .
.
Vv07g1270 . . . . . . .
. . . . . . . .
. . . . .
Vv07g1271 . . . . . . .
. . . . . . . .
. . . . .
Vv07g1272 Tc01_g032930 . . . . .
Vv018rg0082 . . . . . .
. . . . . . .
Vv07g1273 . . . . . . .
. . . . . . . .
. . . . .
Vv07g1274 . . . . . . .
. . . . . . . .
. . . . .
Vv07g1275 . . . . Gorai.003G048800.1
Gorai.011G114900.1 . . . . . .
. . . . . . . .
.
Vv07g1276 . . . . . . .
. . . . . . . .
. . . . .
Vv07g1277 . . . . Gorai.003G048900.1
. . . . . . . .
. . . . . . .
Vv07g1278 Tc01_g032180 . . . . .

. . . . . . . .
. . . . . .
Vv07g1279 . . . . . Gorai.
011G115000.1 . . . . . . .
. . . . . . .
Vv07g1280 Tc01_g040830 . . . Gorai.
003G049000.1 . . . . . . .
. . . . . . . .
Vv07g1281 . . . . . .
Vv018rg0089 . . . . . . .
. . . . . .
Vv07g1282 . Gorai.007G063100.1 . . .
. Vv018rg0091 . . . . .
. Vv18g0206 Tc08_g004090 Gorai.002G121600.1
. Gorai.009G192000.1 . .
Vv07g1283 . . . . . .
Vv018rg0092 . . . . . . .
. . . . . .
Vv07g1284 Tc01_g012330 . . . . .
. . . . . . . .
. . . . . .
Vv07g1285 . . . . . Gorai.
011G115100.1 Vv018rg0096 . . . . .
. . . . . . . .
Vv07g1286 . . . . Gorai.003G049500.1
. Vv018rg0102 . . . . .
. . . . . . . .
Vv07g1287 . . . . . . .
. . . . . . . .
. . . . .
Vv07g1288 . . . . . . .
. . . . . . . .
. . . . .
Vv07g1289 Tc01_g000430 . . . Gorai.
003G001900.1 . . . . . . .
. . . . . . . .
Vv07g1290 Tc01_g000420 . . . Gorai.
003G001800.1 . . . . . . .
. . . . . . . .
Vv07g1291 Tc01_g000400 . . . Gorai.
003G001600.1 . . . . . . .
. . . . . . . .
Vv07g1292 Tc01_g000380 . . . . .
. . . . . . . .
. . . . . .
Vv07g1293 Tc01_g000370 . . . Gorai.
003G001500.1 . . . . . . .
. . . . . . . .
Vv07g1294 Tc01_g000360 . . . Gorai.
003G001400.1 . . . . . . .

. . . . . . . .
Vv07g1295 Tc01_g000340 . . . Gorai.
003G001300.1 . . . . . . .
. . . . . . . .
Vv07g1296 Tc01_g000330 . . . . .
. . . . . . . .
. . . . . .
Vv07g1297 Tc01_g000310 . . . Gorai.
003G001200.1 . . . . . . .
. . . . . . . .
Vv07g1298 . . . . . . .
. . . . . . . .
. . . . .
Vv07g1299 . . . . . . .
. . . . . . . .
. . . . .
Vv07g1300 Tc01_g000300 . . . . .
. . . . . . . .
. . . . . .
Vv07g1301 . . . . . . .
. . . . . . . .
. . . . .
Vv07g1302 Tc01_g000290 . . . . .
. . . . . . . .
. . . . . .
Vv07g1303 Tc01_g000270 . . . Gorai.
003G000900.1 . . . . . . .
. . . . . . . .
Vv07g1304 . . . . . . .
. . . . . . . .
. . . . .
Vv07g1305 Tc01_g032780 . . . . .
. . . . . . . .
. . . . . .
Vv07g1306 Tc01_g000260 . . . . .
. . . . . . . .
. . . . . .
Vv07g1307 Tc01_g000250 . . . . .
. . . . . . . .
. . . . . .
Vv07g1308 . . . . . . .
. . . . . . . .
. . . . .
Vv07g1309 Tc01_g031740 . . . . .
. . . . . . . .
. . . . . .
Vv07g1310 . . . . . .
Vv018rg0142 . . . . . .
Vv18g0232 Tc08_g011440 . . Gorai.009G249600.1
. Gorai.010G050100.1

Vv07g1311 . . . . . . .
. . . . . . . .
. . . . .
Vv07g1312 . . . . . . .
. . . . . . . .
. . . . .
Vv07g1313 . . . . . . .
. . . . . . . .
. . . . .
Vv07g1314 . . . . . . .
. . . . . . . .
. . . . .
Vv07g1315 . . . . . . .
. . . . . . . .
. . . . .
Vv07g1316 Tc01_g019580 . . . . .
. . . . . . . .
. . . . . .
Vv07g1317 Tc01_g012530 . . . . .
. . . . . . . .
. . . . . .
Vv07g1318 . . . . . .
Vv18g0253 . . . . . . .
. . . . . .
Vv07g1319 . . . . . . .
. . . . . . . .
. . . . .
Vv07g1320 . . . . . . .
. . . . . . . .
. . . . .
Vv07g1321 Tc01_g012580 . . . . .
Vv18g0261 . . . Gorai.009G248600.1
. . . . . . . .
.
Vv07g1322 . . . . . . .
. . . . . . . .
. . . . .
Vv07g1323 . . . . . . .
. . . . . . . .
. . . . .
Vv07g1324 Tc01_g012610 . . . . .
. . . . . . . .
. . . . . .
Vv07g1325 . . . . . . .
. . . . . . . .
. . . . .
Vv07g1326 Tc01_g012690 . . . . .
Vv18g0275 Tc08_g010990 . . . .
Gorai.010G052300.1 . . . . .
. .

```
Vv07g1327       .       .       .       .       .       .       .
        .       .       .       .       .       .       .       .
        .       .       .       .       .
Vv07g1328       .       .       .       .       .       .       .
        .       .       .       .       .       .       .       .
        .       .       .       .       .
Vv07g1329       .       .       .       .       .       .       .
        .       .       .       .       .       .       .       .
        .       .       .       .       .
Vv07g1330       .       .       .       .       .       .       .
        .       .       .       .       .       .       .       .
        .       .       .       .       .
Vv07g1331       .       .       .       .       .       .       .
        .       .       .       .       .       .       .       .
        .       .       .       .       .
Vv07g1332       Tc01_g032690    .       .       .       .       .
        .       .       .       .       .       .       .       .
        .       .       .       .       .       .
Vv07g1333       Tc01_g019650    .       .       .       .       .
        .       .       .       .       .       .       .       .
        .       .       .       .       .       .
Vv07g1334       .       .       .       .       .       .       .
        .       .       .       .       .       .       .       .
        .       .       .       .       .
Vv07g1335       .       .       .       .       .       .       .
        .       .       .       .       .       .       .       .
        .       .       .       .       .
Vv07g1336       .       .       .       .       .       .       .
        .       .       .       .       .       .       .       .
        .       .       .       .       .
Vv07g1337       Tc01_g012700    .       .       .       .       .
        Vv18g0276       Tc08_g010980    .       .       .       .
        Gorai.010G052200.1      .       .       .       .       .
        .       .
Vv07g1338       Tc01_g012710    .       .       .       .       .
        .       .       .       .       .       .       .       .
        .       .       .       .       .       .
Vv07g1339       Tc01_g012720    .       .       .       .       .
        Vv18g0286       .       .       Gorai.005G089000.1      .
        .       .       .       .       .       .       .       .
        .
Vv07g1340       .       .       .       .       .       .       .
        .       .       .       .       .       .       .       .
        .       .       .       .       .
Vv07g1341       .       .       .       .       .       .       .
        .       .       .       .       .       .       .       .
        .       .       .       .       .
Vv07g1342       Tc01_g012750    .       .       .       .       .
        Vv18g0287       .       .       Gorai.005G089100.1
Gorai.009G247000.1      .       Gorai.010G051500.1      .       .
```

. . . . .
Vv07g1343 . . . . . . .
. . . . . . . .
. . . . .
Vv07g1344 Tc01_g012760 . . . . .
. . . . . . . .
. . . . . .
Vv07g1345 Tc01_g012770 . . . . .
. . . . . . . .
. . . . . .
Vv07g1346 Tc01_g012790 . . . . .
Vv18g0289 . . . Gorai.009G246800.1
. . . . . . . .
.
Vv07g1347 . . . . . . .
. . . . . . . .
. . . . .
Vv07g1348 . . . . . .
Vv18g0290 . . Gorai.005G089200.1 Gorai.
009G246700.1 Gorai.009G283800.1 . . . .
. . . .
Vv07g1349 . . . . . . .
. . . . . . . .
. . . . .
Vv07g1350 Tc01_g021140 . . . . .
. . . . . . . .
. . . . . .
Vv07g1351 . . . . . . .
. . . . . . . .
. . . . .
Vv07g1352 Tc01_g012850 . . . . .
. . . . . . . .
. . . . . .
Vv07g1353 Tc01_g012860 . . . . .
. . . . . . . .
. . . . . .
Vv07g1354 . . . . . . .
. . . . . . . .
. . . . .
Vv07g1355 Tc01_g012870 . . . . .
Vv18g0303 Tc08_g003390 . . Gorai.
009G245900.1 Gorai.009G280500.1 Gorai.010G050400.1 .
. . . . . .
Vv07g1356 . . . . . . .
. . . . . . . .
. . . . .
Vv07g1357 . . . . . . .
. . . . . . . .
. . . . .
Vv07g1358 Tc01_g012890 . . . . .

. . . . . . . .
. . . . . .
Vv07g1359 . . . . . . .
. . . . . . . .
. . . . .
Vv07g1360 Tc01_g020690 . . . . .
Vv18g0305 . . . . .
Gorai.010G050300.1 . . . . . .
.
Vv07g1361 Tc01_g012920 . . . . .
. . . . . . . .
. . . . . .
Vv07g1362 . . . . . . .
. . . . . . . .
. . . . .
Vv07g1363 . . . . . . .
. . . . . . . .
. . . . .
Vv07g1364 . . . . . . .
. . . . . . . .
. . . . .
Vv07g1365 . . . . . . .
. . . . . . . .
. . . . .
Vv07g1366 . . . . . . .
. . . . . . . .
. . . . .
Vv07g1367 Tc01_g012990 . . . . .
. . . . . . . .
. . . . . .
Vv07g1368 . . . . . . .
. . . . . . . .
. . . . .
Vv07g1369 . . . . . . .
. . . . . . . .
. . . . .
Vv07g1370 . . . . . . .
. . . . . . . .
. . . . .
Vv07g1371 Tc01_g013010 . . . . .
. . . . . . . .
. . . . . .
Vv07g1372 . . . . . . .
. . . . . . . .
. . . . .
Vv07g1373 . . . . . . .
. . . . . . . .
. . . . .
Vv07g1374 . . . . . . .
. . . . . . . .

. . . . .
Vv07g1375 Tc01_g013040 . . . . .
Vv18g0320 . . . . . .
. . . . . . .
Vv07g1376 Tc01_g013050 . . . . .
. . . . . . . .
. . . . . .
Vv07g1377 Tc01_g024230 . . . . .
. . . . . . . .
. . . . . .
Vv07g1378 . . . . . . .
. . . . . . . .
. . . . .
Vv07g1379 . . . . . . .
. . . . . . . .
. . . . .
Vv07g1380 Tc01_g024100 . . . . .
. . . . . . . .
. . . . . .
Vv07g1381 Tc01_g013060 . . . . .
. . . . . . . .
. . . . . .
Vv07g1382 . . . . . . .
. . . . . . . .
. . . . .
Vv07g1383 . . . . . . .
. . . . . . . .
. . . . .
Vv07g1384 . . . . . . .
. . . . . . . .
. . . . .
Vv07g1385 . . . . . . .
. . . . . . . .
. . . . .
Vv07g1386 . . . . . . .
. . . . . . . .
. . . . .
Vv07g1387 . . . . . . .
. . . . . . . .
. . . . .
Vv07g1388 . . . . . . .
. . . . . . . .
. . . . .
Vv07g1389 . . . . . . .
. . . . . . . .
. . . . .
Vv07g1390 Tc01_g023780 . . . . .
. . . . . . . .
. . . . . .
Vv07g1391 . . . . . . .

| | | | | | | | |
|---|---|---|---|---|---|---|---|
| . | . | . | . | . | . | . | . |
| . | . | . | . | . | | | |
| Vv07g1392 | . | . | . | . | . | . | . |
| . | . | . | . | . | . | . | . |
| . | . | . | . | . | | | |
| Vv07g1393 | . | . | . | . | . | . | . |
| . | . | . | . | . | . | . | . |
| . | . | . | . | . | | | |
| Vv07g1394 | . | . | . | . | . | . | . |
| . | . | . | . | . | . | . | . |
| . | . | . | . | . | | | |
| Vv07g1395 | . | . | . | . | . | . | . |
| . | . | . | . | . | . | . | . |
| . | . | . | . | . | | | |
| Vv07g1396 | . | . | . | . | . | . | . |
| . | . | . | . | . | . | . | . |
| . | . | . | . | . | | | |
| Vv07g1397 | . | . | . | . | . | . | . |
| . | . | . | . | . | . | . | . |
| . | . | . | . | . | | | |
| Vv07g1398 | . | . | . | . | . | . | . |
| . | . | . | . | . | . | . | . |
| . | . | . | . | . | | | |
| Vv07g1399 | . | . | . | . | . | . | . |
| . | . | . | . | . | . | . | . |
| . | . | . | . | . | | | |
| Vv07g1400 | . | . | . | . | . | . | . |
| . | . | . | . | . | . | . | . |
| . | . | . | . | . | | | |
| Vv07g1401 | Tc01_g022950 | | . | . | . | . | . |
| . | . | . | . | . | . | . | . |
| . | . | . | . | . | . | | |
| Vv07g1402 | . | . | . | . | . | . | . |
| . | . | . | . | . | . | . | . |
| . | . | . | . | . | | | |
| Vv07g1403 | . | . | . | . | . | . | . |
| . | . | . | . | . | . | . | . |
| . | . | . | . | . | | | |
| Vv07g1404 | . | . | . | . | . | . | . |
| . | . | . | . | . | . | . | . |
| . | . | . | . | . | | | |
| Vv07g1405 | . | . | . | . | . | . | . |
| . | . | . | . | . | . | . | . |
| . | . | . | . | . | | | |
| Vv07g1406 | . | . | . | . | . | . | . |
| . | . | . | . | . | . | . | . |
| . | . | . | . | . | | | |
| Vv07g1407 | . | . | . | . | . | . | . |
| . | . | . | . | . | . | . | . |
| . | . | . | . | . | | | |

| | | | | | | | |
|---|---|---|---|---|---|---|---|
| Vv07g1408 | Tc01_g013440 | | . | . | . | . | . |
| . | . | . | . | . | . | . | . |
| . | . | . | . | . | . | | |
| Vv07g1409 | . | . | . | . | . | . | . |
| . | . | . | . | . | . | . | . |
| . | . | . | . | . | | | |
| Vv08g0001 | . | . | . | . | . | . | . |
| . | . | . | . | . | . | . | . |
| . | . | . | . | . | | | |
| Vv08g0002 | . | . | . | . | . | . | . |
| . | . | . | . | . | . | . | . |
| . | . | . | . | . | | | |
| Vv08g0003 | . | . | . | . | . | . | . |
| . | . | . | . | . | . | . | . |
| . | . | . | . | . | | | |
| Vv08g0004 | . | . | . | . | . | . | . |
| . | . | . | . | . | . | . | . |
| . | . | . | . | . | | | |
| Vv08g0005 | . | . | . | . | . | . | |
| Vv01g0416 | . | . | . | . | . | | . |
| . | . | . | . | . | . | | |
| Vv08g0006 | . | . | . | . | . | . | . |
| . | . | . | . | . | . | . | . |
| . | . | . | . | . | | | |
| Vv08g0007 | . | . | . | . | . | . | . |
| . | . | . | . | . | . | . | . |
| . | . | . | . | . | | | |
| Vv08g0008 | . | . | . | . | . | . | . |
| . | . | . | . | . | . | . | . |
| . | . | . | . | . | | | |
| Vv08g0009 | . | . | . | . | . | . | . |
| . | . | . | . | . | . | . | . |
| . | . | . | . | . | | | |
| Vv08g0010 | . | . | . | . | . | . | . |
| . | . | . | . | . | . | . | . |
| . | . | . | . | . | | | |
| Vv08g0011 | . | . | . | . | . | . | . |
| . | . | . | . | . | . | . | . |
| . | . | . | . | . | | | |
| Vv08g0012 | . | . | . | . | . | . | . |
| . | . | . | . | . | . | . | . |
| . | . | . | . | . | | | |
| Vv08g0013 | . | . | . | . | . | . | . |
| . | . | . | . | . | . | . | . |
| . | . | . | . | . | | | |
| Vv08g0014 | . | . | . | . | . | . | . |
| . | . | . | . | . | . | . | . |
| . | . | . | . | . | | | |
| Vv08g0015 | . | . | . | . | . | . | . |
| . | . | . | . | . | . | . | . |

| | | | | | | | |
|---|---|---|---|---|---|---|---|
| Vv08g0016 | . | . | . | . | . | . | . |
| Vv08g0017 | . | . | . | . | . | . | . |
| Vv08g0018 | . | . | . | . | . | . | . |
| Vv08g0019 | . | . | . | . | . | . | . |
| Vv08g0020 | . | . | . | . | . | . | . |
| Vv08g0021 | . | . | . | . | . | . | . |
| Vv08g0022 | . | . | . | . | . | . | . |
| Vv08g0023 | Tc05_g026350 | | . | . | . | . | . |
| Vv08g0024 | . | . | . | . | . | . | . |
| Vv08g0025 | Tc05_g026080 | | . | . | . | . | . |
| Vv08g0026 | . | . | . | . | . | . | . |
| Vv08g0027 | . | . | . | . | . | . | . |
| Vv08g0028 | . | . | . | . | . | . | . |
| Vv08g0029 | . | . | . | . | . | . | . |
| Vv08g0030 | Tc05_g020760 | | . | . | . | . | . |
| Vv08g0031 | . | . | . | . | . | . | . |
| Vv08g0032 | . | . | . | . | . | . | . |

| | | | | | | | |
|---|---|---|---|---|---|---|---|
| . | . | . | . | . | . | . | . |
| . | . | . | . | . | | | |
| Vv08g0033 | . | . | . | . | . | . | . |
| . | . | . | . | . | . | . | . |
| . | . | . | . | . | | | |
| Vv08g0034 | . | . | . | . | . | . | . |
| . | . | . | . | . | . | . | . |
| . | . | . | . | . | | | |
| Vv08g0035 | . | . | . | . | . | . | . |
| . | . | . | . | . | . | . | . |
| . | . | . | . | . | | | |
| Vv08g0036 | . | . | . | . | . | . | . |
| . | . | . | . | . | . | . | . |
| . | . | . | . | . | | | |
| Vv08g0037 | . | . | . | . | . | . | . |
| . | . | . | . | . | . | . | . |
| . | . | . | . | . | | | |
| Vv08g0038 | . | . | . | . | . | . | . |
| . | . | . | . | . | . | . | . |
| . | . | . | . | . | | | |
| Vv08g0039 | . | . | . | . | . | . | . |
| . | . | . | . | . | . | . | . |
| . | . | . | . | . | | | |
| Vv08g0040 | . | . | . | . | . | . | |
| Vv01g0436 | Tc02_g017610 | | . | . | . | . | . |
| . | . | . | . | . | . | . | |
| Vv08g0041 | . | . | . | . | . | . | . |
| . | . | . | . | . | . | . | . |
| . | . | . | . | . | | | |
| Vv08g0042 | . | . | . | . | . | . | . |
| . | . | . | . | . | . | . | . |
| . | . | . | . | . | | | |
| Vv08g0043 | . | . | . | . | . | . | . |
| . | . | . | . | . | . | . | . |
| . | . | . | . | . | | | |
| Vv08g0044 | . | . | . | . | . | . | . |
| . | . | . | . | . | . | . | . |
| . | . | . | . | . | | | |
| Vv08g0045 | . | . | . | . | . | . | . |
| . | . | . | . | . | . | . | . |
| . | . | . | . | . | | | |
| Vv08g0046 | . | . | . | . | . | . | . |
| . | . | . | . | . | . | . | . |
| . | . | . | . | . | | | |
| Vv08g0047 | . | . | . | . | . | . | . |
| . | . | . | . | . | . | . | . |
| . | . | . | . | . | | | |
| Vv08g0048 | . | . | . | . | . | . | . |
| . | . | . | . | . | . | . | . |
| . | . | . | . | . | | | |

Vv08g0049 . . . . . . .
. . . . . . . .
. . . . .
Vv08g0050 Tc05_g003940 . . . Gorai.
012G106700.1 . . . . . . .
. . . . . . . .
Vv08g0051 Tc05_g003950 . . . . .
. . . . . . . .
. . . . . .
Vv08g0052 Tc05_g004050 . . . . .
. . . . . . . .
. . . . . .
Vv08g0053 . . . . . . .
. . . . . . . .
. . . . .
Vv08g0054 . . . . . . .
. . . . . . . .
. . . . .
Vv08g0055 . . . . . .
Vv12g0469 . . . . . Gorai.
001G104700.1 . . . . . . .
Vv08g0056 Tc05_g021070 . . . . .
. . . . . . . .
. . . . . .
Vv08g0057 . . . . . . .
. . . . . . . .
. . . . .
Vv08g0058 . . . . . . .
. . . . . . . .
. . . . .
Vv08g0059 . . . . . . .
. . . . . . . .
. . . . .
Vv08g0060 . . . . . . .
. . . . . . . .
. . . . .
Vv08g0061 Tc05_g022730 . . . . .
. . . . . . . .
. . . . . .
Vv08g0062 . . . . . . .
. . . . . . . .
. . . . .
Vv08g0063 . . . . . . .
. . . . . . . .
. . . . .
Vv08g0064 . . . . . . .
. . . . . . . .
. . . . .
Vv08g0065 . . . . . . .
. . . . . . . .

| | | | | | | | |
|---|---|---|---|---|---|---|---|
| Vv08g0066 | . | . | . | . | . | . | . |
| Vv08g0067 | . | . | . | . | . | . | . |
| Vv08g0068 | . | . | . | . | . | . | . |
| Vv08g0069 | . | . | . | . | . | . | . |
| Vv08g0070 | . | . | . | . | . | . | . |
| Vv08g0071 | Tc05_g023120 | | . | . | . | . | . |
| Vv08g0072 | Tc05_g023130 | | . | . | . | . | . |
| Vv08g0073 | Tc05_g023140 | | . | . | . | . | . |
| Vv08g0074 | . | . | . | . | . | . | . |
| Vv08g0075 | Tc05_g023150 | | . | . | . | . | . |
| Vv08g0076 | . | . | . | . | . | . | . |
| . | . | . | . | . | . | Vv11g1069 | |
| Vv08g0077 | . | . | . | . | . | . | . |
| Vv08g0078 | . | . | . | . | . | . | . |
| Vv08g0079 | Tc05_g023160 | | . | . | . | . | . |
| Vv08g0080 | . | . | . | . | . | . | . |
| Vv08g0081 | . | . | . | . | . | . | . |
| Vv08g0082 | . | . | . | . | . | . | . |

. . . . . . . .
. . . . .
Vv08g0083 . . . . . . .
. . . . . . . .
. . . . .
Vv08g0084 Tc05_g023190 . . . . .
. . . . . . .
Vv11g1071 Tc09_g016710 . . . . .
Vv08g0085 . . . . . . .
. . . . . . . .
. . . . .
Vv08g0086 Tc05_g023200 . . . . .
. . . . . . .
Vv11g1072 . . . . . .
Vv08g0087 . . . . . . .
. . . . . . . .
. . . . .
Vv08g0088 Tc05_g023210 . . . . .
Vv12g0497 . . . . . .
Vv11g1073 Tc09_g016680 . . . .
.
Vv08g0089 . . . . . . .
. . . . . . . .
. . . . .
Vv08g0090 Tc05_g023240 . . . . .
. . . . . . . .
. . . . . .
Vv08g0091 . . . . . . .
. . . . . . . .
. . . . .
Vv08g0092 . . . . . . .
. . . . . . . .
. . . . .
Vv08g0093 . . . . . . .
. . . . . . . .
. . . . .
Vv08g0094 Tc05_g023410 . . . . .
. . . . . . . .
. . . . . .
Vv08g0095 . . . . . . .
. . . . . . Vv11g1074
Tc09_g016670 . . . . .
Vv08g0096 . . . . . .
Vv12g0498 . . . . . Gorai.
001G098600.1 . . . . . . .
Vv08g0097 . . . . . . .
. . . . . . . .
. . . . .
Vv08g0098 . . . . . . .
. . . . . . Vv11g1075

| | | | | | | | |
|---|---|---|---|---|---|---|---|
| . | . | . | . | . | . | | |
| Vv08g0099 | . | . | . | . | . | . | . |
| . | . | . | . | . | . | . | . |
| . | . | . | . | . | | | |
| Vv08g0100 | . | . | . | . | . | . | . |
| . | . | . | . | . | . | . | . |
| . | . | . | . | . | | | |
| Vv08g0101 | . | . | . | . | . | . | . |
| . | . | . | . | . | . | . | . |
| . | . | . | . | . | | | |
| Vv08g0102 | . | . | . | . | . | . | . |
| . | . | . | . | . | . | . | . |
| . | . | . | . | . | | | |
| Vv08g0103 | . | . | . | . | . | . | |
| Vv12g0499 | . | . | . | . | . | . | |
| Vv11g1076 | Tc09_g016660 | | Gorai.013G246100.1 | | | . | . |
| . | . | | | | | | |
| Vv08g0104 | . | . | . | . | . | . | |
| Vv12g0501 | . | . | . | . | . | . | |
| Vv11g1077 | . | . | . | . | . | . | |
| Vv08g0105 | . | . | . | . | . | . | . |
| . | . | . | . | . | . | . | . |
| . | . | . | . | . | | | |
| Vv08g0106 | . | . | . | . | . | . | |
| Vv12g0503 | . | . | . | . | . | . | . |
| . | . | . | . | . | . | | |
| Vv08g0107 | . | . | . | . | . | . | . |
| . | . | . | . | . | . | . | . |
| . | . | . | . | . | | | |
| Vv08g0108 | . | . | . | . | . | . | . |
| . | . | . | . | . | . | . | . |
| . | . | . | . | . | | | |
| Vv08g0109 | Tc05_g023450 | | . | . | . | . | . |
| . | . | . | . | . | . | . | . |
| . | . | . | . | . | . | | |
| Vv08g0110 | Tc05_g023460 | | . | . | . | . | . |
| . | . | . | . | . | . | . | . |
| . | . | . | . | . | . | | |
| Vv08g0111 | Tc05_g023470 | | Gorai.013G113200.1 | | | . | . |
| . | . | . | . | . | . | . | . |
| . | . | . | . | . | . | . | . |
| Vv08g0112 | . | . | . | . | . | . | . |
| . | . | . | . | . | . | . | . |
| . | . | . | . | . | | | |
| Vv08g0113 | . | . | . | . | . | . | . |
| . | . | . | . | . | . | . | . |
| . | . | . | . | . | | | |
| Vv08g0114 | . | . | . | . | . | . | . |
| . | . | . | . | . | . | . | . |
| . | . | . | . | . | | | |

Vv08g0115 . . . . . . .
. . . . . . . .
. . . . .
Vv08g0116 . . . . . . .
. . . . . . . .
. . . . .
Vv08g0117 . . . . . . .
. . . . . . . .
. . . . .
Vv08g0118 Tc05_g004730 . . . . .
. . . . . . . .
. . . . . .
Vv08g0119 . . . . . . .
. . . . . . . .
. . . . .
Vv08g0120 . . . . . . .
. . . . . . . .
. . . . .
Vv08g0121 . . . . . . .
. . . . . . . .
. . . . .
Vv08g0122 . . . . . . .
. . . . . . . .
. . . . .
Vv08g0123 . . . . . . .
. . . . . . . .
. . . . .
Vv08g0124 . . . . . . .
. . . . . . . .
. . . . .
Vv08g0125 Tc05_g023600 . . . . .
. . . . . . . .
. . . . . .
Vv08g0126 . . . . . . .
. . . . . . . .
. . . . .
Vv08g0127 Tc05_g023650 . . . . .
. . . . . . . .
. . . . . .
Vv08g0128 Tc05_g023660 . . . . .
. . . . . . . .
. . . . . .
Vv08g0129 Tc05_g023670 . . . . .
. . . . . . . .
. . . . . .
Vv08g0130 . . . . . . .
. . . . . . . .
. . . . .
Vv08g0131 Tc05_g023680 Gorai.013G113400.1 . .
. . . . . . . .

| | | | | | | | |
|---|---|---|---|---|---|---|---|
| . | . | . | . | . | . | . | . |
| Vv08g0132 | . | . | . | . | . | . | . |
| . | . | . | . | . | . | . | . |
| . | . | . | . | . | | | |
| Vv08g0133 | Tc05_g023700 | | . | . | . | . | . |
| . | . | . | . | . | . | . | . |
| . | . | . | . | . | . | | |
| Vv08g0134 | . | . | . | . | . | . | . |
| . | . | . | . | . | . | . | . |
| . | . | . | . | . | | | |
| Vv08g0135 | . | . | . | . | . | . | . |
| . | . | . | . | . | . | . | . |
| . | . | . | . | . | | | |
| Vv08g0136 | Tc05_g018910 | | . | . | . | . | . |
| . | . | . | . | . | . | . | . |
| . | . | . | . | . | . | | |
| Vv08g0137 | . | . | . | . | . | . | |
| Vv06g1089 | . | . | . | . | . | . | . |
| . | . | . | . | . | . | | |
| Vv08g0138 | . | . | . | . | . | . | . |
| . | . | . | . | . | . | . | . |
| . | . | . | . | . | | | |
| Vv08g0139 | . | . | . | . | . | . | . |
| . | . | . | . | . | . | . | . |
| . | . | . | . | . | | | |
| Vv08g0140 | . | . | . | . | . | . | . |
| . | . | . | . | . | . | . | . |
| . | . | . | . | . | | | |
| Vv08g0141 | . | . | . | . | . | . | . |
| . | . | . | . | . | . | . | . |
| . | . | . | . | . | | | |
| Vv08g0142 | . | . | . | . | . | . | . |
| . | . | . | . | . | . | . | . |
| . | . | . | . | . | | | |
| Vv08g0143 | Tc05_g004750 | | . | . | . | . | . |
| . | . | . | . | . | . | . | |
| Vv01g0465 | . | Gorai.002G180500.1 | | | Gorai.005G162900.1 | | |
| . | . | . | | | | | |
| Vv08g0144 | . | . | . | . | . | . | . |
| . | . | . | . | . | . | . | . |
| . | . | . | . | . | | | |
| Vv08g0145 | . | . | . | . | . | . | . |
| . | . | . | . | . | . | . | . |
| . | . | . | . | . | | | |
| Vv08g0146 | . | . | . | . | . | . | . |
| . | . | . | . | . | . | . | . |
| . | . | . | . | . | | | |
| Vv08g0147 | . | . | . | . | . | . | |
| Vv06g1087 | Tc09_g026980 | | . | Gorai.001G128200.1 | | | . |
| . | . | . | . | . | . | . | . |

| | | | | | | | |
|---|---|---|---|---|---|---|---|
| . | | | | | | | |
| Vv08g0148 | . | . | . | . | . | . | . |
| . | . | . | . | . | . | . | . |
| . | . | . | . | . | | | |
| Vv08g0149 | . | . | . | . | . | . | . |
| . | . | . | . | . | . | . | . |
| . | . | . | . | . | | | |
| Vv08g0150 | . | . | . | . | . | . | . |
| . | . | . | . | . | . | . | . |
| . | . | . | . | . | | | |
| Vv08g0151 | . | . | . | . | . | . | . |
| . | . | . | . | . | . | . | . |
| . | . | . | . | . | | | |
| Vv08g0152 | Tc05_g023710 | | . | . | . | . | . |
| . | . | . | . | . | . | . | . |
| . | . | . | . | . | . | | |
| Vv08g0153 | . | . | . | . | . | . | . |
| . | . | . | . | . | . | . | . |
| . | . | . | . | . | | | |
| Vv08g0154 | . | . | . | . | . | . | . |
| . | . | . | . | . | . | . | . |
| . | . | . | . | . | | | |
| Vv08g0155 | . | . | . | . | . | . | . |
| . | . | . | . | . | . | . | . |
| . | . | . | . | . | | | |
| Vv08g0156 | . | . | . | . | . | . | . |
| . | . | . | . | . | . | . | . |
| . | . | . | . | . | | | |
| Vv08g0157 | . | . | . | . | . | . | . |
| . | . | . | . | . | . | . | . |
| . | . | . | . | . | | | |
| Vv08g0158 | . | . | . | . | . | . | . |
| . | . | . | . | . | . | . | . |
| . | . | . | . | . | | | |
| Vv08g0159 | . | . | . | . | . | . | |
| Vv06g1081 | . | . | . | . | . | . | . |
| . | . | . | . | . | . | | |
| Vv08g0160 | . | . | . | . | . | . | . |
| . | . | . | . | . | . | . | . |
| . | . | . | . | . | | | |
| Vv08g0161 | . | . | . | . | . | . | . |
| . | . | . | . | . | . | . | . |
| . | . | . | . | . | | | |
| Vv08g0162 | . | . | . | . | . | . | . |
| . | . | . | . | . | . | . | . |
| . | . | . | . | . | | | |
| Vv08g0163 | . | . | . | . | . | . | . |
| . | . | . | . | . | . | . | . |
| . | . | . | . | . | | | |
| Vv08g0164 | . | . | . | . | . | . | . |

```
         .       .       .       .       .       .       .       .
         .       .       .       .       .
Vv08g0165        .       .       .       .       .       .       .
         .       .       .       .       .       .       .       .
         .       .       .       .       .
Vv08g0166        .       .       .       .       .       .       .
         .       .       .       .       .       .       .       .
         .       .       .       .       .
Vv08g0167        Tc05_g005040    .       .       .       .       .
        Vv06g1074        .       .       .       .       .
Gorai.006G146700.1       .       .       .       .       .       .
         .
Vv08g0168        .       .       .       .       .       .       .
         .       .       .       .       .       .       .       .
         .       .       .       .       .
Vv08g0169        .       .       .       .       .       .       .
         .       .       .       .       .       .       .       .
         .       .       .       .       .
Vv08g0170        .       .       .       .       .       .       .
         .       .       .       .       .       .       .       .
         .       .       .       .       .
Vv08g0171        .       .       .       .       .       .       .
         .       .       .       .       .       .       .       .
         .       .       .       .       .
Vv08g0172        .       .       .       .       .       .       .
         .       .       .       .       .       .       .       .
         .       .       .       .       .
Vv08g0173        Tc05_g018650    .       .       .       .       .
         .       .       .       .       .       .       .       .
         .       .       .       .       .       .
Vv08g0174        .       .       .       .       .       .       .
         .       .       .       .       .       .       .       .
         .       .       .       .       .
Vv08g0175        .       .       .       .       .       .       .
         .       .       .       .       .       .       .       .
         .       .       .       .       .
Vv08g0176        .       .       .       .       .       .       .
         .       .       .       .       .       .       .       .
         .       .       .       .       .
Vv08g0177        .       .       .       .       .       .
Vv06g1061        .       .       .       .       .       .       .
         .       .       .       .       .       .
Vv08g0178        .       .       .       .       .       .       .
         .       .       .       .       .       .       .       .
         .       .       .       .       .
Vv08g0179        .       .       .       .       .       .       .
         .       .       .       .       .       .       .       .
         .       .       .       .       .
Vv08g0180        .       .       .       .       .       .       .
         .       .       .       .       .       .       .       .
```

| | | | | | | | |
|---|---|---|---|---|---|---|---|
| Vv08g0181 | . | . | . | . | . | . | . |
| Vv08g0182 | . | . | . | . | . | . | . |
| Vv08g0183 | . | . | . | . | . | . | . |
| Vv08g0184 | . | . | . | . | . | . | . |
| Vv08g0185 | . | . | . | . | . | . | . |
| Vv08g0186 | Tc05_g018260 | | . | Gorai.002G228800.1 | | | . |
| Vv08g0187 | . | . | . | . | . | . | . |
| Vv08g0188 | . | . | . | . | . | . | . |
| Vv08g0189 | . | . | . | . | . | . | . |
| Vv08g0190 | Tc05_g023730 | | . | . | . | . | . |
| Vv08g0191 | . | . | . | . | . | . | . |
| Vv08g0192 | . | . | . | . | . | . | . |
| Vv08g0193 | . | . | . | . | . | . | . |
| Vv08g0194 | Tc05_g023980 | | . | . | . | . | . |
| Vv08g0195 | . | . | . | . | . | . | . |
| Vv08g0196 | . | . | . | . | . | . | . |
| Vv08g0197 | . | . | . | . | . | . | . |

```
        .        .       .        .       .        .       .        .
        .        .       .        .       .
Vv08g0198        Tc05_g020190     Gorai.006G146100.1       .        .
        .        .       .        .       .        .       .        .
        .        .       .        .       .        .       .        .
Vv08g0199        .       .        .       .        .       .        .
        .        .       .        .       .        .       .        .
        .        .       .        .       .
Vv08g0200        .       .        .       .        .       .        .
        .        .       .        .       .        .       .        .
        .        .       .        .       .
Vv08g0201        .       .        .       .        .       .        .
        .        .       .        .       .        .       .        .
        .        .       .        .       .
Vv08g0202        .       .        .       .        .       .        .
        .        .       .        .       .        .       .        .
        .        .       .        .       .
Vv08g0203        .       .        .       .        .       .        .
        .        .       .        .       .        .       .        .
        .        .       .        .       .
Vv08g0204        .       .        .       .        .       .        .
        .        .       .        .       .        .       .        .
        .        .       .        .       .
Vv08g0205        .       .        .       .        .       .        .
        .        .       .        .       .        .       .        .
        .        .       .        .       .
Vv08g0206        .       .        .       .        .       .        .
        .        .       .        .       .        .       .        .
        .        .       .        .       .
Vv08g0207        .       .        .       .        .       .        .
        .        .       .        .       .        .       .        .
        .        .       .        .       .
Vv08g0208        .       .        .       .        .       .        .
        .        .       .        .       .        .       .        .
        .        .       .        .       .
Vv08g0209        .       .        .       .        .       .        .
        .        .       .        .       .        .       .        .
        .        .       .        .       .
Vv08g0210        Tc05_g024040     .       .        .       .        .
        Vv06g1046        Tc09_g030330     .        .       .        .
        .        .       .        .       .        .       .        .
Vv08g0211        Tc05_g024050     .       .        .       .        .
        Vv06g1045        Tc09_g030320     .        .       .        .
        .        .       .        .       .        .       .        .
Vv08g0212        .       .        .       .        .       .        .
        .        .       .        .       .        .       .        .
        .        .       .        .       .
Vv08g0213        .       .        .       .        .       .        .
        .        .       .        .       .        .       .        .
        .        .       .        .       .
```

Vv08g0214 . . . . . . .
. . . . . . . .
. . . . .
Vv08g0215 . . . . . . .
. . . . . . . .
. . . . .
Vv08g0216 . . . . . . .
. . . . . . . .
. . . . .
Vv08g0217 . . . . . . .
. . . . . . . .
. . . . .
Vv08g0218 Tc05_g020180 Gorai.006G146200.1 . .
. . . . . . . .
. . . . . . . .
Vv08g0219 . . . . . . .
. . . . . . . .
. . . . .
Vv08g0220 . . . . . . .
. . . . . . . .
. . . . .
Vv08g0221 Tc05_g020150 Gorai.006G146300.1 . .
. . . . . . . .
. . . . . . . .
Vv08g0222 . . . . . . .
. . . . . . . .
. . . . .
Vv08g0223 Tc05_g020130 . Gorai.002G232700.1 .
. . . . . . . .
. . . . . . . .
Vv08g0224 . . . . . . .
. . . . . . . .
. . . . .
Vv08g0225 . . . . . . .
. . . . . . . .
. . . . .
Vv08g0226 . . . . . . .
. . . . . . . .
. . . . .
Vv08g0227 . . . . . . .
. . . . . . . .
. . . . .
Vv08g0228 . . Gorai.002G232900.1 . .
. . . . . . . .
. . . . . . .
Vv08g0229 . . . . . . .
. . . . . . . .
. . . . .
Vv08g0230 Tc05_g020110 . . . . .
. . . . . . . .

. . . . . .
Vv08g0231 . . . . . . .
. . . . . . . .
. . . . .
Vv08g0232 . . . . . . .
. . . . . . . .
. . . . .
Vv08g0233 . . . . . . .
. . . . . . . .
. . . . .
Vv08g0234 . . . . . . .
. . . . . . . .
. . . . .
Vv08g0235 . . . . . . .
. . . . . . . .
. . . . .
Vv08g0236 Tc05_g020050 Gorai.006G146700.1 . .
. . . . . . . .
. . . . . . . .
Vv08g0237 . . . . . . .
. . . . . . . .
. . . . .
Vv08g0238 . . . . . . .
. . . . . . . .
. . . . .
Vv08g0239 Tc05_g020010 Gorai.006G146900.1 Gorai.
002G233200.1 . . . . . . .
. . . . . . . .
. .
Vv08g0240 . . . . . . .
. . . . . . . .
. . . . .
Vv08g0241 Tc05_g020000 . . . . .
. . . . . . . .
. . . . . .
Vv08g0242 Tc05_g019980 . . . . .
. . . . . . . .
. . . . . .
Vv08g0243 . . . . . . .
. . . . . . . .
. . . . .
Vv08g0244 . . . . . . .
. . . . . . . .
. . . . .
Vv08g0245 . . . . . . .
. . . . . . . .
. . . . .
Vv08g0246 . . . . . . .
. . . . . . . .
. . . . .

| | | | | | | | | | | | | | | | | | | | | |
|---|---|---|---|---|---|---|---|---|---|---|---|---|---|---|---|---|---|---|---|---|
| Vv08g0247 | . | . | . | . | . | . | . | . | . | . | . | . | . | . | . | . | . | . | . | . |
| Vv08g0248 | Tc05_g019540 | . | . | . | . | . | . | . | . | . | . | . | . | . | . | . | . | . | . | . |
| Vv08g0249 | . | . | . | . | . | . | . | . | . | . | . | . | . | . | . | . | . | . | . | . |
| Vv08g0250 | . | . | . | . | . | . | . | . | . | . | . | . | . | . | . | . | . | . | . | . |
| Vv08g0251 | . | . | . | . | . | . | . | . | . | . | . | . | . | . | . | . | . | . | . | . |
| Vv08g0252 | . | . | . | . | . | . | . | . | . | . | . | . | . | . | . | . | . | . | . | . |
| Vv08g0253 | . | . | . | . | . | . | . | . | . | . | . | . | . | . | . | . | . | . | . | . |
| Vv08g0254 | . | . | . | . | . | . | . | . | . | . | . | . | . | . | . | . | . | . | . | . |
| Vv08g0255 | . | . | . | . | . | . | . | . | . | . | . | . | . | . | . | . | . | . | . | . |
| Vv08g0256 | . | . | . | . | . | . | . | . | . | . | . | . | . | . | . | . | . | . | . | . |
| Vv08g0257 | . | . | . | . | . | . | . | . | . | . | . | . | . | . | . | . | . | . | . | . |
| Vv08g0258 | . | . | . | . | . | . | . | . | . | . | . | . | . | . | . | . | . | . | . | . |
| Vv08g0259 | . | . | . | . | . | . | . | . | . | . | . | . | . | . | . | . | . | . | . | . |
| Vv08g0260 | . | . | . | . | . | . | . | . | . | . | . | . | . | . | . | . | . | . | . | . |
| Vv08g0261 | . | . | . | . | . | . | . | . | . | . | . | . | . | . | . | . | . | . | . | . |
| Vv08g0262 | . | . | . | . | . | . | . | . | . | . | . | . | . | . | . | . | . | . | . | . |
| Vv08g0263 | . | . | . | . | . | . | . | . | . | . | . | . | . | . | . | | | | | |

| | | | | | | | |
|---|---|---|---|---|---|---|---|
| . | . | . | . | . | | | |
| Vv08g0264 | . | . | . | . | . | . | . |
| . | . | . | . | . | . | . | . |
| . | . | . | . | . | | | |
| Vv08g0265 | . | Gorai.006G147400.1 | | | . | . | . |
| . | . | . | . | . | . | . | . |
| . | . | . | . | . | . | . | |
| Vv08g0266 | . | Gorai.006G147500.1 | | | . | . | . |
| . | . | . | . | . | . | . | . |
| . | . | . | . | . | . | . | |
| Vv08g0267 | . | . | . | . | . | . | . |
| . | . | . | . | . | . | . | . |
| . | . | . | . | . | | | |
| Vv08g0268 | Tc05_g019360 | | . | . | . | . | . |
| . | . | . | . | . | . | . | . |
| . | . | . | . | . | . | | |
| Vv08g0269 | . | . | . | . | . | . | . |
| . | . | . | . | . | . | . | . |
| . | . | . | . | . | | | |
| Vv08g0270 | . | . | . | . | . | . | . |
| . | . | . | . | . | . | . | . |
| . | . | . | . | . | | | |
| Vv08g0271 | . | . | . | . | . | . | . |
| . | . | . | . | . | . | . | . |
| . | . | . | . | . | | | |
| Vv08g0272 | . | . | . | . | . | . | . |
| . | . | . | . | . | . | . | . |
| . | . | . | . | . | | | |
| Vv08g0273 | . | . | . | . | . | . | . |
| . | . | . | . | . | . | . | . |
| . | . | . | . | . | | | |
| Vv08g0274 | . | . | . | . | . | . | . |
| . | . | . | . | . | . | . | . |
| . | . | . | . | . | | | |
| Vv08g0275 | . | . | . | . | . | . | . |
| . | . | . | . | . | . | . | . |
| . | . | . | . | . | | | |
| Vv08g0276 | . | . | . | . | . | . | . |
| . | . | . | . | . | . | . | . |
| . | . | . | . | . | | | |
| Vv08g0277 | . | . | Gorai.002G233900.1 | | | . | . |
| . | . | . | . | . | . | . | . |
| . | . | . | . | . | . | . | |
| Vv08g0278 | . | . | . | . | . | . | . |
| . | . | . | . | . | . | . | . |
| . | . | . | . | . | | | |
| Vv08g0279 | . | . | . | . | . | . | . |
| . | . | . | . | . | . | . | . |
| . | . | . | . | . | | | |
| Vv08g0280 | Tc05_g017170 | | . | . | . | . | . |

. . . . . . . .
. . . . . .
Vv08g0281 . . . . . . .
. . . . . . . .
. . . . .
Vv08g0282 . . . . . . .
. . . . . . . .
. . . . .
Vv08g0283 . . . . . . .
. . . . . . . .
. . . . .
Vv08g0284 . . . . . . .
. . . . . . . .
. . . . .
Vv08g0285 . . . . . . .
. . . . . . . .
. . . . .
Vv08g0286 . . Gorai.002G234200.1 . .
. . . . . . . .
. . . . . . .
Vv08g0287 . . . . . . .
. . . . . . . .
. . . . .
Vv08g0288 . . . . . . .
. . . . . . . .
. . . . .
Vv08g0289 . . . . . . .
. . . . . . . .
. . . . .
Vv08g0290 . . . . . . .
. . . . . . . .
. . . . .
Vv08g0291 . . . . . . .
. . . . . . . .
. . . . .
Vv08g0292 . . . . . . .
. . . . . . . .
. . . . .
Vv08g0293 . . . . . . .
. . . . . . . .
. . . . .
Vv08g0294 . . . . . . .
. . . . . . . .
. . . . .
Vv08g0295 Tc05_g017210 . . . . .
. . . . . . . .
. . . . . .
Vv08g0296 . . . . . . .
. . . . . . . .
. . . . .

Vv08g0297 . . . . . . .
. . . . . . . .
. . . . .
Vv08g0298 . Gorai.006G147900.1 . . .
. . . . . . . .
. . . . . . .
Vv08g0299 . . . . . . .
. . . . . . . .
. . . . .
Vv08g0300 . . . . . . .
. . . . . . . .
. . . . .
Vv08g0301 . . . . . . .
. . . . . . . .
. . . . .
Vv08g0302 . . . . . . .
. . . . . . . .
. . . . .
Vv08g0303 . . . . . . .
. . . . . . . .
. . . . .
Vv08g0304 . . . . . . .
. . . . . . . .
. . . . .
Vv08g0305 . . . . . . .
. . . . . . . .
. . . . .
Vv08g0306 . . . . . . .
. . . . . . . .
. . . . .
Vv08g0307 . . . . . . .
. . . . . . . .
. . . . .
Vv08g0308 . . . . . . .
. . . . . . . .
. . . . .
Vv08g0309 . . . . . . .
. . . . . . . .
. . . . .
Vv08g0310 . . . . . . .
. . . . . . . .
. . . . .
Vv08g0311 . . . . . . .
. . . . . . . .
. . . . .
Vv08g0312 . . . . . . .
. . . . . . . .
. . . . .
Vv08g0313 . . . . . . .
. . . . . . . .

. . . . .
Vv08g0314 . . . . . . .
. . . . . . . .
. . . . .
Vv08g0315 . . . . . . .
. . . . . . . .
. . . . .
Vv08g0316 . . . . . . .
. . . . . . . .
. . . . .
Vv08g0317 . . . . . . .
. . . . . . . .
. . . . .
Vv08g0318 . . . . . . .
. . . . . . . .
. . . . .
Vv08g0319 . . . . . . .
. . . . . . . .
. . . . .
Vv08g0320 . . . . . . .
. . . . . . . .
. . . . .
Vv08g0321 . . . . . . .
. . . . . . . .
. . . . .
Vv08g0322 . . . . . . .
. . . . . . . .
. . . . .
Vv08g0323 . . . . . . .
. . . . . . . .
. . . . .
Vv08g0324 . . . . . . .
. . . . . . . .
. . . . .
Vv08g0325 . . . . . . .
. . . . . . . .
. . . . .
Vv08g0326 . . . . . . .
. . . . . . . .
. . . . .
Vv08g0327 . . . . . . .
. . . . . . . .
. . . . .
Vv08g0328 . . . . . . .
. . . . . . . .
. . . . .
Vv08g0329 Tc05_g010960 Gorai.006G148800.1 Gorai.
002G234700.1 . . . . . . .
. . . . . . . .
. .

Vv08g0330 Tc05_g010950 . . . . .
. . . . . . . .
. . . . . .
Vv08g0331 Tc05_g010920 Gorai.006G148900.1 Gorai.
002G234800.1 . . . . . . .
. . . . . . . .
. .
Vv08g0332 . . . . . . .
. . . . . . . .
. . . . .
Vv08g0333 . Gorai.006G149000.1 . . .
. . . . . . . .
. . . . . . .
Vv08g0334 Tc05_g010840 . . . . .
. . . . . . . .
. . . . . .
Vv08g0335 Tc05_g010830 Gorai.006G149100.1 Gorai.
002G234900.1 . . . . . . .
. . . . . . . .
. .
Vv08g0336 . . . . . . .
. . . . . . . .
. . . . .
Vv08g0337 Tc05_g010780 Gorai.006G149200.1 . .
. . . . . . . .
. . . . . . . .
Vv08g0338 . . Gorai.002G235100.1 . .
. . . . . . . .
. . . . . . .
Vv08g0339 . . . . . . .
. . . . . . . .
. . . . .
Vv08g0340 . . . . . . .
. . . . . . . .
. . . . .
Vv08g0341 . Gorai.006G149300.1 . . .
. . . . . . . .
. . . . . . .
Vv08g0342 . Gorai.006G149400.1 Gorai.002G235200.1
. . . . . . . .
. . . . . . . .
.
Vv08g0343 . . . . . . .
. . . . . . . .
. . . . .
Vv08g0344 Tc05_g002480 . Gorai.002G263500.1 .
. . Vv06g0774 Tc09_g029900 . .
. Gorai.009G092000.1 . . . .
. . . .
Vv08g0345 . . . . . .

```
Vv06g0780       Tc09_g029870     .       .       .       Gorai.
009G091600.1     .       Vv13g0059       Tc10_g001480     .
Gorai.011G288000.1       .       Gorai.010G197900.1       .
Vv08g0346       Tc05_g002460     Gorai.006G182700.1       .       .
        .       .       .       .       .       .       .       .
        .       .       .       .       .       .       .       .
Vv08g0347       Tc05_g002450     .       Gorai.002G263300.1       .
        .       .       .       .       .       .       .       .
        .       .       .       .       .       .       .       .
Vv08g0348       .       .       .       .       .       .       .
        .       .       .       .       .       .       Vv13g0062
        Tc10_g001430     Gorai.013G018000.1       Gorai.011G288400.1
        .       .       .
Vv08g0349       Tc05_g002440     .       Gorai.002G263200.1       .
        Gorai.012G114200.1       .       .       .       .       .
        .       .       .       .       .       .       .       .
        .       .
Vv08g0350       Tc05_g002430     .       .       .       Gorai.
012G114500.1     .       .       .       .       .       .       .
        .       Vv13g0066       Tc10_g001400     Gorai.013G017700.1
        Gorai.011G289200.1       .       Gorai.010G196600.1       .
Vv08g0351       Tc05_g002420     Gorai.006G182800.1       .       .
        .       .       .       .       .       .       .       .
        .       .       .       .       .       .       .       .
Vv08g0352       .       .       .       .       .       .       .
        .       .       .       .       .       .       .       .
        .       .       .       .       .
Vv08g0353       Tc05_g002410     Gorai.006G182900.1       Gorai.
002G263100.1     .       .       .       .       .       .       .
        .       .       .       .       .       .       .       .
        .       .
Vv08g0354       Tc05_g002380     .       .       .       Gorai.
012G114600.1     .       .       .       .       .       .       .
        .       .       .       .       .       .       .       .
Vv08g0355       Tc05_g002370     Gorai.006G183000.1       Gorai.
002G262900.1     .       Gorai.012G114700.1       .       .       .
        .       .       .       .       .       .       .       .
        .       .       .       .
Vv08g0356       .       .       .       .       .       .       .
        .       .       .       .       .       .       .       .
        .       .       .       .       .
Vv08g0357       Tc05_g002310     .       Gorai.002G262800.1       .
        Gorai.012G115000.1       .       Vv06g0788
Tc09_g029750     .       Gorai.001G116500.1       .       .
Gorai.006G106100.1       Vv13g0073       Tc10_g001360     .
Gorai.011G289600.1       .       .       .
Vv08g0358       .       .       .       .       .       .       .
        .       .       .       .       .       .       .       .
        .       .       .       .       .
Vv08g0359       .       .       .       .       .       .       .
```

. . . . . . . .
. . . . .
Vv08g0360 Tc05_g002290 . . . Gorai.
012G115100.1 . . . . . . .
. Vv13g0075 . . . . .
.
Vv08g0361 Tc05_g002270 Gorai.006G183300.1 . .
. . . . . . . .
. . . . . . . .
Vv08g0362 . . . . . . .
. . . . . . . .
. . . . .
Vv08g0363 . . . . . . .
. . . . . . . .
. . . . .
Vv08g0364 . . . . . . .
. . . . . . . .
. . . . .
Vv08g0365 Tc05_g002250 . . . Gorai.
012G115300.1 . . . . . . .
. . . . . . . .
Vv08g0366 Tc05_g002240 Gorai.006G183400.1 . .
. . . . . . . .
. Vv13g0111 Tc10_g001070 Gorai.013G016800.1
. . Gorai.010G195600.1 .
Vv08g0367 . . . . . . .
. . . . . . . .
. . . . .
Vv08g0368 Tc05_g002230 . . . Gorai.
012G115400.1 . Vv06g0798 Tc09_g029660 .
Gorai.001G116900.1 . . Gorai.006G105300.1
Vv13g0130 Tc10_g000890 . . . Gorai.
010G195000.1 .
Vv08g0369 . . . . . . .
. . . . . . . .
. . . . .
Vv08g0370 . . . . . . .
. . . . . . . .
. . . . .
Vv08g0371 Tc05_g002210 . . . Gorai.
012G115600.1 . . . . . . .
. . . . . . . .
Vv08g0372 Tc05_g002200 Gorai.006G183500.1 . .
Gorai.012G115700.1 . . . . .
. . . . . . . .
. .
Vv08g0373 . . . . . . .
. . . . . . . .
. . . . .
Vv08g0374 Tc05_g002170 . . . Gorai.

012G116000.1 . . . . . . .
. . . . . . . .
Vv08g0375 . . . . . . .
. . . . . . . .
. . . . .
Vv08g0376 . . . . . . .
. . . . . . . .
. . . . .
Vv08g0377 . . . . . . .
. . . . . . . .
. . . . .
Vv08g0378 . . . . . . .
. . . . . . . .
. . . . .
Vv08g0379 . . . . . . .
. . . . . . . .
. . . . .
Vv08g0380 . . . . . . .
. . . . . . . .
. . . . .
Vv08g0381 Tc05_g002150 Gorai.006G183700.1 Gorai.
002G262700.1 . . . Vv06g0805 . .
. . . . . . . .
. . .
Vv08g0382 Tc05_g002140 . Gorai.002G262600.1 .
. . Vv06g0806 . Gorai.013G269700.1
. . Gorai.009G111700.1 . . .
. . . . .
Vv08g0383 Tc05_g002130 . Gorai.002G262500.1 .
Gorai.012G116700.1 . . . . .
. . . . . . . .
. .
Vv08g0384 . . . . . . .
. . . . . . . .
. . . . .
Vv08g0385 Tc05_g002120 Gorai.006G183800.1 .
Gorai.002G153200.1 Gorai.012G116800.1 . . .
. . . . . . . .
. . . .
Vv08g0386 Tc05_g002100 Gorai.006G183900.1 . .
. . Vv06g0808 . . . .
. . . . . . . .
.
Vv08g0387 Tc05_g002080 . . . Gorai.
012G116900.1 . . . . . . .
. . . . . . . .
Vv08g0388 . . . . . . .
. . . . . . . .
. . . . .
Vv08g0389 . . . . . . .

. . . . . . . .
. . . . .
Vv08g0390 . . . . . . .
. . . . . . . .
. . . . .
Vv08g0391 . . Gorai.002G259600.1 . .
. . . . . . . .
. . . . . . .
Vv08g0392 . . . . . . .
. . . . . . . .
. . . . .
Vv08g0393 . . . . . . .
. . . . . . . .
. . . . .
Vv08g0394 . . . . . . .
. . . . . . . .
. . . . .
Vv08g0395 . . . . . . .
. . . . . . . .
. . . . .
Vv08g0396 . . . . . . .
. . . . . . . .
. . . . .
Vv08g0397 . . . . . . .
. . . . . . . .
. . . . .
Vv08g0398 . . . . . . .
. . . . . . . .
. . . . .
Vv08g0399 Tc05_g002040 . Gorai.002G264400.1 .
Gorai.012G117000.1 . . . . .
. . . . . . . .
. .
Vv08g0400 Tc05_g002020 . Gorai.002G264500.1 .
Gorai.012G117100.1 . . . . .
. . . . . . . .
. .
Vv08g0401 . . . . . . .
. . . . . . . .
. . . . .
Vv08g0402 . . . . . . .
. . . . . . . .
. . . . .
Vv08g0403 . . . . . . .
. . . . . . . .
. . . . .
Vv08g0404 . . . . . . .
. . . . . . . .
. . . . .
Vv08g0405 . . . . . . .

. . . . . . . .
. . . . .
Vv08g0406 . . . . . . .
. . . . . . . .
. . . . .
Vv08g0407 . . . . . . .
. . . . . . . .
. . . . .
Vv08g0408 . . . . . . .
. . . . . . . .
. . . . .
Vv08g0409 . . . . . . .
. . . . . . . .
. . . . .
Vv08g0410 . . . . . . .
. . . . . . . .
. . . . .
Vv08g0411 Tc05_g001990 . Gorai.002G264600.1 .
Gorai.012G117200.1 . . . . .
. . . . . . . .
. .
Vv08g0412 . . . . . . .
. . . . . . . .
. . . . .
Vv08g0413 . . . . . . .
. . . . . . . .
. . . . .
Vv08g0414 . . . . . . .
. . . . . . . .
. . . . .
Vv08g0415 . . . . . . .
. . . . . . . .
. . . . .
Vv08g0416 Tc05_g001980 . . . Gorai.
012G117300.1 . . . . . . .
. . . . . . . .
Vv08g0417 Tc05_g001970 . Gorai.002G264900.1 .
Gorai.012G117400.1 . . . . .
. . . . . . . .
. .
Vv08g0418 Tc05_g001960 Gorai.006G184200.1 Gorai.
002G265100.1 . Gorai.012G117500.1 . Vv06g0815
. . . . Gorai.009G111900.1 .
. . . . . . .
Vv08g0419 Tc05_g001950 . . . . .
. . . . . . . .
. . . . . .
Vv08g0420 . . . . . . .
. . . . . . . .
. . . . .

Vv08g0421 . . Gorai.002G265200.1 .
Gorai.012G117600.1 . . . . . .
. . . . . . . .
.
Vv08g0422 . . . . . . .
. . . . . . . .
. . . . .
Vv08g0423 Tc05_g001940 . . . . .
. . . . . . . .
. . . . . .
Vv08g0424 . . . . . . .
. . . . . . . .
. . . . .
Vv08g0425 . . . . . . .
. . . . . . . .
. . . . .
Vv08g0426 . . . . . . .
. . . . . . . .
. . . . .
Vv08g0427 . . . . . . .
. . . . . . . .
. . . . .
Vv08g0428 . . . . . . .
. . . . . . . .
. . . . .
Vv08g0429 . . . . . . .
. . . . . . . .
. . . . .
Vv08g0430 . . . . . . .
. . . . . . . .
. . . . .
Vv08g0431 . . . . . . .
. . . . . . . .
. . . . .
Vv08g0432 . . . . . . .
. . . . . . . .
. . . . .
Vv08g0433 . . . . . . .
. . . . . . . .
. . . . .
Vv08g0434 . . . . . . .
. . . . . . . .
. . . . .
Vv08g0435 Tc05_g001910 . Gorai.002G265400.1 .
Gorai.012G117800.1 . Vv06g0821 . .
. . Gorai.009G112300.1 . Vv13g0101
Tc10_g001130 . . . . .
Vv08g0436 Tc05_g001900 . Gorai.002G265500.1 .
. . Vv06g0823 . Gorai.013G270200.1
. . . Gorai.003G147900.1 . .

. . . . .
Vv08g0437 . . . . . . .
. . . . . . . .
. . . . .
Vv08g0438 Tc05_g001880 . . . Gorai.
012G117900.1 . . . . . . .
. . . . . . . .
Vv08g0439 . . . . . . .
. . . . . . . .
. . . . .
Vv08g0440 Tc05_g001870 Gorai.006G184300.1 . .
. . . . . . . .
. . . . . . . .
Vv08g0441 . . . . . . .
. . . . . . . .
. . . . .
Vv08g0442 . . . . . . .
. . . . . . . .
. . . . .
Vv08g0443 . . . . . . .
. . . . . . . .
. . . . .
Vv08g0444 Tc05_g001860 . . . . .
. . . . . . . .
. . . . . .
Vv08g0445 . Gorai.006G184400.1 . .
Gorai.012G118000.1 . . . . . .
. . . . . . . .
.
Vv08g0446 . . . . . . .
. . . . . . . .
. . . . .
Vv08g0447 Tc05_g001850 . . . . .
. . . . . . . .
. . . . . .
Vv08g0448 . . . . . .
Vv06g0826 . Gorai.013G270400.1 . . .
Gorai.003G148200.1 . . . . .
. .
Vv08g0449 . . . . . . .
. . . . . . . .
. . . . .
Vv08g0450 Tc05_g001840 . Gorai.002G265700.1 .
. . . . . . . .
. . . . . . . .
Vv08g0451 . . . . . . .
. . . . . . . .
. . . . .
Vv08g0452 Tc05_g001810 Gorai.006G184500.1 . .
Gorai.012G118200.1 . Vv06g0833 . .

.	.	.	.	.	.	.	.
.	.	.
Vv08g0453	.	.	.	.	.	.	.
.	.	.	.	.	.	.	.
.	.	.	.	.
Vv08g0454	Tc05_g001790	.	Gorai.002G265900.1	.
Gorai.012G118300.1	.	.	.	.	.
.	.	.	.	.	.	.	.
.	.
Vv08g0455	.	.	.	.	.	.	.
.	.	.	.	.	.	.	.
.	.	.	.	.
Vv08g0456	Tc05_g001770	Gorai.006G184600.1	Gorai.
002G266000.1	.	Gorai.012G118500.1	.	Vv06g0838
.	.	.	.	.	.	.	.
.	.	.	.	.
Vv08g0457	Tc05_g001750	.	.	.	.	.
Vv06g0839	.	.	.	.	.	.
Vv13g0123	Tc10_g001000	.	.	Gorai.
009G322800.1	.	Gorai.012G079300.1
Vv08g0458	.	.	.	.	.	.	.
.	.	.	.	.	.	.	.
.	.	.	.	.
Vv08g0459	.	.	.	.	.	.	.
.	.	.	.	.	.	.	.
.	.	.	.	.
Vv08g0460	.	Gorai.006G184700.1	.	.
Gorai.012G118700.1	.	.	.	.	.	.
.	.	.	.	.	.	.	.
.
Vv08g0461	Tc05_g001740	Gorai.006G184800.1	.	.
.	.	.	.	.	.	.	.
.	.	.	.	.	.	.	.
Vv08g0462	Tc05_g001730	Gorai.006G184900.1	.	.
.	.	.	.	.	.	.	.
.	.	.	.	.	.	.	.
Vv08g0463	Tc05_g001720	Gorai.006G185000.1	Gorai.
002G266100.1	.	.	.	.	.	.	.
.	.	.	.	.	.	.	.
.	.
Vv08g0464	.	.	.	.	.	.	.
.	.	.	.	.	.	.	.
.	.	.	.	.
Vv08g0465	Tc05_g001710	.	.	.	Gorai.
012G118800.1	.	.	.	.	.	.	.
.	Vv13g0130	Tc10_g000890	.	.	.
Gorai.010G195000.1	.
Vv08g0466	.	.	.	.	.	.	.
.	.	.	.	.	.	.	.
.	.	.	.	.

Vv08g0467 Tc05_g001700 Gorai.006G185100.1 Gorai.
002G266200.1 . . . . . . .
. . . . . . . .
. .
Vv08g0468 . . . . . . .
. . . . . . . .
. . . . .
Vv08g0469 Tc05_g001680 . . . Gorai.
012G119100.1 . . . . . . .
. . . . . . . .
Vv08g0470 Tc05_g001670 . . . . .
. . . . . . . .
. . . . . .
Vv08g0471 Tc05_g001650 . Gorai.002G266300.1 .
. . . . . . . .
. . . . . . . .
Vv08g0472 Tc05_g001640 . . . . .
. . . . . . . .
. . . . . .
Vv08g0473 Tc05_g001630 Gorai.006G185200.1 Gorai.
002G266400.1 . . . . . . .
. . . . . . . .
. .
Vv08g0474 Tc05_g001620 . . . . .
. . . . . . . .
. . . . . .
Vv08g0475 . . . . . . .
. . . . . . . .
. . . . .
Vv08g0476 Tc05_g001610 Gorai.006G185300.1 Gorai.
002G266500.1 . . . . . . .
. . . Vv13g0139 Tc10_g000810 .
. . Gorai.010G193900.1 .
Vv08g0477 . . . . . . .
. . . . . . . .
. . . . .
Vv08g0478 Tc05_g001600 . . . . .
. . . . . . . .
. . . . . .
Vv08g0479 . . . . . . .
. . . . . . . .
. . . . .
Vv08g0480 Tc05_g001590 Gorai.006G185500.1 Gorai.
002G266600.1 . . . . . . .
. . . . . . . .
. .
Vv08g0481 Tc05_g001580 . . . Gorai.
012G119200.1 . . . . . . .
. . . . . . . .
Vv08g0482 Tc05_g001570 Gorai.006G185700.1 . .

. . . . . . . .
. . . . . . . .
Vv08g0483 . . . . . . .
. . . . . . . .
. . . . .
Vv08g0484 Tc05_g001560 Gorai.006G185800.1 Gorai.
002G266700.1 . . . . . . .
. . . . . . . .
. .
Vv08g0485 Tc05_g001550 . . . . .
. . . . . . . .
. . . . . .
Vv08g0486 . . . . . . .
. . . . . . . .
. . . . .
Vv08g0487 Tc05_g001540 . . . . .
Vv06g0876 . . . . . .
. . . . . . .
Vv08g0488 Tc05_g001520 Gorai.006G185900.1 . .
Gorai.012G119300.1 . . . . .
. . . . . . . .
. .
Vv08g0489 Tc05_g001510 . . . Gorai.
012G119400.1 . Vv06g0878 . . . .
. . . . . . . .
.
Vv08g0490 Tc05_g001500 . Gorai.002G266800.1 .
Gorai.012G119500.1 . . . . .
. . . . . . . .
. .
Vv08g0491 Tc05_g001480 . . . Gorai.
012G119600.1 . . . . . . .
. . . . . . . .
Vv08g0492 . . . . . . .
. . . . . . . .
. . . . .
Vv08g0493 Tc05_g001470 . Gorai.002G266900.1 .
. . . . . . . .
. . . . . . . .
Vv08g0494 . . . . . . .
. . . . . . . .
. . . . .
Vv08g0495 Tc05_g001450 . . . . .
. . . . . . . .
. . . . . .
Vv08g0496 . . . . . . .
. . . . . . . .
. . . . .
Vv08g0497 Tc05_g001440 . . . . .
. . . . . . . .

.	.	.	.	.	.
Vv08g0498	.	.	.	.	.	.	.
.	.	.	.	.	.	.	.
.	.	.	.	.
Vv08g0499	.	.	.	.	.	.	.
.	.	.	.	.	.	.	.
.	.	.	.	.
Vv08g0500	Tc05_g001430	.	.	.	Gorai.
012G119700.1	.	.	.	.	.	.	.
.	Vv13g0155	.	.	.	.
Gorai.010G193200.1	.
Vv08g0501	.	.	.	.	.	.	.
.	.	.	.	.	.	.	.
.	.	.	.	.
Vv08g0502	Tc05_g001420	Gorai.006G186000.1	Gorai.
002G267000.1	.	.	.	.	.	.	.
.	.	.	.	.	.	.	.
.	.
Vv08g0503	.	.	.	.	.	.	.
.	.	.	.	.	.	.	.
.	.	.	.	.
Vv08g0504	Tc05_g000290	.	Gorai.002G267100.1	.
.	.	.	.	.	.	.	.
.	.	.	.	.	.	.	.
Vv08g0505	.	.	.	.	.	.	.
.	.	.	.	.	.	.	.
.	.	.	.	.
Vv08g0506	Tc05_g000270	.	Gorai.002G267300.1	.
.	.	Vv06g0883	.	.	.	.
.	.	.	.	.	.	.	.
.
Vv08g0507	.	.	.	.	.	.	.
.	.	.	.	.	.	.	.
.	.	.	.	.
Vv08g0508	.	.	.	.	.	.	.
.	.	.	.	.	.	Vv13g0158
Tc10_g000660	.	.	.	Gorai.010G192900.1
.
Vv08g0509	Tc05_g000260	.	.	.	Gorai.
012G119800.1	.	.	.	.	.	.	.
.	.	.	.	.	.	.	.
Vv08g0510	.	.	.	.	.	.	.
.	.	.	.	.	.	Vv13g0159
.	.	.	.	.	.
Vv08g0511	.	.	.	.	.	.	.
.	.	.	.	.	.	.	.
.	.	.	.	.
Vv08g0512	.	.	.	.	.	.	.
.	.	.	.	.	.	.	.
.	.	.	.	.

Vv08g0513 . . . . . . .
. . . . . . . .
. . . . .
Vv08g0514 . . . . . .
Vv06g0891 . . . . . Gorai.
003G151100.1 . . . . . . .
Vv08g0515 . . . . . . .
. . . . . . . .
. . . . .
Vv08g0516 . . . . . . .
. . . . . . . .
. . . . .
Vv08g0517 . . . . . . .
. . . . . . . .
. . . . .
Vv08g0518 . . . . . . .
. . . . . . . .
. . . . .
Vv08g0519 . . . . . . .
. . . . . . . .
. . . . .
Vv08g0520 . . . . . . .
. . . . . . . .
. . . . .
Vv08g0521 . . . . . . .
. . . . . . . .
. . . . .
Vv08g0522 . . . . . . .
. . . . . . . .
. . . . .
Vv08g0523 . . . . . . .
. . . . . . . .
. . . . .
Vv08g0524 . . . . . .
Vv06g0894 . . . . . . .
. . . . . .
Vv08g0525 . . . . . . .
. . . . . . . .
. . . . .
Vv08g0526 . . . . . . .
. . . . . . . .
. . . . .
Vv08g0527 . . . . . . .
. . . . . . . .
. . . . .
Vv08g0528 . . . . . .
Vv06g0899 . . . . . .
Vv13g0164 . . . . . .
Vv08g0529 . . . . . . .
. . . . . . . .

. . . . .
Vv08g0530 . . . . . . .
. . . . . . . .
. . . . .
Vv08g0531 . . . . . . .
. . . . . . . .
. . . . .
Vv08g0532 . . . . . . .
. . . . . . . .
. . . . .
Vv08g0533 . . . . . . .
. . . . . . . .
. . . . .
Vv08g0534 . . . . . . .
. . . . . . . .
. . . . .
Vv08g0535 . . . . . . .
. . . . . . . .
. . . . .
Vv08g0536 . . . . . . .
. . . . . . . .
. . . . .
Vv08g0537 . . . . . . .
. . . . . . Vv13g0171
. . Gorai.011G210600.1 . Gorai.
010G217900.1 Gorai.012G015700.1
Vv08g0538 . . . . . . .
. . . . . . . .
. . . . .
Vv08g0539 . . . . . . .
. . . . . . . .
. . . . .
Vv08g0540 . . . . . . .
. . . . . . . .
. . . . .
Vv08g0541 . . . . . . .
. . . . . . . .
. . . . .
Vv08g0542 . . . . . . .
. . . . . . . .
. . . . .
Vv08g0543 . . . . . . .
. . . . . . . .
. . . . .
Vv08g0544 . . . . . . .
. . . . . . Vv13g0179
. . Gorai.011G210200.1 . .
Gorai.012G015500.1
Vv08g0545 . . . . . . .
. . . . . . . .

| | | | | | | | |
|---|---|---|---|---|---|---|---|
| . | . | . | . | . | | | |
| Vv08g0546 | . | . | . | . | . | . | . |
| . | . | . | . | . | . | . | . |
| . | . | . | . | . | | | |
| Vv08g0547 | . | . | . | . | . | . | . |
| . | . | . | . | . | . | . | . |
| . | . | . | . | . | | | |
| Vv08g0548 | . | . | . | . | . | . | |
| Vv06g0920 | . | . | . | . | . | . | . |
| . | . | . | . | . | . | | |
| Vv08g0549 | . | . | . | . | . | . | . |
| . | . | . | . | . | . | . | . |
| . | . | . | . | . | | | |
| Vv08g0550 | . | . | . | . | . | . | . |
| . | . | . | . | . | . | . | . |
| . | . | . | . | . | | | |
| Vv08g0551 | . | . | . | . | . | . | . |
| . | . | . | . | . | . | . | . |
| . | . | . | . | . | | | |
| Vv08g0552 | . | . | . | . | . | . | |
| Vv06g0923 | . | . | . | . | . | . | |
| Vv13g0187 | . | . | . | . | . | . | |
| Vv08g0553 | . | . | . | . | . | . | . |
| . | . | . | . | . | . | . | . |
| . | . | . | . | . | | | |
| Vv08g0554 | . | . | . | . | . | . | . |
| . | . | . | . | . | . | . | . |
| . | . | . | . | . | | | |
| Vv08g0555 | Tc05_g028090 | | . | . | . | . | . |
| . | . | . | . | . | . | . | . |
| . | . | . | . | . | . | | |
| Vv08g0556 | . | . | . | . | . | . | . |
| . | . | . | . | . | . | . | . |
| . | . | . | . | . | | | |
| Vv08g0557 | Tc05_g024960 | | . | Gorai.007G266900.1 | | | . |
| . | . | Vv06g0927 | | . | Gorai.013G272000.1 | | |
| . | . | . | . | Vv13g0211 | | | |
| Tc10_g016330 | Gorai.013G007000.1 | | | Gorai.011G209100.1 | | | . |
| . | Gorai.012G014400.1 | | | | | | |
| Vv08g0558 | . | . | . | . | . | . | . |
| . | . | . | . | . | . | . | . |
| . | . | . | . | . | | | |
| Vv08g0559 | . | . | . | . | . | . | . |
| . | . | . | . | . | . | . | . |
| . | . | . | . | . | | | |
| Vv08g0560 | . | . | . | . | . | . | . |
| . | . | . | . | . | . | . | . |
| . | . | . | . | . | | | |
| Vv08g0561 | . | . | . | . | . | . | . |
| . | . | . | . | . | . | . | . |

. . . . .
Vv08g0562 . . . . . . .
. . . . . . . .
. . . . .
Vv08g0563 . . . . . . .
. . . . . . . .
. . . . .
Vv08g0564 . . . . . . .
. . . . . . . .
. . . . .
Vv08g0565 . . . . . . .
. . . . . . . .
. . . . .
Vv08g0566 . . . . . . .
. . . . . . . .
. . . . .
Vv08g0567 . . . . . .
Vv06g0938 . . . . . . .
. . . . . .
Vv08g0568 . . . . . . .
. . . . . . . .
. . . . .
Vv08g0569 . . . . . . .
. . . . . . . .
. . . . .
Vv08g0570 . . . . . . .
. . . . . . . .
. . . . .
Vv08g0571 . . . . . . .
. . . . . . . .
. . . . .
Vv08g0572 . . . . . . .
. . . . . . Vv13g0228
. Gorai.013G002900.1 . . . .
Vv08g0573 . . . . . . .
. . . . . . . .
. . . . .
Vv08g0574 Tc05_g005710 . . . . .
. . . . . . .
Vv13g0231 Tc10_g016560 . . . .
Gorai.012G014000.1
Vv08g0575 . . . . . . .
. . . . . . . .
. . . . .
Vv08g0576 . . . . . . .
. . . . . . . .
. . . . .
Vv08g0577 . . . . . . .
. . . . . . Vv13g0235
Tc10_g016600 . . . . .

```
Vv08g0578 . . . . . . .
. . . . . . . .
. . . . .
Vv08g0579 . . . . . . .
. . . . . . . .
. . . . .
Vv08g0580 . . . . . . .
. . . . . . . .
. . . . .
Vv08g0581 . . . . . . .
. . . . . . . .
. . . . .
Vv08g0582 . . . . . . .
. . . . . . . .
. . . . .
Vv08g0583 Tc05_g028320 . . . . .
. . . . . . . .
. . . . . .
Vv08g0584 . . . . . . .
. . . . . . . .
. . . . .
Vv08g0585 . . . . . . .
. . . . . . . .
. . . . .
Vv08g0586 . . . . . . .
. . . . . . . .
. . . . .
Vv08g0587 . . . . . . .
. . . . . . . .
. . . . .
Vv08g0588 Tc05_g005850 Gorai.006G169000.1 . .
. . . . . . . .
. . . . . . . .
Vv08g0589 Tc05_g024980 . Gorai.007G267200.1 .
. . . . . . . .
. . . . . . . .
Vv08g0590 . . . . . . .
. . . . . . . .
. . . . .
Vv08g0591 Tc05_g023640 . . . . .
Vv06g0947 Tc09_g024080 . . . . .
. . . . . . . .
Vv08g0592 . . . . . . .
. . . . . . . .
. . . . .
Vv08g0593 . . . . . . .
. . . . . . . .
. . . . .
Vv08g0594 . . . . . . .
. . . . . . . .
```

. . . . .
Vv08g0595 . . . . . . .
. . . . . . . .
. . . . .
Vv08g0596 . . . . . .
Vv11g0480 Tc09_g010570 . . . Gorai.
009G021900.1 . . . . . . .
.
Vv08g0597 . . . . . . .
. . . . . . . .
. . . . .
Vv08g0598 Tc05_g028350 . . . . .
Vv11g0478 Tc09_g010550 . Gorai.001G002800.1
. Gorai.009G022100.1 Gorai.010G067300.1 .
. . . . . .
Vv08g0599 . . . . . . .
. . . . . . . .
. . . . .
Vv08g0600 Tc05_g028570 . . . . .
Vv11g0459 Tc09_g010330 . . .
Gorai.009G022700.1 Gorai.010G068600.1 . . .
. . . .
Vv08g0601 Tc05_g006090 . Gorai.002G249400.1 .
. . . . . . . .
. . . . . . . .
Vv08g0602 . . . . . . .
. . . . . . . .
. . . . .
Vv08g0603 . . . . . . .
. . . . . . . .
. . . . .
Vv08g0604 . . . . . . .
. . . . . . . .
. . . . .
Vv08g0605 . . . . . . .
. . . . . . . .
. . . . .
Vv08g0606 . . . . . . .
. . . . . . . .
. . . . .
Vv08g0607 . . . . . . .
. . . . . . . .
. . . . .
Vv08g0608 . . . . . . .
. . . . . . . .
. . . . .
Vv08g0609 . . . . . . .
. . . . . . . .
. . . . .
Vv08g0610 . . . . . . .

. . . . . . . .
. . . . .
Vv08g0611 . . . . . . .
. . . . . . . .
. . . . .
Vv08g0612 . . . . . . .
. . . . . . . .
. . . . .
Vv08g0613 . . . . . . .
. . . . . . . .
. . . . .
Vv08g0614 . . . . . . .
. . . . . . . .
. . . . .
Vv08g0615 . . . . . Gorai.
007G351000.1 . . . . . . .
. . . . . . .
Vv08g0616 . . . . . . .
. . . . . . . .
. . . . .
Vv08g0617 Tc05_g006160 . . . . .
. . . . . . . .
. . . . . .
Vv08g0618 . . . . . . .
. . . . . . . .
. . . . .
Vv08g0619 . . . . . . .
. . . . . . . .
. . . . .
Vv08g0620 . . . . . . .
. . . . . . . .
. . . . .
Vv08g0621 . . . . . . .
. . . . . . . .
. . . . .
Vv08g0622 . . . . . . .
. . . . . . . .
. . . . .
Vv08g0623 Tc05_g023620 . Gorai.007G256400.1 .
. Gorai.007G350900.1 . . . .
. . . . . . . .
. .
Vv08g0624 . . . . . . .
. . . . . . . .
. . . . .
Vv08g0625 Tc05_g028670 . . . . .
. . . . . . . .
. . . . . .
Vv08g0626 . . . . . . .
. . . . . . . .

```
          .         .         .         .         .
Vv08g0627           Tc05_g015950        .         .         .         .         .
          .         .         .         .         .         .         .         .
          .         .         .         .         .         .
Vv08g0628           Tc05_g025070        .         .         .         .         .
          .         .         .         .         .         .         .         .
          .         .         .         .         .         .
Vv08g0629           .         .         .         .         .         .         .
          .         .         .         .         .         .         .         .
          .         .         .         .         .
Vv08g0630           .         .         .         .         .         .         .
          .         .         .         .         .         .         .         .
          .         .         .         .         .
Vv08g0631           Tc05_g015920        .         .         .         .         .
          .         .         .         .         .         .         .         .
          .         .         .         .         .         .
Vv08g0632           .         .         .         .         .         .         .
          .         .         .         .         .         .         .         .
          .         .         .         .         .
Vv08g0633           .         .         .         .         .         .         .
          .         .         .         .         .         .         .         .
          .         .         .         .         .
Vv08g0634           Tc05_g015620        .         .         .         .         .
          Vv11g0447           .         .         .         .         .         .
          .         .         .         .         .         .         .
Vv08g0635           .         .         .         .         .         .         .
          .         .         .         .         .         .         .         .
          .         .         .         .         .
Vv08g0636           .         .         .         .         .         Gorai.
007G350800.1        Vv11g0408           Tc09_g009860        .         .         .
          .         Gorai.010G070800.1            .         .         .         .
          .         .         .
Vv08g0637           .         .         .         .         .         .         .
          .         .         .         .         .         .         .         .
          .         .         .         .         .
Vv08g0638           .         .         .         .         .         .         .
          .         .         .         .         .         .         .         .
          .         .         .         .         .
Vv08g0639           .         .         .         .         .         .         .
          .         .         .         .         .         .         .         .
          .         .         .         .         .
Vv08g0640           .         .         .         .         .         .         .
          .         .         .         .         .         .         .         .
          .         .         .         .         .
Vv08g0641           Tc05_g025560        .         .         .         .         .
          .         .         .         .         .         .         .         .
          .         .         .         .         .         .
Vv08g0642           .         .         .         .         .         .
Vv11g0393           Tc09_g009680        .         .         .         Gorai.
009G025400.1        Gorai.010G072100.1            Vv03g0771
```

Tc02_g010000 Gorai.013G049500.1 . . . .
Vv08g0643 . . . . . Gorai.
007G351100.1 . . . . . . .
. . . . . . .
Vv08g0644 . . . . . . .
. . . . . . . .
. . . . .
Vv08g0645 . . . . . . .
. . . . . . . .
. . . . .
Vv08g0646 . . . . . . .
. . . . . . . .
. . . . .
Vv08g0647 . . . . . . .
. . . . . . . .
. . . . .
Vv08g0648 . . . . . . .
. . . . . . . .
. . . . .
Vv08g0649 . . . . . . .
. . . . . . . .
. . . . .
Vv08g0650 Tc05_g020760 . . . . .
. . . . . . . .
. . . . . .
Vv08g0651 . . . . . . .
. . . . . . . .
. . . . .
Vv08g0652 . . . . . . .
. . . . . . . .
. . . . .
Vv08g0653 . . . . . . .
. . . . . . . .
. . . . .
Vv08g0654 . . . . . . .
. . . . . . . .
. . . . .
Vv08g0655 . . . . . . .
. . . . . . . .
. . . . .
Vv08g0656 . . . . . Gorai.
007G351300.1 . . . . . . .
. . . . . . .
Vv08g0657 . . . . . . .
. . . . . . . .
. . . . .
Vv08g0658 . . . . . . .
. . . . . . . .
. . . . .
Vv08g0659 . . . . . . .

. . . . . . . .
. . . . .
Vv08g0660 . . . . . . .
. . . . . . . .
. . . . .
Vv08g0661 . . . . . . .
. . . . . . Vv03g0788
Tc02_g010170 Gorai.013G050300.1 Gorai.005G231800.1
Gorai.002G010300.1 . .
Vv08g0662 Tc05_g025820 . . . . .
. . . . . . . .
. . . . . .
Vv08g0663 . . . . . . .
. . . . . . . .
. . . . .
Vv08g0664 . . . . . . .
. . . . . . . .
. . . . .
Vv08g0665 Tc05_g013910 . . . . .
. . . . . . . .
. . . . . .
Vv08g0666 . . . . . . .
. . . . . . . .
. . . . .
Vv08g0667 Tc05_g013960 . . . . .
. . . . . . . .
. . . . . .
Vv08g0668 Tc05_g013970 . . . . .
. . . . . . . .
. . . . . .
Vv08g0669 . . . . . . .
. . . . . . . .
. . . . .
Vv08g0670 . . . . . . .
. . . . . . . .
. . . . .
Vv08g0671 . . . . . . .
. . . . . . . .
. . . . .
Vv08g0672 . . . . . . .
. . . . . . . .
. . . . .
Vv08g0673 . . . . . . .
. . . . . . . .
. . . . .
Vv08g0674 Tc05_g002920 Gorai.006G180700.1 . .
. . Vv11g0370 . . . .
. . . . . . . .
.
Vv08g0675 . . . . . . .

. . . . . . . .
. . . . .
Vv08g0676 Tc05_g014000 . . . . .
. . . . . . . .
. . . . . .
Vv08g0677 . . . . . . .
. . . . . . . .
. . . . .
Vv08g0678 . . . . . . .
. . . . . . . .
. . . . .
Vv08g0679 . . . . . . .
. . . . . . . .
. . . . .
Vv08g0680 Tc05_g014010 . . . . .
. . . . . . . .
. . . . . .
Vv08g0681 . . . . . . .
. . . . . . . .
. . . . .
Vv08g0682 Tc05_g014020 . . . . .
. . . . . . . .
. . . . . .
Vv08g0683 Tc05_g014150 . . . .
Gorai.007G351500.1 . . . . . .
. . . . . . . .
Vv08g0684 Tc05_g025880 . . . . .
. . . . . . . .
. . . . . .
Vv08g0685 . . . . . . .
. . . . . . . .
. . . . .
Vv08g0686 Tc05_g020840 . Gorai.007G249200.1 .
. Gorai.007G348100.1 . . . .
. . . . . . . .
. .
Vv08g0687 . . . . . Gorai.
007G351700.1 Vv03g0815 . Gorai.013G050900.1
Gorai.005G230300.1 . . . . . .
. . . .
Vv08g0688 . . . . . . .
. . . . . . . .
. . . . .
Vv08g0689 . . . . . . .
. . . . . . . .
. . . . .
Vv08g0690 . . . . . . .
. . . . . . . .
. . . . .
Vv08g0691 . . . . . . .

```
                .       .       .       .       .       .       .       .
                .       .       .       .       .
Vv08g0692               .       .       .       .       .      Gorai.
007G351800.1            .       .       .       .       .       .       .
                .       .       .       .       .       .       .
Vv08g0693       Tc05_g003210            .       .       .       .
Gorai.007G346000.1      Vv03g0870               .       .       .       .
                .       .       .       .       .       .       .       .
                .
Vv08g0694               .       .       .       .       .       .       .
                .       .       .       .       .       .       .       .
                .       .       .       .       .
Vv08g0695               .       .       .       .       .      Gorai.
007G343200.1            .       .       .       .       .       .       .
                .       .       .       .       .       .       .
Vv08g0696               .       .       .       .       .       .       .
                .       .       .       .       .       .       .       .
                .       .       .       .       .
Vv08g0697       Tc05_g010370            .       .       .       .       .
                .       .       .       .       .       .       .       .
                .       .       .       .       .       .
Vv08g0698       Tc05_g025920            .       .       .       .       .
                .       .       .       .       .       .       .       .
                .       .       .       .       .       .
Vv08g0699               .       .       .       .       .       .       .
                .       .       .       .       .       .       .       .
                .       .       .       .       .
Vv08g0700               .       .       .       .       .       .
Vv03g0859       Tc02_g013810            .       .       .       .       .
        Vv03g0827               .       .       .       .       .       .
Vv08g0701               .       .       .       .       .       .
Vv03g0852       Tc02_g013830            .       .       .       .       .
        Vv03g0830               .       .       .       .       .       .
Vv08g0702               .       .       .       .       .       .
Vv03g0844               .       .       .       .       .       .
Vv03g0832               .       .       .       .       .       .
Vv08g0703               .       .       .       .       .       .       .
                .       .       .       .       .       .       .       .
                .       .       .       .       .
Vv08g0704               .       .       .       .       .       .
Vv03g0840       Tc02_g013860            .       .       .       .       .
        Vv03g0834               .       .       .       .       .       .
Vv08g0705               .       .       .       .       .       .       .
                .       .       .       .       .       .       .       .
                .       .       .       .       .
Vv08g0706               .       .       .       .       .       .
Vv03g0832               .       .       .       .       .       .
Vv03g0840       Tc02_g013860            .       .       .       .       .
Vv08g0707               .       .       .       .       .       .       .
                .       .       .       .       .       .       .       .
```

. . . . .
Vv08g0708 . . . . . .
Vv03g0827 . . . . . .
Vv03g0844 . . . . . .
Vv08g0709 . . . . . . .
. . . . . . . .
. . . . .
Vv08g0710 . . . . . . .
. . . . . . . .
. . . . .
Vv08g0711 . . . . . Gorai.
007G352100.1 . . . . . . .
. . . . . . .
Vv08g0712 Tc05_g010580 . . . . .
. . . . . . . .
. . . . . .
Vv08g0713 . . . . . . .
. . . . . . . .
. . . . .
Vv08g0714 Tc05_g010650 . . . . .
. . . . . . . .
. . . . . .
Vv08g0715 . . . . . . .
. . . . . . . .
. . . . .
Vv08g0716 . . . . . . .
. . . . . . . .
. . . . .
Vv08g0717 . . . . . . .
. . . . . . . .
. . . . .
Vv08g0718 . . . . . . .
. . . . . . . .
. . . . .
Vv08g0719 . . . . . Gorai.
007G352200.1 . . . . . . .
. . . . . . .
Vv08g0720 . . . . . . .
. . . . . . . .
. . . . .
Vv08g0721 . . . . . Gorai.
007G352300.1 . . . . . . .
. . . . . . .
Vv08g0722 Tc05_g026340 . . . . .
. . . . . . . .
. . . . . .
Vv08g0723 . . . . . . .
. . . . . . . .
. . . . .
Vv08g0724 . . . . . Gorai.

007G352400.1 . . . . . . .
. . . . . . .
Vv08g0725 . . . . . . .
. . . . . . . .
. . . . .
Vv08g0726 . . . . . . .
. . . . . . . .
. . . . .
Vv08g0727 . . . . . Gorai.
007G352500.1 . . . . . . .
. . . . . . .
Vv08g0728 Tc05_g007050 Gorai.006G163100.1 Gorai.
002G247000.1 . . . . . . .
. . . . . . . .
. .
Vv08g0729 . . . . . .
Vv13g0692 . . . . . . .
. . . . . .
Vv08g0730 Tc05_g007030 . . . . .
. . . . . . . .
. . . . . .
Vv08g0731 Tc05_g007020 . . . . .
. . . . . . . .
. . . . . .
Vv08g0732 Tc05_g007000 Gorai.006G163200.1 . .
. . . . . . . .
. . . . . . . .
Vv08g0733 Tc05_g006970 Gorai.006G163300.1 . .
Gorai.009G414600.1 . . . . .
. . . . . . . .
. .
Vv08g0734 Tc05_g006840 Gorai.006G164000.1 . .
. . . . . . . .
. . . . . . . .
Vv08g0735 Tc05_g006820 Gorai.006G164200.1 . .
. . . . . . . .
. . . . . . . .
Vv08g0736 Tc05_g006810 Gorai.006G164300.1 . .
. . . . . . . .
. . . . . . . .
Vv08g0737 Tc05_g006800 Gorai.006G164400.1 . .
. Gorai.007G373200.1 . . . .
. . . . . . . .
. .
Vv08g0738 Tc05_g006780 Gorai.006G164600.1 . .
. . . . . . . .
. . . . . . . .
Vv08g0739 Tc05_g006770 Gorai.006G164700.1 . .
. . . . . . . .
. . . . . . . .

Vv08g0740 Tc05_g006760 . Gorai.002G247700.1 .
Gorai.009G414900.1 . . . . .
. . . . . . . .
. .
Vv08g0741 Tc05_g006750 . . . . .
. . . . . . . .
. . . . . .
Vv08g0742 Tc05_g006740 Gorai.006G164800.1 . .
. Gorai.007G373300.1 . . . .
. . . . . . . .
. .
Vv08g0743 . . . . . . .
. . . . . . . .
. . . . .
Vv08g0744 . Gorai.006G165000.1 . . .
. . . . . . . .
. . . . . . .
Vv08g0745 . . . . . . .
. . . . . . . .
. . . . .
Vv08g0746 Tc05_g006700 . . . . .
. . . . . . . .
. . . . . .
Vv08g0747 Tc05_g006690 Gorai.006G165100.1 . .
. . . . . . . .
. . . . . . . .
Vv08g0748 . . . . . . .
. . . . . . . .
. . . . .
Vv08g0749 Tc05_g006680 Gorai.006G165200.1 . .
Gorai.009G415400.1 . . . . .
. . . . . . . .
. .
Vv08g0750 . . . . . . .
. . . . . . . .
. . . . .
Vv08g0751 . . . . . . .
. . . . . . . .
. . . . .
Vv08g0752 Tc05_g006660 . . . Gorai.
009G415500.1 . . . . . . .
. . . . . . . .
Vv08g0753 . Gorai.006G165300.1 . . .
. Vv06g0292 Tc09_g033700 . . .
. . . . . . . .
.
Vv08g0754 . . . . . . .
. . . . . . . .
. . . . .
Vv08g0755 Tc05_g006640 . . . . .

. . . . . . . .
. . . . . .
Vv08g0756 Tc05_g006630 . . . Gorai.
009G415700.1 . . . . . . .
. . . . . . . .
Vv08g0757 . . . . . . .
. . . . . . . .
. . . . .
Vv08g0758 Tc05_g006620 . Gorai.002G247800.1 .
. Gorai.007G373500.1 Vv06g0297 . .
. . . . . . . .
. . .
Vv08g0759 . . . . . . .
. . . . . . . .
. . . . .
Vv08g0760 Tc05_g006590 Gorai.006G165700.1 . .
Gorai.009G416200.1 . . . . .
. . . . . . . .
. .
Vv08g0761 . . . . . . .
. . . . . . . .
. . . . .
Vv08g0762 Tc05_g006560 Gorai.006G165900.1 . .
. . . . . . . .
. . . . . . . .
Vv08g0763 Tc05_g006550 Gorai.006G166000.1 Gorai.
002G248000.1 . Gorai.009G416300.1 Gorai.007G373600.1
Vv06g0312 . . . . .
Gorai.006G166000.1 Vv13g0688 Tc10_g005730 .
Gorai.011G238400.1 . . .
Vv08g0764 . . . . . . .
. . . . . . . .
. . . . .
Vv08g0765 Tc05_g006540 . . . . .
. . . . . . . .
. . . . . .
Vv08g0766 . . . . . . .
. . . . . . . .
. . . . .
Vv08g0767 . Gorai.006G166200.1 . .
Gorai.009G416700.1 Gorai.007G373800.1 . . .
. . . . . . . .
. . .
Vv08g0768 Tc05_g006530 . . . . .
. . . . . . . .
. . . . . .
Vv08g0769 . . . . . . .
. . . . . . . .
. . . . .
Vv08g0770 Tc05_g006520 Gorai.006G166300.1 . .

Gorai.009G416900.1 . . . . .
. . . . . . . .
. .
Vv08g0771 Tc05_g006510 . Gorai.002G248200.1 .
. . Vv06g0316 Tc09_g033600 . .
. Gorai.009G108500.1 Gorai.003G143300.1 .
. . . . . .
Vv08g0772 . . . . . . .
. . . . . . . .
. . . . .
Vv08g0773 Tc05_g006490 . Gorai.002G248300.1 .
Gorai.009G417300.1 . Vv06g0317
Tc09_g033590 . . . Gorai.009G108400.1
Gorai.003G143200.1 . . . . . .
.
Vv08g0774 Tc05_g006480 . . . . .
. . . . . . . .
. . . . . .
Vv08g0775 Tc05_g006470 . . . Gorai.
009G417400.1 . . . . . . .
. Vv13g0684 Tc10_g005650 Gorai.013G027500.1
Gorai.011G238600.1 . . .
Vv08g0776 Tc05_g006450 Gorai.006G166400.1 . .
. . Vv06g0326 Tc09_g033510 Gorai.
013G267100.1 . . Gorai.009G107800.1 Gorai.
006G120400.1 Vv13g0680 Tc10_g005610 . Gorai.
011G238900.1 . . Gorai.012G004800.1
Vv08g0777 Tc05_g006440 . . . Gorai.
009G417500.1 . Vv06g0330 Tc09_g033480 . .
. Gorai.009G107600.1 Gorai.003G142300.1
Vv13g0676 . Gorai.013G027400.1 . . .
.
Vv08g0778 Tc05_g006430 Gorai.006G166500.1 . .
Gorai.009G417600.1 . . . . .
. . . . . . . .
. .
Vv08g0779 . . . . . . .
. . . . . . . .
. . . . .
Vv08g0780 Tc05_g006420 Gorai.006G166600.1 . .
Gorai.009G417700.1 . Vv06g0335
Tc09_g033430 . . . . Gorai.006G166600.1
. . . . . . .
Vv08g0781 Tc05_g006410 Gorai.006G166700.1 . .
. . . . . . . .
. Vv13g0672 . Gorai.013G027300.1 .
. . .
Vv08g0782 Tc05_g006380 Gorai.006G166800.1 Gorai.
002G248400.1 . Gorai.009G418100.1 . Vv06g0337
Tc09_g033410 . . . Gorai.009G107200.1

Gorai.006G166800.1 . . . . .
. .
Vv08g0783 . . . . . . .
. . . . . . . .
. . . . .
Vv08g0784 Tc05_g006360 . . . Gorai.
009G418200.1 . . . . . . .
. . . . . . . .
Vv08g0785 Tc05_g006280 Gorai.006G167000.1 . .
. . . . . . . .
. . . . . . . .
Vv08g0786 Tc05_g006270 Gorai.006G167100.1 . .
. . . . . . . .
. . . . . . . .
Vv08g0787 Tc05_g006250 Gorai.006G167300.1 . .
Gorai.009G418300.1 Gorai.007G374000.1 Vv06g0351
. . . . . Gorai.006G167300.1
. . . . . . .
Vv08g0788 Tc05_g006230 . Gorai.002G248600.1 .
. . . . . . . .
. . . . . . . .
Vv08g0789 . Gorai.006G167400.1 . .
Gorai.009G418400.1 . . . . . .
. . . . . . . .
.
Vv08g0790 Tc05_g006220 . . . . .
. . . . . . . .
. . . . . .
Vv08g0791 . . . . . Gorai.
007G374100.1 . . . . . . .
. . . . . . .
Vv08g0792 Tc05_g006210 . . . . .
. . . . . . . .
. . . . . .
Vv08g0793 . . . . . . .
. . . . . . . .
. . . . .
Vv08g0794 . . . . . . .
. . . . . . . .
. . . . .
Vv08g0795 Tc05_g006200 Gorai.006G167500.1 Gorai.
002G248700.1 . . . . . . .
. . . . . . . .
. .
Vv08g0796 Tc05_g006190 Gorai.006G167600.1 . .
Gorai.009G418600.1 . Vv06g0362
Tc09_g025680 . Gorai.001G131000.1 . .
Gorai.006G167600.1 . . . . . .
.
Vv08g0797 Tc05_g006180 . . . . .

. . . . . . . .
. . . . . .
Vv08g0798 . . . . . . .
. . . . . . . .
. . . . .
Vv08g0799 Tc05_g006170 . Gorai.002G248800.1 .
Gorai.009G418800.1 . Vv06g0364
Tc09_g025710 . . . . Gorai.006G120200.1
. . . . . . .
Vv08g0800 . . . . . . .
. . . . . . . .
. . . . .
Vv08g0801 . . . . . . .
. . . . . . . .
. . . . .
Vv08g0802 Tc05_g006160 Gorai.006G167700.1 . .
. . . . . . . .
. . . . . . . .
Vv08g0803 . . . . . . .
. . . . . . . .
. . . . .
Vv08g0804 Tc05_g006150 . . . . .
Vv06g0366 Tc09_g025760 . . .
Gorai.009G078600.1 . Vv13g0646 Tc10_g005220 .
Gorai.011G240700.1 . Gorai.010G205100.1
Gorai.012G004100.1
Vv08g0805 Tc05_g006140 . . . . .
Vv06g0367 . . Gorai.001G130800.1 .
Gorai.009G078700.1 . Vv13g0644 . .
. . Gorai.010G205000.1 .
Vv08g0806 Tc05_g006130 Gorai.006G167900.1 . .
Gorai.009G419000.1 . . . . .
. . . . . . . .
. .
Vv08g0807 Tc05_g006120 . . . . .
. . . . . . . .
. . . . . .
Vv08g0808 Tc05_g006110 . Gorai.002G249200.1 .
. . . . . . . .
. . . . . . . .
Vv08g0809 . . . . . .
Vv06g0371 . . . . . . .
. . . . . .
Vv08g0810 . . . . . . .
. . . . . . . .
. . . . .
Vv08g0811 . . . . . . .
. . . . . . . .
. . . . .
Vv08g0812 . . . . . . .

. . . . . . . .
. . . . .
Vv08g0813 . . . . . . .
. . . . . . . .
. . . . .
Vv08g0814 . . . . . . .
. . . . . . . .
. . . . .
Vv08g0815 Tc05_g006080 . Gorai.002G249500.1 .
. . . . . . . .
. . . . . . . .
Vv08g0816 Tc05_g006070 Gorai.006G168200.1 . .
Gorai.009G419500.1 . . . . .
. . . . . . . .
. .
Vv08g0817 Tc05_g006060 . . . Gorai.
009G419600.1 . . . . . . .
. . . . . . . .
Vv08g0818 . . . . . . .
. . . . . . . .
. . . . .
Vv08g0819 Tc05_g006040 . . . . .
. . . . . . . .
. . . . . .
Vv08g0820 Tc05_g006030 . . . . .
. . . . . . . .
. . . . . .
Vv08g0821 Tc05_g006020 . . . .
Gorai.007G374300.1 . . . . . .
. . . . . . . .
Vv08g0822 Tc05_g031420 . . . . .
. . . . . . . .
. . . . . .
Vv08g0823 . . . . . . .
. . . . . . . .
. . . . .
Vv08g0824 Tc05_g006010 . Gorai.002G249600.1 .
. . Vv06g0377 . . Gorai.
001G130700.1 . . . Vv13g0630
Tc10_g005090 . Gorai.011G241200.1 . Gorai.
010G204400.1 .
Vv08g0825 . . . . . . .
. . . . . . . .
. . . . .
Vv08g0826 Tc05_g006000 . . . . .
. . . . . . . .
. . . . . .
Vv08g0827 . . . . . . .
. . . . . . . .
. . . . .

Vv08g0828 Tc05_g005990 Gorai.006G168400.1 Gorai.
002G249700.1 . . . . . . .
. . . Vv13g0627 Tc10_g005040 .
. Gorai.009G314900.1 Gorai.010G204200.1 .
Vv08g0829 Tc05_g005980 . Gorai.002G249800.1 .
. . . . . . . .
. . . . . . . .
Vv08g0830 Tc05_g005970 . . . . .
Vv06g0391 . . Gorai.001G130100.1 .
. Gorai.006G111500.1 . . . .
. . .
Vv08g0831 . . . . . . .
. . . . . . . .
. . . . .
Vv08g0832 . . . . . . .
. . . . . . . .
. . . . .
Vv08g0833 . . . . . . .
. . . . . . . .
. . . . .
Vv08g0834 . . . . . . .
. . . . . . . .
. . . . .
Vv08g0835 Tc05_g005930 Gorai.006G168700.1 Gorai.
002G250200.1 . . . . . . .
. . . . . . . .
. .
Vv08g0836 Tc05_g005910 Gorai.006G168800.1 Gorai.
002G250300.1 . . Gorai.007G374500.1 Vv06g0408
. . Gorai.001G129300.1 . . .
. . . . . . .
Vv08g0837 . . Gorai.002G250400.1 . .
. . . . . . . .
. . . . . . .
Vv08g0838 . . . . . . .
. . . . . . . .
. . . . .
Vv08g0839 . . . . . . .
. . . . . . . .
. . . . .
Vv08g0840 . . . . . . .
. . . . . . . .
. . . . .
Vv08g0841 . . . . . . .
. . . . . . . .
. . . . .
Vv08g0842 Tc05_g031510 . . . . .
. . . . . . . .
. . . . . .
Vv08g0843 . . . . . .

Vv06g0488 Tc09_g032210 . . . Gorai.
009G101500.1 . . . . . . .
.
Vv08g0844 . . . . . . .
. . . . . . . .
. . . . .
Vv08g0845 . . . . . . .
. . . . . . . .
. . . . .
Vv08g0846 . . . . . . .
. . . . . . . .
. . . . .
Vv08g0847 . . . . . . .
. . . . . . . .
. . . . .
Vv08g0848 . . . . . . .
. . . . . . . .
. . . . .
Vv08g0849 . . . . . . .
. . . . . . . .
. . . . .
Vv08g0850 . . . . . . .
. . . . . . . .
. . . . .
Vv08g0851 . . . . . .
Vv06g0444 Tc09_g032740 . . . Gorai.
009G103300.1 Gorai.003G138600.1 . . . .
. . .
Vv08g0852 . . . . . . .
. . . . . . . .
. . . . .
Vv08g0853 Tc05_g008930 . . . . .
. . . . . . . .
. . . . . .
Vv08g0854 . . . . . . .
. . . . . . . .
. . . . .
Vv08g0855 Tc05_g031590 . . . . .
. . . . . . . .
. . . . . .
Vv08g0856 . . . . . . .
. . . . . . . .
. . . . .
Vv08g0857 . . . . . . .
. . . . . . Vv13g0595
. . . . . .
Vv08g0858 . . . . . . .
. . . . . . . .
. . . . .
Vv08g0859 . . . . . . .

```
        .       .       .       .       .       .       Vv13g0293
        .       .       Gorai.011G246100.1      Gorai.009G316900.1
        .       .
Vv08g0860       .       .       .       .       .       .       .
        .       .       .       .       .       .       .       .
        .       .       .       .       .
Vv08g0861       .       .       .       .       .       .
Vv06g0467       Tc09_g032390    .       Gorai.001G107500.1      .
        Gorai.009G102100.1      Gorai.003G137300.1      .       .
        .       .       .       .       .
Vv08g0862       Tc05_g027430    .       .       .       .       .
        .       .       .       .       .       .       .       .
        .       .       .       .       .       .
Vv08g0863       .       .       .       .       .       .       .
        .       .       .       .       .       .       .       .
        .       .       .       .       .
Vv08g0864       .       .       .       .       Gorai.009G419700.1
        .       .       .       .       .       .       .       .
        .       .       .       .       .       .       .
Vv08g0865       .       .       .       .       .       .
Vv06g0465       Tc09_g032410    .       Gorai.001G107400.1      .
        Gorai.009G102200.1      Gorai.003G137500.1      Vv13g0287
        .       .       .       Gorai.009G316600.1      .       .
Vv08g0866       .       .       .       .       .       .       .
        .       .       .       .       .       .       Vv13g0286
        .       .       .       .       .       .
Vv08g0867       .       .       .       .       .       .       .
        .       .       .       .       .       .       .       .
        .       .       .       .       .
Vv08g0868       .       .       .       .       .       .       .
        .       .       .       .       .       .       .       .
        .       .       .       .       .
Vv08g0869       Tc05_g027130    .       .       .       .       .
        .       .       .       .       .       .       .       .
        .       .       .       .       .       .
Vv08g0870       .       .       .       .       .       .       .
        .       .       .       .       .       .       Vv13g0282
        .       .       Gorai.011G245000.1      Gorai.009G316400.1
        .       Gorai.012G067600.1
Vv08g0871       .       .       .       .       .       .       .
        .       .       .       .       .       .       Vv13g0281
        .       .       Gorai.011G244900.1      .       .
Gorai.012G067500.1
Vv08g0872       .       .       .       .       .       .       .
        .       .       .       .       .       .       .       .
        .       .       .       .       .
Vv08g0873       .       .       .       .       .       .       .
        .       .       .       .       .       .       Vv13g0228
        .       Gorai.013G002900.1      .       .       .       .
Vv08g0874       .       .       .       .       .       .
```

Vv06g0452 Tc09_g032540 . Gorai.001G106900.1
Gorai.004G003100.1 . Gorai.003G137800.1 . .
. . . . .
Vv08g0875 . . . . . . .
. . . . . . . .
. . . . .
Vv08g0876 Tc05_g031680 . . . . .
. . . . . . . .
. . . . . .
Vv08g0877 . . . . . . .
. . . . . . . .
. . . . .
Vv08g0878 . . . . . . .
. . . . . . . .
. . . . .
Vv08g0879 . . Gorai.002G252200.1 . .
. . . . . . . .
. . . . . . .
Vv08g0880 . . . . . . .
. . . . . . . .
. . . . .
Vv08g0881 . . . . . . .
. . . . . . . .
. . . . .
Vv08g0882 . . . . . . .
. . . . . . . .
. . . . .
Vv08g0883 Tc05_g031810 . . . . .
. . . . . . . .
. . . . . .
Vv08g0884 . . . . . . .
. . . . . . . .
. . . . .
Vv08g0885 . . . . . . .
. . . . . . . .
. . . . .
Vv08g0886 . . . . Gorai.012G100800.1
. . . . . . . .
Vv13g0260 Tc10_g004570 . Gorai.011G242300.1
. Gorai.010G203500.1 .
Vv08g0887 . . . . . . .
. . . . . . . .
. . . . .
Vv08g0888 Tc05_g009390 . . . . .
. . . . . . . .
. . . . . .
Vv08g0889 . . . . . . .
. . . . . . . .
. . . . .
Vv08g0890 . . . . . . .

```
        .       .       .       .       .       .       .       .
        .       .       .       .       .
Vv08g0891       .       Gorai.006G177600.1      Gorai.002G252300.1
        .       .       .       Vv06g0432       .       .       .
        .       .       .       Vv13g0294       .       .
Gorai.011G246200.1      .       .       .
Vv08g0892       .       .       .       .       .       .       .
        .       .       .       .       .       .       .       .
        .       .       .       .       .
Vv08g0893       .       .       .       .       .       .       .
        .       .       .       .       .       .       .       .
        .       .       .       .       .
Vv08g0894       Tc05_g027100    .       .       .       .       .
        .       .       .       .       .       .       .       .
        .       .       .       .       .       .
Vv08g0895       .       .       .       .       .       .       .
        .       .       .       .       .       .       .       .
        .       .       .       .       .
Vv08g0896       .       .       .       .       .       .       .
        .       .       .       .       .       .       .       .
        .       .       .       .       .
Vv08g0897       .       .       .       .       .       .       .
        .       .       .       .       .       .       .       .
        .       .       .       .       .
Vv08g0898       .       .       .       .       .       .       .
        .       .       .       .       .       .       .       .
        .       .       .       .       .
Vv08g0899       Tc05_g026640    .       .       .       .       .
        Vv06g0429       Tc09_g032930    .       .       .
Gorai.009G103700.1      .       .       .       .       .       .
        .       .
Vv08g0900       .       .       .       .       .       .       .
        .       .       .       .       .       .       .       .
        .       .       .       .       .
Vv08g0901       .       .       .       .       .       .       .
        .       .       .       .       .       .       .       .
        .       .       .       .       .
Vv08g0902       .       .       .       .       Gorai.009G420500.1
        .       .       .       .       .       .       .       .
        .       .       .       .       .       .       .
Vv08g0903       .       .       .       .       .       .
Vv06g0419       Tc09_g033000    Gorai.013G266000.1      Gorai.
001G105300.1    Gorai.004G002000.1      Gorai.009G104400.1      .
        .       .       .       .       .       .       .
Vv08g0904       .       .       .       .       .       .       .
        .       .       .       .       .       .       .       .
        .       .       .       .       .
Vv08g0905       .       .       .       .       .       .       .
        .       .       .       .       .       .       .       .
        .       .       .       .       .
```

Vv08g0906 . . . . Gorai.009G420600.1
Gorai.007G374700.1 . . . . .
. . . . . . . .
.
Vv08g0907 . . . . . . .
. . . . . . . .
. . . . .
Vv08g0908 . . . . . . .
. . . . . . . .
. . . . .
Vv08g0909 . . . . . . .
. . . . . . . .
. . . . .
Vv08g0910 . . . . Gorai.009G420700.1
. . . . . . . .
. . . . . . .
Vv08g0911 . . . . . . .
. . . . . . . .
. . . . .
Vv08g0912 . . . . Gorai.009G420900.1
. . . . . . . .
. . . . . . .
Vv08g0913 . . . . . . .
. . . . . . . .
. . . . .
Vv08g0914 . . . . . . .
. . . . . . . .
. . . . .
Vv08g0915 . . . . . . .
. . . . . . . .
. . . . .
Vv08g0916 . . . . . . .
. . . . . . . .
. . . . .
Vv08g0917 . . . . . . .
. . . . . . . .
. . . . .
Vv08g0918 Tc05_g005040 . Gorai.002G254100.1 .
. . . . . . . .
. . . . . . . .
Vv08g0919 . . . . . . .
. . . . . . . .
. . . . .
Vv08g0920 . . . . . . .
. . . . . . . .
. . . . .
Vv08g0921 Tc05_g005020 Gorai.006G175100.1 . .
. . . . . . . .
. . . . . . . .
Vv08g0922 Tc05_g005010 . Gorai.002G254200.1 .

Gorai.012G101600.1 . . . . .
. . . . . . . .
. .
Vv08g0923 Tc05_g005000 Gorai.006G175000.1 Gorai.
002G254400.1 . . . Vv06g0488
Tc09_g032210 . . . Gorai.009G101500.1 .
Vv13g0327 Tc10_g003840 Gorai.013G023200.1 .
Gorai.009G318200.1 . .
Vv08g0924 . . . . . . .
. . . . . . . .
. . . . .
Vv08g0925 Tc05_g004990 . Gorai.002G254800.1 .
. . Vv06g0496 Tc09_g032130 Gorai.
013G264800.1 . . . Gorai.003G135600.1
Vv13g0333 Tc10_g003700 Gorai.013G022900.1 . .
. Gorai.012G069600.1
Vv08g0926 Tc05_g004980 . . . Gorai.
012G101800.1 . . . . . . .
. . . . . . . .
Vv08g0927 Tc05_g004970 Gorai.006G174800.1 . .
. . . . . . . .
. . . . . . . .
Vv08g0928 Tc05_g004950 Gorai.006G174600.1 . .
Gorai.012G101900.1 . . . . .
. . . . . . . .
. .
Vv08g0929 Tc05_g004930 Gorai.006G174500.1 Gorai.
002G255000.1 . Gorai.012G102200.1 . . .
. . . . . . . .
. . . .
Vv08g0930 Tc05_g004920 . . . . .
. . . . . . . .
. . . . . .
Vv08g0931 . . . . . . .
. . . . . . . .
. . . . .
Vv08g0932 Tc05_g004910 . Gorai.002G255100.1 .
Gorai.012G102300.1 . . . . .
. . . . . . . .
. .
Vv08g0933 Tc05_g004900 . . . . .
. . . . . . . .
. . . . . .
Vv08g0934 Tc05_g004880 . Gorai.002G255200.1 .
Gorai.012G102800.1 . Vv06g0507
Tc09_g032050 Gorai.013G264400.1 . Gorai.004G004600.1
. Gorai.003G134900.1 Vv13g0347 . .
. . . .
Vv08g0935 . . . . . . .
. . . . . . . .

```
        .       .       .       .       .
Vv08g0936       .       .       .       .       .       .       .
        .       .       .       .       .       .       .       .
        .       .       .       .       .
Vv08g0937       .       .       .       .       .       .       .
        .       .       .       .       .       .       .       .
        .       .       .       .       .
Vv08g0938       Tc05_g004840    .       Gorai.002G255300.1      .
        .       .       .       .       .       .       .       .
        .       .       .       .       .       .       .       .
Vv08g0939       .       Gorai.006G174300.1      .       .       .
        .       .       .       .       .       .       .       .
        .       .       .       .       .       .       .
Vv08g0940       .       .       .       .       .       .       .
        .       .       .       .       .       .       .       .
        .       .       .       .       .
Vv08g0941       Tc05_g004820    .       Gorai.002G258600.1      .
        .       .       Vv06g0510       Tc09_g032020    .
Gorai.001G108600.1      .       .       .       .       .       .
        .       .       .       .
Vv08g0942       Tc05_g004800    Gorai.006G174100.1      .       .
        .       .       .       .       .       .       .       .
        .       .       .       .       .       .       .       .
Vv08g0943       .       .       .       .       .       .
Vv06g0513       Tc09_g031990    Gorai.013G264300.1      Gorai.
001G108800.1    .       Gorai.009G100700.1      Gorai.003G134400.1
        Vv13g0356       Tc10_g003480    .       .       .       .
        .
Vv08g0944       Tc05_g004790    Gorai.006G174000.1      Gorai.
002G258500.1    .       .       Gorai.007G375600.1      Vv06g0516
        Tc09_g031960    .       .       .       Gorai.009G100400.1
        Gorai.003G133700.1      Vv13g0357       Tc10_g003470    .
        Gorai.011G256600.1      .       Gorai.010G202100.1      .
Vv08g0945       Tc05_g004780    .       Gorai.002G258400.1      .
        Gorai.012G103100.1      .       Vv06g0517
Tc09_g031950    .       .       .       Gorai.009G100300.1      .
        Vv13g0358       Tc10_g003460    .       .       Gorai.
009G319000.1    .       .
Vv08g0946       Tc05_g004770    Gorai.006G173800.1      .       .
        Gorai.012G103200.1      .       .       .       .       .
        .       .       .       .       .       .       .       .
        .       .
Vv08g0947       Tc05_g004750    .       .       .       Gorai.
012G103300.1    Gorai.007G375700.1      .       .       .       .
        .       .       .       .       .       .       .       .
        .       .
Vv08g0948       Tc05_g004740    .       .       .       .
Gorai.007G375800.1      .       .       .       .       .       .
        .       .       .       .       .       .       .       .
Vv08g0949       Tc05_g004730    Gorai.006G173700.1      .       .
```

Gorai.012G103400.1 . Vv06g0524
Tc09_g031890 . Gorai.001G109300.1 Gorai.004G004800.1
Gorai.009G100100.1 Gorai.003G133200.1 Vv13g0381
. Gorai.013G022500.1 . Gorai.009G319300.1
. .
Vv08g0950 . . . . . . .
. . . . . . . .
. . . . .
Vv08g0951 Tc05_g004690 Gorai.006G173500.1 . .
. . . . . . . .
. . . . . . . .
Vv08g0952 Tc05_g004680 . . . . .
Vv06g0530 Tc09_g031830 . Gorai.001G110200.1
. Gorai.009G099700.1 . . . .
. . . .
Vv08g0953 Tc05_g004670 Gorai.006G173400.1 . .
. . . . . . . .
. . . . . . . .
Vv08g0954 Tc05_g004650 Gorai.006G173300.1 Gorai.
002G258100.1 . . . . . . .
. . . . . . . .
. .
Vv08g0955 . . . . . . .
. . . . . . . .
. . . . .
Vv08g0956 Tc05_g004620 Gorai.006G173000.1 . .
. . . . . . . .
. . . . . . . .
Vv08g0957 Tc05_g004610 . Gorai.002G257800.1 .
Gorai.012G103700.1 . . . . .
. . . . . . . .
. .
Vv08g0958 Tc05_g004600 Gorai.006G172900.1 . .
Gorai.009G421600.1 . Vv06g0534
Tc09_g031780 . . . Gorai.009G099400.1
Gorai.003G132200.1 . . . . . .
.
Vv08g0959 Tc05_g004590 . . . .
Gorai.007G375900.1 Vv06g0536 Tc09_g031760 . .
. Gorai.009G099300.1 Gorai.003G132000.1 .
. . . . . .
Vv08g0960 Tc05_g004580 . Gorai.002G257700.1 .
. Gorai.007G376000.1 Vv06g0538 . .
. . . . . . . .
. . .
Vv08g0961 Tc05_g004570 . . . . .
. . . . . . . .
. . . . . .
Vv08g0962 . . . . . .
Vv06g0552 Tc09_g031700 Gorai.013G263900.1 . .

.	Gorai.003G131900.1	.	.	.	.
.	.	.
Vv08g0963	Tc05_g004560	Gorai.006G172800.1	.	.
.	.	.	.	.	.	.	.
.	.	.	.	.	.	.	.
Vv08g0964	Tc05_g004550	Gorai.006G172700.1	Gorai.
002G257600.1	.	.	.	.	.	.	.
.	.	.	.	.	.	.	.
.	.
Vv08g0965	Tc05_g004520	.	.	.	Gorai.
012G104100.1	.	.	.	.	.	.	.
.	.	.	.	.	.	.	.
Vv08g0966	Tc05_g004510	Gorai.006G172600.1	Gorai.
002G257500.1	.	Gorai.012G104300.1	.	.	.
.	.	.	.	.	.	.	.
.	.	.	.
Vv08g0967	Tc05_g004480	Gorai.006G172400.1	Gorai.
002G257400.1	.	Gorai.012G104400.1	Gorai.007G376300.1
Vv06g0567	Tc09_g031580	.	Gorai.001G111100.1
.	Gorai.009G098800.1	Gorai.003G131000.1
Vv13g0395	.	Gorai.013G022200.1	Gorai.011G266000.1
.	.	.
Vv08g0968	.	.	.	.	.	.	.
.	.	.	.	.	.	.	.
.	.	.	.	.
Vv08g0969	Tc05_g004470	Gorai.006G172300.1	.	.
.	.	.	.	.	.	.	.
.	.	.	.	.	.	.	.
Vv08g0970	Tc05_g004460	Gorai.006G172200.1	.	.
.	.	Vv06g0570	Tc09_g031570	.	.
.	Gorai.009G098700.1	Gorai.003G130900.1	.
.	.	.	.	.	.
Vv08g0971	Tc05_g004450	Gorai.006G172100.1	.	.
.	.	Vv06g0574	Tc09_g031530	.	.
.	.	.	.	.	.	.	.
.	.
Vv08g0972	.	.	.	.	.	.	.
.	.	.	.	.	.	.	.
.	.	.	.	.
Vv08g0973	Tc05_g004440	.	.	.	.	.
Vv06g0576	Tc09_g031520	.	.	.
Gorai.009G098300.1	Gorai.003G130700.1	Vv13g0396	.
Gorai.013G021300.1	Gorai.011G266100.1	.	.
.
Vv08g0974	.	Gorai.006G172000.1	.	.	.
.	.	.	.	.	.	.	.
.	.	.	.	.	.	.
Vv08g0975	Tc05_g004430	.	.	.	.
Gorai.007G376400.1	Vv06g0579	Tc09_g031490	.
Gorai.001G111600.1	.	.	.	Vv13g0402	.

```
                .        .        .        .        .
Vv08g0976                .        .        .        .        .        .        .
                .        .        .        .        .        .        .        .
                .        .        .        .        .
Vv08g0977                Tc05_g004410      Gorai.006G171900.1        .        .
                .        .        .        .        .        .        .        .
                .        .        .        .        .        .        .        .
Vv08g0978                Tc05_g004400      .        Gorai.002G257300.1        .
        Gorai.012G104700.1        Gorai.007G376500.1        Vv06g0583
        Tc09_g031440      .        .        Gorai.004G005300.1        .
        Gorai.003G130000.1        .        .        .        .        .
                .        .
Vv08g0979                Tc05_g004390      Gorai.006G171800.1        Gorai.
002G257200.1             .        .        .        .        .        .        .
                .        .        .        .        .        .        .        .
                .        .
Vv08g0980                Tc05_g004380      Gorai.006G171700.1        .        .
        Gorai.012G104800.1        .        .        .        .        .
                .        .        .        .        .        .        .        .
                .        .
Vv08g0981                Tc05_g004360      .        Gorai.002G257100.1        .
                .        Gorai.007G376600.1        .        .        .        .
                .        .        .        .        .        .        .        .
                .        .
Vv08g0982                .        .        .        .        .        .        .
                .        .        .        .        .        .        .        .
                .        .        .        .        .
Vv08g0983                Tc05_g004340      .        Gorai.002G257000.1        .
                .        Gorai.007G376700.1        .        .        .        .
                .        .        .        .        .        .        .        .
                .        .
Vv08g0984                .        .        .        .        .        .        .
                .        .        .        .        .        .        .        .
                .        .        .        .        .
Vv08g0985                Tc05_g004330      .        .        .        .        .
                .        .        .        .        .        .        .        .
                .        .        .        .        .        .
Vv08g0986                Tc05_g004320      Gorai.006G171500.1        .        .
                .        Gorai.007G376800.1        .        .        .        .
                .        .        .        .        .        .        .        .
                .        .
Vv08g0987                Tc05_g004310      .        .        .        .        .
                .        .        .        .        .        .        .        .
                .        .        .        .        .        .
Vv08g0988                .        .        .        .        .        .        .
                .        .        .        .        .        .        .        .
                .        .        .        .        .
Vv08g0989                Tc05_g004300      Gorai.006G171400.1        .        .
                .        .        .        .        .        .        .        .
                .        Vv13g0413         Tc10_g002790      .        Gorai.
```

011G266900.1 . Gorai.010G201200.1 .
Vv08g0990 Tc05_g004280 Gorai.006G171200.1 . .
. . . . . . . .
. . . . . . . .
Vv08g0991 Tc05_g004270 Gorai.006G171100.1 . .
Gorai.012G105100.1 . . . . .
. . . . . . . .
. .
Vv08g0992 . . . . . . .
. . . . . . . .
. . . . .
Vv08g0993 Tc05_g004260 . . . . .
. . . . . . . .
. . . . . .
Vv08g0994 . . . . Gorai.012G105200.1
. . . . . . . .
. . . . . . .
Vv08g0995 Tc05_g004250 . . . Gorai.
012G105300.1 . . . . . . .
. . . . . . . .
Vv08g0996 . . . . . . .
. . . . . . . .
. . . . .
Vv08g0997 Tc05_g004240 . . . Gorai.
012G105400.1 . . . . . . .
. Vv13g0419 . . . . .
.
Vv08g0998 Tc05_g004230 . . . . .
. . . . . . . .
. . . . . .
Vv08g0999 Tc05_g004220 . . . . .
. . . . . . . .
. . . . . .
Vv08g1000 . . . . . . .
. . . . . . . .
. . . . .
Vv08g1001 Tc05_g004200 . . . Gorai.
012G105600.1 . . . . . . .
. . . . . . . .
Vv08g1002 Tc05_g004140 . . . . .
. . . . . . . .
. . . . . .
Vv08g1003 Tc05_g004100 Gorai.006G171000.1 Gorai.
002G256700.1 . . . . . . .
. . . . . . . .
. .
Vv08g1004 Tc05_g004060 . . . Gorai.
012G106000.1 . Vv06g0603 . . . .
. . Vv13g0423 . . Gorai.
011G267600.1 . Gorai.010G200600.1 .

Vv08g1005 Tc05_g004050 . . . . .
. . . . . . . .
. . . . . .
Vv08g1006 Tc05_g004030 . Gorai.002G256600.1 .
Gorai.012G106200.1 . . . . .
. . . . . . . .
. .
Vv08g1007 . . . . . . .
. . . . . . . .
. . . . .
Vv08g1008 Tc05_g004020 . . . . .
Vv06g0610 . . . . . .
Vv13g0431 . . Gorai.011G268000.1 .
. .
Vv08g1009 Tc05_g004010 . . . Gorai.
012G106300.1 Gorai.007G377000.1 . . . .
. . . . . . . .
. .
Vv08g1010 . Gorai.006G170900.1 . . .
. . . . . . . .
. . . . . . .
Vv08g1011 . . . . . . .
. . . . . . . .
. . . . .
Vv08g1012 . . . . . . .
. . . . . . . .
. . . . .
Vv08g1013 Tc05_g003990 . . . . .
Vv06g0615 Tc09_g031150 . . . .
. . . . . . . .
Vv08g1014 . . . . . .
Vv06g0621 . Gorai.013G262300.1 . . .
. . . . . . . .
Vv08g1015 Tc05_g003980 . . . . .
. . . . . . . .
. . . . . .
Vv08g1016 Tc05_g003970 Gorai.006G170800.1 . .
. . . . . . . .
. . . . . . . .
Vv08g1017 . . Gorai.002G256400.1 . .
. . . . . . . .
. . . . . . .
Vv08g1018 Tc05_g003960 . Gorai.002G256300.1 .
Gorai.012G106400.1 . . . . .
. . . . . . . .
. .
Vv08g1019 Tc05_g003950 . . . . .
. . . . . . . .
. . . . . .
Vv08g1020 Tc05_g003930 . . . . .

. . . . . . . .
. . . . . .
Vv08g1021 Tc05_g003910 Gorai.006G170500.1 . .
Gorai.012G107000.1 . Vv13g0454
Tc10_g002620 . Gorai.011G269200.1 . . .
. . . . . . .
Vv08g1022 Tc05_g003900 . . . .
Gorai.007G377100.1 . . . . . .
. . . . . . . .
Vv08g1023 Tc05_g003890 Gorai.006G170400.1 . .
Gorai.012G107200.1 . . . . .
. . . . . . . .
. .
Vv08g1024 Tc05_g003880 . . . Gorai.
012G107300.1 . . . . . . .
. . . . . . . .
Vv08g1025 Tc05_g003870 Gorai.006G170300.1 . .
Gorai.012G107500.1 . . . . .
. . . . . . . .
. .
Vv08g1026 Tc05_g003860 . . . Gorai.
012G107600.1 . . . . . . .
. . . . . . . .
Vv08g1027 . . Gorai.002G256200.1 . .
Gorai.007G377200.1 . . . . .
. . . . . . . .
.
Vv08g1028 Tc05_g003840 . . . Gorai.
012G107700.1 . Vv13g0456 Tc10_g002590 .
Gorai.011G269700.1 . . . . . .
. . . .
Vv08g1029 Tc05_g003820 . . . Gorai.
012G107800.1 . . . . . . .
. . . . . . . .
Vv08g1030 Tc05_g003800 Gorai.006G170200.1 . .
. . . . . . . .
. . . . . . . .
Vv08g1031 . . . . . . .
. . . . . . . .
. . . . .
Vv08g1032 Tc05_g003760 . . . Gorai.
012G108100.1 . . . . . . .
. . . . . . . .
Vv08g1033 . . . . . . .
. . . . . . . .
. . . . .
Vv08g1034 Tc05_g003750 . . . . .
. . . . . . . .
. . . . . .
Vv08g1035 . . . . . . .

. . . . . . . .
. . . . .
Vv08g1036 Tc05_g003740 Gorai.006G170000.1 . .
. . . . . . . .
. . . . . . . .
Vv08g1037 Tc05_g003730 . . . . .
. . . . . . . .
. . . . . .
Vv08g1038 . . . . . . .
. . . . . . . .
. . . . .
Vv08g1039 . . . . . . .
. . . . . . . .
. . . . .
Vv08g1040 . . . . . . .
. . . . . . . .
. . . . .
Vv08g1041 Tc05_g003720 Gorai.006G169900.1 . .
. . . . . . . .
. . . . . . . .
Vv08g1042 . . . . . . .
. . . . . . . .
. . . . .
Vv08g1043 Tc05_g003710 . Gorai.002G256100.1 .
. . . . . . . .
. . . . . . . .
Vv08g1044 Tc05_g003690 Gorai.006G169800.1 . .
. . . . . . . .
. . . . . . . .
Vv08g1045 Tc05_g003680 . Gorai.002G255900.1 .
. . . . . . . .
. . . . . . . .
Vv08g1046 Tc05_g003670 Gorai.006G169600.1 . .
. . . . . . . .
. . . . . . . .
Vv08g1047 Tc05_g003660 . Gorai.002G255800.1 .
Gorai.012G108300.1 . Vv13g0465
Tc10_g002390 . Gorai.011G271200.1 . . .
. . . . . . .
Vv08g1048 Tc05_g003650 Gorai.006G169500.1 Gorai.
002G255700.1 . . . . . . .
. . . . . . . .
. .
Vv08g1049 Tc05_g003640 Gorai.006G169400.1 . .
. . Vv13g0466 Tc10_g002380 . .
. . . . . . . .
. .
Vv08g1050 . . . . . . .
. . . . . . . .
. . . . .

Vv08g1051 Tc05_g003630 Gorai.006G169300.1 . .
. . . . . . . .
. . . . . . . .
Vv08g1052 Tc05_g003610 . Gorai.002G255500.1 .
. . . . . . . .
. . . . . . . .
Vv08g1053 Tc05_g003600 . . . Gorai.
012G108500.1 . Vv13g0491 . . . .
. . . . . . . .
.
Vv08g1054 Tc05_g003590 Gorai.006G178100.1 . .
Gorai.012G108600.1 Gorai.007G377300.1 Vv13g0492
. . . . . Gorai.012G073500.1
. . . . . . .
Vv08g1055 Tc05_g003580 Gorai.006G178200.1 . .
. Gorai.007G377400.1 Vv13g0493 . .
Gorai.011G274100.1 . . Gorai.012G073600.1
. . . . . . .
Vv08g1056 Tc05_g003570 . Gorai.002G252500.1 .
Gorai.012G108700.1 . . . . .
. . . . . . . .
. .
Vv08g1057 . Gorai.006G178300.1 Gorai.002G258900.1
. . . Vv13g0495 . .
Gorai.011G274300.1 . Gorai.010G199600.1 . .
. . . . .
Vv08g1058 Tc05_g003540 Gorai.006G178400.1 . .
. . . . . . . .
. . . . . . . .
Vv08g1059 . . . . . . .
. . . . . . . .
. . . . .
Vv08g1060 Tc05_g003530 Gorai.006G178600.1 . .
Gorai.012G108800.1 . . . . .
. . . . . . . .
. .
Vv08g1061 Tc05_g003510 Gorai.006G178700.1 . .
. . . . . . . .
. . . . . . . .
Vv08g1062 Tc05_g003500 . . . . .
Vv06g0671 Tc09_g030850 . . . .
Gorai.003G127400.1 . . . . .
. .
Vv08g1063 . . . . Gorai.012G108900.1
. . . . . . . .
. . . . . . .
Vv08g1064 Tc05_g003490 Gorai.006G178800.1 . .
Gorai.012G109000.1 Gorai.007G377500.1 . .
. . . . . Vv13g0497 .
. Gorai.011G274600.1 . . Gorai.

012G073700.1
Vv08g1065 Tc05_g003480 Gorai.006G178900.1 . .
. . . . . . . .
. . . . . . . .
Vv08g1066 Tc05_g003460 . Gorai.002G259000.1 .
. . Vv06g0675 Tc09_g030810 . .
. . Gorai.003G126900.1 . . .
. . . .
Vv08g1067 . . . . . . .
. . . . . . . .
. . . . .
Vv08g1068 Tc05_g003360 Gorai.006G179200.1 . .
. . Vv06g0679 Tc09_g030660 . .
. Gorai.009G094900.1 Gorai.003G126700.1 .
. . . . . .
Vv08g1069 Tc05_g003350 Gorai.006G179300.1 . .
. . . . . . . .
. . . . . . . .
Vv08g1070 Tc05_g003330 Gorai.006G179400.1 Gorai.
002G259100.1 . Gorai.012G109300.1 Gorai.007G377600.1
Vv06g0680 Tc09_g030650 . . . .
. . . . . . . .
Vv08g1071 Tc05_g003320 Gorai.006G179500.1 Gorai.
002G259200.1 . . . . . . .
. . . . . . . .
. .
Vv08g1072 Tc05_g003310 Gorai.006G179600.1 . .
Gorai.012G109400.1 . Vv06g0684 . .
. . . . . . . .
. . .
Vv08g1073 Tc05_g003300 Gorai.006G179700.1 . .
. . . . . . . .
. . . . . . . .
Vv08g1074 . . . . . . .
. . . . . . . .
. . . . .
Vv08g1075 Tc05_g003290 . . . Gorai.
012G109700.1 . . . . . . .
. . . . . . . .
Vv08g1076 Tc05_g003270 . Gorai.002G259300.1
Gorai.002G148300.1 . Gorai.007G377700.1 Vv06g0685
. . . . . . . .
. . . . .
Vv08g1077 Tc05_g003260 . . . . .
. . . . . . . .
. . . . . .
Vv08g1078 . . . . . . .
. . . . . . . .
. . . . .
Vv08g1079 . . . . . . .

```
        .       .       .       .       .       .       .       .
        .       .       .       .       .
Vv08g1080       .       .       .       .       .       .       .
        .       .       .       .       .       .       .       .
        .       .       .       .       .
Vv08g1081       .       .       .       .       .       .       .
        .       .       .       .       .       .       .       .
        .       .       .       .       .
Vv08g1082       Tc05_g003250    .       .       .       Gorai.
012G110100.1    Gorai.007G377800.1      .       .       .       .
        .       .       .       .       .       .       .       .
        .       .
Vv08g1083       Tc05_g003240    .       Gorai.002G259700.1      .
        Gorai.012G110200.1      .       .       .       .       .
        .       .       .       .       .       .       .       .
        .       .
Vv08g1084       Tc05_g003230    .       .       .       Gorai.
012G110300.1    .       .       .       .       .       .       .
        .       .       .       .       .       .       .       .
Vv08g1085       Tc05_g003220    Gorai.006G179800.1      .       .
        Gorai.012G110400.1      .       .       .       .       .
        .       .       .       .       .       .       .       .
        .       .
Vv08g1086       Tc05_g003210    Gorai.006G179900.1      .       .
        .       .       Vv06g0687       Tc09_g030600    Gorai.
013G261300.1    .       .       Gorai.009G094600.1      Gorai.
003G126300.1    .       .       .       .       .       .       .
Vv08g1087       Tc05_g003200    .       .       .       Gorai.
012G110600.1    .       .       .       .       .       .       .
        .       .       .       .       .       .       .       .
Vv08g1088       Tc05_g003180    Gorai.006G180000.1      .       .
        .       .       .       .       .       .       .       .
        .       .       .       .       .       .       .       .
Vv08g1089       .       .       .       .       .       .       .
        .       .       .       .       .       .       .       .
        .       .       .       .       .
Vv08g1090       Tc05_g003170    Gorai.006G180100.1      .       .
        Gorai.012G110800.1      Gorai.007G377900.1      .       .
        .       .       .       .       .       .       .       .
        .       .       .       .
Vv08g1091       Tc05_g003160    .       .       .       Gorai.
012G110900.1    .       .       .       .       .       .       .
        .       .       .       .       .       .       .       .
Vv08g1092       .       .       .       .       .       .       .
        .       .       .       .       .       .       .       .
        .       .       .       .       .
Vv08g1093       Tc05_g003130    .       Gorai.002G260400.1      .
        .       .       Vv06g0692       Tc09_g030530    .       .
        .       Gorai.009G094400.1      Gorai.003G125400.1      .
        .       .       .       .       .       .
```

Vv08g1094 Tc05_g003120 . Gorai.002G260500.1 .
. . Vv06g0694 Tc09_g030510 . .
Gorai.004G006600.1 . . . . .
. . . .
Vv08g1095 Tc05_g003110 . . . Gorai.
012G111000.1 . . . . . . .
. . . . . . . .
Vv08g1096 Tc05_g003100 Gorai.006G180200.1 . .
Gorai.012G111100.1 . . . . .
. . . . . . . .
. .
Vv08g1097 Tc05_g003080 . . . Gorai.
012G111200.1 . . . . . . .
. . . . . . . .
Vv08g1098 Tc05_g003070 . Gorai.002G260600.1 .
Gorai.012G111300.1 . . . . .
. . . . . . . .
. .
Vv08g1099 Tc05_g003060 . Gorai.002G260800.1 .
. . . . . . . .
. . . . . . . .
Vv08g1100 Tc05_g003050 . . Gorai.002G148400.1
Gorai.012G111400.1 . . . . .
. . . . . . . .
. .
Vv08g1101 Tc05_g003040 . . . Gorai.
012G111500.1 . . . . . . .
. . . . . . . .
Vv08g1102 Tc05_g003020 . Gorai.002G260900.1 .
. Gorai.007G378000.1 . . . .
. . . . . . . .
. .
Vv08g1103 . . . . . . .
. . . . . . . .
. . . . .
Vv08g1104 . . . . Gorai.012G111600.1
. . . . . . . .
. . . . . . .
Vv08g1105 Tc05_g003010 Gorai.006G180300.1 . .
Gorai.012G111700.1 . . . . .
. . . . . . . .
. .
Vv08g1106 Tc05_g003000 Gorai.006G180400.1 . .
. . . . . . . .
. . . . . . . .
Vv08g1107 Tc05_g002990 Gorai.006G180500.1 . .
. . Vv06g0709 Tc09_g030410 . .
. . Gorai.003G124900.1 . . .
. . . .
Vv08g1108 Tc05_g002970 Gorai.006G180600.1 . .

. . . . . . . .
. . . . . . . .
Vv08g1109 . . . . . . .
. . . . . . . .
. . . . .
Vv08g1110 Tc05_g002950 . Gorai.002G261000.1 .
Gorai.012G111800.1 Gorai.007G378100.1 Vv06g0711
Tc09_g030390 . Gorai.001G114900.1 . .
. . . . . . . .
Vv08g1111 Tc05_g002940 . . . . .
. . . . . . . .
. . . . . .
Vv08g1112 Tc05_g002930 . Gorai.002G261300.1 .
. . . . . . . .
. . . . . . . .
Vv08g1113 Tc05_g002920 Gorai.006G180700.1 . .
. . . . . . . .
. . . . . . . .
Vv08g1114 Tc05_g002910 . Gorai.002G261400.1 .
. . Vv06g0714 Tc09_g030360 . .
. Gorai.009G093900.1 . . . .
. . . .
Vv08g1115 Tc05_g002900 . Gorai.002G261500.1 .
Gorai.012G111900.1 Gorai.007G378200.1 . .
. . . . . . . .
. . . .
Vv08g1116 Tc05_g002890 Gorai.006G180900.1 Gorai.
002G261600.1 . Gorai.012G112000.1 Gorai.007G378300.1
Vv06g0716 Tc09_g030340 . . .
Gorai.009G093700.1 . . . . . .
. .
Vv08g1117 Tc05_g002870 . . . Gorai.
012G112100.1 . . . . . .
. . . . . . . .
Vv08g1118 Tc05_g002860 . Gorai.002G261700.1 .
Gorai.012G112200.1 . Vv06g0731 . .
. . . . . . . .
. . .
Vv08g1119 . . . . . . .
. . . . . . . .
. . . . .
Vv08g1120 Tc05_g002850 Gorai.006G181000.1 Gorai.
002G261800.1 . Gorai.012G112300.1 . . .
. . . . . . . .
. . . .
Vv08g1121 Tc05_g002840 Gorai.006G181100.1 . .
. . . . . . . .
. . . . . . . .
Vv08g1122 Tc05_g002830 . Gorai.002G262000.1 .
Gorai.012G112400.1 . . . . .

. . . . . . . .
. .
Vv08g1123 . . . . . . .
. . . . . . . .
. . . . .
Vv08g1124 Tc05_g002820 Gorai.006G181200.1 .
Gorai.002G148500.1 Gorai.012G112800.1 . . .
. . . . . . . .
. . . .
Vv08g1125 Tc05_g002780 Gorai.006G181400.1 Gorai.
002G262100.1 . Gorai.012G112900.1 . . .
. . . . . Vv13g0022
Tc10_g001900 Gorai.013G018900.1 Gorai.011G283900.1 .
. Gorai.012G076100.1
Vv08g1126 . . . . . . .
. . . . . . . .
. . . . .
Vv08g1127 . . . . . . .
. . . . . . . .
. . . . .
Vv08g1128 . . . . . . .
. . . . . . . .
. . . . .
Vv08g1129 Tc05_g002770 . . . . .
. . . . . . . .
. . . . . .
Vv08g1130 Tc05_g002760 Gorai.006G181600.1 . .
Gorai.012G113000.1 . . . . .
. . . . . . . .
. .
Vv08g1131 . . . . . . .
. . . . . . . .
. . . . .
Vv08g1132 . . . . . . .
. . . . . . . .
. . . . .
Vv08g1133 Tc05_g002730 . . . . .
. . . . . . . .
. . . . . .
Vv08g1134 Tc05_g002720 . . . . .
. . . . . . . .
. . . . . .
Vv08g1135 Tc05_g002690 Gorai.006G181700.1 . .
. . . . . . . .
. . . . . . . .
Vv08g1136 Tc05_g002670 . Gorai.002G264300.1 .
. . . . . . . .
. . . . . . . .
Vv08g1137 Tc05_g002660 . . Gorai.002G148600.1
. . . . . . . .

```
        .       Vv13g0031       .       .       .       .       .
        Gorai.012G076300.1
Vv08g1138       Tc05_g002650    .       .       .       .       .
        .       .       .       .       .       .       .       .
        .       .       .       .       .       .
Vv08g1139       Tc05_g002630    .       .       .       Gorai.
012G113200.1    .       .       .       .       .       .       .
        .       Vv13g0032       .       .       Gorai.011G285700.1
        .       .       Gorai.012G076400.1
Vv08g1140       Tc05_g002620    Gorai.006G181800.1      .       .
        .       Gorai.007G378800.1      .       .       .       .
        .       .       .       Vv13g0034       .       Gorai.
013G018700.1    .       .       .       Gorai.012G076700.1
Vv08g1141       Tc05_g002610    .       .       .       .       .
        .       .       .       .       .       .       .       .
        .       .       .       .       .       .
Vv08g1142       Tc05_g002600    .       .       .       .       .
        .       .       .       .       .       .       .       .
        .       .       .       .       .       .
Vv08g1143       .       .       .       .       .       .       .
        .       .       .       .       .       .       .       .
        .       .       .       .       .
Vv08g1144       Tc05_g002580    .       .       .       Gorai.
012G113300.1    .       .       .       .       .       .       .
        .       .       .       .       .       .       .       .
Vv08g1145       Tc05_g002560    .       .       .       Gorai.
012G113400.1    .       .       .       .       .       .       .
        .       .       .       .       .       .       .       .
Vv08g1146       Tc05_g002550    .       .       .       Gorai.
012G113500.1    .       .       .       .       .       .       .
        .       .       .       .       .       .       .       .
Vv08g1147       .       .       .       .       .       .       .
        .       .       .       .       .       .       .       .
        .       .       .       .       .
Vv08g1148       Tc05_g002540    Gorai.006G182300.1      .       .
        Gorai.012G113600.1      .       Vv06g0768
Tc09_g029960    .       .       Gorai.004G007100.1      Gorai.
009G092200.1    .       .       .       .       .       .       .
        .
Vv08g1149       Tc05_g002530    Gorai.006G182400.1      .       .
        .       .       .       .       .       .       .       .
        .       .       .       .       .       .       .       .
Vv08g1150       Tc05_g002500    .       .       .       Gorai.
012G114000.1    .       .       .       .       .       .       .
        .       Vv13g0047       .       Gorai.013G018300.1      .
        .       .       .
Vv08g1151       .       Gorai.006G182500.1      .       .       .
        .       .       .       .       .       .       .       .
        .       .       .       .       .       .       .
Vv08g1152       .       .       .       .       .       .
```

Vv06g0779 Tc09_g029880 . . . Gorai.
009G091700.1 Gorai.006G106400.1 Vv13g0058
Tc10_g001510 . . . Gorai.010G198000.1
Gorai.012G076900.1
Vv08g1153 . . . . . . .
. . . . . . . .
. . . . .
Vv08g1154 . . . . . . .
. . . . . . . .
. . . . .
Vv08g1155 . Gorai.006G149600.1 . . .
. . . . . . . .
. . . . . . .
Vv08g1156 . . . . . . .
. . . . . . . .
. . . . .
Vv08g1157 . . . . . . .
. . . . . . . .
. . . . .
Vv08g1158 . Gorai.006G149900.1 . . .
. . . . . . . .
. . . . . . .
Vv08g1159 Tc05_g011670 Gorai.006G150100.1 Gorai.
002G235800.1 . Gorai.009G397400.1 Gorai.007G365600.1
. . . . . . . .
. . . . . .
Vv08g1160 Tc05_g011630 Gorai.006G150200.1 Gorai.
002G235900.1 . . . . . . .
. . . . . . . .
. .
Vv08g1161 . . . . . . .
. . . . . . . .
. . . . .
Vv08g1162 Tc05_g011570 Gorai.006G150400.1 . .
. . Vv06g0784 Tc09_g029790 . .
. Gorai.009G091300.1 . Vv13g0065
Tc10_g001410 . Gorai.011G289100.1 Gorai.009G322600.1
. .
Vv08g1163 Tc05_g011520 Gorai.006G150500.1 . .
. . . . . . . .
. . . . . . . .
Vv08g1164 . . . . . . .
. . . . . . . .
. . . . .
Vv08g1165 Tc05_g011500 . . . . .
. . . . . . . .
. . . . . .
Vv08g1166 . Gorai.006G150600.1 Gorai.002G236000.1
. . . Vv0Unrg8945 . . .
. . . . . . . .

. .
Vv08g1167 Tc05_g011460 Gorai.006G150700.1 Gorai.
002G236100.1 . Gorai.009G397700.1 . . .
. . . . . . . .
. . . .
Vv08g1168 Tc05_g011450 . . . Gorai.
009G397800.1 . . . . . . .
. . . . . . . .
Vv08g1169 . . . . . . .
. . . . . . . .
. . . . .
Vv08g1170 . . . Gorai.002G157200.1 .
Gorai.007G366600.1 Vv0Unrg8948 . . .
. . . . . . . .
. .
Vv08g1171 . Gorai.006G150800.1 Gorai.002G236300.1
. Gorai.009G397900.1 Gorai.007G366700.1 .
. . . . . . . .
. . . . .
Vv08g1172 . . . . . . .
. . . . . . . .
. . . . .
Vv08g1173 . . . . Gorai.009G398000.1
. . . . . . . .
. . . . . . .
Vv08g1174 . Gorai.006G150900.1 Gorai.002G236400.1
Gorai.002G157300.1 Gorai.009G398100.1 .
Vv0Unrg8950 . . . . . . .
. . . . . .
Vv08g1175 . Gorai.006G151000.1 Gorai.002G236600.1
. . Gorai.007G367100.1 Vv0Unrg8952 .
. . . . . . . .
. . . .
Vv08g1176 . . . . . . .
. . . . . . . .
. . . . .
Vv08g1177 . Gorai.006G151100.1 Gorai.002G236700.1
Gorai.002G157600.1 Gorai.009G398500.1 Gorai.
007G367300.1 . . . . . . .
. . . . . . .
Vv08g1178 . Gorai.006G151400.1 . . .
. . . . . . . .
. . . . . . .
Vv08g1179 . . . . . . .
. . . . . . . .
. . . . .
Vv08g1180 . Gorai.006G151500.1 Gorai.002G267100.1
. . . . . . . .
. . . . . . . .
.

Vv08g1181 . . Gorai.002G236800.1 . .
Gorai.007G367500.1 . . . . .
. . . . . . . .
.
Vv08g1182 . . . . . . .
. . . . . . . .
. . . . .
Vv08g1183 . Gorai.006G151600.1 Gorai.002G236900.1
. . . . . . . .
. . . . . . . .
.
Vv08g1184 . . . . . . .
. . . . . . . .
. . . . .
Vv08g1185 . . Gorai.002G237000.1 . .
. . . . . . . .
. . . . . . .
Vv08g1186 . . . . . . .
. . . . . . . .
. . . . .
Vv08g1187 . Gorai.006G151900.1 Gorai.002G237200.1
. . . . . . . .
. . . . . . . .
.
Vv08g1188 . Gorai.006G152000.1 Gorai.002G237300.1
. . . Vv0Unrg8961 . . .
. . . . . . . .
. .
Vv08g1189 . Gorai.006G152100.1 Gorai.002G237400.1
. . . . . . . .
. . . . . . . .
.
Vv08g1190 Tc05_g010650 Gorai.006G152200.1 Gorai.
002G237500.1 . . . . . . .
. . . . . . . .
. .
Vv08g1191 Tc05_g010580 Gorai.006G152300.1 Gorai.
002G237600.1 . . . . . . .
. . . . . . . .
. .
Vv08g1192 . . . . Gorai.009G398600.1
. . . . . . . .
. . . . . . .
Vv08g1193 Tc05_g010520 . . . . .
. . . . . . . .
. . . . . .
Vv08g1194 . . . . . . .
. . . . . . . .
. . . . .
Vv08g1195 Tc05_g010500 . Gorai.002G237700.1 .

. . . . . . . .
. . . . . . . .
Vv08g1196 Tc05_g010490 . . . Gorai.
009G398800.1 . Vv0Unrg8971 . . . .
. . . . . . . .
.
Vv08g1197 Tc05_g010480 . Gorai.002G237800.1 .
. . . . . . . .
. . . . . . . .
Vv08g1198 Tc05_g010460 Gorai.006G152400.1 Gorai.
002G237900.1 . Gorai.009G399000.1 . . .
. . . . . . . .
. . . .
Vv08g1199 . . . . . . .
. . . . . . . .
. . . . .
Vv08g1200 Tc05_g010390 Gorai.006G152600.1 Gorai.
002G238000.1 . . . Vv06g0798
Tc09_g029660 . Gorai.001G116900.1 . .
Gorai.006G105300.1 Vv13g0077 Tc10_g001320 . .
. . Gorai.012G077500.1
Vv08g1201 Tc05_g010380 . . . . .
. . . . . . . .
. . . . . .
Vv08g1202 . . . . Gorai.009G399100.1
. . . . . . . .
. . . . . . .
Vv08g1203 Tc05_g010360 . . . Gorai.
009G399200.1 . Vv0Unrg8973 . . . .
. . . . . . . .
.
Vv08g1204 . Gorai.006G152700.1 . . .
. . . . . . . .
. . . . . . .
Vv08g1205 . Gorai.006G152800.1 . .
Gorai.009G399300.1 Gorai.007G367700.1 Vv0Unrg8979 .
. . . . . . . .
. . . .
Vv08g1206 . . . Gorai.002G157700.1
Gorai.009G399600.1 Gorai.007G367800.1 Vv0Unrg8981 .
. . . . . . . .
. . . .
Vv08g1207 . . . . Gorai.009G399700.1
. Vv0Unrg8982 . . . . .
. . . . . . . .
Vv08g1208 . . Gorai.002G238100.1 . .
. . . . . . . .
Vv18g1285 . . . . . .
Vv08g1209 . . Gorai.002G238200.1 . .
Gorai.007G367900.1 . . . . .

. . . . . . . .
.
Vv08g1210 . . . . . . .
. . . . . . . .
. . . . .
Vv08g1211 . . . . Gorai.009G399800.1
. . . . . . . .
. . . . . . .
Vv08g1212 . . . . . . .
. . . . . . . .
. . . . .
Vv08g1213 . . . . . . .
. . . . . . . .
. . . . .
Vv08g1214 Tc05_g009810 . Gorai.002G238300.1 .
. . Vv0Unrg8988 . . . .
. . . . . . . .
.
Vv08g1215 Tc05_g009680 . . . . .
Vv0Unrg8993 . . . . . .
. . . . . . .
Vv08g1216 Tc05_g009640 Gorai.006G153000.1 . .
Gorai.009G400000.1 . . . . .
. . . . . . . .
. .
Vv08g1217 Tc05_g009630 Gorai.006G153100.1 . .
. . . . . . . .
. . . . . . . .
Vv08g1218 . . Gorai.002G238500.1 . .
. . . . . . . .
. . . . . . .
Vv08g1219 . . . . . . .
. . . . . . . .
. . . . .
Vv08g1220 Tc05_g009610 . . . Gorai.
009G400100.1 . . . . . . .
. . . . . . . .
Vv08g1221 Tc05_g009600 . . . Gorai.
009G400200.1 . . . . . . .
. . . . . . . .
Vv08g1222 Tc05_g009590 Gorai.006G153200.1 .
Gorai.002G158100.1 Gorai.009G400400.1 Gorai.007G368100.1
Vv0Unrg8994 . . . . . .
. . . . . . .
Vv08g1223 Tc05_g009560 Gorai.006G153300.1 . .
. . . . . . . .
. . . . . . . .
Vv08g1224 . . . . . . .
. . . . . . . .
. . . . .

Vv08g1225 Tc05_g009520 . . . . .
. . . . . . . .
. . . . . .
Vv08g1226 . . . . . . .
. . . . . . . .
. . . . .
Vv08g1227 Tc05_g009490 . . . . .
. . . . . . . .
. . . . . .
Vv08g1228 Tc05_g009430 Gorai.006G153700.1 Gorai.
002G238600.1 . Gorai.009G400500.1 . . .
. . . . . . . .
. . . .
Vv08g1229 . . . . . . .
. . . . . . . .
. . . . .
Vv08g1230 Tc05_g009390 . Gorai.002G238700.1 .
. . . . . . . .
. . . . . . . .
Vv08g1231 Tc05_g009380 Gorai.006G153900.1 Gorai.
002G238800.1 . . . . . . .
. . . Vv18g1304 Tc08_g003800 .
. Gorai.009G193700.1 . .
Vv08g1232 . . . . . . .
. . . . . . . .
. . . . .
Vv08g1233 Tc05_g009360 Gorai.006G154100.1 . .
. . . . . . . .
. . . . . . . .
Vv08g1234 Tc05_g009350 Gorai.006G154200.1 . .
Gorai.009G400800.1 . . . . .
. . . . . . . .
. .
Vv08g1235 Tc05_g009340 Gorai.006G154300.1 . .
Gorai.009G400900.1 . . . . .
. . . . . . . .
. .
Vv08g1236 . . . . . . .
. . . . . . . .
. . . . .
Vv08g1237 Tc05_g009300 Gorai.006G154400.1 Gorai.
002G238900.1 . Gorai.009G401000.1 . . .
. . . . . . . .
. . . .
Vv08g1238 Tc05_g009290 Gorai.006G154600.1 Gorai.
002G239000.1 . . . Vv0Unrg9004 . .
. . . . . . . .
. . .
Vv08g1239 Tc05_g009270 . . . . .
. . . . . . . .

.	.	.	.	.	.
Vv08g1240	Tc05_g009260	Gorai.006G154700.1	.	.
	Gorai.009G401100.1	.	.	.	.	.
	.	.	.	.	.	.	.	.
	.	.
Vv08g1241	Tc05_g009250	.	.	.	.	.
	.	.	.	.	.	.	.	.
	.	.	.	.	.	.
Vv08g1242	.	.	.	.	.	.	.
	.	.	.	.	.	.	.	.
	.	.	.	.	.
Vv08g1243	Tc05_g009230	.	Gorai.002G239200.1	.
	Gorai.009G401300.1	.	.	.	.	.
	.	.	.	.	.	.	.	.
	.	.
Vv08g1244	Tc05_g009210	.	Gorai.002G239400.1	.
	Gorai.009G401400.1	.	.	.	.	.
	.	.	.	.	.	.	.	.
	.	.
Vv08g1245	.	.	.	.	.	.	.
	.	.	.	.	.	.	.	.
	.	.	.	.	.
Vv08g1246	Tc05_g009180	Gorai.006G155000.1	Gorai.
002G239600.1	.	Gorai.009G401500.1	.	.	.
	.	.	.	.	.	.	.	.
	.	.	.	.
Vv08g1247	Tc05_g009170	.	.	.	Gorai.
009G401600.1	.	.	.	.	.	.	.
	.	.	.	.	.	.	.	.
Vv08g1248	Tc05_g009130	.	Gorai.002G239700.1	.
	.	.	.	.	.	.	.	.
	.	Vv18g1318	Tc08_g003940	.	.
Gorai.009G193000.1	.	.
Vv08g1249	Tc05_g009100	.	.	.	Gorai.
009G402400.1	.	.	.	.	.	.	.
	.	.	.	.	.	.	.	.
Vv08g1250	Tc05_g009090	.	.	.	Gorai.
009G402500.1	.	.	.	.	.	.	.
	.	.	.	.	.	.	.	.
Vv08g1251	Tc05_g009070	.	.	.	Gorai.
009G402800.1	.	.	.	.	.	.	.
	.	.	.	.	.	.	.	.
Vv08g1252	.	.	.	.	.	.	.
	.	.	.	.	.	.	.	.
	.	.	.	.	.
Vv08g1253	Tc05_g009050	.	.	Gorai.002G158300.1
	.	.	.	.	.	.	.	.
	.	.	.	.	.	.	.	.
Vv08g1254	Tc05_g009040	.	Gorai.002G239900.1
Gorai.002G158400.1	.	.	.	.	.	.

. . . . . . . .
. .
Vv08g1255 Tc05_g009030 . . . . .
Vv0Unrg9023 . . . . . .
. . . . . . .
Vv08g1256 . . . . . .
Vv0Unrg9024 . . . . . . .
. . . . . .
Vv08g1257 . . . . . . .
. . . . . . . .
. . . . .
Vv08g1258 . . . . . . .
. . . . . . . .
. . . . .
Vv08g1259 . . . . . . .
. . . . . . . .
. . . . .
Vv08g1260 . . . . . . .
. . . . . . . .
. . . . .
Vv08g1261 Tc05_g008950 . Gorai.002G240100.1 .
. . . . . . . .
. . . . . . . .
Vv08g1262 Tc05_g008940 . Gorai.002G240200.1 .
Gorai.009G403000.1 . . . . .
. . . . . . . .
. .
Vv08g1263 Tc05_g008930 . Gorai.002G240300.1 .
. . . . . . . .
. . . . . . . .
Vv08g1264 Tc05_g008920 Gorai.006G155300.1 . .
. Gorai.007G368400.1 . . . .
. . . . . . . .
. .
Vv08g1265 . . Gorai.002G240400.1 . .
. . . . . . . .
. . . . . . .
Vv08g1266 Tc05_g008890 Gorai.006G155500.1 . .
Gorai.009G403300.1 . . . . .
. . . . . . . .
. .
Vv08g1267 . . . . . .
Vv0Unrg9031 . . . . . . .
. . . . . .
Vv08g1268 Tc05_g008870 Gorai.006G155600.1 Gorai.
002G240500.1 . . . Vv0Unrg9034 . .
. . . . Vv18g1339
Tc08_g004010 Gorai.002G122200.1 . Gorai.009G192500.1
. .
Vv08g1269 Tc05_g008860 Gorai.006G155700.1 . .

. . . . . . . .
. . . . . . . .
Vv08g1270 Tc05_g008810 . . Gorai.002G158600.1
Gorai.009G403700.1 . Vv0Unrg9036 . .
. . . . . . . .
. . .
Vv08g1271 Tc05_g008760 Gorai.006G155800.1 . .
Gorai.009G403800.1 . Vv0Unrg9043 . .
. . . . . . . .
. . .
Vv08g1272 Tc05_g008730 . Gorai.002G240700.1
Gorai.002G158800.1 . . . . . .
. . . . . . . .
. .
Vv08g1273 Tc05_g008720 Gorai.006G155900.1 . .
Gorai.009G404000.1 . . . . .
. . . . . . . .
. .
Vv08g1274 . . . . . . .
. . . . . . . .
. . . . .
Vv08g1275 Tc05_g008650 . . . . .
. . . . . . . .
. . . . . .
Vv08g1276 Tc05_g008630 . . . . .
. . . . . . . .
. . . . . .
Vv08g1277 Tc05_g008620 Gorai.006G156100.1 . .
Gorai.009G404200.1 . . . . .
. . . . . . . .
. .
Vv08g1278 . . . . . . .
. . . . . . . .
. . . . .
Vv08g1279 Tc05_g008610 Gorai.006G156200.1 Gorai.
002G240900.1 . Gorai.009G404700.1 . . .
. . . . . . . .
. . . .
Vv08g1280 . . . . Gorai.009G404800.1
. . . . . . . .
. . . . . . .
Vv08g1281 . . . . . . .
. . . . . . . .
. . . . .
Vv08g1282 . . . . . . .
. . . . . . . .
. . . . .
Vv08g1283 . . . . . . .
. . . . . . . .
. . . . .

Vv08g1284 . . . . . . .
. . . . . . . .
. . . . .
Vv08g1285 . . . . . . .
. . . . . . . .
. . . . .
Vv08g1286 . . . . . . .
. . . . . . . .
. . . . .
Vv08g1287 . . . . . . .
. . . . . . . .
. . . . .
Vv08g1288 . . . . . .
Vv13g0904 . . . . . . .
. . . . . .
Vv08g1289 . . . . . . .
. . . . . . . .
. . . . .
Vv08g1290 . . . Gorai.002G158900.1 .
Gorai.007G370500.1 . . . . .
. . . . . . . .
.
Vv08g1291 . . . . Gorai.009G405400.1
. . . . . . . .
. . . . . . .
Vv08g1292 . . . . . . .
. . . . . . . .
. . . . .
Vv08g1293 . Gorai.006G156400.1 Gorai.002G241100.1
. . . . . . . .
. . . . . . . .
.
Vv08g1294 . . . . . . .
. . . . . . . .
. . . . .
Vv08g1295 . . . . . . .
. . . . . . . .
. . . . .
Vv08g1296 . . . . . . .
. . . . . . . .
. . . . .
Vv08g1297 . . . . . . .
. . . . . . . .
. . . . .
Vv08g1298 . . . . . . .
. . . . . . . .
. . . . .
Vv08g1299 . . . . Gorai.009G406100.1
. . . . . . . .
. . . . . . .

Vv08g1300 . . . . . . .
. . . . . . . .
. . . . .
Vv08g1301 . . Gorai.002G241400.1 .
Gorai.009G406400.1 . . . . . .
. . . . . . . .
.
Vv08g1302 . Gorai.006G157300.1 . .
Gorai.009G406500.1 . . . . . .
. . . . . . . .
.
Vv08g1303 . Gorai.006G157400.1 Gorai.002G241500.1
. . . Vv13g0874 . . .
. . . . . . . .
. .
Vv08g1304 . Gorai.006G157500.1 Gorai.002G241600.1
Gorai.002G159100.1 . . . . .
. . . . . . . .
. . .
Vv08g1305 Tc05_g008570 . . . Gorai.
009G406900.1 . . . . . . .
. . . . . . . .
Vv08g1306 Tc05_g008520 . Gorai.002G241800.1
Gorai.002G159400.1 Gorai.009G407200.1 . . .
. . . . . . . .
. . . .
Vv08g1307 Tc05_g008470 . Gorai.002G242000.1
Gorai.002G159600.1 . . Vv02g0331 . .
. . Gorai.008G262400.1 . . .
. . . . .
Vv08g1308 Tc05_g008450 . Gorai.002G242100.1 .
Gorai.009G407400.1 . . . . .
. . . . . . . .
. .
Vv08g1309 Tc05_g008440 . Gorai.002G242200.1
Gorai.002G159700.1 . . . . . .
. . . . . . . .
. .
Vv08g1310 . . . . . . .
. . . . . . . .
. . . . .
Vv08g1311 Tc05_g008420 Gorai.006G157800.1 . .
. . . . . . . .
. . . . . . . .
Vv08g1312 . . . . . . .
. . . . . . . .
. . . . .
Vv08g1313 . . . . Gorai.009G407700.1
. . . . . . . .
. . . . . . .

```
Vv08g1314          .       .       .       .      Gorai.009G407800.1
        .          .       .       .       .       .       .       .
        .          .       .       .       .       .       .
Vv08g1315          Tc05_g008330    .       Gorai.002G242600.1      .
        .          .       .       .       .       .       .       .
        .          .       .       .       .       .       .       .
Vv08g1316          .       Gorai.006G158000.1      .       .       .
        .          .       .       .       .       .       .       .
        .          .       .       .       .       .       .
Vv08g1317          .       .       .       .       .       .       .
        .          .       .       .       .       .       .       .
        .          .       .       .       .
Vv08g1318          Tc05_g008290    Gorai.006G158300.1      .       .
        Gorai.009G408300.1         .       .       .       .       .
        .          .       .       .       .       .       .       .
        .          .
Vv08g1319          .       .       .       .       .       .       .
        .          .       .       .       .       .       .       .
        .          .       .       .       .
Vv08g1320          Tc05_g008270    .       Gorai.002G242700.1      .
        .          .       .       .       .       .       .       .
        .          .       .       .       .       .       .       .
Vv08g1321          .       .       Gorai.002G242900.1      .       .
        .          .       .       .       .       .       .       .
        .          .       .       .       .       .       .
Vv08g1322          Tc05_g008240    .       Gorai.002G243000.1      .
        .          Gorai.007G371300.1      .       .       .       .
        .          .       .       .       .       .       .       .
        .          .
Vv08g1323          .       .       .       .       .       .       .
        .          .       .       .       .       .       .       .
        .          .       .       .       .
Vv08g1324          .       .       .       .       .       .       .
        .          .       .       .       .       .       .       .
        .          .       .       .       .
Vv08g1325          .       .       .       .       .       .       .
        .          .       .       .       .       .       .       .
        .          .       .       .       .
Vv08g1326          Tc05_g008230    Gorai.006G158500.1      .       .
        .          .       .       .       .       .       .       .
        .          .       .       .       .       .       .       .
Vv08g1327          .       .       Gorai.002G243100.1      .       .
        .          .       .       .       .       .       .       .
        .          .       .       .       .       .       .
Vv08g1328          Tc05_g008220    .       Gorai.002G243200.1      .
        .          .       .       .       .       .       .       .
        .          .       .       .       .       .       .       .
Vv08g1329          Tc05_g008210    .       Gorai.002G243300.1      .
        .          .       .       .       .       .       .       .
        .          .       .       .       .       .       .       .
```

Vv08g1330 Tc05_g008200 . Gorai.002G243400.1 .
. . . . . . . .
. Vv13g0848 . . . . .
.
Vv08g1331 Tc05_g008190 Gorai.006G158600.1 . .
Gorai.009G408700.1 . . . . .
. . . . . . . .
. .
Vv08g1332 Tc05_g008180 . . . . .
. . . . . . . .
. . . . . .
Vv08g1333 . . . . . . .
. . . . . . . .
. . . . .
Vv08g1334 Tc05_g008170 Gorai.006G158700.1 .
Gorai.002G160000.1 Gorai.009G408800.1 . Vv02g0370
Tc03_g027340 Gorai.004G161300.1 . Gorai.
007G098600.1 Gorai.008G246700.1 . . . .
. . . .
Vv08g1335 Tc05_g008160 . Gorai.002G243500.1 .
. . . . . . . .
. . . . . . . .
Vv08g1336 . . . . . . .
. . . . . . . .
. . . . .
Vv08g1337 Tc05_g008150 . . . .
Gorai.007G371400.1 . . . . . .
. . . . . . . .
Vv08g1338 Tc05_g008140 . . . . .
. . . . . . . .
. . . . . .
Vv08g1339 . . . . . . .
. . . . . . . .
. . . . .
Vv08g1340 . . . . . . .
. . . . . . . .
. . . . .
Vv08g1341 . . . . . . .
. . . . . . . .
. . . . .
Vv08g1342 Tc05_g008050 . . . . .
. . . . . . . .
. . . . . .
Vv08g1343 . . . . . . .
. . . . . . . .
. . . . .
Vv08g1344 . . . . . . .
. . . . . . . .
. . . . .
Vv08g1345 . . . . . . .

. . . . . . . .
. . . . .
Vv08g1346 Tc05_g008040 . . . . .
. . . . . . . .
. . . . . .
Vv08g1347 . . . . . . .
. . . . . . . .
. . . . .
Vv08g1348 Tc05_g008030 . . . Gorai.
009G409200.1 . . . . . . .
. . . . . . . .
Vv08g1349 Tc05_g008020 . . . Gorai.
009G409300.1 . . . . . . .
. . . . . . . .
Vv08g1350 Tc05_g008010 . Gorai.002G243600.1 .
. . . . . . . .
. . . . . . . .
Vv08g1351 Tc05_g008000 . . . .
Gorai.007G371500.1 . . . . . .
. Vv13g0820 . . . . .
.
Vv08g1352 Tc05_g007990 Gorai.006G158900.1 Gorai.
002G243700.1 . . . . . . .
. . . Vv13g0815 . .
Gorai.011G233000.1 . . .
Vv08g1353 . . Gorai.002G243800.1 . .
. . . . . . . .
Vv13g0812 . . . . . .
Vv08g1354 Tc05_g007980 Gorai.006G159000.1 Gorai.
002G243900.1 . . . . . . .
. . . . . . . .
. .
Vv08g1355 Tc05_g007960 Gorai.006G159200.1 . .
Gorai.009G409500.1 . . . . .
. . . . . . . .
. .
Vv08g1356 Tc05_g007950 . Gorai.002G244200.1 .
Gorai.009G409600.1 . . . . .
. . . . . . . .
. .
Vv08g1357 Tc05_g007940 Gorai.006G159400.1 Gorai.
002G244300.1 . . Gorai.007G371600.1 . .
. . . . . Vv13g0805 .
. . . . .
Vv08g1358 Tc05_g007930 . Gorai.002G244400.1 .
Gorai.009G409800.1 . . . . .
. . . . . . . .
. .
Vv08g1359 . . . . . . .
. . . . . . . .

.	.	.	.	.
Vv08g1360	Tc05_g007920	.	.	Gorai.002G160300.1
Gorai.009G410000.1	.	.	.	.	.
.	.	.	.	.	.	.	.
.	.
Vv08g1361	Tc05_g007830	.	.	.	.	.
.	.	.	.	.	.	.	.
.	.	.	.	.	.
Vv08g1362	Tc05_g007810	Gorai.006G159600.1	Gorai.
002G244600.1	.	.	.	Vv02g0373
Tc03_g027310	Gorai.004G211300.1	.	Gorai.007G007000.1
.	.	.	.	.	.	.	.
.
Vv08g1363	.	.	.	.	.	.	.
.	.	.	.	.	.	.	.
.	.	.	.	.
Vv08g1364	Tc05_g007790	.	Gorai.002G244700.1	.
.	.	.	.	.	.	.	.
.	.	.	.	.	.	.	.
Vv08g1365	Tc05_g007780	.	Gorai.002G245000.1	.
.	.	.	.	.	.	.	.
.	.	.	.	.	.	.	.
Vv08g1366	Tc05_g007760	.	Gorai.002G245100.1	.
.	.	.	.	.	.	.	.
.	.	.	.	.	.	.	.
Vv08g1367	Tc05_g007750	Gorai.006G159700.1	.	.
Gorai.009G410200.1	.	.	.	.	.
.	.	.	.	.	.	.	.
.	.
Vv08g1368	.	.	.	.	Gorai.009G410300.1
.	Vv02g0386	Tc03_g027260	.	.	.
Gorai.008G246100.1	.	.	.	.	.
.	.	.
Vv08g1369	Tc05_g007740	Gorai.006G159800.1	.	.
.	.	.	.	.	.	.	.
.	.	.	.	.	.	.	.
Vv08g1370	Tc05_g007730	.	Gorai.002G245200.1	.
.	.	.	.	.	.	.	.
.	.	.	.	.	.	.	.
Vv08g1371	Tc05_g007720	.	.	.	.	.
.	.	.	.	.	.	.	.
.	.	.	.	.	.
Vv08g1372	Tc05_g007710	.	.	.	Gorai.
009G410400.1	.	.	.	.	.	.	.
.	.	.	.	.	.	.	.
Vv08g1373	Tc05_g007660	.	.	.	Gorai.
009G410600.1	Gorai.007G371700.1	.	.	.	.
.	.	.	.	.	.	.	.
.	.
Vv08g1374	Tc05_g007650	.	Gorai.002G245300.1	.

. . Vv06g1686 Tc09_g029200 Gorai.
013G259200.1 Gorai.001G118500.1 . . Gorai.
006G103200.1 . . . . . . .
Vv08g1375 . . . . . . .
. . . . . . . .
. . . . .
Vv08g1376 . . . . . . .
. . . . . . . .
. . . . .
Vv08g1377 . . . . . . .
. . . . . . . .
. . . . .
Vv08g1378 Tc05_g030100 . . . Gorai.
009G410800.1 . . . . . . .
. . . . . . . .
Vv08g1379 . . . . . . .
. . . . . . . .
. . . . .
Vv08g1380 Tc05_g007620 . . . . .
. . . . . . . .
. . . . . .
Vv08g1381 Tc05_g007610 . . . . .
. . . . . . . .
. . . . . .
Vv08g1382 Tc05_g007600 . . . . .
. . . . . . . .
. . . . . .
Vv08g1383 Tc05_g007590 . . . Gorai.
009G410900.1 . . . . . . .
. . . . . . . .
Vv08g1384 Tc05_g007580 . . . Gorai.
009G411000.1 Gorai.007G372100.1 . . . .
. . . . . . . .
. .
Vv08g1385 . . . . . . .
. . . . . . . .
. . . . .
Vv08g1386 . . . . . .
Vv06g1671 Tc09_g029270 . . . .
Gorai.006G103700.1 . . . . . .
.
Vv08g1387 Tc05_g030480 . . . . .
. . . . . . . .
. . . . . .
Vv08g1388 . . Gorai.002G245400.1 . .
. Vv06g1667 . . Gorai.001G117500.1
. . Gorai.006G104200.1 . . .
. . . .
Vv08g1389 . . Gorai.002G245500.1 . .
. Vv06g1666 Tc09_g029370 . . .

. Gorai.006G158300.1 . . . .
. . .
Vv08g1390 . . . . . . .
. . . . . . . .
. . . . .
Vv08g1391 Tc05_g007510 . . . . .
. . . . . . . .
. . . . . .
Vv08g1392 . . . . . . .
. . . . . . Vv13g0774
Tc10_g007710 . Gorai.011G233700.1 . .
.
Vv08g1393 . . . . . . .
. . . . . . . .
. . . . .
Vv08g1394 Tc05_g007470 . . . . .
. . . . . . . .
. . . . . .
Vv08g1395 . . . . . . .
. . . . . . . .
. . . . .
Vv08g1396 . . . . . . .
. . . . . . . .
. . . . .
Vv08g1397 . Gorai.006G160000.1 . . .
. . . . . . . .
. . . . . . .
Vv08g1398 . . . . Gorai.009G411100.1
. . . . . . . .
. . . . . . .
Vv08g1399 Tc05_g007420 . . . . .
. . . . . . . .
. . . . . .
Vv08g1400 . . . . . . .
. . . . . . . .
. . . . .
Vv08g1401 Tc05_g007410 Gorai.006G160700.1 . .
. . . . . . . .
. . . . . . . .
Vv08g1402 Tc05_g007400 . . . . .
. . . . . . . .
. . . . . .
Vv08g1403 Tc05_g007390 . . . . .
. . . . . . . .
. . . . . .
Vv08g1404 Tc05_g007330 . . . . .
. . . . . . . .
. . . . . .
Vv08g1405 Tc05_g007280 . . . . .
. . . . . . . .

. . . . . .
Vv08g1406 . . . . . Gorai.
007G372400.1 . . . . . . .
. . . . . . .
Vv08g1407 . Gorai.006G160800.1 . . .
. . . . . . . .
. . . . . . .
Vv08g1408 . . . . Gorai.009G412100.1
. . . . . . . .
. . . . . . .
Vv08g1409 . . . . . . .
. . . . . . . .
. . . . .
Vv08g1410 . . . . . . .
. . . . . . . .
. . . . .
Vv08g1411 . . . . . . .
. . . . . . . .
. . . . .
Vv08g1412 . . . . . . .
. . . . . . . .
. . . . .
Vv08g1413 . . . . Gorai.009G412500.1
. . . . . . . .
. . . . . . .
Vv08g1414 . . . . . . .
. . . . . . . .
. . . . .
Vv08g1415 . . . . Gorai.009G412600.1
. . . . . . . .
. . . . . . .
Vv08g1416 . . Gorai.002G245600.1 .
Gorai.009G412700.1 . . . . . .
. . . . . . . .
.
Vv08g1417 . Gorai.006G161000.1 . . .
. . . . . . . .
. . . . . . .
Vv08g1418 . . . . . . .
. . . . . . . .
. . . . .
Vv08g1419 . . . . . . .
. . . . . . . .
. . . . .
Vv08g1420 . . . . . . .
. . . . . . . .
. . . . .
Vv08g1421 . . Gorai.002G245700.1 . .
. . . . . . . .
. . . . . . .

Vv08g1422 . Gorai.006G161200.1 Gorai.002G245800.1
. Gorai.009G412900.1 . . . .
. . . . . . . .
. . .
Vv08g1423 . . Gorai.002G248400.1 .
Gorai.009G413000.1 Gorai.007G372700.1 Vv06g1657 .
. . . . . . . .
. . . .
Vv08g1424 . . . . Gorai.009G413400.1
. Vv06g1655 . . . . .
Gorai.003G146200.1 . . . . .
. .
Vv08g1425 . . Gorai.002G246100.1 .
Gorai.009G413500.1 . . . . . .
. . . . . . . .
.
Vv08g1426 . . . . . . .
. . . . . . Vv13g0732
. . . . . .
Vv08g1427 . . . . . . .
. . . . . . . .
. . . . .
Vv08g1428 . . . . Gorai.009G413700.1
Gorai.007G372800.1 . . . . .
. . . . . . . .
.
Vv08g1429 . Gorai.006G161400.1 Gorai.002G246300.1
. Gorai.009G413800.1 Gorai.007G372900.1 .
. . . . . . Vv13g0733
. . . . . .
Vv08g1430 Tc05_g007230 Gorai.006G161600.1 . .
. . Vv06g1641 . . . .
Gorai.009G111200.1 Gorai.006G161600.1 Vv13g0737
. . . . . .
Vv08g1431 . . . . . . .
. . . . . . . .
. . . . .
Vv08g1432 Tc05_g007220 Gorai.006G161700.1 . .
. . . . . . . .
. . . . . . . .
Vv08g1433 Tc05_g007210 Gorai.006G161800.1 Gorai.
002G246400.1 . Gorai.009G413900.1 . . .
. . . . . Vv13g0745 .
. . . . .
Vv08g1434 Tc05_g007200 Gorai.006G162100.1 . .
Gorai.009G414000.1 . . . . .
. . . . . . . .
. .
Vv08g1435 Tc05_g007190 Gorai.006G162200.1 . .
. . . . . . . .

        .       .       .       .       .       .       .       .
Vv08g1436       Tc05_g007180    Gorai.006G162300.1      .       .
        .       .       .       .       .       .       .       .
        .       .       .       .       .       .       .       .
Vv08g1437       Tc05_g007170    Gorai.006G162400.1      .       .
        Gorai.009G414100.1      Gorai.007G373000.1      Vv06g1636
        Tc09_g009340    .       .       .       Gorai.009G110900.1
        Gorai.006G162400.1      .       .       .       .       .
        .       .
Vv08g1438       .       .       .       .       .       .       .
        .       .       .       .       .       .       .       .
        .       .       .       .       .
Vv08g1439       Tc05_g007150    .       .       .       .       .
        .       .       .       .       .       .       .       .
        .       .       .       .       .       .
Vv08g1440       Tc05_g007140    .       .       .       Gorai.
009G414300.1    .       .       .       .       .       .       .
        .       .       .       .       .       .       .       .
Vv08g1441       Tc05_g007130    Gorai.006G162600.1      .       .
        Gorai.009G414400.1      .       .       .       .       .
        .       .       .       .       .       .       .       .
        .       .
Vv08g1442       Tc05_g007120    Gorai.006G162700.1      .       .
        Gorai.009G414500.1      Gorai.007G373100.1      Vv06g1630
        .       .       .       .       .       Gorai.006G162700.1
        .       .       .       .       .       .       .
Vv08g1443       .       .       .       .       .       .       .
        .       .       .       .       .       .       .       .
        .       .       .       .       .
Vv08g1444       Tc05_g007090    .       Gorai.002G246500.1      .
        .       .       .       .       .       .       .       .
        .       .       .       .       .       .       .       .
Vv08g1445       .       .       Gorai.002G246600.1      .       .
        .       .       .       .       .       .       .       .
        .       .       .       .       .       .       .
Vv08g1446       Tc05_g007080    .       .       .       .       .
        .       .       .       .       .       .       .       .
        .       .       .       .       .       .
Vv08g1447       .       .       .       .       .       .       .
        .       .       .       .       .       .       .       .
        .       .       .       .       .
Vv08g1448       .       .       .       .       .       .       .
        .       .       .       .       .       .       .       .
        .       .       .       .       .
Vv08g1449       .       Gorai.006G162900.1      .       .       .
        .       Vv06g1624       .       Gorai.013G269100.1      .
        .       .       Gorai.006G162900.1      Vv13g0762
Tc10_g007060    .       .       .       .       Gorai.012G007500.1
Vv08g1450       .       .       .       .       .       .
Vv13g0781       .       .       .       .       .       .       .

. . . . . .
Vv08g1451 Tc05_g007070 . . . . .
. . . . . . . .
. . . . . .
Vv08g1452 . . . . . . .
. . . . . . . .
. . . . .
Vv08g1453 . . . . . . .
. . . . . . . .
. . . . .
Vv08g1454 . . . . . . .
. . . . . . . .
. . . . .
Vv08g1455 . . . . . . .
. . . . . . . .
. . . . .
Vv08g1456 . . . . . . .
. . . . . . . .
. . . . .
Vv08g1457 . . . . . . .
. . . . . . . .
. . . . .
Vv08g1458 . . . . . . .
. . . . . . . .
. . . . .
Vv08g1459 . . . . . . .
. . . . . . . .
. . . . .
Vv08g1460 . . . . . . .
. . . . . . . .
. . . . .
Vv08g1461 . . . . . . .
. . . . . . . .
. . . . .
Vv08g1462 . . . . . . .
. . . . . . . .
. . . . .
Vv08g1463 . . . . . . .
. . . . . . . .
. . . . .
Vv08g1464 Tc05_g018200 . . . . .
. . . . . . . .
. . . . . .
Vv08g1465 . . . . . Gorai.
007G352600.1 . . . . . . .
. . . . . . .
Vv08g1466 . . . . . . .
. . . . . . . .
. . . . .
Vv08g1467 . . . . . . .

. . . . . . . .
. . . . .
Vv08g1468 . . . . . . .
. . . . . . . .
. . . . .
Vv08g1469 Tc05_g014680 . . . . .
Vv13g0710 Tc10_g006080 . Gorai.011G238000.1
Gorai.009G311500.1 . . . . .
. . . .
Vv08g1470 . . . . . . .
. . . . . . . .
. . . . .
Vv08g1471 . . Gorai.002G245600.1 . .
. . . . . . . .
. . . . . . .
Vv08g1472 . . . . . . .
. . . . . . . .
. . . . .
Vv08g1473 . . . . . . .
. . . . . . . .
. . . . .
Vv08g1474 . . . . . . .
. . . . . . . .
. . . . .
Vv08g1475 . . . . . . .
. . . . . . . .
. . . . .
Vv08g1476 . . . . . Gorai.
007G353000.1 . . . . . . .
. . . . . . .
Vv08g1477 . . . . . . .
. . . . . . . .
. . . . .
Vv08g1478 . . . . . . .
. . . . . . . .
. . . . .
Vv08g1479 . . . . . . .
. . . . . . . .
. . . . .
Vv08g1480 Tc05_g015090 . . . . .
. . . . . . . .
. . . . . .
Vv08g1481 Tc05_g015110 . . . .
Gorai.007G353100.1 . . . . . .
. . . . . . . .
Vv08g1482 Tc05_g015130 . . . .
Gorai.007G353200.1 . . . . . .
. . . . . . . .
Vv08g1483 Tc05_g015200 . . . . .
. . . . . . . .

```
        .       .       .       .       .       .
Vv08g1484       Tc05_g015240    .       .       .       .       .
        Vv13g0797       .       .       .       .       .       .
        .       .       .       .       .       .       .
Vv08g1485       Tc05_g015300    .       .       .       .       .
        .       .       .       .       .       .       .       .
        .       .       .       .       .       .
Vv08g1486       Tc05_g015330    .       .       .       .
Gorai.007G353400.1      .       .       .       .       .       .
        .       .       .       .       .       .       .       .
Vv08g1487       Tc05_g015340    .       .       .       .
Gorai.007G353500.1      .       .       .       .       .       .
        .       .       .       .       .       .       .       .
Vv08g1488       Tc05_g015350    .       .       .       .       .
        .       .       .       .       .       .       .       .
        .       .       .       .       .       .
Vv08g1489       Tc05_g015360    .       Gorai.002G243800.1      .
        .       .       Vv13g0812       .       .       .       .
        .       .       .       .       .       .       .       .
        .
Vv08g1490       .       .       .       .       .       .       .
        .       .       .       .       .       .       .       .
        .       .       .       .       .
Vv08g1491       Tc05_g018080    .       .       .       .       .
        .       .       .       .       .       .       .       .
        .       .       .       .       .       .
Vv08g1492       .       .       .       .       .       .       .
        .       .       .       .       .       .       .       .
        .       .       .       .       .
Vv08g1493       .       .       .       .       .       .       .
        .       .       .       .       .       .       .       .
        .       .       .       .       .
Vv08g1494       .       .       .       .       .       .       .
        .       .       .       .       .       .       .       .
        .       .       .       .       .
Vv08g1495       .       .       Gorai.002G243700.1      .       .
        .       Vv13g0815       .       .       Gorai.011G233000.1
        .       .       .       .       .       .       .       .
        .       .
Vv08g1496       Tc05_g015400    .       .       .       .
Gorai.007G353800.1      .       .       .       .       .       .
        .       .       .       .       .       .       .       .
Vv08g1497       .       .       .       .       .       .       .
        .       .       .       .       .       .       .       .
        .       .       .       .       .
Vv08g1498       .       .       .       .       .       .       .
        .       .       .       .       .       .       .       .
        .       .       .       .       .
Vv08g1499       .       .       .       .       .       .       .
        .       .       .       .       .       .       .       .
```

. . . . .
Vv08g1500 . . . . . . .
. . . . . . . .
. . . . .
Vv08g1501 . . . . . . .
. . . . . . . .
. . . . .
Vv08g1502 Tc05_g015530 . . . . .
. . . . . . . .
. . . . . .
Vv08g1503 Tc05_g015540 . . . . .
. . . . . . . .
. . . . . .
Vv08g1504 . . . . . . .
. . . . . . . .
. . . . .
Vv08g1505 . . . . . . .
. . . . . . . .
. . . . .
Vv08g1506 . . . . . . .
. . . . . . . .
. . . . .
Vv08g1507 . . . . . . .
. . . . . . . .
. . . . .
Vv08g1508 . . . . . . .
. . . . . . . .
. . . . .
Vv08g1509 . . . . . Gorai.
007G354000.1 . . . . . . .
. . . . . . .
Vv08g1510 . . . . . . .
. . . . . . . .
. . . . .
Vv08g1511 . . . . . Gorai.
007G354100.1 . . . . . . .
. . . . . . .
Vv08g1512 . . . . . . .
. . . . . . . .
. . . . .
Vv08g1513 . . . . . . .
. . . . . . . .
. . . . .
Vv08g1514 . . Gorai.002G240700.1 . .
. . . . . . . .
. . . . . . .
Vv08g1515 Tc05_g020040 . . . . .
. . . . . . . .
. . . . . .
Vv08g1516 . . . . . . .

| | | | | | | | |
|---|---|---|---|---|---|---|---|
| . | . | . | . | . | . | . | . |
| . | . | . | . | . | | | |
| Vv08g1517 | Tc05_g017800 | | . | . | . | . | . |
| . | . | . | . | . | . | . | . |
| . | . | . | . | . | . | | |
| Vv08g1518 | . | . | . | . | . | . | . |
| . | . | . | . | . | . | . | . |
| . | . | . | . | . | | | |
| Vv08g1519 | Tc05_g017760 | | . | . | . | . | . |
| . | . | . | . | . | . | . | . |
| . | . | . | . | . | . | | |
| Vv08g1520 | . | . | . | . | . | . | . |
| . | . | . | . | . | . | . | . |
| . | . | . | . | . | | | |
| Vv08g1521 | Tc05_g017590 | | . | . | . | . | . |
| . | . | . | . | . | . | . | . |
| . | . | . | . | . | . | | |
| Vv08g1522 | Tc05_g017570 | | . | . | . | . | . |
| . | . | . | . | . | . | . | . |
| . | . | . | . | . | . | | |
| Vv08g1523 | . | . | . | . | . | . | . |
| . | . | . | . | . | . | . | . |
| . | . | . | . | . | | | |
| Vv08g1524 | . | . | . | . | . | . | . |
| . | . | . | . | . | . | . | . |
| . | . | . | . | . | | | |
| Vv08g1525 | . | . | . | . | . | . | . |
| . | . | . | . | . | . | . | . |
| . | . | . | . | . | | | |
| Vv08g1526 | . | . | . | . | . | . | . |
| . | . | . | . | . | . | . | . |
| . | . | . | . | . | | | |
| Vv08g1527 | . | . | . | . | . | . | . |
| . | . | . | . | . | . | . | . |
| . | . | . | . | . | | | |
| Vv08g1528 | . | . | . | . | . | . | . |
| . | . | . | . | . | . | . | . |
| . | . | . | . | . | | | |
| Vv08g1529 | . | . | . | . | . | . | . |
| . | . | . | . | . | . | . | . |
| . | . | . | . | . | | | |
| Vv08g1530 | . | . | . | . | . | . | . |
| . | . | . | . | . | . | . | . |
| . | . | . | . | . | | | |
| Vv08g1531 | . | . | . | . | . | . | . |
| . | . | . | . | . | . | . | . |
| . | . | . | . | . | | | |
| Vv08g1532 | . | . | . | . | . | . | . |
| . | . | . | . | . | . | . | . |
| . | . | . | . | . | | | |

| | | | | | | | | | | | | | | | | | | | | |
|---|---|---|---|---|---|---|---|---|---|---|---|---|---|---|---|---|---|---|---|---|
| Vv08g1533 | . | . | . | . | . | . | . | . | . | . | . | . | . | . | . | . | . | . | . | . |
| Vv08g1534 | Tc05_g020020 | . | . | . | . | . | . | . | . | . | . | . | . | . | . | . | . | . | . | . |
| Vv08g1535 | . | . | . | . | . | . | . | . | . | . | . | . | . | . | . | . | . | . | . | . |
| Vv08g1536 | Tc05_g016110 | . | . | . | . | . | . | . | . | . | . | . | . | . | . | . | . | . | . | . |
| Vv08g1537 | Tc05_g016120 | . | . | . | . | . | . | . | . | . | . | . | . | . | . | . | . | . | . | . |
| Vv08g1538 | . | . | . | . | . | . | . | . | . | . | . | . | . | . | . | . | . | . | . | . |
| Vv08g1539 | . | . | . | . | . | . | . | . | . | . | . | . | . | . | . | . | . | . | . | . |
| Vv08g1540 | . | . | . | . | . | . | . | . | . | . | . | . | . | . | . | . | . | . | . | . |
| Vv08g1541 | . | . | . | . | . | . | . | . | . | . | . | . | . | . | . | . | . | . | . | . |
| Vv08g1542 | . | . | . | . | . | . | . | . | . | . | . | . | . | . | . | . | . | . | . | . |
| Vv08g1543 | . | . | . | . | . | . | . | . | . | . | . | . | . | . | . | . | . | . | . | . |
| Vv08g1544 | . | . | . | . | . | . | . | . | . | . | . | . | . | . | . | . | . | . | . | . |
| Vv08g1545 | . | . | . | . | . | . | . | . | . | . | . | . | . | . | . | . | . | . | . | . |
| Vv08g1546 | . | . | . | . | . | . | . | . | . | . | . | . | . | . | . | . | . | . | . | . |
| Vv08g1547 | . | . | . | . | . | . | . | . | . | . | . | . | . | . | . | . | . | . | . | . |
| Vv08g1548 | . | . | . | . | . | . | . | . | . | . | . | . | . | . | . | . | . | . | . | . |
| Vv08g1549 | . | . | . | . | . | . | . | . | . | . | . | . | . | . | . | . | . | . | . | . |

. . . . .
Vv08g1550 . . . . . . .
. . . . . . . .
. . . . .
Vv08g1551 . . . . . . .
. . . . . . . .
. . . . .
Vv08g1552 . . . Gorai.002G156900.1 .
Gorai.007G361900.1 . . . . .
. . . . . . . .
.
Vv08g1553 . . . . . . .
. . . . . . . .
. . . . .
Vv08g1554 . . . . . . .
. . . . . . . .
. . . . .
Vv08g1555 . . . . . . .
. . . . . . . .
. . . . .
Vv08g1556 . . . Gorai.002G156800.1 .
. . . . . . . .
. . . . . . .
Vv08g1557 . . . . . . .
. . . . . . . .
. . . . .
Vv08g1558 . . . . . . .
. . . . . . . .
. . . . .
Vv08g1559 . . . . . . .
. . . . . . . .
. . . . .
Vv08g1560 . . . . . . .
. . . . . . . .
. . . . .
Vv08g1561 . . . . . . .
. . . . . . . .
. . . . .
Vv08g1562 . . . . . . .
. . . . . . . .
. . . . .
Vv08g1563 . . . Gorai.002G156700.1 .
Gorai.007G362000.1 . . . . .
. . . . . . . .
.
Vv08g1564 . . . . . . .
. . . . . . . .
. . . . .
Vv08g1565 . . . . . . .
. . . . . . . .

. . . . .
Vv08g1566 . . . . . . .
. . . . . . . .
. . . . .
Vv08g1567 . . . . . . .
. . . . . . . .
. . . . .
Vv08g1568 Tc05_g018920 . . . . .
. . . . . . . .
. . . . . .
Vv08g1569 . . . . . . .
. . . . . . . .
. . . . .
Vv08g1570 Tc05_g018910 . . . . .
Vv06g0168 . Gorai.013G249400.1 .
Gorai.004G018500.1 Gorai.009G070600.1 . . .
. . . . .
Vv08g1571 Tc05_g018880 . . . . .
. . . . . . . .
. . . . . .
Vv08g1572 . . . . . . .
. . . . . . . .
. . . . .
Vv08g1573 Tc05_g019950 . . . . .
. . . . . . . .
. . . . . .
Vv08g1574 . . . . . . .
. . . . . . . .
. . . . .
Vv08g1575 Tc05_g018870 . . . . .
Vv06g0165 . . . . Gorai.
009G070400.1 . . . . . . .
.
Vv08g1576 Tc05_g018860 . . . . .
Vv06g0164 Tc09_g031150 Gorai.013G249200.1 .
Gorai.004G018400.1 . . . . .
. . . .
Vv08g1577 Tc05_g018850 . . . . .
Vv06g0162 . . . . Gorai.
009G070300.1 . . . . . . .
.
Vv08g1578 Tc05_g019670 . . . . .
. . . . . . . .
. . . . . .
Vv08g1579 Tc05_g018840 . . . . .
. . . . . . . .
. . . . . .
Vv08g1580 . . . . . . .
. . . . . . . .
. . . . .

```
Vv08g1581       .       .       .       .       .       .       .
        .       .       .       .       .       .       .       .
        .       .       .       .       .
Vv08g1582       Tc05_g018800    .       .       Gorai.002G156600.1
        Gorai.007G362300.1      Gorai.007G362300.1      Vv06g0157
        .       Gorai.013G248700.1      .       .       .       .
        .       .       .       .       .       .       .
Vv08g1583       Tc05_g021050    .       .       .       .       .
        .       .       .       .       .       .       .       .
        .       .       .       .       .       .
Vv08g1584       Tc05_g018790    .       .       .       .       .
        .       .       .       .       .       .       .       .
        .       .       .       .       .       .
Vv08g1585       Tc05_g018780    .       .       .       .       .
        Vv06g0154       .       .       .       .       .       .
        .       .       .       .       .       .       .
Vv08g1586       Tc05_g018770    .       .       .       .       .
        .       .       .       .       .       .       .       .
        .       .       .       .       .       .
Vv08g1587       Tc05_g018750    .       .       .       .       .
        .       .       .       .       .       .       .       .
        .       .       .       .       .       .
Vv08g1588       Tc05_g018740    .       .       Gorai.002G156400.1
        .       .       .       .       .       .       .       .
        .       .       .       .       .       .       .       .
Vv08g1589       Tc05_g018730    .       .       .       .       .
        .       .       .       .       .       .       .       .
        .       .       .       .       .       .
Vv08g1590       Tc05_g018720    .       .       .       .       .
        .       .       .       .       .       .       .       .
        .       .       .       .       .       .
Vv08g1591       Tc05_g018680    .       .       .       .       .
        .       .       .       .       .       .       .       .
        .       .       .       .       .       .
Vv08g1592       Tc05_g018670    .       .       .       .       .
        .       .       .       .       .       .       .       .
        .       .       .       .       .       .
Vv08g1593       .       .       .       .       .       .       .
        .       .       .       .       .       .       .       .
        .       .       .       .       .
Vv08g1594       Tc05_g018650    .       .       .       .       .
        .       .       .       .       .       .       .       .
        .       .       .       .       .       .
Vv08g1595       .       .       .       .       .       .       .
        .       .       .       .       .       .       .       .
        .       .       .       .       .
Vv08g1596       .       .       .       .       .       .       .
        .       .       .       .       .       .       .       .
        .       .       .       .       .
Vv08g1597       .       .       .       .       .       .       .
```

. . . . . . . .
. . . . .
Vv08g1598 . . . Gorai.002G156300.1 .
. . . . . . . .
. . . . . . .
Vv08g1599 . . . . . . .
. . . . . . . .
. . . . .
Vv08g1600 . . . Gorai.002G155900.1 .
Gorai.007G362400.1 . . . . .
. . . . . . . .
.
Vv08g1601 . . . Gorai.002G155800.1 .
. . . . . . . .
. . . . . . .
Vv08g1602 . . . . . . .
. . . . . . . .
. . . . .
Vv08g1603 . . . . . . .
. . . . . . . .
. . . . .
Vv08g1604 . . . . . . .
. . . . . . . .
. . . . .
Vv08g1605 . . . . . . .
. . . . . . . .
. . . . .
Vv08g1606 . . . . . . .
. . . . . . . .
. . . . .
Vv08g1607 Tc05_g019640 . . . . .
. . . . . . . .
. . . . . .
Vv08g1608 . . . Gorai.002G155600.1 .
. Vv06g0144 . . . . .
. . . . . . . .
Vv08g1609 . . . . . . .
. . . . . . . .
. . . . .
Vv08g1610 . . . . . .
Vv06g0142 . Gorai.013G248400.1 . . .
. . . . . . . .
Vv08g1611 . . . . . . .
. . . . . . . .
. . . . .
Vv08g1612 . . . Gorai.002G155500.1 .
. . . . . . . .
. . . . . . .
Vv08g1613 . . . Gorai.002G155400.1 .
. . . . . . . .

. . . . . . .
Vv08g1614 . . . . . . .
. . . . . . . .
. . . . .
Vv08g1615 . . . . . .
Vv06g0139 . . . . . . .
. . . . . .
Vv08g1616 . . . . . . .
. . . . . . . .
. . . . .
Vv08g1617 . . . Gorai.002G155200.1 .
. . . . . . . .
. . . . . . .
Vv08g1618 . . . . . . .
. . . . . . . .
. . . . .
Vv08g1619 . . . . . . .
. . . . . . . .
. . . . .
Vv08g1620 . . . Gorai.002G155100.1 .
. . . . . . . .
. . . . . . .
Vv08g1621 . . . . . . .
. . . . . . . .
. . . . .
Vv08g1622 . . . . . . .
. . . . . . . .
. . . . .
Vv08g1623 Tc05_g020680 . . . .
Gorai.007G362500.1 Vv06g0133 . . . .
. . . . . . . .
.
Vv08g1624 Tc05_g020670 . . . . .
Vv06g0132 . . . . . .
. . . . . . .
Vv08g1625 . . . . . Gorai.
007G362600.1 Vv06g0129 . . . .
. . . . . . . .
Vv08g1626 . . . . . . .
. . . . . . . .
. . . . .
Vv08g1627 . . . . . .
Vv06g0128 . . . . . . .
. . . . . .
Vv08g1628 . . . . . . .
. . . . . . . .
. . . . .
Vv08g1629 . . . . . .
Vv06g0127 . . . . . . .
. . . . . .

Vv08g1630 . . . . . . .
. . . . . . . .
. . . . .
Vv08g1631 Tc05_g020650 . . . . .
Vv06g0126 . Gorai.013G248100.1 .
Gorai.004G017700.1 . . . . . .
. . .
Vv08g1632 . . . . . .
Vv06g0125 . . . . . . .
. . . . . .
Vv08g1633 Tc05_g020630 . Gorai.002G231800.1 .
. . . . . . . .
. . . . . . . .
Vv08g1634 Tc05_g020590 . Gorai.002G231900.1 .
. . . . . . . .
. . . . . . . .
Vv08g1635 Tc05_g020560 . . . . .
. . . . . . . .
. . . . . .
Vv08g1636 . . . . . .
Vv06g0117 . . . . . . .
. . . . . .
Vv08g1637 Tc05_g020540 . . . . .
. . . . . . . .
. . . . . .
Vv08g1638 Tc05_g020530 . . . . .
. . . . . . . .
. . . . . .
Vv08g1639 . . . . . . .
. . . . . . . .
. . . . .
Vv08g1640 . . . . . . .
. . . . . . . .
. . . . .
Vv08g1641 . . . . . . .
. . . . . . . .
. . . . .
Vv08g1642 . . . . . . .
. . . . . . . .
. . . . .
Vv08g1643 Tc05_g020510 . . . . .
. . . . . . . .
. . . . . .
Vv08g1644 . . . . . . .
. . . . . . . .
. . . . .
Vv08g1645 Tc05_g020400 . . . . .
. . . . . . . .
. . . . . .
Vv08g1646 Tc05_g020360 . . . . .

```
          .       .       .       .       .       .       .       .
          .       .       .       .       .       .
Vv08g1647       Tc05_g020340      .       .       .       .       .
          .       .       .       .       .       .       .       .
          .       .       .       .       .       .
Vv08g1648       Tc05_g020330      .       .       .       .       .
        Vv06g0112       .       Gorai.013G247700.1        .       .
        Gorai.009G067500.1        .       .       .       .       .
          .       .       .
Vv08g1649       Tc05_g020310      .       .       .       .       .
          .       .       .       .       .       .       .       .
          .       .       .       .       .       .
Vv08g1650       Tc05_g020300      .       .       .       .       .
        Vv06g0078       Tc09_g021690    Gorai.013G244100.1        .
          .       .       .       .       .       .       .       .
          .       .
Vv08g1651       Tc05_g020290      .       .       .       .       .
          .       .       .       .       .       .       .       .
          .       .       .       .       .       .
Vv08g1652       Tc05_g020280      .       .       .       .
Gorai.007G362800.1        .       .       .       .       .       .
          .       .       .       .       .       .       .       .
Vv08g1653       Tc05_g020270      .       .       Gorai.002G154600.1
          .       .       .       .       .       .       .       .
          .       .       .       .       .       .       .       .
Vv08g1654       Tc05_g020250      .       .       .       .       .
          .       .       .       .       .       .       .       .
          .       .       .       .       .       .
Vv08g1655       .       .       .       .       .       .       .
          .       .       .       .       .       .       .       .
          .       .       .       .       .
Vv08g1656       Tc05_g020240      .       Gorai.002G232500.1        .
          .       .       .       .       .       .       .       .
          .       .       .       .       .       .       .       .
Vv08g1657       Tc05_g020230      .       .       .       .       .
          .       .       .       .       .       .       .       .
          .       .       .       .       .       .
Vv08g1658       .       .       .       .       .       .       .
          .       .       .       .       .       .       .       .
          .       .       .       .       .
Vv08g1659       Tc05_g020220      .       .       .       .       .
          .       .       .       .       .       .       .       .
          .       .       .       .       .       .
Vv08g1660       .       .       .       .       .       .       .
          .       .       .       .       .       .       .       .
          .       .       .       .       .
Vv08g1661       .       .       Gorai.002G232600.1        Gorai.
002G154300.1    .       .       .       .       .       .       .
          .       .       .       .       .       .       .       .
          .
```

Vv08g1662 Tc05_g024140 Gorai.013G114900.1 . .
. . . . . . . .
. . . . . . . .
Vv08g1663 Tc05_g024150 . . . . .
Vv06g0070 Tc09_g020260 Gorai.013G244400.1 .
. . . . . . . .
. .
Vv08g1664 Tc05_g024160 Gorai.013G115000.1 . .
. . . . . . . .
. . . . . . . .
Vv08g1665 . . . Gorai.002G151800.1 .
. Vv06g0068 Tc09_g020300 Gorai.013G244600.1
. . . Gorai.003G126300.1 . .
. . . . .
Vv08g1666 Tc05_g024170 Gorai.013G115100.1 . .
Gorai.007G346000.1 . . . . .
. . . . . . . .
. .
Vv08g1667 . . . . . . .
. . . . . . . .
. . . . .
Vv08g1668 Tc05_g024180 . . . . .
. . . . . . . .
. . . . . .
Vv08g1669 Tc05_g024230 Gorai.013G115200.1 . .
. . . . . . . .
. . . . . . . .
Vv08g1670 Tc05_g024240 . . . Gorai.
007G345900.1 . . . . . . .
. . . . . . . .
Vv08g1671 Tc05_g024250 . . . Gorai.
007G345800.1 . . . . . . .
. . . . . . . .
Vv08g1672 Tc05_g024270 Gorai.013G115700.1 . .
. . Vv06g0056 Tc09_g004560 .
Gorai.001G023800.1 . Gorai.009G047000.1 Gorai.
003G150800.1 . . . . . . .
Vv08g1673 Tc05_g024280 . . . . .
. . . . . . . .
. . . . . .
Vv08g1674 . . . . . Gorai.
007G343200.1 . . . . . . .
. . . . . . .
Vv08g1675 Tc05_g024330 . . . . .
. . . . . . . .
. . . . . .
Vv08g1676 . . . . . . .
. . . . . . . .
. . . . .
Vv08g1677 . . . . . . .

. . . . . . . .
. . . . .
Vv08g1678 . . . . . Gorai.
007G343400.1 . . . . . . .
. . . . . . .
Vv08g1679 . . . . . Gorai.
007G343500.1 . . . . . . .
. . . . . . .
Vv08g1680 . . . . . . .
. . . . . . . .
. . . . .
Vv08g1681 . . . . . .
Vv06g0037 . . . . . Gorai.
003G129200.1 . . . . . . .
Vv08g1682 . . . . . . .
. . . . . . . .
. . . . .
Vv08g1683 . . . . . Gorai.
007G343600.1 Vv06g0036 . . . . .
. . . . . . . .
Vv08g1684 . . . . . . .
. . . . . . . .
. . . . .
Vv08g1685 . . . . . . .
. . . . . . . .
. . . . .
Vv08g1686 . . . . . . .
. . . . . . . .
. . . . .
Vv08g1687 . . . . . . .
. . . . . . . .
. . . . .
Vv08g1688 Tc05_g000360 . . . . .
. . . . . . . .
. . . . . .
Vv08g1689 Tc05_g010580 . . . . .
. . . . . . . .
. . . . . .
Vv08g1690 . . . . . . .
. . . . . . . .
. . . . .
Vv08g1691 Tc05_g010650 . . . . .
. . . . . . . .
. . . . . .
Vv08g1692 . . . . . . .
. . . . . . . .
. . . . .
Vv08g1693 . . . . . . .
. . . . . . . .
. . . . .

Vv08g1694 . . . . . . .
. . . . . . . .
. . . . .
Vv08g1695 . . . . . . .
. . . . . . . .
. . . . .
Vv08g1696 . . . . . . .
. . . . . . . .
. . . . .
Vv08g1697 Tc05_g024650 . . . . .
. . . . . . . .
. . . . . .
Vv08g1698 Tc05_g024670 Gorai.013G117600.1 . .
. . Vv06g0023 Tc09_g018470 . .
. . Gorai.003G154200.1 . . .
. . . .
Vv08g1699 Tc05_g024970 . . . . .
. . . . . . . .
. . . . . .
Vv08g1700 Tc05_g024980 Gorai.013G117900.1 . .
. . . . . . . .
. . . . . . . .
Vv08g1701 Tc05_g024990 . . . . .
. . . . . . . .
. . . . . .
Vv08g1702 Tc05_g025020 Gorai.013G118000.1 . .
. . . . . . . .
. . . . . . . .
Vv08g1703 . . . . . . .
. . . . . . . .
. . . . .
Vv08g1704 Tc05_g025030 Gorai.013G118100.1 . .
. . . . . . . .
. . . . . . . .
Vv08g1705 . . . . . . .
. . . . . . . .
. . . . .
Vv08g1706 Tc05_g025040 . . . . .
. . . . . . . .
. . . . . .
Vv08g1707 . . . . . . .
. . . . . . . .
. . . . .
Vv08g1708 Tc05_g025070 . . . . .
. . . . . . . .
. . . . . .
Vv08g1709 . . . . . . .
. . . . . . . .
. . . . .
Vv08g1710 . . . . . . .

```
        .       .       .       .       .       .       .       .
        .       .       .       .       .
Vv08g1711       .       .       Gorai.002G228500.1      .       .
        .       .       .       .       .       .       .       .
        .       .       .       .       .       .       .
Vv08g1712       .       .       .       .       .       .       .
        .       .       .       .       .       .       .       .
        .       .       .       .       .
Vv08g1713       .       .       .       .       .       .       .
        .       .       .       .       .       .       .       .
        .       .       .       .       .
Vv08g1714       Tc05_g008040    .       .       .       .       .
        .       .       .       .       .       .       .       .
        .       .       .       .       .       .
Vv08g1715       Tc05_g008050    .       .       .       .       .
        .       .       .       .       .       .       .       .
        .       .       .       .       .       .
Vv08g1716       Tc05_g008140    .       .       .       .       .
        .       .       .       .       .       .       .       .
        .       .       .       .       .       .
Vv08g1717       Tc05_g000120    .       .       .       .       .
        Vv0Unrg9201     .       .       .       .       .       .
        .       .       .       .       .       .       .
Vv08g1718       .       .       .       .       .       .       .
        .       .       .       .       .       .       .       .
        .       .       .       .       .
Vv08g1719       Tc05_g000110    .       .       .       .       .
        .       .       .       .       .       .       .       .
        .       .       .       .       .       .
Vv08g1720       .       .       .       .       .       .       .
        .       .       .       .       .       .       .       .
        .       .       .       .       .
Vv08g1721       .       .       .       .       .       .
Vv0Unrg9200     .       .       .       .       .       .       .
        .       .       .       .       .       .
Vv08g1722       Tc05_g000110    .       .       .       .       .
        Vv0Unrg9199     .       .       .       .       .       .
        .       .       .       .       .       .       .
Vv08g1723       .       .       .       .       .       .       .
        .       .       .       .       .       .       .       .
        .       .       .       .       .
Vv08g1724       .       .       .       .       .       .       .
        .       .       .       .       .       .       .       .
        .       .       .       .       .
Vv08g1725       .       .       Gorai.002G228900.1      .       .
        .       .       .       .       .       .       .       .
        .       .       .       .       .       .       .
Vv08g1726       Tc05_g004050    .       .       .       .       .
        .       .       .       .       .       .       .       .
        .       .       .       .       .       .
```

Vv08g1727 . . . . . . .
. . . . . . . .
. . . . .
Vv08g1728 . . . . . . .
. . . . . . . .
. . . . .
Vv08g1729 . . . . . . .
. . . . . . . .
. . . . .
Vv08g1730 . . . . . . .
. . . . . . . .
. . . . .
Vv08g1731 . . Gorai.002G229100.1 . .
. . . . . . . .
. . . . . . .
Vv08g1732 Tc05_g000120 . . . . .
Vv0Unrg9198 . . . . . .
. . . . . . .
Vv08g1733 . . . . . . .
. . . . . . . .
. . . . .
Vv08g1734 . . . . . . .
. . . . . . . .
. . . . .
Vv08g1735 . . . . . . .
. . . . . . . .
. . . . .
Vv08g1736 . . . . . . .
. . . . . . . .
. . . . .
Vv08g1737 . . . . . Gorai.
007G357900.1 . . . . . . .
. . . . . . .
Vv08g1738 Tc05_g025560 . . . . .
. . . . . . . .
. . . . . .
Vv08g1739 . . . . . . .
. . . . . . . .
. . . . .
Vv08g1740 . . . . . . .
. . . . . . . .
. . . . .
Vv08g1741 Tc05_g008610 . Gorai.002G229200.1 .
. Gorai.007G358100.1 . . . .
. . . Vv13g1290 . . .
. . .
Vv08g1742 . . Gorai.002G229300.1 . .
. . . . . . . .
. . . . . . .
Vv08g1743 Tc05_g003760 . . . Gorai.

012G108100.1 . . . . . . .

. . . . . . . .

Vv08g1744 . . . . . . .

. . . . . . . .

. . . . .

Vv08g1745 . . . . . . .

. . . . . . . .

. . . . .

Vv08g1746 . . Gorai.002G229800.1 . .

. . . . . . . .

. . . . . . .

Vv08g1747 . . . . . . .

. . . . . . . .

. . . . .

Vv08g1748 . . . . . . .

. . . . . . Vv13g1280

. . . . . .

Vv08g1749 . . Gorai.002G229900.1 . .

. . . . . . . .

. . . . . . .

Vv08g1750 . . . . . . .

. . . . . . . .

. . . . .

Vv08g1751 . . . . . . .

. . . . . . . .

. . . . .

Vv08g1752 . . Gorai.002G230000.1 . .

. . . . . . . .

. . . . . . .

Vv08g1753 . . . . . . .

. . . . . . . .

. . . . .

Vv08g1754 . . . . . . .

. . . . . . . .

. . . . .

Vv08g1755 . . . . . . .

. . . . . . . .

. . . . .

Vv08g1756 . . . . . . .

. . . . . . . .

. . . . .

Vv08g1757 . . . . . . .

. . . . . . . .

. . . . .

Vv08g1758 Tc05_g025670 . . . .

Gorai.007G359400.1 . . . . . .

. . . . . . . .

Vv08g1759 . . . . . . .

. . . . . . . .

. . . . .

Vv08g1760 . . Gorai.002G230200.1 . .
. . . . . . . .
. . . . . . .
Vv08g1761 . . . . . . .
. . . . . . . .
. . . . .
Vv08g1762 Tc05_g008930 . . . . .
. . . . . . . .
. . . . . .
Vv08g1763 . . Gorai.002G230300.1 . .
. Vv0Unrg9174 . . . . .
. Vv13g1264 . . . . .
.
Vv08g1764 . . . . . .
Vv13g1262 . . . . . . .
. . . . . .
Vv08g1765 . . . . . . .
. . . . . . . .
. . . . .
Vv08g1766 . . . . . .
Vv13g1256 . . . . . . .
. . . . . .
Vv08g1767 . . . . . Gorai.
007G359800.1 . . . . . . .
. . . . . . .
Vv08g1768 Tc05_g000360 . . . . .
. . . . . . . .
. . . . . .
Vv08g1769 . . . . . Gorai.
007G359900.1 . . . . . . .
. . . . . . .
Vv08g1770 . . . . . . .
. . . . . . . .
. . . . .
Vv08g1771 . . . . . . .
. . . . . . . .
. . . . .
Vv08g1772 . . . . . . .
. . . . . . . .
. . . . .
Vv08g1773 . . . . . . .
. . . . . . . .
. . . . .
Vv08g1774 . . . . . .
Vv13g1239 . . . . . . .
. . . . . .
Vv08g1775 . . . . . .
Vv13g1237 . . . . . . .
. . . . . .
Vv08g1776 . . . . . Gorai.

007G360200.1 Vv13g1227 . . . . .

. . . . . . . .

Vv08g1777 . . Gorai.002G230400.1 . .

. . . . . . . .

. . . . . . .

Vv08g1778 . . . . . . .

. . . . . . . .

. . . . .

Vv08g1779 . . . . . . .

. . . . . . . .

. . . . .

Vv08g1780 . . . . . . .

. . . . . . . .

. . . . .

Vv08g1781 . . . . . . .

. . . . . . . .

. . . . .

Vv08g1782 . . . . . . .

. . . . . . . .

. . . . .

Vv08g1783 Tc05_g009250 . . . . .

. . . . . . . .

. . . . . .

Vv08g1784 . . . . . . .

. . . . . . . .

. . . . .

Vv08g1785 Tc05_g025840 . . . . .

. . . . . . . .

. . . . . .

Vv08g1786 Tc05_g025870 . . . . .

. . . . . . . .

. . . . . .

Vv08g1787 . . . . . . .

. . . . . . . .

. . . . .

Vv08g1788 Tc05_g016360 . . . . .

. . . . . . . .

. . . . . .

Vv08g1789 . . . . . . .

. . . . . . . .

. . . . .

Vv08g1790 Tc05_g016420 . . . . .

. . . . . . . .

. . . . . .

Vv08g1791 . . . . . . .

. . . . . . . .

. . . . .

Vv08g1792 Tc05_g016430 . . . . .

Vv13g1211 . . . . . .

. . . . . . .

Vv08g1793 Tc05_g016440 . . . . .
Vv13g1208 . . . . . .
. . . . . . .
Vv08g1794 Tc05_g016550 . . . . .
. . . . . . . .
. . . . . .
Vv08g1795 Tc05_g016610 . . . . .
. . . . . . . .
. . . . . .
Vv08g1796 . . . . . . .
. . . . . . . .
. . . . .
Vv08g1797 Tc05_g009270 . . . . .
. . . . . . . .
. . . . . .
Vv08g1798 . . . . . .
Vv13g1207 . . . . . . .
. . . . . .
Vv08g1799 Tc05_g016710 . . . . .
Vv13g1197 . . . . . .
. . . . . . .
Vv08g1800 Tc05_g016720 . . . . .
. . . . . . . .
. . . . . .
Vv08g1801 . . . . . . .
. . . . . . . .
. . . . .
Vv08g1802 Tc05_g016730 . . . . .
. . . . . . . .
. . . . . .
Vv08g1803 Tc05_g016740 . . . . .
. . . . . . . .
. . . . . .
Vv08g1804 . . . . . . .
. . . . . . . .
. . . . .
Vv08g1805 . . . . . . .
. . . . . . . .
. . . . .
Vv08g1806 Tc05_g026230 . . . . .
. . . . . . . .
. . . . . .
Vv08g1807 . . . . . . .
. . . . . . . .
. . . . .
Vv08g1808 . . . . . . .
. . . . . . . .
. . . . .
Vv08g1809 . . . . . . .
. . . . . . . .

. . . . .
Vv08g1810 . . . . . . .
. . . . . . . .
. . . . .
Vv08g1811 . . . . . . .
. . . . . . . .
. . . . .
Vv08g1812 Tc05_g016790 . . . . .
. . . . . . . .
. . . . . .
Vv08g1813 . . Gorai.002G230700.1 . .
Gorai.007G360500.1 . . . . .
. . . . . . . .
.
Vv08g1814 . . . . . . .
. . . . . . . .
. . . . .
Vv08g1815 Tc05_g026240 . . . . .
. . . . . . . .
. . . . . .
Vv08g1816 Tc05_g016970 . . . . .
. . . . . . . .
. . . . . .
Vv08g1817 Tc05_g016980 . . . . .
. . . . . . . .
. . . . . .
Vv08g1818 Tc05_g017060 . . . . .
. . . . . . . .
. . . . . .
Vv08g1819 Tc05_g017140 . . . . .
. . . . . . . .
. . . . . .
Vv08g1820 Tc05_g017160 . . . . .
. . . . . . . .
. . . . . .
Vv08g1821 Tc05_g017170 . . . .
Gorai.007G360800.1 . . . . . .
. . . . . . . .
Vv08g1822 Tc05_g009380 Gorai.006G153900.1 . .
. . . . . . . .
. . . . . . . .
Vv08g1823 Tc05_g017210 . Gorai.002G226000.1 .
. . . . . . . .
. . . . . . . .
Vv08g1824 . . . . . . .
. . . . . . . .
. . . . .
Vv08g1825 . . . . . .
Vv13g1187 . . . . . . .
. . . . . .

Vv08g1826 . . . . . . .
. . . . . . . .
. . . . .
Vv08g1827 . . . . . . .
. . . . . . . .
. . . . .
Vv08g1828 Tc05_g017220 . . . . .
. . . . . . . .
. . . . . .
Vv08g1829 . . . . . . .
. . . . . . . .
. . . . .
Vv08g1830 Tc05_g017270 . . . . .
. . . . . . . .
. . . . . .
Vv08g1831 . . . . . . .
. . . . . . . .
. . . . .
Vv08g1832 Tc05_g017310 . . . . .
. . . . . . . .
. . . . . .
Vv08g1833 Tc05_g017320 . . . . .
. . . . . . . .
. . . . . .
Vv08g1834 Tc05_g017330 . . . . .
. . . . . . . .
. . . . . .
Vv08g1835 Tc05_g017350 . . . . .
. . . . . . . .
. . . . . .
Vv08g1836 . . . . . . .
. . . . . . . .
. . . . .
Vv08g1837 Tc05_g017360 . . . Gorai.
007G357300.1 Gorai.007G357300.1 . . . .
. . . . . . . .
. .
Vv08g1838 Tc05_g017370 . . . . .
. . . . . . . .
. . . . . .
Vv08g1839 . . . . . . .
. . . . . . . .
. . . . .
Vv08g1840 . . . . . . .
. . . . . . . .
. . . . .
Vv08g1841 . . . . . . .
. . . . . . . .
. . . . .
Vv08g1842 . . . . . . .

| | | | | | | | |
|---|---|---|---|---|---|---|---|
| . | . | . | . | . | . | . | . |
| . | . | . | . | . | | | |
| Vv08g1843 | . | . | . | . | . | . | . |
| . | . | . | . | . | . | . | . |
| . | . | . | . | . | | | |
| Vv08g1844 | . | . | . | . | . | . | . |
| . | . | . | . | . | . | . | . |
| . | . | . | . | . | | | |
| Vv08g1845 | Tc05_g017380 | | . | . | . | . | . |
| . | . | . | . | . | . | . | . |
| . | . | . | . | . | . | | |
| Vv08g1846 | Tc05_g017390 | | . | . | . | . | . |
| . | . | . | . | . | . | . | . |
| . | . | . | . | . | . | | |
| Vv08g1847 | . | . | . | . | . | . | . |
| . | . | . | . | . | . | . | . |
| . | . | . | . | . | | | |
| Vv08g1848 | Tc05_g017440 | | . | . | . | . | . |
| . | . | . | . | . | . | . | . |
| . | . | . | . | . | . | | |
| Vv08g1849 | . | . | . | . | . | . | . |
| . | . | . | . | . | . | . | . |
| . | . | . | . | . | | | |
| Vv08g1850 | Tc05_g017460 | | . | . | . | . | . |
| . | . | . | . | . | . | . | . |
| . | . | . | . | . | . | | |
| Vv08g1851 | . | . | . | . | . | . | . |
| . | . | . | . | . | . | . | . |
| . | . | . | . | . | | | |
| Vv08g1852 | . | . | . | . | . | . | . |
| . | . | . | . | . | . | . | . |
| . | . | . | . | . | | | |
| Vv08g1853 | . | . | . | . | . | . | . |
| . | . | . | . | . | . | . | . |
| . | . | . | . | . | | | |
| Vv08g1854 | Tc05_g018000 | | . | . | . | . | . |
| . | . | . | . | . | . | . | . |
| . | . | . | . | . | . | | |
| Vv08g1855 | . | . | . | . | . | . | . |
| . | . | . | . | . | . | . | . |
| . | . | . | . | . | | | |
| Vv08g1856 | . | . | . | . | . | . | . |
| . | . | . | . | . | . | . | . |
| . | . | . | . | . | | | |
| Vv08g1857 | . | . | . | . | . | . | . |
| . | . | . | . | . | . | . | . |
| . | . | . | . | . | | | |
| Vv08g1858 | . | . | . | . | . | . | . |
| . | . | . | . | . | . | . | . |
| . | . | . | . | . | | | |

```
Vv08g1859       .       .       .       .       .       .       .
        .       .       .       .       .       .       .       .
        .       .       .       .       .
Vv08g1860       .       .       .       .       .       .       .
        .       .       .       .       .       .       .       .
        .       .       .       .       .
Vv08g1861       .       .       .       .       .       .       .
        .       .       .       .       .       .       .       .
        .       .       .       .       .
Vv08g1862       .       .       .       .       .       .       .
        .       .       .       .       .       .       .       .
        .       .       .       .       .
Vv08g1863       .       .       .       .       .       .       .
        .       .       .       .       .       .       .       .
        .       .       .       .       .
Vv08g1864       .       .       .       .       .       .       .
        .       .       .       .       .       .       .       .
        .       .       .       .       .
Vv08g1865       .       .       .       .       .       .       .
        .       .       .       .       .       .       .       .
        .       .       .       .       .
Vv08g1866       .       .       .       .       .       .       .
        .       .       .       .       .       .       .       .
        .       .       .       .       .
Vv08g1867       .       .       .       .       .       .       .
        .       .       .       .       .       .       .       .
        .       .       .       .       .
Vv09g0001       .       .       .       .       .       .       .
        .       .       .       .       .       .       .       .
        .       .       .       .       .
Vv09g0002       .       .       .       .       .       .       .
        .       .       .       .       .       .       .       .
        .       .       .       .       .
Vv09g0003       Tc06_g010930    .       Gorai.010G172400.1      .
        Gorai.011G053800.1      .       .       .       .       .
        .       .       .       .       .       .       .       .
        .       .
Vv09g0004       .       .       .       .       .       .       .
        .       .       .       .       .       .       .       .
        .       .       .       .       .
Vv09g0005       Tc06_g010910    .       .       .       Gorai.
011G053900.1    .       .       .       .       .       .       .
        .       .       .       .       .       .       .       .
Vv09g0006       Tc06_g010900    Gorai.006G090900.1      .       .
        Gorai.011G054000.1      .       .       .       .       .
        .       .       .       .       .       .       .       .
        .       .
Vv09g0007       Tc06_g010890    .       Gorai.010G172500.1      .
        Gorai.011G054100.1      .       .       .       .       .
        .       .       .       .       .       .       .       .
```

.	.
Vv09g0008	Tc06_g010880	.	.	.	.	.
.	.	.	.	.	.	.	.
.	.	.	.	.	.
Vv09g0009	Tc06_g010860	.	Gorai.010G236300.1	.
Gorai.011G054300.1	.	.	.	.	.
.	.	.	.	.	.	.	.
.	.
Vv09g0010	Tc06_g010850	Gorai.006G090800.1	.	.
Gorai.011G054400.1	.	.	.	.	.
.	.	.	.	.	.	.	.
.	.
Vv09g0011	.	.	.	.	.	.	.
.	.	.	.	.	.	.	.
.	.	.	.	.
Vv09g0012	Tc06_g010830	.	.	.	Gorai.
011G054500.1	.	.	.	.	.	.	.
.	.	.	.	.	.	.	.
Vv09g0013	Tc06_g010820	.	.	.	Gorai.
011G054800.1	.	.	.	.	.	.	.
.	.	.	.	.	.	.	.
Vv09g0014	.	.	.	.	.	.	.
.	.	.	.	.	.	.	.
.	.	.	.	.
Vv09g0015	.	.	.	.	.	.	.
.	.	.	.	.	.	.	.
.	.	.	.	.
Vv09g0016	Tc06_g010790	.	.	.	.	.
.	.	.	.	.	.	.	.
.	.	.	.	.	.
Vv09g0017	.	.	.	.	.	.	.
.	.	.	.	.	.	.	.
.	.	.	.	.
Vv09g0018	.	.	.	.	.	.	.
.	.	.	.	.	.	.	.
.	.	.	.	.
Vv09g0019	.	.	.	.	.	.
Vv11g0016	Tc09_g006150	.	.	.	Gorai.
009G039100.1	.	.	.	.	.	.	.
.
Vv09g0020	Tc06_g010770	.	.	.	Gorai.
011G055100.1	.	Vv11g0017	Tc09_g006160	.	.
.	.	.	.	.	.	.	.
.	.
Vv09g0021	Tc06_g010760	.	.	.	Gorai.
011G055200.1	.	.	.	.	.	.	.
.	.	.	.	.	.	.	.
Vv09g0022	Tc06_g010750	Gorai.006G090500.1	.	.
Gorai.011G055300.1	.	.	.	.	.
.	.	.	.	.	.	.	.

. .
Vv09g0023 . . . . . . .
. . . . . . . .
. . . . .
Vv09g0024 Tc06_g010740 . . . Gorai.
011G055400.1 . . . . . . .
. . . . . . . .
Vv09g0025 Tc06_g010730 Gorai.006G090400.1 . .
Gorai.011G055500.1 . . . . .
. . . . . . . .
. .
Vv09g0026 Tc06_g010720 Gorai.006G090300.1 . .
. . Vv11g0023 Tc09_g006220 .
Gorai.001G018100.1 . Gorai.009G038700.1 . .
. . . . . .
Vv09g0027 . . . . Gorai.011G055600.1
. Vv11g0027 Tc09_g006260 Gorai.013G222000.1
. . Gorai.009G038300.1 . . .
. . . . .
Vv09g0028 Tc06_g010710 . . . . .
. . . . . . . .
. . . . . .
Vv09g0029 Tc06_g010700 . Gorai.010G236200.1 .
. . . . . . . .
. . . . . . . .
Vv09g0030 . . . . . . .
. . . . . . . .
. . . . .
Vv09g0031 Tc06_g010670 . . . Gorai.
011G055700.1 . . . . . . .
. . . . . . . .
Vv09g0032 . . . . . . .
. . . . . . . .
. . . . .
Vv09g0033 Tc06_g010630 . . . . .
. . . . . . . .
. . . . . .
Vv09g0034 Tc06_g010620 . Gorai.010G236100.1 .
Gorai.011G056300.1 . . . . .
. . . . . . . .
. .
Vv09g0035 Tc06_g010600 . . . . .
. . . . . . . .
. . . . . .
Vv09g0036 Tc06_g010590 . . . . .
. . . . . . . .
. . . . . .
Vv09g0037 Tc06_g010570 . . . . .
. . . . . . . .
. . . . . .

Vv09g0038 Tc06_g010560 . . . . .
. . . . . . . .
. . . . . .
Vv09g0039 . . . . . . .
. . . . . . . .
. . . . .
Vv09g0040 Tc06_g010540 . . . . .
. . . . . . . .
. . . . . .
Vv09g0041 . . . . . . .
. . . . . . . .
. . . . .
Vv09g0042 Tc06_g010530 . . . . .
. . . . . . . .
. . . . . .
Vv09g0043 Tc06_g010520 . Gorai.010G235900.1 .
Gorai.011G056600.1 . Vv11g0030
Tc09_g006300 Gorai.013G222100.1 Gorai.001G017800.1 .
. Gorai.010G092400.1 . . . .
. . .
Vv09g0044 . . . . . . .
. . . . . . . .
. . . . .
Vv09g0045 Tc06_g010510 . . . Gorai.
011G056700.1 . . . . . . .
. . . . . . . .
Vv09g0046 . . . . . . .
. . . . . . . .
. . . . .
Vv09g0047 Tc06_g010500 . . . Gorai.
011G056800.1 . . . . . . .
. . . . . . . .
Vv09g0048 Tc06_g010490 . . . . .
. . . . . . . .
. . . . . .
Vv09g0049 Tc06_g010480 . . . Gorai.
011G056900.1 . Vv11g0040 Tc09_g006350 Gorai.
013G222500.1 Gorai.001G017600.1 . Gorai.009G037800.1
Gorai.010G092200.1 . . . . .
. .
Vv09g0050 Tc06_g010460 . . . . .
. . . . . . . .
. . . . . .
Vv09g0051 Tc06_g010450 . . . Gorai.
011G057100.1 . . . . . . .
. . . . . . . .
Vv09g0052 . . . . . . .
. . . . . . . .
. . . . .
Vv09g0053 . . . . . . .

```
. . . . . . . .
. . . . .
Vv09g0054 Tc06_g010430 . Gorai.010G235700.1 .
. . . . . . . .
. . . . . . . .
Vv09g0055 . . . . . . .
. . . . . . . .
. . . . .
Vv09g0056 Tc06_g010420 . . . Gorai.
011G057200.1 . . . . . . .
. . . . . . . .
Vv09g0057 Tc06_g010410 . . . Gorai.
011G057300.1 . . . . . . .
. . . . . . . .
Vv09g0058 Tc06_g010400 . . . Gorai.
011G057400.1 . . . . . . .
. . . . . . . .
Vv09g0059 Tc06_g010390 . Gorai.010G235600.1 .
. . . . . . . .
. . . . . . . .
Vv09g0060 Tc06_g010380 . . . . .
. . . . . . . .
. . . . . .
Vv09g0061 Tc06_g010360 Gorai.006G090100.1 . .
. . Vv11g0050 Tc09_g006480 . .
. . . . . . . .
. .
Vv09g0062 . . . . . . .
. . . . . . . .
. . . . .
Vv09g0063 Tc06_g010330 . . . . .
. . . . . . . .
. . . . . .
Vv09g0064 . . . . . . .
. . . . . . . .
. . . . .
Vv09g0065 Tc06_g010320 . . . . .
. . . . . . . .
. . . . . .
Vv09g0066 Tc06_g010310 . . . . .
. . . . . . . .
. . . . . .
Vv09g0067 Tc06_g010300 . . . . .
. . . . . . . .
. . . . . .
Vv09g0068 . . . . . . .
. . . . . . Vv04g0248
. . Gorai.001G001600.1 . Gorai.
009G068300.1 .
Vv09g0069 . . . . . . .
```

.	.	.	.	.	.	.	.
.	.	.	.	.
Vv09g0070	Tc06_g010230	.	Gorai.010G235500.1	.
.	.	.	.	.	.	.	.
.	.	.	.	.	.	.	.
Vv09g0071	Tc06_g010220	.	Gorai.010G235400.1	.
.	.	.	.	.	.	.	.
.	.	.	.	.	.	.	.
Vv09g0072	.	.	.	.	.	.	.
.	.	.	.	.	.	.	.
.	.	.	.	.
Vv09g0073	Tc06_g010210	.	.	.	.	.
Vv11g0061	Tc09_g006620	.	.	.
Gorai.009G036800.1	Gorai.010G090600.1	.	.	.
.	.	.	.
Vv09g0074	Tc06_g010180	.	Gorai.010G235300.1	.
.	.	.	.	.	.	.	.
.	.	.	.	.	.	.	.
Vv09g0075	Tc06_g010170	.	.	.	.	.
.	.	.	.	.	.	.
Vv04g0236	.	.	.	.	.	.
Vv09g0076	Tc06_g010160	.	.	.	.	.
.	.	.	.	.	.	.	.
.	.	.	.	.	.
Vv09g0077	Tc06_g010150	.	.	.	.	.
Vv11g0069	Tc09_g006690	.	Gorai.001G016100.1
.	Gorai.009G036400.1	Gorai.010G090100.1	.
.	.	.	.	.	.
Vv09g0078	Tc06_g010140	.	.	.	.	.
Vv11g0070	Tc09_g006700	.	.	.	.
Gorai.010G090000.1	.	.	.	.	.
.	.
Vv09g0079	.	.	.	.	.	.	.
.	.	.	.	.	.	.	.
.	.	.	.	.
Vv09g0080	.	.	.	.	.	.
Vv11g0072	Tc09_g006720	Gorai.013G222900.1	Gorai.
001G016000.1	.	.	Gorai.010G089800.1	.	.
.	.	.	.	.
Vv09g0081	.	.	.	.	.	.	.
.	.	.	.	.	.	.	.
.	.	.	.	.
Vv09g0082	.	.	.	.	.	.	.
.	.	.	.	.	.	.	.
.	.	.	.	.
Vv09g0083	.	.	.	.	.	.	.
.	.	.	.	.	.	.	.
.	.	.	.	.
Vv09g0084	.	.	.	.	.	.	.
.	.	.	.	.	.	.	.

| | | | | | | | |
|---|---|---|---|---|---|---|---|
| . | . | . | . | . | | | |
| Vv09g0085 | . | . | . | . | . | . | |
| Vv11g0082 | . | . | . | . | . | . | . |
| . | . | . | . | . | . | | |
| Vv09g0086 | . | . | . | . | . | . | |
| Vv11g0084 | Tc09_g006790 | | Gorai.013G223600.1 | | | . | . |
| . | . | . | . | . | . | . | . |
| . | | | | | | | |
| Vv09g0087 | Tc06_g000810 | | . | . | Gorai.009G116500.1 | | |
| . | . | . | . | . | . | . | . |
| . | . | . | . | . | . | . | . |
| Vv09g0088 | . | . | . | . | . | . | . |
| . | . | . | . | . | . | . | . |
| . | . | . | . | . | | | |
| Vv09g0089 | . | . | . | . | . | . | . |
| . | . | . | . | . | . | . | . |
| . | . | . | . | . | | | |
| Vv09g0090 | . | . | . | . | . | . | . |
| . | . | . | . | . | . | . | . |
| . | . | . | . | . | | | |
| Vv09g0091 | . | . | . | . | . | . | . |
| . | . | . | . | . | . | . | . |
| . | . | . | . | . | | | |
| Vv09g0092 | . | . | . | . | . | . | . |
| . | . | . | . | . | . | . | . |
| . | . | . | . | . | | | |
| Vv09g0093 | . | . | . | . | . | . | . |
| . | . | . | . | . | . | . | . |
| . | . | . | . | . | | | |
| Vv09g0094 | . | . | . | . | . | . | . |
| . | . | . | . | . | . | . | . |
| . | . | . | . | . | | | |
| Vv09g0095 | . | . | . | . | Gorai.011G059700.1 | | |
| . | . | . | . | . | . | . | . |
| . | . | . | . | . | . | . | |
| Vv09g0096 | Tc06_g000510 | | . | . | Gorai.009G115900.1 | | |
| . | . | . | . | . | . | . | . |
| . | . | . | . | . | . | . | . |
| Vv09g0097 | . | . | . | . | . | . | . |
| . | . | . | . | . | . | . | . |
| . | . | . | . | . | | | |
| Vv09g0098 | . | . | . | . | . | . | . |
| . | . | . | . | . | . | . | . |
| . | . | . | . | . | | | |
| Vv09g0099 | . | . | . | . | . | . | . |
| . | . | . | . | . | . | . | . |
| . | . | . | . | . | | | |
| Vv09g0100 | . | . | . | . | . | . | . |
| . | . | . | . | . | . | . | . |
| . | . | . | . | . | | | |

```
Vv09g0101       .       .       .       .       .       .       .
        .       .       .       .       .       .       .       .
        .       .       .       .       .
Vv09g0102       .       .       Gorai.010G235200.1      .       .
        .       .       .       .       .       .       .       .
        Vv04g0207       .       .       .       .       .       .
Vv09g0103       .       .       .       .       .       .       .
        .       .       .       .       .       .       .       .
        .       .       .       .       .
Vv09g0104       .       .       .       .       .       .       .
        .       .       .       .       .       .       .       .
        .       .       .       .       .
Vv09g0105       Tc06_g010130    .       .       .       .       .
        Vv05g1533       .       .       .       .       .       .
        .       .       .       .       .       .       .
Vv09g0106       .       .       .       .       .       .
Vv05g1519       .       .       .       .       .       .       .
        .       .       .       .       .       .
Vv09g0107       .       .       .       .       .       .
Vv05g1518       .       .       .       .       .       .       .
        .       .       .       .       .       .
Vv09g0108       .       .       .       .       .       .
Vv05g1513       .       .       Gorai.005G039700.1      .       .
        .       .       .       .       .       .       .       .
Vv09g0109       .       .       .       .       .       .       .
        .       .       .       .       .       .       .       .
        .       .       .       .       .
Vv09g0110       .       .       .       .       .       .
Vv05g1510       .       .       Gorai.005G039800.1      .       .
        .       .       .       .       .       .       .       .
Vv09g0111       .       .       .       .       .       .       .
        .       .       .       .       .       .       .       .
        .       .       .       .       .
Vv09g0112       .       .       .       .       .       .
Vv05g1505       .       .       Gorai.005G039900.1      .       .
        .       .       .       .       .       .       .       .
Vv09g0113       .       .       .       .       .       .
Vv05g1503       .       .       Gorai.005G040000.1      .       .
        .       .       .       .       .       .       .       .
Vv09g0114       Tc06_g000370    .       .       .       .       .
        Vv11g0115       Tc09_g007080    .       Gorai.001G015400.1
        .       Gorai.009G035500.1      .       .       .       .
        .       .       .       .
Vv09g0115       .       .       .       .       .       .       .
        .       .       .       .       .       .       .       .
        .       .       .       .       .
Vv09g0116       .       .       .       .       .       .
Vv11g0118       Tc09_g007110    Gorai.013G225300.1      .       .
        Gorai.009G035200.1      .       .       .       .       .
        .       .       .
```

Vv09g0117 . . . . . . .
. . . . . . . .
. . . . .
Vv09g0118 . . . . . . .
. . . . . . . .
. . . . .
Vv09g0119 . . . . . . .
. . . . . . . .
. . . . .
Vv09g0120 . . . . . . .
. . . . . . . .
. . . . .
Vv09g0121 Tc06_g009780 . . . . .
. . . . . . . .
. . . . . .
Vv09g0122 Tc06_g009740 . . . . .
. . . . . . . .
. . . . . .
Vv09g0123 . . . . . . .
. . . . . . . .
. . . . .
Vv09g0124 . . . . . . .
. . . . . . . .
. . . . .
Vv09g0125 . . . . . . .
. . . . . . . .
. . . . .
Vv09g0126 . . . . . . .
. . . . . . . .
. . . . .
Vv09g0127 . . . . . . .
. . . . . . . .
. . . . .
Vv09g0128 . . . . . .
Vv11g0134 Tc09_g007200 . Gorai.001G014700.1 .
Gorai.009G041200.1 . Vv04g0174 . .
Gorai.001G020800.1 . . .
Vv09g0129 . . . . . . .
. . . . . . . .
. . . . .
Vv09g0130 . . . . . . .
. . . . . . . .
. . . . .
Vv09g0131 . . Gorai.010G234400.1 . .
. . . . . . . .
. . . . . . .
Vv09g0132 . . . . . .
Vv11g0141 Tc09_g007270 . . . Gorai.
009G041500.1 Gorai.010G086600.1 Vv04g0169 . .
Gorai.001G021000.1 . . .

Vv09g0133 . . . . . . .
. . . . . . . .
. . . . .
Vv09g0134 . . . . . .
Vv11g0142 Tc09_g007280 Gorai.013G226100.1 . .
Gorai.009G034500.1 Gorai.010G086500.1 . .
. . . . .
Vv09g0135 Tc06_g011930 Gorai.006G095300.1 Gorai.
010G234300.1 . . . Vv11g0143
Tc09_g007290 . . . . Gorai.010G086400.1
. . . . . . .
Vv09g0136 . . Gorai.010G234100.1 . .
. . . . . . . .
. . . . . . .
Vv09g0137 Tc06_g011630 . . . . .
. . . . . . . .
. . . . . .
Vv09g0138 . . . . . . .
. . . . . . . .
. . . . .
Vv09g0139 . . . . . . .
. . . . . . . .
. . . . .
Vv09g0140 . . . . . .
Vv11g0146 Tc09_g007320 Gorai.013G226400.1 . .
Gorai.009G041700.1 Gorai.010G086100.1 Vv04g0166
. Gorai.013G226400.1 Gorai.001G021300.1 .
. .
Vv09g0141 . . . . . . .
. . . . . . . .
. . . . .
Vv09g0142 . . . . Gorai.011G062600.1
. . . . . . . .
. . . . . . .
Vv09g0143 . . . . . . .
. . . . . . . .
. . . . .
Vv09g0144 . . . . . . .
. . . . . . . .
. . . . .
Vv09g0145 . . . . . . .
. . . . . . . .
. . . . .
Vv09g0146 . . . . . . .
. . . . . . . .
. . . . .
Vv09g0147 . . . . . . .
. . . . . . . .
. . . . .
Vv09g0148 . . . . . . .

| | | | | | | | |
|---|---|---|---|---|---|---|---|
| Vv09g0149 | . | . | . | . | . | . | . |
| Vv09g0150 | . | . | . | . | . | . | . |
| Vv09g0151 | . | . | . | . | . | . | . |
| Vv09g0152 | . | . | . | . | . | . | . |
| Vv09g0153 | . | . | . | . | . | . | . |
| Vv09g0154 | . | . | Gorai.010G232800.1 | | | . | . |
| Vv09g0155 | . | . | . | . | . | . | . |
| Vv09g0156 | . | . | . | . | . | . | . |
| Vv09g0157 | Tc06_g019130 | | . | . | . | . | . |
| Vv09g0158 | . | . | . | . | . | . | . |
| Vv09g0159 | Tc06_g009410 | | . | . | . | . | . |
| Vv09g0160 | . | . | . | . | . | . | . |
| Vv09g0161 | . | . | . | . | . | . | . |
| Vv09g0162 | . | . | . | . | . | . | . |
| Vv09g0163 | . | . | . | . | . | . | . |
| Vv09g0164 | . | . | . | . | . | . | . |

Vv09g0165 Tc06_g005930 . . Gorai.009G119900.1
. . . . . . . .
. . . . . . . .
Vv09g0166 . . . . . . .
. . . . . . . .
. . . . .
Vv09g0167 . . . . . . .
. . . . . . . .
. . . . .
Vv09g0168 . . . . . . .
. . . . . . . .
. . . . .
Vv09g0169 . . . . . . .
. . . . . . . .
. . . . .
Vv09g0170 . . . . . . .
. . . . . . Vv04g0141
. Gorai.013G220700.1 Gorai.001G021800.1 .
. .
Vv09g0171 . . . . . .
Vv11g0164 . . . . . . .
. . . . . .
Vv09g0172 Tc06_g018970 . . . Gorai.
011G062700.1 . . . . . . .
. . . . . . . .
Vv09g0173 . . . . . . .
. . . . . . . .
. . . . .
Vv09g0174 . . . . . . .
. . . . . . . .
. . . . .
Vv09g0175 . . . . . . .
. . . . . . . .
. . . . .
Vv09g0176 . . . . . . .
. . . . . . . .
. . . . .
Vv09g0177 . . . . . . .
. . . . . . . .
. . . . .
Vv09g0178 . . . . . . .
. . . . . . . .
. . . . .
Vv09g0179 . . . . . . .
. . . . . . . .
. . . . .
Vv09g0180 . . . . . . .
. . . . . . . .
. . . . .
Vv09g0181 Tc06_g009200 . . . . .

. . . . . . . .
. . . . . .
Vv09g0182 . . . . . . .
. . . . . . . .
. . . . .
Vv09g0183 Tc06_g018960 . . . . .
. . . . . . . .
. . . . . .
Vv09g0184 . . . . . . .
. . . . . . . .
. . . . .
Vv09g0185 Tc06_g009090 . . . Gorai.
011G063400.1 . Vv11g0188 Tc09_g007590 . .
. . . . . . . .
. .
Vv09g0186 . . . . . . .
. . . . . . . .
. . . . .
Vv09g0187 . . . . . . .
. . . . . . . .
. . . . .
Vv09g0188 . . . . . . .
. . . . . . . .
. . . . .
Vv09g0189 . . . . . . .
. . . . . . . .
. . . . .
Vv09g0190 . . . . . . .
. . . . . . . .
. . . . .
Vv09g0191 . . . . . . .
. . . . . . . .
. . . . .
Vv09g0192 . . . . . .
Vv11g0217 Tc09_g007860 . . . Gorai.
009G031600.1 Gorai.010G083100.1 . . . .
. . .
Vv09g0193 . . . . . . .
. . . . . . . .
. . . . .
Vv09g0194 . . . . . . .
. . . . . . . .
. . . . .
Vv09g0195 . . . . . . .
. . . . . . . .
. . . . .
Vv09g0196 . . . . . . .
. . . . . . . .
. . . . .
Vv09g0197 Tc06_g008950 . Gorai.010G232400.1 .

```
        .       .       .       .       .       .       .       .
        .       .       .       .       .       .       .       .
Vv09g0198       Tc06_g008940    .       .       .       Gorai.
011G064300.1    .       .       .       .       .       .       .
        .       .       .       .       .       .       .       .
Vv09g0199       Tc06_g008930    .       .       .       .       .
        .       .       .       .       .       .       .       .
        .       .       .       .       .       .
Vv09g0200       Tc06_g008920    .       Gorai.010G232300.1      .
        Gorai.011G064400.1      .       .       .       .       .
        .       .       .       .       .       .       .       .
        .       .
Vv09g0201       Tc06_g008910    .       .       .       .       .
        .       .       .       .       .       .       .       .
        .       .       .       .       .       .
Vv09g0202       .       .       .       .       .       .       .
        .       .       .       .       .       .       .       .
        .       .       .       .       .
Vv09g0203       Tc06_g006270    .       .       Gorai.009G118300.1
        .       .       .       .       .       .       .       .
        .       .       .       .       .       .       .       .
Vv09g0204       Tc06_g008870    .       .       .       Gorai.
011G064600.1    .       .       .       .       .       .       .
        .       .       .       .       .       .       .       .
Vv09g0205       .       .       .       .       .       .       .
        .       .       .       .       .       .       .       .
        .       .       .       .       .
Vv09g0206       Tc06_g008850    .       .       .       .       .
        Vv11g0221       Tc09_g007920    .       Gorai.001G011600.1
        .       .       Gorai.010G082900.1      .       .       .
        .       .       .       .
Vv09g0207       Tc06_g008840    .       .       .       Gorai.
011G064700.1    .       .       .       .       .       .       .
        .       Vv04g0113       Tc01_g029920    .       .       .
        .       Gorai.011G064700.1
Vv09g0208       Tc06_g008830    .       .       .       Gorai.
011G064800.1    .       .       .       .       .       .       .
        .       .       .       .       .       .       .       .
Vv09g0209       .       .       .       .       Gorai.011G064900.1
        .       .       .       .       .       .       .       .
        Vv04g0111       .       .       .       .       Gorai.
003G057000.1    .
Vv09g0210       .       .       .       .       .       .       .
        .       .       .       .       .       .       .       .
        .       .       .       .       .
Vv09g0211       .       .       .       .       Gorai.011G065100.1
        .       .       .       .       .       .       .       .
        .       .       .       .       .       .       .
Vv09g0212       .       .       .       .       .       .       .
        .       .       .       .       .       .       .       .
```

```
        .        .       .        .       .
Vv09g0213        Tc06_g006880     Gorai.006G094600.1       .        .
        .        .       .        .       .        .       .        .
        .        .       .        .       .        .       .        .
Vv09g0214        .       .        .       .        .       .        .
        .        .       .        .       .        .       .        .
        .        .       .        .       .
Vv09g0215        .       .        .       .        .       .        .
        .        .       .        .       .        .       .        .
        .        .       .        .       .
Vv09g0216        .       .        .       .        Gorai.011G066300.1
        .        Vv11g0236        Tc09_g008010     Gorai.013G227900.1
        .        Gorai.004G284100.1       .        .       .        .
        .        .       .        .       .
Vv09g0217        .       .        .       .        Gorai.011G066400.1
        .        .       .        .       .        .       .        .
        .        .       .        .       .        .       .
Vv09g0218        .       .        .       .        .       .        .
        .        .       .        .       .        .       .        .
        .        .       .        .       .
Vv09g0219        .       .        .       .        .       .        .
        .        .       .        .       .        .       .        .
        .        .       .        .       .
Vv09g0220        .       .        .       .        .       .        .
        .        .       .        .       .        .       .        .
        .        .       .        .       .
Vv09g0221        .       .        .       .        .       .        .
        .        .       .        .       .        .       .        .
        .        .       .        .       .
Vv09g0222        .       .        .       .        Gorai.011G067000.1
        .        .       .        .       .        .       .        .
        .        .       .        .       .        .       .
Vv09g0223        .       .        .       .        Gorai.011G067300.1
        .        Vv11g0250        Tc09_g008190     .       .        .
        Gorai.009G030700.1        .       Vv04g0087       .        .
        .        .       .        Gorai.011G067300.1
Vv09g0224        .       .        .       .        .       .        .
        .        .       .        .       .        .       .        .
        .        .       .        .       .
Vv09g0225        .       .        .       .        Gorai.011G067400.1
        .        Vv11g0258        .       .        Gorai.001G023300.1
        .        Gorai.009G046000.1       .        Vv04g0083        .
        .        Gorai.001G023300.1       .        Gorai.003G060000.1
        Gorai.011G067400.1
Vv09g0226        .       .        .       .        .       .        .
        .        .       .        .       .        .       .        .
        .        .       .        .       .
Vv09g0227        Tc06_g014380     .       .        .       .        .
        .        .       .        .       .        .       .        .
        .        .       .        .       .        .
```

```
Vv09g0228       .       .       .       .       .       .       .
        .       .       .       .       .       .       .       .
        .       .       .       .       .
Vv09g0229       .       .       .       .       .       .       .
        .       .       .       .       .       .       .       .
        .       .       .       .       .
Vv09g0230       .       .       .       .       .       .       .
        .       .       .       .       .       .       .       .
        .       .       .       .       .
Vv09g0231       .       .       .       .       Gorai.011G067700.1
        .       .       .       .       .       .       .       .
        .       .       .       .       .       .       .
Vv09g0232       .       .       .       .       .       .       .
        .       .       .       .       .       .       .       .
        .       .       .       .       .
Vv09g0233       .       .       .       .       Gorai.011G067800.1
        .       .       .       .       .       .       .       .
        .       .       .       .       .       .       .
Vv09g0234       .       .       Gorai.010G231600.1      .
Gorai.011G067900.1      .       .       .       .       .       .
        .       .       Vv04g0078       .       .       .       .
        .       Gorai.011G067900.1
Vv09g0235       .       .       .       .       Gorai.011G068100.1
        .       .       .       .       .       .       .       .
        .       .       .       .       .       .       .
Vv09g0236       .       .       Gorai.010G231400.1      .
Gorai.011G068200.1      .       .       .       .       .       .
        .       .       .       .       .       .       .       .
        .
Vv09g0237       .       .       Gorai.010G229100.1      .       .
        .       .       .       .       .       .       .       .
        .       .       .       .       .       .       .
Vv09g0238       .       .       .       .       Gorai.011G068300.1
        .       .       .       .       .       .       .       .
        .       .       .       .       .       .       .
Vv09g0239       .       .       .       .       Gorai.011G068400.1
        .       Vv11g0275       Tc09_g008440    .       .       .
        Gorai.009G029600.1      Gorai.010G080300.1      .       .
        .       .       .       .       .
Vv09g0240       .       .       .       .       .       .       .
        .       .       .       .       .       .       .       .
        .       .       .       .       .
Vv09g0241       .       .       .       .       .       .       .
        .       .       .       .       .       .       .       .
        .       .       .       .       .
Vv09g0242       .       .       .       .       .       .       .
        .       .       .       .       .       .       .       .
        .       .       .       .       .
Vv09g0243       .       .       .       .       .       .       .
        .       .       .       .       .       .       .       .
```

. . . . .
Vv09g0244 . . . . . . .
. . . . . . . .
. . . . .
Vv09g0245 . . . . Gorai.011G068900.1
. . . . . . . .
Vv04g0069 . . . . Gorai.
003G060300.1 Gorai.011G068900.1
Vv09g0246 . . . . Gorai.011G069100.1
. . . . . . . .
. . . . . . .
Vv09g0247 . . . . . . .
. . . . . . Vv04g0065
. . Gorai.001G024100.1 . . .
Vv09g0248 . . . . . . .
. . . . . . . .
. . . . .
Vv09g0249 . . . . . . .
. . . . . . . .
. . . . .
Vv09g0250 . . . . . . .
. . . . . . Vv04g0064
. . . . Gorai.003G060400.1 .
Vv09g0251 . . . . Gorai.011G069200.1
. . . . . . . .
. . . . . . .
Vv09g0252 . . . . . . .
. . . . . . . .
. . . . .
Vv09g0253 Tc06_g007020 . . Gorai.009G115900.1
. . . . . . . .
. . . . . . . .
Vv09g0254 . . . . . . .
. . . . . . . .
. . . . .
Vv09g0255 . . . . . . .
. . . . . . . .
. . . . .
Vv09g0256 . . . . . .
Vv11g0276 Tc09_g008450 Gorai.013G228900.1 Gorai.
001G010300.1 Gorai.004G283100.1 Gorai.009G029400.1
Gorai.010G080200.1 Vv04g0061 . . . .
. .
Vv09g0257 . . . . . . .
. . . . . . . .
. . . . .
Vv09g0258 . . . . . . .
. . . . . . . .
. . . . .
Vv09g0259 . . . . . . .

```
          .         .         .         .         .         .         .         .
          .         .         .         .         .
Vv09g0260           Tc06_g014130        .         .         .         .         .
          .         .         .         .         .         .         .         .
          .         .         .         .         .         .
Vv09g0261           .         .         .         .         .         .         .
          .         .         .         .         .         .         .         .
          .         .         .         .         .
Vv09g0262           .         .         .         .         .         .         .
          .         .         .         .         .         .         .         .
          .         .         .         .         .
Vv09g0263           Tc06_g014110        .         .         .         .         .
          .         .         .         .         .         .         .         .
          .         .         .         .         .         .
Vv09g0264           .         .         .         .         .         .         .
          .         .         .         .         .         .         .         .
          .         .         .         .         .
Vv09g0265           .         .         .         .         .         .         .
          .         .         .         .         .         .         .         .
          .         .         .         .         .
Vv09g0266           .         .         .         .         .         .         .
          .         .         .         .         .         .         .         .
          .         .         .         .         .
Vv09g0267           .         .         .         .         .         .         .
          .         .         .         .         .         .         .         .
          .         .         .         .         .
Vv09g0268           .         .         .         .         .         .         .
          .         .         .         .         .         .         .         .
          .         .         .         .         .
Vv09g0269           .         .         .         .         .         .         .
          .         .         .         .         .         .         .         .
          .         .         .         .         .
Vv09g0270           Tc06_g013970        .         .         .         .         .
          .         .         .         .         .         .         .         .
          .         .         .         .         .         .
Vv09g0271           .         .         .         .         .         .         .
          .         .         .         .         .         .         .         .
          .         .         .         .         .
Vv09g0272           .         .         .         .         .         .         .
          .         .         .         .         .         .         .         .
          .         .         .         .         .
Vv09g0273           .         .         .         .         .         .         .
          .         .         .         .         .         .         .         .
          .         .         .         .         .
Vv09g0274           Tc06_g013700        .         .         .         .         .
          .         .         .         .         .         .         .         .
          .         .         .         .         .         .
Vv09g0275           .         .         .         .         Gorai.011G069400.1
          .         .         .         .         .         .         .         .
          .         .         .         .         .         .         .
```

Vv09g0276 Tc06_g007030 . . . Gorai.
011G069500.1 . . . . . . .
. . . . . . . .
Vv09g0277 . . . . . . .
. . . . . . . .
. . . . .
Vv09g0278 . . . . . .
Vv11g0282 Tc09_g008510 . . . .
Gorai.010G079700.1 . . . . . .
.
Vv09g0279 . . . . . . .
. . . . . . . .
. . . . .
Vv09g0280 Tc06_g007080 . . . . .
. . . . . . . .
. . . . . .
Vv09g0281 . . . . . . .
. . . . . . . .
. . . . .
Vv09g0282 Tc06_g007100 . . . Gorai.
011G069900.1 . . . . . . .
. . . . . . . .
Vv09g0283 . . . . . . .
. . . . . . . .
. . . . .
Vv09g0284 Tc06_g000510 . . Gorai.009G115900.1
. . . . . . . .
. . . . . . . .
Vv09g0285 . . . . . . .
. . . . . . . .
. . . . .
Vv09g0286 Tc06_g007110 . . . Gorai.
011G070000.1 . . . . . . .
. . . . . . . .
Vv09g0287 . . . . . . .
. . . . . . . .
. . . . .
Vv09g0288 Tc06_g013690 . . . . .
. . . . . . . .
. . . . . .
Vv09g0289 . . . . . . .
. . . . . . . .
. . . . .
Vv09g0290 . . . . . . .
. . . . . . . .
. . . . .
Vv09g0291 . . . . . . .
. . . . . . . .
. . . . .
Vv09g0292 . . . . . . .

. . . . . . . .
. . . . .
Vv09g0293 . . . . . . .
. . . . . . . .
. . . . .
Vv09g0294 Tc06_g013560 . . . . .
. . . . . . . .
. . . . . .
Vv09g0295 . . . . . . .
. . . . . . . .
. . . . .
Vv09g0296 Tc06_g007120 . . . Gorai.
011G070100.1 . . . . . . .
. . . . . . . .
Vv09g0297 Tc06_g007130 . . . . .
. . . . . . . .
. . . . . .
Vv09g0298 . . . . . . .
. . . . . . . .
. . . . .
Vv09g0299 Tc06_g007150 . . . Gorai.
011G070300.1 . . . . . . .
. . . . . . . .
Vv09g0300 . . . . . . .
. . . . . . . .
. . . . .
Vv09g0301 Tc06_g007170 . . . . .
Vv11g0293 Tc09_g008600 Gorai.013G229300.1 .
Gorai.004G282800.1 . . . . .
. . . .
Vv09g0302 . . . . . . .
. . . . . . . .
. . . . .
Vv09g0303 . . . . . . .
. . . . . . . .
. . . . .
Vv09g0304 Tc06_g007180 . . . . .
Vv11g0297 Tc09_g008650 . . . .
Gorai.010G078600.1 . . . . .
. .
Vv09g0305 Tc06_g007190 . . . . .
. . . . . . . .
. . . . . .
Vv09g0306 Tc06_g007200 . . . . .
. . . . . . . .
. . . . . .
Vv09g0307 Tc06_g007230 . . . . .
Vv11g0301 Tc09_g008690 . . Gorai.
004G282400.1 Gorai.009G028400.1 Gorai.010G078400.1 .
. . . . . .

Vv09g0308 Tc06_g000590 . . . . .
. . . . . . . .
. . . . . .
Vv09g0309 . . . . . . .
. . . . . . . .
. . . . .
Vv09g0310 Tc06_g000660 . . . . .
. . . . . . . .
. . . . . .
Vv09g0311 . . . . . . .
. . . . . . . .
. . . . .
Vv09g0312 Tc06_g000670 . . . . .
. . . . . . . .
. . . . . .
Vv09g0313 Tc06_g007240 Gorai.006G096500.1 . .
. . . . . . . .
. . . . . . . .
Vv09g0314 Tc06_g007250 . . . Gorai.
011G070600.1 . . . . . . .
. . . . . . . .
Vv09g0315 Tc06_g007260 . . . . .
Vv11g0311 Tc09_g008800 . Gorai.001G009300.1
Gorai.004G282000.1 Gorai.009G027900.1 . .
. . . . . .
Vv09g0316 Tc06_g007300 Gorai.006G096700.1 . .
. . Vv11g0312 . . Gorai.
001G025700.1 . Gorai.009G048100.1 . . .
. . . . .
Vv09g0317 Tc06_g007320 Gorai.006G096800.1 . .
Gorai.011G070700.1 . Vv11g0314
Tc09_g008850 . . . . Gorai.010G077400.1
. . . . . . .
Vv09g0318 Tc06_g007330 . Gorai.010G231100.1 .
Gorai.011G070800.1 . . . . .
. . . . . . . .
. .
Vv09g0319 . . . . . . .
. . . . . . . .
. . . . .
Vv09g0320 Tc06_g007340 . . . . .
. . . . . . . .
. . . . . .
Vv09g0321 . . . . . . .
. . . . . . . .
. . . . .
Vv09g0322 . . Gorai.010G231000.1 . .
. . . . . . . .
. . . . . . .
Vv09g0323 Tc06_g007350 . Gorai.010G230900.1 .

. . . . . . . .
. . . . . . . .
Vv09g0324 Tc06_g007360 . Gorai.010G230700.1 .
. . . . . . . .
. . . . . . . .
Vv09g0325 Tc06_g007380 . Gorai.010G230600.1 .
. . . . . . . .
. . . . . . . .
Vv09g0326 . . . . . . .
. . . . . . . .
. . . . .
Vv09g0327 . . . . . . .
. . . . . . . .
. . . . .
Vv09g0328 Tc06_g007390 Gorai.006G096900.1 . .
Gorai.011G071100.1 . Vv11g0326
Tc09_g008970 . . . Gorai.009G048500.1 .
. . . . . . .
Vv09g0329 Tc06_g007400 . . . Gorai.
011G071200.1 . . . . . . .
. . . . . . . .
Vv09g0330 . . . . . . .
. . . . . . . .
. . . . .
Vv09g0331 . . . . . . .
. . . . . . . .
. . . . .
Vv09g0332 . . . . . . .
. . . . . . . .
. . . . .
Vv09g0333 . . . . . . .
. . . . . . . .
. . . . .
Vv09g0334 . . . . . . .
. . . . . . . .
. . . . .
Vv09g0335 . . . . . . .
. . . . . . . .
. . . . .
Vv09g0336 Tc06_g007440 . . . Gorai.
011G071300.1 . Vv11g0327 Tc09_g009000 . .
. . Gorai.010G076700.1 . . .
. . . .
Vv09g0337 . . . . . . .
. . . . . . . .
. . . . .
Vv09g0338 . . . . . . .
. . . . . . . .
. . . . .
Vv09g0339 Tc06_g007460 . . . Gorai.

011G071400.1 . Vv11g0329 Tc09_g009030 .
Gorai.001G026200.1 . Gorai.009G048600.1 . .
. . . . . .
Vv09g0340 . . . . . . .
. . . . . . . .
. . . . .
Vv09g0341 Tc06_g007490 . . Gorai.009G128700.1
. . . . . . . .
. . . . . . . .
Vv09g0342 Tc06_g007500 . . Gorai.009G128800.1
Gorai.011G071500.1 . . . . .
. . . . . . . .
. .
Vv09g0343 . . . . . . .
. . . . . . . .
. . . . .
Vv09g0344 . . . . . . .
. . . . . . . .
. . . . .
Vv09g0345 Tc06_g007560 . . . Gorai.
011G071700.1 . . . . . . .
. . . . . . . .
Vv09g0346 Tc06_g007600 . . . Gorai.
011G071900.1 . . . . . . .
. . . . . . . .
Vv09g0347 Tc06_g007610 . . . Gorai.
011G072000.1 . . . . . . .
. . . . . . . .
Vv09g0348 Tc06_g007620 . . . Gorai.
011G072100.1 . . . . . . .
. . . . . . . .
Vv09g0349 . . . . . . .
. . . . . . . .
. . . . .
Vv09g0350 . . . . Gorai.011G072300.1
. . . . . . . .
Vv04g0324 . . . . Gorai.
003G062800.1 Gorai.011G072300.1
Vv09g0351 Tc06_g007700 . . . . .
. . . . . . . .
. . . . . .
Vv09g0352 Tc06_g007710 . . . . .
. . . . . . .
Vv04g0328 Tc01_g036690 . Gorai.001G026800.1 .
. Gorai.010G105900.1
Vv09g0353 Tc06_g007740 . Gorai.010G230500.1 .
Gorai.011G072500.1 . . . . .
. . . Vv04g0330 Tc01_g015300 .
. Gorai.004G288400.1 Gorai.009G049300.1
Gorai.011G072500.1

Vv09g0354 Tc06_g007780 . . . Gorai.
011G072600.1 . . . . . . .
. . . . . . . .
Vv09g0355 . . . . . . .
. . . . . . . .
. . . . .
Vv09g0356 Tc06_g007790 . . . . .
. . . . . . . .
. . . . . .
Vv09g0357 Tc06_g002920 . . . . .
. . . . . . . .
. . . . . .
Vv09g0358 Tc06_g002900 . . . . .
. . . . . . . .
. . . . . .
Vv09g0359 Tc06_g002880 . . . . .
. . . . . . . .
. . . . . .
Vv09g0360 Tc06_g002830 . . . . .
. . . . . . . .
. . . . . .
Vv09g0361 Tc06_g002820 . . . . .
. . . . . . . .
. . . . . .
Vv09g0362 Tc06_g007800 . . . . .
. . . . . . . .
. . . . . .
Vv09g0363 Tc06_g007820 . . . . .
. . . . . . . .
. . . . . .
Vv09g0364 Tc06_g007830 . . . . .
. . . . . . . .
. . . . . .
Vv09g0365 Tc06_g007840 . . . . .
. . . . . . . .
. . . . . .
Vv09g0366 Tc06_g007860 Gorai.006G097000.1 . .
. . . . . . . .
. . . . . . . .
Vv09g0367 Tc06_g007910 . . . . .
. . . . . . . .
. . . . . .
Vv09g0368 Tc06_g007920 . . . . .
. . . . . . . .
. . . . . .
Vv09g0369 Tc06_g007930 . . . . .
. . . . . . . .
. . . . . .
Vv09g0370 . . . . . . .
. . . . . . . .

```
        .       .       .       .       .
Vv09g0371       Tc06_g007940    .       Gorai.010G229800.1      .
        .       .       .       .       .       .       .       .
        .       .       .       .       .       .       .       .
Vv09g0372       Tc06_g007970    .       .       .       Gorai.
011G072800.1    .       .       .       .       .       .       .
        .       .       .       .       .       .       .       .
Vv09g0373       Tc06_g007980    .       .       .       Gorai.
011G072900.1    .       .       .       .       .       .       .
        .       .       .       .       .       .       .       .
Vv09g0374       Tc06_g007990    .       .       .       .       .
        .       .       .       .       .       .       .
Vv04g0357       Tc01_g001220    .       .       .       Gorai.
003G064000.1    Gorai.010G107900.1
Vv09g0375       .       .       .       .       .       .       .
        .       .       .       .       .       .       .       .
        .       .       .       .       .
Vv09g0376       Tc06_g008000    Gorai.006G097100.1      .       .
        .       .       .       .       .       .       .       .
        .       .       .       .       .       .       .       .
Vv09g0377       Tc06_g008010    .       .       .       .       .
        .       .       .       .       .       .       .       .
        .       .       .       .       .       .
Vv09g0378       Tc06_g008020    .       .       .       Gorai.
011G073000.1    .       .       .       .       .       .       .
        .       .       .       .       .       .       .       .
Vv09g0379       Tc06_g008030    .       .       .       .       .
        Vv11g0364       Tc09_g009400    .       .       .
Gorai.009G026700.1      .       .       .       .       .       .
        .       .
Vv09g0380       .       .       .       .       .       .       .
        .       .       .       .       .       .       .       .
        .       .       .       .       .
Vv09g0381       Tc06_g008060    .       .       .       Gorai.
011G073200.1    .       .       .       .       .       .       .
        .       .       .       .       .       .       .       .
Vv09g0382       Tc06_g008070    .       Gorai.010G229600.1      .
        Gorai.011G073300.1      .       .       .       .       .
        .       .       .       .       .       .       .       .
        .       .
Vv09g0383       Tc06_g008080    .       .       .       Gorai.
011G020100.1    .       .       .       .       .       .       .
        .       .       .       .       .       .       .       .
Vv09g0384       .       .       .       .       .       .       .
        .       .       .       .       .       .       .       .
        .       .       .       .       .
Vv09g0385       Tc06_g008150    .       .       .       .       .
        Vv11g0370       .       .       .       .       .       .
        Vv04g0363       .       .       .       .       .       .
Vv09g0386       .       .       .       .       .       .       .
```

```
        .       .       .       .       .       .       .       .
        .       .       .       .       .
Vv09g0387       .       .       .       .       .       .       .
        .       .       .       .       .       .       .       .
        .       .       .       .       .
Vv09g0388       Tc06_g008160    Gorai.006G097200.1      .       .
        Gorai.011G073500.1      .       .       .       .       .
        .       .       .       .       .       .       .       .
        .       .
Vv09g0389       Tc06_g008180    Gorai.006G097300.1      .       .
        Gorai.011G073600.1      .       .       .       .       .
        .       .       .       .       .       .       .       .
        .       .
Vv09g0390       Tc06_g008190    .       .       .       Gorai.
011G073700.1    .       .       .       .       .       .       .
        .       .       .       .       .       .       .       .
Vv09g0391       .       .       .       .       .       .       .
        .       .       .       .       .       .       Vv04g0370
        .       .       Gorai.001G027600.1      .       .       .
Vv09g0392       Tc06_g008200    .       .       .       Gorai.
011G073900.1    .       .       .       .       .       .       .
        .       .       .       .       .       .       .       .
Vv09g0393       .       .       .       .       .       .       .
        .       .       .       .       .       .       .       .
        .       .       .       .       .
Vv09g0394       .       .       .       .       .       .       .
        .       .       .       .       .       .       .       .
        .       .       .       .       .
Vv09g0395       .       .       .       .       .       .       .
        .       .       .       .       .       .       .       .
        .       .       .       .       .
Vv09g0396       .       .       .       .       .       .       .
        .       .       .       .       .       .       .       .
        .       .       .       .       .
Vv09g0397       .       .       .       .       .       .       .
        .       .       .       .       .       .       .       .
        .       .       .       .       .
Vv09g0398       .       .       .       .       Gorai.011G018500.1
        .       .       .       .       .       .       .       .
        Vv04g0386       .       .       Gorai.001G028700.1      .
        Gorai.009G051700.1      .
Vv09g0399       .       .       .       .       .       .       .
        .       .       .       .       .       .       .       .
        .       .       .       .       .
Vv09g0400       .       .       .       .       .       .
Vv11g0389       Tc09_g009630    .       .       .       Gorai.
009G025600.1    .       .       .       .       .       .       .
        .
Vv09g0401       .       .       .       .       .       .       .
        .       .       .       .       .       .       .       .
```

```
        .       .       .       .       .
Vv09g0402       .       .       .       .       .       .       .
        .       .       .       .       .       .       .       .
        .       .       .       .       .
Vv09g0403       Tc06_g008410    .       .       .       .       .
        .       .       .       .       .       .       .       .
        .       .       .       .       .       .
Vv09g0404       .       .       .       .       .       .       .
        .       .       .       .       .       .       .       .
        .       .       .       .       .
Vv09g0405       Tc06_g008420    Gorai.006G097500.1      .       .
        .       .       .       .       .       .       .       .
        .       .       .       .       .       .       .       .
Vv09g0406       .       .       .       .       .       .       .
        .       .       .       .       .       .       .       .
        .       .       .       .       .
Vv09g0407       .       .       .       .       .       .       .
        .       .       .       .       .       .       .       .
        .       .       .       .       .
Vv09g0408       .       .       .       .       Gorai.011G074400.1
        .       .       .       .       .       .       .       .
        .       .       .       .       .       .       .
Vv09g0409       .       .       .       .       Gorai.011G016200.1
        .       .       .       .       .       .       .       .
        .       .       .       .       .       .       .
Vv09g0410       .       .       .       .       .       .       .
        .       .       .       .       .       .       .       .
        .       .       .       .       .
Vv09g0411       .       .       .       .       .       .       .
        .       .       .       .       .       .       .       .
        .       .       .       .       .
Vv09g0412       .       .       .       .       .       .       .
        .       .       .       .       .       .       .       .
        .       .       .       .       .
Vv09g0413       .       .       .       .       .       .       .
        .       .       .       .       .       .       .       .
        .       .       .       .       .
Vv09g0414       .       .       .       .       .       .       .
        .       .       .       .       .       .       .       .
        .       .       .       .       .
Vv09g0415       .       .       .       .       Gorai.011G074800.1
        .       .       .       .       .       .       .       .
        .       .       .       .       .       .       .
Vv09g0416       .       .       .       .       Gorai.011G074900.1
        .       .       .       .       .       .       .       .
        Vv04g0394       .       .       Gorai.001G028800.1      .
        Gorai.009G051900.1      Gorai.011G074900.1
Vv09g0417       Tc06_g008520    .       Gorai.010G228200.1      .
        Gorai.011G075000.1      .       .       .       .       .
        .       .       .       .       .       .       .       .
```

. .
Vv09g0418 Tc06_g008540 . . . . .
. . . . . . . .
. . . . . .
Vv09g0419 . . . . . .
Vv11g0406 Tc09_g009850 . . Gorai.004G280000.1
Gorai.009G024600.1 . . . . .
. . .
Vv09g0420 Tc06_g008550 . . . Gorai.
011G075200.1 . Vv11g0407 . . . .
. . . . . . . .
.
Vv09g0421 Tc06_g008570 . . . Gorai.
011G075300.1 . . . . . . .
. . . . . . . .
Vv09g0422 Tc06_g008580 Gorai.006G098000.1 . .
Gorai.011G075400.1 . . . . .
. . . . . . . .
. .
Vv09g0423 Tc06_g008590 . . . . .
Vv11g0409 Tc09_g009870 . . Gorai.
004G279900.1 Gorai.009G024400.1 Gorai.010G070700.1 .
. . . . . .
Vv09g0424 Tc06_g008600 Gorai.006G098200.1 . .
. . . . . . . .
. . . . . . . .
Vv09g0425 . . . . . . .
. . . . . . . .
. . . . .
Vv09g0426 . . . . . . .
. . . . . . . .
. . . . .
Vv09g0427 . . . . . . .
. . . . . . . .
. . . . .
Vv09g0428 . . Gorai.010G225300.1 . .
. . . . . . . .
. . . . . . .
Vv09g0429 Tc06_g008620 . . . . .
. . . . . . . .
. . . . . .
Vv09g0430 . . . . . . .
. . . . . . . .
. . . . .
Vv09g0431 . . . . . . .
. . . . . . . .
. . . . .
Vv09g0432 . . . . . . .
. . . . . . . .
. . . . .

Vv09g0433 . . . . . . .
. . . . . . . .
. . . . .
Vv09g0434 Tc06_g008650 Gorai.006G098400.1 . .
. . . . . . . .
. . . . . . . .
Vv09g0435 . . . . . .
Vv11g0412 Tc09_g009900 . Gorai.001G005200.1 .
. Gorai.010G070400.1 . . . .
. . .
Vv09g0436 . . . . . . .
. . . . . . . .
. . . . .
Vv09g0437 Tc06_g008660 . . . . .
. . . . . . .
Vv04g0432 . . . . Gorai.009G052500.1
.
Vv09g0438 Tc06_g008670 . . . . .
. . . . . . . .
. . . . . .
Vv09g0439 Tc06_g008690 . . . . .
Vv11g0415 Tc09_g009940 . Gorai.001G004800.1
. . Gorai.010G069900.1 . . .
. . . .
Vv09g0440 . . . . . . .
. . . . . . . .
. . . . .
Vv09g0441 . . . . . . .
. . . . . . . .
. . . . .
Vv09g0442 . . . . . . .
. . . . . . . .
. . . . .
Vv09g0443 . . . . . . .
. . . . . . . .
. . . . .
Vv09g0444 . . . . Gorai.011G012700.1
. . . . . . . .
. . . . . . .
Vv09g0445 . . . . . . .
. . . . . . Vv04g0458
. . . . . .
Vv09g0446 . . . . . . .
. . . . . . . .
. . . . .
Vv09g0447 Tc06_g010430 . . . Gorai.
011G076900.1 . Vv11g0423 Tc09_g010070 .
Gorai.001G004200.1 . . . . . .
. . . .
Vv09g0448 . . . . Gorai.011G077200.1

. . . . . . . .
. . . . . . .
Vv09g0449 . . . . . . .
. . . . . . . .
. . . . .
Vv09g0450 . . . . . . .
. . . . . . . .
. . . . .
Vv09g0451 . . . . . . .
. . . . . . . .
. . . . .
Vv09g0452 . . . . . . .
. . . . . . . .
. . . . .
Vv09g0453 . . . . . . .
. . . . . . . .
. . . . .
Vv09g0454 . . . . . .
Vv11g0426 . . . . . . .
. . . . . .
Vv09g0455 . . . . . . .
. . . . . . . .
. . . . .
Vv09g0456 . . . . . . .
. . . . . . . .
. . . . .
Vv09g0457 . . . . . . .
. . . . . . . .
. . . . .
Vv09g0458 . . . . Gorai.011G077300.1
. . . . . . . .
. . . . . . .
Vv09g0459 . . . . . . .
. . . . . . . .
. . . . .
Vv09g0460 . . . . . . .
. . . . . . . .
. . . . .
Vv09g0461 Tc06_g000240 . . Gorai.009G115200.1
. . Vv11g0432 Tc09_g010120 . .
Gorai.004G279500.1 Gorai.009G012100.1 Gorai.
010G069100.1 Vv04g0459 . . . Gorai.
004G279500.1 . .
Vv09g0462 . . . . . .
Vv11g0433 Tc09_g010130 . . . . .
. . . . . . .
Vv09g0463 . . . . Gorai.011G077400.1
. . . . . . . .
. . . . . . .
Vv09g0464 . . . . Gorai.011G077500.1

| | | | | | | | |
|---|---|---|---|---|---|---|---|
| . | . | . | . | . | . | . | . |
| . | . | . | . | . | . | . | |
| Vv09g0465 | . | . | . | . | . | . | . |
| . | . | . | . | . | . | . | . |
| . | . | . | . | . | | | |
| Vv09g0466 | . | . | . | . | . | . | . |
| . | . | . | . | . | . | . | . |
| . | . | . | . | . | | | |
| Vv09g0467 | Tc06_g000590 | | . | . | . | . | . |
| . | . | . | . | . | . | . | . |
| . | . | . | . | . | . | | |
| Vv09g0468 | . | . | . | . | . | . | . |
| . | . | . | . | . | . | . | . |
| . | . | . | . | . | | | |
| Vv09g0469 | . | . | . | . | . | . | . |
| . | . | . | . | . | . | . | . |
| . | . | . | . | . | | | |
| Vv09g0470 | Tc06_g000660 | | . | . | . | . | . |
| . | . | . | . | . | . | . | . |
| . | . | . | . | . | . | | |
| Vv09g0471 | . | . | . | . | Gorai.011G077800.1 | | |
| . | . | . | . | . | . | . | . |
| . | . | . | . | . | . | . | |
| Vv09g0472 | Tc06_g000670 | | . | . | . | . | . |
| . | . | . | . | . | . | . | . |
| . | . | . | . | . | . | | |
| Vv09g0473 | Tc06_g000690 | | . | . | . | . | . |
| . | . | . | . | . | . | . | . |
| . | . | . | . | . | . | | |
| Vv09g0474 | . | . | . | . | . | . | . |
| . | . | . | . | . | . | . | . |
| . | . | . | . | . | | | |
| Vv09g0475 | . | . | . | . | . | . | . |
| . | . | . | . | . | . | . | . |
| . | . | . | . | . | | | |
| Vv09g0476 | Tc06_g000700 | | . | . | . | . | . |
| . | . | . | . | . | . | . | . |
| . | . | . | . | . | . | | |
| Vv09g0477 | . | . | . | . | . | . | . |
| . | . | . | . | . | . | . | . |
| . | . | . | . | . | | | |
| Vv09g0478 | . | . | . | . | Gorai.011G078000.1 | | |
| . | . | . | . | . | . | . | . |
| . | . | . | . | . | . | . | |
| Vv09g0479 | . | . | . | . | . | . | . |
| . | . | . | . | . | . | . | . |
| . | . | . | . | . | | | |
| Vv09g0480 | . | . | . | . | . | . | . |
| . | . | . | . | . | . | . | . |
| . | . | . | . | . | | | |

Vv09g0481 . . . . Gorai.011G078100.1
. . . . . . . .
. . . . . . .
Vv09g0482 . . . . . . .
. . . . . . . .
. . . . .
Vv09g0483 . . . . Gorai.011G078200.1
. . . . . . . .
. . . . . . .
Vv09g0484 Tc06_g010820 . . . . .
. . . . . . . .
. . . . . .
Vv09g0485 . . . . Gorai.011G078300.1
. . . . . . . .
. . . . . . .
Vv09g0486 Tc06_g010810 . . . . .
. . . . . . . .
. . . . . .
Vv09g0487 Tc06_g010810 . . . . .
. . . . . . . .
. . . . . .
Vv09g0488 Tc06_g010820 . . . . .
. . . . . . . .
. . . . . .
Vv09g0489 . . . . . . .
. . . . . . . .
. . . . .
Vv09g0490 Tc06_g007330 . . . . .
Vv11g0466 Tc09_g010370 . . Gorai.
004G279200.1 . . . . . . .
. .
Vv09g0491 . . . . . . .
. . . . . . . .
. . . . .
Vv09g0492 . . . . . . .
. . . . . . . .
. . . . .
Vv09g0493 . . . . . . .
. . . . . . . .
. . . . .
Vv09g0494 . . . . . . .
. . . . . . . .
. . . . .
Vv09g0495 . . . . . . .
. . . . . . . .
. . . . .
Vv09g0496 . . . . . . .
. . . . . . . .
. . . . .
Vv09g0497 . . . . . . .

. . . . . . . .
. . . . .
Vv09g0498 . . . . . . .
. . . . . . . .
. . . . .
Vv09g0499 . . . . . . .
. . . . . . . .
. . . . .
Vv09g0500 . . . . . . .
. . . . . . . .
. . . . .
Vv09g0501 . . . . Gorai.011G078400.1
. . . . . . . .
. . . . . . .
Vv09g0502 . . . . . . .
. . . . . . . .
. . . . .
Vv09g0503 Tc06_g010760 . . . . .
. . . . . . . .
. . . . . .
Vv09g0504 Tc06_g010870 . . . . .
. . . . . . . .
. . . . . .
Vv09g0505 . . . . . . .
. . . . . . . .
. . . . .
Vv09g0506 . . . . . . .
. . . . . . . .
. . . . .
Vv09g0507 Tc06_g007220 . . . Gorai.
011G078700.1 . . . . . . .
. . . . . . . .
Vv09g0508 Tc06_g007210 . . . . .
. . . . . . . .
. . . . . .
Vv09g0509 Tc06_g013430 . . . . .
. . . . . . . .
. . . . . .
Vv09g0510 . . . . Gorai.011G078800.1
. . . . . . . .
. . . . . . .
Vv09g0511 Tc06_g013400 . . . . .
. . . . . . . .
. . . . . .
Vv09g0512 Tc06_g013390 . . . Gorai.
011G079000.1 . . . . . . .
. . . . . . . .
Vv09g0513 Tc06_g006940 . . . Gorai.
011G079100.1 . . . . . . .
. . . . . . . .

Vv09g0514 . . . . . . .
. . . . . . . .
. . . . .
Vv09g0515 . . . . . . .
. . . . . . . .
. . . . .
Vv09g0516 . . . . . . .
. . . . . . . .
. . . . .
Vv09g0517 . . . . . . .
. . . . . . . .
. . . . .
Vv09g0518 . . . . . . .
. . . . . . . .
. . . . .
Vv09g0519 . . . . . . .
. . . . . . . .
. . . . .
Vv09g0520 . . . . . . .
. . . . . . . .
. . . . .
Vv09g0521 . . . . . . .
. . . . . . . .
. . . . .
Vv09g0522 . . . . . . .
. . . . . . . .
. . . . .
Vv09g0523 . . . . . . .
. . . . . . . .
. . . . .
Vv09g0524 . . . . . . .
. . . . . . . .
. . . . .
Vv09g0525 . . . . . . .
. . . . . . . .
. . . . .
Vv09g0526 . . . . . . .
. . . . . . . .
. . . . .
Vv09g0527 . . . . . . .
. . . . . . . .
. . . . .
Vv09g0528 . . . . . . .
. . . . . . . .
. . . . .
Vv09g0529 . . . . . . .
. . . . . . . .
. . . . .
Vv09g0530 . . . . . . .
. . . . . . . .

. . . . .
Vv09g0531 . . . . . . .
. . . . . . . .
. . . . .
Vv09g0532 . . . . . . .
. . . . . . . .
. . . . .
Vv09g0533 . . . . . . .
. . . . . . . .
. . . . .
Vv09g0534 . . . . . . .
. . . . . . . .
. . . . .
Vv09g0535 . . . . . . .
. . . . . . . .
. . . . .
Vv09g0536 . . . . . . .
. . . . . . . .
. . . . .
Vv09g0537 . . . . . . .
. . . . . . . .
. . . . .
Vv09g0538 . . . . . . .
. . . . . . . .
. . . . .
Vv09g0539 . . . . . . .
. . . . . . . .
. . . . .
Vv09g0540 . . . . . . .
. . . . . . . .
. . . . .
Vv09g0541 . . . . . . .
. . . . . . . .
. . . . .
Vv09g0542 . . . . . . .
. . . . . . . .
. . . . .
Vv09g0543 Tc06_g013380 . . . . .
. . . . . . . .
. . . . . .
Vv09g0544 . . . . . . .
. . . . . . . .
. . . . .
Vv09g0545 . . . . . . .
. . . . . . . .
. . . . .
Vv09g0546 . . . . . . .
. . . . . . . .
. . . . .
Vv09g0547 . . . . . . .

. . . . . . . .
. . . . .
Vv09g0548 . . . . . . .
. . . . . . . .
. . . . .
Vv09g0549 . . . . . . .
. . . . . . . .
. . . . .
Vv09g0550 . . . . Gorai.011G079300.1
. . . . . . . .
. . . . . . .
Vv09g0551 . . . . . . .
. . . . . . . .
. . . . .
Vv09g0552 . . . . Gorai.011G079400.1
. Vv04g0461 . . . . .
. . . . . . . .
Vv09g0553 Tc06_g006890 . . . . .
. . . . . . . .
. . . . . .
Vv09g0554 . . . . . . .
. . . . . . . .
. . . . .
Vv09g0555 Tc06_g006880 Gorai.006G094600.1 . .
. . . . . . . .
. . . . . . . .
Vv09g0556 Tc06_g006850 . . . Gorai.
011G079500.1 . . . . . . .
. . . . . . . .
Vv09g0557 Tc06_g006840 . Gorai.010G227600.1
Gorai.009G129100.1 Gorai.011G079600.1 . . .
. . . . . . . .
. . . .
Vv09g0558 . . . . . . .
. . . . . . . .
. . . . .
Vv09g0559 . . . . . .
Vv04g0487 . . . . . . .
. . . . . .
Vv09g0560 Tc06_g006820 . . . . .
. . . . . . . .
. . . . . .
Vv09g0561 Tc06_g006810 . . Gorai.009G129200.1
Gorai.011G079700.1 . . . . .
. . . . . . . .
. .
Vv09g0562 Tc06_g006800 . . Gorai.009G129300.1
. . . . . . . .
. . . . . . . .
Vv09g0563 Tc06_g006790 . . Gorai.009G129400.1

. . . . . . . .
. . . . . . . .
Vv09g0564 Tc06_g006730 . . . Gorai.
011G079800.1 . . . . . . .
. . . . . . . .
Vv09g0565 . . . . . . .
. . . . . . . .
. . . . .
Vv09g0566 . . . . . . .
. . . . . . . .
. . . . .
Vv09g0567 Tc06_g006590 . Gorai.010G227500.1 .
. . Vv11g0493 Tc09_g010730 . .
. Gorai.009G010600.1 . Vv04g0512 .
. . Gorai.004G274500.1 . .
Vv09g0568 . . . . . . .
. . . . . . . .
. . . . .
Vv09g0569 . . . . . . .
. . . . . . . .
. . . . .
Vv09g0570 . . . . . . .
. . . . . . . .
. . . . .
Vv09g0571 . . . . . . .
. . . . . . . .
. . . . .
Vv09g0572 . . . . . . .
. . . . . . . .
. . . . .
Vv09g0573 . . . . . . .
. . . . . . . .
. . . . .
Vv09g0574 . . Gorai.010G227400.1 .
Gorai.011G080100.1 . . . . . .
. . Vv04g0515 Tc09_g017500 . .
. . Gorai.011G080200.1
Vv09g0575 . . . . . . .
. . . . . . . .
. . . . .
Vv09g0576 . . . . . . .
. . . . . . . .
. . . . .
Vv09g0577 . . . . . . .
. . . . . . . .
. . . . .
Vv09g0578 . . . . . . .
. . . . . . . .
. . . . .
Vv09g0579 . . . . . . .

. . . . . . . .
. . . . .
Vv09g0580 . . Gorai.010G227300.1 Gorai.
009G129600.1 Gorai.011G080500.1 . . . .
. . . . Vv04g0524 . .
. . . Gorai.011G080500.1
Vv09g0581 . . Gorai.010G227200.1 Gorai.
009G129700.1 Gorai.011G080600.1 . Vv11g0506
Tc09_g010830 Gorai.013G233000.1 . . Gorai.
009G020100.1 Gorai.010G065500.1 Vv04g0526 .
Gorai.013G233000.1 . . . Gorai.011G080600.1
Vv09g0582 . . . . . . .
. . . . . . Vv04g0527
Tc09_g003770 . . . . .
Vv09g0583 . . . . . . .
. . . . . . . .
. . . . .
Vv09g0584 . . . . Gorai.011G080700.1
. . . . . . . .
. . . . . . .
Vv09g0585 . . . Gorai.009G129800.1 .
. Vv11g0508 Tc09_g010880 . Gorai.
001G001400.1 Gorai.004G278000.1 Gorai.009G019900.1 .
. . . . . . .
Vv09g0586 . . . . . . .
. . . . . . . .
. . . . .
Vv09g0587 . . . Gorai.009G129900.1 .
. . . . . . . .
. . . . . . .
Vv09g0588 . . . . . . .
. . . . . . . .
. . . . .
Vv09g0589 . . . . . . .
. . . . . . . .
. . . . .
Vv09g0590 . . . . . . .
. . . . . . . .
. . . . .
Vv09g0591 . . . . . . .
. . . . . . . .
. . . . .
Vv09g0592 . . . . . . .
. . . . . . . .
. . . . .
Vv09g0593 . . Gorai.010G227000.1 Gorai.
009G130100.1 . . . . . . .
. . Vv04g0530 . Gorai.013G233200.1
. . . .
Vv09g0594 . . . . . . .

. . . . . . . .
. . . . .
Vv09g0595 . . . . . .
Vv11g0515 Tc09_g010960 . Gorai.001G040700.1 .
. . Vv04g0532 Tc01_g039000 Gorai.
013G182300.1 . . . .
Vv09g0596 . . . . . . .
. . . . . . . .
. . . . .
Vv09g0597 . . . . . . .
. . . . . . . .
. . . . .
Vv09g0598 . . . . . . .
. . . . . . . .
. . . . .
Vv09g0599 . . . . . . .
. . . . . . . .
. . . . .
Vv09g0600 . . . . . . .
. . . . . . . .
. . . . .
Vv09g0601 . . . . . . .
. . . . . . . .
. . . . .
Vv09g0602 . . . . . . .
. . . . . . . .
. . . . .
Vv09g0603 . . . . . . .
. . . . . . . .
. . . . .
Vv09g0604 . . . Gorai.009G130200.1
Gorai.011G081300.1 . . . . . .
. . . . . . . .
.
Vv09g0605 . . . . . . .
. . . . . . . .
. . . . .
Vv09g0606 . . . . . . .
. . . . . . . .
. . . . .
Vv09g0607 . . . . . . .
. . . . . . . .
. . . . .
Vv09g0608 . . . . . . .
. . . . . . . .
. . . . .
Vv09g0609 . . . . . .
Vv11g0517 Tc09_g010980 . Gorai.001G040900.1 .
Gorai.009G019200.1 . . . . .
. . .

```
Vv09g0610 . . . . Gorai.011G081400.1
. . . . . . . .
Vv04g0534 . . . Gorai.004G274300.1
. Gorai.011G081400.1
Vv09g0611 . . . Gorai.009G130300.1 .
. . . . . . . .
. . . . . . .
Vv09g0612 . . . . . . .
. . . . . . . .
. . . . .
Vv09g0613 . . . Gorai.009G130400.1
Gorai.011G081500.1 . . . . . .
. . . . . . . .
.
Vv09g0614 . . . . . .
Vv11g0521 Tc09_g011040 . Gorai.001G041000.1 .
. Gorai.010G064800.1 . . . .
. . .
Vv09g0615 . . . . . . .
. . . . . . . .
. . . . .
Vv09g0616 . . . . . . .
. . . . . . . .
. . . . .
Vv09g0617 . . . . . . .
. . . . . . . .
. . . . .
Vv09g0618 . . . . Gorai.011G081700.1
. . . . . . . .
. . . . . . .
Vv09g0619 . . . . . . .
. . . . . . . .
. . . . .
Vv09g0620 . . . . . .
Vv11g0524 Tc09_g011060 . . . . .
. . . . . . .
Vv09g0621 . . . . Gorai.011G081900.1
. . . . . . . .
. . . . . . .
Vv09g0622 . . . . . . .
. . . . . . . .
. . . . .
Vv09g0623 . . . . . . .
. . . . . . . .
. . . . .
Vv09g0624 . . . . . . .
. . . . . . . .
. . . . .
Vv09g0625 . . . . . . .
. . . . . . . .
```

```
         .       .        .       .        .
Vv09g0626        .        .       .        .       .        .
Vv11g0531        Tc09_g011130     Gorai.013G233800.1        .       .
         Gorai.009G010100.1       Gorai.010G064500.1        Vv04g0542
         .       Gorai.013G233800.1        .       Gorai.004G274000.1
         .       .
Vv09g0627        .        .       .        .       .        .       .
         .       .        .       .        .       .        .       .
         .       .        .       .        .
Vv09g0628        .        .       .        .       .        .       .
         .       .        .       .        .       .        .       .
         .       .        .       .        .
Vv09g0629        .        .       .        .       .        .       .
         .       .        .       .        .       .        .       .
         .       .        .       .        .
Vv09g0630        .        .       .        .       .        .       .
         .       .        .       .        .       .        .       .
         .       .        .       .        .
Vv09g0631        .        .       .        .       Gorai.011G082000.1
         .       .        .       .        .       .        .       .
         Vv04g0545        .       Gorai.013G215500.1        .       .
         .       Gorai.011G082000.1
Vv09g0632        .        .       .        .       .        .       .
         .       .        .       .        .       .        .       .
         .       .        .       .        .
Vv09g0633        .        .       .        .       .        .       .
         .       .        .       .        .       .        .       .
         .       .        .       .        .
Vv09g0634        .        .       .        Gorai.009G130600.1
Gorai.011G082100.1        .       Vv11g0534        Tc09_g011190     .
         .       .        .       Gorai.010G064400.1        .       .
         .       .        .       .        .
Vv09g0635        .        .       Gorai.010G226500.1        .       .
         .       .        .       .        .       .        .       .
         .       .        .       .        .       .        .
Vv09g0636        .        .       Gorai.010G226400.1        .       .
         .       .        .       .        .       .        .       .
         .       .        .       .        .       .        .
Vv09g0637        .        .       .        .       .        .       .
         .       .        .       .        .       .        .       .
         .       .        .       .        .
Vv09g0638        .        .       .        .       .        .       .
         .       .        .       .        .       .        .       .
         .       .        .       .        .
Vv09g0639        .        .       .        .       .        .       .
         .       .        .       .        .       .        .       .
         .       .        .       .        .
Vv09g0640        .        .       .        .       .        .       .
         .       .        .       .        .       .        .       .
         .       .        .       .        .
```

Vv09g0641 . . . . . . .
. . . . . . . .
. . . . .
Vv09g0642 . . . . . . .
. . . . . . . .
. . . . .
Vv09g0643 . . . . . . .
. . . . . . . .
. . . . .
Vv09g0644 . . . . . . .
. . . . . . . .
. . . . .
Vv09g0645 Tc06_g001570 . . . Gorai.
011G082500.1 . . . . . . .
. . . . . . . .
Vv09g0646 . . . . . . .
. . . . . . . .
. . . . .
Vv09g0647 Tc06_g001580 . . Gorai.009G130700.1
. . . . . . . .
. . . . . . . .
Vv09g0648 Tc06_g001600 . . Gorai.009G130800.1
Gorai.011G082600.1 . Vv11g0558
Tc09_g011370 . Gorai.001G042100.1 . Gorai.
009G017600.1 . Vv04g0565 . . Gorai.
001G042100.1 . . Gorai.011G082600.1
Vv09g0649 Tc06_g001610 . . . Gorai.
011G082800.1 . . . . . . .
. . . . . . . .
Vv09g0650 Tc06_g001620 . . . Gorai.
011G082900.1 . . . . . . .
. . . . . . . .
Vv09g0651 . . . . . . .
. . . . . . . .
. . . . .
Vv09g0652 Tc06_g001640 . Gorai.010G225600.1 .
Gorai.011G083000.1 . Vv11g0564 . .
. . . . Vv04g0569 . .
Gorai.001G042400.1 . Gorai.009G057800.1
Gorai.011G083000.1
Vv09g0653 Tc06_g001650 . . Gorai.009G130900.1
Gorai.011G083300.1 . . . . .
. . . . . . . .
. .
Vv09g0654 . . . . . . .
. . . . . . . .
. . . . .
Vv09g0655 . . . . . . .
. . . . . . . .
. . . . .

Vv09g0656 Tc06_g001670 . . Gorai.009G131100.1
. . . . . . . .
. . . . . . . .
Vv09g0657 . . . . . . .
. . . . . . . .
. . . . .
Vv09g0658 Tc06_g001690 . . Gorai.009G131200.1
Gorai.011G083600.1 . . . . .
. . . . . . . .
. .
Vv09g0659 Tc06_g001700 . . Gorai.009G131300.1
. . Vv11g0573 Tc09_g011490 Gorai.
013G234200.1 Gorai.001G033500.1 . Gorai.009G016900.1
Gorai.010G063200.1 . . . . .
. .
Vv09g0660 . . . . . .
Vv11g0575 Tc09_g011500 Gorai.013G234300.1 . .
. Gorai.010G063100.1 . . . .
. . .
Vv09g0661 Tc06_g001710 . . . Gorai.
011G083700.1 . . . . . . .
. . . . . . . .
Vv09g0662 . . . . . . .
. . . . . . . .
. . . . .
Vv09g0663 . . . . . . .
. . . . . . . .
. . . . .
Vv09g0664 . . . . . . .
. . . . . . . .
. . . . .
Vv09g0665 Tc06_g001720 Gorai.006G101100.1 . .
Gorai.011G083900.1 . . . . .
. . . . . . . .
. .
Vv09g0666 Tc06_g001730 . . . Gorai.
011G084000.1 . Vv11g0587 Tc09_g031660 . .
. . . . . . . .
. .
Vv09g0667 . . . . . . .
. . . . . . . .
. . . . .
Vv09g0668 . . . Gorai.009G131500.1 .
. . . . . . . .
. . . . . . .
Vv09g0669 . . . . . . .
. . . . . . . .
. . . . .
Vv09g0670 Tc06_g001740 . . . . .
. . . . . . . .

. . . . . .
Vv09g0671 . . . . . .
Vv11g0623 Tc09_g012000 . . . Gorai.
009G059000.1 Gorai.010G061000.1 . . . .
. . .
Vv09g0672 Tc06_g001760 . . Gorai.009G128500.1
Gorai.011G084100.1 . . . . .
. . . . . . . .
. .
Vv09g0673 . . . . . . .
. . . . . . . .
. . . . .
Vv09g0674 . . . . . . .
. . . . . . . .
. . . . .
Vv09g0675 . . . . . . .
. . . . . . . .
. . . . .
Vv09g0676 . . . . . . .
. . . . . . . .
. . . . .
Vv09g0677 . . . . . . .
. . . . . . . .
. . . . .
Vv09g0678 Tc06_g001910 Gorai.006G101300.1 Gorai.
010G225500.1 . . . . . . .
. . . . . . . .
. .
Vv09g0679 Tc06_g001920 . . Gorai.009G128400.1
. . . . . . . .
. . . . . . . .
Vv09g0680 . . . . . . .
. . . . . . . .
. . . . .
Vv09g0681 Tc06_g001940 . Gorai.010G225300.1
Gorai.009G128300.1 . . . . . .
. . . . . . . .
. .
Vv09g0682 . . . . . . .
. . . . . . . .
. . . . .
Vv09g0683 . . . Gorai.009G128200.1 .
. . . . . . . .
. . . . . . .
Vv09g0684 . . . . . . .
. . . . . . . .
. . . . .
Vv09g0685 . . . . . . .
. . . . . . . .
. . . . .

Vv09g0686 Tc06_g014130 . . . . .
. . . . . . . .
. . . . . .
Vv09g0687 . . . . . . .
. . . . . . . .
. . . . .
Vv09g0688 Tc06_g014110 . . . . .
. . . . . . . .
. . . . . .
Vv09g0689 . . . Gorai.009G127700.1
Gorai.011G084400.1 . . . . . .
. . . . . . . .
.
Vv09g0690 . . . . . . .
. . . . . . . .
. . . . .
Vv09g0691 . . . Gorai.009G127600.1 .
. Vv04g0584 . . Gorai.001G034300.1
. . Gorai.010G115600.1 . . .
. . . .
Vv09g0692 . . . . . . .
. . . . . . . .
. . . . .
Vv09g0693 Tc06_g002930 . Gorai.010G225000.1 .
. . . . . . . .
. . . . . . . .
Vv09g0694 Tc06_g002950 . . Gorai.009G127500.1
. . . . . . . .
. . . . . . . .
Vv09g0695 . . . . . . .
. . . . . . . .
. . . . .
Vv09g0696 . . . . . . .
. . . . . . . .
. . . . .
Vv09g0697 Tc06_g002970 . Gorai.010G224900.1 .
. . . . . . . .
. . . . . . . .
Vv09g0698 . . . . . . .
. . . . . . . .
. . . . .
Vv09g0699 . . . . . . .
. . . . . . . .
. . . . .
Vv09g0700 . . . . . . .
. . . . . . . .
. . . . .
Vv09g0701 Tc06_g002980 . . . .
Gorai.010G140900.1 . . . . . .
. . . . . . . .

Vv09g0702 . . . . . . .
. . . . . . . .
. . . . .
Vv09g0703 Tc06_g003120 . . Gorai.009G127100.1
Gorai.011G084700.1 . . . . .
. . . . . . . .
. .
Vv09g0704 Tc06_g003130 . . . Gorai.
011G084800.1 . Vv04g0589 . . Gorai.
001G034600.1 . . Gorai.011G084800.1 . .
. . . . .
Vv09g0705 . . . . . . .
. . . . . . . .
. . . . .
Vv09g0706 Tc06_g003150 . Gorai.010G224300.1 .
. . . . . . . .
. . . . . . . .
Vv09g0707 Tc06_g003160 . . . .
Gorai.010G138400.1 . . . . . .
. . . . . . . .
Vv09g0708 . Gorai.006G101600.1 . Gorai.
009G127000.1 . . Vv04g0593 . . .
. . Gorai.011G085000.1 . . .
. . . .
Vv09g0709 . . . . Gorai.011G085000.1
. . . . . . . .
. . . . . . .
Vv09g0710 . . . . . . .
. . . . . . . .
. . . . .
Vv09g0711 . . . . . . .
. . . . . . . .
. . . . .
Vv09g0712 . . . . . . .
. . . . . . . .
. . . . .
Vv09g0713 . . . . . . .
. . . . . . . .
. . . . .
Vv09g0714 . . . . . . .
. . . . . . . .
. . . . .
Vv09g0715 . . . . . . .
. . . . . . . .
. . . . .
Vv09g0716 Tc06_g003200 Gorai.006G101700.1 . .
. . . . . . . .
. . . . . . . .
Vv09g0717 Tc06_g014070 . . . . .
. . . . . . . .

. . . . . .
Vv09g0718 Tc06_g003210 . . . . .
. . . . . . . .
. . . . . .
Vv09g0719 Tc06_g003260 . Gorai.010G224200.1 .
. Gorai.010G138100.1 . . . .
. . . . . . . .
. .
Vv09g0720 . . Gorai.010G222000.1 . .
. . . . . . . .
. . . . . . .
Vv09g0721 Tc06_g003280 . . . . .
. . . . . . . .
. . . . . .
Vv09g0722 . . . . . . .
. . . . . . . .
. . . . .
Vv09g0723 . . . . . . .
. . . . . . . .
. . . . .
Vv09g0724 Tc06_g003480 . . . . .
. . . . . . . .
. . . . . .
Vv09g0725 . . . . . . .
. . . . . . . .
. . . . .
Vv09g0726 Tc06_g003500 . . Gorai.009G126300.1
. . . . . . . .
. . . . . . . .
Vv09g0727 Tc06_g003510 . . . . .
. . . . . . . .
. . . . . .
Vv09g0728 . . . . . . .
. . . . . . . .
. . . . .
Vv09g0729 . . . . . . .
. . . . . . . .
. . . . .
Vv09g0730 . . . . . . .
. . . . . . . .
. . . . .
Vv09g0731 . . . . . . .
. . . . . . . .
. . . . .
Vv09g0732 . . . . . . .
. . . . . . . .
. . . . .
Vv09g0733 . . . . . . .
. . . . . . . .
. . . . .

Vv09g0734 . . . . . . .
. . . . . . . .
. . . . .
Vv09g0735 . . . . . . .
. . . . . . . .
. . . . .
Vv09g0736 . . . . . . .
. . . . . . . .
. . . . .
Vv09g0737 . . . . . . .
. . . . . . . .
. . . . .
Vv09g0738 . . . . . . .
. . . . . . . .
. . . . .
Vv09g0739 . . . . . . .
. . . . . . . .
. . . . .
Vv09g0740 . . . . . . .
. . . . . . . .
. . . . .
Vv09g0741 Tc06_g003670 . . . Gorai.
011G085400.1 . Vv04g0654 . . . .
Gorai.009G060000.1 . . . . .
. . .
Vv09g0742 . . . . . . .
. . . . . . . .
. . . . .
Vv09g0743 . . . Gorai.009G125900.1 .
. . . . . . . .
. . . . . . .
Vv09g0744 . . . . . . .
. . . . . . . .
. . . . .
Vv09g0745 . . . . . . .
. . . . . . . .
. . . . .
Vv09g0746 . . . . . . .
. . . . . . . .
. . . . .
Vv09g0747 . . . . . . .
. . . . . . . .
. . . . .
Vv09g0748 . . . . . . .
. . . . . . . .
. . . . .
Vv09g0749 . . . . . . .
. . . . . . . .
. . . . .
Vv09g0750 . . . . . . .

. . . . . . . .
. . . . .
Vv09g0751 . . . . . .
Vv04g0651 . . . . . . .
. . . . . .
Vv09g0752 . . . . . . .
. . . . . . . .
. . . . .
Vv09g0753 . . . . . . .
. . . . . . . .
. . . . .
Vv09g0754 . . . . . . .
. . . . . . . .
. . . . .
Vv09g0755 . . . . . . .
. . . . . . . .
. . . . .
Vv09g0756 . . . . . . .
. . . . . . . .
. . . . .
Vv09g0757 . . . . . . .
. . . . . . . .
. . . . .
Vv09g0758 Tc06_g019920 . Gorai.010G184600.1 .
Gorai.011G011400.1 . . . . .
. . . . . . . .
. .
Vv09g0759 . . . . . . .
. . . . . . . .
. . . . .
Vv09g0760 Tc06_g019910 . Gorai.010G184500.1 .
. . . . . . . .
. . . . . . . .
Vv09g0761 . . . . . . .
. . . . . . . .
. . . . .
Vv09g0762 Tc06_g019900 . . . Gorai.
011G011500.1 . . . . . . .
. . . . . . . .
Vv09g0763 . . . . . . .
. . . . . . . .
. . . . .
Vv09g0764 . . . . . . .
. . . . . . . .
. . . . .
Vv09g0765 . . . . . . .
. . . . . . . .
. . . . .
Vv09g0766 . . . . . . .
. . . . . . . .

| | | | | | | | | |
|---|---|---|---|---|---|---|---|---|
| | . | . | . | . | . | | | |
| Vv09g0767 | | . | . | . | . | . | . | . |
| | . | . | . | . | . | . | . | . |
| | . | . | . | . | . | | | |
| Vv09g0768 | | . | . | . | . | . | . | . |
| | . | . | . | . | . | . | . | . |
| | . | . | . | . | . | | | |
| Vv09g0769 | | . | . | . | . | . | . | . |
| | . | . | . | . | . | . | . | . |
| | . | . | . | . | . | | | |
| Vv09g0770 | | . | . | . | . | . | . | . |
| | . | . | . | . | . | . | . | . |
| | . | . | . | . | . | | | |
| Vv09g0771 | | . | . | . | . | . | . | . |
| | . | . | . | . | . | . | . | . |
| | . | . | . | . | . | | | |
| Vv09g0772 | | . | . | . | . | . | . | . |
| | . | . | . | . | . | . | . | . |
| | . | . | . | . | . | | | |
| Vv09g0773 | | Tc06_g007830 | | . | . | . | . | . |
| | . | . | . | . | . | . | . | . |
| | . | . | . | . | . | . | | |
| Vv09g0774 | | . | . | . | . | . | . | . |
| | . | . | . | . | . | . | . | . |
| | . | . | . | . | . | | | |
| Vv09g0775 | | . | . | . | . | . | . | . |
| | . | . | . | . | . | . | . | . |
| | . | . | . | . | . | | | |
| Vv09g0776 | | . | . | . | . | . | . | . |
| | . | . | . | . | . | . | . | . |
| | . | . | . | . | . | | | |
| Vv09g0777 | | . | . | . | . | . | . | . |
| | . | . | . | . | . | . | . | . |
| | . | . | . | . | . | | | |
| Vv09g0778 | | . | . | . | . | . | . | . |
| | . | . | . | . | . | . | . | . |
| | . | . | . | . | . | | | |
| Vv09g0779 | | . | . | . | . | . | . | . |
| | . | . | . | . | . | . | . | . |
| | . | . | . | . | . | | | |
| Vv09g0780 | | . | . | . | . | . | . | . |
| | . | . | . | . | . | . | . | . |
| | . | . | . | . | . | | | |
| Vv09g0781 | | . | . | . | . | . | . | . |
| | . | . | . | . | . | . | . | . |
| | . | . | . | . | . | | | |
| Vv09g0782 | | . | . | . | . | . | . | . |
| | . | . | . | . | . | . | . | . |
| | . | . | . | . | . | | | |
| Vv09g0783 | | . | . | . | Gorai.009G124900.1 | | | . |

```
        .        .        .        .        .        .        .        .
        .        .        .        .        .        .        .
Vv09g0784        .        .        .        .        .        .        .
        .        .        .        .        .        .        .        .
        .        .        .        .        .
Vv09g0785        .        .        .        .        .        .        .
        .        .        .        .        .        .        .        .
        .        .        .        .        .
Vv09g0786        Tc06_g003980      .        .        Gorai.009G124800.1
        .        .        Vv04g0628        Tc09_g013830      .        .
        .        .        Gorai.010G117900.1        .        .        .
        .        .        .        .
Vv09g0787        .        .        .        .        .        .        .
        .        .        .        .        .        .        .        .
        .        .        .        .        .
Vv09g0788        Tc06_g003990      .        .        .        .        .
        .        .        .        .        .        .        .        .
        .        .        .        .        .        .
Vv09g0789        .        .        .        .        .        .        .
        .        .        .        .        .        .        .        .
        .        .        .        .        .
Vv09g0790        .        .        .        .        .        .        .
        .        .        .        .        .        .        .        .
        .        .        .        .        .
Vv09g0791        Tc06_g004000      .        .        Gorai.009G124700.1
        .        .        Vv04g0626        .        .        .        .
        Gorai.009G060800.1        .        .        .        .        .
        .        .        .
Vv09g0792        Tc06_g004010      .        .        Gorai.009G124600.1
        .        .        .        .        .        .        .        .
        .        .        .        .        .        .        .        .
Vv09g0793        Tc06_g019780      .        Gorai.010G183400.1        .
        Gorai.011G011800.1        .        .        .        .        .
        .        .        .        .        .        .        .        .
        .        .
Vv09g0794        .        .        .        .        .        .        .
        .        .        .        .        .        .        .        .
        .        .        .        .        .
Vv09g0795        .        .        .        .        .        .        .
        .        .        .        .        .        .        .        .
        .        .        .        .        .
Vv09g0796        Tc06_g007860      Gorai.006G097000.1        .        .
        .        .        .        .        .        .        .        .
        .        .        .        .        .        .        .        .
Vv09g0797        .        .        .        .        .        .        .
        .        .        .        .        .        .        .        .
        .        .        .        .        .
Vv09g0798        .        .        .        .        .        .        .
        .        .        .        .        .        .        .        .
        .        .        .        .        .
```

| | | | | | | | | | | | | | | | | | | | | |
|---|---|---|---|---|---|---|---|---|---|---|---|---|---|---|---|---|---|---|---|---|
| Vv09g0799 | . | . | . | . | . | . | . | . | . | . | . | . | . | . | . | . | . | . | . | . |
| Vv09g0800 | . | . | . | . | . | . | . | . | . | . | . | . | . | . | . | . | . | . | . | . |
| Vv09g0801 | . | . | . | . | . | . | . | . | . | . | . | . | . | . | . | . | . | . | . | . |
| Vv09g0802 | . | . | . | . | . | . | . | . | . | . | . | . | . | . | . | . | . | . | . | . |
| Vv09g0803 | . | . | . | . | . | . | . | . | . | . | . | . | . | . | . | . | . | . | . | . |
| Vv09g0804 | . | . | . | . | . | . | . | . | . | . | . | . | . | . | . | . | . | . | . | . |
| Vv09g0805 | . | . | . | . | . | . | . | . | . | . | . | . | . | . | . | . | . | . | . | . |
| Vv09g0806 | . | . | . | . | . | . | . | . | . | . | . | . | . | . | . | . | . | . | . | . |
| Vv09g0807 | . | . | . | . | . | . | . | . | . | . | . | . | . | . | . | . | . | . | . | . |
| Vv09g0808 | . | . | . | . | . | . | . | . | . | . | . | . | . | . | . | . | . | . | . | . |
| Vv09g0809 | . | . | . | . | . | . | . | . | . | . | . | . | . | . | . | . | . | . | . | . |
| Vv09g0810 | . | . | . | . | . | . | . | . | . | . | . | . | . | . | . | . | . | . | . | . |
| Vv09g0811 | . | . | . | . | . | . | . | . | . | . | . | . | . | . | . | . | . | . | . | . |
| Vv09g0812 | . | . | . | . | . | . | . | . | . | . | . | . | . | . | . | . | . | . | . | . |
| Vv09g0813 | . | . | . | . | . | . | . | . | . | . | . | . | . | . | . | . | . | . | . | . |
| Vv09g0814 | . | . | . | . | . | . | . | . | . | . | . | . | . | . | . | . | . | . | . | . |
| Vv09g0815 | . | . | . | . | . | . | . | . | . | . | . | . | . | . | . | | | | | |

. . . . .
Vv09g0816 . . . . . . .
. . . . . . . .
. . . . .
Vv09g0817 . . . . . . .
. . . . . . . .
. . . . .
Vv09g0818 . . . . . . .
. . . . . . . .
. . . . .
Vv09g0819 . . . . . . .
. . . . . . . .
. . . . .
Vv09g0820 . . . . . . .
. . . . . . . .
. . . . .
Vv09g0821 . . . . . . .
. . . . . . . .
. . . . .
Vv09g0822 . . . . . . .
. . . . . . . .
. . . . .
Vv09g0823 . . . . . . .
. . . . . . . .
. . . . .
Vv09g0824 . . . . . . .
. . . . . . . .
. . . . .
Vv09g0825 . . . . . . .
. . . . . . . .
. . . . .
Vv09g0826 . . . . . . .
. . . . . . . .
. . . . .
Vv09g0827 . . . . . . .
. . . . . . . .
. . . . .
Vv09g0828 . . . . . . .
. . . . . . . .
. . . . .
Vv09g0829 . . . . . . .
. . . . . . . .
. . . . .
Vv09g0830 Tc06_g004420 . . Gorai.009G124000.1
. . Vv04g0603 Tc01_g017430 .
Gorai.001G037800.1 . Gorai.009G062400.1 . .
. . . . . .
Vv09g0831 Tc06_g004430 . . Gorai.009G123900.1
. . . . . . . .
. . . . . . . .

Vv09g0832 . . . . . . .
. . . . . . . .
. . . . .
Vv09g0833 Tc06_g004460 . . . . .
. . . . . . . .
. . . . . .
Vv09g0834 . . . Gorai.009G123800.1 .
. . . . . . . .
. . . . . . .
Vv09g0835 Tc06_g004470 . . Gorai.009G123700.1
. . . . . . . .
. . . . . . . .
Vv09g0836 . . . . . . .
. . . . . . . .
. . . . .
Vv09g0837 . . . . . . .
. . . . . . . .
. . . . .
Vv09g0838 . . . . . . .
. . . . . . . .
. . . . .
Vv09g0839 . . . . . . .
. . . . . . . .
. . . . .
Vv09g0840 . . . . . . .
. . . . . . . .
. . . . .
Vv09g0841 . . . . . . .
. . . . . . . .
. . . . .
Vv09g0842 Tc06_g004550 . . Gorai.009G123500.1
. . . . . . . .
. . . . . . . .
Vv09g0843 . . . . . . .
. . . . . . . .
. . . . .
Vv09g0844 . . . . . . .
. . . . . . . .
. . . . .
Vv09g0845 . . . . . . .
. . . . . . . .
. . . . .
Vv09g0846 . . . . . . .
. . . . . . . .
. . . . .
Vv09g0847 Tc06_g004560 . . Gorai.009G123400.1
. . . . . . . .
. . . . . . . .
Vv09g0848 Tc06_g004570 . . Gorai.009G123300.1
. . . . . . . .

| | | | | | | | |
|---|---|---|---|---|---|---|---|
| . | . | . | . | . | . | . | . |
| Vv09g0849 | . | . | . | . | . | . | . |
| . | . | . | . | . | . | . | . |
| . | . | . | . | . | | | |
| Vv09g0850 | . | . | . | . | . | . | . |
| . | . | . | . | . | . | . | . |
| . | . | . | . | . | | | |
| Vv09g0851 | . | . | . | . | . | . | . |
| . | . | . | . | . | . | . | . |
| . | . | . | . | . | | | |
| Vv09g0852 | . | . | . | . | . | . | . |
| . | . | . | . | . | . | . | . |
| . | . | . | . | . | | | |
| Vv09g0853 | . | . | . | . | . | . | . |
| . | . | . | . | . | . | . | . |
| . | . | . | . | . | | | |
| Vv09g0854 | Tc06_g000660 | | . | . | . | . | . |
| . | . | . | . | . | . | . | . |
| . | . | . | . | . | . | | |
| Vv09g0855 | Tc06_g004590 | | . | . | Gorai.009G123100.1 | | |
| . | . | . | . | . | . | . | . |
| . | . | . | . | . | . | . | . |
| Vv09g0856 | Tc06_g004610 | | . | . | Gorai.009G123000.1 | | |
| . | . | . | . | . | . | . | . |
| . | . | . | . | . | . | . | . |
| Vv09g0857 | . | . | . | . | . | . | . |
| . | . | . | . | . | . | . | . |
| . | . | . | . | . | | | |
| Vv09g0858 | Tc06_g004640 | | . | . | . | . | . |
| . | . | . | . | . | . | . | . |
| . | . | . | . | . | . | | |
| Vv09g0859 | . | . | . | . | . | . | . |
| . | . | . | . | . | . | . | . |
| . | . | . | . | . | | | |
| Vv09g0860 | Tc06_g004670 | | . | . | . | . | . |
| . | . | . | . | . | . | . | . |
| . | . | . | . | . | . | | |
| Vv09g0861 | Tc06_g004690 | | . | . | . | . | . |
| . | . | . | . | . | . | . | . |
| . | . | . | . | . | . | | |
| Vv09g0862 | . | . | . | . | . | . | . |
| . | . | . | . | . | . | . | . |
| . | . | . | . | . | | | |
| Vv09g0863 | . | . | . | . | . | . | . |
| . | . | . | . | . | . | . | . |
| . | . | . | . | . | | | |
| Vv09g0864 | . | . | . | . | . | . | . |
| . | . | . | . | . | . | . | . |
| . | . | . | . | . | | | |
| Vv09g0865 | Tc06_g004700 | | . | . | Gorai.009G122900.1 | | |

. Gorai.010G140900.1 . . . .
. . . . . . . .
. .
Vv09g0866 . . . . . . .
. . . . . . . .
. . . . .
Vv09g0867 . . . . . . .
. . . . . . . .
. . . . .
Vv09g0868 . . . Gorai.009G120100.1 .
. . . . . . . .
. . . . . . .
Vv09g0869 . . . . . . .
. . . . . . . .
. . . . .
Vv09g0870 . . . . . . .
. . . . . . . .
. . . . .
Vv09g0871 . . . . . . .
. . . . . . . .
. . . . .
Vv09g0872 . . . . . . .
. . . . . . . .
. . . . .
Vv09g0873 . . . . . . .
. . . . . . . .
. . . . .
Vv09g0874 . . . . . . .
. . . . . . . .
. . . . .
Vv09g0875 . . . . . . .
. . . . . . . .
. . . . .
Vv09g0876 . . . . . . .
. . . . . . . .
. . . . .
Vv09g0877 . . . . . . .
. . . . . . . .
. . . . .
Vv09g0878 . . . . . . .
. . . . . . . .
. . . . .
Vv09g0879 . . . . . . .
. . . . . . . .
. . . . .
Vv09g0880 . . . . . . .
. . . . . . . .
. . . . .
Vv09g0881 . . . . . . .
. . . . . . . .

| | | | | | | | |
|---|---|---|---|---|---|---|---|
| Vv09g0882 | . | . | . | . | . | . | . |
| Vv09g0883 | . | . | . | . | . | . | . |
| Vv09g0884 | . | . | . | . | . | . | . |
| Vv09g0885 | . | . | . | . | . | . | . |
| Vv09g0886 | . | . | . | . | . | . | . |
| Vv09g0887 | . | . | . | . | . | . | . |
| Vv09g0888 | . | . | . | . | . | . | . |
| Vv09g0889 | . | . | . | . | . | . | . |
| Vv09g0890 | . | . | . | . | . | . | . |
| Vv09g0891 | . | . | . | . | . | . | . |
| Vv09g0892 | . | . | . | . | . | . | . |
| Vv09g0893 | . | . | . | . | . | . | . |
| Vv09g0894 | Tc06_g000790 | | . | . | . | . | . |
| Vv09g0895 | . | . | . | . | . | . | . |
| Vv09g0896 | . | . | . | . | . | . | . |
| Vv09g0897 | . | . | . | . | . | . | . |
| Vv09g0898 | . | . | . | . | . | . | . |

| | | | | | | | |
|---|---|---|---|---|---|---|---|
| . | . | . | . | . | . | . | . |
| . | . | . | . | . | | | |
| Vv09g0899 | . | . | . | . | . | . | . |
| . | . | . | . | . | . | . | . |
| . | . | . | . | . | | | |
| Vv09g0900 | . | . | . | . | . | . | . |
| . | . | . | . | . | . | . | . |
| . | . | . | . | . | | | |
| Vv09g0901 | . | . | . | . | . | . | . |
| . | . | . | . | . | . | . | . |
| . | . | . | . | . | | | |
| Vv09g0902 | Tc06_g004910 | | . | . | . | . | . |
| . | . | . | . | . | . | . | . |
| . | . | . | . | . | . | | |
| Vv09g0903 | . | . | . | . | . | . | . |
| . | . | . | . | . | . | . | . |
| . | . | . | . | . | | | |
| Vv09g0904 | . | . | . | . | . | . | . |
| . | . | . | . | . | . | . | . |
| . | . | . | . | . | | | |
| Vv09g0905 | . | . | . | . | . | . | . |
| . | . | . | . | . | . | . | . |
| . | . | . | . | . | | | |
| Vv09g0906 | . | . | . | . | . | . | . |
| . | . | . | . | . | . | . | . |
| . | . | . | . | . | | | |
| Vv09g0907 | . | . | . | . | . | . | . |
| . | . | . | . | . | . | . | . |
| . | . | . | . | . | | | |
| Vv09g0908 | . | . | . | . | . | . | . |
| . | . | . | . | . | . | . | . |
| . | . | . | . | . | | | |
| Vv09g0909 | . | . | . | . | . | . | . |
| . | . | . | . | . | . | . | . |
| . | . | . | . | . | | | |
| Vv09g0910 | . | . | . | . | . | . | . |
| . | . | . | . | . | . | . | . |
| . | . | . | . | . | | | |
| Vv09g0911 | . | . | . | . | . | . | . |
| . | . | . | . | . | . | . | . |
| . | . | . | . | . | | | |
| Vv09g0912 | . | . | . | . | . | . | . |
| . | . | . | . | . | . | . | . |
| . | . | . | . | . | | | |
| Vv09g0913 | . | . | . | . | . | . | . |
| . | . | . | . | . | . | . | . |
| . | . | . | . | . | | | |
| Vv09g0914 | . | . | . | . | . | . | . |
| . | . | . | . | . | . | . | . |
| . | . | . | . | . | | | |

Vv09g0915 Tc06_g000790 . . . . .
. . . . . . . .
. . . . . .
Vv09g0916 . . . . . . .
. . . . . . . .
. . . . .
Vv09g0917 Tc06_g010980 . . . . .
. . . . . . . .
. . . . . .
Vv09g0918 . . . Gorai.009G118900.1 .
. . . . . . . .
. . . . . . .
Vv09g0919 . . . . . . .
. . . . . . . .
. . . . .
Vv09g0920 . . . . . . .
. . . . . . . .
. . . . .
Vv09g0921 . . . . . . .
. . . . . . . .
. . . . .
Vv09g0922 Tc06_g011010 . . Gorai.009G117900.1
. . . . . . . .
. . . . . . . .
Vv09g0923 Tc06_g011020 . . . Gorai.
011G053600.1 . . . . . . .
. . . . . . . .
Vv09g0924 . . . . . . .
. . . . . . . .
. . . . .
Vv09g0925 Tc06_g011030 . . . . .
. . . . . . . .
. . . . . .
Vv09g0926 . . . . . . .
. . . . . . . .
. . . . .
Vv09g0927 Tc06_g011050 . Gorai.010G172200.1 .
Gorai.011G053400.1 . . . . .
. . . . . . . .
. .
Vv09g0928 . . . . . . .
. . . . . . . .
. . . . .
Vv09g0929 . . . . . . .
. . . . . . . .
. . . . .
Vv09g0930 . . . . . . .
. . . . . . . .
. . . . .
Vv09g0931 . . . . . . .

. . . . . . . .
. . . . .
Vv09g0932 . . . . . . .
. . . . . . . .
. . . . .
Vv09g0933 Tc06_g011070 . . . Gorai.
011G053300.1 . . . . . . .
. . . . . . . .
Vv09g0934 . . . . . . .
. . . . . . . .
. . . . .
Vv09g0935 . . . . . . .
. . . . . . . .
. . . . .
Vv09g0936 Tc06_g011080 Gorai.006G091100.1 . .
Gorai.011G053200.1 . . . . .
. . . . . . . .
. .
Vv09g0937 . . . . . . .
. . . . . . . .
. . . . .
Vv09g0938 Tc06_g011090 . . . . .
. . . . . . . .
. . . . . .
Vv09g0939 . . . . . . .
. . . . . . . .
. . . . .
Vv09g0940 . . . . . . .
. . . . . . . .
. . . . .
Vv09g0941 . . . . . . .
. . . . . . . .
. . . . .
Vv09g0942 . . . . . . .
. . . . . . . .
. . . . .
Vv09g0943 . . . . . . .
. . . . . . . .
. . . . .
Vv09g0944 . . . . . . .
. . . . . . . .
. . . . .
Vv09g0945 . . . . . . .
. . . . . . . .
. . . . .
Vv09g0946 . . . . . . .
. . . . . . . .
. . . . .
Vv09g0947 Tc06_g001180 . . . . .
. . . . . . . .

. . . . . .
Vv09g0948 . . . . . . .
. . . . . . . .
. . . . .
Vv09g0949 . . . . . . .
. . . . . . . .
. . . . .
Vv09g0950 Tc06_g011100 Gorai.006G091300.1 . .
Gorai.011G053100.1 . . . . .
. . . . . . . .
. .
Vv09g0951 . . . . . . .
. . . . . . . .
. . . . .
Vv09g0952 . . . . . . .
. . . . . . . .
. . . . .
Vv09g0953 . . . . . . .
. . . . . . . .
. . . . .
Vv09g0954 . . . . . . .
. . . . . . . .
. . . . .
Vv09g0955 . . . Gorai.009G121800.1 .
. . . . . . . .
. . . . . . .
Vv09g0956 . . . . . . .
. . . . . . . .
. . . . .
Vv09g0957 . . . . . . .
. . . . . . . .
. . . . .
Vv09g0958 . . . . . . .
. . . . . . . .
. . . . .
Vv09g0959 . . . . . . .
. . . . . . . .
. . . . .
Vv09g0960 . . . Gorai.009G121900.1 .
. . . . . . . .
. . . . . . .
Vv09g0961 . . . . . . .
. . . . . . . .
. . . . .
Vv09g0962 . . . . . . .
. . . . . . . .
. . . . .
Vv09g0963 . . . Gorai.009G122000.1 .
. . . . . . . .
. . . . . . .

Vv09g0964 . . . . . . .
. . . . . . . .
. . . . .

Vv09g0965 . . . Gorai.009G122100.1 .
. . . . . . . .
. . . . . . .

Vv09g0966 Tc06_g011450 . . . . .
. . . . . . . .
. . . . . .

Vv09g0967 . . . . . . .
. . . . . . . .
. . . . .

Vv09g0968 . . . . . . .
. . . . . . . .
. . . . .

Vv09g0969 . . . . . . .
. . . . . . . .
. . . . .

Vv09g0970 . . . . . . .
. . . . . . . .
. . . . .

Vv09g0971 Tc06_g020500 . Gorai.010G188300.1 .
. . . . . . . .
. . . . . . . .

Vv09g0972 . . . . . . .
. . . . . . . .
. . . . .

Vv09g0973 . . . . . . .
. . . . . . . .
. . . . .

Vv09g0974 . . . Gorai.009G122300.1 .
. . . . . . . .
. . . . . . .

Vv09g0975 . . . . . . .
. . . . . . . .
. . . . .

Vv09g0976 . . . . . . .
. . . . . . . .
. . . . .

Vv09g0977 Tc06_g020480 . Gorai.010G188200.1 .
. . . . . . . .
. . . . . . . .

Vv09g0978 . . . . . . .
. . . . . . . .
. . . . .

Vv09g0979 . . . . . . .
. . . . . . . .
. . . . .

Vv09g0980 . . . . . . .
. . . . . . . .

. . . . .
Vv09g0981 . . . . . . .
. . . . . . . .
. . . . .
Vv09g0982 Tc06_g014190 Gorai.006G089200.1 Gorai.
010G159800.1 . . . . . . .
. . . . . . . .
. .
Vv09g0983 . . . . . . .
. . . . . . . .
. . . . .
Vv09g0984 . . . . . . .
. . . . . . . .
. . . . .
Vv09g0985 . . . . . . .
. . . . . . . .
. . . . .
Vv09g0986 . . . . . . .
. . . . . . . .
. . . . .
Vv09g0987 . . . . . . .
. . . . . . . .
. . . . .
Vv09g0988 . . . . . . .
. . . . . . . .
. . . . .
Vv09g0989 . . . . . . .
. . . . . . . .
. . . . .
Vv09g0990 . . . . . . .
. . . . . . . .
. . . . .
Vv09g0991 . . . . . . .
. . . . . . . .
. . . . .
Vv09g0992 . . . . . . .
. . . . . . . .
. . . . .
Vv09g0993 . . . . . . .
. . . . . . . .
. . . . .
Vv09g0994 . . . . . . .
. . . . . . . .
. . . . .
Vv09g0995 . . . . . . .
. . . . . . . .
. . . . .
Vv09g0996 Tc06_g011800 . . . . .
. . . . . . . .
. . . . . .

Vv09g0997 . . . . . . .
. . . . . . . .
. . . . .
Vv09g0998 . . . . . . .
. . . . . . . .
. . . . .
Vv09g0999 . . . . . . .
. . . . . . . .
. . . . .
Vv09g1000 . . . . . . .
. . . . . . . .
. . . . .
Vv09g1001 . . . . . . .
. . . . . . . .
. . . . .
Vv09g1002 . . . . . . .
. . . . . . . .
. . . . .
Vv09g1003 . . . . . . .
. . . . . . . .
. . . . .
Vv09g1004 . . . . . . .
. . . . . . . .
. . . . .
Vv09g1005 . . . . . . .
. . . . . . . .
. . . . .
Vv09g1006 . . . . . . .
. . . . . . . .
. . . . .
Vv09g1007 . . . . . . .
. . . . . . . .
. . . . .
Vv09g1008 . . . . . . .
. . . . . . . .
. . . . .
Vv09g1009 . . . . . . .
. . . . . . . .
. . . . .
Vv09g1010 . . . . . . .
. . . . . . . .
. . . . .
Vv09g1011 . . . . . . .
. . . . . . . .
. . . . .
Vv09g1012 . . . . . . .
. . . . . . . .
. . . . .
Vv09g1013 . . . . . . .
. . . . . . . .

| | | | | | | | |
|---|---|---|---|---|---|---|---|
| . | . | . | . | . | | | |
| Vv09g1014 | . | . | . | . | . | . | . |
| . | . | . | . | . | . | . | . |
| . | . | . | . | . | | | |
| Vv09g1015 | . | . | . | . | . | . | . |
| . | . | . | . | . | . | . | . |
| . | . | . | . | . | | | |
| Vv09g1016 | . | . | . | . | . | . | . |
| . | . | . | . | . | . | . | . |
| . | . | . | . | . | | | |
| Vv09g1017 | . | . | . | . | . | . | . |
| . | . | . | . | . | . | . | . |
| . | . | . | . | . | | | |
| Vv09g1018 | . | . | . | . | . | . | . |
| . | . | . | . | . | . | . | . |
| . | . | . | . | . | | | |
| Vv09g1019 | Tc06_g014140 | | . | . | . | . | . |
| . | . | . | . | . | . | . | . |
| . | . | . | . | . | . | | |
| Vv09g1020 | . | . | . | . | . | . | . |
| . | . | . | . | . | . | . | . |
| . | . | . | . | . | | | |
| Vv09g1021 | . | . | . | . | . | . | . |
| . | . | . | . | . | . | . | . |
| . | . | . | . | . | | | |
| Vv09g1022 | . | . | . | . | . | . | . |
| . | . | . | . | . | . | . | . |
| . | . | . | . | . | | | |
| Vv09g1023 | . | . | . | . | . | . | . |
| . | . | . | . | . | . | . | . |
| . | . | . | . | . | | | |
| Vv09g1024 | Tc06_g003910 | | . | . | Gorai.009G125200.1 | | |
| . | Gorai.010G136300.1 | | | . | . | . | . |
| . | . | . | . | . | . | . | . |
| . | . | | | | | | |
| Vv09g1025 | . | . | . | . | . | . | . |
| . | . | . | . | . | . | . | . |
| . | . | . | . | . | | | |
| Vv09g1026 | . | . | . | . | . | . | . |
| . | . | . | . | . | . | . | . |
| . | . | . | . | . | | | |
| Vv09g1027 | . | . | . | . | . | . | . |
| . | . | . | . | . | . | . | . |
| . | . | . | . | . | | | |
| Vv09g1028 | Tc06_g013030 | | . | Gorai.010G165300.1 | | | . |
| . | . | . | . | . | . | . | . |
| . | . | . | . | . | . | . | . |
| Vv09g1029 | . | . | . | . | . | . | . |
| . | . | . | . | . | . | . | . |
| . | . | . | . | . | | | |

| | | | | | | | | | | | | | | | | | | | | |
|---|---|---|---|---|---|---|---|---|---|---|---|---|---|---|---|---|---|---|---|---|
| Vv09g1030 | . | . | . | . | . | . | . | . | . | . | . | . | . | . | . | . | . | . | . | . |
| Vv09g1031 | . | . | . | . | . | . | . | . | . | . | . | . | . | . | . | . | . | . | . | . |
| Vv09g1032 | . | . | . | . | . | . | . | . | . | . | . | . | . | . | . | . | . | . | . | . |
| Vv09g1033 | . | . | . | . | . | . | . | . | . | . | . | . | . | . | . | . | . | . | . | . |
| Vv09g1034 | . | . | . | . | . | . | . | . | . | . | . | . | . | . | . | . | . | . | . | . |
| Vv09g1035 | . | . | . | . | . | . | . | . | . | . | . | . | . | . | . | . | . | . | . | . |
| Vv09g1036 | . | . | . | . | . | . | . | . | . | . | . | . | . | . | . | . | . | . | . | . |
| Vv09g1037 | . | . | . | . | . | . | . | . | . | . | . | . | . | . | . | . | . | . | . | . |
| Vv09g1038 | Tc06_g012700 | . | . | . | . | . | . | . | . | . | . | . | . | . | . | . | . | . | . | . |
| Vv09g1039 | . | . | . | . | . | . | . | . | . | . | . | . | . | . | . | . | . | . | . | . |
| Vv09g1040 | . | . | . | . | . | . | . | . | . | . | . | . | . | . | . | . | . | . | . | . |
| Vv09g1041 | . | . | . | . | . | . | . | . | . | . | . | . | . | . | . | . | . | . | . | . |
| Vv09g1042 | . | . | . | . | . | . | . | . | . | . | . | . | . | . | . | . | . | . | . | . |
| Vv09g1043 | . | . | . | . | . | . | . | . | . | . | . | . | . | . | . | . | . | . | . | . |
| Vv09g1044 | . | . | . | . | . | . | . | . | . | . | . | . | . | . | . | . | . | . | . | . |
| Vv09g1045 | . | . | . | . | . | . | . | . | . | . | . | . | . | . | . | . | . | . | . | . |
| Vv09g1046 | . | . | . | . | . | . | . | . | . | . | . | . | . | . | . | | | | | |

. . . . .
Vv09g1047 . . . . . . .
. . . . . . . .
. . . . .
Vv09g1048 . . . . . . .
. . . . . . . .
. . . . .
Vv09g1049 . . . . . . .
. . . . . . . .
. . . . .
Vv09g1050 . . . . . . .
. . . . . . . .
. . . . .
Vv09g1051 . . . . . . .
. . . . . . . .
. . . . .
Vv09g1052 . . . . . . .
. . . . . . . .
. . . . .
Vv09g1053 . . . . . . .
. . . . . . . .
. . . . .
Vv09g1054 . . . . . . .
. . . . . . . .
. . . . .
Vv09g1055 . . . . . . .
. . . . . . . .
. . . . .
Vv09g1056 Tc06_g003390 . . . . .
. . . . . . . .
. . . . . .
Vv09g1057 . . . . . . .
. . . . . . . .
. . . . .
Vv09g1058 . . . . . . .
. . . . . . . .
. . . . .
Vv09g1059 . . . . . . .
. . . . . . . .
. . . . .
Vv09g1060 . . . . . . .
. . . . . . . .
. . . . .
Vv09g1061 . . . . . . .
. . . . . . . .
. . . . .
Vv09g1062 . . . . . . .
. . . . . . . .
. . . . .
Vv09g1063 . . . . . . .

| | | | | | | | |
|---|---|---|---|---|---|---|---|
| . | . | . | . | . | . | . | . |
| . | . | . | . | . | | | |
| Vv09g1064 | . | . | . | . | . | . | . |
| . | . | . | . | . | . | . | . |
| . | . | . | . | . | | | |
| Vv09g1065 | . | . | . | . | . | . | . |
| . | . | . | . | . | . | . | . |
| . | . | . | . | . | | | |
| Vv09g1066 | . | . | . | . | . | . | . |
| . | . | . | . | . | . | . | . |
| . | . | . | . | . | | | |
| Vv09g1067 | . | . | . | . | . | . | . |
| . | . | . | . | . | . | . | . |
| . | . | . | . | . | | | |
| Vv09g1068 | . | . | . | . | . | . | . |
| . | . | . | . | . | . | . | . |
| . | . | . | . | . | | | |
| Vv09g1069 | . | . | . | . | Gorai.011G000500.1 | | |
| . | . | . | . | . | . | . | . |
| . | . | . | . | . | . | . | |
| Vv09g1070 | . | . | . | . | . | . | . |
| . | . | . | . | . | . | . | . |
| . | . | . | . | . | | | |
| Vv09g1071 | . | . | . | . | . | . | . |
| . | . | . | . | . | . | . | . |
| . | . | . | . | . | | | |
| Vv09g1072 | . | . | . | . | . | . | . |
| . | . | . | . | . | . | . | . |
| . | . | . | . | . | | | |
| Vv09g1073 | . | . | . | . | . | . | . |
| . | . | . | . | . | . | . | . |
| . | . | . | . | . | | | |
| Vv09g1074 | . | . | . | . | . | . | . |
| . | . | . | . | . | . | . | . |
| . | . | . | . | . | | | |
| Vv09g1075 | . | . | . | . | . | . | . |
| . | . | . | . | . | . | . | . |
| . | . | . | . | . | | | |
| Vv09g1076 | . | . | . | . | . | . | . |
| . | . | . | . | . | . | . | . |
| . | . | . | . | . | | | |
| Vv09g1077 | . | . | . | . | . | . | . |
| . | . | . | . | . | . | . | . |
| . | . | . | . | . | | | |
| Vv09g1078 | . | . | . | Gorai.009G118800.1 | | | . |
| . | . | . | . | . | . | . | . |
| . | . | . | . | . | . | . | |
| Vv09g1079 | . | . | . | . | . | . | . |
| . | . | . | . | . | . | . | . |
| . | . | . | . | . | | | |

Vv09g1080 . . . . . . .
. . . . . . . .
. . . . .
Vv09g1081 . . . . . . .
. . . . . . . .
. . . . .
Vv09g1082 . . . . . . .
. . . . . . . .
. . . . .
Vv09g1083 . . . . . . .
. . . . . . . .
. . . . .
Vv09g1084 . . . . . . .
. . . . . . . .
. . . . .
Vv09g1085 . . . . . . .
. . . . . . . .
. . . . .
Vv09g1086 . . . . . . .
. . . . . . . .
. . . . .
Vv09g1087 . . . . . . .
. . . . . . . .
. . . . .
Vv09g1088 Tc06_g006030 . . . . .
. . . . . . . .
. . . . . .
Vv09g1089 . . . . Gorai.011G002000.1
Gorai.010G141300.1 . . . . .
. . . . . . . .
.
Vv09g1090 . . . . . . .
. . . . . . . .
. . . . .
Vv09g1091 . . . . . . .
. . . . . . . .
. . . . .
Vv09g1092 Tc06_g005130 . . . . .
. . . . . . . .
. . . . . .
Vv09g1093 Tc06_g005150 . . . Gorai.
011G002200.1 Gorai.010G141400.1 . . . .
. . . . . . . .
. .
Vv09g1094 . . . . . . .
. . . . . . . .
. . . . .
Vv09g1095 Tc06_g005160 . . . .
Gorai.010G141600.1 . . . . . .
. . . . . . . .

Vv09g1096 Tc06_g005220 . . . . .
. . . . . . . .
. . . . . .
Vv09g1097 Tc06_g005250 . . . . .
. . . . . . . .
. . . . . .
Vv09g1098 Tc06_g005260 . . . . .
. . . . . . . .
. . . . . .
Vv09g1099 . . . . . . .
. . . . . . . .
. . . . .
Vv09g1100 Tc06_g005270 . . . . .
. . . . . . . .
. . . . . .
Vv09g1101 . . . . . . .
. . . . . . . .
. . . . .
Vv09g1102 Tc06_g005330 . . . Gorai.
011G002300.1 . . . . . . .
. . . . . . . .
Vv09g1103 Tc06_g005400 . . . .
Gorai.010G141800.1 . . . . . .
. . . . . . . .
Vv09g1104 . . . . . . .
. . . . . . . .
. . . . .
Vv09g1105 Tc06_g005420 . . . Gorai.
011G002500.1 . . . . . . .
. . . . . . . .
Vv09g1106 Tc06_g014990 . . . . .
. . . . . . . .
. . . . . .
Vv09g1107 Tc06_g014980 . . . . .
. . . . . . . .
. . . . . .
Vv09g1108 . . . . . . .
. . . . . . . .
. . . . .
Vv09g1109 . . . . . . .
. . . . . . . .
. . . . .
Vv09g1110 . . . . . . .
. . . . . . . .
. . . . .
Vv09g1111 . . . . . . .
. . . . . . . .
. . . . .
Vv09g1112 Tc06_g005480 . . Gorai.009G119300.1
Gorai.011G002600.1 . . . . .

. . . . . . . .
. .
Vv09g1113 . . . . . . .
. . . . . . . .
. . . . .
Vv09g1114 Tc06_g005520 . . . . .
. . . . . . . .
. . . . . .
Vv09g1115 . . . . . . .
. . . . . . . .
. . . . .
Vv09g1116 . . . . . . .
. . . . . . . .
. . . . .
Vv09g1117 . . . . Gorai.011G002700.1
. . . . . . . .
. . . . . . .
Vv09g1118 . . . . . . .
. . . . . . . .
. . . . .
Vv09g1119 . . . . . . .
. . . . . . . .
. . . . .
Vv09g1120 . . . . . . .
. . . . . . . .
. . . . .
Vv09g1121 . . . . . Gorai.
010G142500.1 . . . . . . .
. . . . . . .
Vv09g1122 . . . . . . .
. . . . . . . .
. . . . .
Vv09g1123 . . . . . . .
. . . . . . . .
. . . . .
Vv09g1124 Tc06_g006020 . . . . .
. . . . . . . .
. . . . . .
Vv09g1125 . . . . . . .
. . . . . . . .
. . . . .
Vv09g1126 Tc06_g005980 . . . . .
. . . . . . . .
. . . . . .
Vv09g1127 Tc06_g005970 . . . . .
. . . . . . . .
. . . . . .
Vv09g1128 . . . . . . .
. . . . . . . .
. . . . .

| | | | | | | | |
|---|---|---|---|---|---|---|---|
| Vv09g1129 | Tc06_g005930 | | . | . | Gorai.009G119900.1 | | |
| . | . | . | . | . | . | . | . |
| . | . | . | . | . | . | . | . |
| Vv09g1130 | Tc06_g005910 | | . | . | Gorai.009G120000.1 | | |
| . | . | . | . | . | . | . | . |
| . | . | . | . | . | . | . | . |
| Vv09g1131 | Tc06_g005880 | | . | . | . | . | . |
| . | . | . | . | . | . | . | . |
| . | . | . | . | . | . | | |
| Vv09g1132 | . | . | . | . | . | . | |
| Vv10g0249 | . | . | . | . | . | . | . |
| . | . | . | . | . | . | | |
| Vv09g1133 | . | . | . | . | . | . | . |
| . | . | . | . | . | . | . | . |
| . | . | . | . | . | | | |
| Vv09g1134 | . | . | . | . | . | . | . |
| . | . | . | . | . | . | . | . |
| . | . | . | . | . | | | |
| Vv09g1135 | . | . | . | . | . | . | . |
| . | . | . | . | . | . | . | . |
| . | . | . | . | . | | | |
| Vv09g1136 | Tc06_g005740 | | . | . | . | . | . |
| . | . | . | . | . | . | . | . |
| . | . | . | . | . | . | | |
| Vv09g1137 | . | . | . | . | . | . | . |
| . | . | . | . | . | . | . | . |
| . | . | . | . | . | | | |
| Vv09g1138 | Tc06_g005790 | | . | . | . | . | . |
| . | . | . | . | . | . | . | . |
| . | . | . | . | . | . | | |
| Vv09g1139 | Tc06_g005800 | | . | . | . | . | . |
| . | . | . | . | . | . | . | . |
| . | . | . | . | . | . | | |
| Vv09g1140 | . | . | . | . | . | . | . |
| . | . | . | . | . | . | . | . |
| . | . | . | . | . | | | |
| Vv09g1141 | . | . | . | . | . | . | . |
| . | . | . | . | . | . | . | . |
| . | . | . | . | . | | | |
| Vv09g1142 | . | . | . | . | . | . | . |
| . | . | . | . | . | . | . | . |
| . | . | . | . | . | | | |
| Vv09g1143 | . | . | . | . | . | . | . |
| . | . | . | . | . | . | . | . |
| . | . | . | . | . | | | |
| Vv09g1144 | . | . | . | . | . | . | . |
| . | . | . | . | . | . | . | . |
| . | . | . | . | . | | | |
| Vv09g1145 | . | . | . | . | . | . | . |
| . | . | . | . | . | . | . | . |

| | | | | | | | |
|---|---|---|---|---|---|---|---|
| . | . | . | . | . | | | |
| Vv09g1146 | Tc06_g005800 | | . | . | . | . | . |
| . | . | . | . | . | . | . | . |
| . | . | . | . | . | . | | |
| Vv09g1147 | . | . | . | . | . | . | . |
| . | . | . | . | . | . | . | . |
| . | . | . | . | . | | | |
| Vv09g1148 | . | . | . | . | . | . | . |
| . | . | . | . | . | . | . | . |
| . | . | . | . | . | | | |
| Vv09g1149 | Tc06_g005790 | | . | . | . | . | . |
| . | . | . | . | . | . | . | . |
| . | . | . | . | . | . | | |
| Vv09g1150 | . | . | . | . | . | . | . |
| . | . | . | . | . | . | . | . |
| . | . | . | . | . | | | |
| Vv09g1151 | . | . | . | . | . | . | . |
| . | . | . | . | . | . | . | . |
| . | . | . | . | . | | | |
| Vv09g1152 | . | . | . | . | . | . | . |
| . | . | . | . | . | . | . | . |
| . | . | . | . | . | | | |
| Vv09g1153 | . | . | . | . | . | . | . |
| . | . | . | . | . | . | . | . |
| . | . | . | . | . | | | |
| Vv09g1154 | . | . | . | . | . | . | . |
| . | . | . | . | . | . | . | . |
| . | . | . | . | . | | | |
| Vv09g1155 | . | . | . | . | . | . | . |
| . | . | . | . | . | . | . | . |
| . | . | . | . | . | | | |
| Vv09g1156 | . | . | . | . | . | . | . |
| . | . | . | . | . | . | . | . |
| . | . | . | . | . | | | |
| Vv09g1157 | . | . | . | . | . | . | . |
| . | . | . | . | . | . | . | . |
| . | . | . | . | . | | | |
| Vv09g1158 | Tc06_g003810 | | . | . | . | . | . |
| . | . | . | . | . | . | . | . |
| . | . | . | . | . | . | | |
| Vv09g1159 | . | . | . | . | . | . | . |
| . | . | . | . | . | . | . | . |
| . | . | . | . | . | | | |
| Vv09g1160 | Tc06_g003840 | | . | . | Gorai.009G120100.1 | | |
| . | . | . | . | . | . | . | . |
| . | . | . | . | . | . | . | . |
| Vv09g1161 | . | . | . | . | . | . | . |
| . | . | . | . | . | . | . | . |
| . | . | . | . | . | | | |
| Vv09g1162 | . | . | . | . | . | . | . |

| | | | | | | | |
|---|---|---|---|---|---|---|---|
| . | . | . | . | . | . | . | . |
| . | . | . | . | . | | | |
| Vv09g1163 | . | . | . | . | . | . | . |
| . | . | . | . | . | . | . | . |
| . | . | . | . | . | | | |
| Vv09g1164 | . | . | . | . | . | . | |
| Vv10g0248 | Tc06_g012600 | | . | . | . | . | . |
| . | . | . | . | . | . | . | |
| Vv09g1165 | . | . | . | . | . | . | . |
| . | . | . | . | . | . | . | . |
| . | . | . | . | . | | | |
| Vv09g1166 | . | . | . | . | . | . | . |
| . | . | . | . | . | . | . | . |
| . | . | . | . | . | | | |
| Vv09g1167 | . | . | . | . | . | . | . |
| . | . | . | . | . | . | . | . |
| . | . | . | . | . | | | |
| Vv09g1168 | . | . | . | . | . | . | . |
| . | . | . | . | . | . | . | . |
| . | . | . | . | . | | | |
| Vv09g1169 | . | . | . | . | . | . | |
| Vv10g0241 | . | . | . | . | . | . | . |
| . | . | . | . | . | . | | |
| Vv09g1170 | . | . | . | . | . | . | . |
| . | . | . | . | . | . | . | . |
| . | . | . | . | . | | | |
| Vv09g1171 | . | . | . | . | . | . | . |
| . | . | . | . | . | . | . | . |
| . | . | . | . | . | | | |
| Vv09g1172 | . | . | . | . | . | . | |
| Vv10g0240 | . | . | . | . | . | . | . |
| . | . | . | . | . | . | | |
| Vv09g1173 | . | . | . | . | . | . | . |
| . | . | . | . | . | . | . | . |
| . | . | . | . | . | | | |
| Vv09g1174 | . | . | . | . | . | . | |
| Vv10g0239 | . | . | . | . | . | . | . |
| . | . | . | . | . | . | | |
| Vv09g1175 | . | . | . | . | . | . | . |
| . | . | . | . | . | . | . | . |
| . | . | . | . | . | | | |
| Vv09g1176 | . | . | . | . | . | . | |
| Vv10g0237 | . | . | . | . | . | . | . |
| . | . | . | . | . | . | | |
| Vv09g1177 | . | . | . | . | . | . | |
| Vv10g0234 | . | . | . | . | . | . | . |
| . | . | . | . | . | . | | |
| Vv09g1178 | . | . | . | . | . | . | . |
| . | . | . | . | . | . | . | . |
| . | . | . | . | . | | | |

Vv09g1179 . . . . . . .
. . . . . . . .
. . . . .
Vv09g1180 . . . . . . .
. . . . . . . .
. . . . .
Vv09g1181 . . . . . . .
. . . . . . . .
. . . . .
Vv09g1182 . . . . . . .
. . . . . . . .
. . . . .
Vv09g1183 . . . . . . .
. . . . . . . .
. . . . .
Vv09g1184 . . . . . . .
. . . . . . . .
. . . . .
Vv09g1185 . . . . . . .
. . . . . . . .
. . . . .
Vv09g1186 Tc06_g005720 . . . . .
. . . . . . . .
. . . . . .
Vv09g1187 . . . . . . .
. . . . . . . .
. . . . .
Vv09g1188 . . . . . . .
. . . . . . . .
. . . . .
Vv09g1189 . . . . . . .
. . . . . . . .
. . . . .
Vv09g1190 . . . . . . .
. . . . . . . .
. . . . .
Vv09g1191 . . . . . . .
. . . . . . . .
. . . . .
Vv09g1192 . . . . . . .
. . . . . . . .
. . . . .
Vv09g1193 . . . . . . .
. . . . . . . .
. . . . .
Vv09g1194 . . . . . . .
. . . . . . . .
. . . . .
Vv09g1195 . . . . . . .
. . . . . . . .

| | | | | | | | |
|---|---|---|---|---|---|---|---|
| . | . | . | . | . | | | |
| Vv09g1196 | Tc06_g003870 | | . | . | . | . | . |
| . | . | . | . | . | . | . | . |
| . | . | . | . | . | . | | |
| Vv09g1197 | . | . | . | Gorai.009G120600.1 | | | . |
| . | . | . | . | . | . | . | . |
| . | . | . | . | . | . | . | |
| Vv09g1198 | . | . | . | . | . | . | . |
| . | . | . | . | . | . | . | . |
| . | . | . | . | . | | | |
| Vv09g1199 | . | . | . | Gorai.009G120700.1 | | | . |
| . | . | . | . | . | . | . | . |
| . | . | . | . | . | . | . | |
| Vv09g1200 | . | . | . | Gorai.009G120800.1 | | | . |
| . | . | . | . | . | . | . | . |
| . | . | . | . | . | . | . | |
| Vv09g1201 | . | . | . | Gorai.009G120900.1 | | | . |
| . | . | . | . | . | . | . | . |
| . | . | . | . | . | . | . | |
| Vv09g1202 | . | . | . | . | . | . | . |
| . | . | . | . | . | . | . | . |
| . | . | . | . | . | | | |
| Vv09g1203 | Tc06_g005350 | | . | . | . | . | . |
| . | . | . | . | . | . | . | . |
| . | . | . | . | . | . | | |
| Vv09g1204 | . | . | . | Gorai.009G121000.1 | | | . |
| . | . | . | . | . | . | . | . |
| . | . | . | . | . | . | . | |
| Vv09g1205 | . | . | . | . | . | . | . |
| . | . | . | . | . | . | . | . |
| . | . | . | . | . | | | |
| Vv09g1206 | . | . | . | Gorai.009G121100.1 | | | . |
| . | . | . | . | . | . | . | . |
| . | . | . | . | . | . | . | |
| Vv09g1207 | . | . | . | . | . | . | . |
| . | . | . | . | . | . | . | . |
| . | . | . | . | . | | | |
| Vv09g1208 | . | . | . | . | . | . | . |
| . | . | . | . | . | . | . | . |
| . | . | . | . | . | | | |
| Vv09g1209 | . | . | . | Gorai.009G121200.1 | | | . |
| . | . | . | . | . | . | . | . |
| . | . | . | . | . | . | . | |
| Vv09g1210 | . | . | . | Gorai.009G121300.1 | | | . |
| . | . | . | . | . | . | . | . |
| . | . | . | . | . | . | . | |
| Vv09g1211 | . | . | . | . | . | . | . |
| . | . | . | . | . | . | . | . |
| . | . | . | . | . | | | |
| Vv09g1212 | . | . | . | Gorai.009G121500.1 | | | . |

. . . . . . . .
. . . . . . .
Vv09g1213 . . . Gorai.009G121600.1 .
. . . . . . . .
. . . . . . .
Vv09g1214 . . . Gorai.009G121700.1 .
. . . . . . . .
. . . . . . .
Vv09g1215 . . . . . . .
. . . . . . . .
. . . . .
Vv09g1216 . . . . . . .
. . . . . . . .
. . . . .
Vv09g1217 . . . . . . .
. . . . . . . .
. . . . .
Vv09g1218 . . . . . . .
. . . . . . . .
. . . . .
Vv09g1219 . . . . . . .
. . . . . . . .
. . . . .
Vv09g1220 . . . . . . .
. . . . . . . .
. . . . .
Vv09g1221 . . . . . . .
. . . . . . . .
. . . . .
Vv10g0001 Tc06_g015710 . . . Gorai.
011G028300.1 . . . . . . .
. . . . . . . .
Vv10g0002 . . . . . . .
. . . . . . . .
. . . . .
Vv10g0003 . . . . . . .
. . . . . . . .
. . . . .
Vv10g0004 Tc06_g015680 . Gorai.010G154300.1
Gorai.009G168900.1 . Gorai.010G154300.1 . .
. . . . . . . .
. . . .
Vv10g0005 Tc06_g015670 . Gorai.010G154500.1
Gorai.009G168800.1 . Gorai.010G154500.1 Vv19g0246
. . . Gorai.002G069400.1 Gorai.
009G384400.1 . . . . . . .
.
Vv10g0006 Tc06_g015660 . Gorai.010G154800.1 .
. Gorai.010G154800.1 Vv12g0350 . .
. . . . . . . .

. . .
Vv10g0007 Tc06_g015650 . Gorai.010G155000.1 .
. Gorai.010G155000.1 . . . .
. . . . . . . .
. .
Vv10g0008 Tc06_g015630 . . Gorai.009G168700.1
. Gorai.010G256300.1 . . . .
. . . . . . . .
. .
Vv10g0009 Tc06_g015600 . . Gorai.009G168600.1
. Gorai.010G256400.1 . . . .
. . . . . . . .
. .
Vv10g0010 Tc06_g015580 . . . Gorai.
011G029100.1 . . . . . . .
. . . . . . . .
Vv10g0011 Tc06_g015570 . Gorai.010G155200.1
Gorai.009G168400.1 Gorai.011G029200.1 . . .
. . . . . . . .
. . . .
Vv10g0012 . . . . . . .
. . . . . . . .
. . . . .
Vv10g0013 Tc06_g015560 . . Gorai.009G168300.1
Gorai.011G029300.1 . . . . .
. . . . . . . .
. .
Vv10g0014 Tc06_g015550 . . . Gorai.
011G029400.1 . Vv12g0338 Tc01_g000650 . .
. Gorai.003G003200.1 . Vv19g0234
Tc07_g008410 . . Gorai.002G068300.1 . .
Vv10g0015 Tc06_g015540 . Gorai.010G155300.1 .
. . . . . . . .
. . . . . . . .
Vv10g0016 Tc06_g015530 . Gorai.010G155400.1 .
. . . . . . . .
. . . . . . . .
Vv10g0017 Tc06_g015520 . . . . .
. . . . . . . .
. . . . . .
Vv10g0018 . . Gorai.010G155500.1 . .
. Vv12g0337 . . . . .
. . . . . . . .
Vv10g0019 Tc06_g015510 . Gorai.010G155600.1 .
. . . . . . . .
. . . . . . . .
Vv10g0020 Tc06_g015500 . . Gorai.009G167600.1
. Gorai.010G256500.1 . . . .
. . . . . . . .
. .

Vv10g0021 Tc06_g015480 Gorai.006G087900.1 . .
Gorai.011G029600.1 . . . . .
. . . . . . . .
. .
Vv10g0022 . . . . . . .
. . . . . . . .
. . . . .
Vv10g0023 Tc06_g015470 Gorai.006G088000.1 Gorai.
010G155900.1 . Gorai.011G029800.1 Gorai.010G256600.1
. . . . . . . .
. . . . . .
Vv10g0024 Tc06_g015440 . . Gorai.009G167300.1
. Gorai.010G256700.1 . . . .
. . . . . . . .
. .
Vv10g0025 Tc06_g015420 . . . . .
. . . . . . . .
. . . . . .
Vv10g0026 Tc06_g015410 Gorai.006G088100.1 . .
. . . . . . . .
. . . . . . . .
Vv10g0027 . . . . . . .
. . . . . . Vv19g0216
. . . . . .
Vv10g0028 Tc06_g015400 . . Gorai.009G167200.1
. Gorai.010G256800.1 Vv12g0320 . .
. . . . . . . .
. . .
Vv10g0029 . . . . . . .
. . . . . . . .
. . . . .
Vv10g0030 . . . . . . .
. . . . . . . .
. . . . .
Vv10g0031 Tc06_g015350 . . Gorai.009G167000.1
Gorai.011G030000.1 . . . . .
. . . . . . . .
. .
Vv10g0032 Tc06_g015340 . . Gorai.009G166900.1
. . . . . . . .
. . . . . . . .
Vv10g0033 Tc06_g015330 . . . Gorai.
011G030100.1 . . . . . . .
. . . . . . . .
Vv10g0034 Tc06_g015320 . . Gorai.009G166800.1
. . . . . . . .
. . . . . . . .
Vv10g0035 Tc06_g015310 . . Gorai.009G166700.1
. . . . . . . .
. . . . . . . .

Vv10g0036 Tc06_g015300 . . . .
Gorai.010G256900.1 . . . . . .
. . . . . . . .
Vv10g0037 . . . . . Gorai.
010G243500.1 Vv12g0309 . . . . .
. . . . . . . .
Vv10g0038 Tc06_g015290 Gorai.006G088300.1 . .
Gorai.011G030200.1 . . . . .
. . . . . . . .
. .
Vv10g0039 . . . . . . .
. . . . . . . .
. . . . .
Vv10g0040 Tc06_g015270 . . . Gorai.
011G030300.1 . Vv12g0308 . . . .
. . . . . . . .
.
Vv10g0041 Tc06_g015260 . . Gorai.009G166500.1
Gorai.011G030400.1 . . . . .
. . . . . . . .
. .
Vv10g0042 Tc06_g015250 . . Gorai.009G166400.1
. . . . . . . .
. . . . . . . .
Vv10g0043 Tc06_g015240 Gorai.006G088400.1 .
Gorai.009G166300.1 . . Vv12g0306 . .
. . . . . . . .
. . .
Vv10g0044 Tc06_g015230 . . . Gorai.
011G030500.1 . . . . . . .
. . . . . . . .
Vv10g0045 . . . . . . .
. . . . . . . .
. . . . .
Vv10g0046 Tc06_g015210 . . Gorai.009G166200.1
Gorai.011G030700.1 . . . . .
. . . . . . . .
. .
Vv10g0047 Tc06_g015200 Gorai.006G088500.1 . .
Gorai.011G030800.1 . Vv12g0302 . .
. . . . . . . .
. . .
Vv10g0048 Tc06_g015190 . . Gorai.009G166100.1
Gorai.011G030900.1 . . . . .
. . . . . . . .
. .
Vv10g0049 Tc06_g015180 . . . .
Gorai.010G243400.1 . . . . . .
. Vv19g0199 Tc07_g008870 Gorai.013G171700.1
. . . .

Vv10g0050 . . . . . .
Vv12g0289 . . . . . . .
. . . . . .
Vv10g0051 Tc06_g015170 . . . . .
Vv12g0286 . . . . . .
. . . . . . .
Vv10g0052 . . . . . . .
. . . . . . . .
. . . . .
Vv10g0053 Tc06_g015160 . . . . .
Vv12g0284 . . . . . .
. . . . . . .
Vv10g0054 Tc06_g015150 . . . . .
Vv12g0282 . . . . . .
. . . . . . .
Vv10g0055 Tc06_g015140 . . . . .
Vv12g0277 . . . . . .
. . . . . . .
Vv10g0056 Tc06_g015120 . . Gorai.009G165900.1
. . Vv12g0275 . . . .
. . . . . . . .
.
Vv10g0057 Tc06_g015110 . . Gorai.009G165800.1
. . Vv12g0274 . . . .
. . . . . . . .
.
Vv10g0058 . . . . . . .
. . . . . . . .
. . . . .
Vv10g0059 . . . . . . .
. . . . . . . .
. . . . .
Vv10g0060 Tc06_g015090 . . . . .
. . . . . . . .
. . . . . .
Vv10g0061 Tc06_g015070 . . . Gorai.
011G031100.1 . . . . . . .
. . . . . . . .
Vv10g0062 . . . . . . .
. . . . . . . .
. . . . .
Vv10g0063 Tc06_g015050 . . . Gorai.
011G031200.1 . . . . . . .
. . . . . . . .
Vv10g0064 . . . . . . .
. . . . . . . .
. . . . .
Vv10g0065 Tc06_g015040 . . Gorai.009G165700.1
Gorai.011G031300.1 . . . . .
. . . . . . . .

. .
Vv10g0066 Tc06_g015030 . . . Gorai.
011G031400.1 Gorai.010G242900.1 Vv12g0270
Tc01_g040940 . . . . . . .
. . . . .
Vv10g0067 Tc06_g015010 . . Gorai.009G165500.1
. . . . . . . .
. . . . . . . .
Vv10g0068 Tc06_g015000 . Gorai.010G156500.1
Gorai.009G165400.1 . . . . . .
. . . . . . . .
. .
Vv10g0069 Tc06_g014990 . . Gorai.009G165300.1
. . . . . . . .
. . . . . . . .
Vv10g0070 . . . . . . .
. . . . . . . .
. . . . .
Vv10g0071 Tc06_g014980 . . Gorai.009G165200.1
. . . . . . . .
. . . . . . . .
Vv10g0072 Tc06_g014970 . . . Gorai.
011G031600.1 . . . . . . .
. . . . . . . .
Vv10g0073 Tc06_g014960 . . . Gorai.
011G031700.1 . . . . . . .
. . . . . . . .
Vv10g0074 Tc06_g014950 . . Gorai.009G165100.1
. . . . . . . .
. . . . . . . .
Vv10g0075 Tc06_g014940 . . Gorai.009G165000.1
. . . . . . . .
. . . . . . . .
Vv10g0076 . . . . Gorai.011G031800.1
Gorai.010G242700.1 . . . . .
. . Vv19g0183 . . . .
. .
Vv10g0077 . . . . . . .
. . . . . . . .
. . . . .
Vv10g0078 . . . . . . .
. . . . . . . .
. . . . .
Vv10g0079 . . . . . . .
. . . . . . . .
. . . . .
Vv10g0080 Tc06_g014930 . . . . .
. . . . . . . .
. . . . . .
Vv10g0081 Tc06_g014920 . . Gorai.009G164900.1

Gorai.011G031900.1 . . . . .
. . . Vv19g0181 . . .
. . .
Vv10g0082 . . . . Gorai.011G032000.1
. . . . . . . .
Vv19g0179 . . . . . .
Vv10g0083 . . . . . . .
. . . . . . . .
. . . . .
Vv10g0084 Tc06_g014910 . . . . .
. . . . . . .
Vv19g0178 Tc07_g009220 . . . . .
Vv10g0085 . . . . Gorai.011G032200.1
. . . . . . . .
Vv19g0176 Tc07_g009230 . . . .
.
Vv10g0086 Tc06_g014900 . . . Gorai.
011G032400.1 . . . . . . .
. . . . . . . .
Vv10g0087 Tc06_g014890 . . . . .
Vv12g0258 . . . . . .
Vv19g0175 Tc07_g009250 . . Gorai.
002G064800.1 . .
Vv10g0088 . . . . . . .
. . . . . . Vv19g0173
Tc07_g009270 . . Gorai.002G064700.1 .
Gorai.004G029900.1
Vv10g0089 Tc06_g014860 . . Gorai.009G164700.1
Gorai.011G032700.1 . . . . .
. . . Vv19g0172 Tc07_g009280 .
. Gorai.002G064600.1 . .
Vv10g0090 . . . . . .
Vv12g0255 . . . . . .
Vv19g0171 Tc07_g009290 Gorai.013G172800.1 .
Gorai.002G064500.1 Gorai.009G373000.1 .
Vv10g0091 Tc06_g014850 . . . Gorai.
011G032800.1 Gorai.010G242600.1 . . . .
. . . . . . . .
. .
Vv10g0092 Tc06_g014840 . . . .
Gorai.010G242500.1 . . . . . .
. . . . . . . .
Vv10g0093 Tc06_g014830 . . . Gorai.
011G032900.1 Gorai.010G242400.1 . . . .
. . . . . . . .
. .
Vv10g0094 . . . . . . .
. . . . . . . .
. . . . .
Vv10g0095 Tc06_g014820 . . . . .

. . . . . . . .
. . . . . .
Vv10g0096 . . . . . . .
. . . . . . . .
. . . . .
Vv10g0097 Tc06_g014810 . . . Gorai.
011G033000.1 . . . . . . .
. . . . . . . .
Vv10g0098 . . . . . . .
. . . . . . . .
. . . . .
Vv10g0099 . . . . . . .
. . . . . . . .
. . . . .
Vv10g0100 . . . . . . .
. . . . . . . .
. . . . .
Vv10g0101 . . . . . . .
. . . . . . . .
. . . . .
Vv10g0102 . . . . . . .
. . . . . . . .
. . . . .
Vv10g0103 Tc06_g014760 . . . Gorai.
011G033300.1 . . . . . . .
. . . . . . . .
Vv10g0104 Tc06_g014750 . . Gorai.009G164300.1
. . . . . . . .
. . . . . . . .
Vv10g0105 Tc06_g014740 . . Gorai.009G164200.1
. . Vv12g0251 . . . .
. . . . . . . .
.
Vv10g0106 . . . Gorai.009G164100.1 .
. . . . . . . .
. . . . . . .
Vv10g0107 Tc06_g014730 . . . Gorai.
011G033400.1 . . . . . . .
. . . . . . . .
Vv10g0108 . . . . . . .
. . . . . . . .
. . . . .
Vv10g0109 Tc06_g014720 . . . . .
. . . . . . .
Vv19g0167 Tc07_g009370 . . Gorai.002G063300.1
Gorai.009G372600.1 Gorai.004G030200.1
Vv10g0110 Tc06_g014710 . . . . .
. . . . . . . .
. . . . . .
Vv10g0111 Tc06_g014700 . . Gorai.009G164000.1

. . . . . . . .
. . . . . . . .
Vv10g0112 Tc06_g014690 . . . . .
. . . . . . . .
. . . . . .
Vv10g0113 . . . . . . .
. . . . . . . .
. . . . .
Vv10g0114 . . . . . . .
. . . . . . . .
. . . . .
Vv10g0115 . . . . . . .
. . . . . . . .
. . . . .
Vv10g0116 Tc06_g014640 . . Gorai.009G163800.1
Gorai.011G033600.1 . Vv12g0249 . .
. . . . . . . .
. . .
Vv10g0117 Tc06_g014620 Gorai.006G088700.1 Gorai.
010G158300.1 . Gorai.011G033700.1 . . .
. . . . . . . .
. . . .
Vv10g0118 Tc06_g014610 . Gorai.010G158600.1 .
. . . . . . . .
. . . . . . . .
Vv10g0119 Tc06_g014600 Gorai.006G088800.1 . .
Gorai.011G033800.1 Gorai.010G242200.1 Vv12g0242
Tc01_g013500 . . . . .
Vv19g0164 Tc07_g009400 . . . . .
Vv10g0120 . . . . . . .
. . . . . . . .
. . . . .
Vv10g0121 Tc06_g014580 . . . . .
Vv12g0233 . . . . . .
. . . . . . .
Vv10g0122 Tc06_g014560 . . . . .
Vv12g0232 . . . . . .
. . . . . . .
Vv10g0123 Tc06_g014540 . Gorai.010G158900.1 .
. . . . . . . .
. . . . . . . .
Vv10g0124 Tc06_g014520 . Gorai.010G159100.1
Gorai.009G163600.1 . . . . . .
. . . . . . . .
. .
Vv10g0125 . . . . . . .
. . . . . . . .
. . . . .
Vv10g0126 Tc06_g014510 . . Gorai.009G163500.1
. . . . . . . .

```
         .       .        .       .        .       .        .        .
Vv10g0127        Tc06_g014500     .        .       Gorai.009G163400.1
        Gorai.011G034000.1        .        .       .        .        .
         .       .        .       .        .       .        .        .
         .       .
Vv10g0128        Tc06_g014490     .        .       .        .        .
         .       .        .       .        .       .        .        .
         .       .        .       .        .       .
Vv10g0129        .        .       Gorai.010G159200.1        .        .
         .       .        .       .        .       .        .        .
         .       .        .       .        .       .        .
Vv10g0130        .        .       .        .       .        .        .
         .       .        .       .        .       .        .        .
         .       .        .       .        .
Vv10g0131        Tc06_g014450     .        .       Gorai.009G163100.1
         .       Gorai.010G242000.1        Vv12g0225
Tc01_g013620     .        .       .        .       .        .        .
         .       .        .       .        .
Vv10g0132        Tc06_g014440     Gorai.006G088900.1        .
Gorai.009G162900.1        .       .        Vv12g0220
Tc01_g001320     .        .       .        .       .        .        .
         .       .        .       .        .
Vv10g0133        Tc06_g014430     .        .       .        .
Gorai.010G241900.1        .       .        .       .        .        .
         .       .        .       .        .       .        .        .
Vv10g0134        Tc06_g014420     .        .       Gorai.009G162800.1
         .       .        .       .        .       .        .        .
         .       Vv19g0146        .        .       .        .        .
         .
Vv10g0135        .        .       .        .       .        .        .
         .       .        .       .        .       .        .        .
         .       .        .       .        .
Vv10g0136        Tc06_g014410     .        .       Gorai.009G162700.1
         .       .        .       .        .       .        .        .
         .       .        .       .        .       .        .        .
Vv10g0137        Tc06_g014380     .        Gorai.010G159300.1
Gorai.009G162500.1        Gorai.011G040200.1       .        .        .
         .       .        .       .        .       .        .        .
         .       .        .       .
Vv10g0138        Tc06_g014370     .        .       Gorai.009G162400.1
        Gorai.011G040000.1        .        .       .        .        .
         .       .        .       .        .       .        .        .
         .       .
Vv10g0139        .        .       .        .       .        .        .
         .       .        .       .        .       .        .        .
         .       .        .       .        .
Vv10g0140        Tc06_g014340     .        .       Gorai.009G162300.1
        Gorai.011G039700.1        .        .       .        .        .
         .       .        .       .        .       .        .        .
         .       .
```

Vv10g0141 Tc06_g014330 . . Gorai.009G162200.1
Gorai.011G039600.1 . Vv12g0216 . .
. . . . . . . .
. . .
Vv10g0142 Tc06_g014320 . Gorai.010G159400.1
Gorai.009G162100.1 Gorai.011G039500.1 . . .
. . . . . . . .
. . . .
Vv10g0143 Tc06_g014310 . . . Gorai.
011G039400.1 . . . . . . .
. . . . . . . .
Vv10g0144 Tc06_g014290 . . Gorai.009G162000.1
. . . . . . . .
. . . . . . . .
Vv10g0145 . . . . . . .
. . . . . . . .
. . . . .
Vv10g0146 . . . . . . .
. . . . . . . .
. . . . .
Vv10g0147 Tc06_g014280 . Gorai.010G159600.1 .
Gorai.011G034200.1 . . . . .
. . . . . . . .
. .
Vv10g0148 . . . . . . .
. . . . . . . .
. . . . .
Vv10g0149 Tc06_g014260 . . Gorai.009G161900.1
Gorai.011G039100.1 . Vv12g0212
Tc01_g013820 . . . . . . .
. . . . .
Vv10g0150 Tc06_g014250 . . . . .
. . . . . . . .
. . . . . .
Vv10g0151 . . Gorai.010G159700.1 Gorai.
009G161800.1 Gorai.011G039000.1 . Vv12g0209 .
. . . . . . . .
. . . .
Vv10g0152 Tc06_g014230 . . . . .
Vv12g0208 . . . . . .
. . . . . . .
Vv10g0153 Tc06_g014220 Gorai.006G089100.1 . .
. Gorai.010G241800.1 . . . .
. . . . . . . .
. .
Vv10g0154 Tc06_g014210 . . . Gorai.
011G038900.1 . . . . . . .
. Vv19g0126 . . . . .
.
Vv10g0155 Tc06_g014190 Gorai.006G089200.1 Gorai.

010G159800.1 . . . . . . .
. . . . . . . .
. .
Vv10g0156 Tc06_g014180 . . Gorai.009G161600.1
. Gorai.010G241700.1 . . . .
. . . . . . . .
. .
Vv10g0157 . . . . . . .
. . . . . . . .
. . . . .
Vv10g0158 Tc06_g014170 . Gorai.010G160000.1 .
. . . . . . . .
. . . . . . . .
Vv10g0159 . . . . . . .
. . . . . . . .
. . . . .
Vv10g0160 Tc06_g014160 . . . Gorai.
011G038700.1 . . . . . . .
. . . . . . . .
Vv10g0161 Tc06_g014150 . . Gorai.009G161500.1
Gorai.011G038600.1 . . . . .
. . . . . . . .
. .
Vv10g0162 Tc06_g014140 . . Gorai.009G161400.1
. . . . . . . .
. . . . . . . .
Vv10g0163 . . . . . . .
. . . . . . . .
. . . . .
Vv10g0164 . . . . . . .
. . . . . . . .
. . . . .
Vv10g0165 Tc06_g014120 Gorai.006G089400.1 . .
. Gorai.010G241600.1 . . . .
. . . . . . . .
. .
Vv10g0166 Tc06_g014110 . . . . .
. . . . . . . .
. . . . . .
Vv10g0167 Tc06_g014100 Gorai.006G089600.1 . .
Gorai.011G038300.1 Gorai.010G241500.1 . .
. . . . . . . .
. . . .
Vv10g0168 . . . . . . .
. . . . . . . .
. . . . .
Vv10g0169 Tc06_g014090 Gorai.006G089700.1 Gorai.
010G160100.1 Gorai.009G161300.1 Gorai.011G038200.1
Gorai.010G241400.1 Vv12g0178 . . . .
. . . . . . . .

.
Vv10g0170 Tc06_g014080 . Gorai.010G160200.1 .
. . . . . . . .
. . . . . . . .
Vv10g0171 Tc06_g014070 . . . . .
. . . . . . . .
. . . . . .
Vv10g0172 Tc06_g014060 . Gorai.010G160400.1 .
Gorai.011G038000.1 . . . . .
. . . . . . . .
. .
Vv10g0173 Tc06_g014050 . . Gorai.009G161200.1
. . . . . . . .
. . . . . . . .
Vv10g0174 Tc06_g014040 . . . Gorai.
011G037800.1 . . . . . . .
. . . . . . . .
Vv10g0175 Tc06_g014020 . Gorai.010G160700.1
Gorai.009G161100.1 . . Vv12g0172 . .
. . . . . . . .
. . .
Vv10g0176 Tc06_g014000 . Gorai.010G160900.1
Gorai.009G160900.1 Gorai.011G037600.1 . . .
. . . . . . . .
. . . .
Vv10g0177 . . . . . . .
. . . . . . . .
. . . . .
Vv10g0178 Tc06_g013990 . Gorai.010G161000.1
Gorai.009G160800.1 Gorai.011G037500.1 . Vv12g0170
Tc01_g014750 . . . . . .
. . . . . .
Vv10g0179 Tc06_g013980 . . Gorai.009G160700.1
. Gorai.010G241300.1 . . . .
. . . . . . . .
. .
Vv10g0180 Tc06_g013960 . Gorai.010G161300.1
Gorai.009G160600.1 . . . . . .
. . . . . . . .
. .
Vv10g0181 Tc06_g013950 . Gorai.010G161400.1 .
. . . . . . . .
. . . . . . . .
Vv10g0182 Tc06_g013910 . . . Gorai.
011G037100.1 . . . . . . .
. . . . . . . .
Vv10g0183 Tc06_g013530 . . Gorai.009G160400.1
Gorai.011G037000.1 Gorai.010G241200.1 Vv12g0169
. . . . . . . .
. . . . .

Vv10g0184 . . . Gorai.009G160300.1
Gorai.011G036900.1 Gorai.010G241100.1 . . .
. . . . Vv19g0109 . .
. Gorai.002G050200.1 . .
Vv10g0185 . . . Gorai.009G160100.1
Gorai.011G036800.1 . . . . . .
. . . . . . . .
.
Vv10g0186 . . . Gorai.009G160000.1
Gorai.011G036700.1 . . . . . .
. . Vv19g0105 . . .
Gorai.002G050900.1 . .
Vv10g0187 . Gorai.006G089800.1 . .
Gorai.011G036600.1 Gorai.010G240900.1 . . .
. . . . . . . .
. . .
Vv10g0188 . Gorai.006G089900.1 Gorai.010G161700.1
. Gorai.011G036500.1 . . . .
. . . . . . . .
. . .
Vv10g0189 . . . . . . .
. . . . . . . .
. . . . .
Vv10g0190 . . . . . . .
. . . . . . . .
. . . . .
Vv10g0191 . . . . . . .
. . . . . . . .
. . . . .
Vv10g0192 . . . . Gorai.011G036400.1
Gorai.010G240800.1 Vv12g0142 . . .
. . . . . . . .
. .
Vv10g0193 . . . . . . .
. . . . . . . .
. . . . .
Vv10g0194 . . . . Gorai.011G036300.1
Gorai.010G240700.1 Vv19g0089 . . .
Gorai.002G052400.1 . . . . .
. . . .
Vv10g0195 . . . . Gorai.011G036200.1
Gorai.010G240600.1 Vv19g0088 . . .
Gorai.002G052500.1 . . . . .
. . . .
Vv10g0196 . . . . . Gorai.
010G240500.1 . . . . . . .
. . . . . . .
Vv10g0197 Tc06_g013270 . Gorai.010G162300.1 .
Gorai.011G036000.1 . . . . .
. . . . . . . .

```
        .        .
Vv10g0198        .       .        Gorai.010G162400.1       .
Gorai.011G035800.1       Gorai.010G240400.1        .       .        .
        .        .       .        .       .        .       .        .
        .        .       .
Vv10g0199        .       .        .       .        Gorai.011G035700.1
        Gorai.010G240300.1        Vv19g0086        .       .        .
        .        .       .        .       .        .       .        .
        .        .
Vv10g0200        .       .        .       .        .       .        .
        .        .       .        .       .        .       .        .
        .        .       .        .       .
Vv10g0201        .       .        .       .        .       .        .
        .        .       .        .       .        .       .        .
        .        .       .        .       .
Vv10g0202        .       .        .       .        .       .        .
        .        .       .        .       .        .       .        .
        .        .       .        .       .
Vv10g0203        .       .        .       .        .       .        .
        .        .       .        .       .        .       .        .
        .        .       .        .       .
Vv10g0204        .       .        .       .        .       .        .
        .        .       .        .       .        .       .        .
        .        .       .        .       .
Vv10g0205        .       .        .       .        .       .        .
        .        .       .        .       .        .       .        .
        .        .       .        .       .
Vv10g0206        Tc06_g013030     .       .        .       .        .
        .        .       .        .       .        .       .        .
        .        .       .        .       .        .
Vv10g0207        .       .        .       .        .       .        .
        .        .       .        .       .        .       .        .
        .        .       .        .       .
Vv10g0208        .       .        .       .        .       .        .
        .        .       .        .       .        .       .        .
        .        .       .        .       .
Vv10g0209        .       .        .       .        .       .        .
        .        .       .        .       .        .       .        .
        .        .       .        .       .
Vv10g0210        .       .        .       .        .       .        .
        .        .       .        .       .        .       .        .
        .        .       .        .       .
Vv10g0211        .       .        .       .        .       .        .
        .        .       .        .       .        .       .        .
        .        .       .        .       .
Vv10g0212        .       .        .       .        .       .        .
        .        .       .        .       .        .       .        .
        .        .       .        .       .
Vv10g0213        .       .        .       .        .       .        .
        .        .       .        .       .        .       .        .
```

. . . . .
Vv10g0214 . . . . . . .
. . . . . . . .
. . . . .
Vv10g0215 . . . . . . .
. . . . . . . .
. . . . .
Vv10g0216 . . . . . . .
. . . . . . . .
. . . . .
Vv10g0217 . . . . . . .
. . . . . . . .
. . . . .
Vv10g0218 . . . . . . .
. . . . . . . .
. . . . .
Vv10g0219 . . . Gorai.009G159000.1
Gorai.011G032700.1 Gorai.010G237400.1 . . .
. . . . . . . .
. . .
Vv10g0220 . . . . . . .
. . . . . . . .
. . . . .
Vv10g0221 . . . . . . .
. . . . . . . .
. . . . .
Vv10g0222 . . Gorai.010G163400.1 . .
. . . . . . . .
. . . . . . .
Vv10g0223 . . Gorai.010G163600.1 . .
. . . . . . . .
. . . . . . .
Vv10g0224 . . . . . . .
. . . . . . . .
. . . . .
Vv10g0225 . . . . . . .
. . . . . . . .
. . . . .
Vv10g0226 . . . . . . .
. . . . . . . .
. . . . .
Vv10g0227 . . . . . . .
. . . . . . . .
. . . . .
Vv10g0228 . . . . . . .
. . . . . . . .
. . . . .
Vv10g0229 Tc06_g012640 . . . Gorai.
011G043800.1 . . . . . . .
. . . . . . . .

Vv10g0230 . . . . . . .
. . . . . . . .
. . . . .
Vv10g0231 Tc06_g012630 . Gorai.010G166700.1 .
Gorai.011G044200.1 . . . . .
. . . . . . . .
. .
Vv10g0232 Tc06_g012620 . . Gorai.009G154900.1
Gorai.011G044400.1 . . . . .
. . . . . . . .
. .
Vv10g0233 . . . . . . .
. . . . . . . .
. . . . .
Vv10g0234 . . . . . .
Vv09g1177 . . . . . . .
. . . . . .
Vv10g0235 . . . . Gorai.011G044500.1
. . . . . . . .
. . . . . . .
Vv10g0236 . . . . . . .
. . . . . . . .
. . . . .
Vv10g0237 . . . . . .
Vv09g1176 . . . . . . .
. . . . . .
Vv10g0238 . . . . . . .
. . . . . . . .
. . . . .
Vv10g0239 . . . . . .
Vv09g1174 . . . . . . .
. . . . . .
Vv10g0240 . . . . . .
Vv09g1172 . . . . . . .
. . . . . .
Vv10g0241 . . . . . .
Vv09g1169 . . . . . . .
. . . . . .
Vv10g0242 . . . . . . .
. . . . . . . .
. . . . .
Vv10g0243 . . . . . . .
. . . . . . . .
. . . . .
Vv10g0244 . . . . . . .
. . . . . . . .
. . . . .
Vv10g0245 . . . . . . .
. . . . . . . .
. . . . .

Vv10g0246 . . . . . . .
. . . . . . . .
. . . . .
Vv10g0247 . . . . . . .
. . . . . . . .
. . . . .
Vv10g0248 Tc06_g012600 . . . . .
Vv09g1164 . . . . . .
. . . . . . .
Vv10g0249 . . . . . .
Vv09g1132 . . . . . . .
. . . . . .
Vv10g0250 Tc06_g012590 . . . Gorai.
011G044600.1 . . . . . . .
. . . . . . . .
Vv10g0251 Tc06_g012580 . . Gorai.009G154800.1
Gorai.011G044700.1 . . . . .
. . . . . . . .
. .
Vv10g0252 . . . . . . .
. . . . . . . .
. . . . .
Vv10g0253 . . . . . . .
. . . . . . . .
. . . . .
Vv10g0254 . . . . . . .
. . . . . . . .
. . . . .
Vv10g0255 Tc06_g012540 . . . Gorai.
011G044900.1 . . . . . . .
. . . . . . . .
Vv10g0256 Tc06_g012530 . . . . .
. . . . . . . .
. . . . . .
Vv10g0257 Tc06_g012520 Gorai.006G092600.1 . .
Gorai.011G045000.1 . . . . .
. . . . . . . .
. .
Vv10g0258 Tc06_g012500 . Gorai.010G166900.1
Gorai.009G154600.1 Gorai.011G045200.1 . . .
. . . . . . . .
. . . .
Vv10g0259 . . . . . . .
. . . . . . . .
. . . . .
Vv10g0260 Tc06_g012490 . Gorai.010G167000.1
Gorai.009G154500.1 . . . . . .
. . . . . . . .
. .
Vv10g0261 . . . Gorai.009G154400.1 .

. . . . . . . .
. . . . . . .
Vv10g0262 . Gorai.006G092400.1 . Gorai.
009G154300.1 . . . . . . .
. . . . . . . .
.
Vv10g0263 Tc06_g012480 . . . . .
. . . . . . . .
. . . . . .
Vv10g0264 . . . . . . .
. . . . . . . .
. . . . .
Vv10g0265 . . . . Gorai.011G045300.1
. . . . . . . .
. . . . . . .
Vv10g0266 Tc06_g012460 . . . . .
. . . . . . . .
. . . . . .
Vv10g0267 . . . . . . .
. . . . . . . .
. . . . .
Vv10g0268 . . . . . . .
. . . . . . . .
. . . . .
Vv10g0269 Tc06_g012450 Gorai.006G092300.1 .
Gorai.009G154200.1 . . . . . .
. . . . . . . .
. .
Vv10g0270 Tc06_g012440 . . . Gorai.
011G045500.1 . . . . . . .
. . . . . . . .
Vv10g0271 Tc06_g012410 . . . Gorai.
011G045700.1 . . . . . . .
. . . . . . . .
Vv10g0272 . . . . . . .
. . . . . . . .
. . . . .
Vv10g0273 . . . . . . .
. . . . . . . .
. . . . .
Vv10g0274 Tc06_g012390 Gorai.006G092200.1 . .
. . . . . . . .
. . . . . . . .
Vv10g0275 Tc06_g012380 Gorai.006G089600.1 Gorai.
010G167200.1 . . . . . . .
. . . . . . . .
. .
Vv10g0276 . . . . . . .
. . . . . . . .
. . . . .

Vv10g0277 Tc06_g012370 . Gorai.010G167400.1
Gorai.009G154000.1 Gorai.011G045900.1 Gorai.010G238100.1
. . . . . . . .
. . . . . .
Vv10g0278 Tc06_g012360 . . Gorai.009G153900.1
Gorai.011G046000.1 . . . . .
. . . . . . . .
. .
Vv10g0279 Tc06_g012350 . . Gorai.009G153800.1
Gorai.011G046200.1 . . . . .
. . . . . . . .
. .
Vv10g0280 Tc06_g012340 . . Gorai.009G153700.1
Gorai.011G046300.1 . . . . .
. . . . . . . .
. .
Vv10g0281 Tc06_g012320 . . Gorai.009G153500.1
Gorai.011G046400.1 . . . . .
. . . . . . . .
. .
Vv10g0282 Tc06_g012310 . Gorai.010G167600.1
Gorai.009G153400.1 . . . . . .
. . . . . . . .
. .
Vv10g0283 Tc06_g012300 . . . Gorai.
011G046500.1 Gorai.010G238000.1 . . . .
. . . . . . . .
. .
Vv10g0284 . . . . . . .
. . . . . . . .
. . . . .
Vv10g0285 Tc06_g012280 . . . Gorai.
011G046700.1 . . . . . . .
. . . . . . . .
Vv10g0286 Tc06_g012270 . . . Gorai.
011G047100.1 Gorai.010G237900.1 . . . .
. . . . . . . .
. .
Vv10g0287 Tc06_g012220 . . Gorai.009G153000.1
. . . . . . . .
. . . . . . . .
Vv10g0288 . . . . . . .
. . . . . . . .
. . . . .
Vv10g0289 Tc06_g012200 . Gorai.010G168100.1 .
Gorai.011G047700.1 . . . . .
. . . . . . . .
. .
Vv10g0290 . . . . . . .
. . . . . . . .

. . . . .
Vv10g0291 Tc06_g012190 . Gorai.010G168200.1 .
Gorai.011G047800.1 . . . . .
. . . . . . . .
. .
Vv10g0292 Tc06_g012180 . . . Gorai.
011G047900.1 Gorai.010G237700.1 . . . .
. . . . . . . .
. .
Vv10g0293 Tc06_g012160 . . Gorai.009G152900.1
. Gorai.010G237600.1 . . . .
. . . . . . . .
. .
Vv10g0294 Tc06_g012150 . . . Gorai.
011G048000.1 . . . . . . .
. . . . . . . .
Vv10g0295 Tc06_g012140 . . . . .
. . . . . . . .
. . . . . .
Vv10g0296 Tc06_g012130 . Gorai.010G168300.1 .
. Gorai.010G237500.1 . . . .
. . . . . . . .
. .
Vv10g0297 Tc06_g012120 . . . .
Gorai.010G237400.1 . . . . . .
. . . . . . . .
Vv10g0298 Tc06_g012110 . . Gorai.009G152800.1
Gorai.011G048100.1 . . . . .
. . . . . . . .
. .
Vv10g0299 Tc06_g012100 . . Gorai.009G152700.1
Gorai.011G048200.1 . . . . .
. . . . . . . .
. .
Vv10g0300 Tc06_g012090 . . Gorai.009G152600.1
. . . . . . . .
. . . . . . . .
Vv10g0301 . . . . . . .
. . . . . . . .
. . . . .
Vv10g0302 Tc06_g012080 . . . Gorai.
011G048300.1 . . . . . . .
. . . . . . . .
Vv10g0303 . . . . . . .
. . . . . . . .
. . . . .
Vv10g0304 . . . . . . .
. . . . . . . .
. . . . .
Vv10g0305 . . . . . . .

. . . . . . . .
. . . . .
Vv10g0306 . . . . . . .
. . . . . . . .
. . . . .
Vv10g0307 Tc06_g012070 . Gorai.010G168400.1
Gorai.009G152500.1 . Gorai.010G234500.1 . .
. . . . . . . .
. . . .
Vv10g0308 . . . . . . .
. . . . . . . .
. . . . .
Vv10g0309 Tc06_g012060 . . . . .
. . . . . . . .
. . . . . .
Vv10g0310 Tc06_g012050 . . Gorai.009G152400.1
Gorai.011G048400.1 . . . . .
. . . . . . . .
. .
Vv10g0311 . . . . . . .
. . . . . . . .
. . . . .
Vv10g0312 . . . . . . .
. . . . . . . .
. . . . .
Vv10g0313 . . . . . . .
. . . . . . . .
. . . . .
Vv10g0314 . . . . . . .
. . . . . . . .
. . . . .
Vv10g0315 . . . . . . .
. . . . . . . .
. . . . .
Vv10g0316 . . . . . . .
. . . . . . . .
. . . . .
Vv10g0317 . . . . . . .
. . . . . . . .
. . . . .
Vv10g0318 . . . . . . .
. . . . . . . .
. . . . .
Vv10g0319 Tc06_g002530 . . . . .
. . . . . . . .
. . . . . .
Vv10g0320 . . . . . . .
. . . . . . . .
. . . . .
Vv10g0321 . . . . . . .

| | | | | | | | |
|---|---|---|---|---|---|---|---|
| . | . | . | . | . | . | . | . |
| . | . | . | . | . | | | |
| Vv10g0322 | . | . | . | . | . | . | . |
| . | . | . | . | . | . | . | . |
| . | . | . | . | . | | | |
| Vv10g0323 | . | . | . | . | . | . | . |
| . | . | . | . | . | . | . | . |
| . | . | . | . | . | | | |
| Vv10g0324 | . | . | . | . | . | . | . |
| . | . | . | . | . | . | . | . |
| . | . | . | . | . | | | |
| Vv10g0325 | . | . | . | . | . | . | . |
| . | . | . | . | . | . | . | . |
| . | . | . | . | . | | | |
| Vv10g0326 | Tc06_g002590 | | . | . | Gorai.009G117800.1 | | |
| . | . | . | . | . | . | . | . |
| . | . | . | . | . | . | . | . |
| Vv10g0327 | Tc06_g011990 | | . | . | Gorai.009G151900.1 | | |
| . | . | . | . | . | . | . | . |
| . | . | . | . | . | . | . | . |
| Vv10g0328 | . | . | . | . | . | . | . |
| . | . | . | . | . | . | . | . |
| . | . | . | . | . | | | |
| Vv10g0329 | . | . | . | . | . | . | . |
| . | . | . | . | . | . | . | . |
| . | . | . | . | . | | | |
| Vv10g0330 | . | . | . | . | . | . | . |
| . | . | . | . | . | . | . | . |
| . | . | . | . | . | | | |
| Vv10g0331 | . | . | . | . | . | . | . |
| . | . | . | . | . | . | . | . |
| . | . | . | . | . | | | |
| Vv10g0332 | . | . | . | . | . | . | . |
| . | . | . | . | . | . | . | . |
| . | . | . | . | . | | | |
| Vv10g0333 | . | . | . | . | . | . | . |
| . | . | . | . | . | . | . | . |
| . | . | . | . | . | | | |
| Vv10g0334 | . | . | . | . | . | . | . |
| . | . | . | . | . | . | . | . |
| . | . | . | . | . | | | |
| Vv10g0335 | . | . | . | . | . | . | . |
| . | . | . | . | . | . | . | . |
| . | . | . | . | . | | | |
| Vv10g0336 | . | . | . | . | . | . | . |
| . | . | . | . | . | . | . | . |
| . | . | . | . | . | | | |
| Vv10g0337 | . | . | . | . | . | . | . |
| . | . | . | . | . | . | . | . |
| . | . | . | . | . | | | |

Vv10g0338 . . . . . . .
. . . . . . . .
. . . . .
Vv10g0339 . . . . . . .
. . . . . . . .
. . . . .
Vv10g0340 . . . . . . .
. . . . . . . .
. . . . .
Vv10g0341 . . . . . . .
. . . . . . . .
. . . . .
Vv10g0342 Tc06_g008660 . . . . .
. . . . . . . .
. . . . . .
Vv10g0343 . . . . . . .
. . . . . . . .
. . . . .
Vv10g0344 . . . . . . .
. . . . . . . .
. . . . .
Vv10g0345 . . . . . . .
. . . . . . . .
. . . . .
Vv10g0346 . . . . . . .
. . . . . . . .
. . . . .
Vv10g0347 . . . . . . .
. . . . . . . .
. . . . .
Vv10g0348 . . . . . . .
. . . . . . . .
. . . . .
Vv10g0349 . . . . . . .
. . . . . . . .
. . . . .
Vv10g0350 . . . . . . .
. . . . . . . .
. . . . .
Vv10g0351 . . . . . . .
. . . . . . . .
. . . . .
Vv10g0352 Tc06_g002650 . . . . .
. . . . . . . .
. . . . . .
Vv10g0353 . . . . . . .
. . . . . . . .
. . . . .
Vv10g0354 . . . . . . .
. . . . . . . .

. . . . .
Vv10g0355 . . . . . . .
. . . . . . . .
. . . . .
Vv10g0356 . . . . . . .
. . . . . . . .
. . . . .
Vv10g0357 . . . . . . .
. . . . . . . .
. . . . .
Vv10g0358 . . . . . . .
. . . . . . . .
. . . . .
Vv10g0359 . . . . . . .
. . . . . . . .
. . . . .
Vv10g0360 . . . . . . .
. . . . . . . .
. . . . .
Vv10g0361 . . . . . . .
. . . . . . . .
. . . . .
Vv10g0362 . . . . . . .
. . . . . . . .
. . . . .
Vv10g0363 . . . . Gorai.011G004000.1
. . . . . . . .
. . . . . . .
Vv10g0364 Tc06_g002770 . . Gorai.009G117200.1
. Gorai.010G142800.1 . . . .
. . . . . . . .
. .
Vv10g0365 . . . . Gorai.011G004100.1
. . . . . . . .
. . . . . . .
Vv10g0366 . . . . . . .
. . . . . . . .
. . . . .
Vv10g0367 . . . . . . .
. . . . . . . .
. . . . .
Vv10g0368 . . . . . . .
. . . . . . . .
. . . . .
Vv10g0369 Tc06_g008310 . . Gorai.009G135100.1
Gorai.011G004200.1 . . . . .
. . . . . . . .
. .
Vv10g0370 . . . . . . .
. . . . . . . .

| | | | | | | | |
|---|---|---|---|---|---|---|---|
| Vv10g0371 | . | . | . | . | . | . | . |
| Vv10g0372 | . | . | . | . | . | . | . |
| Vv10g0373 | . | . | . | . | . | . | . |
| Vv10g0374 | Tc06_g000790 | | . | . | . | . | . |
| Vv10g0375 | Tc06_g000780 | | . | . | . | . | . |
| Vv10g0376 | . | . | . | . | . | . | . |
| Vv10g0377 | . | . | . | . | . | . | . |
| Vv10g0378 | . | . | . | . | . | . | . |
| Vv10g0379 | . | . | . | . | . | . | . |
| Vv10g0380 | . | . | . | . | . | . | . |
| Vv10g0381 | . | . | . | . | . | . | . |
| Vv10g0382 | . | . | . | . | . | . | . |
| Vv10g0383 | . | . | . | . | . | . | . |
| Vv10g0384 | Tc06_g018320 | | . | . | . | . | . |
| Vv10g0385 | . | . | . | . | . | . | . |
| Vv10g0386 | . | . | . | . | . | . | . |
| Vv10g0387 | . | . | . | . | . | . | . |

. . . . . . . .
. . . . .
Vv10g0388 . . . . . . .
. . . . . . . .
. . . . .
Vv10g0389 . . . . . . .
. . . . . . . .
. . . . .
Vv10g0390 . . . . . . .
. . . . . . . .
. . . . .
Vv10g0391 . . . . . . .
. . . . . . . .
. . . . .
Vv10g0392 . . . . . . .
. . . . . . . .
. . . . .
Vv10g0393 . . . . Gorai.011G004500.1
. . . . . . . .
. . . . . . .
Vv10g0394 . . . . . . .
. . . . . . . .
. . . . .
Vv10g0395 . . . . . . .
. . . . . . . .
. . . . .
Vv10g0396 . . . . Gorai.011G004700.1
. . . . . . . .
. . . . . . .
Vv10g0397 . . . . . . .
. . . . . . . .
. . . . .
Vv10g0398 . . . . . . .
. . . . . . . .
. . . . .
Vv10g0399 Tc06_g000510 . . Gorai.009G115900.1
. . . . . . . .
. . . . . . . .
Vv10g0400 Tc06_g000500 . . Gorai.009G115800.1
. . . . . . . .
. . . . . . . .
Vv10g0401 . . . . . . .
. . . . . . . .
. . . . .
Vv10g0402 Tc06_g000480 . . . Gorai.
011G005000.1 . . . . . . .
. . . . . . . .
Vv10g0403 Tc06_g000470 . . . . .
. . . . . . . .
. . . . . .

| | | | | | | | | | | | | | | | | | | | | |
|---|---|---|---|---|---|---|---|---|---|---|---|---|---|---|---|---|---|---|---|---|
| Vv10g0404 | Tc06_g000460 | . | . | . | . | . | . | . | . | . | . | . | . | . | . | . | . | . | . | . |
| Vv10g0405 | . | . | . | . | . | . | . | . | . | . | . | . | . | . | . | . | . | . | . | . |
| Vv10g0406 | . | . | . | . | . | . | . | . | . | . | . | . | . | . | . | . | . | . | . | . |
| Vv10g0407 | . | . | . | . | . | . | . | . | . | . | . | . | . | . | . | . | . | . | . | . |
| Vv10g0408 | . | . | . | . | . | . | . | . | . | . | . | . | . | . | . | . | . | . | . | . |
| Vv10g0409 | . | . | . | . | . | . | . | . | . | . | . | . | . | . | . | . | . | . | . | . |
| Vv10g0410 | . | . | . | . | . | . | . | . | . | . | . | . | . | . | . | . | . | . | . | . |
| Vv10g0411 | . | . | . | . | . | . | . | . | . | . | . | . | . | . | . | . | . | . | . | . |
| Vv10g0412 | Tc06_g000450 | . | . | . | . | . | . | . | . | . | . | . | . | . | . | . | . | . | . | . |
| Vv10g0413 | Tc06_g018270 | . | . | . | . | . | . | . | . | . | . | . | . | . | . | . | . | . | . | . |
| Vv10g0414 | . | . | . | . | . | . | . | . | . | . | . | . | . | . | . | . | . | . | . | . |
| Vv10g0415 | . | . | . | . | . | . | . | . | . | . | . | . | . | . | . | . | . | . | . | . |
| Vv10g0416 | . | . | . | . | . | . | . | . | . | . | . | . | . | . | . | . | . | . | . | . |
| Vv10g0417 | . | . | . | . | . | . | . | . | . | . | . | . | . | . | . | . | . | . | . | . |
| Vv10g0418 | . | . | . | . | . | . | . | . | . | . | . | . | . | . | . | . | . | . | . | . |
| Vv10g0419 | . | . | . | . | . | . | . | . | . | . | . | . | . | . | . | . | . | . | . | . |
| Vv10g0420 | . | . | . | . | . | . | . | . | . | . | . | . | . | . | . | | | | | |

. . . . .

Vv10g0421 . . . . . . .

. . . . . . . .

. . . . .

Vv10g0422 . . . . . . .

. . . . . . . .

. . . . .

Vv10g0423 . . . . . . .

. . . . . . . .

. . . . .

Vv10g0424 . . . . . . .

. . . . . . . .

. . . . .

Vv10g0425 . . . . . . .

. . . . . . . .

. . . . .

Vv10g0426 . . . . . . .

. . . . . . . .

. . . . .

Vv10g0427 Tc06_g018250 . . . . .

. . . . . . . .

. . . . . .

Vv10g0428 . . . . . . .

. . . . . . . .

. . . . .

Vv10g0429 . . . . . . .

. . . . . . . .

. . . . .

Vv10g0430 . . . . . . .

. . . . . . . .

. . . . .

Vv10g0431 . . . . . . .

. . . . . . . .

. . . . .

Vv10g0432 . . . . . . .

. . . . . . . .

. . . . .

Vv10g0433 . . . . . . .

. . . . . . . .

. . . . .

Vv10g0434 . . . . . . .

. . . . . . . .

. . . . .

Vv10g0435 . . . . . . .

. . . . . . . .

. . . . .

Vv10g0436 . . . . . . .

. . . . . . . .

. . . . .

Vv10g0437 . . . . . . .

| | | | | | | | |
|---|---|---|---|---|---|---|---|
| . | . | . | . | . | . | . | . |
| . | . | . | . | . | | | |
| Vv10g0438 | . | . | . | . | . | . | . |
| . | . | . | . | . | . | . | . |
| . | . | . | . | . | | | |
| Vv10g0439 | Tc06_g018160 | | . | . | . | . | . |
| . | . | . | . | . | . | . | . |
| . | . | . | . | . | . | | |
| Vv10g0440 | . | . | . | . | . | . | . |
| . | . | . | . | . | . | . | . |
| . | . | . | . | . | | | |
| Vv10g0441 | Tc06_g000440 | | . | . | Gorai.009G115700.1 | | |
| . | . | . | . | . | . | . | . |
| . | . | . | . | . | . | . | . |
| Vv10g0442 | Tc06_g000430 | | . | . | . | . | . |
| . | . | . | . | . | . | . | . |
| . | . | . | . | . | . | | |
| Vv10g0443 | Tc06_g000400 | | . | . | . | . | . |
| . | . | . | . | . | . | . | . |
| . | . | . | . | . | . | | |
| Vv10g0444 | . | . | . | . | . | . | . |
| . | . | . | . | . | . | . | . |
| . | . | . | . | . | | | |
| Vv10g0445 | . | . | . | . | . | . | . |
| . | . | . | . | . | . | . | . |
| . | . | . | . | . | | | |
| Vv10g0446 | . | . | . | . | . | . | . |
| . | . | . | . | . | . | . | . |
| . | . | . | . | . | | | |
| Vv10g0447 | . | . | . | . | . | . | . |
| . | . | . | . | . | . | . | . |
| . | . | . | . | . | | | |
| Vv10g0448 | . | . | . | . | . | . | . |
| . | . | . | . | . | . | . | . |
| . | . | . | . | . | | | |
| Vv10g0449 | . | . | . | . | . | . | . |
| . | . | . | . | . | . | . | . |
| . | . | . | . | . | | | |
| Vv10g0450 | . | . | . | . | . | . | . |
| . | . | . | . | . | . | . | . |
| . | . | . | . | . | | | |
| Vv10g0451 | Tc06_g000370 | | . | . | . | . | . |
| . | . | . | . | . | . | . | . |
| . | . | . | . | . | . | | |
| Vv10g0452 | . | . | . | . | . | . | . |
| . | . | . | . | . | . | . | . |
| . | . | . | . | . | | | |
| Vv10g0453 | . | . | . | . | . | . | . |
| . | . | . | . | . | . | . | . |
| . | . | . | . | . | | | |

Vv10g0454 Tc06_g000360 . . Gorai.009G115600.1

. . . . . . . .

. . . . . . . .

Vv10g0455 . . . . . . .

. . . . . . . .

. . . . .

Vv10g0456 Tc06_g000340 . . . . .

. . . . . . . .

. . . . . .

Vv10g0457 . . . . . . .

. . . . . . . .

. . . . .

Vv10g0458 Tc06_g000370 . . . . .

. . . . . . . .

. . . . . .

Vv10g0459 . . . . . . .

. . . . . . . .

. . . . .

Vv10g0460 . . . . . . .

. . . . . . . .

. . . . .

Vv10g0461 Tc06_g000400 . . . . .

. . . . . . . .

. . . . . .

Vv10g0462 . . . . . . .

. . . . . . . .

. . . . .

Vv10g0463 . . . . . . .

. . . . . . . .

. . . . .

Vv10g0464 . . . . . . .

. . . . . . . .

. . . . .

Vv10g0465 . . . . . . .

. . . . . . . .

. . . . .

Vv10g0466 Tc06_g002050 . . . . .

. . . . . . . .

. . . . . .

Vv10g0467 Tc06_g002060 . . . . .

. . . . . . . .

. . . . . .

Vv10g0468 . . . . . . .

. . . . . . . .

. . . . .

Vv10g0469 . . . . . . .

. . . . . . . .

. . . . .

Vv10g0470 Tc06_g002080 . . . Gorai.

011G007800.1 . . . . . . .

. . . . . . . .
Vv10g0471 . . . . . . .
. . . . . . . .
. . . . .
Vv10g0472 Tc06_g002090 . . . Gorai.
011G007700.1 . . . . . . .
. . . . . . . .
Vv10g0473 . . . . . . .
. . . . . . . .
. . . . .
Vv10g0474 . . . . . . .
. . . . . . . .
. . . . .
Vv10g0475 . . . . . . .
. . . . . . . .
. . . . .
Vv10g0476 . . . . . . .
. . . . . . . .
. . . . .
Vv10g0477 Tc06_g018910 . . . .
Gorai.010G179300.1 . . . . . .
. . . . . . . .
Vv10g0478 . . . . . . .
. . . . . . . .
. . . . .
Vv10g0479 . . . . . . .
. . . . . . . .
. . . . .
Vv10g0480 Tc06_g002200 . . . Gorai.
011G007500.1 . . . . . . .
. . . . . . . .
Vv10g0481 . . . . . . .
. . . . . . . .
. . . . .
Vv10g0482 Tc06_g002220 . . . Gorai.
011G007400.1 . . . . . . .
. . . . . . . .
Vv10g0483 . . . . . . .
. . . . . . . .
. . . . .
Vv10g0484 . . . . . . .
. . . . . . . .
. . . . .
Vv10g0485 . . . . . . .
. . . . . . . .
. . . . .
Vv10g0486 . . . . . . .
. . . . . . . .
. . . . .
Vv10g0487 Tc06_g002250 . . . Gorai.

011G007300.1 . . . . . . .
. . . . . . . .
Vv10g0488 Tc06_g002270 . . . . .
. . . . . . . .
. . . . . .
Vv10g0489 Tc06_g002280 . . . Gorai.
011G007200.1 . . . . . . .
. . . . . . . .
Vv10g0490 . . . . . . .
. . . . . . . .
. . . . .
Vv10g0491 Tc06_g002300 . . . .
Gorai.010G221400.1 . . . . . .
. . . . . . . .
Vv10g0492 Tc06_g002320 . . . Gorai.
011G007100.1 Gorai.010G221500.1 . . . .
. . . . . . . .
. .
Vv10g0493 Tc06_g002770 . . Gorai.009G117200.1
Gorai.011G007000.1 Gorai.010G142800.1 . .
. . . . . . . .
. . . .
Vv10g0494 . . . . . . .
. . . . . . . .
. . . . .
Vv10g0495 Tc06_g000830 . . Gorai.009G116600.1
. . . . . . . .
. . . . . . . .
Vv10g0496 . . . . . . .
. . . . . . . .
. . . . .
Vv10g0497 Tc06_g000890 . . . . .
. . . . . . . .
. . . . . .
Vv10g0498 . . . . . . .
. . . . . . . .
. . . . .
Vv10g0499 . . . . . . .
. . . . . . . .
. . . . .
Vv10g0500 . . . . . . .
. . . . . . . .
. . . . .
Vv10g0501 . . . . . . .
. . . . . . . .
. . . . .
Vv10g0502 Tc06_g000910 . . . . .
. . . . . . . .
. . . . . .
Vv10g0503 Tc06_g000920 . . Gorai.009G116700.1

. . . . . . . .
. . . . . . . .
Vv10g0504 . . . . . . .
. . . . . . . .
. . . . .
Vv10g0505 . . . . . . .
. . . . . . . .
. . . . .
Vv10g0506 Tc06_g000940 . . . . .
. . . . . . . .
. . . . . .
Vv10g0507 . . . . . . .
. . . . . . . .
. . . . .
Vv10g0508 Tc06_g000960 . . . . .
. . . . . . . .
. . . . . .
Vv10g0509 . . . . . . .
. . . . . . . .
. . . . .
Vv10g0510 . . . . . . .
. . . . . . . .
. . . . .
Vv10g0511 . . . . . . .
. . . . . . . .
. . . . .
Vv10g0512 . . . . . . .
. . . . . . . .
. . . . .
Vv10g0513 . . . . . . .
. . . . . . . .
. . . . .
Vv10g0514 Tc06_g000970 . . Gorai.009G116800.1
. . . . . . . .
. . . . . . . .
Vv10g0515 Tc06_g018870 . . . . .
. . . . . . . .
. . . . . .
Vv10g0516 . . . . . . .
. . . . . . . .
. . . . .
Vv10g0517 . . . . . . .
. . . . . . . .
. . . . .
Vv10g0518 Tc06_g000980 . . . . .
. . . . . . . .
. . . . . .
Vv10g0519 Tc06_g003150 . . . .
Gorai.010G224300.1 . . . . . .
. . . . . . . .

Vv10g0520 . . . . Gorai.011G006900.1
. . . . . . . .
. . . . . . .
Vv10g0521 Tc06_g001040 . . . Gorai.
011G006700.1 . . . . . . .
. . . . . . . .
Vv10g0522 . . . . . . .
. . . . . . . .
. . . . .
Vv10g0523 . . . . . . .
. . . . . . . .
. . . . .
Vv10g0524 . . . . . . .
. . . . . . . .
. . . . .
Vv10g0525 . . . . . . .
. . . . . . . .
. . . . .
Vv10g0526 . . . . . . .
. . . . . . . .
. . . . .
Vv10g0527 . . . . . . .
. . . . . . . .
. . . . .
Vv10g0528 . . . . . . .
. . . . . . . .
. . . . .
Vv10g0529 . . . . . . .
. . . . . . . .
. . . . .
Vv10g0530 . . . . . . .
. . . . . . . .
. . . . .
Vv10g0531 . . . . . . .
. . . . . . . .
. . . . .
Vv10g0532 . . . . . . .
. . . . . . . .
. . . . .
Vv10g0533 . . . . . . .
. . . . . . . .
. . . . .
Vv10g0534 . . . . . . .
. . . . . . . .
. . . . .
Vv10g0535 . . . . . . .
. . . . . . . .
. . . . .
Vv10g0536 . . . . . . .
. . . . . . . .

| | | | | | | | |
|---|---|---|---|---|---|---|---|
| . | . | . | . | . | | | |
| Vv10g0537 | . | . | . | . | Gorai.011G004000.1 | | |
| . | . | . | . | . | . | . | . |
| . | . | . | . | . | . | . | |
| Vv10g0538 | . | . | . | . | . | . | . |
| . | . | . | . | . | . | . | . |
| . | . | . | . | . | | | |
| Vv10g0539 | . | . | . | . | . | . | . |
| . | . | . | . | . | . | . | . |
| . | . | . | . | . | | | |
| Vv10g0540 | . | . | . | . | . | . | . |
| . | . | . | . | . | . | . | . |
| . | . | . | . | . | | | |
| Vv10g0541 | . | . | . | . | . | . | . |
| . | . | . | . | . | . | . | . |
| . | . | . | . | . | | | |
| Vv10g0542 | . | . | . | . | . | . | . |
| . | . | . | . | . | . | . | . |
| . | . | . | . | . | | | |
| Vv10g0543 | . | . | . | . | . | . | . |
| . | . | . | . | . | . | . | . |
| . | . | . | . | . | | | |
| Vv10g0544 | . | . | . | . | . | . | . |
| . | . | . | . | . | . | . | . |
| . | . | . | . | . | | | |
| Vv10g0545 | . | . | . | . | . | . | . |
| . | . | . | . | . | . | . | . |
| . | . | . | . | . | | | |
| Vv10g0546 | . | . | . | . | . | . | . |
| . | . | . | . | . | . | . | . |
| . | . | . | . | . | | | |
| Vv10g0547 | . | . | . | . | . | . | . |
| . | . | . | . | . | . | . | . |
| . | . | . | . | . | | | |
| Vv10g0548 | . | . | . | . | . | . | . |
| . | . | . | . | . | . | . | . |
| . | . | . | . | . | | | |
| Vv10g0549 | Tc06_g018470 | | . | . | . | . | . |
| . | . | . | . | . | . | . | . |
| . | . | . | . | . | . | | |
| Vv10g0550 | . | . | . | . | . | . | . |
| . | . | . | . | . | . | . | . |
| . | . | . | . | . | | | |
| Vv10g0551 | . | . | . | . | . | . | . |
| . | . | . | . | . | . | . | . |
| . | . | . | . | . | | | |
| Vv10g0552 | . | . | . | . | . | . | . |
| . | . | . | . | . | . | . | . |
| . | . | . | . | . | | | |
| Vv10g0553 | . | . | . | . | . | . | . |

| | | | | | | | |
|---|---|---|---|---|---|---|---|
| . | . | . | . | . | . | . | . |
| . | . | . | . | . | | | |
| Vv10g0554 | Tc06_g018460 | | . | . | . | . | . |
| . | . | . | . | . | . | . | . |
| . | . | . | . | . | . | | |
| Vv10g0555 | Tc06_g018450 | | . | . | . | . | . |
| . | . | . | . | . | . | . | . |
| . | . | . | . | . | . | | |
| Vv10g0556 | . | . | . | . | . | . | . |
| . | . | . | . | . | . | . | . |
| . | . | . | . | . | | | |
| Vv10g0557 | Tc06_g018370 | | . | . | . | . | . |
| . | . | . | . | . | . | . | . |
| . | . | . | . | . | . | | |
| Vv10g0558 | Tc06_g018360 | | . | . | . | . | . |
| . | . | . | . | . | . | . | . |
| . | . | . | . | . | . | | |
| Vv10g0559 | . | . | . | . | . | . | . |
| . | . | . | . | . | . | . | . |
| . | . | . | . | . | | | |
| Vv10g0560 | . | . | . | . | . | . | . |
| . | . | . | . | . | . | . | . |
| . | . | . | . | . | | | |
| Vv10g0561 | . | . | . | . | . | . | . |
| . | . | . | . | . | . | . | . |
| . | . | . | . | . | | | |
| Vv10g0562 | . | . | . | . | . | . | . |
| . | . | . | . | . | . | . | . |
| . | . | . | . | . | | | |
| Vv10g0563 | . | . | . | . | . | . | . |
| . | . | . | . | . | . | . | . |
| . | . | . | . | . | | | |
| Vv10g0564 | . | . | . | . | . | . | . |
| . | . | . | . | . | . | . | . |
| . | . | . | . | . | | | |
| Vv10g0565 | . | . | . | . | . | . | . |
| . | . | . | . | . | . | . | . |
| . | . | . | . | . | | | |
| Vv10g0566 | . | . | . | . | . | . | . |
| . | . | . | . | . | . | . | . |
| . | . | . | . | . | | | |
| Vv10g0567 | Tc06_g018310 | | . | . | . | . | |
| Gorai.010G220800.1 | | . | . | . | . | . | . |
| . | . | . | . | . | . | . | . |
| Vv10g0568 | . | . | . | . | . | . | . |
| . | . | . | . | . | . | . | . |
| . | . | . | . | . | | | |
| Vv10g0569 | . | . | . | . | . | . | . |
| . | . | . | . | . | . | . | . |
| . | . | . | . | . | | | |

Vv10g0570 . . . . . . .
. . . . . . . .
. . . . .
Vv10g0571 . . . . . . .
. . . . . . . .
. . . . .
Vv10g0572 . . . . . . .
. . . . . . . .
. . . . .
Vv10g0573 . . . . . . .
. . . . . . . .
. . . . .
Vv10g0574 . . . . . . .
. . . . . . . .
. . . . .
Vv10g0575 . . . . . . .
. . . . . . . .
. . . . .
Vv10g0576 . . . . . . .
. . . . . . . .
. . . . .
Vv10g0577 . . . . . . .
. . . . . . . .
. . . . .
Vv10g0578 . . . . . . .
. . . . . . . .
. . . . .
Vv10g0579 . . . . . . .
. . . . . . . .
. . . . .
Vv10g0580 . . . . . . .
. . . . . . . .
. . . . .
Vv10g0581 . . . . . . .
. . . . . . . .
. . . . .
Vv10g0582 . . . . . . .
. . . . . . . .
. . . . .
Vv10g0583 . . . . . . .
. . . . . . . .
. . . . .
Vv10g0584 . . . . . . .
. . . . . . . .
. . . . .
Vv10g0585 . . . . . . .
. . . . . . . .
. . . . .
Vv10g0586 . . . . . . .
. . . . . . . .

. . . . .
Vv10g0587 . . . . Gorai.011G005200.1
. . . . . . . .
. . . . . . .
Vv10g0588 . . . . . . .
. . . . . . . .
. . . . .
Vv10g0589 . . . . . . .
. . . . . . . .
. . . . .
Vv10g0590 Tc06_g002720 . . Gorai.009G117500.1
. . . . . . . .
. . . . . . . .
Vv10g0591 . . . . . . .
. . . . . . . .
. . . . .
Vv10g0592 . . . . . . .
. . . . . . . .
. . . . .
Vv10g0593 . . . . . . .
. . . . . . . .
. . . . .
Vv10g0594 . . . . . . .
. . . . . . . .
. . . . .
Vv10g0595 . . . . . . .
. . . . . . . .
. . . . .
Vv10g0596 . . . . . . .
. . . . . . . .
. . . . .
Vv10g0597 . . . . . . .
. . . . . . . .
. . . . .
Vv10g0598 Tc06_g000320 . . Gorai.009G115500.1
. . . . . . . .
. . . . . . . .
Vv10g0599 . . . . . . .
. . . . . . . .
. . . . .
Vv10g0600 . . . . . . .
. . . . . . . .
. . . . .
Vv10g0601 . . . . . . .
. . . . . . . .
. . . . .
Vv10g0602 Tc06_g000530 . . Gorai.009G116000.1
Gorai.011G005300.1 . . . . .
. . . . . . . .
. .

Vv10g0603 Tc06_g000540 . . . . .
. . . . . . . .
. . . . . .
Vv10g0604 . . . . . . .
. . . . . . . .
. . . . .
Vv10g0605 Tc06_g000550 . . Gorai.009G116100.1
Gorai.011G005400.1 . . . . .
. . . . . . . .
. .
Vv10g0606 . . . . . . .
. . . . . . . .
. . . . .
Vv10g0607 . . . . . . .
. . . . . . . .
. . . . .
Vv10g0608 . . . . . . .
. . . . . . . .
. . . . .
Vv10g0609 . . . . . . .
. . . . . . . .
. . . . .
Vv10g0610 Tc06_g000560 . . . . .
. . . . . . . .
. . . . . .
Vv10g0611 . . . . . . .
. . . . . . . .
. . . . .
Vv10g0612 Tc06_g000810 . . Gorai.009G116500.1
Gorai.011G006000.1 . . . . .
. . . . . . . .
. .
Vv10g0613 . . . . . . .
. . . . . . . .
. . . . .
Vv10g0614 Tc06_g002710 . . Gorai.009G117600.1
. . . . . . . .
. . . . . . . .
Vv10g0615 . . . . . . .
. . . . . . . .
. . . . .
Vv10g0616 . . . . . . .
. . . . . . . .
. . . . .
Vv10g0617 . . . . . . .
. . . . . . . .
. . . . .
Vv10g0618 . . . . . . .
. . . . . . . .
. . . . .

Vv10g0619 Tc06_g000850 . . . . .
. . . . . . . .
. . . . . .
Vv10g0620 . . . . . . .
. . . . . . . .
. . . . .
Vv10g0621 . . . . . . .
. . . . . . . .
. . . . .
Vv10g0622 Tc06_g000860 . . . Gorai.
011G006100.1 . . . . . . .
. . . . . . . .
Vv10g0623 . . . . . . .
. . . . . . . .
. . . . .
Vv10g0624 . . . . . . .
. . . . . . . .
. . . . .
Vv10g0625 . . . . . . .
. . . . . . . .
. . . . .
Vv10g0626 . . . . . . .
. . . . . . . .
. . . . .
Vv10g0627 . . . . . . .
. . . . . . . .
. . . . .
Vv10g0628 . . . . . . .
. . . . . . . .
. . . . .
Vv10g0629 . . . . . . .
. . . . . . . .
. . . . .
Vv10g0630 . . . . . . .
. . . . . . . .
. . . . .
Vv10g0631 . . . . . . .
. . . . . . . .
. . . . .
Vv10g0632 Tc06_g001020 . . . Gorai.
011G006700.1 . . . . . . .
. . . . . . . .
Vv11g0001 . . . . . . .
. . . . . . . .
. . . . .
Vv11g0002 . . . . . . .
. . . . . . . .
. . . . .
Vv11g0003 . . . Gorai.004G285700.1
Gorai.009G039400.1 . . . . . .

. . . . . . . .
.
Vv11g0004 . . . . . . .
. . . . . . . .
. . . . .
Vv11g0005 . . . . . . .
. . . . . . . .
. . . . .
Vv11g0006 Tc09_g006050 . Gorai.001G018700.1 .
Gorai.009G039300.1 . . . . .
. . . . . . . .
. .
Vv11g0007 . . . . . . .
. . . . . . . .
. . . . .
Vv11g0008 Tc09_g006070 . Gorai.001G018600.1 .
Gorai.009G039200.1 . . . . .
. . . . . . . .
. .
Vv11g0009 Tc09_g006080 Gorai.013G221700.1 . .
. . . . . . . .
. . . . . . . .
Vv11g0010 . . . . . . .
. . . . . . . .
. . . . .
Vv11g0011 Tc09_g006090 . Gorai.001G018400.1 .
. . . . . . . .
. . . . . . . .
Vv11g0012 Tc09_g006100 . . . . .
. . . . . . . .
. . . . . .
Vv11g0013 . . . . . . .
. . . . . . . .
. . . . .
Vv11g0014 Tc09_g006130 . . . .
Gorai.010G094700.1 . . . . . .
. . . . . . . .
Vv11g0015 Tc09_g006140 Gorai.013G221800.1 . .
. . . . . . . .
. . . . . . . .
Vv11g0016 Tc09_g006150 . . . Gorai.
009G039100.1 . Vv09g0019 . . . .
. . . . . . . .
.
Vv11g0017 Tc09_g006160 . . . . .
Vv09g0020 Tc06_g010770 . . .
Gorai.011G055100.1 . . . . . .
. .
Vv11g0018 . . . . . . .
. . . . . . . .

. . . . .
Vv11g0019 Tc09_g006170 . Gorai.001G018300.1 .
Gorai.009G039000.1 . . . . .
. . . . . . . .
. .
Vv11g0020 Tc09_g006190 . Gorai.001G018200.1 .
Gorai.009G038900.1 . . . . .
. . . . . . . .
. .
Vv11g0021 Tc09_g006200 . . . Gorai.
009G038800.1 Gorai.010G095300.1 . . . .
. . . . . . . .
. .
Vv11g0022 Tc09_g006210 Gorai.013G221900.1 . .
. Gorai.010G095200.1 . . . .
. . . . . . . .
. .
Vv11g0023 Tc09_g006220 . Gorai.001G018100.1 .
Gorai.009G038700.1 . Vv09g0026
Tc06_g010720 Gorai.006G090300.1 . . . .
. . . . . . .
Vv11g0024 Tc09_g006230 . Gorai.001G018000.1 .
Gorai.009G038600.1 . . . . .
. . . . . . . .
. .
Vv11g0025 Tc09_g006240 . Gorai.001G017900.1 .
Gorai.009G038500.1 . . . . .
. . . . . . . .
. .
Vv11g0026 Tc09_g006250 . . . Gorai.
009G038400.1 . . . . . . .
. . . . . . . .
Vv11g0027 Tc09_g006260 Gorai.013G222000.1 . .
Gorai.009G038300.1 . Vv09g0027 . .
. . Gorai.011G055600.1 . . .
. . . . .
Vv11g0028 Tc09_g006280 . . . Gorai.
009G038200.1 . . . . . . .
. . . . . . . .
Vv11g0029 Tc09_g006290 . . . . .
. . . . . . . .
. . . . . .
Vv11g0030 Tc09_g006300 Gorai.013G222100.1 Gorai.
001G017800.1 . . Gorai.010G092400.1 Vv09g0043
Tc06_g010520 . Gorai.010G235900.1 .
Gorai.011G056600.1 . . . . . .
. .
Vv11g0031 Tc09_g006310 . . . Gorai.
009G038100.1 . . . . . . .
. . . . . . . .

Vv11g0032 Tc09_g006320 Gorai.013G222300.1 . .
. . Vv17g0386 . . . .
. . . . . . . .
.
Vv11g0033 . . . . . . .
. . . . . . . .
. . . . .
Vv11g0034 . . . . . . .
. . . . . . . .
. . . . .
Vv11g0035 . . . . . . .
. . . . . . . .
. . . . .
Vv11g0036 Tc09_g006330 Gorai.013G222400.1 Gorai.
001G017700.1 . Gorai.009G038000.1 Gorai.010G092300.1
. . . . . . . .
. . . . . .
Vv11g0037 . . . . . . .
. . . . . . . .
. . . . .
Vv11g0038 Tc09_g006340 . . . . .
. . . . . . . .
. . . . . .
Vv11g0039 . . . . Gorai.009G037900.1
. . . . . . . .
. . . . . . .
Vv11g0040 Tc09_g006350 Gorai.013G222500.1 Gorai.
001G017600.1 . Gorai.009G037800.1 Gorai.010G092200.1
. . . . . . .
Vv09g0049 Tc06_g010480 . . . Gorai.
011G056900.1 .
Vv11g0041 Tc09_g006360 . . . . .
. . . . . . . .
. . . . . .
Vv11g0042 Tc09_g006380 . Gorai.001G017500.1
Gorai.004G285600.1 Gorai.009G037700.1 Gorai.010G091700.1
. . . . . . . .
. . . . . .
Vv11g0043 Tc09_g006390 . . . Gorai.
009G037600.1 Gorai.010G091600.1 . . . .
. . . . . . . .
. .
Vv11g0044 Tc09_g006400 . Gorai.001G017400.1 .
. . . . . . . .
. . . . . . . .
Vv11g0045 Tc09_g006410 . . Gorai.004G285500.1
. . . . . . . .
. . . . . . . .
Vv11g0046 Tc09_g006420 . . Gorai.004G285400.1
. . . . . . . .

. . . . . . . .
Vv11g0047 Tc09_g006430 . . . Gorai.
009G037500.1 . Vv04g0226 . . . .
. . . . . . . .
.
Vv11g0048 Tc09_g006460 . Gorai.001G017200.1 .
Gorai.009G037300.1 . . . . .
. . . . . . . .
. .
Vv11g0049 . Gorai.013G222600.1 . Gorai.
004G285300.1 . Gorai.010G091400.1 . . .
. . . . . . . .
. . .
Vv11g0050 Tc09_g006480 . . . . .
. . . . . . .
Vv09g0061 Tc06_g010360 Gorai.006G090100.1 . .
. .
Vv11g0051 Tc09_g006490 . . . Gorai.
009G037200.1 . . . . . . .
. . . . . . . .
Vv11g0052 . . . . . . .
. . . . . . . .
. . . . .
Vv11g0053 . . . . . .
Vv04g0218 Tc01_g016100 Gorai.013G221400.1 . .
. . . . . . . .
.
Vv11g0054 Tc09_g006500 . Gorai.001G017000.1 .
Gorai.009G037100.1 Gorai.010G091300.1 . .
. . . . . . . .
. . . .
Vv11g0055 Tc09_g006510 . Gorai.001G016900.1 .
. . . . . . . .
. . . . . . . .
Vv11g0056 Tc09_g006520 . . Gorai.004G285200.1
. Gorai.010G091100.1 . . . .
. . . . . . . .
. .
Vv11g0057 Tc09_g006530 Gorai.013G222700.1 Gorai.
001G016800.1 Gorai.004G282200.1 Gorai.009G037000.1 .
. . . . . . . .
. . . . . .
Vv11g0058 Tc09_g006550 Gorai.013G222800.1 . .
. . . . . . . .
. . . . . . . .
Vv11g0059 Tc09_g006580 . Gorai.001G016700.1 .
. Gorai.010G090800.1 . . . .
. . . . . . . .
. .
Vv11g0060 Tc09_g006590 . Gorai.001G016600.1 .

```
        .       Gorai.010G090700.1      .       .       .       .
        .       .       .       .       .       .       .       .
        .       .
Vv11g0061       Tc09_g006620    .       .       .       Gorai.
009G036800.1    Gorai.010G090600.1      .       .       .       .
        .       .       .       Vv09g0073       Tc06_g010210    .
        .       .       .       .
Vv11g0062       Tc09_g006630    .       Gorai.001G016500.1      .
        Gorai.009G036700.1      Gorai.010G090400.1      .       .
        .       .       .       .       .       .       .       .
        .       .       .       .
Vv11g0063       Tc09_g006640    .       Gorai.001G016400.1      .
        .       .       .       .       .       .       .       .
        .       .       .       .       .       .       .       .
Vv11g0064       Tc09_g006650    .       Gorai.001G016300.1      .
        Gorai.009G036600.1      .       .       .       .       .
        .       .       .       .       .       .       .       .
        .       .
Vv11g0065       .       .       .       .       .       .       .
        .       .       .       .       .       .       .       .
        .       .       .       .       .
Vv11g0066       Tc09_g006660    .       .       .       .
Gorai.010G090300.1      .       .       .       .       .       .
        .       .       .       .       .       .       .       .
Vv11g0067       Tc09_g006670    .       Gorai.001G016200.1      .
        .       .       Vv17g0422       .       .       .       .
        .       .       .       .       .       .       .       .
        .
Vv11g0068       Tc09_g006680    .       .       .       Gorai.
009G036500.1    Gorai.010G090200.1      .       .       .       .
        .       .       .       .       .       .       .       .
        .       .
Vv11g0069       Tc09_g006690    .       Gorai.001G016100.1      .
        Gorai.009G036400.1      Gorai.010G090100.1      .       .
        .       .       .       .       .       Vv09g0077
Tc06_g010150    .       .       .       .       .
Vv11g0070       Tc09_g006700    .       .       .       .
Gorai.010G090000.1      .       .       .       .       .       .
        .       Vv09g0078       Tc06_g010140    .       .       .
        .       .
Vv11g0071       Tc09_g006710    .       .       .       .
Gorai.010G089900.1      .       .       .       .       .       .
        .       .       .       .       .       .       .       .
Vv11g0072       Tc09_g006720    Gorai.013G222900.1      Gorai.
001G016000.1    .       .       Gorai.010G089800.1      .       .
        .       .       .       .       .       Vv09g0080       .
        .       .       .       .       .
Vv11g0073       .       .       .       .       .       .       .
        .       .       .       .       .       .       .       .
        .       .       .       .       .
```

Vv11g0074 Tc09_g006740 Gorai.013G223100.1 Gorai.
001G015800.1 . . . Vv17g0446 . .
. . . . . . . .
. . .
Vv11g0075 . . . . . . .
. . . . . . . .
. . . . .
Vv11g0076 . . . . . .
Vv17g0454 Tc03_g013510 . . . . .
. . . . . . .
Vv11g0077 . . . . . . .
. . . . . . . .
. . . . .
Vv11g0078 . Gorai.013G223300.1 . . .
. Vv17g0483 . . . .
Gorai.008G201500.1 . . . . . .
. .
Vv11g0079 Tc09_g006760 . . . . .
Vv17g0485 . . . . . .
. . . . . . .
Vv11g0080 . . . . . .
Vv17g0487 Tc03_g013310 . . . . .
. . . . . . .
Vv11g0081 Tc09_g006770 . . . Gorai.
009G036300.1 Gorai.010G089300.1 Vv04g0212 .
Gorai.013G221200.1 Gorai.001G019300.1 . . .
. . . . . . .
Vv11g0082 . . . . . . .
. . . . . . Vv09g0085
. . . . . .
Vv11g0083 Tc09_g006780 . . . . .
. . . . . . . .
. . . . . .
Vv11g0084 Tc09_g006790 Gorai.013G223600.1 . .
. . . . . . . .
. Vv09g0086 . . . . .
.
Vv11g0085 . . . . . . .
. . . . . . . .
. . . . .
Vv11g0086 Tc09_g006810 . . . .
Gorai.010G089100.1 . . . . . .
. . . . . . . .
Vv11g0087 Tc09_g006820 Gorai.013G223800.1 Gorai.
001G015700.1 . . Gorai.010G088900.1 . .
. . . . . . . .
. . . .
Vv11g0088 Tc09_g006830 Gorai.013G223900.1 . .
. . . . . . . .
. . . . . . . .

Vv11g0089 Tc09_g006840 . Gorai.001G015600.1 .
Gorai.009G036200.1 . . . . .
. . . . . . . .
. .
Vv11g0090 . . . . . . .
. . . . . . . .
. . . . .
Vv11g0091 . . . . . . .
. . . . . . . .
. . . . .
Vv11g0092 . . . . . . .
. . . . . . . .
. . . . .
Vv11g0093 . . . . . . .
. . . . . . . .
. . . . .
Vv11g0094 . . . . . . .
. . . . . . . .
. . . . .
Vv11g0095 Tc09_g006880 . . . .
Gorai.010G088700.1 . . . . . .
. . . . . . . .
Vv11g0096 . . . . . . .
. . . . . . . .
. . . . .
Vv11g0097 . . . . . . .
. . . . . . . .
. . . . .
Vv11g0098 Tc09_g006890 Gorai.013G224000.1 . .
. Gorai.010G088600.1 . . . .
. . . . . . . .
. .
Vv11g0099 . . . . . . .
. . . . . . . .
. . . . .
Vv11g0100 Tc09_g006900 Gorai.013G224100.1 . .
. Gorai.010G088500.1 . . . .
. . . . . . . .
. .
Vv11g0101 Tc09_g006910 Gorai.013G224200.1 . .
. . . . . . . .
. . . . . . . .
Vv11g0102 . . . . . . .
. . . . . . . .
. . . . .
Vv11g0103 Tc09_g006920 Gorai.013G224300.1 . .
. . . . . . . .
. . . . . . . .
Vv11g0104 Tc09_g006930 . . . .
Gorai.010G088400.1 . . . . . .

. . . . . . . .
Vv11g0105 Tc09_g006940 . Gorai.001G015500.1 .
Gorai.009G040300.1 . Vv04g0202 . .
Gorai.001G019700.1 . . . . .
. . . . .
Vv11g0106 Tc09_g006980 Gorai.013G224400.1 . .
. Gorai.010G088300.1 . . . .
. . . . . . . .
. .
Vv11g0107 Tc09_g007010 . . . .
Gorai.010G088200.1 . . . . . .
. . . . . . . .
Vv11g0108 Tc09_g007020 Gorai.013G224500.1 . .
. Gorai.010G088100.1 . . . .
. . . . . . . .
. .
Vv11g0109 . . . . . . .
. . . . . . . .
. . . . .
Vv11g0110 Tc09_g007050 . . . .
Gorai.010G087700.1 . . . . . .
. . . . . . . .
Vv11g0111 . . . . . . .
. . . . . . . .
. . . . .
Vv11g0112 Tc09_g007060 Gorai.013G225200.1 . .
. Gorai.010G087600.1 . . . .
. . . . . . . .
. .
Vv11g0113 Tc09_g007070 . . . .
Gorai.010G087500.1 . . . . . .
. . . . . . . .
Vv11g0114 . . . . . . .
. . . . . . . .
. . . . .
Vv11g0115 Tc09_g007080 . Gorai.001G015400.1 .
Gorai.009G035500.1 . . . . .
. . . Vv09g0114 Tc06_g000370 .
. . . .
Vv11g0116 Tc09_g007090 . . . Gorai.
009G035400.1 Gorai.010G087400.1 . . . .
. . . . . . . .
. .
Vv11g0117 Tc09_g007100 . . . Gorai.
009G035300.1 . . . . . . .
. . . . . . . .
Vv11g0118 Tc09_g007110 Gorai.013G225300.1 . .
Gorai.009G035200.1 . . . . .
. . . Vv09g0116 . . .
. . .

Vv11g0119 . . Gorai.001G015300.1 . .
. . . . . . . .
. . . . . . .
Vv11g0120 Tc09_g007120 . . . . .
. . . . . . . .
. . . . . .
Vv11g0121 Tc09_g007130 Gorai.013G225400.1 . .
. . . . . . . .
. . . . . . . .
Vv11g0122 Tc09_g007140 Gorai.013G225500.1 . .
Gorai.009G035100.1 . . . . .
. . . . . . . .
. .
Vv11g0123 Tc09_g007150 . . . Gorai.
009G035000.1 . . . . . . .
. . . . . . . .
Vv11g0124 . . . . . . .
. . . . . . . .
. . . . .
Vv11g0125 Tc09_g007160 . Gorai.001G015200.1 .
. Gorai.010G087200.1 . . . .
. . . . . . . .
. .
Vv11g0126 . . . . . . .
. . . . . . . .
. . . . .
Vv11g0127 . . . . . . .
. . . . . . . .
. . . . .
Vv11g0128 . . . . . . .
. . . . . . . .
. . . . .
Vv11g0129 . . . . . . .
. . . . . . . .
. . . . .
Vv11g0130 . . Gorai.001G015100.1 .
Gorai.009G034900.1 . . . . . .
. . . . . . . .
.
Vv11g0131 Tc09_g007180 . Gorai.001G014900.1 .
. . . . . . . .
. . . . . . . .
Vv11g0132 Tc09_g007190 Gorai.013G225600.1 Gorai.
001G014800.1 . Gorai.009G034800.1 Gorai.010G087000.1
. . . . . . . .
. . . . . .
Vv11g0133 . . . . . . .
. . . . . . . .
. . . . .
Vv11g0134 Tc09_g007200 . Gorai.001G014700.1 .

Gorai.009G041200.1 . Vv04g0174 . .
Gorai.001G020800.1 . . . Vv09g0128
. . . . . .
Vv11g0135 Tc09_g007210 Gorai.013G225700.1 . .
. . . . . . . .
. . . . . . . .
Vv11g0136 Tc09_g007220 Gorai.013G225800.1 Gorai.
001G014500.1 . . . Vv04g0172 . .
. . . . . . . .
. . .
Vv11g0137 . . . . . . .
. . . . . . . .
. . . . .
Vv11g0138 Tc09_g007230 . . . Gorai.
009G041300.1 Gorai.010G086800.1 Vv04g0171 . .
Gorai.001G020900.1 . . . . .
. . . . .
Vv11g0139 Tc09_g007250 . Gorai.001G014400.1 .
. . . . . . . .
. . . . . . . .
Vv11g0140 Tc09_g007260 . Gorai.001G014300.1 .
. Gorai.010G086700.1 . . . .
. . . . . . . .
. .
Vv11g0141 Tc09_g007270 . . . Gorai.
009G041500.1 Gorai.010G086600.1 Vv04g0169 . .
Gorai.001G021000.1 . . . Vv09g0132
. . . . . .
Vv11g0142 Tc09_g007280 Gorai.013G226100.1 . .
Gorai.009G034500.1 Gorai.010G086500.1 . .
. . . . . Vv09g0134 .
. . . . .
Vv11g0143 Tc09_g007290 . . . .
Gorai.010G086400.1 . . . . . .
. Vv09g0135 Tc06_g011930 Gorai.006G095300.1
Gorai.010G234300.1 . . .
Vv11g0144 . . . . . . .
. . . . . . . .
. . . . .
Vv11g0145 Tc09_g007310 Gorai.013G226300.1 . .
. . . . . . . .
. . . . . . . .
Vv11g0146 Tc09_g007320 Gorai.013G226400.1 . .
Gorai.009G041700.1 Gorai.010G086100.1 Vv04g0166
. Gorai.013G226400.1 Gorai.001G021300.1 .
. . Vv09g0140 . . . .
. .
Vv11g0147 . . . . . . .
. . . . . . . .
. . . . .

Vv11g0148 . . . . . . .
. . . . . . . .
. . . . .
Vv11g0149 . . . . . . .
. . . . . . . .
. . . . .
Vv11g0150 . . . . . Gorai.
010G086000.1 . . . . . . .
. . . . . . .
Vv11g0151 Tc09_g007330 . . . .
Gorai.010G085900.1 . . . . . .
. . . . . . . .
Vv11g0152 . . . . . . .
. . . . . . . .
. . . . .
Vv11g0153 . . . . . . .
. . . . . . . .
. . . . .
Vv11g0154 Tc09_g007340 Gorai.013G226500.1 . .
Gorai.009G034400.1 Gorai.010G085800.1 . .
. . . . . . . .
. . . .
Vv11g0155 . . . . . . .
. . . . . . . .
. . . . .
Vv11g0156 . . . . . . .
. . . . . . . .
. . . . .
Vv11g0157 . . . . . . .
. . . . . . . .
. . . . .
Vv11g0158 . . . . . . .
. . . . . . . .
. . . . .
Vv11g0159 . . . . . .
Vv04g0145 . Gorai.013G226500.1 Gorai.001G021600.1
. . . . . . . .
. .
Vv11g0160 Tc09_g007350 . . . . .
. . . . . . . .
. . . . . .
Vv11g0161 . . . . . . .
. . . . . . . .
. . . . .
Vv11g0162 . Gorai.013G226600.1 . . .
Gorai.010G085700.1 . . . . .
. . . . . . . .
.
Vv11g0163 Tc09_g007360 Gorai.013G226700.1 Gorai.
001G014100.1 . Gorai.009G034300.1 . . .

. . . . . . . .
. . . .
Vv11g0164 . . . . . . .
. . . . . . Vv09g0171
. . . . . .
Vv11g0165 Tc09_g007370 . . . Gorai.
009G034200.1 Gorai.010G085500.1 . . . .
. . . . . . . .
. .
Vv11g0166 Tc09_g007380 . Gorai.001G014000.1 .
. Gorai.010G085400.1 . . . .
. . . . . . . .
. .
Vv11g0167 Tc09_g007390 . . . Gorai.
009G034000.1 . . . . . . .
. . . . . . . .
Vv11g0168 Tc09_g007400 . Gorai.001G013900.1 .
. . . . . . . .
. . . . . . . .
Vv11g0169 . . . . . . .
. . . . . . . .
. . . . .
Vv11g0170 . . . . . . .
. . . . . . . .
. . . . .
Vv11g0171 Tc09_g007420 . Gorai.001G013700.1 .
Gorai.009G033900.1 . . . . .
. . . . . . . .
. .
Vv11g0172 Tc09_g007440 . . . Gorai.
009G033800.1 . . . . . . .
. . . . . . . .
Vv11g0173 Tc09_g007450 . Gorai.001G013500.1 .
. . Vv04g0140 . . . .
. . . . . . . .
.
Vv11g0174 Tc09_g007460 . Gorai.001G013400.1 .
Gorai.009G033700.1 Gorai.010G084900.1 . .
. . . . . . . .
. . . .
Vv11g0175 Tc09_g007470 . . . . .
. . . . . . . .
. . . . . .
Vv11g0176 . Gorai.013G226800.1 . . .
. Vv04g0136 . Gorai.013G226800.1 .
. . . . . . . .
. .
Vv11g0177 Tc09_g007490 . . . .
Gorai.010G084800.1 . . . . . .
. . . . . . . .

Vv11g0178 . . . . . . .
. . . . . . . .
. . . . .
Vv11g0179 Tc09_g007500 . . . Gorai.
009G042800.1 Gorai.010G084700.1 . . . .
. . . . . . . .
. .
Vv11g0180 Tc09_g007510 . . . Gorai.
009G033300.1 . . . . . . .
. . . . . . . .
Vv11g0181 Tc09_g007530 . . . Gorai.
009G033100.1 . . . . . . .
. . . . . . . .
Vv11g0182 Tc09_g007540 . . . Gorai.
009G033000.1 . . . . . . .
. . . . . . . .
Vv11g0183 . . . . . . .
. . . . . . . .
. . . . .
Vv11g0184 Tc09_g007550 . . . Gorai.
009G032900.1 . . . . . . .
. . . . . . . .
Vv11g0185 Tc09_g007560 . . . . .
. . . . . . . .
. . . . . .
Vv11g0186 Tc09_g007570 Gorai.013G227000.1 . .
Gorai.009G032800.1 Gorai.010G084600.1 . .
. . . . . . . .
. . . .
Vv11g0187 Tc09_g007580 . . . .
Gorai.010G084500.1 . . . . . .
. . . . . . . .
Vv11g0188 Tc09_g007590 . . . . .
. . . . . . .
Vv09g0185 Tc06_g009090 . . . Gorai.
011G063400.1 .
Vv11g0189 Tc09_g007600 . . . . .
. . . . . . . .
. . . . . .
Vv11g0190 Tc09_g007610 . Gorai.001G013200.1 .
Gorai.009G043500.1 . . . . .
. . . . . . . .
. .
Vv11g0191 Tc09_g007620 Gorai.013G227100.1 . .
Gorai.009G032600.1 . . . . .
. . . . . . . .
. .
Vv11g0192 Tc09_g007640 Gorai.013G227200.1 . .
Gorai.009G043900.1 Gorai.010G084300.1 . .
. . . . . . . .

. . . .
Vv11g0193 Tc09_g007650 . Gorai.001G012800.1 .
. . . . . . . .
. . . . . . . .
Vv11g0194 Tc09_g007660 . . Gorai.004G284300.1
. . . . . . . .
. . . . . . . .
Vv11g0195 Tc09_g007670 . Gorai.001G012700.1
Gorai.004G284200.1 Gorai.009G032300.1 . . .
. . . . . . . .
. . . .
Vv11g0196 . . . . . . .
. . . . . . . .
. . . . .
Vv11g0197 Tc09_g007680 . . . .
Gorai.010G084200.1 . . . . . .
. . . . . . . .
Vv11g0198 Tc09_g007690 . . . Gorai.
009G032200.1 . . . . . . .
. . . . . . . .
Vv11g0199 . . . . . . .
. . . . . . . .
. . . . .
Vv11g0200 Tc09_g007700 . . . Gorai.
009G032100.1 . . . . . . .
. . . . . . . .
Vv11g0201 Tc09_g007710 . Gorai.001G012500.1 .
Gorai.009G032000.1 Gorai.010G084100.1 . .
. . . . . . . .
. . . .
Vv11g0202 Tc09_g007720 Gorai.013G227300.1 . .
. Gorai.010G084000.1 . . . .
. . . . . . . .
. .
Vv11g0203 Tc09_g007730 . Gorai.001G012400.1 .
Gorai.009G031900.1 Gorai.010G083900.1 . .
. . . . . . . .
. . . .
Vv11g0204 . . . . . . .
. . . . . . . .
. . . . .
Vv11g0205 Tc09_g007740 . . . .
Gorai.010G083800.1 . . . . . .
. . . . . . . .
Vv11g0206 Tc09_g007750 . . . Gorai.
009G044400.1 Gorai.010G083600.1 Vv04g0116 . .
. . . Gorai.011G061800.1 . .
. . . . .
Vv11g0207 Tc09_g007760 . . . .
Gorai.010G083500.1 . . . . . .

. . . . . . . .
Vv11g0208 Tc09_g007770 Gorai.013G227400.1 Gorai.
001G012300.1 . . Gorai.010G083400.1 . .
. . . . . . . .
. . . .
Vv11g0209 Tc09_g007780 . Gorai.001G012200.1 .
. . . . . . . .
. . . . . . . .
Vv11g0210 Tc09_g007790 . . . . .
. . . . . . . .
. . . . . .
Vv11g0211 . . . . . Gorai.
010G083200.1 . . . . . . .
. . . . . . .
Vv11g0212 Tc09_g007810 . . . . .
. . . . . . . .
. . . . . .
Vv11g0213 . . . . . . .
. . . . . . . .
. . . . .
Vv11g0214 Tc09_g001690 . . . . .
. . . . . . . .
. . . . . .
Vv11g0215 Tc09_g007820 . Gorai.001G012000.1 .
. . . . . . . .
. . . . . . . .
Vv11g0216 Tc09_g007850 Gorai.013G227700.1 . .
Gorai.009G031700.1 . . . . .
. . . . . . . .
. .
Vv11g0217 Tc09_g007860 . . . Gorai.
009G031600.1 Gorai.010G083100.1 . . . .
. . . Vv09g0192 . . .
. . .
Vv11g0218 . . . . . . .
. . . . . . . .
. . . . .
Vv11g0219 Tc09_g007890 . Gorai.001G011800.1 .
Gorai.009G031500.1 . . . . .
. . . . . . . .
. .
Vv11g0220 Tc09_g007910 . Gorai.001G011700.1 .
. . . . . . . .
. . . . . . . .
Vv11g0221 Tc09_g007920 . Gorai.001G011600.1 .
. Gorai.010G082900.1 . . . .
. . . Vv09g0206 Tc06_g008850 .
. . . .
Vv11g0222 Tc09_g007930 . . . Gorai.
009G045000.1 Gorai.010G082800.1 . . . .

```
        .       .       .       .       .       .       .       .
        .       .
Vv11g0223       Tc09_g001550    .       .       .       .       .
        .       .       .       .       .       .       .       .
        .       .       .       .       .       .
Vv11g0224       .       .       .       .       .       .       .
        .       .       .       .       .       .       .       .
        .       .       .       .       .
Vv11g0225       .       .       .       .       .       .       .
        .       .       .       .       .       .       .       .
        .       .       .       .       .
Vv11g0226       .       .       .       .       .       .       .
        .       .       .       .       .       .       .       .
        .       .       .       .       .
Vv11g0227       .       .       .       .       .       .       .
        .       .       .       .       .       .       .       .
        .       .       .       .       .
Vv11g0228       .       .       .       .       .       .       .
        .       .       .       .       .       .       .       .
        .       .       .       .       .
Vv11g0229       Tc09_g007960    .       Gorai.001G011300.1      .
        .       .       .       .       .       .       .       .
        .       .       .       .       .       .       .       .
Vv11g0230       .       .       .       .       Gorai.009G045100.1
        .       .       .       .       .       .       .       .
        .       .       .       .       .       .       .
Vv11g0231       Tc09_g007970    .       .       .       .
Gorai.010G082500.1      .       .       .       .       .       .
        .       .       .       .       .       .       .       .
Vv11g0232       .       .       .       .       .       .       .
        .       .       .       .       .       .       .       .
        .       .       .       .       .
Vv11g0233       Tc09_g007980    .       .       .       Gorai.
009G031300.1    Gorai.010G082300.1      .       .       .       .
        .       .       .       .       .       .       .       .
        .       .
Vv11g0234       Tc09_g007990    .       .       .       Gorai.
009G031200.1    Gorai.010G082200.1      .       .       .       .
        .       .       .       .       .       .       .       .
        .       .
Vv11g0235       Tc09_g008000    .       .       .       .       .
        .       .       .       .       .       .       .       .
        .       .       .       .       .       .
Vv11g0236       Tc09_g008010    Gorai.013G227900.1      .
Gorai.004G284100.1      .       .       .       .       .       .
        .       .       Vv09g0216       .       .       .
        .       Gorai.011G066300.1      .
Vv11g0237       Tc09_g008020    Gorai.013G228000.1      .
Gorai.004G284000.1      Gorai.009G031100.1      .       .       .
        .       .       .       .       .       .       .       .
```

. . . .
Vv11g0238 Tc09_g008030 . . . .
Gorai.010G082100.1 . . . . . . .
. . . . . . . .
Vv11g0239 Tc09_g008040 . Gorai.001G011200.1 .
Gorai.009G031000.1 Gorai.010G082000.1 . .
. . . . . . . .
. . . .
Vv11g0240 Tc09_g008050 . . . .
Gorai.010G081900.1 . . . . . . .
. . . . . . . .
Vv11g0241 Tc09_g008080 . . . . .
. . . . . . . .
. . . . . .
Vv11g0242 Tc09_g008110 Gorai.013G228100.1 .
Gorai.004G283900.1 . . . . . . .
. . . . . . . .
. .
Vv11g0243 . . . . Gorai.009G030900.1
Gorai.010G081800.1 Vv04g0090 . . .
. . . . . . . .
. .
Vv11g0244 Tc09_g008130 . . . .
Gorai.010G081700.1 . . . . . . .
. . . . . . . .
Vv11g0245 Tc09_g008140 . . . .
Gorai.010G081600.1 . . . . . . .
. . . . . . . .
Vv11g0246 Tc09_g008150 . . . . .
. . . . . . . .
. . . . . .
Vv11g0247 Tc09_g008160 Gorai.013G228200.1 . .
. . . . . . . .
. . . . . . . .
Vv11g0248 Tc09_g008170 Gorai.013G228400.1 . .
. . . . . . . .
. . . . . . . .
Vv11g0249 Tc09_g008180 Gorai.013G228500.1 . .
Gorai.009G030800.1 . . . . .
. . . . . . . .
. .
Vv11g0250 Tc09_g008190 . . . Gorai.
009G030700.1 . Vv04g0087 . . . .
. Gorai.011G067300.1 Vv09g0223 . .
. . Gorai.011G067300.1 .
Vv11g0251 Tc09_g008200 . Gorai.001G023200.1 .
Gorai.009G045800.1 Gorai.010G081500.1 Vv04g0085
. . Gorai.001G011000.1 . . .
. . . . . . .
Vv11g0252 . . . . . . .

. . . . . . . .
. . . . .
Vv11g0253 Tc09_g008210 . . . . .
. . . . . . . .
. . . . . .
Vv11g0254 Tc09_g008220 . Gorai.001G010900.1 .
Gorai.009G045900.1 . . . . .
. . . . . . . .
. .
Vv11g0255 Tc09_g008230 . Gorai.001G010800.1 .
. . . . . . . .
. . . . . . . .
Vv11g0256 Tc09_g008240 . . . Gorai.
009G030600.1 Gorai.010G081400.1 . . . .
. . . . . . . .
. .
Vv11g0257 Tc09_g008250 . . . .
Gorai.010G081300.1 . . . . . .
. . . . . . . .
Vv11g0258 . . Gorai.001G023300.1 .
Gorai.009G046000.1 . Vv04g0083 . .
Gorai.001G023300.1 . Gorai.003G060000.1 Gorai.
011G067400.1 Vv09g0225 . . . .
Gorai.011G067400.1 .
Vv11g0259 Tc09_g008260 . Gorai.001G010700.1 .
Gorai.009G030500.1 . . . . .
. . . . . . . .
. .
Vv11g0260 Tc09_g008280 Gorai.013G228600.1 . .
Gorai.009G030300.1 . . . . .
. . . . . . . .
. .
Vv11g0261 Tc09_g008290 . Gorai.001G010500.1 .
Gorai.009G030200.1 Gorai.010G081100.1 . .
. . . . . . . .
. . . .
Vv11g0262 . . . Gorai.004G283700.1 .
. . . . . . . .
. . . . . . .
Vv11g0263 Tc09_g008300 . . . . .
. . . . . . . .
. . . . . .
Vv11g0264 . . . . . Gorai.
010G081000.1 . . . . . . .
. . . . . . .
Vv11g0265 Tc09_g008310 . . . . .
. . . . . . . .
. . . . . .
Vv11g0266 Tc09_g008340 . . . .
Gorai.010G080700.1 . . . . . .

. . . . . . . .
Vv11g0267 Tc09_g008350 . . Gorai.004G283500.1
. . . . . . . .
. . . . . . . .
Vv11g0268 Tc09_g008370 . . Gorai.004G283400.1
Gorai.009G030000.1 . . . . .
. . . . . . . .
. .
Vv11g0269 Tc09_g008390 Gorai.013G228700.1 .
Gorai.004G283300.1 Gorai.009G029900.1 Gorai.010G080500.1
. . . . . . . .
. . . . . .
Vv11g0270 . . . . . . .
. . . . . . . .
. . . . .
Vv11g0271 Tc09_g008400 . . . Gorai.
009G029800.1 . . . . . . .
. . . . . . . .
Vv11g0272 . . . Gorai.004G283200.1 .
. . . . . . . .
. . . . . . .
Vv11g0273 Tc09_g008410 . . . .
Gorai.010G080400.1 . . . . . .
. . . . . . . .
Vv11g0274 Tc09_g008420 Gorai.013G228800.1 . .
Gorai.009G029700.1 . . . . .
. . . . . . . .
. .
Vv11g0275 Tc09_g008440 . . . Gorai.
009G029600.1 Gorai.010G080300.1 . . . .
. . . Vv09g0239 . . .
. Gorai.011G068400.1 .
Vv11g0276 Tc09_g008450 Gorai.013G228900.1 Gorai.
001G010300.1 Gorai.004G283100.1 Gorai.009G029400.1
Gorai.010G080200.1 Vv04g0061 . . . .
. . Vv09g0256 . . . .
. .
Vv11g0277 . . . . . . .
. . . . . . . .
. . . . .
Vv11g0278 . . . . . . .
. . . . . . . .
. . . . .
Vv11g0279 . . . . . . .
. . . . . . . .
. . . . .
Vv11g0280 Tc09_g008490 . . . .
Gorai.010G079900.1 . . . . . .
. . . . . . . .
Vv11g0281 Tc09_g008500 Gorai.013G229000.1 Gorai.

001G024400.1	Gorai.004G283000.1	Gorai.009G047600.1	.
Vv04g0057	.	Gorai.013G229000.1	Gorai.
001G009900.1	Gorai.004G283000.1	.	.	.	.
.	.	.	.	.
Vv11g0282	Tc09_g008510	.	.	.	.
Gorai.010G079700.1	.	.	.	.	.	.
.	Vv09g0278	.	.	.	.	.
.
Vv11g0283	.	.	.	.	.	.	.
.	.	.	.	.	.	.	.
.	.	.	.	.
Vv11g0284	.	.	.	.	Gorai.009G047700.1
.	.	.	.	.	.	.	.
.	.	.	.	.	.	.
Vv11g0285	.	.	.	.	.	Gorai.
010G079600.1	Vv04g0055	.	Gorai.013G219900.1	.
.	.	.	.	.	.	.	.
.	.
Vv11g0286	.	.	.	.	.	.	.
.	.	.	.	.	.	.	.
.	.	.	.	.
Vv11g0287	Tc09_g008540	Gorai.013G219900.1	.	.
.	.	.	.	.	.	.	.
.	.	.	.	.	.	.	.
Vv11g0288	Tc09_g008550	.	.	.	.
Gorai.010G079400.1	.	.	.	.	.	.
.	.	.	.	.	.	.	.
Vv11g0289	Tc09_g008560	Gorai.013G229100.1	.	.
Gorai.009G029200.1	.	.	.	.	.
.	.	.	.	.	.	.	.
.	.
Vv11g0290	.	.	.	.	.	.	.
.	.	.	.	.	.	.	.
.	.	.	.	.
Vv11g0291	Tc09_g008570	Gorai.013G229200.1	Gorai.
001G025000.1	.	Gorai.009G029100.1	Gorai.010G079100.1
Vv04g0049	.	Gorai.013G229200.1	Gorai.
001G025000.1	.	Gorai.003G061100.1	.	.	.
.	.	.	.	.
Vv11g0292	Tc09_g008580	.	.	.	.	.
.	.	.	.	.	.	.	.
.	.	.	.	.	.
Vv11g0293	Tc09_g008600	Gorai.013G229300.1	.
Gorai.004G282800.1	.	.	.	.	.	.
.	.	.	Vv09g0301	Tc06_g007170	.
.	.	.	.
Vv11g0294	Tc09_g008620	.	.	.	Gorai.
009G028900.1	Gorai.010G078900.1	.	.	.	.
.	.	.	.	.	.	.	.
.	.

Vv11g0295 Tc09_g008630 . . . Gorai.
009G028800.1 Gorai.010G078800.1 . . . .
. . . . . . . .
. .
Vv11g0296 Tc09_g008640 Gorai.013G229400.1 . .
Gorai.009G028700.1 Gorai.010G078700.1 . .
. . . . . . . .
. . . .
Vv11g0297 Tc09_g008650 . . . .
Gorai.010G078600.1 . . . . . .
. Vv09g0304 Tc06_g007180 . . .
. .
Vv11g0298 Tc09_g008660 . Gorai.001G009800.1 .
Gorai.009G028600.1 . . . . .
. . . . . . . .
. .
Vv11g0299 . . . . . . .
. . . . . . . .
. . . . .
Vv11g0300 Tc09_g008680 Gorai.013G229500.1 Gorai.
001G025300.1 . Gorai.009G028500.1 Gorai.010G078500.1
Vv04g0046 . Gorai.013G229500.1 Gorai.
001G025300.1 Gorai.004G282500.1 Gorai.003G061200.1 .
. . . . . . .
Vv11g0301 Tc09_g008690 . . Gorai.004G282400.1
Gorai.009G028400.1 Gorai.010G078400.1 . .
. . . . . Vv09g0307
Tc06_g007230 . . . . .
Vv11g0302 Tc09_g008700 . . Gorai.004G282300.1
Gorai.009G028300.1 Gorai.010G078300.1 . .
. . . . . . . .
. . . .
Vv11g0303 Tc09_g008710 . Gorai.001G009700.1 .
. . . . . . . .
. . . . . . . .
Vv11g0304 . . . . . . .
. . . . . . . .
. . . . .
Vv11g0305 . . . . . . .
. . . . . . . .
. . . . .
Vv11g0306 Tc09_g008730 . . Gorai.004G282200.1
Gorai.009G028100.1 . . . . .
. . . . . . . .
. .
Vv11g0307 Tc09_g008750 . . . Gorai.
009G028000.1 Gorai.010G078000.1 . . . .
. . . . . . . .
. .
Vv11g0308 . . . . . . .

.	.	.	.	.	.	.	.
.	.	.	.	.
Vv11g0309	Tc09_g008770	.	.	.	.
Gorai.010G077900.1	.	.	.	.	.	.
.	.	.	.	.	.	.	.
Vv11g0310	Tc09_g008790	.	Gorai.001G009400.1	.
.	Gorai.010G077800.1	.	.	.	.
.	.	.	.	.	.	.	.
.	.
Vv11g0311	Tc09_g008800	.	Gorai.001G009300.1
Gorai.004G282000.1	Gorai.009G027900.1	.	.	.
.	.	.	.	.	Vv09g0315
Tc06_g007260	.	.	.	.	.
Vv11g0312	.	.	Gorai.001G025700.1	.
Gorai.009G048100.1	.	.	.	.	.	.
.	.	Vv09g0316	Tc06_g007300	Gorai.
006G096700.1	.	.	.	.
Vv11g0313	Tc09_g008830	.	.	Gorai.004G281900.1
.	Gorai.010G077700.1	.	.	.	.
.	.	.	.	.	.	.	.
.	.
Vv11g0314	Tc09_g008850	.	.	.	.
Gorai.010G077400.1	.	.	.	.	.	.
.	Vv09g0317	Tc06_g007320	Gorai.006G096800.1
.	.	Gorai.011G070700.1	.
Vv11g0315	Tc09_g008880	.	.	.	.	.
.	.	.	.	.	.	.	.
.	.	.	.	.	.
Vv11g0316	.	.	.	.	.	.	.
.	.	.	.	.	.	.	.
.	.	.	.	.
Vv11g0317	.	.	.	.	.	Gorai.
010G077300.1	.	.	.	.	.	.	.
.	.	.	.	.	.	.
Vv11g0318	Tc09_g008900	Gorai.013G219800.1	.	.
Gorai.009G048300.1	.	.	.	.	.
.	.	.	.	.	.	.	.
.	.
Vv11g0319	.	.	.	.	.	.	.
.	.	.	.	.	.	.	.
.	.	.	.	.
Vv11g0320	Tc09_g008910	.	.	.	.	.
.	.	.	.	.	.	.	.
.	.	.	.	.	.
Vv11g0321	.	.	.	.	Gorai.009G027500.1
Gorai.010G077100.1	.	.	.	.	.
.	.	.	.	.	.	.	.
.
Vv11g0322	Tc09_g008920	.	.	.	.	.
.	.	.	.	.	.	.	.

. . . . . .
Vv11g0323 Tc09_g008940 . . Gorai.004G281800.1
. Gorai.010G077000.1 . . . .
. . . . . . . .
. .
Vv11g0324 Tc09_g008950 . . . Gorai.
009G027400.1 . . . . . . .
. . . . . . . .
Vv11g0325 Tc09_g008960 . . . . .
. . . . . . . .
. . . . . .
Vv11g0326 Tc09_g008970 . . . Gorai.
009G048500.1 . Vv04g0012 . . . .
. Gorai.011G068900.1 Vv09g0328
Tc06_g007390 Gorai.006G096900.1 . . Gorai.
011G071100.1 .
Vv11g0327 Tc09_g009000 . . . .
Gorai.010G076700.1 . . . . . .
. Vv09g0336 Tc06_g007440 . . .
Gorai.011G071300.1 .
Vv11g0328 Tc09_g009020 . . . .
Gorai.010G076300.1 . . . . . .
. . . . . . . .
Vv11g0329 Tc09_g009030 . Gorai.001G026200.1 .
Gorai.009G048600.1 . Vv04g0008 . .
Gorai.001G008900.1 . Gorai.003G061800.1
Gorai.011G071400.1 Vv09g0339 Tc06_g007460 . .
. Gorai.011G071400.1 .
Vv11g0330 . . . . . . .
. . . . . . . .
. . . . .
Vv11g0331 Tc09_g009050 . Gorai.001G008800.1 .
Gorai.009G057700.1 . . . . .
. . . . . . . .
. .
Vv11g0332 Tc09_g009060 . . Gorai.004G281700.1
. Gorai.010G076200.1 . . . .
. . . . . . . .
. .
Vv11g0333 Tc09_g009070 . Gorai.001G008700.1 .
. . Vv04g0004 . . Gorai.
001G008700.1 . Gorai.003G062600.1 Gorai.011G072200.1
. . . . . . .
Vv11g0334 Tc09_g009090 Gorai.013G219400.1 Gorai.
001G008600.1 . . . Vv04g0322 .
Gorai.013G219300.1 Gorai.001G008600.1 . . .
. . . . . . .
Vv11g0335 Tc09_g009100 . . . Gorai.
009G027300.1 . . . . . . .
. . . . . . . .

Vv11g0336 Tc09_g009110 . Gorai.001G008500.1 .
. . . . . . . .
. . . . . . . .
Vv11g0337 Tc09_g009120 . . . .
Gorai.010G076100.1 . . . . . . .
. . . . . . . .
Vv11g0338 Tc09_g009130 . . . .
Gorai.010G076000.1 . . . . . . .
. . . . . . . .
Vv11g0339 Tc09_g009140 Gorai.013G229700.1 . .
. Gorai.010G075800.1 . . . .
. . . . . . . .
. .
Vv11g0340 Tc09_g009150 Gorai.013G219200.1 Gorai.
001G008200.1 . Gorai.009G027200.1 Gorai.010G075600.1
Vv04g0337 . Gorai.013G219200.1 Gorai.
001G008200.1 Gorai.004G288600.1 Gorai.009G049700.1 .
. . . . . . .
Vv11g0341 Tc09_g009160 . . . .
Gorai.010G075400.1 . . . . . . .
. . . . . . . .
Vv11g0342 . . . . . . .
. . . . . . . .
. . . . .
Vv11g0343 Tc09_g009180 . Gorai.001G008000.1 .
. . . . . . . .
. . . . . . . .
Vv11g0344 Tc09_g009190 Gorai.013G219000.1 . .
Gorai.009G059200.1 . Vv04g0342 . .
. . . . . . . .
. . .
Vv11g0345 Tc09_g009200 Gorai.013G216000.1 Gorai.
001G007900.1 . Gorai.009G059300.1 . Vv04g0343
. Gorai.013G219000.1 Gorai.001G007900.1 .
. Gorai.010G107300.1 . . . .
. . .
Vv11g0346 Tc09_g009210 . . . .
Gorai.010G075200.1 . . . . . . .
. . . . . . . .
Vv11g0347 Tc09_g009220 . Gorai.001G007800.1 .
. Gorai.010G075100.1 . . . .
. . . . . . . .
. .
Vv11g0348 Tc09_g009240 . . . Gorai.
009G027000.1 Gorai.010G074900.1 . . . .
. . . . . . . .
. .
Vv11g0349 Tc09_g009250 . Gorai.001G007700.1 .
. Gorai.010G074800.1 . . . .
. . . . . . . .

. .
Vv11g0350 . . . . . . .
. . . . . . . .
. . . . .
Vv11g0351 Tc09_g009260 . Gorai.001G007600.1 .
. Gorai.010G074600.1 . . . .
. . . . . . . .
. .
Vv11g0352 Tc09_g009270 . . Gorai.004G281400.1
Gorai.009G026900.1 Gorai.010G074500.1 Vv04g0359
. . . Gorai.004G281400.1 .
Gorai.010G108000.1 . . . . . .
.
Vv11g0353 . . . . . . .
. . . . . . . .
. . . . .
Vv11g0354 Tc09_g009280 . Gorai.001G007500.1 .
. . . . . . . .
. . . . . . . .
Vv11g0355 Tc09_g009300 . Gorai.001G026300.1 .
Gorai.009G048800.1 Gorai.010G074400.1 . .
. . . . . . . .
. . . .
Vv11g0356 . . . . . . .
. . . . . . . .
. . . . .
Vv11g0357 Tc09_g009340 . . . .
Gorai.010G074000.1 . . . . . .
. . . . . . . .
Vv11g0358 Tc09_g009350 . . Gorai.004G281000.1
. . Vv04g0361 . . .
Gorai.004G281000.1 . . . . . .
. . .
Vv11g0359 Tc09_g009360 . Gorai.001G007300.1 .
Gorai.009G026800.1 Gorai.010G073900.1 . .
. . . . . . . .
. . . .
Vv11g0360 . . . . . . .
. . . . . . . .
. . . . .
Vv11g0361 Tc09_g009370 Gorai.013G230500.1 . .
. Gorai.010G073800.1 . . . .
. . . . . . . .
. .
Vv11g0362 Tc09_g009380 . . . .
Gorai.010G073700.1 . . . . . .
. . . . . . . .
Vv11g0363 Tc09_g009390 . Gorai.001G007200.1
Gorai.004G280900.1 . . . . . .
. . . . . . . .

. .
Vv11g0364 Tc09_g009400 . . . Gorai.
009G026700.1 . . . . . . .
. Vv09g0379 Tc06_g008030 . . .
. .
Vv11g0365 Tc09_g009410 . Gorai.001G007100.1 .
. . . . . . . .
. . . . . . . .
Vv11g0366 Tc09_g009420 . Gorai.001G007000.1 .
Gorai.009G026600.1 Gorai.010G073600.1 . .
. . . . . . . .
. . . .
Vv11g0367 Tc09_g009430 . Gorai.001G027100.1 .
. . . . . . . .
. . . . . . . .
Vv11g0368 Tc09_g009440 . . . Gorai.
009G026500.1 . . . . . . .
. . . . . . . .
Vv11g0369 . . . . . .
Vv04g0362 Tc01_g015360 . . . . .
. . . . . . .
Vv11g0370 . . . . . .
Vv04g0363 . . . . . .
Vv09g0385 Tc06_g008150 . . . . .
Vv11g0371 Tc09_g009460 . . Gorai.004G280800.1
Gorai.009G026300.1 . . . . .
. . . . . . . .
. .
Vv11g0372 Tc09_g009470 . . . Gorai.
009G026200.1 Gorai.010G073300.1 . . . .
. . . . . . . .
. .
Vv11g0373 Tc09_g009480 . Gorai.001G006600.1 .
Gorai.009G026100.1 . . . . .
. . . . . . . .
. .
Vv11g0374 Tc09_g009510 Gorai.013G230700.1 . .
Gorai.009G026000.1 . . . . .
. . . . . . . .
. .
Vv11g0375 Tc09_g009520 . . Gorai.004G280600.1
. . . . . . . .
. . . . . . . .
Vv11g0376 Tc09_g009530 . Gorai.001G006500.1 .
Gorai.009G025900.1 Gorai.010G073200.1 Vv04g0371
. . Gorai.001G006500.1 . .
Gorai.011G074200.1 . . . . . .
.
Vv11g0377 Tc09_g009550 . Gorai.001G006400.1
Gorai.004G280500.1 . Gorai.010G073100.1 . .

. . . . . . . .
. . . .
Vv11g0378 . . . . . . .
. . . . . . . .
. . . . .
Vv11g0379 Tc09_g009560 . Gorai.001G006100.1 .
. . . . . . . .
. . . . . . . .
Vv11g0380 Tc09_g009570 . . . Gorai.
009G025800.1 . . . . . . .
. . . . . . . .
Vv11g0381 Tc09_g009580 . Gorai.001G028000.1 .
Gorai.009G060500.1 Gorai.010G072900.1 Vv04g0379
. . Gorai.001G028100.1 . . .
. . . . . . .
Vv11g0382 Tc09_g009590 . Gorai.001G006000.1
Gorai.004G280400.1 . Gorai.010G072800.1 . .
. . . . . . . .
. . . .
Vv11g0383 . . Gorai.001G028200.1 . .
. Vv04g0382 . . . . .
. . . . . . . .
Vv11g0384 . . . . . . .
. . . . . . . .
. . . . .
Vv11g0385 Tc09_g009600 . Gorai.001G005900.1 .
. Gorai.010G072700.1 . . . .
. . . . . . . .
. .
Vv11g0386 Tc09_g009610 . . . .
Gorai.010G072600.1 . . . . . .
. . . . . . . .
Vv11g0387 Tc09_g009620 Gorai.013G230800.1 Gorai.
001G005800.1 . Gorai.009G025700.1 Gorai.010G072300.1
. . . . . . . .
. . . . . .
Vv11g0388 . . . . . . .
. . . . . . . .
. . . . .
Vv11g0389 Tc09_g009630 . . . Gorai.
009G025600.1 . . . . . . .
. Vv09g0400 . . . . .
.
Vv11g0390 Tc09_g009640 . . . Gorai.
009G025500.1 . . . . . . .
. . . . . . . .
Vv11g0391 Tc09_g009670 . . . .
Gorai.010G072200.1 . . . . . .
. . . . . . . .
Vv11g0392 . . . . . . .

. . . . . . . .
. . . . .
Vv11g0393 Tc09_g009680 . . . Gorai.
009G025400.1 Gorai.010G072100.1 Vv04g0390 . .
. Gorai.004G290800.1 . . . .
. . . . .
Vv11g0394 Tc09_g009690 . . . Gorai.
009G025300.1 . . . . . . .
. . . . . . . .
Vv11g0395 Tc09_g009700 . . Gorai.004G280300.1
. . . . . . . .
. . . . . . . .
Vv11g0396 . . . . Gorai.009G025200.1
. . . . . . . .
. . . . . . .
Vv11g0397 Tc09_g009720 . Gorai.001G005700.1
Gorai.004G280200.1 Gorai.009G025100.1 Gorai.010G072000.1
. . . . . . . .
. . . . . .
Vv11g0398 Tc09_g009730 . . . Gorai.
009G025000.1 . . . . . . .
. . . . . . . .
Vv11g0399 Tc09_g009740 Gorai.013G230900.1 Gorai.
001G005600.1 . . . . . . .
. . . . . . . .
. .
Vv11g0400 Tc09_g009750 . Gorai.001G005500.1 .
. . . . . . . .
. . . . . . . .
Vv11g0401 Tc09_g009770 . . . .
Gorai.010G071400.1 . . . . . .
. . . . . . . .
Vv11g0402 Tc09_g009780 . Gorai.001G005400.1 .
Gorai.009G024900.1 Gorai.010G071300.1 . .
. . . . . . . .
. . . .
Vv11g0403 Tc09_g009820 . Gorai.001G028900.1 .
Gorai.009G024800.1 Gorai.010G071100.1 Vv04g0396
. . Gorai.001G028900.1 . Gorai.
009G052000.1 Gorai.010G109400.1 . . . .
. . .
Vv11g0404 Tc09_g009830 . . Gorai.004G280100.1
Gorai.009G024700.1 Gorai.010G070900.1 . .
. . . . . . . .
. . . .
Vv11g0405 . . . . . .
Vv04g0427 . . . . . . .
. . . . . .
Vv11g0406 Tc09_g009850 . . Gorai.004G280000.1
Gorai.009G024600.1 . . . . .

. . . Vv09g0419 . . .
. . .
Vv11g0407 . . . . . . .
. . . . . . Vv09g0420
Tc06_g008550 . . . Gorai.011G075200.1
.
Vv11g0408 Tc09_g009860 . . . .
Gorai.010G070800.1 . . . . . .
. . . . . . . .
Vv11g0409 Tc09_g009870 . . Gorai.004G279900.1
Gorai.009G024400.1 Gorai.010G070700.1 . .
. . . . . Vv09g0423
Tc06_g008590 . . . . .
Vv11g0410 . . . . . . .
. . . . . . . .
. . . . .
Vv11g0411 Tc09_g009890 . . . .
Gorai.010G070600.1 . . . . . .
. . . . . . . .
Vv11g0412 Tc09_g009900 . Gorai.001G005200.1 .
. Gorai.010G070400.1 . . . .
. . . Vv09g0435 . . .
. . .
Vv11g0413 Tc09_g009920 . Gorai.001G005000.1 .
Gorai.009G024300.1 Gorai.010G070200.1 . .
. . . . . . . .
. . . .
Vv11g0414 . . . . . . .
. . . . . . . .
. . . . .
Vv11g0415 Tc09_g009940 . Gorai.001G004800.1 .
. Gorai.010G069900.1 . . . .
. . . Vv09g0439 Tc06_g008690 .
. . . .
Vv11g0416 Tc09_g009980 . . Gorai.004G279600.1
Gorai.009G024100.1 . . . . .
. . . . . . . .
. .
Vv11g0417 Tc09_g010000 . Gorai.001G004500.1 .
. . . . . . . .
. . . . . . . .
Vv11g0418 Tc09_g010030 . . . .
Gorai.010G069500.1 . . . . . .
. . . . . . . .
Vv11g0419 . . . . . . .
. . . . . . . .
. . . . .
Vv11g0420 . . . . . . .
. . . . . . . .
. . . . .

Vv11g0421 Tc09_g010040 . . . .
Gorai.010G069400.1 . . . . . . .
. . . . . . . .
Vv11g0422 Tc09_g010050 . . . Gorai.
009G023900.1 . . . . . . .
. . . . . . . .
Vv11g0423 Tc09_g010070 . Gorai.001G004200.1 .
. . . . . . . .
. Vv09g0447 Tc06_g010430 . . .
Gorai.011G076900.1 .
Vv11g0424 . . . . Gorai.009G023800.1
. . . . . . . .
. . . . . . .
Vv11g0425 Tc09_g010080 . Gorai.001G004100.1 .
. . . . . . . .
. . . . . . . .
Vv11g0426 . . . . . . .
. . . . . . Vv09g0454
. . . . . .
Vv11g0427 . . . . . . .
. . . . . . . .
. . . . .
Vv11g0428 . . . . . . .
. . . . . . . .
. . . . .
Vv11g0429 Tc09_g010090 . Gorai.001G004000.1 .
Gorai.009G023700.1 . . . . .
. . . . . . . .
. .
Vv11g0430 . . . . . . .
. . . . . . . .
. . . . .
Vv11g0431 Tc09_g010110 . Gorai.001G003900.1 .
Gorai.009G023600.1 . . . . .
. . . . . . . .
. .
Vv11g0432 Tc09_g010120 . . Gorai.004G279500.1
Gorai.009G012100.1 Gorai.010G069100.1 . .
. . . . . Vv09g0461
Tc06_g000240 . . Gorai.009G115200.1 . .
Vv11g0433 Tc09_g010130 . . . . .
. . . . . . .
Vv09g0462 . . . . . .
Vv11g0434 Tc09_g010140 . Gorai.001G003800.1 .
Gorai.009G023400.1 . . . . .
. . . . . . . .
. .
Vv11g0435 . . . . . . .
. . . . . . . .
. . . . .

```
Vv11g0436       Tc09_g010160    .       .       .       Gorai.
009G023200.1    .       Vv04g0455       .       .       .       .
        .       .       .       .       .       .       .       .
        .
Vv11g0437       .       .       .       .       .       .       .
        .       .       .       .       .       .       .       .
        .       .       .       .       .
Vv11g0438       Tc09_g010170    .       .       .       Gorai.
009G023100.1    .       .       .       .       .       .       .
        .       .       .       .       .       .       .       .
Vv11g0439       .       .       .       .       .       .       .
        .       .       .       .       .       .       .       .
        .       .       .       .       .
Vv11g0440       Tc09_g010180    .       .       .       Gorai.
009G023000.1    Gorai.010G069000.1      .       .       .       .
        .       .       .       .       .       .       .       .
        .       .
Vv11g0441       .       .       .       .       .       .       .
        .       .       .       .       .       .       .       .
        .       .       .       .       .
Vv11g0442       Tc09_g010190    Gorai.013G231000.1      Gorai.
001G003700.1    .       .       .       .       .       .       .
        .       .       .       .       .       .       .       .
        .       .
Vv11g0443       Tc09_g010200    .       .       Gorai.004G279400.1
        .       .       .       .       .       .       .       .
        .       .       .       .       .       .       .       .
Vv11g0444       Tc09_g010210    .       .       .       .       .
        .       .       .       .       .       .       .       .
        .       .       .       .       .       .
Vv11g0445       .       .       .       .       .       .       .
        .       .       .       .       .       .       .       .
        .       .       .       .       .
Vv11g0446       .       .       .       .       .       .       .
        .       .       .       .       .       .       .       .
        .       .       .       .       .
Vv11g0447       .       .       .       .       .       .       .
        .       .       .       .       .       .       .       .
        .       .       .       .       .
Vv11g0448       .       .       .       .       .       Gorai.
010G068900.1    .       .       .       .       .       .       .
        .       .       .       .       .       .       .
Vv11g0449       .       .       .       .       .       .       .
        .       .       .       .       .       .       .       .
        .       .       .       .       .
Vv11g0450       .       .       .       .       .       .       .
        .       .       .       .       .       .       .       .
        .       .       .       .       .
Vv11g0451       Tc09_g010230    .       .       .       .
Gorai.010G068800.1      .       .       .       .       .       .
```

. . . . . . . .
Vv11g0452 . . . . . . .
. . . . . . . .
. . . . .
Vv11g0453 Tc09_g010260 . . . . .
. . . . . . . .
. . . . . .
Vv11g0454 Tc09_g010270 . . . Gorai.
009G012000.1 . . . . . . .
. . . . . . . .
Vv11g0455 Tc09_g010280 . Gorai.001G003600.1 .
Gorai.009G022800.1 . . . . .
. . . . . . . .
. .
Vv11g0456 Tc09_g010290 . . . . .
. . . . . . . .
. . . . . .
Vv11g0457 Tc09_g010300 . . . .
Gorai.010G068700.1 Vv04g0492 . . . .
. . . . . . . .
.
Vv11g0458 . . . . . . .
. . . . . . . .
. . . . .
Vv11g0459 Tc09_g010330 . . . Gorai.
009G022700.1 Gorai.010G068600.1 . . . .
. . . . . . . .
. .
Vv11g0460 . . . . . . .
. . . . . . . .
. . . . .
Vv11g0461 . . . . . . .
. . . . . . . .
. . . . .
Vv11g0462 Tc09_g010340 . . . . .
. . . . . . . .
. . . . . .
Vv11g0463 Tc09_g010350 . . . Gorai.
009G011400.1 . . . . . . .
. . . . . . . .
Vv11g0464 . . . . . . .
. . . . . . . .
. . . . .
Vv11g0465 Tc09_g010360 Gorai.013G231200.1 Gorai.
001G003300.1 Gorai.004G279300.1 . Gorai.010G068500.1
. . . . . . . .
. . . . . .
Vv11g0466 Tc09_g010370 . . Gorai.004G279200.1
. . . . . . . .
. Vv09g0490 Tc06_g007330 . . .

. .
Vv11g0467 Tc09_g010380 Gorai.013G231300.1 . .
Gorai.009G022600.1 Gorai.010G068400.1 . .
. . . . . . . .
. . . .
Vv11g0468 Tc09_g010390 . . . .
Gorai.010G068300.1 . . . . . .
. . . . . . . .
Vv11g0469 . . . . . . .
. . . . . . . .
. . . . .
Vv11g0470 Tc09_g010400 . . . .
Gorai.010G068100.1 . . . . . .
. . . . . . . .
Vv11g0471 Tc09_g010420 . . . Gorai.
009G022400.1 Gorai.010G067900.1 . . . .
. . . . . . . .
. .
Vv11g0472 . . . . . . .
. . . . . . . .
. . . . .
Vv11g0473 Tc09_g010440 . . . .
Gorai.010G067800.1 . . . . . .
. . . . . . . .
Vv11g0474 Tc09_g010450 Gorai.013G231400.1 . .
. Gorai.010G067700.1 . . . .
. . . . . . . .
. .
Vv11g0475 Tc09_g010460 Gorai.013G231600.1 Gorai.
001G003200.1 Gorai.004G278900.1 Gorai.009G022300.1
Gorai.010G067400.1 . . . . . .
. . . . . . . .
Vv11g0476 Tc09_g010470 . . . Gorai.
009G022200.1 . . . . . . .
. . . . . . . .
Vv11g0477 Tc09_g010480 . Gorai.001G003100.1 .
. . . . . . . .
. . . . . . . .
Vv11g0478 Tc09_g010550 . Gorai.001G002800.1 .
Gorai.009G022100.1 Gorai.010G067300.1 . .
. . . . . . . .
. . . .
Vv11g0479 Tc09_g010560 . . . Gorai.
009G022000.1 . . . . . . .
. . . . . . . .
Vv11g0480 Tc09_g010570 . . . Gorai.
009G021900.1 . . . . . . .
. . . . . . . .
Vv11g0481 . . . . . . .
. . . . . . . .

. . . . .
Vv11g0482 Tc09_g010590 . . . .
Gorai.010G066900.1 . . . . . .
. . . . . . . .
Vv11g0483 . . . . . . .
. . . . . . . .

. . . . .
Vv11g0484 Tc09_g010600 Gorai.013G232400.1 .
Gorai.004G278800.1 Gorai.009G021600.1 . . .
. . . . . . . .

. . . .
Vv11g0485 Tc09_g010610 . . . Gorai.
009G021500.1 . . . . . . .
. . . . . . . .
Vv11g0486 . . . . . . .
. . . . . . . .

. . . . .
Vv11g0487 Tc09_g010630 . . . Gorai.
009G021100.1 . . . . . . .
. . . . . . . .
Vv11g0488 Tc09_g010640 . . Gorai.004G278700.1
Gorai.009G021000.1 Gorai.010G066800.1 . .
. . . . . . . .

. . . .
Vv11g0489 Tc09_g010660 . . Gorai.004G278600.1
Gorai.009G020900.1 Gorai.010G066700.1 . .
. . . . . . . .

. . . .
Vv11g0490 . . . . . . .
. . . . . . . .

. . . . .
Vv11g0491 . Gorai.013G232700.1 . . .
. . . . . . . .

. . . . . . .
Vv11g0492 Tc09_g010720 . . . Gorai.
009G020600.1 . . . . . . .
. . . . . . . .
Vv11g0493 Tc09_g010730 . . . Gorai.
009G010600.1 . Vv04g0512 . . .
Gorai.004G274500.1 . . Vv09g0567
Tc06_g006590 . Gorai.010G227500.1 . . .
Vv11g0494 Tc09_g010740 Gorai.013G218200.1 Gorai.
001G002700.1 Gorai.004G278500.1 . Gorai.010G066400.1
. . . . . . . .

. . . . . .
Vv11g0495 Tc09_g010750 Gorai.013G232900.1 .
Gorai.004G278400.1 . Gorai.010G066300.1 . .
. . . . . . . .

. . . .
Vv11g0496 Tc09_g010760 . . . .

Gorai.010G066200.1 . . . . . .
. . . . . . . .
Vv11g0497 Tc09_g010770 . . Gorai.004G278300.1
. Gorai.010G066100.1 . . . .
. . . . . . . .
. .
Vv11g0498 Tc09_g010780 . Gorai.001G002600.1
Gorai.004G278200.1 . Gorai.010G065800.1 . .
. . . . . . . .
. . . .
Vv11g0499 . . . . . . .
. . . . . . . .
. . . . .
Vv11g0500 Tc09_g010790 . . . .
Gorai.010G065700.1 Vv04g0518 . . . .
. Gorai.011G080400.1 . . . .
. . .
Vv11g0501 . . . . . . .
. . . . . . . .
. . . . .
Vv11g0502 Tc09_g010810 . . . Gorai.
009G020400.1 . . . . . . .
. . . . . . . .
Vv11g0503 Tc09_g010820 . Gorai.001G002500.1 .
. Gorai.010G065600.1 . . . .
. . . . . . . .
. .
Vv11g0504 . . . . . . .
. . . . . . . .
. . . . .
Vv11g0505 . . . . . . .
. . . . . . . .
. . . . .
Vv11g0506 Tc09_g010830 Gorai.013G233000.1 . .
Gorai.009G020100.1 Gorai.010G065500.1 Vv04g0526
. Gorai.013G233000.1 . . .
Gorai.011G080600.1 Vv09g0581 . . Gorai.
010G227200.1 Gorai.009G129700.1 Gorai.011G080600.1 .
Vv11g0507 Tc09_g010840 Gorai.013G233100.1 Gorai.
001G002400.1 . Gorai.009G020000.1 . . .
. . . . . . . .
. . . .
Vv11g0508 Tc09_g010880 . Gorai.001G001400.1
Gorai.004G278000.1 Gorai.009G019900.1 . . .
. . . . . Vv09g0585 .
. . Gorai.009G129800.1 . .
Vv11g0509 Tc09_g010890 . . . . .
. . . . . . . .
. . . . . .
Vv11g0510 . . . . . . .

. . . . . . . .
. . . . .
Vv11g0511 Tc09_g010900 . . . Gorai.
009G019800.1 . . . . . . .
. . . . . . . .
Vv11g0512 Tc09_g010920 . . . Gorai.
009G019600.1 . . . . . . .
. . . . . . . .
Vv11g0513 . . . . . . .
. . . . . . . .
. . . . .
Vv11g0514 . . . . Gorai.009G019400.1
Gorai.010G065100.1 . . . . .
. . . . . . . .
.
Vv11g0515 Tc09_g010960 . Gorai.001G040700.1 .
. . . . . . . .
. Vv09g0595 . . . . .
.
Vv11g0516 Tc09_g010970 Gorai.013G233600.1 .
Gorai.004G277900.1 . . . . . .
. . . . . . . .
. .
Vv11g0517 Tc09_g010980 . Gorai.001G040900.1 .
Gorai.009G019200.1 . . . . .
. . . Vv09g0609 . . .
. . .
Vv11g0518 . . . . . . .
. . . . . . . .
. . . . .
Vv11g0519 Tc09_g011000 . . Gorai.004G277800.1
. Gorai.010G064900.1 . . . .
. . . . . . . .
. .
Vv11g0520 Tc09_g011030 Gorai.013G233700.1 . .
. . . . . . . .
. . . . . . . .
Vv11g0521 Tc09_g011040 . Gorai.001G041000.1 .
. Gorai.010G064800.1 . . . .
. . . Vv09g0614 . . .
. . .
Vv11g0522 Tc09_g011050 . . . . .
. . . . . . . .
. . . . . .
Vv11g0523 . . . . . . .
. . . . . . . .
. . . . .
Vv11g0524 Tc09_g011060 . . . . .
. . . . . .
Vv09g0620 . . . . . .

Vv11g0525	Tc09_g011070	.	Gorai.001G041100.1	.
Gorai.009G019100.1	.	.	.	.	.
.	.	.	.	.	.	.	.
.	.
Vv11g0526	Tc09_g011080	.	Gorai.001G041200.1
Gorai.004G277700.1	.	.	.	.	.	.
.	.	.	.	.	.	.	.
.	.
Vv11g0527	Tc09_g011090	.	Gorai.001G041300.1	.
.	Gorai.010G064700.1	.	.	.	.
.	.	.	.	.	.	.	.
.	.
Vv11g0528	Tc09_g011100	.	Gorai.001G041400.1
Gorai.004G277600.1	.	Gorai.010G064600.1	.	.
.	.	.	.	.	.	.	.
.	.	.	.
Vv11g0529	Tc09_g011110	.	.	.	Gorai.
009G018600.1	.	.	.	.	.	.	.
.	.	.	.	.	.	.	.
Vv11g0530	Tc09_g011120	.	.	Gorai.004G277500.1
.	.	.	.	.	.	.	.
.	.	.	.	.	.	.	.
Vv11g0531	Tc09_g011130	Gorai.013G233800.1	.	.
Gorai.009G010100.1	Gorai.010G064500.1	Vv04g0542
.	Gorai.013G233800.1	.	Gorai.004G274000.1
.	.	Vv09g0626	.	.	.	.
.	.
Vv11g0532	Tc09_g011140	.	Gorai.001G041500.1	.
Gorai.009G010000.1	.	.	.	.	.
.	.	.	.	.	.	.	.
.	.
Vv11g0533	Tc09_g011170	Gorai.013G233900.1	.	.
Gorai.009G007300.1	.	.	.	.	.
.	.	.	.	.	.	.	.
.	.
Vv11g0534	Tc09_g011190	.	.	.	.
Gorai.010G064400.1	.	.	.	.	.	.
.	Vv09g0634	.	.	.	Gorai.
009G130600.1	Gorai.011G082100.1	.
Vv11g0535	Tc09_g011200	.	.	Gorai.004G277400.1
.	Gorai.010G064300.1	.	.	.	.
.	.	.	.	.	.	.	.
.	.
Vv11g0536	Tc09_g011220	.	.	.	.	.
.	.	.	.	.	.	.	.
.	.	.	.	.	.
Vv11g0537	.	.	.	.	.	.	.
.	.	.	.	.	.	.	.
.	.	.	.	.
Vv11g0538	Tc09_g011230	.	Gorai.001G041900.1	.

. . . . . . . .
. . . . . . . .
Vv11g0539 . . . . . . .
. . . . . . . .
. . . . .
Vv11g0540 Tc09_g011240 . . . .
Gorai.010G064200.1 Vv04g0548 . Gorai.013G215400.1
. . . . . . . .
. . .
Vv11g0541 Tc09_g011250 Gorai.013G215300.1 . .
Gorai.009G057400.1 . Vv04g0549 .
Gorai.013G215300.1 . . Gorai.009G057400.1
Gorai.010G114100.1 . . . . . .
.
Vv11g0542 Tc09_g011260 . . Gorai.004G277100.1
. . . . . . . .
. . . . . . . .
Vv11g0543 . . . . . . .
. . . . . . . .
. . . . .
Vv11g0544 Tc09_g011290 . . . Gorai.
009G017900.1 . . . . . . .
. . . . . . . .
Vv11g0545 Tc09_g011300 . . . . .
. . . . . . . .
. . . . . .
Vv11g0546 . . . . . . .
. . . . . . . .
. . . . .
Vv11g0547 . . Gorai.001G042000.1 . .
. . . . . . . .
. . . . . . .
Vv11g0548 Tc09_g011320 . . . .
Gorai.010G064100.1 . . . . . .
. . . . . . . .
Vv11g0549 Tc09_g011330 . . . .
Gorai.010G064000.1 . . . . . .
. . . . . . . .
Vv11g0550 Tc09_g011340 Gorai.013G234000.1 . .
. . . . . . . .
. . . . . . . .
Vv11g0551 . . . . . . .
. . . . . . . .
. . . . .
Vv11g0552 . . . . . . .
. . . . . . . .
. . . . .
Vv11g0553 . . . . . . .
. . . . . . . .
. . . . .

Vv11g0554 . . . . . Gorai.
010G063900.1 Vv04g0555 . Gorai.013G234000.1 .
. . Gorai.010G114400.1 . . .
. . . .
Vv11g0555 Tc09_g011350 Gorai.013G234100.1 . .
Gorai.009G017800.1 Gorai.010G063800.1 . .
. . . . . . . .
. . . .
Vv11g0556 . . . . . . .
. . . . . . . .
. . . . .
Vv11g0557 Tc09_g011360 . . . Gorai.
009G017700.1 . . . . . . .
. . . . . . . .
Vv11g0558 Tc09_g011370 . Gorai.001G042100.1 .
Gorai.009G017600.1 . Vv04g0565 . .
Gorai.001G042100.1 . . Gorai.011G082600.1
Vv09g0648 Tc06_g001600 . . Gorai.
009G130800.1 Gorai.011G082600.1 .
Vv11g0559 . . . . . . .
. . . . . . . .
. . . . .
Vv11g0560 Tc09_g011380 . Gorai.001G033200.1 .
Gorai.009G017500.1 . . . . .
. . . . . . . .
. .
Vv11g0561 Tc09_g011390 . . . Gorai.
009G017400.1 . . . . . . .
. . . . . . . .
Vv11g0562 . . . . . . .
. . . . . . . .
. . . . .
Vv11g0563 . . . . . . .
. . . . . . . .
. . . . .
Vv11g0564 . . . . . . .
. . . . . . Vv09g0652
Tc06_g001640 . Gorai.010G225600.1 .
Gorai.011G083000.1 .
Vv11g0565 . . . . . . .
. . . . . . . .
. . . . .
Vv11g0566 . . . . . . .
. . . . . . . .
. . . . .
Vv11g0567 Tc09_g011400 . Gorai.001G033300.1 .
Gorai.009G057800.1 . Vv04g0569 . .
Gorai.001G042400.1 . Gorai.009G057800.1
Gorai.011G083000.1 . . . . . .
.

Vv11g0568 . . . . . . .
. . . . . . . .
. . . . .
Vv11g0569 Tc09_g011420 . . . .
Gorai.010G063500.1 . . . . . .
. . . . . . . .
Vv11g0570 . . . . . . .
. . . . . . . .
. . . . .
Vv11g0571 Tc09_g011440 Gorai.013G215000.1 Gorai.
001G033400.1 Gorai.004G276800.1 Gorai.009G058000.1 .
. . . . . . . .
. . . . . .
Vv11g0572 Tc09_g011460 . . . .
Gorai.010G063300.1 . . . . . .
. . . . . . . .
Vv11g0573 Tc09_g011490 Gorai.013G234200.1 Gorai.
001G033500.1 . Gorai.009G016900.1 Gorai.010G063200.1
. . . . . . .
Vv09g0659 Tc06_g001700 . . Gorai.009G131300.1
. .
Vv11g0574 . . . . . . .
. . . . . . . .
. . . . .
Vv11g0575 Tc09_g011500 Gorai.013G234300.1 . .
. Gorai.010G063100.1 . . . .
. . . Vv09g0660 . . .
. . .
Vv11g0576 . Gorai.013G214800.1 Gorai.001G033600.1
. Gorai.009G058200.1 . Vv04g0573 .
Gorai.013G185900.1 Gorai.001G033600.1 .
Gorai.009G058200.1 Gorai.010G115100.1 . . .
. . . .
Vv11g0577 Tc09_g011540 . . . .
Gorai.010G063000.1 . . . . . .
. . . . . . . .
Vv11g0578 . . . . . . .
. . . . . . . .
. . . . .
Vv11g0579 . . . . . . .
. . . . . . . .
. . . . .
Vv11g0580 . . . . . . .
. . . . . . . .
. . . . .
Vv11g0581 Tc09_g031810 Gorai.013G212100.1 . .
. . . . . . . .
. . . . . . . .
Vv11g0582 . . . Gorai.004G276600.1 .
. . . . . . . .

. . . . . . .
Vv11g0583 . . Gorai.001G033800.1 .
Gorai.009G058400.1 . Vv04g0577 . Gorai.
013G234700.1 Gorai.001G033800.1 . Gorai.009G058400.1
Gorai.010G115300.1 . . . . .
. .
Vv11g0584 Tc09_g011650 . . . . .
. . . . . . . .
. . . . . .
Vv11g0585 Tc09_g011660 Gorai.013G234400.1 . .
Gorai.009G016500.1 . . . . .
. . . . . . . .
. .
Vv11g0586 Tc09_g011670 Gorai.013G234500.1 . .
Gorai.009G016300.1 Gorai.010G062600.1 . .
. . . . . . . .
. . . .
Vv11g0587 Tc09_g031660 . . . . .
. . . . . . .
Vv09g0666 Tc06_g001730 . . . Gorai.
011G084000.1 .
Vv11g0588 . . . . . . .
. . . . . . . .
. . . . .
Vv11g0589 . . Gorai.001G043000.1 . .
. . . . . . . .
. . . . . . .
Vv11g0590 . . . . . . .
. . . . . . . .
. . . . .
Vv11g0591 . . . . . . .
. . . . . . . .
. . . . .
Vv11g0592 . . . . . . .
. . . . . . . .
. . . . .
Vv11g0593 . . . . . . .
. . . . . . . .
. . . . .
Vv11g0594 . . Gorai.001G043700.1 . .
. . . . . . . .
. . . . . . .
Vv11g0595 . . . . . . .
. . . . . . . .
. . . . .
Vv11g0596 Tc09_g011740 . . . Gorai.
009G059700.1 Gorai.010G062200.1 . . . .
. . . . . . . .
. .
Vv11g0597 Tc09_g011760 . Gorai.001G043800.1 .

. . . . . . . .
. . . . . . . .
Vv11g0598 . . . . . . .
. . . . . . . .
. . . . .
Vv11g0599 . . . . . . .
. . . . . . . .
. . . . .
Vv11g0600 . . . . . . .
. . . . . . . .
. . . . .
Vv11g0601 . . . . . . .
. . . . . . . .
. . . . .
Vv11g0602 . . . . . . .
. . . . . . . .
. . . . .
Vv11g0603 . . . Gorai.004G276400.1
Gorai.009G016100.1 . . . . . .
. . . . . . . .
.
Vv11g0604 . . . . . . .
. . . . . . . .
. . . . .
Vv11g0605 Tc09_g011800 . . . . .
. . . . . . . .
. . . . . .
Vv11g0606 Tc09_g011810 . . . .
Gorai.010G061900.1 . . . . . .
. . . . . . . .
Vv11g0607 . . . . . . .
. . . . . . . .
. . . . .
Vv11g0608 Tc09_g011820 . Gorai.001G034200.1 .
Gorai.009G016000.1 . Vv04g0583 . .
Gorai.001G034200.1 . . . . .
. . . . .
Vv11g0609 . . . . . . .
. . . . . . . .
. . . . .
Vv11g0610 Tc09_g011840 . Gorai.001G044200.1 .
Gorai.009G015800.1 . . . . .
. . . . . . . .
. .
Vv11g0611 Tc09_g011850 Gorai.013G234800.1 . .
Gorai.009G015700.1 . . . . .
. . . . . . . .
. .
Vv11g0612 . . . . . . .
. . . . . . . .

. . . . .
Vv11g0613 . . Gorai.004G107300.1 . .
Gorai.010G061600.1 . . . . .
. . . . . . . .
.
Vv11g0614 Tc09_g011880 . . . Gorai.
009G015600.1 . . . . . . .
. . . . . . . .
Vv11g0615 Tc09_g011890 . Gorai.004G107400.1 .
. . . . . . . .
. . . . . . . .
Vv11g0616 Tc09_g011920 Gorai.013G235200.1 . .
. . . . . . . .
. . . . . . . .
Vv11g0617 . Gorai.013G218200.1 Gorai.001G029400.1
. . . . . . . .
. . . . . . . .
.
Vv11g0618 Tc09_g011930 Gorai.013G235300.1 Gorai.
004G107500.1 . . . . . . .
. . . . . . . .
. .
Vv11g0619 . . . . . . .
. . . . . . . .
. . . . .
Vv11g0620 Tc09_g011940 . Gorai.004G107700.1
Gorai.004G275600.1 Gorai.009G015500.1 . Vv04g0590
Tc01_g038070 Gorai.013G214600.1 Gorai.001G034700.1
. . . . . . . .
. .
Vv11g0621 Tc09_g011950 . Gorai.001G034800.1 .
Gorai.009G058900.1 Gorai.010G061200.1 Vv04g0592
Tc09_g003010 Gorai.013G214400.1 Gorai.001G034800.1
. Gorai.009G058900.1 . . . .
. . . .
Vv11g0622 Tc09_g011990 . . Gorai.004G274700.1
. . . . . . . .
. . . . . . . .
Vv11g0623 Tc09_g012000 . . . Gorai.
009G059000.1 Gorai.010G061000.1 Vv04g0595 . .
. . Gorai.009G059000.1 . Vv09g0671
. . . . . .
Vv11g0624 . . . . . . .
. . . . . . . .
. . . . .
Vv11g0625 Tc09_g012010 . . . .
Gorai.010G060900.1 . . . . . .
. . . . . . . .
Vv11g0626 Tc09_g012020 . . Gorai.004G271900.1
Gorai.009G058500.1 Gorai.010G060800.1 . .

. . . . . . . .
. . . .
Vv11g0627 Tc09_g012030 . Gorai.004G107900.1 .
. . . . . . . .
. . . . . . . .
Vv11g0628 . . . . . . .
. . . . . . . .
. . . . .
Vv11g0629 . . . . . . .
. . . . . . . .
. . . . .
Vv11g0630 . . . . . Gorai.
010G058500.1 . . . . . . .
. . . . . . .
Vv11g0631 . . . . . . .
. . . . . . . .
. . . . .
Vv11g0632 . . . . . . .
. . . . . . . .
. . . . .
Vv11g0633 . . . . . . .
. . . . . . . .
. . . . .
Vv11g0634 . . . . . . .
. . . . . . . .
. . . . .
Vv11g0635 . . . . . .
Vv04g0609 . . Gorai.001G037200.1 .
Gorai.009G061900.1 . . . . . .
. .
Vv11g0636 . . . . . . .
. . . . . . . .
. . . . .
Vv11g0637 . . . . . .
Vv04g0611 Tc01_g002260 . Gorai.001G031700.1 .
Gorai.009G061700.1 . . . . .
. . .
Vv11g0638 . . . Gorai.004G291600.1 .
. . . . . . . .
. . . . . . .
Vv11g0639 . . . . . . .
. . . . . . . .
. . . . .
Vv11g0640 . . . . . . .
. . . . . . . .
. . . . .
Vv11g0641 . . . . . . .
. . . . . . . .
. . . . .
Vv11g0642 . . Gorai.001G031500.1 .

Gorai.009G061200.1 . Vv04g0617 Tc09_g027670 .
Gorai.001G031500.1 . Gorai.009G061200.1 .
. . . . . . .
Vv11g0643 . Gorai.013G217200.1 . . .
. . . . . . . .
. . . . . . .
Vv11g0644 . . . . . . .
. . . . . . . .
. . . . .
Vv11g0645 . . . . . . .
. . . . . . . .
. . . . .
Vv11g0646 Tc09_g005290 . Gorai.001G032200.1 .
. . . . . . . .
. . . . . . . .
Vv11g0647 . Gorai.013G216100.1 . .
Gorai.009G057100.1 . . . . . .
. . . . . . . .
.
Vv11g0648 . . . . Gorai.009G057000.1
. . . . . . . .
. . . . . . .
Vv11g0649 . . . . . . .
. . . . . . . .
. . . . .
Vv11g0650 . Gorai.013G216000.1 Gorai.001G032300.1
. . . . . . . .
. . . . . . . .
.
Vv11g0651 . . . Gorai.004G291800.1 .
. . . . . . . .
. . . . . . .
Vv11g0652 . . . . . . .
. . . . . . . .
. . . . .
Vv11g0653 . . . . . . .
. . . . . . . .
. . . . .
Vv11g0654 . Gorai.013G215900.1 . .
Gorai.009G063000.1 . . . . . .
. . . . . . . .
.
Vv11g0655 . . . . . . .
. . . . . . . .
. . . . .
Vv11g0656 . Gorai.013G215800.1 . .
Gorai.009G056700.1 . . . . . .
. . . . . . . .
.
Vv11g0657 . . . . . . .

. . . . . . . .
. . . . .
Vv11g0658 . . . . Gorai.009G056600.1
. . . . . . . .
. . . . . . .
Vv11g0659 . . . Gorai.004G291900.1 .
. . . . . . . .
. . . . . . .
Vv11g0660 . . . . . . .
. . . . . . . .
. . . . .
Vv11g0661 . . . . . . .
. . . . . . . .
. . . . .
Vv11g0662 . . . . . . .
. . . . . . . .
. . . . .
Vv11g0663 . . Gorai.001G038300.1 .
Gorai.009G063200.1 . . . . . .
. . . . . . . .
.
Vv11g0664 . . . . . . .
. . . . . . . .
. . . . .
Vv11g0665 . . . . . . .
. . . . . . . .
. . . . .
Vv11g0666 . . Gorai.001G032400.1 . .
. . . . . . . .
. . . . . . .
Vv11g0667 . . . Gorai.004G292100.1 .
. . . . . . . .
. . . . . . .
Vv11g0668 . . . . . . .
. . . . . . . .
. . . . .
Vv11g0669 . . . . . . .
. . . . . . . .
. . . . .
Vv11g0670 . . . . . . .
. . . . . . . .
. . . . .
Vv11g0671 . . . . . . .
. . . . . . . .
. . . . .
Vv11g0672 . . . . . . .
. . . . . . . .
. . . . .
Vv11g0673 . . . . . . .
. . . . . . . .

. . . . .
Vv11g0674 . . . . . . .
. . . . . . . .
. . . . .
Vv11g0675 . . Gorai.001G032800.1 Gorai.
004G292200.1 Gorai.009G056200.1 . . . .
. . . . . . . .
. . .
Vv11g0676 . . Gorai.001G032900.1 . .
. . . . . . . .
. . . . . . .
Vv11g0677 . . . . Gorai.009G056100.1
. . . . . . . .
. . . . . . .
Vv11g0678 . . . . . . .
. . . . . . . .
. . . . .
Vv11g0679 Tc09_g005640 . . . Gorai.
009G056000.1 . . . . . . .
. . . . . . . .
Vv11g0680 . Gorai.013G214400.1 . .
Gorai.009G055900.1 . . . . . .
. . . . . . . .
.
Vv11g0681 . . . . . . .
. . . . . . . .
. . . . .
Vv11g0682 Tc09_g014730 Gorai.013G241400.1 . .
. . . . . . . .
. . . . . . . .
Vv11g0683 . . . . . . .
. . . . . . . .
. . . . .
Vv11g0684 Tc09_g014720 Gorai.013G241300.1 .
Gorai.004G273900.1 Gorai.009G009800.1 . . .
. . . . . . . .
. . . .
Vv11g0685 . . . . . . .
. . . . . . . .
. . . . .
Vv11g0686 Tc09_g014710 . . . . .
. . . . . . . .
. . . . . .
Vv11g0687 . . . . . . .
. . . . . . . .
. . . . .
Vv11g0688 Tc09_g014700 . . . . .
. . . . . . . .
. . . . . .
Vv11g0689 Tc09_g014690 . . . Gorai.

009G009700.1 . . . . . . .
. . . . . . . .
Vv11g0690 Tc09_g014680 . . . Gorai.
009G009600.1 . . . . . . .
. . . . . . . .
Vv11g0691 Tc09_g014670 . . Gorai.004G273800.1
. . . . . . . .
. . . . . . . .
Vv11g0692 . . . . . . .
. . . . . . . .
. . . . .
Vv11g0693 . . . . . . .
. . . . . . . .
. . . . .
Vv11g0694 . . . . . . .
. . . . . . . .
. . . . .
Vv11g0695 . . . . . . .
. . . . . . . .
. . . . .
Vv11g0696 . . . . . . .
. . . . . . . .
. . . . .
Vv11g0697 . . . . . . .
. . . . . . . .
. . . . .
Vv11g0698 . . . . . . .
. . . . . . . .
. . . . .
Vv11g0699 Tc09_g014570 . . . . .
. . . . . . . .
. . . . . .
Vv11g0700 Tc09_g014560 . Gorai.004G107100.1
Gorai.004G273600.1 . . . . . .
. . . . . . . .
. .
Vv11g0701 Tc09_g014550 . . . . .
. . . . . . . .
. . . . . .
Vv11g0702 . . . . . . .
. . . . . . . .
. . . . .
Vv11g0703 Tc09_g014520 . . . Gorai.
009G009300.1 . . . . . . .
. . . . . . . .
Vv11g0704 . . . . . . .
. . . . . . . .
. . . . .
Vv11g0705 Tc09_g005700 . . . . .
. . . . . . . .

. . . . . .
Vv11g0706 . . . . . . .
. . . . . . . .
. . . . .
Vv11g0707 . . . . . . .
. . . . . . . .
. . . . .
Vv11g0708 . . . . . . .
. . . . . . . .
. . . . .
Vv11g0709 . . . . . . .
. . . . . . . .
. . . . .
Vv11g0710 . . . . . . .
. . . . . . . .
. . . . .
Vv11g0711 . . . . . . .
. . . . . . . .
. . . . .
Vv11g0712 . . . . . . .
. . . . . . . .
. . . . .
Vv11g0713 . . . . . . .
. . . . . . . .
. . . . .
Vv11g0714 . . . . . . .
. . . . . . . .
. . . . .
Vv11g0715 . . . . . . .
. . . . . . . .
. . . . .
Vv11g0716 . . . . . . .
. . . . . . . .
. . . . .
Vv11g0717 . . . . . . .
. . . . . . . .
. . . . .
Vv11g0718 . . . . . . .
. . . . . . . .
. . . . .
Vv11g0719 . . . . Gorai.009G009100.1
. . . . . . . .
. . . . . . .
Vv11g0720 . . . . . . .
. . . . . . . .
. . . . .
Vv11g0721 . . . . . . .
. . . . . . . .
. . . . .
Vv11g0722 . . . . . . .

. . . . . . . .
. . . . .
Vv11g0723 . . . . . . .
. . . . . . . .
. . . . .
Vv11g0724 Tc09_g014470 . . . . .
. . . . . . . .
. . . . . .
Vv11g0725 . . . . . . .
. . . . . . . .
. . . . .
Vv11g0726 Tc09_g026970 . . . . .
. . . . . . . .
. . . . . .
Vv11g0727 . . . . . . .
. . . . . . . .
. . . . .
Vv11g0728 . . . . . . .
. . . . . . . .
. . . . .
Vv11g0729 . . . . . . .
. . . . . . . .
. . . . .
Vv11g0730 . . . . . . .
. . . . . . . .
. . . . .
Vv11g0731 . . . . . . .
. . . . . . . .
. . . . .
Vv11g0732 Tc09_g026960 . . . . .
. . . . . . . .
. . . . . .
Vv11g0733 . . . . . . .
. . . . . . . .
. . . . .
Vv11g0734 Tc09_g014430 . Gorai.004G106800.1 .
. . . . . . . .
. . . . . . . .
Vv11g0735 Tc09_g014410 . . . Gorai.
009G008900.1 . . . . . . .
. . . . . . . .
Vv11g0736 Tc09_g014400 . . . . .
. . . . . . . .
. . . . . .
Vv11g0737 Tc09_g014390 . . . . .
. . . . . . . .
. . . . . .
Vv11g0738 Tc09_g014380 . Gorai.004G106400.1 .
. . . . . . . .
. . . . . . . .

Vv11g0739 . . . . . . .
. . . . . . .
. . . . .
Vv11g0740 . . . . . . .
. . . . . . .
. . . . .
Vv11g0741 Tc09_g014350 . . . Gorai.
009G008800.1 . . . . . . .
. . . . . . .
Vv11g0742 . . . . . . .
. . . . . . .
. . . . .
Vv11g0743 Tc09_g014340 Gorai.013G240900.1 . .
. . . . . . .
. . . . . . .
Vv11g0744 . . . . . . .
. . . . . . .
. . . . .
Vv11g0745 Tc09_g014330 . Gorai.004G106300.1 .
. . . . . . .
. . . . . . .
Vv11g0746 . . . . . . .
. . . . . . .
. . . . .
Vv11g0747 Tc09_g014300 . . . Gorai.
009G008600.1 . . . . . . .
. . . . . . .
Vv11g0748 . . . . . . .
. . . . . . .
. . . . .
Vv11g0749 . . . . . . .
. . . . . . .
. . . . .
Vv11g0750 Tc09_g014290 . . . Gorai.
009G008500.1 . . . . . . .
. . . . . . .
Vv11g0751 . . . . . . .
. . . . . . .
. . . . .
Vv11g0752 Tc09_g014280 . . . Gorai.
009G008400.1 . . . . . . .
. . . . . . .
Vv11g0753 . . . . . . .
. . . . . . .
. . . . .
Vv11g0754 . . . . . . .
. . . . . . .
. . . . .
Vv11g0755 . . Gorai.004G105600.1 . .
. . . . . . .

. . . . . . .
Vv11g0756 Tc09_g015170 . . . . .
. . . . . . . .
. . . . . .
Vv11g0757 . . . . . . .
. . . . . . . .
. . . . .
Vv11g0758 Tc09_g015180 . . . . .
. . . . . . . .
. . . . . .
Vv11g0759 Tc09_g015190 . Gorai.004G105500.1 .
. . . . . . . .
. . . . . . . .
Vv11g0760 . . . . . . .
. . . . . . . .
. . . . .
Vv11g0761 Tc09_g015200 . . . Gorai.
009G008200.1 . . . . . . .
. . . . . . . .
Vv11g0762 Tc09_g015210 . . . . .
. . . . . . . .
. . . . . .
Vv11g0763 . . . . . . .
. . . . . . . .
. . . . .
Vv11g0764 . . . . . . .
. . . . . . . .
. . . . .
Vv11g0765 . . . . . . .
. . . . . . . .
. . . . .
Vv11g0766 Tc09_g015300 . Gorai.004G104700.1 .
. . . . . . . .
. . . . . . . .
Vv11g0767 . . . . . . .
. . . . . . . .
. . . . .
Vv11g0768 Tc09_g015310 . Gorai.004G104300.1
Gorai.004G273300.1 Gorai.009G008000.1 . . .
. . . . . . . .
. . . .
Vv11g0769 . . . . . . .
. . . . . . . .
. . . . .
Vv11g0770 Tc09_g015390 . . . . .
. . . . . . . .
. . . . . .
Vv11g0771 . . . . . . .
. . . . . . . .
. . . . .

Vv11g0772 . . . . . . .
. . . . . . . .
. . . . .
Vv11g0773 . . . . . . .
. . . . . . . .
. . . . .
Vv11g0774 . . . . . . .
. . . . . . . .
. . . . .
Vv11g0775 . . . . . . .
. . . . . . . .
. . . . .
Vv11g0776 . . . . . . .
. . . . . . . .
. . . . .
Vv11g0777 . . . . . . .
. . . . . . . .
. . . . .
Vv11g0778 . . Gorai.004G104100.1 Gorai.
004G273200.1 . . . . . . .
. . . . . . . .
.
Vv11g0779 . . . . . . .
. . . . . . . .
. . . . .
Vv11g0780 . . . . Gorai.009G005000.1
. . . . . . . .
. . . . . . .
Vv11g0781 . . . . . . .
. . . . . . . .
. . . . .
Vv11g0782 . . . . . . .
. . . . . . . .
. . . . .
Vv11g0783 Tc09_g015900 . Gorai.004G104000.1
Gorai.004G273100.1 . . . . . .
. . . . . . . .
. .
Vv11g0784 . . . . . . .
. . . . . . . .
. . . . .
Vv11g0785 Tc09_g015970 . Gorai.004G103900.1 .
. . . . . . . .
. . . . . . . .
Vv11g0786 Tc09_g015980 . . . . .
. . . . . . . .
. . . . . .
Vv11g0787 . . . . . . .
. . . . . . . .
. . . . .

Vv11g0788 . . . . . . .
. . . . . . . .
. . . . .
Vv11g0789 . . . . . . .
. . . . . . . .
. . . . .
Vv11g0790 . . . . . . .
. . . . . . . .
. . . . .
Vv11g0791 . . Gorai.004G103800.1 . .
. . . . . . . .
. . . . . . .
Vv11g0792 . . . . . . .
. . . . . . . .
. . . . .
Vv11g0793 Tc09_g007440 . . . . .
. . . . . . . .
. . . . . .
Vv11g0794 . . . . . . .
. . . . . . . .
. . . . .
Vv11g0795 . . . . . . .
. . . . . . . .
. . . . .
Vv11g0796 Tc09_g007330 . . . . .
. . . . . . . .
. . . . . .
Vv11g0797 . . . . . . .
. . . . . . . .
. . . . .
Vv11g0798 . . . . . . .
. . . . . . . .
. . . . .
Vv11g0799 . . . . . . .
. . . . . . . .
. . . . .
Vv11g0800 Tc09_g007040 . . . . .
. . . . . . . .
. . . . . .
Vv11g0801 . . . . . . .
. . . . . . . .
. . . . .
Vv11g0802 . . . . . . .
. . . . . . . .
. . . . .
Vv11g0803 . . . . . . .
. . . . . . . .
. . . . .
Vv11g0804 . . . . . . .
. . . . . . . .

. . . . .
Vv11g0805 . . . . . . .
. . . . . . . .
. . . . .
Vv11g0806 . . . . . . .
. . . . . . . .
. . . . .
Vv11g0807 . . . . . . .
. . . . . . . .
. . . . .
Vv11g0808 Tc09_g016000 . Gorai.004G103200.1
Gorai.004G273000.1 . . . . . .
. . . . . . . .
. .
Vv11g0809 . . . . . . .
. . . . . . . .
. . . . .
Vv11g0810 . . . . . . .
. . . . . . . .
. . . . .
Vv11g0811 Tc09_g016020 . Gorai.004G103100.1 .
. . . . . . . .
. . . . . . . .
Vv11g0812 . . . . . . .
. . . . . . . .
. . . . .
Vv11g0813 . . . . . . .
. . . . . . . .
. . . . .
Vv11g0814 Tc09_g021880 . Gorai.004G100200.1 .
. . . . . . . .
. . . . . . . .
Vv11g0815 Tc09_g020720 . . Gorai.004G020800.1
. . . . . . . .
. . . . . . . .
Vv11g0816 . . . . . . .
. . . . . . . .
. . . . .
Vv11g0817 . . . . . . .
. . . . . . . .
. . . . .
Vv11g0818 . . . . . . .
. . . . . . . .
. . . . .
Vv11g0819 . . . . . . .
. . . . . . . .
. . . . .
Vv11g0820 . . . . . . .
. . . . . . . .
. . . . .

Vv11g0821 . . . . . . .
. . . . . . . .
. . . . .
Vv11g0822 . . . . . . .
. . . . . . . .
. . . . .
Vv11g0823 . . . . . . .
. . . . . . . .
. . . . .
Vv11g0824 . . . . . . .
. . . . . . . .
. . . . .
Vv11g0825 . . . . . . .
. . . . . . . .
. . . . .
Vv11g0826 . . . . . . .
. . . . . . . .
. . . . .
Vv11g0827 . . . . . . .
. . . . . . . .
. . . . .
Vv11g0828 . . . . . . .
. . . . . . . .
. . . . .
Vv11g0829 . . . . . . .
. . . . . . . .
. . . . .
Vv11g0830 . . . . . . .
. . . . . . . .
. . . . .
Vv11g0831 . . . . . . .
. . . . . . . .
. . . . .
Vv11g0832 . . . . . . .
. . . . . . . .
. . . . .
Vv11g0833 . . . . . . .
. . . . . . . .
. . . . .
Vv11g0834 . . . . . . .
. . . . . . . .
. . . . .
Vv11g0835 . . . . . . .
. . . . . . . .
. . . . .
Vv11g0836 Tc09_g020730 . . Gorai.004G020600.1
. . . . . . . .
. . . . . . . .
Vv11g0837 . . . . . . .
. . . . . . . .

```
         .       .        .       .        .
Vv11g0838        .        .       .        .       .        .       .
         .       .        .       .        .       .        .       .
         .       .        .       .        .
Vv11g0839        .        .       .        .       .        .       .
         .       .        .       .        .       .        .       .
         .       .        .       .        .
Vv11g0840        .        .       .        .       .        .       .
         .       .        .       .        .       .        .       .
         .       .        .       .        .
Vv11g0841        .        .       .        .       .        .       .
         .       .        .       .        .       .        .       .
         .       .        .       .        .
Vv11g0842        Tc09_g020790     .        .       .        .       .
         .       .        .       .        .       .        .       .
         .       .        .       .        .       .
Vv11g0843        .        .       .        .       .        .       .
         .       .        .       .        .       .        .       .
         .       .        .       .        .
Vv11g0844        .        .       .        .       .        .       .
         .       .        .       .        .       .        .       .
         .       .        .       .        .
Vv11g0845        .        .       .        .       .        .       .
         .       .        .       .        .       .        .       .
         .       .        .       .        .
Vv11g0846        .        .       .        Gorai.004G272800.1       .
         .       Vv17g0798        .        Gorai.001G077600.1       .
         .       .        .       .        .       .        .       .
         .       .
Vv11g0847        .        .       .        .       .        .       .
         .       .        .       .        .       .        .       .
         .       .        .       .        .
Vv11g0848        .        .       .        .       .        .       .
         .       .        .       .        .       .        .       .
         .       .        .       .        .
Vv11g0849        Tc09_g000280     .        .       .        .       .
         .       .        .       .        .       .        .       .
         .       .        .       .        .       .
Vv11g0850        Tc09_g021860     .        .       .        .       .
         .       .        .       .        .       .        .       .
         .       .        .       .        .       .
Vv11g0851        Tc09_g000260     .        .       .        .       .
         .       .        .       .        .       .        .       .
         .       .        .       .        .       .
Vv11g0852        .        .       .        .       .        .       .
         .       .        .       .        .       .        .       .
         .       .        .       .        .
Vv11g0853        Tc09_g021760     .        .       .        .       .
         Vv17g0804        Tc03_g011980     .       .        .
Gorai.008G198400.1        .       .        .       .        .       .
```

| | | | | | | | |
|---|---|---|---|---|---|---|---|
| . | . | | | | | | |
| Vv11g0854 | . | . | . | . | . | . | . |
| . | . | . | . | . | . | . | . |
| . | . | . | . | . | | | |
| Vv11g0855 | . | . | . | . | . | . | . |
| . | . | . | . | . | . | . | . |
| . | . | . | . | . | | | |
| Vv11g0856 | . | . | . | Gorai.004G270800.1 | | | . |
| . | . | . | . | . | . | . | . |
| . | . | . | . | . | . | . | |
| Vv11g0857 | . | . | . | . | . | . | . |
| . | . | . | . | . | . | . | . |
| . | . | . | . | . | | | |
| Vv11g0858 | . | . | . | . | . | . | . |
| . | . | . | . | . | . | . | . |
| . | . | . | . | . | | | |
| Vv11g0859 | Tc09_g003550 | | . | . | . | . | . |
| . | . | . | . | . | . | . | . |
| . | . | . | . | . | . | | |
| Vv11g0860 | Tc09_g000030 | | . | . | . | . | . |
| . | . | . | . | . | . | . | . |
| . | . | . | . | . | . | | |
| Vv11g0861 | . | . | . | . | . | . | . |
| . | . | . | . | . | . | . | . |
| . | . | . | . | . | | | |
| Vv11g0862 | . | . | . | . | . | . | . |
| . | . | . | . | . | . | . | . |
| . | . | . | . | . | | | |
| Vv11g0863 | . | . | . | . | . | . | . |
| . | . | . | . | . | . | . | . |
| . | . | . | . | . | | | |
| Vv11g0864 | . | . | . | . | . | . | . |
| . | . | . | . | . | . | . | . |
| . | . | . | . | . | | | |
| Vv11g0865 | . | . | . | Gorai.004G022300.1 | | | . |
| . | Vv17g0832 | | Tc03_g000240 | | . | . | . |
| . | . | . | . | . | . | . | . |
| . | | | | | | | |
| Vv11g0866 | Tc09_g003530 | | . | . | . | . | . |
| . | . | . | . | . | . | . | . |
| . | . | . | . | . | . | | |
| Vv11g0867 | . | . | . | . | . | . | . |
| . | . | . | . | . | . | . | . |
| . | . | . | . | . | | | |
| Vv11g0868 | . | . | . | . | . | . | . |
| . | . | . | . | . | . | . | . |
| . | . | . | . | . | | | |
| Vv11g0869 | . | . | . | . | . | . | . |
| . | . | . | . | . | . | . | . |
| . | . | . | . | . | | | |

Vv11g0870 . . . . . . .
. . . . . . . .
. . . . .
Vv11g0871 . . . . . . .
. . . . . . . .
. . . . .
Vv11g0872 . . . . . . .
. . . . . . . .
. . . . .
Vv11g0873 . . . . . . .
. . . . . . . .
. . . . .
Vv11g0874 . . . . . . .
. . . . . . . .
. . . . .
Vv11g0875 . . . . . . .
. . . . . . . .
. . . . .
Vv11g0876 . . . . . . .
. . . . . . . .
. . . . .
Vv11g0877 . . . . . . .
. . . . . . . .
. . . . .
Vv11g0878 . . . . . . .
. . . . . . . .
. . . . .
Vv11g0879 . . . . . . .
. . . . . . . .
. . . . .
Vv11g0880 . . . . . . .
. . . . . . . .
. . . . .
Vv11g0881 . . . . . . .
. . . . . . . .
. . . . .
Vv11g0882 . . . . . . .
. . . . . . . .
. . . . .
Vv11g0883 . . . . . . .
. . . . . . . .
. . . . .
Vv11g0884 . . . . . . .
. . . . . . . .
. . . . .
Vv11g0885 . . . . . . .
. . . . . . . .
. . . . .
Vv11g0886 . Gorai.013G241900.1 . Gorai.
004G022500.1 . . . . . . .

| | | | | | | | |
|---|---|---|---|---|---|---|---|
| . | . | . | . | . | . | . | . |
| . | | | | | | | |
| Vv11g0887 | . | . | . | . | . | . | . |
| . | . | . | . | . | . | . | . |
| . | . | . | . | . | | | |
| Vv11g0888 | . | . | . | . | . | . | |
| Vv17g0849 | . | . | . | . | . | . | . |
| . | . | . | . | . | . | | |
| Vv11g0889 | . | . | . | . | . | . | . |
| . | . | . | . | . | . | . | . |
| . | . | . | . | . | | | |
| Vv11g0890 | . | . | . | . | . | . | . |
| . | . | . | . | . | . | . | . |
| . | . | . | . | . | | | |
| Vv11g0891 | . | . | . | . | . | . | . |
| . | . | . | . | . | . | . | . |
| . | . | . | . | . | | | |
| Vv11g0892 | . | . | . | . | . | . | . |
| . | . | . | . | . | . | . | . |
| . | . | . | . | . | | | |
| Vv11g0893 | . | . | . | . | . | . | . |
| . | . | . | . | . | . | . | . |
| . | . | . | . | . | | | |
| Vv11g0894 | . | . | . | . | . | . | . |
| . | . | . | . | . | . | . | . |
| . | . | . | . | . | | | |
| Vv11g0895 | . | . | . | . | . | . | . |
| . | . | . | . | . | . | . | . |
| . | . | . | . | . | | | |
| Vv11g0896 | . | . | . | . | . | . | . |
| . | . | . | . | . | . | . | . |
| . | . | . | . | . | | | |
| Vv11g0897 | . | . | . | . | . | . | . |
| . | . | . | . | . | . | . | . |
| . | . | . | . | . | | | |
| Vv11g0898 | Tc09_g003340 | | . | . | . | . | . |
| . | . | . | . | . | . | . | . |
| . | . | . | . | . | . | | |
| Vv11g0899 | . | . | . | . | . | . | . |
| . | . | . | . | . | . | . | . |
| . | . | . | . | . | | | |
| Vv11g0900 | . | . | . | . | . | . | . |
| . | . | . | . | . | . | . | . |
| . | . | . | . | . | | | |
| Vv11g0901 | Tc09_g003230 | | . | . | Gorai.004G024800.1 | | |
| . | . | . | . | . | . | . | . |
| . | . | . | . | . | . | . | . |
| Vv11g0902 | . | . | . | . | . | . | . |
| . | . | . | . | . | . | . | . |
| . | . | . | . | . | | | |

Vv11g0903 . . . . . . .
. . . . . . . .
. . . . .
Vv11g0904 . . . . . . .
. . . . . . . .
. . . . .
Vv11g0905 . . . . . . .
. . . . . . . .
. . . . .
Vv11g0906 . . . . . . .
. . . . . . . .
. . . . .
Vv11g0907 . . . . . . .
. . . . . . . .
. . . . .
Vv11g0908 . . . . . . .
. . . . . . . .
. . . . .
Vv11g0909 . . . . . . .
. . . . . . . .
. . . . .
Vv11g0910 . . . . . . .
. . . . . . . .
. . . . .
Vv11g0911 . . . . . . .
. . . . . . . .
. . . . .
Vv11g0912 . . . . . . .
. . . . . . . .
. . . . .
Vv11g0913 . . . . . . .
. . . . . . . .
. . . . .
Vv11g0914 . . . . . . .
. . . . . . . .
. . . . .
Vv11g0915 Tc09_g025210 . . . . .
. . . . . . . .
. . . . . .
Vv11g0916 . . . . . . .
. . . . . . . .
. . . . .
Vv11g0917 . . . . . . .
. . . . . . . .
. . . . .
Vv11g0918 Tc09_g018340 Gorai.013G242600.1 . .
. . . . . . . .
. . . . . . . .
Vv11g0919 Tc09_g018280 Gorai.013G242700.1 . .
. . . . . . . .

```
                    .        .        .        .        .        .        .        .
Vv11g0920                    .        .        .        .        .        .        .
                    .        .        .        .        .        .        .        .
                    .        .        .        .        .
Vv11g0921        Tc09_g018210         .        .        .        .        .
         Vv17g0861        Tc03_g000540         .        .        .        .
         Gorai.006G187900.1           .        .        .        .        .
                    .        .
Vv11g0922                    .        .        .        .        .        .        .
                    .        .        .        .        .        .        .        .
                    .        .        .        .        .
Vv11g0923                    .        .        .        .        .        .        .
                    .        .        .        .        .        .        .        .
                    .        .        .        .        .
Vv11g0924                    .        .        .        .        .        .        .
                    .        .        .        .        .        .        .        .
                    .        .        .        .        .
Vv11g0925        Tc09_g018190         .        .        .        .        .
                    .        .        .        .        .        .        .        .
                    .        .        .        .        .        .
Vv11g0926                    .        .        .        .        .        .        .
                    .        .        .        .        .        .        .        .
                    .        .        .        .        .
Vv11g0927        Tc09_g002900     Gorai.013G242800.1         .        .
         Gorai.009G002200.1           .        .        .        .        .
                    .        .        .        .        .        .        .        .
                    .        .
Vv11g0928                    .        .        .        .        .        .        .
                    .        .        .        .        .        .        .        .
                    .        .        .        .        .
Vv11g0929                    .    Gorai.013G243000.1         .        .        .
                    .        .        .        .        .        .        .        .
                    .        .        .        .        .        .        .
Vv11g0930                    .    Gorai.013G243100.1         .        .        .
                    .        .        .        .        .        .        .        .
                    .        .        .        .        .        .        .
Vv11g0931                    .        .        .        .        .        .        .
                    .        .        .        .        .        .        .        .
                    .        .        .        .        .
Vv11g0932                    .        .        .        .        .        .        .
                    .        .        .        .        .        .        .        .
                    .        .        .        .        .
Vv11g0933                    .        .        .    Gorai.004G025000.1
Gorai.009G002300.1            .        .        .        .        .        .
                    .        .        .        .        .        .        .        .
                    .
Vv11g0934                    .        .        .        .        .        .        .
                    .        .        .        .        .        .        .        .
                    .        .        .        .        .
Vv11g0935                    .        .        .        .        .        .        .
```

| | | | | | | | |
|---|---|---|---|---|---|---|---|
| . | . | . | . | . | . | . | . |
| . | . | . | . | . | | | |
| Vv11g0936 | . | . | . | . | . | . | . |
| . | . | . | . | . | . | . | . |
| . | . | . | . | . | | | |
| Vv11g0937 | . | . | . | . | . | . | . |
| . | . | . | . | . | . | . | . |
| . | . | . | . | . | | | |
| Vv11g0938 | Tc09_g004330 | | . | . | . | . | . |
| . | . | . | . | . | . | . | . |
| . | . | . | . | . | . | | |
| Vv11g0939 | Tc09_g002700 | | . | . | . | . | . |
| . | . | . | . | . | . | . | . |
| . | . | . | . | . | . | | |
| Vv11g0940 | . | . | . | . | . | . | . |
| . | . | . | . | . | . | . | . |
| . | . | . | . | . | | | |
| Vv11g0941 | . | . | . | . | . | . | . |
| . | . | . | . | . | . | . | . |
| . | . | . | . | . | | | |
| Vv11g0942 | . | . | . | . | . | . | . |
| . | . | . | . | . | . | . | . |
| . | . | . | . | . | | | |
| Vv11g0943 | . | . | . | . | . | . | . |
| . | . | . | . | . | . | . | . |
| . | . | . | . | . | | | |
| Vv11g0944 | . | . | . | . | . | . | . |
| . | . | . | . | . | . | . | . |
| . | . | . | . | . | | | |
| Vv11g0945 | . | . | . | . | . | . | . |
| . | . | . | . | . | . | . | . |
| . | . | . | . | . | | | |
| Vv11g0946 | . | . | . | . | . | . | . |
| . | . | . | . | . | . | . | . |
| . | . | . | . | . | | | |
| Vv11g0947 | . | . | . | . | . | . | . |
| . | . | . | . | . | . | . | . |
| . | . | . | . | . | | | |
| Vv11g0948 | . | . | . | . | . | . | . |
| . | . | . | . | . | . | . | . |
| . | . | . | . | . | | | |
| Vv11g0949 | Tc09_g002430 | | . | . | . | . | . |
| . | . | . | . | . | . | . | . |
| . | . | . | . | . | . | | |
| Vv11g0950 | . | . | . | . | . | . | . |
| . | . | . | . | . | . | . | . |
| . | . | . | . | . | | | |
| Vv11g0951 | . | . | . | . | . | . | . |
| . | . | . | . | . | . | . | . |
| . | . | . | . | . | | | |

| | | | | | | | |
|---|---|---|---|---|---|---|---|
| Vv11g0952 | . | . | . | . | . | . | . |
| . | . | . | . | . | . | . | . |
| . | . | . | . | . | | | |
| Vv11g0953 | Tc09_g024850 | | . | . | . | . | . |
| . | . | . | . | . | . | . | . |
| . | . | . | . | . | . | | |
| Vv11g0954 | . | . | . | . | . | . | . |
| . | . | . | . | . | . | . | . |
| . | . | . | . | . | | | |
| Vv11g0955 | . | . | . | . | . | . | . |
| . | . | . | . | . | . | . | . |
| . | . | . | . | . | | | |
| Vv11g0956 | . | . | . | . | . | . | . |
| . | . | . | . | . | . | . | . |
| . | . | . | . | . | | | |
| Vv11g0957 | . | . | . | . | . | . | . |
| . | . | . | . | . | . | . | . |
| . | . | . | . | . | | | |
| Vv11g0958 | . | . | . | . | . | . | . |
| . | . | . | . | . | . | . | . |
| . | . | . | . | . | | | |
| Vv11g0959 | Tc09_g003930 | | . | . | . | . | . |
| . | . | . | . | . | . | . | . |
| . | . | . | . | . | . | | |
| Vv11g0960 | Tc09_g018120 | | . | . | . | . | . |
| . | . | . | . | . | . | . | . |
| . | . | . | . | . | . | | |
| Vv11g0961 | . | . | . | . | . | . | |
| Vv17g0877 | Tc03_g000690 | | . | . | . | . | . |
| . | . | . | . | . | . | . | |
| Vv11g0962 | . | . | . | . | . | . | . |
| . | . | . | . | . | . | . | . |
| . | . | . | . | . | | | |
| Vv11g0963 | . | . | . | . | . | . | . |
| . | . | . | . | . | . | . | . |
| . | . | . | . | . | | | |
| Vv11g0964 | . | . | . | . | . | . | . |
| . | . | . | . | . | . | . | . |
| . | . | . | . | . | | | |
| Vv11g0965 | . | . | . | . | . | . | . |
| . | . | . | . | . | . | . | . |
| . | . | . | . | . | | | |
| Vv11g0966 | Tc09_g017950 | | . | . | . | . | . |
| . | . | . | . | . | . | . | . |
| . | . | . | . | . | . | | |
| Vv11g0967 | Tc09_g017840 | | . | . | . | . | . |
| . | . | . | . | . | . | . | . |
| . | . | . | . | . | . | | |
| Vv11g0968 | . | . | . | . | . | . | . |
| . | . | . | . | . | . | . | . |

. . . . .
Vv11g0969 Tc09_g017780 . . . . .
. . . . . . . .
. . . . . .
Vv11g0970 . . . . . . .
. . . . . . . .
. . . . .
Vv11g0971 Tc09_g017760 . . . . .
Vv17g0882 Tc03_g000730 . . . .
. . . . . . . .
Vv11g0972 Tc09_g017700 . . . . .
. . . . . . . .
. . . . . .
Vv11g0973 Tc09_g017500 . . . . .
. . . . . . . .
. . . . . .
Vv11g0974 Tc09_g017490 . . . Gorai.
009G003700.1 . . . . . . .
. . . . . . . .
Vv11g0975 . . . Gorai.004G026700.1 .
. . . . . . . .
. . . . . . .
Vv11g0976 . . . Gorai.004G027200.1
Gorai.009G004000.1 . . . . . .
. . . . . . . .
.
Vv11g0977 . . . . . . .
. . . . . . . .
. . . . .
Vv11g0978 . . . . . . .
. . . . . . . .
. . . . .
Vv11g0979 . . . . . . .
. . . . . . . .
. . . . .
Vv11g0980 . . . . . . .
. . . . . . . .
. . . . .
Vv11g0981 . . . Gorai.004G027400.1
Gorai.009G004400.1 . . . . . .
. . . . . . . .
.
Vv11g0982 . . . . . . .
. . . . . . . .
. . . . .
Vv11g0983 . . . . . . .
. . . . . . . .
. . . . .
Vv11g0984 . . . . . . .
. . . . . . . .

. . . . .
Vv11g0985 . . . . . . .
. . . . . . . .
. . . . .
Vv11g0986 . . . . . . .
. . . . . . . .
. . . . .
Vv11g0987 . . . . . . .
. . . . . . . .
. . . . .
Vv11g0988 . . . . . . .
. . . . . . . .
. . . . .
Vv11g0989 . . . . . . .
. . . . . . . .
. . . . .
Vv11g0990 . . . Gorai.004G027600.1
Gorai.009G004900.1 . . . . . .
. . . . . . . .
.
Vv11g0991 Tc09_g017450 . Gorai.004G099100.1 .
. . . . . . . .
. . . . . . . .
Vv11g0992 Tc09_g017430 . . . Gorai.
009G005000.1 . . . . . . .
. . . . . . . .
Vv11g0993 . . . . Gorai.009G005400.1
. . . . . . . .
. . . . . . .
Vv11g0994 . . . . . . .
. . . . . . . .
. . . . .
Vv11g0995 Tc09_g017350 . . . Gorai.
009G005600.1 . . . . . . .
. . . . . . . .
Vv11g0996 . . . . . . .
. . . . . . . .
. . . . .
Vv11g0997 . . . . . . .
. . . . . . . .
. . . . .
Vv11g0998 . . Gorai.004G099500.1 . .
. . . . . . . .
. . . . . . .
Vv11g0999 . . . . . . .
. . . . . . . .
. . . . .
Vv11g1000 . . Gorai.004G099900.1 . .
. . . . . . . .
. . . . . . .

Vv11g1001 Tc09_g017120 . Gorai.004G100200.1 .
. . . . . . . .
. . . . . . . .
Vv11g1002 . . . . . . .
. . . . . . . .
. . . . .
Vv11g1003 Tc09_g017090 . . . . .
. . . . . . . .
. . . . . .
Vv11g1004 . . . . . . .
. . . . . . . .
. . . . .
Vv11g1005 . . Gorai.004G100300.1 . .
. . . . . . . .
. . . . . . .
Vv11g1006 . . . . . . .
. . . . . . . .
. . . . .
Vv11g1007 . . . . . . .
. . . . . . . .
. . . . .
Vv11g1008 . . . . . . .
. . . . . . . .
. . . . .
Vv11g1009 . . . . . . .
. . . . . . . .
. . . . .
Vv11g1010 . . . . . . .
. . . . . . . .
. . . . .
Vv11g1011 . . Gorai.004G100400.1 . .
. . . . . . . .
. . . . . . .
Vv11g1012 . . . . . . .
. . . . . . . .
. . . . .
Vv11g1013 . . . . . . .
. . . . . . . .
. . . . .
Vv11g1014 . . . . . . .
. . . . . . . .
. . . . .
Vv11g1015 . . . . . . .
. . . . . . . .
. . . . .
Vv11g1016 . . Gorai.004G100500.1 . .
. . . . . . . .
. . . . . . .
Vv11g1017 . . . . . . .
. . . . . . . .

. . . . .
Vv11g1018 . . . . Gorai.009G005900.1
. . . . . . . .
. . . . . . .
Vv11g1019 . . . . . . .
. . . . . . . .
. . . . .
Vv11g1020 . . . . . . .
. . . . . . . .
. . . . .
Vv11g1021 . . Gorai.004G100800.1 . .
. . . . . . . .
. . . . . . .
Vv11g1022 . . . . . . .
. . . . . . . .
. . . . .
Vv11g1023 . . . . Gorai.009G006000.1
. . . . . . . .
. . . . . . .
Vv11g1024 . . . . . . .
. . . . . . . .
. . . . .
Vv11g1025 . . . . Gorai.009G006100.1
. . . . . . . .
. . . . . . .
Vv11g1026 . . . . Gorai.009G006200.1
. . . . . . . .
. . . . . . .
Vv11g1027 Tc09_g016990 . Gorai.004G100900.1 .
. . . . . . . .
. . . . . . . .
Vv11g1028 Tc09_g016970 . . . Gorai.
009G006300.1 . . . . . . .
. . . . . . . .
Vv11g1029 Tc09_g016960 . . . . .
. . . . . . . .
. . . . . .
Vv11g1030 . . . . . . .
. . . . . . . .
. . . . .
Vv11g1031 Tc09_g016950 . . . . .
. . . . . . . .
. . . . . .
Vv11g1032 Tc09_g016940 . . . Gorai.
009G006400.1 . . . . . . .
. . . . . . . .
Vv11g1033 Tc09_g016930 . . . . .
. . . . . . . .
. . . . . .
Vv11g1034 . . . . . . .

| | | | | | | | |
|---|---|---|---|---|---|---|---|
| . | . | . | . | . | . | . | . |
| . | . | . | . | . | | | |
| Vv11g1035 | . | . | . | . | . | . | . |
| . | . | . | . | . | . | . | . |
| . | . | . | . | . | | | |
| Vv11g1036 | . | . | . | . | . | . | . |
| . | . | . | . | . | . | . | . |
| . | . | . | . | . | | | |
| Vv11g1037 | . | . | . | . | . | . | . |
| . | . | . | . | . | . | . | . |
| . | . | . | . | . | | | |
| Vv11g1038 | . | . | . | Gorai.004G030400.1 | | | . |
| . | . | . | . | . | . | . | . |
| . | . | . | . | . | . | . | |
| Vv11g1039 | . | . | . | . | . | . | . |
| . | . | . | . | . | . | . | . |
| . | . | . | . | . | | | |
| Vv11g1040 | . | . | . | . | . | . | . |
| . | . | . | . | . | . | . | . |
| . | . | . | . | . | | | |
| Vv11g1041 | . | . | . | Gorai.004G030500.1 | | | . |
| . | . | . | . | . | . | . | . |
| . | . | . | . | . | . | . | |
| Vv11g1042 | . | . | . | . | . | . | . |
| . | . | . | . | . | . | . | . |
| . | . | . | . | . | | | |
| Vv11g1043 | . | . | . | Gorai.004G030600.1 | | | . |
| . | . | . | . | . | . | . | . |
| . | . | . | . | . | . | . | |
| Vv11g1044 | . | . | . | . | . | . | . |
| . | . | . | . | . | . | . | . |
| . | . | . | . | . | | | |
| Vv11g1045 | . | . | . | . | . | . | . |
| . | . | . | . | . | . | . | . |
| . | . | . | . | . | | | |
| Vv11g1046 | . | . | . | Gorai.004G030700.1 | | | . |
| . | . | . | . | . | . | . | . |
| . | . | . | . | . | . | . | |
| Vv11g1047 | . | . | . | . | . | . | . |
| . | . | . | . | . | . | . | . |
| . | . | . | . | . | | | |
| Vv11g1048 | Tc09_g016910 | | . | . | . | . | . |
| . | . | . | . | . | . | . | . |
| . | . | . | . | . | . | | |
| Vv11g1049 | . | . | . | . | . | . | . |
| . | . | . | . | . | . | . | . |
| . | . | . | . | . | | | |
| Vv11g1050 | . | . | . | . | . | . | . |
| . | . | . | . | . | . | . | . |
| . | . | . | . | . | | | |

Vv11g1051 . . . . . . .
. . . . . . . .
. . . . .
Vv11g1052 . . . . . . .
. . . . . . . .
. . . . .
Vv11g1053 . . . . . . .
. . . . . . . .
. . . . .
Vv11g1054 . . . . . . .
. . . . . . . .
. . . . .
Vv11g1055 Tc09_g016880 Gorai.013G246400.1 . .
. . . . . . . .
. . . . . . . .
Vv11g1056 Tc09_g016870 Gorai.013G246300.1 . .
. . . . . . . .
. . . . . . . .
Vv11g1057 Tc09_g016790 . . . Gorai.
009G006500.1 . . . . . . .
. . . . . . . .
Vv11g1058 . . . . . . .
. . . . . . . .
. . . . .
Vv11g1059 . . . . . . .
. . . . . . . .
. . . . .
Vv11g1060 . . . . . . .
. . . . . . . .
. . . . .
Vv11g1061 . . . . . . .
. . . . . . . .
. . . . .
Vv11g1062 . . . . . . .
. . . . . . . .
. . . . .
Vv11g1063 . . . . . . .
. . . . . . . .
. . . . .
Vv11g1064 . . . . . . .
. . . . . . . .
. . . . .
Vv11g1065 Tc09_g016780 . Gorai.004G101000.1 .
Gorai.009G006600.1 . . . . .
. . . . . . . .
. .
Vv11g1066 . . . . . . .
. . . . . . . .
. . . . .
Vv11g1067 . . . . . . .

. . . . . . . .
. . . . .
Vv11g1068 Tc09_g016760 Gorai.013G246200.1 . .
Gorai.009G006700.1 . . . . .
. . . . . . . .
. .
Vv11g1069 . . . . . .
Vv08g0076 . . . . . . .
. . . . . .
Vv11g1070 . . . . . . .
. . . . . . . .
. . . . .
Vv11g1071 Tc09_g016710 . . . . .
Vv08g0084 Tc05_g023190 . . . .
. . . . . . . .
Vv11g1072 . . . . . .
Vv08g0086 Tc05_g023200 . . . . .
. . . . . . .
Vv11g1073 Tc09_g016680 . . . . .
Vv08g0088 Tc05_g023210 . . . .
. . . . . . . .
Vv11g1074 Tc09_g016670 . . . . .
Vv08g0095 . . . . . .
. . . . . . .
Vv11g1075 . . . . . .
Vv08g0098 . . . . . . .
. . . . . .
Vv11g1076 Tc09_g016660 Gorai.013G246100.1 . .
. . Vv08g0103 . . . .
. . . . . . . .
.
Vv11g1077 . . . . . .
Vv08g0104 . . . . . . .
. . . . . .
Vv11g1078 Tc09_g016650 . Gorai.004G101100.1 .
. . . . . . . .
. . . . . . . .
Vv11g1079 . . . . . . .
. . . . . . . .
. . . . .
Vv11g1080 . . . . . . .
. . . . . . . .
. . . . .
Vv11g1081 . . . . . . .
. . . . . . . .
. . . . .
Vv11g1082 Tc09_g016580 . Gorai.004G101300.1 .
. . . . . . . .
. . . . . . . .
Vv11g1083 . . . . . . .

. . . . . . . .
. . . . .
Vv11g1084 . . . . . . .
. . . . . . . .
. . . . .
Vv11g1085 . . . . . . .
. . . . . . . .
. . . . .
Vv11g1086 Tc09_g016550 . Gorai.004G101500.1 .
. . . . . . . .
. . . . . . . .
Vv11g1087 . . . . . . .
. . . . . . . .
. . . . .
Vv11g1088 Tc09_g016540 . Gorai.004G101600.1 .
. . . . . . . .
. . . . . . . .
Vv11g1089 . . . . . . .
. . . . . . . .
. . . . .
Vv11g1090 . . . . . . .
. . . . . . . .
. . . . .
Vv11g1091 . . . . . . .
. . . . . . . .
. . . . .
Vv11g1092 Tc09_g016360 . Gorai.004G101800.1 .
Gorai.009G006900.1 . . . . .
. . . . . . . .
. .
Vv11g1093 . . . Gorai.004G030800.1 .
. . . . . . . .
. . . . . . .
Vv11g1094 . . . . . . .
. . . . . . . .
. . . . .
Vv11g1095 . . . . . . .
. . . . . . . .
. . . . .
Vv11g1096 . . . . . . .
. . . . . . . .
. . . . .
Vv11g1097 . . . Gorai.004G030900.1 .
. . . . . . . .
. . . . . . .
Vv11g1098 . . . . . . .
. . . . . . . .
. . . . .
Vv11g1099 Tc09_g016270 Gorai.013G246000.1 Gorai.
004G102000.1 . . . . . . .

```
        .       .       .       .       .       .       .       .
        .       .
Vv11g1100       Tc09_g016230    .       Gorai.004G102100.1      .
        .       .       .       .       .       .       .       .
        .       .       .       .       .       .       .       .
Vv11g1101       Tc09_g016200    .       Gorai.004G102300.1      .
        .       .       .       .       .       .       .       .
        .       .       .       .       .       .       .       .
Vv11g1102       .       .       .       .       .       .       .
        .       .       .       .       .       .       .       .
        .       .       .       .       .
Vv11g1103       .       .       .       .       .       .       .
        .       .       .       .       .       .       .       .
        .       .       .       .       .
Vv11g1104       .       .       Gorai.004G103700.1      .       .
        .       .       .       .       .       .       .       .
        .       .       .       .       .       .       .
Vv11g1105       .       .       .       .       .       .       .
        .       .       .       .       .       .       .       .
        .       .       .       .       .
Vv11g1106       .       Gorai.013G243100.1      .       .
Gorai.009G007300.1      .       .       .       .       .       .
        .       .       .       .       .       .       .       .
        .
Vv11g1107       .       .       .       .       .       .       .
        .       .       .       .       .       .       .       .
        .       .       .       .       .
Vv12g0001       .       .       .       .       .       .       .
        .       .       .       .       .       .       .       .
        .       .       .       .       .
Vv12g0002       Tc01_g040310    .       .       .       .       .
        .       .       .       .       .       .       .       .
        .       .       .       .       .       .
Vv12g0003       .       .       .       .       .       .       .
        .       .       .       .       .       .       .       .
        .       .       .       .       .
Vv12g0004       .       .       .       .       .       .       .
        .       .       .       .       .       .       .       .
        .       .       .       .       .
Vv12g0005       .       .       .       .       .       .       .
        .       .       .       .       .       .       .       .
        .       .       .       .       .
Vv12g0006       .       .       .       .       .       .       .
        .       .       .       .       .       .       .       .
        .       .       .       .       .
Vv12g0007       .       .       .       .       .       .       .
        .       .       .       .       .       .       .       .
        .       .       .       .       .
Vv12g0008       Tc01_g002220    .       Gorai.008G064700.1      .
        .       .       .       .       .       .       .       .
```

| | | | | | | | |
|---|---|---|---|---|---|---|---|
| . | . | . | . | . | . | . | . |
| Vv12g0009 | . | . | . | . | . | . | . |
| . | . | . | . | . | . | . | . |
| . | . | . | . | . | | | |
| Vv12g0010 | . | . | . | . | . | . | . |
| . | . | . | . | . | . | . | . |
| . | . | . | . | . | | | |
| Vv12g0011 | . | . | . | . | . | . | . |
| . | . | . | . | . | . | . | . |
| . | . | . | . | . | | | |
| Vv12g0012 | Tc01_g040190 | | . | . | . | . | . |
| . | . | . | . | . | . | . | . |
| . | . | . | . | . | . | | |
| Vv12g0013 | . | . | . | . | . | . | . |
| . | . | . | . | . | . | . | . |
| . | . | . | . | . | | | |
| Vv12g0014 | . | . | . | . | . | . | . |
| . | . | . | . | . | . | . | . |
| . | . | . | . | . | | | |
| Vv12g0015 | . | . | . | . | . | . | . |
| . | . | . | . | . | . | . | . |
| . | . | . | . | . | | | |
| Vv12g0016 | . | . | . | . | . | . | . |
| . | . | . | . | . | . | . | . |
| . | . | . | . | . | | | |
| Vv12g0017 | . | . | . | . | . | . | . |
| . | . | . | . | . | . | . | . |
| . | . | . | . | . | | | |
| Vv12g0018 | . | . | . | . | . | . | . |
| . | . | . | . | . | . | . | . |
| . | . | . | . | . | | | |
| Vv12g0019 | . | . | . | . | . | . | . |
| . | . | . | . | . | . | . | . |
| . | . | . | . | . | | | |
| Vv12g0020 | . | . | . | . | . | . | . |
| . | . | . | . | . | . | . | . |
| . | . | . | . | . | | | |
| Vv12g0021 | . | . | . | . | . | . | . |
| . | . | . | . | . | . | . | . |
| . | . | . | . | . | | | |
| Vv12g0022 | Tc01_g002250 | | Gorai.007G043400.1 | | | . | . |
| . | . | . | . | . | . | . | . |
| . | . | . | . | . | . | . | . |
| Vv12g0023 | . | . | . | . | . | . | . |
| . | . | . | . | . | . | . | . |
| . | . | . | . | . | | | |
| Vv12g0024 | . | . | . | . | . | . | . |
| . | . | . | . | . | . | . | . |
| . | . | . | . | . | | | |
| Vv12g0025 | . | . | . | . | . | . | . |

. . . . . . . .
. . . . .
Vv12g0026 . . . . . . .
. . . . . . . .
. . . . .
Vv12g0027 . . . . . . .
. . . . . . . .
. . . . .
Vv12g0028 . . . . . . .
. . . . . . . .
. . . . .
Vv12g0029 . . . . . . .
. . . . . . . .
. . . . .
Vv12g0030 . . . . . . .
. . . . . . . .
. . . . .
Vv12g0031 . . . . . . .
. . . . . . . .
. . . . .
Vv12g0032 . . . . . . .
. . . . . . . .
. . . . .
Vv12g0033 . . . . . . .
. . . . . . . .
. . . . .
Vv12g0034 Tc01_g002410 . . . Gorai.
003G012100.1 . . . . . . .
. . . . . . . .
Vv12g0035 . . . . . . .
. . . . . . . .
. . . . .
Vv12g0036 . . . . . . .
. . . . . . . .
. . . . .
Vv12g0037 . . . . . . .
. . . . . . . .
. . . . .
Vv12g0038 . . . . . . .
. . . . . . . .
. . . . .
Vv12g0039 . . . . . . .
. . . . . . . .
. . . . .
Vv12g0040 Tc01_g002590 Gorai.007G050000.1 Gorai.
008G065400.1 . Gorai.003G012800.1 . . .
. . . . . . . .
. . . .
Vv12g0041 . . . . . . .
. . . . . . . .

. . . . .
Vv12g0042 . . . . . . .
. . . . . . . .
. . . . .
Vv12g0043 . . . . . . .
. . . . . . . .
. . . . .
Vv12g0044 . . . . . . .
. . . . . . . .
. . . . .
Vv12g0045 . . . . . . .
. . . . . . . .
. . . . .
Vv12g0046 . . . . . . .
. . . . . . . .
. . . . .
Vv12g0047 . . . . . . .
. . . . . . . .
. . . . .
Vv12g0048 . . . . . . .
. . . . . . . .
. . . . .
Vv12g0049 Tc01_g002880 . . . . .
. . . . . . .
. . . . . .
Vv12g0050 . . . . . . .
. . . . . . . .
. . . . .
Vv12g0051 . . . . . . .
. . . . . . . .
. . . . .
Vv12g0052 . . . . . . .
. . . . . . . .
. . . . .
Vv12g0053 . . . . . . .
. . . . . . . .
. . . . .
Vv12g0054 . . . . . Gorai.
001G093000.1 . . . . . . .
. . . . . . .
Vv12g0055 . . . . . . .
. . . . . . . .
. . . . .
Vv12g0056 . . . . . . .
. . . . . . . .
. . . . .
Vv12g0057 . . . . . . .
. . . . . . . .
. . . . .
Vv12g0058 . . . . . . .

. . . . . . . .
. . . . .
Vv12g0059 . . . . . . .
. . . . . . . .
. . . . .
Vv12g0060 . . . . . . .
. . . . . . . .
. . . . .
Vv12g0061 . . . . . . .
. . . . . . . .
. . . . .
Vv12g0062 . . . . . . .
. . . . . . . .
. . . . .
Vv12g0063 . . . . . . .
. . . . . . . .
. . . . .
Vv12g0064 . . . . . . .
. . . . . . . .
. . . . .
Vv12g0065 Tc01_g031820 . Gorai.008G138500.1 .
. . . . . . . .
. . . . . . . .
Vv12g0066 . . . . . . .
. . . . . . . .
. . . . .
Vv12g0067 . . . . . . .
. . . . . . . .
. . . . .
Vv12g0068 . . . . . . .
. . . . . . . .
. . . . .
Vv12g0069 Tc01_g016860 . . . . .
. . . . . . . .
. . . . . .
Vv12g0070 . . . . . . .
. . . . . . . .
. . . . .
Vv12g0071 . . . . . . .
. . . . . . . .
. . . . .
Vv12g0072 . . . . . . .
. . . . . . . .
. . . . .
Vv12g0073 . . . . . . .
. . . . . . . .
. . . . .
Vv12g0074 Tc01_g016800 . . . .
Gorai.001G092400.1 . . . . . .
. . . . . . . .

Vv12g0075 Tc01_g016790 . . . .
Gorai.001G092300.1 . . . . . .
. . . . . . . .
Vv12g0076 Tc01_g016780 . Gorai.001G211700.1 .
. Gorai.001G092200.1 . . . .
. . . . . . . .
. .
Vv12g0077 . . . . . . .
. . . . . . . .
. . . . .
Vv12g0078 Tc01_g016760 . Gorai.001G211500.1 .
. . . . . . . .
. . . . . . . .
Vv12g0079 . Gorai.007G173500.1 . . .
. . . . . . . .
. . . . . . .
Vv12g0080 Tc01_g016730 . . . .
Gorai.001G092100.1 . . . . . .
. . . . . . . .
Vv12g0081 Tc01_g016720 . . . . .
. . . . . . . .
. . . . . .
Vv12g0082 Tc01_g016710 Gorai.007G174900.1 . .
. . . . . . . .
. . . . . . . .
Vv12g0083 . . . . . . .
. . . . . . . .
. . . . .
Vv12g0084 Tc01_g015210 . . . .
Gorai.001G092000.1 . . . . . .
. . . . . . . .
Vv12g0085 . . . . . . .
. . . . . . . .
. . . . .
Vv12g0086 Tc01_g015200 . . . . .
. . . . . . . .
. . . . . .
Vv12g0087 . . . . . . .
. . . . . . . .
. . . . .
Vv12g0088 . Gorai.007G175000.1 . . .
. . . . . . . .
. . . . . . .
Vv12g0089 Tc01_g015170 . . . . .
. . . . . . . .
. . . . . .
Vv12g0090 Tc01_g015160 . . . . .
. . . . . . . .
. . . . . .
Vv12g0091 . . . . . . .

| | | | | | | | |
|---|---|---|---|---|---|---|---|
| . | . | . | . | . | . | . | . |
| . | . | . | . | . | | | |
| Vv12g0092 | . | . | . | . | . | . | . |
| . | . | . | . | . | . | . | . |
| . | . | . | . | . | | | |
| Vv12g0093 | Tc01_g015140 | | Gorai.007G175100.1 | | | . | . |
| . | . | . | . | . | . | . | . |
| . | . | . | . | . | . | . | . |
| Vv12g0094 | Tc01_g015120 | | . | . | . | . | . |
| . | . | . | . | . | . | . | . |
| . | . | . | . | . | . | | |
| Vv12g0095 | . | . | . | . | . | . | . |
| . | . | . | . | . | . | . | . |
| . | . | . | . | . | | | |
| Vv12g0096 | . | . | . | . | . | . | . |
| . | . | . | . | . | . | . | . |
| . | . | . | . | . | | | |
| Vv12g0097 | Tc01_g015090 | | . | . | . | . | . |
| . | . | . | . | . | . | . | . |
| . | . | . | . | . | . | | |
| Vv12g0098 | Tc01_g015070 | | . | . | . | . | . |
| . | . | . | . | . | . | . | . |
| . | . | . | . | . | . | | |
| Vv12g0099 | . | . | . | . | . | . | . |
| . | . | . | . | . | . | . | . |
| . | . | . | . | . | | | |
| Vv12g0100 | . | . | . | . | . | . | . |
| . | . | . | . | . | . | . | . |
| . | . | . | . | . | | | |
| Vv12g0101 | . | . | . | . | . | . | . |
| . | . | . | . | . | . | . | . |
| . | . | . | . | . | | | |
| Vv12g0102 | . | . | . | . | . | . | . |
| . | . | . | . | . | . | . | . |
| . | . | . | . | . | | | |
| Vv12g0103 | . | . | . | . | . | . | . |
| . | . | . | . | . | . | . | . |
| . | . | . | . | . | | | |
| Vv12g0104 | Tc01_g032050 | | . | Gorai.008G137800.1 | | | . |
| . | . | . | . | . | . | . | . |
| . | . | . | . | . | . | . | . |
| Vv12g0105 | Tc01_g015060 | | . | . | . | . | . |
| . | . | . | . | . | . | . | . |
| . | . | . | . | . | . | | |
| Vv12g0106 | Tc01_g010590 | | . | . | . | . | . |
| . | . | . | . | . | . | . | . |
| . | . | . | . | . | . | | |
| Vv12g0107 | Tc01_g015050 | | . | . | . | . | . |
| . | . | . | . | . | . | . | . |
| . | . | . | . | . | . | | |

Vv12g0108 Tc01_g015040 . . . . .
. . . . . . . .
. . . . . .
Vv12g0109 Tc01_g015000 . . . . .
. . . . . . . .
. . . . . .
Vv12g0110 Tc01_g014990 . . . . .
. . . . . . . .
. . . . . .
Vv12g0111 Tc01_g014950 . Gorai.008G099600.1 .
. . . . . . . .
. . . . . . . .
Vv12g0112 Tc01_g039880 . Gorai.008G111100.1 .
. . . . . . . .
. . . . . . . .
Vv12g0113 Tc01_g014920 . . . . .
Vv0Unrg9177 . . . . . .
. . . . . . .
Vv12g0114 . . . . . . .
. . . . . . . .
. . . . .
Vv12g0115 Tc01_g014910 . Gorai.008G097100.1 .
. . Vv0Unrg9178 . . . .
. . . . . . . .
.
Vv12g0116 . . . . . . .
. . . . . . . .
. . . . .
Vv12g0117 . . . . . . .
. . . . . . . .
. . . . .
Vv12g0118 . . . . . . .
. . . . . . . .
. . . . .
Vv12g0119 . . . . . .
Vv0Unrg9191 . . . . . . .
. . . . . .
Vv12g0120 Tc01_g014840 . . . . .
. . . . . . . .
. . . . . .
Vv12g0121 . . . . . . .
. . . . . . . .
. . . . .
Vv12g0122 . . . . . . .
. . . . . . . .
. . . . .
Vv12g0123 . . . . . . .
. . . . . . . .
. . . . .
Vv12g0124 . . . . . . .

```
        .        .       .        .       .        .       .        .
        .        .       .        .       .
Vv12g0125        .       .        .       .        .       .        .
        .        .       .        .       .        .       .        .
        .        .       .        .       .
Vv12g0126        .       .        .       .        .       .        .
        .        .       .        .       .        .       .        .
        .        .       .        .       .
Vv12g0127        .       .        .       .        .       .        .
        .        .       .        .       .        .       .        .
        .        .       .        .       .
Vv12g0128        Tc01_g032480     .       Gorai.008G136700.1        .
        .        .       Vv0Unrg9198      .        .       .        .
        .        .       .        .       .        .       .        .
        .
Vv12g0129        .       .        .       .        .       .        .
        .        .       .        .       .        .       .        .
        .        .       .        .       .
Vv12g0130        .       .        .       .        .       .
Vv0Unrg9199      .       .        .       .        .       .        .
        .        .       .        .       .        .
Vv12g0131        .       .        .       .        .       .
Vv0Unrg9200      .       .        .       .        .       .        .
        .        .       .        .       .        .
Vv12g0132        .       .        .       .        .       .        .
        .        .       .        .       .        .       .        .
        .        .       .        .       .
Vv12g0133        .       .        .       .        .       .        .
        .        .       .        .       .        .       .        .
        .        .       .        .       .
Vv12g0134        .       .        .       .        .       .        .
        .        .       .        .       .        .       .        .
        .        .       .        .       .
Vv12g0135        .       .        .       .        .       .        .
        .        .       .        .       .        .       .        .
        .        .       .        .       .
Vv12g0136        .       .        .       .        .       .        .
        .        .       .        .       .        .       .        .
        .        .       .        .       .
Vv12g0137        .       .        .       .        .       .        .
        .        .       .        .       .        .       .        .
        .        .       .        .       .
Vv12g0138        .       .        .       .        .       .        .
        .        .       .        .       .        .       .        .
        .        .       .        .       .
Vv12g0139        .       .        .       .        .       .        .
        .        .       .        .       .        .       .        .
        .        .       .        .       .
Vv12g0140        .       .        .       .        .       .        .
        .        .       .        .       .        .       .        .
```

. . . . .
Vv12g0141 Tc01_g010170 . . . . .
. . . . . . . .
. . . . . .
Vv12g0142 . . . . . . .
. . . . . . Vv10g0192
. . . . Gorai.011G036400.1
Gorai.010G240800.1
Vv12g0143 . . . . . . .
. . . . . . . .
. . . . .
Vv12g0144 . . . . . . .
. . . . . . . .
. . . . .
Vv12g0145 Tc01_g039490 . . . . .
. . . . . . . .
. . . . . .
Vv12g0146 Tc01_g039480 . . . . .
. . . . . . . .
. . . . . .
Vv12g0147 . . . . . . .
. . . . . . . .
. . . . .
Vv12g0148 Tc01_g039470 . . . . .
. . . . . . . .
. . . . . .
Vv12g0149 . . . . . . .
. . . . . . . .
. . . . .
Vv12g0150 . . . . . . .
. . . . . . . .
. . . . .
Vv12g0151 . . . . . . .
. . . . . . . .
. . . . .
Vv12g0152 . . . . . . .
. . . . . . . .
. . . . .
Vv12g0153 Tc01_g039420 . . . . .
. . . . . . . .
. . . . . .
Vv12g0154 Tc01_g039410 . . . . .
. . . . . . . .
. . . . . .
Vv12g0155 . . . . . . .
. . . . . . . .
. . . . .
Vv12g0156 . . . . . . .
. . . . . . . .
. . . . .

Vv12g0157 . . . . . . .
. . . . . . . .
. . . . .
Vv12g0158 . . . . . . .
. . . . . . . .
. . . . .
Vv12g0159 Tc01_g014770 . . . . .
. . . . . . . .
. . . . . .
Vv12g0160 . . . . . . .
. . . . . . . .
. . . . .
Vv12g0161 . . . . . . .
. . . . . . . .
. . . . .
Vv12g0162 . . . . . . .
. . . . . . . .
. . . . .
Vv12g0163 Tc01_g006040 . Gorai.008G079700.1 .
Gorai.003G028100.1 . . . . .
. . . . . . . .
. .
Vv12g0164 . . . . . . .
. . . . . . . .
. . . . .
Vv12g0165 Tc01_g005950 . Gorai.008G079500.1 .
. . . . . . . .
. . . . . . . .
Vv12g0166 . . . . . . .
. . . . . . . .
. . . . .
Vv12g0167 Tc01_g039170 . . . . .
. . . . . . . .
. . . . . .
Vv12g0168 . . . . . . .
. . . . . . . .
. . . . .
Vv12g0169 . . . . . . .
. . . . . . Vv10g0183
Tc06_g013530 . . Gorai.009G160400.1
Gorai.011G037000.1 Gorai.010G241200.1
Vv12g0170 Tc01_g014750 . . . . .
Vv0Unrg9240 . . . . . .
Vv10g0178 Tc06_g013990 . Gorai.010G161000.1
Gorai.009G160800.1 Gorai.011G037500.1 .
Vv12g0171 . . . . . . .
. . . . . . . .
. . . . .
Vv12g0172 . . . . . .
Vv10g0175 Tc06_g014020 . Gorai.010G160700.1

Gorai.009G161100.1 . . . . . .
. . .
Vv12g0173 . . . . . . .
. . . . . . . .
. . . . .
Vv12g0174 . . . . . . .
. . . . . . . .
. . . . .
Vv12g0175 . . . . . . .
. . . . . . . .
. . . . .
Vv12g0176 . . . . . . .
. . . . . . . .
. . . . .
Vv12g0177 . . . . . . .
. . . . . . . .
. . . . .
Vv12g0178 . . . . . .
Vv10g0169 Tc06_g014090 Gorai.006G089700.1 Gorai.
010G160100.1 Gorai.009G161300.1 Gorai.011G038200.1
Gorai.010G241400.1 . . . . . .
.
Vv12g0179 . . . . . . .
. . . . . . . .
. . . . .
Vv12g0180 Tc01_g014580 . . . . .
. . . . . . . .
. . . . . .
Vv12g0181 . . . . . . .
. . . . . . . .
. . . . .
Vv12g0182 . . . . . . .
. . . . . . . .
. . . . .
Vv12g0183 . . . . . . .
. . . . . . . .
. . . . .
Vv12g0184 . . . . . . .
. . . . . . . .
. . . . .
Vv12g0185 Tc01_g001190 . . . Gorai.
003G006600.1 . . . . . . .
. . . . . . . .
Vv12g0186 Tc01_g014260 . . . . .
. . . . . . . .
. . . . . .
Vv12g0187 . . . . . . .
. . . . . . . .
. . . . .
Vv12g0188 . . . . . . .

| | | | | | | | |
|---|---|---|---|---|---|---|---|
| . | . | . | . | . | . | . | . |
| . | . | . | . | . | | | |
| Vv12g0189 | . | . | . | . | . | . | . |
| . | . | . | . | . | . | . | . |
| . | . | . | . | . | | | |
| Vv12g0190 | . | . | . | . | . | . | . |
| . | . | . | . | . | . | . | . |
| . | . | . | . | . | | | |
| Vv12g0191 | . | . | . | . | . | . | . |
| . | . | . | . | . | . | . | . |
| . | . | . | . | . | | | |
| Vv12g0192 | . | . | . | . | . | . | . |
| . | . | . | . | . | . | . | . |
| . | . | . | . | . | | | |
| Vv12g0193 | . | . | . | . | . | . | . |
| . | . | . | . | . | . | . | . |
| . | . | . | . | . | | | |
| Vv12g0194 | . | . | . | . | . | . | . |
| . | . | . | . | . | . | . | . |
| . | . | . | . | . | | | |
| Vv12g0195 | . | . | . | . | . | . | . |
| . | . | . | . | . | . | . | . |
| . | . | . | . | . | | | |
| Vv12g0196 | . | . | . | . | . | . | . |
| . | . | . | . | . | . | . | . |
| . | . | . | . | . | | | |
| Vv12g0197 | . | . | . | . | . | . | . |
| . | . | . | . | . | . | . | . |
| . | . | . | . | . | | | |
| Vv12g0198 | . | . | . | . | . | . | . |
| . | . | . | . | . | . | . | . |
| . | . | . | . | . | | | |
| Vv12g0199 | . | . | . | . | . | . | . |
| . | . | . | . | . | . | . | . |
| . | . | . | . | . | | | |
| Vv12g0200 | . | . | . | . | . | . | . |
| . | . | . | . | . | . | . | . |
| . | . | . | . | . | | | |
| Vv12g0201 | . | . | . | . | . | . | . |
| . | . | . | . | . | . | . | . |
| . | . | . | . | . | | | |
| Vv12g0202 | Tc01_g013990 | | . | . | . | . | . |
| . | . | . | . | . | . | . | . |
| . | . | . | . | . | . | | |
| Vv12g0203 | . | . | . | . | . | . | |
| Vv19g0130 | . | . | . | . | Gorai.009G367300.1 | | |
| . | . | . | . | . | . | . | . |
| Vv12g0204 | . | . | . | . | . | . | . |
| . | . | . | . | . | . | . | . |
| . | . | . | . | . | | | |

Vv12g0205 . . . . . . .
. . . . . . .
. . . . .
Vv12g0206 . . . . . . .
. . . . . . .
. . . . .
Vv12g0207 . . . . . . .
. . . . . . .
. . . . .
Vv12g0208 . . . . . . .
. . . . . . Vv10g0152
Tc06_g014230 . . . . .
Vv12g0209 . . . . . . .
. . . . . . Vv10g0151
. . Gorai.010G159700.1 Gorai.009G161800.1
Gorai.011G039000.1 .
Vv12g0210 . . . . . . .
. . . . . . .
. . . . .
Vv12g0211 . . . . . . .
. . . . . . .
. . . . .
Vv12g0212 Tc01_g013820 . . . . .
. . . . . . .
Vv10g0149 Tc06_g014260 . . Gorai.009G161900.1
Gorai.011G039100.1 .
Vv12g0213 . . . . . . .
. . . . . . .
. . . . .
Vv12g0214 Tc01_g013810 . . . . .
. . . . . . .
. . . . . .
Vv12g0215 Tc01_g013800 . . . . .
. . . . . . .
. . . . . .
Vv12g0216 . . . . . . .
. . . . . . Vv10g0141
Tc06_g014330 . . Gorai.009G162200.1
Gorai.011G039600.1 .
Vv12g0217 . . . . . . .
. . . . . . .
. . . . .
Vv12g0218 Tc01_g013690 . . . . .
. . . . . . .
. . . . . .
Vv12g0219 . . . . . . .
Vv19g0143 . Gorai.013G173500.1 . Gorai.
002G061400.1 . . . . . . .
. .
Vv12g0220 Tc01_g001320 . . . . .

. . . . . . .
Vv10g0132 Tc06_g014440 Gorai.006G088900.1 .
Gorai.009G162900.1 . .
Vv12g0221 . . . . . . .
. . . . . . . .
. . . . .
Vv12g0222 . . . . . . .
. . . . . . . .
. . . . .
Vv12g0223 . . . . . . .
. . . . . . . .
. . . . .
Vv12g0224 Tc01_g001340 . . Gorai.007G192200.1
. . Vv19g0150 . Gorai.013G173200.1
Gorai.007G326600.1 Gorai.002G061700.1 Gorai.
009G368300.1 . . . . . . .
.
Vv12g0225 Tc01_g013620 . . . . .
. . . . . . .
Vv10g0131 Tc06_g014450 . . Gorai.009G163100.1
. Gorai.010G242000.1
Vv12g0226 Tc01_g013610 . . . . .
. . . . . . . .
. . . . . .
Vv12g0227 Tc01_g013590 . . . . .
. . . . . . . .
. . . . . .
Vv12g0228 Tc01_g013580 . . . . .
. . . . . . . .
. . . . . .
Vv12g0229 . . . . . . .
. . . . . . . .
. . . . .
Vv12g0230 . . . . . . .
. . . . . . . .
. . . . .
Vv12g0231 Tc01_g001670 . . Gorai.007G194500.1
Gorai.003G009500.1 Gorai.011G098300.1 . .
. . . . . . . .
. . . .
Vv12g0232 . . . . . . .
. . . . . . Vv10g0122
Tc06_g014560 . . . . .
Vv12g0233 . . . . . . .
. . . . . . Vv10g0121
Tc06_g014580 . . . . .
Vv12g0234 Tc01_g019120 . . . . .
. . . . . . . .
. . . . . .
Vv12g0235 Tc01_g013560 . . . . .

. . . . . . . .
. . . . . .
Vv12g0236 . . . . . . .
. . . . . . . .
. . . . .
Vv12g0237 Tc01_g013550 . . . . .
. . . . . . . .
. . . . . .
Vv12g0238 Tc01_g013540 . . . . .
. . . . . . . .
. . . . . .
Vv12g0239 Tc01_g013530 . . . . .
. . . . . . . .
. . . . . .
Vv12g0240 Tc01_g013520 . . . . .
. . . . . . . .
. . . . . .
Vv12g0241 . . . . . . .
. . . . . . . .
. . . . .
Vv12g0242 Tc01_g013500 . . . . .
. . . . . . .
Vv10g0119 Tc06_g014600 Gorai.006G088800.1 . .
Gorai.011G033800.1 Gorai.010G242200.1
Vv12g0243 Tc01_g001700 . . . . .
. . . . . . . .
. . . . . .
Vv12g0244 . . . . . . .
. . . . . . . .
. . . . .
Vv12g0245 . . . . . . .
. . . . . . . .
. . . . .
Vv12g0246 Tc01_g013490 . . . . .
Vv19g0164 Tc07_g009400 . . . .
. . . . . . . .
Vv12g0247 . . . . . . .
. . . . . . . .
. . . . .
Vv12g0248 . . . . . . .
. . . . . . . .
. . . . .
Vv12g0249 . . . . . . .
. . . . . . Vv10g0116
Tc06_g014640 . . Gorai.009G163800.1
Gorai.011G033600.1 .
Vv12g0250 . . . . . . .
. . . . . . . .
. . . . .
Vv12g0251 . . . . . . .

. . . . . . Vv10g0105
Tc06_g014740 . . Gorai.009G164200.1 .
.
Vv12g0252 . . . . . . .
. . . . . . . .
. . . . .
Vv12g0253 . . . . . .
Vv19g0168 Tc07_g009360 . . Gorai.002G063500.1
Gorai.009G372700.1 . . . . .
. . .
Vv12g0254 Tc01_g018850 . . . . .
. . . . . . . .
. . . . . .
Vv12g0255 . . . . . .
Vv19g0171 Tc07_g009290 Gorai.013G172800.1 .
Gorai.002G064500.1 Gorai.009G373000.1 . Vv10g0090
. . . . . .
Vv12g0256 . . . . . . .
. . . . . . . .
. . . . .
Vv12g0257 . . . . . . .
. . . . . . . .
. . . . .
Vv12g0258 . . . . . .
Vv19g0174 Tc07_g009260 . Gorai.007G325800.1 .
. . Vv10g0087 Tc06_g014890 . .
. . .
Vv12g0259 . . . . . .
Vv19g0175 Tc07_g009250 . . Gorai.002G064800.1
. . . . . . . .
.
Vv12g0260 . . . . . .
Vv19g0177 . . . . . . .
. . . . . .
Vv12g0261 . . . . . . .
. . . . . . . .
. . . . .
Vv12g0262 . . . . . . .
. . . . . . . .
. . . . .
Vv12g0263 . . . . . . .
. . . . . . . .
. . . . .
Vv12g0264 . . . . . . .
. . . . . . . .
. . . . .
Vv12g0265 . . . . . .
Vv19g0182 Tc07_g009200 Gorai.013G172500.1 Gorai.
007G325700.1 Gorai.002G064900.1 . . . .
. . . . .

Vv12g0266 . . . . . . .
. . . . . . . .
. . . . .
Vv12g0267 Tc01_g018550 . . . . .
. . . . . . . .
. . . . . .
Vv12g0268 . . . . . . .
. . . . . . . .
. . . . .
Vv12g0269 . . . . . .
Vv06g0393 . . . . . . .
. . . . . .
Vv12g0270 Tc01_g040940 . . . . .
. . . . . . .
Vv10g0066 Tc06_g015030 . . . Gorai.
011G031400.1 Gorai.010G242900.1
Vv12g0271 . . . . . .
Vv06g0377 . . Gorai.001G130700.1 . .
. . . . . . . .
Vv12g0272 Tc01_g010210 . . . . .
. . . . . . . .
. . . . . .
Vv12g0273 . . . . . .
Vv06g0369 . . . . . Gorai.
006G120500.1 . . . . . . .
Vv12g0274 . . . . . . .
. . . . . . Vv10g0057
Tc06_g015110 . . Gorai.009G165800.1 .
.
Vv12g0275 . . . . . . .
. . . . . . Vv10g0056
Tc06_g015120 . . Gorai.009G165900.1 .
.
Vv12g0276 . . . . . . .
. . . . . . . .
. . . . .
Vv12g0277 . . . . . . .
. . . . . . Vv10g0055
Tc06_g015140 . . . . .
Vv12g0278 . . . . . . .
. . . . . . . .
. . . . .
Vv12g0279 . . . . . . .
. . . . . . . .
. . . . .
Vv12g0280 . . . . . . .
. . . . . . . .
. . . . .
Vv12g0281 . . . . . . .
. . . . . . . .

. . . . .
Vv12g0282 . . . . . . .
. . . . . . Vv10g0054
Tc06_g015150 . . . . .
Vv12g0283 . . . . . . .
. . . . . . . .
. . . . .
Vv12g0284 . . . . . . .
. . . . . . Vv10g0053
Tc06_g015160 . . . . .
Vv12g0285 . . . . . . .
. . . . . . . .
. . . . .
Vv12g0286 . . . . . . .
. . . . . . Vv10g0051
Tc06_g015170 . . . . .
Vv12g0287 . . . . . . .
. . . . . . . .
. . . . .
Vv12g0288 . . . . . . .
. . . . . . . .
. . . . .
Vv12g0289 . . . . . . .
. . . . . . Vv10g0050
. . . . . .
Vv12g0290 . . . . . . .
. . . . . . . .
. . . . .
Vv12g0291 . . . . . . .
. . . . . . . .
. . . . .
Vv12g0292 . . . . . . .
. . . . . . . .
. . . . .
Vv12g0293 . . . . . . .
. . . . . . . .
. . . . .
Vv12g0294 . . . . . . .
. . . . . . . .
. . . . .
Vv12g0295 . . . . . . .
. . . . . . . .
. . . . .
Vv12g0296 . . . . . . .
. . . . . . . .
. . . . .
Vv12g0297 . . . . . . .
. . . . . . . .
. . . . .
Vv12g0298 . . . . . . .

. . . . . . . .
. . . . .
Vv12g0299 . . . . . . .
. . . . . . . .
. . . . .
Vv12g0300 . . . . . . .
. . . . . . . .
. . . . .
Vv12g0301 . . . . . . .
. . . . . . . .
. . . . .
Vv12g0302 . . . . . .
Vv06g0367 . . Gorai.001G130800.1 .
Gorai.009G078700.1 . Vv10g0047 Tc06_g015200
Gorai.006G088500.1 . . Gorai.011G030800.1 .
Vv12g0303 . . . . . . .
. . . . . . . .
. . . . .
Vv12g0304 Tc01_g040750 . . . . .
Vv06g0362 Tc09_g025680 . Gorai.001G131000.1
. . Gorai.006G167600.1 . . .
. . . .
Vv12g0305 . . . . . . .
. . . . . . . .
. . . . .
Vv12g0306 . . . . . . .
. . . . . . Vv10g0043
Tc06_g015240 Gorai.006G088400.1 . Gorai.
009G166300.1 . .
Vv12g0307 Tc01_g010390 . . . . .
. . . . . . . .
. . . . . .
Vv12g0308 . . . . . . .
. . . . . . Vv10g0040
Tc06_g015270 . . . Gorai.011G030300.1
.
Vv12g0309 . . . . . . .
. . . . . . Vv10g0037
. . . . . Gorai.010G243500.1
Vv12g0310 . . . . . . .
. . . . . . . .
. . . . .
Vv12g0311 . . . . . . .
. . . . . . . .
. . . . .
Vv12g0312 . . . . . . .
. . . . . . . .
. . . . .
Vv12g0313 . . . . . . .
. . . . . . . .

| | | | | | | | | |
|---|---|---|---|---|---|---|---|---|
| | . | . | . | . | . | | | |
| Vv12g0314 | | . | . | . | . | . | . | . |
| | . | . | . | . | . | . | . | . |
| | . | . | . | . | . | | | |
| Vv12g0315 | | . | . | . | . | . | . | . |
| | . | . | . | . | . | . | . | . |
| | . | . | . | . | . | | | |
| Vv12g0316 | | Tc01_g010590 | | . | . | . | . | . |
| | . | . | . | . | . | . | . | . |
| | . | . | . | . | . | . | | |
| Vv12g0317 | | . | . | . | . | . | . | . |
| | . | . | . | . | . | . | . | . |
| | . | . | . | . | . | | | |
| Vv12g0318 | | . | . | . | . | . | . | . |
| | . | . | . | . | . | . | . | . |
| | . | . | . | . | . | | | |
| Vv12g0319 | | . | . | . | . | . | . | . |
| | . | . | . | . | . | . | . | . |
| | . | . | . | . | . | | | |
| Vv12g0320 | | . | . | . | . | . | . | . |
| | . | . | . | . | . | . | Vv10g0028 | |
| | Tc06_g015400 | | . | . | Gorai.009G167200.1 | | | . |
| | Gorai.010G256800.1 | | | | | | | |
| Vv12g0321 | | . | . | . | . | . | . | . |
| | . | . | . | . | . | . | . | . |
| | . | . | . | . | . | | | |
| Vv12g0322 | | . | . | . | . | . | . | . |
| | . | . | . | . | . | . | . | . |
| | . | . | . | . | . | | | |
| Vv12g0323 | | . | . | . | . | . | . | . |
| | . | . | . | . | . | . | . | . |
| | . | . | . | . | . | | | |
| Vv12g0324 | | . | . | . | . | . | . | . |
| | . | . | . | . | . | . | . | . |
| | . | . | . | . | . | | | |
| Vv12g0325 | | . | . | . | . | . | . | . |
| | . | . | . | . | . | . | . | . |
| | . | . | . | . | . | | | |
| Vv12g0326 | | . | . | . | . | . | . | . |
| | . | . | . | . | . | . | . | . |
| | . | . | . | . | . | | | |
| Vv12g0327 | | . | . | . | . | . | . | . |
| | . | . | . | . | . | . | . | . |
| | . | . | . | . | . | | | |
| Vv12g0328 | | . | . | . | . | . | . | . |
| | . | . | . | . | . | . | . | . |
| | . | . | . | . | . | | | |
| Vv12g0329 | | . | . | . | . | . | . | . |
| | . | . | . | . | . | . | . | . |
| | . | . | . | . | . | | | |

Vv12g0330 . . . . . . .
. . . . . . .
. . . . .
Vv12g0331 . . . . . . .
. . . . . . .
. . . . .
Vv12g0332 . . . . . . .
. . . . . . .
. . . . .
Vv12g0333 . . . . . . .
. . . . . . .
. . . . .
Vv12g0334 Tc01_g040660 . . . . .
Vv06g0347 . . . . .
Gorai.003G141000.1 . . . . . .
.
Vv12g0335 Tc01_g000370 . . . Gorai.
003G001500.1 . . . . . . .
. . . . . . . .
Vv12g0336 . . . . . . .
. . . . . . .
. . . . .
Vv12g0337 . . . . . . .
. . . . . . Vv10g0018
. . Gorai.010G155500.1 . . .
Vv12g0338 Tc01_g000650 . . . Gorai.
003G003200.1 . . . . . . .
. Vv10g0014 Tc06_g015550 . . .
Gorai.011G029400.1 .
Vv12g0339 . . . . . . .
. . . . . . .
. . . . .
Vv12g0340 . . . . . . .
. . . . . . .
. . . . .
Vv12g0341 . . . . . . .
. . . . . . .
. . . . .
Vv12g0342 . . . . . . .
. . . . . . .
. . . . .
Vv12g0343 . . . . . . .
. . . . . . .
. . . . .
Vv12g0344 . . . . . . .
. . . . . . .
. . . . .
Vv12g0345 . . . . . . .
. . . . . . .
. . . . .

Vv12g0346 . . . . . . .
. . . . . . . .
. . . . .
Vv12g0347 . . . . . . .
. . . . . . . .
. . . . .
Vv12g0348 . . . . .
Vv06g0310 . Gorai.013G252300.1 . . .
Gorai.006G132200.1 . . . . .
. .
Vv12g0349 Tc01_g027630 . . . . .
. . . . . . . .
. . . . . .
Vv12g0350 . . . . . . .
. . . . . . Vv10g0006
Tc06_g015660 . Gorai.010G154800.1 . .
Gorai.010G154800.1
Vv12g0351 . . . . . .
Vv19g0245 . Gorai.013G169100.1 . Gorai.
002G069300.1 . . . . . . .
. .
Vv12g0352 . . . . . . .
. . . . . . . .
. . . . .
Vv12g0353 . . . . . . .
. . . . . . . .
. . . . .
Vv12g0354 . . . . . . .
. . . . . . . .
. . . . .
Vv12g0355 . . . . . . .
. . . . . . . .
. . . . .
Vv12g0356 Tc01_g011040 . . . . .
. . . . . . . .
. . . . . .
Vv12g0357 . . . . . . .
. . . . . . . .
. . . . .
Vv12g0358 Tc01_g011390 . . . . .
. . . . . . . .
. . . . . .
Vv12g0359 Tc01_g011770 . . . . .
. . . . . . . .
. . . . . .
Vv12g0360 . . . . . . .
. . . . . . . .
. . . . .
Vv12g0361 Tc01_g011790 . . . . .
. . . . . . . .

. . . . . .
Vv12g0362 . . . . . .
Vv19g0270 . . . Gorai.002G070000.1
Gorai.009G388000.1 . . . . . .
. .
Vv12g0363 . . . . . . .
. . . . . . . .
. . . . .
Vv12g0364 . . . . . . .
. . . . . . . .
. . . . .
Vv12g0365 . . . . . . .
. . . . . . . .
. . . . .
Vv12g0366 . . . . . .
Vv19g0279 . . . Gorai.002G070200.1 .
. . . . . . . .
Vv12g0367 . . . . . . .
. . . . . . . .
. . . . .
Vv12g0368 . . . . . . .
. . . . . . . .
. . . . .
Vv12g0369 . . . . . . .
. . . . . . . .
. . . . .
Vv12g0370 . . . . . . .
. . . . . . . .
. . . . .
Vv12g0371 . . . . . . .
. . . . . . . .
. . . . .
Vv12g0372 Tc01_g014820 . . . . .
. . . . . . . .
. . . . . .
Vv12g0373 Tc01_g014810 . . . . .
. . . . . . . .
. . . . . .
Vv12g0374 . . . . . . .
. . . . . . . .
. . . . .
Vv12g0375 . . . . . . .
. . . . . . . .
. . . . .
Vv12g0376 . . . . . . .
. . . . . . . .
. . . . .
Vv12g0377 . . . . . . .
. . . . . . . .
. . . . .

| | | | | | | | |
|---|---|---|---|---|---|---|---|
| Vv12g0378 | . | . | . | . | . | . | . |
| . | . | . | . | . | . | . | . |
| . | . | . | . | . | | | |
| Vv12g0379 | . | . | . | . | . | . | . |
| . | . | . | . | . | . | . | . |
| . | . | . | . | . | | | |
| Vv12g0380 | . | . | . | . | . | . | . |
| . | . | . | . | . | . | . | . |
| . | . | . | . | . | | | |
| Vv12g0381 | . | . | . | . | . | . | . |
| . | . | . | . | . | . | . | . |
| . | . | . | . | . | | | |
| Vv12g0382 | . | . | . | . | . | . | . |
| . | . | . | . | . | . | . | . |
| . | . | . | . | . | | | |
| Vv12g0383 | . | . | . | . | . | . | . |
| . | . | . | . | . | . | . | . |
| . | . | . | . | . | | | |
| Vv12g0384 | . | . | . | . | . | . | |
| Vv19g0299 | . | Gorai.013G168000.1 | | | . | . | . |
| . | . | . | . | . | . | . | . |
| Vv12g0385 | . | . | . | . | . | . | . |
| . | . | . | . | . | . | . | . |
| . | . | . | . | . | | | |
| Vv12g0386 | Tc01_g027480 | | . | . | . | . | . |
| . | . | . | . | . | . | . | . |
| . | . | . | . | . | . | | |
| Vv12g0387 | Tc01_g027240 | | . | . | . | . | . |
| . | . | . | . | . | . | . | . |
| . | . | . | . | . | . | | |
| Vv12g0388 | . | . | . | . | . | . | . |
| . | . | . | . | . | . | . | . |
| . | . | . | . | . | | | |
| Vv12g0389 | . | . | . | . | . | . | . |
| . | . | . | . | . | . | . | . |
| . | . | . | . | . | | | |
| Vv12g0390 | . | . | . | . | . | . | . |
| . | . | . | . | . | . | . | . |
| . | . | . | . | . | | | |
| Vv12g0391 | . | . | . | . | . | . | . |
| . | . | . | . | . | . | . | . |
| . | . | . | . | . | | | |
| Vv12g0392 | . | . | . | . | . | . | . |
| . | . | . | . | . | . | . | . |
| . | . | . | . | . | | | |
| Vv12g0393 | Tc01_g011840 | | . | . | . | . | . |
| . | . | . | . | . | . | . | . |
| . | . | . | . | . | . | | |
| Vv12g0394 | Tc01_g011850 | | . | . | . | . | . |
| . | . | . | . | . | . | . | . |

. . . . . .
Vv12g0395 Tc01_g011860 . . . . .
. . . . . . . .
. . . . . .
Vv12g0396 Tc01_g011890 . . . . .
. . . . . . . .
. . . . . .
Vv12g0397 . . . . . . .
. . . . . . . .
. . . . .
Vv12g0398 . . . . . . .
. . . . . . . .
. . . . .
Vv12g0399 . . . . . . .
. . . . . . . .
. . . . .
Vv12g0400 . . . . . . .
. . . . . . . .
. . . . .
Vv12g0401 . . . . . . .
. . . . . . . .
. . . . .
Vv12g0402 . . . . . . .
. . . . . . . .
. . . . .
Vv12g0403 . . . . . . .
. . . . . . . .
. . . . .
Vv12g0404 . . . . . . .
. . . . . . . .
. . . . .
Vv12g0405 . . . . . . .
. . . . . . . .
. . . . .
Vv12g0406 . . . . Gorai.004G028700.1
. Vv19g0477 . Gorai.013G165400.1 .
Gorai.002G078700.1 . . . . .
. . . .
Vv12g0407 Tc01_g030220 . . . Gorai.
004G029600.1 . Vv19g0482 . . .
Gorai.002G079500.1 . . . . . .
. . .
Vv12g0408 . . . . . . .
. . . . . . . .
. . . . .
Vv12g0409 . . . . . . .
. . . . . . . .
. . . . .
Vv12g0410 . . . . . . .
. . . . . . . .

. . . . .
Vv12g0411 . . . . . . .
. . . . . . . .
. . . . .
Vv12g0412 Tc01_g026880 . . . Gorai.
004G031100.1 . . . . . . .
. . . . . . . .
Vv12g0413 . . . . . . .
. . . . . . . .
. . . . .
Vv12g0414 . . . . . . .
. . . . . . . .
. . . . .
Vv12g0415 . . . . . . .
. . . . . . . .
. . . . .
Vv12g0416 . . . . . . .
. . . . . . . .
. . . . .
Vv12g0417 . . . . . . .
. . . . . . . .
. . . . .
Vv12g0418 . . . . . . .
. . . . . . . .
. . . . .
Vv12g0419 . . . . . . .
. . . . . . . .
. . . . .
Vv12g0420 . . . . . . .
. . . . . . . .
. . . . .
Vv12g0421 . . . . . . .
. . . . . . . .
. . . . .
Vv12g0422 . . . . . . .
. . . . . . . .
. . . . .
Vv12g0423 . . . . . . .
. . . . . . . .
. . . . .
Vv12g0424 . . . . . . .
. . . . . . . .
. . . . .
Vv12g0425 . . . . . . .
. . . . . . . .
. . . . .
Vv12g0426 . . . . . .
Vv19g0488 . . . . . . .
. . . . . .
Vv12g0427 . . . . . . .

| | | | | | | | |
|---|---|---|---|---|---|---|---|
| . | . | . | . | . | . | . | . |
| . | . | . | . | . | | | |
| Vv12g0428 | . | . | . | . | . | . | . |
| . | . | . | . | . | . | . | . |
| . | . | . | . | . | | | |
| Vv12g0429 | . | . | . | . | . | . | . |
| . | . | . | . | . | . | . | . |
| . | . | . | . | . | | | |
| Vv12g0430 | . | . | . | . | . | . | . |
| . | . | . | . | . | . | . | . |
| . | . | . | . | . | | | |
| Vv12g0431 | . | . | . | . | . | . | . |
| . | . | . | . | . | . | . | . |
| . | . | . | . | . | | | |
| Vv12g0432 | . | . | . | . | . | . | . |
| . | . | . | . | . | . | . | . |
| . | . | . | . | . | | | |
| Vv12g0433 | . | . | . | . | . | . | . |
| . | . | . | . | . | . | . | . |
| . | . | . | . | . | | | |
| Vv12g0434 | Tc01_g029790 | | . | . | . | . | . |
| . | . | . | . | . | . | . | . |
| . | . | . | . | . | . | | |
| Vv12g0435 | . | . | . | . | . | . | |
| Vv19g0510 | . | . | . | . | . | . | . |
| . | . | . | . | . | . | | |
| Vv12g0436 | . | . | . | . | . | . | . |
| . | . | . | . | . | . | . | . |
| . | . | . | . | . | | | |
| Vv12g0437 | . | . | . | . | . | . | . |
| . | . | . | . | . | . | . | . |
| . | . | . | . | . | | | |
| Vv12g0438 | . | . | . | . | . | . | |
| Vv19g0515 | . | . | . | . | . | . | . |
| . | . | . | . | . | . | | |
| Vv12g0439 | . | . | . | . | . | . | . |
| . | . | . | . | . | . | . | . |
| . | . | . | . | . | | | |
| Vv12g0440 | . | . | . | . | . | . | . |
| . | . | . | . | . | . | . | . |
| . | . | . | . | . | | | |
| Vv12g0441 | . | . | . | . | . | . | . |
| . | . | . | . | . | . | . | . |
| . | . | . | . | . | | | |
| Vv12g0442 | Tc01_g026460 | | . | . | . | . | . |
| . | . | . | . | . | . | . | . |
| . | . | . | . | . | . | | |
| Vv12g0443 | . | . | . | . | . | . | . |
| . | . | . | . | . | . | . | . |
| . | . | . | . | . | | | |

Vv12g0444 . . . . . .
Vv19g0517 Tc07_g005200 Gorai.013G164400.1 . .
. . . . . . . .
.
Vv12g0445 . . . . . . .
. . . . . . . .
. . . . .
Vv12g0446 . . . . Gorai.004G033500.1
. . . . . . . .
. . . . . . .
Vv12g0447 . . . . . . .
. . . . . . . .
. . . . .
Vv12g0448 . . . . . . .
. . . . . . . .
. . . . .
Vv12g0449 . . . . . .
Vv19g0522 Tc07_g005150 Gorai.013G164100.1 Gorai.
007G314800.1 Gorai.002G081900.1 . . . .
. . . . .
Vv12g0450 Tc01_g039350 . . . . .
. . . . . . . .
. . . . . .
Vv12g0451 Tc01_g025940 . . . . .
. . . . . . . .
. . . . . .
Vv12g0452 . . . . . . .
. . . . . . . .
. . . . .
Vv12g0453 . . . . . . .
. . . . . . . .
. . . . .
Vv12g0454 . . . . . . .
. . . . . . . .
. . . . .
Vv12g0455 . . . . . . .
. . . . . . . .
. . . . .
Vv12g0456 . . . . . . .
. . . . . . . .
. . . . .
Vv12g0457 . . . . . . .
. . . . . . . .
. . . . .
Vv12g0458 . . . . . . .
. . . . . . . .
. . . . .
Vv12g0459 . . . . . Gorai.
001G105800.1 . . . . . . .
. . . . . . .

Vv12g0460 Tc01_g029690 . . . . .
Vv19g0524 . . . . . .
. . . . . . .
Vv12g0461 . . . . . . .
. . . . . . . .
. . . . .
Vv12g0462 Tc01_g036940 . Gorai.008G116200.1 .
. . . . . . . .
. . . . . . . .
Vv12g0463 Tc01_g039540 . . . . .
. . . . . . . .
. . . . . .
Vv12g0464 . . . . . . .
. . . . . . . .
. . . . .
Vv12g0465 . . . . . . .
. . . . . . . .
. . . . .
Vv12g0466 . . . . . . .
. . . . . . . .
. . . . .
Vv12g0467 . . . . . . .
. . . . . . . .
. . . . .
Vv12g0468 . . . . . . .
. . . . . . . .
. . . . .
Vv12g0469 . . . . . Gorai.
001G104700.1 Vv08g0055 . . . . .
. . . . . . . .
Vv12g0470 . . . . . . .
. . . . . . . .
. . . . .
Vv12g0471 . . . . . . .
. . . . . . . .
. . . . .
Vv12g0472 . . . . . . .
. . . . . . . .
. . . . .
Vv12g0473 . . . . . . .
. . . . . . . .
. . . . .
Vv12g0474 Tc01_g004590 . Gorai.008G073800.1 .
. . . . . . . .
. . . . . . . .
Vv12g0475 . . . . . . .
. . . . . . . .
. . . . .
Vv12g0476 Tc01_g029350 . . . . .
. . . . . . . .

| | | | | | | | | |
|---|---|---|---|---|---|---|---|---|
| | . | . | . | . | . | . | | |
| Vv12g0477 | | . | . | . | . | . | . | . |
| | . | . | . | . | . | . | . | . |
| | . | . | . | . | . | | | |
| Vv12g0478 | | . | . | . | . | . | . | . |
| | . | . | . | . | . | . | . | . |
| | . | . | . | . | . | | | |
| Vv12g0479 | | . | . | . | . | . | . | . |
| | . | . | . | . | . | . | . | . |
| | . | . | . | . | . | | | |
| Vv12g0480 | | . | . | . | . | . | . | . |
| | . | . | . | . | . | . | . | . |
| | . | . | . | . | . | | | |
| Vv12g0481 | | . | . | . | . | . | . | . |
| | . | . | . | . | . | . | . | . |
| | . | . | . | . | . | | | |
| Vv12g0482 | | Tc01_g029340 | | . | . | . | . | . |
| | . | . | . | . | . | . | . | . |
| | . | . | . | . | . | . | | |
| Vv12g0483 | | . | . | . | . | . | . | . |
| | . | . | . | . | . | . | . | . |
| | . | . | . | . | . | | | |
| Vv12g0484 | | . | . | . | . | . | . | . |
| | . | . | . | . | . | . | . | . |
| | . | . | . | . | . | | | |
| Vv12g0485 | | . | . | . | . | . | . | . |
| | . | . | . | . | . | . | . | . |
| | . | . | . | . | . | | | |
| Vv12g0486 | | . | . | . | . | . | . | . |
| | . | . | . | . | . | . | . | . |
| | . | . | . | . | . | | | |
| Vv12g0487 | | . | . | . | . | . | . | . |
| | . | . | . | . | . | . | . | . |
| | . | . | . | . | . | | | |
| Vv12g0488 | | . | . | . | . | . | . | . |
| | . | . | . | . | . | . | . | . |
| | . | . | . | . | . | | | |
| Vv12g0489 | | Tc01_g036690 | | . | Gorai.008G117200.1 | | | . |
| | . | . | . | . | . | . | . | . |
| | . | . | . | . | . | . | . | . |
| Vv12g0490 | | . | . | . | . | . | . | . |
| | . | . | . | . | . | . | . | . |
| | . | . | . | . | . | | | |
| Vv12g0491 | | . | . | . | . | . | . | . |
| | . | . | . | . | . | . | . | . |
| | . | . | . | . | . | | | |
| Vv12g0492 | | Tc01_g039780 | | . | . | . | . | . |
| | . | . | . | . | . | . | . | . |
| | . | . | . | . | . | . | | |
| Vv12g0493 | | Tc01_g004990 | | . | . | . | . | |

Gorai.001G101700.1 . . . . . .
. . . . . . . .
Vv12g0494 Tc01_g005000 . . . .
Gorai.001G101500.1 . . . . . .
. . . . . . . .
Vv12g0495 . . . . . . .
. . . . . . . .
. . . . .
Vv12g0496 . . . . . . .
. . . . . . . .
. . . . .
Vv12g0497 . . . . . .
Vv08g0088 Tc05_g023210 . . . . .
. . . . . . .
Vv12g0498 . . . . . Gorai.
001G098600.1 Vv08g0096 . . . . .
. . . . . . . .
Vv12g0499 . . . . . .
Vv08g0103 . . . . . . .
. . . . . .
Vv12g0500 . . . . . . .
. . . . . . . .
. . . . .
Vv12g0501 . . . . . .
Vv08g0104 . . . . . . .
. . . . . .
Vv12g0502 . . . . . . .
. . . . . . . .
. . . . .
Vv12g0503 . . . . . .
Vv08g0106 . . . . . . .
. . . . . .
Vv12g0504 . . . . . . .
. . . . . . . .
. . . . .
Vv12g0505 . Gorai.007G167700.1 Gorai.001G219600.1
. . . . . . . .
. . . . . . . .
.
Vv12g0506 Tc01_g031210 . . . . .
. . . . . . . .
. . . . . .
Vv12g0507 . . . . . . .
. . . . . . . .
. . . . .
Vv12g0508 Tc01_g018680 . . . . .
. . . . . . . .
. . . . . .
Vv12g0509 . . . . . . .
. . . . . . . .

| | | | | | | | |
|---|---|---|---|---|---|---|---|
| . | . | . | . | . | | | |
| Vv12g0510 | Tc01_g031170 | | . | . | . | . | . |
| . | . | . | . | . | . | . | . |
| . | . | . | . | . | . | | |
| Vv12g0511 | . | . | . | . | . | . | . |
| . | . | . | . | . | . | . | . |
| . | . | . | . | . | | | |
| Vv12g0512 | . | . | . | . | . | . | . |
| . | . | . | . | . | . | . | . |
| . | . | . | . | . | | | |
| Vv12g0513 | . | . | . | . | . | . | . |
| . | . | . | . | . | . | . | . |
| . | . | . | . | . | | | |
| Vv12g0514 | . | . | . | . | . | . | . |
| . | . | . | . | . | . | . | . |
| . | . | . | . | . | | | |
| Vv12g0515 | . | . | . | . | . | . | |
| Vv08g0143 | Tc05_g004750 | | . | . | . | . | . |
| . | . | . | . | . | . | . | |
| Vv12g0516 | . | . | . | . | . | . | . |
| . | . | . | . | . | . | . | . |
| . | . | . | . | . | | | |
| Vv12g0517 | . | . | . | . | . | . | . |
| . | . | . | . | . | . | . | . |
| . | . | . | . | . | | | |
| Vv12g0518 | . | . | . | . | . | . | . |
| . | . | . | . | . | . | . | . |
| . | . | . | . | . | | | |
| Vv12g0519 | . | . | . | . | . | . | . |
| . | . | . | . | . | . | . | . |
| . | . | . | . | . | | | |
| Vv12g0520 | Tc01_g039880 | | . | . | . | . | . |
| . | . | . | . | . | . | . | . |
| . | . | . | . | . | . | | |
| Vv12g0521 | . | . | . | . | . | . | . |
| . | . | . | . | . | . | . | . |
| . | . | . | . | . | | | |
| Vv12g0522 | . | . | . | . | . | . | . |
| . | . | . | . | . | . | . | . |
| . | . | . | . | . | | | |
| Vv12g0523 | . | . | . | . | . | . | . |
| . | . | . | . | . | . | . | . |
| . | . | . | . | . | | | |
| Vv12g0524 | . | . | . | . | . | . | . |
| . | . | . | . | . | . | . | . |
| . | . | . | . | . | | | |
| Vv12g0525 | . | . | . | . | . | . | . |
| . | . | . | . | . | . | . | . |
| . | . | . | . | . | | | |
| Vv12g0526 | . | . | . | . | . | . | . |

```
          .        .        .        .        .        .        .        .
          .        .        .        .        .
Vv12g0527          .        .        .        .        .        .        .
          .        .        .        .        .        .        .        .
          .        .        .        .        .
Vv12g0528          Tc01_g018550      .        .        .        .        .
          .        .        .        .        .        .        .        .
          .        .        .        .        .        .
Vv12g0529          .        .        .        .        .        .        .
          .        .        .        .        .        .        .        .
          .        .        .        .        .
Vv12g0530          .        .        .        .        .        .        .
          .        .        .        .        .        .        .        .
          .        .        .        .        .
Vv12g0531          .        .        .        .        .        .        .
          .        .        .        .        .        .        .        .
          .        .        .        .        .
Vv12g0532          Tc01_g031140      .        .        .        .        .
          .        .        .        .        .        .        .        .
          .        .        .        .        .        .
Vv12g0533          .        .        .        .        .        .        .
          .        .        .        .        .        .        .        .
          .        .        .        .        .
Vv12g0534          .        .        .        .        .        .        .
          .        .        .        .        .        .        .        .
          .        .        .        .        .
Vv12g0535          .        .        .        .        .        .        .
          .        .        .        .        .        .        .        .
          .        .        .        .        .
Vv12g0536          .        .        .        .        .        .        .
          .        .        .        .        .        .        .        .
          .        .        .        .        .
Vv12g0537          .        .        .        .        .        .        .
          .        .        .        .        .        .        .        .
          .        .        .        .        .
Vv12g0538          .        .        .        .        .        .        .
          .        .        .        .        .        .        .        .
          .        .        .        .        .
Vv12g0539          .        .        .        .        .        .        .
          .        .        .        .        .        .        .        .
          .        .        .        .        .
Vv12g0540          .        .        .        .        .        .        .
          .        .        .        .        .        .        .        .
          .        .        .        .        .
Vv12g0541          .        .        .        .        .        .        .
          .        .        .        .        .        .        .        .
          .        .        .        .        .
Vv12g0542          .        .        .        .        .        .        .
          .        .        .        .        .        .        .        .
          .        .        .        .        .
```

Vv12g0543 . . . . . . .
. . . . . . . .
. . . . .
Vv12g0544 . Gorai.007G169000.1 . . .
. . . . . . . .
. . . . . . .
Vv12g0545 . Gorai.007G169100.1 . . .
. Vv19g0704 Tc07_g002460 Gorai.013G157300.1
Gorai.007G169100.1 . . . . .
. . . . .
Vv12g0546 . . . . . Gorai.
001G097300.1 . . . . . . .
. . . . . . .
Vv12g0547 Tc01_g018300 Gorai.007G169200.1 . .
. . . . . . . .
. . . . . . . .
Vv12g0548 Tc01_g018010 Gorai.007G169300.1 Gorai.
001G218300.1 . . Gorai.001G097000.1 . .
. . . . . . . .
. . . .
Vv12g0549 . . . . . . .
. . . . . . . .
. . . . .
Vv12g0550 Tc01_g018000 . Gorai.001G218200.1 .
. . . . . . . .
. . . . . . . .
Vv12g0551 Tc01_g017990 . . . . .
Vv19g0692 . . . . . .
. . . . . . .
Vv12g0552 Tc01_g017980 . Gorai.001G218100.1 .
. . . . . . . .
. . . . . . . .
Vv12g0553 Tc01_g017960 Gorai.007G169400.1 Gorai.
001G218000.1 . . . . . . .
. . . . . . . .
. .
Vv12g0554 . . . . . . .
. . . . . . . .
. . . . .
Vv12g0555 Tc01_g017870 . Gorai.001G217800.1 .
. . . . . . . .
. . . . . . . .
Vv12g0556 Tc01_g017860 Gorai.007G169500.1 . .
. . . . . . . .
. . . . . . . .
Vv12g0557 Tc01_g017850 . . Gorai.001G195000.1
. . . . . . . .
. . . . . . . .
Vv12g0558 Tc01_g017830 Gorai.007G169700.1 .
Gorai.001G195100.1 . . . . . .

. . . . . . . .
. .
Vv12g0559 . . Gorai.001G217700.1 Gorai.
001G195200.1 . . . . . . .
. . . . . . . .
.
Vv12g0560 . . . . . . .
. . . . . . . .
. . . . .
Vv12g0561 . . . . . . .
. . . . . . . .
. . . . .
Vv12g0562 . . . . . . .
. . . . . . . .
. . . . .
Vv12g0563 Tc01_g017800 Gorai.007G169900.1 .
Gorai.001G195400.1 . . . . . .
. . . . . . . .
. .
Vv12g0564 . . . . . . .
. . . . . . . .
. . . . .
Vv12g0565 . . . . . . .
. . . . . . . .
. . . . .
Vv12g0566 Tc01_g017790 . . . . .
. . . . . . . .
. . . . . .
Vv12g0567 Tc01_g017780 . . Gorai.001G195500.1
. . . . . . . .
. . . . . . . .
Vv12g0568 Tc01_g030980 . . . . .
. . . . . . . .
. . . . . .
Vv12g0569 Tc01_g017770 . . Gorai.001G195600.1
. Gorai.001G096900.1 . . . .
. . . . . . . .
. .
Vv12g0570 Tc01_g017760 . . . . .
. . . . . . . .
. . . . . .
Vv12g0571 . . . Gorai.001G195700.1 .
. . . . . . . .
. . . . . . .
Vv12g0572 Tc01_g017730 Gorai.007G170000.1 . .
. . . . . . . .
. . . . . . . .
Vv12g0573 . . . . . . .
. . . . . . . .
. . . . .

Vv12g0574 Tc01_g017720 . Gorai.001G217500.1
Gorai.001G195900.1 . Gorai.001G096500.1 . .
. . . . . . . .
. . . .
Vv12g0575 Tc01_g017710 Gorai.007G170100.1 Gorai.
001G217400.1 . . Gorai.001G096400.1 . .
. . . . . . . .
. . . .
Vv12g0576 Tc01_g017690 Gorai.007G170200.1 . .
. . . . . . . .
. . . . . . . .
Vv12g0577 Tc01_g017680 . . . . .
. . . . . . . .
. . . . . .
Vv12g0578 . . . . . . .
. . . . . . . .
. . . . .
Vv12g0579 Tc01_g017670 Gorai.007G170300.1 Gorai.
001G217000.1 . . . . . . .
. . . . . . . .
. .
Vv12g0580 Tc01_g017660 Gorai.007G170400.1 . .
. . . . . . . .
. . . . . . . .
Vv12g0581 Tc01_g017650 . Gorai.001G216900.1 .
. . Vv19g0685 Tc07_g002650 . .
Gorai.002G092600.1 Gorai.009G442700.1 . .
. . . . . .
Vv12g0582 Tc01_g017640 . Gorai.001G216800.1 .
. . . . . . . .
. . . . . . . .
Vv12g0583 Tc01_g017630 . . Gorai.001G196200.1
. . . . . . . .
. . . . . . . .
Vv12g0584 Tc01_g017600 . Gorai.001G216700.1 .
. . . . . . . .
. . . . . . . .
Vv12g0585 . . . . . . .
. . . . . . . .
. . . . .
Vv12g0586 Tc01_g017590 Gorai.007G170500.1 . .
. . . . . . . .
. . . . . . . .
Vv12g0587 . . Gorai.001G216600.1 . .
. . . . . . . .
. . . . . . .
Vv12g0588 Tc01_g003440 . . . . .
. . . . . . . .
. . . . . .
Vv12g0589 Tc01_g030880 Gorai.007G170600.1 . .

```
        .        Gorai.001G095700.1       Vv19g0683
Tc07_g002680     .       Gorai.007G170600.1       Gorai.002G092400.1
        .        .       .        .       .        .       .        .
        .
Vv12g0590        .       .        .       .        .       .        .
        .        .       .        .       .        .       .        .
        .        .       .        .       .
Vv12g0591        .       .        .       .        .       .        .
        .        .       .        .       .        .       .        .
        .        .       .        .       .
Vv12g0592        .       Gorai.007G170900.1        .       .        .
        .        .       .        .       .        .       .        .
        .        .       .        .       .        .       .
Vv12g0593        .       .        .       .        .       .        .
        .        .       .        .       .        .       .        .
        .        .       .        .       .
Vv12g0594        Tc01_g003360     .       .        .       .        .
        .        .       .        .       .        .       .        .
        .        .       .        .       .        .
Vv12g0595        .       .        .       .        .       .        .
        .        .       .        .       .        .       .        .
        .        .       .        .       .
Vv12g0596        .       .        .       .        .       .        .
        .        .       .        .       .        .       .        .
        .        .       .        .       .
Vv12g0597        .       .        .       .        .       .        .
        .        .       .        .       .        .       .        .
        .        .       .        .       .
Vv12g0598        .       .        .       .        .       .        .
        .        .       .        .       .        .       .        .
        .        .       .        .       .
Vv12g0599        .       Gorai.007G171000.1       Gorai.001G216400.1
        .        .       .        .       .        .       .        .
        .        .       .        .       .        .       .        .
        .
Vv12g0600        .       .        Gorai.001G216300.1       .        .
        .        .       .        .       .        .       .        .
        .        .       .        .       .        .       .
Vv12g0601        .       .        Gorai.001G216200.1       .        .
        .        .       .        .       .        .       .        .
        .        .       .        .       .        .       .
Vv12g0602        .       Gorai.007G171100.1        .       .        .
        .        .       .        .       .        .       .        .
        .        .       .        .       .        .       .
Vv12g0603        .       .        .       .        .       .        .
        .        .       .        .       .        .       .        .
        .        .       .        .       .
Vv12g0604        .       Gorai.007G171400.1       Gorai.001G215700.1
        .        .       .        .       .        .       .        .
        .        .       .        .       .        .       .        .
```

.
Vv12g0605 . . Gorai.001G215600.1 . .
. . . . . . . .
. . . . . . .
Vv12g0606 . Gorai.007G171500.1 Gorai.001G215500.1
. . . . . . . .
. . Vv010rg0095 . . . .
. .
Vv12g0607 . . . Gorai.001G197700.1 .
. Vv19g0678 Tc07_g002770 Gorai.013G158700.1
. . . . . . . .
. . .
Vv12g0608 . . . . . Gorai.
001G094600.1 Vv19g0677 Tc07_g002780 . . .
. . . . . . . .
.
Vv12g0609 Tc01_g003310 Gorai.007G171600.1 . .
. . . . . . . .
. . . . . . . .
Vv12g0610 . . . . . . .
. . . . . . . .
. . . . .
Vv12g0611 . . . . . . .
. . . . . . . .
. . . . .
Vv12g0612 . . . . . Gorai.
001G094400.1 . . . . . . .
. . . . . . .
Vv12g0613 . . Gorai.001G215000.1 . .
. Vv19g0674 . . . . .
. . . . . . . .
Vv12g0614 . . . . . . .
. . . . . . . .
. . . . .
Vv12g0615 . . . . . . .
. . . . . . . .
. . . . .
Vv12g0616 . . . . . . .
. . . . . . . .
. . . . .
Vv12g0617 . . . . . . .
. . . . . . . .
. . . . .
Vv12g0618 Tc01_g025770 . . . . .
. . . . . . . .
. . . . . .
Vv12g0619 . . . . . . .
. . . . . . . .
. . . . .
Vv12g0620 . . . . . . .

. . . . . . . .
. . . . .
Vv12g0621 Tc01_g017560 . . . . .
. . . . . . . .
. . . . . .
Vv12g0622 Tc01_g017540 . . . . .
. . . . . . . .
. . . . . .
Vv12g0623 . . . . . . .
. . . . . . . .
. . . . .
Vv12g0624 Tc01_g017530 . . . . .
. . . . . . . .
. . . . . .
Vv12g0625 Tc01_g003020 . . . . .
. . . . . . . .
. . . . . .
Vv12g0626 Tc01_g017490 Gorai.007G171800.1 . .
. . . . . . . .
. . . . . . . .
Vv12g0627 Tc01_g002880 . . . . .
. . . . . . . .
. . . . . .
Vv12g0628 Tc01_g017460 . . Gorai.001G197600.1
. . . . . . . .
. . . . . . . .
Vv12g0629 Tc01_g017430 Gorai.007G171900.1 Gorai.
001G213700.1 . . Gorai.001G094300.1 Vv19g0667
Tc07_g002910 . Gorai.007G171900.1 Gorai.
002G091300.1 . . Vv010rg0082 . . .
. . .
Vv12g0630 . . . . . . .
. . . . . . . .
. . . . .
Vv12g0631 . . . . . . .
. . . . . . . .
. . . . .
Vv12g0632 . . . . . . .
. . . . . . Vv010rg0077
. . . . . .
Vv12g0633 . . . . . . .
. . . . . . . .
. . . . .
Vv12g0634 . . . . . Gorai.
001G092000.1 . . . . . . .
Vv010rg0076 . . . . . .
Vv12g0635 . . . . . . .
. . . . . . . .
. . . . .
Vv12g0636 . . . . . . .

```
        .        .        .        .        .        .        .        .
        .        .        .        .        .
Vv12g0637        .        .        .        .        .        .        .
        .        .        .        .        .        .        .        .
        .        .        .        .        .
Vv12g0638        .        .        .        .        .        .        .
        .        .        .        .        .        .        .        .
        .        .        .        .        .
Vv12g0639        .        .        .        .        .        .        .
        .        .        .        .        .        .       Vv010rg0050
        .        .        .        .        .        .
Vv12g0640        .        .        .        .        .        .        .
        .        .        .        .        .        .        .        .
        .        .        .        .        .
Vv12g0641        .        .        .        .        .        .        .
        .        .        .        .        .        .        .        .
        .        .        .        .        .
Vv12g0642        .        .        .        .        .        .        .
        .        .        .        .        .        .        .        .
        .        .        .        .        .
Vv12g0643        .        .        .        .        .        .        .
        .        .        .        .        .        .        .        .
        .        .        .        .        .
Vv12g0644        .        .        .        .        .        .        .
        .        .        .        .        .        .        .        .
        .        .        .        .        .
Vv12g0645        .        .        .        .        .        .        .
        .        .        .        .        .        .        .        .
        .        .        .        .        .
Vv12g0646        .        .        .        .        .        .        .
        .        .        .        .        .        .        .        .
        .        .        .        .        .
Vv12g0647        .        .        .        .        .        .        .
        .        .        .        .        .        .        .        .
        .        .        .        .        .
Vv12g0648        .        .        .        .        .        .        .
        .        .        .        .        .        .        .        .
        .        .        .        .        .
Vv12g0649        .        .        .        .        .        .
Vv19g0645        .        .        .        .        .        .
Vv010rg0046      .        .        .        .        .        .
Vv12g0650        .        .        .        .        .        .
Vv19g0642        Tc07_g003130      .        .        Gorai.002G089700.1
        Gorai.009G440200.1         .        Vv010rg0044       .        .
        .        .        .        .
Vv12g0651        .        .        .        .        .        .        .
        .        .        .        .        .        .        .        .
        .        .        .        .        .
Vv12g0652        .        .        .        .        .        .        .
        .        .        .        .        .        .        .        .
```

| | | | | | | | |
|---|---|---|---|---|---|---|---|
| . | . | . | . | . | | | |
| Vv12g0653 | . | . | . | . | . | . | . |
| . | . | . | . | . | . | . | . |
| . | . | . | . | . | | | |
| Vv12g0654 | . | . | . | . | . | . | . |
| . | . | . | . | . | . | . | . |
| . | . | . | . | . | | | |
| Vv12g0655 | . | . | . | . | . | . | . |
| . | . | . | . | . | . | . | . |
| . | . | . | . | . | | | |
| Vv12g0656 | . | . | . | . | . | . | . |
| . | . | . | . | . | . | . | . |
| . | . | . | . | . | | | |
| Vv12g0657 | Tc01_g025640 | | . | . | . | . | . |
| . | . | . | . | . | . | . | . |
| . | . | . | . | . | . | | |
| Vv12g0658 | . | . | . | . | . | . | . |
| . | . | . | . | . | . | . | . |
| . | . | . | . | . | | | |
| Vv12g0659 | . | . | . | . | . | . | |
| Vv010rg0022 | . | . | . | . | . | . | . |
| . | . | . | . | . | . | | |
| Vv12g0660 | . | . | . | . | . | . | . |
| . | . | . | . | . | . | . | . |
| . | . | . | . | . | | | |
| Vv12g0661 | . | . | . | . | . | . | . |
| . | . | . | . | . | . | . | . |
| . | . | . | . | . | | | |
| Vv12g0662 | . | . | . | . | . | . | . |
| . | . | . | . | . | . | . | . |
| . | . | . | . | . | | | |
| Vv12g0663 | . | . | . | . | . | . | . |
| . | . | . | . | . | . | . | . |
| . | . | . | . | . | | | |
| Vv12g0664 | . | . | . | . | . | . | . |
| . | . | . | . | . | . | . | . |
| . | . | . | . | . | | | |
| Vv12g0665 | . | . | . | . | . | . | . |
| . | . | . | . | . | . | . | . |
| . | . | . | . | . | | | |
| Vv12g0666 | . | . | . | . | . | . | . |
| . | . | . | . | . | . | . | . |
| . | . | . | . | . | | | |
| Vv12g0667 | . | . | . | . | . | . | . |
| . | . | . | . | . | . | . | . |
| . | . | . | . | . | | | |
| Vv12g0668 | . | . | . | . | . | . | . |
| . | . | . | . | . | . | . | . |
| . | . | . | . | . | | | |
| Vv12g0669 | . | . | . | . | . | . | . |

```
. . . . . . . .
. . . . .
Vv12g0670 . . . . . . .
. . . . . . . .
. . . . .
Vv12g0671 . . . . . . .
. . . . . . . .
. . . . .
Vv12g0672 . . . . . . .
. . . . . . . .
. . . . .
Vv12g0673 . . . . . . .
. . . . . . . .
. . . . .
Vv12g0674 . . . Gorai.001G194900.1 .
. . . . . . . .
. . . . . . .
Vv12g0675 . . . Gorai.001G194800.1 .
. . . . . . . .
. . . . . . .
Vv12g0676 . . . Gorai.001G191800.1 .
. . . . . . . .
. . . . . . .
Vv12g0677 . . . . . . .
. . . . . . . .
. . . . .
Vv12g0678 . . . . . . .
. . . . . . . .
. . . . .
Vv12g0679 . . . . . . .
. . . . . . . .
. . . . .
Vv12g0680 . . . . . . .
. . . . . . . .
. . . . .
Vv12g0681 . . . . . . .
. . . . . . . .
. . . . .
Vv12g0682 . . . . . . .
. . . . . . . .
. . . . .
Vv12g0683 . . . . . . .
. . . . . . . .
. . . . .
Vv12g0684 . . . . . . .
. . . . . . . .
. . . . .
Vv12g0685 . . . . . . .
. . . . . . . .
. . . . .
```

Vv12g0686 . . . . . . .
. . . . . . . .
. . . . .
Vv12g0687 . . . . . . .
. . . . . . . .
. . . . .
Vv12g0688 . . . . . . .
. . . . . . . .
. . . . .
Vv12g0689 . . . . . . .
. . . . . . . .
. . . . .
Vv12g0690 . . . . . . .
. . . . . . . .
. . . . .
Vv12g0691 Tc01_g029130 . . . . .
. . . . . . . .
. . . . . .
Vv12g0692 . . . . . . .
. . . . . . . .
. . . . .
Vv12g0693 . . . . . . .
. . . . . . . .
. . . . .
Vv12g0694 . . . . . . .
. . . . . . . .
. . . . .
Vv12g0695 . . . . . . .
. . . . . . . .
. . . . .
Vv12g0696 . . . . . . .
. . . . . . . .
. . . . .
Vv12g0697 . . . . . . .
. . . . . . . .
. . . . .
Vv12g0698 . . . . . . .
. . . . . . . .
. . . . .
Vv12g0699 . . . . Gorai.004G057900.1
. . . . . . . .
. . . . . . .
Vv12g0700 . . . . . . .
. . . . . . . .
. . . . .
Vv12g0701 Tc01_g029240 . . . . .
. . . . . . . .
. . . . . .
Vv12g0702 . . . . . . .
. . . . . . . .

```
        .        .       .        .       .
Vv12g0703        .       .        .       .        .       .        .
        .        .       .        .       .        .       .        .
        .        .       .        .       .
Vv12g0704        .       .        .       .        .       .        .
        .        .       .        .       .        .       .        .
        .        .       .        .       .
Vv12g0705        .       .        .       .        .       .        .
        .        .       .        .       .        .       .        .
        .        .       .        .       .
Vv12g0706        Tc01_g011850     .       .        .       .        .
        .        .       .        .       .        .       .        .
        .        .       .        .       .        .
Vv12g0707        .       .        .       .        .       .        .
        .        .       .        .       .        .       .        .
        .        .       .        .       .
Vv12g0708        Tc01_g011860     .       .        .       .        .
        .        .       .        .       .        .       .        .
        .        .       .        .       .        .
Vv12g0709        Tc01_g011890     .       .        .       .        .
        .        .       .        .       .        .       .        .
        .        .       .        .       .        .
Vv12g0710        .       .        .       .        .       .        .
        .        .       .        .       .        .       .        .
        .        .       .        .       .
Vv12g0711        .       .        .       .        .       .        .
        .        .       .        .       .        .       .        .
        .        .       .        .       .
Vv12g0712        .       .        .       .        .       .        .
        .        .       .        .       .        .       .        .
        .        .       .        .       .
Vv12g0713        .       .        .       .        .       .        .
        .        .       .        .       .        .       .        .
        .        .       .        .       .
Vv12g0714        .       .        .       .        .       .        .
        .        .       .        .       .        .       .        .
        .        .       .        .       .
Vv12g0715        .       .        .       .        .       .        .
        .        .       .        .       .        .       .        .
        .        .       .        .       .
Vv12g0716        .       .        .       .        .       .        .
        .        .       .        .       .        .       .        .
        .        .       .        .       .
Vv12g0717        .       .        .       .        .       .        .
        .        .       .        .       .        .       .        .
        .        .       .        .       .
Vv12g0718        .       .        .       .        .       .        .
        .        .       .        .       .        .       .        .
        .        .       .        .       .
Vv12g0719        .       .        .       .        .       .        .
```

. . . . . . . .
. . . . .
Vv12g0720 . . . . . . .
. . . . . . . .
. . . . .
Vv12g0721 . . . . . . .
. . . . . . . .
. . . . .
Vv12g0722 . . . . . . .
. . . . . . . .
. . . . .
Vv12g0723 . . . . . . .
. . . . . . . .
. . . . .
Vv12g0724 . . . . . . .
. . . . . . . .
. . . . .
Vv12g0725 . . . . . . .
. . . . . . . .
. . . . .
Vv12g0726 . . . . . . .
. . . . . . . .
. . . . .
Vv12g0727 . . . . . . .
. . . . . . . .
. . . . .
Vv12g0728 . . . . . . .
. . . . . . . .
. . . . .
Vv12g0729 . . . . . . .
. . . . . . . .
. . . . .
Vv12g0730 . . . . . . .
. . . . . . . .
. . . . .
Vv12g0731 . . . . . . .
. . . . . . . .
. . . . .
Vv12g0732 . . . . . . .
. . . . . . . .
. . . . .
Vv12g0733 . . . . . . .
. . . . . . . .
. . . . .
Vv12g0734 . . . . . . .
. . . . . . . .
. . . . .
Vv12g0735 . . . . . . .
. . . . . . . .
. . . . .

Vv12g0736 . . . . . . .
. . . . . . . .
. . . . .
Vv12g0737 . . . . . . .
. . . . . . . .
. . . . .
Vv12g0738 . . . . . . .
. . . . . . . .
. . . . .
Vv12g0739 Tc01_g019650 Gorai.007G163100.1 . .
. . . . . . . .
. . . . . . . .
Vv12g0740 . Gorai.007G163300.1 Gorai.001G225600.1
. . Gorai.001G100100.1 Vv01g0681
Tc02_g007920 Gorai.002G170500.1 . Gorai.009G329300.1
. . . . . . . .
.
Vv12g0741 . . . . . . .
. . . . . . . .
. . . . .
Vv12g0742 . . Gorai.001G225300.1 . .
. . . . . . . .
. . . . . . .
Vv12g0743 . . . . Gorai.004G060600.1
. . . . . . . .
. . . . . . .
Vv12g0744 . . . . . .
Vv19g0905 . . . . . . .
. . . . . .
Vv12g0745 . . . . . . .
. . . . . . . .
. . . . .
Vv12g0746 . Gorai.007G163700.1 Gorai.001G224500.1
. . . . . . . .
. . . . . . . .
.
Vv12g0747 Tc01_g011970 . . . . .
. . . . . . . .
. . . . . .
Vv12g0748 . . . . . . .
. . . . . . . .
. . . . .
Vv12g0749 . . . . . . .
. . . . . . . .
. . . . .
Vv12g0750 . . . . . . .
. . . . . . . .
. . . . .
Vv12g0751 . . . . . . .
. . . . . . . .

. . . . .
Vv12g0752 . . . . . . .
. . . . . . . .
. . . . .
Vv12g0753 . . . Gorai.001G189500.1 .
. . . . . . . .
. . . . . . .
Vv12g0754 . Gorai.007G164400.1 . Gorai.
001G189600.1 . . . . . . .
. . Vv01g0656 . . Gorai.
005G153900.1 . . .
Vv12g0755 . . . . . . .
. . . . . . . .
. . . . .
Vv12g0756 . . Gorai.001G224100.1 . .
. . . . . . . .
. . . . . . .
Vv12g0757 . . Gorai.001G224000.1 Gorai.
001G189700.1 Gorai.004G060700.1 . Vv19g0884
Tc07_g000630 . . . Gorai.009G452100.1 .
. . . . . . .
Vv12g0758 . . . Gorai.001G189900.1 .
. Vv19g0882 Tc07_g000650 . .
Gorai.002G103200.1 Gorai.009G451900.1 . . .
. . . . .
Vv12g0759 Tc01_g019330 . Gorai.001G223900.1 .
. . . . . . . .
. . . . . . . .
Vv12g0760 Tc01_g019310 Gorai.007G164500.1 . .
Gorai.004G060900.1 . . . . .
. . . . . . . .
. .
Vv12g0761 Tc01_g019300 Gorai.007G164600.1 Gorai.
001G223800.1 Gorai.001G190000.1 Gorai.004G061000.1
Gorai.001G099700.1 . . . . . .
. . . . . . . .
Vv12g0762 Tc01_g019290 Gorai.007G164700.1 .
Gorai.001G190100.1 . . . . . .
. . . . . . . .
. .
Vv12g0763 Tc01_g019270 Gorai.007G165000.1 Gorai.
001G223700.1 Gorai.001G190300.1 . Gorai.001G099600.1
. . . . . . . .
. . . . . .
Vv12g0764 Tc01_g019260 Gorai.007G165100.1 . .
. . . . . . . .
. . . . . . . .
Vv12g0765 Tc01_g019250 . Gorai.001G223300.1
Gorai.001G190400.1 . Gorai.001G099500.1 . .
. . . . . . . .

.       .       .       .
Vv12g0766       Tc01_g019240    .       .       Gorai.001G190700.1
.       .       .       .       .       .       .       .
.       .       .       .       .       .       .       .
Vv12g0767       Tc01_g019220    Gorai.007G165200.1      .       .
.       Gorai.001G098400.1      Vv19g0863
Tc07_g000790    Gorai.013G150900.1      Gorai.007G165200.1
Gorai.002G102300.1      .       .       .       .       .       .
.       .       .
Vv12g0768       Tc01_g041020    Gorai.007G167500.1      .
Gorai.001G190800.1      Gorai.004G061200.1      Gorai.001G098300.1
.       .       .       .       .       .       .       .
.       .       .       .       .       .
Vv12g0769       .       .       .       .       .       .       .
.       .       .       .       .       .       .       .
.       .       .       .       .
Vv12g0770       .       .       .       .       .       Gorai.
001G095400.1    .       .       .       .       .       .       .
.       .       .       .       .       .       .
Vv12g0771       .       .       Gorai.001G223100.1      .       .
.       .       .       .       .       .       .       .
.       .       .       .       .       .       .
Vv12g0772       Tc01_g019180    .       .       Gorai.001G190900.1
Gorai.004G061300.1      .       .       .       .       .
.       .       .       .       .       .       .       .
.       .
Vv12g0773       Tc01_g019170    Gorai.007G165300.1      .
Gorai.001G191000.1      .       .       .       .       .       .
.       .       .       .       .       .       .       .
.       .
Vv12g0774       Tc01_g019150    .       Gorai.001G223000.1      .
.       .       Vv19g0836       Tc07_g001020    Gorai.
013G151700.1    .       Gorai.002G101200.1      .       .       .
.       .       .       .       .       .
Vv12g0775       Tc01_g019140    Gorai.007G165500.1      .       .
.       .       Vv19g0835       Tc07_g001030    Gorai.
013G151900.1    Gorai.007G310200.1      Gorai.002G101100.1      .
.       .       .       .       .       .       .       .
Vv12g0776       Tc01_g019130    Gorai.007G165600.1      .       .
.       .       .       .       .       .       .       .
.       .       .       .       .       .       .       .
Vv12g0777       .       .       .       .       .       .       .
.       .       .       .       .       .       .       .
.       .       .       .       .
Vv12g0778       Tc01_g016800    .       .       .       .       .
.       .       .       .       .       .       .       .
.       .       .       .       .       .
Vv12g0779       Tc01_g019120    .       .       .       .       .
.       .       .       .       .       .       .       .
.       .       .       .       .       .

Vv12g0780 . . . Gorai.001G191200.1 .
. . . . . . . .
. . . . . . .
Vv12g0781 . . . . . . .
. . . . . . . .
. . . . .
Vv12g0782 . . . . . . .
. . . . . . . .
. . . . .
Vv12g0783 . . . . . . .
. . . . . . . .
. . . . .
Vv12g0784 Tc01_g019110 . . . . .
. . . . . . . .
. . . . . .
Vv12g0785 . . . . . . .
. . . . . . . .
. . . . .
Vv12g0786 . Gorai.007G165700.1 Gorai.001G222500.1
. . . . . . . .
. . . . . . . .
.
Vv12g0787 Tc01_g019040 . . . . .
. . . . . . . .
. . . . . .
Vv12g0788 . . . . . .
Vv19g0818 Tc07_g001220 Gorai.013G152800.1 Gorai.
007G165700.1 Gorai.002G100400.1 . . . .
. . . . .
Vv12g0789 Tc01_g019030 . . . . .
. . . . . . . .
. . . . . .
Vv12g0790 Tc01_g001370 . . . . .
. . . . . . .
Vv01g0617 . . . . . .
Vv12g0791 Tc01_g001380 . . . . .
. . . . . . .
Vv01g0616 . Gorai.013G029300.1 . . .
.
Vv12g0792 . . . . . . .
. . . . . . . .
. . . . .
Vv12g0793 . . . . . . .
. . . . . . . .
. . . . .
Vv12g0794 . Gorai.007G166500.1 . . .
. . . . . . . .
. . . . . . .
Vv12g0795 Tc01_g019010 Gorai.007G165800.1 Gorai.
001G222400.1 . . . Vv19g0817

Tc07_g001240 Gorai.013G152900.1 Gorai.007G165800.1
Gorai.002G100100.1 . . . . . .
. . .
Vv12g0796 Tc01_g018980 Gorai.007G166100.1 Gorai.
001G222300.1 . . . Vv19g0809
Tc07_g001290 Gorai.013G153100.1 . Gorai.002G099900.1
. . . . . . . .
.
Vv12g0797 Tc01_g018950 . Gorai.001G222200.1 .
. . . . . . . .
. . . . . . . .
Vv12g0798 Tc01_g018900 . Gorai.001G222000.1 .
. . . . . . . .
. . . . . . . .
Vv12g0799 Tc01_g018880 . Gorai.001G221800.1 .
. . . . . . . .
. . . . . . . .
Vv12g0800 Tc01_g018870 Gorai.007G166200.1 Gorai.
001G221700.1 . . . . . . .
. . . . . . . .
. .
Vv12g0801 Tc01_g018860 Gorai.007G166300.1 . .
. . . . . . . .
. . . . . . . .
Vv12g0802 Tc01_g018850 Gorai.007G166400.1 . .
. . . . . . . .
. . . . . . . .
Vv12g0803 . . . . . . .
. . . . . . . .
. . . . .
Vv12g0804 . . . . . . .
. . . . . . . .
. . . . .
Vv12g0805 Tc01_g018820 Gorai.007G166500.1 . .
. . . . . . . .
. . . . . . . .
Vv12g0806 Tc01_g040790 . . . . .
. . . . . . . .
. . . . . .
Vv12g0807 . . . . . . .
. . . . . . . .
. . . . .
Vv12g0808 Tc01_g018760 . Gorai.001G221400.1 .
. . . . . . . .
. . . . . . . .
Vv12g0809 . . . . . .
Vv19g0797 Tc07_g001430 . . Gorai.002G099000.1
Gorai.009G448800.1 Gorai.004G033300.1 . .
. . . . .
Vv12g0810 Tc01_g016500 . . . . .

. . . . . . . .
. . . . . .
Vv12g0811 . . . . . . .
. . . . . . . .
. . . . .
Vv12g0812 . . . . . . .
. . . . . . . .
. . . . .
Vv12g0813 . . . . . . .
. . . . . . . .
. . . . .
Vv12g0814 . . . . . . .
. . . . . . . .
. . . . .
Vv12g0815 . . . . . . .
. . . . . . . .
. . . . .
Vv12g0816 . . . . . . .
. . . . . . . .
. . . . .
Vv12g0817 . . . . . . .
. . . . . . . .
. . . . .
Vv12g0818 . . . . . . .
. . . . . . . .
. . . . .
Vv12g0819 . . . . . . .
. . . . . . . .
. . . . .
Vv12g0820 . . . . . . .
. . . . . . . .
. . . . .
Vv12g0821 . . . . . . .
. . . . . . . .
. . . . .
Vv12g0822 . . . . . . .
. . . . . . . .
. . . . .
Vv12g0823 . . . . . . .
. . . . . . . .
. . . . .
Vv12g0824 . . . . . . .
. . . . . . . .
. . . . .
Vv12g0825 . . . . . . .
. . . . . . . .
. . . . .
Vv12g0826 . . . . . . .
. . . . . . . .
. . . . .

Vv12g0827 Tc01_g018670 . . . . .
. . . . . . . .
. . . . . .
Vv12g0828 Tc01_g018650 . . Gorai.001G191700.1
. . . . . . . .
. . . . . . . .
Vv12g0829 . . . . . . .
. . . . . . Vv01g0579
Tc02_g028070 Gorai.002G174100.1 Gorai.005G159100.1
. . .
Vv12g0830 Tc01_g001470 . . . . .
. . . . . . . .
. . . . . .
Vv12g0831 . . . . . Gorai.
001G099400.1 . . . . . . .
. . . . . . .
Vv12g0832 . . . . . . .
. . . . . . . .
. . . . .
Vv12g0833 . . . . . . .
. . . . . . . .
. . . . .
Vv12g0834 . . . . . . .
. . . . . . . .
. . . . .
Vv12g0835 Tc01_g018560 . . . . .
. . . . . . . .
. . . . . .
Vv12g0836 . Gorai.007G163200.1 . Gorai.
001G192000.1 . . . . . . .
. . . . . . . .
.
Vv12g0837 . . . . . . .
. . . . . . . .
. . . . .
Vv12g0838 . . Gorai.001G221100.1 . .
. . . . . . . .
. . . . . . .
Vv12g0839 . . . . . Gorai.
001G099100.1 . . . . . . .
. . . . . . .
Vv12g0840 . Gorai.007G166700.1 . . .
. . . . . . . .
. . . . . . .
Vv12g0841 . . . . . .
Vv19g0757 Tc07_g001980 . Gorai.007G166600.1
Gorai.002G096600.1 Gorai.009G446200.1 . . .
. . . . .
Vv12g0842 Tc01_g018470 Gorai.007G167000.1 . .
. . Vv19g0749 Tc07_g002000 . .

Gorai.002G096300.1 Gorai.009G446100.1 .
Vv01g0547 Tc02_g028520 Gorai.002G177000.1 . .
. .
Vv12g0843 Tc01_g018460 Gorai.007G167100.1 . .
. . . . . . . .
. . . . . . . .
Vv12g0844 . . . . . . .
. . . . . . . .
. . . . .
Vv12g0845 . Gorai.007G167200.1 . . .
. . . . . . . .
. . . . . . .
Vv12g0846 Tc01_g034170 Gorai.007G167300.1 . .
. . . . . . . .
. . . . . . . .
Vv12g0847 . . Gorai.001G220400.1 . .
. . . . . . . .
. . . . . . .
Vv12g0848 . Gorai.007G167400.1 . . .
. . . . . . . .
. . . . . . .
Vv12g0849 Tc01_g016320 Gorai.007G167500.1 Gorai.
001G220100.1 Gorai.001G193100.1 . Gorai.001G098600.1
. . . . . . . .
. . . . . .
Vv12g0850 . . . . . .
Vv19g0741 Tc07_g002070 Gorai.013G156200.1 .
Gorai.002G095700.1 Gorai.009G445900.1 . . .
. . . . .
Vv12g0851 . . . . . . .
. . . . . . . .
. . . . .
Vv12g0852 . . Gorai.001G217500.1 Gorai.
001G193200.1 . . . . . . .
. . . . . . . .
.
Vv12g0853 Tc01_g034450 Gorai.007G162400.1 Gorai.
008G125300.1 Gorai.001G193600.1 . . . .
. . . . . . . .
. . . .
Vv12g0854 . . . . . .
Vv19g0734 . . . . . . .
. . . . . .
Vv12g0855 . . . . . . .
. . . . . . . .
. . . . .
Vv12g0856 . . . . . . .
. . . . . . . .
. . . . .
Vv12g0857 . . . . . . .

. . . . . . . .
. . . . .
Vv12g0858 . . . . . . .
. . . . . . . .
. . . . .
Vv12g0859 . . . Gorai.001G193700.1 .
. . . . . . . .
Vv0Unrg9182 . . . . . .
Vv12g0860 . . . . . .
Vv19g0730 Tc07_g002200 . . . . .
. . . . . . .
Vv12g0861 . . . . . .
Vv0Unrg9198 . . . . . . .
. . . . . .
Vv12g0862 . . . . . . .
. . . . . . . .
. . . . .
Vv12g0863 . . . . . . .
. . . . . . . .
. . . . .
Vv12g0864 . Gorai.007G159700.1 . Gorai.
001G193800.1 . Gorai.001G098100.1 . . .
. . . . . . . .
. . .
Vv12g0865 . . . . . .
Vv0Unrg9199 . . . . . . .
. . . . . .
Vv12g0866 . . . . . Gorai.
001G095100.1 . . . . . . .
. . . . . . .
Vv12g0867 . . . . . . .
. . . . . . . .
. . . . .
Vv12g0868 . . . . . .
Vv0Unrg9200 . . . . . . .
. . . . . .
Vv12g0869 . . . . . .
Vv0Unrg9201 . . . . . . .
. . . . . .
Vv12g0870 . . . . . . .
. . . . . . . .
. . . . .
Vv12g0871 . . . . . . .
. . . . . . . .
. . . . .
Vv12g0872 . . . . . .
Vv01g0509 . . . . . . .
. . . . . .
Vv12g0873 . . . . . . .
. . . . . . . .

| | | | | | | | |
|---|---|---|---|---|---|---|---|
| . | . | . | . | . | | | |
| Vv12g0874 | . | . | . | . | . | . | . |
| . | . | . | . | . | . | . | . |
| . | . | . | . | . | | | |
| Vv12g0875 | . | . | . | . | . | . | . |
| . | . | . | . | . | . | . | . |
| . | . | . | . | . | | | |
| Vv12g0876 | . | . | . | . | . | . | . |
| . | . | . | . | . | . | . | . |
| . | . | . | . | . | | | |
| Vv12g0877 | . | . | . | . | . | . | . |
| . | . | . | . | . | . | . | . |
| . | . | . | . | . | | | |
| Vv12g0878 | . | . | . | . | . | . | . |
| . | . | . | . | . | . | . | . |
| . | . | . | . | . | | | |
| Vv12g0879 | . | . | . | . | . | . | . |
| . | . | . | . | . | . | . | . |
| . | . | . | . | . | | | |
| Vv12g0880 | . | . | . | . | . | . | . |
| . | . | . | . | . | . | . | . |
| . | . | . | . | . | | | |
| Vv12g0881 | . | . | . | . | . | . | . |
| . | . | . | . | . | . | . | . |
| . | . | . | . | . | | | |
| Vv12g0882 | . | . | . | . | . | . | . |
| . | . | . | . | . | . | . | . |
| . | . | . | . | . | | | |
| Vv12g0883 | . | . | . | . | . | . | . |
| . | . | . | . | . | . | . | . |
| . | . | . | . | . | | | |
| Vv12g0884 | . | . | . | . | . | . | . |
| . | . | . | . | . | . | . | . |
| . | . | . | . | . | | | |
| Vv12g0885 | . | . | . | . | . | . | . |
| . | . | . | . | . | . | . | . |
| . | . | . | . | . | | | |
| Vv12g0886 | . | . | . | . | . | . | . |
| . | . | . | . | . | . | . | . |
| . | . | . | . | . | | | |
| Vv12g0887 | . | . | . | . | . | . | . |
| . | . | . | . | . | . | . | . |
| . | . | . | . | . | | | |
| Vv12g0888 | . | . | . | . | . | . | . |
| . | . | . | . | . | . | . | . |
| . | . | . | . | . | | | |
| Vv12g0889 | . | . | . | . | . | . | . |
| . | . | . | . | . | . | . | . |
| . | . | . | . | . | | | |
| Vv12g0890 | . | . | . | . | . | . | . |

. . . . . . . .
. . . . .
Vv12g0891 . . . . . . .
. . . . . . . .
. . . . .
Vv12g0892 . . . . . . .
. . . . . . . .
. . . . .
Vv12g0893 . . . . . . .
. . . . . . . .
. . . . .
Vv12g0894 . . . . . . .
. . . . . . . .
. . . . .
Vv12g0895 . . . . . . .
. . . . . . . .
. . . . .
Vv12g0896 . . . . . . .
. . . . . . . .
. . . . .
Vv12g0897 . . . . . . .
. . . . . . . .
. . . . .
Vv12g0898 . . . . . . .
. . . . . . . .
. . . . .
Vv12g0899 . . . . . . .
. . . . . . . .
. . . . .
Vv12g0900 . . . . . . .
. . . . . . . .
. . . . .
Vv12g0901 . . . . . .
Vv01g0486 . . . . . . .
. . . . . .
Vv12g0902 . . . . . . .
. . . . . . . .
. . . . .
Vv12g0903 . . . . . . .
. . . . . . . .
. . . . .
Vv12g0904 . . . . . . .
. . . . . . . .
. . . . .
Vv12g0905 . . . . . . .
. . . . . . . .
. . . . .
Vv12g0906 . . . . . . .
. . . . . . . .
. . . . .

Vv12g0907 . . . . . . .
. . . . . . . .
. . . . .
Vv12g0908 Tc01_g005340 . . . . .
. . . . . . . .
. . . . . .
Vv12g0909 Tc01_g005380 . . . . .
. . . . . . . .
. . . . . .
Vv12g0910 . . . . . . .
. . . . . . . .
. . . . .
Vv12g0911 . . . . . . .
. . . . . . . .
. . . . .
Vv12g0912 . . . . . . .
. . . . . . . .
. . . . .
Vv12g0913 Tc01_g008850 . . . . .
. . . . . . . .
. . . . . .
Vv12g0914 . . . . . . .
. . . . . . . .
. . . . .
Vv12g0915 . . . . . . .
. . . . . . . .
. . . . .
Vv12g0916 . . . . . . .
. . . . . . . .
. . . . .
Vv12g0917 . . . . . . .
. . . . . . . .
. . . . .
Vv12g0918 Tc01_g005650 . Gorai.008G078400.1 .
. . . . . . . .
. . . . . . . .
Vv12g0919 . . . . . . .
. . . . . . . .
. . . . .
Vv12g0920 . . . . . . .
. . . . . . . .
. . . . .
Vv12g0921 . . . . . . .
. . . . . . . .
. . . . .
Vv12g0922 . . . . . . .
. . . . . . . .
. . . . .
Vv12g0923 Tc01_g011090 . . . . .
. . . . . . . .

. . . . . .
Vv12g0924 . . . . . . .
. . . . . . . .
. . . . .
Vv12g0925 . . . . . . .
. . . . . . . .
. . . . .
Vv12g0926 . . . . . . .
. . . . . . . .
. . . . .
Vv12g0927 . . . . . . .
. . . . . . . .
. . . . .
Vv12g0928 . . . . . . .
. . . . . . . .
. . . . .
Vv12g0929 . . . . . . .
. . . . . . . .
. . . . .
Vv12g0930 . . . . . . .
. . . . . . . .
. . . . .
Vv12g0931 . . . . . . .
. . . . . . . .
. . . . .
Vv12g0932 . . . . . . .
. . . . . . . .
. . . . .
Vv12g0933 . . . . . . .
. . . . . . . .
. . . . .
Vv12g0934 Tc01_g011070 . . . . .
. . . . . . . .
. . . . . .
Vv12g0935 . . . . . . .
. . . . . . . .
. . . . .
Vv12g0936 . . . . . . .
. . . . . . . .
. . . . .
Vv12g0937 . . . . . . .
. . . . . . . .
. . . . .
Vv12g0938 . . . . . . .
. . . . . . . .
. . . . .
Vv12g0939 . . . . . . .
. . . . . . . .
. . . . .
Vv12g0940 Tc01_g007670 . . . . .

. . . . . . . .
. . . . . .
Vv12g0941 . . . . . . .
. . . . . . . .
. . . . .
Vv12g0942 . . . . . . .
. . . . . . . .
. . . . .
Vv12g0943 . . . . . . .
. . . . . . . .
. . . . .
Vv12g0944 . . . . . . .
. . . . . . . .
. . . . .
Vv12g0945 . . . . . . .
. . . . . . . .
. . . . .
Vv12g0946 . . . . . . .
. . . . . . . .
. . . . .
Vv12g0947 . . . . . . .
. . . . . . . .
. . . . .
Vv12g0948 . . . . . . .
. . . . . . . .
. . . . .
Vv12g0949 . . . . . . .
. . . . . . . .
. . . . .
Vv12g0950 Tc01_g008780 Gorai.007G060500.1 . .
. . . . . . . .
. . . . . . . .
Vv12g0951 Tc01_g005680 . Gorai.008G078600.1 .
Gorai.003G027300.1 . . . . .
. . . . . . . .
. .
Vv12g0952 Tc01_g007620 . . . . .
. . . . . . . .
. . . . . .
Vv12g0953 . . . . . . .
. . . . . . . .
. . . . .
Vv12g0954 . . . . . . .
. . . . . . . .
. . . . .
Vv12g0955 Tc01_g028030 . . . . .
. . . . . . . .
. . . . . .
Vv12g0956 . . . . . . .
. . . . . . . .

| | | | | | | | |
|---|---|---|---|---|---|---|---|
| Vv12g0957 | . | . | . | . | . | . | . |
| Vv12g0958 | . | . | . | . | . | . | . |
| Vv12g0959 | . | . | . | . | . | . | . |
| Vv12g0960 | Tc01_g019660 | | . | . | . | . | . |
| Vv12g0961 | . | . | . | . | . | . | . |
| Vv12g0962 | . | . | . | . | . | . | . |
| Vv12g0963 | . | . | . | . | . | . | . |
| Vv12g0964 | . | . | . | . | . | . | . |
| Vv12g0965 | . | . | . | . | . | . | . |
| Vv12g0966 | . | . | . | . | . | . | . |
| Vv12g0967 | . | . | . | . | . | . | . |
| Vv12g0968 | . | . | . | . | . | . | . |
| Vv12g0969 | . | . | . | . | . | . | . |
| Vv12g0970 | . | . | . | . | . | . | . |
| Vv12g0971 | . | . | . | . | . | . | . |
| Vv12g0972 | . | . | . | . | . | . | . |
| Vv12g0973 | . | . | . | . | . | . | . |

| | | | | | | | |
|---|---|---|---|---|---|---|---|
| . | . | . | . | . | . | . | . |
| . | . | . | . | . | | | |
| Vv12g0974 | . | . | . | . | . | . | . |
| . | . | . | . | . | . | . | . |
| . | . | . | . | . | | | |
| Vv12g0975 | . | . | . | . | . | . | . |
| . | . | . | . | . | . | . | . |
| . | . | . | . | . | | | |
| Vv12g0976 | . | . | . | . | . | . | . |
| . | . | . | . | . | . | . | . |
| . | . | . | . | . | | | |
| Vv12g0977 | Tc01_g008530 | | . | . | . | . | . |
| . | . | . | . | . | . | . | . |
| . | . | . | . | . | . | | |
| Vv12g0978 | . | . | . | . | . | . | . |
| . | . | . | . | . | . | . | . |
| . | . | . | . | . | | | |
| Vv12g0979 | . | . | . | . | . | . | . |
| . | . | . | . | . | . | . | . |
| . | . | . | . | . | | | |
| Vv12g0980 | . | . | . | . | . | . | . |
| . | . | . | . | . | . | . | . |
| . | . | . | . | . | | | |
| Vv12g0981 | . | . | . | . | . | . | . |
| . | . | . | . | . | . | . | . |
| . | . | . | . | . | | | |
| Vv12g0982 | Tc01_g002490 | | Gorai.007G049900.1 | | | . | . |
| . | . | . | . | . | . | . | . |
| . | . | . | . | . | . | . | . |
| Vv12g0983 | Tc01_g018670 | | . | . | . | . | . |
| . | . | . | . | . | . | . | . |
| . | . | . | . | . | . | | |
| Vv12g0984 | . | . | . | . | . | . | . |
| . | . | . | . | . | . | . | . |
| . | . | . | . | . | | | |
| Vv12g0985 | . | . | . | . | . | . | . |
| . | . | . | . | . | . | . | . |
| . | . | . | . | . | | | |
| Vv12g0986 | Tc01_g007500 | | . | Gorai.008G088500.1 | | | . |
| . | . | . | . | . | . | . | . |
| . | . | . | . | . | . | . | . |
| Vv12g0987 | Tc01_g009400 | | . | . | . | . | . |
| . | . | . | . | . | . | . | . |
| . | . | . | . | . | . | | |
| Vv12g0988 | . | . | . | . | . | . | . |
| . | . | . | . | . | . | . | . |
| . | . | . | . | . | | | |
| Vv12g0989 | Tc01_g009520 | | . | . | . | . | . |
| . | . | . | . | . | . | . | . |
| . | . | . | . | . | . | | |

Vv12g0990 Tc01_g008300 . . . . .
. . . . . . . .
. . . . . .
Vv12g0991 . . . . . . .
. . . . . . . .
. . . . .
Vv12g0992 Tc01_g028020 Gorai.007G138500.1 . .
. . . . . . . .
. . . . . . . .
Vv12g0993 Tc01_g007270 . . . . .
. . . . . . . .
. . . . . .
Vv12g0994 . . . . . . .
. . . . . . . .
. . . . .
Vv12g0995 . . . . . . .
. . . . . . . .
. . . . .
Vv12g0996 . . . . . . .
. . . . . . . .
. . . . .
Vv12g0997 . . . . . . .
. . . . . . . .
. . . . .
Vv12g0998 . . . . . . .
. . . . . . . .
. . . . .
Vv12g0999 . . . . . . .
. . . . . . . .
. . . . .
Vv12g1000 Tc01_g020070 . . . . .
. . . . . . . .
. . . . . .
Vv12g1001 . . . . . . .
. . . . . . . .
. . . . .
Vv12g1002 Tc01_g013330 . . . . .
. . . . . . . .
. . . . . .
Vv12g1003 . . . . . . .
. . . . . . . .
. . . . .
Vv12g1004 . . . . . . .
. . . . . . . .
. . . . .
Vv12g1005 . . . . . . .
. . . . . . . .
. . . . .
Vv12g1006 . . . . . . .
. . . . . . . .

. . . . .
Vv12g1007 . . . . . . .
. . . . . . . .
. . . . .
Vv12g1008 . . . . . . .
. . . . . . . .
. . . . .
Vv12g1009 . . . . . . .
. . . . . . . .
. . . . .
Vv12g1010 . . . . . . .
. . . . . . . .
. . . . .
Vv12g1011 . . . . . . .
. . . . . . . .
. . . . .
Vv12g1012 . . . . . . .
. . . . . . . .
. . . . .
Vv12g1013 . . . . . . .
. . . . . . . .
. . . . .
Vv12g1014 Tc01_g019120 . . . . .
. . . . . . . .
. . . . . .
Vv12g1015 . . . . . . .
. . . . . . . .
. . . . .
Vv12g1016 . . . . . . .
. . . . . . . .
. . . . .
Vv12g1017 . . . . . . .
. . . . . . . .
. . . . .
Vv12g1018 . . . . . . .
. . . . . . . .
. . . . .
Vv12g1019 . . . . . . .
. . . . . . . .
. . . . .
Vv12g1020 . . . . . . .
. . . . . . . .
. . . . .
Vv12g1021 Tc01_g014900 . . . . .
. . . . . . . .
. . . . . .
Vv12g1022 Tc01_g002230 Gorai.007G045900.1 Gorai.
008G064800.1 . . . . . . .
. . . . . . . .
. .

Vv12g1023 . . . . . . .
. . . . . . . .
. . . . .
Vv12g1024 . . . . . . .
. . . . . . . .
. . . . .
Vv12g1025 . . . . . . .
. . . . . . . .
. . . . .
Vv12g1026 Tc01_g013100 . . . . .
. . . . . . . .
. . . . . .
Vv12g1027 . . . . . . .
. . . . . . . .
. . . . .
Vv12g1028 . . . . . . .
. . . . . . . .
. . . . .
Vv12g1029 Tc01_g013090 . . . . .
. . . . . . . .
. . . . . .
Vv12g1030 . . . . . . .
. . . . . . . .
. . . . .
Vv12g1031 Tc01_g002030 . . . Gorai.
003G011000.1 . . . . . . .
. . . . . . . .
Vv12g1032 . . . . . . .
. . . . . . . .
. . . . .
Vv12g1033 . . . . . . .
. . . . . . . .
. . . . .
Vv12g1034 . . . . . . .
. . . . . . . .
. . . . .
Vv12g1035 . . . . . . .
. . . . . . . .
. . . . .
Vv12g1036 . . . . . . .
. . . . . . . .
. . . . .
Vv12g1037 . . . . . . .
. . . . . . . .
. . . . .
Vv12g1038 . . . . . . .
. . . . . . . .
. . . . .
Vv12g1039 . . . . . . .
. . . . . . . .

| | | | | | | | |
|---|---|---|---|---|---|---|---|
| . | . | . | . | . | | | |
| Vv12g1040 | . | . | . | . | . | . | . |
| . | . | . | . | . | . | . | . |
| . | . | . | . | . | | | |
| Vv12g1041 | . | . | . | . | . | . | . |
| . | . | . | . | . | . | . | . |
| . | . | . | . | . | | | |
| Vv12g1042 | . | . | . | . | . | . | . |
| . | . | . | . | . | . | . | . |
| . | . | . | . | . | | | |
| Vv12g1043 | . | . | . | . | . | . | . |
| . | . | . | . | . | . | . | . |
| . | . | . | . | . | | | |
| Vv12g1044 | . | . | . | . | . | . | . |
| . | . | . | . | . | . | . | . |
| . | . | . | . | . | | | |
| Vv12g1045 | . | . | . | . | . | . | . |
| . | . | . | . | . | . | . | . |
| . | . | . | . | . | | | |
| Vv12g1046 | . | . | . | . | . | . | . |
| . | . | . | . | . | . | . | . |
| . | . | . | . | . | | | |
| Vv12g1047 | Tc01_g014770 | | . | . | . | . | . |
| . | . | . | . | . | . | . | . |
| . | . | . | . | . | . | | |
| Vv12g1048 | . | . | . | . | . | . | . |
| . | . | . | . | . | . | . | . |
| . | . | . | . | . | | | |
| Vv12g1049 | . | . | . | . | . | . | . |
| . | . | . | . | . | . | . | . |
| . | . | . | . | . | | | |
| Vv12g1050 | . | . | . | . | . | . | . |
| . | . | . | . | . | . | . | . |
| . | . | . | . | . | | | |
| Vv12g1051 | . | . | . | . | . | . | . |
| . | . | . | . | . | . | . | . |
| . | . | . | . | . | | | |
| Vv12g1052 | . | . | . | . | . | . | . |
| . | . | . | . | . | . | . | . |
| . | . | . | . | . | | | |
| Vv12g1053 | . | . | . | . | . | . | . |
| . | . | . | . | . | . | . | . |
| . | . | . | . | . | | | |
| Vv12g1054 | . | . | . | . | . | . | . |
| . | . | . | . | . | . | . | . |
| . | . | . | . | . | | | |
| Vv12g1055 | . | . | . | . | . | . | . |
| . | . | . | . | . | . | . | . |
| . | . | . | . | . | | | |
| Vv12g1056 | . | . | . | . | . | . | . |

| | | | | | | | |
|---|---|---|---|---|---|---|---|
| . | . | . | . | . | . | . | . |
| . | . | . | . | . | | | |
| Vv12g1057 | . | . | . | . | . | . | . |
| . | . | . | . | . | . | . | . |
| . | . | . | . | . | | | |
| Vv12g1058 | . | . | . | . | . | . | . |
| . | . | . | . | . | . | . | . |
| . | . | . | . | . | | | |
| Vv12g1059 | . | . | . | . | . | . | . |
| . | . | . | . | . | . | . | . |
| . | . | . | . | . | | | |
| Vv12g1060 | . | . | . | . | . | . | . |
| . | . | . | . | . | . | . | . |
| . | . | . | . | . | | | |
| Vv12g1061 | . | . | . | . | . | . | . |
| . | . | . | . | . | . | . | . |
| . | . | . | . | . | | | |
| Vv12g1062 | . | . | . | . | . | . | . |
| . | . | . | . | . | . | . | . |
| . | . | . | . | . | | | |
| Vv12g1063 | . | . | . | . | . | . | . |
| . | . | . | . | . | . | . | . |
| . | . | . | . | . | | | |
| Vv12g1064 | . | . | . | . | . | . | . |
| . | . | . | . | . | . | . | . |
| . | . | . | . | . | | | |
| Vv12g1065 | . | . | . | . | . | . | . |
| . | . | . | . | . | . | . | . |
| . | . | . | . | . | | | |
| Vv12g1066 | . | . | . | . | . | . | . |
| . | . | . | . | . | . | . | . |
| . | . | . | . | . | | | |
| Vv12g1067 | . | . | . | . | . | . | . |
| . | . | . | . | . | . | . | . |
| . | . | . | . | . | | | |
| Vv12g1068 | . | . | . | . | . | . | . |
| . | . | . | . | . | . | . | . |
| . | . | . | . | . | | | |
| Vv12g1069 | . | . | . | . | . | . | . |
| . | . | . | . | . | . | . | . |
| . | . | . | . | . | | | |
| Vv12g1070 | Tc01_g020550 | | . | . | . | . | . |
| . | . | . | . | . | . | . | . |
| . | . | . | . | . | . | | |
| Vv12g1071 | Tc01_g003700 | | . | . | . | . | . |
| . | . | . | . | . | . | . | . |
| . | . | . | . | . | . | | |
| Vv12g1072 | . | . | . | . | . | . | . |
| . | . | . | . | . | . | . | . |
| . | . | . | . | . | | | |

Vv12g1073 Tc01_g003690 . . . . .
. . . . . . . .
. . . . . .
Vv12g1074 Tc01_g003680 . Gorai.008G069600.1 .
. . . . . . . .
. . . . . . . .
Vv12g1075 . . . . . . .
. . . . . . . .
. . . . .
Vv12g1076 . . . . . . .
. . . . . . . .
. . . . .
Vv12g1077 . . . . . . .
. . . . . . . .
. . . . .
Vv12g1078 Tc01_g003670 . . . . .
. . . . . . . .
. . . . . .
Vv12g1079 . . . . . . .
. . . . . . . .
. . . . .
Vv12g1080 . . . . . . .
. . . . . . . .
. . . . .
Vv12g1081 Tc01_g003660 . Gorai.008G069500.1 .
. . . . . . . .
. . . . . . . .
Vv12g1082 Tc01_g003650 . . . . .
. . . . . . . .
. . . . . .
Vv12g1083 . . . . . . .
. . . . . . . .
. . . . .
Vv12g1084 . . . . . . .
. . . . . . . .
. . . . .
Vv12g1085 . . . . . . .
. . . . . . . .
. . . . .
Vv12g1086 Tc01_g003640 . Gorai.008G069400.1 .
Gorai.003G016800.1 . . . . .
. . . . . . . .
. .
Vv12g1087 . . . . . . .
. . . . . . . .
. . . . .
Vv12g1088 . . . . . . .
. . . . . . . .
. . . . .
Vv12g1089 . . . . . . .

| | | | | | | | |
|---|---|---|---|---|---|---|---|
| . | . | . | . | . | . | . | . |
| . | . | . | . | . | | | |
| Vv12g1090 | . | . | . | . | . | . | . |
| . | . | . | . | . | . | . | . |
| . | . | . | . | . | | | |
| Vv12g1091 | . | . | . | . | . | . | . |
| . | . | . | . | . | . | . | . |
| . | . | . | . | . | | | |
| Vv12g1092 | . | . | . | . | . | . | . |
| . | . | . | . | . | . | . | . |
| . | . | . | . | . | | | |
| Vv12g1093 | . | . | . | . | . | . | . |
| . | . | . | . | . | . | . | . |
| . | . | . | . | . | | | |
| Vv12g1094 | . | . | . | . | . | . | . |
| . | . | . | . | . | . | . | . |
| . | . | . | . | . | | | |
| Vv12g1095 | . | . | . | . | . | . | . |
| . | . | . | . | . | . | . | . |
| . | . | . | . | . | | | |
| Vv12g1096 | . | . | . | . | . | . | . |
| . | . | . | . | . | . | . | . |
| . | . | . | . | . | | | |
| Vv12g1097 | . | . | . | . | . | . | . |
| . | . | . | . | . | . | . | . |
| . | . | . | . | . | | | |
| Vv12g1098 | . | . | . | . | . | . | . |
| . | . | . | . | . | . | . | . |
| . | . | . | . | . | | | |
| Vv12g1099 | . | . | . | . | . | . | . |
| . | . | . | . | . | . | . | . |
| . | . | . | . | . | | | |
| Vv12g1100 | . | . | . | . | . | . | . |
| . | . | . | . | . | . | . | . |
| . | . | . | . | . | | | |
| Vv12g1101 | . | . | . | . | . | . | . |
| . | . | . | . | . | . | . | . |
| . | . | . | . | . | | | |
| Vv12g1102 | . | . | . | . | . | . | . |
| . | . | . | . | . | . | . | . |
| . | . | . | . | . | | | |
| Vv12g1103 | . | . | . | . | . | . | . |
| . | . | . | . | . | . | . | . |
| . | . | . | . | . | | | |
| Vv12g1104 | Tc01_g020510 | | . | . | . | . | . |
| . | . | . | . | . | . | . | . |
| . | . | . | . | . | . | | |
| Vv12g1105 | . | . | . | . | . | . | . |
| . | . | . | . | . | . | . | . |
| . | . | . | . | . | | | |

Vv12g1106 . . . . . . .
. . . . . . . .
. . . . .
Vv12g1107 . . . . . . .
. . . . . . . .
. . . . .
Vv12g1108 . . . . . . .
. . . . . . . .
. . . . .
Vv12g1109 . . . . . . .
. . . . . . . .
. . . . .
Vv12g1110 . . . . . . .
. . . . . . . .
. . . . .
Vv12g1111 . . . . . . .
. . . . . . . .
. . . . .
Vv12g1112 . . . . . . .
. . . . . . . .
. . . . .
Vv12g1113 . . . . . . .
. . . . . . . .
. . . . .
Vv12g1114 . . . . . . .
. . . . . . . .
. . . . .
Vv12g1115 . . . . . . .
. . . . . . . .
. . . . .
Vv12g1116 . . . . . . .
. . . . . . . .
. . . . .
Vv12g1117 . . . . . . .
. . . . . . . .
. . . . .
Vv12g1118 . . . . . . .
. . . . . . . .
. . . . .
Vv12g1119 . . . . . . .
. . . . . . . .
. . . . .
Vv12g1120 Tc01_g004580 Gorai.007G053300.1 Gorai.
008G073600.1 . . . . . . .
. . . . . . . .
. .
Vv12g1121 Tc01_g001220 . . Gorai.007G191600.1
. . . . . . . .
. . . . . . . .
Vv12g1122 . . . . . . .

. . . . . . . .
. . . . .
Vv12g1123 . . . . . . .
. . . . . . . .
. . . . .
Vv12g1124 . . . . . . .
. . . . . . . .
. . . . .
Vv12g1125 . . . . . . .
. . . . . . . .
. . . . .
Vv12g1126 . . . . . . .
. . . . . . . .
. . . . .
Vv12g1127 . . . . . . .
. . . . . . . .
. . . . .
Vv12g1128 . . . . . . .
. . . . . . . .
. . . . .
Vv12g1129 . . . . . . .
. . . . . . . .
. . . . .
Vv12g1130 . . . . . . .
. . . . . . . .
. . . . .
Vv12g1131 . . . . . . .
. . . . . . . .
. . . . .
Vv12g1132 . . . . . . .
. . . . . . . .
. . . . .
Vv12g1133 . . . . . . .
. . . . . . . .
. . . . .
Vv12g1134 . . . . . . .
. . . . . . . .
. . . . .
Vv12g1135 . . . . . . .
. . . . . . . .
. . . . .
Vv12g1136 . . . . . . .
. . . . . . . .
. . . . .
Vv12g1137 . . . . . . .
. . . . . . . .
. . . . .
Vv12g1138 . . . . . . .
. . . . . . . .
. . . . .

Vv12g1139 . . . . . . .
. . . . . . . .
. . . . .
Vv12g1140 . . . . . . .
. . . . . . . .
. . . . .
Vv12g1141 . . . . . . .
. . . . . . . .
. . . . .
Vv12g1142 . . . . . . .
. . . . . . . .
. . . . .
Vv12g1143 . . . . . . .
. . . . . . . .
. . . . .
Vv12g1144 . . . . . . .
. . . . . . . .
. . . . .
Vv12g1145 . . . . . . .
. . . . . . . .
. . . . .
Vv12g1146 . . . . . . .
. . . . . . . .
. . . . .
Vv12g1147 . . . . . . .
. . . . . . . .
. . . . .
Vv12g1148 . . . . . . .
. . . . . . . .
. . . . .
Vv12g1149 . . . . . . .
. . . . . . . .
. . . . .
Vv12g1150 . . . . . . .
. . . . . . . .
. . . . .
Vv12g1151 . . . . . . .
. . . . . . . .
. . . . .
Vv12g1152 . . . . . . .
. . . . . . . .
. . . . .
Vv12g1153 . . . . . . .
. . . . . . . .
. . . . .
Vv12g1154 . . . . . . .
. . . . . . . .
. . . . .
Vv12g1155 Tc01_g000990 . . Gorai.007G190200.1
Gorai.003G005100.1 . . . . .

. . . . . . . .
. .
Vv12g1156 Tc01_g004520 Gorai.007G053000.1 Gorai.
008G073200.1 . . . . . . .
. . . . . . . .
. .
Vv12g1157 Tc01_g040190 . . . . .
. . . . . . . .
. . . . . .
Vv12g1158 . . . . . . .
. . . . . . . .
. . . . .
Vv12g1159 . . . . . . .
. . . . . . . .
. . . . .
Vv12g1160 . . . . . . .
. . . . . . . .
. . . . .
Vv12g1161 . . . . . . .
. . . . . . . .
. . . . .
Vv12g1162 . . . . . . .
. . . . . . . .
. . . . .
Vv12g1163 . . . . . . .
. . . . . . . .
. . . . .
Vv12g1164 Tc01_g040200 . . . . .
. . . . . . . .
. . . . . .
Vv12g1165 . . . . . . .
. . . . . . . .
. . . . .
Vv12g1166 . . . . . . .
. . . . . . . .
. . . . .
Vv12g1167 . . . . . . .
. . . . . . . .
. . . . .
Vv12g1168 . . . . . . .
. . . . . . . .
. . . . .
Vv12g1169 . . . . . . .
. . . . . . . .
. . . . .
Vv12g1170 . . . . . . .
. . . . . . . .
. . . . .
Vv12g1171 . . . . . . .
. . . . . . . .

| | | | | | | | |
|---|---|---|---|---|---|---|---|
| . | . | . | . | . | | | |
| Vv12g1172 | Tc01_g040220 | | . | . | . | . | . |
| . | . | . | . | . | . | . | . |
| . | . | . | . | . | . | | |
| Vv12g1173 | . | . | . | . | . | . | . |
| . | . | . | . | . | . | . | . |
| . | . | . | . | . | | | |
| Vv12g1174 | . | . | . | . | . | . | . |
| . | . | . | . | . | . | . | . |
| . | . | . | . | . | | | |
| Vv12g1175 | . | . | . | . | . | . | . |
| . | . | . | . | . | . | . | . |
| . | . | . | . | . | | | |
| Vv12g1176 | . | . | . | . | . | . | . |
| . | . | . | . | . | . | . | . |
| . | . | . | . | . | | | |
| Vv12g1177 | . | . | . | . | . | . | . |
| . | . | . | . | . | . | . | . |
| . | . | . | . | . | | | |
| Vv12g1178 | . | . | . | . | . | . | . |
| . | . | . | . | . | . | . | . |
| . | . | . | . | . | | | |
| Vv12g1179 | . | . | . | . | . | . | . |
| . | . | . | . | . | . | . | . |
| . | . | . | . | . | | | |
| Vv12g1180 | . | . | . | . | . | . | . |
| . | . | . | . | . | . | . | . |
| . | . | . | . | . | | | |
| Vv12g1181 | . | . | . | . | . | . | . |
| . | . | . | . | . | . | . | . |
| . | . | . | . | . | | | |
| Vv12g1182 | . | . | . | . | . | . | . |
| . | . | . | . | . | . | . | . |
| . | . | . | . | . | | | |
| Vv12g1183 | . | . | . | . | . | . | . |
| . | . | . | . | . | . | . | . |
| . | . | . | . | . | | | |
| Vv12g1184 | . | . | . | . | . | . | . |
| . | . | . | . | . | . | . | . |
| . | . | . | . | . | | | |
| Vv12g1185 | . | . | . | . | . | . | . |
| . | . | . | . | . | . | . | . |
| . | . | . | . | . | | | |
| Vv12g1186 | . | . | . | . | . | . | . |
| . | . | . | . | . | . | . | . |
| . | . | . | . | . | | | |
| Vv12g1187 | . | . | . | . | . | . | . |
| . | . | . | . | . | . | . | . |
| . | . | . | . | . | | | |
| Vv12g1188 | . | . | . | . | . | . | . |

. . . . . . . .
. . . . .
Vv12g1189 . . . . . . .
. . . . . . . .
. . . . .
Vv12g1190 . . . . . . .
. . . . . . . .
. . . . .
Vv12g1191 . . . . . . .
. . . . . . . .
. . . . .
Vv12g1192 . . . . . . .
. . . . . . . .
. . . . .
Vv12g1193 . . . . . . .
. . . . . . . .
. . . . .
Vv12g1194 . . . . . . .
. . . . . . . .
. . . . .
Vv12g1195 . . . . . . .
. . . . . . . .
. . . . .
Vv12g1196 . . . . . . .
. . . . . . . .
. . . . .
Vv12g1197 . . . . . . .
. . . . . . . .
. . . . .
Vv12g1198 . . . . . . .
. . . . . . . .
. . . . .
Vv12g1199 . . . . . . .
. . . . . . . .
. . . . .
Vv12g1200 . . . . . . .
. . . . . . . .
. . . . .
Vv12g1201 . . . . . . .
. . . . . . . .
. . . . .
Vv12g1202 . . . . . . .
. . . . . . . .
. . . . .
Vv12g1203 . . . . . . .
. . . . . . . .
. . . . .
Vv12g1204 . . . . . . .
. . . . . . . .
. . . . .

| | | | | | | | |
|---|---|---|---|---|---|---|---|
| Vv12g1205 | . | . | . | . | . | . | . |
| . | . | . | . | . | . | . | . |
| . | . | . | . | . | | | |
| Vv12g1206 | . | . | . | . | . | . | . |
| . | . | . | . | . | . | . | . |
| . | . | . | . | . | | | |
| Vv12g1207 | . | . | . | . | . | . | . |
| . | . | . | . | . | . | . | . |
| . | . | . | . | . | | | |
| Vv12g1208 | . | . | . | . | . | . | . |
| . | . | . | . | . | . | . | . |
| . | . | . | . | . | | | |
| Vv12g1209 | . | . | . | . | . | . | . |
| . | . | . | . | . | . | . | . |
| . | . | . | . | . | | | |
| Vv12g1210 | . | . | . | . | . | . | . |
| . | . | . | . | . | . | . | . |
| . | . | . | . | . | | | |
| Vv12g1211 | . | . | . | . | . | . | . |
| . | . | . | . | . | . | . | . |
| . | . | . | . | . | | | |
| Vv12g1212 | . | . | . | . | . | . | . |
| . | . | . | . | . | . | . | . |
| . | . | . | . | . | | | |
| Vv12g1213 | . | . | . | . | . | . | . |
| . | . | . | . | . | . | . | . |
| . | . | . | . | . | | | |
| Vv12g1214 | . | . | . | . | . | . | . |
| . | . | . | . | . | . | . | . |
| . | . | . | . | . | | | |
| Vv12g1215 | . | . | . | . | . | . | . |
| . | . | . | . | . | . | . | . |
| . | . | . | . | . | | | |
| Vv12g1216 | . | . | . | . | . | . | . |
| . | . | . | . | . | . | . | . |
| . | . | . | . | . | | | |
| Vv12g1217 | . | . | . | . | . | . | . |
| . | . | . | . | . | . | . | . |
| . | . | . | . | . | | | |
| Vv12g1218 | Tc01_g040290 | | . | . | . | . | . |
| . | . | . | . | . | . | . | . |
| . | . | . | . | . | . | | |
| Vv12g1219 | . | . | . | . | . | . | . |
| . | . | . | . | . | . | . | . |
| . | . | . | . | . | | | |
| Vv12g1220 | Tc01_g010920 | | . | . | . | . | . |
| . | . | . | . | . | . | . | . |
| . | . | . | . | . | . | | |
| Vv12g1221 | . | . | . | . | . | . | . |
| . | . | . | . | . | . | . | . |

| | | | | | | | |
|---|---|---|---|---|---|---|---|
| Vv12g1222 | . | . | . | . | . | . | . |
| Vv12g1223 | . | . | . | . | . | . | . |
| Vv12g1224 | . | . | . | . | . | . | . |
| Vv12g1225 | . | . | . | . | . | . | . |
| Vv12g1226 | . | . | . | . | . | . | . |
| Vv12g1227 | . | . | . | . | . | . | . |
| Vv12g1228 | Tc01_g040220 | | . | . | . | . | . |
| Vv12g1229 | . | . | . | . | . | . | . |
| Vv12g1230 | Tc01_g040210 | | . | . | . | . | . |
| Vv12g1231 | . | . | . | . | . | . | . |
| Vv12g1232 | Tc01_g040200 | | . | . | . | . | . |
| Vv12g1233 | Tc01_g040190 | | . | . | . | . | . |
| Vv12g1234 | . | . | . | . | . | . | . |
| Vv12g1235 | Tc01_g040180 | | . | . | . | . | . |
| Vv12g1236 | . | . | . | . | . | . | . |
| Vv12g1237 | . | . | . | . | . | . | . |
| Vv12g1238 | . | . | . | . | . | . | . |

| | | | | | | | |
|---|---|---|---|---|---|---|---|
| Vv12g1239 | Tc01_g040170 | | . | . | . | . | . |
| Vv12g1240 | Tc01_g040160 | | . | Gorai.008G112600.1 | | | . |
| Vv12g1241 | . | . | . | . | . | . | . |
| Vv12g1242 | Tc01_g040150 | | . | . | . | . | . |
| Vv12g1243 | . | . | . | . | . | . | . |
| Vv12g1244 | . | . | . | . | . | . | . |
| Vv12g1245 | . | . | . | . | . | . | . |
| Vv12g1246 | . | . | . | . | . | . | . |
| Vv12g1247 | . | . | . | . | . | . | . |
| Vv12g1248 | Tc01_g040050 | | . | . | . | . | . |
| Vv12g1249 | . | . | . | . | . | . | . |
| Vv12g1250 | Tc01_g040010 | | . | . | . | . | . |
| Vv12g1251 | . | . | . | . | . | . | . |
| Vv12g1252 | . | . | . | . | . | . | . |
| Vv12g1253 | . | . | . | . | . | . | . |
| Vv12g1254 | Tc01_g011350 | | . | . | . | . | . |

Vv12g1255 . . . . . . .
. . . . . . . .
. . . . .
Vv12g1256 Tc01_g039880 . Gorai.008G111100.1 .
. . . . . . . .
. . . . . . . .
Vv12g1257 Tc01_g038610 . . . . .
. . . . . . . .
. . . . . .
Vv12g1258 Tc01_g007650 . . . . .
. . . . . . . .
. . . . . .
Vv12g1259 . . . . . . .
. . . . . . . .
. . . . .
Vv12g1260 . . . . . . .
. . . . . . . .
. . . . .
Vv12g1261 . . . . . . .
. . . . . . . .
. . . . .
Vv12g1262 . . . . . . .
. . . . . . . .
. . . . .
Vv12g1263 . . . . . . .
. . . . . . . .
. . . . .
Vv12g1264 . . . . . . .
. . . . . . . .
. . . . .
Vv12g1265 . . . . . . .
. . . . . . . .
. . . . .
Vv12g1266 . . . . . . .
. . . . . . . .
. . . . .
Vv12g1267 . . . . . . .
. . . . . . . .
. . . . .
Vv12g1268 . . . . . . .
. . . . . . . .
. . . . .
Vv12g1269 . . . . . . .
. . . . . . . .
. . . . .
Vv12g1270 . . . . . . .
. . . . . . . .
. . . . .
Vv12g1271 . . . . . . .
. . . . . . . .

| | | | | | | | |
|---|---|---|---|---|---|---|---|
| . | . | . | . | . | | | |
| Vv12g1272 | . | . | . | . | . | . | . |
| . | . | . | . | . | . | . | . |
| . | . | . | . | . | | | |
| Vv12g1273 | . | . | . | . | . | . | . |
| . | . | . | . | . | . | . | . |
| . | . | . | . | . | | | |
| Vv12g1274 | . | . | . | . | . | . | . |
| . | . | . | . | . | . | . | . |
| . | . | . | . | . | | | |
| Vv12g1275 | . | . | . | . | . | . | . |
| . | . | . | . | . | . | . | . |
| . | . | . | . | . | | | |
| Vv12g1276 | . | . | . | . | . | . | . |
| . | . | . | . | . | . | . | . |
| . | . | . | . | . | | | |
| Vv12g1277 | . | . | . | . | . | . | . |
| . | . | . | . | . | . | . | . |
| . | . | . | . | . | | | |
| Vv12g1278 | . | . | . | . | . | . | . |
| . | . | . | . | . | . | . | . |
| . | . | . | . | . | | | |
| Vv12g1279 | . | . | . | . | . | . | . |
| . | . | . | . | . | . | . | . |
| . | . | . | . | . | | | |
| Vv12g1280 | . | . | . | . | . | . | . |
| . | . | . | . | . | . | . | . |
| . | . | . | . | . | | | |
| Vv12g1281 | Tc01_g040010 | | . | . | . | . | . |
| . | . | . | . | . | . | . | . |
| . | . | . | . | . | . | | |
| Vv12g1282 | . | . | . | . | . | . | . |
| . | . | . | . | . | . | . | . |
| . | . | . | . | . | | | |
| Vv12g1283 | . | . | . | . | . | . | . |
| . | . | . | . | . | . | . | . |
| . | . | . | . | . | | | |
| Vv12g1284 | . | . | . | . | . | . | . |
| . | . | . | . | . | . | . | . |
| . | . | . | . | . | | | |
| Vv12g1285 | . | . | . | . | . | . | . |
| . | . | . | . | . | . | . | . |
| . | . | . | . | . | | | |
| Vv12g1286 | . | . | . | . | . | . | . |
| . | . | . | . | . | . | . | . |
| . | . | . | . | . | | | |
| Vv12g1287 | . | . | . | . | . | . | . |
| . | . | . | . | . | . | . | . |
| . | . | . | . | . | | | |
| Vv12g1288 | . | . | . | . | . | . | . |

. . . . . . . .
. . . . .
Vv12g1289 . . . . . . .
. . . . . . . .
. . . . .
Vv12g1290 . . . . . . .
. . . . . . . .
. . . . .
Vv12g1291 . . . . . . .
. . . . . . . .
. . . . .
Vv12g1292 . . . . . . .
. . . . . . . .
. . . . .
Vv12g1293 . . . . . . .
. . . . . . . .
. . . . .
Vv12g1294 . . . . . . .
. . . . . . . .
. . . . .
Vv12g1295 . . . . . . .
. . . . . . . .
. . . . .
Vv12g1296 . . . . . . .
. . . . . . . .
. . . . .
Vv12g1297 Tc01_g038650 . . . . .
. . . . . . . .
. . . . . .
Vv12g1298 Tc01_g007620 . . . . .
. . . . . . . .
. . . . . .
Vv12g1299 . . . . . . .
. . . . . . . .
. . . . .
Vv12g1300 Tc01_g007590 . Gorai.008G088900.1 .
Gorai.003G036300.1 . . . . .
. . . . . . . .
. .
Vv12g1301 . . . . . . .
. . . . . . . .
. . . . .
Vv12g1302 . . . . . . .
. . . . . . . .
. . . . .
Vv12g1303 . . . . . . .
. . . . . . . .
. . . . .
Vv12g1304 . . . . . . .
. . . . . . . .

. . . . .
Vv12g1305 Tc01_g040050 . . . . .
. . . . . . . .
. . . . . .
Vv12g1306 . . . . . . .
. . . . . . . .
. . . . .
Vv12g1307 . . . . . . .
. . . . . . . .
. . . . .
Vv12g1308 . . . . . . .
. . . . . . . .
. . . . .
Vv12g1309 . . . . . . .
. . . . . . . .
. . . . .
Vv12g1310 . . . . . . .
. . . . . . . .
. . . . .
Vv12g1311 . . . . . . .
. . . . . . . .
. . . . .
Vv12g1312 . . . . . . .
. . . . . . . .
. . . . .
Vv12g1313 . . . . . . .
. . . . . . . .
. . . . .
Vv12g1314 . . . . . . .
. . . . . . . .
. . . . .
Vv12g1315 . . . . . . .
. . . . . . . .
. . . . .
Vv12g1316 Tc01_g040150 . . . . .
. . . . . . . .
. . . . . .
Vv12g1317 Tc01_g040160 . Gorai.008G112600.1 .
. . . . . . . .
. . . . . . . .
Vv12g1318 Tc01_g012740 . . . . .
. . . . . . . .
. . . . . .
Vv12g1319 Tc01_g012730 . . . . .
. . . . . . . .
. . . . . .
Vv12g1320 . . . . . . .
. . . . . . . .
. . . . .
Vv12g1321 . . . . . . .

| | | | | | | | |
|---|---|---|---|---|---|---|---|
| . | . | . | . | . | . | . | . |
| . | . | . | . | . | | | |
| Vv12g1322 | Tc01_g007530 | | . | . | . | . | . |
| . | . | . | . | . | . | . | . |
| . | . | . | . | . | . | | |
| Vv12g1323 | . | . | . | . | . | . | . |
| . | . | . | . | . | . | . | . |
| . | . | . | . | . | | | |
| Vv12g1324 | . | . | . | . | . | . | . |
| . | . | . | . | . | . | . | . |
| . | . | . | . | . | | | |
| Vv12g1325 | . | . | . | . | . | . | . |
| . | . | . | . | . | . | . | . |
| . | . | . | . | . | | | |
| Vv12g1326 | . | . | . | . | . | . | . |
| . | . | . | . | . | . | . | . |
| . | . | . | . | . | | | |
| Vv12g1327 | Tc01_g040170 | | . | . | . | . | . |
| . | . | . | . | . | . | . | . |
| . | . | . | . | . | . | | |
| Vv12g1328 | Tc01_g040180 | | . | . | . | . | . |
| . | . | . | . | . | . | . | . |
| . | . | . | . | . | . | | |
| Vv12g1329 | . | . | . | . | . | . | . |
| . | . | . | . | . | . | . | . |
| . | . | . | . | . | | | |
| Vv12g1330 | Tc01_g040190 | | . | . | . | . | . |
| . | . | . | . | . | . | . | . |
| . | . | . | . | . | . | | |
| Vv12g1331 | . | . | . | . | . | . | . |
| . | . | . | . | . | . | . | . |
| . | . | . | . | . | | | |
| Vv12g1332 | . | . | . | . | . | . | . |
| . | . | . | . | . | . | . | . |
| . | . | . | . | . | | | |
| Vv12g1333 | Tc01_g040200 | | . | . | . | . | . |
| . | . | . | . | . | . | . | . |
| . | . | . | . | . | . | | |
| Vv12g1334 | . | . | . | . | . | . | . |
| . | . | . | . | . | . | . | . |
| . | . | . | . | . | | | |
| Vv12g1335 | . | . | . | . | . | . | . |
| . | . | . | . | . | . | . | . |
| . | . | . | . | . | | | |
| Vv12g1336 | . | . | . | . | . | . | . |
| . | . | . | . | . | . | . | . |
| . | . | . | . | . | | | |
| Vv12g1337 | . | . | . | . | . | . | . |
| . | . | . | . | . | . | . | . |
| . | . | . | . | . | | | |

Vv12g1338 Tc01_g040210 . . . . .
. . . . . . . .
. . . . . .
Vv12g1339 Tc01_g040220 . . . . .
. . . . . . . .
. . . . . .
Vv12g1340 . . . . . . .
. . . . . . . .
. . . . .
Vv12g1341 . . . . . . .
. . . . . . . .
. . . . .
Vv12g1342 . . . . . . .
. . . . . . . .
. . . . .
Vv12g1343 . . . . . . .
. . . . . . . .
. . . . .
Vv12g1344 . . . . . . .
. . . . . . . .
. . . . .
Vv12g1345 . . . . . . .
. . . . . . . .
. . . . .
Vv12g1346 Tc01_g037320 . . . . .
. . . . . . . .
. . . . . .
Vv12g1347 . . . . . . .
. . . . . . . .
. . . . .
Vv12g1348 . . . . . . .
. . . . . . . .
. . . . .
Vv12g1349 . . . . . . .
. . . . . . . .
. . . . .
Vv12g1350 . . . . . . .
. . . . . . . .
. . . . .
Vv12g1351 . . . . . . .
. . . . . . . .
. . . . .
Vv12g1352 Tc01_g007220 . . . Gorai.
003G034500.1 . . . . . . .
. . . . . . . .
Vv12g1353 . . . . . . .
. . . . . . . .
. . . . .
Vv12g1354 . . . . . . .
. . . . . . . .

. . . . .
Vv12g1355 Tc01_g002030 . . . Gorai.
003G011000.1 . . . . . . .
. . . . . . . .
Vv12g1356 . . . . . . .
. . . . . . . .
. . . . .
Vv12g1357 Tc01_g007200 . Gorai.008G087500.1 .
Gorai.003G034200.1 . . . . .
. . . . . . . .
. .
Vv12g1358 Tc01_g002220 . Gorai.008G064700.1 .
. . . . . . . .
. . . . . . . .
Vv12g1359 . . . . . . .
. . . . . . . .
. . . . .
Vv12g1360 . . . . . . .
. . . . . . . .
. . . . .
Vv12g1361 . . . . . . .
. . . . . . . .
. . . . .
Vv12g1362 Tc01_g025760 . . . . .
. . . . . . . .
. . . . . .
Vv12g1363 Tc01_g038770 . . . . .
. . . . . . . .
. . . . . .
Vv12g1364 . . . . . . .
. . . . . . . .
. . . . .
Vv12g1365 . . . . . . .
. . . . . . . .
. . . . .
Vv12g1366 . . . . . . .
. . . . . . . .
. . . . .
Vv12g1367 . . . . . . .
. . . . . . . .
. . . . .
Vv12g1368 Tc01_g038760 . . . . .
. . . . . . . .
. . . . . .
Vv12g1369 Tc01_g038750 . . . . .
. . . . . . . .
. . . . . .
Vv12g1370 . . . . . . .
. . . . . . . .
. . . . .

| | | | | | | | | | | | | | | | | | | | | |
|---|---|---|---|---|---|---|---|---|---|---|---|---|---|---|---|---|---|---|---|---|
| Vv12g1371 | . | . | . | . | . | . | . | . | . | . | . | . | . | . | . | . | . | . | . | . |
| Vv12g1372 | . | . | . | . | . | . | . | . | . | . | . | . | . | . | . | . | . | . | . | . |
| Vv12g1373 | . | . | . | . | . | . | . | . | . | . | . | . | . | . | . | . | . | . | . | . |
| Vv12g1374 | . | . | . | . | . | . | . | . | . | . | . | . | . | . | . | . | . | . | . | . |
| Vv12g1375 | . | . | . | . | . | . | . | . | . | . | . | . | . | . | . | . | . | . | . | . |
| Vv12g1376 | . | . | . | . | . | . | . | . | . | . | . | . | . | . | . | . | . | . | . | . |
| Vv12g1377 | . | . | . | . | . | . | . | . | . | . | . | . | . | . | . | . | . | . | . | . |
| Vv12g1378 | . | . | . | . | . | . | . | . | . | . | . | . | . | . | . | . | . | . | . | . |
| Vv12g1379 | Tc01_g026140 | . | . | . | . | . | . | . | . | . | . | . | . | . | . | . | . | . | . | . |
| Vv12g1380 | . | . | . | . | . | . | . | . | . | . | . | . | . | . | . | . | . | . | . | . |
| Vv12g1381 | Tc01_g037000 | . | . | . | . | . | . | . | . | . | . | . | . | . | . | . | . | . | . | . |
| Vv12g1382 | . | . | . | . | . | . | . | . | . | . | . | . | . | . | . | . | . | . | . | . |
| Vv12g1383 | . | . | . | . | . | . | . | . | . | . | . | . | . | . | . | . | . | . | . | . |
| Vv12g1384 | . | . | . | . | . | . | . | . | . | . | . | . | . | . | . | . | . | . | . | . |
| Vv12g1385 | Tc01_g002530 | . | . | . | Gorai.003G012700.1 | . | . | . | . | . | . | . | . | . | . | . | . | . | . | |
| Vv12g1386 | . | . | . | . | . | . | . | . | . | . | . | . | . | . | . | . | . | . | . | . |
| Vv12g1387 | . | . | . | . | . | . | . | . | . | . | . | . | . | . | . | | | | | |

| | | | | | | | |
|---|---|---|---|---|---|---|---|
| . | . | . | . | . | | | |
| Vv12g1388 | . | . | . | . | . | . | . |
| . | . | . | . | . | . | . | . |
| . | . | . | . | . | | | |
| Vv12g1389 | . | . | . | . | . | . | . |
| . | . | . | . | . | . | . | . |
| . | . | . | . | . | | | |
| Vv12g1390 | . | . | . | . | . | . | . |
| . | . | . | . | . | . | . | . |
| . | . | . | . | . | | | |
| Vv12g1391 | . | . | . | . | . | . | . |
| . | . | . | . | . | . | . | . |
| . | . | . | . | . | | | |
| Vv12g1392 | Tc01_g026460 | | Gorai.007G152300.1 | | | . | . |
| . | . | . | . | . | . | . | . |
| . | . | . | . | . | . | . | . |
| Vv12g1393 | Tc01_g026890 | | . | . | . | . | . |
| . | . | . | . | . | . | . | . |
| . | . | . | . | . | . | | |
| Vv12g1394 | . | . | . | . | . | . | . |
| . | . | . | . | . | . | . | . |
| . | . | . | . | . | | | |
| Vv12g1395 | . | . | . | . | . | . | . |
| . | . | . | . | . | . | . | . |
| . | . | . | . | . | | | |
| Vv12g1396 | Tc01_g002670 | | . | . | . | . | . |
| . | . | . | . | . | . | . | . |
| . | . | . | . | . | . | | |
| Vv12g1397 | . | . | . | . | . | . | . |
| . | . | . | . | . | . | . | . |
| . | . | . | . | . | | | |
| Vv12g1398 | Tc01_g002680 | | . | . | . | . | . |
| . | . | . | . | . | . | . | . |
| . | . | . | . | . | . | | |
| Vv12g1399 | . | . | . | . | . | . | . |
| . | . | . | . | . | . | . | . |
| . | . | . | . | . | | | |
| Vv12g1400 | . | . | . | . | . | . | . |
| . | . | . | . | . | . | . | . |
| . | . | . | . | . | | | |
| Vv12g1401 | . | . | . | . | . | . | . |
| . | . | . | . | . | . | . | . |
| . | . | . | . | . | | | |
| Vv12g1402 | . | . | . | . | . | . | . |
| . | . | . | . | . | . | . | . |
| . | . | . | . | . | | | |
| Vv12g1403 | . | . | . | . | . | . | . |
| . | . | . | . | . | . | . | . |
| . | . | . | . | . | | | |
| Vv12g1404 | . | . | . | . | . | . | . |

. . . . . . . .
. . . . .
Vv12g1405 . . . . . . .
. . . . . . . .
. . . . .
Vv12g1406 . . . . . . .
. . . . . . . .
. . . . .
Vv12g1407 . . . . . . .
. . . . . . . .
. . . . .
Vv12g1408 . . . . . . .
. . . . . . . .
. . . . .
Vv12g1409 . . . . . . .
. . . . . . . .
. . . . .
Vv12g1410 . . . . . . .
. . . . . . . .
. . . . .
Vv12g1411 . . . . . . .
. . . . . . . .
. . . . .
Vv12g1412 . . . . . . .
. . . . . . . .
. . . . .
Vv12g1413 . . . . . . .
. . . . . . . .
. . . . .
Vv12g1414 . . . . . . .
. . . . . . . .
. . . . .
Vv12g1415 . . . . . . .
. . . . . . . .
. . . . .
Vv12g1416 . . . . . . .
. . . . . . . .
. . . . .
Vv12g1417 . . . . . . .
. . . . . . . .
. . . . .
Vv12g1418 . . . . . . .
. . . . . . . .
. . . . .
Vv12g1419 . . . . . . .
. . . . . . . .
. . . . .
Vv12g1420 . . . . . . .
. . . . . . . .
. . . . .

Vv12g1421 . . . . . . .
. . . . . . . .
. . . . .
Vv12g1422 . . . . . . .
. . . . . . . .
. . . . .
Vv12g1423 Tc01_g021790 . . . . .
. . . . . . . .
. . . . . .
Vv12g1424 . . . . . . .
. . . . . . . .
. . . . .
Vv12g1425 Tc01_g021730 Gorai.007G156800.1 . .
. . . . . . . .
. . . . . . . .
Vv12g1426 . . . . . . .
. . . . . . . .
. . . . .
Vv12g1427 . . . . . . .
. . . . . . . .
. . . . .
Vv12g1428 Tc01_g002880 . . . . .
. . . . . . . .
. . . . . .
Vv12g1429 . . . . . . .
. . . . . . . .
. . . . .
Vv12g1430 . . . . . . .
. . . . . . . .
. . . . .
Vv12g1431 Tc01_g021630 Gorai.007G151200.1 . .
. . . . . . . .
. . . . . . . .
Vv12g1432 . . . . . . .
. . . . . . . .
. . . . .
Vv12g1433 . . . . . . .
. . . . . . . .
. . . . .
Vv12g1434 . . . . . . .
. . . . . . . .
. . . . .
Vv12g1435 . . . . . . .
. . . . . . . .
. . . . .
Vv12g1436 . . . . . . .
. . . . . . . .
. . . . .
Vv12g1437 . . . . . . .
. . . . . . . .

. . . . .
Vv12g1438 Tc01_g003110 . . . . .
. . . . . . . .
. . . . . .
Vv12g1439 . . . . . . .
. . . . . . . .
. . . . .
Vv12g1440 . . . . . . .
. . . . . . . .
. . . . .
Vv12g1441 Tc01_g003370 . . . Gorai.
003G015600.1 . . . . . . .
. . . . . . . .
Vv12g1442 . . . . . . .
. . . . . . . .
. . . . .
Vv12g1443 . . . . . . .
. . . . . . . .
. . . . .
Vv12g1444 . . . . . . .
. . . . . . . .
. . . . .
Vv12g1445 . . . . . . .
. . . . . . . .
. . . . .
Vv12g1446 . . . . . . .
. . . . . . . .
. . . . .
Vv12g1447 . . . . . . .
. . . . . . . .
. . . . .
Vv12g1448 . . . . . . .
. . . . . . . .
. . . . .
Vv12g1449 . . . . . . .
. . . . . . . .
. . . . .
Vv12g1450 . . . . . . .
. . . . . . . .
. . . . .
Vv12g1451 . . . . . . .
. . . . . . . .
. . . . .
Vv12g1452 . . . . . . .
. . . . . . . .
. . . . .
Vv12g1453 . . . . . . .
. . . . . . . .
. . . . .
Vv12g1454 . . . . . . .

. . . . . . . .
. . . . .
Vv12g1455 . . . . . . .
. . . . . . . .
. . . . .
Vv12g1456 . . . . . . .
. . . . . . . .
. . . . .
Vv12g1457 . . . . . . .
. . . . . . . .
. . . . .
Vv12g1458 . . . . . . .
. . . . . . . .
. . . . .
Vv12g1459 . . . . . . .
. . . . . . . .
. . . . .
Vv12g1460 . . . . . . .
. . . . . . . .
. . . . .
Vv12g1461 . . . . . . .
. . . . . . . .
. . . . .
Vv12g1462 . . . . . . .
. . . . . . . .
. . . . .
Vv12g1463 . . . . . . .
. . . . . . . .
. . . . .
Vv12g1464 . . . . . . .
. . . . . . . .
. . . . .
Vv12g1465 . . . . . . .
. . . . . . . .
. . . . .
Vv12g1466 . . . . . . .
. . . . . . . .
. . . . .
Vv12g1467 . . . . . . .
. . . . . . . .
. . . . .
Vv12g1468 . . . . . . .
. . . . . . . .
. . . . .
Vv12g1469 . . . . . . .
. . . . . . . .
. . . . .
Vv12g1470 . . . . . . .
. . . . . . . .
. . . . .

Vv12g1471 . . . . . . .
. . . . . . . .
. . . . .
Vv12g1472 . . . . . . .
. . . . . . . .
. . . . .
Vv12g1473 . . . . . . .
. . . . . . . .
. . . . .
Vv12g1474 . . . . . . .
. . . . . . . .
. . . . .
Vv12g1475 . . . . . . .
. . . . . . . .
. . . . .
Vv12g1476 . . . . . . .
. . . . . . . .
. . . . .
Vv12g1477 . . . . . . .
. . . . . . . .
. . . . .
Vv12g1478 . . . . . . .
. . . . . . . .
. . . . .
Vv12g1479 . . . . . . .
. . . . . . . .
. . . . .
Vv12g1480 . . . . . . .
. . . . . . . .
. . . . .
Vv12g1481 . . . . . . .
. . . . . . . .
. . . . .
Vv13g0001 . . . . . . .
. . . . . . . .
. . . . .
Vv13g0002 . Gorai.013G021900.1 . . .
. . . . . . . .
. . . . . . .
Vv13g0003 . Gorai.013G010300.1 . . .
. . . . . . . .
. . . . . . .
Vv13g0004 . Gorai.013G010200.1 . . .
. . . . . . . .
. . . . . . .
Vv13g0005 . Gorai.013G010100.1 . Gorai.
009G291800.1 . . . . . . .
. . . . . . . .
.
Vv13g0006 Tc10_g010020 . . . . .

. . . . . . . .
. . . . . .
Vv13g0007 . . . Gorai.009G291900.1 .
. . . . . . . .
. . . . . . .
Vv13g0008 Tc10_g002060 . . . . .
Vv06g0723 . . . . . .
. . . . . . .
Vv13g0009 . Gorai.013G010000.1 . . .
. . . . . . . .
. . . . . . .
Vv13g0010 . Gorai.013G009900.1 . Gorai.
009G292000.1 . . . . . . .
. . . . . . . .
.
Vv13g0011 . Gorai.013G006900.1 . Gorai.
009G292100.1 . . . . . . .
. . . . . . . .
.
Vv13g0012 . . . . . . .
. . . . . . . .
. . . . .
Vv13g0013 . . . . . . .
. . . . . . . .
. . . . .
Vv13g0014 . Gorai.013G019300.1 Gorai.011G283400.1
. Gorai.010G198800.1 . . . .
. . . . . . . .
. . .
Vv13g0015 . . . . . Gorai.
012G075900.1 . . . . . . .
. . . . . . .
Vv13g0016 . . . . . . .
. . . . . . . .
. . . . .
Vv13g0017 . . Gorai.011G283500.1 .
Gorai.010G198700.1 . Vv06g0735 Tc09_g030170 .
Gorai.001G115400.1 Gorai.004G007000.1 .
Gorai.003G123600.1 . . . . . .
.
Vv13g0018 . . . . . . .
. . . . . . . .
. . . . .
Vv13g0019 . Gorai.013G019200.1 Gorai.011G283600.1
Gorai.009G322100.1 . . . . .
. . . . . . . .
. . .
Vv13g0020 . Gorai.013G019000.1 Gorai.011G283700.1
. . Gorai.012G076000.1 Vv06g0737
Tc09_g030150 . . . . Gorai.003G123400.1

. . . . . . .
Vv13g0021 . . Gorai.011G283800.1 . .
. . . . . . . .
. . . . . . .
Vv13g0022 Tc10_g001900 Gorai.013G018900.1 Gorai.
011G283900.1 . . Gorai.012G076100.1 Vv08g1125
Tc05_g002780 Gorai.006G181400.1 Gorai.002G262100.1
. Gorai.012G112900.1 . . . .
. . . .
Vv13g0023 . . . . . . .
. . . . . . . .
. . . . .
Vv13g0024 . . . . . Gorai.
012G076200.1 . . . . . . .
. . . . . . .
Vv13g0025 . . . Gorai.009G322200.1 .
. . . . . . . .
Vv06g0738 Tc09_g030140 . Gorai.001G115500.1
. . .
Vv13g0026 Tc10_g001670 . . . . .
. . . . . . . .
. . . . . .
Vv13g0027 Tc10_g001660 . . . . .
. . . . . . . .
. . . . . .
Vv13g0028 Tc10_g001620 . . Gorai.009G322300.1
. . . . . . . .
. Vv06g0740 Tc09_g030120 Gorai.013G260600.1
Gorai.001G115600.1 . . .
Vv13g0029 Tc10_g001610 . . . . .
. . . . . . . .
. . . . . .
Vv13g0030 . . . . . . .
. . . . . . . .
. . . . .
Vv13g0031 . . . . . Gorai.
012G076300.1 Vv08g1137 Tc05_g002660 . .
Gorai.002G148600.1 . . . . . .
. . .
Vv13g0032 . . Gorai.011G285700.1 . .
Gorai.012G076400.1 Vv08g1139 Tc05_g002630 .
. . Gorai.012G113200.1 . . .
. . . . .
Vv13g0033 . . Gorai.011G285800.1 . .
. . . . . . . .
. . . . . . .
Vv13g0034 . Gorai.013G018700.1 . . .
Gorai.012G076700.1 Vv08g1140 Tc05_g002620
Gorai.006G181800.1 . . . Gorai.007G378800.1
. . . . . . .

Vv13g0035 . . . . . . .
. . . . . . . .
. . . . .
Vv13g0036 . . . . . . .
. . . . . . . .
. . . . .
Vv13g0037 . . . . . . .
. . . . . . . .
. . . . .
Vv13g0038 . . . . . . .
. . . . . . . .
. . . . .
Vv13g0039 . . . . . . .
. . . . . . . .
. . . . .
Vv13g0040 . . Gorai.011G286200.1 . .
. . . . . . . .
. . . . . . .
Vv13g0041 . Gorai.013G018400.1 Gorai.011G286300.1
. Gorai.010G198400.1 Gorai.012G076800.1 .
. . . . . . . .
. . . . .
Vv13g0042 . . . . . . .
. . . . . . . .
. . . . .
Vv13g0043 . . . . Gorai.010G198300.1
. . . . . . . .
. . . . . . .
Vv13g0044 . . . . . . .
. . . . . . . .
. . . . .
Vv13g0045 Tc10_g009860 . . . . .
. . . . . . . .
. . . . . .
Vv13g0046 . . . . . . .
. . . . . . . .
. . . . .
Vv13g0047 . Gorai.013G018300.1 . . .
. Vv08g1150 Tc05_g002500 . . .
Gorai.012G114000.1 . . . . .
. . .
Vv13g0048 . . . . . . .
. . . . . . . .
. . . . .
Vv13g0049 . . . . . . .
. . . . . . . .
. . . . .
Vv13g0050 . . . . . . .
. . . . . . . .
. . . . .

Vv13g0051 . . . . . . .
. . . . . . . .
. . . . .
Vv13g0052 . . . . . . .
. . . . . . . .
. . . . .
Vv13g0053 . . . . . . .
. . . . . . . .
. . . . .
Vv13g0054 . . . . . . .
. . . . . . . .
. . . . .
Vv13g0055 Tc10_g001560 . . . Gorai.
010G198200.1 . . . . . . .
. . . . . . . .
Vv13g0056 Tc10_g001550 . Gorai.011G287800.1 .
Gorai.010G198100.1 . . . . .
. . . Vv06g0772 Tc09_g029920 .
. . Gorai.009G092100.1 .
Vv13g0057 Tc10_g001540 . Gorai.011G287900.1 .
. . . . . . . .
. Vv06g0773 Tc09_g029910 . . .
. Gorai.006G106600.1
Vv13g0058 Tc10_g001510 . . . Gorai.
010G198000.1 Gorai.012G076900.1 Vv08g1152 . .
. . . . Vv06g0779
Tc09_g029880 . . . Gorai.009G091700.1
Gorai.006G106400.1
Vv13g0059 Tc10_g001480 . Gorai.011G288000.1 .
Gorai.010G197900.1 . Vv08g0345 . .
. . . . Vv06g0781
Tc09_g029860 . . . Gorai.009G091400.1 .
Vv13g0060 Tc10_g001460 . . . Gorai.
010G197800.1 . . . . . . .
. . . . . . . .
Vv13g0061 Tc10_g001450 Gorai.013G018100.1 Gorai.
011G288100.1 . Gorai.010G197700.1 . . .
. . . . . . . .
. . . .
Vv13g0062 Tc10_g001430 Gorai.013G018000.1 Gorai.
011G288400.1 . . . Vv08g0348 . .
. . . . . . . .
. . .
Vv13g0063 Tc10_g001420 . . . . .
. . . . . . . .
. . . . . .
Vv13g0064 . . Gorai.011G288600.1 . .
. . . . . . . .
. . . . . . .
Vv13g0065 Tc10_g001410 . Gorai.011G289100.1

Gorai.009G322600.1 . . . . . .
. . . Vv06g0784 Tc09_g029790 .
. . Gorai.009G091300.1 .
Vv13g0066 Tc10_g001400 Gorai.013G017700.1 Gorai.
011G289200.1 . Gorai.010G196600.1 . Vv08g0350
Tc05_g002430 . . . Gorai.012G114500.1
. . . . . . . .
Vv13g0067 Tc10_g001390 . Gorai.011G289300.1 .
. Gorai.012G077000.1 . . . .
. . . . . . . .
. .
Vv13g0068 Tc10_g001380 Gorai.013G017600.1 Gorai.
011G289400.1 . Gorai.010G196500.1 . . .
. . . . . Vv06g0785
Tc09_g029780 . Gorai.001G116400.1 Gorai.004G007300.1
Gorai.009G091200.1 Gorai.006G106300.1
Vv13g0069 . . . . . . .
. . . . . . . .
. . . . .
Vv13g0070 . . . . . . .
. . . . . . . .
. . . . .
Vv13g0071 . . . . . . .
. . . . . . . .
. . . . .
Vv13g0072 Tc10_g001370 . Gorai.011G289500.1 .
. . . . . . . .
. . . . . . . .
Vv13g0073 Tc10_g001360 . Gorai.011G289600.1 .
. . Vv08g0357 Tc05_g002310 .
Gorai.002G262800.1 . Gorai.012G115000.1 .
Vv06g0788 Tc09_g029750 . Gorai.001G116500.1 .
. Gorai.006G106100.1
Vv13g0074 Tc10_g001350 . . . .
Gorai.012G077100.1 . . . . . .
. . . . . . . .
Vv13g0075 . . . . . .
Vv08g0360 Tc05_g002290 . . . Gorai.
012G115100.1 . . . . . . .
.
Vv13g0076 Tc10_g001340 Gorai.013G017500.1 . .
. . . . . . . .
. . . . . . . .
Vv13g0077 Tc10_g001320 . . . .
Gorai.012G077500.1 . . . . . .
. Vv06g0798 Tc09_g029660 . Gorai.
001G116900.1 . . Gorai.006G105300.1
Vv13g0078 . . . . . . .
. . . . . . . .
. . . . .

Vv13g0079 . . . . . . .
. . . . . . . .
. . . . .
Vv13g0080 Tc10_g001300 . . . . .
. . . . . . . .
. . . . .
Vv13g0081 Tc10_g001290 . . . Gorai.
010G196300.1 . . . . . . .
. . . . . . . .
Vv13g0082 Tc10_g001280 . . . . .
. . . . . . . .
. . . . . .
Vv13g0083 . . . . . . .
. . . . . . . .
. . . . .
Vv13g0084 Tc10_g001270 . . . .
Gorai.012G077700.1 . . . . . .
. Vv06g0801 . . . . .
.
Vv13g0085 Tc10_g001260 Gorai.013G017300.1 .
Gorai.009G322700.1 Gorai.010G196100.1 Gorai.012G077900.1
. . . . . . .
Vv06g0804 . . . . . .
Vv13g0086 Tc10_g001250 Gorai.013G017200.1 . .
. . . . . . . .
. . . . . . . .
Vv13g0087 Tc10_g001240 . . . .
Gorai.012G078000.1 . . . . . .
. . . . . . . .
Vv13g0088 Tc10_g001230 . . . .
Gorai.012G078100.1 . . . . . .
. . . . . . . .
Vv13g0089 Tc10_g001220 . . . .
Gorai.012G078200.1 . . . . . .
. . . . . . . .
Vv13g0090 . . . . . . .
. . . . . . . .
. . . . .
Vv13g0091 . . . . . . .
. . . . . . . .
. . . . .
Vv13g0092 . . . . . . .
. . . . . . Vv06g0813
. . . . . Gorai.003G147200.1
Vv13g0093 Tc10_g001190 Gorai.013G017100.1 . .
. Gorai.012G078300.1 . . . .
. . . . . . . .
. .
Vv13g0094 . . . . . . .
. . . . . . . .

. . . . .
Vv13g0095 . . . . . . .
. . . . . . . .
. . . . .
Vv13g0096 Tc10_g001180 . . . . .
. . . . . . . .
. . . . . .
Vv13g0097 Tc10_g001170 Gorai.013G017000.1 . .
. . . . . . . .
. . . . . . . .
Vv13g0098 Tc10_g001160 . Gorai.011G290800.1 .
. . . . . . . .
. . . . . . . .
Vv13g0099 Tc10_g001150 . . . . .
. . . . . . . .
. . . . . .
Vv13g0100 Tc10_g001140 . . . . .
. . . . . . . .
. . . . . .
Vv13g0101 Tc10_g001130 . . . . .
. . . . . . .
Vv06g0821 . . . . Gorai.009G112300.1
.
Vv13g0102 Tc10_g001120 . . . Gorai.
010G195800.1 . . . . . . .
. . . . . . . .
Vv13g0103 Tc10_g001110 . . . .
Gorai.012G078400.1 . . . . . .
. . . . . . . .
Vv13g0104 Tc10_g001100 . Gorai.011G291100.1 .
. . . . . . . .
. . . . . . . .
Vv13g0105 Tc10_g001090 . . . Gorai.
010G195700.1 Gorai.012G078500.1 . . . .
. . . . . . . .
. .
Vv13g0106 . . . . . . .
. . . . . . . .
. . . . .
Vv13g0107 . . . . . . .
. . . . . . . .
. . . . .
Vv13g0108 . . . . . . .
. . . . . . . .
. . . . .
Vv13g0109 . . . . . . .
. . . . . . . .
. . . . .
Vv13g0110 . . . . . . .
. . . . . . . .

.	.	.	.	.
Vv13g0111	Tc10_g001070	Gorai.013G016800.1	.	.
Gorai.010G195600.1	.	Vv08g0366
Tc05_g002240	Gorai.006G183400.1	.	.	.	.
.	.	.	.	.	.	.
Vv13g0112	Tc10_g001060	.	.	.	.
Gorai.012G078600.1	.	.	.	.	.	.
.	.	.	.	.	.	.	.
Vv13g0113	Tc10_g001050	.	.	.	.
Gorai.012G078700.1	.	.	.	.	.	.
.	.	.	.	.	.	.	.
Vv13g0114	Tc10_g001040	.	.	.	Gorai.
010G195500.1	.	.	.	.	.	.	.
.	.	.	.	.	.	.	.
Vv13g0115	Tc10_g001030	.	.	.	Gorai.
010G195400.1	Gorai.012G078800.1	.	.	.	.
.	.	.	.	.	.	.	.
.	.
Vv13g0116	.	.	.	.	.	.	.
.	.	.	.	.	.	Vv06g0831
.	.	.	.	.	.
Vv13g0117	Tc10_g001020	.	.	.	.	.
.	.	.	.	.	.	.	.
.	.	.	.	.	.
Vv13g0118	.	.	.	.	.	.	.
.	.	.	.	.	.	.	.
.	.	.	.	.
Vv13g0119	Tc10_g001010	.	.	.	Gorai.
010G195300.1	Gorai.012G079000.1	.	.	.	.
.	.	.	.	.	.	.	.
.	.
Vv13g0120	.	.	.	.	.	.	.
.	.	.	.	.	.	.	.
.	.	.	.	.
Vv13g0121	.	.	.	.	.	.	.
.	.	.	.	.	.	.	.
.	.	.	.	.
Vv13g0122	.	.	.	.	.	.	.
.	.	.	.	.	.	.	.
.	.	.	.	.
Vv13g0123	Tc10_g001000	.	.	Gorai.009G322800.1
.	Gorai.012G079300.1	.	.	.	.
.	.	.	Vv06g0839	.	.	.
.	.	.
Vv13g0124	.	.	.	.	.	.	.
.	.	.	.	.	.	.	.
.	.	.	.	.
Vv13g0125	Tc10_g000990	.	Gorai.011G291500.1	.
Gorai.010G195100.1	.	.	.	.	.
.	.	.	Vv06g0844	.	.	.

. . .
Vv13g0126 Tc10_g000970 . . . .
Gorai.012G079500.1 . . . . . .
. . . . . . . .
Vv13g0127 Tc10_g000960 . . . .
Gorai.012G079600.1 . . . . . .
. . . . . . . .
Vv13g0128 . . . . . . .
. . . . . . . .
. . . . .
Vv13g0129 Tc10_g000950 . . . . .
. . . . . . . .
. . . . . .
Vv13g0130 Tc10_g000890 . . . Gorai.
010G195000.1 . Vv08g0368 Tc05_g002230 . .
. Gorai.012G115400.1 . . . .
. . . .
Vv13g0131 Tc10_g000870 . . Gorai.009G323000.1
Gorai.010G194800.1 . . . . .
. . . . . . . .
. .
Vv13g0132 Tc10_g000860 . . . Gorai.
010G194700.1 . . . . . . .
. . . . . . . .
Vv13g0133 . . . . . . .
. . . . . . . .
. . . . .
Vv13g0134 . . . . . . .
. . . . . . . .
. . . . .
Vv13g0135 Tc10_g000830 . . . Gorai.
010G194300.1 . . . . . . .
. . . . . . . .
Vv13g0136 . . . . . . .
. . . . . . . .
. . . . .
Vv13g0137 . . . . . .
Vv06g0868 . . . . . Gorai.
003G150300.1 . . . . . . .
Vv13g0138 Tc10_g000820 . . . Gorai.
010G194100.1 . . . . . . .
. . . . . . . .
Vv13g0139 Tc10_g000810 . . . Gorai.
010G193900.1 . . . . . . .
. . . . . . . .
Vv13g0140 . . . . . . .
. . . . . . . .
. . . . .
Vv13g0141 Tc10_g000800 . . . Gorai.
010G193800.1 . . . . . . .

. . . . . . . .
Vv13g0142 Tc10_g000790 Gorai.013G016600.1 . .
Gorai.010G193700.1 . . . . .
. . . . . . . .
. .
Vv13g0143 . . . . . . .
. . . . . . . .
. . . . .
Vv13g0144 . . . . . . .
. . . . . . . .
. . . . .
Vv13g0145 . Gorai.013G014500.1 . . .
. . . . . . . .
. . . . . . .
Vv13g0146 . . . . . . .
. . . . . . . .
. . . . .
Vv13g0147 . . . . . . .
. . . . . . . .
. . . . .
Vv13g0148 . . . . . . .
. . . . . . . .
. . . . .
Vv13g0149 . . . . . . .
. . . . . . . .
. . . . .
Vv13g0150 Tc10_g000740 . . . . .
. . . . . . . .
. . . . . .
Vv13g0151 . . . . . . .
. . . . . . . .
. . . . .
Vv13g0152 . . . . . . .
. . . . . . . .
. . . . .
Vv13g0153 Tc10_g000730 . Gorai.011G292600.1 .
Gorai.010G193300.1 . . . . .
. . . . . . . .
. .
Vv13g0154 . . . . . . .
. . . . . . . .
. . . . .
Vv13g0155 . . . . Gorai.010G193200.1
. . . . . . . .
. . . . . . .
Vv13g0156 . . . . . . .
. . . . . . . .
. . . . .
Vv13g0157 . . . . . . .
. . . . . . . .

. . . . .
Vv13g0158 Tc10_g000660 . . . Gorai.
010G192900.1 . . . . . . .
. . . . . . . .
Vv13g0159 . . . . . . .
. . . . . . . .

. . . . .
Vv13g0160 Tc10_g000640 . . . Gorai.
010G192600.1 Gorai.012G079900.1 . . . .
. . . . . . . .

. .
Vv13g0161 Tc10_g000610 . . . .
Gorai.012G080100.1 . . . . . .
. . . . . . . .
Vv13g0162 . . Gorai.011G293300.1 . .
. . . . . . . .

. . . . . . .
Vv13g0163 . . . . . . .
. . . . . . . .

. . . . .
Vv13g0164 . . . . . .
Vv06g0899 . . . . . . .
. . . . . .
Vv13g0165 . . . . . Gorai.
012G016300.1 . . . . . . .
. . . . . . .
Vv13g0166 . . Gorai.011G211500.1 . .
. . . . . . . .

. . . . . . .
Vv13g0167 . . . . . . .
. . . . . . . .

. . . . .
Vv13g0168 . . . . . . .
. . . . . . . .

. . . . .
Vv13g0169 Tc10_g016040 . Gorai.011G211400.1 .
. . . . . . . .

. . . . . . . .
Vv13g0170 . . . . . .
Vv06g0908 Tc09_g004920 . . . . .
. . . . . . .
Vv13g0171 . . Gorai.011G210600.1 .
Gorai.010G217900.1 Gorai.012G015700.1 . . .
. . . . . . . .

. . .
Vv13g0172 . . . . . . .
. . . . . . . .

. . . . .
Vv13g0173 . . . . . . .
. . . . . . . .

. . . . .

Vv13g0174 . . . . . . .

. . . . . . . .

. . . . .

Vv13g0175 . . . . . . .

. . . . . . . .

. . . . .

Vv13g0176 . . . . . . .

. . . . . . . .

. . . . .

Vv13g0177 . . . . . . .

. . . . . . . .

. . . . .

Vv13g0178 . . . . . . .

. . . . . . . .

. . . . .

Vv13g0179 . . Gorai.011G210200.1 . .

Gorai.012G015500.1 . . . . .

. . . . . . . .

.

Vv13g0180 . . . . . . .

. . . . . . . .

. . . . .

Vv13g0181 . . Gorai.011G210100.1 . .

. . . . . . . .

. . . . . . .

Vv13g0182 . Gorai.013G008100.1 . . .

. . . . . . . .

. . . . . . .

Vv13g0183 . . . . . . .

. . . . . . . .

. . . . .

Vv13g0184 . . . . . Gorai.

012G082500.1 . . . . . . .

. . . . . . .

Vv13g0185 . . . . . . .

. . . . . . . .

. . . . .

Vv13g0186 . . . . . . .

. . . . . . . .

. . . . .

Vv13g0187 . . . . . .

Vv06g0923 . . . . . . .

. . . . . .

Vv13g0188 . . . . . . .

. . . . . . . .

. . . . .

Vv13g0189 . Gorai.013G007300.1 . . .

. Vv06g0924 . . . . .

. . . . . . . .

Vv13g0190 . Gorai.013G006900.1 . . .
Gorai.012G014700.1 . . . . .
. . . . . . . .
.
Vv13g0191 Tc10_g016160 . . . . .
. . . . . . . .
. . . . . .
Vv13g0192 Tc10_g016170 . . . . .
. . . . . . . .
. . . . . .
Vv13g0193 Tc10_g016240 . . . . .
. . . . . . . .
. . . . . .
Vv13g0194 . . . . . . .
. . . . . . . .
. . . . .
Vv13g0195 . . . . . . .
. . . . . . . .
. . . . .
Vv13g0196 . . . . . . .
. . . . . . . .
. . . . .
Vv13g0197 . . . . . . .
. . . . . . . .
. . . . .
Vv13g0198 . . . . . . .
. . . . . . . .
. . . . .
Vv13g0199 . . . . . . .
. . . . . . . .
. . . . .
Vv13g0200 . . . . . . .
. . . . . . . .
. . . . .
Vv13g0201 . . . . . . .
. . . . . . . .
. . . . .
Vv13g0202 . . . . . . .
. . . . . . . .
. . . . .
Vv13g0203 . . . . . . .
. . . . . . . .
. . . . .
Vv13g0204 Tc10_g016280 . . . Gorai.
010G218500.1 . . . . . . .
. . . . . . . .
Vv13g0205 . . . . . . .
. . . . . . . .
. . . . .
Vv13g0206 Tc10_g016290 . . . .

Gorai.012G014600.1 . . . . . .
. . . . . . . .
Vv13g0207 Tc10_g016300 . Gorai.011G209400.1 .
Gorai.010G218600.1 . . . . .
. . . . . . . .
. .
Vv13g0208 Tc10_g016310 . Gorai.011G209200.1 .
Gorai.010G218700.1 Gorai.012G014500.1 . .
. . . . . . . .
. . . .
Vv13g0209 Tc10_g016320 . . . . .
. . . . . . . .
. . . . . .
Vv13g0210 . . . . . . .
. . . . . . . .
. . . . .
Vv13g0211 Tc10_g016330 Gorai.013G007000.1 Gorai.
011G209100.1 . . Gorai.012G014400.1 . .
. . . . . . . .
. . . .
Vv13g0212 Tc10_g016390 Gorai.013G007200.1 Gorai.
011G208100.1 . . . . . . .
. . . . . . . .
. .
Vv13g0213 Tc10_g016400 . Gorai.011G208000.1 .
. . . . . . . .
. . . . . . . .
Vv13g0214 . . . . . . .
. . . . . . . .
. . . . .
Vv13g0215 . . . . . . .
. . . . . . . .
. . . . .
Vv13g0216 Tc10_g016410 . . . . .
. . . . . . . .
. . . . . .
Vv13g0217 Tc10_g016420 . Gorai.011G207900.1 .
. . Vv06g0925 . . . .
. . . . . . . .
.
Vv13g0218 . . . . . . .
. . . . . . . .
. . . . .
Vv13g0219 . . . . . . .
. . . . . . . .
. . . . .
Vv13g0220 . Gorai.013G006800.1 . . .
. . . . . . . .
. . . . . . .
Vv13g0221 . . . . . . .

.	.	.	.	.	.	.	.
.	.	.	.	.
Vv13g0222	.	.	.	.	.	.	.
.	.	.	.	.	.	.	.
.	.	.	.	.
Vv13g0223	.	.	.	.	.	.	.
.	.	.	.	.	.	.	.
.	.	.	.	.
Vv13g0224	Tc10_g014960	Gorai.013G004700.1	.	.
.	Gorai.012G017600.1	Vv08g0899
Tc05_g026640	.	.	.	.	.	.	.
.	.	.	.	.
Vv13g0225	.	.	.	.	.	.	.
.	.	.	.	.	.	.	.
.	.	.	.	.
Vv13g0226	.	.	.	.	.	Gorai.
012G066600.1	.	.	.	.	.	.	.
.	.	.	.	.	.	.
Vv13g0227	.	.	.	.	.	.	.
.	.	.	.	.	.	.	.
.	.	.	.	.
Vv13g0228	.	Gorai.013G002900.1	.	.	.
.	Vv08g0873	.	.	.	.	.
.	.	.	.	.	.	.	.
Vv13g0229	.	.	.	.	.	.	.
.	.	.	.	.	.	.	.
.	.	.	.	.
Vv13g0230	Tc10_g016530	.	.	.	Gorai.
010G219100.1	.	.	.	.	.	.	.
.	.	.	.	.	.	.	.
Vv13g0231	Tc10_g016560	.	.	.	.
Gorai.012G014000.1	.	.	.	.	.	.
.	.	.	.	.	.	.	.
Vv13g0232	Tc10_g016570	Gorai.013G008300.1	.	.
Gorai.010G219200.1	Gorai.012G013900.1	.	.
.	.	.	.	.	.	.	.
.	.	.	.
Vv13g0233	Tc10_g016580	.	Gorai.011G206700.1	.
.	.	.	.	.	.	.	.
.	.	.	.	.	.	.	.
Vv13g0234	Tc10_g016590	Gorai.013G008400.1	.	.
.	.	.	.	.	.	.	.
.	.	.	.	.	.	.	.
Vv13g0235	Tc10_g016600	.	.	.	.	.
.	.	.	.	.	.	.	.
.	.	.	.	.	.
Vv13g0236	.	.	.	.	.	.	.
.	.	.	.	.	.	.	.
.	.	.	.	.
Vv13g0237	Tc10_g015000	.	Gorai.011G206500.1	.

.        .        .        .        .        .        .        .
.        .        .        .        .        .        .        .
Vv13g0238        Tc10_g016620     .        .        .        .        .
.        .        .        .        .        .        .        .
.        .        .        .        .        .
Vv13g0239        .        .        Gorai.011G240500.1        .        .
Gorai.012G013600.1        .        .        .        .        .
.        .        .        .        .        .        .        .
.
Vv13g0240        Tc10_g016640     .        .        .        .        .
.        .        .        .        .        .        .        .
.        .        .        .        .        .
Vv13g0241        Tc10_g016650     Gorai.013G008500.1        Gorai.
011G206300.1     .        .        Gorai.012G013500.1        .        .
.        .        .        .        .        .        .        .
.        .        .        .
Vv13g0242        Tc10_g016660     Gorai.013G008700.1        .        .
.        .        .        .        .        .        .        .
.        Vv06g0943        .        .        .        .        .
.
Vv13g0243        Tc10_g016670     .        Gorai.011G203600.1        .
.        .        .        .        .        .        .        .
.        .        .        .        .        .        .        .
Vv13g0244        .        .        .        .        .        .        .
.        .        .        .        .        .        .        .
.        .        .        .        .
Vv13g0245        Tc10_g016690     .        .        .        .        .
.        .        .        .        .        .        .        .
.        .        .        .        .        .
Vv13g0246        Tc10_g004690     .        .        Gorai.009G315800.1
.        .        .        .        .        .        .        .
.        .        .        .        .        .        .        .
Vv13g0247        Tc10_g004680     Gorai.013G024500.1        .        .
.        .        .        .        .        .        .        .
.        .        .        .        .        .        .        .
Vv13g0248        .        .        .        .        .        .        .
.        .        .        .        .        .        .        .
.        .        .        .        .
Vv13g0249        Tc10_g004670     .        Gorai.011G241800.1        .
.        Gorai.012G010900.1        .        .        .        .
.        .        .        .        .        .        .        .
.        .
Vv13g0250        .        .        .        .        .        .        .
.        .        .        .        .        .        .        .
.        .        .        .        .
Vv13g0251        Tc10_g004660     .        Gorai.011G241900.1        .
.        .        .        .        .        .        .        .
.        .        .        .        .        .        .        .
Vv13g0252        Tc10_g004650     .        .        .        .        .
.        .        .        .        .        .        .

Vv06g0429 Tc09_g032930 . . . Gorai.
009G103700.1 .
Vv13g0253 Tc10_g004640 . . . Gorai.
010G203600.1 . . . . . . .
. . . . . . . .
Vv13g0254 Tc10_g004630 . Gorai.011G242000.1
Gorai.009G315900.1 . . . . . .
. . . . . . . .
. .
Vv13g0255 Tc10_g004610 Gorai.013G024400.1 Gorai.
011G242200.1 Gorai.009G316000.1 . . . .
. . . . . . . .
. . . .
Vv13g0256 Tc10_g004590 Gorai.013G024300.1 . .
. . . . . . . .
. . . . . . . .
Vv13g0257 . . . . . . .
. . . . . . . .
. . . . .
Vv13g0258 Tc10_g004580 Gorai.013G024200.1 . .
. . . . . . . .
. Vv06g0432 . . . . .
.
Vv13g0259 . . . . . . .
. . . . . . . .
. . . . .
Vv13g0260 Tc10_g004570 . Gorai.011G242300.1 .
Gorai.010G203500.1 . Vv08g0886 . .
. . Gorai.012G100800.1 . . .
. . . . .
Vv13g0261 Tc10_g004560 . Gorai.011G242400.1 .
. . . . . . . .
. . . . . . . .
Vv13g0262 Tc10_g004540 . Gorai.011G242500.1 .
Gorai.010G203400.1 . . . . .
. . . . . . . .
. .
Vv13g0263 Tc10_g004530 . Gorai.011G242600.1 .
. . . . . . . .
. . . . . . . .
Vv13g0264 . Gorai.013G024100.1 Gorai.011G242700.1
. Gorai.010G203100.1 . . . .
. . . . . . . .
. . .
Vv13g0265 . . . . . . .
. . . . . . . .
. . . . .
Vv13g0266 . . . . . . .
. . . . . . . .
. . . . .

Vv13g0267 . . . . . Gorai.
012G066900.1 . . . . . . .
. . . . . . .
Vv13g0268 . . . . . . .
. . . . . . . .
. . . . .
Vv13g0269 Tc10_g004500 . Gorai.011G242800.1 .
. . . . . . . .
. . . . . . . .
Vv13g0270 . . . . . . .
. . . . . . . .
. . . . .
Vv13g0271 Tc10_g004440 Gorai.013G023900.1 . .
. Gorai.012G067300.1 . . . .
. . . . . . . .
. .
Vv13g0272 Tc10_g004430 . Gorai.011G244700.1 .
Gorai.010G203000.1 . . . . .
. . . . . . . .
. .
Vv13g0273 Tc10_g004420 . . . .
Gorai.012G067400.1 . . . . . .
. . . . . . . .
Vv13g0274 . . . . . . .
. . . . . . . .
. . . . .
Vv13g0275 Tc10_g004410 . . . . .
. . . . . . . .
. . . . . .
Vv13g0276 . . . . . . .
. . . . . . . .
. . . . .
Vv13g0277 . . . . . . .
. . . . . . . .
. . . . .
Vv13g0278 . . . . . . .
. . . . . . Vv06g0456
Tc09_g032510 . . . Gorai.009G102800.1
.
Vv13g0279 Tc10_g004370 Gorai.013G023400.1 . .
. . . . . . . .
. . . . . . . .
Vv13g0280 . . . . . . .
. . . . . . . .
. . . . .
Vv13g0281 . . Gorai.011G244900.1 . .
Gorai.012G067500.1 . . . . .
. . . . . . . .
.
Vv13g0282 . . Gorai.011G245000.1 Gorai.

009G316400.1 . Gorai.012G067600.1 . . .
. . . . . . . .
. . .
Vv13g0283 Tc10_g004310 . . . Gorai.
010G202900.1 Gorai.012G067700.1 . . . .
. . . . . . . .
. .
Vv13g0284 . . Gorai.011G245100.1 . .
. . . . . . . .
. . . . . . .
Vv13g0285 . . . . Gorai.010G202800.1
. . . . . . . .
Vv06g0463 Tc09_g032420 . Gorai.001G107300.1
. Gorai.009G102300.1 .
Vv13g0286 . . . . . . .
. . . . . . . .
. . . . .
Vv13g0287 . . . Gorai.009G316600.1 .
. . . . . . . .
Vv06g0465 Tc09_g032410 . Gorai.001G107400.1
. Gorai.009G102200.1 Gorai.003G137500.1
Vv13g0288 . . Gorai.011G245300.1 Gorai.
009G316700.1 . . . . . . .
. . . . . . . .
.
Vv13g0289 . . Gorai.011G245500.1 .
Gorai.010G202700.1 . . . . . .
. . . . . . . .
.
Vv13g0290 . . Gorai.011G245700.1 . .
Gorai.012G068000.1 . . . . .
. . . . . . . .
.
Vv13g0291 Tc10_g000510 . Gorai.011G245900.1
Gorai.009G316800.1 . . . . . .
. . . . . . . .
. .
Vv13g0292 Tc10_g000500 . Gorai.011G246000.1 .
. . . . . . . .
. Vv06g0468 Tc09_g032380 . . .
. Gorai.003G137200.1
Vv13g0293 . . Gorai.011G246100.1 Gorai.
009G316900.1 . . . . . . .
. . . . . . . .
.
Vv13g0294 . . Gorai.011G246200.1 . .
. Vv08g0891 . Gorai.006G177600.1
Gorai.002G252300.1 . . . . . .
. . . .
Vv13g0295 . . . . Gorai.010G202600.1

. . . . . . . .
. . . . . . .
Vv13g0296 . . . . . . .
. . . . . . . .
. . . . .
Vv13g0297 . . . . . . .
. . . . . . . .
. . . . .
Vv13g0298 . . Gorai.011G246300.1 . .
. . . . . . . .
. . . . . . .
Vv13g0299 . . Gorai.011G246400.1 Gorai.
009G317000.1 . . . . . . .
. . . . . . . .
.
Vv13g0300 . . . . . . .
. . . . . . . .
. . . . .
Vv13g0301 . . Gorai.011G246500.1 . .
. . . . . . . .
. . . . . . .
Vv13g0302 . . Gorai.011G246600.1 . .
Gorai.012G068100.1 . . . . .
. . . . . . . .
.
Vv13g0303 . . Gorai.011G247000.1 . .
. . . . . . . .
. . . . . . .
Vv13g0304 . . . . . . .
. . . . . . . .
. . . . .
Vv13g0305 . . . . . . .
. . . . . . . .
. . . . .
Vv13g0306 . . . . . . .
. . . . . . . .
. . . . .
Vv13g0307 . . . . . . .
. . . . . . . .
. . . . .
Vv13g0308 . . . . . . .
. . . . . . . .
. . . . .
Vv13g0309 . . . . . . .
. . . . . . . .
. . . . .
Vv13g0310 . . Gorai.011G247700.1 . .
. . . . . . . .
. . . . . . .
Vv13g0311 . . . . . . .

```
        .       .       .       .       .       .       .       .
        .       .       .       .       .
Vv13g0312       Tc10_g004240    .       .       Gorai.009G317700.1
        .       .       .       .       .       .       .       .
        .       .       .       .       .       .       .       .
Vv13g0313       Tc10_g004230    .       Gorai.011G252900.1      .
        .       Gorai.012G069000.1      .       .       .       .
        .       .       .       .       .       .       .       .
        .       .
Vv13g0314       Tc10_g004220    .       .       .       .
Gorai.012G069100.1      .       .       .       .       .       .
        .       .       .       .       .       .       .       .
Vv13g0315       Tc10_g004200    .       Gorai.011G253100.1      .
        .       .       .       .       .       .       .       .
        .       Vv06g0479       Tc09_g032280    Gorai.013G265400.1
        .       .       Gorai.009G101700.1      Gorai.003G136600.1
Vv13g0316       .       .       .       .       .       .       .
        .       .       .       .       .       .       .       .
        .       .       .       .       .
Vv13g0317       Tc10_g003910    .       Gorai.011G253200.1
Gorai.009G317800.1      .       .       .       .       .       .
        .       .       .       .       .       .       .       .
        .       .
Vv13g0318       .       .       .       .       .       .       .
        .       .       .       .       .       .       .       .
        .       .       .       .       .
Vv13g0319       .       .       .       .       .       .       .
        .       .       .       .       .       .       .       .
        .       .       .       .       .
Vv13g0320       .       .       .       .       .       .       .
        .       .       .       .       .       .       .       .
        .       .       .       .       .
Vv13g0321       .       .       .       .       .       .       .
        .       .       .       .       .       .       .       .
        .       .       .       .       .
Vv13g0322       .       .       Gorai.011G253900.1      .       .
        .       .       .       .       .       .       .       .
        .       .       .       .       .       .       .
Vv13g0323       Tc10_g003860    .       Gorai.011G254000.1      .
        .       .       .       .       .       .       .       .
        .       Vv06g0483       Tc09_g032240    .       Gorai.
001G108000.1    .       Gorai.009G101600.1      Gorai.003G136300.1
Vv13g0324       .       .       .       .       .       .       .
        .       .       .       .       .       .       Vv06g0484
        .       .       .       .       .       .
Vv13g0325       .       .       .       .       .       .       .
        .       .       .       .       .       .       .       .
        .       .       .       .       .
Vv13g0326       Tc10_g003850    Gorai.013G023300.1      Gorai.
011G254300.1    .       Gorai.010G202400.1      .       .       .
```

. . . . . . . .
. . . .
Vv13g0327 Tc10_g003840 Gorai.013G023200.1 .
Gorai.009G318200.1 . . Vv08g0923
Tc05_g005000 Gorai.006G175000.1 Gorai.002G254400.1 .
. . Vv06g0488 Tc09_g032210 . .
. Gorai.009G101500.1 .
Vv13g0328 Tc10_g003790 . . . . .
. . . . . . . .
. . . . . .
Vv13g0329 Tc10_g003760 . Gorai.011G255300.1 .
. Gorai.012G069500.1 . . . .
. . . . . . . .
. .
Vv13g0330 Tc10_g003730 Gorai.013G023100.1 Gorai.
011G255400.1 . . . . . . .
. . . . . . . .
. .
Vv13g0331 . . . . . . .
. . . . . . . .
. . . . .
Vv13g0332 . . . . . . .
. . . . . . . .
. . . . .
Vv13g0333 Tc10_g003700 Gorai.013G022900.1 . .
. Gorai.012G069600.1 Vv08g0925
Tc05_g004990 . Gorai.002G254800.1 . . .
Vv06g0496 Tc09_g032130 Gorai.013G264800.1 .
. . Gorai.003G135600.1
Vv13g0334 . . . . . . .
. . . . . . . .
. . . . .
Vv13g0335 . . . . . . .
. . . . . . . .
. . . . .
Vv13g0336 . . . . . . .
. . . . . . . .
. . . . .
Vv13g0337 . . . . . . .
. . . . . . . .
. . . . .
Vv13g0338 Tc10_g003670 . . . . .
. . . . . . . .
. . . . . .
Vv13g0339 . . . . . . .
. . . . . . . .
. . . . .
Vv13g0340 . . . . . Gorai.
012G069700.1 . . . . . . .
. . . . . . .

Vv13g0341 . . . . . . .
. . . . . . . .
. . . . .
Vv13g0342 . . . Gorai.009G318900.1 .
. . . . . . . .
. . . . . . .
Vv13g0343 . . Gorai.011G255700.1 . .
. . . . . . . .
. . . . . . .
Vv13g0344 Tc10_g003640 Gorai.013G022700.1 . .
. . . . . . . .
. Vv06g0506 Tc09_g032060 Gorai.013G264500.1
. . . Gorai.003G135000.1
Vv13g0345 . . . . . . .
. . . . . . . .
. . . . .
Vv13g0346 Tc10_g003630 . . . . .
. . . . . . .
Vv06g0507 Tc09_g032050 Gorai.013G264400.1 .
Gorai.004G004600.1 . Gorai.003G134900.1
Vv13g0347 . . . . . .
Vv08g0934 Tc05_g004880 . Gorai.002G255200.1 .
Gorai.012G102800.1 . . . . .
. . .
Vv13g0348 Tc10_g003620 . . . . .
. . . . . . . .
. . . . . .
Vv13g0349 . . Gorai.011G256000.1 . .
. . . . . . . .
. . . . . . .
Vv13g0350 Tc10_g003610 . Gorai.011G256100.1 .
. Gorai.012G070000.1 . . . .
. . . Vv06g0509 Tc09_g032030 .
. . Gorai.009G100900.1 Gorai.003G134600.1
Vv13g0351 . . . . . . .
. . . . . . . .
. . . . .
Vv13g0352 . . . . . . .
. . . . . . . .
. . . . .
Vv13g0353 . . . . . . .
. . . . . . . .
. . . . .
Vv13g0354 . . . . . . .
. . . . . . . .
. . . . .
Vv13g0355 Tc10_g003590 . Gorai.011G256300.1 .
. . . . . . . .
. . . . . . . .
Vv13g0356 Tc10_g003480 . . . . .

Vv08g0943 . . . . . .
Vv06g0513 Tc09_g031990 Gorai.013G264300.1
Gorai.001G108800.1 . Gorai.009G100700.1 Gorai.
003G134400.1
Vv13g0357 Tc10_g003470 . Gorai.011G256600.1 .
Gorai.010G202100.1 . Vv08g0944
Tc05_g004790 Gorai.006G174000.1 Gorai.002G258500.1 .
. Gorai.007G375600.1 Vv06g0516
Tc09_g031960 . . . Gorai.009G100400.1
Gorai.003G133700.1
Vv13g0358 Tc10_g003460 . . Gorai.009G319000.1
. . Vv08g0945 Tc05_g004780 .
Gorai.002G258400.1 . Gorai.012G103100.1 .
Vv06g0517 Tc09_g031950 . . . Gorai.
009G100300.1 .
Vv13g0359 Tc10_g003450 . Gorai.011G256700.1 .
. . . . . . . .
. . . . . . . .
Vv13g0360 Tc10_g003430 Gorai.013G022600.1 Gorai.
011G256800.1 Gorai.009G319200.1 . . . .
. . . . . Vv06g0519
Tc09_g031930 . Gorai.001G109100.1 . Gorai.
009G100200.1 Gorai.003G133600.1
Vv13g0361 . . . . . . .
. . . . . . . .
. . . . .
Vv13g0362 . . . . . . .
. . . . . . . .
. . . . .
Vv13g0363 . . . . . . .
. . . . . . . .
. . . . .
Vv13g0364 . . . . . . .
. . . . . . . .
. . . . .
Vv13g0365 . . . . . . .
. . . . . . . .
. . . . .
Vv13g0366 . . . . . . .
. . . . . . . .
. . . . .
Vv13g0367 . . . . . . .
. . . . . . . .
. . . . .
Vv13g0368 . . . . . . .
. . . . . . . .
. . . . .
Vv13g0369 . . . . . . .
. . . . . . . .
. . . . .

Vv13g0370 . . . . . . .
. . . . . . . .
. . . . .
Vv13g0371 . . . . . . .
. . . . . . . .
. . . . .
Vv13g0372 . . . . . . .
. . . . . . . .
. . . . .
Vv13g0373 . . . . . . .
. . . . . . . .
. . . . .
Vv13g0374 Tc10_g003330 . . . .
Gorai.012G070100.1 . . . . . .
. . . . . . . .
Vv13g0375 Tc10_g003320 . . . .
Gorai.012G070200.1 . . . . . .
. . . . . . . .
Vv13g0376 . . . . . . .
. . . . . . . .
. . . . .
Vv13g0377 . . Gorai.011G259600.1 . .
. . . . . . . .
. . . . . . .
Vv13g0378 . . . . . . .
. . . . . . . .
. . . . .
Vv13g0379 . . Gorai.011G260000.1 . .
. . . . . . . .
. . . . . . .
Vv13g0380 . . . . . . .
. . . . . . . .
. . . . .
Vv13g0381 . Gorai.013G022500.1 . Gorai.
009G319300.1 . . Vv08g0949 Tc05_g004730
Gorai.006G173700.1 . . Gorai.012G103400.1 .
Vv06g0524 Tc09_g031890 . Gorai.001G109300.1
Gorai.004G004800.1 Gorai.009G100100.1 Gorai.
003G133200.1
Vv13g0382 . . . . . . .
. . . . . . . .
. . . . .
Vv13g0383 . . . . . . .
. . . . . . . .
. . . . .
Vv13g0384 . . . . . . .
. . . . . . . .
. . . . .
Vv13g0385 . . . . . . .
. . . . . . . .

. . . . .
Vv13g0386 . . . . . . .
. . . . . . . .
. . . . .
Vv13g0387 Tc10_g003090 . Gorai.011G262500.1 .
. . . . . . . .
. . . . . . . .
Vv13g0388 Tc10_g003080 . . . Gorai.
010G201500.1 . . . . . . .
. . . . . . . .
Vv13g0389 Tc10_g003070 . . Gorai.009G319600.1
. Gorai.012G070900.1 . . . .
. . . . . . . .
. .
Vv13g0390 . . . . . . .
. . . . . . . .
. . . . .
Vv13g0391 . . . . . . .
. . . . . . . .
. . . . .
Vv13g0392 Tc10_g003060 . Gorai.011G265400.1 .
Gorai.010G201400.1 Gorai.012G071000.1 . .
. . . . . Vv06g0564 .
Gorai.013G263400.1 . . . .
Vv13g0393 . . . . . . .
. . . . . . . .
. . . . .
Vv13g0394 . . . . . . .
. . . . . . Vv06g0566
. . . . . .
Vv13g0395 . Gorai.013G022200.1 Gorai.011G266000.1
. . . Vv08g0967 Tc05_g004480
Gorai.006G172400.1 Gorai.002G257400.1 . Gorai.
012G104400.1 Gorai.007G376300.1 Vv06g0567
Tc09_g031580 . Gorai.001G111100.1 . Gorai.
009G098800.1 Gorai.003G131000.1
Vv13g0396 . Gorai.013G021300.1 Gorai.011G266100.1
. . . Vv08g0973 Tc05_g004440 .
. . . . Vv06g0576
Tc09_g031520 . . . Gorai.009G098300.1
Gorai.003G130700.1
Vv13g0397 Tc10_g000490 . . . . .
. . . . . . . .
. . . . . .
Vv13g0398 . . . . . . .
. . . . . . Vv06g0578
Tc09_g031500 . . . Gorai.009G098200.1
Gorai.003G130500.1
Vv13g0399 . Gorai.013G018300.1 Gorai.011G266200.1
. . . . . . . .

. . . . . . . .
.
Vv13g0400 . . . . . . .
. . . . . . Vv06g0579
Tc09_g031490 . Gorai.001G111600.1 . .
.
Vv13g0401 . . . . . . .
. . . . . . . .
. . . . .
Vv13g0402 . . . . . .
Vv08g0975 Tc05_g004430 . . . .
Gorai.007G376400.1 Vv06g0580 Tc09_g031480 . .
. Gorai.009G098100.1 Gorai.003G130400.1
Vv13g0403 . . . . . . .
. . . . . . . .
. . . . .
Vv13g0404 . . Gorai.011G266300.1 . .
. . . . . . . .
. . . . . . .
Vv13g0405 . . Gorai.011G266400.1 Gorai.
009G319700.1 . . . . . . .
. . . . . . . .
.
Vv13g0406 . . . . . . .
. . . . . . . .
. . . . .
Vv13g0407 . . . . . . .
. . . . . . . .
. . . . .
Vv13g0408 . . . . . . .
. . . . . . . .
. . . . .
Vv13g0409 Tc10_g000830 . . . . .
. . . . . . . .
. . . . . .
Vv13g0410 . . . . . . .
. . . . . . . .
. . . . .
Vv13g0411 . . . . . . .
. . . . . . . .
. . . . .
Vv13g0412 Tc10_g002800 . Gorai.011G266800.1 .
. . . . . . . .
. . . . . . . .
Vv13g0413 Tc10_g002790 . Gorai.011G266900.1 .
Gorai.010G201200.1 . Vv08g0989
Tc05_g004300 Gorai.006G171400.1 . . . .
. . . . . . .
Vv13g0414 . . . . . . .
. . . . . . . .

.	.	.	.	.
Vv13g0415	.	.	Gorai.011G267200.1	.	.
	Gorai.012G071300.1	.	.	.	.	.
	.	.	.	.	.	.	.	.
	.
Vv13g0416	.	.	.	.	.	.	.
	.	.	.	.	.	.	Vv06g0592
	Tc09_g031320	.	.	.	.	.
Vv13g0417	.	.	.	.	.	.	.
	.	.	.	.	.	.	Vv06g0593
	.	.	Gorai.001G112000.1	Gorai.004G005400.1
	Gorai.009G097200.1	Gorai.003G129200.1
Vv13g0418	.	.	.	.	.	.	.
	.	.	.	.	.	.	.	.
	.	.	.	.	.
Vv13g0419	.	.	.	.	.	.
Vv08g0997	Tc05_g004240	.	.	.	Gorai.
012G105400.1	.	.	.	.	.	.	.
	.
Vv13g0420	.	.	.	.	.	.	.
	.	.	.	.	.	.	.	.
	.	.	.	.	.
Vv13g0421	.	.	.	.	.	.	.
	.	.	.	.	.	.	.	.
	.	.	.	.	.
Vv13g0422	Tc10_g000980	.	.	.	.	.
	.	.	.	.	.	.	.	.
	.	.	.	.	.	.
Vv13g0423	.	.	Gorai.011G267600.1	.
Gorai.010G200600.1	.	Vv08g1004	Tc05_g004060	.
	.	.	Gorai.012G106000.1	.	Vv06g0603
	.	.	.	.	.	.
Vv13g0424	Tc10_g001160	.	Gorai.011G267700.1	.
	.	.	.	.	.	.	.	.
	.	.	.	.	.	.	.	.
Vv13g0425	.	.	.	.	.	.	.
	.	.	.	.	.	.	.	.
	.	.	.	.	.
Vv13g0426	.	.	.	.	.	.	.
	.	.	.	.	.	.	.	.
	.	.	.	.	.
Vv13g0427	.	.	.	.	.	.	.
	.	.	.	.	.	.	.	.
	.	.	.	.	.
Vv13g0428	.	.	.	.	.	.	.
	.	.	.	.	.	.	.	.
	.	.	.	.	.
Vv13g0429	.	.	Gorai.011G267800.1	.	.
	.	.	.	.	.	.	.	.
	.	.	.	.	.	.	.

Vv13g0430 . . Gorai.011G267900.1 . .
. . . . . . . .
. . . . . . .
Vv13g0431 . . Gorai.011G268000.1 . .
. Vv08g1008 Tc05_g004020 . . .
. . Vv06g0610 . . . .
. .
Vv13g0432 . . . . . . .
. . . . . . . .
. . . . .
Vv13g0433 . . . . . . .
. . . . . . . .
. . . . .
Vv13g0434 . . . . . . .
. . . . . . . .
. . . . .
Vv13g0435 . . . . . . .
. . . . . . . .
. . . . .
Vv13g0436 . . . . . . .
. . . . . . . .
. . . . .
Vv13g0437 . . . . . . .
. . . . . . . .
. . . . .
Vv13g0438 . . . . . . .
. . . . . . . .
. . . . .
Vv13g0439 . . . . . Gorai.
012G072100.1 . . . . . . .
. . . . . . .
Vv13g0440 Tc10_g001270 . . . . .
. . . . . . .
Vv06g0638 Tc09_g031060 . . . Gorai.
009G096200.1 Gorai.003G128900.1
Vv13g0441 . . . . . . .
. . . . . . Vv06g0639
Tc09_g031050 Gorai.013G261900.1 . . .
Gorai.003G128800.1
Vv13g0442 . . Gorai.011G268300.1 . .
. . . . . . . .
. . . . . . .
Vv13g0443 . . . . . . .
. . . . . . . .
. . . . .
Vv13g0444 . . . . . . .
. . . . . . . .
. . . . .
Vv13g0445 . . Gorai.011G268400.1 . .
Gorai.012G072300.1 . . . . .

. . . . . . . .
.
Vv13g0446 . . Gorai.011G268500.1 .
Gorai.010G200500.1 . . . . . .
. . . . . . . .
.
Vv13g0447 . . . . . . .
. . . . . . . .
. . . . .
Vv13g0448 . . Gorai.011G268700.1 . .
Gorai.012G072600.1 . . . . .
. . . . . . . .
.
Vv13g0449 . . . . . . .
. . . . . . . .
. . . . .
Vv13g0450 . . . . . . .
. . . . . . . .
. . . . .
Vv13g0451 . . . . . . .
. . . . . . . .
. . . . .
Vv13g0452 . . Gorai.011G268900.1 .
Gorai.010G200400.1 . . . . . .
. . . . . . . .
.
Vv13g0453 Tc10_g002650 . Gorai.011G269100.1 .
. Gorai.012G072800.1 . . . .
. . . . . . . .
. .
Vv13g0454 Tc10_g002620 . Gorai.011G269200.1 .
. . Vv08g1021 Tc05_g003910 Gorai.
006G170500.1 . . Gorai.012G107000.1 . .
. . . . . .
Vv13g0455 Tc10_g002600 . Gorai.011G269600.1 .
. Gorai.012G073000.1 . . . .
. . . . . . . .
. .
Vv13g0456 Tc10_g002590 . Gorai.011G269700.1 .
. . Vv08g1028 Tc05_g003840 . .
. Gorai.012G107700.1 . . . .
. . . .
Vv13g0457 . . . . . . .
. . . . . . . .
. . . . .
Vv13g0458 Tc10_g002580 . Gorai.011G269800.1 .
. Gorai.012G073100.1 . . . .
. . . . . . . .
. .
Vv13g0459 Tc10_g002530 . Gorai.011G269900.1 .

```
        .        .       .        .       .        .       .        .
        .        .       .        .       .        .       .        .
Vv13g0460        Tc10_g002450     .       Gorai.011G270500.1        .
        .        .       .        .       .        .       .        .
        .        .       .        .       .        .       .        .
Vv13g0461        .       .        .       .        .       .        .
        .        .       .        .       .        .       .        .
        .        .       .        .       .
Vv13g0462        Tc10_g002430     .       .        .       .        .
        .        .       .        .       .        .       .        .
        .        .       .        .       .        .
Vv13g0463        Tc10_g002410     .       Gorai.011G270900.1        .
        .        .       .        .       .        .       .        .
        .        .       .        .       .        .       .        .
Vv13g0464        Tc10_g002400     .       Gorai.011G271000.1        .
        .        .       .        .       .        .       .        .
        .        .       .        .       .        .       .        .
Vv13g0465        Tc10_g002390     .       Gorai.011G271200.1        .
        .        .       Vv08g1047        Tc05_g003660     .
Gorai.002G255800.1       .        Gorai.012G108300.1       .        .
        .        .       .        .       .        .
Vv13g0466        Tc10_g002380     .       .        .       .        .
        Vv08g1049        Tc05_g003640     Gorai.006G169400.1        .
        .        .       .        .       .        .       .        .
        .        .
Vv13g0467        Tc10_g002370     .       Gorai.011G271400.1        .
        .        Gorai.012G073300.1       .        .       .        .
        .        .       .        .       .        .       .        .
        .        .
Vv13g0468        Tc10_g002350     .       Gorai.011G272000.1        .
        Gorai.010G200300.1        .       .        .       .        .
        .        .       .        .       .        .       .        .
        .        .
Vv13g0469        Tc10_g002340     .       Gorai.011G272100.1        .
        Gorai.010G200200.1        .       .        .       .        .
        .        .       .        .       .        .       .        .
        .        .
Vv13g0470        .       .        .       .        .       .        .
        .        .       .        .       .        .       .        .
        .        .       .        .       .
Vv13g0471        Tc10_g002320     .       Gorai.011G272200.1        .
        Gorai.010G200000.1        .       .        .       .        .
        .        .       .        .       .        .       .        .
        .        .
Vv13g0472        .       .        .       .        .       .        .
        .        .       .        .       .        .       .        .
        .        .       .        .       .
Vv13g0473        Tc10_g002310     .       Gorai.011G272300.1        .
        .        .       .        .       .        .       .        .
        .        .       .        .       .        .       .        .
```

Vv13g0474 . . . . . . .
. . . . . . . .
. . . . .
Vv13g0475 . . . . . . .
. . . . . . . .
. . . . .
Vv13g0476 . . . . . . .
. . . . . . . .
. . . . .
Vv13g0477 . . . . . . .
. . . . . . . .
. . . . .
Vv13g0478 . . . . . . .
. . . . . . . .
. . . . .
Vv13g0479 . . . . . . .
. . . . . . . .
. . . . .
Vv13g0480 . . . . . . .
. . . . . . . .
. . . . .
Vv13g0481 . . . . . . .
. . . . . . . .
. . . . .
Vv13g0482 . . . . . . .
. . . . . . . .
. . . . .
Vv13g0483 . . . . . . .
. . . . . . . .
. . . . .
Vv13g0484 . . . . . . .
. . . . . . . .
. . . . .
Vv13g0485 . . . . . . .
. . . . . . . .
. . . . .
Vv13g0486 . . . . . . .
. . . . . . . .
. . . . .
Vv13g0487 . . . . . . .
. . . . . . . .
. . . . .
Vv13g0488 . . . . . . .
. . . . . . . .
. . . . .
Vv13g0489 . . . . . . .
. . . . . . . .
. . . . .
Vv13g0490 . . . . . . .
. . . . . . . .

.	.	.	.	.
Vv13g0491	.	.	.	.	.	.
Vv08g1053	Tc05_g003600	.	.	.	Gorai.
012G108500.1	.	.	.	.	.	.	.
.
Vv13g0492	.	.	.	.	.	Gorai.
012G073500.1	Vv08g1054	Tc05_g003590	Gorai.006G178100.1
.	.	Gorai.012G108600.1	Gorai.007G377300.1
.	.	.	.	.	.	.
Vv13g0493	.	.	Gorai.011G274100.1	.	.
Gorai.012G073600.1	Vv08g1055	Tc05_g003580
Gorai.006G178200.1	.	.	.	Gorai.007G377400.1
.	.	.	.	.	.	.
Vv13g0494	.	.	Gorai.011G274200.1	.	.
.	.	.	.	.	.	.	.
.	.	.	.	.	.	.
Vv13g0495	.	.	Gorai.011G274300.1	.
Gorai.010G199600.1	.	Vv08g1057	.	Gorai.
006G178300.1	Gorai.002G258900.1	.	.	.	.
.	.	.	.	.	.
Vv13g0496	.	.	Gorai.011G274500.1	.	.
.	.	.	.	.	.	.	.
.	.	.	.	.	.	.
Vv13g0497	.	.	Gorai.011G274600.1	.	.
Gorai.012G073700.1	Vv08g1064	Tc05_g003490
Gorai.006G178800.1	.	.	Gorai.012G109000.1
Gorai.007G377500.1	.	.	.	.	.	.
.
Vv13g0498	.	.	.	.	.	.
Vv04g0160	.	.	.	.	.	.	.
.	.	.	.	.	.
Vv13g0499	.	.	.	.	.	.
Vv04g0159	.	.	.	.	.	.	.
.	.	.	.	.	.
Vv13g0500	.	.	.	.	.	.
Vv04g0158	.	.	.	.	.	.	.
.	.	.	.	.	.
Vv13g0501	.	.	.	.	.	.
Vv04g0157	.	.	.	.	.	.	.
.	.	.	.	.	.
Vv13g0502	.	.	.	.	.	.	.
.	.	.	.	.	.	.	.
.	.	.	.	.
Vv13g0503	.	.	.	.	.	.	.
.	.	.	.	.	.	.	.
.	.	.	.	.
Vv13g0504	.	.	.	.	.	.	.
.	.	.	.	.	.	.	.
.	.	.	.	.
Vv13g0505	.	.	.	.	.	.	.

. . . . . . . .
. . . . .
Vv13g0506 . . . . . .
Vv04g0156 . . . . . . .
. . . . . .
Vv13g0507 . . . . . . .
. . . . . . . .
. . . . .
Vv13g0508 . . . . . .
Vv04g0155 . . . . . . .
. . . . . .
Vv13g0509 . . . . . . .
. . . . . . . .
. . . . .
Vv13g0510 . . . . . . .
. . . . . . . .
. . . . .
Vv13g0511 . . . . . . .
. . . . . . . .
. . . . .
Vv13g0512 . . . . . . .
. . . . . . . .
. . . . .
Vv13g0513 . . Gorai.011G275500.1 . .
. . . . . . . .
. . . . . . .
Vv13g0514 . . . . . .
Vv04g0151 . . . . . . .
. . . . . .
Vv13g0515 . . . . . . .
. . . . . . . .
. . . . .
Vv13g0516 . . . . . . .
. . . . . . . .
. . . . .
Vv13g0517 . . . . . . .
. . . . . . . .
. . . . .
Vv13g0518 . . . . . . .
. . . . . . . .
. . . . .
Vv13g0519 . . . . . . .
. . . . . . . .
. . . . .
Vv13g0520 . . . . . . .
. . . . . . . .
. . . . .
Vv13g0521 . . . . . . .
. . . . . . . .
. . . . .

Vv13g0522 . . . . . . .
. . . . . . . .
. . . . .
Vv13g0523 . . . . . . .
. . . . . . . .
. . . . .
Vv13g0524 . . . . . . .
. . . . . . . .
. . . . .
Vv13g0525 . . . . . . .
. . . . . . . .
. . . . .
Vv13g0526 . . . . . . .
. . . . . . . .
. . . . .
Vv13g0527 . . . . . . .
. . . . . . . .
. . . . .
Vv13g0528 . . . . . . .
. . . . . . . .
. . . . .
Vv13g0529 . . . . . . .
. . . . . . . .
. . . . .
Vv13g0530 . . . . . . .
. . . . . . . .
. . . . .
Vv13g0531 . . . . . . .
. . . . . . . .
. . . . .
Vv13g0532 . . . . . .
Vv04g0150 . . . . . . .
. . . . . .
Vv13g0533 . . . . . . .
. . . . . . . .
. . . . .
Vv13g0534 . . . . . . .
. . . . . . . .
. . . . .
Vv13g0535 . . Gorai.011G275900.1 . .
. Vv04g0149 . . Gorai.001G021400.1
. . . . . . . .
. .
Vv13g0536 . . . . . . .
. . . . . . . .
. . . . .
Vv13g0537 . . . . . . .
. . . . . . . .
. . . . .
Vv13g0538 . . . . . . .

. . . . . . . .
. . . . .
Vv13g0539 . . . . . . .
. . . . . . . .
. . . . .
Vv13g0540 . . . . . . .
. . . . . . . .
. . . . .
Vv13g0541 . . . . . . .
. . . . . . . .
. . . . .
Vv13g0542 . . . . . . .
. . . . . . . .
. . . . .
Vv13g0543 . . . . . . .
. . . . . . . .
. . . . .
Vv13g0544 . . . . . . .
. . . . . . . .
. . . . .
Vv13g0545 . . . . . . .
. . . . . . . .
. . . . .
Vv13g0546 . . . . . . .
. . . . . . . .
. . . . .
Vv13g0547 . . . . . . .
. . . . . . . .
. . . . .
Vv13g0548 . . . . . . .
. . . . . . . .
. . . . .
Vv13g0549 . . . . . . .
. . . . . . . .
. . . . .
Vv13g0550 . . . . . . .
. . . . . . . .
. . . . .
Vv13g0551 . . . . . . .
. . . . . . . .
. . . . .
Vv13g0552 . . . . . . .
. . . . . . . .
. . . . .
Vv13g0553 . . . . . . .
. . . . . . . .
. . . . .
Vv13g0554 . . . . . . .
. . . . . . . .
. . . . .

Vv13g0555 . . . . . .
Vv04g0148 . . . . . . .
. . . . . .
Vv13g0556 . . Gorai.011G278300.1 . .
. . . . . . . .
. . . . . . .
Vv13g0557 . Gorai.013G020500.1 Gorai.011G278400.1
. . . . . . . .
. . . . . . . .
.
Vv13g0558 . . . . . . .
. . . . . . . .
. . . . .
Vv13g0559 Tc10_g002290 Gorai.013G020300.1 Gorai.
011G278600.1 . . . . . . .
. . . . . . . .
. .
Vv13g0560 Tc10_g002270 . Gorai.011G278700.1 .
. . . . . . . .
. . . . . . . .
Vv13g0561 . . . . . . .
. . . . . . . .
. . . . .
Vv13g0562 Tc10_g013130 . Gorai.011G278800.1 .
. . . . . . . .
. . . . . . . .
Vv13g0563 Tc10_g002260 Gorai.013G020100.1 . .
. . . . . . . .
. . . . . . . .
Vv13g0564 . . . . . . .
. . . . . . . .
. . . . .
Vv13g0565 Tc10_g002220 Gorai.013G019900.1 Gorai.
011G279000.1 . . . . . . .
. . . . . . . .
. .
Vv13g0566 Tc10_g002200 Gorai.013G019800.1 Gorai.
011G279100.1 . . . . . . .
. . . . . . . .
. .
Vv13g0567 . . . . . . .
. . . . . . . .
. . . . .
Vv13g0568 Tc10_g002190 Gorai.013G019700.1 . .
. . . . . . . .
. . . . . . . .
Vv13g0569 Tc10_g013030 . . . . .
. . . . . . . .
. . . . . .
Vv13g0570 Tc10_g002180 . Gorai.011G279300.1 .

. . . . . . . .
. . . . . . . .
Vv13g0571 Tc10_g002170 . . . . .
. . . . . . . .
. . . . . .
Vv13g0572 Tc10_g002120 . Gorai.011G279900.1 .
. . . . . . . .
. . . . . . . .
Vv13g0573 Tc10_g002100 . Gorai.011G280200.1 .
. . . . . . . .
. . . . . . . .
Vv13g0574 . . . . . . .
. . . . . . . .
. . . . .
Vv13g0575 Tc10_g002090 Gorai.013G019600.1 Gorai.
011G280300.1 . . . . . . .
. . . . . . . .
. .
Vv13g0576 Tc10_g002080 . Gorai.011G280400.1 .
. . . . . . . .
. . . . . . . .
Vv13g0577 . . . . . . .
. . . . . . . .
. . . . .
Vv13g0578 Tc10_g002060 Gorai.013G019500.1 Gorai.
011G280500.1 . . . . . . .
. . . . . . . .
. .
Vv13g0579 Tc10_g002040 . Gorai.011G282700.1 .
. . . . . . . .
. . . . . . . .
Vv13g0580 . . . . . . .
. . . . . . . .
. . . . .
Vv13g0581 Tc10_g002020 . Gorai.011G283000.1 .
. . Vv06g0441 Tc09_g032770 . .
. Gorai.009G103500.1 Gorai.003G138900.1 .
. . . . . .
Vv13g0582 . Gorai.013G019400.1 Gorai.011G283100.1
. . . . . . . .
. . . . . . . .
.
Vv13g0583 . . . Gorai.009G315700.1 .
. Vv06g0427 Tc09_g032950 . .
Gorai.004G002200.1 . . . . . .
. . .
Vv13g0584 Tc10_g004710 . . . . .
. . . . . . . .
. . . . . .
Vv13g0585 Tc10_g004730 . . . Gorai.

```
010G203700.1     .        .        .        .        .        .        .
         .       .        .        .        .        .        .        .
Vv13g0586        Tc10_g004740      .        Gorai.011G241700.1         .
         .       .        .        .        .        .        .        .
         .       .        .        .        .        .        .        .
Vv13g0587        Tc10_g004750      Gorai.013G024700.1         .        .
         .       .        .        .        .        .        .        .
         .       .        .        .        .        .        .        .
Vv13g0588        Tc10_g004760      Gorai.013G024800.1         .        .
         .       .        .        .        .        .        .        .
         .       .        .        .        .        .        .        .
Vv13g0589        Tc10_g004860      .        .        .        .        .
         .       .        .        .        .        .        .        .
         .       .        .        .        .        .
Vv13g0590        Tc10_g004870      .        Gorai.011G241600.1
Gorai.009G315600.1        .        .        Vv06g0410         .        .
         Gorai.001G126500.1        Gorai.004G011500.1         .
Gorai.006G116300.1        .        .        .        .        .        .
         .
Vv13g0591        Tc10_g004890      .        Gorai.011G241500.1
Gorai.009G315100.1        .        .        .        .        .        .
         .       .        .        .        .        .        .        .
         .       .
Vv13g0592        Tc10_g004900      .        .        .        .        .
         .       .        .        .        .        .        .        .
         .       .        .        .        .        .
Vv13g0593        .        .        .        .        .        .        .
         .       .        .        .        .        .        .        .
         .       .        .        .        .
Vv13g0594        .        .        .        .        .        .        .
         .       .        .        .        .        .        .        .
         .       .        .        .        .
Vv13g0595        .        .        .        .        .        .
Vv08g0857        .        .        .        .        .        .        .
         .       .        .        .        .        .
Vv13g0596        Tc10_g004940      .        .        .        .        .
         .       .        .        .        .        .        .        .
         .       .        .        .        .        .
Vv13g0597        .        Gorai.013G025000.1         .        .        .
         .       .        .        .        .        .        .        .
         .       .        .        .        .        .        .
Vv13g0598        Tc10_g004980      .        .        .        .        .
         .       .        .        .        .        .        .        .
         .       .        .        .        .        .
Vv13g0599        Tc10_g004990      .        .        .        .        .
         .       .        .        .        .        .        .        .
         .       .        .        .        .        .
Vv13g0600        .        .        .        .        .        .        .
         .       .        .        .        .        .        .        .
         .       .        .        .        .
```

Vv13g0601 . . . . . . .

. . . . . . . .

. . . . .

Vv13g0602 . . . . . . .

. . . . . . . .

. . . . .

Vv13g0603 Tc10_g005000 . . . . .

. . . . . . . .

. . . . . .

Vv13g0604 . . . . . . .

. . . . . . . .

. . . . .

Vv13g0605 . Gorai.013G016900.1 . .

Gorai.010G203800.1 . . . . . .

. . . . . . . .

.

Vv13g0606 . Gorai.013G014500.1 . . .

. . . . . . . .

. . . . . . .

Vv13g0607 . . . . . . .

. . . . . . . .

. . . . .

Vv13g0608 . . . . . . .

. . . . . . . .

. . . . .

Vv13g0609 . . . . . . .

. . . . . . . .

. . . . .

Vv13g0610 . . . . . . .

. . . . . . . .

. . . . .

Vv13g0611 . . . . . . .

. . . . . . . .

. . . . .

Vv13g0612 . . . . . . .

. . . . . . . .

. . . . .

Vv13g0613 . . . . . . .

. . . . . . . .

. . . . .

Vv13g0614 . . . . . . .

. . . . . . . .

. . . . .

Vv13g0615 . . . . . . .

. . . . . . . .

. . . . .

Vv13g0616 . . . . . . .

. . . . . . Vv06g0395

. . Gorai.001G129700.1 Gorai.004G012800.1

. Gorai.006G120900.1

Vv13g0617 . . . . . . .
. . . . . . . .
. . . . .
Vv13g0618 . . . . . . .
. . . . . . Vv06g0393
. . . . . .
Vv13g0619 . . . . . . .
. . . . . . . .
. . . . .
Vv13g0620 . . . . . . .
. . . . . . . .
. . . . .
Vv13g0621 . . . . . . .
. . . . . . . .
. . . . .
Vv13g0622 . . . . . . .
. . . . . . . .
. . . . .
Vv13g0623 Tc10_g005010 . . . . .
. . . . . . . .
. . . . . .
Vv13g0624 Tc10_g005020 . . . Gorai.
010G204000.1 . . . . . . .
. . . . . . . .
Vv13g0625 . . . . . . .
. . . . . . . .
. . . . .
Vv13g0626 . . . . . . .
. . . . . . . .
. . . . .
Vv13g0627 Tc10_g005040 . . Gorai.009G314900.1
Gorai.010G204200.1 . Vv08g0828
Tc05_g005990 Gorai.006G168400.1 Gorai.002G249700.1 .
. . . . . . . .
.
Vv13g0628 Tc10_g005070 . . . . .
. . . . . . . .
. . . . . .
Vv13g0629 . . . . . . .
. . . . . . . .
. . . . .
Vv13g0630 Tc10_g005090 . Gorai.011G241200.1 .
Gorai.010G204400.1 . Vv08g0824
Tc05_g006010 . Gorai.002G249600.1 . . .
Vv06g0382 . . . . . .
Vv13g0631 Tc10_g005100 . . . Gorai.
010G204700.1 . . . . . . .
. . . . . . . .
Vv13g0632 Tc10_g005120 . Gorai.011G241100.1 .
. . . . . . . .

.	.	.	.	.	.	.	.
Vv13g0633	.	.	.	.	.	.	.
.	.	.	.	.	.	.	.
.	.	.	.	.
Vv13g0634	Tc10_g005150	.	.	Gorai.009G314800.1
.	.	.	.	.	.	.	.
.	.	.	.	.	.	.	.
Vv13g0635	.	.	.	.	.	.	.
.	.	.	.	.	.	.	.
.	.	.	.	.
Vv13g0636	Tc10_g005160	.	Gorai.011G240900.1	.
.	.	.	.	.	.	.	.
.	.	.	.	.	.	.	.
Vv13g0637	Tc10_g005170	.	.	Gorai.009G314700.1
.	.	.	.	.	.	.	.
.	.	.	.	.	.	.	.
Vv13g0638	Tc10_g005180	.	.	.	Gorai.
010G204900.1	.	.	.	.	.	.	.
.	.	.	.	.	.	.	.
Vv13g0639	Tc10_g005190	Gorai.013G026300.1	Gorai.
011G240800.1	Gorai.009G314600.1	.	.	.	.
.	.	.	.	.	.	.	.
.	.	.	.
Vv13g0640	.	.	.	.	.	.	.
.	.	.	.	.	.	.	.
.	.	.	.	.
Vv13g0641	Tc10_g005200	.	.	.	.	.
.	.	.	.	.	.	.	.
.	.	.	.	.	.
Vv13g0642	Tc10_g005210	.	.	.	.	.
.	.	.	.	.	.	.	.
.	.	.	.	.	.
Vv13g0643	.	.	.	.	.	Gorai.
012G004000.1	.	.	.	.	.	.	.
.	.	.	.	.	.	.
Vv13g0644	.	.	.	.	Gorai.010G205000.1
.	Vv08g0805	Tc05_g006140	.	.	.
.	.	Vv06g0367	.	.	Gorai.
001G130800.1	.	Gorai.009G078700.1	.
Vv13g0645	.	.	.	.	.	.	.
.	.	.	.	.	.	.	.
.	.	.	.	.
Vv13g0646	Tc10_g005220	.	Gorai.011G240700.1	.
Gorai.010G205100.1	Gorai.012G004100.1	Vv08g0804
Tc05_g006150	.	.	.	.	.
Vv06g0366	Tc09_g025760	.	.	.	Gorai.
009G078600.1	.
Vv13g0647	.	.	.	.	.	.	.
.	.	.	.	.	.	Vv06g0365
.	.	.	.	.	.

Vv13g0648 Tc10_g005230 . . Gorai.009G314500.1
. Gorai.012G004200.1 . . . .
. . . . . . . .
. .
Vv13g0649 Tc10_g005240 . . . . .
. . . . . . . .
. . . . . .
Vv13g0650 . . . . . . .
. . . . . . . .
. . . . .
Vv13g0651 Tc10_g005250 . Gorai.011G240600.1 .
. Gorai.012G004300.1 . . . .
. . . . . . . .
. .
Vv13g0652 Tc10_g005260 . Gorai.011G240500.1 .
. . . . . . . .
. . . . . . . .
Vv13g0653 . . . . . . .
. . . . . . . .
. . . . .
Vv13g0654 Tc10_g005330 . . Gorai.009G314300.1
. . . . . . . .
. . . . . . . .
Vv13g0655 Tc10_g005350 . Gorai.011G240400.1 .
. . . . . . . .
. . . . . . . .
Vv13g0656 . . . . . . .
. . . . . . . .
. . . . .
Vv13g0657 Tc10_g005360 . Gorai.011G240300.1 .
. . . . . . . .
. . . . . . . .
Vv13g0658 . . . . . . .
. . . . . . . .
. . . . .
Vv13g0659 . . . . . . .
. . . . . . . .
. . . . .
Vv13g0660 . . . . . . .
. . . . . . . .
. . . . .
Vv13g0661 . . . . . . .
. . . . . . . .
. . . . .
Vv13g0662 . . . . . . .
. . . . . . . .
. . . . .
Vv13g0663 . . . . . . .
. . . . . . . .
. . . . .

Vv13g0664 Tc10_g005370 . Gorai.011G240100.1
Gorai.009G314200.1 . . . . . .
. . . Vv06g0350 . . .
. Gorai.009G103400.1 .
Vv13g0665 Tc10_g005380 . Gorai.011G240000.1 .
. Gorai.012G004400.1 . . . .
. . . . . . . .
. .
Vv13g0666 Tc10_g005390 . . . . .
. . . . . . . .
. . . . . .
Vv13g0667 . . . . . . .
. . . . . . . .
. . . . .
Vv13g0668 Tc10_g005420 . . . . .
. . . . . . .
Vv06g0340 Tc09_g033360 . . . Gorai.
009G106600.1 Gorai.003G141300.1
Vv13g0669 Tc10_g005430 . . . Gorai.
010G205300.1 . . . . . . .
. . . . . . . .
Vv13g0670 Tc10_g005440 . . . .
Gorai.012G004700.1 . . . . . .
. . . . . . . .
Vv13g0671 . . . . . . .
. . . . . . . .
. . . . .
Vv13g0672 . Gorai.013G027300.1 . . .
. Vv08g0781 Tc05_g006410 Gorai.006G166700.1
. . . . . . . .
. . .
Vv13g0673 Tc10_g005470 . Gorai.011G239400.1 .
. . . . . . . .
. . . . . . . .
Vv13g0674 . . . . . . .
. . . . . . . .
. . . . .
Vv13g0675 . . . . . . .
. . . . . . Vv06g0331
Tc09_g033470 Gorai.013G266900.1 . .
Gorai.009G107500.1 Gorai.003G142200.1
Vv13g0676 . Gorai.013G027400.1 . . .
. Vv08g0777 Tc05_g006440 . . .
Gorai.009G417500.1 . Vv06g0330
Tc09_g033480 . . . Gorai.009G107600.1
Gorai.003G142300.1
Vv13g0677 Tc10_g005520 . . . . .
. . . . . . . .
. . . . . .
Vv13g0678 Tc10_g005550 . Gorai.011G239300.1 .

. . . . . . . .
. . . . . . . .
Vv13g0679 Tc10_g005570 . Gorai.011G239000.1 .
. . . . . . . .
. . . . . . . .
Vv13g0680 Tc10_g005610 . Gorai.011G238900.1 .
. Gorai.012G004800.1 Vv08g0776
Tc05_g006450 Gorai.006G166400.1 . . . .
Vv06g0326 Tc09_g033510 Gorai.013G267100.1 .
. Gorai.009G107800.1 Gorai.006G120400.1
Vv13g0681 Tc10_g005620 . Gorai.011G238800.1
Gorai.009G313700.1 . . . . . .
. . . Vv06g0325 Tc09_g033520
Gorai.013G267200.1 . . Gorai.009G107900.1
Gorai.003G142600.1
Vv13g0682 Tc10_g005630 . . Gorai.009G313600.1
Gorai.010G205600.1 . . . . .
. . . Vv06g0323 Tc09_g033540 .
. . Gorai.009G108000.1 Gorai.003G142800.1
Vv13g0683 Tc10_g005640 . Gorai.011G238700.1
Gorai.009G313500.1 . . . . . .
. . . . . . . .
. .
Vv13g0684 Tc10_g005650 Gorai.013G027500.1 Gorai.
011G238600.1 . . . Vv08g0775
Tc05_g006470 . . . Gorai.009G417400.1 .
. . . . . . .
Vv13g0685 Tc10_g005670 . . . Gorai.
010G205700.1 . . . . . . .
. . . . . . . .
Vv13g0686 Tc10_g005680 . . . . .
. . . . . . . .
. . . . . .
Vv13g0687 Tc10_g005690 . Gorai.011G238500.1 .
. Gorai.012G005000.1 . . . .
. . . . . . . .
. .
Vv13g0688 Tc10_g005730 . Gorai.011G238400.1 .
. . Vv08g0763 Tc05_g006550 Gorai.
006G166000.1 Gorai.002G248000.1 . Gorai.009G416300.1
Gorai.007G373600.1 Vv06g0312 . . .
. . Gorai.006G166000.1
Vv13g0689 . . . Gorai.009G313300.1 .
. . . . . . . .
. . . . . . .
Vv13g0690 . . . . . . .
. . . . . . . .
. . . . .
Vv13g0691 . . . . . . .
. . . . . . . .

. . . . .
Vv13g0692 . . . . . .
Vv08g0729 . . . . . . .
. . . . . .
Vv13g0693 Tc10_g005740 . . . . .
. . . . . . . .
. . . . . .
Vv13g0694 . . . . . . .
. . . . . . . .
. . . . .
Vv13g0695 Tc10_g005760 . . . . .
. . . . . . . .
. . . . . .
Vv13g0696 Tc10_g005770 . . Gorai.009G313100.1
. . . . . . . .
. . . . . . . .
Vv13g0697 Tc10_g005780 . . . . .
. . . . . . . .
. . . . . .
Vv13g0698 . . . . . . .
. . . . . . . .
. . . . .
Vv13g0699 . . . Gorai.009G313000.1 .
. . . . . . . .
. . . . . . .
Vv13g0700 Tc10_g005950 . . . . .
. . . . . . . .
. . . . . .
Vv13g0701 . . Gorai.011G238300.1 Gorai.
009G312500.1 . . Vv06g0295 . . .
. Gorai.009G109800.1 . . . .
. . . .
Vv13g0702 Tc10_g005970 . Gorai.011G238200.1 .
. . . . . . . .
. . . . . . . .
Vv13g0703 Tc10_g005980 . Gorai.011G238100.1
Gorai.009G312200.1 . . . . . .
. . . . . . . .
. .
Vv13g0704 . . . . . .
Vv06g0293 Tc09_g033690 Gorai.013G268100.1 Gorai.
001G134600.1 . . Gorai.006G165400.1 . .
. . . . .
Vv13g0705 . . . Gorai.009G312000.1 .
. . . . . . . .
. . . . . . .
Vv13g0706 Tc10_g006020 . . . .
Gorai.012G005300.1 . . . . . .
. . . . . . . .
Vv13g0707 . . . . . . .

. . . . . . . .
. . . . .
Vv13g0708 Tc10_g006040 . . . .
Gorai.012G005400.1 . . . . . .
. . . . . . . .
Vv13g0709 Tc10_g006060 . . Gorai.009G311900.1
. . . . . . . .
. . . . . . . .
Vv13g0710 Tc10_g006080 . Gorai.011G238000.1
Gorai.009G311500.1 . . Vv08g1469
Tc05_g014680 . . . . . . .
. . . . .
Vv13g0711 Tc10_g006110 . Gorai.011G237900.1 .
. . . . . . . .
. . . . . . . .
Vv13g0712 Tc10_g006250 . . . . .
. . . . . . . .
. . . . . .
Vv13g0713 Tc10_g006320 . Gorai.011G237300.1 .
. . . . . . . .
. . . . . . . .
Vv13g0714 . . . . . Gorai.
012G005800.1 . . . . . . .
. . . . . . .
Vv13g0715 Tc10_g006330 . . . . .
. . . . . . . .
. . . . . .
Vv13g0716 Tc10_g006340 . . . Gorai.
010G205900.1 Gorai.012G005900.1 . . . .
. . . . . . . .
. .
Vv13g0717 Tc10_g006350 . Gorai.011G237100.1 .
. . . . . . . .
. . . . . . . .
Vv13g0718 Tc10_g006370 . Gorai.011G236400.1 .
. Gorai.012G006000.1 . . . .
. . . . . . . .
. .
Vv13g0719 . . . . . . .
. . . . . . . .
. . . . .
Vv13g0720 . . . . . . .
. . . . . . . .
. . . . .
Vv13g0721 Tc10_g006430 . Gorai.011G236100.1 .
. . . . . . . .
. . . . . . . .
Vv13g0722 . . . . . . .
. . . . . . . .
. . . . .

Vv13g0723 Tc10_g006440 . . . Gorai.
010G206000.1 . . . . . . .
. . . . . . . .
Vv13g0724 Tc10_g006450 . Gorai.011G236000.1 .
Gorai.010G207000.1 . . . . .
. . . . . . . .
. .
Vv13g0725 Tc10_g006530 . . Gorai.009G310600.1
. . . . . . . .
. . . . . . . .
Vv13g0726 . . . . . . .
. . . . . . . .
. . . . .
Vv13g0727 . . . . . . .
. . . . . . . .
. . . . .
Vv13g0728 . . . . . . .
. . . . . . . .
. . . . .
Vv13g0729 . . . . . . .
. . . . . . . .
. . . . .
Vv13g0730 . . . . . . .
. . . . . . . .
. . . . .
Vv13g0731 . . . . . . .
. . . . . . . .
. . . . .
Vv13g0732 . . . . . . .
. . . . . . . .
. . . . .
Vv13g0733 . . . . . . .
. . . . . . . .
. . . . .
Vv13g0734 . . . . . . .
. . . . . . . .
. . . . .
Vv13g0735 . . . . . . .
. . . . . . . .
. . . . .
Vv13g0736 . . . . . . .
. . . . . . . .
. . . . .
Vv13g0737 . . . . . . .
. . . . . . . .
. . . . .
Vv13g0738 . . . . . . .
. . . . . . . .
. . . . .
Vv13g0739 . . . . . . .

```
         .         .         .         .         .         .         .         .
         .         .         .         .         .
Vv13g0740          .         .         .         .         .         .         .
         .         .         .         .         .         .         .         .
         .         .         .         .         .
Vv13g0741          .         .         Gorai.011G235000.1            .         .
         .         .         .         .         .         .         .         .
         .         .         .         .         .         .         .
Vv13g0742          .         .         Gorai.011G234900.1            .         .
         .         Vv08g1430           Tc05_g007230        Gorai.006G161600.1
         .         .         .         .         .         .         .         .
         .         .         .
Vv13g0743          .         .         .         .         .         .         .
         .         .         .         .         .         .         .         .
         .         .         .         .         .
Vv13g0744          .         .         .         .         .         .         .
         .         .         .         .         .         .         .         .
         .         .         .         .         .
Vv13g0745          .         .         .         .         .         .         .
         .         .         .         .         .         .         .         .
         .         .         .         .         .
Vv13g0746          .         .         .         .         .         .         .
         .         .         .         .         .         .         .         .
         .         .         .         .         .
Vv13g0747          .         .         .         .         .         .         .
         .         .         .         .         .         .         .         .
         .         .         .         .         .
Vv13g0748          .         .         .         .         .         .         .
         .         .         .         .         .         .         .         .
         .         .         .         .         .
Vv13g0749          Tc10_g006570        .         .         .         .         .
         .         .         .         .         .         .         .         .
         .         .         .         .         .         .
Vv13g0750          Tc10_g006590        .         .         .         .         .
         .         .         .         .         .         .         .         .
         .         .         .         .         .         .
Vv13g0751          .         .         .         .         .         .         .
         .         .         .         .         .         .         .         .
         .         .         .         .         .
Vv13g0752          Tc10_g006620        .         .         .         .         .
         .         .         .         .         .         .         .         .
         .         .         .         .         .         .
Vv13g0753          Tc10_g006910        .         .         .         .         .
         .         .         .         .         .         .         .         .
         .         .         .         .         .         .
Vv13g0754          Tc10_g006940        .         Gorai.011G234400.1            .
         .         Gorai.012G006900.1            .         .         .         .
         .         .         .         .         .         .         .         .
         .         .
Vv13g0755          Tc10_g006970        .         .         .         .         .
```

```
        .        .       .        .       .        .       .        .
        .        .       .        .       .        .
Vv13g0756        Tc10_g006980     .       .        .       .        .
        .        .       .        .       .        .       .        .
        .        .       .        .       .        .
Vv13g0757        .       .        .       .        .       .        .
        .        .       .        .       .        .       .        .
        .        .       .        .       .
Vv13g0758        .       .        .       Gorai.009G310500.1        .
        .        .       .        .       .        .       .        .
        .        .       .        .       .        .       .
Vv13g0759        Tc10_g007000     .       .        Gorai.009G309300.1
        .        Gorai.012G007000.1       .        .       .        .
        .        .       .        .       .        .       .        .
        .        .
Vv13g0760        Tc10_g007040     .       .        Gorai.009G309000.1
        .        .       .        .       .        .       .        .
        .        .       .        .       .        .       .        .
Vv13g0761        Tc10_g007050     .       .        .       .
Gorai.012G007300.1       .        .       .        .       .        .
        .        .       .        .       .        .       .        .
Vv13g0762        Tc10_g007060     .       .        .       .
Gorai.012G007500.1       .        .       .        .       .        .
        .        .       .        .       .        .       .        .
Vv13g0763        .       .        .       .        .       .        .
        .        .       .        .       .        .       .        .
        .        .       .        .       .
Vv13g0764        .       .        Gorai.011G234300.1       .        .
        .        .       .        .       .        .       .        .
        .        .       .        .       .        .       .
Vv13g0765        Tc10_g007080     .       .        Gorai.009G308900.1
        .        .       .        .       .        .       .        .
        .        .       .        .       .        .       .        .
Vv13g0766        .       .        .       .        .       .        .
        .        .       .        .       .        .       .        .
        .        .       .        .       .
Vv13g0767        .       .        .       .        .       .        .
        .        .       .        .       .        .       .        .
        .        .       .        .       .
Vv13g0768        .       .        .       .        .       .        .
        .        .       .        .       .        .       .        .
        .        .       .        .       .
Vv13g0769        Tc10_g007170     .       Gorai.011G234000.1
Gorai.009G308500.1       Gorai.010G207600.1        .       .        .
        .        .       .        .       .        .       .        .
        .        .       .        .
Vv13g0770        Tc10_g007200     .       .        .       .        .
        .        .       .        .       .        .       .        .
        .        .       .        .       .        .
Vv13g0771        Tc10_g007210     .       .        .       .        .
```

| | | | | | | | |
|---|---|---|---|---|---|---|---|
| . | . | . | . | . | . | . | . |
| . | . | . | . | . | . | | |
| Vv13g0772 | Tc10_g007220 | | . | . | Gorai.009G308200.1 | | |
| . | . | . | . | . | . | . | . |
| . | . | . | . | . | . | . | . |
| Vv13g0773 | . | . | Gorai.011G233900.1 | | | . | . |
| . | . | . | . | . | . | . | . |
| . | . | . | . | . | . | . | |
| Vv13g0774 | Tc10_g007710 | | . | Gorai.011G233700.1 | | | . |
| . | . | Vv08g1392 | | . | . | . | . |
| . | . | . | . | . | . | . | . |
| . | | | | | | | |
| Vv13g0775 | . | . | . | . | . | . | . |
| . | . | . | . | . | . | . | . |
| . | . | . | . | . | | | |
| Vv13g0776 | . | . | . | . | . | . | . |
| . | . | . | . | . | . | . | . |
| . | . | . | . | . | | | |
| Vv13g0777 | . | . | . | . | . | . | . |
| . | . | . | . | . | . | . | . |
| . | . | . | . | . | | | |
| Vv13g0778 | . | . | . | . | . | . | . |
| . | . | . | . | . | . | . | . |
| . | . | . | . | . | | | |
| Vv13g0779 | . | . | . | . | . | . | . |
| . | . | . | . | . | . | . | . |
| . | . | . | . | . | | | |
| Vv13g0780 | . | . | . | . | . | . | . |
| . | . | . | . | . | . | . | . |
| . | . | . | . | . | | | |
| Vv13g0781 | . | . | . | . | . | . | . |
| . | . | . | . | . | . | . | . |
| . | . | . | . | . | | | |
| Vv13g0782 | Tc10_g015110 | | . | . | . | . | . |
| . | . | . | . | . | . | . | . |
| . | . | . | . | . | . | | |
| Vv13g0783 | . | . | . | . | . | . | . |
| . | . | . | . | . | . | . | . |
| . | . | . | . | . | | | |
| Vv13g0784 | Tc10_g007850 | | . | . | . | . | . |
| . | . | . | . | . | . | . | . |
| . | . | . | . | . | . | | |
| Vv13g0785 | Tc10_g007880 | | . | . | Gorai.009G307600.1 | | |
| . | . | . | . | . | . | . | . |
| . | . | . | . | . | . | . | . |
| Vv13g0786 | . | . | . | . | . | . | . |
| . | . | . | . | . | . | . | . |
| . | . | . | . | . | | | |
| Vv13g0787 | Tc10_g007950 | | . | . | . | . | . |
| . | . | . | . | . | . | . | . |

. . . . . .
Vv13g0788 . . . . . . .
. . . . . . . .
. . . . .
Vv13g0789 . . . . . . .
. . . . . . . .
. . . . .
Vv13g0790 . . . . . . .
. . . . . . . .
. . . . .
Vv13g0791 Tc10_g008060 . . . . .
. . . . . . . .
. . . . . .
Vv13g0792 . . . . . . .
. . . . . . . .
. . . . .
Vv13g0793 . . . . . . .
. . . . . . . .
. . . . .
Vv13g0794 Tc10_g008100 . . Gorai.009G307400.1
. . . . . . . .
. . . . . . . .
Vv13g0795 . . . . . . .
. . . . . . . .
. . . . .
Vv13g0796 . . . . . . .
. . . . . . . .
. . . . .
Vv13g0797 . . . . . . .
. . . . . . . .
. . . . .
Vv13g0798 . . . . . . .
. . . . . . . .
. . . . .
Vv13g0799 . . . . . . .
. . . . . . . .
. . . . .
Vv13g0800 Tc10_g008510 . . Gorai.009G307300.1
. . . . . . . .
. . . . . . . .
Vv13g0801 Tc10_g009130 . . . . .
. . . . . . . .
. . . . . .
Vv13g0802 . . Gorai.011G233500.1 . .
. . . . . . . .
. . . . . . .
Vv13g0803 . . . . . . .
. . . . . . . .
. . . . .
Vv13g0804 Tc10_g014650 . . . . .

. . . . . . . .
. . . . . .
Vv13g0805 . . . . . .
Vv08g1357 Tc05_g007940 Gorai.006G159400.1 Gorai.
002G244300.1 . . Gorai.007G371600.1 . .
. . . . .
Vv13g0806 . . . . . . .
. . . . . . . .
. . . . .
Vv13g0807 . . Gorai.011G233300.1 . .
. . . . . . . .
. . . . . . .
Vv13g0808 . . Gorai.011G233200.1 . .
. . . . . . . .
. . . . . . .
Vv13g0809 . . . . . . .
. . . . . . . .
. . . . .
Vv13g0810 . . . . . . .
. . . . . . . .
. . . . .
Vv13g0811 . . Gorai.011G233100.1 . .
. . . . . . . .
. . . . . . .
Vv13g0812 . . . . . .
Vv08g1353 . . Gorai.002G243800.1 . .
. . . . . . . .
Vv13g0813 . . . . . . .
. . . . . . . .
. . . . .
Vv13g0814 . . . . . . .
. . . . . . . .
. . . . .
Vv13g0815 . . Gorai.011G233000.1 . .
. Vv08g1352 Tc05_g007990 Gorai.006G158900.1
Gorai.002G243700.1 . . . . .
. . . . .
Vv13g0816 . . . . . . .
. . . . . . . .
. . . . .
Vv13g0817 . . . . . . .
. . . . . . . .
. . . . .
Vv13g0818 . . . . . . .
. . . . . . . .
. . . . .
Vv13g0819 . . . . . . .
. . . . . . . .
. . . . .
Vv13g0820 . . . . . .

```
Vv08g1351       Tc05_g008000    .       .       .       .
Gorai.007G371500.1      .       .       .       .       .       .
        .
Vv13g0821       .       .       .       .       .       .       .
        .       .       .       .       .       .       .       .
        .       .       .       .       .
Vv13g0822       .       .       .       .       .       .       .
        .       .       .       .       .       .       .       .
        .       .       .       .       .
Vv13g0823       .       .       .       .       .       .       .
        .       .       .       .       .       .       .       .
        .       .       .       .       .
Vv13g0824       .       .       .       .       .       .       .
        .       .       .       .       .       .       .       .
        .       .       .       .       .
Vv13g0825       .       .       .       .       .       .       .
        .       .       .       .       .       .       .       .
        .       .       .       .       .
Vv13g0826       .       .       .       .       .       .       .
        .       .       .       .       .       .       .       .
        .       .       .       .       .
Vv13g0827       .       .       .       .       .       .       .
        .       .       .       .       .       .       .       .
        .       .       .       .       .
Vv13g0828       .       .       .       .       .       .       .
        .       .       .       .       .       .       .       .
        .       .       .       .       .
Vv13g0829       .       .       .       .       .       .       .
        .       .       .       .       .       .       .       .
        .       .       .       .       .
Vv13g0830       .       .       .       .       .       .       .
        .       .       .       .       .       .       .       .
        .       .       .       .       .
Vv13g0831       .       .       .       .       .       .       .
        .       .       .       .       .       .       .       .
        .       .       .       .       .
Vv13g0832       Tc10_g011760    .       .       Gorai.009G304700.1
        .       .       .       .       .       .       .       .
        .       .       .       .       .       .       .       .
Vv13g0833       .       .       .       .       .       .       .
        .       .       .       .       .       .       .       .
        .       .       .       .       .
Vv13g0834       Tc10_g011460    .       .       .       .       .
        .       .       .       .       .       .       .       .
        .       .       .       .       .       .
Vv13g0835       .       .       .       .       .       .       .
        .       .       .       .       .       .       .       .
        .       .       .       .       .
Vv13g0836       .       .       .       Gorai.009G304600.1      .
        .       .       .       .       .       .       .       .
```

| | | | | | | | | |
|---|---|---|---|---|---|---|---|---|
| | . | . | . | . | . | . | . | |
| Vv13g0837 | | . | . | . | . | . | . | . |
| | . | . | . | . | . | . | . | . |
| | . | . | . | . | . | | | |
| Vv13g0838 | | . | . | . | Gorai.009G302500.1 | | | . |
| | . | . | . | . | . | . | . | . |
| | . | . | . | . | . | . | . | |
| Vv13g0839 | | . | . | . | . | . | . | . |
| | . | . | . | . | . | . | . | . |
| | . | . | . | . | . | | | |
| Vv13g0840 | | . | . | . | . | . | . | . |
| | . | . | . | . | . | . | . | . |
| | . | . | . | . | . | | | |
| Vv13g0841 | | Tc10_g003050 | | . | . | . | . | . |
| | . | . | . | . | . | . | . | . |
| | . | . | . | . | . | . | | |
| Vv13g0842 | | . | . | . | . | . | . | . |
| | . | . | . | . | . | . | . | . |
| | . | . | . | . | . | | | |
| Vv13g0843 | | . | . | . | . | . | . | . |
| | . | . | . | . | . | . | . | . |
| | . | . | . | . | . | | | |
| Vv13g0844 | | Tc10_g010120 | | . | . | . | . | . |
| | . | . | . | . | . | . | . | . |
| | . | . | . | . | . | . | | |
| Vv13g0845 | | . | . | . | . | . | . | . |
| | . | . | . | . | . | . | . | . |
| | . | . | . | . | . | | | |
| Vv13g0846 | | Tc10_g010250 | | . | . | . | . | . |
| | . | . | . | . | . | . | . | . |
| | . | . | . | . | . | . | | |
| Vv13g0847 | | Tc10_g010670 | | . | . | . | . | . |
| | . | . | . | . | . | . | . | . |
| | . | . | . | . | . | . | | |
| Vv13g0848 | | . | . | . | . | . | . | |
| Vv08g1330 | | Tc05_g008200 | | . | Gorai.002G243400.1 | | | . |
| | . | . | . | . | . | . | . | . |
| | . | | | | | | | |
| Vv13g0849 | | . | . | . | . | . | . | . |
| | . | . | . | . | . | . | . | . |
| | . | . | . | . | . | | | |
| Vv13g0850 | | . | . | . | . | . | . | . |
| | . | . | . | . | . | . | . | . |
| | . | . | . | . | . | | | |
| Vv13g0851 | | . | . | . | . | . | . | . |
| | . | . | . | . | . | . | . | . |
| | . | . | . | . | . | | | |
| Vv13g0852 | | Tc10_g003140 | | . | Gorai.011G232200.1 | | | . |
| | . | . | . | . | . | . | . | . |
| | . | . | . | . | . | . | . | . |

Vv13g0853 . . . . . . .
. . . . . . . .
. . . . .
Vv13g0854 . . . . . . .
. . . . . . . .
. . . . .
Vv13g0855 . . . . . . .
. . . . . . . .
. . . . .
Vv13g0856 . . . . . . .
. . . . . . . .
. . . . .
Vv13g0857 . . . . . . .
. . . . . . . .
. . . . .
Vv13g0858 . . . . . . .
. . . . . . . .
. . . . .
Vv13g0859 . . . . . Gorai.
012G025000.1 . . . . . . .
. . . . . . .
Vv13g0860 . . . . . . .
. . . . . . . .
. . . . .
Vv13g0861 . . . . . Gorai.
012G024300.1 . . . . . . .
. . . . . . .
Vv13g0862 . . . . . . .
. . . . . . . .
. . . . .
Vv13g0863 . . . . . . .
. . . . . . . .
. . . . .
Vv13g0864 . . . . . . .
. . . . . . . .
. . . . .
Vv13g0865 . . . . . . .
. . . . . . . .
. . . . .
Vv13g0866 . . . . . . .
. . . . . . . .
. . . . .
Vv13g0867 . . . . . . .
. . . . . . . .
. . . . .
Vv13g0868 . . . . . . .
. . . . . . . .
. . . . .
Vv13g0869 . . . . . . .
. . . . . . . .

. . . . .
Vv13g0870 . . . . . . .
. . . . . . . .
. . . . .
Vv13g0871 . . . . . . .
. . . . . . . .
. . . . .
Vv13g0872 . . . . . . .
. . . . . . . .
. . . . .
Vv13g0873 . . . . . . .
. . . . . . . .
. . . . .
Vv13g0874 . . . . . .
Vv08g1303 . Gorai.006G157400.1 Gorai.002G241500.1
. . . . . . . .
. .
Vv13g0875 . . . . . . .
. . . . . . . .
. . . . .
Vv13g0876 . . . . . . .
. . . . . . . .
. . . . .
Vv13g0877 . . . . . . .
. . . . . . . .
. . . . .
Vv13g0878 . . . . . . .
. . . . . . . .
. . . . .
Vv13g0879 . . . . . . .
. . . . . . . .
. . . . .
Vv13g0880 . . . . . . .
. . . . . . . .
. . . . .
Vv13g0881 . . . . . Gorai.
012G023700.1 . . . . . . .
. . . . . . .
Vv13g0882 . . . . . . .
. . . . . . . .
. . . . .
Vv13g0883 Tc10_g008990 . . . . .
. . . . . . . .
. . . . . .
Vv13g0884 . . . . . . .
. . . . . . . .
. . . . .
Vv13g0885 . . . . . . .
. . . . . . . .
. . . . .

Vv13g0886 . . . . . . .
. . . . . . . .
. . . . .
Vv13g0887 Tc10_g009000 . . . . .
. . . . . . . .
. . . . . .
Vv13g0888 . . . . . . .
. . . . . . . .
. . . . .
Vv13g0889 . . . . . . .
. . . . . . . .
. . . . .
Vv13g0890 . . . . . . .
. . . . . . . .
. . . . .
Vv13g0891 . . . . . . .
. . . . . . . .
. . . . .
Vv13g0892 . . . . . . .
. . . . . . . .
. . . . .
Vv13g0893 Tc10_g009030 . Gorai.011G229600.1 .
. . . . . . . .
. . . . . . . .
Vv13g0894 . . . . . . .
. . . . . . . .
. . . . .
Vv13g0895 . . . . . . .
. . . . . . . .
. . . . .
Vv13g0896 . . . . . . .
. . . . . . . .
. . . . .
Vv13g0897 . . . . . . .
. . . . . . . .
. . . . .
Vv13g0898 Tc10_g009120 . . . .
Gorai.012G023500.1 . . . . . .
. . . . . . . .
Vv13g0899 Tc10_g009130 . . . . .
. . . . . . . .
. . . . . .
Vv13g0900 . . . . . . .
. . . . . . . .
. . . . .
Vv13g0901 . . . . . . .
. . . . . . . .
. . . . .
Vv13g0902 . . . . . . .
. . . . . . . .

| | | | | | | | |
|---|---|---|---|---|---|---|---|
| . | . | . | . | . | | | |
| Vv13g0903 | . | . | . | . | . | . | . |
| . | . | . | . | . | . | . | . |
| . | . | . | . | . | | | |
| Vv13g0904 | . | . | . | . | . | . | |
| Vv08g1288 | . | . | . | . | . | . | . |
| . | . | . | . | . | . | | |
| Vv13g0905 | . | . | . | . | . | . | . |
| . | . | . | . | . | . | . | . |
| . | . | . | . | . | | | |
| Vv13g0906 | . | . | . | . | . | . | . |
| . | . | . | . | . | . | . | . |
| . | . | . | . | . | | | |
| Vv13g0907 | Tc10_g000740 | | . | . | . | . | . |
| . | . | . | . | . | . | . | . |
| . | . | . | . | . | . | | |
| Vv13g0908 | . | . | . | . | . | . | . |
| . | . | . | . | . | . | . | . |
| . | . | . | . | . | | | |
| Vv13g0909 | . | . | . | . | . | . | . |
| . | . | . | . | . | . | . | . |
| . | . | . | . | . | | | |
| Vv13g0910 | . | . | . | . | . | . | . |
| . | . | . | . | . | . | . | . |
| . | . | . | . | . | | | |
| Vv13g0911 | . | . | . | . | . | . | . |
| . | . | . | . | . | . | . | . |
| . | . | . | . | . | | | |
| Vv13g0912 | . | . | . | . | . | Gorai. | |
| 012G021500.1 | . | . | . | . | . | . | . |
| . | . | . | . | . | . | . | |
| Vv13g0913 | . | . | . | . | . | . | . |
| . | . | . | . | . | . | . | . |
| . | . | . | . | . | | | |
| Vv13g0914 | Tc10_g000360 | | . | . | . | . | . |
| . | . | . | . | . | . | . | . |
| . | . | . | . | . | . | | |
| Vv13g0915 | . | . | . | . | . | . | . |
| . | . | . | . | . | . | . | . |
| . | . | . | . | . | | | |
| Vv13g0916 | . | . | . | . | . | . | . |
| . | . | . | . | . | . | . | . |
| . | . | . | . | . | | | |
| Vv13g0917 | . | . | . | . | . | . | . |
| . | . | . | . | . | . | . | . |
| . | . | . | . | . | | | |
| Vv13g0918 | . | . | . | . | . | . | . |
| . | . | . | . | . | . | . | . |
| . | . | . | . | . | | | |
| Vv13g0919 | . | . | . | . | . | . | . |

. . . . . . . .
. . . . .
Vv13g0920 . . . . . . .
. . . . . . . .
. . . . .
Vv13g0921 . . . . . . .
. . . . . . . .
. . . . .
Vv13g0922 . . . . . . .
. . . . . . . .
. . . . .
Vv13g0923 . . . . . . .
. . . . . . . .
. . . . .
Vv13g0924 . . . . . . .
. . . . . . . .
. . . . .
Vv13g0925 . . . . . Gorai.
012G021400.1 . . . . . . .
. . . . . . .
Vv13g0926 . . . . . . .
. . . . . . . .
. . . . .
Vv13g0927 . . . . . . .
. . . . . . . .
. . . . .
Vv13g0928 . . . . . . .
. . . . . . . .
. . . . .
Vv13g0929 . . . . . . .
. . . . . . . .
. . . . .
Vv13g0930 . . . . . . .
. . . . . . . .
. . . . .
Vv13g0931 . . . . . . .
. . . . . . . .
. . . . .
Vv13g0932 . . . . . . .
. . . . . . . .
. . . . .
Vv13g0933 . . . . . . .
. . . . . . . .
. . . . .
Vv13g0934 Tc10_g000320 . . . . .
. . . . . . . .
. . . . . .
Vv13g0935 Tc10_g012360 . . . . .
. . . . . . . .
. . . . . .

| | | | | | | | |
|---|---|---|---|---|---|---|---|
| Vv13g0936 | . | . | . | . | . | . | . |
| . | . | . | . | . | . | . | . |
| . | . | . | . | . | | | |
| Vv13g0937 | . | . | . | . | . | . | . |
| . | . | . | . | . | . | . | . |
| . | . | . | . | . | | | |
| Vv13g0938 | . | . | . | . | . | . | . |
| . | . | . | . | . | . | . | . |
| . | . | . | . | . | | | |
| Vv13g0939 | . | . | . | . | . | . | . |
| . | . | . | . | . | . | . | . |
| . | . | . | . | . | | | |
| Vv13g0940 | . | . | . | . | . | . | . |
| . | . | . | . | . | . | . | . |
| . | . | . | . | . | | | |
| Vv13g0941 | . | . | . | . | . | . | . |
| . | . | . | . | . | . | . | . |
| . | . | . | . | . | | | |
| Vv13g0942 | Tc10_g012460 | | . | . | . | . | . |
| . | . | . | . | . | . | . | . |
| . | . | . | . | . | . | | |
| Vv13g0943 | Tc10_g012510 | | . | . | Gorai.009G301100.1 | | |
| . | . | . | . | . | . | . | . |
| . | . | . | . | . | . | . | . |
| Vv13g0944 | . | . | . | . | . | . | . |
| . | . | . | . | . | . | . | . |
| . | . | . | . | . | | | |
| Vv13g0945 | . | . | . | . | . | . | . |
| . | . | . | . | . | . | . | . |
| . | . | . | . | . | | | |
| Vv13g0946 | . | . | . | . | . | . | . |
| . | . | . | . | . | . | . | . |
| . | . | . | . | . | | | |
| Vv13g0947 | . | . | . | . | . | . | . |
| . | . | . | . | . | . | . | . |
| . | . | . | . | . | | | |
| Vv13g0948 | . | . | . | . | . | . | . |
| . | . | . | . | . | . | . | . |
| . | . | . | . | . | | | |
| Vv13g0949 | . | . | . | . | . | . | . |
| . | . | . | . | . | . | . | . |
| . | . | . | . | . | | | |
| Vv13g0950 | . | . | . | . | . | . | . |
| . | . | . | . | . | . | . | . |
| . | . | . | . | . | | | |
| Vv13g0951 | Tc10_g013030 | | . | . | Gorai.009G300000.1 | | |
| . | . | . | . | . | . | . | . |
| . | . | . | . | . | . | . | . |
| Vv13g0952 | . | . | . | . | . | . | . |
| . | . | . | . | . | . | . | . |

| | | | | | | | |
|---|---|---|---|---|---|---|---|
| Vv13g0953 | . | . | . | . | . | . | . |
| Vv13g0954 | . | . | . | . | . | . | . |
| Vv13g0955 | . | . | . | . | . | . | . |
| Vv13g0956 | . | . | . | . | . | . | . |
| Vv13g0957 | Tc10_g013060 | | . | . | . | . | . |
| Vv13g0958 | . | . | . | . | . | . | . |
| Vv13g0959 | . | . | . | . | . | . | . |
| Vv13g0960 | . | . | . | . | . | . | . |
| Vv13g0961 | . | . | . | . | . | . | . |
| Vv13g0962 | . | . | . | . | . | . | . |
| Vv13g0963 | . | . | . | . | . | . | . |
| Vv13g0964 | . | . | . | . | . | . | . |
| Vv13g0965 | . | . | . | . | . | . | . |
| Vv13g0966 | . | . | . | . | . | . | . |
| Vv13g0967 | . | . | . | . | . | . | . |
| Vv13g0968 | . | . | . | . | . | . | . |
| Vv13g0969 | . | . | . | . | . | . | . |

. . . . . . . .
. . . . .
Vv13g0970 . . . . . . .
. . . . . . . .
. . . . .
Vv13g0971 . . . . . Gorai.
012G019000.1 . . . . . . .
. . . . . . .
Vv13g0972 . . . . . . .
. . . . . . . .
. . . . .
Vv13g0973 Tc10_g000220 . . . . .
. . . . . . . .
. . . . . .
Vv13g0974 Tc10_g016270 . . . . .
. . . . . . . .
. . . . . .
Vv13g0975 . . . . . . .
. . . . . . . .
. . . . .
Vv13g0976 . . . . . . .
. . . . . . . .
. . . . .
Vv13g0977 . . . . . . .
. . . . . . . .
. . . . .
Vv13g0978 . . . . . . .
. . . . . . . .
. . . . .
Vv13g0979 . . . . . . .
. . . . . . . .
. . . . .
Vv13g0980 . . . . . . .
. . . . . . . .
. . . . .
Vv13g0981 . . . . . . .
. . . . . . . .
. . . . .
Vv13g0982 . . . . . . .
. . . . . . . .
. . . . .
Vv13g0983 . . . . . . .
. . . . . . . .
. . . . .
Vv13g0984 . . . . . . .
. . . . . . . .
. . . . .
Vv13g0985 . . . Gorai.009G294600.1 .
. . . . . . . .
. . . . . . .

Vv13g0986 . . . . . . .
. . . . . . . .
. . . . .
Vv13g0987 . . . . . . .
. . . . . . . .
. . . . .
Vv13g0988 . . . . . . .
. . . . . . . .
. . . . .
Vv13g0989 . . . . . . .
. . . . . . . .
. . . . .
Vv13g0990 . . . . . . .
. . . . . . . .
. . . . .
Vv13g0991 Tc10_g016140 . . . . .
. . . . . . . .
. . . . . .
Vv13g0992 . . . . . . .
. . . . . . . .
. . . . .
Vv13g0993 . . . . . . .
. . . . . . . .
. . . . .
Vv13g0994 . . . . . . .
. . . . . . . .
. . . . .
Vv13g0995 . . . . . . .
. . . . . . . .
. . . . .
Vv13g0996 . . . . . . .
. . . . . . . .
. . . . .
Vv13g0997 . . . . . . .
. . . . . . . .
. . . . .
Vv13g0998 . . . . . . .
. . . . . . . .
. . . . .
Vv13g0999 . . . . . . .
. . . . . . . .
. . . . .
Vv13g1000 . . . . . . .
. . . . . . . .
. . . . .
Vv13g1001 Tc10_g016130 . . . . .
. . . . . . . .
. . . . . .
Vv13g1002 . . . . . . .
. . . . . . . .

. . . . .
Vv13g1003 . . . . . . .
. . . . . . . .
. . . . .
Vv13g1004 . . . Gorai.009G295800.1 .
. . . . . . . .
. . . . . . .
Vv13g1005 . . . Gorai.009G295900.1 .
. . . . . . . .
. . . . . . .
Vv13g1006 . . . . . . .
. . . . . . . .
. . . . .
Vv13g1007 . . . Gorai.009G296000.1 .
. . . . . . . .
. . . . . . .
Vv13g1008 . . . . . . .
. . . . . . . .
. . . . .
Vv13g1009 . . . . . . .
. . . . . . . .
. . . . .
Vv13g1010 . . . . . . .
. . . . . . . .
. . . . .
Vv13g1011 . . . . . . .
. . . . . . . .
. . . . .
Vv13g1012 . . . . . . .
. . . . . . . .
. . . . .
Vv13g1013 . . . . . . .
. . . . . . . .
. . . . .
Vv13g1014 . . . . . . .
. . . . . . . .
. . . . .
Vv13g1015 . . . . . . .
. . . . . . . .
. . . . .
Vv13g1016 . . . . . . .
. . . . . . . .
. . . . .
Vv13g1017 . . . . . . .
. . . . . . . .
. . . . .
Vv13g1018 . . . . . . .
. . . . . . . .
. . . . .
Vv13g1019 . . . . . . .

| | | | | | | | |
|---|---|---|---|---|---|---|---|
| . | . | . | . | . | . | . | . |
| . | . | . | . | . | | | |
| Vv13g1020 | . | . | . | . | . | . | . |
| . | . | . | . | . | . | . | . |
| . | . | . | . | . | | | |
| Vv13g1021 | . | . | . | . | . | . | . |
| . | . | . | . | . | . | . | . |
| . | . | . | . | . | | | |
| Vv13g1022 | . | . | . | . | . | . | . |
| . | . | . | . | . | . | . | . |
| . | . | . | . | . | | | |
| Vv13g1023 | . | . | . | . | . | . | . |
| . | . | . | . | . | . | . | . |
| . | . | . | . | . | | | |
| Vv13g1024 | . | . | . | . | . | . | . |
| . | . | . | . | . | . | . | . |
| . | . | . | . | . | | | |
| Vv13g1025 | . | . | . | . | . | . | . |
| . | . | . | . | . | . | . | . |
| . | . | . | . | . | | | |
| Vv13g1026 | . | . | . | . | . | . | . |
| . | . | . | . | . | . | . | . |
| . | . | . | . | . | | | |
| Vv13g1027 | . | . | . | . | . | . | . |
| . | . | . | . | . | . | . | . |
| . | . | . | . | . | | | |
| Vv13g1028 | . | . | . | . | . | . | . |
| . | . | . | . | . | . | . | . |
| . | . | . | . | . | | | |
| Vv13g1029 | . | . | . | . | . | . | . |
| . | . | . | . | . | . | . | . |
| . | . | . | . | . | | | |
| Vv13g1030 | . | . | . | . | . | . | . |
| . | . | . | . | . | . | . | . |
| . | . | . | . | . | | | |
| Vv13g1031 | . | . | . | . | . | . | . |
| . | . | . | . | . | . | . | . |
| . | . | . | . | . | | | |
| Vv13g1032 | . | . | . | . | . | . | . |
| . | . | . | . | . | . | . | . |
| . | . | . | . | . | | | |
| Vv13g1033 | . | . | . | . | . | . | . |
| . | . | . | . | . | . | . | . |
| . | . | . | . | . | | | |
| Vv13g1034 | Tc10_g015880 | | . | . | . | . | . |
| . | . | . | . | . | . | . | . |
| . | . | . | . | . | . | | |
| Vv13g1035 | Tc10_g015870 | | . | . | . | . | . |
| . | . | . | . | . | . | . | . |
| . | . | . | . | . | . | | |

```
Vv13g1036          .        .        .        .        .        .        .
         .         .        .        .        .        .        .        .
         .         .        .        .        .
Vv13g1037          .        .        .        .        .        .        .
         .         .        .        .        .        .        .        .
         .         .        .        .        .
Vv13g1038          .        .        .        .        .        .        .
         .         .        .        .        .        .        .        .
         .         .        .        .        .
Vv13g1039          .        .        .        .        .        .        .
         .         .        .        .        .        .        .        .
         .         .        .        .        .
Vv13g1040          .        .        .        .        .        .        .
         .         .        .        .        .        .        .        .
         .         .        .        .        .
Vv13g1041          .        .        .        .        .        .        .
         .         .        .        .        .        .        .        .
         .         .        .        .        .
Vv13g1042          .        .        .        .        .        .        .
         .         .        .        .        .        .        .        .
         .         .        .        .        .
Vv13g1043          .        .        .        .        .        .        .
         .         .        .        .        .        .        .        .
         .         .        .        .        .
Vv13g1044          .        .        .        .        .        .        .
         .         .        .        .        .        .        .        .
         .         .        .        .        .
Vv13g1045          .        .        .        .        .        .        .
         .         .        .        .        .        .        .        .
         .         .        .        .        .
Vv13g1046          .        .        .        .        .        .        .
         .         .        .        .        .        .        .        .
         .         .        .        .        .
Vv13g1047          Tc10_g015870      .        .        .        .        .
         .         .        .        .        .        .        .        .
         .         .        .        .        .        .
Vv13g1048          Tc10_g015880      .        .        Gorai.009G296000.1
         .         .        .        .        .        .        .        .
         .         .        .        .        .        .        .        .
Vv13g1049          .        .        .        .        .        .        .
         .         .        .        .        .        .        .        .
         .         .        .        .        .
Vv13g1050          .        .        .        .        .        .        .
         .         .        .        .        .        .        .        .
         .         .        .        .        .
Vv13g1051          Tc10_g015890      .        .        .        .        .
         .         .        .        .        .        .        .        .
         .         .        .        .        .        .
Vv13g1052          .        .        .        Gorai.009G295900.1       .
         .         .        .        .        .        .        .        .
```

. . . . . . .
Vv13g1053 . . . Gorai.009G295800.1 .
. . . . . . . .
. . . . . . .
Vv13g1054 Tc10_g015910 . . . . .
. . . . . . . .
. . . . . .
Vv13g1055 . . . Gorai.009G295700.1 .
. . . . . . . .
. . . . . . .
Vv13g1056 . . . Gorai.009G292700.1 .
. . . . . . . .
. . . . . . .
Vv13g1057 Tc10_g000590 . . . Gorai.
010G192500.1 . . . . . . .
. . . . . . . .
Vv13g1058 . . . . . . .
. . . . . . . .
. . . . .
Vv13g1059 . . . . . .
Vv14g0398 . . . . . . .
. . . . . .
Vv13g1060 Tc10_g000570 Gorai.013G015600.1 . .
. . . . . . . .
. . . . . . . .
Vv13g1061 Tc10_g000540 . Gorai.011G293500.1 .
. . . . . . . .
. . . . . . . .
Vv13g1062 Tc10_g016110 . . . . .
. . . . . . . .
. . . . . .
Vv13g1063 . . . . . . .
. . . . . . . .
. . . . .
Vv13g1064 . . . . . . .
. . . . . . . .
. . . . .
Vv13g1065 . . . . . . .
. . . . . . . .
. . . . .
Vv13g1066 Tc10_g016120 . . . . .
. . . . . . . .
. . . . . .
Vv13g1067 Tc10_g016130 . . . . .
. . . . . . . .
. . . . . .
Vv13g1068 Tc10_g001330 . . . . .
. . . . . . . .
. . . . . .
Vv13g1069 Tc10_g001320 . . . . .

```
        .        .       .        .       .        .       .        .
        .        .       .        .       .        .
Vv13g1070        .       .        .       .        .       .        .
        .        .       .        .       .        .       .        .
        .        .       .        .       .
Vv13g1071        .       .        .       .        .       .        .
        .        .       .        .       .        .       .        .
        .        .       .        .       .
Vv13g1072        .       .        .       .        .       .        .
        .        .       .        .       .        .       .        .
        .        .       .        .       .
Vv13g1073        Tc10_g000500     .       .        .       .        .
        .        .       .        .       .        .       .        .
        .        .       .        .       .        .
Vv13g1074        Tc10_g000490     .       .        .       .        .
        .        .       .        .       .        .       .        .
        .        .       .        .       .        .
Vv13g1075        Tc10_g000480     Gorai.013G015400.1       .        .
        .        .       .        .       .        .       .        .
        .        .       .        .       .        .       .        .
Vv13g1076        Tc10_g000470     Gorai.013G015300.1       .        .
        .        .       .        .       .        .       .        .
        .        .       .        .       .        .       .        .
Vv13g1077        .       .        .       .        .       .        .
        .        .       .        .       .        .       .        .
        .        .       .        .       .
Vv13g1078        .       .        .       .        .       .        .
        .        .       .        .       .        .       .        .
        .        .       .        .       .
Vv13g1079        Tc10_g000460     .       .        .       .        .
        .        .       .        .       .        .       .        .
        .        .       .        .       .        .
Vv13g1080        .       .        .       .        .       .
Vv14g0418        Tc04_g023450     .       .        .       .        .
        .        .       .        .       .        .       .
Vv13g1081        .       .        .       .        .       .
Vv14g0419        .       .        .       .        .       .        .
        .        .       .        .       .        .
Vv13g1082        .       .        .       .        .       .
Vv14g0421        .       .        Gorai.007G292400.1       .        .
        .        .       .        .       .        .       .        .
Vv13g1083        .       .        .       .        .       .        .
        .        .       .        .       .        .       .        .
        .        .       .        .       .
Vv13g1084        .       .        .       .        .       .
Vv14g0431        Tc04_g027160     .       Gorai.007G292300.1       .
        Gorai.011G183500.1        .       .        .       .        .
        .        .       .
Vv13g1085        .       .        .       .        .       .        .
        .        .       .        .       .        .       .        .
```

. . . . .
Vv13g1086 . . . . . . .
. . . . . . . .
. . . . .
Vv13g1087 . . . . . . .
. . . . . . . .
. . . . .
Vv13g1088 . . . . . . .
. . . . . . . .
. . . . .
Vv13g1089 . . . . . .
Vv14g0433 . . . . . . .
. . . . . .
Vv13g1090 . . . . . .
Vv14g0434 . . Gorai.007G292200.1 Gorai.
013G203800.1 . . . . . . .
. .
Vv13g1091 . . . . . . .
. . . . . . . .
. . . . .
Vv13g1092 . . . . . . .
. . . . . . . .
. . . . .
Vv13g1093 . . . . . . .
. . . . . . . .
. . . . .
Vv13g1094 . . . . . . .
. . . . . . . .
. . . . .
Vv13g1095 . . . . . . .
. . . . . . . .
. . . . .
Vv13g1096 . . . . . . .
. . . . . . . .
. . . . .
Vv13g1097 . . . . . . .
. . . . . . . .
. . . . .
Vv13g1098 . . . . . . .
. . . . . . . .
. . . . .
Vv13g1099 . . . . . . .
. . . . . . . .
. . . . .
Vv13g1100 . . . . . . .
. . . . . . . .
. . . . .
Vv13g1101 Tc10_g000450 . Gorai.011G294900.1 .
. . . . . . . .
. . . . . . . .

Vv13g1102 Tc10_g000440 . . . Gorai.
010G192300.1 . . . . . . .
. . . . . . . .
Vv13g1103 . . . . . . .
. . . . . . . .
. . . . .
Vv13g1104 Tc10_g000410 Gorai.013G014900.1 . .
. . . . . . . .
. . . . . . . .
Vv13g1105 Tc10_g000370 . . . . .
. . . . . . . .
. . . . . .
Vv13g1106 . . . . . . .
. . . . . . . .
. . . . .
Vv13g1107 . . Gorai.011G295300.1 .
Gorai.010G192000.1 . . . . . .
. . . . . . . .
.
Vv13g1108 . . . . . . .
. . . . . . . .
. . . . .
Vv13g1109 Tc10_g000360 . . . . .
. . . . . . . .
. . . . . .
Vv13g1110 Tc10_g000340 . . . . .
. . . . . . . .
. . . . . .
Vv13g1111 Tc10_g000320 . . . . .
. . . . . . . .
. . . . . .
Vv13g1112 . . . . . . .
. . . . . . . .
. . . . .
Vv13g1113 . . . . . . .
. . . . . . . .
. . . . .
Vv13g1114 . . . . . . .
. . . . . . . .
. . . . .
Vv13g1115 . . . . . . .
. . . . . . . .
. . . . .
Vv13g1116 . . . . . . .
. . . . . . . .
. . . . .
Vv13g1117 . . . . . . .
. . . . . . . .
. . . . .
Vv13g1118 . . . . . . .

. . . . . . . .
. . . . .
Vv13g1119 . . . . . . .
. . . . . . . .
. . . . .
Vv13g1120 . . . . . . .
. . . . . . . .
. . . . .
Vv13g1121 . . . . . . .
. . . . . . . .
. . . . .
Vv13g1122 . Gorai.013G013400.1 . .
Gorai.010G189600.1 . . . . . .
. . . . . . . .
.
Vv13g1123 Tc10_g000210 . . . . .
. . . . . . . .
. . . . . .
Vv13g1124 . . . . . . .
. . . . . . . .
. . . . .
Vv13g1125 . . . . . . .
. . . . . . . .
. . . . .
Vv13g1126 . . Gorai.011G295400.1 . .
. . . . . . . .
. . . . . . .
Vv13g1127 . . . . . . .
. . . . . . . .
. . . . .
Vv13g1128 . . . . . . .
. . . . . . . .
. . . . .
Vv13g1129 . . . . . . .
. . . . . . . .
. . . . .
Vv13g1130 . . . . . . .
. . . . . . . .
. . . . .
Vv13g1131 . . . . . . .
. . . . . . . .
. . . . .
Vv13g1132 . . . . . . .
. . . . . . . .
. . . . .
Vv13g1133 . . . . . . .
. . . . . . . .
. . . . .
Vv13g1134 . . . . . . .
. . . . . . . .

| | | | | | | | |
|---|---|---|---|---|---|---|---|
| . | . | . | . | . | | | |
| Vv13g1135 | . | . | . | . | . | . | . |
| . | . | . | . | . | . | . | . |
| . | . | . | . | . | | | |
| Vv13g1136 | . | . | . | . | . | . | . |
| . | . | . | . | . | . | . | . |
| . | . | . | . | . | | | |
| Vv13g1137 | . | . | . | . | . | . | . |
| . | . | . | . | . | . | . | . |
| . | . | . | . | . | | | |
| Vv13g1138 | . | . | . | . | . | . | . |
| . | . | . | . | . | . | . | . |
| . | . | . | . | . | | | |
| Vv13g1139 | . | . | . | . | . | . | . |
| . | . | . | . | . | . | . | . |
| . | . | . | . | . | | | |
| Vv13g1140 | . | . | . | . | . | . | . |
| . | . | . | . | . | . | . | . |
| . | . | . | . | . | | | |
| Vv13g1141 | . | . | . | . | . | . | . |
| . | . | . | . | . | . | . | . |
| . | . | . | . | . | | | |
| Vv13g1142 | . | . | . | . | . | . | . |
| . | . | . | . | . | . | . | . |
| . | . | . | . | . | | | |
| Vv13g1143 | Tc10_g005330 | | . | . | . | . | . |
| . | . | . | . | . | . | . | . |
| . | . | . | . | . | . | | |
| Vv13g1144 | . | . | . | . | . | . | . |
| . | . | . | . | . | . | . | . |
| . | . | . | . | . | | | |
| Vv13g1145 | . | . | . | . | . | . | . |
| . | . | . | . | . | . | . | . |
| . | . | . | . | . | | | |
| Vv13g1146 | . | . | . | . | . | . | . |
| . | . | . | . | . | . | . | . |
| . | . | . | . | . | | | |
| Vv13g1147 | . | . | . | . | . | . | . |
| . | . | . | . | . | . | . | . |
| . | . | . | . | . | | | |
| Vv13g1148 | . | . | . | . | . | . | . |
| . | . | . | . | . | . | . | . |
| . | . | . | . | . | | | |
| Vv13g1149 | Tc10_g006540 | | . | . | . | . | |
| Gorai.012G006300.1 | | . | . | . | . | . | . |
| . | . | . | . | . | . | . | . |
| Vv13g1150 | Tc10_g005210 | | . | . | . | . | . |
| . | . | . | . | . | . | . | . |
| . | . | . | . | . | . | | |
| Vv13g1151 | Tc10_g005200 | | . | . | . | . | . |

| | | | | | | | |
|---|---|---|---|---|---|---|---|
| . | . | . | . | . | . | . | . |
| . | . | . | . | . | . | | |
| Vv13g1152 | . | . | . | . | . | . | . |
| . | . | . | . | . | . | . | . |
| . | . | . | . | . | | | |
| Vv13g1153 | . | . | . | . | . | . | . |
| . | . | . | . | . | . | . | . |
| . | . | . | . | . | | | |
| Vv13g1154 | . | . | . | . | . | . | . |
| . | . | . | . | . | . | . | . |
| . | . | . | . | . | | | |
| Vv13g1155 | . | . | . | . | . | . | . |
| . | . | . | . | . | . | . | . |
| . | . | . | . | . | | | |
| Vv13g1156 | . | . | . | . | . | . | . |
| . | . | . | . | . | . | . | . |
| . | . | . | . | . | | | |
| Vv13g1157 | . | . | . | . | . | . | . |
| . | . | . | . | . | . | . | . |
| . | . | . | . | . | | | |
| Vv13g1158 | . | . | . | . | . | . | . |
| . | . | . | . | . | . | . | . |
| . | . | . | . | . | | | |
| Vv13g1159 | . | . | . | . | . | . | . |
| . | . | . | . | . | . | . | . |
| . | . | . | . | . | | | |
| Vv13g1160 | . | . | . | . | . | . | . |
| . | . | . | . | . | . | . | . |
| . | . | . | . | . | | | |
| Vv13g1161 | . | . | . | . | . | . | . |
| . | . | . | . | . | . | . | . |
| . | . | . | . | . | | | |
| Vv13g1162 | . | . | . | . | . | . | . |
| . | . | . | . | . | . | . | . |
| . | . | . | . | . | | | |
| Vv13g1163 | . | . | . | . | . | . | . |
| . | . | . | . | . | . | . | . |
| . | . | . | . | . | | | |
| Vv13g1164 | . | . | . | . | . | . | . |
| . | . | . | . | . | . | . | . |
| . | . | . | . | . | | | |
| Vv13g1165 | Tc10_g006230 | | . | . | . | . | . |
| . | . | . | . | . | . | . | . |
| . | . | . | . | . | . | | |
| Vv13g1166 | . | . | . | . | . | . | . |
| . | . | . | . | . | . | . | . |
| . | . | . | . | . | | | |
| Vv13g1167 | Tc10_g006220 | | . | . | . | . | . |
| . | . | . | . | . | . | . | . |
| . | . | . | . | . | . | | |

Vv13g1168 . . . . . . .
. . . . . . . .
. . . . .
Vv13g1169 . . . . . . .
. . . . . . . .
. . . . .
Vv13g1170 . . . . . . .
. . . . . . . .
. . . . .
Vv13g1171 . . . . . . .
. . . . . . . .
. . . . .
Vv13g1172 . . . . . . .
. . . . . . . .
. . . . .
Vv13g1173 . . . . . . .
. . . . . . . .
. . . . .
Vv13g1174 . . . . . . .
. . . . . . . .
. . . . .
Vv13g1175 . . . . . . .
. . . . . . . .
. . . . .
Vv13g1176 . . . . . . .
. . . . . . . .
. . . . .
Vv13g1177 . . . . . . .
. . . . . . . .
. . . . .
Vv13g1178 . . . . . . .
. . . . . . . .
. . . . .
Vv13g1179 . . . . . . .
. . . . . . . .
. . . . .
Vv13g1180 . . . . . . .
. . . . . . . .
. . . . .
Vv13g1181 . . . . . . .
. . . . . . . .
. . . . .
Vv13g1182 . . . . . . .
. . . . . . . .
. . . . .
Vv13g1183 . . . . . . .
. . . . . . . .
. . . . .
Vv13g1184 . . . . . . .
. . . . . . . .

| | | | | | | | |
|---|---|---|---|---|---|---|---|
| . | . | . | . | . | | | |
| Vv13g1185 | . | . | . | . | . | . | . |
| . | . | . | . | . | . | . | . |
| . | . | . | . | . | | | |
| Vv13g1186 | . | . | . | . | . | . | . |
| . | . | . | . | . | . | . | . |
| . | . | . | . | . | | | |
| Vv13g1187 | . | . | . | . | . | . | |
| Vv08g1825 | . | . | . | . | . | . | . |
| . | . | . | . | . | . | | |
| Vv13g1188 | . | . | . | . | . | . | . |
| . | . | . | . | . | . | . | . |
| . | . | . | . | . | | | |
| Vv13g1189 | . | . | . | . | . | . | . |
| . | . | . | . | . | . | . | . |
| . | . | . | . | . | | | |
| Vv13g1190 | . | . | . | . | . | . | . |
| . | . | . | . | . | . | . | . |
| . | . | . | . | . | | | |
| Vv13g1191 | . | . | . | . | . | . | . |
| . | . | . | . | . | . | . | . |
| . | . | . | . | . | | | |
| Vv13g1192 | . | . | . | . | . | . | . |
| . | . | . | . | . | . | . | . |
| . | . | . | . | . | | | |
| Vv13g1193 | Tc10_g006210 | | . | . | . | . | . |
| . | . | . | . | . | . | . | . |
| . | . | . | . | . | . | | |
| Vv13g1194 | . | . | . | . | . | . | . |
| . | . | . | . | . | . | . | . |
| . | . | . | . | . | | | |
| Vv13g1195 | . | . | . | . | . | . | . |
| . | . | . | . | . | . | . | . |
| . | . | . | . | . | | | |
| Vv13g1196 | . | . | . | . | . | . | . |
| . | . | . | . | . | . | . | . |
| . | . | . | . | . | | | |
| Vv13g1197 | . | . | . | . | . | . | |
| Vv08g1799 | Tc05_g016710 | | . | . | . | . | . |
| . | . | . | . | . | . | . | |
| Vv13g1198 | . | . | . | . | . | . | . |
| . | . | . | . | . | . | . | . |
| . | . | . | . | . | | | |
| Vv13g1199 | . | . | . | . | . | . | . |
| . | . | . | . | . | . | . | . |
| . | . | . | . | . | | | |
| Vv13g1200 | . | . | . | . | . | . | . |
| . | . | . | . | . | . | . | . |
| . | . | . | . | . | | | |
| Vv13g1201 | Tc10_g006160 | | . | . | . | . | . |

| | | | | | | | |
|---|---|---|---|---|---|---|---|
| . | . | . | . | . | . | . | . |
| . | . | . | . | . | . | | |
| Vv13g1202 | Tc10_g006150 | | . | . | . | . | . |
| . | . | . | . | . | . | . | . |
| . | . | . | . | . | . | | |
| Vv13g1203 | Tc10_g013490 | | . | . | . | . | . |
| . | . | . | . | . | . | . | . |
| . | . | . | . | . | . | | |
| Vv13g1204 | Tc10_g013480 | | . | . | . | . | . |
| . | . | . | . | . | . | . | . |
| . | . | . | . | . | . | | |
| Vv13g1205 | . | . | . | . | . | . | . |
| . | . | . | . | . | . | . | . |
| . | . | . | . | . | | | |
| Vv13g1206 | Tc10_g013470 | | . | . | . | . | . |
| . | . | . | . | . | . | . | . |
| . | . | . | . | . | . | | |
| Vv13g1207 | . | . | . | . | . | . | |
| Vv08g1798 | . | . | . | . | . | . | . |
| . | . | . | . | . | . | | |
| Vv13g1208 | . | . | . | . | . | . | |
| Vv08g1793 | Tc05_g016440 | | . | . | . | . | . |
| . | . | . | . | . | . | . | |
| Vv13g1209 | . | . | . | . | . | . | . |
| . | . | . | . | . | . | . | . |
| . | . | . | . | . | | | |
| Vv13g1210 | . | . | . | . | . | . | . |
| . | . | . | . | . | . | . | . |
| . | . | . | . | . | | | |
| Vv13g1211 | . | . | . | . | . | . | |
| Vv08g1792 | Tc05_g016430 | | . | . | . | . | . |
| . | . | . | . | . | . | . | |
| Vv13g1212 | . | . | . | . | . | . | . |
| . | . | . | . | . | . | . | . |
| . | . | . | . | . | | | |
| Vv13g1213 | . | . | . | . | . | . | . |
| . | . | . | . | . | . | . | . |
| . | . | . | . | . | | | |
| Vv13g1214 | . | . | . | . | . | . | . |
| . | . | . | . | . | . | . | . |
| . | . | . | . | . | | | |
| Vv13g1215 | . | . | . | . | . | . | . |
| . | . | . | . | . | . | . | . |
| . | . | . | . | . | | | |
| Vv13g1216 | . | . | . | . | . | . | . |
| . | . | . | . | . | . | . | . |
| . | . | . | . | . | | | |
| Vv13g1217 | Tc10_g006120 | | . | . | . | . | . |
| . | . | . | . | . | . | . | . |
| . | . | . | . | . | . | | |

Vv13g1218 . . . . . . .
. . . . . . . .
. . . . .
Vv13g1219 . . . . . . .
. . . . . . . .
. . . . .
Vv13g1220 . . . . . . .
. . . . . . . .
. . . . .
Vv13g1221 . . . . . . .
. . . . . . . .
. . . . .
Vv13g1222 . . . . . . .
. . . . . . . .
. . . . .
Vv13g1223 . . . . . . .
. . . . . . . .
. . . . .
Vv13g1224 . . . . . . .
. . . . . . . .
. . . . .
Vv13g1225 . . . . . . .
. . . . . . . .
. . . . .
Vv13g1226 . . . . . . .
. . . . . . . .
. . . . .
Vv13g1227 . . . . . .
Vv08g1776 . . . . . Gorai.
007G360200.1 . . . . . . .
Vv13g1228 . . . . . . .
. . . . . . . .
. . . . .
Vv13g1229 . . . . . . .
. . . . . . . .
. . . . .
Vv13g1230 . . . . . . .
. . . . . . . .
. . . . .
Vv13g1231 . . . . . . .
. . . . . . . .
. . . . .
Vv13g1232 . . . . . . .
. . . . . . . .
. . . . .
Vv13g1233 . . . . . . .
. . . . . . . .
. . . . .
Vv13g1234 . . . . . . .
. . . . . . . .

| | | | | | | | |
|---|---|---|---|---|---|---|---|
| . | . | . | . | . | | | |
| Vv13g1235 | . | . | . | . | . | . | . |
| . | . | . | . | . | . | . | . |
| . | . | . | . | . | | | |
| Vv13g1236 | . | . | . | . | . | . | . |
| . | . | . | . | . | . | . | . |
| . | . | . | . | . | | | |
| Vv13g1237 | . | . | . | . | . | . | |
| Vv08g1775 | . | . | . | . | . | . | . |
| . | . | . | . | . | . | | |
| Vv13g1238 | Tc10_g013170 | | . | Gorai.011G222300.1 | | | . |
| . | . | . | . | . | . | . | . |
| . | . | . | . | . | . | . | . |
| Vv13g1239 | . | . | . | . | . | . | |
| Vv08g1774 | . | . | . | . | . | . | . |
| . | . | . | . | . | . | | |
| Vv13g1240 | . | . | . | . | . | . | . |
| . | . | . | . | . | . | . | . |
| . | . | . | . | . | | | |
| Vv13g1241 | . | . | . | . | . | . | . |
| . | . | . | . | . | . | . | . |
| . | . | . | . | . | | | |
| Vv13g1242 | . | . | . | . | . | . | . |
| . | . | . | . | . | . | . | . |
| . | . | . | . | . | | | |
| Vv13g1243 | . | . | . | . | . | . | . |
| . | . | . | . | . | . | . | . |
| . | . | . | . | . | | | |
| Vv13g1244 | Tc10_g006080 | | . | . | . | . | . |
| . | . | . | . | . | . | . | . |
| . | . | . | . | . | . | | |
| Vv13g1245 | Tc10_g006070 | | . | . | . | . | . |
| . | . | . | . | . | . | . | . |
| . | . | . | . | . | . | | |
| Vv13g1246 | . | . | . | . | . | . | . |
| . | . | . | . | . | . | . | . |
| . | . | . | . | . | | | |
| Vv13g1247 | . | . | . | . | . | . | . |
| . | . | . | . | . | . | . | . |
| . | . | . | . | . | | | |
| Vv13g1248 | . | . | . | . | . | . | . |
| . | . | . | . | . | . | . | . |
| . | . | . | . | . | | | |
| Vv13g1249 | . | . | . | . | . | . | . |
| . | . | . | . | . | . | . | . |
| . | . | . | . | . | | | |
| Vv13g1250 | . | . | . | . | . | . | . |
| . | . | . | . | . | . | . | . |
| . | . | . | . | . | | | |
| Vv13g1251 | . | . | . | . | . | . | . |

. . . . . . . .
. . . . .
Vv13g1252 . . . . . . .
. . . . . . . .
. . . . .
Vv13g1253 . . . . . . .
. . . . . . . .
. . . . .
Vv13g1254 . . . . . . .
. . . . . . . .
. . . . .
Vv13g1255 . . . . . . .
. . . . . . . .
. . . . .
Vv13g1256 . . . . . .
Vv08g1766 . . . . . . .
. . . . . .
Vv13g1257 . . . . . . .
. . . . . . Vv06g0272
. Gorai.013G251200.1 . . . .
Vv13g1258 . . . . . . .
. . . . . . . .
. . . . .
Vv13g1259 . . . . . . .
. . . . . . . .
. . . . .
Vv13g1260 . . . . . . .
. . . . . . . .
. . . . .
Vv13g1261 . . . . . . .
. . . . . . Vv06g0269
. . . . Gorai.009G073300.1
Gorai.006G128600.1
Vv13g1262 . . . . . .
Vv08g1764 . . . . . .
Vv06g0266 . . Gorai.001G138300.1 Gorai.
004G037500.1 . Gorai.006G138100.1
Vv13g1263 . . . . . . .
. . . . . . . .
. . . . .
Vv13g1264 . . . . . .
Vv08g1763 . . Gorai.002G230300.1 . .
. . . . . . . .
Vv13g1265 . . . . . . .
. . . . . . Vv06g0264
. . . Gorai.004G037400.1 .
Gorai.006G128800.1
Vv13g1266 . . . . . . .
. . . . . . . .
. . . . .

Vv13g1267 . . . . . . .
. . . . . . . .
. . . . .
Vv13g1268 . . . . . . .
. . . . . . . .
. . . . .
Vv13g1269 . . . . . . .
. . . . . . . .
. . . . .
Vv13g1270 . . . . . . .
. . . . . . . .
. . . . .
Vv13g1271 . . . . . . .
. . . . . . . .
. . . . .
Vv13g1272 . . . . . . .
. . . . . . . .
. . . . .
Vv13g1273 . . . . . . .
. . . . . . . .
. . . . .
Vv13g1274 . . . . . . .
. . . . . . . .
. . . . .
Vv13g1275 . . . . . . .
. . . . . . . .
. . . . .
Vv13g1276 . . . . . . .
. . . . . . . .
. . . . .
Vv13g1277 . . . . . . .
. . . . . . . .
. . . . .
Vv13g1278 . . . . . . .
. . . . . . . .
. . . . .
Vv13g1279 . . . . . . .
. . . . . . . .
. . . . .
Vv13g1280 . . . . . .
Vv08g1748 . . . . . .
Vv06g0236 . Gorai.013G250800.1 . Gorai.
004G037000.1 . Gorai.006G138700.1
Vv13g1281 . . . . . . .
. . . . . . . .
. . . . .
Vv13g1282 . . . . . . .
. . . . . . . .
. . . . .
Vv13g1283 . . . . . . .

. . . . . . . .
. . . . .
Vv13g1284 . . . . . . .
. . . . . . . .
. . . . .
Vv13g1285 . . . . . . .
. . . . . . . .
. . . . .
Vv13g1286 . . . . . . .
. . . . . . . .
. . . . .
Vv13g1287 . . . . . . .
. . . . . . . .
. . . . .
Vv13g1288 . . . . . . .
. . . . . . . .
. . . . .
Vv13g1289 . . . . . . .
. . . . . . . .
. . . . .
Vv13g1290 . . . . . .
Vv08g1741 Tc05_g008610 . Gorai.002G229200.1 .
. Gorai.007G358100.1 Vv06g0229 . .
. Gorai.004G036200.1 . .
Vv13g1291 . . . . . . .
. . . . . . . .
. . . . .
Vv13g1292 . . . . . . .
. . . . . . . .
. . . . .
Vv13g1293 . . . . . . .
. . . . . . . .
. . . . .
Vv13g1294 . . . . . . .
. . . . . . . .
. . . . .
Vv13g1295 . . . . . . .
. . . . . . . .
. . . . .
Vv13g1296 . . . . . . .
. . . . . . . .
. . . . .
Vv13g1297 . . . . . . .
. . . . . . . .
. . . . .
Vv13g1298 . . . . . . .
. . . . . . . .
. . . . .
Vv13g1299 . . . . . . .
. . . . . . . .

. . . . .
Vv13g1300 Tc10_g006250 . . . . .
. . . . . . . .
. . . . . .
Vv13g1301 . . . . . . .
. . . . . . . .
. . . . .
Vv13g1302 . . . . . . .
. . . . . . . .
. . . . .
Vv13g1303 . . . . . . .
. . . . . . . .
. . . . .
Vv13g1304 . . . . . . .
. . . . . . . .
. . . . .
Vv13g1305 . . . . . . .
. . . . . . . .
. . . . .
Vv13g1306 . . . . . .
Vv06g0219 . . . . . . .
. . . . . .
Vv13g1307 . . . . . . .
. . . . . . . .
. . . . .
Vv13g1308 . . . . . . .
. . . . . . . .
. . . . .
Vv13g1309 . . . . . . .
. . . . . . . .
. . . . .
Vv13g1310 . . . . . . .
. . . . . . . .
. . . . .
Vv13g1311 . . . . . . .
. . . . . . . .
. . . . .
Vv13g1312 . . . . . . .
. . . . . . . .
. . . . .
Vv13g1313 . . . . . . .
. . . . . . . .
. . . . .
Vv13g1314 . . . . . . .
. . . . . . . .
. . . . .
Vv13g1315 . . . . . . .
. . . . . . . .
. . . . .
Vv13g1316 . . . . . . .

. . . . . . . .
. . . . .
Vv13g1317 . . . . . . .
. . . . . . . .
. . . . .
Vv13g1318 . . . . . . .
. . . . . . . .
. . . . .
Vv13g1319 . . . . . . .
. . . . . . . .
. . . . .
Vv13g1320 . . . . . . .
. . . . . . . .
. . . . .
Vv13g1321 . . . . . . .
. . . . . . . .
. . . . .
Vv13g1322 . . . . . . .
. . . . . . . .
. . . . .
Vv13g1323 Tc10_g006390 . . . . .
Vv06g0194 . . . . . .
. . . . . . .
Vv13g1324 . . . . . .
Vv06g0192 . . . . . . .
. . . . . .
Vv13g1325 . . . . . .
Vv06g0191 . . . . Gorai.009G071200.1
. . . . . . . .
Vv13g1326 . . . . . . .
. . . . . . . .
. . . . .
Vv13g1327 . . . . . . .
. . . . . . . .
. . . . .
Vv13g1328 . . . . . .
Vv06g0187 . . . . . . .
. . . . . .
Vv13g1329 . . . . . . .
. . . . . . . .
. . . . .
Vv14g0001 . . . . . . .
. . . . . . . .
. . . . .
Vv14g0002 . . . . . . .
. . . . . . . .
. . . . .
Vv14g0003 . . . . . . .
. . . . . . . .
. . . . .

Vv14g0004 Tc05_g004980 . Gorai.002G261200.1 .
Gorai.012G101800.1 . . . . .
. . . . . . . .
. .
Vv14g0005 Tc04_g005500 . . . . .
. . . . . . . .
. . . . . .
Vv14g0006 . . . . . . .
. . . . . . . .
. . . . .
Vv14g0007 . . . . . . .
. . . . . . . .
. . . . .
Vv14g0008 . . . . . .
Vv05g0331 . Gorai.006G263500.1 . . .
Gorai.002G028200.1 . . . . .
. .
Vv14g0009 . . . . . . .
. . . . . . . .
. . . . .
Vv14g0010 . . . . . . .
. . . . . . . .
. . . . .
Vv14g0011 . . . . . . .
. . . . . . . .
. . . . .
Vv14g0012 . . . . . . .
. . . . . . . .
. . . . .
Vv14g0013 . . . . . . .
. . . . . . . .
. . . . .
Vv14g0014 . . . . . . .
. . . . . . . .
. . . . .
Vv14g0015 . . . . . . .
. . . . . . . .
. . . . .
Vv14g0016 . . . . . . .
. . . . . . . .
. . . . .
Vv14g0017 . . . . . . .
. . . . . . . .
. . . . .
Vv14g0018 Tc04_g005140 . Gorai.001G275900.1 .
. Gorai.012G082800.1 . . . .
. . . . . . . .
. .
Vv14g0019 . . . . . . .
. . . . . . . .

.       .       .       .       .
Vv14g0020       .       Gorai.013G131700.1       .       .       .
.       .       .       .       .       .       .       .
.       .       .       .       .       .       .
Vv14g0021       .       .       .       .       .       .       .
.       .       .       .       .       .       .       .
.       .       .       .       .
Vv14g0022       .       .       .       .       .       .       .
.       .       .       .       .       .       .       .
.       .       .       .       .
Vv14g0023       .       .       .       .       .       .       .
.       .       .       .       .       .       .       .
.       .       .       .       .
Vv14g0024       .       .       .       .       .       .       .
.       .       .       .       .       .       .       .
.       .       .       .       .
Vv14g0025       .       .       .       .       .       .       .
.       .       .       .       .       .       .       .
.       .       .       .       .
Vv14g0026       .       .       .       .       .       .       .
.       .       .       .       .       .       .       .
.       .       .       .       .
Vv14g0027       Tc05_g004610       Gorai.013G131600.1       .
Gorai.013G211000.1       .       .       Vv05g0304       .       .
.       .       .       .       Vv07g0619       .
Gorai.007G118400.1       .       .       .       .
Vv14g0028       .       .       .       .       .       .       .
.       .       .       .       .       .       .       .
.       .       .       .       .
Vv14g0029       .       .       .       .       .       .       .
.       .       .       .       .       .       .       .
.       .       .       .       .
Vv14g0030       Tc04_g005090       .       Gorai.007G309400.1
Gorai.007G118100.1       .       .       .       .       .       .
.       .       .       .       .       .       .       .
.       .
Vv14g0031       .       .       .       .       .       .       .
.       .       .       .       .       .       .       .
.       .       .       .       .
Vv14g0032       .       .       .       .       .       .       .
.       .       .       .       .       .       .       .
.       .       .       .       .
Vv14g0033       .       .       .       .       .       .       .
.       .       .       .       .       .       .       .
.       .       .       .       .
Vv14g0034       .       .       .       Gorai.013G210200.1       .
.       Vv05g0297       .       .       .       .       .
.       .       .       .       .       .       .       .
Vv14g0035       .       .       .       .       .       .       .
.       .       .       .       .       .       .       .

```
          .         .         .         .         .
Vv14g0036           .         .         .         Gorai.013G210000.1            .
          .         .         .         .         .         .         .         .
          .         .         .         .         .         .         .
Vv14g0037           Tc04_g030740        .         .         .         .         .
          .         .         .         .         .         .         .         .
          .         .         .         .         .         .
Vv14g0038           .         .         .         .         .         .         .
          .         .         .         .         .         .         .         .
          .         .         .         .         .
Vv14g0039           Tc04_g030730        .         .         .         .         .
          .         .         .         .         .         .         .         .
          .         .         .         .         .         .
Vv14g0040           .         .         .         .         .         .
Vv05g0292           Tc04_g030740        .         Gorai.005G073900.1            .
          .         .         .         .         .         .         .         .
          .
Vv14g0041           .         .         .         .         .         .         .
          .         .         .         .         .         .         .         .
          .         .         .         .         .
Vv14g0042           .         .         .         .         .         .         .
          .         .         .         .         .         .         .         .
          .         .         .         .         .
Vv14g0043           Tc04_g030610        .         .         Gorai.013G209800.1
          .         .         .         .         .         .         .         .
          .         .         .         .         .         .         .         .
Vv14g0044           Tc04_g030600        .         .         .         .         .
          Vv05g0278           Tc04_g030600        .         .         .         .
          .         .         .         .         .         .         .         .
Vv14g0045           .         .         .         .         .         .         .
          .         .         .         .         .         .         .         .
          .         .         .         .         .
Vv14g0046           .         .         .         .         .         .         .
          .         .         .         .         .         .         Vv07g0598
          Tc01_g032740        .         Gorai.008G135500.1            .         .
          .
Vv14g0047           Tc04_g030560        .         .         .         .
Gorai.002G032100.1            Vv05g0275           Tc04_g030560        .         .
          .         Gorai.011G205200.1            .         .         .         .
          .         .         .         .
Vv14g0048           .         .         .         .         .         .         .
          .         .         .         .         .         .         .         .
          .         .         .         .         .
Vv14g0049           .         Gorai.013G131200.1            .         .         .
          .         .         .         .         .         .         .         .
          Vv07g0597           .         .         .         .         .         .
Vv14g0050           .         .         Gorai.007G309000.1            .         .
          .         .         .         .         .         .         .         .
          .         .         .         .         .         .         .
Vv14g0051           .         Gorai.013G131100.1            .         .         .
```

```
        .       .       .       .       .       .       .       .
        .       .       .       .       .       .       .
Vv14g0052       Tc05_g004510    .       .       .       Gorai.
012G104300.1    .       .       .       .       .       .       .
        .       .       .       .       .       .       .       .
Vv14g0053       Tc04_g030480    .       .       .       Gorai.
011G205000.1    .       .       .       .       .       .       .
        .       .       .       .       .       .       .       .
Vv14g0054       Tc04_g030470    .       .       .       Gorai.
011G204900.1    .       .       .       .       .       .       .
        .       .       .       .       .       .       .       .
Vv14g0055       Tc04_g030460    .       .       Gorai.007G119200.1
        .       .       Vv05g0266       Tc04_g030460    .
Gorai.005G071300.1      .       .       .       Vv07g0590
Tc01_g032660    Gorai.007G119200.1      Gorai.008G135900.1      .
        .       Gorai.004G146800.1
Vv14g0056       .       .       .       .       .       .       .
        .       .       .       .       .       .       .       .
        .       .       .       .       .
Vv14g0057       Tc04_g016760    .       .       .       Gorai.
011G129900.1    .       .       .       .       .       .       .
        .       .       .       .       .       .       .       .
Vv14g0058       .       .       .       .       .       .       .
        .       .       .       .       .       .       .       .
        .       .       .       .       .
Vv14g0059       Tc04_g016740    .       .       .       Gorai.
011G129800.1    .       .       .       .       .       .       .
        .       .       .       .       .       .       .       .
Vv14g0060       .       .       .       .       .       .       .
        .       .       .       .       .       .       .       .
        .       .       .       .       .
Vv14g0061       Tc04_g016340    .       .       .       .       .
        .       .       .       .       .       .       .       .
        .       .       .       .       .       .
Vv14g0062       .       .       Gorai.007G308700.1      .       .
        .       .       .       .       .       .       .       .
        .       .       .       .       .       .       .
Vv14g0063       .       .       .       .       .       .       .
        .       .       .       .       .       .       .       .
        .       .       .       .       .
Vv14g0064       .       .       .       Gorai.013G209300.1      .
        .       .       .       .       .       .       .       .
        .       .       .       .       .       .       .
Vv14g0065       .       .       Gorai.007G308600.1      Gorai.
013G209200.1    .       .       .       .       .       .       .
        .       .       .       .       .       .       .       .
        .
Vv14g0066       .       .       .       .       .       .       .
        .       .       .       .       .       .       .       .
        .       .       .       .       .
```

Vv14g0067 . . . . . . .
. . . . . . . .
. . . . .
Vv14g0068 Tc04_g016080 . . . . .
. . . . . . . .
. . . . . .
Vv14g0069 . . . . . . .
. . . . . . . .
. . . . .
Vv14g0070 . . . . . . .
. . . . . . . .
. . . . .
Vv14g0071 . . . . . . .
. . . . . . . .
. . . . .
Vv14g0072 Tc04_g030230 . . . . .
Vv05g0237 Tc04_g030230 Gorai.006G266100.1
Gorai.005G069900.1 . . . Vv07g0575
Tc01_g032480 . Gorai.008G136700.1 . . .
Vv14g0073 . . . Gorai.013G209000.1 .
. . . . . . . .
. . . . . . .
Vv14g0074 . . Gorai.007G308400.1 . .
. . . . . . . .
. . . . . . .
Vv14g0075 Tc05_g009390 . . . . .
. . . . . . . .
. . . . . .
Vv14g0076 . . . . . . .
. . . . . . . .
. . . . .
Vv14g0077 . . . . . . .
. . . . . . . .
. . . . .
Vv14g0078 . . . . . . .
. . . . . . . .
. . . . .
Vv14g0079 . Gorai.013G131000.1 . Gorai.
007G120600.1 . . . . . . .
. . . . . . . .
.
Vv14g0080 Tc05_g004500 . . . . .
. . . . . . . .
. . . . . .
Vv14g0081 . . . . . . .
. . . . . . . .
. . . . .
Vv14g0082 Tc04_g030130 Gorai.013G130900.1 .
Gorai.007G120900.1 . . Vv05g0228 . .
. . . . Vv07g0565

Tc01_g032360 . . . . .
Vv14g0083 Tc04_g030120 . . . . .
. . . . . . . .
. . . . . .
Vv14g0084 . . . . . . .
. . . . . . . .
. . . . .
Vv14g0085 . Gorai.013G130800.1 . . .
. . . . . . . .
Vv07g0563 . Gorai.007G120900.1 . .
. .
Vv14g0086 . . . . . . .
. . . . . . . .
. . . . .
Vv14g0087 . . . . . . .
. . . . . . . .
. . . . .
Vv14g0088 . . . . . . .
. . . . . . . .
. . . . .
Vv14g0089 . . . . . . .
. . . . . . . .
. . . . .
Vv14g0090 . . . . . . .
. . . . . . . .
. . . . .
Vv14g0091 . . . . . . .
. . . . . . . .
. . . . .
Vv14g0092 . . . . . . .
. . . . . . . .
. . . . .
Vv14g0093 . . . . . . .
. . . . . . . .
. . . . .
Vv14g0094 . . . . . .
Vv05g0193 Tc04_g030120 Gorai.006G265600.1 . .
. . . . . . . .
.
Vv14g0095 . . Gorai.007G308300.1 . .
. . . . . . . .
. . . . . . .
Vv14g0096 . . Gorai.007G308200.1 . .
. . . . . . . .
Vv07g0562 Tc01_g032350 . . . .
Gorai.004G145900.1
Vv14g0097 . . . Gorai.013G208400.1 .
. . . . . . . .
. . . . . . .
Vv14g0098 . . . Gorai.013G208300.1 .

. . . . . . . .
. . . . . . .
Vv14g0099 Tc04_g030070 Gorai.013G130600.1 Gorai.
007G308100.1 Gorai.013G208200.1 . . Vv05g0188
Tc04_g030070 . . . Gorai.011G203600.1
. . . . . . . .
Vv14g0100 Tc04_g023940 . . . . .
. . . . . . . .
. . . . . .
Vv14g0101 Tc04_g024140 . . . Gorai.
011G165700.1 . . . . . . .
. . . . . . . .
Vv14g0102 Tc04_g030020 . . Gorai.013G208100.1
. Gorai.002G031600.1 . . . .
. . . Vv07g0546 Tc01_g032270 .
. . . Gorai.004G145200.1
Vv14g0103 Tc04_g030010 Gorai.013G130500.1 .
Gorai.013G208000.1 Gorai.011G203200.1 . . .
. . . . . . . .
. . . .
Vv14g0104 . Gorai.013G130400.1 . . .
. . . . . . . .
. . . . . . .
Vv14g0105 . Gorai.013G130300.1 . . .
. . . . . . . .
. . . . . . .
Vv14g0106 Tc04_g029940 . . . . .
. . . . . . . .
. . . . . .
Vv14g0107 . . . . . . .
. . . . . . . .
. . . . .
Vv14g0108 Tc05_g009270 . . . . .
. . . . . . . .
. . . . . .
Vv14g0109 . . . . . . .
. . . . . . . .
. . . . .
Vv14g0110 . . . . . . .
. . . . . . . .
. . . . .
Vv14g0111 Tc04_g029860 . . . Gorai.
011G202000.1 . Vv05g0171 Tc04_g029860 Gorai.
006G265400.1 . Gorai.006G080200.1 Gorai.011G202000.1
Gorai.002G031000.1 Vv07g0540 Tc01_g032010 .
. . . .
Vv14g0112 Tc04_g029780 . . . . .
Vv05g0163 Tc04_g029780 Gorai.006G265000.1
Gorai.005G067900.1 . Gorai.011G201700.1 Gorai.
002G030700.1 . . . . . . .

Vv14g0113 . Gorai.013G130100.1 . . .
. . . . . . . .
. . . . . . .
Vv14g0114 . . . . . . .
. . . . . . . .
. . . . .
Vv14g0115 Tc04_g029610 . . . .
Gorai.002G030400.1 Vv05g0148 Tc04_g029610 . .
. . Gorai.002G030400.1 . . .
. . . .
Vv14g0116 . Gorai.013G129700.1 . . .
. . . . . . . .
. . . . . . .
Vv14g0117 . . Gorai.007G307200.1 . .
. . . . . . . .
. . . . . . .
Vv14g0118 . . . . . . .
. . . . . . . .
. . . . .
Vv14g0119 . . . . . . .
. . . . . . . .
. . . . .
Vv14g0120 Tc04_g024520 . . . . .
. . . . . . . .
. . . . . .
Vv14g0121 . . . . . . .
. . . . . . . .
. . . . .
Vv14g0122 Tc04_g029410 . Gorai.007G306800.1 .
. . Vv05g0131 Tc04_g029410 .
Gorai.005G066400.1 . . . Vv07g0522
Tc01_g031800 . . . . .
Vv14g0123 . . . . . . .
. . . . . . . .
. . . . .
Vv14g0124 . . . . . . .
. . . . . . . .
. . . . .
Vv14g0125 . . Gorai.007G306700.1 . .
. . . . . . . .
. . . . . . .
Vv14g0126 . Gorai.013G129500.1 Gorai.007G306600.1
. . . . . . . .
. . . . . . . .
.
Vv14g0127 . . . . . . .
. . . . . . . .
. . . . .
Vv14g0128 . Gorai.013G129400.1 Gorai.007G306500.1
. . . . . . . .

. . . . . . . .
.
Vv14g0129 Tc04_g029370 . Gorai.007G306400.1
Gorai.013G207700.1 . . . . . .
. . . . . . . .
. .
Vv14g0130 . . . Gorai.013G207000.1 .
. . . . . . . .
. . . . . . .
Vv14g0131 Tc04_g029350 . . Gorai.007G122800.1
. . Vv05g0126 Tc04_g029350 . .
Gorai.006G079800.1 . . Vv07g0509
Tc01_g031670 Gorai.007G122800.1 Gorai.008G139700.1 .
Gorai.004G050400.1 .
Vv14g0132 . . . . . . .
. . . . . . . .
. . . . .
Vv14g0133 . . Gorai.007G306200.1 . .
. . . . . . . .
. . . . . . .
Vv14g0134 . . . . . . .
. . . . . . . .
. . . . .
Vv14g0135 . . Gorai.007G306100.1 . .
. . . . . . . .
. . . . . . .
Vv14g0136 . . . . . . .
. . . . . . . .
. . . . .
Vv14g0137 . . Gorai.007G306000.1 . .
. . . . . . . .
. . . . . . .
Vv14g0138 . . . . . . .
. . . . . . . .
. . . . .
Vv14g0139 . . . . . . .
. . . . . . . .
. . . . .
Vv14g0140 . . . . . . .
. . . . . . . .
. . . . .
Vv14g0141 . . . . . . .
. . . . . . . .
. . . . .
Vv14g0142 . . . . . . .
. . . . . . . .
. . . . .
Vv14g0143 . Gorai.013G129200.1 Gorai.007G305800.1
Gorai.013G206900.1 . . . .
. . . . Vv07g0501 . .

. . . .
Vv14g0144 . . . . . . .
. . . . . Vv07g0497
Tc01_g031580 . Gorai.008G140200.1 . .
Gorai.004G144200.1
Vv14g0145 . . . . . . .
. . . . . . . .
. . . . .
Vv14g0146 . . Gorai.007G305600.1 . .
. . . . . . . .
. . . . . . .
Vv14g0147 Tc04_g029230 . . . . .
Vv05g0119 Tc04_g029230 Gorai.006G259300.1 .
Gorai.006G079100.1 . . . . .
. . . .
Vv14g0148 Tc04_g029200 Gorai.013G129100.1 . .
. . Vv05g0116 Tc04_g029210 .
Gorai.005G065800.1 . Gorai.011G198700.1 . .
. . . . . .
Vv14g0149 . . . . . . .
. . . . . . . .
. . . . .
Vv14g0150 Tc04_g029140 . . . . .
. . . . . . . .
. . . . . .
Vv14g0151 Tc04_g024580 . . . . .
. . . . . . . .
. . . . . .
Vv14g0152 . . . . . . .
. . . . . . . .
. . . . .
Vv14g0153 . . . . . . .
. . . . . . . .
. . . . .
Vv14g0154 . . . Gorai.007G124200.1 .
. . . . . . . .
. . . . . . .
Vv14g0155 Tc04_g024840 . . . . .
. . . . . . . .
. . . . . .
Vv14g0156 . . . . . . .
. . . . . . . .
. . . . .
Vv14g0157 . . . . . . .
. . . . . . . .
. . . . .
Vv14g0158 . Gorai.013G128900.1 Gorai.007G305200.1
Gorai.007G124400.1 . . . . .
. . . . Vv07g0477
Tc01_g031420 Gorai.007G124400.1 . . . .

Vv14g0159 Tc04_g029090 Gorai.013G128700.1 . .
. . Vv05g0101 Tc04_g029090 . .
. . . . . . . .
. .
Vv14g0160 . . . . . . .
. . . . . . . .
. . . . .
Vv14g0161 . . . . . . .
. . . . . . . .
. . . . .
Vv14g0162 . . . . . . .
. . . . . . . .
. . . . .
Vv14g0163 . Gorai.013G128600.1 . . .
. . . . . . . .
. . . . . . .
Vv14g0164 . . . . . . .
. . . . . . . .
. . . . .
Vv14g0165 . Gorai.013G128200.1 . . .
. . . . . . . .
. . . . . . .
Vv14g0166 . Gorai.013G128100.1 . . .
. . . . . . . .
. . . . . . .
Vv14g0167 . . . . . . .
. . . . . . . .
. . . . .
Vv14g0168 Tc05_g002120 . Gorai.007G304100.1 .
. . . . . . . .
. . . . . . . .
Vv14g0169 . . . . . . .
. . . . . . . .
. . . . .
Vv14g0170 . . . . . . .
. . . . . . . .
. . . . .
Vv14g0171 Tc04_g029030 Gorai.013G128000.1 .
Gorai.007G124900.1 . . Vv05g0097
Tc04_g029030 . Gorai.005G065400.1 . .
Gorai.002G029800.1 . . . . . .
.
Vv14g0172 Tc04_g029020 . . . . .
Vv05g0096 Tc04_g029020 . Gorai.005G065300.1
. . Gorai.002G029400.1 Vv07g0465
Tc01_g031290 . . . . .
Vv14g0173 . . . . . . .
. . . . . . . .
. . . . .
Vv14g0174 . Gorai.013G127800.1 Gorai.007G303900.1

Gorai.007G125900.1 . . . . .
. . . . Vv07g0457
Tc01_g031170 Gorai.007G125900.1 . . . .
Vv14g0175 . . . . . . .
. . . . . . . .
. . . . .
Vv14g0176 . . . . . . .
. . . . . . . .
. . . . .
Vv14g0177 . . . . . . .
. . . . . . . .
. . . . .
Vv14g0178 . . . . . . .
. . . . . . . .
. . . . .
Vv14g0179 Tc04_g028970 . . . . .
Vv05g0092 Tc04_g028970 Gorai.006G259800.1
Gorai.005G065100.1 . . . Vv07g0456
Tc01_g031160 Gorai.007G126000.1 . . .
Gorai.004G237800.1
Vv14g0180 . Gorai.013G127600.1 Gorai.007G303700.1
Gorai.013G206200.1 . . . . .
. . . . . . . .
. . .
Vv14g0181 Tc04_g020020 . . . Gorai.
011G150300.1 . . . . . . .
. Vv07g0452 Tc01_g031120 . . .
. .
Vv14g0182 . . . . . . .
. . . . . . . .
. . . . .
Vv14g0183 . . Gorai.007G303500.1 . .
. Vv05g0054 Tc04_g028650 . Gorai.
005G062200.1 . Gorai.011G194000.1 . . .
. . . . .
Vv14g0184 . . Gorai.007G303400.1 . .
. . . . . . . .
. . . . . . .
Vv14g0185 . . . . . . .
. . . . . . . .
. . . . .
Vv14g0186 . . . . . . .
. . . . . . . .
. . . . .
Vv14g0187 . . . . . . .
. . . . . . . .
. . . . .
Vv14g0188 . . Gorai.007G303300.1 . .
. . . . . . . .
. . . . . . .

Vv14g0189 . . . . . . .
. . . . . . .
. . . . .
Vv14g0190 . . . . . . .
. . . . . . .
. . . . .
Vv14g0191 . . . . . . .
. . . . . . .
. . . . .
Vv14g0192 Tc04_g020200 . . . . .
. . . . . . .
. . . . . .
Vv14g0193 Tc04_g028700 . . . Gorai.
011G194100.1 . . . . . . .
. . . . . . .
Vv14g0194 . . . . . . .
. . . . . Vv07g0446
Tc01_g031090 . . . Gorai.004G051500.1
.
Vv14g0195 . . . . . . .
. . . . . . .
. . . . .
Vv14g0196 . . . . . . .
. . . . . . .
. . . . .
Vv14g0197 . . . . . . .
. . . . . . .
. . . . .
Vv14g0198 . . . . . . .
. . . . . . .
. . . . .
Vv14g0199 Tc05_g001730 . . . . .
. . . . . . .
. . . . . .
Vv14g0200 . . . . . . .
. . . . . . .
. . . . .
Vv14g0201 . . . . . . .
. . . . . . .
. . . . .
Vv14g0202 . . . . . . .
. . . . . . .
. . . . .
Vv14g0203 . Gorai.013G126600.1 Gorai.007G301500.1
. . . . . . .
. . . . . . .
.
Vv14g0204 . . . . . . .
. . . . . . .
. . . . .

Vv14g0205 Tc04_g000790 . . . . .
. . . . . . . .
. . . . . .
Vv14g0206 . Gorai.013G126400.1 Gorai.007G301200.1
. . . . . . . .
. . . . . . . .
.
Vv14g0207 . . . . . . .
. . . . . . . .
. . . . .
Vv14g0208 . . . Gorai.007G127300.1 .
. . . . . . . .
. . . . . . .
Vv14g0209 . . . . . .
Vv05g0031 . . . . Gorai.011G192300.1
. . . . . . . .
Vv14g0210 . . . . . . .
. . . . . . . .
. . . . .
Vv14g0211 Tc04_g028490 . . . . .
. . . . . . .
Vv07g0432 Tc01_g030870 . . . . .
Vv14g0212 . . . . . . .
. . . . . . Vv07g0431
Tc01_g030860 . . . . .
Vv14g0213 . . . . . . .
. . . . . . . .
. . . . .
Vv14g0214 . . . . . . .
. . . . . . . .
. . . . .
Vv14g0215 Tc05_g023060 . . . . .
. . . . . . . .
. . . . . .
Vv14g0216 . . . . . . .
. . . . . . . .
. . . . .
Vv14g0217 Tc05_g023040 . . . . .
. . . . . . . .
. . . . . .
Vv14g0218 . . . . . . .
. . . . . . . .
. . . . .
Vv14g0219 Tc04_g028320 . Gorai.007G300700.1
Gorai.007G128000.1 . . Vv05g0010
Tc04_g028320 . . . . . Vv07g0413
Tc01_g030610 Gorai.007G128000.1 . .
Gorai.004G090300.1 .
Vv14g0220 . . . . . . .
. . . . . . . .

```
        .       .       .       .       .
Vv14g0221       .       .       .       .       .       .       .
        .       .       .       .       .       .       .       .
        .       .       .       .       .
Vv14g0222       Tc05_g005630    .       .       .       .       .
        .       .       .       .       .       .       .       .
        .       .       .       .       .       .
Vv14g0223       .       .       .       .       .       .       .
        .       .       .       .       .       .       .       .
        .       .       .       .       .
Vv14g0224       Tc04_g000930    .       .       .       .
Gorai.012G061700.1      .       .       .       .       .       .
        .       .       .       .       .       .       .       .
Vv14g0225       Tc05_g005300    .       .       .       .       .
        .       .       .       .       .       .       .       .
        .       .       .       .       .       .
Vv14g0226       .       .       .       .       .       .       .
        .       .       .       .       .       .       .       .
        .       .       .       .       .
Vv14g0227       .       .       .       .       .       .       .
        .       .       .       .       .       .       .       .
        .       .       .       .       .
Vv14g0228       .       .       .       .       .       .       .
        .       .       .       .       .       .       .       .
        .       .       .       .       .
Vv14g0229       .       .       .       .       .       .       .
        .       .       .       .       .       .       .       .
        .       .       .       .       .
Vv14g0230       .       .       Gorai.007G300000.1      .       .
        .       .       .       .       .       .       .       .
        .       .       .       .       .       .       .
Vv14g0231       .       .       .       .       .       .       .
        .       .       .       .       .       .       .       .
        .       .       .       .       .
Vv14g0232       Tc04_g020360    .       Gorai.007G299900.1
Gorai.007G129000.1      .       .       .       .       .       .
        .       .       .       .       .       .       .       .
        .       .
Vv14g0233       .       .       .       .       .       .       .
        .       .       .       .       .       .       .       .
        .       .       .       .       .
Vv14g0234       .       .       .       .       .       .       .
        .       .       .       .       .       .       .       .
        .       .       .       .       .
Vv14g0235       .       .       .       .       .       .       .
        .       .       .       .       .       .       .       .
        .       .       .       .       .
Vv14g0236       .       .       .       .       .       .       .
        .       .       .       .       .       .       .       .
        .       .       .       .       .
```

Vv14g0237 . . . . . . .
. . . . . . . .
. . . . .
Vv14g0238 . . . . . . .
. . . . . . . .
. . . . .
Vv14g0239 . . . . . . .
. . . . . . . .
. . . . .
Vv14g0240 . . . . . . .
. . . . . . . .
. . . . .
Vv14g0241 . . . . . . .
. . . . . . Vv07g0377
Tc01_g030370 . Gorai.008G146200.1 .
Gorai.004G052500.1 .
Vv14g0242 . . . . . . .
. . . . . . . .
. . . . .
Vv14g0243 Tc04_g003320 . . . . .
. . . . . . . .
. . . . . .
Vv14g0244 Tc04_g028210 Gorai.013G126000.1 . .
. . . . . . . .
. . . . . . . .
Vv14g0245 . . . . . . .
. . . . . . . .
. . . . .
Vv14g0246 . . . Gorai.013G205600.1 .
. . . . . . . .
. . . . . . .
Vv14g0247 . . . . . . .
. . . . . . . .
. . . . .
Vv14g0248 . . . . . .
Vv05g0006 . . . . . . .
. . . . . .
Vv14g0249 . . . . . . .
. . . . . . . .
. . . . .
Vv14g0250 . . . . . . .
. . . . . . . .
. . . . .
Vv14g0251 . . . . . . .
. . . . . . . .
. . . . .
Vv14g0252 . . Gorai.007G299500.1 . .
. . . . . . . .
. . . . . . .
Vv14g0253 . . . . . . .

```
        .       .       .       .       .       .       .       .
        .       .       .       .       .
Vv14g0254       .       Gorai.013G125400.1      Gorai.007G299400.1
        .       .       .       .       .       .       .       .
        .       .       .       .       .       .       .       .
        .
Vv14g0255       Tc04_g017400    Gorai.013G125300.1      .       .
        Gorai.011G134000.1      .       .       .       .       .
        .       .       .       .       .       .       .       .
        .       .
Vv14g0256       Tc04_g001160    .       Gorai.007G299200.1      .
        Gorai.006G031200.1      Gorai.012G063000.1      .       .
        .       .       .       .       .       .       .       .
        .       .       .       .
Vv14g0257       Tc04_g017700    .       Gorai.007G299100.1      .
        Gorai.011G136900.1      .       .       .       .       .
        .       .       .       .       .       .       .       .
        .       .
Vv14g0258       .       .       .       .       .       .       .
        .       .       .       .       .       .       .       .
        .       .       .       .       .
Vv14g0259       Tc05_g028760    .       Gorai.007G299000.1      .
        .       .       .       .       .       .       .       .
        .       .       .       .       .       .       .       .
Vv14g0260       .       .       .       .       .       .       .
        .       .       .       .       .       .       .       .
        .       .       .       .       .
Vv14g0261       Tc05_g028810    Gorai.013G125200.1      .
Gorai.007G129500.1      .       .       Vv07g0345
Tc01_g030060    Gorai.007G129500.1      .       .       .       .
        .       .       .       .       .       .       .
Vv14g0262       .       .       .       .       .       .       .
        .       .       .       .       .       .       .       .
        .       .       .       .       .
Vv14g0263       Tc04_g027960    .       Gorai.007G298900.1      .
        .       .       .       .       .       .       .       .
        .       .       .       .       .       .       .       .
Vv14g0264       Tc04_g027950    .       .       .       .       .
        .       .       .       .       .       .       .       .
        .       .       .       .       .       .
Vv14g0265       .       Gorai.013G125100.1      Gorai.007G298800.1
        Gorai.013G205300.1      .       .       .       .       .
        .       .       .       .       .       .       .       .
        .       .       .
Vv14g0266       .       .       .       .       .       .       .
        .       .       .       .       .       .       .       .
        .       .       .       .       .
Vv14g0267       .       .       Gorai.007G298700.1      .       .
        .       .       .       .       .       .       .       .
        .       .       .       .       .       .       .
```

Vv14g0268 . . Gorai.007G298600.1 . .
. . . . . . . .
. . . . . . .
Vv14g0269 . . . . . . .
. . . . . . . .
. . . . .
Vv14g0270 Tc04_g003160 . . . . .
. . . . . . . .
. . . . . .
Vv14g0271 . . . . . . .
. . . . . . . .
. . . . .
Vv14g0272 . . . . . . .
. . . . . . . .
. . . . .
Vv14g0273 . . . . . . .
. . . . . . . .
. . . . .
Vv14g0274 . . . . . . .
. . . . . . . .
. . . . .
Vv14g0275 . . . . . . .
. . . . . . . .
. . . . .
Vv14g0276 . . . . . . .
. . . . . . . .
. . . . .
Vv14g0277 . . . . . . .
. . . . . . . .
. . . . .
Vv14g0278 . . . . . . .
. . . . . . . .
. . . . .
Vv14g0279 . . . . . . .
. . . . . . . .
. . . . .
Vv14g0280 . . . . . . .
. . . . . . . .
. . . . .
Vv14g0281 . . . . . . .
. . . . . . . .
. . . . .
Vv14g0282 . . . . . . .
. . . . . . . .
. . . . .
Vv14g0283 . . . . . . .
. . . . . . . .
. . . . .
Vv14g0284 Tc04_g003140 . . . . .
. . . . . . . .

.	.	.	.	.	.
Vv14g0285	.	.	.	.	.	.	.
.	.	.	.	.	.	.	.
.	.	.	.	.
Vv14g0286	Tc04_g027920	Gorai.013G124900.1	.	.
.	.	Vv07g0329	Tc01_g029930	Gorai.
007G130000.1	.	.	Gorai.004G053000.1	.	.
.	.	.	.	.	.
Vv14g0287	.	.	.	.	.	.	.
.	.	.	.	.	.	.	.
.	.	.	.	.
Vv14g0288	Tc04_g027900	.	.	Gorai.007G129900.1
Gorai.011G189100.1	.	.	.	.	.
.	.	.	.	.	.	.	.
.	.
Vv14g0289	.	.	.	.	.	.	.
.	.	.	.	.	.	.	.
.	.	.	.	.
Vv14g0290	.	.	Gorai.007G298500.1	.	.
.	.	.	.	.	.	.	.
.	.	.	.	.	.	.
Vv14g0291	.	.	.	.	.	.	.
.	.	.	.	.	.	.	.
.	.	.	.	.
Vv14g0292	.	.	.	.	.	.	.
.	.	.	.	.	.	.	.
.	.	.	.	.
Vv14g0293	.	.	.	.	.	.	.
.	.	.	.	.	.	.	.
.	.	.	.	.
Vv14g0294	.	.	.	.	.	.	.
.	.	.	.	.	.	.	.
.	.	.	.	.
Vv14g0295	.	.	.	.	.	.	.
.	.	.	.	.	.	.	.
.	.	.	.	.
Vv14g0296	.	.	.	.	.	.	.
.	.	.	.	.	.	.	.
.	.	.	.	.
Vv14g0297	.	.	.	.	.	.	.
.	.	.	.	.	.	.	.
.	.	.	.	.
Vv14g0298	.	.	.	.	.	.	.
.	.	.	.	.	.	.	.
.	.	.	.	.
Vv14g0299	.	.	.	.	.	.	.
.	.	.	.	.	.	.	.
.	.	.	.	.
Vv14g0300	.	.	.	.	.	.	.
.	.	.	.	.	.	.	.

```
        .        .       .        .       .
Vv14g0301        .       .        .       .        .       .        .
        .        .       .        .       .        .       .        .
        .        .       .        .       .
Vv14g0302        .       .        .       .        .       .        .
        .        .       .        .       .        .       .        .
        .        .       .        .       .
Vv14g0303        .       .        .       .        .       .        .
        .        .       .        .       .        .       .        .
        .        .       .        .       .
Vv14g0304        .       .        .       .        .       .        .
        .        .       .        .       .        .       .        .
        .        .       .        .       .
Vv14g0305        .       .        .       .        .       .        .
        .        .       .        .       .        .       .        .
        .        .       .        .       .
Vv14g0306        .       .        .       .        .       .        .
        .        .       .        .       .        .       .        .
        .        .       .        .       .
Vv14g0307        .       .        .       .        .       .        .
        .        .       .        .       .        .       .        .
        .        .       .        .       .
Vv14g0308        .       .        .       .        .       .        .
        .        .       .        .       .        .       .        .
        .        .       .        .       .
Vv14g0309        .       .        .       .        .       .        .
        .        .       .        .       .        .       .        .
        .        .       .        .       .
Vv14g0310        .       .        .       .        .       .        .
        .        .       .        .       .        .       .        .
        .        .       .        .       .
Vv14g0311        .       .        .       .        .       .        .
        .        .       .        .       .        .       .        .
        .        .       .        .       .
Vv14g0312        .       .        .       .        .       .        .
        .        .       .        .       .        .       .        .
        .        .       .        .       .
Vv14g0313        .       .        .       .        .       .        .
        .        .       .        .       .        .       .        .
        .        .       .        .       .
Vv14g0314        .       .        .       Gorai.007G130300.1        .
        .        Vv07g0325        .       Gorai.007G130300.1        .
        .        Gorai.004G053100.1       .        .       .        .
        .        .       .        .
Vv14g0315        Tc04_g027850     .       .        .       .        .
        .        .       .        .       .        .       .        .
        .        .       .        .       .        .
Vv14g0316        Tc04_g027840     .       Gorai.007G298100.1        .
        Gorai.007G342100.1        .       .        .       .        .
        .        .       .        .       .        .       .        .
```

. .
Vv14g0317 . Gorai.013G124600.1 . Gorai.
013G202200.1 Gorai.007G342200.1 . . . .
. . . . . . . .
. . .
Vv14g0318 . . . . . . .
. . . . . . . .
. . . . .
Vv14g0319 . . Gorai.007G297700.1 . .
. . . . . . . .
. . . . . . .
Vv14g0320 . . . . . . .
. . . . . . . .
. . . . .
Vv14g0321 . . . . . . .
. . . . . . . .
. . . . .
Vv14g0322 Tc04_g023850 . . . . .
. . . . . . . .
. . . . . .
Vv14g0323 . . . . . .
Vv05g0519 Tc04_g023490 Gorai.006G238200.1 Gorai.
005G041900.1 . . . . . . .
. . .
Vv14g0324 . Gorai.013G124500.1 . . .
. . . . . . . .
. . . . . . .
Vv14g0325 Tc04_g023490 . . . . .
. . . . . . . .
. . . . . .
Vv14g0326 . . . . . . .
. . . . . . . .
. . . . .
Vv14g0327 . Gorai.013G124300.1 . . .
. . . . . . . .
. . . . . . .
Vv14g0328 . . Gorai.007G297300.1 .
Gorai.007G342300.1 . . . . . .
. . . . . . . .
.
Vv14g0329 . . . . . . .
. . . . . . . .
. . . . .
Vv14g0330 . . . . . . .
. . . . . . . .
. . . . .
Vv14g0331 Tc04_g023400 . . . Gorai.
007G342400.1 Gorai.002G023900.1 Vv05g0508
Tc04_g023400 . . Gorai.006G052200.1 .
Gorai.002G023900.1 . . . . . .

| | | | | | | | |
|---|---|---|---|---|---|---|---|
| . | | | | | | | |
| Vv14g0332 | . | . | . | . | . | . | . |
| . | . | . | . | . | . | . | . |
| . | . | . | . | . | | | |
| Vv14g0333 | . | Gorai.013G124200.1 | | | Gorai.007G297200.1 | | |
| . | . | . | . | . | . | . | . |
| . | . | . | . | . | . | . | . |
| . | | | | | | | |
| Vv14g0334 | Tc04_g023370 | | . | . | . | . | . |
| . | . | . | . | . | . | . | . |
| . | . | . | . | . | . | | |
| Vv14g0335 | . | . | . | . | . | . | . |
| . | . | . | . | . | . | . | . |
| . | . | . | . | . | | | |
| Vv14g0336 | . | . | . | . | . | . | . |
| . | . | . | . | . | . | . | . |
| . | . | . | . | . | | | |
| Vv14g0337 | . | . | . | . | . | . | . |
| . | . | . | . | . | . | . | . |
| . | . | . | . | . | | | |
| Vv14g0338 | . | . | . | . | . | . | . |
| . | . | . | . | . | . | . | . |
| . | . | . | . | . | | | |
| Vv14g0339 | . | . | . | . | . | . | . |
| . | . | . | . | . | . | . | . |
| . | . | . | . | . | | | |
| Vv14g0340 | . | . | . | . | . | . | . |
| . | . | . | . | . | . | . | . |
| . | . | . | . | . | | | |
| Vv14g0341 | . | . | . | . | . | . | . |
| . | . | . | . | . | . | . | . |
| . | . | . | . | . | | | |
| Vv14g0342 | . | . | . | . | . | . | . |
| . | . | . | . | . | . | . | . |
| . | . | . | . | . | | | |
| Vv14g0343 | . | . | . | . | . | . | . |
| . | . | . | . | . | . | . | . |
| . | . | . | . | . | | | |
| Vv14g0344 | . | . | . | . | . | . | . |
| . | . | . | . | . | . | . | . |
| . | . | . | . | . | | | |
| Vv14g0345 | . | . | . | . | . | . | . |
| . | . | . | . | . | . | . | . |
| . | . | . | . | . | | | |
| Vv14g0346 | . | . | . | . | . | . | . |
| . | . | . | . | . | . | . | . |
| . | . | . | . | . | | | |
| Vv14g0347 | . | . | . | . | . | . | . |
| . | . | . | . | . | . | . | . |
| . | . | . | . | . | | | |

Vv14g0348 . . . . . . .
. . . . . . . .
. . . . .
Vv14g0349 Tc04_g021990 . . . . .
. . . . . . . .
. . . . . .
Vv14g0350 . . . . . . .
. . . . . . . .
. . . . .
Vv14g0351 . . . . . . .
. . . . . . . .
. . . . .
Vv14g0352 . . . . . . .
. . . . . . . .
. . . . .
Vv14g0353 . . . . . . .
. . . . . . . .
. . . . .
Vv14g0354 Tc04_g021800 . . . . .
. . . . . . . .
. . . . . .
Vv14g0355 . . Gorai.007G297100.1 . .
. . . . . . . .
. . . . . . .
Vv14g0356 . . . . . . .
. . . . . . . .
. . . . .
Vv14g0357 . . . . . . .
. . . . . . . .
. . . . .
Vv14g0358 . . Gorai.007G297000.1 . .
. . . . . . . .
. . . . . . .
Vv14g0359 Tc04_g025820 . . . . .
. . . . . . . .
. . . . . .
Vv14g0360 . . Gorai.007G296800.1 . .
. . . . . . . .
. . . . . . .
Vv14g0361 Tc04_g023240 . . . . .
. . . . . . . .
. . . . . .
Vv14g0362 Tc05_g031480 Gorai.013G123900.1 Gorai.
007G296600.1 . . . . . . .
. . . . . . . .
. .
Vv14g0363 . . . . . . .
. . . . . . . .
. . . . .
Vv14g0364 . . . . Gorai.007G342600.1

. . . . . . . .
Vv07g0218 . . . . .
Gorai.004G241600.1
Vv14g0365 Tc04_g023230 . Gorai.007G296500.1 .
. . Vv05g0490 Tc04_g023230 .
Gorai.005G041300.1 . . . . . .
. . . .
Vv14g0366 . . Gorai.007G296400.1 . .
. . . . . . . .
. . . . . . .
Vv14g0367 Tc04_g023220 . . . . .
. . . . . . . .
. . . . . .
Vv14g0368 . . Gorai.007G296200.1 . .
. . . . . . . .
. . . . . . .
Vv14g0369 . . . . . . .
. . . . . . . .
. . . . .
Vv14g0370 Tc04_g023210 Gorai.013G123800.1 . .
Gorai.006G052800.1 . Vv05g0489
Tc04_g023210 . Gorai.005G041200.1 . . .
. . . . . . .
Vv14g0371 Tc04_g023170 . Gorai.007G296100.1 .
. . . . . . . .
. . . . . . . .
Vv14g0372 . . Gorai.007G296000.1 .
Gorai.007G342800.1 . . . . . .
. . . . . . . .
.
Vv14g0373 . . Gorai.007G295900.1 . .
. . . . . . . .
. . . . . . .
Vv14g0374 . . . . . . .
. . . . . . . .
. . . . .
Vv14g0375 . . . . . . .
. . . . . . . .
. . . . .
Vv14g0376 . . . . Gorai.007G343000.1
. . . . . . . .
. . . . . . .
Vv14g0377 . . . . . . .
. . . . . . . .
. . . . .
Vv14g0378 . . . . . . .
. . . . . . . .
. . . . .
Vv14g0379 . . . . . . .
. . . . . . . .

.		.		.		.		.
Vv14g0380		.		.		.		.		.		.		.
.		.		.		.		.		.		.		.
.		.		.		.		.
Vv14g0381		.		.		Gorai.007G295500.1		.		.
.		.		.		.		.		.		.		.
.		.		.		.		.		.		.
Vv14g0382		.		.		.		.		.		.		.
.		.		.		.		.		.		.		.
.		.		.		.		.
Vv14g0383		.		.		.		.		.		.		.
.		.		.		.		.		.		.		.
.		.		.		.		.
Vv14g0384		.		.		.		.		.		.		.
.		.		.		.		.		.		.		.
.		.		.		.		.
Vv14g0385		.		.		.		.		.		.
Vv05g0470		.		.		.		.		.		.		.
.		.		.		.		.		.
Vv14g0386		Tc04_g022980		.		.		.		.		.
Vv05g0468		Tc04_g022990		.		.		.		.
.		.		.		.		.		.		.		.
Vv14g0387		Tc04_g022960		.		Gorai.007G295400.1		.
.		Gorai.004G215700.1		Vv05g0467
Tc04_g022970		.		.		.		.		.		.		.
.		.		.		.		.
Vv14g0388		Tc04_g022920		.		Gorai.007G295300.1		.
.		.		Vv05g0464		Tc04_g022920		.		.
Gorai.006G054000.1		.		.		.		.		.
.		.		.		.
Vv14g0389		Tc04_g025620		.		.		.		Gorai.
006G053800.1		.		Vv05g0463		Tc04_g022910		.		.
Gorai.006G053800.1		.		.		Vv07g0236
Tc01_g028920		Gorai.007G133700.1		.		.		Gorai.
004G054800.1		.
Vv14g0390		.		.		.		.		.		.		.
.		.		.		.		.		.		.		.
.		.		.		.		.
Vv14g0391		Tc04_g027640		.		.		.		.		.
.		.		.		.		.		.		.		.
.		.		.		.		.		.
Vv14g0392		.		.		.		Gorai.013G204500.1		.
.		.		.		.		.		.		.		.
.		.		.		.		.		.		.
Vv14g0393		Tc04_g016350		.		Gorai.007G294900.1
Gorai.013G204400.1		.		.		.		.		.		.
.		.		.		.		.		.		.		.
.		.
Vv14g0394		.		.		Gorai.007G294800.1		Gorai.
013G204300.1		Gorai.006G054300.1		.		.		.		.

. . . . . . . .
. . .
Vv14g0395 . . . . . . .
. . . . . . . .
. . . . .
Vv14g0396 . Gorai.013G123700.1 . . .
. . . . . . . .
. . . . . . .
Vv14g0397 . . . . . . .
. . . . . . . .
. . . . .
Vv14g0398 . . . . . .
Vv13g1059 . . . . . . .
. . . . . .
Vv14g0399 . . . . . . .
. . . . . . . .
. . . . .
Vv14g0400 Tc05_g031530 . . . . .
. . . . . . . .
. . . . . .
Vv14g0401 . . Gorai.007G294200.1 . .
. . . . . . . .
. . . . . . .
Vv14g0402 . . Gorai.007G294100.1 Gorai.
013G204100.1 . . . . . . .
. . . . . . . .
.
Vv14g0403 . . . . . . .
. . . . . . . .
. . . . .
Vv14g0404 Tc04_g027370 . Gorai.007G294000.1 .
. . . . . . . .
. . . . . . . .
Vv14g0405 . . . . . . .
. . . . . . . .
. . . . .
Vv14g0406 . . . . . . .
. . . . . . . .
. . . . .
Vv14g0407 . . . . . . .
. . . . . . . .
. . . . .
Vv14g0408 . . . . . . .
. . . . . . . .
. . . . .
Vv14g0409 . . . . . . .
. . . . . . . .
. . . . .
Vv14g0410 . . . . . . .
. . . . . . . .

. . . . .
Vv14g0411 . . . . . . .
. . . . . . . .
. . . . .
Vv14g0412 Tc04_g016370 . . . Gorai.
011G127800.1 . . . . . . .
. . . . . . . .
Vv14g0413 . . . . . . .
. . . . . . . .
. . . . .
Vv14g0414 . . . . . . .
. . . . . . . .
. . . . .
Vv14g0415 . Gorai.013G123500.1 Gorai.007G293800.1
. . . . . . . .
. . . . . . . .
.
Vv14g0416 . Gorai.013G123400.1 . Gorai.
007G133300.1 . Gorai.004G215100.1 . . .
. . . . Vv07g0246
Tc01_g029050 Gorai.007G133300.1 . . .
Gorai.004G215100.1
Vv14g0417 . . . . . . .
. . . . . . . .
. . . . .
Vv14g0418 Tc04_g023450 . . . . .
Vv13g1080 . . . . . .
. . . . . . .
Vv14g0419 . . . . . .
Vv13g1081 . . . . . . .
. . . . . .
Vv14g0420 . Gorai.013G120800.1 Gorai.007G292500.1
. . . . . . . .
. . . . . . . .
.
Vv14g0421 . . Gorai.007G292400.1 . .
. Vv13g1082 . . . . .
. . . . . . . .
Vv14g0422 . . . . . . .
. . . . . . . .
. . . . .
Vv14g0423 Tc04_g023430 . . . . .
. . . . . . . .
. . . . . .
Vv14g0424 Tc04_g023420 . . . . .
. . . . . . . .
. . . . . .
Vv14g0425 . . . . . . .
. . . . . . . .
. . . . .

Vv14g0426 . . . . . . .
. . . . . . . .
. . . . .
Vv14g0427 . . . . . . .
. . . . . . . .
. . . . .
Vv14g0428 . . . . . . .
. . . . . . . .
. . . . .
Vv14g0429 . . . . . . .
. . . . . . . .
. . . . .
Vv14g0430 . . . . . . .
. . . . . . . .
. . . . .
Vv14g0431 Tc04_g027160 . Gorai.007G292300.1 .
Gorai.011G183500.1 . Vv13g1084 . .
. . . . . . . .
. . .
Vv14g0432 . . . . . . .
. . . . . . . .
. . . . .
Vv14g0433 . . . . . .
Vv13g1089 . . . . . . .
. . . . . .
Vv14g0434 . . Gorai.007G292200.1 Gorai.
013G203800.1 . . Vv13g1090 . . .
. . . . . . . .
. .
Vv14g0435 . . . . . . .
. . . . . . . .
. . . . .
Vv14g0436 . . . . . . .
. . . . . . . .
. . . . .
Vv14g0437 . . . . . . .
. . . . . . . .
. . . . .
Vv14g0438 Tc05_g031690 . . . . .
. . . . . . . .
. . . . . .
Vv14g0439 . . . . . . .
. . . . . . . .
. . . . .
Vv14g0440 . . Gorai.007G291800.1 Gorai.
013G203700.1 . . Vv07g0258 Tc01_g029170 .
. . . . . . . .
. . .
Vv14g0441 Tc04_g027120 . Gorai.007G291700.1
Gorai.007G132900.1 . . Vv07g0259

Tc01_g029180 Gorai.007G132900.1 . . . .
. . . . . . .
Vv14g0442 Tc04_g027130 . Gorai.007G291600.1 .
Gorai.011G183200.1 . . . . .
. . . . . . . .
. .
Vv14g0443 . . Gorai.007G291400.1 . .
. . . . . . . .
. . . . . . .
Vv14g0444 . . . Gorai.007G132800.1 .
Gorai.004G214300.1 Vv07g0264 Tc01_g029220
Gorai.007G132800.1 . . . Gorai.004G214300.1
. . . . . . .
Vv14g0445 . . . . . . .
. . . . . . . .
. . . . .
Vv14g0446 . . Gorai.007G291200.1 . .
Gorai.004G214100.1 Vv07g0267 Tc01_g029250 .
. . . Gorai.004G239900.1 . .
. . . . .
Vv14g0447 . . Gorai.007G291100.1 . .
. . . . . . . .
. . . . . . .
Vv14g0448 . . Gorai.007G288200.1 . .
. . . . . . . .
. . . . . . .
Vv14g0449 . . . . . Gorai.
004G211800.1 . . . . . . .
. . . . . . .
Vv14g0450 . . . . . . .
. . . . . . . .
. . . . .
Vv14g0451 . . . Gorai.013G203400.1 .
. . . . . . . .
. . . . . . .
Vv14g0452 . . . . . . .
. . . . . . . .
. . . . .
Vv14g0453 . . . Gorai.013G200700.1 .
. . . . . . . .
. . . . . . .
Vv14g0454 . . . . . . .
. . . . . . . .
. . . . .
Vv14g0455 . . . . . . .
. . . . . . . .
. . . . .
Vv14g0456 . . . . . . .
. . . . . . . .
. . . . .

Vv14g0457 . . . . . . .
. . . . . . . .
. . . . .
Vv14g0458 . . . . . . .
. . . . . . . .
. . . . .
Vv14g0459 . . . . . . .
. . . . . . . .
. . . . .
Vv14g0460 . . . . . . .
. . . . . . . .
. . . . .
Vv14g0461 . . . . . . .
. . . . . . . .
. . . . .
Vv14g0462 . Gorai.013G115800.1 . . .
. . . . . . . .
. . . . . . .
Vv14g0463 . . . . . . .
. . . . . . . .
. . . . .
Vv14g0464 . . . . . . .
. . . . . . . .
. . . . .
Vv14g0465 . . . . . . .
. . . . . . . .
. . . . .
Vv14g0466 . . . . . . .
. . . . . . . .
. . . . .
Vv14g0467 . . . . . . .
. . . . . . . .
. . . . .
Vv14g0468 . . . . . . .
. . . . . . . .
. . . . .
Vv14g0469 . Gorai.013G115900.1 . . .
. . . . . . . .
. . . . . . .
Vv14g0470 . . . . . . .
. . . . . . . .
. . . . .
Vv14g0471 . Gorai.013G116000.1 . . .
. Vv07g0285 . . . . .
. . . . . . . .
Vv14g0472 Tc05_g030310 Gorai.013G116100.1 . .
. . . . . . . .
. . . . . . . .
Vv14g0473 Tc04_g027280 . . . . .
. . . . . . . .

. . . . . .
Vv14g0474 . . . . . . .
. . . . . . . .
. . . . .
Vv14g0475 . . . . . . .
. . . . . . . .
. . . . .
Vv14g0476 . . . . . . .
. . . . . . . .
. . . . .
Vv14g0477 Tc04_g027300 Gorai.013G116200.1 . .
. . . . . . . .
. . . . . . . .
Vv14g0478 . . . Gorai.007G132000.1 .
. . . . . . . .
. . . . . . .
Vv14g0479 . . . . . . .
. . . . . . . .
. . . . .
Vv14g0480 . Gorai.013G116700.1 . . .
. . . . . . . .
. . . . . . .
Vv14g0481 . . . . . . .
. . . . . . . .
. . . . .
Vv14g0482 . . . . . .
Vv07g0294 Tc01_g029560 . . . . .
. . . . . . .
Vv14g0483 . . . . . . .
. . . . . . . .
. . . . .
Vv14g0484 . . Gorai.007G264500.1 . .
. . . . . . . .
. . . . . . .
Vv14g0485 . . . . . . .
. . . . . . . .
. . . . .
Vv14g0486 . . Gorai.007G264400.1 . .
. . . . . . . .
. . . . . . .
Vv14g0487 . . . . . . .
. . . . . . . .
. . . . .
Vv14g0488 . . . . . . .
. . . . . . . .
. . . . .
Vv14g0489 . . . . . . .
. . . . . . . .
. . . . .
Vv14g0490 Tc04_g027340 . Gorai.007G264300.1 .

. . . . . . . .

. . . . . . . .

Vv14g0491 . . Gorai.007G264200.1 . .

. . . . . . . .

. . . . . . .

Vv14g0492 . . . . . . .

. . . . . . . .

. . . . .

Vv14g0493 . . . . . . .

. . . . . . . .

. . . . .

Vv14g0494 . . . . . . .

. . . . . . . .

. . . . .

Vv14g0495 . . Gorai.007G263800.1 . .

. . . . . . . .

. . . . . . .

Vv14g0496 . . . . . . .

. . . . . . . .

. . . . .

Vv14g0497 . . . . . . .

. . . . . . . .

. . . . .

Vv14g0498 . . . . . . .

. . . . . . . .

. . . . .

Vv14g0499 . . . . . . .

. . . . . . . .

. . . . .

Vv14g0500 . Gorai.013G116800.1 Gorai.007G261000.1

. . . . . . . .

. . . . . . . .

.

Vv14g0501 Tc05_g030670 . . . . .

. . . . . . . .

. . . . . .

Vv14g0502 Tc05_g030680 . . . . .

. . . . . . . .

. . . . . .

Vv14g0503 . . . . . . .

. . . . . . . .

. . . . .

Vv14g0504 . . . . . . .

. . . . . . . .

. . . . .

Vv14g0505 . . . . . . .

. . . . . . . .

. . . . .

Vv14g0506 Tc05_g030690 . . . . .

. . . . . . . .

| | | | | | | | |
|---|---|---|---|---|---|---|---|
| . | . | . | . | . | . | | |
| Vv14g0507 | . | . | . | . | . | . | . |
| . | . | . | . | . | . | . | . |
| . | . | . | . | . | | | |
| Vv14g0508 | . | . | . | . | . | . | . |
| . | . | . | . | . | . | . | . |
| . | . | . | . | . | | | |
| Vv14g0509 | . | . | . | . | . | . | . |
| . | . | . | . | . | . | . | . |
| . | . | . | . | . | | | |
| Vv14g0510 | . | . | . | . | . | . | . |
| . | . | . | . | . | . | . | . |
| . | . | . | . | . | | | |
| Vv14g0511 | . | . | . | . | . | . | . |
| . | . | . | . | . | . | . | . |
| . | . | . | . | . | | | |
| Vv14g0512 | . | . | . | . | . | . | . |
| . | . | . | . | . | . | . | . |
| . | . | . | . | . | | | |
| Vv14g0513 | Tc05_g030700 | | . | . | . | . | . |
| . | . | . | . | . | . | . | . |
| . | . | . | . | . | . | | |
| Vv14g0514 | . | . | . | . | . | . | . |
| . | . | . | . | . | . | . | . |
| . | . | . | . | . | | | |
| Vv14g0515 | . | . | . | . | . | . | . |
| . | . | . | . | . | . | . | . |
| . | . | . | . | . | | | |
| Vv14g0516 | . | . | . | . | . | . | . |
| . | . | . | . | . | . | . | . |
| . | . | . | . | . | | | |
| Vv14g0517 | . | . | . | . | . | . | . |
| . | . | . | . | . | . | . | . |
| . | . | . | . | . | | | |
| Vv14g0518 | . | . | . | . | . | . | . |
| . | . | . | . | . | . | . | . |
| . | . | . | . | . | | | |
| Vv14g0519 | . | . | . | . | . | . | . |
| . | . | . | . | . | . | . | . |
| . | . | . | . | . | | | |
| Vv14g0520 | . | . | . | . | . | . | . |
| . | . | . | . | . | . | . | . |
| . | . | . | . | . | | | |
| Vv14g0521 | . | . | . | . | . | . | . |
| . | . | . | . | . | . | . | . |
| . | . | . | . | . | | | |
| Vv14g0522 | . | . | . | . | . | . | . |
| . | . | . | . | . | . | . | . |
| . | . | . | . | . | | | |
| Vv14g0523 | Tc05_g030740 | | . | . | . | . | . |

. . . . . . . .
. . . . . .
Vv14g0524 . . . . . . .
. . . . . . . .
. . . . .
Vv14g0525 . . . . . . .
. . . . . . . .
. . . . .
Vv14g0526 . . . . . . .
. . . . . . . .
. . . . .
Vv14g0527 Tc04_g004860 . . Gorai.007G131300.1
. . . . . . . .
. . . . . . . .
Vv14g0528 . Gorai.013G122800.1 . Gorai.
013G202700.1 . . . . . . .
. . . . . . . .
.
Vv14g0529 Tc04_g027460 . . . Gorai.
011G185800.1 . . . . . . .
. . . . . . . .
Vv14g0530 . . Gorai.007G287100.1 Gorai.
007G129000.1 . . . . . . .
. . . . . . . .
.
Vv14g0531 Tc04_g023370 . . . . .
. . . . . . . .
. . . . . .
Vv14g0532 Tc04_g027500 . Gorai.007G287000.1
Gorai.013G202400.1 . . . . . .
. . . . . . . .
. .
Vv14g0533 . . . . . . .
. . . . . . . .
. . . . .
Vv14g0534 . . . . . . .
. . . . . . . .
. . . . .
Vv14g0535 Tc04_g004760 . . . . .
. . . . . . . .
. . . . . .
Vv14g0536 . . . . . . .
. . . . . . . .
. . . . .
Vv14g0537 Tc04_g021290 Gorai.013G122600.1 Gorai.
007G286900.1 Gorai.013G202200.1 Gorai.011G159400.1 .
. . . . . . . .
. . . . . .
Vv14g0538 . . . . . . .
. . . . . . . .

```
             .        .        .        .        .
Vv14g0539           Tc05_g030820     .        .        .        .        .
             .        .        .        .        .        .        .        .
             .        .        .        .        .        .
Vv14g0540    .        .        Gorai.007G286600.1         .        .
             .        .        .        .        .        .        .        .
             .        .        .        .        .        .        .
Vv14g0541    .        .        .        .        .        .        .
             .        .        .        .        .        .        .        .
             .        .        .        .        .
Vv14g0542    .        .        .        .        .        .        .
             .        .        .        .        .        .        .        .
             .        .        .        .        .
Vv14g0543    .        Gorai.013G122400.1       Gorai.007G286500.1
             .        .        .        .        .        .        .        .
             .        .        .        .        .        .        .        .
             .
Vv14g0544    .        .        .        .        .        .        .
             .        .        .        .        .        .        .        .
             .        .        .        .        .
Vv14g0545    .        Gorai.013G122300.1       .        .        .
             .        .        .        .        .        .        .        .
             .        .        .        .        .        .        .
Vv14g0546           Tc04_g021720     .        .        .        .        .
             .        .        .        .        .        .        .        .
             .        .        .        .        .        .
Vv14g0547    .        .        Gorai.007G286100.1         .        .
             .        .        .        .        .        .        .        .
             .        .        .        .        .        .        .
Vv14g0548    .        .        .        .        Gorai.007G344500.1
             .        .        .        .        .        .        .        .
             .        .        .        .        .        .        .
Vv14g0549    .        .        .        .        .        .        .
             .        .        .        .        .        .        .        .
             .        .        .        .        .
Vv14g0550    .        Gorai.013G122200.1       .        .
Gorai.007G344600.1    .        .        .        .        .        .
             .        .        .        .        .        .        .        .
             .
Vv14g0551           Tc04_g023790     .        .        Gorai.013G202100.1
             .        .        .        .        .        .        .        .
             .        .        .        .        .        .        .        .
Vv14g0552    .        .        .        .        .        .        .
             .        .        .        .        .        .        .        .
             .        .        .        .        .
Vv14g0553    .        Gorai.013G122000.1       .        .        .
             .        .        .        .        .        .        .        .
             .        .        .        .        .        .        .
Vv14g0554    .        .        .        Gorai.013G202000.1        .
             .        .        .        .        .        .        .        .
```

. . . . . . .
Vv14g0555 . . Gorai.007G286000.1 . .
. . . . . . . .
. . . . . . .
Vv14g0556 . . . . . . .
. . . . . . . .
. . . . .
Vv14g0557 . . . . . . .
. . . . . . . .
. . . . .
Vv14g0558 . . . . . . .
. . . . . . . .
. . . . .
Vv14g0559 . . . . . . .
. . . . . . . .
. . . . .
Vv14g0560 . . . . . . .
. . . . . . . .
. . . . .
Vv14g0561 . . . . . . .
. . . . . . . .
. . . . .
Vv14g0562 . . . . . . .
. . . . . . . .
. . . . .
Vv14g0563 . . . . . . .
. . . . . . . .
. . . . .
Vv14g0564 . . . . . . .
. . . . . . . .
. . . . .
Vv14g0565 . . . . . . .
. . . . . . . .
. . . . .
Vv14g0566 . . . . . . .
. . . . . . . .
. . . . .
Vv14g0567 . . . . . . .
. . . . . . . .
. . . . .
Vv14g0568 . . . . . . .
. . . . . . . .
. . . . .
Vv14g0569 . . . . . . .
. . . . . . . .
. . . . .
Vv14g0570 . Gorai.013G121900.1 Gorai.007G285900.1
. . . . . . . .
. . . . . . . .
.

Vv14g0571 . . . . . . .
. . . . . . . .
. . . .
Vv14g0572 . Gorai.013G121800.1 Gorai.007G285700.1
. . . . . . . .
. . . . . . . .
.
Vv14g0573 . . Gorai.007G285600.1 . .
. . . . . . . .
. . . . . . .
Vv14g0574 . Gorai.013G121400.1 Gorai.007G285500.1
Gorai.013G201800.1 . . . . .
. . . . . . . .
. . .
Vv14g0575 . . . . . . .
. . . . . . . .
. . . . .
Vv14g0576 . . . . . . .
. . . . . . . .
. . . . .
Vv14g0577 . . Gorai.007G285400.1 .
Gorai.007G344700.1 . . . . . .
. . . . . . . .
.
Vv14g0578 Tc04_g024160 . . . . .
. . . . . . . .
. . . . . .
Vv14g0579 . . . . . . .
. . . . . . . .
. . . . .
Vv14g0580 . . . . . . .
. . . . . . . .
. . . . .
Vv14g0581 . . . . . . .
. . . . . . . .
. . . . .
Vv14g0582 . . . . . . .
. . . . . . . .
. . . . .
Vv14g0583 . . . . . . .
. . . . . . . .
. . . . .
Vv14g0584 . . . . . . .
. . . . . . . .
. . . . .
Vv14g0585 . . Gorai.007G285000.1 . .
. . . . . . . .
. . . . . . .
Vv14g0586 . . Gorai.007G284900.1 Gorai.
013G201600.1 . . . . . . .

. . . . . . . .
.
Vv14g0587 . . . . . . .
. . . . . . . .
. . . . .
Vv14g0588 . . . . . . .
. . . . . . . .
. . . . .
Vv14g0589 Tc05_g008440 . Gorai.007G284600.1
Gorai.007G137800.1 Gorai.007G344900.1 . . .
. . . . . . . .
. . . .
Vv14g0590 . . . . . . .
. . . . . . . .
. . . . .
Vv14g0591 . . Gorai.007G284500.1 . .
. . . . . . . .
. . . . . . .
Vv14g0592 Tc04_g024170 Gorai.013G121300.1 Gorai.
007G284200.1 . . . Vv05g0595
Tc04_g024170 . Gorai.005G044800.1 . . .
. . . . . . .
Vv14g0593 . . . . . . .
. . . . . . . .
. . . . .
Vv14g0594 . . . . . . .
. . . . . . . .
. . . . .
Vv14g0595 Tc04_g024190 . . . Gorai.
011G167500.1 . . . . . . .
. . . . . . . .
Vv14g0596 . . Gorai.007G284100.1 . .
. . . . . . . .
. . . . . . .
Vv14g0597 . . . . . . .
. . . . . . . .
. . . . .
Vv14g0598 . . Gorai.007G284000.1 . .
. . . . . . . .
. . . . . . .
Vv14g0599 Tc05_g008470 . Gorai.007G283800.1 .
. . . . . . . .
. . . . . . . .
Vv14g0600 Tc04_g009660 . . . Gorai.
006G013500.1 . . . . . . .
. . . . . . . .
Vv14g0601 Tc04_g024240 . . Gorai.013G201400.1
Gorai.011G167700.1 . Vv05g0603
Tc04_g024240 Gorai.006G241800.1 . . Gorai.
011G167700.1 . . . . . . .

.
Vv14g0602 . . Gorai.007G283700.1 . .
. . . . . . . .
. . . . . . .
Vv14g0603 . . Gorai.007G283600.1 Gorai.
013G201200.1 . . . . . . .
. . . . . . . .
.
Vv14g0604 . . Gorai.007G283500.1 . .
. . . . . . . .
. . . . . . .
Vv14g0605 . . . . . . .
. . . . . . . .
. . . . .
Vv14g0606 . . . . . . .
. . . . . . . .
. . . . .
Vv14g0607 . . . . . . .
. . . . . . . .
. . . . .
Vv14g0608 Tc04_g020550 . . . . .
. . . . . . . .
. . . . . .
Vv14g0609 Tc04_g024380 . Gorai.007G283400.1 .
. . . . . . . .
. Vv01g0679 . . Gorai.005G186700.1
. . .
Vv14g0610 . . . . . . .
. . . . . . . .
. . . . .
Vv14g0611 Tc04_g024390 . Gorai.007G283200.1 .
. . . . . . . .
. Vv01g0678 . . Gorai.005G186800.1
. . .
Vv14g0612 . . Gorai.007G283000.1 . .
. . . . . . . .
Vv01g0677 . . Gorai.005G186900.1 .
. .
Vv14g0613 . . . . . . .
. . . . . . . .
. . . . .
Vv14g0614 Tc04_g024400 . Gorai.007G282900.1 .
. . . . . . . .
. Vv01g0676 . Gorai.002G170600.1
Gorai.005G187000.1 . . .
Vv14g0615 . . . . . . .
. . . . . . . .
. . . . .
Vv14g0616 . . Gorai.007G282600.1 . .
. . . . . . . .

. . . . . . .
Vv14g0617 Tc04_g024410 . Gorai.007G282500.1 .
. . . . . . . .
. Vv01g0675 . . . . .
.
Vv14g0618 . . . . . . .
. . . . . . . .
. . . . .
Vv14g0619 . . . . . . .
. . . . . . . .
. . . . .
Vv14g0620 . . Gorai.007G282100.1 . .
. . . . . . . .
Vv01g0674 . Gorai.002G170700.1 Gorai.
005G187000.1 . . .
Vv14g0621 . . . . . . .
. . . . . . . .
. . . . .
Vv14g0622 . Gorai.013G121000.1 Gorai.007G282000.1
. . . Vv05g0619 Tc04_g024410
Gorai.006G242800.1 Gorai.005G045500.1 . . .
Vv01g0672 . . Gorai.005G186900.1 .
. .
Vv14g0623 . . . . . . .
. . . . . . . .
. . . . .
Vv14g0624 . . . . . . .
. . . . . . . .
. . . . .
Vv14g0625 Tc05_g026700 . . . . .
. . . . . . . .
. . . . . .
Vv14g0626 Tc05_g017380 . Gorai.007G279900.1 .
. . Vv05g0632 . . . .
. . . . . . . .
.
Vv14g0627 . . . . . .
Vv05g0641 . Gorai.006G243500.1 . . .
. . . . . . . .
Vv14g0628 . . . . . .
Vv05g0642 . . . . . . .
. . . . . .
Vv14g0629 . . . . . . .
. . . . . . . .
. . . . .
Vv14g0630 . . . . . . .
. . . . . . . .
. . . . .
Vv14g0631 . . . . . . .
. . . . . . . .

```
        .        .       .        .       .
Vv14g0632        Tc05_g017900     .       .        .       .        .
        .        .       .        .       .        .       .        .
        .        .       .        .       .        .
Vv14g0633        Tc04_g009190     .       .        Gorai.007G140300.1
        .        .       .        .       .        .       .        .
        .        .       .        .       .        .       .        .
Vv14g0634        .       .        .       Gorai.007G140600.1        .
        .        .       .        .       .        .       .        .
        .        .       .        .       .        .       .
Vv14g0635        .       Gorai.013G120900.1        .       .        .
        .        .       .        .       .        .       .        .
        .        .       .        .       .        .       .
Vv14g0636        Tc04_g020510     Gorai.013G120800.1       .        .
        .        .       Vv05g0663        .        .       .        .
        .        .       .        .       .        .       .        .
        .
Vv14g0637        .       .        .       .        .       .        .
        .        .       .        .       .        .       .        .
        .        .       .        .       .
Vv14g0638        .       .        .       .        .       .        .
        .        .       .        .       .        .       .        .
        .        .       .        .       .
Vv14g0639        Tc04_g008940     .       .        .       .        .
        .        .       .        .       .        .       .        .
        .        .       .        .       .        .
Vv14g0640        .       .        .       .        .       .        .
        .        .       .        .       .        .       .        .
        .        .       .        .       .
Vv14g0641        .       .        .       .        .       .        .
        .        .       .        .       .        .       .        .
        .        .       .        .       .
Vv14g0642        .       .        .       Gorai.007G141500.1        .
        .        .       .        .       .        .       .        .
        .        .       .        .       .        .       .
Vv14g0643        Tc04_g020330     .       .        .       .        .
        .        .       .        .       .        .       .        .
        .        .       .        .       .        .
Vv14g0644        .       .        .       Gorai.013G200900.1        .
        .        .       .        .       .        .       .        .
        .        .       .        .       .        .       .
Vv14g0645        Tc05_g018100     .       .        Gorai.007G141800.1
        .        .       .        .       .        .       .        .
        .        .       .        .       .        .       .        .
Vv14g0646        .       .        .       .        .       .        .
        .        .       .        .       .        .       .        .
        .        .       .        .       .
Vv14g0647        Tc05_g027190     .       .        .       .        .
        .        .       .        .       .        .       .        .
        .        .       .        .       .        .
```

```
Vv14g0648       Tc04_g023370     .       .       .       .       .
       .       .       .       .       .       .       .       .
       .       .       .       .       .       .
Vv14g0649       Tc05_g027370     .       .       .       .       .
       .       .       .       .       .       .       .       .
       .       .       .       .       .       .
Vv14g0650       .       .       .       .       .       .       .
       .       .       .       .       .       .       .       .
       .       .       .       .       .
Vv14g0651       Tc04_g024590     .       .       Gorai.007G141900.1
       .       .       Vv05g0679       Tc04_g024590     Gorai.
006G245900.1     Gorai.005G046400.1      .       .       .       .
       .       .       .       .       .       .
Vv14g0652       .       .       .       .       .       .       .
       .       .       .       .       .       .       .       .
       .       .       .       .       .
Vv14g0653       .       .       .       .       .       .       .
       .       .       .       .       .       .       .       .
       .       .       .       .       .
Vv14g0654       .       .       .       .       .       .       .
       .       .       .       .       .       .       .       .
       .       .       .       .       .
Vv14g0655       .       .       .       .       .       .       .
       .       .       .       .       .       .       .       .
       .       .       .       .       .
Vv14g0656       .       .       .       .       .       .       .
       .       .       .       .       .       .       .       .
       .       .       .       .       .
Vv14g0657       .       .       .       .       .       .       .
       .       .       .       .       .       .       .       .
       .       .       .       .       .
Vv14g0658       .       .       .       .       .       .
Vv01g0664       .       .       .       .       .       .       .
       .       .       .       .       .       .
Vv14g0659       .       .       .       .       .       .       .
       .       .       .       .       .       .       .       .
       .       .       .       .       .
Vv14g0660       .       .       .       .       .       .       .
       .       .       .       .       .       .       .       .
       .       .       .       .       .
Vv14g0661       Tc04_g024710     .       .       .       .       .
       .       .       .       .       .       .       .       .
       .       .       .       .       .       .
Vv14g0662       .       .       .       .       .       .       .
       .       .       .       .       .       .       .       .
       .       .       .       .       .
Vv14g0663       .       .       .       .       .       .       .
       .       .       .       .       .       .       .       .
       .       .       .       .       .
Vv14g0664       .       .       .       .       .       .       .
```

. . . . . . . .
. . . . .
Vv14g0665 Tc04_g024840 . . . . .
. . . . . . . .
. . . . . .
Vv14g0666 Tc04_g023010 . . . . .
. . . . . . . .
. . . . . .
Vv14g0667 . . . . . . .
. . . . . . . .
. . . . .
Vv14g0668 . . . . . . .
. . . . . . . .
. . . . .
Vv14g0669 . . . . . . .
. . . . . . . .
. . . . .
Vv14g0670 . . . . . . .
. . . . . . . .
. . . . .
Vv14g0671 . . . . . . .
. . . . . . . .
. . . . .
Vv14g0672 . . . . . . .
. . . . . . . .
. . . . .
Vv14g0673 . . . . . . .
. . . . . . . .
. . . . .
Vv14g0674 . . . . . . .
. . . . . . . .
. . . . .
Vv14g0675 . . . . . . .
. . . . . . . .
. . . . .
Vv14g0676 . . . . . . .
. . . . . . . .
. . . . .
Vv14g0677 . . . . . . .
. . . . . . . .
. . . . .
Vv14g0678 . . . . . . .
. . . . . . . .
. . . . .
Vv14g0679 . . . . . . .
. . . . . . . .
. . . . .
Vv14g0680 . . . . . . .
. . . . . . . .
. . . . .

Vv14g0681 Tc04_g022630 . . . . .
. . . . . . . .
. . . . . .
Vv14g0682 . . . . . . .
. . . . . . . .
. . . . .
Vv14g0683 . . . . . . .
. . . . . . . .
. . . . .
Vv14g0684 . . . . . . .
. . . . . . . .
. . . . .
Vv14g0685 . . . . . . .
. . . . . . . .
. . . . .
Vv14g0686 . . . . . . .
. . . . . . . .
. . . . .
Vv14g0687 . . . . . . .
. . . . . . . .
. . . . .
Vv14g0688 . . . . . . .
. . . . . . . .
. . . . .
Vv14g0689 . . . . . . .
. . . . . . . .
. . . . .
Vv14g0690 . . . . . . .
. . . . . . . .
. . . . .
Vv14g0691 . . . . . . .
. . . . . . . .
. . . . .
Vv14g0692 . . . . . . .
. . . . . . . .
. . . . .
Vv14g0693 . . . . . . .
. . . . . . . .
. . . . .
Vv14g0694 . . . . . . .
. . . . . . . .
. . . . .
Vv14g0695 . . . . . . .
. . . . . . . .
. . . . .
Vv14g0696 . . . . . . .
. . . . . . . .
. . . . .
Vv14g0697 . . . . . . .
. . . . . . . .

| | | | | | | | |
|---|---|---|---|---|---|---|---|
| . | . | . | . | . | | | |
| Vv14g0698 | . | . | . | . | . | . | . |
| . | . | . | . | . | . | . | . |
| . | . | . | . | . | | | |
| Vv14g0699 | . | . | . | . | . | . | . |
| . | . | . | . | . | . | . | . |
| . | . | . | . | . | | | |
| Vv14g0700 | . | . | . | . | . | . | . |
| . | . | . | . | . | . | . | . |
| . | . | . | . | . | | | |
| Vv14g0701 | . | . | . | . | . | . | . |
| . | . | . | . | . | . | . | . |
| . | . | . | . | . | | | |
| Vv14g0702 | . | . | . | . | . | . | . |
| . | . | . | . | . | . | . | . |
| . | . | . | . | . | | | |
| Vv14g0703 | . | . | . | . | . | . | . |
| . | . | . | . | . | . | . | . |
| . | . | . | . | . | | | |
| Vv14g0704 | . | . | . | . | . | . | . |
| . | . | . | . | . | . | . | . |
| . | . | . | . | . | | | |
| Vv14g0705 | . | . | . | . | . | . | . |
| . | . | . | . | . | . | . | . |
| . | . | . | . | . | | | |
| Vv14g0706 | . | . | . | . | . | . | . |
| . | . | . | . | . | . | . | . |
| . | . | . | . | . | | | |
| Vv14g0707 | . | . | . | . | . | . | . |
| . | . | . | . | . | . | . | . |
| . | . | . | . | . | | | |
| Vv14g0708 | Tc04_g029300 | | . | . | . | . | . |
| . | . | . | . | . | . | . | . |
| . | . | . | . | . | . | | |
| Vv14g0709 | . | . | . | . | . | . | . |
| . | . | . | . | . | . | . | . |
| . | . | . | . | . | | | |
| Vv14g0710 | . | . | . | . | . | . | . |
| . | . | . | . | . | . | . | . |
| . | . | . | . | . | | | |
| Vv14g0711 | . | . | . | . | . | . | . |
| . | . | . | . | . | . | . | . |
| . | . | . | . | . | | | |
| Vv14g0712 | . | . | . | . | . | . | . |
| . | . | . | . | . | . | . | . |
| . | . | . | . | . | | | |
| Vv14g0713 | . | . | . | . | . | . | . |
| . | . | . | . | . | . | . | . |
| . | . | . | . | . | | | |
| Vv14g0714 | . | . | . | . | . | . | . |

| | | | | | | | |
|---|---|---|---|---|---|---|---|
| Vv14g0715 | . | . | . | . | . | . | . |
| Vv14g0716 | . | . | . | . | . | . | . |
| Vv14g0717 | . | . | . | . | . | . | . |
| Vv14g0718 | Tc04_g029380 | | . | . | . | . | . |
| Vv14g0719 | . | . | . | . | . | . | . |
| Vv14g0720 | . | . | . | . | . | . | . |
| Vv14g0721 | . | . | . | . | . | . | . |
| Vv14g0722 | . | . | . | . | . | . | . |
| Vv14g0723 | . | . | . | . | . | . | . |
| Vv14g0724 | . | . | . | . | . | . | . |
| Vv14g0725 | . | . | . | . | . | . | . |
| Vv14g0726 | . | . | . | . | . | . | . |
| Vv14g0727 | . | . | . | . | . | . | . |
| Vv14g0728 | . | . | . | . | . | . | . |
| Vv14g0729 | . | . | . | . | . | . | . |
| Vv14g0730 | . | . | . | . | . | . | . |

Vv14g0731 . . . . . . .
. . . . . . . .
. . . . .
Vv14g0732 . . . . . .
Vv01g0427 . . Gorai.005G193700.1 . .
. . . . . . . .
Vv14g0733 . . . . . . .
. . . . . . . .
. . . . .
Vv14g0734 . . . . . . .
. . . . . . . .
. . . . .
Vv14g0735 . . . . . . .
. . . . . . . .
. . . . .
Vv14g0736 Tc04_g013830 . . . . .
Vv17g0322 . Gorai.001G084000.1 . .
. . Vv01g0421 . . . .
. .
Vv14g0737 . . . . . . .
. . . . . . . .
. . . . .
Vv14g0738 . . . . . . .
. . . . . . . .
. . . . .
Vv14g0739 . . . . . . .
. . . . . . . .
. . . . .
Vv14g0740 Tc04_g013880 . . Gorai.007G184400.1
. . . . . . . .
. . . . . . . .
Vv14g0741 . . . . . . .
. . . . . . . .
. . . . .
Vv14g0742 . . . . . . .
. . . . . . . .
. . . . .
Vv14g0743 . . . . . . .
. . . . . . . .
. . . . .
Vv14g0744 Tc04_g013950 . . . . .
. . . . . . . .
. . . . . .
Vv14g0745 . . . . . . .
. . . . . . . .
. . . . .
Vv14g0746 Tc04_g013970 . . . . .
. . . . . . .
Vv01g0407 . . . . . .
Vv14g0747 . . . . . . .

. . . . . . . .
. . . . .
Vv14g0748 . . . . . . .
. . . . . . . .
. . . . .
Vv14g0749 Tc04_g029540 . . . . .
. . . . . . . .
. . . . . .
Vv14g0750 . . . . . . .
. . . . . . . .
. . . . .
Vv14g0751 Tc04_g014300 . . Gorai.007G183400.1
. . . . . . . .
. . . . . . . .
Vv14g0752 . . . . . . .
. . . . . . . .
. . . . .
Vv14g0753 . . . . . . .
. . . . . . . .
. . . . .
Vv14g0754 . . . . . . .
. . . . . . . .
. . . . .
Vv14g0755 Tc04_g014320 . . Gorai.007G182700.1
. . Vv17g0344 . . Gorai.
006G059300.1 . . . . . . .
. . .
Vv14g0756 Tc04_g014330 . . Gorai.007G182500.1
. . . . . . . .
. . . . . . . .
Vv14g0757 . . . . . . .
. . . . . . . .
. . . . .
Vv14g0758 Tc04_g014340 . . Gorai.007G182400.1
. . . . . . . .
. . . . . . . .
Vv14g0759 Tc04_g014350 . . . . .
. . . . . . .
Vv01g0389 . . . . . .
Vv14g0760 . . . . . . .
. . . . . . . .
. . . . .
Vv14g0761 . . . . . . .
. . . . . . . .
. . . . .
Vv14g0762 . . . . . . .
. . . . . . . .
. . . . .
Vv14g0763 . . . . . . .
. . . . . . . .

. . . . .
Vv14g0764 Tc04_g014420 . . . Gorai.
011G228800.1 . Vv17g0349 . . Gorai.
006G060000.1 . . . Vv01g0384 . .
Gorai.005G192100.1 Gorai.009G347900.1 .
Gorai.012G124300.1
Vv14g0765 Tc04_g014430 . . Gorai.007G182300.1
. . . . . . . .
. . . . . . . .
Vv14g0766 Tc04_g014440 . . Gorai.007G182200.1
. . . . . . . .
. . . . . . . .
Vv14g0767 Tc04_g014560 . . . . .
. . . . . . .
Vv01g0377 Tc02_g009740 . . . . .
Vv14g0768 Tc04_g014570 . . . . .
. . . . . . . .
. . . . . .
Vv14g0769 . . . . . . .
. . . . . . . .
. . . . .
Vv14g0770 Tc04_g014590 . . Gorai.007G181900.1
. . . . . . . .
. . . . . . . .
Vv14g0771 Tc04_g014610 . . . . .
Vv17g0355 . Gorai.001G081000.1 . .
. . Vv01g0374 . . . .
. .
Vv14g0772 . . . . . . .
. . . . . . . .
. . . . .
Vv14g0773 . . . . . . .
. . . . . . . .
. . . . .
Vv14g0774 . . . . . . .
. . . . . . . .
. . . . .
Vv14g0775 . . . . . . .
. . . . . . . .
. . . . .
Vv14g0776 . . . . . . .
. . . . . . . .
. . . . .
Vv14g0777 Tc04_g014640 . . . . .
Vv17g0361 . . Gorai.006G060100.1 .
. . . . . . . .
.
Vv14g0778 Tc04_g014660 . . . . .
. . . . . . . .
. . . . . .

Vv14g0779 Tc04_g014670 . . Gorai.007G181800.1
. . . . . . . .
. Vv01g0372 . . . . .
.
Vv14g0780 Tc04_g014680 . . . . .
Vv17g0362 . Gorai.001G075100.1 Gorai.
006G060200.1 . . Gorai.006G194400.1 . .
. . . . .
Vv14g0781 Tc04_g014710 . . Gorai.007G181600.1
. . . . . . . .
. . . . . . . .
Vv14g0782 Tc04_g014730 . . . . .
. . . . . . . .
. . . . . .
Vv14g0783 . . . . . . .
. . . . . . . .
. . . . .
Vv14g0784 Tc04_g014740 . . . . .
. . . . . . . .
. . . . . .
Vv14g0785 Tc04_g014750 . . . . .
. . . . . . . .
. . . . . .
Vv14g0786 Tc04_g014760 . . . . .
. . . . . . . .
. . . . . .
Vv14g0787 . . . . . . .
. . . . . . . .
. . . . .
Vv14g0788 Tc04_g014770 . . . . .
. . . . . . .
Vv01g0357 . . Gorai.005G191800.1 . .
.
Vv14g0789 . . . . . . .
. . . . . . . .
. . . . .
Vv14g0790 Tc04_g014790 . . Gorai.007G181500.1
. . Vv17g0383 . . . .
. . Vv01g0353 . . Gorai.
005G190600.1 . . .
Vv14g0791 . . . . . . .
. . . . . . . .
. . . . .
Vv14g0792 . . . . . . .
. . . . . . . .
. . . . .
Vv14g0793 . . . . . . .
. . . . . . . .
. . . . .
Vv14g0794 . . . . . . .

. . . . . . . .
. . . . .
Vv14g0795 Tc04_g014830 . . . . .
Vv17g0390 . . Gorai.006G061200.1 .
. . Vv01g0351 Tc02_g010100 Gorai.
002G217800.1 Gorai.005G190300.1 . . .
Vv14g0796 Tc04_g014850 . . Gorai.007G181200.1
. . . . . . . .
. . . . . . . .
Vv14g0797 . . . . . . .
. . . . . . . .
. . . . .
Vv14g0798 Tc04_g014860 . . Gorai.007G181100.1
Gorai.011G231100.1 . . . . .
. . . . . . . .
. .
Vv14g0799 . . . . . . .
. . . . . . . .
. . . . .
Vv14g0800 . . . . . . .
. . . . . . . .
. . . . .
Vv14g0801 . . . . . . .
. . . . . . Vv01g0344
. Gorai.002G218500.1 . . . .
Vv14g0802 . . . . . . .
. . . . . . . .
. . . . .
Vv14g0803 Tc04_g014870 . . . . .
. . . . . . . .
. . . . . .
Vv14g0804 . . . . . . .
. . . . . . . .
. . . . .
Vv14g0805 . . . . . . .
. . . . . . . .
. . . . .
Vv14g0806 Tc04_g014900 . . . . .
. . . . . . .
Vv01g0337 Tc02_g034190 Gorai.013G076800.1 . .
. .
Vv14g0807 . . . . . . .
. . . . . . . .
. . . . .
Vv14g0808 Tc04_g014920 . . . . .
. . . . . . .
Vv01g0328 Tc02_g034280 Gorai.013G081600.1 Gorai.
005G138400.1 . . .
Vv14g0809 . . . . . . .
. . . . . . . .

. . . . .
Vv14g0810 . . . Gorai.007G180900.1 .
. . . . . . . .
Vv01g0325 Tc02_g034310 . Gorai.005G138300.1
. . .
Vv14g0811 . . . . . . .
. . . . . . . .
. . . . .
Vv14g0812 . . . . . . .
. . . . . . . .
. . . . .
Vv14g0813 . . . . . . .
. . . . . . . .
. . . . .
Vv14g0814 . . . . . . .
. . . . . . . .
. . . . .
Vv14g0815 Tc04_g015090 . . . . .
. . . . . . . .
. . . . . .
Vv14g0816 Tc04_g015100 . . . . .
. . . . . . .
Vv01g0321 Tc02_g034360 . . . Gorai.
008G184600.1 .
Vv14g0817 . . . . . .
Vv17g0417 . . Gorai.006G062300.1 .
Gorai.008G184700.1 Gorai.006G195300.1 Vv01g0319
Tc02_g034390 . . . . .
Vv14g0818 . . . . . . .
. . . . . . . .
. . . . .
Vv14g0819 Tc04_g015110 . . . . .
. . . . . . . .
. . . . . .
Vv14g0820 Tc04_g015120 . . . . .
. . . . . . . .
. . . . . .
Vv14g0821 Tc04_g015130 . Gorai.001G201400.1 .
. . . . . . . .
. . . . . . . .
Vv14g0822 Tc04_g015140 . . Gorai.007G189000.1
. . . . . . . .
. . . . . . . .
Vv14g0823 Tc04_g015150 . . . . .
. . . . . . . .
. . . . . .
Vv14g0824 Tc04_g015160 . . Gorai.007G180600.1
. . . . . . . .
. . . . . . . .
Vv14g0825 . . . . . . .

. . . . . . . .
. . . . .
Vv14g0826 . . . . . . .
. . . . . . . .
. . . . .
Vv14g0827 . . . . . . .
. . . . . . . .
. . . . .
Vv14g0828 Tc04_g023450 . . . . .
. . . . . . . .
. . . . . .
Vv14g0829 . . . . . . .
. . . . . . . .
. . . . .
Vv14g0830 Tc04_g023430 . . . . .
. . . . . . . .
. . . . . .
Vv14g0831 Tc04_g023420 . . . . .
. . . . . . . .
. . . . . .
Vv14g0832 Tc04_g015170 . . . . .
Vv17g0428 Tc03_g007010 . . Gorai.
007G003000.1 . . . . . . .
. .
Vv14g0833 Tc04_g015190 . . Gorai.007G180300.1
. . . . . . . .
. . . . . . . .
Vv14g0834 . . . . . . .
. . . . . . . .
. . . . .
Vv14g0835 . . . . . . .
. . . . . . . .
. . . . .
Vv14g0836 . . . Gorai.007G179100.1 .
. . . . . . . .
. . . . . . .
Vv14g0837 Tc04_g015210 . . Gorai.007G176300.1
. . . . . . . .
. . . . . . . .
Vv14g0838 . . . . . . .
. . . . . . . .
. . . . .
Vv14g0839 Tc04_g015230 . . . . .
. . . . . . . .
. . . . . .
Vv14g0840 Tc04_g015240 . . . . .
. . . . . . . .
. . . . . .
Vv14g0841 Tc04_g015260 . . . . .
. . . . . . .

Vv01g0294 Tc02_g034590 . . . Gorai.
008G186200.1 .
Vv14g0842 Tc04_g015290 . . . . .
. . . . . . .
Vv01g0287 Tc02_g034650 . . . Gorai.
008G186800.1 .
Vv14g0843 . . . . . . .
. . . . . . . .
. . . . .
Vv14g0844 Tc04_g015300 . . . . .
. . . . . . . .
. . . . . .
Vv14g0845 . . . . . .
Vv17g0445 . . Gorai.006G063500.1 . .
. . . . . . . .
Vv14g0846 Tc04_g015310 . . . . .
Vv17g0446 . . . . . .
. . . . . . .
Vv14g0847 . . . . . . .
. . . . . . . .
. . . . .
Vv14g0848 Tc04_g015320 . . . . .
. . . . . . .
Vv01g0280 Tc02_g034710 . . . Gorai.
008G187200.1 .
Vv14g0849 . . . . . . .
. . . . . . . .
. . . . .
Vv14g0850 . . . . . . .
. . . . . . . .
. . . . .
Vv14g0851 . . . . . . .
. . . . . . . .
. . . . .
Vv14g0852 Tc04_g015480 . . . . .
. . . . . . .
Vv01g0273 . . . . . .
Vv14g0853 . . . . . . .
. . . . . . . .
. . . . .
Vv14g0854 . . . . . . .
. . . . . . . .
. . . . .
Vv14g0855 . . . . . . .
. . . . . . . .
. . . . .
Vv14g0856 . . . . . . .
. . . . . . . .
. . . . .
Vv14g0857 . . . . . . .

. . . . . . . .
. . . . .
Vv14g0858 . . . . . . .
. . . . . . . .
. . . . .
Vv14g0859 . . Gorai.001G203600.1 . .
. . . . . . . .
. . . . . . .
Vv14g0860 . . . . . . .
. . . . . . . .
. . . . .
Vv14g0861 . . . . . . .
. . . . . . . .
. . . . .
Vv14g0862 . . . . . . .
. . . . . . . .
. . . . .
Vv14g0863 . . . . . . .
. . . . . . . .
. . . . .
Vv14g0864 . . . Gorai.007G187100.1 .
. . . . . . . .
. . . . . . .
Vv14g0865 Tc05_g001710 . . . Gorai.
012G118800.1 . . . . . . .
. . . . . . . .
Vv14g0866 . . . . . . .
. . . . . . . .
. . . . .
Vv14g0867 Tc05_g001700 . . . . .
. . . . . . . .
. . . . . .
Vv14g0868 . . . . . . .
. . . . . . . .
. . . . .
Vv14g0869 . . . . . . .
. . . . . . . .
. . . . .
Vv14g0870 . . . . . . .
. . . . . . . .
. . . . .
Vv14g0871 . . . . . . .
. . . . . . . .
. . . . .
Vv14g0872 . . . . . . .
. . . . . . . .
. . . . .
Vv14g0873 . . . . . . .
. . . . . . . .
. . . . .

Vv14g0874 Tc04_g015580 . . . . .
. . . . . . . .
. . . . . .
Vv14g0875 . . . . . . .
. . . . . . . .
. . . . .
Vv14g0876 . . . Gorai.007G186800.1 .
. . . . . . . .
. . . . . . .
Vv14g0877 . . . . . . .
. . . . . . . .
. . . . .
Vv14g0878 . . . . . .
Vv17g0482 . . . . . . .
. . . . . .
Vv14g0879 Tc04_g015610 . Gorai.001G203800.1
Gorai.007G186600.1 . . . . . .
. . . . . . . .
. .
Vv14g0880 Tc04_g015620 . . . . .
. . . . . . . .
. . . . . .
Vv14g0881 Tc04_g017870 . . . . .
. . . . . . . .
. . . . . .
Vv14g0882 Tc04_g015630 . . . . .
. . . . . . . .
. . . . . .
Vv14g0883 . . . . . . .
. . . . . . . .
. . . . .
Vv14g0884 . . . . . . .
. . . . . . . .
. . . . .
Vv14g0885 . . . . . . .
. . . . . . . .
. . . . .
Vv14g0886 Tc04_g015640 . . . . .
. . . . . . . .
. . . . . .
Vv14g0887 Tc04_g015650 . . . . .
. . . . . . . .
. . . . . .
Vv14g0888 . . . . . . .
. . . . . . . .
. . . . .
Vv14g0889 . . . . . . .
. . . . . . . .
. . . . .
Vv14g0890 Tc04_g015680 . . . . .

. . . . . . . .
. . . . . .
Vv14g0891 . . . . . . .
. . . . . . . .
. . . . .
Vv14g0892 . . . . . . .
. . . . . . . .
. . . . .
Vv14g0893 . . . . . . .
. . . . . . . .
. . . . .
Vv14g0894 Tc04_g004790 . . . . .
. . . . . . . .
. . . . . .
Vv14g0895 . . . . . . .
. . . . . . . .
. . . . .
Vv14g0896 . . . . . . .
. . . . . . Vv01g0001
. . . . . .
Vv14g0897 . . Gorai.001G205700.1 Gorai.
007G184400.1 . . . . . . .
. . . . . . . .
.
Vv14g0898 . . Gorai.001G205800.1 . .
. . . . . . . .
. . . . . . .
Vv14g0899 Tc04_g015740 . . . . .
. . . . . . . .
. . . . . .
Vv14g0900 Tc04_g005140 . . . .
Gorai.012G082800.1 . . . . . .
. . . . . . . .
Vv14g0901 . . . . . . .
. . . . . . . .
. . . . .
Vv14g0902 . . Gorai.001G206000.1 . .
. . . . . . . .
. . . . . . .
Vv14g0903 . . . . . . .
. . . . . . . .
. . . . .
Vv14g0904 . . . . . . .
. . . . . . . .
. . . . .
Vv14g0905 . . . . . . .
. . . . . . . .
. . . . .
Vv14g0906 . . Gorai.001G206200.1 . .
. . . . . . . .

. . . . . . .
Vv14g0907 . . . . . . .
. . . . . . . .
. . . . .
Vv14g0908 Tc04_g002220 . . . . .
. . . . . . . .
. . . . . .
Vv14g0909 Tc05_g023260 . . . . .
. . . . . . . .
. . . . . .
Vv14g0910 Tc04_g017860 . . . . .
. . . . . . . .
. . . . . .
Vv14g0911 . . . . . . .
. . . . . . . .
. . . . .
Vv14g0912 Tc04_g002020 . . . . .
. . . . . . . .
. . . . . .
Vv14g0913 Tc04_g002010 Gorai.011G094300.1 . .
. . . . . . . .
. . . . . . . .
Vv14g0914 Tc04_g002000 Gorai.011G094400.1 . .
. . . . . . . .
. . . . . . . .
Vv14g0915 . . . . . . .
. . . . . . . .
. . . . .
Vv14g0916 Tc04_g015880 . . Gorai.007G175600.1
. . . . . . . .
. . . . . . . .
Vv14g0917 . . . . . .
Vv17g0512 Tc03_g012770 Gorai.001G066500.1 . .
Gorai.008G175000.1 . . . . .
. . .
Vv14g0918 . . . . . . .
. . . . . . . .
. . . . .
Vv14g0919 Tc04_g020920 . . . Gorai.
011G156300.1 . . . . . . .
. . . . . . . .
Vv14g0920 . . . . . . .
. . . . . . . .
. . . . .
Vv14g0921 . . . Gorai.007G040600.1 .
. Vv17g0513 Tc03_g012760 . . .
Gorai.008G175100.1 Gorai.006G269200.1 . .
. . . . .
Vv14g0922 . . . . . .
Vv17g0514 Tc03_g012750 Gorai.001G066600.1 .

```
Gorai.007G040500.1       .        .        .        .        .        .
        .        .        .
Vv14g0923        .        .        .        .        .        .        .
        .        .        .        .        .        .        .        .
        .        .        .        .        .
Vv14g0924        .        .        Gorai.001G206500.1        .        .
        .        .        .        .        .        .        .        .
        .        .        .        .        .        .        .
Vv14g0925        .        .        .        .        .        .        .
        .        .        .        .        .        .        .        .
        .        .        .        .        .
Vv14g0926        .        .        .        .        .        .
Vv17g0525        .        Gorai.001G067500.1        .        .        .
        .        .        .        .        .        .        .        .
Vv14g0927        .        .        .        .        .        .        .
        .        .        .        .        .        .        .        .
        .        .        .        .        .
Vv14g0928        .        .        .        .        .        .        .
        .        .        .        .        .        .        .        .
        .        .        .        .        .
Vv14g0929        .        .        Gorai.001G206900.1        .        .
        .        .        .        .        .        .        .        .
        .        .        .        .        .        .        .
Vv14g0930        .        .        Gorai.001G207000.1        .        .
        .        .        .        .        .        .        .        .
        .        .        .        .        .        .        .
Vv14g0931        .        .        Gorai.001G207100.1        .        .
        .        .        .        .        .        .        .        .
        .        .        .        .        .        .        .
Vv14g0932        .        .        Gorai.001G207200.1        .        .
        .        .        .        .        .        .        .        .
        .        .        .        .        .        .        .
Vv14g0933        .        .        .        .        .        .        .
        .        .        .        .        .        .        .        .
        .        .        .        .        .
Vv14g0934        Tc05_g031790        .        .        .        .        .
        Vv17g0538        Tc03_g012620        .        .        .        .
        .        Vv01g0014        Tc02_g031670        Gorai.013G096100.1
        Gorai.005G147200.1        .        .        .
Vv14g0935        .        .        .        .        .        .        .
        .        .        .        .        .        .        .        .
        .        .        .        .        .
Vv14g0936        .        .        .        .        .        .
Vv17g0539        Tc03_g012600        Gorai.001G075800.1        .        .
        .        Gorai.006G268400.1        Vv01g0017
Tc02_g031690        Gorai.013G096000.1        .        .        .        .
Vv14g0937        .        .        .        .        .        .        .
        .        .        .        .        .        .        .        .
        .        .        .        .        .
Vv14g0938        Tc05_g023430        Gorai.013G113000.1        .        .
```

. . . . . . . .
. . . . . . . .
Vv14g0939 . . . . . . .
. . . . . . . .
. . . . .
Vv14g0940 . . Gorai.001G207700.1 . .
. . . . . . . .
. . . . . . .
Vv14g0941 . . Gorai.001G208000.1 Gorai.
007G039800.1 . . Vv17g0542 Tc03_g012560
Gorai.001G076300.1 . . Gorai.008G200200.1 .
Vv01g0021 Tc02_g031760 Gorai.013G095500.1 .
. Gorai.008G200200.1 .
Vv14g0942 Tc04_g021220 . . . Gorai.
011G158000.1 . . . . . . .
. . . . . . . .
Vv14g0943 . . Gorai.001G208200.1 . .
. . . . . . . .
. . . . . . .
Vv14g0944 . . . . . . .
. . . . . . . .
. . . . .
Vv14g0945 . . . . . . .
. . . . . . . .
. . . . .
Vv14g0946 . . . . . . .
. . . . . . . .
. . . . .
Vv14g0947 . . . . . . .
. . . . . . . .
. . . . .
Vv14g0948 . . . . . .
Vv17g0546 Tc03_g012490 Gorai.001G076200.1 . .
Gorai.008G200100.1 . . . . .
. . .
Vv14g0949 . . Gorai.001G208300.1 . .
. Vv17g0547 Tc03_g012460 . . .
Gorai.008G200000.1 . . . . .
. . .
Vv14g0950 . . . . . . .
. . . . . . . .
. . . . .
Vv14g0951 . . . . . . .
. . . . . . . .
. . . . .
Vv14g0952 . . Gorai.001G208400.1 . .
. . . . . . . .
. . . . . . .
Vv14g0953 . . Gorai.001G208500.1 . .
. . . . . . . .

. . . . . . .
Vv14g0954 . . . . . . .
. . . . . . . .
. . . . .
Vv14g0955 Tc04_g015890 . . . Gorai.
011G124900.1 . . . . . . .
. . . . . . . .
Vv14g0956 . . . . . . .
. . . . . . . .
. . . . .
Vv14g0957 . . . . . . .
. . . . . . . .
. . . . .
Vv14g0958 . . . . . . .
. . . . . . . .
. . . . .
Vv14g0959 . . . . . . .
. . . . . . . .
. . . . .
Vv14g0960 . . Gorai.001G208900.1 . .
. . . . . . . .
Vv01g0030 . . . . . .
Vv14g0961 . . . . . . .
. . . . . . . .
. . . . .
Vv14g0962 . . . . . . .
. . . . . . . .
. . . . .
Vv14g0963 . . . . . . .
. . . . . . . .
. . . . .
Vv14g0964 . . . . . . .
. . . . . . . .
. . . . .
Vv14g0965 Tc04_g020290 . . . Gorai.
011G151900.1 . . . . . . .
. . . . . . . .
Vv14g0966 . . . . . . .
. . . . . . . .
. . . . .
Vv14g0967 Tc05_g031690 . . . . .
. . . . . . . .
. . . . . .
Vv14g0968 . . . . . . .
. . . . . . . .
. . . . .
Vv14g0969 . . . . . . .
. . . . . . . .
. . . . .
Vv14g0970 Tc04_g016050 . . . . .

. . . . . . . .
. . . . . .
Vv14g0971 . . . . . . .
. . . . . . . .
. . . . .
Vv14g0972 . . . . . . .
. . . . . . . .
. . . . .
Vv14g0973 . . . . . . .
. . . . . . . .
. . . . .
Vv14g0974 . . . . . . .
. . . . . . . .
. . . . .
Vv14g0975 . . . . . . .
. . . . . . . .
. . . . .
Vv14g0976 . . . Gorai.007G082800.1 .
. . . . . . . .
. . . . . . .
Vv14g0977 . . . . . . .
. . . . . . . .
. . . . .
Vv14g0978 . . . . Gorai.006G010500.1
. . . . . . . .
. . . . . . .
Vv14g0979 Tc05_g019280 . . . . .
. . . . . . . .
. . . . . .
Vv14g0980 . . . Gorai.007G082700.1 .
. . . . . . . .
. . . . . . .
Vv14g0981 . . . . Gorai.006G010200.1
. . . . . . . .
. . . . . . .
Vv14g0982 . . . . . . .
. . . . . . . .
. . . . .
Vv14g0983 Tc04_g026560 . . . . .
. . . . . . . .
. . . . . .
Vv14g0984 . . . . Gorai.006G010000.1
. . . . . . . .
Vv01g0039 Tc02_g031980 Gorai.013G094600.1 .
. Gorai.008G176700.1 .
Vv14g0985 Tc04_g026400 . . Gorai.007G039600.1
Gorai.006G009900.1 . Vv17g0567
Tc03_g012260 . . . . . . .
. . . . .
Vv14g0986 . . . . . . .

```
        .        .       .        .       .        .       .        .
        .        .       .        .       .
Vv14g0987        .       .        .       .        Gorai.006G009800.1
        .        .       .        .       .        .       .        .
        .        .       .        .       .        .       .
Vv14g0988        .       .        .       .        .       .        .
        .        .       .        .       .        .       .        .
        .        .       .        .       .
Vv14g0989        .       .        .       .        Gorai.006G009600.1
        .        .       .        .       .        .       .        .
        .        .       .        .       .        .       .
Vv14g0990        .       .        .       .        .       .        .
        .        .       .        .       .        .       Vv01g0054
        Tc02_g032160     .        .       .        Gorai.008G177500.1
        .
Vv14g0991        .       .        .       .        Gorai.006G007300.1
        .        .       .        .       .        .       .        .
        .        .       .        .       .        .       .
Vv14g0992        .       .        .       .        .       .        .
        .        .       .        .       .        .       .        .
        .        .       .        .       .
Vv14g0993        Tc04_g026240     .       .        .       .        .
        .        .       .        .       .        .       .        .
        .        .       .        .       .        .
Vv14g0994        .       .        .       .        .       .        .
        .        .       .        .       .        .       .        .
        .        .       .        .       .
Vv14g0995        .       .        .       Gorai.007G039300.1        .
        .        .       .        .       .        .       .        .
        .        .       .        .       .        .       .
Vv14g0996        Tc04_g020280     .       .        .       .        .
        .        .       .        .       .        .       .        .
        .        .       .        .       .        .
Vv14g0997        Tc05_g019240     .       .        .       .        .
        .        .       .        .       .        .       .        .
        .        .       .        .       .        .
Vv14g0998        Tc05_g022410     .       .        .       .        .
        .        .       .        .       .        .       .        .
        .        .       .        .       .        .
Vv14g0999        .       .        .       Gorai.007G036500.1        .
        .        .       .        .       .        .       .        .
        .        .       .        .       .        .       .
Vv14g1000        Tc05_g022430     .       .        .       .        .
        .        .       .        .       .        .       .        .
        .        .       .        .       .        .
Vv14g1001        .       .        .       Gorai.007G082000.1        .
        .        .       .        .       .        .       .        .
        .        .       .        .       .        .       .
Vv14g1002        .       .        .       Gorai.007G079000.1        .
        .        .       .        .       .        .       .        .
```

```
        .        .       .        .       .        .       .
Vv14g1003        Tc04_g016460     .       .        .       .        .
        .        .       .        .       .        .       .        .
        .        .       .        .       .        .
Vv14g1004        .       .        .       .        .       .        .
        .        .       .        .       .        .       .        .
        .        .       .        .       .
Vv14g1005        .       .        .       .        .       .        .
        .        .       .        .       .        .       .        .
        .        .       .        .       .
Vv14g1006        .       .        .       .        .       .        .
        .        .       .        .       .        .       .        .
        .        .       .        .       .
Vv14g1007        .       .        .       .        .       .        .
        .        .       .        .       .        .       .        .
        .        .       .        .       .
Vv14g1008        .       .        .       .        .       .        .
        .        .       .        .       .        .       .        .
        .        .       .        .       .
Vv14g1009        .       .        .       .        .       .        .
        .        .       .        .       .        .       .        .
        .        .       .        .       .
Vv14g1010        .       .        .       .        .       .        .
        .        .       .        .       .        .       .        .
        .        .       .        .       .
Vv14g1011        .       .        .       .        .       .        .
        .        .       .        .       .        .       .        .
        .        .       .        .       .
Vv14g1012        .       .        .       .        .       .
Vv17g0163        Tc03_g014500     Gorai.001G060700.1       .        .
        Gorai.008G202600.1        Gorai.006G271000.1       .        .
        .        .       .        .       .
Vv14g1013        .       .        .       .        .       .        .
        .        .       .        .       .        .       .        .
        .        .       .        .       .
Vv14g1014        .       .        .       .        .       .        .
        .        .       .        .       .        .       .        .
        .        .       .        .       .
Vv14g1015        .       .        .       .        .       .        .
        .        .       .        .       .        .       .        .
        .        .       .        .       .
Vv14g1016        .       .        .       .        .       .        .
        .        .       .        .       .        .       .        .
        .        .       .        .       .
Vv14g1017        .       Gorai.011G088800.1        Gorai.001G276400.1
        Gorai.007G074000.1        Gorai.006G038900.1       .        .
        .        .       .        .       .        .       Vv01g1311
        .        .       .        .       .        .
Vv14g1018        .       .        Gorai.001G276300.1       .        .
        .        .       .        .       .        .       .        .
```

. . . . . . .
Vv14g1019 . . . . . . .
. . . . . . . .
. . . . .
Vv14g1020 . . . . . . .
. . . . . . . .
. . . . .
Vv14g1021 . . . . . . .
. . . . . . . .
. . . . .
Vv14g1022 . . . Gorai.007G045600.1
Gorai.006G040900.1 . . . . . .
. . . . . . . .
.
Vv14g1023 . . . . . . .
. . . . . . . .
. . . . .
Vv14g1024 Tc04_g005560 . . . . .
. . . . . . .
Vv01g1298 . . . . . .
Vv14g1025 Tc05_g022730 . . . . .
. . . . . . . .
. . . . . .
Vv14g1026 . . . . . . .
. . . . . . . .
. . . . .
Vv14g1027 Tc04_g005550 . . . . .
. . . . . . . .
. . . . . .
Vv14g1028 . . . . . . .
. . . . . . . .
. . . . .
Vv14g1029 Tc04_g005520 . . . . .
. . . . . . . .
. . . . . .
Vv14g1030 . . . . . . .
. . . . . . . .
. . . . .
Vv14g1031 . . . . . . .
. . . . . . . .
. . . . .
Vv14g1032 Tc04_g005510 . . . . .
. . . . . . . .
. . . . . .
Vv14g1033 Tc04_g005500 . . . . .
. . . . . . . .
. . . . . .
Vv14g1034 . . . . Gorai.006G000100.1
. . . . . . . .
. . . . . . .

Vv14g1035 . . . . . . .
. . . . . . . .
. . . . .
Vv14g1036 . . . . . . .
. . . . . . . .
. . . . .
Vv14g1037 . . . . . . .
. . . . . . . .
. . . . .
Vv14g1038 Tc04_g005480 . . . .
Gorai.012G082500.1 Vv17g0177 Tc03_g007940 . .
. . . . . . . .
. .
Vv14g1039 Tc04_g005470 . . . . .
. . . . . . . .
. . . . . .
Vv14g1040 Tc04_g005460 . . . . .
Vv17g0189 . . . Gorai.005G133500.1
. . Vv01g1294 . . . .
Gorai.008G155700.1 .
Vv14g1041 Tc04_g005410 . . . . .
Vv01g1293 . . . . . .
. . . . . . .
Vv14g1042 Tc04_g005400 . . . . .
. . . . . . . .
. . . . . .
Vv14g1043 Tc04_g005390 . . . . .
. . . . . . . .
. . . . . .
Vv14g1044 Tc04_g005360 . . . Gorai.
006G000200.1 . . . . . . .
. . . . . . . .
Vv14g1045 . . . . . . .
. . . . . . . .
. . . . .
Vv14g1046 . . . . . . .
. . . . . . . .
. . . . .
Vv14g1047 Tc04_g005330 . . . . .
. . . . . . . .
. . . . . .
Vv14g1048 Tc04_g005320 . . Gorai.007G073200.1
. . . . . . . .
. . . . . . . .
Vv14g1049 Tc04_g005310 . . Gorai.007G073100.1
. . . . . . . .
. . . . . . . .
Vv14g1050 Tc04_g005300 . . . . .
. . . . . . . .
. . . . . .

Vv14g1051 Tc04_g005290 . . . . .
. . . . . . . .
. . . . .
Vv14g1052 Tc04_g005280 Gorai.011G088500.1 .
Gorai.007G073000.1 . . . . . .
. . . . . . . .
. .
Vv14g1053 Tc04_g005270 . . Gorai.007G072900.1
. . . . . . . .
. . . . . . . .
Vv14g1054 Tc04_g005260 . . Gorai.007G072800.1
. . Vv01g1277 . . . .
. . . . . . . .
.
Vv14g1055 Tc05_g025670 . . . . .
. . . . . . . .
. . . . . .
Vv14g1056 Tc04_g005230 . Gorai.001G276200.1 .
. . . . . . . .
. . . . . . . .
Vv14g1057 Tc04_g005210 . . . .
Gorai.012G082600.1 . . . . . .
. . . . . . . .
Vv14g1058 Tc05_g025990 . . . . .
Vv01g1266 Tc02_g002960 . Gorai.005G196900.1
. . . . . . . .
. .
Vv14g1059 . . . . . . .
. . . . . . . .
. . . . .
Vv14g1060 Tc04_g005190 . . . Gorai.
006G000300.1 Gorai.012G082700.1 . . . .
. . . . . . . .
. .
Vv14g1061 Tc04_g005180 . . . . .
Vv17g0028 Tc03_g015790 . . . .
. . . . . . . .
Vv14g1062 . . . . . .
Vv17g0029 Tc03_g015780 . . . . .
. . . . . . .
Vv14g1063 Tc04_g005170 Gorai.011G088400.1 . .
. . . . . . . .
. . . . . . . .
Vv14g1064 Tc04_g005160 . . . . .
. . . . . . . .
. . . . . .
Vv14g1065 . . . . . . .
. . . . . . . .
. . . . .
Vv14g1066 Tc04_g005150 . . Gorai.007G072700.1

. . . . . . . .
. Vv01g1259 . . . . .
Gorai.012G125000.1
Vv14g1067 Tc04_g005140 . Gorai.001G275900.1 .
. Gorai.012G082800.1 . . . .
. . . . . . . .
. .
Vv14g1068 Tc04_g005120 . Gorai.001G275800.1 .
. . . . . . . .
. . . . . . . .
Vv14g1069 Tc04_g005100 . Gorai.001G275700.1 .
Gorai.006G000400.1 . . . . .
. . . Vv01g1255 Tc02_g019700 .
. . . .
Vv14g1070 . . . . . . .
. . . . . . . .
. . . . .
Vv14g1071 Tc04_g005090 . Gorai.001G275600.1
Gorai.007G044800.1 . . . . . .
. . . . . . . .
. .
Vv14g1072 Tc04_g005080 . Gorai.001G275500.1 .
Gorai.006G041300.1 . . . . .
. . . . . . . .
. .
Vv14g1073 Tc04_g005070 . . . . .
Vv17g0036 . . . . .
Gorai.006G273600.1 . . . . . .
.
Vv14g1074 . . . . . . .
. . . . . . . .
. . . . .
Vv14g1075 Tc04_g005050 Gorai.011G088100.1 Gorai.
001G275400.1 . . . . . . .
. . . . . . . .
. .
Vv14g1076 Tc04_g005020 . . . . .
. . . . . . . .
. . . . . .
Vv14g1077 . . . . . . .
. . . . . . . .
. . . . .
Vv14g1078 Tc04_g004990 . . Gorai.007G044500.1
. . . . . . . .
. . . . . . . .
Vv14g1079 . . . . . . .
. . . . . . . .
. . . . .
Vv14g1080 Tc04_g004980 . . Gorai.007G072500.1
. Gorai.012G082900.1 Vv17g0045 . .

. . . . . . . .
. . .
Vv14g1081 Tc04_g004970 . . . . .
. . . . . . . .
. . . . . .
Vv14g1082 Tc04_g004880 . . . . .
Vv17g0048 Tc03_g015590 . . .
Gorai.008G209600.1 . . . . . .
. .
Vv14g1083 . . . . . . .
. . . . . . . .
. . . . .
Vv14g1084 Tc04_g004860 . . Gorai.007G072400.1
. . . . . . . .
. Vv01g1237 . . . . .
.
Vv14g1085 . . . . . . .
. . . . . . . .
. . . . .
Vv14g1086 . . . . . . .
. . . . . . . .
. . . . .
Vv14g1087 Tc04_g004840 . . . . .
. . . . . . .
Vv01g1233 . . Gorai.005G196000.1 . .
.
Vv14g1088 Tc04_g004800 . . . Gorai.
006G000600.1 Gorai.012G083000.1 . . . .
. . . . . . . .
. .
Vv14g1089 Tc04_g004790 . . . .
Gorai.012G083100.1 . . . . . .
. Vv01g1225 . . Gorai.005G195800.1
. . .
Vv14g1090 Tc04_g004780 . . . . .
. . . . . . . .
. . . . . .
Vv14g1091 Tc04_g004770 . . Gorai.007G044200.1
Gorai.006G000900.1 . Vv17g0051
Tc03_g015560 . . . . Gorai.006G272700.1
. . . . . . .
Vv14g1092 . . . . . . .
. . . . . . . .
. . . . .
Vv14g1093 Tc04_g004760 . . Gorai.007G072100.1
Gorai.006G001000.1 . . . . .
. . . . . . . .
. .
Vv14g1094 Tc04_g004750 . . . . .
. . . . . . . .

. . . . . .
Vv14g1095 Tc04_g004740 . . . . .
. . . . . . . .
. . . . . .
Vv14g1096 . . . . . . .
. . . . . . . .
. . . . .
Vv14g1097 . . . . . . .
. . . . . . . .
. . . . .
Vv14g1098 Tc04_g004730 . . . .
Gorai.012G083200.1 . . . . . . .
. . . . . . . .
Vv14g1099 Tc04_g004720 . . . .
Gorai.012G083300.1 Vv17g0052 Tc03_g015550 . .
. . . . . . . .
. .
Vv14g1100 Tc04_g004710 . . . .
Gorai.012G083400.1 . . . . . .
. . . . . . . .
Vv14g1101 Tc04_g004700 . . . .
Gorai.012G083500.1 . . . . . .
. Vv01g1222 . . Gorai.005G195700.1
. Gorai.008G156600.1 .
Vv14g1102 Tc04_g004670 . . . .
Gorai.012G083700.1 . . . . . .
. . . . . . . .
Vv14g1103 Tc04_g004640 Gorai.011G087800.1 Gorai.
001G274600.1 . . . . . . .
. . . . . . . .
. .
Vv14g1104 . . . . . .
Vv17g0070 Tc03_g015480 . . . Gorai.
008G209300.1 . . . . . . .
.
Vv14g1105 Tc04_g004620 . . . . .
. . . . . . . .
. . . . . .
Vv14g1106 Tc04_g004610 . . . Gorai.
006G044900.1 . Vv17g0071 Tc03_g015470 . .
. Gorai.008G209200.1 . . . .
. . . .
Vv14g1107 Tc04_g004590 . . Gorai.007G071900.1
. . . . . . . .
. . . . . . . .
Vv14g1108 Tc04_g004580 . . . Gorai.
006G044700.1 . Vv17g0074 . . . .
. . . . . . . .
.
Vv14g1109 Tc04_g004560 . . . Gorai.

```
006G044500.1     Gorai.012G083900.1       Vv17g0075         .       .
        .        .        .        .        .        .        .       .
        .        .        .
Vv14g1110        .        .        .        .        .       Gorai.
012G084000.1     .        .        .        .        .        .       .
        Vv01g1211        Tc02_g018390      .        Gorai.005G195000.1
        .        Gorai.008G156900.1       Gorai.012G124500.1
Vv14g1111        .        .        .        .        .        .       .
        .        .        .        .        .        .        .       .
        .        .        .        .        .
Vv14g1112        .        .        .        .        .        .       .
        .        .        .        .        .        .        .       .
        .        .        .        .        .
Vv14g1113        .        .        .        .        .        .       .
        .        .        .        .        .        .        .       .
        .        .        .        .        .
Vv14g1114        .        .        .        .        .        .       .
        .        .        .        .        .        .        .       .
        .        .        .        .        .
Vv14g1115        .        .        .        .        .       Gorai.
012G084100.1     .        .        .        .        .        .       .
        .        .        .        .        .        .        .
Vv14g1116        .        .        .        .        .        .
Vv17g0084        Tc03_g015350      .        .        .        .       .
        .        .        .        .        .        .        .
Vv14g1117        .        .        .        .        .        .       .
        .        .        .        .        .        .        .       .
        .        .        .        .        .
Vv14g1118        .        .        .       Gorai.007G043600.1        .
        .        Vv17g0086        Tc03_g015330     Gorai.001G056300.1
        .        .       Gorai.008G208100.1       Gorai.006G272400.1
        .        .        .        .        .        .        .
Vv14g1119        .        .        .       Gorai.007G071700.1
Gorai.006G044200.1        .        .        .        .        .       .
        .        .       Vv01g1205          .        .        .       .
        .        .
Vv14g1120        .        .        .        .        .        .       .
        .        .        .        .        .        .        .       .
        .        .        .        .        .
Vv14g1121        Tc04_g029970      .        .        .        .       .
        .        .        .        .        .        .        .       .
        .        .        .        .        .        .
Vv14g1122        .        .        .       Gorai.007G068800.1
Gorai.006G043900.1       Gorai.012G084400.1       Vv17g0088
Tc03_g015290     Gorai.001G056500.1       .        .        .       .
        Vv01g1200         .        .        .        .        .       .
Vv14g1123        .        .        .        .        .        .       .
        .        .        .        .        .        .        .       .
        .        .        .        .        .
Vv14g1124        .       Gorai.011G086900.1       Gorai.001G273300.1
```

```
        .       Gorai.006G043800.1      Gorai.012G084600.1      .
        .       .       .       .       .       .       .       .
        .       .       .       .       .
Vv14g1125       .       .       .       .       .       .       .
        .       .       .       .       .       .       .       .
        .       .       .       .       .
Vv14g1126       .       .       .       .       .       .       .
        .       .       .       .       .       .       .       .
        .       .       .       .       .
Vv14g1127       .       Gorai.011G086700.1      .       .       .
        .       .       .       .       .       .       .       .
        .       .       .       .       .       .       .
Vv14g1128       .       Gorai.011G086600.1      .       .       .
        Gorai.012G084800.1      .       .       .       .       .
        .       .       .       .       .       .       .       .
        .
Vv14g1129       Tc05_g016970    Gorai.011G086500.1      .       .
        .       .       .       .       .       .       .       .
        .       .       .       .       .       .       .       .
Vv14g1130       .       .       Gorai.001G273200.1      .       .
        Gorai.012G085000.1      .       .       .       .       .
        .       .       .       .       .       .       .       .
        .
Vv14g1131       .       .       .       .       .       .       .
        .       .       .       .       .       .       .       .
        .       .       .       .       .
Vv14g1132       Tc04_g029800    .       .       .       .       .
        .       .       .       .       .       .       .
Vv1rg0354       .       .       .       .       .       .
Vv14g1133       .       .       .       .       .       .       .
        .       .       .       .       .       .       .       .
        .       .       .       .       .
Vv14g1134       .       .       .       Gorai.007G043500.1      .
        .       .       .       .       .       .       .       .
        Vv1rg0357       .       .       .       .       .       .
Vv14g1135       .       .       .       .       .       .       .
        .       .       .       .       .       .       .       .
        .       .       .       .       .
Vv14g1136       Tc05_g017170    .       .       .       .       .
        .       .       .       .       .       .       .
Vv1rg0360       .       .       .       .       .       .
Vv14g1137       Tc04_g004290    .       Gorai.001G272800.1
Gorai.007G043300.1      .       Gorai.012G086000.1      Vv17g0107
        Tc03_g015050    Gorai.001G057900.1      .       .       .
        .       Vv1rg0365       .       .       .       .       .
        .
Vv14g1138       Tc04_g004280    .       .       .       Gorai.
006G043700.1    .       .       .       .       .       .       .
        .       Vv1rg0366       .       .       .       .       .
        .
```

Vv14g1139 . . . . . . .
. . . . . . . .
. . . . .
Vv14g1140 . . . . . . .
. . . . . . . .
. . . . .
Vv14g1141 . . Gorai.001G272600.1 . .
. . . . . . . .
. . . . . . .
Vv14g1142 Tc05_g007270 . . . Gorai.
006G042600.1 Gorai.012G086100.1 . . . .
. . . . . . . .
. .
Vv14g1143 . . . . . . .
. . . . . . . .
. . . . .
Vv14g1144 . . . . . . .
. . . . . . . .
. . . . .
Vv14g1145 Tc05_g007230 . Gorai.001G272000.1 .
Gorai.006G042500.1 . . . . .
. . . . . . . .
. .
Vv14g1146 . . . . . . .
. . . . . . . .
. . . . .
Vv14g1147 Tc04_g016340 . . . . .
. . . . . . . .
. . . . . .
Vv14g1148 . . Gorai.001G271700.1 . .
. . . . . . . .
. . . . . . .
Vv14g1149 . Gorai.011G086200.1 Gorai.001G271600.1
. . Gorai.012G086400.1 . . .
. . . . . . . .
. . .
Vv14g1150 . . . . . . .
. . . . . . . .
. . . . .
Vv14g1151 . . . . Gorai.006G042400.1
. . . . . . . .
. . . . . . .
Vv14g1152 Tc05_g002060 . . . . .
Vv17g0116 . . . . . .
. . . . . . .
Vv14g1153 . . . Gorai.007G042800.1 .
. . . . . . . .
Vv1rg0387 . . . . . .
Vv14g1154 . . . . . . .
. . . . . . . .

. . . . .
Vv14g1155 . . . . Gorai.006G042300.1
. . . . . . . .
. . . . . . .
Vv14g1156 . Gorai.011G086100.1 Gorai.001G271500.1
. . . . . . . .
. . . . . . . .
.
Vv14g1157 . . . . . Gorai.
012G086500.1 . . . . . . .
. . . . . . .
Vv14g1158 . . . . Gorai.006G042000.1
. . . . . . . .
. . . . . . .
Vv14g1159 . Gorai.011G085900.1 . .
Gorai.006G041900.1 Gorai.012G086800.1 . . .
. . . . . . . .
. . .
Vv14g1160 . . Gorai.001G271300.1 Gorai.
007G039800.1 . Gorai.012G086900.1 . . .
. . . . . . . .
. . .
Vv14g1161 . . . . . . .
. . . . . . . .
. . . . .
Vv14g1162 . . . . . . .
. . . . . . . .
. . . . .
Vv14g1163 . . Gorai.001G271200.1 . .
Gorai.012G087000.1 . . . . .
. . . . . . . .
.
Vv14g1164 . . Gorai.001G271100.1 . .
. . . . . . . .
. . . . . . .
Vv14g1165 Tc05_g007150 . . . . .
. . . . . . . .
. . . . . .
Vv14g1166 . . . . Gorai.006G041700.1
. . . . . . . .
. . . . . . .
Vv14g1167 . . Gorai.001G270800.1 . .
. . . . . . . .
. . . . . . .
Vv14g1168 . . . . . . .
. . . . . . . .
. . . . .
Vv14g1169 Tc05_g006740 . . . . .
. . . . . . . .
. . . . . .

Vv14g1170 . . . . Gorai.006G041500.1
. . . . . . . .
. . . . . . .
Vv14g1171 . Gorai.011G084300.1 Gorai.001G270700.1
. Gorai.006G041400.1 . Vv17g0133
Tc03_g014740 . . . Gorai.008G205600.1 .
. . . . . . .
Vv14g1172 . . Gorai.001G270600.1 . .
Gorai.012G087300.1 . . . . .
. . Vv1rg0424 . . . .
. .
Vv14g1173 . . . . Gorai.006G041200.1
Gorai.012G087400.1 . . . . .
. . . . . . . .
.
Vv14g1174 . . . . . . .
. . . . . . . .
. . . . .
Vv14g1175 . . Gorai.001G270200.1 . .
Gorai.012G087500.1 . . . . .
. . . . . . . .
.
Vv14g1176 . Gorai.011G091800.1 . . .
. . . . . . . .
. . . . . . .
Vv14g1177 Tc04_g016210 . . . . .
. . . . . . . .
. . . . . .
Vv14g1178 Tc05_g002040 . . . . .
. . . . . . . .
. . . . . .
Vv14g1179 . . . . . . .
. . . . . . . .
. . . . .
Vv14g1180 Tc05_g002020 . . . . .
. . . . . . . .
. . . . . .
Vv14g1181 . . . . . . .
. . . . . . . .
. . . . .
Vv14g1182 . . . . . . .
. . . . . . . .
. . . . .
Vv14g1183 . . . . . . .
. . . . . . . .
. . . . .
Vv14g1184 . . . . . . .
. . . . . . . .
. . . . .
Vv14g1185 . . . . . . .

. . . . . . . .
. . . . .
Vv14g1186 Tc04_g003440 . . . . .
. . . . . . . .
. . . . . .
Vv14g1187 . . . . . . .
. . . . . . . .
. . . . .
Vv14g1188 . . . . . . .
. . . . . . . .
. . . . .
Vv14g1189 Tc04_g003430 . . . . .
. . . . . . . .
. . . . . .
Vv14g1190 . . . . . . .
. . . . . . . .
. . . . .
Vv14g1191 Tc04_g003390 . . . . .
. . . . . . . .
. . . . . .
Vv14g1192 . . . . . . .
. . . . . . . .
. . . . .
Vv14g1193 . . . . . . .
. . . . . . . .
. . . . .
Vv14g1194 . . . . . . .
. . . . . . . .
. . . . .
Vv14g1195 . . . . . . .
. . . . . . . .
. . . . .
Vv14g1196 . . . . . . .
. . . . . . . .
. . . . .
Vv14g1197 . . . . . . .
. . . . . . . .
. . . . .
Vv14g1198 . . . . . .
Vv17g0141 Tc03_g014680 . . . . .
. . . . . . .
Vv14g1199 . Gorai.011G091700.1 Gorai.001G270100.1
. Gorai.006G041100.1 . Vv17g0144 .
. . . . . . . .
. . . .
Vv14g1200 . . Gorai.001G270000.1 . .
. . . . . . . .
. . . . . . .
Vv14g1201 . Gorai.011G091500.1 . .
Gorai.006G041000.1 . . . . . .

. . . . . . . .
.
Vv14g1202 . . Gorai.001G269900.1 .
Gorai.006G040600.1 Gorai.012G087600.1 . . .
. . . . . . . .
. . .
Vv14g1203 . . . . . . .
. . . . . . . .
. . . . .
Vv14g1204 . Gorai.011G091900.1 Gorai.001G269600.1
. . . . . . . .
. . . . . . . .
.
Vv14g1205 Tc04_g015880 . . . . .
. . . . . . . .
. . . . . .
Vv14g1206 Tc04_g015760 . Gorai.001G269500.1 .
. Gorai.012G087800.1 . . . .
. . . . . . . .
. .
Vv14g1207 Tc04_g002990 . Gorai.001G269400.1 .
. Gorai.012G088300.1 Vv17g0150 . .
. . . . . . . .
. . .
Vv14g1208 Tc04_g002980 Gorai.011G092300.1 Gorai.
001G269300.1 . . . . . . .
. . . Vv1rg0244 . . .
. . .
Vv14g1209 Tc04_g002970 . Gorai.001G269200.1 .
. Gorai.012G088400.1 . . . .
. . . . . . . .
. .
Vv14g1210 Tc04_g002950 . . . .
Gorai.012G088600.1 . . . . . .
. . . . . . . .
Vv14g1211 . . . . . . .
. . . . . . . .
. . . . .
Vv14g1212 Tc04_g002940 . . . Gorai.
006G040200.1 Gorai.012G088700.1 . . . .
. . . . . . . .
. .
Vv14g1213 . . . . . . .
. . . . . . . .
. . . . .
Vv14g1214 . . . . . . .
. . . . . . . .
. . . . .
Vv14g1215 . . . . . . .
. . . . . . . .

. . . . .
Vv14g1216 . . . . . . .
. . . . . . . .
. . . . .
Vv14g1217 Tc04_g002920 . . . . .
. . . . . . . .
. . . . . .
Vv14g1218 Tc04_g002880 . . . Gorai.
006G040100.1 Gorai.012G088800.1 . . . .
. . . . . . . .
. .
Vv14g1219 Tc04_g002870 Gorai.011G092400.1 . .
Gorai.006G040000.1 Gorai.012G088900.1 . .
. . . . . . . .
. . . .
Vv14g1220 Tc04_g002860 . . . Gorai.
006G039900.1 . . . . . . .
. . . . . . . .
Vv14g1221 Tc04_g002850 . . . . .
. . . . . . .
Vv1rg0253 . . . . . .
Vv14g1222 . . . . . .
Vv17g0163 Tc03_g014500 Gorai.001G060700.1 . .
Gorai.008G202600.1 Gorai.006G271000.1 . .
. . . . .
Vv14g1223 Tc04_g002840 . . Gorai.007G073800.1
. Gorai.012G089000.1 . . . .
. . . . . . . .
. .
Vv14g1224 Tc04_g002830 . . . Gorai.
006G039800.1 Gorai.012G089100.1 . . . .
. . . . . . . .
. .
Vv14g1225 Tc04_g002820 Gorai.011G092500.1 . .
Gorai.006G039700.1 . . . . .
. . . . . . . .
. .
Vv14g1226 . . . . . . .
. . . . . . . .
. . . . .
Vv14g1227 Tc04_g002810 Gorai.011G092600.1 . .
Gorai.006G039500.1 . Vv17g0167
Tc03_g014450 Gorai.001G061000.1 . . .
Gorai.006G268000.1 . . . . . .
.
Vv14g1228 . . . . . . .
. . . . . . . .
. . . . .
Vv14g1229 . . . . . . .
. . . . . . . .

. . . . .
Vv14g1230 Tc04_g002790 . Gorai.001G268800.1 .
Gorai.006G039400.1 . Vv17g0168
Tc03_g014440 . . . . . . .
. . . . .
Vv14g1231 Tc04_g002780 . . . Gorai.
006G039300.1 . Vv1rg0258 . . . .
. . . . . . . .
.
Vv14g1232 Tc04_g002770 . Gorai.001G268600.1 .
Gorai.006G039200.1 Gorai.012G089200.1 Vv1rg0261
. . . . . . . .
. . . . .
Vv14g1233 . . . . . . .
. . . . . . . .
. . . . .
Vv14g1234 Tc04_g002750 . . Gorai.007G073900.1
. Gorai.012G089300.1 . . . .
. . . . . . . .
. .
Vv14g1235 . . . . . . .
. . . . . . . .
. . . . .
Vv14g1236 Tc04_g002710 Gorai.011G092900.1 . .
Gorai.006G039000.1 Gorai.012G067800.1 . .
. . . . . . . .
. . . .
Vv14g1237 Tc04_g002700 Gorai.011G088800.1 Gorai.
001G268500.1 Gorai.007G074000.1 Gorai.006G038900.1
Gorai.012G089500.1 Vv17g1164 Tc03_g014400 . .
Gorai.007G041900.1 . . Vv1rg0282 .
. . . . .
Vv14g1238 Tc04_g002690 . . . . .
Vv17g1162 . . . . . .
Vv1rg0286 . . . . . .
Vv14g1239 . . . . Gorai.006G038800.1
. . . . . . . .
. . . . . . .
Vv14g1240 Tc04_g002680 Gorai.011G085900.1 Gorai.
001G268400.1 . . Gorai.012G089600.1 . .
. . . . . Vv1rg0289 .
. . . . .
Vv14g1241 Tc04_g002670 . Gorai.001G268300.1 .
Gorai.006G038700.1 Gorai.012G089700.1 . .
. . . . . . . .
. . . .
Vv14g1242 Tc04_g002660 Gorai.011G093000.1 . .
. Gorai.012G089800.1 . . . .
. . . . . . . .
. .

Vv14g1243	Tc04_g002650	Gorai.011G093100.1	.
Gorai.007G074100.1	Gorai.006G038600.1	.	.	.
.	.	.	.	.	.	.	.
.	.	.	.
Vv14g1244	Tc04_g002640	.	Gorai.001G268200.1
Gorai.007G074200.1	Gorai.006G038500.1	.	.	.
.	.	.	.	.	Vv1rg0292	.
.	.	.	.	.
Vv14g1245	Tc04_g002630	Gorai.011G093200.1	Gorai.
001G268100.1	Gorai.007G074300.1	Gorai.006G038400.1	.
.	.	.	.	.	.	.
Vv1rg0301	.	.	.	.	.	.
Vv14g1246	Tc04_g002610	.	.	Gorai.007G074400.1
Gorai.006G037700.1	.	.	.	.	.
.	.	.	.	.	.	.	.
.	.
Vv14g1247	.	.	.	.	Gorai.006G037600.1
.	.	.	.	.	.	.	.
.	.	.	.	.	.	.
Vv14g1248	.	.	.	.	.	.	.
.	.	.	.	.	.	.	.
.	.	.	.	.
Vv14g1249	.	.	Gorai.001G267900.1	.	.
.	.	.	.	.	.	.	.
.	.	.	.	.	.	.
Vv14g1250	.	.	.	.	.	Gorai.
012G090700.1	Vv17g1141	Tc03_g014190	Gorai.001G061600.1
.	.	.	.	.	.	.	.
.	.	.
Vv14g1251	.	.	.	.	.	.	.
.	.	.	.	.	.	.	.
.	.	.	.	.
Vv14g1252	.	.	.	.	.	.	.
.	.	.	.	.	.	.	.
.	.	.	.	.
Vv14g1253	.	.	Gorai.001G267700.1	.
Gorai.006G037200.1	.	.	.	.	.	.
.	.	.	.	.	.	.	.
.
Vv14g1254	Tc04_g002530	.	Gorai.001G267600.1
Gorai.007G074700.1	.	.	.	.	.	.
.	.	.	Vv1rg0302	.	.	.
.	.	.
Vv14g1255	.	.	.	.	Gorai.006G037100.1
.	.	.	.	.	.	.	.
.	.	.	.	.	.	.
Vv14g1256	.	.	.	Gorai.007G074900.1
Gorai.006G037000.1	Gorai.012G091100.1	.	.	.
.	.	.	.	.	.	.	.
.	.	.

Vv14g1257 . . . . . . .
. . . . . . . .
. . . . .
Vv14g1258 . . . Gorai.007G075000.1 .
. . . . . . . .
. . . . . . .
Vv14g1259 . . . . . Gorai.
012G091200.1 . . . . . . .
. . . . . . .
Vv14g1260 . . Gorai.001G267500.1 . .
Gorai.012G091400.1 . . . . .
. . . . . . . .
.
Vv14g1261 . . . . . .
Vv17g1118 Tc03_g014120 . . . Gorai.
008G204500.1 Gorai.006G270200.1 . . . .
. . .
Vv14g1262 . . . . Gorai.006G036900.1
. Vv17g1117 Tc03_g014110 Gorai.001G061800.1
. Gorai.007G041600.1 Gorai.008G204400.1 .
. . . . . . .
Vv14g1263 . . . . . . .
. . . . . . . .
. . . . .
Vv14g1264 Tc04_g002380 . . . Gorai.
006G036800.1 Gorai.012G091500.1 . . . .
. . . . . . . .
. .
Vv14g1265 Tc04_g002370 . . . . .
. . . . . . . .
. . . . . .
Vv14g1266 Tc04_g002360 . Gorai.001G267400.1 .
Gorai.006G036700.1 . . . . .
. . . . . . . .
. .
Vv14g1267 Tc04_g002330 . . . Gorai.
006G036400.1 . . . . . . .
. . . . . . . .
Vv14g1268 Tc04_g002320 . Gorai.001G267300.1 .
Gorai.006G036300.1 . Vv17g1097 . .
. . . . Vv01g0714
Tc02_g025720 Gorai.002G189900.1 Gorai.005G174600.1 .
Gorai.008G162500.1 .
Vv14g1269 Tc04_g002240 . Gorai.001G267200.1 .
. . . . . . . .
. . . . . . . .
Vv14g1270 . . . . . . .
. . . . . . . .
. . . . .
Vv14g1271 . . . . . . .

. . . . . . Vv01g0725
Tc02_g025570 . Gorai.005G175000.1 .
Gorai.008G162000.1 .
Vv14g1272 . . . . . . .
. . . . . . . .
. . . . .
Vv14g1273 Tc04_g002230 . . . Gorai.
006G036200.1 Gorai.012G066600.1 . . . .
. . . . . . . .
. .
Vv14g1274 Tc04_g002220 . . . .
Gorai.012G066500.1 Vv17g1087 Tc03_g013920 Gorai.
001G062700.1 . . . Gorai.006G269800.1 .
. . . . . .
Vv14g1275 . . . . . . .
. . . . . . . .
. . . . .
Vv14g1276 . Gorai.011G093700.1 . . .
. Vv17g1083 Tc03_g013840 . .
Gorai.007G041500.1 . . Vv01g0730
Tc02_g025410 . Gorai.005G183600.1 . . .
Vv14g1277 . . . . . Gorai.
012G066400.1 . . . . . . .
. . . . . . .
Vv14g1278 . . . . . . .
. . . . . . . .
. . . . .
Vv14g1279 . . . . . . .
. . . . . . Vv01g0731
Tc02_g025400 . Gorai.005G175200.1 . .
.
Vv14g1280 . . . . . . .
. . . . . . . .
. . . . .
Vv14g1281 . . . . . Gorai.
012G066300.1 . . . . . . .
Vv01g0732 . . Gorai.005G175300.1 .
. .
Vv14g1282 . . . . . . .
. . . . . . . .
. . . . .
Vv14g1283 . . . . . . .
. . . . . . . .
. . . . .
Vv14g1284 . . . . . . .
. . . . . . . .
. . . . .
Vv14g1285 . . . . . Gorai.
012G066200.1 . . . . . . .
. . . . . . .

Vv14g1286 Tc04_g002200 . . . . .
. . . . . . . .
. . . . . .
Vv14g1287 . . . . . . .
. . . . . . . .
. . . . .
Vv14g1288 . . . . . . .
. . . . . . . .
. . . . .
Vv14g1289 Tc04_g002190 . . . . .
. . . . . . . .
. . . . . .
Vv14g1290 . . . . . . .
. . . . . . . .
. . . . .
Vv14g1291 Tc04_g002180 . . . .
Gorai.012G066100.1 . . . . . .
. . . . . . . .
Vv14g1292 Tc04_g002170 . . . .
Gorai.012G066000.1 . . . . . .
. . . . . . . .
Vv14g1293 Tc04_g002150 . . . .
Gorai.012G065900.1 . . . . . .
. . . . . . . .
Vv14g1294 Tc04_g002130 Gorai.011G093900.1 Gorai.
001G267000.1 . Gorai.006G035100.1 . . .
. . . . . . . .
. . . .
Vv14g1295 Tc04_g002100 Gorai.011G094000.1 . .
Gorai.006G035000.1 . . . . .
. . . . . . . .
. .
Vv14g1296 . . Gorai.001G266500.1 Gorai.
007G075200.1 . . . . . . .
. . . . . . . .
.
Vv14g1297 . . . . . . .
. . . . . . . .
. . . . .
Vv14g1298 . . . . . .
Vv17g1043 Tc03_g002290 . . . . .
. . . . . . .
Vv14g1299 . . . . . . .
. . . . . . . .
. . . . .
Vv14g1300 Tc04_g002020 . . . . .
. . . . . . . .
. . . . . .
Vv14g1301 Tc04_g002010 . . . . .
. . . . . . . .

. . . . . .
Vv14g1302 . Gorai.011G094100.1 . . .
. . . . . . . .
. . . . . . .
Vv14g1303 . . . . . . .
. . . . . . . .
. . . . .
Vv14g1304 . . . . . Gorai.
012G065600.1 . . . . . . .
. . . . . . .
Vv14g1305 . . . . . . .
. . . . . . . .
. . . . .
Vv14g1306 . . . . Gorai.006G034900.1
. . . . . . . .
Vv01g0737 Tc02_g025310 . Gorai.005G175700.1
Gorai.009G335200.1 . .
Vv14g1307 . . . . . . .
. . . . . . . .
. . . . .
Vv14g1308 Tc04_g001980 Gorai.011G094500.1 Gorai.
001G266300.1 . . Gorai.012G065300.1 . .
. . . . . Vv01g0774
Tc02_g024980 . Gorai.005G177400.1 . Gorai.
008G161300.1 .
Vv14g1309 Tc04_g001970 . . . .
Gorai.012G065200.1 . . . . . .
. . . . . . . .
Vv14g1310 . . . . . . .
. . . . . . . .
. . . . .
Vv14g1311 Tc04_g001960 . . . Gorai.
006G034800.1 . . . . . . .
. . . . . . . .
Vv14g1312 Tc04_g001940 . . . Gorai.
006G034700.1 Gorai.012G065000.1 . . . .
. . . Vv01g0777 Tc02_g024950
Gorai.002G187800.1 . Gorai.009G335700.1 Gorai.
008G161200.1 .
Vv14g1313 Tc04_g001930 . . . . .
. . . . . . . .
. . . . . .
Vv14g1314 . . . . . Gorai.
012G064900.1 . . . . . . .
. . . . . . .
Vv14g1315 Tc04_g001920 . . Gorai.007G075300.1
Gorai.006G034600.1 . . . . .
. . . . . . . .
. .
Vv14g1316 Tc04_g001910 . Gorai.001G266100.1 .

Gorai.006G034500.1 Gorai.012G064800.1 . .
. . . . . . . .
. . . .
Vv14g1317 . . . . . . .
. . . . . . . .
. . . . .
Vv14g1318 Tc04_g001900 . . . .
Gorai.012G064700.1 . . . . . .
. . . . . . . .
Vv14g1319 . . Gorai.001G266000.1 . .
. . . . . . . .
. . . . . . .
Vv14g1320 Tc04_g001880 . . . .
Gorai.012G064600.1 . . . . . .
. . . . . . . .
Vv14g1321 . . . . . . .
. . . . . . . .
. . . . .
Vv14g1322 . . . . . . .
. . . . . . . .
. . . . .
Vv14g1323 Tc04_g001790 . . . . .
. . . . . . . .
. . . . . .
Vv14g1324 Tc04_g001770 . Gorai.001G265900.1 .
. . . . . . . .
. . . . . . . .
Vv14g1325 Tc04_g001760 . Gorai.001G265500.1 .
. . . . . . . .
. . . . . . . .
Vv14g1326 Tc04_g001750 . . . Gorai.
006G034300.1 Gorai.012G064500.1 . . . .
. . . . . . . .
. .
Vv14g1327 Tc04_g001740 Gorai.011G094600.1 .
Gorai.007G075400.1 Gorai.006G034200.1 Gorai.012G064400.1
. . . . . . . .
. . . . . .
Vv14g1328 Tc04_g001730 . Gorai.001G265400.1 .
. . . . . . . .
. . . . . . . .
Vv14g1329 Tc04_g001720 . . . Gorai.
006G034100.1 . . . . . . .
. . . . . . . .
Vv14g1330 Tc04_g001700 . . . Gorai.
006G033800.1 . . . . . . .
. . . . . . . .
Vv14g1331 Tc04_g001680 . . . . .
. . . . . . . .
. . . . . .

Vv14g1332 . . . . Gorai.006G033600.1
. . . . . . . .
. . . . . . .
Vv14g1333 . . . . . .
Vv17g1017 . . . . . .
. . . . . .
Vv14g1334 Tc04_g001670 . . . .
Gorai.012G064300.1 . . . . . .
. . . . . . . .
Vv14g1335 Tc04_g001660 . Gorai.001G265000.1 .
. Gorai.012G064200.1 . . . .
. . . . . . . .
. .
Vv14g1336 Tc04_g001650 Gorai.011G094700.1 . .
Gorai.006G033300.1 Gorai.012G064100.1 . .
. . . . . . . .
. . . .
Vv14g1337 . . . . . . .
. . . . . . . .
. . . . .
Vv14g1338 Tc04_g001640 . Gorai.001G264900.1 .
. Gorai.012G064000.1 . . . .
. . . . . . . .
. .
Vv14g1339 Tc04_g001620 . . . . .
. . . . . . . .
. . . . . .
Vv14g1340 Tc04_g001610 . Gorai.001G264700.1 .
. . . . . . . .
. . . . . . . .
Vv14g1341 Tc04_g001590 . Gorai.001G264600.1
Gorai.007G075500.1 . . . . . .
. . . . . . . .
. .
Vv14g1342 Tc04_g001580 . . Gorai.007G075600.1
. . . . . . . .
. . . . . . . .
Vv14g1343 . . . . . . .
. . . . . . . .
. . . . .
Vv14g1344 Tc04_g001570 . . Gorai.007G075700.1
Gorai.006G033100.1 Gorai.012G063800.1 Vv17g1007
Tc03_g001980 . . . . . .
. . . . . .
Vv14g1345 Tc04_g001560 Gorai.011G094900.1 Gorai.
001G264500.1 . Gorai.006G033000.1 . . .
. . . . . . . .
. . . .
Vv14g1346 Tc04_g001550 Gorai.011G095000.1 . .
. . . . . . . .

. . . . . . . .
Vv14g1347 Tc04_g001540 . . . . .
. . . . . . . .
. . . . . .
Vv14g1348 . . . . . . .
. . . . . . . .
. . . . .
Vv14g1349 . . . . . . .
. . . . . . . .
. . . . .
Vv14g1350 Tc04_g001520 . . . . .
. . . . . . . .
. . . . . .
Vv14g1351 . . Gorai.001G264400.1 Gorai.
007G075900.1 Gorai.006G032800.1 Gorai.012G063700.1 .
. . . . . . . .
. . . . .
Vv14g1352 . . . . . . .
. . . . . . . .
. . . . .
Vv14g1353 Tc04_g001510 . . Gorai.007G076000.1
. Gorai.012G063600.1 . . . .
. . . . . . . .
. .
Vv14g1354 Tc04_g001490 . . . .
Gorai.012G063500.1 Vv17g1003 . . . .
. . . . . . . .
.
Vv14g1355 Tc04_g001480 . Gorai.001G264300.1 .
. . . . . . . .
. . . . . . . .
Vv14g1356 Tc04_g001470 . Gorai.001G264200.1 .
. . . . . . . .
. . . . . . . .
Vv14g1357 . . . . . . .
. . . . . . . .
. . . . .
Vv14g1358 Tc04_g001440 . Gorai.001G264100.1 .
. . . . . . . .
. . . . . . . .
Vv14g1359 Tc04_g001430 . Gorai.001G264000.1 .
Gorai.006G032200.1 . . . . .
. . . . . . . .
. .
Vv14g1360 . . . . . . .
. . . . . . . .
. . . . .
Vv14g1361 Tc04_g001420 . . . . .
Vv17g0978 Tc03_g001550 . . . .
. . . . . . . .

Vv14g1362 Tc04_g001390 . . . .
Gorai.012G063400.1 . . . . . .
. . . . . . . .
Vv14g1363 Tc04_g001380 Gorai.011G095200.1 . .
. . . . . . . .
. . . . . . . .
Vv14g1364 Tc04_g001370 Gorai.011G095300.1 Gorai.
001G263700.1 . . . . . . .
. . . . . . . .
. .
Vv14g1365 . . . . Gorai.006G032100.1
. . . . . . . .
. . . . . . .
Vv14g1366 . . . . . . .
. . . . . . . .
. . . . .
Vv14g1367 Tc04_g001360 Gorai.011G095400.1 . .
. . . . . . . .
. . . . . . . .
Vv14g1368 . . . . . . .
. . . . . . . .
. . . . .
Vv14g1369 . . . . Gorai.006G032000.1
. . . . . . . .
. . . . . . .
Vv14g1370 Tc04_g001350 . . . Gorai.
006G031800.1 . Vv17g0967 Tc03_g001450 . .
. . . . . . . .
. .
Vv14g1371 . . . . . .
Vv17g0966 Tc03_g001440 . . . . .
. . . . . . .
Vv14g1372 Tc04_g001340 . . . . .
Vv17g0960 Tc03_g001390 . . . .
. . . . . . . .
Vv14g1373 Tc04_g001320 . . . Gorai.
006G031600.1 . . . . . . .
. . . . . . . .
Vv14g1374 Tc04_g001310 . . . .
Gorai.012G063100.1 Vv17g0955 Tc03_g001360 . .
. . . . . . . .
. .
Vv14g1375 . . . . . .
Vv17g0953 . Gorai.001G071400.1 . . .
. . . . . . . .
Vv14g1376 Tc04_g001170 . Gorai.001G263600.1 .
. . . . . . . .
. . . . . . . .
Vv14g1377 Tc04_g001160 . Gorai.001G263500.1 .
Gorai.006G031200.1 Gorai.012G063000.1 . .

. . . . . . . .
. . . .
Vv14g1378 . . . . . . .
. . . . . . . .
. . . . .
Vv14g1379 Tc04_g001150 . . . Gorai.
006G031000.1 Gorai.012G062900.1 . . . .
. . . . . . . .
. .
Vv14g1380 Tc04_g001140 . . . . .
. . . . . . . .
. . . . . .
Vv14g1381 Tc04_g001130 . Gorai.001G263400.1 .
. . . . . . . .
. . . . . . . .
Vv14g1382 Tc04_g001120 Gorai.011G095500.1 . .
. . . . . . . .
. . . . . . . .
Vv14g1383 . . . . . . .
. . . . . . . .
. . . . .
Vv14g1384 Tc04_g001110 . Gorai.001G263300.1 .
. Gorai.012G062700.1 . . . .
. . . . . . . .
. .
Vv14g1385 . . . . . . .
. . . . . . . .
. . . . .
Vv14g1386 Tc04_g001100 . Gorai.001G263200.1 .
Gorai.006G030800.1 . . . . .
. . . . . . . .
. .
Vv14g1387 Tc04_g001090 Gorai.011G095600.1 . .
Gorai.006G030700.1 . . . . .
. . . . . . . .
. .
Vv14g1388 Tc04_g001080 . Gorai.001G263100.1 .
. Gorai.012G062600.1 Vv17g0943
Tc03_g001290 . . . . . . .
. . . . .
Vv14g1389 . . . . . . .
. . . . . . . .
. . . . .
Vv14g1390 Tc04_g001070 . . . .
Gorai.012G062400.1 . . . . . .
. . . . . . . .
Vv14g1391 Tc04_g001060 . Gorai.001G263000.1 .
. Gorai.012G062300.1 . . . .
. . . . . . . .
. .

Vv14g1392 Tc04_g001050 . . Gorai.007G076200.1
Gorai.006G030600.1 . . . . .
. . . . . . . .
. .
Vv14g1393 Tc04_g001040 . . . Gorai.
006G030500.1 Gorai.012G062200.1 . . . .
. . . . . . . .
. .
Vv14g1394 Tc04_g001030 . Gorai.001G262800.1 .
. . . . . . . .
. . . . . . . .
Vv14g1395 Tc04_g001020 Gorai.011G095700.1 .
Gorai.007G076300.1 Gorai.006G030400.1 . . .
. . . . . . . .
. . . .
Vv14g1396 Tc04_g001010 . Gorai.001G262700.1 .
. Gorai.012G062000.1 . . . .
. . . . . . . .
. .
Vv14g1397 Tc04_g001000 Gorai.011G095800.1 Gorai.
001G262600.1 . . . Vv17g0931 . .
. . . . . . . .
. . .
Vv14g1398 Tc04_g000990 . Gorai.001G262500.1 .
. . . . . . . .
. Vv01g0753 Tc02_g025190 Gorai.002G188400.1
Gorai.005G176500.1 . . .
Vv14g1399 Tc04_g000980 . Gorai.001G262400.1 .
Gorai.006G030300.1 Gorai.012G061900.1 . .
. . . . . . . .
. . . .
Vv14g1400 Tc04_g000970 . . . .
Gorai.012G061800.1 . . . . . .
. . . . . . . .
Vv14g1401 Tc04_g000950 . Gorai.001G262300.1 .
. . . . . . . .
. . . . . . . .
Vv14g1402 . . . . . . .
. . . . . . . .
. . . . .
Vv14g1403 Tc04_g000940 . . . . .
. . . . . . . .
. . . . . .
Vv14g1404 . . Gorai.001G262200.1 . .
. . . . . . . .
. . . . . . .
Vv14g1405 Tc04_g000930 . Gorai.001G262100.1 .
. Gorai.012G061700.1 . . . .
. . . . . . . .
. .

Vv14g1406 . . . . . . .
. . . . . . . .
. . . . .
Vv14g1407 Tc04_g000920 . . . Gorai.
006G030200.1 Gorai.012G061500.1 Vv17g0922
Tc03_g001070 . . . . . . .
. . . . .
Vv14g1408 . . . . . . .
. . . . . . . .
. . . . .
Vv14g1409 Tc04_g000910 . Gorai.001G262000.1 .
Gorai.006G030100.1 . . . . .
. . . . . . . .
. .
Vv14g1410 Tc04_g000880 . Gorai.001G261500.1 .
. . Vv17g0916 Tc03_g001050 . .
. . . . . . . .
. .
Vv14g1411 Tc04_g000870 . . . Gorai.
006G029800.1 . . . . . . .
. . . . . . . .
Vv14g1412 Tc04_g000850 . Gorai.001G261400.1 .
. . . . . . . .
. . . . . . . .
Vv14g1413 . . . . . . .
. . . . . . . .
. . . . .
Vv14g1414 . . . . . . .
. . . . . . . .
. . . . .
Vv14g1415 . . . . . . .
. . . . . . . .
. . . . .
Vv14g1416 Tc04_g000840 . . Gorai.007G076500.1
. . . . . . . .
. . . . . . . .
Vv14g1417 . . . . . . .
. . . . . . . .
. . . . .
Vv14g1418 Tc04_g000830 . . . . .
. . . . . . .
Vv01g0785 Tc02_g023410 . Gorai.005G182900.1 .
. .
Vv14g1419 Tc04_g000810 . Gorai.001G261300.1
Gorai.007G076700.1 . Gorai.012G061300.1 . .
. . . . . . . .
. . . .
Vv14g1420 Tc04_g000800 . Gorai.001G261200.1 .
Gorai.006G029700.1 . . . . .
. . . . . . . .

.        .
Vv14g1421        Tc04_g000790      .        .        .        .
Gorai.012G061200.1        Vv17g0904        Tc03_g000950      Gorai.
001G068400.1      .        .        .        .        .        .        .
.        .        .        .
Vv14g1422        .        .        .        .        .        .        .
.        .        .        .        .        .        .        .
.        .        .        .        .
Vv14g1423        .        .        .        .        .        .        .
.        .        .        .        .        .        .        .
.        .        .        .        .
Vv14g1424        Tc04_g000770      Gorai.011G095900.1        .        .
.        .        .        .        .        .        .        .
.        .        .        .        .        .        .        .
Vv14g1425        Tc04_g000760      .        .        .        Gorai.
006G029600.1      .        .        .        .        .        .        .
.        Vv01g0791        Tc02_g000580      .        .        .
.        .
Vv14g1426        .        .        .        .        .        .        .
.        .        .        .        .        .        .        .
.        .        .        .        .
Vv14g1427        Tc04_g000750      .        .        .        Gorai.
006G029400.1      Gorai.012G061000.1        .        .        .        .
.        .        .        .        .        .        .        .
.        .
Vv14g1428        .        .        .        .        .        .        .
.        .        .        .        .        .        .        .
.        .        .        .        .
Vv14g1429        .        .        .        .        .        .        .
.        .        .        .        .        .        .        .
.        .        .        .        .
Vv14g1430        .        .        .        .        .        .        .
.        .        .        .        .        .        .        .
.        .        .        .        .
Vv14g1431        Tc04_g000730      .        .        .        .        .
.        .        .        .        .        .        .        .
.        .        .        .        .        .
Vv14g1432        .        .        .        .        .        .        .
.        .        .        .        .        .        .        .
.        .        .        .        .
Vv14g1433        .        .        .        .        .        .
Vv17g0898        .        .        .        .        .        .        .
.        .        .        .        .        .
Vv14g1434        Tc04_g000720      .        .        .        Gorai.
006G029200.1      .        .        .        .        .        .        .
.        .        .        .        .        .        .        .
Vv14g1435        Tc04_g000710      .        Gorai.001G261100.1        .
.        .        Vv17g0896        Tc03_g000920      .        .
.        Gorai.008G158500.1        .        Vv01g0800        .
.        .        .        Gorai.008G155700.1        .

Vv14g1436 Tc04_g000700 . . . .
Gorai.012G060800.1 Vv17g0895 . . . .
. Gorai.006G187100.1 . . . .
. . .
Vv14g1437 . . . . . . .
. . . . . . . .
. . . . .
Vv14g1438 Tc04_g000680 . . . .
Gorai.012G060600.1 . . . . . .
. . . . . . . .
Vv14g1439 Tc04_g000670 . . . Gorai.
006G029100.1 Gorai.012G060500.1 . . . .
. . . Vv01g0805 Tc02_g000600
Gorai.002G213100.1 . . . Gorai.012G171200.1
Vv14g1440 Tc04_g000660 . Gorai.001G260900.1 .
. . . . . . . .
. Vv01g0814 . . . Gorai.
009G343600.1 . Gorai.012G171100.1
Vv14g1441 Tc04_g000630 . . Gorai.007G076900.1
. . Vv17g0889 Tc03_g000800 . .
. . . . . . . .
. .
Vv14g1442 . . . . . . .
. . . . . . . .
. . . . .
Vv14g1443 Tc04_g000610 . Gorai.001G260600.1 .
. . . . . . . .
. . . . . . . .
Vv14g1444 . . . . . . .
. . . . . . . .
. . . . .
Vv14g1445 Tc04_g000590 . Gorai.001G260400.1
Gorai.007G077000.1 . Gorai.012G060200.1 . .
. . . . . . . .
. . . .
Vv14g1446 Tc04_g000550 . . . .
Gorai.012G060000.1 Vv17g0879 Tc03_g000710 . .
. . Gorai.006G188000.1 Vv01g0821
Tc02_g023300 . Gorai.005G184300.1 . . .
Vv14g1447 Tc04_g000520 . . . .
Gorai.012G059900.1 Vv17g0877 Tc03_g000690 . .
. . . Vv01g0832 Tc02_g023290 .
Gorai.005G184400.1 . . .
Vv14g1448 Tc04_g000500 . . Gorai.007G077100.1
. . Vv17g0876 Tc03_g000680 Gorai.
001G070400.1 . . . . . . .
. . . .
Vv14g1449 Tc04_g000490 . . . .
Gorai.012G059800.1 . . . . . .
. . . . . . . .

Vv14g1450 . . . . . . .
. . . . . . . .
. . . . .
Vv14g1451 . . . . . . .
. . . . . . . .
. . . . .
Vv14g1452 Tc04_g000480 . . . .
Gorai.012G059700.1 . . . . . .
. . . . . . . .
Vv14g1453 Tc04_g000460 Gorai.011G096000.1 . .
Gorai.006G028500.1 Gorai.012G059600.1 . .
. . . . . . . .
. . . .
Vv14g1454 Tc04_g000450 . . Gorai.007G077200.1
. Gorai.012G059500.1 . . . .
. . . . . . . .
. .
Vv14g1455 . . . . . .
Vv17g0873 Tc03_g000650 . . . .
Gorai.006G187700.1 . . . . . .
.
Vv14g1456 . . . . . . .
. . . . . . . .
. . . . .
Vv14g1457 Tc04_g000420 . . . Gorai.
006G028400.1 . . . . . . .
. . . . . . . .
Vv14g1458 . . . . . . .
. . . . . . . .
. . . . .
Vv14g1459 . . . . . . .
. . . . . . . .
. . . . .
Vv14g1460 . . . . . . .
. . . . . . . .
. . . . .
Vv14g1461 Tc04_g000400 . Gorai.001G259900.1 .
. . . . . . . .
. . . . . . . .
Vv14g1462 Tc04_g000390 . . Gorai.007G077300.1
. . . . . . . .
. Vv01g0850 Tc02_g023050 . Gorai.
005G185700.1 . . .
Vv14g1463 Tc04_g000380 . Gorai.001G259800.1 .
. . . . . . . .
. . . . . . . .
Vv14g1464 . . . . . . .
. . . . . . . .
. . . . .
Vv14g1465 . . Gorai.001G259700.1 . .

. Vv17g0862 . . . . .
Gorai.006G187800.1 . . . . .
. .
Vv14g1466 Tc04_g000360 . Gorai.001G259600.1 .
. . . . . . . .
. . . . . . . .
Vv14g1467 . . . . . .
Vv17g0841 Tc03_g000280 . . . . .
. . . . . . .
Vv14g1468 . . . . . . .
. . . . . . . .
. . . . .
Vv14g1469 . . . . . . .
. . . . . . . .
. . . . .
Vv14g1470 . . Gorai.001G259500.1 . .
. Vv17g0828 . . . . .
. . . . . . . .
Vv14g1471 Tc04_g000350 . . . . .
Vv17g0825 . . . . . .
. . . . . . .
Vv14g1472 . . . . . .
Vv17g0823 . . . . . . .
. . . . . .
Vv14g1473 Tc04_g000340 . Gorai.001G259400.1 .
. Gorai.012G059300.1 . . . .
. . . . . . . .
. .
Vv14g1474 . . . . . . .
. . . . . . . .
. . . . .
Vv14g1475 Tc04_g000330 . Gorai.001G259300.1 .
. . . . . . . .
. . . . . . . .
Vv14g1476 Tc04_g000320 . Gorai.001G259200.1 .
. . . . . . . .
. . . . . . . .
Vv14g1477 Tc04_g000310 . . . Gorai.
006G028100.1 . . . . . . .
. Vv01g0868 . . . . .
Gorai.012G123100.1
Vv14g1478 Tc04_g000300 . Gorai.001G259100.1 .
Gorai.006G028000.1 . . . . .
. . . . . . . .
. .
Vv14g1479 Tc04_g000290 . . . Gorai.
006G027700.1 . . . . . . .
. . . . . . . .
Vv14g1480 Tc04_g000280 . Gorai.001G259000.1 .
. Gorai.012G059200.1 . . . .

. . . . . . . .
. .
Vv14g1481 . . . . . Gorai.
012G058700.1 . . . . . . .
Vv01g0871 . . . Gorai.009G345000.1
. .
Vv14g1482 . . . . . . .
. . . . . . . .
. . . . .
Vv14g1483 Tc04_g015390 . Gorai.001G258900.1 .
. . . . . . . .
. . . . . . . .
Vv14g1484 Tc04_g015410 . . . . .
. . . . . . . .
. . . . . .
Vv14g1485 . . . . . . .
. . . . . . . .
. . . . .
Vv14g1486 . . Gorai.001G258800.1 . .
. . . . . . . .
. . . . . . .
Vv14g1487 Tc04_g007060 . . . . .
. . . . . . . .
. . . . . .
Vv14g1488 . . . . . . .
. . . . . . . .
. . . . .
Vv14g1489 . . . . . . .
. . . . . . . .
. . . . .
Vv14g1490 Tc04_g007120 . . . . .
. . . . . . . .
. . . . . .
Vv14g1491 . . . . . . .
. . . . . . . .
. . . . .
Vv14g1492 Tc04_g007210 . . . . .
. . . . . . . .
. . . . . .
Vv14g1493 . . . . . . .
. . . . . . . .
. . . . .
Vv14g1494 . . Gorai.001G258100.1 . .
. . . . . . . .
. . . . . . .
Vv14g1495 Tc04_g015620 . Gorai.001G257600.1
Gorai.007G201000.1 . . . . . .
. . . . . . . .
. .
Vv14g1496 . . . . . . .

. . . . . . . .
. . . . .
Vv14g1497 . . . . . . .
. . . . . . . .
. . . . .
Vv14g1498 . . . Gorai.007G078200.1 .
. . . . . . . .
. . . . . . .
Vv14g1499 . . . Gorai.007G078100.1 .
. . . . . . . .
. . . . . . .
Vv14g1500 Tc04_g000970 . . . . .
. . . . . . . .
. . . . . .
Vv14g1501 . . . Gorai.007G078000.1 .
. . . . . . . .
. . . . . . .
Vv14g1502 . . . . . . .
. . . . . . . .
. . . . .
Vv14g1503 . . . . . . .
. . . . . . . .
. . . . .
Vv14g1504 . . . . . .
Vv17g0576 . . . . Gorai.008G189700.1
. Vv01g0892 . . . Gorai.
009G346100.1 Gorai.008G189700.1 .
Vv14g1505 Tc04_g015680 . . . . .
. . . . . . . .
. . . . . .
Vv14g1506 . . . Gorai.007G033900.1 .
. . . . . . . .
. . . . . . .
Vv14g1507 . . . . . . .
. . . . . . . .
. . . . .
Vv14g1508 . . . . . . .
. . . . . . . .
. . . . .
Vv14g1509 . . . . . . .
. . . . . . . .
. . . . .
Vv14g1510 . . . . . . .
. . . . . . . .
. . . . .
Vv14g1511 . . . . . . .
. . . . . . . .
. . . . .
Vv14g1512 . . . . . . .
. . . . . . . .

. . . . .
Vv14g1513 Tc04_g012930 . . . . .
. . . . . . . .
. . . . . .
Vv14g1514 . . . . . . .
. . . . . . . .
. . . . .
Vv14g1515 . . . . . . .
. . . . . . . .
. . . . .
Vv14g1516 Tc04_g012950 . . . . .
. . . . . . . .
. . . . . .
Vv14g1517 . . . . . . .
. . . . . . . .
. . . . .
Vv14g1518 . . . Gorai.007G077800.1
Gorai.006G026900.1 . . . . . .
. . . . . . . .
.
Vv14g1519 . . . . . . .
. . . . . . . .
. . . . .
Vv14g1520 . . . . . . .
. . . . . . . .
. . . . .
Vv14g1521 . . . . . Gorai.
012G058500.1 . . . . . . .
. . . . . . .
Vv14g1522 . . . . . . .
. . . . . . . .
. . . . .
Vv14g1523 . . . Gorai.007G077700.1 .
. . . . . . . .
. . . . . . .
Vv14g1524 . . . . . . .
. . . . . . . .
. . . . .
Vv14g1525 . . . . . . .
. . . . . . . .
. . . . .
Vv14g1526 Tc04_g000710 . . Gorai.007G074700.1
. . . . . . . .
. . . . . . . .
Vv14g1527 . . Gorai.001G257500.1 Gorai.
007G200200.1 Gorai.006G026100.1 . . . .
. . . . . . . .
. . .
Vv14g1528 . . Gorai.001G257400.1 Gorai.
007G200000.1 Gorai.006G026000.1 . . . .

. . . . . . . .
. . .
Vv14g1529 Tc04_g007270 . . . Gorai.
006G025900.1 . . . . . . .
. Vv01g0911 . . . . .
.
Vv14g1530 Tc04_g007280 . . . . .
. . . . . . . .
. . . . . .
Vv14g1531 Tc04_g007300 . . . . .
. . . . . . .
Vv01g0921 Tc02_g022210 . . . . .
Vv14g1532 . . . . . . .
. . . . . . Vv01g0922
Tc02_g022200 Gorai.002G164600.1 Gorai.005G189500.1
. . .
Vv14g1533 . . . . . .
Vv17g0596 . . . . . . .
. . . . . .
Vv14g1534 Tc04_g007880 . . . Gorai.
006G025600.1 . . . . . . .
. . . . . . . .
Vv14g1535 . . . Gorai.007G034900.1 .
. . . . . . . .
. . . . . . .
Vv14g1536 . . . . . . .
. . . . . . . .
. . . . .
Vv14g1537 . . . . . . .
. . . . . . . .
. . . . .
Vv14g1538 Tc04_g025710 . . . . .
. . . . . . . .
. . . . . .
Vv14g1539 . . . . . .
Vv17g0600 . . . . . . .
. . . . . .
Vv14g1540 Tc04_g016040 . . Gorai.007G198800.1
. . . . . . . .
. Vv01g0926 Tc02_g022140 . .
Gorai.009G346700.1 . .
Vv14g1541 Tc04_g013320 . . Gorai.007G196200.1
. . . . . . . .
. . . . . . . .
Vv14g1542 Tc04_g017620 . . . . .
. . . . . . . .
. . . . . .
Vv14g1543 Tc04_g025970 . . . . .
Vv17g0616 Tc03_g009550 . . . .
. Vv01g0940 Tc02_g034120 . . .

.	.
Vv14g1544	.	.	.	.	.	.	.
.	.	.	.	.	.	.	.
.	.	.	.	.
Vv14g1545	.	.	.	.	.	.	.
.	.	.	.	.	.	.	.
.	.	.	.	.
Vv14g1546	.	.	.	.	.	.	.
.	.	.	.	.	.	.	.
.	.	.	.	.
Vv14g1547	.	.	.	.	.	.	.
.	.	.	.	.	.	.	.
.	.	.	.	.
Vv14g1548	.	.	.	.	.	.	.
.	.	.	.	.	.	.	.
.	.	.	.	.
Vv14g1549	.	.	.	.	.	.	.
.	.	.	.	.	.	.	.
.	.	.	.	.
Vv14g1550	Tc04_g016210	.	.	.	.	.
.	.	.	.	.	.	.	.
.	.	.	.	.	.
Vv14g1551	.	.	.	.	.	.
Vv17g0621	Tc03_g009580	.	.	.	Gorai.
008G182700.1	.	Vv01g0951	Tc02_g034040	.	.
.	Gorai.008G190600.1	.
Vv14g1552	.	.	.	.	.	.	.
.	.	.	.	.	.	.	.
.	.	.	.	.
Vv14g1553	.	.	.	.	.	.	.
.	.	.	.	.	.	.	.
.	.	.	.	.
Vv14g1554	.	.	.	.	.	.
Vv17g0625	Tc03_g009670	.	.	.	.	.
Vv01g0953	Tc02_g034020	.	.	.	.
.
Vv14g1555	.	.	.	.	.	.	.
.	.	.	.	.	.	.	.
.	.	.	.	.
Vv14g1556	.	.	.	.	.	.	.
.	.	.	.	.	.	.	.
.	.	.	.	.
Vv14g1557	Tc04_g007900	.	.	.	Gorai.
006G025400.1	.	.	.	.	.	.	.
.	.	.	.	.	.	.	.
Vv14g1558	Tc04_g007930	.	.	.	Gorai.
006G025300.1	.	.	.	.	.	.	.
.	.	.	.	.	.	.	.
Vv14g1559	Tc05_g023660	.	.	.	.	.
Vv17g0630	.	.	.	.	.	.

. . . . . . .
Vv14g1560 . . . . . . .
. . . . . . . .
. . . . .
Vv14g1561 . . . . . . .
. . . . . . . .
. . . . .
Vv14g1562 . . . . . .
Vv17g0634 Tc03_g009820 . . . Gorai.
008G191200.1 . Vv01g0961 Tc02_g033950 Gorai.
013G074800.1 . . Gorai.008G191200.1 Gorai.
012G117800.1
Vv14g1563 . . . . . . .
. . . . . . . .
. . . . .
Vv14g1564 Tc05_g023640 . . . . .
Vv17g0636 . . . . Gorai.
008G182300.1 . Vv01g0964 Tc02_g033920 . .
. Gorai.008G191300.1 .
Vv14g1565 Tc04_g017560 . . . . .
Vv17g0638 Tc03_g009840 . . . .
. Vv01g0966 Tc02_g033900 Gorai.013G082700.1
. Gorai.009G323400.1 . .
Vv14g1566 . . . Gorai.007G035800.1 .
. Vv17g0639 . Gorai.001G072400.1 .
. . . . . . . .
. .
Vv14g1567 . . Gorai.001G257300.1 . .
Gorai.012G057200.1 . . . . .
. . . . . . . .
.
Vv14g1568 . . . . . Gorai.
012G057100.1 . . . . . . .
. . . . . . .
Vv14g1569 . . . . . Gorai.
012G057000.1 . . . . . . .
. . . . . . .
Vv14g1570 . . . . . . .
. . . . . . Vv1rg0202
. . . . . .
Vv14g1571 . . . . . Gorai.
012G056900.1 . . . . . . .
Vv1rg0201 . . . . . .
Vv14g1572 . . . Gorai.007G036000.1 .
. . . . . . . .
. . . . . . .
Vv14g1573 . . . . . . .
. . . . . . . .
. . . . .
Vv14g1574 . . . . . . .

. . . . . . . .
. . . . .
Vv14g1575 . . . . . . .
. . . . . . . .
. . . . .
Vv14g1576 . . . . . . .
. . . . . . . .
. . . . .
Vv14g1577 . . . . . . .
. . . . . . . .
. . . . .
Vv14g1578 . . . . . . .
. . . . . . . .
. . . . .
Vv14g1579 . . . . . . .
. . . . . . . .
. . . . .
Vv14g1580 . . . . Gorai.006G023000.1
Gorai.012G055600.1 Vv17g0643 . . .
. . . . . . . .
. .
Vv14g1581 . . . . . . .
. . . . . . . .
. . . . .
Vv14g1582 . . . . . . .
. . . . . . . .
. . . . .
Vv14g1583 . . . . . . .
. . . . . . . .
. . . . .
Vv14g1584 . . . . . . .
. . . . . . . .
. . . . .
Vv14g1585 . . . . . . .
. . . . . . . .
. . . . .
Vv14g1586 Tc05_g004120 . . . . .
. . . . . . . .
. . . . . .
Vv14g1587 . . . . . . .
. . . . . . . .
. . . . .
Vv14g1588 . . Gorai.001G256400.1 . .
. . . . . . . .
. . . . . . .
Vv14g1589 Tc05_g004140 . . . . .
. . . . . . . .
. . . . . .
Vv14g1590 Tc04_g008100 . . . .
Gorai.012G055500.1 . . . . . .

```
        .       .       .       .       .       .       .       .
Vv14g1591       .       .       Gorai.001G256300.1      .
Gorai.006G022100.1      Gorai.012G055400.1      .       .       .
        .       .       .       .       Vv1rg0191       .       .
        .       .       .       .
Vv14g1592       .       .       .       .       Gorai.006G022000.1
        Gorai.012G055300.1      .       .       .       .       .
        .       .       .       .       .       .       .       .
        .
Vv14g1593       .       .       .       .       Gorai.006G021900.1
        .       .       .       .       .       .       .       .
        Vv1rg0189       .       .       .       .       .       .
Vv14g1594       .       .       Gorai.001G256200.1      Gorai.
007G036200.1    Gorai.006G021800.1      .       Vv17g0647
Tc03_g009870    .       .       .       Gorai.008G181800.1      .
        Vv1rg0186       .       .       .       .       .       .
Vv14g1595       .       .       .       .       .       .       .
        .       .       .       .       .       .       .       .
        .       .       .       .       .
Vv14g1596       Tc05_g023350    .       .       .       .       .
        .       .       .       .       .       .       .       .
        .       .       .       .       .       .
Vv14g1597       .       .       Gorai.001G255600.1      .       .
        .       .       .       .       .       .       .       .
        .       .       .       .       .       .       .
Vv14g1598       .       .       .       .       .       .       .
        .       .       .       .       .       .       .       .
        .       .       .       .       .
Vv14g1599       .       .       Gorai.001G238700.1      Gorai.
007G036500.1    Gorai.006G020700.1      Gorai.012G055000.1
Vv17g0651       Tc03_g010150    .       .       .       .       .
        Vv1rg0180       .       .       .       .       .       .
Vv14g1600       .       .       .       .       Gorai.006G020600.1
        .       .       .       .       .       .       .       .
        .       .       .       .       .       .       .
Vv14g1601       .       .       .       Gorai.007G036700.1      .
        .       Vv17g0654       Tc03_g010220    .       .       .
        .       .       .       .       .       .       .       .
        .
Vv14g1602       .       .       .       .       Gorai.006G020500.1
        .       .       .       .       .       .       .       .
        Vv1rg0167       .       .       .       .       .       .
Vv14g1603       .       .       .       .       Gorai.006G018000.1
        .       .       .       .       .       .       .       .
        .       .       .       .       .       .       .
Vv14g1604       .       .       .       Gorai.007G036900.1      .
        .       .       .       .       .       .       .       .
        .       .       .       .       .       .       .
Vv14g1605       .       .       .       .       .       .       .
        .       .       .       .       .       .       .       .
```

```
         .     .     .     .     .
Vv14g1606      .     .     .     .     .     .     .
         .     .     .     .     .     .     .     .
         .     .     .     .     .
Vv14g1607      .     .     Gorai.001G238800.1    .     .
         .     .     .     .     .     .     .     .
         .     .     .     .     .     .     .
Vv14g1608      .     .     .     .     Gorai.006G017900.1
         .     .     .     .     .     .     .     .
         .     .     .     .     .     .     .
Vv14g1609      .     .     .     .     Gorai.006G017800.1
         .     .     .     .     .     .     .     .
         .     .     .     .     .     .     .
Vv14g1610      .     .     .     Gorai.007G076400.1    .
         .     .     .     .     .     .     .     .
         .     .     .     .     .     .     .
Vv14g1611      .     .     .     .     .     .     .
         .     .     .     .     .     .     .     .
         .     .     .     .     .
Vv14g1612      .     .     .     .     .     .     .
         .     .     .     .     .     .     .     .
         .     .     .     .     .
Vv14g1613      .     .     .     .     .     .     .
         .     .     .     .     .     .     .     .
         .     .     .     .     .
Vv14g1614      .     .     .     .     .     .     .
         .     .     .     .     .     .     .     .
         .     .     .     .     .
Vv14g1615      Tc05_g004390      .     .     .     .     .
         .     .     .     .     .     .     .     .
         .     .     .     .     .     .
Vv14g1616      .     .     .     .     .     .     .
         .     .     .     .     .     .     .     .
         .     .     .     .     .
Vv14g1617      .     .     .     .     .     .     .
         .     .     .     .     .     .     .     .
         .     .     .     .     .
Vv14g1618      .     .     .     .     .     .     .
         .     .     .     .     .     .     .     .
         .     .     .     .     .
Vv14g1619      .     .     .     .     .     .     .
         .     .     .     .     .     .     .     .
         .     .     .     .     .
Vv14g1620      .     .     .     .     .     .     .
         .     .     .     .     .     .     .     .
         .     .     .     .     .
Vv14g1621      .     .     .     .     .     .     .
         .     .     .     .     .     .     .     .
         .     .     .     .     .
Vv14g1622      .     .     .     .     .     .     .
```

```
        .       .       .       .       .       .       .       .
        .       .       .       .       .
Vv14g1623       Tc04_g008890    .       .       .       Gorai.
006G016800.1    .       .       .       .       .       .       .
        .       .       .       .       .       .       .       .
Vv14g1624       .       .       .       .       .       Gorai.
012G054100.1    .       .       .       .       .       .       .
        .       .       .       .       .       .       .
Vv14g1625       Tc04_g008920    .       .       Gorai.007G078900.1
        Gorai.006G016700.1      Gorai.012G054000.1      .       .
        .       .       .       .       .       .       .       .
        .       .       .       .
Vv14g1626       .       .       .       .       .       .       .
        .       .       .       .       .       .       .       .
        .       .       .       .       .
Vv14g1627       .       .       .       .       .       .       .
        .       .       .       .       .       .       .       .
        .       .       .       .       .
Vv14g1628       .       .       .       .       .       .       .
        .       .       .       .       .       .       .       .
        .       .       .       .       .
Vv14g1629       .       .       .       .       .       .       .
        .       .       .       .       .       .       .       .
        .       .       .       .       .
Vv14g1630       .       .       .       .       .       .       .
        .       .       .       .       .       .       Vv1rg0138
        .       .       .       .       .       .
Vv14g1631       .       .       Gorai.001G239100.1      Gorai.
007G037300.1    .       .       Vv17g0681       .       .       .
        .       .       .       Vv1rg0137       .       .       .
        .       .       .
Vv14g1632       .       .       .       .       .       .       .
        .       .       .       .       .       .       .       .
        .       .       .       .       .
Vv14g1633       .       .       .       .       .       .       .
        .       .       .       .       .       .       .       .
        .       .       .       .       .
Vv14g1634       .       .       .       .       .       .       .
        .       .       .       .       .       .       .       .
        .       .       .       .       .
Vv14g1635       Tc04_g001100    .       .       .       .       .
        .       .       .       .       .       .       .       .
        .       .       .       .       .       .
Vv14g1636       .       .       .       .       .       .       .
        .       .       .       .       .       .       .       .
        .       .       .       .       .
Vv14g1637       .       .       .       .       .       .       .
        .       .       .       .       .       .       .       .
        .       .       .       .       .
Vv14g1638       .       .       .       .       .       .       .
```

. . . . . . . .
. . . . .
Vv14g1639 . . . Gorai.007G037800.1 .
. . . . . . . .
. . . . . . .
Vv14g1640 . . . . . .
Vv17g0688 Tc03_g025210 . . . . .
. . . . . . .
Vv14g1641 . . . . . . .
. . . . . . . .
. . . . .
Vv14g1642 . . . . . . .
. . . . . . . .
. . . . .
Vv14g1643 Tc04_g008960 . . . Gorai.
006G016600.1 . . . . . . .
. Vv1rg0126 . . . . .
.
Vv14g1644 . . . . . . .
. . . . . . . .
. . . . .
Vv14g1645 . . . . . . .
. . . . . . . .
. . . . .
Vv14g1646 . . . . . . .
. . . . . . . .
. . . . .
Vv14g1647 . . . Gorai.007G079000.1 .
. . . . . . . .
. . . . . . .
Vv14g1648 Tc04_g009000 . . . . .
. . . . . . . .
. . . . . .
Vv14g1649 . . . . . . .
. . . . . . . .
. . . . .
Vv14g1650 Tc04_g009010 . Gorai.001G239200.1 .
Gorai.006G016400.1 . . . . .
. . . . . . . .
. .
Vv14g1651 Tc04_g009020 . . Gorai.007G079100.1
. Gorai.012G053600.1 . . . .
. . . . . . . .
. .
Vv14g1652 . . . . . . .
. . . . . . . .
. . . . .
Vv14g1653 Tc04_g009040 . . Gorai.007G079200.1
Gorai.006G015600.1 Gorai.012G053300.1 . .
. . . . . . . .

. . . .
Vv14g1654 Tc05_g004730 . . Gorai.007G079300.1
Gorai.006G015500.1 . Vv17g0693 . .
. . . . Vv1rg0123 . .
. . . .
Vv14g1655 . . . . . . .
. . . . . . . .
. . . . .
Vv14g1656 . . . Gorai.007G079700.1
Gorai.006G015300.1 Gorai.012G053200.1 . . .
. . . . . . . .
. . .
Vv14g1657 Tc04_g000970 . . . . .
. . . . . . . .
. . . . . .
Vv14g1658 Tc04_g009150 . Gorai.001G239700.1 .
Gorai.006G015100.1 . Vv17g0699 . .
. . Gorai.008G194200.1 . Vv1rg0119
. . . . . .
Vv14g1659 . . . . . Gorai.
012G052900.1 . . . . . . .
. . . . . . .
Vv14g1660 . . . . Gorai.006G014900.1
. . . . . . . .
. . . . . . .
Vv14g1661 . . . . Gorai.006G014800.1
Gorai.012G052800.1 . . . . .
. . Vv1rg0114 . . . .
. .
Vv14g1662 Tc05_g004980 . . . . .
. . . . . . . .
. . . . . .
Vv14g1663 Tc04_g009200 . . . .
Gorai.012G052700.1 . . . . . .
. . . . . . . .
Vv14g1664 Tc05_g020930 . . . . .
. . . . . . . .
. . . . . .
Vv14g1665 Tc04_g009220 . . Gorai.007G079900.1
. . . . . . . .
. . . . . . . .
Vv14g1666 . . . . . . .
. . . . . . . .
. . . . .
Vv14g1667 Tc04_g009420 . . . .
Gorai.012G052500.1 . . . . . .
. . . . . . . .
Vv14g1668 . . . . . . .
. . . . . . . .
. . . . .

Vv14g1669 Tc04_g009470 . Gorai.001G240300.1 .
. Gorai.012G052100.1 . . . .
. . . . . . . .
. .
Vv14g1670 Tc04_g009480 . Gorai.001G240400.1 .
. . . . . . . .
. . . . . . . .
Vv14g1671 Tc04_g009500 . . . . .
. . . . . . . .
. . . . . .
Vv14g1672 Tc04_g009520 . . . Gorai.
006G014300.1 Gorai.012G052000.1 . . . .
. . . . . . . .
. .
Vv14g1673 Tc04_g009560 . . . Gorai.
006G014200.1 Gorai.012G051900.1 . . . .
. . . . . . . .
. .
Vv14g1674 . . . . . . .
. . . . . . . .
. . . . .
Vv14g1675 Tc04_g009570 . Gorai.001G240600.1 .
Gorai.006G014100.1 . . . . .
. . . . . . . .
. .
Vv14g1676 Tc04_g009590 . . . Gorai.
006G014000.1 . . . . . . .
. . . . . . . .
Vv14g1677 Tc04_g009600 . Gorai.001G240700.1
Gorai.007G080000.1 . . . . . .
. . . . . . . .
. .
Vv14g1678 Tc04_g009610 . Gorai.001G240800.1 .
. . . . . . . .
. . . . . . . .
Vv14g1679 . . . . . . .
. . . . . . . .
. . . . .
Vv14g1680 Tc04_g009620 . Gorai.001G240900.1
Gorai.007G038300.1 . Gorai.012G051800.1 Vv17g0722
. . . . Gorai.008G179200.1 .
. . . . . . .
Vv14g1681 . . . . . . .
. . . . . . . .
. . . . .
Vv14g1682 Tc04_g009640 . . . .
Gorai.012G051600.1 . . . . . .
. . . . . . . .
Vv14g1683 Tc04_g009650 . . . Gorai.
006G013600.1 . . . . . . .

. . . . . . . .
Vv14g1684 Tc04_g009660 . Gorai.001G241000.1
Gorai.007G080200.1 Gorai.006G013500.1 . . .
. . . . . . . .
. . . .
Vv14g1685 Tc04_g009670 . . Gorai.007G080300.1
. . . . . . . .
. . . . . . . .
Vv14g1686 Tc04_g009690 . Gorai.001G241100.1
Gorai.007G080500.1 Gorai.006G013400.1 . . .
. . . . . . . .
. . . .
Vv14g1687 Tc04_g009720 . . . Gorai.
006G013200.1 . . . . . . .
. . . . . . . .
Vv14g1688 Tc05_g020810 . . . . .
. . . . . . . .
. . . . . .
Vv14g1689 Tc04_g009730 . . . .
Gorai.012G051400.1 . . . . . .
. . . . . . . .
Vv14g1690 . . . . . . .
. . . . . . . .
. . . . .
Vv14g1691 Tc04_g009750 . . Gorai.007G080600.1
. . . . . . . .
. . . . . . . .
Vv14g1692 Tc04_g009760 . . Gorai.007G080700.1
. Gorai.012G051300.1 . . . .
. . . . . . . .
. .
Vv14g1693 Tc04_g009770 . . Gorai.007G080800.1
. . . . . . . .
. . . . . . . .
Vv14g1694 Tc04_g009780 . . . Gorai.
006G013100.1 Gorai.012G051200.1 . . . .
. . . . . . . .
. .
Vv14g1695 . . . . . . .
. . . . . . . .
. . . . .
Vv14g1696 Tc04_g009800 . . . . .
. . . . . . . .
. . . . . .
Vv14g1697 Tc04_g009810 . . . .
Gorai.012G051000.1 . . . . . .
. . . . . . . .
Vv14g1698 Tc04_g009880 . . Gorai.007G080900.1
. . . . . . . .
. . . . . . . .

Vv14g1699 Tc04_g009890 . . . Gorai.
006G012900.1 . . . . . . .
. . . . . . . .
Vv14g1700 Tc04_g009910 . . Gorai.007G081000.1
. . . . . . . .
. . . . . . . .
Vv14g1701 Tc04_g010040 . . . Gorai.
006G012500.1 Gorai.012G050600.1 . . . .
. . . . . . . .
. .
Vv14g1702 . . . . . . .
. . . . . . . .
. . . . .
Vv14g1703 Tc04_g010050 . . Gorai.007G081200.1
Gorai.006G012300.1 Gorai.012G050400.1 . .
. . . . . . . .
. . . .
Vv14g1704 Tc04_g010060 . . Gorai.007G081300.1
. . . . . . . .
. . . . . . . .
Vv14g1705 Tc04_g010070 . . . Gorai.
006G012200.1 Gorai.012G050300.1 Vv17g0746 . .
. . . . . . . .
. . .
Vv14g1706 . . . . . . .
. . . . . . . .
. . . . .
Vv14g1707 Tc04_g010090 . . . Gorai.
006G012000.1 . . . . . . .
. . . . . . . .
Vv14g1708 Tc04_g010100 . . . Gorai.
006G011900.1 . . . . . . .
. . . . . . . .
Vv14g1709 Tc04_g010110 . . . Gorai.
006G011800.1 Gorai.012G050100.1 . . . .
. . . . . . . .
. .
Vv14g1710 . . . . . . .
. . . . . . . .
. . . . .
Vv14g1711 Tc04_g010130 . Gorai.001G243400.1
Gorai.007G040000.1 . Gorai.012G049900.1 . .
. . . . . . . .
. . . .
Vv14g1712 Tc04_g010140 . . Gorai.007G081600.1
. . . . . . . .
. . . . . . . .
Vv14g1713 . . . Gorai.007G081700.1 .
. . . . . . . .
. . . . . . .

Vv14g1714 Tc04_g010150 . . . Gorai.
006G011600.1 . . . . . . .
. . . . . . . .
Vv14g1715 . . . . . . .
. . . . . . . .
. . . . .
Vv14g1716 Tc04_g010170 . . . Gorai.
006G011500.1 . . . . . . .
. . . . . . . .
Vv14g1717 Tc04_g010180 . . . . .
Vv17g0749 . Gorai.001G078200.1 . .
. . . . . . . .
.
Vv14g1718 Tc04_g010190 . . . .
Gorai.012G049800.1 . . . . . .
. . . . . . . .
Vv14g1719 . . . . . . .
. . . . . . . .
. . . . .
Vv14g1720 Tc04_g010210 . . Gorai.007G081800.1
. . Vv17g0764 Tc03_g011610 Gorai.
001G077900.1 . . Gorai.008G177900.1 . .
. . . . . .
Vv14g1721 . . . . . . .
. . . . . . . .
. . . . .
Vv14g1722 . . . . . . .
. . . . . . . .
. . . . .
Vv14g1723 Tc04_g010220 . . . Gorai.
006G011300.1 Gorai.012G049600.1 . . . .
. . . . . . . .
. .
Vv14g1724 . . . . . . .
. . . . . . . .
. . . . .
Vv14g1725 Tc04_g010230 . . Gorai.007G081900.1
Gorai.006G011200.1 Gorai.012G049500.1 . .
. . . . . . . .
. . . .
Vv14g1726 . . . . . . .
. . . . . . . .
. . . . .
Vv14g1727 Tc04_g010240 . . . Gorai.
006G011100.1 Gorai.012G049300.1 . . . .
. . . . . . . .
. .
Vv14g1728 Tc04_g010250 . . . Gorai.
006G010900.1 . . . . . . .
. . . . . . . .

Vv14g1729 . . . . . . .
. . . . . . . .
. . . . .
Vv15g0001 . . . . . . .
. . . . . . . .
. . . . .
Vv15g0002 Tc01_g040010 . . . . .
. . . . . . . .
. . . . . .
Vv15g0003 . . . . . . .
. . . . . . . .
. . . . .
Vv15g0004 . . . . . . .
. . . . . . . .
. . . . .
Vv15g0005 . . . . . . .
. . . . . . . .
. . . . .
Vv15g0006 Tc01_g009040 . . . . .
. . . . . . . .
. . . . . .
Vv15g0007 . . . . . . .
. . . . . . . .
. . . . .
Vv15g0008 . . . . . . .
. . . . . . . .
. . . . .
Vv15g0009 . . . . . . .
. . . . . . . .
. . . . .
Vv15g0010 . . . . . . .
. . . . . . . .
. . . . .
Vv15g0011 . . . . . . .
. . . . . . . .
. . . . .
Vv15g0012 Tc01_g003310 . . . . .
. . . . . . . .
. . . . . .
Vv15g0013 . . . . . . .
. . . . . . . .
. . . . .
Vv15g0014 . . . . . . .
. . . . . . . .
. . . . .
Vv15g0015 . . . . . .
Vv02g0740 . . . . . . .
. . . . . .
Vv15g0016 Tc01_g003020 . Gorai.008G067300.1 .
. . . . . . . .

. . . . . . . .
Vv15g0017 . . . . . . .
. . . . . . . .
. . . . .
Vv15g0018 . . . . . . .
. . . . . . . .
. . . . .
Vv15g0019 . . . . . . .
. . . . . . . .
. . . . .
Vv15g0020 . . . . . . .
. . . . . . . .
. . . . .
Vv15g0021 . . . . . . .
. . . . . . . .
. . . . .
Vv15g0022 . . . . . . .
. . . . . . . .
. . . . .
Vv15g0023 . . . . . . .
. . . . . . . .
. . . . .
Vv15g0024 . . . . . . .
. . . . . . . .
. . . . .
Vv15g0025 . . . . . . .
. . . . . . . .
. . . . .
Vv15g0026 Tc01_g008680 . . . . .
Vv02g0704 . Gorai.004G189000.1 . .
. . . . . . . .
.
Vv15g0027 . . . . . .
Vv02g0703 . . . . Gorai.008G229500.1
. . . . . . . .
Vv15g0028 . . . . . . .
. . . . . . . .
. . . . .
Vv15g0029 . . . . . . .
. . . . . . . .
. . . . .
Vv15g0030 . . . . . . .
. . . . . . . .
. . . . .
Vv15g0031 . . . . . . .
. . . . . . . .
. . . . .
Vv15g0032 . . . . . . .
. . . . . . . .
. . . . .

Vv15g0033 . . . . . . .
. . . . . . . .
. . . . .
Vv15g0034 . . . . . . .
. . . . . . . .
. . . . .
Vv15g0035 Tc01_g040000 . Gorai.008G111700.1 .
. Gorai.004G168400.1 . . . .
. . . . . . . .
. .
Vv15g0036 . . . . . . .
. . . . . . . .
. . . . .
Vv15g0037 . . . . . Gorai.
004G168200.1 Vv02g0683 . . Gorai.003G179700.1
. . Gorai.006G215400.1 . . .
. . . .
Vv15g0038 . . . . . . .
. . . . . . . .
. . . . .
Vv15g0039 Tc01_g039970 . . . . .
. . . . . . . .
. . . . . .
Vv15g0040 . . . . . . .
. . . . . . . .
. . . . .
Vv15g0041 . . . . . . .
. . . . . . . .
. . . . .
Vv15g0042 . . . . . . .
. . . . . . . .
. . . . .
Vv15g0043 . . . . . . .
. . . . . . . .
. . . . .
Vv15g0044 Tc01_g008550 . . . . .
. . . . . . . .
. . . . . .
Vv15g0045 . . Gorai.008G111600.1 . .
. . . . . . . .
. . . . . . .
Vv15g0046 . . . . . . .
. . . . . . . .
. . . . .
Vv15g0047 . . . . . . .
. . . . . . . .
. . . . .
Vv15g0048 . . . . . . .
. . . . . . . .
. . . . .

Vv15g0049 . . . . . . .
. . . . . . . .
. . . . .
Vv15g0050 . . . . . . .
. . . . . . . .
. . . . .
Vv15g0051 . . . . . . .
. . . . . . . .
. . . . .
Vv15g0052 . . . . . . .
. . . . . . . .
. . . . .
Vv15g0053 Tc01_g039940 . . . .
Gorai.004G168100.1 . . . . . .
. . . . . . . .
Vv15g0054 . . . . . . .
. . . . . . . .
. . . . .
Vv15g0055 . . . . . . .
. . . . . . . .
. . . . .
Vv15g0056 . . . . . . .
. . . . . . . .
. . . . .
Vv15g0057 . . . . . . .
. . . . . . . .
. . . . .
Vv15g0058 Tc01_g039930 . . . . .
. . . . . . . .
. . . . . .
Vv15g0059 . . . . . . .
. . . . . . . .
. . . . .
Vv15g0060 . . Gorai.008G111300.1 . .
Gorai.004G168000.1 . . . . .
. . . . . . . .
.
Vv15g0061 . . . . . . .
. . . . . . . .
. . . . .
Vv15g0062 . . . . . . .
. . . . . . . .
. . . . .
Vv15g0063 . . . . . . .
. . . . . . . .
. . . . .
Vv15g0064 Tc01_g039900 . . . .
Gorai.004G167800.1 . . . . . .
. . . . . . . .
Vv15g0065 . . . . . . .

. . . . . . . .
. . . . .
Vv15g0066 Tc01_g039890 . . . . .
Vv02g0660 . Gorai.004G190400.1 Gorai.
003G179300.1 . . Gorai.006G215200.1 . .
. . . . .
Vv15g0067 . . . . . . .
. . . . . . . .
. . . . .
Vv15g0068 . . . . . . .
. . . . . . . .
. . . . .
Vv15g0069 . . . . . . .
. . . . . . . .
. . . . .
Vv15g0070 Tc01_g039870 . . . . .
. . . . . . . .
. . . . . .
Vv15g0071 . . . . . . .
. . . . . . . .
. . . . .
Vv15g0072 Tc01_g039860 . Gorai.008G111200.1 .
Gorai.004G038100.1 Gorai.004G167700.1 . .
. . . . . . . .
. . . .
Vv15g0073 Tc01_g039850 . . . . .
. . . . . . . .
. . . . . .
Vv15g0074 . . . . . . .
. . . . . . . .
. . . . .
Vv15g0075 . . . . . Gorai.
004G167600.1 . . . . . . .
. . . . . . .
Vv15g0076 . . . . . . .
. . . . . . . .
. . . . .
Vv15g0077 Tc01_g039840 . . . .
Gorai.004G167500.1 Vv02g0647 . Gorai.004G190800.1
. . . Gorai.006G214900.1 . .
. . . . .
Vv15g0078 Tc01_g039830 . Gorai.008G110800.1 .
. Gorai.004G167400.1 Vv02g0646 .
Gorai.004G190900.1 . . . Gorai.006G214800.1
. . . . . . .
Vv15g0079 Tc01_g039820 . Gorai.008G110700.1 .
Gorai.004G038500.1 Gorai.004G167300.1 Vv02g0642
. . . . . . . .
. . . . .
Vv15g0080 . . . . . . .

. . . . . . . .
. . . . .
Vv15g0081 Tc01_g039810 . . . . .
. . . . . . . .
. . . . . .
Vv15g0082 . . . . . . .
. . . . . . . .
. . . . .
Vv15g0083 . . . . . . .
. . . . . . . .
. . . . .
Vv15g0084 Tc01_g039800 . Gorai.008G110600.1 .
. . . . . . . .
. . . . . . . .
Vv15g0085 . . . . . . .
. . . . . . . .
. . . . .
Vv15g0086 Tc01_g039790 . Gorai.008G110500.1 .
. . . . . . . .
. . . . . . . .
Vv15g0087 . . . . . . .
. . . . . . . .
. . . . .
Vv15g0088 Tc01_g039780 . . . .
Gorai.004G167200.1 . . . . . .
. . . . . . . .
Vv15g0089 Tc01_g039770 . Gorai.008G110400.1 .
. . . . . . . .
. . . . . . . .
Vv15g0090 Tc01_g039760 . . . . .
. . . . . . . .
. . . . . .
Vv15g0091 Tc01_g039750 . . . . .
. . . . . . . .
. . . . . .
Vv15g0092 . . . . . . .
. . . . . . . .
. . . . .
Vv15g0093 . . . . . . .
. . . . . . . .
. . . . .
Vv15g0094 Tc01_g039740 . . . . .
. . . . . . . .
. . . . . .
Vv15g0095 . . . . . . .
. . . . . . . .
. . . . .
Vv15g0096 . . . . . . .
. . . . . . . .
. . . . .

```
Vv15g0097        .       .        .       .        .       .        .
        .        .       .        .       .        .       .        .
        .        .       .        .       .
Vv15g0098        .       .        .       .        .       .        .
        .        .       .        .       .        .       .        .
        .        .       .        .       .
Vv15g0099        .       .        .       .        .       .        .
        .        .       .        .       .        .       .        .
        .        .       .        .       .
Vv15g0100        Tc01_g039560     .       Gorai.008G109800.1        .
        .        .       .        .       .        .       .        .
        .        .       .        .       .        .       .        .
Vv15g0101        Tc01_g039540     .       Gorai.008G109700.1        .
        .        Gorai.004G167000.1       .        .       .        .
        .        .       .        .       .        .       .        .
        .        .
Vv15g0102        Tc01_g039530     .       .        .       .        .
        .        .       .        .       .        .       .        .
        .        .       .        .       .        .
Vv15g0103        .       .        .       .        .       .        .
        .        .       .        .       .        .       .        .
        .        .       .        .       .
Vv15g0104        .       .        .       .        .       .        .
        .        .       .        .       .        .       .        .
        .        .       .        .       .
Vv15g0105        .       .        .       .        .       .        .
        .        .       .        .       .        .       .        .
        .        .       .        .       .
Vv15g0106        Tc01_g039520     .       .        .       .
Gorai.004G166900.1       .        .       .        .       .        .
        .        .       .        .       .        .       .        .
Vv15g0107        .       .        .       .        .       .        .
        .        .       .        .       .        .       .        .
        .        .       .        .       .
Vv15g0108        .       .        .       .        .       Gorai.
004G166600.1     .       .        .       .        .       .        .
        .        .       .        .       .        .       .
Vv15g0109        .       .        .       .        Gorai.004G038600.1
        .        .       .        .       .        .       .        .
        .        .       .        .       .        .       .
Vv15g0110        .       .        .       .        .       .        .
        .        .       .        .       .        .       .        .
        .        .       .        .       .
Vv15g0111        Tc01_g039390     .       Gorai.008G109500.1        .
        .        .       .        .       .        .       .        .
        .        .       .        .       .        .       .        .
Vv15g0112        .       .        .       .        .       .        .
        .        .       .        .       .        .       .        .
        .        .       .        .       .
Vv15g0113        Tc01_g039350     .       .        .       .        .
```

. . . . . . . .
. . . . . .
Vv15g0114 Tc01_g039340 . . . . .
. . . . . . . .
. . . . . .
Vv15g0115 . . . . Gorai.004G038700.1
Gorai.004G166300.1 . . . . .
. . . . . . . .
.
Vv15g0116 Tc01_g039330 . . . .
Gorai.004G166200.1 . . . . . .
. . . . . . . .
Vv15g0117 . . . . . . .
. . . . . . . .
. . . . .
Vv15g0118 . . . . Gorai.004G038800.1
. . . . . . . .
. . . . . . .
Vv15g0119 . . . . . . .
. . . . . . . .
. . . . .
Vv15g0120 . . . . . . .
. . . . . . . .
. . . . .
Vv15g0121 . . . . . . .
. . . . . . . .
. . . . .
Vv15g0122 . . . . . . .
. . . . . . . .
. . . . .
Vv15g0123 . . . . . . .
. . . . . . . .
. . . . .
Vv15g0124 Tc01_g039320 . . . . .
. . . . . . . .
. . . . . .
Vv15g0125 . . . . . . .
. . . . . . . .
. . . . .
Vv15g0126 . . . . . . .
. . . . . . . .
. . . . .
Vv15g0127 . . . . . . .
. . . . . . . .
. . . . .
Vv15g0128 Tc01_g039310 . Gorai.008G109100.1 .
. . . . . . . .
. . . . . . . .
Vv15g0129 . . . . . . .
. . . . . . . .

. . . . .
Vv15g0130 . . . . . . .
. . . . . . . .
. . . . .
Vv15g0131 Tc01_g039300 . Gorai.008G109000.1 .
. . . . . . . .
. . . . . . . .
Vv15g0132 Tc01_g039290 . Gorai.008G108900.1 .
. . . . . . . .
. . . . . . . .
Vv15g0133 Tc01_g039280 . Gorai.008G108800.1 .
. Gorai.004G166100.1 . . . .
. . . . . . . .
. .
Vv15g0134 Tc01_g039270 . . . . .
. . . . . . . .
. . . . . .
Vv15g0135 Tc01_g039260 . . . .
Gorai.004G165900.1 . . . . . .
. . . . . . . .
Vv15g0136 Tc01_g039250 . . . Gorai.
004G039000.1 . . . . . . .
. . . . . . . .
Vv15g0137 . . . . . . .
. . . . . . . .
. . . . .
Vv15g0138 Tc01_g039240 . Gorai.008G108700.1 .
Gorai.004G039100.1 . . . . .
. . . . . . . .
. .
Vv15g0139 Tc01_g039230 . Gorai.008G108600.1 .
. . . . . . . .
. . . . . . . .
Vv15g0140 Tc01_g039150 . . . . .
. . . . . . . .
. . . . . .
Vv15g0141 . . . . . . .
. . . . . . . .
. . . . .
Vv15g0142 . . . . . . .
. . . . . . . .
. . . . .
Vv15g0143 . . . . . . .
. . . . . . . .
. . . . .
Vv15g0144 Tc01_g039120 . . . . .
. . . . . . . .
. . . . . .
Vv15g0145 . . . . . . .
. . . . . . . .

. . . . .
Vv15g0146 . . . . . . .
. . . . . . . .
. . . . .
Vv15g0147 . . Gorai.008G108300.1 . .
Gorai.004G165600.1 . . . . .
. . . . . . . .
.
Vv15g0148 . . . . . . .
. . . . . . . .
. . . . .
Vv15g0149 . . . . . . .
. . . . . . . .
. . . . .
Vv15g0150 . . . . . . .
. . . . . . . .
. . . . .
Vv15g0151 . . . . . . .
. . . . . . . .
. . . . .
Vv15g0152 Tc01_g039110 . . . Gorai.
004G039300.1 . . . . . . .
. . . . . . . .
Vv15g0153 . . Gorai.008G108200.1 . .
. . . . . . . .
. . . . . . .
Vv15g0154 . . . . . . .
. . . . . . . .
. . . . .
Vv15g0155 . . . . . . .
. . . . . . . .
. . . . .
Vv15g0156 Tc01_g039090 . . . .
Gorai.004G165500.1 . . . . . .
. . . . . . . .
Vv15g0157 Tc01_g039080 . . . . .
. . . . . . . .
. . . . . .
Vv15g0158 Tc01_g039070 . . . . .
. . . . . . . .
. . . . . .
Vv15g0159 Tc01_g039060 . . . . .
. . . . . . . .
. . . . . .
Vv15g0160 . . . . . . .
. . . . . . . .
. . . . .
Vv15g0161 Tc01_g039050 . Gorai.008G108000.1 .
. . . . . . . .
. . . . . . . .

| | | | | | | | |
|---|---|---|---|---|---|---|---|
| Vv15g0162 | . | . | . | . | . | . | . |
| . | . | . | . | . | . | . | . |
| . | . | . | . | . | | | |
| Vv15g0163 | . | . | . | . | . | . | . |
| . | . | . | . | . | . | . | . |
| . | . | . | . | . | | | |
| Vv15g0164 | Tc01_g039040 | | . | Gorai.008G107900.1 | | | . |
| . | . | . | . | . | . | . | . |
| . | . | . | . | . | . | . | . |
| Vv15g0165 | . | . | . | . | . | . | . |
| . | . | . | . | . | . | . | . |
| . | . | . | . | . | | | |
| Vv15g0166 | . | . | . | . | . | . | . |
| . | . | . | . | . | . | . | . |
| . | . | . | . | . | | | |
| Vv15g0167 | . | . | . | . | Gorai.004G039400.1 | | |
| . | . | . | . | . | . | . | . |
| . | . | . | . | . | . | . | |
| Vv15g0168 | Tc01_g039030 | | . | . | . | . | . |
| . | . | . | . | . | . | . | . |
| . | . | . | . | . | . | | |
| Vv15g0169 | . | . | . | . | . | . | . |
| . | . | . | . | . | . | . | . |
| . | . | . | . | . | | | |
| Vv15g0170 | . | . | . | . | . | . | . |
| . | . | . | . | . | . | . | . |
| . | . | . | . | . | | | |
| Vv15g0171 | . | . | . | . | . | . | . |
| . | . | . | . | . | . | . | . |
| . | . | . | . | . | | | |
| Vv15g0172 | . | . | . | . | . | . | . |
| . | . | . | . | . | . | . | . |
| . | . | . | . | . | | | |
| Vv15g0173 | . | . | . | . | . | . | . |
| . | . | . | . | . | . | . | . |
| . | . | . | . | . | | | |
| Vv15g0174 | . | . | . | . | . | . | . |
| . | . | . | . | . | . | . | . |
| . | . | . | . | . | | | |
| Vv15g0175 | . | . | . | . | . | . | . |
| . | . | . | . | . | . | . | . |
| . | . | . | . | . | | | |
| Vv15g0176 | Tc01_g038810 | | . | . | . | . | . |
| . | . | . | . | . | . | . | . |
| . | . | . | . | . | . | | |
| Vv15g0177 | Tc01_g038800 | | . | . | . | . | . |
| . | . | . | . | . | . | . | . |
| . | . | . | . | . | . | | |
| Vv15g0178 | . | . | . | . | . | . | . |
| . | . | . | . | . | . | . | . |

. . . . .
Vv15g0179 Tc01_g017270 . . . . .
. . . . . . . .
. . . . . .
Vv15g0180 . . . . . . .
. . . . . . . .
. . . . .
Vv15g0181 . . . . . . .
. . . . . . . .
. . . . .
Vv15g0182 . . . . . . .
. . . . . . . .
. . . . .
Vv15g0183 Tc01_g038650 . . . . .
. . . . . . . .
. . . . . .
Vv15g0184 . . . . . . .
. . . . . . . .
. . . . .
Vv15g0185 Tc01_g038630 . . Gorai.001G176400.1
. Gorai.004G165200.1 . . . .
. . . . . . . .
. .
Vv15g0186 Tc01_g038620 . . . Gorai.
004G039600.1 Gorai.004G165100.1 . . . .
. . . . . . . .
. .
Vv15g0187 . . . . . . .
. . . . . . . .
. . . . .
Vv15g0188 . . . . Gorai.004G039700.1
. . . . . . . .
. . . . . . .
Vv15g0189 . . . . . . .
. . . . . . . .
. . . . .
Vv15g0190 . . . . . . .
. . . . . . . .
. . . . .
Vv15g0191 Tc01_g038610 . . . .
Gorai.004G165000.1 . . . . . .
. . . . . . . .
Vv15g0192 Tc01_g038600 . . . . .
. . . . . . . .
. . . . . .
Vv15g0193 Tc01_g038590 . Gorai.008G107600.1 .
. . . . . . . .
. . . . . . . .
Vv15g0194 Tc01_g038570 . . . . .
. . . . . . . .

. . . . . .
Vv15g0195 Tc01_g038560 . . . . .
. . . . . . . .
. . . . . .
Vv15g0196 . . . . . . .
. . . . . . . .
. . . . .
Vv15g0197 Tc01_g011350 . . . . .
. . . . . . . .
. . . . . .
Vv15g0198 Tc01_g038530 . . . . .
. . . . . . . .
. . . . . .
Vv15g0199 . . . . . . .
. . . . . . . .
. . . . .
Vv15g0200 . . . . . . .
. . . . . . . .
. . . . .
Vv15g0201 . . . . . . .
. . . . . . . .
. . . . .
Vv15g0202 . . . . . Gorai.
004G164800.1 . . . . . . .
. . . . . . .
Vv15g0203 . . . . . . .
. . . . . . . .
. . . . .
Vv15g0204 Tc01_g038490 . Gorai.008G107400.1 .
. . . . . . . .
. . . . . . . .
Vv15g0205 Tc01_g038480 . Gorai.008G107300.1
Gorai.001G176000.1 . . . . . .
. . . . . . . .
. .
Vv15g0206 . . . . . . .
. . . . . . . .
. . . . .
Vv15g0207 . . . . . . .
. . . . . . . .
. . . . .
Vv15g0208 . . . . . . .
. . . . . . . .
. . . . .
Vv15g0209 . . . . . . .
. . . . . . . .
. . . . .
Vv15g0210 . . . . . . .
. . . . . . . .
. . . . .

Vv15g0211 . . . . . . .
. . . . . . . .
. . . . .
Vv15g0212 . . . . . . .
. . . . . . . .
. . . . .
Vv15g0213 . . . . . . .
. . . . . . . .
. . . . .
Vv15g0214 . . . . . . .
. . . . . . . .
. . . . .
Vv15g0215 . . . . . . .
. . . . . . . .
. . . . .
Vv15g0216 . . . . . . .
. . . . . . . .
. . . . .
Vv15g0217 . . . . . . .
. . . . . . . .
. . . . .
Vv15g0218 . . . . . . .
. . . . . . . .
. . . . .
Vv15g0219 Tc01_g017110 . . . . .
. . . . . . . .
. . . . . .
Vv15g0220 Tc01_g011440 . . . . .
. . . . . . . .
. . . . . .
Vv15g0221 . . . . Gorai.004G039800.1
. . . . . . . .
. . . . . . .
Vv15g0222 . . . . . . .
. . . . . . . .
. . . . .
Vv15g0223 . . . . . . .
. . . . . . . .
. . . . .
Vv15g0224 . . . . . . .
. . . . . . . .
. . . . .
Vv15g0225 . . . . . . .
. . . . . . . .
. . . . .
Vv15g0226 Tc01_g038420 . . . .
Gorai.004G164700.1 . . . . . .
. . . . . . . .
Vv15g0227 Tc01_g038410 . . Gorai.001G175600.1
. . . . . . . .

. . . . . . . .
Vv15g0228 Tc01_g038400 . Gorai.008G107100.1 .
. . . . . . . .

. . . . . . . .
Vv15g0229 . . . . . . .
. . . . . . . .

. . . . .
Vv15g0230 . . . . . . .
. . . . . . . .

. . . . .
Vv15g0231 . . . . . . .
. . . . . . . .

. . . . .
Vv15g0232 . . . . . . .
. . . . . . . .

. . . . .
Vv15g0233 Tc01_g038380 . Gorai.008G107000.1 .
. Gorai.004G164500.1 . . . .
. . . . . . . .

. .
Vv15g0234 . . . . . . .
. . . . . . . .

. . . . .
Vv15g0235 . . . . . . .
. . . . . . . .

. . . . .
Vv15g0236 . . . . . . .
. . . . . . . .

. . . . .
Vv15g0237 Tc01_g038360 . . . .
Gorai.004G164400.1 . . . . . .
. . . . . . . .

Vv15g0238 . . . . . . .
. . . . . . . .

. . . . .
Vv15g0239 Tc01_g011460 . Gorai.008G106900.1 .
. . . . . . . .

. . . . . . . .
Vv15g0240 . . . . . . .
. . . . . . . .

. . . . .
Vv15g0241 Tc01_g038340 . Gorai.008G106700.1 .
. Gorai.004G164200.1 . . . .
. . . . . . . .

. .
Vv15g0242 Tc01_g038310 . Gorai.008G106600.1
Gorai.001G175500.1 Gorai.004G039900.1 . . .
. . . . . . . .

. . . .
Vv15g0243 Tc01_g038300 . Gorai.008G106500.1

```
Gorai.001G175400.1       .        Gorai.004G164000.1       .        .
        .        .       .        .       .        .       .        .
        .        .       .        .
Vv15g0244        Tc01_g038290     .       .        Gorai.001G175300.1
        .        .       .        .       .        .       .        .
        .        .       .        .       .        .       .        .
Vv15g0245        Tc01_g038280     .       Gorai.008G106400.1        .
        .        .       .        .       .        .       .        .
        .        .       .        .       .        .       .        .
Vv15g0246        Tc01_g038270     .       .        .       .        .
        .        .       .        .       .        .       .        .
        .        .       .        .       .        .
Vv15g0247        .       .        .       .        .       .        .
        .        .       .        .       .        .       .        .
        .        .       .        .       .
Vv15g0248        .       .        .       .        .       .        .
        .        .       .        .       .        .       .        .
        .        .       .        .       .
Vv15g0249        .       .        .       .        .       .        .
        .        .       .        .       .        .       .        .
        .        .       .        .       .
Vv15g0250        .       .        .       .        .       .        .
        .        .       .        .       .        .       .        .
        .        .       .        .       .
Vv15g0251        .       .        .       .        .       .        .
        .        .       .        .       .        .       .        .
        .        .       .        .       .
Vv15g0252        .       .        .       .        .       .        .
        .        .       .        .       .        .       .        .
        .        .       .        .       .
Vv15g0253        .       .        .       .        .       .        .
        .        .       .        .       .        .       .        .
        .        .       .        .       .
Vv15g0254        .       .        .       .        .       .        .
        .        .       .        .       .        .       .        .
        .        .       .        .       .
Vv15g0255        .       .        .       .        .       .        .
        .        .       .        .       .        .       .        .
        .        .       .        .       .
Vv15g0256        .       .        .       .        .       .        .
        .        .       .        .       .        .       .        .
        .        .       .        .       .
Vv15g0257        .       .        .       .        .       .        .
        .        .       .        .       .        .       .        .
        .        .       .        .       .
Vv15g0258        .       .        .       .        .       .        .
        .        .       .        .       .        .       .        .
        .        .       .        .       .
Vv15g0259        .       .        .       .        .       .        .
        .        .       .        .       .        .       .        .
```

. . . . .
Vv15g0260 . . . . . . .
. . . . . . . .
. . . . .
Vv15g0261 . . . . . . .
. . . . . . . .
. . . . .
Vv15g0262 . . . . . . .
. . . . . . . .
. . . . .
Vv15g0263 . . . . . . .
. . . . . . . .
. . . . .
Vv15g0264 Tc01_g038180 . . . . .
. . . . . . . .
. . . . . .
Vv15g0265 Tc01_g038170 . . . .
Gorai.004G163900.1 . . . . . .
. . . . . . . .
Vv15g0266 Tc01_g038160 . Gorai.008G113000.1 .
Gorai.004G040100.1 Gorai.004G163800.1 Vv02g0623
Tc03_g025100 Gorai.004G163800.1 Gorai.003G170900.1
Gorai.007G028100.1 Gorai.008G235700.1 Gorai.
006G213500.1 . . . . . . .
Vv15g0267 Tc01_g038150 . . . . .
. . . . . . . .
. . . . . .
Vv15g0268 Tc01_g038140 . . Gorai.001G175000.1
. . . . . . . .
. . . . . . . .
Vv15g0269 Tc01_g038130 . Gorai.008G106100.1 .
. . Vv02g0616 Tc03_g025170 .
Gorai.003G170700.1 . . . . . .
. . . .
Vv15g0270 . . . . . . .
. . . . . . . .
. . . . .
Vv15g0271 . . . . . . .
. . . . . . . .
. . . . .
Vv15g0272 . . . . . . .
. . . . . . . .
. . . . .
Vv15g0273 . . . . . . .
. . . . . . . .
. . . . .
Vv15g0274 . . . . . . .
. . . . . . . .
. . . . .
Vv15g0275 . . . . . . .

. . . . . . . .
. . . . .
Vv15g0276 . . . . . . .
. . . . . . . .
. . . . .
Vv15g0277 . . . . . . .
. . . . . . . .
. . . . .
Vv15g0278 . . Gorai.008G113600.1 . .
. . . . . . . .
. . . . . . .
Vv15g0279 . . . . . . .
. . . . . . . .
. . . . .
Vv15g0280 . . Gorai.008G113700.1 . .
. . . . . . . .
. . . . . . .
Vv15g0281 . . Gorai.008G113800.1 . .
. . . . . . . .
. . . . . . .
Vv15g0282 . . . . . . .
. . . . . . . .
. . . . .
Vv15g0283 . . . . . . .
. . . . . . . .
. . . . .
Vv15g0284 . . . . . . .
. . . . . . . .
. . . . .
Vv15g0285 . . . . . . .
. . . . . . . .
. . . . .
Vv15g0286 . . . . . . .
. . . . . . . .
. . . . .
Vv15g0287 . . . . . .
Vv02g0601 Tc03_g025320 . Gorai.003G170400.1 .
. . . . . . . .
.
Vv15g0288 . . . . . . .
. . . . . . . .
. . . . .
Vv15g0289 . . . . . . .
. . . . . . . .
. . . . .
Vv15g0290 . . . . . . .
. . . . . . . .
. . . . .
Vv15g0291 . . . . . . .
. . . . . . . .

. . . . .
Vv15g0292 Tc01_g037990 . . . . .
. . . . . . . .
. . . . . .
Vv15g0293 . . . . . . .
. . . . . . . .
. . . . .
Vv15g0294 . . . . . . .
. . . . . . . .
. . . . .
Vv15g0295 . . . . . . .
. . . . . . . .
. . . . .
Vv15g0296 Tc01_g037960 . . . . .
. . . . . . . .
. . . . . .
Vv15g0297 Tc01_g037930 . . . . .
Vv02g0566 Tc03_g025600 Gorai.004G201400.1
Gorai.003G114700.1 . Gorai.008G238500.1 Gorai.
006G212100.1 . . . . . . .
Vv15g0298 . . . . . . .
. . . . . . . .
. . . . .
Vv15g0299 Tc01_g037920 . Gorai.008G113200.1 .
. . Vv02g0564 Tc03_g025620 Gorai.
004G201500.1 Gorai.003G114800.1 Gorai.007G090200.1
Gorai.008G238600.1 Gorai.006G212000.1 . . .
. . . .
Vv15g0300 . . . . . . .
. . . . . . . .
. . . . .
Vv15g0301 . . . . . Gorai.
004G163300.1 . . . . . . .
. . . . . . .
Vv15g0302 . . . . . . .
. . . . . . . .
. . . . .
Vv15g0303 . . . Gorai.001G174700.1 .
. . . . . . . .
. . . . . . .
Vv15g0304 . . . . . . .
. . . . . . . .
. . . . .
Vv15g0305 Tc01_g037860 . . . Gorai.
004G040500.1 Gorai.004G163100.1 . . . .
. . . . . . . .
. .
Vv15g0306 . . . . . . .
. . . . . . . .
. . . . .

Vv15g0307 . . . . . . .
. . . . . . . .
. . . . .
Vv15g0308 . . . . . . .
. . . . . . . .
. . . . .
Vv15g0309 . . . . . . .
. . . . . . . .
. . . . .
Vv15g0310 . . . . . . .
. . . . . . . .
. . . . .
Vv15g0311 . . . . . . .
. . . . . . . .
. . . . .
Vv15g0312 . . Gorai.008G105800.1 . .
. . . . . . . .
. . . . . . .
Vv15g0313 . . . . . . .
. . . . . . . .
. . . . .
Vv15g0314 Tc01_g037770 . . Gorai.001G174200.1
Gorai.004G040700.1 Gorai.004G162400.1 Vv02g0542
Tc03_g025870 Gorai.004G162400.1 . Gorai.
007G090600.1 Gorai.008G284100.1 Gorai.006G195900.1 .
. . . . . .
Vv15g0315 Tc01_g037760 . . . . .
. . . . . . . .
. . . . . .
Vv15g0316 Tc01_g037750 . . . . .
. . . . . . . .
. . . . . .
Vv15g0317 Tc01_g037740 . Gorai.008G113900.1 .
. Gorai.004G162000.1 Vv02g0537
Tc03_g025910 Gorai.004G162000.1 . . Gorai.
008G239700.1 . . . . . . .
.
Vv15g0318 Tc01_g037710 . Gorai.008G114100.1
Gorai.001G174100.1 . . . . . .
. . . . . . . .
. .
Vv15g0319 . . . . . . .
. . . . . . . .
. . . . .
Vv15g0320 . . . . . . .
. . . . . . . .
. . . . .
Vv15g0321 . . . . . . .
. . . . . . . .
. . . . .

Vv15g0322 . . . . . . .
. . . . . . . .
. . . . .
Vv15g0323 Tc01_g007280 . . . . .
. . . . . . . .
. . . . . .
Vv15g0324 Tc01_g037530 . . . . .
Vv02g0534 Tc03_g026070 Gorai.004G161300.1 .
Gorai.007G021700.1 Gorai.008G283600.1 . .
. . . . . .
Vv15g0325 . . . . . Gorai.
004G160800.1 . . . . . . .
. . . . . . .
Vv15g0326 . . . . . . .
. . . . . . . .
. . . . .
Vv15g0327 . . . . . . .
. . . . . . . .
. . . . .
Vv15g0328 Tc01_g037490 . . . . .
. . . . . . . .
. . . . . .
Vv15g0329 . . . . . . .
. . . . . . . .
. . . . .
Vv15g0330 Tc01_g037470 . . . . .
. . . . . . . .
. . . . . .
Vv15g0331 . . . . . . .
. . . . . . . .
. . . . .
Vv15g0332 . . . . . . .
. . . . . . . .
. . . . .
Vv15g0333 . . . . . . .
. . . . . . . .
. . . . .
Vv15g0334 . . . . . . .
. . . . . . . .
. . . . .
Vv15g0335 Tc01_g037430 . . . .
Gorai.004G158000.1 Vv02g0531 Tc03_g026120 Gorai.
004G160700.1 . Gorai.007G091800.1 Gorai.008G240700.1
Gorai.006G211500.1 . . . . .
. .
Vv15g0336 . . . . . . .
. . . . . . . .
. . . . .
Vv15g0337 . . . . . . .
. . . . . . . .

```
       .       .       .       .       .
Vv15g0338      .       .       .       .       .       .       .
       .       .       .       .       .       .       .       .
       .       .       .       .       .
Vv15g0339      .       .       .       .       .       .       .
       .       .       .       .       .       .       .       .
       .       .       .       .       .
Vv15g0340      .       .       .       .       .       .       .
       .       .       .       .       .       .       .       .
       .       .       .       .       .
Vv15g0341      .       .       Gorai.008G114200.1      .       .
       .       .       .       .       .       .       .       .
       .       .       .       .       .       .       .
Vv15g0342      Tc01_g007270    .       .       .       Gorai.
004G040800.1   .       .       .       .       .       .       .
       .       .       .       .       .       .       .       .
Vv15g0343      .       .       .       .       .       .       .
       .       .       .       .       .       .       .       .
       .       .       .       .       .
Vv15g0344      .       .       .       Gorai.001G174000.1      .
       .       .       .       .       .       .       .       .
       .       .       .       .       .       .       .
Vv15g0345      .       .       .       .       .       .       .
       .       .       .       .       .       .       .       .
       .       .       .       .       .
Vv15g0346      .       .       .       .       .       .       .
       .       .       .       .       .       .       .       .
       .       .       .       .       .
Vv15g0347      .       .       .       Gorai.001G173900.1      .
       .       .       .       .       .       .       .       .
       .       .       .       .       .       .       .
Vv15g0348      .       .       .       Gorai.001G173700.1      .
       .       .       .       .       .       .       .       .
       .       .       .       .       .       .       .
Vv15g0349      .       .       .       .       .       Gorai.
004G156300.1   .       .       .       .       .       .       .
       .       .       .       .       .       .       .
Vv15g0350      .       .       .       .       .       .       .
       .       .       .       .       .       .       .       .
       .       .       .       .       .
Vv15g0351      .       .       .       .       .       .
Vv02g0465      Tc03_g026680    Gorai.004G207700.1      .       .
       Gorai.008G243500.1      .       .       .       .       .
       .       .       .
Vv15g0352      .       .       Gorai.008G116300.1      .       .
       Gorai.004G156100.1      Vv02g0467       Tc03_g026670    .
       .       .       .       Gorai.006G210200.1      .       .
       .       .       .       .       .
Vv15g0353      .       .       .       .       .       Gorai.
004G156000.1   .       .       .       .       .       .       .
```

. . . . . . .
Vv15g0354 . . . . . . .
. . . . . . . .
. . . . .
Vv15g0355 . . Gorai.008G115900.1 . .
. Vv02g0473 Tc03_g026620 . Gorai.
003G162900.1 . Gorai.008G243400.1 . . .
. . . . .
Vv15g0356 . . Gorai.008G115800.1 . .
Gorai.004G155900.1 . . . . .
. . . . . . . .
.
Vv15g0357 . . . . . . .
. . . . . . . .
. . . . .
Vv15g0358 . . . . . . .
. . . . . . . .
. . . . .
Vv15g0359 . . . . . . .
. . . . . . . .
. . . . .
Vv15g0360 . . . . . . .
. . . . . . . .
. . . . .
Vv15g0361 . . . . . . .
. . . . . . . .
. . . . .
Vv15g0362 . . . . . . .
. . . . . . . .
. . . . .
Vv15g0363 . . . . .
Vv02g0478 Tc03_g026580 Gorai.004G206900.1 .
Gorai.007G094200.1 Gorai.008G243000.1 Gorai.006G197000.1
. . . . . .
Vv15g0364 . . Gorai.008G115700.1 . .
. Vv02g0481 Tc03_g026510 Gorai.004G206700.1
. Gorai.007G094100.1 . . . .
. . . . .
Vv15g0365 . . . . . . .
. . . . . . . .
. . . . .
Vv15g0366 . . . . . . .
. . . . . . . .
. . . . .
Vv15g0367 . . . . . . .
. . . . . . . .
. . . . .
Vv15g0368 Tc01_g037040 . Gorai.008G115400.1 .
. . . . . . . .
. . . . . . . .

```
Vv15g0369       Tc01_g037030     .       .       .       .       .
        .       .       .        .       .       .       .       .
        .       .       .        .       .       .
Vv15g0370       Tc01_g037020     .       Gorai.008G115500.1      .
        .       .       .        .       .       .       .       .
        .       .       .        .       .       .       .       .
Vv15g0371       .       .        .       .       .       .       .
        .       .       .        .       .       .       .       .
        .       .       .        .       .
Vv15g0372       .       .        .       .       .       .       .
        .       .       .        .       .       .       .       .
        .       .       .        .       .
Vv15g0373       .       .        Gorai.008G115300.1      .       .
        .       .       .        .       .       .       .       .
        .       .       .        .       .       .       .
Vv15g0374       .       .        .       .       .       .       .
        .       .       .        .       .       .       .       .
        .       .       .        .       .
Vv15g0375       .       .        .       .       .       .
Vv02g0484       Tc03_g026480     .       Gorai.003G163900.1
Gorai.007G009800.1      Gorai.008G242500.1       .       .       .
        .       .       .        .       .
Vv15g0376       .       .        .       .       .       .       .
        .       .       .        .       .       .       .       .
        .       .       .        .       .
Vv15g0377       .       .        .       .       .       .       .
        .       .       .        .       .       .       .       .
        .       .       .        .       .
Vv15g0378       .       .        .       .       .       .       .
        .       .       .        .       .       .       .       .
        .       .       .        .       .
Vv15g0379       .       .        .       .       .       .       .
        .       .       .        .       .       .       .       .
        .       .       .        .       .
Vv15g0380       Tc01_g021930     .       .       .       .
Gorai.004G155600.1      .        .       .       .       .       .
        .       .       .        .       .       .       .       .
Vv15g0381       .       .        .       .       .       .       .
        .       .       .        .       .       .       .       .
        .       .       .        .       .
Vv15g0382       .       .        .       .       .       .       .
        .       .       .        .       .       .       .       .
        .       .       .        .       .
Vv15g0383       .       .        .       .       .       .
Vv02g0492       Tc03_g026410     .       Gorai.003G164300.1      .
        .       .       .        .       .       .       .       .
        .
Vv15g0384       .       .        Gorai.008G115100.1      .       .
        .       .       .        .       .       .       .       .
        .       .       .        .       .       .       .
```

Vv15g0385 . . . . . . .
. . . . . . . .
. . . . .
Vv15g0386 . . . . . . .
. . . . . . . .
. . . . .
Vv15g0387 . . . . . . .
. . . . . . . .
. . . . .
Vv15g0388 . . . . . . .
. . . . . . . .
. . . . .
Vv15g0389 . . . . . . .
. . . . . . . .
. . . . .
Vv15g0390 . . . . . . .
. . . . . . . .
. . . . .
Vv15g0391 . . . . . . .
. . . . . . . .
. . . . .
Vv15g0392 . . . . . . .
. . . . . . . .
. . . . .
Vv15g0393 . . Gorai.008G115000.1 . .
. . . . . . . .
. . . . . . .
Vv15g0394 . . . Gorai.001G173300.1 .
. . . . . . . .
. . . . . . .
Vv15g0395 . . . . . . .
. . . . . . . .
. . . . .
Vv15g0396 . . . . . . .
. . . . . . . .
. . . . .
Vv15g0397 . . . . . . .
. . . . . . . .
. . . . .
Vv15g0398 . . . . . . .
. . . . . . . .
. . . . .
Vv15g0399 . . . . . . .
. . . . . . . .
. . . . .
Vv15g0400 . . . . . . .
. . . . . . . .
. . . . .
Vv15g0401 Tc01_g034320 . . . . .
. . . . . . . .

. . . . . .
Vv15g0402 . . . . . . .
. . . . . . . .
. . . . .
Vv15g0403 . . . . . . .
. . . . . . . .
. . . . .
Vv15g0404 . . . . . . .
. . . . . . . .
. . . . .
Vv15g0405 . . . . . . .
. . . . . . . .
. . . . .
Vv15g0406 . . . . . . .
. . . . . . . .
. . . . .
Vv15g0407 Tc01_g021730 . . . . .
. . . . . . . .
. . . . . .
Vv15g0408 . . . . . . .
. . . . . . . .
. . . . .
Vv15g0409 . . . . . . .
. . . . . . . .
. . . . .
Vv15g0410 Tc01_g021630 . . . . .
. . . . . . . .
. . . . . .
Vv15g0411 . . . . . . .
. . . . . . . .
. . . . .
Vv15g0412 . . . . . . .
. . . . . . . .
. . . . .
Vv15g0413 . . . . . .
Vv02g0512 . . . . . . .
. . . . . .
Vv15g0414 . . . . . . .
. . . . . . . .
. . . . .
Vv15g0415 . . . . . . .
. . . . . . . .
. . . . .
Vv15g0416 . . . . . . .
. . . . . . . .
. . . . .
Vv15g0417 . . . . . Gorai.
004G155500.1 . . . . . . .
. . . . . . .
Vv15g0418 . . . . . . .

```
        .        .       .        .       .        .       .        .
        .        .       .        .       .
Vv15g0419        Tc01_g037280    .       .        .       .        .
        .        .       .        .       .        .       .        .
        .        .       .        .       .        .
Vv15g0420        Tc01_g037270    .       .        .       .        .
        .        .       .        .       .        .       .        .
        .        .       .        .       .        .
Vv15g0421        Tc01_g037260    .       .        .       .
Gorai.004G155400.1       .        .       .        .       .        .
        .        .       .        .       .        .       .        .
Vv15g0422        .       .        .       .        .       .        .
        .        .       .        .       .        .       .        .
        .        .       .        .       .
Vv15g0423        Tc01_g037250    .       .        .       .        .
        .        .       .        .       .        .       .        .
        .        .       .        .       .        .
Vv15g0424        Tc01_g037240    .       .        .       .        .
        .        .       .        .       .        .       .        .
        .        .       .        .       .        .
Vv15g0425        .       .        .       .        .       .        .
        .        .       .        .       .        .       .        .
        .        .       .        .       .
Vv15g0426        .       .        .       .        .       .        .
        .        .       .        .       .        .       .        .
        .        .       .        .       .
Vv15g0427        .       .        .       .        .       .        .
        .        .       .        .       .        .       .        .
        .        .       .        .       .
Vv15g0428        Tc01_g004660    .       .        .       .        .
        .        .       .        .       .        .       .        .
        .        .       .        .       .        .
Vv15g0429        .       .        .       .        .       .
Vv02g0514        Tc03_g026260    Gorai.004G204600.1      .
Gorai.007G092400.1       Gorai.008G241800.1       .       .        .
        .        .       .        .       .
Vv15g0430        .       .        Gorai.008G114900.1      .        .
        .        .       .        .       .        .       .        .
        .        .       .        .       .        .       .
Vv15g0431        .       .        .       .        .       .        .
        .        .       .        .       .        .       .        .
        .        .       .        .       .
Vv15g0432        Tc01_g004600    .       Gorai.008G112900.1        .
        Gorai.003G021400.1       .       .        .       .        .
        .        .       .        .       .        .       .        .
        .        .
Vv15g0433        Tc01_g036870    .       .        .       .        .
        .        .       .        .       .        .       .        .
        .        .       .        .       .        .
Vv15g0434        Tc01_g036850    .       .        .       .        .
```

. . . . . . . .
. . . . . .
Vv15g0435 . . . . . . .
. . . . . . . .
. . . . .
Vv15g0436 Tc01_g004480 . Gorai.008G073100.1 .
. . . . . . . .
. . . . . . . .
Vv15g0437 Tc01_g036830 . . . . .
. . . . . . . .
. . . . . .
Vv15g0438 . . . . . . .
. . . . . . . .
. . . . .
Vv15g0439 . . . . . . .
. . . . . . . .
. . . . .
Vv15g0440 Tc01_g034130 . . . . .
. . . . . . . .
. . . . . .
Vv15g0441 Tc01_g036810 . . . .
Gorai.004G156500.1 . . . . . .
. . . . . . . .
Vv15g0442 . . . . . . .
. . . . . . . .
. . . . .
Vv15g0443 . . . . . . .
. . . . . . . .
. . . . .
Vv15g0444 Tc01_g036790 . . Gorai.001G171700.1
. . . . . . . .
. . . . . . . .
Vv15g0445 Tc01_g036780 . . . . .
. . . . . . . .
. . . . . .
Vv15g0446 Tc01_g036750 . . . .
Gorai.004G156600.1 . . . . . .
. . . . . . . .
Vv15g0447 Tc01_g036730 . . . . .
. . . . . . . .
. . . . . .
Vv15g0448 Tc01_g036720 . . . . .
. . . . . . . .
. . . . . .
Vv15g0449 Tc01_g036710 . . . . .
. . . . . . . .
. . . . . .
Vv15g0450 Tc01_g036700 . . Gorai.001G171500.1
. . . . . . . .
. . . . . . . .

```
Vv15g0451       Tc01_g036690    .       .       Gorai.001G171000.1
        .       .       Vv02g0528       Tc03_g026150    .       .
        Gorai.007G092100.1      Gorai.008G241000.1      .       .
        .       .       .       .       .       .
Vv15g0452       Tc01_g036680    .       .       .       .       .
        .       .       .       .       .       .       .       .
        .       .       .       .       .       .
Vv15g0453       Tc01_g036670    .       .       .       .       .
        .       .       .       .       .       .       .       .
        .       .       .       .       .       .
Vv15g0454       Tc01_g036660    .       .       .       .       .
        .       .       .       .       .       .       .       .
        .       .       .       .       .       .
Vv15g0455       .       .       .       .       .       .       .
        .       .       .       .       .       .       .       .
        .       .       .       .       .
Vv15g0456       .       .       .       .       .       .       .
        .       .       .       .       .       .       .       .
        .       .       .       .       .
Vv15g0457       .       .       .       Gorai.001G168700.1      .
        .       .       .       .       .       .       .       .
        .       .       .       .       .       .       .
Vv15g0458       .       .       .       .       .       .       .
        .       .       .       .       .       .       .       .
        .       .       .       .       .
Vv15g0459       Tc01_g036640    .       .       .       .       .
        .       .       .       .       .       .       .       .
        .       .       .       .       .       .
Vv15g0460       Tc01_g036630    .       .       .       .       .
        .       .       .       .       .       .       .       .
        .       .       .       .       .       .
Vv15g0461       Tc01_g036620    .       .       .       .
Gorai.004G156800.1      .       .       .       .       .       .
        .       .       .       .       .       .       .       .
Vv15g0462       Tc01_g036610    .       .       .       .       .
        .       .       .       .       .       .       .       .
        .       .       .       .       .       .
Vv15g0463       .       .       .       .       .       .       .
        .       .       .       .       .       .       .       .
        .       .       .       .       .
Vv15g0464       .       .       .       .       .       .       .
        .       .       .       .       .       .       .       .
        .       .       .       .       .
Vv15g0465       .       .       .       .       .       .       .
        .       .       .       .       .       .       .       .
        .       .       .       .       .
Vv15g0466       .       .       .       .       .       .       .
        .       .       .       .       .       .       .       .
        .       .       .       .       .
Vv15g0467       .       .       .       .       .       .       .
```

. . . . . . . .
. . . . .
Vv15g0468 . . . . . . .
. . . . . . . .
. . . . .
Vv15g0469 . . . . . . .
. . . . . . . .
. . . . .
Vv15g0470 . . . . . . .
. . . . . . . .
. . . . .
Vv15g0471 . . . . . . .
. . . . . . . .
. . . . .
Vv15g0472 Tc01_g033300 . . . . .
. . . . . . . .
. . . . . .
Vv15g0473 Tc01_g033310 . . . . .
. . . . . . . .
. . . . . .
Vv15g0474 Tc01_g033320 . . . . .
. . . . . . . .
. . . . . .
Vv15g0475 Tc01_g033340 . . . . .
. . . . . . . .
. . . . . .
Vv15g0476 Tc01_g033370 . . . . .
. . . . . . . .
. . . . . .
Vv15g0477 . . . . . . .
. . . . . . . .
. . . . .
Vv15g0478 Tc01_g033380 . . . . .
. . . . . . . .
. . . . . .
Vv15g0479 . . . . . . .
. . . . . . . .
. . . . .
Vv15g0480 Tc01_g033390 . . . . .
. . . . . . . .
. . . . . .
Vv15g0481 Tc01_g033440 . . . . .
. . . . . . . .
. . . . . .
Vv15g0482 Tc01_g033450 . . . . .
Vv16g0531 Tc03_g021990 . . Gorai.
007G114600.1 . . . . . . .
. .
Vv15g0483 Tc01_g033470 . . . . .
. . . . . . . .

. . . . . .
Vv15g0484 . . . . . . .
. . . . . . . .
. . . . .
Vv15g0485 . . . . . . .
. . . . . . . .
. . . . .
Vv15g0486 Tc01_g033480 . . . . .
. . . . . . . .
. . . . . .
Vv15g0487 . . . . . . .
. . . . . . . .
. . . . .
Vv15g0488 . . . . . . .
. . . . . . . .
. . . . .
Vv15g0489 . . . . . . .
. . . . . . . .
. . . . .
Vv15g0490 . . . . . . .
. . . . . . . .
. . . . .
Vv15g0491 Tc01_g033490 . . . . .
. . . . . . . .
. . . . . .
Vv15g0492 . . . . . . .
. . . . . . . .
. . . . .
Vv15g0493 . . . . . . .
. . . . . . . .
. . . . .
Vv15g0494 . . . . . . .
. . . . . . . .
. . . . .
Vv15g0495 . . . . . . .
. . . . . . . .
. . . . .
Vv15g0496 . . . . . . .
. . . . . . . .
. . . . .
Vv15g0497 . . . . . . .
. . . . . . . .
. . . . .
Vv15g0498 Tc01_g033520 . . . . .
. . . . . . . .
. . . . . .
Vv15g0499 Tc01_g033530 . . . . .
Vv02g0306 . . . . . .
. . . . . . .
Vv15g0500 Tc01_g033540 . . . . .

. . . . . . . .
. . . . . .
Vv15g0501 Tc01_g033550 . . . . .
. . . . . . . .
. . . . . .
Vv15g0502 Tc01_g033560 . . . .
Gorai.004G159700.1 . . . . . .
. . . . . . . .
Vv15g0503 . . . . . . .
. . . . . . . .
. . . . .
Vv15g0504 Tc01_g033570 . . . . .
. . . . . . . .
. . . . . .
Vv15g0505 . . . . . . .
. . . . . . Vv16g0544
Tc03_g021850 . . . Gorai.008G260800.1
.
Vv15g0506 Tc01_g033580 . . . . .
Vv02g0303 . . . . Gorai.
008G260800.1 . . . . . . .
.
Vv15g0507 . . . . . . .
. . . . . . . .
. . . . .
Vv15g0508 . . . . . . .
. . . . . . Vv16g0545
. . . . Gorai.008G260600.1 .
Vv15g0509 . . . . . . .
. . . . . . . .
. . . . .
Vv15g0510 Tc01_g033590 . . . . .
Vv02g0302 . . . . Gorai.
008G264900.1 . . . . . . .
.
Vv15g0511 Tc01_g033600 . . . . .
. . . . . . . .
. . . . . .
Vv15g0512 Tc01_g033610 . . . . .
. . . . . . . .
. . . . . .
Vv15g0513 . . . . . .
Vv02g0300 . Gorai.004G128400.1 . . .
. . . . . . . .
Vv15g0514 Tc01_g033620 . . . . .
. . . . . . . .
. . . . . .
Vv15g0515 Tc01_g033640 . . . . .
. . . . . . .
Vv16g0549 Tc03_g021790 . . Gorai.007G017200.1

. .
Vv15g0516 Tc01_g033650 . . . . .
. . . . . . .
Vv16g0550 Tc03_g021780 . . Gorai.007G113000.1
Gorai.008G265100.1 .
Vv15g0517 Tc01_g033660 . . . . .
. . . . . . . .
. . . . . .
Vv15g0518 . . . . . . .
. . . . . . . .
. . . . .
Vv15g0519 . . . . . . .
. . . . . . . .
. . . . .
Vv15g0520 . . . . . . .
. . . . . . . .
. . . . .
Vv15g0521 Tc01_g033670 . . . . .
. . . . . . . .
. . . . . .
Vv15g0522 . . . . . . .
. . . . . . . .
. . . . .
Vv15g0523 Tc01_g033680 . . . . .
. . . . . . . .
. . . . . .
Vv15g0524 . . . . . . .
. . . . . . . .
. . . . .
Vv15g0525 . . . . . . .
. . . . . . . .
. . . . .
Vv15g0526 Tc01_g033730 . . . . .
. . . . . . . .
. . . . . .
Vv15g0527 Tc01_g033750 . . . . .
Vv02g0278 . Gorai.004G129100.1 .
Gorai.007G112600.1 . Gorai.006G205200.1 . .
. . . . .
Vv15g0528 Tc01_g033780 . . . . .
. . . . . . . .
. . . . . .
Vv15g0529 . . . . . . .
. . . . . . . .
. . . . .
Vv15g0530 . . . . . . .
. . . . . . . .
. . . . .
Vv15g0531 Tc01_g033790 . . . . .
Vv02g0275 . . . Gorai.007G019100.1

```
          Gorai.008G259600.1            .         Vv16g0567
Tc03_g021610        .         .         .         .         .
Vv15g0532           Tc01_g033820        .         .         .         .         .
          .         .         .         .         .         .         .         .
          .         .         .         .         .         .
Vv15g0533           .         .         .         .         .         .         .
          .         .         .         .         .         .         .         .
          .         .         .         .         .
Vv15g0534           Tc01_g033830        .         .         .         .         .
          .         .         .         .         .         .         .         .
          .         .         .         .         .         .
Vv15g0535           Tc01_g033850        .         .         .         .         .
          .         .         .         .         .         .         .         .
          .         .         .         .         .         .
Vv15g0536           .         .         .         .         .         .         .
          .         .         .         .         .         .         .         .
          .         .         .         .         .
Vv15g0537           .         .         .         .         .         .         .
          .         .         .         .         .         .         .         .
          .         .         .         .         .
Vv15g0538           .         .         .         .         .         .         .
          .         .         .         .         .         .         .         .
          .         .         .         .         .
Vv15g0539           Tc01_g033860        .         .         .         .         .
          .         .         .         .         .         .         .         .
          .         .         .         .         .         .
Vv15g0540           Tc01_g033880        .         .         .         .         .
          .         .         .         .         .         .         .         .
          .         .         .         .         .         .
Vv15g0541           Tc01_g033890        .         .         .         .         .
          .         .         .         .         .         .         .
Vv16g0572           Tc03_g021570        .         Gorai.003G122900.1            .
          .         .
Vv15g0542           Tc01_g033900        .         .         .         .         .
          .         .         .         .         .         .         .         .
          .         .         .         .         .         .
Vv15g0543           .         .         .         .         .         .         .
          .         .         .         .         .         .         .         .
          .         .         .         .         .
Vv15g0544           .         .         .         .         .         .         .
          .         .         .         .         .         .         .         .
          .         .         .         .         .
Vv15g0545           Tc01_g033910        .         .         .         .         .
          .         .         .         .         .         .         .         .
          .         .         .         .         .         .
Vv15g0546           .         .         .         .         .         .         .
          .         .         .         .         .         .         .         .
          .         .         .         .         .
Vv15g0547           Tc01_g033920        .         .         .         .         .
          .         .         .         .         .         .         .         .
```

. . . . . .
Vv15g0548 Tc01_g033940 . . . . .
. . . . . . . .
. . . . . .
Vv15g0549 Tc01_g033960 . . . . .
. . . . . . . .
. . . . . .
Vv15g0550 Tc01_g033970 . . . . .
. . . . . . . .
. . . . . .
Vv15g0551 . . . . . . .
. . . . . . . .
. . . . .
Vv15g0552 Tc01_g033980 . . . . .
Vv02g0264 Tc03_g029890 . . Gorai.
007G111400.1 Gorai.008G258900.1 . Vv16g0578
Tc03_g021520 . . Gorai.007G111400.1 Gorai.
008G258900.1 .
Vv15g0553 Tc01_g034000 . . . . .
. . . . . . . .
. . . . . .
Vv15g0554 . . . . . .
Vv16g0580 Tc03_g021500 . . Gorai.007G016700.1
Gorai.008G266000.1 . . . . .
. . .
Vv15g0555 Tc01_g034020 . . . . .
. . . . . . . .
. . . . . .
Vv15g0556 Tc01_g034030 . . . . .
. . . . . . . .
. . . . . .
Vv15g0557 . . . . . . .
. . . . . . . .
. . . . .
Vv15g0558 Tc01_g034050 . . . . .
. . . . . . . .
. . . . . .
Vv15g0559 Tc01_g034060 . . . . .
. . . . . . . .
. . . . . .
Vv15g0560 Tc01_g034070 . . . . .
. . . . . . . .
. . . . . .
Vv15g0561 . . . . . . .
. . . . . . . .
. . . . .
Vv16g0001 . . . . . . .
. . . . . . . .
. . . . .
Vv16g0002 . . . . . . .

| | | | | | | | |
|---|---|---|---|---|---|---|---|
| Vv16g0003 | . | . | . | . | . | . | . |
| Vv16g0004 | . | . | . | . | . | . | . |
| Vv16g0005 | . | . | . | . | . | . | . |
| Vv16g0006 | . | . | . | . | . | . | . |
| Vv16g0007 | Tc03_g024460 | | . | . | . | . | . |
| Vv16g0008 | . | . | . | . | . | . | . |
| Vv16g0009 | Tc03_g026200 | | . | . | . | . | . |
| Vv16g0010 | . | . | . | . | . | . | . |
| Vv16g0011 | . | . | . | . | . | . | . |
| Vv16g0012 | . | . | . | . | . | . | . |
| Vv16g0013 | . | . | . | . | . | . | . |
| Vv16g0014 | . | . | . | . | . | . | . |
| Vv16g0015 | . | . | . | . | . | . | . |
| Vv16g0016 | Tc03_g023800 | | . | . | . | . | . |
| Vv16g0017 | . | . | . | . | . | . | . |
| Vv16g0018 | . | . | . | . | . | . | . |

Vv16g0019 . . . . . . .
. . . . . . . .
. . . . .
Vv16g0020 . . . . . . .
. . . . . . . .
. . . . .
Vv16g0021 . . . . . . .
. . . . . . . .
. . . . .
Vv16g0022 . . . . . . .
. . . . . . . .
. . . . .
Vv16g0023 . . . . . . .
. . . . . . . .
. . . . .
Vv16g0024 . . . . . . .
. . . . . . . .
. . . . .
Vv16g0025 Tc03_g023790 . . . . .
. . . . . . . .
. . . . . .
Vv16g0026 . . . . . . .
. . . . . . . .
. . . . .
Vv16g0027 . . . . . . .
. . . . . . . .
. . . . .
Vv16g0028 . . . . . . .
. . . . . . . .
. . . . .
Vv16g0029 Tc03_g024420 . . Gorai.007G029700.1
. . . . . . . .
. . . . . . . .
Vv16g0030 . . . . . . .
. . . . . . . .
. . . . .
Vv16g0031 . . . . . . .
. . . . . . . .
. . . . .
Vv16g0032 Tc03_g013680 . Gorai.003G107300.1 .
. . . . . . . .
. . . . . . . .
Vv16g0033 . . . . . . .
. . . . . . . .
. . . . .
Vv16g0034 . . . . . . .
. . . . . . . .
. . . . .
Vv16g0035 . . . . . . .
. . . . . . . .

. . . . .
Vv16g0036 . . . . . . .
. . . . . . . .
. . . . .
Vv16g0037 . . . . . . .
. . . . . . . .
. . . . .
Vv16g0038 . . . . . . .
. . . . . . . .
. . . . .
Vv16g0039 . . . . . . .
. . . . . . . .
. . . . .
Vv16g0040 . . . . . . .
. . . . . . . .
. . . . .
Vv16g0041 Tc03_g013700 . Gorai.003G107400.1 .
Gorai.008G202400.1 . . . . .
. . . . . . . .
. .
Vv16g0042 . . . . . . .
. . . . . . . .
. . . . .
Vv16g0043 . . . . . . .
. . . . . . . .
. . . . .
Vv16g0044 Tc03_g013710 . . . Gorai.
008G202500.1 . . . . . . .
. . . . . . . .
Vv16g0045 Tc03_g025810 . . . . .
. . . . . . . .
. . . . . .
Vv16g0046 . . . . . . .
. . . . . . . .
. . . . .
Vv16g0047 . . . . . . .
. . . . . . . .
. . . . .
Vv16g0048 Tc03_g013740 . . . . .
. . . . . . . .
. . . . . .
Vv16g0049 . . . . . . .
. . . . . . . .
. . . . .
Vv16g0050 . . . . . . .
. . . . . . . .
. . . . .
Vv16g0051 Tc03_g025790 . . . . .
. . . . . . . .
. . . . . .

| | | | | | | | |
|---|---|---|---|---|---|---|---|
| Vv16g0052 | . | . | . | . | . | . | . |
| . | . | . | . | . | . | . | . |
| . | . | . | . | . | | | |
| Vv16g0053 | . | . | . | . | . | . | . |
| . | . | . | . | . | . | . | . |
| . | . | . | . | . | | | |
| Vv16g0054 | Tc03_g025780 | | . | . | . | . | . |
| . | . | . | . | . | . | . | . |
| . | . | . | . | . | . | | |
| Vv16g0055 | . | . | . | . | . | . | . |
| . | . | . | . | . | . | . | . |
| . | . | . | . | . | | | |
| Vv16g0056 | . | . | . | . | . | . | . |
| . | . | . | . | . | . | . | . |
| . | . | . | . | . | | | |
| Vv16g0057 | . | . | . | . | . | . | . |
| . | . | . | . | . | . | . | . |
| . | . | . | . | . | | | |
| Vv16g0058 | Tc03_g025770 | | . | . | . | . | . |
| . | . | . | . | . | . | . | . |
| . | . | . | . | . | . | | |
| Vv16g0059 | . | . | . | . | . | . | . |
| . | . | . | . | . | . | . | . |
| . | . | . | . | . | | | |
| Vv16g0060 | . | . | . | . | . | . | . |
| . | . | . | . | . | . | . | . |
| . | . | . | . | . | | | |
| Vv16g0061 | Tc03_g025760 | | . | . | . | . | . |
| . | . | . | . | . | . | . | . |
| . | . | . | . | . | . | | |
| Vv16g0062 | . | . | . | . | . | . | . |
| . | . | . | . | . | . | . | . |
| . | . | . | . | . | | | |
| Vv16g0063 | Tc03_g025740 | | . | . | . | . | . |
| . | . | . | . | . | . | . | . |
| . | . | . | . | . | . | | |
| Vv16g0064 | . | . | . | . | . | . | . |
| . | . | . | . | . | . | . | . |
| . | . | . | . | . | | | |
| Vv16g0065 | . | . | . | . | . | . | . |
| . | . | . | . | . | . | . | . |
| . | . | . | . | . | | | |
| Vv16g0066 | . | . | . | . | . | . | . |
| . | . | . | . | . | . | . | . |
| . | . | . | . | . | | | |
| Vv16g0067 | . | . | . | . | . | . | . |
| . | . | . | . | . | . | . | . |
| . | . | . | . | . | | | |
| Vv16g0068 | . | . | . | . | . | . | . |
| . | . | . | . | . | . | . | . |

. . . . .
Vv16g0069 . . . . . . .
. . . . . . . .
. . . . .
Vv16g0070 . . . . . . .
. . . . . . . .
. . . . .
Vv16g0071 Tc03_g030330 . . . Gorai.
008G222500.1 . . . . . . .
. . . . . . . .
Vv16g0072 . . . . . . .
. . . . . . . .
. . . . .
Vv16g0073 . . . . . . .
. . . . . . . .
. . . . .
Vv16g0074 . . . . . . .
. . . . . . . .
. . . . .
Vv16g0075 Tc03_g022880 . Gorai.003G077000.1 .
. . . . . . . .
. . . . . . . .
Vv16g0076 . . . . . . .
. . . . . . . .
. . . . .
Vv16g0077 . . . . . . .
. . . . . . . .
. . . . .
Vv16g0078 . . . . . . .
. . . . . . . .
. . . . .
Vv16g0079 . . . . . . .
. . . . . . . .
. . . . .
Vv16g0080 . . . . . . .
. . . . . . . .
. . . . .
Vv16g0081 . . . . . . .
. . . . . . . .
. . . . .
Vv16g0082 . . . . . . .
. . . . . . . .
. . . . .
Vv16g0083 . . . . . . .
. . . . . . . .
. . . . .
Vv16g0084 . . . . . . .
. . . . . . . .
. . . . .
Vv16g0085 . . . . . . .

| | | | | | | | |
|---|---|---|---|---|---|---|---|
| . | . | . | . | . | . | . | . |
| . | . | . | . | . | | | |
| Vv16g0086 | . | . | . | . | . | . | . |
| . | . | . | . | . | . | . | . |
| . | . | . | . | . | | | |
| Vv16g0087 | Tc03_g003090 | | . | . | . | . | . |
| . | . | . | . | . | . | . | . |
| . | . | . | . | . | . | | |
| Vv16g0088 | . | . | . | . | . | . | . |
| . | . | . | . | . | . | . | . |
| . | . | . | . | . | | | |
| Vv16g0089 | . | . | . | . | . | . | . |
| . | . | . | . | . | . | . | . |
| . | . | . | . | . | | | |
| Vv16g0090 | . | . | . | . | . | . | . |
| . | . | . | . | . | . | . | . |
| . | . | . | . | . | | | |
| Vv16g0091 | . | . | . | . | . | . | . |
| . | . | . | . | . | . | . | . |
| . | . | . | . | . | | | |
| Vv16g0092 | . | . | . | . | . | . | . |
| . | . | . | . | . | . | . | . |
| . | . | . | . | . | | | |
| Vv16g0093 | . | . | . | . | . | . | . |
| . | . | . | . | . | . | . | . |
| . | . | . | . | . | | | |
| Vv16g0094 | . | . | . | . | . | . | . |
| . | . | . | . | . | . | . | . |
| . | . | . | . | . | | | |
| Vv16g0095 | Tc03_g030740 | | . | . | . | . | . |
| . | . | . | . | . | . | . | . |
| . | . | . | . | . | . | | |
| Vv16g0096 | . | . | . | . | . | . | . |
| . | . | . | . | . | . | . | . |
| . | . | . | . | . | | | |
| Vv16g0097 | . | . | . | . | . | . | . |
| . | . | . | . | . | . | . | . |
| . | . | . | . | . | | | |
| Vv16g0098 | . | . | . | . | . | . | . |
| . | . | . | . | . | . | . | . |
| . | . | . | . | . | | | |
| Vv16g0099 | . | . | . | . | . | . | . |
| . | . | . | . | . | . | . | . |
| . | . | . | . | . | | | |
| Vv16g0100 | . | . | . | . | . | . | . |
| . | . | . | . | . | . | . | . |
| . | . | . | . | . | | | |
| Vv16g0101 | . | . | . | . | . | . | . |
| . | . | . | . | . | . | . | . |
| . | . | . | . | . | | | |

Vv16g0102 . . . . . . .
. . . . . . . .
. . . . .
Vv16g0103 Tc03_g030750 . . . . .
. . . . . . . .
. . . . . .
Vv16g0104 . . . . . . .
. . . . . . . .
. . . . .
Vv16g0105 . . . . . . .
. . . . . . . .
. . . . .
Vv16g0106 . . . . . . .
. . . . . . . .
. . . . .
Vv16g0107 . . . . . . .
. . . . . . . .
. . . . .
Vv16g0108 . . . . . . .
. . . . . . . .
. . . . .
Vv16g0109 Tc03_g003550 . . . . .
. . . . . . . .
. . . . . .
Vv16g0110 . . . . . . .
. . . . . . . .
. . . . .
Vv16g0111 . . . . . . .
. . . . . . . .
. . . . .
Vv16g0112 . . . . . . .
. . . . . . . .
. . . . .
Vv16g0113 . . . . . . .
. . . . . . . .
. . . . .
Vv16g0114 Tc03_g022750 . . . . .
. . . . . . . .
. . . . . .
Vv16g0115 . . . . . . .
. . . . . . . .
. . . . .
Vv16g0116 . . . . . . .
. . . . . . . .
. . . . .
Vv16g0117 . . . . . . .
. . . . . . . .
. . . . .
Vv16g0118 . . . . . . .
. . . . . . . .

.	.	.	.	.
Vv16g0119	.	.	.	.	Gorai.008G226700.1
.	Vv02g0747	Tc03_g012040	.	.	.
.	.	.	.	.	.	.	.
.
Vv16g0120	.	.	.	.	.	.	.
.	.	.	.	.	.	.	.
.	.	.	.	.
Vv16g0121	.	.	.	.	.	.	.
.	.	.	.	.	.	.	.
.	.	.	.	.
Vv16g0122	.	.	.	.	.	.	.
.	.	.	.	.	.	.	.
.	.	.	.	.
Vv16g0123	.	.	.	.	.	.	.
.	.	.	.	.	.	.	.
.	.	.	.	.
Vv16g0124	.	.	.	.	.	.	.
.	.	.	.	.	.	.	.
.	.	.	.	.
Vv16g0125	.	.	.	.	.	.	.
.	.	.	.	.	.	.	.
.	.	.	.	.
Vv16g0126	.	.	.	.	.	.	.
.	.	.	.	.	.	.	.
.	.	.	.	.
Vv16g0127	.	.	.	Gorai.007G032400.1
Gorai.008G226100.1	.	Vv02g0761	.	Gorai.
004G163500.1	Gorai.003G183000.1	Gorai.007G032400.1
Gorai.008G226100.1	Gorai.006G217100.1	.	.	.
.	.	.	.
Vv16g0128	.	.	.	.	.	.	.
.	.	.	.	.	.	.	.
.	.	.	.	.
Vv16g0129	.	.	.	.	.	.	.
.	.	.	.	.	.	.	.
.	.	.	.	.
Vv16g0130	.	.	.	Gorai.007G032800.1
Gorai.008G225600.1	.	Vv02g0774	.	.	.
Gorai.007G032800.1	Gorai.008G225600.1	.	.
.	.	.	.	.	.
Vv16g0131	.	.	.	.	.	.	.
.	.	.	.	.	.	.	.
.	.	.	.	.
Vv16g0132	.	.	.	.	.	.	.
.	.	.	.	.	.	.	.
.	.	.	.	.
Vv16g0133	.	.	.	.	.	.	.
.	.	.	.	.	.	.	.
.	.	.	.	.

Vv16g0134 . . . . . . .
. . . . . . . .
. . . . .
Vv16g0135 . . . . . . .
. . . . . . . .
. . . . .
Vv16g0136 . . . . . . .
. . . . . . . .
. . . . .
Vv16g0137 . . . . . . .
. . . . . . . .
. . . . .
Vv16g0138 . . . . . . .
. . . . . . . .
. . . . .
Vv16g0139 . . . . . . .
. . . . . . . .
. . . . .
Vv16g0140 . . . . . . .
. . . . . . . .
. . . . .
Vv16g0141 . . . . . .
Vv02g0778 . Gorai.004G184100.1 . . .
. . . . . . . .
Vv16g0142 . . . . . . .
. . . . . . . .
. . . . .
Vv16g0143 . . . . . . .
. . . . . . . .
. . . . .
Vv16g0144 . . . . . . .
. . . . . . . .
. . . . .
Vv16g0145 . . . Gorai.007G033100.1 .
. Vv02g0785 Tc03_g029660 . .
Gorai.007G033100.1 . Gorai.006G217500.1 . .
. . . . .
Vv16g0146 Tc03_g029670 . . . . .
. . . . . . . .
. . . . . .
Vv16g0147 . . . . . . .
. . . . . . . .
. . . . .
Vv16g0148 Tc03_g029680 . . . . .
. . . . . . . .
. . . . . .
Vv16g0149 Tc03_g029690 . . . . .
. . . . . . . .
. . . . . .
Vv16g0150 . . . . . . .

. . . . . . . .
. . . . .
Vv16g0151 . . . . . . .
. . . . . . . .
. . . . .
Vv16g0152 Tc03_g018360 . Gorai.006G064000.1 .
. . . . . . . .
. . . . . . . .
Vv16g0153 . . . . . . .
. . . . . . . .
. . . . .
Vv16g0154 . . . . . . .
. . . . . . . .
. . . . .
Vv16g0155 . . . . . . .
. . . . . . . .
. . . . .
Vv16g0156 . . . . . . .
. . . . . . . .
. . . . .
Vv16g0157 . . . . . . .
. . . . . . . .
. . . . .
Vv16g0158 . . . . . . .
. . . . . . . .
. . . . .
Vv16g0159 . . . . . . .
. . . . . . . .
. . . . .
Vv16g0160 . . . . . . .
. . . . . . . .
. . . . .
Vv16g0161 Tc03_g029760 . . . Gorai.
008G225200.1 . . . . . . .
. . . . . . . .
Vv16g0162 . . . Gorai.007G033300.1 .
. Vv02g0806 Tc03_g029760 . Gorai.
003G183900.1 Gorai.007G033300.1 Gorai.008G225200.1 .
. . . . . . .
Vv16g0163 Tc03_g029800 . . . Gorai.
008G224800.1 . Vv02g0810 Tc03_g029800 Gorai.
004G183100.1 Gorai.003G184100.1 . Gorai.008G224800.1
Gorai.006G217600.1 . . . . .
. .
Vv16g0164 Tc03_g029810 . . . Gorai.
008G224700.1 . Vv02g0812 Tc03_g029810 .
Gorai.003G184200.1 . Gorai.008G224700.1 . .
. . . . . .
Vv16g0165 . . . . . . .
. . . . . . . .

. . . . .
Vv16g0166 Tc03_g029870 . . . Gorai.
008G224600.1 . . . . . . .
. . . . . . . .
Vv16g0167 Tc03_g029910 . . . Gorai.
008G224500.1 . . . . . . .
. . . . . . . .
Vv16g0168 . . . . Gorai.008G224400.1
. Vv02g0824 Tc03_g030440 . . .
Gorai.008G224400.1 . . . . .
. . .
Vv16g0169 Tc03_g029920 . . . . .
. . . . . . . .
. . . . . .
Vv16g0170 . . . . . . .
. . . . . . . .
. . . . .
Vv16g0171 . . . . . . .
. . . . . . . .
. . . . .
Vv16g0172 . . . . . . .
. . . . . . . .
. . . . .
Vv16g0173 . . . . . . .
. . . . . . . .
. . . . .
Vv16g0174 . . . . . . .
. . . . . . . .
. . . . .
Vv16g0175 . . . . . . .
. . . . . . . .
. . . . .
Vv16g0176 . . . . . . .
. . . . . . . .
. . . . .
Vv16g0177 . . . . . . .
. . . . . . . .
. . . . .
Vv16g0178 . . . . . . .
. . . . . . . .
. . . . .
Vv16g0179 . . . . . . .
. . . . . . . .
. . . . .
Vv16g0180 . . . . . . .
. . . . . . . .
. . . . .
Vv16g0181 . . . . . . .
. . . . . . . .
. . . . .

Vv16g0182 . . . . . . .
. . . . . . .
. . . . .
Vv16g0183 Tc03_g030010 . . . Gorai.
008G223700.1 . . . . . . .
. . . . . . .
Vv16g0184 . . . . . . .
. . . . . . .
. . . . .
Vv16g0185 . . . . . . .
. . . . . . .
. . . . .
Vv16g0186 Tc03_g030140 . . Gorai.007G033600.1
. . . . . . .
. . . . . . .
Vv16g0187 . . . . . . .
. . . . . . .
. . . . .
Vv16g0188 . . . . . . .
. . . . . . .
. . . . .
Vv16g0189 . . . . . . .
. . . . . . .
. . . . .
Vv16g0190 . . . . . . .
. . . . . . .
. . . . .
Vv16g0191 Tc03_g018230 . . . . .
. . . . . . .
. . . . . .
Vv16g0192 Tc03_g030350 . . . Gorai.
008G219800.1 . . . . . . .
. . . . . . .
Vv16g0193 . . . . . . .
. . . . . . .
. . . . .
Vv16g0194 . . . . . . .
. . . . . . .
. . . . .
Vv16g0195 . . . . . . .
. . . . . . .
. . . . .
Vv16g0196 . . . . . . .
. . . . . . .
. . . . .
Vv16g0197 . . . . . . .
. . . . . . .
. . . . .
Vv16g0198 Tc03_g018140 Gorai.001G048500.1 . .
. . . . . . .

| | | | | | | | |
|---|---|---|---|---|---|---|---|
| Vv16g0199 | . | . | . | . | . | . | . |
| Vv16g0200 | . | . | . | . | . | . | . |
| Vv16g0201 | Tc03_g018020 | | . | . | . | . | . |
| Vv16g0202 | . | . | . | . | . | . | . |
| Vv16g0203 | . | . | . | . | . | . | . |
| Vv16g0204 | . | . | . | . | . | . | . |
| Vv16g0205 | . | . | . | . | . | . | . |
| Vv16g0206 | . | . | . | . | . | . | . |
| Vv16g0207 | . | . | . | . | . | . | . |
| Vv16g0208 | . | . | . | . | . | . | . |
| Vv16g0209 | . | . | . | . | . | . | . |
| Vv16g0210 | . | . | . | . | . | . | . |
| Vv16g0211 | . | . | . | . | . | . | . |
| Vv16g0212 | . | . | . | . | . | . | . |
| Vv16g0213 | . | . | . | . | . | . | . |
| Vv16g0214 | . | . | . | . | . | . | . |
| Vv16g0215 | . | . | . | . | . | . | . |

| | | | | | | | |
|---|---|---|---|---|---|---|---|
| . | . | . | . | . | . | . | . |
| . | . | . | . | . | | | |
| Vv16g0216 | . | . | . | . | . | . | . |
| . | . | . | . | . | . | . | . |
| . | . | . | . | . | | | |
| Vv16g0217 | . | . | . | . | . | . | . |
| . | . | . | . | . | . | . | . |
| . | . | . | . | . | | | |
| Vv16g0218 | . | . | . | . | . | . | . |
| . | . | . | . | . | . | . | . |
| . | . | . | . | . | | | |
| Vv16g0219 | . | . | . | . | . | . | . |
| . | . | . | . | . | . | . | . |
| . | . | . | . | . | | | |
| Vv16g0220 | . | . | . | . | . | . | . |
| . | . | . | . | . | . | . | . |
| . | . | . | . | . | | | |
| Vv16g0221 | . | . | . | . | . | . | . |
| . | . | . | . | . | . | . | . |
| . | . | . | . | . | | | |
| Vv16g0222 | . | . | . | . | . | . | . |
| . | . | . | . | . | . | . | . |
| . | . | . | . | . | | | |
| Vv16g0223 | Tc03_g030420 | | Gorai.001G052000.1 | | | . | . |
| . | . | . | . | . | . | . | . |
| . | . | . | . | . | . | . | . |
| Vv16g0224 | . | . | . | . | . | . | . |
| . | . | . | . | . | . | . | . |
| . | . | . | . | . | | | |
| Vv16g0225 | . | . | . | . | . | . | . |
| . | . | . | . | . | . | . | . |
| . | . | . | . | . | | | |
| Vv16g0226 | . | . | . | . | . | . | . |
| . | . | . | . | . | . | . | . |
| . | . | . | . | . | | | |
| Vv16g0227 | . | . | . | . | . | . | . |
| . | . | . | . | . | . | . | . |
| . | . | . | . | . | | | |
| Vv16g0228 | . | . | . | . | . | . | . |
| . | . | . | . | . | . | . | . |
| . | . | . | . | . | | | |
| Vv16g0229 | . | . | . | . | . | . | . |
| . | . | . | . | . | . | . | . |
| . | . | . | . | . | | | |
| Vv16g0230 | . | . | . | . | . | . | . |
| . | . | . | . | . | . | . | . |
| . | . | . | . | . | | | |
| Vv16g0231 | . | . | . | . | . | . | . |
| . | . | . | . | . | . | . | . |
| . | . | . | . | . | | | |

Vv16g0232 . . . . . . .
. . . . . . . .
. . . . .
Vv16g0233 . . . . . . .
. . . . . . . .
. . . . .
Vv16g0234 . . . . . . .
. . . . . . . .
. . . . .
Vv16g0235 . . . . . . .
. . . . . . . .
. . . . .
Vv16g0236 . . . . . . .
. . . . . . . .
. . . . .
Vv16g0237 . . . . . . .
. . . . . . . .
. . . . .
Vv16g0238 . . . . . . .
. . . . . . . .
. . . . .
Vv16g0239 . . . . . . .
. . . . . . . .
. . . . .
Vv16g0240 . . . . . . .
. . . . . . . .
. . . . .
Vv16g0241 . . . . . . .
. . . . . . . .
. . . . .
Vv16g0242 . . . . . . .
. . . . . . . .
. . . . .
Vv16g0243 . . . . . . .
. . . . . . . .
. . . . .
Vv16g0244 . . . . . . .
. . . . . . . .
. . . . .
Vv16g0245 . Gorai.001G052100.1 . . .
. . . . . . . .
. . . . . . .
Vv16g0246 Tc03_g024210 . . . Gorai.
008G231200.1 . . . . . . .
. . . . . . . .
Vv16g0247 . Gorai.001G052200.1 . . .
. . . . . . . .
. . . . . . .
Vv16g0248 . . . . . . .
. . . . . . . .

. . . . .
Vv16g0249 Tc03_g024200 Gorai.001G052300.1 . .
Gorai.008G231100.1 . . . . .
. . . . . . . .
. .
Vv16g0250 . . . . . . .
. . . . . . . .
. . . . .
Vv16g0251 Tc03_g024190 . . . . .
. . . . . . . .
. . . . . .
Vv16g0252 . . . . . . .
. . . . . . . .
. . . . .
Vv16g0253 . . . . . . .
. . . . . . . .
. . . . .
Vv16g0254 . . . . . . .
. . . . . . . .
. . . . .
Vv16g0255 . . . . Gorai.008G231000.1
. . . . . . . .
. . . . . . .
Vv16g0256 . . . . . . .
. . . . . . . .
. . . . .
Vv16g0257 . . . . . . .
. . . . . . . .
. . . . .
Vv16g0258 . . . . Gorai.008G213200.1
. . . . . . . .
. . . . . . .
Vv16g0259 . . . . . . .
. . . . . . . .
. . . . .
Vv16g0260 . Gorai.001G052500.1 . .
Gorai.008G213100.1 . . . . . .
. . . . . . . .
.
Vv16g0261 . . . . . . .
. . . . . . . .
. . . . .
Vv16g0262 . . . . . . .
. . . . . . . .
. . . . .
Vv16g0263 . . . . . . .
. . . . . . . .
. . . . .
Vv16g0264 . . . . . . .
. . . . . . . .

| | | | | | | | |
|---|---|---|---|---|---|---|---|
| Vv16g0265 | . | . | . | . | . | . | . |
| Vv16g0266 | . | . | . | . | . | . | . |
| Vv16g0267 | . | . | . | . | . | . | . |
| Vv16g0268 | . | . | . | . | . | . | . |
| Vv16g0269 | . | . | . | . | . | . | . |
| Vv16g0270 | . | . | . | . | . | . | . |
| Vv16g0271 | . | . | . | . | Gorai.008G230500.1 | | |
| Vv16g0272 | . | . | . | . | . | . | . |
| Vv16g0273 | . | . | . | . | . | . | . |
| Vv16g0274 | . | . | . | . | . | . | . |
| Vv16g0275 | Tc03_g016120 | | . | . | . | . | . |
| Vv16g0276 | . | . | . | . | . | . | . |
| Vv16g0277 | . | . | . | . | . | . | . |
| Vv16g0278 | . | . | . | . | . | . | . |
| Vv16g0279 | . | . | . | . | . | . | . |
| Vv16g0280 | . | . | . | . | . | . | . |
| Vv16g0281 | . | . | . | . | . | . | . |

| | | | | | | | | |
|---|---|---|---|---|---|---|---|---|
| | . | . | . | . | . | . | . | . |
| Vv16g0282 | . | . | . | . | . | . | . | . |
| Vv16g0283 | . | . | . | . | . | . | . | . |
| Vv16g0284 | . | . | . | . | . | . | . | . |
| Vv16g0285 | . | . | . | . | . | . | . | . |
| Vv16g0286 | . | . | . | . | . | . | . | . |
| Vv16g0287 | . | . | . | . | . | . | . | . |
| Vv16g0288 | . | . | . | . | . | . | . | . |
| Vv16g0289 | . | . | . | . | . | . | . | . |
| Vv16g0290 | . | . | . | . | . | . | . | . |
| Vv16g0291 | . | . | . | . | . | . | . | . |
| Vv16g0292 | . | . | . | . | . | . | . | . |
| Vv16g0293 | . | . | . | . | . | . | . | . |
| Vv16g0294 | . | . | . | . | . | . | . | . |
| Vv16g0295 | . | . | . | . | . | . | . | . |
| Vv16g0296 | . | . | . | . | . | . | . | . |
| Vv16g0297 | . | . | . | . | . | . | . | . |

Vv16g0298 . . . . . . .
. . . . . . . .
. . . . .
Vv16g0299 . . . . . . .
. . . . . . . .
. . . . .
Vv16g0300 Tc03_g018250 . . . . .
. . . . . . . .
. . . . . .
Vv16g0301 . . . . . . .
. . . . . . . .
. . . . .
Vv16g0302 . . . . . . .
. . . . . . . .
. . . . .
Vv16g0303 . . . . . . .
. . . . . . . .
. . . . .
Vv16g0304 . . . . . . .
. . . . . . . .
. . . . .
Vv16g0305 . . . . . . .
. . . . . . . .
. . . . .
Vv16g0306 Tc03_g018240 . . . . .
. . . . . . . .
. . . . . .
Vv16g0307 . . . . . . .
. . . . . . . .
. . . . .
Vv16g0308 . . . . . . .
. . . . . . . .
. . . . .
Vv16g0309 . . . . . . .
. . . . . . . .
. . . . .
Vv16g0310 Tc03_g016210 . . . Gorai.
008G212300.1 . . . . . . .
. . . . . . . .
Vv16g0311 . . . . . . .
. . . . . . . .
. . . . .
Vv16g0312 Tc03_g016220 . . . . .
. . . . . . . .
. . . . . .
Vv16g0313 . . . . . . .
. . . . . . . .
. . . . .
Vv16g0314 . . . . . . .
. . . . . . . .

. . . . .
Vv16g0315 . Gorai.001G052600.1 . . .
. . . . . . . .
. . . . . . .
Vv16g0316 . . . . . . .
. . . . . . . .
. . . . .
Vv16g0317 . . . . . . .
. . . . . . . .
. . . . .
Vv16g0318 . . . . . . .
. . . . . . . .
. . . . .
Vv16g0319 . . . . . . .
. . . . . . . .
. . . . .
Vv16g0320 Tc03_g002720 . . . Gorai.
008G212200.1 . . . . . . .
. . . . . . . .
Vv16g0321 . . Gorai.003G111300.1 . .
. . . . . . . .
. . . . . . .
Vv16g0322 . . . . . . .
. . . . . . . .
. . . . .
Vv16g0323 . . . . . . .
. . . . . . . .
. . . . .
Vv16g0324 Tc03_g016340 . . . . .
. . . . . . . .
. . . . . .
Vv16g0325 . . . . . . .
. . . . . . . .
. . . . .
Vv16g0326 . . . . . . .
. . . . . . . .
. . . . .
Vv16g0327 . . Gorai.003G111600.1 . .
. . . . . . . .
. . . . . . .
Vv16g0328 . . . . . . .
. . . . . . . .
. . . . .
Vv16g0329 . . . . . . .
. . . . . . . .
. . . . .
Vv16g0330 . . . . . . .
. . . . . . . .
. . . . .
Vv16g0331 . . . . . . .

. . . . . . . .
. . . . .
Vv16g0332 . . . . . . .
. . . . . . . .
. . . . .
Vv16g0333 . . . . . . .
. . . . . . . .
. . . . .
Vv16g0334 Tc03_g016350 Gorai.001G051800.1 . .
. . . . . . . .
. . . . . . . .
Vv16g0335 . . . . . . .
. . . . . . . .
. . . . .
Vv16g0336 . . . . . . .
. . . . . . . .
. . . . .
Vv16g0337 . . . . . . .
. . . . . . . .
. . . . .
Vv16g0338 . . . . . . .
. . . . . . . .
. . . . .
Vv16g0339 . . . . . . .
. . . . . . . .
. . . . .
Vv16g0340 . . . . . . .
. . . . . . . .
. . . . .
Vv16g0341 . . . . . . .
. . . . . . . .
. . . . .
Vv16g0342 . . . . . . .
. . . . . . . .
. . . . .
Vv16g0343 . . . . . . .
. . . . . . . .
. . . . .
Vv16g0344 . . . . . . .
. . . . . . . .
. . . . .
Vv16g0345 . . . . . . .
. . . . . . . .
. . . . .
Vv16g0346 . . . . . . .
. . . . . . . .
. . . . .
Vv16g0347 . . . . . . .
. . . . . . . .
. . . . .

Vv16g0348 . . . . . . .
. . . . . . . .
. . . . .
Vv16g0349 . . . . . . .
. . . . . . . .
. . . . .
Vv16g0350 Tc03_g002470 . . . Gorai.
008G233500.1 . . . . . . .
. . . . . . . .
Vv16g0351 . . . . . . .
. . . . . . . .
. . . . .
Vv16g0352 . . . . . . .
. . . . . . . .
. . . . .
Vv16g0353 . . . . . . .
. . . . . . . .
. . . . .
Vv16g0354 . . . . . . .
. . . . . . . .
. . . . .
Vv16g0355 . . . . . . .
. . . . . . . .
. . . . .
Vv16g0356 . . . . . . .
. . . . . . . .
. . . . .
Vv16g0357 . . . . . . .
. . . . . . . .
. . . . .
Vv16g0358 . . . . . . .
. . . . . . . .
. . . . .
Vv16g0359 Tc03_g013950 . Gorai.003G111700.1 .
. . . . . . . .
. . . . . . . .
Vv16g0360 . . . . . . .
. . . . . . . .
. . . . .
Vv16g0361 . . . . . . .
. . . . . . . .
. . . . .
Vv16g0362 Tc03_g016390 . . . Gorai.
008G213300.1 . . . . . . .
. . . . . . . .
Vv16g0363 Tc03_g016400 . . . . .
. . . . . . . .
. . . . . .
Vv16g0364 . . . . . . .
. . . . . . . .

. . . . .
Vv16g0365 . . . . . . .
. . . . . . . .
. . . . .
Vv16g0366 . . . . . . .
. . . . . . . .
. . . . .
Vv16g0367 . . . . . . .
. . . . . . . .
. . . . .
Vv16g0368 . . . . . . .
. . . . . . . .
. . . . .
Vv16g0369 Tc03_g016480 Gorai.001G051700.1 . .
. . . . . . . .
. . . . . . . .
Vv16g0370 . . . . . . .
. . . . . . . .
. . . . .
Vv16g0371 . . . . . . .
. . . . . . . .
. . . . .
Vv16g0372 . . . . . . .
. . . . . . . .
. . . . .
Vv16g0373 . . . . . . .
. . . . . . . .
. . . . .
Vv16g0374 . . . . . . .
. . . . . . . .
. . . . .
Vv16g0375 . Gorai.001G051500.1 . . .
. . . . . . . .
. . . . . . .
Vv16g0376 . . . . . . .
. . . . . . . .
. . . . .
Vv16g0377 Tc03_g016520 . Gorai.003G112000.1 .
. . . . . . . .
. . . . . . . .
Vv16g0378 . . . . . . .
. . . . . . . .
. . . . .
Vv16g0379 . . . . . . .
. . . . . . . .
. . . . .
Vv16g0380 Tc03_g016530 . . . Gorai.
008G213800.1 . . . . . . .
. . . . . . . .
Vv16g0381 . . . . . . .

. . . . . . . .
. . . . .
Vv16g0382 Tc03_g016560 Gorai.001G051400.1 . .
. . . . . . . .
. . . . . . . .
Vv16g0383 . Gorai.001G051300.1 . .
Gorai.008G213900.1 . . . . . .
. . . . . . . .
.
Vv16g0384 . . . . . . .
. . . . . . . .
. . . . .
Vv16g0385 Tc03_g016570 . Gorai.003G112200.1 .
Gorai.008G232700.1 . . . . .
. . . . . . . .
. .
Vv16g0386 . . . . . . .
. . . . . . . .
. . . . .
Vv16g0387 Tc03_g013540 . . . . .
. . . . . . . .
. . . . . .
Vv16g0388 . . . . . . .
. . . . . . . .
. . . . .
Vv16g0389 . . . . . . .
. . . . . . . .
. . . . .
Vv16g0390 . . . . . . .
. . . . . . . .
. . . . .
Vv16g0391 Tc03_g016600 Gorai.001G051200.1 . .
Gorai.008G214000.1 . . . . .
. . . . . . . .
. .
Vv16g0392 . . . . Gorai.008G232600.1
. . . . . . . .
. . . . . . .
Vv16g0393 . . . . . . .
. . . . . . . .
. . . . .
Vv16g0394 Tc03_g016630 Gorai.001G051100.1 . .
. . . . . . . .
. . . . . . . .
Vv16g0395 . . . . . . .
. . . . . . . .
. . . . .
Vv16g0396 Tc03_g013430 . . . Gorai.
008G232500.1 . . . . . . .
. . . . . . . .

Vv16g0397 . . . . . . .
. . . . . . . .
. . . . .
Vv16g0398 . . . . Gorai.008G230500.1
. . . . . . . .
. . . . . . .
Vv16g0399 . . . . . . .
. . . . . . . .
. . . . .
Vv16g0400 . . . . . . .
. . . . . . . .
. . . . .
Vv16g0401 . . . . . . .
. . . . . . . .
. . . . .
Vv16g0402 . . . . . . .
. . . . . . . .
. . . . .
Vv16g0403 . . . . . . .
. . . . . . . .
. . . . .
Vv16g0404 . . . . . . .
. . . . . . . .
. . . . .
Vv16g0405 . . . . . . .
. . . . . . . .
. . . . .
Vv16g0406 . . . . . . .
. . . . . . . .
. . . . .
Vv16g0407 Tc03_g019360 . . . . .
. . . . . . . .
. . . . . .
Vv16g0408 . . . . . . .
. . . . . . . .
. . . . .
Vv16g0409 . . . . . . .
. . . . . . . .
. . . . .
Vv16g0410 . . . . . . .
. . . . . . . .
. . . . .
Vv16g0411 . . . . . . .
. . . . . . . .
. . . . .
Vv16g0412 . . . . . . .
. . . . . . . .
. . . . .
Vv16g0413 . . . . . . .
. . . . . . . .

```
                    .        .        .        .        .
Vv16g0414           Tc03_g019370      .        .        .        .        .
                    .        .        .        .        .        .        .        .
                    .        .        .        .        .        .
Vv16g0415           .        .        .        .        .        .        .
                    .        .        .        .        .        .        .        .
                    .        .        .        .        .
Vv16g0416           .        .        .        .        .        .        .
                    .        .        .        .        .        .        .        .
                    .        .        .        .        .
Vv16g0417           .        .        .        .        .        .        .
                    .        .        .        .        .        .        .        .
                    .        .        .        .        .
Vv16g0418           .        .        .        .        .        .        .
                    .        .        .        .        .        .        .        .
                    .        .        .        .        .
Vv16g0419           .        .        .        .        .        .        .
                    .        .        .        .        .        .        .        .
                    .        .        .        .        .
Vv16g0420           .        .        .        .        .        .        .
                    .        .        .        .        .        .        .        .
                    .        .        .        .        .
Vv16g0421           .        .        .        .        .        .        .
                    .        .        .        .        .        .        .        .
                    .        .        .        .        .
Vv16g0422           .        .        .        .        .        .        .
                    .        .        .        .        .        .        .        .
                    .        .        .        .        .
Vv16g0423           .        .        .        .        .        .        .
                    .        .        .        .        .        .        .        .
                    .        .        .        .        .
Vv16g0424           Tc03_g016640      Gorai.001G051000.1         .        .
                    .        .        .        .        .        .        .        .
                    .        .        .        .        .        .        .        .
Vv16g0425           .        .        .        .        .        .        .
                    .        .        .        .        .        .        .        .
                    .        .        .        .        .
Vv16g0426           .        .        .        .        .        .        .
                    .        .        .        .        .        .        .        .
                    .        .        .        .        .
Vv16g0427           .        .        .        .        .        .        .
                    .        .        .        .        .        .        .        .
                    .        .        .        .        .
Vv16g0428           .        .        .        .        Gorai.008G215500.1
                    .        .        .        .        .        .        .        .
                    .        .        .        .        .        .        .
Vv16g0429           .        .        .        .        .        .        .
                    .        .        .        .        .        .        .        .
                    .        .        .        .        .
Vv16g0430           .        .        Gorai.003G113000.1         .
```

Gorai.008G215600.1 . . . . . .
. . . . . . . .
.
Vv16g0431 . . . . . . .
. . . . . . . .
. . . . .
Vv16g0432 . . Gorai.003G113100.1 .
Gorai.008G215700.1 . . . . . .
. . . . . . . .
.
Vv16g0433 . . . . . . .
. . . . . . . .
. . . . .
Vv16g0434 . . Gorai.003G113200.1 . .
. . . . . . . .
. . . . . . .
Vv16g0435 . . . . . . .
. . . . . . . .
. . . . .
Vv16g0436 . . . . . . .
. . . . . . . .
. . . . .
Vv16g0437 . . . . . . .
. . . . . . . .
. . . . .
Vv16g0438 Tc03_g019480 . . . . .
. . . . . . . .
. . . . . .
Vv16g0439 . . . . . . .
. . . . . . . .
. . . . .
Vv16g0440 Tc03_g016990 Gorai.001G049000.1 . .
. . . . . . . .
. . . . . . . .
Vv16g0441 . . . . . . .
. . . . . . . .
. . . . .
Vv16g0442 . . . . . . .
. . . . . . . .
. . . . .
Vv16g0443 . . . . . . .
. . . . . . . .
. . . . .
Vv16g0444 . . . . . . .
. . . . . . . .
. . . . .
Vv16g0445 . . . . . . .
. . . . . . . .
. . . . .
Vv16g0446 . . . . . . .

. . . . . . . .
. . . . .
Vv16g0447 . . . . . . .
. . . . . . . .
. . . . .
Vv16g0448 . . . . . . .
. . . . . . . .
. . . . .
Vv16g0449 . . . . . . .
. . . . . . . .
. . . . .
Vv16g0450 . . . . . . .
. . . . . . . .
. . . . .
Vv16g0451 . . . . . . .
. . . . . . . .
. . . . .
Vv16g0452 . . . . . . .
. . . . . . . .
. . . . .
Vv16g0453 . . . . . . .
. . . . . . . .
. . . . .
Vv16g0454 . . . . . . .
. . . . . . . .
. . . . .
Vv16g0455 . . . . . . .
. . . . . . . .
. . . . .
Vv16g0456 . . . . . . .
. . . . . . . .
. . . . .
Vv16g0457 . . . . . . .
. . . . . . . .
. . . . .
Vv16g0458 . . . . . . .
. . . . . . . .
. . . . .
Vv16g0459 . . . . . . .
. . . . . . . .
. . . . .
Vv16g0460 . . . . . . .
. . . . . . . .
. . . . .
Vv16g0461 . . . . . . .
. . . . . . . .
. . . . .
Vv16g0462 . . . . . . .
. . . . . . . .
. . . . .

Vv16g0463 . . . . . . .
. . . . . . . .
. . . . .
Vv16g0464 . . . . . . .
. . . . . . . .
. . . . .
Vv16g0465 . . . . . . .
. . . . . . . .
. . . . .
Vv16g0466 Tc03_g004410 . . . . .
. . . . . . . .
. . . . . .
Vv16g0467 . . . . . . .
. . . . . . . .
. . . . .
Vv16g0468 . . . . . . .
. . . . . . . .
. . . . .
Vv16g0469 . . . . . . .
. . . . . . . .
. . . . .
Vv16g0470 . . . . . . .
. . . . . . . .
. . . . .
Vv16g0471 Tc03_g004110 . . . . .
. . . . . . . .
. . . . . .
Vv16g0472 Tc03_g004080 . . . . .
. . . . . . . .
. . . . . .
Vv16g0473 . . . . . . .
. . . . . . . .
. . . . .
Vv16g0474 Tc03_g017330 . . . . .
. . . . . . . .
. . . . . .
Vv16g0475 . . . . . . .
. . . . . . . .
. . . . .
Vv16g0476 . . . . . . .
. . . . . . . .
. . . . .
Vv16g0477 Tc03_g017340 . . . . .
. . . . . . . .
. . . . . .
Vv16g0478 . . . . . . .
. . . . . . . .
. . . . .
Vv16g0479 Tc03_g017360 . . . . .
. . . . . . . .

| | | | | | | | |
|---|---|---|---|---|---|---|---|
| . | . | . | . | . | . | | |
| Vv16g0480 | . | . | . | . | . | . | . |
| . | . | . | . | . | . | . | . |
| . | . | . | . | . | | | |
| Vv16g0481 | Tc03_g017370 | | . | . | . | . | . |
| . | . | . | . | . | . | . | . |
| . | . | . | . | . | . | | |
| Vv16g0482 | Tc03_g022290 | | . | . | Gorai.007G018200.1 | | |
| . | . | . | . | . | . | . | . |
| . | . | . | . | . | . | . | . |
| Vv16g0483 | . | . | . | . | . | . | . |
| . | . | . | . | . | . | . | . |
| . | . | . | . | . | | | |
| Vv16g0484 | . | . | . | . | . | . | . |
| . | . | . | . | . | . | . | . |
| . | . | . | . | . | | | |
| Vv16g0485 | . | . | . | . | . | . | . |
| . | . | . | . | . | . | . | . |
| . | . | . | . | . | | | |
| Vv16g0486 | . | . | . | . | . | . | . |
| . | . | . | . | . | . | . | . |
| . | . | . | . | . | | | |
| Vv16g0487 | . | . | . | . | . | . | . |
| . | . | . | . | . | . | . | . |
| . | . | . | . | . | | | |
| Vv16g0488 | . | . | . | . | . | . | . |
| . | . | . | . | . | . | . | . |
| . | . | . | . | . | | | |
| Vv16g0489 | . | . | . | . | . | . | . |
| . | . | . | . | . | . | . | . |
| . | . | . | . | . | | | |
| Vv16g0490 | . | . | . | . | . | . | . |
| . | . | . | . | . | . | . | . |
| . | . | . | . | . | | | |
| Vv16g0491 | . | . | . | . | . | . | . |
| . | . | . | . | . | . | . | . |
| . | . | . | . | . | | | |
| Vv16g0492 | . | . | . | . | . | . | . |
| . | . | . | . | . | . | . | . |
| . | . | . | . | . | | | |
| Vv16g0493 | . | . | . | . | . | . | . |
| . | . | . | . | . | . | . | . |
| . | . | . | . | . | | | |
| Vv16g0494 | . | . | . | . | . | . | . |
| . | . | . | . | . | . | . | . |
| . | . | . | . | . | | | |
| Vv16g0495 | . | . | . | . | . | . | . |
| . | . | . | . | . | . | . | . |
| . | . | . | . | . | | | |
| Vv16g0496 | . | . | . | . | . | . | . |

. . . . . . . .
. . . . .
Vv16g0497 . . . . . . .
. . . . . . . .
. . . . .
Vv16g0498 Tc03_g017380 . . . . .
. . . . . . . .
. . . . . .
Vv16g0499 . . . . . . .
. . . . . . . .
. . . . .
Vv16g0500 . . . . . . .
. . . . . . . .
. . . . .
Vv16g0501 . . . . . . .
. . . . . . . .
. . . . .
Vv16g0502 . . . . . . .
. . . . . . . .
. . . . .
Vv16g0503 . . . . . . .
. . . . . . . .
. . . . .
Vv16g0504 . . . . . . .
. . . . . . . .
. . . . .
Vv16g0505 . . . . . . .
. . . . . . . .
. . . . .
Vv16g0506 . . . . . . .
. . . . . . . .
. . . . .
Vv16g0507 . . . . . . .
. . . . . . . .
. . . . .
Vv16g0508 . . . . . . .
. . . . . . . .
. . . . .
Vv16g0509 . . . . . . .
. . . . . . . .
. . . . .
Vv16g0510 Tc03_g017710 . . . . .
. . . . . . . .
. . . . . .
Vv16g0511 Tc03_g022180 . . . . .
. . . . . . . .
. . . . . .
Vv16g0512 Tc03_g022170 . . . Gorai.
008G262800.1 . . . . . . .
. . . . . . . .

Vv16g0513 Tc03_g022160 . . . . .
. . . . . . . .
. . . . . .
Vv16g0514 . . . . . . .
. . . . . . . .
. . . . .
Vv16g0515 Tc03_g022140 . . Gorai.007G017700.1
Gorai.008G262900.1 . . . . .
. . . . . . . .
. .
Vv16g0516 . . . . . . .
. . . . . . . .
. . . . .
Vv16g0517 . . . . . . .
. . . . . . . .
. . . . .
Vv16g0518 . . . . . . .
. . . . . . . .
. . . . .
Vv16g0519 Tc03_g022120 . . . . .
. . . . . . . .
. . . . . .
Vv16g0520 . . . . . . .
. . . . . . . .
. . . . .
Vv16g0521 . . . . . . .
. . . . . . . .
. . . . .
Vv16g0522 Tc03_g022100 . . . Gorai.
008G263000.1 . . . . . . .
. . . . . . . .
Vv16g0523 . . . . . . .
. . . . . . . .
. . . . .
Vv16g0524 Tc03_g022070 . . . Gorai.
008G263400.1 . . . . . . .
. . . . . . . .
Vv16g0525 . . . . Gorai.008G263400.1
. . . . . . . .
. . . . . . .
Vv16g0526 . . . Gorai.007G115000.1 .
. . . . . . . .
. . . . . . .
Vv16g0527 . . . . . . .
. . . . . . . .
. . . . .
Vv16g0528 Tc03_g022050 . . Gorai.007G114700.1
Gorai.008G263600.1 . . . . .
. . . . . . . .
. .

```
Vv16g0529	Tc03_g022030	.	.	.	.	.
	.	.	.	.	.	.	.	.
	.	.	.	.	.	.
Vv16g0530	Tc03_g022020	.	.	.	Gorai.
008G262000.1	.	.	.	.	.	.	.
	.	.	.	.	.	.	.	.
Vv16g0531	Tc03_g021990	.	.	Gorai.007G114600.1
	.	.	Vv15g0482	Tc01_g033450	.	.
	.	.	.	.	.	.	.	.
	.	.
Vv16g0532	Tc03_g021980	.	.	Gorai.007G017500.1
	.	.	.	.	.	.	.	.
	.	.	.	.	.	.	.	.
Vv16g0533	.	.	.	.	.	.	.
	.	.	.	.	.	.	.	.
	.	.	.	.	.
Vv16g0534	Tc03_g021930	.	.	.	.	.
	.	.	.	.	.	.	.	.
	.	.	.	.	.	.
Vv16g0535	.	.	.	.	.	.	.
	.	.	.	.	.	.	.	.
	.	.	.	.	.
Vv16g0536	Tc03_g021920	.	.	.	Gorai.
008G263900.1	.	.	.	.	.	.	.
	.	.	.	.	.	.	.	.
Vv16g0537	.	.	.	.	.	.	.
	.	.	.	.	.	.	.	.
	.	.	.	.	.
Vv16g0538	Tc03_g021910	.	.	.	Gorai.
008G264000.1	.	Vv02g0309	Tc03_g015160	Gorai.
004G128100.1	.	.	Gorai.008G261300.1	Gorai.
006G204300.1	.	.	.	.	.	.	.
Vv16g0539	.	.	.	.	.	.	.
	.	.	.	.	.	.	.	.
	.	.	.	.	.
Vv16g0540	Tc03_g021880	.	.	.	Gorai.
008G264300.1	.	.	.	.	.	.	.
	.	.	.	.	.	.	.	.
Vv16g0541	Tc03_g021870	.	.	.	Gorai.
008G264600.1	.	.	.	.	.	.	.
	.	.	.	.	.	.	.	.
Vv16g0542	.	.	.	.	.	.	.
	.	.	.	.	.	.	.	.
	.	.	.	.	.
Vv16g0543	Tc03_g021860	.	.	Gorai.007G017400.1
	.	.	.	.	.	.	.	.
	.	.	.	.	.	.	.	.
Vv16g0544	Tc03_g021850	.	.	.	Gorai.
008G260800.1	.	Vv02g0303	.	.	.	.
	Gorai.008G260800.1	.	Vv15g0505	.	.
```

.	.	.	.
Vv16g0545	.	.	.	.	Gorai.008G260600.1
.	Vv02g0302	.	.	.	.
Gorai.008G264900.1	.	Vv15g0508	.	.	.
.	.	.
Vv16g0546	Tc03_g021840	.	.	.	Gorai.
008G265000.1	.	.	.	.	.	.	.
.	.	.	.	.	.	.	.
Vv16g0547	Tc03_g021830	.	.	.	.	.
.	.	.	.	.	.	.	.
.	.	.	.	.	.
Vv16g0548	Tc03_g021800	.	.	Gorai.007G017300.1
.	.	.	.	.	.	.	.
.	.	.	.	.	.	.	.
Vv16g0549	Tc03_g021790	.	.	Gorai.007G017200.1
.	.	.	.	.	.	.	.
.	Vv15g0515	Tc01_g033640	.	.	.
.	.
Vv16g0550	Tc03_g021780	.	.	Gorai.007G113000.1
Gorai.008G265100.1	.	.	.	.	.
.	.	.	Vv15g0516	Tc01_g033650	.
.	.	.	.
Vv16g0551	.	.	.	.	.	.	.
.	.	.	.	.	.	.	.
.	.	.	.	.
Vv16g0552	Tc03_g021770	.	.	.	.	.
.	.	.	.	.	.	.	.
.	.	.	.	.	.
Vv16g0553	Tc03_g021760	.	.	.	.	.
.	.	.	.	.	.	.	.
.	.	.	.	.	.
Vv16g0554	.	.	.	.	.	.	.
.	.	.	.	.	.	.	.
.	.	.	.	.
Vv16g0555	.	.	.	.	.	.	.
.	.	.	.	.	.	.	.
.	.	.	.	.
Vv16g0556	.	.	.	.	.	.	.
.	.	.	.	.	.	.	.
.	.	.	.	.
Vv16g0557	Tc03_g021740	.	.	.	.	.
.	.	.	.	.	.	.	.
.	.	.	.	.	.
Vv16g0558	.	.	.	.	.	.	.
.	.	.	.	.	.	.	.
.	.	.	.	.
Vv16g0559	Tc03_g021690	.	.	.	.	.
Vv02g0287	.	.	.	.	.	.
.	.	.	.	.	.	.
Vv16g0560	.	.	.	.	.	.	.

. . . . . . . .
. . . . .
Vv16g0561 Tc03_g021670 . . Gorai.007G017000.1
. . . . . . . .
. . . . . . . .
Vv16g0562 . . . . . . .
. . . . . . . .
. . . . .
Vv16g0563 Tc03_g021660 . . . . .
. . . . . . . .
. . . . . .
Vv16g0564 Tc03_g021650 . . Gorai.007G016900.1
. . . . . . . .
. . . . . . . .
Vv16g0565 Tc03_g021640 . . . . .
. . . . . . . .
. . . . . .
Vv16g0566 Tc03_g021620 . . . Gorai.
008G265500.1 . . . . . . .
. . . . . . . .
Vv16g0567 Tc03_g021610 . . . . .
Vv02g0275 . . . Gorai.007G019100.1
Gorai.008G259600.1 . Vv15g0531
Tc01_g033790 . . . . .
Vv16g0568 Tc03_g021600 . . . Gorai.
008G265600.1 . . . . . . .
. . . . . . . .
Vv16g0569 . . . . . . .
. . . . . . . .
. . . . .
Vv16g0570 Tc03_g021590 . . . . .
. . . . . . . .
. . . . . .
Vv16g0571 Tc03_g021580 . . . Gorai.
008G265700.1 . . . . . . .
. . . . . . . .
Vv16g0572 Tc03_g021570 . Gorai.003G122900.1 .
. . . . . . . .
. Vv15g0541 Tc01_g033890 . . .
. .
Vv16g0573 Tc03_g021560 . . . Gorai.
008G265800.1 . . . . . . .
. . . . . . . .
Vv16g0574 Tc03_g021550 . . . . .
. . . . . . . .
. . . . . .
Vv16g0575 Tc03_g021540 . . . Gorai.
008G265900.1 . . . . . . .
. . . . . . . .
Vv16g0576 Tc03_g021530 . . . . .

. . . . . . . .
. . . . . .
Vv16g0577 . . . . . . .
. . . . . . . .
. . . . .
Vv16g0578 Tc03_g021520 . . Gorai.007G111400.1
Gorai.008G258900.1 . Vv02g0264
Tc03_g029890 . . Gorai.007G111400.1 Gorai.
008G258900.1 . Vv15g0552 Tc01_g033980 . .
. . .
Vv16g0579 Tc03_g021510 . . . . .
. . . . . . . .
. . . . . .
Vv16g0580 Tc03_g021500 . . Gorai.007G016700.1
Gorai.008G266000.1 . . . . .
. . . Vv15g0554 . . .
. . .
Vv16g0581 Tc03_g021480 . . . Gorai.
008G266100.1 . . . . . . .
. . . . . . . .
Vv16g0582 Tc03_g021470 . . Gorai.007G016600.1
Gorai.008G258300.1 . Vv02g0258 . .
. Gorai.007G020000.1 Gorai.008G258300.1 .
. . . . . . .
Vv16g0583 Tc03_g021460 . Gorai.003G122800.1 .
Gorai.008G266200.1 . . . . .
. . . . . . . .
. .
Vv16g0584 . . . . . . .
. . . . . . . .
. . . . .
Vv16g0585 Tc03_g021450 . Gorai.003G122700.1 .
. . Vv02g0254 . . Gorai.
003G122700.1 . . . . . . .
. . .
Vv16g0586 . . . . . . .
. . . . . . . .
. . . . .
Vv16g0587 Tc03_g021440 . . . . .
. . . . . . . .
. . . . . .
Vv16g0588 Tc03_g021430 . Gorai.003G122600.1
Gorai.007G110900.1 . . . . . .
. . . . . . . .
. .
Vv16g0589 Tc03_g021420 . . . Gorai.
008G266300.1 . . . . . . .
. . . . . . . .
Vv16g0590 Tc03_g021400 . . . . .
. . . . . . . .

. . . . . .
Vv16g0591 Tc03_g021390 . . Gorai.007G016500.1
Gorai.008G266400.1 . Vv02g0245 .
Gorai.004G130100.1 . . Gorai.008G266400.1 .
. . . . . . .
Vv16g0592 Tc03_g021380 . Gorai.003G122500.1 .
. . . . . . . .
. . . . . . . .
Vv16g0593 Tc03_g021370 . . . Gorai.
008G266500.1 . . . . . . .
. . . . . . . .
Vv16g0594 Tc03_g021340 . . . . .
. . . . . . . .
. . . . . .
Vv16g0595 . . . . . . .
. . . . . . . .
. . . . .
Vv16g0596 Tc03_g021330 . . . . .
. . . . . . . .
. . . . . .
Vv16g0597 . . . Gorai.007G016300.1 .
. Vv02g0242 . Gorai.004G130200.1 .
. . . . . . . .
. .
Vv16g0598 Tc03_g021320 . . Gorai.007G016200.1
. . . . . . . .
. . . . . . . .
Vv16g0599 Tc03_g021300 . Gorai.003G122200.1
Gorai.007G016100.1 Gorai.008G257400.1 . Vv02g0241
. Gorai.004G130300.1 Gorai.003G122200.1 .
Gorai.008G257400.1 . Vv0Unrg3035 . .
. . . .
Vv16g0600 . . . . . . .
. . . . . . . .
. . . . .
Vv16g0601 Tc03_g021280 . . . . .
. . . . . . . .
. . . . . .
Vv16g0602 Tc03_g021270 . . . Gorai.
008G266900.1 . . . . . . .
. . . . . . . .
Vv16g0603 Tc03_g021260 . . . Gorai.
008G267000.1 . Vv02g0229 Tc03_g012980 .
Gorai.003G076400.1 . Gorai.008G256800.1 Gorai.
006G206100.1 Vv0Unrg3026 . . . . .
.
Vv16g0604 . . . . . . .
. . . . . . . .
. . . . .
Vv16g0605 Tc03_g021250 . . . . .

. . . . . . . .
. . . . . .
Vv16g0606 Tc03_g021240 . Gorai.003G122100.1 .
. . . . . . . .
. . . . . . . .
Vv16g0607 Tc03_g021230 . Gorai.003G122000.1
Gorai.007G110600.1 Gorai.008G267100.1 . . .
. . . . . . . .
. . . .
Vv16g0608 . . . . . . .
. . . . . . . .
. . . . .
Vv16g0609 Tc03_g021210 . . . . .
. . . . . . . .
. . . . . .
Vv16g0610 . . . . . . .
. . . . . . . .
. . . . .
Vv16g0611 Tc03_g021200 . Gorai.003G121900.1 .
. . . . . . . .
. . . . . . . .
Vv16g0612 Tc03_g021190 . . . . .
. . . . . . . .
. . . . . .
Vv16g0613 Tc03_g021180 . . . . .
Vv02g0217 Tc03_g029430 Gorai.004G131400.1 .
Gorai.007G020600.1 . . . . .
. . . .
Vv16g0614 . . . . . . .
. . . . . . . .
. . . . .
Vv16g0615 . . . . . . .
. . . . . . . .
. . . . .
Vv16g0616 . . . . . . .
. . . . . . . .
. . . . .
Vv16g0617 Tc03_g021160 . . . Gorai.
008G267400.1 . . . . . . .
. . . . . . . .
Vv16g0618 Tc03_g021140 . . . Gorai.
008G267600.1 . . . . . . .
. . . . . . . .
Vv16g0619 . . . . Gorai.008G267800.1
. . . . . . . .
. . . . . . .
Vv16g0620 . . . . . . .
. . . . . . . .
. . . . .
Vv16g0621 Tc03_g021120 . . . . .

Vv02g0215 Tc03_g029400 . . Gorai.
007G110100.1 . Gorai.006G206300.1 Vv0Unrg3017 .
. . . . .
Vv16g0622 Tc03_g021100 . . Gorai.007G109800.1
. . . . . . . .
. Vv0Unrg3013 . . . . .
.
Vv16g0623 Tc03_g021090 . . . Gorai.
008G267900.1 . . . . . . .
. . . . . . . .
Vv16g0624 Tc03_g021080 . . . . .
. . . . . . . .
. . . . . .
Vv16g0625 Tc03_g021070 . Gorai.003G121700.1 .
. . . . . . . .
. . . . . . . .
Vv16g0626 . . . . Gorai.008G256500.1
. . . . . . . .
. . . . . . .
Vv16g0627 Tc03_g021060 . Gorai.003G121600.1 .
. . Vv02g0213 . . . .
. . . . . . . .
.
Vv16g0628 Tc03_g021050 . Gorai.003G121500.1 .
Gorai.008G268100.1 . . . . .
. . . Vv0Unrg3010 . . .
. . .
Vv16g0629 Tc03_g021040 . Gorai.003G119000.1 .
Gorai.008G268200.1 . . . . .
. . . . . . . .
. .
Vv16g0630 Tc03_g021030 . . . Gorai.
008G268300.1 . . . . . . .
. . . . . . . .
Vv16g0631 Tc03_g021020 . . . Gorai.
008G268400.1 . . . . . . .
. . . . . . . .
Vv16g0632 Tc03_g021010 . . . . .
. . . . . . . .
. . . . . .
Vv16g0633 Tc03_g021000 . . . Gorai.
008G268500.1 . . . . . . .
. . . . . . . .
Vv16g0634 . . . . . . .
. . . . . . . .
. . . . .
Vv16g0635 . . . Gorai.007G015700.1
Gorai.008G268600.1 . . . . . .
. . . . . . . .
.

Vv16g0636 . . . . . . .
. . . . . . .
. . . . .
Vv16g0637 . . . Gorai.007G109100.1
Gorai.008G268700.1 . . . . . .
. . Vv0Unrg3004 . . . .
. .
Vv16g0638 Tc03_g020920 . . . . .
. . . . . . .
. . . . . .
Vv16g0639 Tc03_g020910 . . Gorai.007G108500.1
. . Vv02g0173 . . .
Gorai.007G108500.1 . . Vv0Unrg2999 . .
. . . .
Vv16g0640 . . . . Gorai.008G268800.1
. . . . . . .
. . . . . . .
Vv16g0641 . . . . . . .
. . . . . . .
. . . . .
Vv16g0642 . . . . . . .
. . . . . . .
. . . . .
Vv16g0643 . . . . . . .
. . . . . . .
. . . . .
Vv16g0644 . . . Gorai.007G108300.1
Gorai.008G255200.1 . Vv02g0167 Tc03_g029130
Gorai.004G132400.1 Gorai.003G078600.1 Gorai.007G108300.1
Gorai.008G255600.1 . Vv0Unrg2994 . .
. . . .
Vv16g0645 . . . . . . .
. . . . . . .
. . . . .
Vv16g0646 . . . . . . .
. . . . . . .
. . . . .
Vv16g0647 . . . . . . .
. . . . . . .
. . . . .
Vv17g0001 . . . . . . .
. . . . . . .
. . . . .
Vv17g0002 Tc03_g016040 . . . Gorai.
008G211400.1 . . . . . . .
. . . . . . .
Vv17g0003 . . . . Gorai.008G211300.1
. . . . . . .
. . . . . . .
Vv17g0004 . . . . . . .

. . . . . . . .
. . . . .
Vv17g0005 . Gorai.001G053500.1 . . .
. . . . . . . .
. . . . . . .
Vv17g0006 Tc03_g019260 . . . . .
. . . . . . . .
. . . . . .
Vv17g0007 . . . . . . .
. . . . . . . .
. . . . .
Vv17g0008 Tc03_g018890 . . . .
Gorai.006G196400.1 . . . . . .
. . . . . . . .
Vv17g0009 Tc03_g016000 . . Gorai.007G045200.1
. . . . . . . .
. . . . . . . .
Vv17g0010 Tc03_g015990 . . . Gorai.
008G211100.1 . . . . . . .
. . . . . . . .
Vv17g0011 Tc03_g015980 . . . Gorai.
008G156000.1 . Vv01g1280 . . . .
Gorai.008G156000.1 . . . . .
. . .
Vv17g0012 Tc03_g015970 Gorai.001G053800.1 . .
. . Vv01g1278 . . . .
. . . . . . . .
.
Vv17g0013 . . . . . . .
. . . . . . . .
. . . . .
Vv17g0014 . . . . . . .
. . . . . . . .
. . . . .
Vv17g0015 Tc03_g015960 . . . . .
. . . . . . . .
. . . . . .
Vv17g0016 Tc03_g015950 Gorai.001G053900.1 . .
. . . . . . . .
. . . . . . . .
Vv17g0017 Tc03_g015940 . . . . .
Vv01g1276 . . . . .
Gorai.012G125800.1 . . . . . .
.
Vv17g0018 Tc03_g015920 . . . Gorai.
008G210800.1 . . . . . . .
. . . . . . . .
Vv17g0019 . . . . . . .
. . . . . . . .
. . . . .

Vv17g0020 Tc03_g015910 . . . Gorai.
008G210700.1 . . . . . . .
. . . . . . . .
Vv17g0021 . . . . . . .
. . . . . . . .
. . . . .
Vv17g0022 Tc03_g015870 . . Gorai.007G045000.1
. Gorai.006G273100.1 . . . .
. . . . . . . .
. .
Vv17g0023 Tc03_g015850 Gorai.001G054100.1 . .
. . . . . . . .
. . . . . . . .
Vv17g0024 Tc03_g015830 Gorai.001G054200.1 . .
Gorai.008G210400.1 . . . . .
. . . . . . . .
. .
Vv17g0025 Tc03_g015820 Gorai.001G054300.1 . .
. . . . . . . .
. . . . . . . .
Vv17g0026 Tc03_g015810 . . . . .
Vv01g1264 . . . . . .
. . . . . . .
Vv17g0027 Tc03_g015800 Gorai.001G054400.1 . .
. . . . . . . .
. . . . . . . .
Vv17g0028 Tc03_g015790 . . . . .
Vv14g1061 Tc04_g005180 . . . .
. . . . . . . .
Vv17g0029 Tc03_g015780 . . . . .
Vv14g1062 . . . . . .
. . . . . . .
Vv17g0030 Tc03_g015770 Gorai.001G054500.1 . .
. . . . . . . .
. . . . . . . .
Vv17g0031 . . . . . . .
. . . . . . . .
. . . . .
Vv17g0032 Tc03_g015740 . . . . .
. . . . . . . .
. . . . . .
Vv17g0033 Tc03_g015720 Gorai.001G054600.1 .
Gorai.007G044900.1 . . . . . .
. . . Vv01g1257 . . .
. . .
Vv17g0034 Tc03_g015710 . . . . .
. . . . . . . .
. . . . . .
Vv17g0035 Tc03_g015700 Gorai.001G054700.1 . .
. . . . . . . .

.	.	.	.	.	.	.	.
Vv17g0036	.	.	.	.	.	Gorai.
006G273600.1	Vv14g1073	Tc04_g005070	.	.	.
.	.	.	.	.	.	.	.
.
Vv17g0037	Tc03_g015690	.	.	.	.
Gorai.006G273300.1	.	.	.	.	.	.
.	.	.	.	.	.	.	.
Vv17g0038	Tc03_g015680	.	.	Gorai.007G044700.1
.	.	.	.	.	.	.	.
.	Vv01g1251	Tc02_g019750	.	Gorai.
005G196500.1	.	.	.
Vv17g0039	Tc03_g015670	.	.	Gorai.007G044600.1
Gorai.008G210100.1	.	.	.	.	.
.	.	.	Vv01g1249	Tc02_g019760	.
.	.	Gorai.008G210100.1	.
Vv17g0040	.	.	.	.	.	.	.
.	.	.	.	.	.	.	.
.	.	.	.	.
Vv17g0041	Tc03_g015650	Gorai.001G054800.1	.	.
.	.	.	.	.	.	.	.
.	.	.	.	.	.	.	.
Vv17g0042	.	.	.	.	.	.	.
.	.	.	.	.	.	.	.
.	.	.	.	.
Vv17g0043	Tc03_g015620	Gorai.001G054900.1	.
Gorai.007G044400.1	.	.	.	.	.	.
.	.	.	.	.	.	.	.
.	.
Vv17g0044	.	.	.	.	.	.	.
.	.	.	.	.	.	.	.
.	.	.	.	.
Vv17g0045	.	.	.	.	.	.
Vv14g1080	Tc04_g004980	.	.	Gorai.007G072500.1
.	Gorai.012G082900.1	.	.	.	.
.	.	.
Vv17g0046	Tc03_g015610	.	.	.	Gorai.
008G209800.1	.	.	.	.	.	.	.
.	.	.	.	.	.	.	.
Vv17g0047	Tc03_g015600	Gorai.001G055000.1	.
Gorai.007G044300.1	Gorai.008G209700.1	Gorai.006G272900.1
.	.	.	.	.	.	.
Vv01g1242	.	.	.	.	Gorai.008G209700.1
.
Vv17g0048	Tc03_g015590	.	.	.	Gorai.
008G209600.1	.	Vv14g1082	Tc04_g004880	.	.
.	.	.	.	.	.	.	.
.	.
Vv17g0049	.	.	.	.	.	.	.
.	.	.	.	.	.	.	.

. . . . .
Vv17g0050 Tc03_g015570 Gorai.001G055200.1 . .
. Gorai.006G272800.1 . . . .
. . . Vv01g1226 . . .
. . .
Vv17g0051 Tc03_g015560 . . . .
Gorai.006G272700.1 Vv14g1091 Tc04_g004770 . .
Gorai.007G044200.1 Gorai.006G000900.1 . .
. . . . . .
Vv17g0052 Tc03_g015550 . . . . .
Vv14g1099 Tc04_g004720 . . . .
Gorai.012G083300.1 . . . . .
. .
Vv17g0053 . . . . . . .
. . . . . . . .
. . . . .
Vv17g0054 Tc03_g015540 . . Gorai.007G044100.1
Gorai.008G209500.1 . . . . .
. . . . . . . .
. .
Vv17g0055 Tc03_g015530 Gorai.001G055300.1 . .
. Gorai.006G272600.1 . . . .
. . . . . . . .
. .
Vv17g0056 . . . . . . .
. . . . . . . .
. . . . .
Vv17g0057 . . . . . . .
. . . . . . . .
. . . . .
Vv17g0058 . . . . . . .
. . . . . . . .
. . . . .
Vv17g0059 . . . . . . .
. . . . . . . .
. . . . .
Vv17g0060 Tc03_g015520 . . . Gorai.
008G156700.1 . . . . . . .
. Vv01g1220 . . Gorai.005G195600.1
. Gorai.008G156700.1 .
Vv17g0061 . . . . . . .
. . . . . . . .
. . . . .
Vv17g0062 . . . . . . .
. . . . . . . .
. . . . .
Vv17g0063 . . . . . . .
. . . . . . . .
. . . . .
Vv17g0064 . . . . . . .

```
        .       .       .       .       .       .       .       .
        .       .       .       .       .
Vv17g0065       .       .       .       .       .       .       .
        .       .       .       .       .       .       .       .
        .       .       .       .       .
Vv17g0066       Tc03_g015510    .       .       Gorai.007G044000.1
        .       .       .       .       .       .       .       .
        .       .       .       .       .       .       .       .
Vv17g0067       .       .       .       .       .       .       .
        .       .       .       .       .       .       .       .
        .       .       .       .       .
Vv17g0068       .       .       .       .       .       .       .
        .       .       .       .       .       .       .       .
        .       .       .       .       .
Vv17g0069       Tc03_g015490    .       .       Gorai.007G043900.1
        .       .       .       .       .       .       .       .
        .       .       .       .       .       .       .       .
Vv17g0070       Tc03_g015480    .       .       .       Gorai.
008G209300.1    .       Vv14g1104       .       .       .       .
        .       .       .       .       .       .       .       .
        .
Vv17g0071       Tc03_g015470    .       .       .       Gorai.
008G209200.1    .       Vv14g1106       Tc04_g004610    .       .
        .       Gorai.006G044900.1      .       .       .       .
        .       .       .       .
Vv17g0072       Tc03_g015460    Gorai.001G055500.1      .       .
        Gorai.008G209100.1      .       .       .       .       .
        .       .       .       Vv01g1214       .       .
Gorai.005G195100.1      .       Gorai.008G209000.1      Gorai.
012G124700.1
Vv17g0073       Tc03_g015450    Gorai.001G055600.1      .       .
        Gorai.008G209000.1      .       .       .       .       .
        .       .       .       .       .       .       .       .
        .       .
Vv17g0074       .       .       .       .       .       .
Vv14g1108       Tc04_g004580    .       .       .       Gorai.
006G044700.1    .       .       .       .       .       .       .
        .
Vv17g0075       .       .       .       .       .       .
Vv14g1109       Tc04_g004560    .       .       .       Gorai.
006G044500.1    Gorai.012G083900.1      .       .       .       .
        .       .       .
Vv17g0076       Tc03_g015430    .       .       .       .       .
        .       .       .       .       .       .       .       .
        .       .       .       .       .       .
Vv17g0077       Tc03_g015420    .       .       .       Gorai.
008G208900.1    .       .       .       .       .       .       .
        .       .       .       .       .       .       .       .
Vv17g0078       Tc03_g015400    Gorai.001G055800.1      .       .
        .       .       .       .       .       .       .       .
```

. . . . . . . .
Vv17g0079 . . . . . . .
. . . . . . . .
. . . . .
Vv17g0080 Tc03_g015390 . . Gorai.007G043700.1
Gorai.008G208800.1 . . . . .
. . . . . . . .
. .
Vv17g0081 Tc03_g015380 . . . Gorai.
008G208700.1 . . . . . . .
. . . . . . . .
Vv17g0082 . Gorai.001G055900.1 . .
Gorai.008G157100.1 . . . . . .
. . . . . . . .
.
Vv17g0083 . . . . . . .
. . . . . . . .
. . . . .
Vv17g0084 Tc03_g015350 . . . . .
Vv14g1116 . . . . . .
. . . . . . .
Vv17g0085 Tc03_g015340 Gorai.001G056200.1 . .
Gorai.008G208200.1 . . . . .
. . . . . . . .
. .
Vv17g0086 Tc03_g015330 Gorai.001G056300.1 . .
Gorai.008G208100.1 Gorai.006G272400.1 Vv14g1118
. . . Gorai.007G043600.1 . .
. . . . . . .
Vv17g0087 Tc03_g015300 . . . Gorai.
008G207800.1 . . . . . . .
. . . . . . . .
Vv17g0088 Tc03_g015290 Gorai.001G056500.1 . .
. . Vv14g1122 . . .
Gorai.007G068800.1 Gorai.006G043900.1 Gorai.012G084400.1
Vv01g1200 . . . . . .
Vv17g0089 Tc03_g015270 . . . . .
. . . . . . . .
. . . . . .
Vv17g0090 . Gorai.001G056700.1 . . .
. . . . . . . .
. . . . . . .
Vv17g0091 Tc03_g015250 Gorai.001G056800.1 . .
. . . . . . . .
. . . . . . . .
Vv17g0092 Tc03_g015240 Gorai.001G056900.1 . .
. . . . . . . .
. . . . . . . .
Vv17g0093 . . . . . . .
. . . . . . . .

. . . . .
Vv17g0094 . . . . . . .
. . . . . . . .
. . . . .
Vv17g0095 . . . . Gorai.008G157700.1
. . . . . . . .
. . . . . . .
Vv17g0096 Tc03_g015170 . . . . .
. . . . . . . .
. . . . . .
Vv17g0097 Tc03_g015160 Gorai.001G057100.1 . .
Gorai.008G207300.1 Gorai.006G272200.1 . .
. . . . . Vv01g1193 .
. . . . .
Vv17g0098 Tc03_g015150 Gorai.001G057500.1 . .
. . . . . . . .
. . . . . . . .
Vv17g0099 . . . . . . .
. . . . . . . .
. . . . .
Vv17g0100 . . . . . . .
. . . . . . . .
. . . . .
Vv17g0101 . . . . . . .
. . . . . . . .
. . . . .
Vv17g0102 . . . . . . .
. . . . . . . .
. . . . .
Vv17g0103 . . . . . . .
. . . . . . . .
. . . . .
Vv17g0104 . . . . . . .
. . . . . . . .
. . . . .
Vv17g0105 . . . . . . .
. . . . . . . .
. . . . .
Vv17g0106 . . . . . . .
. . . . . . . .
. . . . .
Vv17g0107 Tc03_g015050 Gorai.001G057900.1 . .
. . Vv14g1137 Tc04_g004290 .
Gorai.001G272800.1 Gorai.007G043300.1 . Gorai.
012G086000.1 . . . . . . .
Vv17g0108 Tc03_g012600 . . . . .
. . . . . . .
Vv01g1157 . . . . . .
Vv17g0109 . . . . . . .
. . . . . . . .

.        .        .        .        .
Vv17g0110        .        .        .        .        .        .        .
        .        .        .        .        .        .        .        .
        .        .        .        .        .
Vv17g0111        Tc03_g012570        .        .        .        .        .
        .        .        .        .        .        .        .        .
        .        .        .        .        .        .
Vv17g0112        .        .        .        .        Gorai.008G207100.1
        .        .        .        .        .        .        .        .
        .        .        .        .        .        .        .
Vv17g0113        .        .        .        .        .        .        .
        .        .        .        .        .        .        .        .
        .        .        .        .        .
Vv17g0114        Tc03_g012560        .        .        .        .        .
        .        .        .        .        .        .        .        .
        .        .        .        .        .        .
Vv17g0115        .        .        .        .        .        .        .
        .        .        .        .        .        .        .        .
        .        .        .        .        .
Vv17g0116        .        .        .        .        .        .
Vv14g1152        Tc05_g002060        .        .        .        .        .
        .        .        .        .        .        .        .
Vv17g0117        Tc03_g014940        .        .        .        Gorai.
008G206500.1        .        .        .        .        .        .        .
        .        .        .        .        .        .        .        .
Vv17g0118        .        .        .        .        .        .        .
        .        .        .        .        .        .        .        .
        .        .        .        .        .
Vv17g0119        Tc03_g014910        Gorai.001G058900.1        .        .
        .        Gorai.006G272000.1        .        .        .        .
        .        .        .        .        .        .        .        .
        .        .
Vv17g0120        Tc03_g014890        .        .        .        Gorai.
008G206400.1        .        .        .        .        .        .        .
        .        .        .        .        .        .        .        .
Vv17g0121        Tc03_g014880        Gorai.001G059000.1        .        .
        Gorai.008G206300.1        .        .        .        .        .
        .        .        .        .        .        .        .        .
        .        .
Vv17g0122        Tc03_g014860        .        .        .        .        .
        .        .        .        .        .        .        .        .
        .        .        .        .        .        .
Vv17g0123        Tc03_g014850        .        .        .        Gorai.
008G206100.1        Gorai.006G271900.1        .        .        .        .
        .        .        .        .        .        .        .        .
        .        .
Vv17g0124        Tc03_g014840        .        .        .        .        .
        .        .        .        .        .        .        .        .
        .        .        .        .        .        .
Vv17g0125        Tc03_g014820        .        .        .        Gorai.

008G206000.1 . . . . . . .
. . . . . . . .
Vv17g0126 Tc03_g014810 . . . Gorai.
008G205900.1 . . . . . . .
. . . . . . . .
Vv17g0127 . . . . . . .
. . . . . . . .
. . . . .
Vv17g0128 Tc03_g014790 . . . Gorai.
008G205800.1 . . . . . . .
. . . . . . . .
Vv17g0129 Tc03_g014780 Gorai.001G059200.1 .
Gorai.007G042700.1 . . . . . .
. . . . . . . .
. .
Vv17g0130 . . . . Gorai.008G205700.1
. . . . . . . .
. . . . . . .
Vv17g0131 Tc03_g014760 . . Gorai.007G042600.1
. Gorai.006G271700.1 . . . .
. . . . . . . .
. .
Vv17g0132 Tc03_g014750 . . Gorai.007G042500.1
. . . . . . . .
. . . . . . . .
Vv17g0133 Tc03_g014740 . . . Gorai.
008G205600.1 . Vv14g1171 . Gorai.011G084300.1
Gorai.001G270700.1 . Gorai.006G041400.1 .
. . . . . . .
Vv17g0134 Tc03_g014730 . . . .
Gorai.006G271600.1 . . . . . .
. . . . . . . .
Vv17g0135 Tc03_g014720 . . . . .
. . . . . . . .
. . . . . .
Vv17g0136 Tc03_g014710 . . . Gorai.
008G205500.1 . . . . . . .
. . . . . . . .
Vv17g0137 . . . . . . .
. . . . . . . .
. . . . .
Vv17g0138 Tc03_g014690 Gorai.001G059400.1 . .
Gorai.008G205400.1 . . . . .
. . . . . . . .
. .
Vv17g0139 . . . . . . .
. . . . . . . .
. . . . .
Vv17g0140 . . . . . . .
. . . . . . . .

```
        .       .       .       .       .
Vv17g0141       Tc03_g014680    .       .       .       .       .
        Vv14g1198       .       .       .       .       .       .
        .       .       .       .       .       .       .
Vv17g0142       .       .       .       .       .       .       .
        .       .       .       .       .       .       .       .
        .       .       .       .       .
Vv17g0143       .       .       .       .       .       .       .
        .       .       .       .       .       .       .       .
        .       .       .       .       .
Vv17g0144       .       .       .       .       .       .
Vv14g1199       .       Gorai.011G091700.1      Gorai.001G270100.1
        .       Gorai.006G041100.1      .       .       .       .
        .       .       .       .
Vv17g0145       Tc03_g014670    Gorai.001G059500.1      .
Gorai.007G042400.1      .       .       .       .       .       .
        .       .       .       .       .       .       .       .
        .       .
Vv17g0146       Tc03_g014660    Gorai.001G059600.1      .       .
        .       .       .       .       .       .       .       .
        .       .       .       .       .       .       .       .
Vv17g0147       .       .       .       .       .       .       .
        .       .       .       .       .       .       .       .
        .       .       .       .       .
Vv17g0148       Tc03_g014650    Gorai.001G059700.1      .       .
        .       .       .       .       .       .       .       .
        .       .       .       .       .       .       .       .
Vv17g0149       Tc03_g014640    .       .       .       .       .
        .       .       .       .       .       .       .       .
        .       .       .       .       .       .
Vv17g0150       .       .       .       .       .       .
Vv14g1207       Tc04_g002990    .       Gorai.001G269400.1      .
        .       Gorai.012G088300.1      .       .       .       .
        .       .       .
Vv17g0151       Tc03_g014630    Gorai.001G059900.1      .       .
        .       .       .       .       .       .       .       .
        .       .       .       .       .       .       .       .
Vv17g0152       .       .       .       .       .       .       .
        .       .       .       .       .       .       .       .
        .       .       .       .       .
Vv17g0153       Tc03_g014610    Gorai.001G060100.1      .       .
        Gorai.008G205300.1      .       .       .       .       .
        .       .       .       .       .       .       .       .
        .       .
Vv17g0154       Tc03_g014600    Gorai.001G060200.1      .
Gorai.007G042300.1      .       .       .       .       .       .
        .       .       .       .       .       .       .       .
        .       .
Vv17g0155       Tc03_g014590    Gorai.001G060300.1      .       .
        Gorai.008G205200.1      Gorai.006G271500.1      .       .
```

. . . . . . . .
. . . .
Vv17g0156 Tc03_g014580 . . . . .
. . . . . . . .
. . . . . .
Vv17g0157 . . . . . . .
. . . . . . . .
. . . . .
Vv17g0158 Tc03_g014570 Gorai.001G060400.1 . .
. . . . . . . .
. . . . . . . .
Vv17g0159 Tc03_g014530 . . . . .
. . . . . . . .
. . . . . .
Vv17g0160 . . . . . . .
. . . . . . . .
. . . . .
Vv17g0161 Tc03_g014520 Gorai.001G060500.1 . .
. Gorai.006G271400.1 . . . .
. . . . . . . .
. .
Vv17g0162 . . . . . . .
. . . . . . . .
. . . . .
Vv17g0163 Tc03_g014500 Gorai.001G060700.1 . .
Gorai.008G202600.1 Gorai.006G271000.1 Vv14g1222
. . . . . . . .
. . . . .
Vv17g0164 Tc03_g014480 . . . . .
. . . . . . . .
. . . . . .
Vv17g0165 Tc03_g014470 Gorai.001G060800.1 . .
. . . . . . . .
. . . . . . . .
Vv17g0166 Tc03_g014460 Gorai.001G060900.1 . .
. . . . . . . .
. . . . . . . .
Vv17g0167 Tc03_g014450 Gorai.001G061000.1 . .
. Gorai.006G268000.1 Vv14g1227
Tc04_g002810 Gorai.011G092600.1 . . Gorai.
006G039500.1 . . . . . . .
.
Vv17g0168 Tc03_g014440 . . . . .
Vv14g1230 Tc04_g002790 . Gorai.001G268800.1
. Gorai.006G039400.1 . . . .
. . . .
Vv17g0169 . . . . . . .
. . . . . . . .
. . . . .
Vv17g0170 . Gorai.001G061100.1 . . .

. . . . . . . .
. . . . . . .
Vv17g0171 . . . . . . .
. . . . . . . .
. . . . .
Vv17g0172 . . . Gorai.005G134600.1 .
Gorai.006G190500.1 . . . . .
. . . . . . . .
.
Vv17g0173 Tc03_g008530 . . . . .
. . . . . . . .
. . . . . .
Vv17g0174 . . . . . Gorai.
006G191100.1 . . . . . . .
. . . . . . .
Vv17g0175 . . . . . . .
. . . . . . . .
. . . . .
Vv17g0176 . . . . . . .
. . . . . . . .
. . . . .
Vv17g0177 Tc03_g007940 . . . . .
Vv14g1038 Tc04_g005480 . . . .
Gorai.012G082500.1 . . . . .
. .
Vv17g0178 . . . . . . .
. . . . . . . .
. . . . .
Vv17g0179 Tc03_g007920 . . . .
Gorai.006G191200.1 . . . . . .
. . . . . . . .
Vv17g0180 . . . . . . .
. . . . . . . .
. . . . .
Vv17g0181 Tc03_g007870 . . . . .
. . . . . . . .
. . . . . .
Vv17g0182 Tc03_g007830 . . Gorai.005G133600.1
. . . . . . . .
. . . . . . . .
Vv17g0183 . . . . . . .
. . . . . . . .
. . . . .
Vv17g0184 . . . . . . .
. . . . . . . .
. . . . .
Vv17g0185 . . . . . . .
. . . . . . . .
. . . . .
Vv17g0186 Tc03_g007760 . . . . .

. . . . . . . .
. . . . . .
Vv17g0187 . . . . . . .
. . . . . . . .
. . . . .
Vv17g0188 . . . . . . .
. . . . . . . .
. . . . .
Vv17g0189 . . . Gorai.005G133500.1 .
. Vv14g1040 Tc04_g005460 . . .
. . Vv01g0590 Tc02_g027880 Gorai.
002G172600.1 . . Gorai.008G291200.1 .
Vv17g0190 . . . . . . .
. . . . . . . .
. . . . .
Vv17g0191 . . . . . . .
. . . . . . . .
. . . . .
Vv17g0192 . . . . . . .
. . . . . . . .
. . . . .
Vv17g0193 . . . . . . .
. . . . . . . .
. . . . .
Vv17g0194 . . . . . . .
. . . . . . . .
. . . . .
Vv17g0195 . . . . . . .
. . . . . . . .
. . . . .
Vv17g0196 . . . . . . .
. . . . . . . .
. . . . .
Vv17g0197 . . . . . . .
. . . . . . . .
. . . . .
Vv17g0198 Tc03_g007360 . . . . .
. . . . . . . .
. . . . . .
Vv17g0199 Tc03_g007300 . . . . .
. . . . . . . .
. . . . . .
Vv17g0200 . . . Gorai.005G132400.1 .
. . . . . . . .
. . . . . . .
Vv17g0201 . . . . . . .
. . . . . . . .
. . . . .
Vv17g0202 Tc03_g007130 . . . . .
. . . . . . . .

. . . . . .
Vv17g0203 Tc03_g007120 . . Gorai.005G132200.1
. . . . . . . .
. . . . . . . .
Vv17g0204 . . . . . Gorai.
006G191400.1 . . . . . . .
. . . . . . .
Vv17g0205 . . . . . . .
. . . . . . . .
. . . . .
Vv17g0206 . . . . . . .
. . . . . . . .
. . . . .
Vv17g0207 . . . . . . .
. . . . . . . .
. . . . .
Vv17g0208 . . . . . . .
. . . . . . . .
. . . . .
Vv17g0209 . . . . . . .
. . . . . . . .
. . . . .
Vv17g0210 . . . . . . .
. . . . . . . .
. . . . .
Vv17g0211 . . . . . . .
. . . . . . . .
. . . . .
Vv17g0212 . . . . . . .
. . . . . . . .
. . . . .
Vv17g0213 Tc03_g006820 . . . . .
Vv01g0581 Tc02_g028050 Gorai.002G173700.1 .
Gorai.009G330500.1 Gorai.008G291000.1 . .
. . . . . .
Vv17g0214 . . . . . . .
. . . . . . . .
. . . . .
Vv17g0215 . . . . . . .
. . . . . . . .
. . . . .
Vv17g0216 . . . . . . .
. . . . . . . .
. . . . .
Vv17g0217 . . . . . . .
. . . . . . . .
. . . . .
Vv17g0218 . . . . . . .
. . . . . . . .
. . . . .

Vv17g0219 . . . . . . .
. . . . . . . .
. . . . .
Vv17g0220 . . . . . Gorai.
006G191700.1 . . . . . . .
. . . . . . .
Vv17g0221 . . . . . . .
. . . . . . . .
. . . . .
Vv17g0222 . . . . . . .
. . . . . . . .
. . . . .
Vv17g0223 Tc03_g003450 . . . . .
. . . . . . . .
. . . . . .
Vv17g0224 . . . . . . .
. . . . . . . .
. . . . .
Vv17g0225 . . . . . . .
. . . . . . . .
. . . . .
Vv17g0226 . . . . . . .
. . . . . . . .
. . . . .
Vv17g0227 . . . . . . .
. . . . . . . .
. . . . .
Vv17g0228 Tc03_g001650 . . . . .
. . . . . . . .
. . . . . .
Vv17g0229 . . . . . . .
. . . . . . . .
. . . . .
Vv17g0230 . . . . . . .
. . . . . . . .
. . . . .
Vv17g0231 . . . . . . .
. . . . . . . .
. . . . .
Vv17g0232 . . . . . . .
. . . . . . . .
. . . . .
Vv17g0233 . . . . . . .
. . . . . . . .
. . . . .
Vv17g0234 . . . . . . .
. . . . . . . .
. . . . .
Vv17g0235 . . . . . . .
. . . . . . . .

. . . . .
Vv17g0236 . . . . . . .
. . . . . . . .
. . . . .
Vv17g0237 . . . . . . .
. . . . . . . .
. . . . .
Vv17g0238 Tc03_g006240 . . . . .
. . . . . . . .
. . . . . .
Vv17g0239 Tc03_g001500 . . . . .
. . . . . . . .
. . . . . .
Vv17g0240 . . . . . Gorai.
006G191800.1 . . . . . . .
. . . . . . .
Vv17g0241 . . . . . . .
. . . . . . . .
. . . . .
Vv17g0242 Tc03_g003550 . . Gorai.005G132000.1
. . Vv01g0542 . . . .
Gorai.008G289900.1 . . . . .
. . .
Vv17g0243 . . . Gorai.005G131900.1 .
. Vv01g0520 . . . . .
. . . . . . . .
Vv17g0244 Tc03_g005600 . Gorai.006G049000.1 .
. Gorai.006G179900.1 . . . .
. . . . . . . .
. .
Vv17g0245 . . . . . . .
. . . . . . . .
. . . . .
Vv17g0246 Tc03_g005580 . . . . .
Vv01g0516 Tc02_g027160 . . .
Gorai.008G289800.1 . . . . . .
. .
Vv17g0247 . . . . . . .
. . . . . . . .
. . . . .
Vv17g0248 Tc03_g005540 . . . . .
. . . . . . . .
. . . . . .
Vv17g0249 Tc03_g005490 Gorai.001G087100.1 .
Gorai.005G131800.1 . . . . . .
. . . . . . . .
. .
Vv17g0250 . . . . . . .
. . . . . . . .
. . . . .

```
Vv17g0251        Tc03_g005400     .        .        .        .        .
        .        .        .        .        .        .        .        .
        .        .        .        .        .        .
Vv17g0252        Tc03_g005380     .        .        .        .
Gorai.006G192100.1        .        .        .        .        .        .
        .        .        .        .        .        .        .        .
Vv17g0253        .        .        .        .        .        .        .
        .        .        .        .        .        .        .        .
        .        .        .        .        .
Vv17g0254        Tc03_g005360     .        .        Gorai.005G131600.1
        .        .        .        .        .        .        .        .
        .        .        .        .        .        .        .        .
Vv17g0255        Tc03_g005310     .        .        .        .        .
        .        .        .        .        .        .        .        .
        .        .        .        .        .        .
Vv17g0256        Tc03_g005300     .        .        .        .        .
        .        .        .        .        .        .        .        .
        .        .        .        .        .        .
Vv17g0257        Tc03_g005280     .        .        .        .        .
        .        .        .        .        .        .        .        .
        .        .        .        .        .        .
Vv17g0258        .        .        .        .        .        .        .
        .        .        .        .        .        .        .        .
        .        .        .        .        .
Vv17g0259        Tc03_g005260     .        .        .        .        .
        .        .        .        .        .        .        .        .
        .        .        .        .        .        .
Vv17g0260        Tc03_g005250     .        .        .        .        .
        .        .        .        .        .        .        .        .
        .        .        .        .        .        .
Vv17g0261        .        .        .        .        .        .        .
        .        .        .        .        .        .        .        .
        .        .        .        .        .
Vv17g0262        .        .        .        Gorai.005G128600.1        .
        .        .        .        .        .        .        .        .
        .        .        .        .        .        .        .
Vv17g0263        .        .        .        .        .        .        .
        .        .        .        .        .        .        .        .
        .        .        .        .        .
Vv17g0264        .        .        .        .        .        Gorai.
006G192300.1     .        .        .        .        .        .        .
        .        .        .        .        .        .        .
Vv17g0265        .        Gorai.001G086600.1        .        .        .
        .        Vv01g0480        Tc02_g027530     Gorai.002G180100.1
        Gorai.005G162500.1        .        Gorai.008G166500.1        .
        .        .        .        .        .        .        .
Vv17g0266        .        .        .        .        .        .        .
        .        .        .        .        .        .        .        .
        .        .        .        .        .
Vv17g0267        .        .        .        .        .        .        .
```

. . . . . . . .
. . . . .
Vv17g0268 . . . . . . .
. . . . . . . .
. . . . .
Vv17g0269 . Gorai.001G086300.1 Gorai.006G057000.1
. . . . . . . .
. . . . . . . .
.
Vv17g0270 Tc03_g004840 . Gorai.006G057100.1 .
. . . . . . . .
. . . . . . . .
Vv17g0271 . . . . . Gorai.
006G192500.1 . . . . . . .
. . . . . . .
Vv17g0272 Tc03_g001100 . . Gorai.007G001800.1
. . . . . . . .
. . . . . . . .
Vv17g0273 . Gorai.001G069800.1 . . .
Gorai.006G192600.1 . . . . .
. . . . . . . .
.
Vv17g0274 . . Gorai.006G057200.1 . .
. . . . . . . .
. . . . . . .
Vv17g0275 . . . . . . .
. . . . . . . .
. . . . .
Vv17g0276 . . . . . . .
. . . . . . . .
. . . . .
Vv17g0277 . . . . . . .
. . . . . . . .
. . . . .
Vv17g0278 . . . . . . .
. . . . . . . .
. . . . .
Vv17g0279 . . . . . . .
. . . . . . . .
. . . . .
Vv17g0280 . . . . . . .
. . . . . . . .
. . . . .
Vv17g0281 . . . . . . .
. . . . . . . .
. . . . .
Vv17g0282 . . . . . . .
. . . . . . . .
. . . . .
Vv17g0283 . . . . . . .

. . . . . . . .
. . . . .
Vv17g0284 . . . . . . .
. . . . . . . .
. . . . .
Vv17g0285 . . . . . . .
. . . . . . . .
. . . . .
Vv17g0286 . . . . . . .
. . . . . . . .
. . . . .
Vv17g0287 . . . . . Gorai.
006G193100.1 . . . . . . .
. . . . . . .
Vv17g0288 . Gorai.001G084500.1 . . .
. . . . . . . .
. . . . . . .
Vv17g0289 . . . . . . .
. . . . . . . .
. . . . .
Vv17g0290 . . . . . . .
. . . . . . . .
. . . . .
Vv17g0291 . . . . . . .
. . . . . . . .
. . . . .
Vv17g0292 . . . . . .
Vv01g0449 . . . . . Gorai.
012G123700.1 . . . . . . .
Vv17g0293 Tc03_g004820 . . . . .
. . . . . . . .
. . . . . .
Vv17g0294 Tc03_g004810 . . . .
Gorai.006G178700.1 . . . . . .
. . . . . . . .
Vv17g0295 . . . . . Gorai.
006G178600.1 . . . . . . .
. . . . . . .
Vv17g0296 . . . . . . .
. . . . . . . .
. . . . .
Vv17g0297 Tc03_g004780 . . . .
Gorai.006G177800.1 . . . . . .
. . . . . . . .
Vv17g0298 . . . . . . .
. . . . . . . .
. . . . .
Vv17g0299 Tc03_g004770 . . . .
Gorai.006G193300.1 . . . . . .
. . . . . . . .

Vv17g0300 Tc03_g004760 . . . . .
Vv01g0446 . . . . . .
. . . . . . .
Vv17g0301 . . . . . . .
. . . . . . . .
. . . . .
Vv17g0302 . . . . . . .
. . . . . . . .
. . . . .
Vv17g0303 Tc03_g004730 . . . . .
Vv01g0445 Tc02_g024630 . Gorai.005G178900.1
. . . . . . . .
. .
Vv17g0304 . . . . . . .
. . . . . . . .
. . . . .
Vv17g0305 Tc03_g004720 . . . . .
Vv01g0443 Tc02_g024580 . Gorai.005G179100.1
. . . . . . . .
. .
Vv17g0306 . . Gorai.006G057500.1 . .
. . . . . . . .
. . . . . . .
Vv17g0307 . . . . . .
Vv01g0437 Tc02_g017590 . . . . .
. . . . . . .
Vv17g0308 . . . . . .
Vv01g0436 Tc02_g017610 . . . . .
. . . . . . .
Vv17g0309 . . . . . . .
. . . . . . . .
. . . . .
Vv17g0310 . . . . . Gorai.
006G193500.1 . . . . . . .
. . . . . . .
Vv17g0311 Tc03_g000690 . . . . .
. . . . . . . .
. . . . . .
Vv17g0312 . . . . . . .
. . . . . . . .
. . . . .
Vv17g0313 . . . . . . .
. . . . . . . .
. . . . .
Vv17g0314 . . . . . . .
. . . . . . . .
. . . . .
Vv17g0315 . . . . . . .
. . . . . . . .
. . . . .

Vv17g0316 . . . . . . .
. . . . . . .
. . . . .
Vv17g0317 . . . . . .
Vv01g0434 . . . . . . .
. . . . . .
Vv17g0318 . . . . . Gorai.
006G193600.1 Vv01g0433 . . . . .
. . . . . . .
Vv17g0319 . . . . . . .
. . . . . . .
. . . . .
Vv17g0320 . Gorai.001G068100.1 Gorai.006G057900.1
. . . . . . .
. . . . . . .
.
Vv17g0321 . . . . . . .
. . . . . . .
. . . . .
Vv17g0322 . Gorai.001G084000.1 . . .
. Vv14g0736 Tc04_g013830 . . .
. . Vv01g0422 . . . .
. .
Vv17g0323 . Gorai.001G068000.1 . . .
. . . . . . .
Vv01g0418 . . . Gorai.009G348900.1
. .
Vv17g0324 . Gorai.001G067900.1 . . .
. . . . . . .
. . . . . . .
Vv17g0325 . . . . . . .
. . . . . . Vv01g0415
. . . Gorai.009G348200.1 .
Gorai.012G124000.1
Vv17g0326 . . Gorai.006G058300.1 . .
. . . . . . .
. . . . . . .
Vv17g0327 Tc03_g027480 Gorai.001G067700.1 Gorai.
006G058500.1 . . . . . . .
. . . . . . .
. .
Vv17g0328 Tc03_g015780 . . . . .
. . . . . . .
. . . . . .
Vv17g0329 . . . . . . .
. . . . . . .
. . . . .
Vv17g0330 . Gorai.001G064700.1 . . .
. . . . . . .
. . . . . . .

```
Vv17g0331             .          .          .          .          .          .          .
            .         .          .          .          .          .          .          .
            .         .          .          .          .
Vv17g0332             Tc03_g004130          Gorai.001G067600.1               .          .
            .         .          .          .          .          .          .          .
            .         .          .          .          .          .          .          .
Vv17g0333             .          Gorai.001G075400.1               Gorai.006G059000.1
            .         .          .          .          .          .          .          .
            .         .          Vv01g0409             Tc02_g009450          .          .
            .         .          .
Vv17g0334             .          Gorai.001G075300.1               .          .          .
            .         .          .          .          .          .          .          .
            .         .          .          .          .          .          .
Vv17g0335             .          .          .          .          .          .          .
            .         .          .          .          .          .          .          .
            .         .          .          .          .
Vv17g0336             Tc03_g027470          .          .          .          .          .
            .         .          .          .          .          .          .          .
            .         .          .          .          .          .
Vv17g0337             .          .          .          .          .          .          .
            .         .          .          .          .          .          Vv01g0406
            .         .          .          .          .          .
Vv17g0338             .          .          .          .          .          .          .
            .         .          .          .          .          .          .          .
            .         .          .          .          .
Vv17g0339             .          .          .          .          .          .          .
            .         .          .          .          .          .          .          .
            .         .          .          .          .
Vv17g0340             .          .          .          .          .          .          .
            .         .          .          .          .          .          .          .
            .         .          .          .          .
Vv17g0341             .          .          .          .          .          .          .
            .         .          .          .          .          .          .          .
            .         .          .          .          .
Vv17g0342             .          .          .          .          .          .          .
            .         .          .          .          .          .          .          .
            .         .          .          .          .
Vv17g0343             .          .          .          .          .          .          .
            .         .          .          .          .          .          .          .
            .         .          .          .          .
Vv17g0344             .          .          Gorai.006G059300.1               .          .
            .         Vv14g0755             Tc04_g014320          .          .
Gorai.007G182700.1               .          .          .          .          .          .
            .         .          .
Vv17g0345             .          .          .          .          .          .          .
            .         .          .          .          .          .          .          .
            .         .          .          .          .
Vv17g0346             .          .          Gorai.006G059900.1               .          .
            .         .          .          .          .          .          .          .
            .         .          .          .          .          .          .
```

Vv17g0347 . . . . . . .
. . . . . . . .
. . . . .
Vv17g0348 . . . . . . .
. . . . . . . .
. . . . .
Vv17g0349 . . Gorai.006G060000.1 . .
. Vv14g0764 Tc04_g014420 . . .
Gorai.011G228800.1 . . . . .
. . .
Vv17g0350 . Gorai.001G081500.1 . . .
. . . . . . . .
Vv01g0387 . . . . .
Gorai.012G124200.1
Vv17g0351 . Gorai.001G081300.1 . . .
. . . . . . . .
Vv01g0384 . . Gorai.005G192100.1
Gorai.009G347900.1 . Gorai.012G124300.1
Vv17g0352 . . . . . . .
. . . . . . . .
. . . . .
Vv17g0353 . . . . . . .
. . . . . . Vv01g0381
. . . . . .
Vv17g0354 . . . . . . .
. . . . . . . .
. . . . .
Vv17g0355 . Gorai.001G081000.1 . . .
. Vv14g0771 Tc04_g014610 . . .
. . Vv01g0374 . . . .
. .
Vv17g0356 . . . . . . .
. . . . . . . .
. . . . .
Vv17g0357 . . . . . . .
. . . . . . . .
. . . . .
Vv17g0358 . . . . . Gorai.
006G194200.1 . . . . . . .
. . . . . . .
Vv17g0359 . . . . . . .
. . . . . . . .
. . . . .
Vv17g0360 . . . . . . .
. . . . . . . .
. . . . .
Vv17g0361 . . Gorai.006G060100.1 . .
. Vv14g0777 Tc04_g014640 . . .
. . . . . . . .
.

Vv17g0362 . Gorai.001G075100.1 Gorai.006G060200.1
. . Gorai.006G194400.1 Vv14g0780
Tc04_g014680 . . . . . . .
. . . . .
Vv17g0363 . . . . . . .
. . . . . . . .
. . . . .
Vv17g0364 Tc03_g015710 . . . . .
. . . . . . . .
. . . . . .
Vv17g0365 . . . . . . .
. . . . . . . .
. . . . .
Vv17g0366 . . . . . . .
. . . . . . . .
. . . . .
Vv17g0367 . . . . . . .
. . . . . . . .
. . . . .
Vv17g0368 . . . . . . .
. . . . . . . .
. . . . .
Vv17g0369 Tc03_g015620 Gorai.001G074900.1 Gorai.
006G060400.1 Gorai.007G044400.1 . Gorai.006G194500.1
. . . . . . . .
. . . . . .
Vv17g0370 . . Gorai.006G060500.1 . .
. . . . . . . .
. . . . . . .
Vv17g0371 . . . . . . .
. . . . . . . .
. . . . .
Vv17g0372 . . . . . . .
. . . . . . . .
. . . . .
Vv17g0373 . . . . . . .
. . . . . . . .
. . . . .
Vv17g0374 . Gorai.001G071900.1 . . .
. . . . . . . .
. . . . . . .
Vv17g0375 . . Gorai.006G060600.1 . .
. . . . . . . .
. . . . . . .
Vv17g0376 . . . . . . .
. . . . . . . .
. . . . .
Vv17g0377 . . . . . . .
. . . . . . . .
. . . . .

Vv17g0378 Tc03_g023390 . . Gorai.007G031300.1
. . . . . . . .
. . . . . . . .
Vv17g0379 . . . . . . .
. . . . . . . .
. . . . .
Vv17g0380 . . . . . . .
. . . . . . . .
. . . . .
Vv17g0381 . . . . . Gorai.
006G194600.1 . . . . . . .
. . . . . . .
Vv17g0382 . . . . . . .
. . . . . . . .
. . . . .
Vv17g0383 . . . . . .
Vv14g0790 Tc04_g014790 . . Gorai.007G181500.1
. . Vv01g0353 . . Gorai.
005G190600.1 . . .
Vv17g0384 . . . . . . .
. . . . . . . .
. . . . .
Vv17g0385 . . . . . . .
. . . . . . . .
. . . . .
Vv17g0386 . . . . . . .
. . . . . . . .
. . . . .
Vv17g0387 . . . . . . .
. . . . . . . .
. . . . .
Vv17g0388 . . . . . . .
. . . . . . . .
. . . . .
Vv17g0389 . . . . . . .
. . . . . . . .
. . . . .
Vv17g0390 . . Gorai.006G061200.1 . .
. Vv14g0795 Tc04_g014830 . . .
. . Vv01g0351 Tc02_g010100 Gorai.
002G217800.1 Gorai.005G190300.1 . . .
Vv17g0391 . . . . . . .
. . . . . . . .
. . . . .
Vv17g0392 . . Gorai.006G061400.1 . .
. . . . . . . .
. . . . . . .
Vv17g0393 . . . . . . .
. . . . . . . .
. . . . .

Vv17g0394 . . . . . . .
. . . . . . Vv01g0347
. Gorai.002G218400.1 . . . .
Vv17g0395 . . . . . . .
. . . . . . . .
. . . . .
Vv17g0396 . . . . . . .
. . . . . . . .
. . . . .
Vv17g0397 . . . . . . .
. . . . . . . .
. . . . .
Vv17g0398 . . . . . . .
. . . . . . . .
. . . . .
Vv17g0399 . . . . . . .
. . . . . . . .
. . . . .
Vv17g0400 . . . . . . .
. . . . . . . .
. . . . .
Vv17g0401 . . . . . Gorai.
006G194900.1 . . . . . . .
. . . . . . .
Vv17g0402 . . . . . . .
. . . . . . . .
. . . . .
Vv17g0403 . . Gorai.006G061500.1 .
Gorai.008G184200.1 Gorai.006G195000.1 . . .
. . . . . . . .
. . .
Vv17g0404 Tc03_g023370 . . . Gorai.
008G184400.1 Gorai.006G195100.1 . . . .
. . . Vv01g0323 Tc02_g034330 .
. . Gorai.008G184400.1 .
Vv17g0405 . . . . . . .
. . . . . . . .
. . . . .
Vv17g0406 . . . . Gorai.008G184500.1
. . . . . . . .
Vv01g0322 Tc02_g034340 Gorai.013G081300.1 .
. Gorai.008G184500.1 .
Vv17g0407 . . . . . . .
. . . . . . . .
. . . . .
Vv17g0408 Tc03_g013740 . . . . .
. . . . . . . .
. . . . . .
Vv17g0409 . . . . . . .
. . . . . . . .

. . . . .
Vv17g0410 Tc03_g013710 . . . . . .
. . . . . . . .
. . . . . .
Vv17g0411 Tc03_g013700 . . . . . .
. . . . . . . .
. . . . . .
Vv17g0412 . . . . . . .
. . . . . . . .
. . . . .
Vv17g0413 Tc03_g013660 . . . . .
. . . . . . . .
. . . . . .
Vv17g0414 . . . . . . .
. . . . . . . .
. . . . .
Vv17g0415 Tc03_g013620 . . Gorai.007G041400.1
. . . . . . . .
. . . . . . . .
Vv17g0416 Tc03_g007350 . . . .
Gorai.006G195200.1 . . . . . .
. . . . . . . .
Vv17g0417 . . Gorai.006G062300.1 .
Gorai.008G184700.1 Gorai.006G195300.1 Vv14g0817 .
. . . . . Vv01g0318
Tc02_g034400 . . . Gorai.008G184800.1 .
Vv17g0418 . . . . . . .
. . . . . . . .
. . . . .
Vv17g0419 . . Gorai.006G062600.1 . .
. . . . . . . .
. . . . . . .
Vv17g0420 . . . . . Gorai.
006G195500.1 . . . . . . .
. . . . . . .
Vv17g0421 Tc03_g031200 . . . . .
. . . . . . .
Vv01g0314 Tc02_g034440 . . . . .
Vv17g0422 . . . . . . .
. . . . . . . .
. . . . .
Vv17g0423 . . . . . . .
. . . . . . . .
. . . . .
Vv17g0424 . . . . . . .
. . . . . . . .
. . . . .
Vv17g0425 . . . . . . .
. . . . . . . .
. . . . .

Vv17g0426 . . . . . . .
. . . . . . . .
. . . . .
Vv17g0427 . . . . . . .
. . . . . . Vv01g0309
Tc02_g034480 Gorai.013G080300.1 Gorai.005G138200.1
. . .
Vv17g0428 Tc03_g007010 . . Gorai.007G003000.1
. . Vv14g0832 Tc04_g015170 . .
. . . . . . . .
. .
Vv17g0429 . . . . . . .
. . . . . . . .
. . . . .
Vv17g0430 . . . . . . .
. . . . . . . .
. . . . .
Vv17g0431 . . . . . . .
. . . . . . . .
. . . . .
Vv17g0432 . . . . . . .
. . . . . . . .
. . . . .
Vv17g0433 . . Gorai.006G063000.1 . .
. . . . . . . .
. . . . . . .
Vv17g0434 . . . . . . .
. . . . . . . .
. . . . .
Vv17g0435 . . . . . . .
. . . . . . . .
. . . . .
Vv17g0436 . . . . Gorai.008G186500.1
. . . . . . . .
Vv01g0291 Tc02_g034610 . . .
Gorai.008G186500.1 .
Vv17g0437 . . Gorai.006G063200.1 . .
. . . . . . . .
Vv01g0290 Tc02_g034630 Gorai.013G079900.1
Gorai.005G137800.1 . . .
Vv17g0438 . . . . . . .
. . . . . . . .
. . . . .
Vv17g0439 . . Gorai.006G063300.1 . .
. . . . . . . .
. . . . . . .
Vv17g0440 . . . . Gorai.008G186800.1
. . . . . . . .
Vv01g0287 Tc02_g034650 . . .
Gorai.008G186800.1 .

Vv17g0441 . . . . . . .
. . . . . . . .
. . . . .
Vv17g0442 . . . . . . .
. . . . . . . .
. . . . .
Vv17g0443 . . . . . . .
. . . . . . . .
. . . . .
Vv17g0444 . . . . Gorai.008G187000.1
. . . . . . . .
Vv01g0282 Tc02_g034690 . . .
Gorai.008G187000.1 .
Vv17g0445 . . Gorai.006G063500.1 . .
. Vv14g0845 . . . . .
. . . . . . . .
Vv17g0446 . . . . . .
Vv14g0846 Tc04_g015310 . . . . .
. . . . . . .
Vv17g0447 . . . . . . .
. . . . . . . .
. . . . .
Vv17g0448 . . . . . . .
. . . . . . . .
. . . . .
Vv17g0449 . . . . . . .
. . . . . . . .
. . . . .
Vv17g0450 . . . . . . .
. . . . . . . .
. . . . .
Vv17g0451 . . . . . . .
. . . . . . . .
. . . . .
Vv17g0452 Tc03_g013520 . . Gorai.007G041300.1
. . . . . . . .
. . . . . . . .
Vv17g0453 . . . . . . .
. . . . . . . .
. . . . .
Vv17g0454 Tc03_g013510 . . . . .
. . . . . . . .
. . . . . .
Vv17g0455 . . . . . . .
. . . . . . . .
. . . . .
Vv17g0456 Tc03_g013500 . . . . .
. . . . . . . .
. . . . . .
Vv17g0457 Tc03_g013490 . . Gorai.007G041200.1

```
        Gorai.008G187800.1      .       .       .       .       .
        .       .       .       Vv01g0274       Tc02_g034770    .
        .       .       Gorai.008G187800.1      .
Vv17g0458       Tc03_g013480    Gorai.001G064700.1      .       .
        .       Gorai.006G269400.1      .       .       .       .
        .       .       .       .       .       .       .       .
        .       .
Vv17g0459       .       .       .       .       .       .       .
        .       .       .       .       .       .       .       .
        .       .       .       .       .
Vv17g0460       Tc03_g031220    .       .       .       .       .
        .       .       .       .       .       .       .       .
        .       .       .       .       .       .
Vv17g0461       .       .       .       .       .       .       .
        .       .       .       .       .       .       .       .
        .       .       .       .       .
Vv17g0462       .       .       .       .       .       .       .
        .       .       .       .       .       .       .       .
        .       .       .       .       .
Vv17g0463       .       .       .       .       .       .       .
        .       .       .       .       .       .       .       .
        .       .       .       .       .
Vv17g0464       .       .       .       .       .       .       .
        .       .       .       .       .       .       .       .
        .       .       .       .       .
Vv17g0465       .       .       .       .       .       .       .
        .       .       .       .       .       .       .       .
        .       .       .       .       .
Vv17g0466       .       .       .       .       .       .       .
        .       .       .       .       .       .       .       .
        .       .       .       .       .
Vv17g0467       Tc03_g013470    .       .       .       .       .
        .       .       .       .       .       .       .       .
        .       .       .       .       .       .
Vv17g0468       Tc03_g013460    .       .       .       .       .
        .       .       .       .       .       .       .
Vv01g0251       Tc02_g034900    .       .       .       Gorai.
008G188500.1    .
Vv17g0469       .       Gorai.001G064800.1      .       .
Gorai.008G188500.1      .       .       .       .       .       .
        .       .       .       .       .       .       .       .
        .
Vv17g0470       Tc03_g013450    .       .       Gorai.007G041100.1
        Gorai.008G188600.1      .       .       .       .       .
        .       .       .       .       .       .       .       .
        .       .
Vv17g0471       Tc03_g013430    Gorai.001G064900.1      .       .
        .       .       .       .       .       .       .       .
        .       .       .       .       .       .       .       .
Vv17g0472       Tc03_g013410    .       .       .       .       .
```

```
         .         .         .         .         .         .         .         .
         .         .         .         .         .         .
Vv17g0473           Tc03_g013400        Gorai.001G065100.1           .         .
         .         .         .         .         .         .         .         .
         .         .         .         .         .         .         .         .
Vv17g0474           Tc03_g013390       .         .         .         .         .
         .         .         .         .         .         .         .         .
         .         .         .         .         .         .
Vv17g0475          .         .         .         .         .         .         .
         .         .         .         .         .         .         .         .
         .         .         .         .         .
Vv17g0476           Tc03_g013380       .         .         .         .         .
         .         .         .         .         .         .         .         .
         .         .         .         .         .         .
Vv17g0477           Tc03_g013370        Gorai.001G065200.1           .         .
         .         .         .         .         .         .         .         .
         .         .         .         .         .         .         .         .
Vv17g0478          .         .         .         .         .         .         .
         .         .         .         .         .         .         .         .
         .         .         .         .         .
Vv17g0479           Tc03_g013340       .         .         .         .         .
         .         .         .         .         .         .         .         .
         .         .         .         .         .         .
Vv17g0480           Tc03_g013330        Gorai.001G065400.1           .
Gorai.007G040900.1           .         .         .         .         .         .
         .         .         .         .         .         .         .         .
         .         .
Vv17g0481           Tc03_g013320       .         .         .         .         .
         .         .         .         .         .         .         .         .
         .         .         .         .         .         .
Vv17g0482          .         .         .         .         .         .
Vv14g0878          .         .         .         .         .         .         .
         .         .         .         .         .         .
Vv17g0483          .         .         .         .          Gorai.008G201500.1
         .         .         .         .         .         .         .         .
         .         .         .         .         .         .         .
Vv17g0484          .          Gorai.001G065600.1           .         .         .
         .         .         .         .         .         .         .         .
         .         .         .         .         .         .         .
Vv17g0485          .         .         .         .         .         .         .
         .         .         .         .         .         .         .         .
         .         .         .         .         .
Vv17g0486          .          Gorai.001G065700.1           .         .         .
         .         .         .         .         .         .         .         .
         .         .         .         .         .         .         .
Vv17g0487           Tc03_g013310       .         .         .         .         .
         .         .         .         .         .         .         .         .
         .         .         .         .         .         .
Vv17g0488          .         .         .         .         .         .         .
         .         .         .         .         .         .         .         .
```

. . . . .
Vv17g0489 Tc03_g013300 . . . Gorai.
008G173900.1 . . . . . . .
. . . . . . . .
Vv17g0490 . . . . . . .
. . . . . . . .
. . . . .
Vv17g0491 . . . . . . .
. . . . . . . .
. . . . .
Vv17g0492 Tc03_g013270 Gorai.001G065800.1 . .
Gorai.008G201400.1 . . . . .
. . . . . . . .
. .
Vv17g0493 Tc03_g013260 . . . Gorai.
008G174100.1 . . . . . . .
. . . . . . . .
Vv17g0494 Tc03_g013240 Gorai.001G065900.1 . .
. . . . . . . .
. . . . . . . .
Vv17g0495 Tc03_g013230 Gorai.001G066000.1 . .
Gorai.008G201200.1 . . . . .
. . . . . . . .
. .
Vv17g0496 . . . . . . .
. . . . . . . .
. . . . .
Vv17g0497 . . . . . Gorai.
006G269300.1 . . . . . . .
. . . . . . .
Vv17g0498 . . . . . . .
. . . . . . . .
. . . . .
Vv17g0499 Tc03_g013160 . . . . .
. . . . . . . .
. . . . . .
Vv17g0500 Tc03_g013150 . . . . .
. . . . . . . .
. . . . . .
Vv17g0501 . . . . . . .
. . . . . . . .
. . . . .
Vv17g0502 . . . . . . .
. . . . . . . .
. . . . .
Vv17g0503 . . . . . . .
. . . . . . . .
. . . . .
Vv17g0504 . . . . . . .
. . . . . . . .

```
       .        .        .        .        .
Vv17g0505        Tc03_g012880     .        .        Gorai.007G040800.1
        Gorai.008G200700.1        .        .        .        .        .
        .        .        .        .        .        .        .        .
        .        .
Vv17g0506        .        .        .        .        .        .        .
        .        .        .        .        .        .        .        .
        .        .        .        .        .
Vv17g0507        Tc03_g012860     .        .        .        .        .
        .        .        .        .        .        .        .        .
        .        .        .        .        .        .
Vv17g0508        Tc03_g012850     .        .        .        .        .
        .        .        .        .        .        .        .        .
        .        .        .        .        .        .
Vv17g0509        Tc03_g012800     .        .        .        .        .
        .        .        .        .        .        .        .        .
        .        .        .        .        .        .
Vv17g0510        .        .        .        .        .        .        .
        .        .        .        .        .        .        .        .
        .        .        .        .        .
Vv17g0511        Tc03_g012780     Gorai.001G066400.1        .        .
        .        .        .        .        .        .        .        .
        .        .        .        .        .        .        .        .
Vv17g0512        Tc03_g012770     Gorai.001G066500.1        .        .
        Gorai.008G175000.1        .        Vv14g0917        .        .
        .        .        .        .        .        .        .        .
        .        .        .
Vv17g0513        Tc03_g012760     .        .        .        Gorai.
008G175100.1     Gorai.006G269200.1        Vv14g0921        .        .
        .        Gorai.007G040600.1        .        .        .        .
        .        .        .        .        .
Vv17g0514        Tc03_g012750     Gorai.001G066600.1        .
Gorai.007G040500.1        .        .        Vv14g0922        .        .
        .        .        .        .        .        .        .        .
        .        .        .
Vv17g0515        .        .        .        .        .        .        .
        .        .        .        .        .        .        .        .
        .        .        .        .        .
Vv17g0516        .        .        .        .        .        .        .
        .        .        .        .        .        .        .        .
        .        .        .        .        .
Vv17g0517        Tc03_g012730     Gorai.001G066900.1        .        .
        .        .        .        .        .        .        .        .
        .        .        .        .        .        .        .        .
Vv17g0518        .        Gorai.001G067000.1        .        .        .
        Gorai.006G268800.1        .        .        .        .        .
        .        .        .        .        .        .        .        .
        .
Vv17g0519        .        Gorai.001G067300.1        .        .        .
        Gorai.006G268700.1        .        .        .        .        .
```

| | | | | | | | |
|---|---|---|---|---|---|---|---|
| . | . | . | . | . | . | . | . |
| . | | | | | | | |
| Vv17g0520 | . | . | . | . | Gorai.008G200500.1 | | |
| . | . | . | . | . | . | . | . |
| . | . | . | . | . | . | . | |
| Vv17g0521 | . | . | . | . | . | . | . |
| . | . | . | . | . | . | . | . |
| . | . | . | . | . | | | |
| Vv17g0522 | . | . | . | . | . | . | . |
| . | . | . | . | . | . | . | . |
| . | . | . | . | . | | | |
| Vv17g0523 | . | . | . | . | . | . | . |
| . | . | . | . | . | . | . | . |
| . | . | . | . | . | | | |
| Vv17g0524 | . | . | . | . | Gorai.008G200400.1 | | |
| . | . | . | . | . | . | . | . |
| . | . | . | . | . | . | . | |
| Vv17g0525 | . | Gorai.001G067500.1 | | | . | . | . |
| . | Vv14g0926 | | . | . | . | . | . |
| . | . | . | . | . | . | . | . |
| Vv17g0526 | . | . | . | . | . | . | . |
| . | . | . | . | . | . | . | . |
| . | . | . | . | . | | | |
| Vv17g0527 | . | . | . | . | . | . | . |
| . | . | . | . | . | . | . | . |
| . | . | . | . | . | | | |
| Vv17g0528 | . | . | . | . | . | . | . |
| . | . | . | . | . | . | . | . |
| . | . | . | . | . | | | |
| Vv17g0529 | . | . | . | . | . | . | . |
| . | . | . | . | . | . | . | . |
| . | . | . | . | . | | | |
| Vv17g0530 | . | . | . | . | . | . | . |
| . | . | . | . | . | . | . | . |
| . | . | . | . | . | | | |
| Vv17g0531 | . | . | . | . | Gorai.008G200300.1 | | |
| . | . | . | . | . | . | . | . |
| . | . | . | . | . | . | . | |
| Vv17g0532 | . | . | . | . | . | . | . |
| . | . | . | . | . | . | . | . |
| . | . | . | . | . | | | |
| Vv17g0533 | . | . | . | . | . | . | . |
| . | . | . | . | . | . | . | . |
| . | . | . | . | . | | | |
| Vv17g0534 | . | . | . | . | . | . | . |
| . | . | . | . | . | . | . | . |
| . | . | . | . | . | | | |
| Vv17g0535 | . | . | . | . | . | . | . |
| . | . | . | . | . | . | . | . |
| . | . | . | . | . | | | |

Vv17g0536 . . . . . . .
. . . . . . . .
. . . . .
Vv17g0537 Tc03_g012650 Gorai.001G075600.1 .
Gorai.007G039900.1 . . . . . .
. . . . . . . .
. .
Vv17g0538 Tc03_g012620 . . . . .
Vv14g0934 Tc05_g031790 . . . .
. . . . . . . .
Vv17g0539 Tc03_g012600 Gorai.001G075800.1 . .
. Gorai.006G268400.1 Vv14g0936 . .
. . . . . . . .
. . .
Vv17g0540 . . . . . . .
. . . . . . . .
. . . . .
Vv17g0541 . . . . . . .
. . . . . . . .
. . . . .
Vv17g0542 Tc03_g012560 Gorai.001G076300.1 . .
Gorai.008G200200.1 . Vv14g0941 . .
Gorai.001G208000.1 Gorai.007G039800.1 . .
. . . . . . .
Vv17g0543 . . . . . . .
. . . . . . . .
. . . . .
Vv17g0544 Tc03_g012500 . . . . .
. . . . . . . .
. . . . . .
Vv17g0545 . . . . . . .
. . . . . . . .
. . . . .
Vv17g0546 Tc03_g012490 Gorai.001G076200.1 . .
Gorai.008G200100.1 . Vv14g0948 . .
. . . . . . . .
. . .
Vv17g0547 Tc03_g012460 . . . Gorai.
008G200000.1 . Vv14g0949 . . Gorai.
001G208300.1 . . . . . . .
. . .
Vv17g0548 . . . . . . .
. . . . . . . .
. . . . .
Vv17g0549 Tc03_g012440 Gorai.001G076000.1 . .
. . . . . . . .
. . . . . . . .
Vv17g0550 Tc03_g012430 . . . Gorai.
008G199900.1 . . . . . . .
. . . . . . . .

Vv17g0551 . . . . . . .
. . . . . . . .
. . . . .
Vv17g0552 . . . . . . .
. . . . . . . .
. . . . .
Vv17g0553 . . . . . . .
. . . . . . . .
. . . . .
Vv17g0554 Tc03_g012420 . . . . .
. . . . . . . .
. . . . . .
Vv17g0555 . . . . . . .
. . . . . . . .
. . . . .
Vv17g0556 . . . . . . .
. . . . . . . .
. . . . .
Vv17g0557 . . . . . . .
. . . . . . . .
. . . . .
Vv17g0558 . . . . . . .
. . . . . . . .
. . . . .
Vv17g0559 . . . . . . .
. . . . . . . .
. . . . .
Vv17g0560 Tc03_g012380 . . . . .
. . . . . . . .
. . . . . .
Vv17g0561 . . . . . . .
. . . . . . . .
. . . . .
Vv17g0562 Tc03_g012370 . . . . .
. . . . . . . .
. . . . . .
Vv17g0563 Tc03_g012360 . . . .
Gorai.006G268300.1 . . . . . .
. . . . . . . .
Vv17g0564 . . . . . . .
. . . . . . . .
. . . . .
Vv17g0565 Tc03_g012320 . . . Gorai.
008G199700.1 . . . . . . .
. . . . . . . .
Vv17g0566 Tc03_g012300 . . . .
Gorai.006G268200.1 . . . . . .
. . . . . . . .
Vv17g0567 Tc03_g012260 . . . . .
Vv14g0985 Tc04_g026400 . . Gorai.

007G039600.1 Gorai.006G009900.1 . . . .
. . . .
Vv17g0568 . . . . Gorai.008G199600.1
Gorai.006G265200.1 . . . . .
. . . . . . . .
.
Vv17g0569 Tc03_g012250 . . . . .
. . . . . . . .
. . . . . .
Vv17g0570 Tc03_g012240 . . . . .
. . . . . . . .
. . . . . .
Vv17g0571 . . . . . . .
. . . . . . . .
. . . . .
Vv17g0572 . . . . . . .
. . . . . . . .
. . . . .
Vv17g0573 . . . . Gorai.008G196700.1
. . . . . . . .
. . . . . . .
Vv17g0574 . . . . Gorai.008G189500.1
. . . . . . . .
. . . . . . .
Vv17g0575 . . . . Gorai.008G189600.1
. . . . . . . .
. . . . . . .
Vv17g0576 . . . . Gorai.008G189700.1
. Vv14g1504 . . . . .
. Vv01g0892 . . . Gorai.
009G346100.1 Gorai.008G189700.1 .
Vv17g0577 . . . . . . .
. . . . . . . .
. . . . .
Vv17g0578 . . . . . . .
. . . . . . . .
. . . . .
Vv17g0579 . Gorai.001G074900.1 . . .
. . . . . . . .
. . . . . . .
Vv17g0580 . . . . Gorai.008G189800.1
. . . . . . . .
. . . . . . .
Vv17g0581 . . . . . . .
. . . . . . . .
. . . . .
Vv17g0582 Tc03_g029670 . . . . .
. . . . . . . .
. . . . . .
Vv17g0583 . . . . . . .

```
          .         .         .         .         .         .         Vv01g0907
          Tc02_g022530        .         Gorai.005G189000.1            .         .
          .
Vv17g0584           .         .         .         .         .         .         .
          .         .         .         .         .         .         .         .
          .         .         .         .         .
Vv17g0585           .         .         .         .         .         .         .
          .         .         .         .         .         .         .         .
          .         .         .         .         .
Vv17g0586           .         .         .         .         .         .         .
          .         .         .         .         .         .         .         .
          .         .         .         .         .
Vv17g0587           .         .         .         .         Gorai.008G189900.1
          .         .         .         .         .         .         .         .
          .         .         .         .         .         .         .
Vv17g0588           .         .         .         .         .         .         .
          .         .         .         .         .         .         .         .
          .         .         .         .         .
Vv17g0589           Tc03_g009010        .         .         .         .         .
          .         .         .         .         .         .         .
Vv01g0912           .         .         .         .         .         .
Vv17g0590           .         .         .         .         .         .         .
          .         .         .         .         .         .         .         .
          .         .         .         .         .
Vv17g0591           Tc03_g009030        Gorai.001G074100.1            .         .
          .         .         .         .         .         .         .         .
          .         .         .         .         .         .         .         .
Vv17g0592           .         .         .         .         .         .         .
          .         .         .         .         .         .         .         .
          .         .         .         .         .
Vv17g0593           Tc03_g009040        .         .         Gorai.007G034700.1
          Gorai.008G190000.1           .         .         .         .         .
          .         .         .         .         .         .         .         .
          .         .
Vv17g0594           .         .         .         .         Gorai.008G190100.1
          .         .         .         .         .         .         .         .
          .         .         .         .         .         .         .
Vv17g0595           .         .         .         .         .         .         .
          .         .         .         .         .         .         .         .
          .         .         .         .         .
Vv17g0596           .         .         .         .         .         .
Vv14g1533           .         .         .         .         .         .         .
          .         .         .         .         .         .
Vv17g0597           Tc03_g029740        .         .         .         .         .
          .         .         .         .         .         .         .         .
          .         .         .         .         .         .
Vv17g0598           .         .         .         .         .         .         .
          .         .         .         .         .         .         .         .
          .         .         .         .         .
Vv17g0599           Tc03_g030060        .         .         .         .         .
```

```
        .        .       .        .       .        .       .        .
        .        .       .        .       .        .
Vv17g0600        .       .        .       .        .       .
Vv14g1539        .       .        .       .        .       .        .
        .        .       .        .       .        .
Vv17g0601        .       .        .       .        .       .        .
        .        .       .        .       .        .       .        .
        .        .       .        .       .
Vv17g0602        .       .        .       .        .       .        .
        .        .       .        .       .        .       .        .
        .        .       .        .       .
Vv17g0603        .       .        .       .        .       .        .
        .        .       .        .       .        .       Vv01g0931
        Tc02_g034200     .        .       .        .       .
Vv17g0604        .       .        .       .        Gorai.008G190200.1
        .        .       .        .       .        .       .        .
        .        .       .        .       .        .       .
Vv17g0605        Tc03_g009500     Gorai.001G074000.1       .
Gorai.007G035200.1       .        .       .        .       .        .
        .        .       .        .       .        .       .        .
        .        .
Vv17g0606        .       Gorai.001G073900.1        .       .        .
        .        .       .        .       .        .       .        .
        .        .       .        .       .        .       .
Vv17g0607        Tc03_g009510     .       .        Gorai.007G035300.1
        .        .       .        .       .        .       .        .
        .        .       .        .       .        .       .        .
Vv17g0608        .       .        .       .        .       .        .
        .        .       .        .       .        .       .        .
        .        .       .        .       .
Vv17g0609        .       .        .       .        .       .        .
        .        .       .        .       .        .       .        .
        .        .       .        .       .
Vv17g0610        .       .        .       .        .       .        .
        .        .       .        .       .        .       .        .
        .        .       .        .       .
Vv17g0611        .       .        .       .        .       .        .
        .        .       .        .       .        .       .        .
        .        .       .        .       .
Vv17g0612        .       .        .       .        .       .        .
        .        .       .        .       .        .       .        .
        .        .       .        .       .
Vv17g0613        .       .        .       .        .       .        .
        .        .       .        .       .        .       .        .
        .        .       .        .       .
Vv17g0614        Tc03_g009530     Gorai.001G073800.1       .        .
        .        .       .        .       .        .       .        .
        .        .       .        .       .        .       .        .
Vv17g0615        Tc03_g009540     Gorai.001G073600.1       .        .
        Gorai.008G190300.1        .       .        .       .        .
```

. . . Vv01g0937 Tc02_g021810 .
Gorai.005G189800.1 . Gorai.008G190300.1 .
Vv17g0616 Tc03_g009550 . . . . .
Vv14g1543 Tc04_g025970 . . . .
. Vv01g0940 Tc02_g034120 . . .
. .
Vv17g0617 Tc03_g009560 . . . . .
. . . . . . . .
. . . . . .
Vv17g0618 . . . . . . .
. . . . . . . .
. . . . .
Vv17g0619 . . . . . . .
. . . . . . . .
. . . . .
Vv17g0620 Tc03_g009570 Gorai.001G073500.1 . .
Gorai.008G182800.1 . . . . .
. . . Vv01g0950 Tc02_g034050 .
. . Gorai.008G182800.1 .
Vv17g0621 Tc03_g009580 . . . Gorai.
008G182700.1 . Vv14g1551 . . . .
. . Vv01g0951 Tc02_g034040 . .
. Gorai.008G190600.1 .
Vv17g0622 Tc03_g009590 Gorai.001G073200.1 . .
. . . . . . . .
. . . . . . . .
Vv17g0623 . . . . . . .
. . . . . . . .
. . . . .
Vv17g0624 . . . . . . .
. . . . . . . .
. . . . .
Vv17g0625 Tc03_g009670 . . . . .
Vv14g1554 . . . . . .
Vv01g0953 Tc02_g034020 . . . .
.
Vv17g0626 Tc03_g009680 . . . Gorai.
008G190900.1 . . . . . . .
. . . . . . . .
Vv17g0627 Tc03_g009710 . . . Gorai.
008G191000.1 . . . . . . .
. . . . . . . .
Vv17g0628 . . . . . . .
. . . . . . . .
. . . . .
Vv17g0629 Tc03_g009780 . . Gorai.007G035700.1
. . . . . . . .
. . . . . . . .
Vv17g0630 . . . . . .
Vv14g1559 Tc05_g023660 . . . . .

. . . . . . .
Vv17g0631 Tc03_g009790 . . . . .
. . . . . . . .
. . . . . .
Vv17g0632 . . . . . . .
. . . . . . . .
. . . . .
Vv17g0633 . . . . . . .
. . . . . . . .
. . . . .
Vv17g0634 Tc03_g009820 . . . Gorai.
008G191200.1 . Vv14g1562 . . . .
. . Vv01g0961 Tc02_g033950 Gorai.
013G074800.1 . . Gorai.008G191200.1 Gorai.
012G117800.1
Vv17g0635 . . . . . . .
. . . . . . . .
. . . . .
Vv17g0636 . . . . Gorai.008G182300.1
. Vv14g1564 Tc05_g023640 . . .
. . Vv01g0964 Tc02_g033920 . .
. Gorai.008G191300.1 .
Vv17g0637 . . . . . . .
. . . . . . . .
. . . . .
Vv17g0638 Tc03_g009840 . . . . .
Vv14g1565 Tc04_g017560 . . . .
. Vv01g0966 Tc02_g033900 Gorai.013G082700.1
. Gorai.009G323400.1 . .
Vv17g0639 . Gorai.001G072400.1 . . .
. Vv14g1566 . . . Gorai.
007G035800.1 . . . . . . .
. .
Vv17g0640 . . . . . . .
. . . . . . . .
. . . . .
Vv17g0641 . . . . . . .
. . . . . . . .
. . . . .
Vv17g0642 . . . . Gorai.008G191700.1
. . . . . . . .
. . . . . . .
Vv17g0643 . . . . . .
Vv14g1580 . . . . Gorai.006G023000.1
Gorai.012G055600.1 . . . . .
. .
Vv17g0644 . . . . . . .
. . . . . . . .
. . . . .
Vv17g0645 . . . . Gorai.008G182100.1

. . . . . . . .
Vv1rg0199 . . . . . .
Vv17g0646 . . . . . . .
. . . . . . . .
. . . . .
Vv17g0647 Tc03_g009870 . . . Gorai.
008G181800.1 . Vv14g1594 . . Gorai.
001G256200.1 Gorai.007G036200.1 Gorai.006G021800.1 .
Vv1rg0186 . . . . . .
Vv17g0648 Tc03_g009920 . . . . .
. . . . . . .
Vv1rg0183 . . . . . .
Vv17g0649 Tc03_g010030 . . . Gorai.
008G191900.1 . . . . . . .
. . . . . . . .
Vv17g0650 Tc03_g010060 . . Gorai.007G036300.1
Gorai.008G192000.1 . . . . .
. . . . . . . .
. .
Vv17g0651 Tc03_g010150 . . . . .
Vv14g1599 . . Gorai.001G238700.1
Gorai.007G036500.1 Gorai.006G020700.1 Gorai.012G055000.1
Vv1rg0180 . . . . . .
Vv17g0652 Tc03_g010200 . . Gorai.007G036600.1
. . . . . . . .
. . . . . . . .
Vv17g0653 Tc03_g010210 . . . Gorai.
008G192100.1 . . . . . . .
. Vv1rg0172 . . . . .
.
Vv17g0654 Tc03_g010220 . . . . .
Vv14g1601 . . . Gorai.007G036700.1
. . . . . . . .
.
Vv17g0655 Tc03_g010230 . . . Gorai.
008G192300.1 . . . . . . .
. . . . . . . .
Vv17g0656 Tc03_g010240 Gorai.001G072200.1 .
Gorai.007G036800.1 Gorai.008G181600.1 . . .
. . . . . Vv1rg0171 .
. . . . .
Vv17g0657 . . . . . . .
. . . . . . . .
. . . . .
Vv17g0658 Tc03_g010320 Gorai.001G071900.1 . .
Gorai.008G192500.1 . . . . .
. . . Vv1rg0157 . . .
. . .
Vv17g0659 Tc03_g010330 Gorai.001G071800.1 . .
. . . . . . . .

. Vv1rg0156 . . . . .
.
Vv17g0660 . . . . . . .
. . . . . . . .
. . . . .
Vv17g0661 Tc03_g010340 . . . . .
. . . . . . . .
. . . . . .
Vv17g0662 . . . . . . .
. . . . . . . .
. . . . .
Vv17g0663 . . . . . . .
. . . . . . . .
. . . . .
Vv17g0664 . . . . . . .
. . . . . . . .
. . . . .
Vv17g0665 Tc03_g010380 . . . . .
. . . . . . . .
. . . . . .
Vv17g0666 . Gorai.001G069500.1 . . .
. . . . . . . .
. . . . . . .
Vv17g0667 Tc03_g010390 . . . . .
. . . . . . . .
. . . . . .
Vv17g0668 . . . . . . .
. . . . . . . .
. . . . .
Vv17g0669 Tc03_g010400 . . . . .
. . . . . . . .
. . . . . .
Vv17g0670 . . . . . Gorai.
006G178700.1 . . . . . . .
. . . . . . .
Vv17g0671 . . . . . . .
. . . . . . . .
. . . . .
Vv17g0672 Tc03_g010420 . . . .
Gorai.006G178600.1 . . . . . .
. . . . . . . .
Vv17g0673 Tc03_g010430 . . . .
Gorai.006G178400.1 . . . . . .
. . . . . . . .
Vv17g0674 . . . . . . .
. . . . . . . .
. . . . .
Vv17g0675 . . . . . . .
. . . . . . . .
. . . . .

Vv17g0676 . . . . . . .
. . . . . . . .
. . . . .
Vv17g0677 . . . . . . .
. . . . . . . .
. . . . .
Vv17g0678 Tc03_g010450 . . . . .
. . . . . . . .
. . . . . .
Vv17g0679 Tc03_g010460 . . . . .
. . . . . . . .
. . . . . .
Vv17g0680 . . . . . . .
. . . . . . . .
. . . . .
Vv17g0681 . . . . . .
Vv14g1631 . . Gorai.001G239100.1 Gorai.
007G037300.1 . . Vv1rg0138 . . .
. . .
Vv17g0682 . . . . . . .
. . . . . . . .
. . . . .
Vv17g0683 Tc03_g024750 . . . . .
. . . . . . . .
. . . . . .
Vv17g0684 . . . . Gorai.008G181000.1
. . . . . . . .
Vv1rg0135 . . . . . .
Vv17g0685 . . . . . . .
. . . . . . . .
. . . . .
Vv17g0686 Tc03_g024920 . . . Gorai.
008G193400.1 Gorai.006G266900.1 . . . .
. . . . . . . .
. .
Vv17g0687 . . . . . . .
. . . . . . . .
. . . . .
Vv17g0688 Tc03_g025210 . . . . .
Vv14g1640 . . . . . .
. . . . . . .
Vv17g0689 . . . . . . .
. . . . . . . .
. . . . .
Vv17g0690 . . . . . . .
. . . . . . . .
. . . . .
Vv17g0691 . . . . . . .
. . . . . . . .
. . . . .

Vv17g0692 . . . . . . .
. . . . . . .
. . . . .
Vv17g0693 . . . . . .
Vv14g1654 Tc05_g004730 . . Gorai.007G079300.1
Gorai.006G015500.1 . Vv1rg0123 . .
. . . .
Vv17g0694 Tc03_g010570 . . . Gorai.
008G193800.1 . . . . . . .
. . . . . . .
Vv17g0695 . . . . . . .
. . . . . . .
. . . . .
Vv17g0696 . . . . . . .
. . . . . . .
. . . . .
Vv17g0697 . . . . . . .
. . . . . . .
. . . . .
Vv17g0698 . . . . Gorai.008G194000.1
. . . . . . .
. . . . . .
Vv17g0699 . . . . Gorai.008G194200.1
. Vv14g1658 Tc04_g009150 . Gorai.
001G239700.1 . Gorai.006G015100.1 . Vv1rg0119
. . . . . .
Vv17g0700 . . . . . . .
. . . . . . .
. . . . .
Vv17g0701 . . . . Gorai.008G194300.1
. . . . . . .
. . . . . .
Vv17g0702 . . . . . . .
. . . . . . .
. . . . .
Vv17g0703 . . . . Gorai.008G180100.1
. . . . . . .
Vv1rg0110 . . . . . .
Vv17g0704 . . . . . . .
. . . . . . .
. . . . .
Vv17g0705 Tc03_g025530 . . Gorai.007G017500.1
. . . . . . .
. . . . . . .
Vv17g0706 Tc03_g025710 . . . . .
. . . . . . .
. . . . . .
Vv17g0707 . . . . . . .
. . . . . . .
. . . . .

Vv17g0708 . . . . . . .
. . . . . . . .
. . . . .
Vv17g0709 . . . . . . .
. . . . . . . .
. . . . .
Vv17g0710 . . . . Gorai.008G194500.1
. . . . . . . .
. . . . . . .
Vv17g0711 . . . . . . .
. . . . . . . .
. . . . .
Vv17g0712 . . . . . . .
. . . . . . . .
. . . . .
Vv17g0713 . . . . . . .
. . . . . . . .
. . . . .
Vv17g0714 . . . . . . .
. . . . . . . .
. . . . .
Vv17g0715 . . . . . . .
. . . . . . . .
. . . . .
Vv17g0716 . . . . . . .
. . . . . . . .
. . . . .
Vv17g0717 . . . . . . .
. . . . . . . .
. . . . .
Vv17g0718 . . . . . . .
. . . . . . . .
. . . . .
Vv17g0719 . . . . . . .
. . . . . . . .
. . . . .
Vv17g0720 . . . . . . .
. . . . . . . .
. . . . .
Vv17g0721 Tc03_g027750 . . . .
Gorai.006G177800.1 . . . . . .
. . . . . . . .
Vv17g0722 . . . . Gorai.008G179200.1
. Vv14g1680 Tc04_g009620 . Gorai.
001G240900.1 Gorai.007G038300.1 . Gorai.012G051800.1
. . . . . . .
Vv17g0723 . . . . Gorai.008G195000.1
. . . . . . . .
. . . . . . .
Vv17g0724 Tc03_g010620 Gorai.001G079100.1 . .

. . . . . . . .
. . . . . . . .
Vv17g0725 Tc03_g010630 . . . . .
. . . . . . . .
. . . . . .
Vv17g0726 . Gorai.001G079000.1 . .
Gorai.008G195500.1 . . . . . .
. . . . . . . .
.
Vv17g0727 . . . . . Gorai.
006G267200.1 . . . . . . .
. . . . . . .
Vv17g0728 . Gorai.001G078900.1 . .
Gorai.008G195600.1 . . . . . .
. . . . . . . .
.
Vv17g0729 . . . . . . .
. . . . . . . .
. . . . .
Vv17g0730 Tc03_g027590 . . . . .
. . . . . . . .
. . . . . .
Vv17g0731 . . . . Gorai.008G195700.1
. . . . . . . .
. . . . . . .
Vv17g0732 . Gorai.001G078800.1 . .
Gorai.008G178700.1 Gorai.006G267400.1 . . .
. . . . . . . .
. . .
Vv17g0733 . . . . . . .
. . . . . . . .
. . . . .
Vv17g0734 . . . . . . .
. . . . . . . .
. . . . .
Vv17g0735 . . . . . . .
. . . . . . . .
. . . . .
Vv17g0736 . . . . . . .
. . . . . . . .
. . . . .
Vv17g0737 . . . . . . .
. . . . . . . .
. . . . .
Vv17g0738 . . . . . . .
. . . . . . . .
. . . . .
Vv17g0739 . Gorai.001G078700.1 . . .
. . . . . . . .
. . . . . . .

Vv17g0740 Tc03_g011010 . . . . .
. . . . . . . .
. . . . . .
Vv17g0741 . . . . . . .
. . . . . . . .
. . . . .
Vv17g0742 . . . . . . .
. . . . . . . .
. . . . .
Vv17g0743 . . . . Gorai.008G196200.1
. . . . . . . .
. . . . . . .
Vv17g0744 . . . . . . .
. . . . . . . .
. . . . .
Vv17g0745 . . . . . . .
. . . . . . . .
. . . . .
Vv17g0746 . . . . . .
Vv14g1705 Tc04_g010070 . . . Gorai.
006G012200.1 Gorai.012G050300.1 . . . .
. . .
Vv17g0747 . . . . Gorai.008G178000.1
. . . . . . . .
. . . . . . .
Vv17g0748 . . . . . . .
. . . . . . . .
. . . . .
Vv17g0749 . Gorai.001G078200.1 . . .
. Vv14g1717 Tc04_g010180 . . .
. . . . . . . .
.
Vv17g0750 . . . . . . .
. . . . . . . .
. . . . .
Vv17g0751 . Gorai.001G078100.1 . .
Gorai.008G196700.1 . . . . . .
. . . . . . . .
.
Vv17g0752 . . . . . . .
. . . . . . . .
. . . . .
Vv17g0753 . . . . . Gorai.
006G267500.1 . . . . . . .
. . . . . . .
Vv17g0754 Tc03_g011510 . . . . .
. . . . . . . .
. . . . . .
Vv17g0755 . . . . . . .
. . . . . . . .

. . . . .
Vv17g0756 . . . . . . .
. . . . . . . .
. . . . .
Vv17g0757 . . . . . . .
. . . . . . . .
. . . . .
Vv17g0758 . . . . . . .
. . . . . . . .
. . . . .
Vv17g0759 . . . . . . .
. . . . . . . .
. . . . .
Vv17g0760 Tc03_g011590 . . . Gorai.
008G197100.1 Gorai.006G267600.1 . . . .
. . . . . . . .
. .
Vv17g0761 . . . . . . .
. . . . . . . .
. . . . .
Vv17g0762 . . . . . . .
. . . . . . . .
. . . . .
Vv17g0763 Tc03_g011600 . . . Gorai.
008G197200.1 . . . . . . .
. . . . . . . .
Vv17g0764 Tc03_g011610 Gorai.001G077900.1 . .
Gorai.008G177900.1 . Vv14g1720
Tc04_g010210 . . Gorai.007G081800.1 . .
. . . . . .
Vv17g0765 Tc03_g011640 Gorai.001G077800.1 . .
Gorai.008G197400.1 . . . . .
. . . . . . . .
. .
Vv17g0766 Tc03_g011650 . . . .
Gorai.006G267700.1 . . . . . .
. . . . . . . .
Vv17g0767 Tc03_g011700 . . . Gorai.
008G197500.1 Gorai.006G267800.1 . . . .
. . . . . . . .
. .
Vv17g0768 . . . . . . .
. . . . . . . .
. . . . .
Vv17g0769 . . . . . . .
. . . . . . . .
. . . . .
Vv17g0770 . . . . . . .
. . . . . . . .
. . . . .

Vv17g0771 . . . . . . .
. . . . . . . .
. . . . .
Vv17g0772 Tc03_g011720 . . . Gorai.
008G197700.1 . . . . . . .
. . . . . . . .
Vv17g0773 Tc03_g011770 . . . Gorai.
008G197800.1 . . . . . . .
. . . . . . . .
Vv17g0774 . . . . . . .
. . . . . . . .
. . . . .
Vv17g0775 . . . . . . .
. . . . . . . .
. . . . .
Vv17g0776 . . . . . . .
. . . . . . . .
. . . . .
Vv17g0777 . . . . . . .
. . . . . . . .
. . . . .
Vv17g0778 . . . . . . .
. . . . . . . .
. . . . .
Vv17g0779 . . . . . . .
. . . . . . . .
. . . . .
Vv17g0780 . . . . . . .
. . . . . . . .
. . . . .
Vv17g0781 Tc03_g000570 . . . . .
. . . . . . . .
. . . . . .
Vv17g0782 . . . . . . .
. . . . . . . .
. . . . .
Vv17g0783 . . . . . . .
. . . . . . . .
. . . . .
Vv17g0784 . . . . . . .
. . . . . . . .
. . . . .
Vv17g0785 . . . . . . .
. . . . . . . .
. . . . .
Vv17g0786 . . . . . . .
. . . . . . . .
. . . . .
Vv17g0787 . . . . . . .
. . . . . . . .

. . . . .
Vv17g0788 Tc03_g011830 . . . Gorai.
008G197900.1 . . . . . . .
. . . . . . . .
Vv17g0789 Tc03_g011840 . . Gorai.007G039200.1
. . . . . . . .
. . . . . . . .
Vv17g0790 Tc03_g011850 . . . . .
. . . . . . . .
. . . . . .
Vv17g0791 . . . . . . .
. . . . . . . .
. . . . .
Vv17g0792 . . . . . . .
. . . . . . . .
. . . . .
Vv17g0793 Tc03_g011860 . . . .
Gorai.006G268000.1 . . . . . .
. . . . . . . .
Vv17g0794 . . . . Gorai.008G198000.1
. . . . . . . .
. . . . . . .
Vv17g0795 . . . . Gorai.008G198100.1
. . . . . . . .
. . . . . . .
Vv17g0796 . . . . . . .
. . . . . . . .
. . . . .
Vv17g0797 . . . . . . .
. . . . . . . .
. . . . .
Vv17g0798 . Gorai.001G077600.1 . . .
. Vv11g0846 . . . Gorai.
004G272800.1 . . . . . . .
. .
Vv17g0799 . . . . . . .
. . . . . . . .
. . . . .
Vv17g0800 Tc03_g011930 . . . Gorai.
008G198200.1 . . . . . . .
. . . . . . . .
Vv17g0801 . . . . . . .
. . . . . . . .
. . . . .
Vv17g0802 . . . . . . .
. . . . . . . .
. . . . .
Vv17g0803 . . . . . . .
. . . . . . . .
. . . . .

Vv17g0804 Tc03_g011980 . . . Gorai.
008G198400.1 . Vv11g0853 Tc09_g021760 . .
. . . . . . . .
. .

Vv17g0805 . . . . . . .
. . . . . . . .
. . . . .

Vv17g0806 . . . . Gorai.008G177700.1
. . . . . . . .
. . . . . . .

Vv17g0807 . . . . . . .
. . . . . . . .
. . . . .

Vv17g0808 . . . . . . .
. . . . . . . .
. . . . .

Vv17g0809 . . . . . . .
. . . . . . . .
. . . . .

Vv17g0810 . . . . . . .
. . . . . . . .
. . . . .

Vv17g0811 Tc03_g012060 . . . Gorai.
008G175000.1 . . . . . . .
. . . . . . . .

Vv17g0812 Tc03_g012080 . . . Gorai.
008G198900.1 . . . . . . .
. . . . . . . .

Vv17g0813 Tc03_g012100 Gorai.001G077400.1 . .
Gorai.008G199000.1 . . . . .
. . . . . . . .
. .

Vv17g0814 Tc03_g012130 Gorai.001G077300.1 . .
. . . . . . . .
. . . . . . . .

Vv17g0815 Tc03_g012140 Gorai.001G077200.1 . .
Gorai.008G199200.1 . . . . .
. . . . . . . .
. .

Vv17g0816 Tc03_g012150 . . Gorai.007G039400.1
. . . . . . . .
. . . . . . . .

Vv17g0817 . Gorai.001G077100.1 . . .
. . . . . . . .
. . . . . . .

Vv17g0818 Tc03_g012160 . . . Gorai.
008G199300.1 Gorai.006G268100.1 . . . .
. . . . . . . .
. .

Vv17g0819 . . . . . . .

. . . . . . . .
. . . . .
Vv17g0820 Tc03_g000280 . . . . .
Vv14g1467 . . . . . .
. . . . . . .
Vv17g0821 . . . . . .
Vv14g1470 . . Gorai.001G259500.1 . .
. . . . . . . .
Vv17g0822 . . . . . . .
. . . . . . . .
. . . . .
Vv17g0823 . . . . . .
Vv14g1472 . . . . . . .
. . . . . .
Vv17g0824 . . . . . . .
. . . . . . . .
. . . . .
Vv17g0825 . . . . . .
Vv14g1471 Tc04_g000350 . . . . .
. . . . . . .
Vv17g0826 . . . . . . .
. . . . . . . .
. . . . .
Vv17g0827 . . . . . . .
. . . . . . . .
. . . . .
Vv17g0828 . . . . . .
Vv14g1470 . . Gorai.001G259500.1 . .
. . . . . . . .
Vv17g0829 . . . . . . .
. . . . . . . .
. . . . .
Vv17g0830 Tc03_g012200 . . . . .
. . . . . . . .
. . . . . .
Vv17g0831 Tc03_g012210 Gorai.001G076900.1 . .
. . . . . . . .
. . . . . . . .
Vv17g0832 Tc03_g000240 . . . . .
. . . . . . .
Vv01g0885 . . Gorai.005G188000.1 Gorai.
009G345700.1 . .
Vv17g0833 Tc03_g000230 . . . . .
. . . . . . . .
. . . . . .
Vv17g0834 Tc03_g000220 . . . . .
. . . . . . . .
. . . . . .
Vv17g0835 . . . . . . .
. . . . . . . .

. . . . .
Vv17g0836 . . . . . . .
. . . . . . . .
. . . . .
Vv17g0837 . . . . . . .
. . . . . . . .
. . . . .
Vv17g0838 . Gorai.001G073900.1 . . .
. . . . . . . .
. . . . . . .
Vv17g0839 . . . . . .
. . . . . . Vv01g0883
. . Gorai.005G187800.1 . . .
Vv17g0840 Tc03_g000270 . . . .
Gorai.006G187300.1 . . . . . .
. . . . . . . .
Vv17g0841 Tc03_g000280 . . . . .
Vv14g1467 . . . . . .
. . . . . . .
Vv17g0842 Tc03_g000300 . . . . .
. . . . . . .
Vv01g0876 . . Gorai.005G187300.1 . .
.
Vv17g0843 Tc03_g000310 . . . .
Gorai.006G187400.1 . . . . . .
. Vv01g0875 . . . . .
.
Vv17g0844 . . . . . . .
. . . . . . . .
. . . . .
Vv17g0845 Tc03_g000320 . . . . .
. . . . . . . .
. . . . . .
Vv17g0846 Tc03_g000330 . . . . .
. . . . . . . .
. . . . . .
Vv17g0847 Tc03_g000350 . . . . .
. . . . . . . .
. . . . . .
Vv17g0848 . . . . . . .
. . . . . . . .
. . . . .
Vv17g0849 . . . . . . .
. . . . . . . .
. . . . .
Vv17g0850 Tc03_g000360 . . . . .
. . . . . . . .
. . . . . .
Vv17g0851 Tc03_g000370 Gorai.001G071200.1 . .
. . . . . . . .

```
        .        Vv01g0872        .        .       .        .       .
        .
Vv17g0852        Tc03_g000430     .        .       .        .       .
        .        .        .       .        .       .        .       .
        .        .        .       .        .       .
Vv17g0853        .        .       .        .       .        .       .
        .        .        .       .        .       .        .       .
        .        .        .       .        .
Vv17g0854        Tc03_g000470     .        .       .        .       .
        .        .        .       .        .       .        .       .
        .        .        .       .        .       .
Vv17g0855        Tc03_g000480     .        .       .        .       .
        .        .        .       .        .       .        .       .
        .        .        .       .        .       .
Vv17g0856        .        .       .        .       .        .       .
        .        .        .       .        .       .        .       .
        .        .        .       .        .
Vv17g0857        .        .       .        .       Gorai.008G158300.1
        .        .        .       .        .       .        .       .
        Vv01g0868         .       .        .       .        .
Gorai.012G123100.1
Vv17g0858        Tc03_g000500     .        .       .        .       .
        .        .        .       .        .       .        .       .
        .        .        .       .        .       .
Vv17g0859        Tc03_g000510     .        .       .        .       .
        .        .        .       .        .       .        .       .
        .        .        .       .        .       .
Vv17g0860        Tc03_g000520     .        .       .        .       .
        .        .        .       .        .       .        .       .
        .        .        .       .        .       .
Vv17g0861        Tc03_g000540     .        .       .        .
Gorai.006G187900.1        .       .        .       .        .       .
        .        .        .       .        .       .        .       .
Vv17g0862        .        .       .        .       .        Gorai.
006G187800.1     Vv14g1465        .        .       Gorai.001G259700.1
        .        .        .       Vv01g0842        .        .       .
        .        .        .
Vv17g0863        Tc03_g000550     .        .       .        Gorai.
008G201000.1     .        .       .        .       .        .       .
        .        .        .       .        .       .        .       .
Vv17g0864        Tc03_g000570     .        .       .        .       .
        .        .        .       .        .       .        .       .
        .        .        .       .        .       .
Vv17g0865        Tc03_g000580     .        .       .        .       .
        .        .        .       .        .       .        .       .
        .        .        .       .        .       .
Vv17g0866        Tc03_g000590     .        .       .        .       .
        .        .        .       .        .       .        .       .
        .        .        .       .        .       .
Vv17g0867        Tc03_g000600     .        .       .        .       .
```

. . . . . . . .
. . . . . .
Vv17g0868 . . . . . . .
. . . . . . . .
. . . . .
Vv17g0869 . . . . . . .
. . . . . . . .
. . . . .
Vv17g0870 Tc03_g000610 . . . . .
. . . . . . . .
. . . . . .
Vv17g0871 Tc03_g000620 . . . . .
. . . . . . . .
. . . . . .
Vv17g0872 Tc03_g000640 . . . . .
. . . . . . . .
. . . . . .
Vv17g0873 Tc03_g000650 . . . .
Gorai.006G187700.1 Vv14g1455 . . . .
. . Vv01g0824 . . . .
. .
Vv17g0874 Tc03_g000670 . . . . .
. . . . . . . .
. . . . . .
Vv17g0875 . . . . . . .
. . . . . . . .
. . . . .
Vv17g0876 Tc03_g000680 Gorai.001G070400.1 . .
. . Vv14g1448 Tc04_g000500 . .
Gorai.007G077100.1 . . . . .
. . . .
Vv17g0877 Tc03_g000690 . . . . .
Vv14g1447 Tc04_g000520 . . . .
Gorai.012G059900.1 Vv01g0821 Tc02_g023300 .
Gorai.005G184300.1 . . .
Vv17g0878 . . . . . . .
. . . . . . . .
. . . . .
Vv17g0879 Tc03_g000710 . . . .
Gorai.006G188000.1 Vv14g1446 Tc04_g000550 . .
. . Gorai.012G060000.1 . . .
. . . .
Vv17g0880 . . . . . . .
. . . . . . . .
. . . . .
Vv17g0881 . . . . . . .
. . . . . . . .
. . . . .
Vv17g0882 Tc03_g000730 . . . . .
. . . . . . .

Vv01g0808 . . . . . .
Vv17g0883 . . . . . . .
. . . . . . . .
. . . . .
Vv17g0884 . . . . . . .
. . . . . . . .
. . . . .
Vv17g0885 Tc03_g000760 Gorai.001G070300.1 . .
. Gorai.006G188200.1 . . . .
. . . . . . . .
. .
Vv17g0886 Tc03_g000770 Gorai.001G070200.1 . .
. Gorai.006G188100.1 . . . .
. . . . . . . .
. .
Vv17g0887 Tc03_g000780 . . . . .
. . . . . . . .
. . . . . .
Vv17g0888 Tc03_g000790 . . . . .
. . . . . . . .
. . . . . .
Vv17g0889 Tc03_g000800 . . . . .
Vv14g1441 Tc04_g000630 . . Gorai.
007G076900.1 . . . . . . .
. .
Vv17g0890 . . . . . . .
. . . . . . . .
. . . . .
Vv17g0891 Tc03_g000820 . . . . .
. . . . . . .
Vv01g0807 . . Gorai.005G183800.1 . .
.
Vv17g0892 Tc03_g000830 . . . . .
. . . . . . . .
. . . . . .
Vv17g0893 Tc03_g000880 Gorai.001G068600.1 . .
. . . . . . . .
. Vv01g0802 . . Gorai.005G183500.1
. . .
Vv17g0894 Tc03_g000890 . . Gorai.007G002000.1
. . . . . . . .
. . . . . . . .
Vv17g0895 . . . . . Gorai.
006G187100.1 Vv14g1436 Tc04_g000700 . . .
. Gorai.012G060800.1 . . . .
. . .
Vv17g0896 Tc03_g000920 . . . Gorai.
008G158500.1 . Vv14g1435 Tc04_g000710 .
Gorai.001G261100.1 . . . Vv01g0800 .
. . . Gorai.008G155700.1 .

Vv17g0897 Tc03_g000930 . . . . .
. . . . . . . .
. . . . . .
Vv17g0898 . . . . . .
Vv14g1433 . . . . . . .
. . . . . .
Vv17g0899 . . . . . Gorai.
006G185400.1 . . . . . . .
. . . . . . .
Vv17g0900 . . . . . . .
. . . . . . . .
. . . . .
Vv17g0901 Tc03_g000940 . . . .
Gorai.006G182700.1 . . . . . .
. . . . . . . .
Vv17g0902 . Gorai.001G068400.1 . . .
. . . . . . . .
. . . . . . .
Vv17g0903 . . . . . . .
. . . . . . . .
. . . . .
Vv17g0904 Tc03_g000950 Gorai.001G068400.1 . .
. . Vv14g1421 Tc04_g000790 . .
. . Gorai.012G061200.1 . . .
. . . .
Vv17g0905 Tc03_g000960 . . . . .
. . . . . . . .
. . . . . .
Vv17g0906 Tc03_g000990 . . . . .
. . . . . . . .
. . . . . .
Vv17g0907 . . . . . . .
. . . . . . . .
. . . . .
Vv17g0908 Tc03_g001010 . . . . .
. . . . . . .
Vv1rg0006 . . . . . .
Vv17g0909 . . . . . . .
. . . . . . . .
. . . . .
Vv17g0910 . . . . . . .
. . . . . . . .
. . . . .
Vv17g0911 . . . . . . .
. . . . . . . .
. . . . .
Vv17g0912 . . . . . . .
. . . . . . . .
. . . . .
Vv17g0913 Tc03_g001020 . . Gorai.007G001900.1

. . . . . . . .
. . . . . . . .
Vv17g0914 Tc03_g001030 . . . . .
. . . . . . . .
. . . . . .
Vv17g0915 Tc03_g001040 . . . . .
. . . . . . . .
. . . . . .
Vv17g0916 Tc03_g001050 . . . . .
Vv14g1410 Tc04_g000880 . Gorai.001G261500.1
. . . . . . . .
. .
Vv17g0917 . . . . . . .
. . . . . . . .
. . . . .
Vv17g0918 Tc03_g001060 . . . . .
. . . . . . . .
. . . . . .
Vv17g0919 . . . . . . .
. . . . . . . .
. . . . .
Vv17g0920 . . . . . . .
. . . . . . . .
. . . . .
Vv17g0921 . . . . . . .
. . . . . . . .
. . . . .
Vv17g0922 Tc03_g001070 . . . . .
Vv14g1407 Tc04_g000920 . . .
Gorai.006G030200.1 Gorai.012G061500.1 . . .
. . . .
Vv17g0923 Tc03_g001080 . . . . .
. . . . . . . .
. . . . . .
Vv17g0924 . . . . . . .
. . . . . . . .
. . . . .
Vv17g0925 . . . . . . .
. . . . . . . .
. . . . .
Vv17g0926 . . . . . . .
. . . . . . . .
. . . . .
Vv17g0927 . . . . . . .
. . . . . . . .
. . . . .
Vv17g0928 . . . . . . .
. . . . . . . .
. . . . .
Vv17g0929 . . . . . Gorai.

006G189100.1 . . . . . . .
. . . . . . .
Vv17g0930 Tc03_g029940 . . . Gorai.
008G158900.1 . . . . . . .
. . . . . . . .
Vv17g0931 . . . . . .
Vv14g1397 Tc04_g001000 Gorai.011G095800.1 Gorai.
001G262600.1 . . . . . . .
. . .
Vv17g0932 . . . . . Gorai.
006G189200.1 . . . . . . .
. . . . . . .
Vv17g0933 . . . . . . .
. . . . . . . .
. . . . .
Vv17g0934 Tc03_g030120 . . . . .
. . . . . . . .
. . . . . .
Vv17g0935 . . . . . . .
. . . . . . . .
. . . . .
Vv17g0936 . . . . . . .
. . . . . . . .
. . . . .
Vv17g0937 Tc03_g001110 . . . . .
. . . . . . .
Vv1rg0021 . . . . . .
Vv17g0938 . . . . . . .
. . . . . . . .
. . . . .
Vv17g0939 Tc03_g001120 . . . . .
. . . . . . .
Vv1rg0022 . . . . . .
Vv17g0940 Tc03_g001130 . . . . .
. . . . . . . .
. . . . . .
Vv17g0941 . . . . . . .
. . . . . . . .
. . . . .
Vv17g0942 . . . . . . .
. . . . . . . .
. . . . .
Vv17g0943 Tc03_g001290 . . . . .
Vv14g1388 Tc04_g001080 . Gorai.001G263100.1
. . Gorai.012G062600.1 . . .
. . . .
Vv17g0944 . . . . . . .
. . . . . . . .
. . . . .
Vv17g0945 . . . . . . .

. . . . . . . .
. . . . .
Vv17g0946 Tc03_g001300 . . Gorai.007G001600.1
. . . . . . . .
. . . . . . . .
Vv17g0947 . . . . Gorai.008G159500.1
. . . . . . . .
Vv1rg0044 . . . . . .
Vv17g0948 . . . . . . .
. . . . . . . .
. . . . .
Vv17g0949 . . . . . . .
. . . . . . . .
. . . . .
Vv17g0950 Tc03_g001330 . . Gorai.007G001500.1
. . . . . . . .
. . . . . . . .
Vv17g0951 Tc03_g001340 . . . . .
. . . . . . . .
. . . . . .
Vv17g0952 Tc03_g001350 . . . . .
. . . . . . . .
. . . . . .
Vv17g0953 . Gorai.001G071400.1 . . .
. Vv14g1375 . . . . .
. . . . . . . .
Vv17g0954 . . . . . . .
. . . . . . . .
. . . . .
Vv17g0955 Tc03_g001360 . . . . .
Vv14g1374 Tc04_g001310 . . . .
Gorai.012G063100.1 . . . . .
. .
Vv17g0956 . . . . . . .
. . . . . . . .
. . . . .
Vv17g0957 Tc03_g001370 . . . Gorai.
008G159600.1 . . . . . . .
. Vv1rg0062 . . . . .
.
Vv17g0958 . . . . . . .
. . . . . . . .
. . . . .
Vv17g0959 Tc03_g001380 . . . . .
. . . . . . . .
. . . . . .
Vv17g0960 Tc03_g001390 . . . . .
Vv14g1372 Tc04_g001340 . . . .
. Vv1rg0067 . . . . .
.

```
Vv17g0961          .        .        .        .        .        .        .
         .         .        .        .        .        .       Vv1rg0068
         .         .        .        .        .        .
Vv17g0962          Tc03_g001400      .        .        .        .        .
         .         .        .        .        .        .        .        .
         .         .        .        .        .        .
Vv17g0963          Tc03_g001410      .        .        .        .        .
         .         .        .        .        .        .        .        .
         .         .        .        .        .        .
Vv17g0964          Tc03_g001420      .        .        .        .        .
         .         .        .        .        .        .        .        .
         .         .        .        .        .        .
Vv17g0965          Tc03_g001430      Gorai.001G071500.1         .        .
         .         .        .        .        .        .        .        .
         .         .        .        .        .        .        .        .
Vv17g0966          Tc03_g001440      .        .        .        .        .
         Vv14g1371          .        .        .        .        .        .
         .         .        .        .        .        .        .
Vv17g0967          Tc03_g001450      .        .        .        .        .
         Vv14g1370          Tc04_g001350      .        .        .
Gorai.006G031800.1          .        .        .        .        .        .
         .         .
Vv17g0968          Tc03_g001470      .        .        .        .        .
         .         .        .        .        .        .        .        .
         .         .        .        .        .        .
Vv17g0969          Tc03_g001480      .        .        .        .        .
         .         .        .        .        .        .        .        .
         .         .        .        .        .        .
Vv17g0970          .        .        .        .        .        .        .
         .         .        .        .        .        .        .        .
         .         .        .        .        .
Vv17g0971          Tc03_g001490      .        .        .        .        .
         .         .        .        .        .        .        .
Vv1rg0073          .        .        .        .        .        .
Vv17g0972          .        .        .        .        .        .        .
         .         .        .        .        .        .        .        .
         .         .        .        .        .
Vv17g0973          Tc03_g001500      .        .        Gorai.007G001400.1
         .         .        .        .        .        .        .        .
         .         .        .        .        .        .        .        .
Vv17g0974          .        .        .        .        .        .        .
         .         .        .        .        .        .        .        .
         .         .        .        .        .
Vv17g0975          Tc03_g001510      .        .        .        .        .
         .         .        .        .        .        .        .        .
         .         .        .        .        .        .
Vv17g0976          .        .        .        .        .        .        .
         .         .        .        .        .        .        .        .
         .         .        .        .        .
Vv17g0977          .        .        .        .        .        .        .
```

. . . . . . . .

. . . . .

Vv17g0978 Tc03_g001550 . . . . .

Vv14g1361 Tc04_g001420 . . . .

. . . . . . . .

Vv17g0979 Tc03_g001650 . . . . .

. . . . . . . .

. . . . . .

Vv17g0980 . . . . . . .

. . . . . . . .

. . . . .

Vv17g0981 Tc03_g001660 . . . .

Gorai.006G190000.1 . . . . . .

. . . . . . . .

Vv17g0982 Tc03_g001680 . . . . .

. . . . . . . .

. . . . . .

Vv17g0983 . . . . . . .

. . . . . . . .

. . . . .

Vv17g0984 Tc03_g001690 . . . Gorai.

008G219700.1 . . . . . . .

. . . . . . . .

Vv17g0985 Tc03_g001700 . . . . .

. . . . . . .

Vv1rg0088 . . . . . .

Vv17g0986 . . . . . . .

. . . . . . . .

. . . . .

Vv17g0987 . . . . . . .

. . . . . . . .

. . . . .

Vv17g0988 . . . . . . .

. . . . . . . .

. . . . .

Vv17g0989 Tc03_g001710 . . . . .

. . . . . . . .

. . . . . .

Vv17g0990 Tc03_g001720 . . . . .

. . . . . . . .

. . . . . .

Vv17g0991 Tc03_g001730 . . Gorai.007G001100.1

. . . . . . . .

. Vv1rg0091 . . . . .

.

Vv17g0992 Tc03_g001740 . . . . .

. . . . . . . .

. . . . . .

Vv17g0993 Tc03_g001750 . . . . .

. . . . . . . .

. . . . . .
Vv17g0994 Tc03_g009100 . . . . .
. . . . . . . .
. . . . . .
Vv17g0995 . . . . . . .
. . . . . . . .
. . . . .
Vv17g0996 . . . . . . .
. . . . . . . .
. . . . .
Vv17g0997 . Gorai.001G072300.1 . . .
. . . . . . . .
. . . . . . .
Vv17g0998 . . . . . . .
. . . . . . . .
. . . . .
Vv17g0999 . . . . . . .
. . . . . . . .
. . . . .
Vv17g1000 . . . . . . .
. . . . . . . .
. . . . .
Vv17g1001 . . . . . . .
. . . . . . . .
. . . . .
Vv17g1002 . . . . . . .
. . . . . . . .
. . . . .
Vv17g1003 . . . . . .
Vv14g1354 Tc04_g001490 . . . .
Gorai.012G063500.1 . . . . . .
.
Vv17g1004 . . . . . . .
. . . . . . . .
. . . . .
Vv17g1005 Tc03_g008690 . . . . .
. . . . . . . .
. . . . . .
Vv17g1006 . . . . . . .
. . . . . . . .
. . . . .
Vv17g1007 Tc03_g001980 . . . . .
Vv14g1344 Tc04_g001570 . . Gorai.
007G075700.1 Gorai.006G033100.1 Gorai.012G063800.1 .
. . . . . .
Vv17g1008 . . . . . . .
. . . . . . . .
. . . . .
Vv17g1009 . . . . . . .
. . . . . . . .

. . . . .
Vv17g1010 . . . . . . .
. . . . . . . .
. . . . .
Vv17g1011 . . . . . . .
. . . . . . . .
. . . . .
Vv17g1012 . . . . . . .
. . . . . . . .
. . . . .
Vv17g1013 Tc03_g008530 . . . . .
. . . . . . . .
. . . . . .
Vv17g1014 . . . . . . .
. . . . . . . .
. . . . .
Vv17g1015 . . . . . . .
. . . . . . . .
. . . . .
Vv17g1016 . . . . . . .
. . . . . . . .
. . . . .
Vv17g1017 . . . . . .
Vv14g1333 . . . . . . .
. . . . . .
Vv17g1018 . . . . . . .
. . . . . . . .
. . . . .
Vv17g1019 . . . . . . .
. . . . . . . .
. . . . .
Vv17g1020 . . . . . . .
. . . . . . . .
. . . . .
Vv17g1021 Tc03_g002130 . . . Gorai.
008G199100.1 . . . . . . .
. . . . . . . .
Vv17g1022 . . . . . . .
. . . . . . . .
. . . . .
Vv17g1023 . . . . . . .
. . . . . . . .
. . . . .
Vv17g1024 Tc03_g002140 . . . . .
. . . . . . . .
. . . . . .
Vv17g1025 Tc03_g002150 . . . . .
. . . . . . . .
. . . . . .
Vv17g1026 . . . . . . .

. . . . . . . .
. . . . .
Vv17g1027 Tc03_g002160 . . . Gorai.
008G217300.1 . . . . . . .
. . . . . . . .
Vv17g1028 . . . . . . .
. . . . . . . .
. . . . .
Vv17g1029 Tc03_g002170 . . . Gorai.
008G217200.1 . . . . . . .
. . . . . . . .
Vv17g1030 . . . . . . .
. . . . . . . .
. . . . .
Vv17g1031 Tc03_g002180 . . . Gorai.
008G217100.1 . . . . . . .
. . . . . . . .
Vv17g1032 . . . . . . .
. . . . . . . .
. . . . .
Vv17g1033 . . . . . . .
. . . . . . . .
. . . . .
Vv17g1034 Tc03_g002190 . . . . .
. . . . . . . .
. . . . . .
Vv17g1035 . . . . . . .
. . . . . . . .
. . . . .
Vv17g1036 . . . . . . .
. . . . . . . .
. . . . .
Vv17g1037 Tc03_g002220 . . . . .
. . . . . . . .
. . . . . .
Vv17g1038 Tc03_g002230 . . . . .
. . . . . . . .
. . . . . .
Vv17g1039 . . . . . . .
. . . . . . . .
. . . . .
Vv17g1040 Tc03_g002260 . . . . .
. . . . . . . .
. . . . . .
Vv17g1041 Tc03_g002270 . . . . .
. . . . . . . .
. . . . . .
Vv17g1042 . . . . . . .
. . . . . . . .
. . . . .

Vv17g1043 Tc03_g002290 . . . . .
Vv14g1298 . . . . . .
. . . . . . .
Vv17g1044 . . . . . . .
. . . . . . .
. . . . .
Vv17g1045 Tc03_g013540 Gorai.001G064400.1 . .
. . . . . . .
. . . . . . .
Vv17g1046 Tc03_g013550 Gorai.001G064300.1 . .
Gorai.008G201800.1 . . . . .
. . . . . . .
. .
Vv17g1047 . . . . . . .
. . . . . . .
. . . . .
Vv17g1048 Tc03_g013560 Gorai.001G064200.1 . .
Gorai.008G201900.1 . . . . .
. . . Vv01g0770 Tc02_g025050 .
Gorai.005G177200.1 . . .
Vv17g1049 Tc03_g013570 . . . . .
. . . . . . .
. . . . . .
Vv17g1050 . . . . . . .
. . . . . . .
. . . . .
Vv17g1051 . . . . . . .
. . . . . . .
. . . . .
Vv17g1052 Tc03_g013580 Gorai.001G064100.1 . .
. . . . . . .
. . . . . . .
Vv17g1053 Tc03_g013590 . . . Gorai.
008G202100.1 . . . . . . .
. . . . . . .
Vv17g1054 . . . . . . .
. . . . . . .
. . . . .
Vv17g1055 Tc03_g013600 Gorai.001G064000.1 . .
. . . . . . .
. Vv01g0763 Tc02_g025140 . .
Gorai.009G335400.1 . .
Vv17g1056 Tc03_g013610 . . . . .
. . . . . . .
. . . . . .
Vv17g1057 . . . . . . .
. . . . . . .
. . . . .
Vv17g1058 Tc03_g013680 Gorai.001G063800.1 . .
. . . . . . .

. Vv01g0747 . . . . .
.
Vv17g1059 . . . . . . .
. . . . . . . .
. . . . .
Vv17g1060 . . . . . . .
. . . . . . . .
. . . . .
Vv17g1061 Tc03_g013690 Gorai.001G063600.1 . .
. . . . . . . .
. . . . . . . .
Vv17g1062 . . . . . . .
. . . . . . . .
. . . . .
Vv17g1063 . . . . . . .
. . . . . . . .
. . . . .
Vv17g1064 . . . . . . .
. . . . . . . .
. . . . .
Vv17g1065 . . . . . . .
. . . . . . . .
. . . . .
Vv17g1066 . . . . . . .
. . . . . . . .
. . . . .
Vv17g1067 Tc03_g013700 . . . Gorai.
008G202400.1 . . . . . . .
. . . . . . . .
Vv17g1068 . . . . . Gorai.
006G266700.1 . . . . . . .
. . . . . . .
Vv17g1069 . . . . . . .
. . . . . . . .
. . . . .
Vv17g1070 . . . . . . .
. . . . . . . .
. . . . .
Vv17g1071 Tc03_g013710 . . . Gorai.
008G202500.1 . . . . . . .
. . . . . . . .
Vv17g1072 Tc03_g013720 . . . Gorai.
008G202600.1 . . . . . . .
. . . . . . . .
Vv17g1073 . . . . . . .
. . . . . . . .
. . . . .
Vv17g1074 Tc03_g013740 . . . . .
. . . . . . . .
. . . . . .

Vv17g1075 . . . . . . .
. . . . . . . .
. . . . .
Vv17g1076 . . . . . . .
. . . . . . . .
. . . . .
Vv17g1077 Tc03_g013760 . . . Gorai.
008G202700.1 . . . . . . .
. Vv01g0737 Tc02_g025310 . Gorai.
005G175700.1 Gorai.009G335200.1 . .
Vv17g1078 Tc03_g013770 . . . Gorai.
008G202800.1 . . . . . . .
. . . . . . . .
Vv17g1079 Tc03_g013810 . . . Gorai.
008G203100.1 . . . . . . .
. . . . . . . .
Vv17g1080 Tc03_g013820 Gorai.001G063100.1 . .
. . . . . . . .
. . . . . . . .
Vv17g1081 Tc03_g013830 Gorai.001G063000.1 . .
Gorai.008G203200.1 . . . . .
. . . . . . . .
. .
Vv17g1082 . . . . . . .
. . . . . . . .
. . . . .
Vv17g1083 Tc03_g013840 . . Gorai.007G041500.1
. . Vv14g1276 . Gorai.011G093700.1
. . . . Vv01g0731
Tc02_g025400 . Gorai.005G175200.1 . . .
Vv17g1084 Tc03_g013850 . . . Gorai.
008G203300.1 . . . . . . .
. . . . . . . .
Vv17g1085 Tc03_g013860 . . . Gorai.
008G203400.1 Gorai.006G269700.1 . . . .
. . . . . . . .
. .
Vv17g1086 Tc03_g013890 . . . . .
. . . . . . . .
. . . . . .
Vv17g1087 Tc03_g013920 Gorai.001G062700.1 . .
. Gorai.006G269800.1 Vv14g1274
Tc04_g002220 . . . . Gorai.012G066500.1
. . . . . . .
Vv17g1088 . . . . Gorai.008G203700.1
. . . . . . . .
. . . . . . .
Vv17g1089 Tc03_g013940 . . . .
Gorai.006G269900.1 . . . . . .
. . . . . . . .

Vv17g1090 . . . . . . .
. . . . . . . .
. . . . .
Vv17g1091 . . . . . . .
. . . . . . . .
. . . . .
Vv17g1092 . . . . . . .
. . . . . . . .
. . . . .
Vv17g1093 Tc03_g013960 . . . Gorai.
008G203800.1 . . . . . . .
. Vv01g0726 Tc02_g025520 . . .
Gorai.008G161800.1 .
Vv17g1094 . Gorai.001G062600.1 . . .
. . . . . . . .
. . . . . . .
Vv17g1095 Tc03_g013990 Gorai.001G062300.1 . .
Gorai.008G204000.1 . . . . .
. . . . . . . .
. .
Vv17g1096 . . . . . . .
. . . . . . . .
. . . . .
Vv17g1097 . . . . . .
Vv14g1268 Tc04_g002320 . Gorai.001G267300.1 .
Gorai.006G036300.1 . Vv01g0714
Tc02_g025720 Gorai.002G189900.1 Gorai.005G174600.1 .
Gorai.008G162500.1 .
Vv17g1098 . . . . . . .
. . . . . . . .
. . . . .
Vv17g1099 Tc03_g014000 Gorai.001G062200.1 . .
. . . . . . . .
. . . . . . . .
Vv17g1100 . . . . . . .
. . . . . . . .
. . . . .
Vv17g1101 Tc03_g014010 . . . . .
. . . . . . . .
. . . . . .
Vv17g1102 Tc03_g014030 . . . . .
. . . . . . . .
. . . . . .
Vv17g1103 . . . . . . .
. . . . . . . .
. . . . .
Vv17g1104 Tc03_g014040 Gorai.001G062100.1 . .
. . . . . . . .
. . . . . . . .
Vv17g1105 Tc03_g014050 Gorai.001G062000.1 . .

. . . . . . . .
. . . . . . . .
Vv17g1106 . . . . . . .
. . . . . . . .
. . . . .
Vv17g1107 Tc03_g014060 . . . Gorai.
008G204100.1 . . . . . . .
. . . . . . . .
Vv17g1108 Tc03_g014070 . . . Gorai.
008G204200.1 . . . . . . .
. Vv01g0709 Tc02_g025770 . Gorai.
005G174300.1 . . Gorai.012G173900.1
Vv17g1109 Tc03_g014080 . . . .
Gorai.006G270000.1 . . . . . .
. . . . . . . .
Vv17g1110 . . . . . . .
. . . . . . . .
. . . . .
Vv17g1111 . . . . . . .
. . . . . . . .
. . . . .
Vv17g1112 . . . . . . .
. . . . . . . .
. . . . .
Vv17g1113 . . . . . . .
. . . . . . . .
. . . . .
Vv17g1114 Tc03_g014090 . . . Gorai.
008G204300.1 . . . . . . .
. . . . . . . .
Vv17g1115 . . . . . . .
. . . . . . . .
. . . . .
Vv17g1116 Tc03_g014100 Gorai.001G061900.1 . .
. Gorai.006G270100.1 . . . .
. . . . . . . .
. .
Vv17g1117 Tc03_g014110 Gorai.001G061800.1 .
Gorai.007G041600.1 Gorai.008G204400.1 . Vv14g1262
. . . . Gorai.006G036900.1 .
. . . . . . .
Vv17g1118 Tc03_g014120 . . . Gorai.
008G204500.1 Gorai.006G270200.1 Vv14g1261 . .
. . . . . . . .
. . .
Vv17g1119 . . . . . . .
. . . . . . . .
. . . . .
Vv17g1120 . . . . . . .
. . . . . . . .

. . . . .
Vv17g1121 . . . . . . .
. . . . . . . .
. . . . .
Vv17g1122 . . . . . . .
. . . . . . . .
. . . . .
Vv17g1123 . . . . . . .
. . . . . . . .
. . . . .
Vv17g1124 Tc03_g014130 . . . . .
. . . . . . . .
. . . . . .
Vv17g1125 . . . . . . .
. . . . . . . .
. . . . .
Vv17g1126 . . . . . . .
. . . . . . . .
. . . . .
Vv17g1127 . . . . . . .
. . . . . . . .
. . . . .
Vv17g1128 . . . . . . .
. . . . . . . .
. . . . .
Vv17g1129 . . . . . . .
. . . . . . . .
. . . . .
Vv17g1130 Tc03_g014140 . . . Gorai.
008G204600.1 . . . . . . .
. . . . . . . .
Vv17g1131 Tc03_g014150 . . . . .
. . . . . . . .
. . . . . .
Vv17g1132 . . . . . . .
. . . . . . . .
. . . . .
Vv17g1133 Tc03_g014160 Gorai.001G061700.1 . .
. . . . . . . .
. . . . . . . .
Vv17g1134 . . . . . . .
. . . . . . . .
. . . . .
Vv17g1135 . . . . . . .
. . . . . . . .
. . . . .
Vv17g1136 . . . . . . .
. . . . . . Vv01g0697
Tc02_g025980 Gorai.002G169300.1 . Gorai.
009G329400.1 . .

Vv17g1137 . . . . . . .
. . . . . . . .
. . . . .
Vv17g1138 . . . . . . .
. . . . . . . .
. . . . .
Vv17g1139 Tc03_g014180 . . . Gorai.
008G204700.1 . . . . . . .
. . . . . . . .
Vv17g1140 . . . . . . .
. . . . . . . .
. . . . .
Vv17g1141 Tc03_g014190 Gorai.001G061600.1 . .
. . Vv14g1250 . . . .
. Gorai.012G090700.1 . . . .
. . .
Vv17g1142 Tc03_g014200 . . . .
Gorai.006G270300.1 . . . . . .
. . . . . . . .
Vv17g1143 Tc03_g014210 Gorai.001G061500.1 .
Gorai.007G041800.1 . . . . . .
. . . . . . . .
. .
Vv17g1144 . . . . . . .
. . . . . . . .
. . . . .
Vv17g1145 . . . . . . .
. . . . . . . .
. . . . .
Vv17g1146 . . . . . . .
. . . . . . . .
. . . . .
Vv17g1147 Tc03_g014220 Gorai.001G061400.1 . .
. . . . . . . .
. . . . . . . .
Vv17g1148 Tc03_g014240 Gorai.001G059300.1 . .
. . . . . . . .
. . . . . . . .
Vv17g1149 Tc03_g014250 . . . Gorai.
008G204800.1 Gorai.006G270400.1 . . . .
. . . . . . . .
. .
Vv17g1150 Tc03_g014260 Gorai.001G056300.1 . .
. Gorai.006G270500.1 . . . .
. . . . . . . .
. .
Vv17g1151 . . . . . . .
. . . . . . . .
. . . . .
Vv17g1152 . . . . . . .

. . . . . . . .
. . . . .
Vv17g1153 Tc03_g014280 . . . .
Gorai.006G270700.1 . . . . . .
. . . . . . . .
Vv17g1154 . . . . . . .
. . . . . . . .
. . . . .
Vv17g1155 Tc03_g014290 . . . . .
. . . . . . . .
. . . . . .
Vv17g1156 . . . . . . .
. . . . . . . .
. . . . .
Vv17g1157 . . . . . . .
. . . . . . . .
. . . . .
Vv17g1158 . . . . . . .
. . . . . . . .
. . . . .
Vv17g1159 . . . . . . .
. . . . . . . .
. . . . .
Vv17g1160 . . . . . . .
. . . . . . . .
. . . . .
Vv17g1161 . . . . . . .
. . . . . . . .
. . . . .
Vv17g1162 . . . . . .
Vv14g1238 Tc04_g002690 . . . . .
. . . . . . .
Vv17g1163 . . . . . . .
. . . . . . . .
. . . . .
Vv17g1164 Tc03_g014400 . . Gorai.007G041900.1
. . Vv14g1237 Tc04_g002700 Gorai.
011G088800.1 Gorai.001G268500.1 Gorai.007G074000.1
Gorai.006G038900.1 Gorai.012G089500.1 . . .
. . . .
Vv17g1165 . . . . . . .
. . . . . . . .
. . . . .
Vv17g1166 . . . . . .
Vv01g0683 . Gorai.002G170400.1 . .
Gorai.008G291400.1 . . . . . .
. .
Vv17g1167 . . . . . . .
. . . . . . . .
. . . . .

Vv17g1168 . . . . . . .
. . . . . . . .
. . . . .
Vv18g0001 . . . . Gorai.009G291700.1
. . . . . . . .
. . . . . . .
Vv18g0002 . . . . . . .
. . . . . . . .
. . . . .
Vv18g0003 . . . . . . .
. . . . . . . .
. . . . .
Vv18g0004 . . . . . .
Vv03g0423 Tc02_g006510 Gorai.013G035100.1 . .
Gorai.008G030600.1 Gorai.012G154900.1 . .
. . . . .
Vv18g0005 . . . . . . .
. . . . . . . .
. . . . .
Vv18g0006 . . . . . . .
. . . . . . . .
. . . . .
Vv18g0007 . . . . . . .
. . . . . . . .
. . . . .
Vv18g0008 . . . . . . .
. . . . . . . .
. . . . .
Vv18g0009 . . . . . . .
. . . . . . . .
. . . . .
Vv18g0010 . . . . . . .
. . . . . . . .
. . . . .
Vv18g0011 . Gorai.002G111500.1 . . .
. . . . . . . .
. . . . . . .
Vv18g0012 . Gorai.002G111600.1 . . .
. . . . . . . .
. . . . . . .
Vv18g0013 Tc08_g009970 . . . . .
. . . . . . . .
. . . . . .
Vv18g0014 . . . . . . .
. . . . . . . .
. . . . .
Vv18g0015 . . . . . . .
. . . . . . . .
. . . . .
Vv18g0016 . Gorai.002G111700.1 . . .

. . . . . . . .
. . . . . . .
Vv18g0017 Tc08_g010590 . . . . .
. . . . . . . .
. . . . . .
Vv18g0018 . . . Gorai.009G185700.1 .
. . . . . . . .
. . . . . . .
Vv18g0019 Tc08_g009680 . . . . .
. . . . . . . .
. . . . . .
Vv18g0020 . Gorai.002G112000.1 . . .
. . . . . . . .
. . . . . . .
Vv18g0021 . Gorai.002G112100.1 . . .
. . . . . . . .
. . . . . . .
Vv18g0022 . . . . . . .
. . . . . . . .
. . . . .
Vv18g0023 . . . . . . .
. . . . . . . .
. . . . .
Vv18g0024 . . . . . . .
. . . . . . . .
. . . . .
Vv18g0025 . . . . . .
Vv03g0395 Tc02_g012710 . . . Gorai.
008G012900.1 . . . . . . .
.
Vv18g0026 . . . . . . .
. . . . . . . .
. . . . .
Vv18g0027 . Gorai.002G112200.1 . . .
. . . . . . . .
. . . . . . .
Vv18g0028 . . . . . . .
. . . . . . . .
. . . . .
Vv18g0029 . . . . . . .
. . . . . . . .
. . . . .
Vv18g0030 . . . . . . .
. . . . . . . .
. . . . .
Vv18g0031 . . . . . . .
. . . . . . . .
. . . . .
Vv18g0032 . . . . . . .
. . . . . . . .

. . . . .
Vv18g0033 . . . . . . .
. . . . . . . .
. . . . .
Vv18g0034 . . . . . . .
. . . . . . . .
. . . . .
Vv18g0035 . . . . . . .
. . . . . . . .
. . . . .
Vv18g0036 . . . . . . .
. . . . . . . .
. . . . .
Vv18g0037 . . . . . . .
. . . . . . . .
. . . . .
Vv18g0038 . . . . . . .
. . . . . . . .
. . . . .
Vv18g0039 . . . . . .
Vv03g0393 Tc02_g027460 Gorai.013G065300.1 . .
. Gorai.012G137600.1 . . . .
. . .
Vv18g0040 Tc08_g005540 . . . . .
. . . . . . . .
. . . . . .
Vv18g0041 . Gorai.002G112400.1 . . .
. . . . . . . .
. . . . . . .
Vv18g0042 . . . . . .
Vv03g0389 Tc02_g012650 . Gorai.005G215400.1 .
. Gorai.012G138000.1 . . . .
. . .
Vv18g0043 . . . . . . .
. . . . . . . .
. . . . .
Vv18g0044 . . . . . .
Vv03g0388 Tc02_g012640 Gorai.013G065200.1 Gorai.
005G215700.1 Gorai.002G014700.1 Gorai.008G013000.1
Gorai.012G138100.1 . . . . . .
.
Vv18g0045 . . . . Gorai.009G291200.1
. . . . . . . .
. . . . . . .
Vv18g0046 . . . . Gorai.009G291100.1
. . . . . . . .
. . . . . . .
Vv18g0047 . . . . . . .
. . . . . . . .
. . . . .

Vv18g0048 . . . . Gorai.009G290900.1
. . . . . . . .
. . . . . . .
Vv18g0049 . . . . Gorai.009G288000.1
. . . . . . . .
. . . . . . .
Vv18g0050 . . . . . . .
. . . . . . . .
. . . . .
Vv18g0051 . Gorai.002G112500.1 Gorai.005G117700.1
. . . . . . . .
. . . . . . . .
.
Vv18g0052 Tc08_g010960 . . Gorai.009G185900.1
. . . . . . . .
. . . . . . . .
Vv18g0053 . . . . . . .
. . . . . . . .
. . . . .
Vv18g0054 . Gorai.002G112700.1 . . .
. . . . . . . .
. . . . . . .
Vv18g0055 . Gorai.002G112800.1 . . .
. . . . . . . .
. . . . . . .
Vv18g0056 . . . . . . .
. . . . . . . .
. . . . .
Vv18g0057 . Gorai.002G113200.1 . . .
. . . . . . . .
. . . . . . .
Vv18g0058 . . . . . . .
. . . . . . . .
. . . . .
Vv18g0059 . . Gorai.005G115700.1 . .
. . . . . . . .
. . . . . . .
Vv18g0060 . . . . . .
Vv03g0376 Tc02_g012530 . . . Gorai.
008G013500.1 Gorai.012G138700.1 . . . .
. . .
Vv18g0061 . . . . . . .
. . . . . . . .
. . . . .
Vv18g0062 Tc08_g011030 . . . . .
. . . . . . . .
. . . . . .
Vv18g0063 Tc08_g005370 . . . . .
. . . . . . . .
. . . . . .

Vv18g0064 . . . . . . .
. . . . . . . .
. . . . .
Vv18g0065 . . . . . . .
. . . . . . . .
. . . . .
Vv18g0066 . . . . . . .
. . . . . . . .
. . . . .
Vv18g0067 Tc08_g005360 Gorai.002G113400.1 . .
. . . . . . . .
. . . . . . . .
Vv18g0068 Tc08_g005350 . . Gorai.009G186100.1
. . . . . . . .
. . . . . . . .
Vv18g0069 Tc08_g005340 Gorai.002G113500.1 . .
. . . . . . . .
. . . . . . . .
Vv18g0070 Tc08_g005330 . . Gorai.009G186200.1
. . . . . . . .
. . . . . . . .
Vv18g0071 Tc08_g005320 Gorai.002G113600.1 Gorai.
005G114900.1 . . . . . . .
. . . . . . . .
. .
Vv18g0072 Tc08_g005310 Gorai.002G113700.1 . .
. . . . . . . .
. . . . . . . .
Vv18g0073 . . . . . . .
. . . . . . . .
. . . . .
Vv18g0074 Tc08_g005290 . . Gorai.009G186300.1
. . . . . . . .
. . . . . . . .
Vv18g0075 Tc08_g005280 Gorai.002G113800.1 . .
. . . . . . . .
. . . . . . . .
Vv18g0076 Tc08_g005270 Gorai.002G113900.1 Gorai.
005G114700.1 . . . . . . .
. . . . . . . .
. .
Vv18g0077 Tc08_g005260 Gorai.002G114000.1 .
Gorai.009G186400.1 . . . . . .
. . . . . . . .
. .
Vv18g0078 Tc08_g005250 Gorai.002G114100.1 . .
. . . . . . . .
. . . . . . . .
Vv18g0079 Tc08_g005240 Gorai.002G114200.1 . .
. . . . . . . .

```
        .       Vv01g0579       Tc02_g028070     Gorai.002G174100.1
        Gorai.005G159100.1      .       .        .
Vv18g0080       Tc08_g005230    .       .        Gorai.009G186500.1
        .       .       Vv03g0369       Tc02_g012440     Gorai.
013G064100.1    .       .       Gorai.008G013900.1       .       .
        .       .       .       .       .       .
Vv18g0081       Tc08_g005220    Gorai.002G114300.1       Gorai.
005G114600.1    .       .       .       .       .       .       .
        .       .       .       .       .       .       .       .
        .       .
Vv18g0082       Tc08_g005210    Gorai.002G114400.1       .       .
        .       .       .       .       .       .       .       .
        .       .       .       .       .       .       .       .
Vv18g0083       Tc08_g005200    Gorai.002G114500.1       .       .
        .       .       .       .       .       .       .       .
        .       .       .       .       .       .       .       .
Vv18g0084       Tc08_g005190    Gorai.002G114600.1       .       .
        .       .       .       .       .       .       .       .
        .       Vv01g0551       Tc02_g028440     .       .       .
        .       .
Vv18g0085       .       .       .       .       .       .       .
        .       .       .       .       .       .       .       .
        .       .       .       .       .
Vv18g0086       Tc08_g005180    Gorai.002G114700.1       .       .
        .       .       .       .       .       .       .       .
        .       .       .       .       .       .       .       .
Vv18g0087       Tc08_g005170    Gorai.002G114800.1       .       .
        .       .       Vv03g0365       .       .       .       .
        .       .       .       .       .       .       .       .
        .
Vv18g0088       .       .       .       .       .       .       .
        .       .       .       .       .       .       .       .
        .       .       .       .       .
Vv18g0089       Tc08_g005160    .       .       Gorai.009G186600.1
        .       .       .       .       .       .       .       .
        .       .       .       .       .       .       .       .
Vv18g0090       Tc08_g005140    .       .       Gorai.009G186700.1
        .       .       .       .       .       .       .       .
        .       .       .       .       .       .       .       .
Vv18g0091       Tc08_g005130    .       .       .       .       .
        .       .       .       .       .       .       .
Vv01g0516       Tc02_g027160    .       .       .       Gorai.
008G289800.1    .
Vv18g0092       Tc08_g005120    .       .       .       .       .
        .       .       .       .       .       .       .       .
        .       .       .       .       .       .
Vv18g0093       .       Gorai.002G115000.1      .       .       .
        .       .       .       .       .       .       .       .
        .       .       .       .       .       .       .
Vv18g0094       Tc08_g005110    Gorai.002G115100.1       Gorai.
```

005G114500.1 Gorai.009G186800.1 . . . .
. . . . . . . .
. . . .
Vv18g0095 Tc08_g005100 . . Gorai.009G186900.1
. . . . . . . .
. . . . . . . .
Vv18g0096 Tc08_g005090 Gorai.002G115300.1 . .
. . Vv03g0362 Tc02_g012360 . .
. . Gorai.012G139400.1 . . .
. . . .
Vv18g0097 Tc08_g005080 . Gorai.005G114300.1
Gorai.009G187000.1 . . Vv03g0361 . .
. . Gorai.008G014100.1 . . .
. . . . .
Vv18g0098 Tc08_g005060 Gorai.002G115500.1 . .
. . . . . . . .
. . . . . . . .
Vv18g0099 Tc08_g005050 Gorai.002G115600.1 . .
. . . . . . . .
. . . . . . . .
Vv18g0100 . . . . . . .
. . . . . . . .
. . . . .
Vv18g0101 Tc08_g005030 . Gorai.005G114200.1 .
. . . . . . . .
. . . . . . . .
Vv18g0102 . . . . . . .
. . . . . . . .
. . . . .
Vv18g0103 Tc08_g005020 Gorai.002G115700.1 . .
. . Vv03g0353 . Gorai.002G206800.1
. . . . . . . .
. . .
Vv18g0104 Tc08_g005000 . . . . .
Vv03g0351 Tc02_g003500 Gorai.002G206700.1
Gorai.005G259100.1 . Gorai.008G041100.1 . .
. . . . . .
Vv18g0105 . Gorai.002G115800.1 . Gorai.
009G187500.1 . . . . . . .
. . . . . . . .
.
Vv18g0106 Tc08_g004990 Gorai.002G115900.1 .
Gorai.009G187600.1 . . . . . .
. . . . . . . .
. .
Vv18g0107 Tc08_g004960 . . Gorai.009G187700.1
. . . . . . . .
. . . . . . . .
Vv18g0108 Tc08_g004950 Gorai.002G116800.1 Gorai.
005G113900.1 Gorai.009G187800.1 . . Vv03g0341

. . . . . . . .
. . . . .
Vv18g0109 . . . . . . .
. . . . . . . .
. . . . .
Vv18g0110 . . . . . . .
. . . . . . . .
. . . . .
Vv18g0111 . . . . . . .
. . . . . . . .
. . . . .
Vv18g0112 . Gorai.002G117000.1 . . .
. . . . . . . .
. . . . . . .
Vv18g0113 . . . . . . .
. . . . . . . .
. . . . .
Vv18g0114 . . . . . . .
. . . . . . . .
. . . . .
Vv18g0115 Tc08_g004890 . . . . .
. . . . . . . .
. . . . . .
Vv18g0116 . . . . . . .
. . . . . . . .
. . . . .
Vv18g0117 Tc08_g007990 . . . . .
. . . . . . . .
. . . . . .
Vv18g0118 . . . . . . .
. . . . . . . .
. . . . .
Vv18g0119 . . . . . . .
. . . . . . . .
. . . . .
Vv18g0120 . . . . . . .
. . . . . . . .
. . . . .
Vv18g0121 Tc08_g007910 . . . . .
. . . . . . . .
. . . . . .
Vv18g0122 . . . . . . .
. . . . . . . .
. . . . .
Vv18g0123 . . . . . . .
. . . . . . . .
. . . . .
Vv18g0124 . . . Gorai.009G187900.1 .
. . . . . . . .
. . . . . . .

Vv18g0125 . . Gorai.005G113700.1 . .
. . . . . . . .
. . . . . . .
Vv18g0126 . . . . . . .
. . . . . . . .
. . . . .
Vv18g0127 Tc08_g004800 Gorai.002G118000.1 . .
. . . . . . . .
. . . . . . . .
Vv18g0128 Tc08_g004790 Gorai.002G118100.1 . .
. . . . . . . .
. . . . . . . .
Vv18g0129 Tc08_g004780 Gorai.002G118200.1 . .
. . . . . . . .
. Vv01g0514 Tc02_g027180 . . .
. .
Vv18g0130 Tc08_g004770 Gorai.002G118400.1 .
Gorai.009G188200.1 . . . . . .
. . . . . . . .
. .
Vv18g0131 Tc08_g004760 . . . Gorai.
009G267800.1 . . . . . . .
. . . . . . . .
Vv18g0132 Tc08_g004750 Gorai.002G118500.1 .
Gorai.009G188300.1 . . . . . .
. . . . . . . .
. .
Vv18g0133 . . . . . . .
. . . . . . . .
. . . . .
Vv18g0134 . . . . . . .
. . . . . . . .
. . . . .
Vv18g0135 . . . . . . .
. . . . . . . .
. . . . .
Vv18g0136 Tc08_g004720 . . Gorai.009G188600.1
. . . . . . . .
. . . . . . . .
Vv18g0137 Tc08_g004710 Gorai.002G118800.1 .
Gorai.009G188700.1 . . . . . .
. . . . . . . .
. .
Vv18g0138 . . . . . . .
. . . . . . . .
. . . . .
Vv18g0139 Tc08_g004660 Gorai.002G119100.1 .
Gorai.009G188900.1 . . Vv03g0311 . .
. . . . . . . .
. . .

Vv18g0140 . . . . . . .
. . . . . . . .
. . . . .
Vv18g0141 . . . . . . .
. . . . . . . .
. . . . .
Vv18g0142 . . . . . . .
. . . . . . . .
. . . . .
Vv18g0143 Tc08_g004630 . Gorai.005G113500.1 .
. . . . . . . .
. . . . . . . .
Vv18g0144 Tc08_g004620 Gorai.002G119500.1 Gorai.
005G113000.1 . . . . . . .
. . . . . . . .
. .
Vv18g0145 . . . . . . .
. . . . . . . .
. . . . .
Vv18g0146 Tc08_g004590 . . . . .
. . . . . . . .
. . . . . .
Vv18g0147 . . . . . . .
. . . . . . . .
. . . . .
Vv18g0148 Tc08_g004580 . . . . .
. . . . . . . .
. . . . . .
Vv18g0149 Tc08_g004560 . Gorai.005G112900.1
Gorai.009G189400.1 Gorai.009G267900.1 . . .
. . . . . . . .
. . . .
Vv18g0150 . . . . . . .
. . . . . . . .
. . . . .
Vv18g0151 . . . . . . .
. . . . . . . .
. . . . .
Vv18g0152 Tc08_g004540 . . Gorai.009G189600.1
. . . . . . . .
. . . . . . . .
Vv18g0153 Tc08_g004530 Gorai.002G119700.1 . .
. . . . . . . .
. Vv04g1420 Tc01_g010700 . . .
. .
Vv18g0154 . . . . . . .
. . . . . . Vv04g1419
. . . . . .
Vv18g0155 . . . . . . .
. . . . . . . .

. . . . .
Vv18g0156 Tc08_g004510 . . Gorai.009G189800.1
. . . . . . . .
. . . . . . . .
Vv18g0157 . . . . . . .
. . . . . . . .
. . . . .
Vv18g0158 Tc08_g004490 Gorai.002G120000.1 .
Gorai.009G189900.1 Gorai.009G268100.1 . . .
. . . . . . . .
. . . .
Vv18g0159 Tc08_g004480 . . Gorai.009G190000.1
Gorai.009G268200.1 . . . . .
. . . . . . . .
. .
Vv18g0160 Tc08_g004470 . . . . .
. . . . . . . .
. . . . . .
Vv18g0161 . . . . Gorai.009G268300.1
. . . . . . . .
. . . . . . .
Vv18g0162 Tc08_g004460 . . Gorai.009G190100.1
. . . . . . . .
. . . . . . . .
Vv18g0163 Tc08_g004450 Gorai.002G120100.1 . .
. . . . . . . .
. . . . . . . .
Vv18g0164 Tc08_g004430 . . Gorai.009G190200.1
. . . . . . . .
. . . . . . . .
Vv18g0165 Tc08_g004420 . . . Gorai.
009G268400.1 Gorai.010G001000.1 . . . .
. . . . . . . .
. .
Vv18g0166 . . . . . . .
. . . . . . . .
. . . . .
Vv18g0167 . . . . . . .
. . . . . . . .
. . . . .
Vv18g0168 Tc08_g004400 Gorai.002G120400.1 . .
. . . . . . . .
. . . . . . . .
Vv18g0169 Tc08_g004390 Gorai.002G120500.1 . .
. . . . . . . .
. . . . . . . .
Vv18g0170 Tc08_g004380 Gorai.002G120600.1 . .
. . . . . . . .
. . . . . . . .
Vv18g0171 Tc08_g004370 . . Gorai.009G190400.1

```
         .        .        .        .        .        .        .        .
         .        Vv04g1409         Tc01_g000500      .        .        .
         .        .
Vv18g0172         Tc08_g004350      .        .        .        .        .
         .        .        .        .        .        .        .        .
         .        .        .        .        .        .
Vv18g0173         Tc08_g004340      .        .        .        .        .
         .        .        .        .        .        .        .        .
         .        .        .        .        .        .
Vv18g0174         Tc08_g004330      .        .        Gorai.009G190500.1
         .        .        Vv03g0272         Tc02_g004330      .        .
         .        .        .        .        .        .        .        .
         .        .
Vv18g0175         .        .        .        .        .        .        .
         .        .        .        .        .        .        .        .
         .        .        .        .        .
Vv18g0176         Tc08_g004310      .        .        .        .        .
         .        .        .        .        .        .        .        .
         .        .        .        .        .        .
Vv18g0177         Tc08_g004300      Gorai.002G120700.1       .        .
         .        .        .        .        .        .        .        .
         .        .        .        .        .        .        .        .
Vv18g0178         .        .        .        .        .        .        .
         .        .        .        .        .        .        .        .
         .        .        .        .        .
Vv18g0179         Tc08_g004290      .        .        Gorai.009G190800.1
         .        .        .        .        .        .        .        .
         .        .        .        .        .        .        .        .
Vv18g0180         .        .        .        .        Gorai.009G268500.1
         .        .        .        .        .        .        .        .
         Vv04g1378         Tc01_g001040      .        Gorai.008G061600.1
         .        .        .
Vv18g0181         Tc08_g004280      Gorai.002G120800.1       .        .
         .        .        .        .        .        .        .        .
         .        Vv04g1376         .        .        .        .
Gorai.003G005500.1         .
Vv18g0182         .        .        .        .        .        .        .
         .        .        .        .        .        .        .        .
         .        .        .        .        .
Vv18g0183         Tc08_g004270      Gorai.002G120900.1       .
Gorai.009G190900.1         .        .        Vv03g0260
Tc02_g004390      Gorai.002G200900.1       Gorai.005G255500.1       .
         Gorai.008G037600.1       .        .        .        .        .
         .        .        .
Vv18g0184         Tc08_g004260      .        .        Gorai.009G191000.1
         Gorai.009G268600.1       .        Vv03g0259         .        .
         .        .        .        .        .        .        .        .
         .        .        .
Vv18g0185         Tc08_g004250      .        .        Gorai.009G191100.1
         .        .        .        .        .        .        .        .
```

. . . . . . . .
Vv18g0186 . . . . . . .
. . . . . . . .
. . . . .
Vv18g0187 Tc08_g004240 . . Gorai.009G191200.1
Gorai.009G268900.1 . . . . .
. . . . . . . .
. .
Vv18g0188 . . . . . . .
. . . . . . . .
. . . . .
Vv18g0189 . . . . . . .
. . . . . . . .
. . . . .
Vv18g0190 . . . . . . .
. . . . . . Vv04g1374
. . . Gorai.007G190500.1 Gorai.
003G005700.1 .
Vv18g0191 . . . . . . .
. . . . . . Vv04g1373
. . . . . .
Vv18g0192 . . . . . . .
. . . . . . . .
. . . . .
Vv18g0193 Tc08_g004220 . . Gorai.009G191300.1
. . . . . . . .
. . . . . . . .
Vv18g0194 Tc08_g004200 . . . . .
Vv03g0252 Tc02_g004550 Gorai.002G200500.1
Gorai.005G255100.1 Gorai.002G002200.1 . . .
. . . . . .
Vv18g0195 Tc08_g004190 . Gorai.005G112700.1
Gorai.009G191400.1 Gorai.009G269000.1 . . .
. . . . . Vv04g1371
Tc01_g001090 . Gorai.008G061800.1 Gorai.007G190600.1
. .
Vv18g0196 Tc08_g004180 . . . . .
. . . . . . . .
. . . . . .
Vv18g0197 . . . . . . .
. . . . . . . .
. . . . .
Vv18g0198 Tc08_g004170 . . Gorai.009G191500.1
. Gorai.010G001300.1 . . . .
. . . . . . . .
. .
Vv18g0199 Tc08_g004150 Gorai.002G121100.1 . .
Gorai.009G269100.1 . Vv03g0245 . .
. . . . . . . .
. . .

Vv18g0200 Tc08_g004140 Gorai.002G121200.1 Gorai.
005G109800.1 Gorai.009G191800.1 . . . .
. . . . . . . .
. . . .
Vv18g0201 Tc08_g008510 . . . .
Gorai.010G001500.1 . . . . . .
. . . . . . . .
Vv18g0202 . . . Gorai.009G191900.1 .
. . . . . . . .
. . . . . . .
Vv18g0203 Tc08_g004130 . . . . .
. . . . . . . .
. . . . . .
Vv18g0204 . . . . . . .
. . . . . . . .
. . . . .
Vv18g0205 Tc08_g004120 . . . .
Gorai.010G001700.1 . . . . . .
. . . . . . . .
Vv18g0206 Tc08_g004090 Gorai.002G121600.1 .
Gorai.009G192000.1 . . . . . .
. . . Vv07g1282 . Gorai.
007G063100.1 . . . .
Vv18g0207 . . . . . . .
. . . . . . . .
. . . . .
Vv18g0208 . . . . . .
Vv03g0229 . Gorai.002G199300.1 . . .
. . . . . . . .
Vv18g0209 Tc08_g004080 . . . . .
Vv03g0228 Tc02_g004740 Gorai.002G199200.1 .
. Gorai.008G037000.1 . . . .
. . . .
Vv18g0210 Tc08_g004070 . . Gorai.009G192100.1
Gorai.009G269200.1 Gorai.010G001800.1 . .
. . . . . . . .
. . . .
Vv18g0211 Tc08_g004060 . . Gorai.009G192200.1
. Gorai.010G001900.1 . . . .
. . . . . . . .
. .
Vv18g0212 Tc08_g004030 . . Gorai.009G192400.1
. . . . . . . .
. . . . . . . .
Vv18g0213 . . . Gorai.009G250700.1 .
. . . . . . . .
. . . . . . .
Vv18g0214 . . . . . . .
. . . . . . . .
. . . . .

Vv18g0215 . . . . . . .
. . . . . . . .
. . . . .
Vv18g0216 . . . . . . .
. . . . . . . .
. . . . .
Vv18g0217 . . . . . . .
. . . . . . . .
. . . . .
Vv18g0218 Tc08_g008900 . Gorai.005G086100.1
Gorai.009G250600.1 . . . . . .
. . . . . . . .
. .
Vv18g0219 . . . . . . .
. . . . . . . .
. . . . .
Vv18g0220 . . . . . . .
. . . . . . . .
. . . . .
Vv18g0221 . . . . . . .
. . . . . . . .
. . . . .
Vv18g0222 . . . . . . .
. . . . . . . .
. . . . .
Vv18g0223 . . . . . . .
. . . . . . . .
. . . . .
Vv18g0224 . . . . . . .
. . . . . . . .
. . . . .
Vv18g0225 . . . . . . .
. . . . . . . .
. . . . .
Vv18g0226 . . . . . . .
. . . . . . . .
. . . . .
Vv18g0227 . . . . . . .
. . . . . . . .
. . . . .
Vv18g0228 . . . . . . .
. . . . . . . .
. . . . .
Vv18g0229 Tc08_g011790 . . . . .
. . . . . . . .
. . . . . .
Vv18g0230 . . . . . . .
. . . . . . . .
. . . . .
Vv18g0231 . . . . . . .

```
        .       .       .       .       .       .       .       .
        .       .       .       .       .
Vv18g0232       Tc08_g011440    .       .       Gorai.009G249600.1
        .       Gorai.010G050100.1      Vv07g1310       .       .
        .       .       .       .       .       .       .       .
        .       .       .
Vv18g0233       .       .       .       Gorai.009G249500.1      .
        .       .       .       .       .       .       .       .
        .       .       .       .       .       .       .
Vv18g0234       .       .       .       Gorai.009G249400.1      .
        .       .       .       .       .       .       .       .
        .       .       .       .       .       .       .
Vv18g0235       .       .       .       .       .       .       .
        .       .       .       .       .       .       .       .
        .       .       .       .       .
Vv18g0236       .       .       Gorai.005G087000.1      Gorai.
009G249300.1    .       .       .       .       .       .       .
        .       .       .       .       .       .       .       .
        .
Vv18g0237       .       .       Gorai.005G087100.1      Gorai.
009G249200.1    .       .       .       .       .       .       .
        .       .       .       .       .       .       .       .
        .
Vv18g0238       .       .       Gorai.005G087200.1      .       .
        .       .       .       .       .       .       .       .
        .       .       .       .       .       .       .
Vv18g0239       .       .       .       .       .       .       .
        .       .       .       .       .       .       .       .
        .       .       .       .       .
Vv18g0240       .       .       .       .       .       .       .
        .       .       .       .       .       .       .       .
        .       .       .       .       .
Vv18g0241       .       .       .       Gorai.009G249000.1      .
        .       .       .       .       .       .       .       .
        .       .       .       .       .       .       .
Vv18g0242       .       .       .       .       .       .       .
        .       .       .       .       .       .       .       .
        .       .       .       .       .
Vv18g0243       .       .       .       .       .       .       .
        .       .       .       .       .       .       .       .
        .       .       .       .       .
Vv18g0244       .       .       Gorai.005G087500.1      Gorai.
009G248900.1    .       .       .       .       .       .       .
        .       .       .       .       .       .       .       .
        .
Vv18g0245       .       .       .       .       .       .       .
        .       .       .       .       .       .       .       .
        .       .       .       .       .
Vv18g0246       Tc08_g002950    .       Gorai.005G087600.1      .
        Gorai.009G271100.1      .       .       .       .       .
```

. . . . . . . .
. .
Vv18g0247 . . . . . . .
. . . . . . . .
. . . . .
Vv18g0248 . . Gorai.005G087700.1 . .
. . . . . . . .
. . . . . . .
Vv18g0249 . . Gorai.005G087800.1 . .
. . . . . . . .
. . . . . . .
Vv18g0250 . . . . . . .
. . . . . . . .
. . . . .
Vv18g0251 . . . Gorai.009G248800.1 .
. . . . . . . .
. . . . . . .
Vv18g0252 . . . . . . .
. . . . . . . .
. . . . .
Vv18g0253 . . . . . .
Vv07g1318 . . . . . . .
. . . . . .
Vv18g0254 . . . . . . .
. . . . . . . .
. . . . .
Vv18g0255 Tc08_g000320 . Gorai.005G088100.1 .
. . . . . . . .
. . . . . . . .
Vv18g0256 . . . . . . .
. . . . . . . .
. . . . .
Vv18g0257 Tc08_g000220 . . Gorai.009G213700.1
Gorai.009G276000.1 . . . . .
. . . . . . . .
. .
Vv18g0258 . . . . . . .
. . . . . . . .
. . . . .
Vv18g0259 . . . Gorai.009G195000.1 .
. . . . . . . .
. . . . . . .
Vv18g0260 . . . . . . .
. . . . . . . .
. . . . .
Vv18g0261 . . . Gorai.009G248600.1 .
. Vv07g1321 Tc01_g012580 . . .
. . . . . . . .
.
Vv18g0262 . . . Gorai.009G248500.1 .

```
        .       .       .       .       .       .       .       .
        .       .       .       .       .       .       .
Vv18g0263       .       .       Gorai.005G088400.1      .       .
        .       .       .       .       .       .       .       .
        .       .       .       .       .       .       .
Vv18g0264       .       .       .       .       .       Gorai.
010G053300.1    .       .       .       .       .       .       .
        .       .       .       .       .       .       .
Vv18g0265       .       .       .       Gorai.009G248300.1      .
        Gorai.010G052900.1      .       .       .       .       .
        .       .       .       .       .       .       .       .
        .
Vv18g0266       Tc08_g011050    .       .       .       .
Gorai.010G052800.1      Vv04g1342       Tc01_g001390    .       .
        .       .       Gorai.011G098200.1      .       .       .
        .       .       .       .
Vv18g0267       .       .       .       Gorai.009G248100.1      .
        .       .       .       .       .       .       .       .
        .       .       .       .       .       .       .
Vv18g0268       .       .       .       .       .       Gorai.
010G052500.1    .       .       .       .       .       .       .
        .       .       .       .       .       .       .
Vv18g0269       .       .       .       .       .       .       .
        .       .       .       .       .       .       .       .
        .       .       .       .       .
Vv18g0270       .       .       .       .       .       .       .
        .       .       .       .       .       .       .       .
        .       .       .       .       .
Vv18g0271       .       .       .       .       .       .       .
        .       .       .       .       .       .       .       .
        .       .       .       .       .
Vv18g0272       .       .       Gorai.005G088500.1      .
Gorai.009G284000.1      .       .       .       .       .       .
        .       .       .       .       .       .       .       .
        .
Vv18g0273       .       .       .       .       .       .       .
        .       .       .       .       .       .       .       .
        .       .       .       .       .
Vv18g0274       .       .       .       Gorai.009G248000.1      .
        .       .       .       .       .       .       .       .
        .       .       .       .       .       .       .
Vv18g0275       Tc08_g010990    .       .       .       .
Gorai.010G052300.1      Vv04g1350       .       .       .       .
        .       .       Vv07g1326       Tc01_g012690    .       .
        .       .       .
Vv18g0276       Tc08_g010980    .       .       .       .
Gorai.010G052200.1      Vv04g1351       .       .       .       .
        .       .       Vv07g1337       Tc01_g012700    .       .
        .       .       .
Vv18g0277       .       .       Gorai.005G088700.1      .
```

Gorai.009G283900.1 Gorai.010G052100.1 . . .
. . . . . . . .
. . .
Vv18g0278 . . . . . . .
. . . . . . . .
. . . . .
Vv18g0279 . . . Gorai.009G247900.1 .
. . . . . . . .
. . . . . . .
Vv18g0280 . . . . . . .
. . . . . . . .
. . . . .
Vv18g0281 Tc08_g000180 . . . . .
. . . . . . . .
. . . . . .
Vv18g0282 . . . . . . .
. . . . . . . .
. . . . .
Vv18g0283 . . . Gorai.009G247700.1 .
Gorai.010G051700.1 . . . . .
. . . . . . . .
.
Vv18g0284 Tc08_g010880 . . Gorai.009G247600.1
. . . . . . . .
. . . . . . . .
Vv18g0285 Tc08_g003030 . . . . .
. . . . . . . .
. . . . . .
Vv18g0286 . . Gorai.005G089000.1 . .
. . . . . . . .
Vv07g1339 Tc01_g012720 . . . .
.
Vv18g0287 . . Gorai.005G089100.1 Gorai.
009G247000.1 . Gorai.010G051500.1 . . .
. . . . Vv07g1342
Tc01_g012750 . . . . .
Vv18g0288 . . . Gorai.009G246900.1 .
Gorai.010G051400.1 . . . . .
. . . . . . . .
.
Vv18g0289 . . . Gorai.009G246800.1 .
. . . . . . . .
Vv07g1346 Tc01_g012790 . . . .
.
Vv18g0290 . . Gorai.005G089200.1 Gorai.
009G246700.1 Gorai.009G283800.1 . . . .
. . . . Vv07g1348 . .
. . . .
Vv18g0291 . . . Gorai.009G246600.1 .
Gorai.010G051300.1 . . . . .

. . . . . . . .
.
Vv18g0292 . . . . . . .
. . . . . . . .
. . . . .
Vv18g0293 . . . Gorai.009G246300.1 .
Gorai.010G051100.1 . . . . .
. . . . . . . .
.
Vv18g0294 . . . . . . .
. . . . . . . .
. . . . .
Vv18g0295 . . . . . . .
. . . . . . . .
. . . . .
Vv18g0296 . . . . . Gorai.
010G051000.1 . . . . . . .
. . . . . . .
Vv18g0297 . . . . . . .
. . . . . . . .
. . . . .
Vv18g0298 Tc08_g003070 . . . . .
. . . . . . . .
. . . . . .
Vv18g0299 Tc08_g010510 . Gorai.005G089300.1 .
. . . . . . . .
. . . . . . . .
Vv18g0300 . . . . . Gorai.
010G050500.1 . . . . . . .
. . . . . . .
Vv18g0301 . . Gorai.005G089400.1 Gorai.
009G246000.1 . . . . . . .
. . . . . . . .
.
Vv18g0302 . . . . Gorai.009G283400.1
. . . . . . . .
. . . . . . .
Vv18g0303 Tc08_g003390 . . Gorai.009G245900.1
Gorai.009G280500.1 Gorai.010G050400.1 . .
. . . . . Vv07g1355
Tc01_g012870 . . . . .
Vv18g0304 . . . Gorai.009G245800.1 .
. . . . . . . .
. . . . . . .
Vv18g0305 . . . . . Gorai.
010G050300.1 Vv04g1372 Tc01_g001070 . . .
Gorai.003G005800.1 . Vv07g1360
Tc01_g020690 . . . . .
Vv18g0306 . . . . . . .
. . . . . . . .

. . . . .
Vv18g0307 . . . . . . .
. . . . . . . .
. . . . .
Vv18g0308 . . . . . . .
. . . . . . . .
. . . . .
Vv18g0309 . . Gorai.005G089800.1 Gorai.
009G245700.1 . . . . . . .
. . . . . . . .
.
Vv18g0310 Tc08_g010480 . . Gorai.009G245600.1
. Gorai.010G050200.1 . . . .
. . . . . . . .
. .
Vv18g0311 . . . . . . .
. . . . . . . .
. . . . .
Vv18g0312 . . . . . . .
. . . . . . . .
. . . . .
Vv18g0313 . . . . . . .
. . . . . . . .
. . . . .
Vv18g0314 . . . . . . .
. . . . . . . .
. . . . .
Vv18g0315 . . . . . Gorai.
010G050000.1 . . . . . . .
. . . . . . .
Vv18g0316 . . Gorai.005G089900.1 Gorai.
009G245400.1 . . . . . . .
. . . . . . . .
.
Vv18g0317 . . Gorai.005G090000.1 . .
Gorai.010G049900.1 . . . . .
. . . . . . . .
.
Vv18g0318 . . . . . Gorai.
010G049800.1 . . . . . . .
. . . . . . .
Vv18g0319 . . . . . Gorai.
010G049700.1 . . . . . . .
. . . . . . .
Vv18g0320 . . . . . . .
. . . . . . Vv07g1375
Tc01_g013040 . . . . .
Vv18g0321 . . . . . . .
. . . . . . . .
. . . . .

Vv18g0322 . . . Gorai.009G245300.1 .
. . . . . . . .
. . . . . . .
Vv18g0323 . . . . . . .
. . . . . . . .
. . . . .
Vv18g0324 . . . Gorai.009G245200.1 .
. . . . . . . .
. . . . . . .
Vv18g0325 . . . Gorai.009G245100.1 .
Gorai.010G049600.1 . . . . .
. . . . . . . .
.
Vv18g0326 . . . . . Gorai.
010G049400.1 . . . . . . .
. . . . . . .
Vv18g0327 . . . . . . .
. . . . . . . .
. . . . .
Vv18g0328 . . . . . .
Vv04g1408 . . . . . . .
. . . . . .
Vv18g0329 . . . . . .
Vv04g1436 Tc01_g010750 . . . . .
Vv1rg0413 . . . . . .
Vv18g0330 . . . . . .
Vv04g1441 . . . . . .
Vv1rg0409 . . . . . .
Vv18g0331 . . . Gorai.009G244600.1 .
. . . . . . . .
. . . . . . .
Vv18g0332 . . . . . Gorai.
010G049000.1 . . . . . . .
. . . . . . .
Vv18g0333 . . . . . . .
. . . . . . . .
. . . . .
Vv18g0334 . . . . . . .
. . . . . . . .
. . . . .
Vv18g0335 . . . . . . .
. . . . . . . .
. . . . .
Vv18g0336 . . . Gorai.009G244500.1 .
Gorai.010G048900.1 . . . . .
. . . . . . . .
.
Vv18g0337 . . Gorai.005G090400.1 Gorai.
009G244400.1 . . . . . . .
. . . . . . . .

.
Vv18g0338 . . Gorai.005G090500.1 . .
. . . . . . . .
. . . . . . .
Vv18g0339 . . . . . . .
. . . . . . . .
. . . . .
Vv18g0340 . . . Gorai.009G244300.1 .
Gorai.010G048700.1 . . . . .
. . . . . . . .
.
Vv18g0341 . . Gorai.005G090600.1 . .
. . . . . . . .
. . . . . . .
Vv18g0342 Tc08_g010330 . . . . .
Vv04g1469 . . . . . .
. . . . . . .
Vv18g0343 Tc08_g010320 . . Gorai.009G244100.1
. . . . . . . .
. . . . . . . .
Vv18g0344 Tc08_g010300 . . Gorai.009G244000.1
. . . . . . . .
. . . . . . . .
Vv18g0345 Tc08_g010290 . . . . .
. . . . . . . .
. . . . . .
Vv18g0346 . . . Gorai.009G243900.1 .
. . . . . . . .
. . . . . . .
Vv18g0347 Tc08_g010280 . . Gorai.009G243800.1
. Gorai.010G048500.1 . . . .
. . . . . . . .
. .
Vv18g0348 . . . . . . .
. . . . . . . .
. . . . .
Vv18g0349 . . . . . . .
. . . . . . . .
. . . . .
Vv18g0350 Tc08_g010270 . . . . .
. . . . . . . .
. . . . . .
Vv18g0351 . . . . . . .
. . . . . . . .
. . . . .
Vv18g0352 . . . . . . .
. . . . . . . .
. . . . .
Vv18g0353 Tc08_g010260 . Gorai.005G090800.1
Gorai.009G243700.1 . . . . . .

```
        .       .       .       .       .       .       .       .
        .       .
Vv18g0354       .       .       .       .       .       .       .
        .       .       .       .       .       .       .       .
        .       .       .       .       .
Vv18g0355       .       .       .       .       .       .       .
        .       .       .       .       .       .       .       .
        .       .       .       .       .
Vv18g0356       .       .       .       .       .       .       .
        .       .       .       .       .       .       .       .
        .       .       .       .       .
Vv18g0357       Tc08_g010240    .       .       Gorai.009G243600.1
        .       .       .       .       .       .       .       .
        .       .       .       .       .       .       .       .
Vv18g0358       .       .       .       .       .       .       .
        .       .       .       .       .       .       .       .
        .       .       .       .       .
Vv18g0359       Tc08_g010230    .       .       .       .
Gorai.010G048400.1      .       .       .       .       .       .
        .       .       .       .       .       .       .       .
Vv18g0360       .       .       .       Gorai.009G243500.1      .
        Gorai.010G048300.1      .       .       .       .       .
        .       .       .       .       .       .       .       .
        .
Vv18g0361       Tc08_g010200    .       .       .       .
Gorai.010G048200.1      .       .       .       .       .       .
        .       .       .       .       .       .       .       .
Vv18g0362       Tc08_g010190    .       Gorai.005G090900.1
Gorai.009G243400.1      .       .       .       .       .       .
        .       .       .       .       .       .       .       .
        .       .
Vv18g0363       Tc08_g010180    .       Gorai.005G091100.1      .
        .       Gorai.010G048100.1      .       .       .       .
        .       .       .       .       .       .       .       .
        .       .
Vv18g0364       Tc08_g010170    .       .       Gorai.009G243300.1
        .       .       .       .       .       .       .       .
        .       .       .       .       .       .       .       .
Vv18g0365       .       .       .       .       .       .       .
        .       .       .       .       .       .       .       .
        .       .       .       .       .
Vv18g0366       Tc08_g010160    .       .       Gorai.009G243200.1
        .       Gorai.010G048000.1      Vv02g0595
Tc03_g025370    .       Gorai.003G170100.1      .       .       .
        .       .       .       .       .       .       .
Vv18g0367       Tc08_g010150    .       .       .       .       .
        .       .       .       .       .       .       .       .
        .       .       .       .       .       .
Vv18g0368       Tc08_g010090    .       .       Gorai.009G243100.1
        .       Gorai.010G047900.1      .       .       .       .
```

. . . . . . . .
. .
Vv18g0369 Tc08_g010060 . Gorai.005G091200.1 .
. . . . . . . .
. Vv1rg0392 . . . . .
.
Vv18g0370 Tc08_g010040 . . Gorai.009G243000.1
. . . . . . . .
. . . . . . . .
Vv18g0371 . . . . . . .
. . . . . . . .
. . . . .
Vv18g0372 Tc08_g010030 . Gorai.005G091400.1
Gorai.009G242900.1 Gorai.009G283100.1 Gorai.010G047800.1
Vv02g0599 Tc03_g025330 . Gorai.003G170200.1
. . . . . . . .
. .
Vv18g0373 Tc08_g010020 . . Gorai.009G242800.1
Gorai.009G283000.1 . . . . .
. . . . . . . .
. .
Vv18g0374 Tc08_g010010 . . . .
Gorai.010G047700.1 . . . . . .
. . . . . . . .
Vv18g0375 Tc08_g010000 . Gorai.005G091500.1 .
. Gorai.010G047600.1 . . . .
. . . . . . . .
. .
Vv18g0376 . . . . . . .
. . . . . . . .
. . . . .
Vv18g0377 Tc08_g009960 . Gorai.005G091600.1 .
. . . . . . . .
. . . . . . . .
Vv18g0378 Tc08_g009950 . . . Gorai.
009G282900.1 Gorai.010G047400.1 . . . .
. . . . . . . .
. .
Vv18g0379 Tc08_g009940 . . Gorai.009G242600.1
. . . . . . . .
. . . . . . . .
Vv18g0380 . . . . . . .
. . . . . . . .
. . . . .
Vv18g0381 Tc08_g009930 . Gorai.005G091900.1 .
Gorai.009G282700.1 Gorai.010G047200.1 . .
. . . . . . . .
. . . .
Vv18g0382 Tc08_g009890 . Gorai.005G092000.1 .
. Gorai.010G047100.1 . . . .

```
        .        .       .        .       .        .       .        .
        .        .
Vv18g0383        Tc08_g009880     .       .        .       .
Gorai.010G047000.1       .        .       .        .       .        .
        .        .       .        .       .        .       .        .
Vv18g0384        Tc08_g009870     .       .        Gorai.009G242400.1
        .        Gorai.010G046900.1       .        .       .        .
        .        .       .        .       .        .       .        .
        .        .
Vv18g0385        .       .        .       .        .       .        .
        .        .       .        .       .        .       .        .
        .        .       .        .       .
Vv18g0386        Tc08_g009850     .       .        Gorai.009G242200.1
        .        Gorai.010G046700.1       .        .       .        .
        .        .       .        .       .        .       .        .
        .        .
Vv18g0387        Tc08_g009840     .       .        Gorai.009G242100.1
        .        Gorai.010G046600.1       .        .       .        .
        .        .       .        .       .        .       .        .
        .        .
Vv18g0388        Tc08_g009830     .       .        Gorai.009G242000.1
        .        Gorai.010G046500.1       .        .       .        .
        .        .       .        .       .        .       .        .
        .        .
Vv18g0389        .       .        .       .        .       .        .
        .        .       .        .       .        .       .        .
        .        .       .        .       .
Vv18g0390        Tc08_g009820     .       .        Gorai.009G241900.1
        .        Gorai.010G046000.1       .        .       .        .
        .        .       .        .       .        .       .        .
        .        .
Vv18g0391        Tc08_g009810     .       .        Gorai.009G241800.1
        .        .       .        .       .        .       .        .
        .        .       .        .       .        .       .        .
Vv18g0392        .       .        .       .        .       .        .
        .        .       .        .       .        .       .        .
        .        .       .        .       .
Vv18g0393        .       .        .       .        .       .        .
        .        .       .        .       .        .       .        .
        .        .       .        .       .
Vv18g0394        .       .        .       .        .       .        .
        .        .       .        .       .        .       .        .
        .        .       .        .       .
Vv18g0395        .       .        .       .        .       .        .
        .        .       .        .       .        .       .        .
        .        .       .        .       .
Vv18g0396        .       .        .       .        .       .        .
        .        .       .        .       .        .       .        .
        .        .       .        .       .
Vv18g0397        Tc08_g009790     .       Gorai.005G092100.1
```

```
Gorai.009G241600.1        .       .        .       .        .       .
         .       .        .       .        .       .        .       .
         .       .
Vv18g0398        Tc08_g009780     .        Gorai.005G092200.1
Gorai.009G241500.1        .       Gorai.010G045900.1        .       .
         .       .        .       .        .       .        .       .
         .       .        .       .
Vv18g0399        Tc08_g009770     .        .       .        .
Gorai.010G045800.1        .       .        .       .        .       .
         .       .        .       .        .       .        .       .
Vv18g0400        Tc08_g009760     .        .       .        .       .
         .       .        .       .        .       .        .       .
         .       .        .       .        .       .
Vv18g0401        Tc08_g009750     .        .       .        .       .
         Vv02g0628        Tc03_g025050     .       Gorai.003G171200.1
         .       .        .       Vv1rg0375        .        .       .
         .       .        .
Vv18g0402        Tc08_g009740     .        Gorai.005G092400.1       .
         .       Gorai.010G045700.1        .       .        .       .
         .       .        .       .        .       .        .       .
         .       .
Vv18g0403        Tc08_g009730     .        .       .        .
Gorai.010G045600.1        .       .        .       .        .       .
         .       Vv07g1202        .        .       .        .       .
         .
Vv18g0404        .        .       .        .       .        .       .
         .       .        .       .        .       .        Vv07g1201
         .       .        .       .        .       .
Vv18g0405        Tc08_g009710     .        Gorai.005G092500.1       .
         Gorai.009G282600.1       .        .       .        .       .
         .       .        .       .        .       .        .       .
         .       .
Vv18g0406        Tc08_g009700     .        .       Gorai.009G241300.1
         .       .        .       .        .       .        .       .
         .       .        .       .        .       .        .       .
Vv18g0407        Tc08_g009690     .        .       Gorai.009G241200.1
         .       .        .       .        .       .        .       .
         .       .        .       .        .       .        .       .
Vv18g0408        Tc08_g009680     .        .       Gorai.009G241100.1
         .       .        .       .        .       .        .       .
         .       .        .       .        .       .        .       .
Vv18g0409        Tc08_g009660     .        .       .        .
Gorai.010G045400.1        .       .        .       .        .       .
         .       .        .       .        .       .        .       .
Vv18g0410        Tc08_g009650     .        .       Gorai.009G241000.1
         .       .        .       .        .       .        .       .
         .       .        .       .        .       .        .       .
Vv18g0411        Tc08_g009620     .        Gorai.005G092600.1       .
         .       Gorai.010G045100.1        .       .        .       .
         .       .        .       .        .       .        .       .
```

. .
Vv18g0412 Tc08_g009610 . . Gorai.009G240600.1
. Gorai.010G045000.1 . . . .
. . . . . . . .
. .
Vv18g0413 . . . . . Gorai.
010G044900.1 . . . . . . .
. . . . . . .
Vv18g0414 Tc08_g009590 . . Gorai.009G240500.1
Gorai.009G282400.1 . . . . .
. . . . . . . .
. .
Vv18g0415 . . Gorai.005G092700.1 . .
Gorai.010G044800.1 . . . . .
. . . . . . . .
.
Vv18g0416 . . . . . . .
. . . . . . Vv07g1194
. Gorai.007G061800.1 . . . .
Vv18g0417 . . . . . . .
. . . . . . Vv07g1193
. . . . Gorai.003G045500.1 .
Vv18g0418 . . . . . . .
. . . . . . . .
. . . . .
Vv18g0419 Tc08_g009580 . . . . .
. . . . . . .
Vv07g1188 . . . . . .
Vv18g0420 Tc08_g009570 . . Gorai.009G240400.1
. . . . . . . .
. Vv07g1187 . . . .
Gorai.003G045300.1 .
Vv18g0421 Tc08_g009560 . Gorai.005G093000.1 .
. . . . . . . .
. Vv07g1185 . . . . .
.
Vv18g0422 . . . . . . .
. . . . . . Vv07g1172
. . . . . .
Vv18g0423 . . . . . . .
. . . . . . Vv07g1170
. . . . . .
Vv18g0424 Tc08_g009520 . . . .
Gorai.010G044500.1 . . . . . .
. . . . . . . .
Vv18g0425 Tc08_g009510 . . Gorai.009G240100.1
. . . . . . . .
. Vv07g1168 . . . . .
.
Vv18g0426 Tc08_g009490 . . Gorai.009G240000.1

. Gorai.010G044300.1 . . . .
. . . . . . . .
. .
Vv18g0427 Tc08_g009470 . . Gorai.009G239900.1
. Gorai.010G044200.1 . . . .
. . . . . . . .
. .
Vv18g0428 Tc08_g009450 . . . Gorai.
009G282300.1 . . . . . . .
. Vv07g0162 Tc01_g027840 Gorai.007G137600.1
. . . Gorai.004G216500.1
Vv18g0429 . . . . . . .
. . . . . . Vv07g0123
Tc01_g028230 . . . Gorai.004G077800.1
.
Vv18g0430 Tc08_g009430 . . Gorai.009G239700.1
. Gorai.010G044000.1 Vv02g0660 .
Gorai.004G190400.1 Gorai.003G179300.1 . .
Gorai.006G215200.1 . . . . . .
.
Vv18g0431 Tc08_g009420 . . Gorai.009G239600.1
. . . . . . . .
. . . . . . . .
Vv18g0432 . . Gorai.005G093100.1 .
Gorai.009G282200.1 . . . . . .
. . . . . . . .
.
Vv18g0433 . . . . . . .
. . . . . . . .
. . . . .
Vv18g0434 . . . . . . .
. . . . . . . .
. . . . .
Vv18g0435 Tc08_g009400 . . Gorai.009G239400.1
. Gorai.010G043600.1 . . . .
. . . . . . . .
. .
Vv18g0436 Tc08_g009390 . . Gorai.009G239300.1
. Gorai.010G043500.1 . . . .
. . . . . . . .
. .
Vv18g0437 . . . . . . .
. . . . . . . .
. . . . .
Vv18g0438 Tc08_g009380 . . Gorai.009G239200.1
Gorai.009G282100.1 . Vv02g0681 .
Gorai.004G189700.1 Gorai.003G179500.1 . .
Gorai.006G215300.1 . . . . . .
.
Vv18g0439 Tc08_g009370 . . . . .

.       .       .       .       .       .       .       .
.       .       .       .       .       .
Vv18g0440       Tc08_g009360    .       Gorai.005G093200.1      .
.       .       .       .       .       .       .       .
.       .       .       .       .       .       .       .
Vv18g0441       Tc08_g009350    .       Gorai.005G093300.1
Gorai.009G239100.1      Gorai.009G282000.1      .       .       .
.       .       .       .       .       .       .       .
.       .       .       .
Vv18g0442       .       .       .       .       .       .       .
.       .       .       .       .       .       .       .
.       .       .       .       .
Vv18g0443       Tc08_g009320    .       .       Gorai.009G238900.1
.       Gorai.010G043300.1      .       .       .       .
.       .       .       .       .       .       .       .
.       .
Vv18g0444       Tc08_g009310    .       Gorai.005G093500.1
Gorai.009G238800.1      .       Gorai.010G043200.1      .       .
.       .       .       .       .       .       .       .
.       .       .       .
Vv18g0445       Tc08_g009300    .       .       Gorai.009G238700.1
Gorai.009G281900.1      .       .       .       .       .
.       .       .       .       .       .       .       .
.       .
Vv18g0446       Tc08_g009290    .       .       Gorai.009G238600.1
.       Gorai.010G042600.1      .       .       .       .
.       .       .       .       .       .       .       .
.       .
Vv18g0447       Tc08_g009270    .       .       .       .
Gorai.010G042500.1      .       .       .       .       .       .
.       .       .       .       .       .       .       .
Vv18g0448       Tc08_g009250    .       .       Gorai.009G238400.1
.       Gorai.010G042400.1      .       .       .       .
.       .       .       .       .       .       .       .
.       .
Vv18g0449       Tc08_g009240    .       .       .       .
Gorai.010G042300.1      .       .       .       .       .       .
.       .       .       .       .       .       .       .
Vv18g0450       Tc08_g009230    .       .       Gorai.009G238300.1
Gorai.009G281800.1      Gorai.010G042200.1      .       .
.       .       .       .       .       .       .       .
.       .       .       .
Vv18g0451       Tc08_g009220    .       .       Gorai.009G238200.1
.       .       .       .       .       .       .       .
.       .       .       .       .       .       .       .
Vv18g0452       Tc08_g009210    .       .       .       Gorai.
009G281700.1    .       Vv02g0693       Tc03_g022270    .       .
.       Gorai.008G220500.1      .       .       .       .
.       .       .       .
Vv18g0453       .       .       .       .       .       .       .

. . . . . . . .
. . . . .
Vv18g0454 Tc08_g009200 . . Gorai.009G238100.1
Gorai.009G281600.1 Gorai.010G042000.1 . .
. . . . . . . .
. . . .
Vv18g0455 Tc08_g009190 . . Gorai.009G238000.1
. Gorai.010G041900.1 . . . .
. . . . . . . .
. .
Vv18g0456 Tc08_g009180 . . Gorai.009G237900.1
. . . . . . . .
. . . . . . . .
Vv18g0457 . . . . . . .
. . . . . . . .
. . . . .
Vv18g0458 Tc08_g009120 . . Gorai.009G237600.1
. . . . . . . .
. . . . . . . .
Vv18g0459 Tc08_g009100 . Gorai.005G094000.1
Gorai.009G237500.1 . . . . . .
. . . . . . . .
. .
Vv18g0460 Tc08_g009090 . . . . .
. . . . . . . .
. . . . . .
Vv18g0461 Tc08_g009060 . . Gorai.009G237200.1
. . . . . . . .
. . . . . . . .
Vv18g0462 Tc08_g009050 . . Gorai.009G237100.1
. . . . . . . .
. . . . . . . .
Vv18g0463 Tc08_g009040 . Gorai.005G094100.1
Gorai.009G237000.1 . Gorai.010G040300.1 . .
. . . . . . . .
. . . .
Vv18g0464 Tc08_g009010 . . Gorai.009G236900.1
Gorai.009G281000.1 . . . . .
. . . . . . . .
. .
Vv18g0465 Tc08_g009000 . . Gorai.009G236700.1
Gorai.009G280900.1 . . . . .
. . . . . . . .
. .
Vv18g0466 Tc08_g008980 . . Gorai.009G236500.1
. Gorai.010G040100.1 Vv02g0733 . .
. . . . Vv07g0089
Tc01_g028380 . . . Gorai.004G056100.1 .
Vv18g0467 Tc08_g008970 . . Gorai.009G236400.1
. . . . . . . .

. . . . . . . .
Vv18g0468 . . . . . . .
. . . . . . . .
. . . . .
Vv18g0469 Tc08_g008960 . . Gorai.009G236300.1
. Gorai.010G040000.1 . . . .
. . . . . . . .
. .
Vv18g0470 Tc08_g008940 . Gorai.005G094200.1 .
. Gorai.010G039900.1 . . . .
. . . . . . . .
. .
Vv18g0471 . . . . . . .
. . . . . . . .
. . . . .
Vv18g0472 Tc08_g008930 . . . . .
. . . . . . . .
. . . . . .
Vv18g0473 Tc08_g008920 . . Gorai.009G236200.1
. . . . . . . .
. . . . . . . .
Vv18g0474 . . . . . . .
. . . . . . . .
. . . . .
Vv18g0475 Tc08_g008900 . . Gorai.009G236100.1
Gorai.009G280800.1 Gorai.010G039800.1 . .
. . . . . . . .
. . . .
Vv18g0476 . . . . . . .
. . . . . . . .
. . . . .
Vv18g0477 Tc08_g008870 . . . .
Gorai.010G039600.1 Vv07g0087 Tc01_g028410 Gorai.
007G139800.1 . . Gorai.004G076800.1 . .
. . . . . .
Vv18g0478 . . . . . . .
. . . . . . . .
. . . . .
Vv18g0479 . . . . . . .
. . . . . . . .
. . . . .
Vv18g0480 Tc08_g008850 . . Gorai.009G235700.1
. . . . . . . .
. . . . . . . .
Vv18g0481 . . . . . . .
. . . . . . . .
. . . . .
Vv18g0482 . . . . . . .
. . . . . . . .
. . . . .

Vv18g0483 . . . . . . .
. . . . . . . .
. . . . .
Vv18g0484 Tc08_g008840 . Gorai.005G094300.1
Gorai.009G235600.1 . . . . . .
. . . . . . . .
. .
Vv18g0485 Tc08_g008830 . . . . .
. . . . . . . .
. . . . . .
Vv18g0486 . . . . . Gorai.
010G039500.1 . . . . . . .
. . . . . . .
Vv18g0487 . . . . . . .
. . . . . . . .
. . . . .
Vv18g0488 . . . . . . .
. . . . . . . .
. . . . .
Vv18g0489 . . . . . . .
. . . . . . . .
. . . . .
Vv18g0490 . . . . . . .
. . . . . . . .
. . . . .
Vv18g0491 . . . . . . .
. . . . . . . .
. . . . .
Vv18g0492 . . . . . . .
. . . . . . . .
. . . . .
Vv18g0493 Tc08_g008820 . Gorai.005G094400.1 .
. . . . . . . .
. . . . . . . .
Vv18g0494 . . . Gorai.009G235500.1 .
. . . . . . . .
. . . . . . .
Vv18g0495 . . . . . . .
. . . . . . . .
. . . . .
Vv18g0496 Tc08_g008810 . . Gorai.009G235400.1
. . . . . . . .
. . . . . . . .
Vv18g0497 . . . Gorai.009G235300.1 .
. . . . . . . .
. . . . . . .
Vv18g0498 . . . . . . .
. . . . . . . .
. . . . .
Vv18g0499 Tc08_g008800 . . Gorai.009G235200.1

. . . . . . . .
. . . . . . . .
Vv18g0500 Tc08_g008790 . . Gorai.009G235100.1
. Gorai.010G038800.1 . . . .
. . . . . . . .
. .
Vv18g0501 Tc08_g008780 . . Gorai.009G235000.1
. . . . . . . .
. . . . . . . .
Vv18g0502 Tc08_g008770 . . Gorai.009G234900.1
. . Vv07g0086 Tc01_g028430 . .
. Gorai.004G076600.1 . . . .
. . . .
Vv18g0503 . . . . . . .
. . . . . . . .
. . . . .
Vv18g0504 Tc08_g008760 . . . .
Gorai.010G038700.1 . . . . . .
. . . . . . . .
Vv18g0505 . . . . . . .
. . . . . . . .
. . . . .
Vv18g0506 Tc08_g008750 . Gorai.005G094500.1
Gorai.009G234800.1 . . . . . .
. . . . . . . .
. .
Vv18g0507 Tc08_g008740 . . Gorai.009G234700.1
. . . . . . . .
. . . . . . . .
Vv18g0508 Tc08_g008730 . . Gorai.009G234600.1
Gorai.009G280500.1 Gorai.010G038600.1 . .
. . . . . . . .
. . . .
Vv18g0509 . . . . . . .
. . . . . . . .
. . . . .
Vv18g0510 . . . . . . .
. . . . . . . .
. . . . .
Vv18g0511 Tc08_g008710 . . . .
Gorai.010G038400.1 . . . . . .
. . . . . . . .
Vv18g0512 Tc08_g008660 . . . .
Gorai.010G038200.1 . . . . . .
. . . . . . . .
Vv18g0513 Tc08_g008650 . . . Gorai.
009G280200.1 Gorai.010G038100.1 . . . .
. . . . . . . .
. .
Vv18g0514 Tc08_g008630 . . Gorai.009G234000.1

```
        .        Gorai.010G037900.1       .        .       .        .
        .        .       .        .       .        .       .        .
        .        .
Vv18g0515        .       .        .       .        .       .        .
        .        .       .        .       .        .       .        .
        .        .       .        .       .
Vv18g0516        Tc08_g008620     .       .        Gorai.009G233900.1
        .        Gorai.010G037800.1       .        .       .        .
        .        .       .        .       .        .       .        .
        .        .
Vv18g0517        Tc08_g008610     .       .        .       .        .
        .        .       .        .       .        .       .        .
        .        .       .        .       .        .
Vv18g0518        .       .        .       .        .       .        .
        .        .       .        .       .        .       .        .
        .        .       .        .       .
Vv18g0519        Tc08_g008600     .       .        .       .
Gorai.010G037700.1       .        .       .        .       .        .
        .        .       .        .       .        .       .        .
Vv18g0520        Tc08_g008590     .       .        Gorai.009G233800.1
        .        .       Vv0Unrg4486      .        .       .        .
        .        .       .        .       .        .       .        .
        .
Vv18g0521        .       .        .       .        .       .        .
        .        .       .        .       .        .       .        .
        .        .       .        .       .
Vv18g0522        .       .        .       .        .       .        .
        .        .       .        .       .        .       .        .
        .        .       .        .       .
Vv18g0523        .       .        .       .        .       .        .
        .        .       .        .       .        .       .        .
        .        .       .        .       .
Vv18g0524        .       .        .       .        .       .        .
        .        .       .        .       .        .       .        .
        .        .       .        .       .
Vv18g0525        Tc08_g008550     .       .        .       .        .
        .        .       .        .       .        .       .        .
        .        .       .        .       .        .
Vv18g0526        Tc08_g008540     .       .        Gorai.009G233400.1
        .        Gorai.010G037300.1       .        .       .        .
        .        .       .        .       .        .       .        .
        .        .
Vv18g0527        .       .        .       .        .       .        .
        .        .       .        .       .        .       .        .
        .        .       .        .       .
Vv18g0528        .       .        .       .        .       .        .
        .        .       .        .       .        .       .        .
        .        .       .        .       .
Vv18g0529        .       .        .       .        .       .        .
        .        .       .        .       .        .       .        .
```

. . . . .
Vv18g0530 Tc08_g008510 . . Gorai.009G233100.1
. . . . . . . .
. . . . . . . .
Vv18g0531 . . Gorai.005G094800.1 Gorai.
009G233000.1 . . Vv0Unrg4509 . . .
. . . . . . . .
. .
Vv18g0532 . . Gorai.005G095100.1 . .
. . . . . . . .
. . . . . . .
Vv18g0533 . . . . . . .
. . . . . . . .
. . . . .
Vv18g0534 . . . Gorai.009G232900.1 .
Gorai.010G037200.1 Vv0Unrg4515 . . .
. . . . . . . .
. .
Vv18g0535 . . . . . . .
. . . . . . . .
. . . . .
Vv18g0536 . . . . . .
Vv0Unrg4516 . . . . . . .
. . . . . .
Vv18g0537 . . . . . . .
. . . . . . . .
. . . . .
Vv18g0538 . . . . . . .
. . . . . . . .
. . . . .
Vv18g0539 . . . . . . .
. . . . . . . .
. . . . .
Vv18g0540 . . . . . . .
. . . . . . . .
. . . . .
Vv18g0541 . . . . . . .
. . . . . . . .
. . . . .
Vv18g0542 . . . Gorai.009G232600.1 .
. . . . . . . .
. . . . . . .
Vv18g0543 . . . Gorai.009G232500.1 .
. . . . . . . .
. . . . . . .
Vv18g0544 . . . Gorai.009G232400.1 .
. . . . . . . .
. . . . . . .
Vv18g0545 Tc08_g008120 . . . .
Gorai.010G036800.1 . . . . . .

.	.	.	.	.	.	.	.
Vv18g0546	.	.	.	.	.	.	.
.	.	.	.	.	.	.	.
.	.	.	.	.
Vv18g0547	.	.	.	Gorai.009G232300.1	.
.	.	.	.	.	.	.	.
.	.	.	.	.	.	.
Vv18g0548	.	.	.	Gorai.009G232200.1	.
.	.	.	.	.	.	.	.
.	.	.	.	.	.	.
Vv18g0549	Tc08_g000420	.	.	.	.	.
.	.	.	.	.	.	.	.
.	.	.	.	.	.
Vv18g0550	.	.	.	.	.	Gorai.
010G036600.1	Vv0Unrg4518	.	.	.	.	.
.	.	.	.	.	.	.	.
Vv18g0551	.	.	.	.	Gorai.009G279900.1
.	Vv0Unrg4520	.	.	.	.	.
.	.	.	.	.	.	.	.
Vv18g0552	.	.	.	.	Gorai.009G279800.1
.	.	.	.	.	.	.	.
.	.	.	.	.	.	.	.
Vv18g0553	.	.	.	Gorai.009G232000.1	.
Gorai.010G036400.1	.	.	.	.	.
.	.	.	.	.	.	.	.
.
Vv18g0554	.	.	.	.	.	.	.
.	.	.	.	.	.	.	.
.	.	.	.	.
Vv18g0555	.	.	Gorai.005G095200.1	Gorai.
009G231900.1	.	.	Vv0Unrg4521	.	.	.
.	.	.	.	.	.	.	.
.	.
Vv18g0556	Tc08_g002200	.	.	Gorai.009G231800.1
.	Gorai.010G036300.1	.	.	.	.
.	.	.	.	.	.	.	.
.	.
Vv18g0557	.	.	.	.	.	.	.
.	.	.	.	.	.	.	.
.	.	.	.	.
Vv18g0558	.	.	.	.	.	.	.
.	.	.	.	.	.	.	.
.	.	.	.	.
Vv18g0559	.	.	.	.	.	.	.
.	.	.	.	.	.	.	.
.	.	.	.	.
Vv18g0560	.	.	Gorai.005G095300.1	Gorai.
009G231700.1	.	.	.	.	.	.	.
.	.	.	.	.	.	.	.
.

Vv18g0561 . . . . . . .
. . . . . . . .
. . . . .
Vv18g0562 . . . . . . .
. . . . . . . .
. . . . .
Vv18g0563 . . . Gorai.009G231600.1 .
Gorai.010G036000.1 . . . . .
. . . . . . . .
.
Vv18g0564 . . . . . . .
. . . . . . . .
. . . . .
Vv18g0565 Tc08_g007970 . . Gorai.009G231300.1
. Gorai.010G035800.1 . . . .
. . . . . . . .
. .
Vv18g0566 . . . Gorai.009G231100.1 .
Gorai.010G035700.1 . . . . .
. . . . . . . .
.
Vv18g0567 . . Gorai.005G095400.1 Gorai.
009G231000.1 . . . . . . .
. . . . . . . .
.
Vv18g0568 . . . . . . .
. . . . . . . .
. . . . .
Vv18g0569 Tc08_g003700 . Gorai.005G095700.1 .
. . . . . . . .
. . . . . . . .
Vv18g0570 . . . . . . .
. . . . . . . .
. . . . .
Vv18g0571 . . . . . . .
. . . . . . . .
. . . . .
Vv18g0572 Tc08_g001960 . . Gorai.009G230800.1
. Gorai.010G035600.1 . . . .
. . . . . . . .
. .
Vv18g0573 . . . Gorai.009G230700.1 .
. . . . . . . .
. . . . . . .
Vv18g0574 . . . Gorai.009G230600.1
Gorai.009G279500.1 . . . . . .
. . . . . . . .
.
Vv18g0575 . . Gorai.005G096000.1 Gorai.
009G230500.1 . . . . . . .

. . . . . . . .
.
Vv18g0576 . . . . . . .
. . . . . . . .
. . . . .
Vv18g0577 . . . . . . .
. . . . . . . .
. . . . .
Vv18g0578 . . Gorai.005G096300.1 .
Gorai.009G279400.1 . . . . . .
. . . . . . . .
.
Vv18g0579 . . Gorai.005G096500.1 Gorai.
009G230400.1 . . . . . . .
. . . . . . . .
.
Vv18g0580 . . . . . Gorai.
010G035500.1 Vv05g0313 Tc04_g001060 . . .
. . . . . . . .
.
Vv18g0581 . . Gorai.005G096600.1 . .
Gorai.010G035400.1 . . . . .
. . . . . . . .
.
Vv18g0582 . . Gorai.005G096700.1 Gorai.
009G230300.1 . Gorai.010G035300.1 . . .
. . . . . . . .
. . .
Vv18g0583 . . . . . . .
. . . . . . . .
. . . . .
Vv18g0584 . . . Gorai.009G230100.1 .
. . . . . . . .
. . . . . . . .
Vv18g0585 Tc08_g000200 . . Gorai.009G230000.1
. . . . . . . .
. . . . . . . .
Vv18g0586 . . . . . . .
. . . . . . . .
. . . . .
Vv18g0587 . . . . . . .
. . . . . . . .
. . . . .
Vv18g0588 . . Gorai.005G096800.1 Gorai.
009G229800.1 . Gorai.010G035100.1 . . .
. . . . . . . .
. . .
Vv18g0589 . . . . . Gorai.
010G035000.1 Vv05g0297 . . . . .
. . . . . . . .

Vv18g0590 . . . . . . .
. . . . . . . .
. . . . .
Vv18g0591 . . . . . . .
. . . . . . . .
. . . . .
Vv18g0592 . . . . . . .
. . . . . . . .
. . . . .
Vv18g0593 . . . . . Gorai.
010G034900.1 . . . . . . .
. . . . . . .
Vv18g0594 . . . . . .
Vv05g0261 Tc04_g030360 . . . . .
. . . . . . .
Vv18g0595 . . . . . Gorai.
010G034700.1 . . . . . . .
. . . . . . .
Vv18g0596 . . . . . . .
. . . . . . . .
. . . . .
Vv18g0597 . . . . . Gorai.
010G034600.1 . . . . . . .
. . . . . . .
Vv18g0598 . . . . Gorai.009G279300.1
Gorai.010G034500.1 . . . . .
. . . . . . . .
.
Vv18g0599 . . . . . . .
. . . . . . . .
. . . . .
Vv18g0600 Tc08_g003720 . . Gorai.009G229300.1
. . . . . . . .
. . . . . . . .
Vv18g0601 . . . . . . .
. . . . . . . .
. . . . .
Vv18g0602 . . . . . . .
. . . . . . . .
. . . . .
Vv18g0603 . . . . . . .
. . . . . . . .
. . . . .
Vv18g0604 . . . . . . .
. . . . . . . .
. . . . .
Vv18g0605 . . . Gorai.009G229100.1 .
Gorai.010G034400.1 . . . . .
. . . . . . . .
.

Vv18g0606 . . . . . Gorai.
010G034200.1 . . . . . . .
. . . . . . .
Vv18g0607 . . . . . . .
. . . . . . . .
. . . . .
Vv18g0608 . . . . . . .
. . . . . . . .
. . . . .
Vv18g0609 . . Gorai.005G097000.1 . .
Gorai.010G033800.1 . . . . .
. . . . . . . .
.
Vv18g0610 . . . . . Gorai.
010G033700.1 . . . . . . .
. . . . . . .
Vv18g0611 . . . . . . .
. . . . . . . .
. . . . .
Vv18g0612 . . . . . . .
. . . . . . . .
. . . . .
Vv18g0613 . . Gorai.005G097100.1 .
Gorai.009G279200.1 Gorai.010G033400.1 . . .
. . . . . . . .
. . .
Vv18g0614 . . . . . . .
. . . . . . . .
. . . . .
Vv18g0615 . . . . . . .
. . . . . . . .
. . . . .
Vv18g0616 . . . Gorai.009G228700.1 .
. . . . . . . .
. . . . . . .
Vv18g0617 . . . . Gorai.009G279000.1
. . . . . . . .
. . . . . . .
Vv18g0618 . . . Gorai.009G228600.1 .
. . . . . . . .
. . . . . . .
Vv18g0619 . . . Gorai.009G228500.1 .
. . . . . . . .
. . . . . . .
Vv18g0620 . . . . . Gorai.
010G033300.1 . . . . . . .
. . . . . . .
Vv18g0621 . . . . . . .
. . . . . . . .
. . . . .

Vv18g0622 Tc08_g003800 . . . . .
. . . . . . . .
. . . . . .
Vv18g0623 . . . . Gorai.009G278900.1
. Vv05g0227 . Gorai.006G265700.1 .
. . . . . . . .
. .
Vv18g0624 . . . Gorai.009G228200.1 .
Gorai.010G033100.1 . . . . .
. . . . . . . .
.
Vv18g0625 . . . . . . .
. . . . . . . .
. . . . .
Vv18g0626 . . . Gorai.009G228000.1
Gorai.009G278800.1 . . . . . .
. . . . . . . .
.
Vv18g0627 . . . . . . .
. . . . . . . .
. . . . .
Vv18g0628 . . . Gorai.009G227800.1 .
Gorai.010G033000.1 . . . . .
. . . . . . . .
.
Vv18g0629 . . . . . . .
. . . . . . . .
. . . . .
Vv18g0630 . . . . . . .
. . . . . . . .
. . . . .
Vv18g0631 . . . Gorai.009G227600.1 .
Gorai.010G032800.1 . . . . .
. . . . . . . .
.
Vv18g0632 Tc08_g002950 . . . . .
Vv03g0892 Tc02_g002320 . Gorai.005G263100.1
. . Gorai.012G163200.1 . . .
. . . .
Vv18g0633 . . . . . . .
. . . . . . . .
. . . . .
Vv18g0634 . . . . . . .
. . . . . . . .
. . . . .
Vv18g0635 . . . Gorai.009G227200.1 .
. . . . . . . .
. . . . . . .
Vv18g0636 . . . Gorai.009G227100.1 .
. . . . . . . .

```
        .       .       .       .       .       .       .
Vv18g0637       .       .       .       .       .       .       .
        .       .       .       .       .       .       .       .
        .       .       .       .       .
Vv18g0638       .       .       .       Gorai.009G227000.1      .
        .       .       .       .       .       .       .       .
        .       .       .       .       .       .       .
Vv18g0639       .       .       Gorai.005G097200.1      .       .
        .       .       .       .       .       .       .       .
        .       .       .       .       .       .       .
Vv18g0640       .       .       .       .       .       .       .
        .       .       .       .       .       .       .       .
        .       .       .       .       .
Vv18g0641       Tc08_g004170    .       .       .       .       .
        .       .       .       .       .       .       .       .
        .       .       .       .       .       .
Vv18g0642       .       .       .       .       .       .       .
        .       .       .       .       .       .       .       .
        .       .       .       .       .
Vv18g0643       .       .       .       Gorai.009G226800.1
Gorai.009G278600.1      .       .       .       .       .       .
        .       .       .       .       .       .       .       .
        .
Vv18g0644       .       .       .       Gorai.009G226700.1      .
        .       .       .       .       .       .       .       .
        .       .       .       .       .       .       .
Vv18g0645       .       .       .       Gorai.009G226400.1      .
        Gorai.010G031900.1      .       .       .       .       .
        .       .       .       .       .       .       .       .
        .
Vv18g0646       .       .       .       .       .       .       .
        .       .       .       .       .       .       .       .
        .       .       .       .       .
Vv18g0647       .       .       .       Gorai.009G226200.1      .
        Gorai.010G031800.1      .       .       .       .       .
        .       .       .       .       .       .       .       .
        .
Vv18g0648       .       .       .       .       .       .       .
        .       .       .       .       .       .       .       .
        .       .       .       .       .
Vv18g0649       .       .       .       Gorai.009G226000.1      .
        Gorai.010G031600.1      .       .       .       .       .
        .       .       .       .       .       .       .       .
        .
Vv18g0650       .       .       .       .       .       .       .
        .       .       .       .       .       .       Vv07g0889
        Tc01_g007370    .       .       .       Gorai.003G035600.1
        .
Vv18g0651       .       .       .       .       .       .       .
        .       .       .       .       .       .       .       .
```

. . . . .
Vv18g0652 . . . Gorai.009G225900.1 .
. . . . . . . .
. . . . . . .
Vv18g0653 . . . . Gorai.009G278400.1
Gorai.010G031300.1 . . . . .
. . . . . . . .
.
Vv18g0654 . . . . . . .
. . . . . . . .
. . . . .
Vv18g0655 . . . Gorai.009G225700.1 .
. . . . . . . .
. . . . . . .
Vv18g0656 . . Gorai.005G097300.1 . .
Gorai.010G031000.1 . . . . .
. . . . . . . .
.
Vv18g0657 . . . . . . .
. . . . . . . .
. . . . .
Vv18g0658 Tc08_g014580 . . . . .
. . . . . . . .
. . . . . .
Vv18g0659 . . . . . . .
. . . . . . . .
. . . . .
Vv18g0660 . . . . . . .
. . . . . . . .
. . . . .
Vv18g0661 . . . . . . .
. . . . . . . .
. . . . .
Vv18g0662 . . . Gorai.009G225500.1 .
Gorai.010G030700.1 . . . . .
. . . . . . . .
.
Vv18g0663 . . . . . . .
. . . . . . . .
. . . . .
Vv18g0664 Tc08_g003050 . . . . .
. . . . . . . .
. . . . . .
Vv18g0665 . . Gorai.005G097800.1 Gorai.
009G224800.1 . . . . . . .
. . Vv07g0912 Tc01_g007180 .
Gorai.008G087400.1 . . .
Vv18g0666 . . . Gorai.009G224700.1 .
. . . . . . . .
. . . . . . .

Vv18g0667 . . . . . . .
. . . . . . . .
. . . . .
Vv18g0668 Tc08_g004570 . . Gorai.009G224500.1
Gorai.009G278100.1 Gorai.010G029800.1 . .
. . . . . . . .
. . . .
Vv18g0669 . . . . . . .
. . . . . . . .
. . . . .
Vv18g0670 . . . . . Gorai.
010G029700.1 . . . . . . .
. . . . . . .
Vv18g0671 . . . . . . .
. . . . . . . .
. . . . .
Vv18g0672 . . . . . .
Vv03g0873 . . . . Gorai.008G019300.1
. . . . . . . .
Vv18g0673 . . . Gorai.009G224300.1
Gorai.009G277900.1 Gorai.010G029600.1 . . .
. . . . . . . .
. . .
Vv18g0674 . . . . . . .
. . . . . . . .
. . . . .
Vv18g0675 . . . Gorai.009G224000.1 .
Gorai.010G029500.1 . . . . .
. . . . . . . .
.
Vv18g0676 . . . Gorai.009G223900.1 .
Gorai.010G029400.1 . . . . .
. . . . . . . .
.
Vv18g0677 . . . . . . .
. . . . . . Vv07g0917
Tc01_g007120 Gorai.007G057500.1 Gorai.008G087000.1
Gorai.007G228200.1 Gorai.003G033800.1 .
Vv18g0678 . . Gorai.005G098000.1 Gorai.
009G223700.1 . . . . . . .
. . . . . . . .
.
Vv18g0679 . . . Gorai.009G223500.1 .
Gorai.010G029100.1 . . . . .
. . . . . . . .
.
Vv18g0680 . . Gorai.005G098100.1 Gorai.
009G223200.1 . Gorai.010G029000.1 . . .
. . . . . . . .
. . .

Vv18g0681 . . . . . . .
. . . . . . . .
. . . . .
Vv18g0682 . . Gorai.005G098200.1 Gorai.
009G223100.1 . . . . . . .
. . . . . . . .
.
Vv18g0683 . . . . Gorai.009G277800.1
. . . . . . . .
Vv07g0924 Tc01_g007050 . Gorai.008G086600.1
Gorai.007G227600.1 . .
Vv18g0684 . . . . . . .
. . . . . . . .
. . . . .
Vv18g0685 . . Gorai.005G098300.1 Gorai.
009G223000.1 . Gorai.010G028900.1 . . .
. . . . . . . .
. . .
Vv18g0686 . . . . . . .
. . . . . . . .
. . . . .
Vv18g0687 Tc08_g014670 . . . . .
. . . . . . . .
. . . . . .
Vv18g0688 . . . . . . .
. . . . . . . .
. . . . .
Vv18g0689 . . . Gorai.009G222800.1 .
Gorai.010G028700.1 . . . . .
. . . . . . . .
.
Vv18g0690 . . . . . . .
. . . . . . . .
. . . . .
Vv18g0691 . . . . . . .
. . . . . . . .
. . . . .
Vv18g0692 . . . Gorai.009G222700.1
Gorai.009G277700.1 . . . . . .
. . . . . . . .
.
Vv18g0693 . . . Gorai.009G222600.1 .
. . . . . . . .
Vv07g0926 Tc01_g007010 . . Gorai.
007G227300.1 Gorai.003G032800.1 .
Vv18g0694 . . . Gorai.009G222500.1 .
Gorai.010G028600.1 Vv03g0871 . . .
. . . . . . . .
. .
Vv18g0695 . . . Gorai.009G222400.1 .

Gorai.010G028500.1 . . . . .
. . Vv07g0933 Tc01_g006960 . .
Gorai.007G226900.1 Gorai.003G032500.1 .
Vv18g0696 . . Gorai.005G098400.1 . .
. . . . . . . .
. . . . . . .
Vv18g0697 . . Gorai.005G098500.1 . .
Gorai.010G028400.1 . . . . .
. . . . . . . .
.
Vv18g0698 . . Gorai.005G098600.1 Gorai.
009G222300.1 Gorai.009G277600.1 Gorai.010G028300.1 .
. . . . . . . .
. . . . .
Vv18g0699 Tc08_g015140 . . . .
Gorai.010G028200.1 . . . . . .
. . . . . . . .
Vv18g0700 . . . . . Gorai.
010G028100.1 . . . . . . .
. . . . . . .
Vv18g0701 . . . . . . .
. . . . . . . .
. . . . .
Vv18g0702 . . . . . Gorai.
010G028000.1 . . . . . . .
. . . . . . .
Vv18g0703 . . . . . Gorai.
010G027900.1 . . . . . . .
. . . . . . .
Vv18g0704 . . Gorai.005G098700.1 Gorai.
009G222200.1 Gorai.009G277500.1 Gorai.010G027700.1 .
. . . . . . . .
. . . . .
Vv18g0705 . . . . . Gorai.
010G027200.1 . . . . . . .
. . . . . . .
Vv18g0706 . . . . . . .
. . . . . . . .
. . . . .
Vv18g0707 . . . . . . .
. . . . . . . .
. . . . .
Vv18g0708 . . . . . . .
. . . . . . . .
. . . . .
Vv18g0709 . . . . . . .
. . . . . . . .
. . . . .
Vv18g0710 . . . Gorai.009G221700.1 .
Gorai.010G026900.1 . . . . .

. . . . . . . .
.
Vv18g0711 . . . . . . .
. . . . . . . .
. . . . .
Vv18g0712 . . . . . .
Vv03g0863 . . . . . . .
. . . . . .
Vv18g0713 . . . . . Gorai.
010G026600.1 . . . . . . .
Vv07g0939 Tc01_g006800 . . Gorai.
007G225900.1 . .
Vv18g0714 . . . Gorai.009G221400.1 .
Gorai.010G026500.1 . . . . .
. . Vv07g0941 Tc01_g006780 . .
Gorai.007G225700.1 Gorai.003G031700.1 .
Vv18g0715 . . . . Gorai.009G277400.1
Gorai.010G026400.1 . . . . .
. . Vv07g0944 Tc01_g006750 .
Gorai.008G084500.1 Gorai.007G187800.1 . .
Vv18g0716 . . . Gorai.009G221300.1 .
. . . . . . . .
. . . . . . .
Vv18g0717 . . . . . . .
. . . . . . . .
. . . . .
Vv18g0718 . . . . . . .
. . . . . . . .
. . . . .
Vv18g0719 . . . . . . .
. . . . . . . .
. . . . .
Vv18g0720 . . . Gorai.009G221100.1 .
. . . . . . . .
. . . . . . .
Vv18g0721 . . . . . .
Vv03g0827 . . . . . . .
. . . . . .
Vv18g0722 . . . Gorai.009G220900.1 .
. . . . . . . .
. . . . . . .
Vv18g0723 . . . . . . .
. . . . . . . .
. . . . .
Vv18g0724 . . . . . . .
. . . . . . . .
. . . . .
Vv18g0725 . . . . . . .
. . . . . . Vv07g0949
Tc01_g006690 Gorai.007G056700.1 . Gorai.

007G224700.1 Gorai.003G031300.1 .
Vv18g0726 . . . . . . .
. . . . . . . .
. . . . .
Vv18g0727 . . . . . .
Vv03g0814 . . Gorai.005G230600.1 .
Gorai.008G019900.1 . . . . . .
. .
Vv18g0728 . . . . . . .
. . . . . . . .
. . . . .
Vv18g0729 . . . . . . .
. . . . . . . .
. . . . .
Vv18g0730 . . . Gorai.009G220700.1 .
Gorai.010G026200.1 . . . . .
. . . . . . . .
.
Vv18g0731 . . . Gorai.009G220500.1 .
. . . . . . . .
. . . . . . .
Vv18g0732 . . . Gorai.009G220400.1 .
. . . . . . . .
. . . . . . .
Vv18g0733 . . . . . Gorai.
010G026100.1 Vv03g0810 Tc02_g010330 . Gorai.
005G230900.1 . . Gorai.012G147400.1 . .
. . . . .
Vv18g0734 . . . . . Gorai.
010G026000.1 . . . . . . .
. . . . . . .
Vv18g0735 . . . Gorai.009G220300.1 .
. . . . . . . .
. . . . . . .
Vv18g0736 . . . . . . .
. . . . . . . .
. . . . .
Vv18g0737 . . Gorai.005G099400.1 Gorai.
009G220100.1 . . . . . . .
. . . . . . . .
.
Vv18g0738 . . . Gorai.009G219900.1 .
. . . . . . . .
. . . . . . .
Vv18g0739 . . . Gorai.009G219800.1 .
Gorai.010G025800.1 . . . . .
. . . . . . . .
.
Vv18g0740 . . . Gorai.009G219600.1 .
Gorai.010G025700.1 . . . . .

.	.	.	.	.	.	.	.
.
Vv18g0741	.	.	.	Gorai.009G219500.1	.
Gorai.010G025600.1	.	.	.	.	.
.	.	.	.	.	.	.	.
.
Vv18g0742	.	.	.	.	.	.	.
.	.	.	.	.	.	.	.
.	.	.	.	.
Vv18g0743	.	.	.	.	.	Gorai.
010G025500.1	.	.	.	.	.	.	.
.	.	.	.	.	.	.
Vv18g0744	.	.	.	Gorai.009G219400.1	.
.	.	.	.	.	.	.	.
Vv07g0951	Tc01_g006680	.	.	Gorai.
007G224600.1	Gorai.003G031200.1	.
Vv18g0745	.	.	Gorai.005G099600.1	Gorai.
009G219300.1	Gorai.009G277100.1	.	Vv03g0790
Tc02_g010210	Gorai.013G050400.1	.	.	.	.
.	.	.	.	.	.	.
Vv18g0746	.	.	.	.	.	.	.
.	.	.	.	.	.	.	.
.	.	.	.	.
Vv18g0747	.	.	.	Gorai.009G219200.1	.
.	.	.	.	.	.	.	.
.	.	.	.	.	.	.
Vv18g0748	.	.	.	Gorai.009G219100.1	.
Gorai.010G025400.1	.	.	.	.	.
.	.	.	.	.	.	.	.
.
Vv18g0749	.	.	.	Gorai.009G219000.1	.
Gorai.010G025300.1	.	.	.	.	.
.	.	.	.	.	.	.	.
.
Vv18g0750	.	Gorai.002G132600.1	.	Gorai.
009G218900.1	.	Gorai.010G025200.1	.	.	.
.	.	.	.	.	.	.	.
.	.	.
Vv18g0751	.	.	.	Gorai.009G218800.1	.
Gorai.010G025100.1	Vv03g0778	Tc02_g010100	.
Gorai.005G232500.1	.	.	.	Vv07g0982
Tc01_g006300	Gorai.007G056300.1	Gorai.008G081100.1
Gorai.007G222000.1	.	.
Vv18g0752	.	.	Gorai.005G099700.1	Gorai.
009G218600.1	.	Gorai.010G025000.1	Vv03g0777
Tc02_g010090	Gorai.013G049900.1	Gorai.005G232600.1	.
.	.	.	.	.	.	.	.
.
Vv18g0753	.	.	.	.	.	.	.
.	.	.	.	.	.	.	.

. . . . .
Vv18g0754 . . Gorai.005G099800.1 . .
. . . . . . . .
. . . . . . .
Vv18g0755 . . . Gorai.009G218300.1 .
. Vv03g0771 Tc02_g010000 Gorai.013G049500.1
. . . . . . . .
. . .
Vv18g0756 . . . . . . .
. . . . . . . .
. . . . .
Vv18g0757 . Gorai.002G132700.1 . .
Gorai.009G277000.1 . . . . . .
. . . . . . . .
.
Vv18g0758 Tc08_g004170 . . . . .
. . . . . . . .
. . . . . .
Vv18g0759 . . . Gorai.009G218200.1
Gorai.009G274000.1 Gorai.010G024800.1 . . .
. . . . Vv07g0991
Tc01_g006230 . Gorai.008G080400.1 . Gorai.
003G028800.1 .
Vv18g0760 . . . . . Gorai.
010G024700.1 . . . . . . .
Vv07g0992 Tc01_g006220 . Gorai.008G080300.1
Gorai.007G221500.1 . .
Vv18g0761 Tc08_g004000 . . . . .
. . . . . . . .
. . . . . .
Vv18g0762 . . . Gorai.009G217900.1 .
. . . . . . . .
. . . . . . .
Vv18g0763 . . . . . . .
. . . . . . . .
. . . . .
Vv18g0764 . . Gorai.005G100000.1 Gorai.
009G217800.1 . . . . . . .
. . . . . . . .
.
Vv18g0765 . . . . . .
Vv03g0759 Tc02_g009910 Gorai.013G049000.1 Gorai.
005G233000.1 . . . Vv07g1000
Tc01_g006140 . . Gorai.007G190900.1 . .
Vv18g0766 . . Gorai.005G100100.1 Gorai.
009G217700.1 . . . . . . .
. . . . . . . .
.
Vv18g0767 . . . . . . .
. . . . . . . .

. . . . .
Vv18g0768 . . Gorai.005G100200.1 Gorai.
009G217600.1 . . . . . . .
. . . . . . . .
.
Vv18g0769 . . . . . . .
. . . . . . . .
. . . . .
Vv18g0770 . . . . . Gorai.
010G024500.1 . . . . . . .
Vv07g1003 Tc01_g006100 . . Gorai.
007G220600.1 . .
Vv18g0771 . Gorai.002G132800.1 . . .
Gorai.010G024300.1 . . . . .
. . . . . . . .
.
Vv18g0772 . . . . . . .
. . . . . . . .
. . . . .
Vv18g0773 . Gorai.002G132900.1 . Gorai.
009G217300.1 . . . . . . .
. . Vv07g1009 Tc01_g006030 Gorai.
007G056000.1 . Gorai.007G191200.1 Gorai.003G027900.1
.
Vv18g0774 . . Gorai.005G100300.1 Gorai.
009G217200.1 . . . . . . .
. . . . . . . .
.
Vv18g0775 . . . Gorai.009G217100.1 .
. . . . . . . .
Vv07g1011 Tc01_g006010 . Gorai.008G079600.1
Gorai.007G219700.1 Gorai.003G027800.1 .
Vv18g0776 Tc08_g001370 . . . . .
. . . . . . . .
. . . . . .
Vv18g0777 Tc08_g001360 . . . . .
. . . . . . . .
. . . . . .
Vv18g0778 Tc08_g001340 . . . . .
. . . . . . . .
. . . . . .
Vv18g0779 . . Gorai.005G100500.1 . .
. . . . . . . .
Vv07g1012 Tc01_g006000 . . .
Gorai.003G027700.1 .
Vv18g0780 . . . . . . .
. . . . . . . .
. . . . .
Vv18g0781 . . . . . . .
. . . . . . Vv07g1013

Tc01_g005980 . . Gorai.007G219600.1 .
.
Vv18g0782 . . Gorai.005G100600.1 Gorai.
009G216900.1 . . . . . . .
. . . . . . . .
.
Vv18g0783 . . . . . . .
. . . . . . . .
. . . . .
Vv18g0784 . . . . . . .
. . . . . . . .
. . . . .
Vv18g0785 . . . . . . .
. . . . . . . .
. . . . .
Vv18g0786 . . . . . . .
. . . . . . . .
. . . . .
Vv18g0787 . . . . . . .
. . . . . . . .
. . . . .
Vv18g0788 . . . Gorai.009G216800.1 .
Gorai.010G024100.1 Vv03g0725 Tc02_g009300
Gorai.013G046400.1 Gorai.005G235600.1 . .
Gorai.012G149300.1 . . . . . .
.
Vv18g0789 . . . Gorai.009G216700.1 .
Gorai.010G024000.1 . . . . .
. . . . . . . .
.
Vv18g0790 . . . . . . .
. . . . . . . .
. . . . .
Vv18g0791 . . . . . . .
. . . . . . . .
. . . . .
Vv18g0792 Tc08_g001300 . . . . .
. . . . . . . .
. . . . . .
Vv18g0793 . . . . . . .
. . . . . . . .
. . . . .
Vv18g0794 Tc08_g001290 . . . . .
. . . . . . . .
. . . . . .
Vv18g0795 . . . . . . .
. . . . . . . .
. . . . .
Vv18g0796 . . . . . . .
. . . . . . . .

. . . . .
Vv18g0797 . . . . . . .
. . . . . . Vv07g1054
Tc01_g005750 . . . . .
Vv18g0798 . . . . . . .
. . . . . . Vv07g1056
. Gorai.007G055700.1 . . . .
Vv18g0799 . . . . . . .
. . . . . . . .
. . . . .
Vv18g0800 . . . . . . .
. . . . . . . .
. . . . .
Vv18g0801 . . . . . . .
. . . . . . . .
. . . . .
Vv18g0802 . . . . . . .
. . . . . . . .
. . . . .
Vv18g0803 . . . . . . .
. . . . . . . .
. . . . .
Vv18g0804 . . . . . . .
. . . . . . . .
. . . . .
Vv18g0805 . . . . . . .
. . . . . . . .
. . . . .
Vv18g0806 . . . . . . .
. . . . . . . .
. . . . .
Vv18g0807 . . . . . . .
. . . . . . . .
. . . . .
Vv18g0808 . . . . . . .
. . . . . . . .
. . . . .
Vv18g0809 . . . . . . .
. . . . . . . .
. . . . .
Vv18g0810 Tc08_g001040 . . . Gorai.
009G275100.1 . . . . . . .
. . . . . . . .
Vv18g0811 . . . . . Gorai.
010G023400.1 . . . . . . .
. . . . . . .
Vv18g0812 . . . Gorai.009G215800.1 .
Gorai.010G023300.1 . . . . .
. . . . . . . .
.

Vv18g0813 . . . . . Gorai.
010G023200.1 . . . . . . .
. . . . . . .
Vv18g0814 . . . . . . .
. . . . . . . .
. . . . .
Vv18g0815 . . Gorai.005G101000.1 Gorai.
009G215700.1 . Gorai.010G023000.1 . . .
. . . . . . . .
. . .
Vv18g0816 . Gorai.002G133500.1 Gorai.005G101100.1
Gorai.009G215600.1 . . . . .
. . . . Vv07g1065
Tc01_g005650 . . . Gorai.003G027100.1
Gorai.011G106700.1
Vv18g0817 . . Gorai.005G101200.1 Gorai.
009G215500.1 . . . . . . .
. . . . . . . .
.
Vv18g0818 . Gorai.002G133700.1 . Gorai.
009G215400.1 . Gorai.010G022900.1 . . .
. . . . . . . .
. . .
Vv18g0819 . . . Gorai.009G215300.1 .
. . . . . . . .
. . . . . . .
Vv18g0820 . . . Gorai.009G215200.1 .
Gorai.010G022800.1 Vv03g0724 Tc02_g009280
Gorai.013G046300.1 Gorai.005G235700.1 Gorai.002G008800.1
. . . . . . . .
.
Vv18g0821 . . . Gorai.009G215100.1 .
. . . . . . . .
. . . . . . .
Vv18g0822 Tc08_g000020 Gorai.002G133800.1 .
Gorai.009G214900.1 . Gorai.010G022700.1 Vv03g0722
Tc02_g009260 . Gorai.005G235900.1 . .
. Vv07g1072 Tc01_g005600 . . .
. .
Vv18g0823 . . . . . . .
. . . . . . . .
. . . . .
Vv18g0824 . . . . . . .
. . . . . . . .
. . . . .
Vv18g0825 Tc08_g000030 . . Gorai.009G214800.1
. Gorai.010G022600.1 Vv07g1076
Tc01_g005580 . . Gorai.007G216700.1 Gorai.
003G026600.1 . . . . . . .
.

Vv18g0826	Tc08_g000050	.	.	.	.	.
	.	.	.	.	.	.	.	.
	.	.	.	.	.	.
Vv18g0827	Tc08_g000060	.	.	Gorai.009G214700.1
	.	.	.	.	.	.	.	.
	.	.	.	.	.	.	.	.
Vv18g0828	Tc08_g000070	.	.	.	.
Gorai.010G022500.1	.	.	.	.	.	.
	.	.	.	.	.	.	.	.
Vv18g0829	Tc08_g000080	.	.	Gorai.009G214600.1
	.	Gorai.010G022400.1	Vv07g1081
Tc01_g005510	.	.	Gorai.007G216400.1	Gorai.
003G026200.1	.	.	.	.	.	.	.
	.
Vv18g0830	.	.	.	.	.	.	.
	.	.	.	.	.	.	.	.
	.	.	.	.	.
Vv18g0831	Tc08_g000090	.	.	Gorai.009G214500.1
	.	.	Vv07g1082	Tc01_g005500	.	.
	Gorai.007G216300.1	.	.	.	.	.
	.	.	.	.
Vv18g0832	Tc08_g000100	.	.	.	.
Gorai.010G022300.1	.	.	.	.	.	.
	.	.	.	.	.	.	.	.
Vv18g0833	Tc08_g000110	.	.	Gorai.009G214400.1
	.	Gorai.010G022200.1	.	.	.	.
	.	.	.	.	.	.	.	.
	.	.
Vv18g0834	Tc08_g000130	Gorai.002G134600.1	.
Gorai.009G214300.1	.	Gorai.010G021900.1	.	.
	.	.	.	.	.	.	.	.
	.	.	.	.
Vv18g0835	Tc08_g000140	Gorai.002G134800.1	Gorai.
005G101400.1	Gorai.009G214200.1	.	.	.	.
	.	.	.	.	.	.	.	.
	.	.	.	.
Vv18g0836	Tc08_g000150	.	.	.	.
Gorai.010G021800.1	.	.	.	.	.	.
	.	.	.	.	.	.	.	.
Vv18g0837	Tc08_g000160	.	.	.	.
Gorai.010G021700.1	Vv07g1097	Tc01_g005380	.	.
	Gorai.007G215600.1	.	.	.	.	.
	.	.	.	.
Vv18g0838	.	Gorai.002G134900.1	Gorai.005G101600.1
	Gorai.009G214100.1	.	.	.	.	.
	.	.	.	.	.	.	.	.
	.	.	.
Vv18g0839	.	.	.	.	.	.	.
	.	.	.	.	.	.	.	.
	.	.	.	.	.

Vv18g0840 Tc08_g000170 . . . .
Gorai.010G021400.1 Vv07g1098 Tc01_g005370 . .
Gorai.007G215500.1 . . . . .
. . . .
Vv18g0841 . . . . . . .
. . . . . . . .
. . . . .
Vv18g0842 . . . . . . .
. . . . . . . .
. . . . .
Vv18g0843 . . . . . Gorai.
010G021300.1 Vv07g1099 . . . Gorai.
007G215400.1 . . . . . . .
. .
Vv18g0844 . . . . . Gorai.
010G021200.1 Vv07g1101 Tc01_g005330 . . .
. . . . . . . .
.
Vv18g0845 Tc08_g000180 . . Gorai.009G214000.1
. . . . . . . .
. . . . . . . .
Vv18g0846 Tc08_g000190 . Gorai.005G101700.1
Gorai.009G213900.1 . . . . . .
. . . . . . . .
. .
Vv18g0847 Tc08_g000200 . . Gorai.009G213800.1
. . . . . . . .
. . . . . . . .
Vv18g0848 . . . . . . .
. . . . . . . .
. . . . .
Vv18g0849 Tc08_g000210 Gorai.002G135000.1 . .
. . . . . . . .
. . . . . . . .
Vv18g0850 Tc08_g000220 . . Gorai.009G213700.1
. . . . . . . .
. . . . . . . .
Vv18g0851 Tc08_g000230 . . Gorai.009G213600.1
. . . . . . . .
. . . . . . . .
Vv18g0852 Tc08_g000240 . . . .
Gorai.010G021100.1 . . . . . .
. . . . . . . .
Vv18g0853 . . . . . . .
. . . . . . . .
. . . . .
Vv18g0854 Tc08_g000250 . . Gorai.009G213500.1
. . . . . . . .
. . . . . . . .
Vv18g0855 . . . . . . .

```
       .       .       .       .       .       .       .       .
       .       .       .       .       .
Vv18g0856      Tc08_g000260    .       .       .       .       .
       .       .       .       .       .       .       .       .
       .       .       .       .       .       .
Vv18g0857      Tc08_g000270    .       .       Gorai.009G213400.1
       .       .       .       .       .       .       .       .
       .       .       .       .       .       .       .       .
Vv18g0858      .       .       .       Gorai.009G213300.1      .
       .       .       .       .       .       .       .       .
       .       .       .       .       .       .       .
Vv18g0859      Tc08_g000280    .       Gorai.005G101900.1      .
       .       Gorai.010G020900.1      Vv07g1120
Tc01_g005200   .       Gorai.008G077100.1      Gorai.007G214000.1
       .       .       .       .       .       .       .       .
       .
Vv18g0860      Tc08_g000300    .       .       Gorai.009G213200.1
       .       .       .       .       .       .       .       .
       .       .       .       .       .       .       .       .
Vv18g0861      Tc08_g000310    Gorai.002G135200.1      Gorai.
005G102000.1   Gorai.009G213100.1      .       Gorai.010G020700.1
       .       .       .       .       .       .       .       .
       .       .       .       .       .       .
Vv18g0862      Tc08_g000320    .       .       .       .
Gorai.010G020600.1     .       .       .       .       .       .
       .       .       .       .       .       .       .       .
Vv18g0863      Tc08_g000330    .       .       Gorai.009G212900.1
       .       Gorai.010G020500.1      .       .       .       .
       .       .       .       .       .       .       .       .
       .       .
Vv18g0864      Tc08_g000340    .       Gorai.005G102200.1      .
       .       .       Vv07g1127       Tc01_g005120    Gorai.
007G054400.1   .       .       .       .       .       .       .
       .       .       .       .
Vv18g0865      Tc08_g000350    .       .       Gorai.009G212800.1
       .       Gorai.010G020400.1      Vv07g1130
Tc01_g005070   .       Gorai.008G076600.1      .       .       .
       .       .       .       .       .       .       .
Vv18g0866      Tc08_g000360    .       .       .       .       .
       .       .       .       .       .       .       .       .
       .       .       .       .       .       .
Vv18g0867      .       .       .       .       .       Gorai.
010G020300.1   .       .       .       .       .       .       .
       .       .       .       .       .       .       .
Vv18g0868      Tc08_g000380    .       .       Gorai.009G212700.1
       .       .       .       .       .       .       .       .
       .       .       .       .       .       .       .       .
Vv18g0869      Tc08_g000390    .       Gorai.005G102300.1
Gorai.009G212600.1     .       .       Vv03g0691
Tc02_g008840   Gorai.013G044500.1      Gorai.005G237400.1      .
```

Gorai.008G024700.1 . . . . .
. . .
Vv18g0870 . . . . . . .
. . . . . . . .
. . . . .
Vv18g0871 . . . . . . .
. . . . . . . .
. . . . .
Vv18g0872 . . . . . . .
. . . . . . . .
. . . . .
Vv18g0873 Tc08_g000400 . Gorai.005G102400.1 .
. Gorai.010G020200.1 . . . .
. . . Vv07g1135 . . .
. . .
Vv18g0874 Tc08_g000410 . . Gorai.009G212500.1
. . . . . . . .
. . . . . . . .
Vv18g0875 Tc08_g000420 . . Gorai.009G212400.1
. Gorai.010G020100.1 . . . .
. . . . . . . .
. .
Vv18g0876 Tc08_g000430 Gorai.002G132500.1 .
Gorai.009G212200.1 . Gorai.010G020000.1 . .
. . . . . . . .
. . . .
Vv18g0877 Tc08_g000440 . . Gorai.009G212100.1
. Gorai.010G019900.1 . . . .
. . . . . . . .
. .
Vv18g0878 . . . . . . .
. . . . . . . .
. . . . .
Vv18g0879 Tc08_g000450 . . Gorai.009G211900.1
. . . . . . . .
. . . . . . . .
Vv18g0880 Tc08_g000460 . . Gorai.009G211800.1
. . . . . . . .
. . . . . . . .
Vv18g0881 Tc08_g000470 Gorai.002G132400.1 .
Gorai.009G211700.1 . Gorai.010G019800.1 . .
. . . . . . . .
. . . .
Vv18g0882 Tc08_g000510 Gorai.002G132200.1 . .
. . . . . . . .
. . . . . . . .
Vv18g0883 Tc08_g000520 . . . .
Gorai.010G019700.1 Vv03g0675 Tc02_g008750 .
Gorai.005G238100.1 Gorai.002G008100.1 . . .
. . . . . .

Vv18g0884 Tc08_g000530 Gorai.002G132100.1 . .
. . . . . . . .
. . . . . . . .
Vv18g0885 Tc08_g000540 Gorai.002G132000.1 .
Gorai.009G211500.1 . Gorai.010G019600.1 . .
. . . . . . . .
. . . .
Vv18g0886 Tc08_g000550 . . Gorai.009G211400.1
. Gorai.010G019500.1 . . . .
. . . . . . . .
. .
Vv18g0887 Tc08_g000560 Gorai.002G131900.1 . .
. . . . . . . .
. . . . . . . .
Vv18g0888 . . . . . . .
. . . . . . . .
. . . . .
Vv18g0889 . . . . . . .
. . . . . . . .
. . . . .
Vv18g0890 . . . . . . .
. . . . . . . .
. . . . .
Vv18g0891 . . . . . . .
. . . . . . . .
. . . . .
Vv18g0892 . . . . . . .
. . . . . . . .
. . . . .
Vv18g0893 . . . . . . .
. . . . . . . .
. . . . .
Vv18g0894 . . Gorai.005G103000.1 . .
. . . . . . . .
. . . . . . .
Vv18g0895 Tc08_g000690 . . Gorai.009G211300.1
. Gorai.010G018400.1 . . . .
. . . . . . . .
. .
Vv18g0896 Tc08_g000700 Gorai.002G131700.1 .
Gorai.009G211200.1 . Gorai.010G018300.1 . .
. . . . . . . .
. . . .
Vv18g0897 Tc08_g000710 . Gorai.005G103200.1
Gorai.009G211100.1 . Gorai.010G018200.1 . .
. . . . . . . .
. . . .
Vv18g0898 Tc08_g000720 Gorai.002G128700.1 . .
. . Vv03g0658 . . Gorai.
005G238600.1 Gorai.002G007600.1 . . Vv07g1148

Tc01_g004850 . . Gorai.007G212100.1 .
.
Vv18g0899 Tc08_g000730 . . Gorai.009G211000.1
. . . . . . . .
. . . . . . . .
Vv18g0900 Tc08_g000740 . . Gorai.009G210900.1
. . Vv03g0656 . . Gorai.
005G238800.1 Gorai.002G007500.1 . . . .
. . . . .
Vv18g0901 . . . . . . .
. . . . . . . .
. . . . .
Vv18g0902 Tc08_g000750 . . . .
Gorai.010G018100.1 . . . . . .
. . . . . . . .
Vv18g0903 Tc08_g000790 . . . .
Gorai.010G017800.1 . . . . . .
. . . . . . . .
Vv18g0904 . . . . . . .
. . . . . . . .
. . . . .
Vv18g0905 Tc08_g000810 . Gorai.005G103700.1
Gorai.009G210700.1 . . Vv03g0649
Tc02_g008630 . Gorai.005G239100.1 . . .
. . . . . . .
Vv18g0906 Tc08_g000820 . Gorai.005G103800.1
Gorai.009G210600.1 . Gorai.010G017700.1 . .
. . . . . . . .
. . . .
Vv18g0907 . . . . . . .
. . . . . . . .
. . . . .
Vv18g0908 . . . . . Gorai.
010G017600.1 . . . . . . .
Vv07g1153 Tc01_g004810 Gorai.007G051000.1 .
Gorai.007G211600.1 . Gorai.011G105000.1
Vv18g0909 Tc08_g000830 . Gorai.005G103900.1
Gorai.009G210500.1 . . . . . .
. . . Vv07g1156 Tc01_g004780
Gorai.007G053800.1 . . Gorai.003G021700.1
Gorai.011G102000.1
Vv18g0910 . . . . . . .
. . . . . . . .
. . . . .
Vv18g0911 . . . . . . .
. . . . . . . .
. . . . .
Vv18g0912 . . . . . . .
. . . . . . . .
. . . . .

Vv18g0913 Tc08_g000840 . . . . .
. . . . . . .
Vv07g1158 Tc01_g004630 . . . Gorai.
003G018700.1 .
Vv18g0914 . . Gorai.005G104200.1 Gorai.
009G210400.1 . . . . . . .
. . . . . . . .
.
Vv18g0915 Tc08_g000850 . . . . .
. . . . . . .
Vv07g1159 Tc01_g004620 . . . . .
Vv18g0916 . . . . . . .
. . . . . . . .
. . . . .
Vv18g0917 . . . Gorai.009G210300.1 .
. . . . . . . .
. . . . . . .
Vv18g0918 . . . . . . .
. . . . . . . .
. . . . .
Vv18g0919 . . . . . . .
. . . . . . . .
. . . . .
Vv18g0920 Tc08_g000860 . . . . .
. . . . . . . .
. . . . . .
Vv18g0921 . . . Gorai.009G210000.1 .
. . . . . . . .
. . . . . . .
Vv18g0922 Tc08_g000870 . . . . .
. . . . . . . .
. . . . . .
Vv18g0923 . . . . . . .
. . . . . . . .
. . . . .
Vv18g0924 Tc08_g000880 . . . . .
. . . . . . . .
. . . . . .
Vv18g0925 . . . . . . .
. . . . . . . .
. . . . .
Vv18g0926 . . . . . . .
. . . . . . . .
. . . . .
Vv18g0927 . . . . . . .
. . . . . . . .
. . . . .
Vv18g0928 Tc08_g000890 . . . . .
. . . . . . . .
. . . . . .

Vv18g0929 . . . . . . .
. . . . . . . .
. . . . .
Vv18g0930 Tc08_g000900 . . . . .
. . . . . . . .
. . . . .
Vv18g0931 Tc08_g000910 . . . Gorai.
009G275400.1 . . . . . . .
. . . . . . . .
Vv18g0932 Tc08_g000920 . . . . .
Vv03g0640 Tc02_g008590 Gorai.013G042400.1 .
. Gorai.008G025300.1 . Vv07g1160 .
. . Gorai.007G210500.1 . .
Vv18g0933 Tc08_g000930 . . Gorai.009G209900.1
. Gorai.010G017500.1 . . . .
. . . . . . . .
. .
Vv18g0934 Tc08_g000960 . . Gorai.009G209600.1
Gorai.009G275200.1 Gorai.010G017300.1 . .
. . . . . . . .
. . . .
Vv18g0935 . . . . . . .
. . . . . . . .
. . . . .
Vv18g0936 Tc08_g000970 . . Gorai.009G209500.1
. . . . . . . .
. . . . . . . .
Vv18g0937 Tc08_g000990 . . . . .
. . . . . . . .
. . . . . .
Vv18g0938 Tc08_g001000 . . Gorai.009G209300.1
. Gorai.010G017000.1 . . . .
. . . . . . . .
. .
Vv18g0939 Tc08_g001010 . Gorai.005G104600.1
Gorai.009G209200.1 . . Vv03g0636
Tc02_g008530 Gorai.013G042200.1 . Gorai.002G007300.1
Gorai.008G025600.1 . . . . .
. . .
Vv18g0940 . . . . . . .
. . . . . . Vv07g0510
Tc01_g031710 . Gorai.008G139300.1 . .
.
Vv18g0941 Tc08_g001020 . . . .
Gorai.010G016900.1 . . . . . .
. . . . . . . .
Vv18g0942 Tc08_g001030 . Gorai.005G104800.1
Gorai.009G209000.1 . . . . . .
. . . . . . . .
. .

Vv18g0943 Tc08_g001040 . . Gorai.009G208900.1
Gorai.009G275100.1 Gorai.010G016800.1 . .
. . . . . . . .
. . . .
Vv18g0944 Tc08_g001050 . . . .
Gorai.010G016700.1 Vv03g0627 Tc02_g008460 Gorai.
013G041800.1 . . . . . . .
. . . .
Vv18g0945 Tc08_g001060 . . . .
Gorai.010G016600.1 . . . . . .
. . . . . . . .
Vv18g0946 Tc08_g001080 . . . . .
. . . . . . . .
. . . . . .
Vv18g0947 . . . . . . .
. . . . . . . .
. . . . .
Vv18g0948 Tc08_g001100 . . Gorai.009G208700.1
. . . . . . . .
. . . . . . . .
Vv18g0949 . . . . . . .
. . . . . . . .
. . . . .
Vv18g0950 Tc08_g001110 . . Gorai.009G208600.1
Gorai.009G275000.1 . . . . .
. . . . . . . .
. .
Vv18g0951 Tc08_g001120 . . Gorai.009G208500.1
. Gorai.010G016200.1 . . . .
. . . . . . . .
. .
Vv18g0952 Tc08_g001140 . . Gorai.009G208400.1
. . . . . . . .
. . . . . . . .
Vv18g0953 Tc08_g001160 . . Gorai.009G208200.1
. . . . . . . .
. . . . . . . .
Vv18g0954 Tc08_g001190 . . Gorai.009G208000.1
. Gorai.010G016100.1 . . . .
. . . . . . . .
. .
Vv18g0955 Tc08_g001200 . . Gorai.009G207900.1
. Gorai.010G016000.1 . . . .
. . . . . . . .
. .
Vv18g0956 Tc08_g001210 . Gorai.005G105200.1
Gorai.009G207800.1 . . Vv03g0587 . .
. . . . . . . .
. . .
Vv18g0957 Tc08_g001220 . . Gorai.009G207500.1

```
        .       Gorai.010G015900.1      .       .       .       .
        .       .       .       .       .       .       .       .
        .       .
Vv18g0958       Tc08_g001230    .       .       .       .       .
        .       .       .       .       .       .       .       .
        .       .       .       .       .       .
Vv18g0959       Tc08_g001240    .       .       .       .
Gorai.010G015800.1      .       .       .       .       .       .
        .       .       .       .       .       .       .       .
Vv18g0960       .       .       .       .       .       .       .
        .       .       .       .       .       .       .       .
        .       .       .       .       .
Vv18g0961       Tc08_g001250    .       Gorai.005G105400.1
Gorai.009G207400.1      .       .       Vv03g0578
Tc02_g008170    .       .       .       .       .       .       .
        .       .       .       .       .
Vv18g0962       Tc08_g001260    .       .       .       .
Gorai.010G015700.1      Vv03g0574       .       Gorai.002G214600.1
        Gorai.005G264100.1      .       .       .       .       .
        .       .       .       .       .
Vv18g0963       Tc08_g001270    .       .       Gorai.009G207300.1
        .       Gorai.010G015600.1      .       .       .       .
        .       .       .       .       .       .       .       .
        .       .
Vv18g0964       .       .       .       .       .       .       .
        .       .       .       .       .       .       .       .
        .       .       .       .       .
Vv18g0965       Tc08_g001290    .       .       .       .
Gorai.010G015400.1      .       .       .       .       .       .
        .       .       .       .       .       .       .       .
Vv18g0966       Tc08_g001300    .       .       .       .       .
        .       .       .       .       .       .       .       .
        .       .       .       .       .       .
Vv18g0967       Tc08_g001310    .       .       Gorai.009G207100.1
        .       Gorai.010G015200.1      .       .       .       .
        .       .       .       .       .       .       .       .
        .       .
Vv18g0968       Tc08_g001320    .       .       .       .
Gorai.010G015000.1      .       .       .       .       .       .
        .       .       .       .       .       .       .       .
Vv18g0969       Tc08_g001330    .       .       Gorai.009G207000.1
        .       Gorai.010G014900.1      .       .       .       .
        .       .       .       .       .       .       .       .
        .       .
Vv18g0970       .       .       .       .       .       .       .
        .       .       .       .       .       .       .       .
        .       .       .       .       .
Vv18g0971       .       .       .       .       .       .       .
        .       .       .       .       .       .       .       .
        .       .       .       .       .
```

```
Vv18g0972       Tc08_g001340    .       .       .       .
Gorai.010G014800.1      .       .       .       .       .       .
        .       .       .       .       .       .       .       .
Vv18g0973       .       .       .       .       .       .       .
        .       .       .       .       .       .       .       .
        .       .       .       .       .
Vv18g0974       Tc08_g001360    .       .       .       .
Gorai.010G014600.1      .       .       .       .       .       .
        .       .       .       .       .       .       .       .
Vv18g0975       Tc08_g001370    .       .       Gorai.009G206700.1
        .       Gorai.010G014500.1      .       .       .       .
        .       .       .       .       .       .       .       .
        .       .
Vv18g0976       .       .       .       .       .       .       .
        .       .       .       .       .       .       .       .
        .       .       .       .       .
Vv18g0977       Tc08_g001380    .       .       .       .
Gorai.010G014400.1      .       .       .       .       .       .
        .       .       .       .       .       .       .       .
Vv18g0978       Tc08_g001390    .       .       Gorai.009G206600.1
        .       Gorai.010G014300.1      .       .       .       .
        .       .       .       .       .       .       .       .
        .       .
Vv18g0979       .       .       .       .       .       .       .
        .       .       .       .       .       .       .       .
        .       .       .       .       .
Vv18g0980       Tc08_g001400    .       .       .       .
Gorai.010G014200.1      .       .       .       .       .       .
        .       .       .       .       .       .       .       .
Vv18g0981       Tc08_g001410    .       .       Gorai.009G206500.1
        Gorai.009G274900.1      .       .       .       .       .
        .       .       .       .       .       .       .       .
        .       .
Vv18g0982       .       .       .       .       .       .       .
        .       .       .       .       .       .       .       .
        .       .       .       .       .
Vv18g0983       Tc08_g001420    .       .       .       .
Gorai.010G014100.1      .       .       .       .       .       .
        .       .       .       .       .       .       .       .
Vv18g0984       Tc08_g001430    .       Gorai.005G105700.1
Gorai.009G206400.1      Gorai.009G274800.1      Gorai.010G014000.1
        .       .       .       .       .       .       .       .
        .       .       .       .       .       .
Vv18g0985       .       .       .       .       .       .       .
        .       .       .       .       .       .       .       .
        .       .       .       .       .
Vv18g0986       Tc08_g001450    .       .       Gorai.009G206300.1
        .       Gorai.010G013800.1      .       .       .       .
        .       .       .       .       .       .       .       .
        .       .
```

Vv18g0987 Tc08_g001460 . Gorai.005G105800.1 .
Gorai.009G274700.1 Gorai.010G013600.1 . .
. . . . . . . .
. . . .
Vv18g0988 . . . . . . .
. . . . . . . .
. . . . .
Vv18g0989 Tc08_g001470 . . Gorai.009G206200.1
. Gorai.010G013500.1 . . . .
. . . . . . . .
. .
Vv18g0990 . . . . . . .
. . . . . . . .
. . . . .
Vv18g0991 . . . . . . .
. . . . . . . .
. . . . .
Vv18g0992 . . . . . . .
. . . . . . . .
. . . . .
Vv18g0993 Tc08_g001490 . . . .
Gorai.010G013400.1 . . . . . .
. . . . . . . .
Vv18g0994 . . . . . . .
. . . . . . . .
. . . . .
Vv18g0995 Tc08_g001510 . . Gorai.009G205900.1
Gorai.009G274500.1 . . . . .
. . . . . . . .
. .
Vv18g0996 . . . . . . .
. . . . . . . .
. . . . .
Vv18g0997 Tc08_g001530 . . Gorai.009G205800.1
. Gorai.010G013300.1 . . . .
. . . . . . . .
. .
Vv18g0998 Tc08_g001540 . . . . .
Vv03g0526 . Gorai.013G049300.1 Gorai.
005G263200.1 . . Gorai.012G163300.1 . .
. . . . .
Vv18g0999 Tc08_g001550 . . . .
Gorai.010G013000.1 . . . . . .
. . . . . . . .
Vv18g1000 Tc08_g001560 . . . . .
. . . . . . . .
. . . . . .
Vv18g1001 Tc08_g001570 . . . Gorai.
009G274400.1 . . . . . . .
. . . . . . . .

Vv18g1002	Tc08_g001580	.	.	Gorai.009G205700.1
	.	Gorai.010G012900.1	.	.	.	.
	.	.	.	.	.	.	.	.
	.	.
Vv18g1003	.	.	.	.	.	.	.
	.	.	.	.	.	.	Vv04g1021
	Tc01_g003880	.	Gorai.008G070800.1	Gorai.
007G207200.1	Gorai.003G018100.1	.
Vv18g1004	.	.	.	.	.	.	.
	.	.	.	.	.	.	.	.
	.	.	.	.	.
Vv18g1005	Tc08_g001590	Gorai.002G129900.1	.
Gorai.009G205600.1	.	.	.	.	.	.
	.	.	.	Vv04g1023	.	.
Gorai.008G070600.1	.	.	.
Vv18g1006	Tc08_g001600	.	.	Gorai.009G205500.1
	.	.	.	.	.	.	.	.
	.	.	.	.	.	.	.	.
Vv18g1007	Tc08_g001620	.	.	Gorai.009G205400.1
	.	.	.	.	.	.	.	.
	.	.	.	.	.	.	.	.
Vv18g1008	Tc08_g001630	Gorai.002G129800.1	Gorai.
005G106100.1	Gorai.009G205300.1	Gorai.009G274300.1
Gorai.010G012700.1	.	.	.	.	.	.
	.	Vv04g1035	Tc01_g003810	.	Gorai.
008G070000.1	Gorai.007G207000.1	Gorai.003G017900.1	.
Vv18g1009	.	.	.	.	.	.	.
	.	.	.	.	.	.	.	.
	.	.	.	.	.
Vv18g1010	.	.	.	.	.	.	.
	.	.	.	.	.	.	.	.
	.	.	.	.	.
Vv18g1011	.	.	.	.	.	.	.
	.	.	.	.	.	.	.	.
	.	.	.	.	.
Vv18g1012	.	.	.	.	.	.	.
	.	.	.	.	.	.	.	.
	.	.	.	.	.
Vv18g1013	Tc08_g001640	Gorai.002G129700.1	.	.
	.	Gorai.010G012500.1	.	.	.	.
	.	.	.	.	.	.	.	.
	.	.
Vv18g1014	Tc08_g001650	.	Gorai.005G106300.1	.
	.	Gorai.010G012400.1	.	.	.	.
	.	.	.	Vv04g1037	Tc01_g003790	.
	.	Gorai.007G206900.1	Gorai.003G017700.1	.
Vv18g1015	Tc08_g001660	.	.	.	.
Gorai.010G012300.1	Vv03g0519	.	Gorai.013G052400.1
	.	.	.	.	.	.	.	.
	.	.	.

Vv18g1016 . . . . . . .
. . . . . . . .
. . . . .
Vv18g1017 Tc08_g001670 . Gorai.005G106400.1
Gorai.009G205200.1 Gorai.009G274200.1 . . .
. . . . . . . .
. . . .
Vv18g1018 Tc08_g001680 . . Gorai.009G205100.1
Gorai.009G274100.1 Gorai.010G012200.1 . .
. . . . . . . .
. . . .
Vv18g1019 Tc08_g001690 . . Gorai.009G205000.1
. . . . . . . .
. Vv04g1038 Tc01_g003770 . .
Gorai.007G206800.1 Gorai.003G017600.1 Gorai.011G103100.1
Vv18g1020 Tc08_g001700 . Gorai.005G106500.1
Gorai.009G204900.1 . Gorai.010G012100.1 . .
. . . . . Vv04g1040 .
. . . . .
Vv18g1021 Tc08_g001710 . . Gorai.009G204700.1
. Gorai.010G012000.1 . . . .
. . . . . . . .
. .
Vv18g1022 . . . . . Gorai.
010G011900.1 . . . . . . .
. . . . . . .
Vv18g1023 . . . . . . .
. . . . . . Vv04g1043
Tc01_g003740 Gorai.007G051300.1 . Gorai.
007G206700.1 . Gorai.011G102800.1
Vv18g1024 Tc08_g001720 . . . . .
. . . . . . . .
. . . . . .
Vv18g1025 Tc08_g001730 . Gorai.005G107000.1
Gorai.009G204600.1 Gorai.009G274000.1 . . .
. . . . . Vv04g1047 .
. . . . .
Vv18g1026 Tc08_g001740 . . Gorai.009G204500.1
. . . . . . . .
. . . . . . . .
Vv18g1027 Tc08_g012960 . . . . .
. . . . . . . .
. . . . . .
Vv18g1028 Tc08_g001750 . . . . .
. . . . . . . .
. . . . . .
Vv18g1029 . . . . . . .
. . . . . . . .
. . . . .
Vv18g1030 . . . . . . .

. . . . . . . .
. . . . .
Vv18g1031 . . . Gorai.009G204400.1 .
. . . . . . . .
. . . . . . .
Vv18g1032 . . . . . . .
. . . . . . . .
. . . . .
Vv18g1033 . . . . . . .
. . . . . . . .
. . . . .
Vv18g1034 . . . . . . .
. . . . . . . .
. . . . .
Vv18g1035 Tc08_g001760 Gorai.002G129600.1 . .
Gorai.009G273900.1 Gorai.010G011800.1 . .
. . . . . . . .
. . . .
Vv18g1036 Tc08_g001770 Gorai.002G129500.1 .
Gorai.009G204300.1 . Gorai.010G011700.1 Vv03g0511
. Gorai.013G052800.1 . . Gorai.
008G018900.1 . . . . . . .
.
Vv18g1037 . . . . . . .
. . . . . . . .
. . . . .
Vv18g1038 . . . . . . .
. . . . . . . .
. . . . .
Vv18g1039 . . . . . . .
. . . . . . . .
. . . . .
Vv18g1040 . . . . . . .
. . . . . . Vv04g1059
Tc01_g003580 . Gorai.008G068900.1 Gorai.
007G205700.1 . .
Vv18g1041 Tc08_g001790 . . . .
Gorai.010G011500.1 Vv03g0510 . . . .
. Gorai.012G146400.1 . . . .
. . .
Vv18g1042 Tc08_g001800 Gorai.002G129300.1 .
Gorai.009G204100.1 . . . . . .
. . . Vv04g1063 Tc01_g003540 .
. . Gorai.003G016400.1 .
Vv18g1043 . . . . . . .
. . . . . . . .
. . . . .
Vv18g1044 . . . . . . .
. . . . . . . .
. . . . .

Vv18g1045 . . . . . . .
. . . . . . .
. . . . .
Vv18g1046 . . . . . . .
. . . . . . .
. . . . .
Vv18g1047 Tc08_g001810 Gorai.002G129200.1 . .
. . . . . . .
. . . . . . .
Vv18g1048 . . . . . . .
. . . . . . .
. . . . .
Vv18g1049 Tc08_g012870 . . . . .
. . . . . . .
. . . . . .
Vv18g1050 . . . Gorai.009G204000.1 .
. . . . . . .
. . . . . . .
Vv18g1051 . . . . . . .
. . . . . . .
. . . . .
Vv18g1052 Tc08_g001820 . . . . .
. . . . . . .
. . . . . .
Vv18g1053 . . . Gorai.009G203800.1 .
Gorai.010G011200.1 . . . . .
. . Vv04g1067 Tc01_g003520 . .
Gorai.007G205300.1 Gorai.003G016200.1 .
Vv18g1054 Tc08_g001830 Gorai.002G129100.1 . .
. . . . . . .
. . . . . . .
Vv18g1055 . . . . . . .
. . . . . . .
. . . . .
Vv18g1056 Tc08_g001850 Gorai.002G128900.1 Gorai.
005G107200.1 . Gorai.009G273800.1 . . .
. . . . . . .
. . . .
Vv18g1057 . . . . . . .
. . . . . . .
. . . . .
Vv18g1058 Tc08_g001860 . . Gorai.009G203700.1
. . . . . . .
. . . . . . .
Vv18g1059 Tc08_g001870 . . Gorai.009G203600.1
. Gorai.010G011100.1 . . . .
. . . . . . .
. .
Vv18g1060 Tc08_g001880 Gorai.002G128800.1 . .
. . . . . . .

. . . . . . . .
Vv18g1061 Tc08_g001890 . Gorai.005G107300.1
Gorai.009G203500.1 Gorai.009G273700.1 Gorai.010G010900.1
. . . . . . . .
. . . . . .
Vv18g1062 Tc08_g001900 . Gorai.005G107400.1 .
. Gorai.010G010800.1 . . . .
. . . . . . . .
. .
Vv18g1063 Tc08_g001910 . . Gorai.009G203400.1
Gorai.009G273600.1 . . . . .
. . . Vv04g1077 Tc01_g003470 .
. Gorai.007G205100.1 Gorai.003G016100.1 .
Vv18g1064 Tc08_g001920 Gorai.002G128700.1 .
Gorai.009G203300.1 . . . . . .
. . . Vv04g1080 Tc01_g003450 .
Gorai.008G068600.1 . . .
Vv18g1065 . . . . . . .
. . . . . . . .
. . . . .
Vv18g1066 Tc08_g001930 . . Gorai.009G203200.1
. Gorai.010G010700.1 . . . .
. . . Vv04g1081 Tc01_g003430 .
. Gorai.007G204600.1 Gorai.003G015900.1 .
Vv18g1067 Tc08_g001940 Gorai.002G128600.1 . .
. . . . . . . .
. . . . . . . .
Vv18g1068 . Gorai.002G128500.1 . . .
. . . . . . . .
. . . . . . .
Vv18g1069 . . . Gorai.009G203100.1 .
. . . . . . . .
. . . . . . .
Vv18g1070 . . . . . . .
. . . . . . . .
. . . . .
Vv18g1071 . . . . . . .
. . . . . . . .
. . . . .
Vv18g1072 . . . . . . .
. . . . . . . .
. . . . .
Vv18g1073 Tc08_g001960 Gorai.002G128400.1 .
Gorai.009G203000.1 . . . . . .
. . . . . . . .
. .
Vv18g1074 Tc08_g001970 Gorai.002G128300.1 . .
Gorai.009G273500.1 . . . . .
. . . . . . . .
. .

Vv18g1075 Tc08_g001980 . . Gorai.009G202900.1
. . Vv03g0480 Tc02_g007080 .
Gorai.005G243100.1 . . . . . .
. . . .
Vv18g1076 Tc08_g001990 . Gorai.005G107800.1
Gorai.009G202800.1 . Gorai.010G010600.1 . .
. . . . . . . .
. . . .
Vv18g1077 . . . . . . .
. . . . . . . .
. . . . .
Vv18g1078 . . . . . . .
. . . . . . . .
. . . . .
Vv18g1079 Tc08_g002030 . . Gorai.009G202400.1
. . Vv03g0475 Tc02_g007030 Gorai.
013G037200.1 . . . . . . .
. . . .
Vv18g1080 Tc08_g002050 Gorai.002G128000.1 .
Gorai.009G202100.1 . . . . . .
. . . . . . . .
. .
Vv18g1081 Tc08_g002060 . . Gorai.009G202000.1
. . . . . . . .
. . . . . . . .
Vv18g1082 Tc08_g002070 . . Gorai.009G201900.1
. Gorai.010G010300.1 . . . .
. . . . . . . .
. .
Vv18g1083 Tc08_g002080 . . . . .
. . . . . . . .
. . . . . .
Vv18g1084 Tc08_g002090 Gorai.002G127900.1 . .
. . . . . . . .
. . . . . . . .
Vv18g1085 Tc08_g002100 . . Gorai.009G201800.1
. Gorai.010G010200.1 . . . .
. . . . . . . .
. .
Vv18g1086 . . . . . . .
. . . . . . . .
. . . . .
Vv18g1087 Tc08_g002130 Gorai.002G127700.1 . .
. Gorai.010G010100.1 . . . .
. . . . . . . .
. .
Vv18g1088 Tc08_g002140 . . Gorai.009G201700.1
Gorai.009G273300.1 Gorai.010G010000.1 . .
. . . . . . . .
. . . .

Vv18g1089 Tc08_g002150 . Gorai.005G107900.1 .
Gorai.009G273200.1 . . . . .
. . . Vv04g1117 Tc01_g003180 .
. . Gorai.003G014500.1 .
Vv18g1090 Tc08_g002160 . Gorai.005G108000.1
Gorai.009G201600.1 . . Vv03g0464
Tc02_g006950 . Gorai.005G244100.1 . . .
. . . . . . .
Vv18g1091 . . . . . . .
. . . . . . . .
. . . . .
Vv18g1092 Tc08_g002170 . Gorai.005G108100.1 .
. Gorai.010G009900.1 . . . .
. . . Vv04g1123 Tc01_g003130
Gorai.007G050600.1 . Gorai.007G202700.1 . .
Vv18g1093 . . . . . . .
. . . . . . . .
. . . . .
Vv18g1094 Tc08_g002200 Gorai.002G127500.1 .
Gorai.009G201300.1 . . . . . .
. . . Vv04g1125 Tc01_g003110
Gorai.007G050500.1 . Gorai.007G202500.1 Gorai.
003G014100.1 .
Vv18g1095 Tc08_g002220 Gorai.002G127300.1 .
Gorai.009G201200.1 Gorai.009G273000.1 . Vv03g0461
Tc02_g006880 Gorai.013G036700.1 . . .
Gorai.012G155400.1 . . . . .
. .
Vv18g1096 Tc08_g002230 Gorai.002G127200.1 . .
. . . . . . . .
. Vv04g1129 Tc01_g003080 . .
Gorai.007G202100.1 Gorai.003G014000.1 Gorai.011G101500.1
Vv18g1097 Tc08_g002240 . . . .
Gorai.010G009700.1 . . . . . .
. . . . . . . .
Vv18g1098 Tc08_g002250 . . . .
Gorai.010G009600.1 . . . . . .
. . . . . . . .
Vv18g1099 Tc08_g002260 . Gorai.005G108300.1 .
. Gorai.010G009500.1 . . . .
. . . . . . . .
. .
Vv18g1100 Tc08_g002270 . . Gorai.009G201100.1
Gorai.009G272900.1 Gorai.010G009400.1 . .
. . . . . Vv04g1132
Tc01_g003050 Gorai.007G050300.1 Gorai.008G067400.1 .
. Gorai.011G101400.1
Vv18g1101 Tc08_g002280 . . Gorai.009G201000.1
Gorai.009G272700.1 . Vv03g0454
Tc02_g006830 . Gorai.005G244300.1 . . .

. . . . . . .
Vv18g1102 Tc08_g002290 Gorai.002G127100.1 .
Gorai.009G200900.1 Gorai.009G272400.1 . . .
. . . . . . . .
. . . .
Vv18g1103 Tc08_g002300 Gorai.002G127000.1 . .
. . Vv03g0450 . . . .
. . . . . . . .
.
Vv18g1104 Tc08_g002320 . . . .
Gorai.010G009200.1 Vv03g0441 Tc02_g006720 Gorai.
013G035900.1 Gorai.005G244700.1 . Gorai.008G029700.1
. Vv04g1140 Tc01_g002960 . . .
Gorai.003G013600.1 .
Vv18g1105 . Gorai.002G126800.1 . . .
. . . . . . . .
. . . . . . .
Vv18g1106 Tc08_g002330 . . . . .
. . . . . . .
Vv04g1142 Tc01_g002940 . . Gorai.007G201900.1
Gorai.003G013500.1 .
Vv18g1107 Tc08_g002340 . . Gorai.009G200800.1
Gorai.009G272300.1 . . . . .
. . . . . . . .
. .
Vv18g1108 Tc08_g002350 Gorai.002G126700.1 . .
Gorai.009G272200.1 Gorai.010G009100.1 . .
. . . . . . . .
. . . .
Vv18g1109 Tc08_g002360 . . . .
Gorai.010G009000.1 . . . . . .
. Vv04g1144 . . . Gorai.
007G201800.1 Gorai.003G013400.1 .
Vv18g1110 Tc08_g002370 . . Gorai.009G200700.1
. . . . . . . .
. . . . . . . .
Vv18g1111 . . . . . . .
. . . . . . Vv04g1145
. . . . . .
Vv18g1112 . . . . . . .
. . . . . . . .
. . . . .
Vv18g1113 Tc08_g002380 . . Gorai.009G200600.1
. Gorai.010G008900.1 . . . .
. . . . . . . .
. .
Vv18g1114 . . . . . . .
. . . . . . . .
. . . . .
Vv18g1115 Tc08_g002390 . . . .

Gorai.010G008800.1 . . . . . .
. . . . . . . .
Vv18g1116 . . . . . . .
. . . . . . . .
. . . . .
Vv18g1117 Tc08_g002400 . . Gorai.009G200500.1
. . . . . . . .
. Vv04g1149 Tc01_g002880 . .
Gorai.007G201600.1 . .
Vv18g1118 Tc08_g002410 . . Gorai.009G200400.1
. Gorai.010G008700.1 Vv03g0436
Tc02_g006690 Gorai.013G035800.1 Gorai.005G244900.1 .
. . . . . . . .
.
Vv18g1119 Tc08_g002420 . . Gorai.009G200300.1
. . . . . . . .
. . . . . . . .
Vv18g1120 Tc08_g002440 . . . Gorai.
009G272000.1 . . . . . . .
. . . . . . . .
Vv18g1121 Tc08_g002450 Gorai.002G126600.1 Gorai.
005G109100.1 Gorai.009G200200.1 Gorai.009G271900.1 .
. . . . . . .
Vv04g1152 Tc01_g002860 . Gorai.008G066400.1
Gorai.007G201500.1 . Gorai.011G100900.1
Vv18g1122 Tc08_g002460 . . . . .
. . . . . . . .
. . . . . .
Vv18g1123 . . . . . . .
. . . . . . Vv04g1154
Tc01_g002800 . Gorai.008G066300.1 . .
.
Vv18g1124 Tc08_g002470 . . Gorai.009G200100.1
. Gorai.010G008500.1 Vv03g0433
Tc02_g006610 . Gorai.005G245200.1 . . .
Vv04g1159 . . Gorai.008G066100.1 .
. .
Vv18g1125 . . . . . . .
. . . . . . Vv04g1161
. . . . . .
Vv18g1126 Tc08_g002490 . . . . .
. . . . . . .
Vv04g1163 Tc01_g002770 . . . . .
Vv18g1127 . . . . . . .
. . . . . . . .
. . . . .
Vv18g1128 Tc08_g002500 . . Gorai.009G200000.1
. Gorai.010G008400.1 . . . .
. . . . . . . .
. .

Vv18g1129 . . . . . .
Vv03g0431 Tc02_g006560 . Gorai.005G245400.1 .
. . . . . . . .
.
Vv18g1130 . . . . . .
Vv03g0430 . . . . . . .
. . . . . .
Vv18g1131 Tc08_g002530 . Gorai.005G109200.1
Gorai.009G199800.1 . . Vv03g0428
Tc02_g006540 Gorai.013G035400.1 Gorai.005G245500.1 .
Gorai.008G030300.1 Gorai.012G155100.1 Vv04g1184
Tc01_g002610 . . Gorai.007G200700.1
Gorai.003G013000.1 Gorai.011G100700.1
Vv18g1132 Tc08_g002540 . Gorai.005G109300.1
Gorai.009G199700.1 . Gorai.010G008300.1 . .
. . . . . . . .
. . . .
Vv18g1133 Tc08_g002550 . . Gorai.009G199600.1
. Gorai.010G008200.1 . . . .
. . . . . . . .
. .
Vv18g1134 Tc08_g002560 . . Gorai.009G199500.1
. . . . . . . .
. . . . . . . .
Vv18g1135 Tc08_g002570 . . . .
Gorai.010G008100.1 . . . . . .
. . . . . . . .
Vv18g1136 . . . . . . .
. . . . . . . .
. . . . .
Vv18g1137 Tc08_g002580 . . . . .
. . . . . . . .
. . . . . .
Vv18g1138 Tc08_g002590 . Gorai.005G109500.1
Gorai.009G199400.1 . . . . . .
. . . . . . . .
. .
Vv18g1139 Tc08_g002600 Gorai.002G126500.1 Gorai.
005G109600.1 Gorai.009G199300.1 . Gorai.010G008000.1
Vv03g0425 Tc02_g006520 Gorai.013G035200.1
Gorai.005G245700.1 . . Gorai.012G155000.1
Vv04g1187 Tc01_g002580 . Gorai.008G065300.1
Gorai.007G200600.1 . .
Vv18g1140 Tc08_g002610 . . . .
Gorai.010G007900.1 . . . . . .
. . . . . . . .
Vv18g1141 Tc08_g002620 . Gorai.005G109800.1
Gorai.009G199100.1 . . Vv03g0421 .
Gorai.013G034900.1 . . Gorai.008G030700.1 .
. . . . . . .

Vv18g1142 Tc08_g002630 . . . Gorai.
009G271700.1 . . . . . . .
. . . . . . . .
Vv18g1143 Tc08_g002650 . . Gorai.009G199000.1
. . . . . . . .
. . . . . . . .
Vv18g1144 . . . . . . .
. . . . . . . .
. . . . .
Vv18g1145 Tc08_g002660 . Gorai.005G109900.1
Gorai.009G198900.1 Gorai.009G271600.1 . . .
. . . . . . . .
. . . .
Vv18g1146 Tc08_g002670 . . Gorai.009G198800.1
. . Vv04g1197 Tc01_g002520 . .
Gorai.007G199800.1 Gorai.003G012600.1 . .
. . . . . .
Vv18g1147 Tc08_g002680 . . Gorai.009G198700.1
. . . . . . . .
. . . . . . . .
Vv18g1148 . . . . . . .
. . . . . . . .
. . . . .
Vv18g1149 Tc08_g002690 . . Gorai.009G198600.1
. . . . . . . .
. . . . . . . .
Vv18g1150 Tc08_g002700 . . . .
Gorai.010G007800.1 Vv03g0006 Tc02_g007610 Gorai.
013G034600.1 Gorai.005G246400.1 . Gorai.008G031100.1
. . . . . . . .
Vv18g1151 Tc08_g002710 . . Gorai.009G198500.1
. Gorai.010G007700.1 . . . .
. . . . . . . .
. .
Vv18g1152 . . . . . . .
. . . . . . . .
. . . . .
Vv18g1153 Tc08_g002730 . . . .
Gorai.010G007600.1 . . . . . .
. . . . . . . .
Vv18g1154 . Gorai.002G126400.1 . Gorai.
009G198400.1 . . . . . .
. . Vv04g1217 . . . .
Gorai.003G012200.1 .
Vv18g1155 . . . . . . .
. . . . . . . .
. . . . .
Vv18g1156 Tc08_g002740 Gorai.002G126300.1 .
Gorai.009G198300.1 . Gorai.010G007500.1 . .
. . . . . . . .

```
        .       .       .       .
Vv18g1157       Tc08_g002750    .       .       Gorai.009G198200.1
        .       Gorai.010G007400.1      .       .       .       .
        .       .       .       .       .       .       .       .
        .       .
Vv18g1158       Tc08_g002760    .       .       .       .       .
        .       .       .       .       .       .       .       .
        .       .       .       .       .       .
Vv18g1159       Tc08_g002770    .       .       Gorai.009G198100.1
        .       Gorai.010G007300.1      .       .       .       .
        .       .       .       .       .       .       .       .
        .       .
Vv18g1160       .       .       .       .       .       .       .
        .       .       .       .       .       .       .       .
        .       .       .       .       .
Vv18g1161       .       .       .       .       .       .       .
        .       .       .       .       .       .       .       .
        .       .       .       .       .
Vv18g1162       .       .       .       .       .       .       .
        .       .       .       .       .       .       .       .
        .       .       .       .       .
Vv18g1163       .       .       .       .       .       .       .
        .       .       .       .       .       .       .       .
        .       .       .       .       .
Vv18g1164       .       .       .       .       .       .
Vv03g0011       Tc02_g007580    .       Gorai.005G246600.1      .
        .       .       Vv04g1226       Tc01_g002380    .       .
        Gorai.007G198900.1      Gorai.003G011900.1      .
Vv18g1165       Tc08_g002780    Gorai.002G126200.1      Gorai.
005G110000.1    .       .       .       .       .       .
        .       .       .       Vv04g1227       Tc01_g002370    .
        .       Gorai.007G198800.1      .       Gorai.011G100200.1
Vv18g1166       Tc08_g002790    Gorai.002G126100.1      .
Gorai.009G198000.1      .       Gorai.010G007200.1      .       .
        .       .       .       .       .       .       .       .
        .       .       .       .
Vv18g1167       Tc08_g002800    .       .       Gorai.009G197900.1
        .       .       .       .       .       .       .       .
        .       .       .       .       .       .       .       .
Vv18g1168       Tc08_g002810    Gorai.002G126000.1      .       .
        .       .       .       .       .       .       .       .
        .       .       .       .       .       .       .       .
Vv18g1169       .       .       .       .       .       .       .
        .       .       .       .       .       .       .       .
        .       .       .       .       .
Vv18g1170       .       .       .       .       Gorai.009G271500.1
        .       .       .       .       .       .       .       .
        .       .       .       .       .       .       .
Vv18g1171       Tc08_g002820    .       .       .       .       .
        .       .       .       .       .       .       .       .
```

. . . . . .
Vv18g1172 . . . . . . .
. . . . . . . .
. . . . .
Vv18g1173 Tc08_g002830 . Gorai.005G110100.1
Gorai.009G197800.1 Gorai.009G271400.1 . . .
. . . . . . . .
. . . .
Vv18g1174 Tc08_g002840 . . Gorai.009G197700.1
. . Vv03g0015 . Gorai.013G034000.1
Gorai.005G246800.1 . . . . .
. . . . .
Vv18g1175 Tc08_g002850 . . Gorai.009G197600.1
. Gorai.010G007100.1 Vv03g0016
Tc02_g007550 . . . . . . .
. . . . .
Vv18g1176 . . . . . . .
. . . . . . . .
. . . . .
Vv18g1177 . . . . . . .
. . . . . . . .
. . . . .
Vv18g1178 Tc08_g002860 . . . Gorai.
009G271300.1 . . . . . . .
. . . . . . . .
Vv18g1179 Tc08_g002870 Gorai.002G125900.1 .
Gorai.009G197500.1 . Gorai.010G006900.1 . .
. . . . . Vv04g1230
Tc01_g002360 . . Gorai.007G198700.1 .
Gorai.011G100100.1
Vv18g1180 Tc08_g002880 Gorai.002G125800.1 . .
Gorai.009G271200.1 . . . . .
. . . . . . . .
. .
Vv18g1181 . . Gorai.005G110200.1 Gorai.
009G197400.1 . . . . . . .
. . . . . . . .
.
Vv18g1182 . . . . . . .
. . . . . . . .
. . . . .
Vv18g1183 Tc08_g002890 . . . . .
. . . . . . . .
. . . . . .
Vv18g1184 . . . . . . .
. . . . . . . .
. . . . .
Vv18g1185 . . . . . .
Vv03g0020 Tc02_g007540 Gorai.013G033800.1 Gorai.
005G247000.1 . . . . . . .

. . .
Vv18g1186 Tc08_g002900 Gorai.002G125700.1 . .
. . Vv03g0021 Tc02_g007530 Gorai.
013G033700.1 . Gorai.002G004800.1 . . .
. . . . . .
Vv18g1187 . . . . . . .
. . . . . . . .
. . . . .
Vv18g1188 Tc08_g002910 Gorai.002G125600.1 Gorai.
005G110600.1 Gorai.009G197300.1 . Gorai.010G006800.1
Vv03g0023 Tc02_g007510 Gorai.013G033500.1
Gorai.005G247100.1 . . . Vv04g1235
Tc01_g002320 . . Gorai.007G198100.1 Gorai.
003G011800.1 .
Vv18g1189 Tc08_g002920 . . . . .
. . . . . . . .
. . . . . .
Vv18g1190 Tc08_g002940 Gorai.002G125500.1 .
Gorai.009G197200.1 . . . . . .
. . . . . . . .
. .
Vv18g1191 Tc08_g002950 . . . Gorai.
009G271100.1 . . . . . . .
. Vv04g1240 Tc01_g002250 . Gorai.
008G064900.1 . Gorai.003G011500.1 .
Vv18g1192 . Gorai.002G125300.1 . . .
. . . . . . . .
Vv04g1241 Tc01_g002240 . . Gorai.
007G198000.1 . .
Vv18g1193 Tc08_g002960 . . . . .
. . . . . . . .
. . . . . .
Vv18g1194 Tc08_g002970 . . . .
Gorai.010G006700.1 . . . . . .
. . . . . . . .
Vv18g1195 Tc08_g002980 . Gorai.005G110700.1
Gorai.009G197000.1 . Gorai.010G006600.1 Vv03g0025
Tc02_g007440 Gorai.013G033400.1 . Gorai.
002G004700.1 . Gorai.012G154400.1 Vv04g1263
Tc01_g002070 . Gorai.008G064100.1 Gorai.007G196900.1
Gorai.003G011200.1 Gorai.011G099600.1
Vv18g1196 . . . . . .
Vv03g0034 Tc02_g007310 . Gorai.005G247800.1
Gorai.002G004300.1 . . . . . .
. . .
Vv18g1197 . . . . . . .
. . . . . . . .
. . . . .
Vv18g1198 . . . . . . .
. . . . . . . .

. . . . .
Vv18g1199 Tc08_g003020 Gorai.002G125100.1 .
Gorai.009G196800.1 . . . . . .
. . . . . . . .
. .
Vv18g1200 . . . . . . .
. . . . . . . .
. . . . .
Vv18g1201 . . . . . . .
. . . . . . Vv04g1265
Tc01_g002040 . Gorai.008G063800.1 Gorai.
007G196800.1 . .
Vv18g1202 Tc08_g003030 . . Gorai.009G196700.1
. Gorai.010G006400.1 Vv03g0052
Tc02_g006380 . Gorai.005G248400.1 Gorai.002G004600.1
. Gorai.012G154300.1 . . . .
. . .
Vv18g1203 Tc08_g003050 . . . Gorai.
009G271000.1 Gorai.010G006200.1 . . . .
. . . . . . . .
. .
Vv18g1204 Tc08_g003060 . . Gorai.009G196500.1
Gorai.009G270900.1 . . . . .
. . . . . . . .
. .
Vv18g1205 . . . . . . .
. . . . . . . .
. . . . .
Vv18g1206 Tc08_g003070 . . . .
Gorai.010G006100.1 Vv03g0060 Tc02_g006320 .
Gorai.005G248800.1 Gorai.002G004000.1 . Gorai.
012G154000.1 . . . . . . .
Vv18g1207 Tc08_g003080 . Gorai.005G110900.1 .
. . . . . . . .
. . . . . . . .
Vv18g1208 . . . . . . .
. . . . . . . .
. . . . .
Vv18g1209 Tc08_g003090 . . . .
Gorai.010G006000.1 . . . . . .
. . . . . . . .
Vv18g1210 Tc08_g003110 Gorai.002G125000.1 .
Gorai.009G196400.1 Gorai.009G270800.1 . . .
. . . . . . . .
. . . .
Vv18g1211 . . . . . . .
. . . . . . . .
. . . . .
Vv18g1212 . . Gorai.005G111200.1 . .
. . . . . . . .

Vv04g1273 Tc01_g001970 . . . .
.
Vv18g1213 Tc08_g003130 . . . . .
. . . . . . . .
. . . . . .
Vv18g1214 Tc08_g003140 Gorai.002G124900.1 . .
. . . . . . . .
. . . . . . . .
Vv18g1215 . . . . . . .
. . . . . . . .
. . . . .
Vv18g1216 . . . . . . .
. . . . . . . .
. . . . .
Vv18g1217 Tc08_g003160 . . . . .
Vv03g0064 Tc02_g006270 . Gorai.005G248900.1
. . . . . . . .
. .
Vv18g1218 Tc08_g003170 . . . Gorai.
009G270600.1 Gorai.010G005900.1 . . . .
. . . . . . . .
. .
Vv18g1219 . . . . . . .
. . . . . . . .
. . . . .
Vv18g1220 Tc08_g003180 . . . . .
. . . . . . . .
. . . . . .
Vv18g1221 Tc08_g003190 . . . . .
. . . . . . . .
. . . . . .
Vv18g1222 Tc08_g003200 . . . . .
. . . . . . . .
. . . . . .
Vv18g1223 Tc08_g003220 . . . . .
. . . . . . . .
. . . . . .
Vv18g1224 Tc08_g003250 . . . . .
. . . . . . . .
. . . . . .
Vv18g1225 Tc08_g003270 . . . . .
. . . . . . . .
. . . . . .
Vv18g1226 . . . . . . .
. . . . . . . .
. . . . .
Vv18g1227 . . . . . . .
. . . . . . . .
. . . . .
Vv18g1228 Tc08_g003290 . . Gorai.009G196300.1

. . . . . . . .
. . . . . . . .
Vv18g1229 . . . . . . .
. . . . . . . .
. . . . .
Vv18g1230 . . . . . . .
. . . . . . . .
. . . . .
Vv18g1231 . . . . . . .
. . . . . . . .
. . . . .
Vv18g1232 Tc08_g003310 Gorai.002G124800.1 .
Gorai.009G196200.1 . Gorai.010G005800.1 . .
. . . . . . . .
. . . .
Vv18g1233 Tc08_g003320 Gorai.002G124700.1 Gorai.
005G111400.1 . . Gorai.010G005700.1 . .
. . . . . Vv04g1281
Tc01_g001920 . . Gorai.007G196000.1 . .
Vv18g1234 Tc08_g003330 . Gorai.005G111500.1 .
. . . . . . . .
. . . . . . . .
Vv18g1235 Tc08_g003340 Gorai.002G124600.1 . .
. . . . . . . .
. . . . . . . .
Vv18g1236 . . . . . . .
. . . . . . . .
. . . . .
Vv18g1237 Tc08_g003360 Gorai.002G124500.1 . .
. Gorai.010G005600.1 . . . .
. . . . . . . .
. .
Vv18g1238 Tc08_g003370 Gorai.002G124400.1 .
Gorai.009G196100.1 . . . . . .
. . . . . . . .
. .
Vv18g1239 . . . . . . .
. . . . . . . .
. . . . .
Vv18g1240 Tc08_g003380 . Gorai.005G111600.1 .
. . . . . . . .
. . . . . . . .
Vv18g1241 . Gorai.002G124300.1 . Gorai.
009G196000.1 Gorai.009G270500.1 Gorai.010G005500.1 .
. . . . . . . .
. . . . .
Vv18g1242 Tc08_g003390 Gorai.002G124100.1 .
Gorai.009G195900.1 . Gorai.010G005400.1 . .
. . . . . Vv04g1285
Tc01_g001880 Gorai.007G048300.1 . . . .

Vv18g1243 . Gorai.002G124000.1 . Gorai.
009G195800.1 Gorai.009G270400.1 Gorai.010G005300.1 .
. . . . . . . .
. . . . .
Vv18g1244 . . . . . . .
. . . . . . . .
. . . . .
Vv18g1245 Tc08_g003400 . . . . .
. . . . . . . .
. . . . . .
Vv18g1246 Tc08_g003410 . . Gorai.009G195700.1
. . . . . . . .
. . . . . . . .
Vv18g1247 . . . . . . .
. . . . . . . .
. . . . .
Vv18g1248 . . . . . .
Vv03g0074 . . . . . . .
. . . . . .
Vv18g1249 . . . Gorai.009G195600.1
Gorai.009G270300.1 . . . . . .
. . . . . . . .
.
Vv18g1250 Tc08_g003430 Gorai.002G123800.1 .
Gorai.009G195500.1 . . . . . .
. . . . . . . .
. .
Vv18g1251 . . . . . . .
. . . . . . . .
. . . . .
Vv18g1252 Tc08_g003440 Gorai.002G123700.1 .
Gorai.009G195400.1 . Gorai.010G005200.1 . .
. . . . . Vv04g1290 .
. . Gorai.007G195700.1 . .
Vv18g1253 . . . . . . .
. . . . . . . .
. . . . .
Vv18g1254 . . . . . . .
. . . . . . . .
. . . . .
Vv18g1255 . . . . . . .
. . . . . . . .
. . . . .
Vv18g1256 Tc08_g003470 . . . . .
Vv03g0106 . . . . . .
. . . . . . .
Vv18g1257 . . . . . . .
. . . . . . . .
. . . . .
Vv18g1258 . . . . . . .

. . . . . . . .
. . . . .
Vv18g1259 . . . . . . .
. . . . . . . .
. . . . .
Vv18g1260 . . . Gorai.009G195300.1 .
Gorai.010G005100.1 Vv03g0107 . . .
. . . . . . . .
. .
Vv18g1261 . . . . . . .
. . . . . . . .
. . . . .
Vv18g1262 . . . . . . .
. . . . . . . .
. . . . .
Vv18g1263 Tc08_g003490 . . Gorai.009G195100.1
. . . . . . . .
. . . . . . . .
Vv18g1264 Tc08_g003500 Gorai.002G123600.1 .
Gorai.009G195000.1 . . . . . .
. . . . . . . .
. .
Vv18g1265 . . . . . . .
. . . . . . . .
. . . . .
Vv18g1266 . . . . . . .
. . . . . . . .
. . . . .
Vv18g1267 . . . . . . .
. . . . . . . .
. . . . .
Vv18g1268 . . . . . . .
. . . . . . . .
. . . . .
Vv18g1269 Tc08_g003520 . . . Gorai.
009G270100.1 Gorai.010G004800.1 . . . .
. . . Vv04g1298 Tc01_g001750 .
. Gorai.007G195200.1 Gorai.003G009800.1 .
Vv18g1270 Tc08_g003530 . . . .
Gorai.010G004700.1 . . . . . .
. . . . . . . .
Vv18g1271 . . . . . . .
. . . . . . . .
. . . . .
Vv18g1272 Tc08_g003540 . . . .
Gorai.010G004600.1 . . . . . .
. Vv04g1299 . . . . .
.
Vv18g1273 . . . . . . .
. . . . . . . .

. . . . .
Vv18g1274 Tc08_g003570 . . Gorai.009G194700.1
. Gorai.010G004400.1 . . . .
. . . . . . . .
. .
Vv18g1275 Tc08_g003580 . . . .
Gorai.010G004300.1 Vv03g0115 Tc02_g005630 Gorai.
002G192600.1 Gorai.005G252000.1 . Gorai.008G033400.1
. . . . . . . .
Vv18g1276 Tc08_g003590 Gorai.002G123500.1 . .
. . Vv03g0116 Tc02_g005620 .
Gorai.005G252100.1 . . . . . .
. . . .
Vv18g1277 Tc08_g003600 . . Gorai.009G194600.1
Gorai.009G270000.1 . . . . .
. . . . . . . .
. .
Vv18g1278 . . . . . . .
. . . . . . . .
. . . . .
Vv18g1279 Tc08_g003610 . . . .
Gorai.010G004200.1 . . . . . .
. . . . . . . .
Vv18g1280 Tc08_g003620 . . . Gorai.
009G269900.1 . . . . . . .
. . . . . . . .
Vv18g1281 . . . . . . .
. . . . . . . .
. . . . .
Vv18g1282 Tc08_g003640 Gorai.002G123400.1 .
Gorai.009G194500.1 Gorai.009G269800.1 Gorai.010G003800.1
. . . . . . . .
. . . . . .
Vv18g1283 Tc08_g003650 Gorai.002G123300.1 . .
. . . . . . . .
. . . . . . . .
Vv18g1284 . . . . . . .
. . . . . . . .
. . . . .
Vv18g1285 . . . . . .
Vv03g0156 Tc02_g005490 Gorai.002G193600.1 .
Gorai.002G003800.1 Gorai.008G034100.1 . . .
. . . . .
Vv18g1286 . . . . . . .
. . . . . . . .
. . . . .
Vv18g1287 Tc08_g003660 . Gorai.005G111800.1
Gorai.009G194400.1 . Gorai.010G003300.1 . .
. . . . . . . .
. . . .

Vv18g1288 Tc08_g003690 Gorai.002G123200.1 . .
. . . . . . . .
. . . . . . . .
Vv18g1289 . . . . . . .
. . . . . . . .
. . . . .
Vv18g1290 . . . . . . .
. . . . . . . .
. . . . .
Vv18g1291 . . . Gorai.009G194200.1 .
. Vv04g1312 Tc01_g001610 . Gorai.
008G062600.1 Gorai.007G193800.1 . . . .
. . . . .
Vv18g1292 Tc08_g003720 Gorai.002G123100.1 . .
. . . . . . . .
. . . . . . . .
Vv18g1293 . . . . . . .
. . . . . . . .
. . . . .
Vv18g1294 Tc08_g003730 Gorai.002G122900.1 Gorai.
005G112000.1 Gorai.009G194100.1 . Gorai.010G003100.1
Vv04g1314 Tc01_g001590 Gorai.007G048900.1
Gorai.008G062300.1 Gorai.007G193600.1 Gorai.003G009200.1
. . . . . . . .
Vv18g1295 Tc08_g003740 . . Gorai.009G194000.1
. . Vv04g1315 Tc01_g001580 . .
Gorai.007G193400.1 . . . . .
. . . .
Vv18g1296 Tc08_g003750 Gorai.002G122800.1 . .
. Gorai.010G003000.1 . . . .
. . . . . . . .
. .
Vv18g1297 . . . . . . .
. . . . . . . .
. . . . .
Vv18g1298 . . . . . . .
. . . . . . . .
. . . . .
Vv18g1299 . . . . . . .
. . . . . . . .
. . . . .
Vv18g1300 . . . . . . .
. . . . . . . .
. . . . .
Vv18g1301 Tc08_g003770 . . Gorai.009G193900.1
. . . . . . . .
. . . . . . . .
Vv18g1302 . . . . . . .
. . . . . . . .
. . . . .

Vv18g1303 . . . . . . .
. . . . . . .
. . . . .
Vv18g1304 Tc08_g003800 . . Gorai.009G193700.1
. . . . . . . .
. . . . . . . .
Vv18g1305 . . Gorai.005G112200.1 . .
. . . . . . .
. . . . . . .
Vv18g1306 . . . . . . .
. . . . . . .
. . . . .
Vv18g1307 Tc08_g003840 . . Gorai.009G193600.1
Gorai.009G269700.1 . . . . .
. . . . . . .
. .
Vv18g1308 . . . . . . .
. . . . . . .
. . . . .
Vv18g1309 Tc08_g003850 . . Gorai.009G193500.1
. . . . . . .
. . . . . . .
Vv18g1310 . . . . . . .
. . . . . . .
. . . . .
Vv18g1311 Tc08_g003880 . . . .
Gorai.010G002700.1 . . . . . .
. . . . . . .
Vv18g1312 Tc08_g003890 . Gorai.005G112500.1
Gorai.009G193300.1 Gorai.009G269600.1 Gorai.010G002600.1
Vv03g0204 Tc02_g005020 Gorai.002G197500.1 .
. Gorai.008G036100.1 . Vv04g1352
Tc01_g001350 . . Gorai.007G192300.1 Gorai.
003G008000.1 .
Vv18g1313 Tc08_g003910 Gorai.002G122600.1 .
Gorai.009G193200.1 . . . . . .
. . . Vv04g1353 Tc01_g001330
Gorai.007G049400.1 . . . .
Vv18g1314 . . . . . . .
. . . . . . .
. . . . .
Vv18g1315 Tc08_g003920 Gorai.002G122500.1 . .
. . . . . . .
. . . . . . .
Vv18g1316 . . . . . . .
. . . . . . .
. . . . .
Vv18g1317 . . . . . . .
. . . . . . .
. . . . .

Vv18g1318 Tc08_g003940 . . Gorai.009G193000.1
. . . . . . . .
. . . . . . . .
Vv18g1319 Tc08_g003950 Gorai.002G122300.1 Gorai.
005G112600.1 Gorai.009G192800.1 Gorai.009G269400.1
Gorai.010G002400.1 Vv03g0210 Tc02_g004940 Gorai.
002G198200.1 Gorai.005G254300.1 Gorai.002G002800.1 .
. . . . . . . .
Vv18g1320 . . . . . . .
. . . . . . . .
. . . . .
Vv18g1321 . . . . . . .
. . . . . . . .
. . . . .
Vv18g1322 . . . . . . .
. . . . . . . .
. . . . .
Vv18g1323 . . . . . . .
. . . . . . . .
. . . . .
Vv18g1324 . . . . . . .
. . . . . . . .
. . . . .
Vv18g1325 . . . . . . .
. . . . . . . .
. . . . .
Vv18g1326 . . . . . . .
. . . . . . . .
. . . . .
Vv18g1327 . . . . . . .
. . . . . . . .
. . . . .
Vv18g1328 . . . . . . .
. . . . . . . .
. . . . .
Vv18g1329 . . . . . . .
. . . . . . . .
. . . . .
Vv18g1330 Tc08_g003980 . . Gorai.009G192700.1
. Gorai.010G002200.1 . . . .
. . . . . . . .
. .
Vv18g1331 . . . . . . .
. . . . . . . .
. . . . .
Vv18g1332 . . . . . .
Vv03g0212 . Gorai.002G198400.1 . . .
. . . . . . . .
Vv18g1333 . . . . . . .
. . . . . . . .

. . . . .
Vv18g1334 . . . . Gorai.009G266600.1
. . . . . . . .
. . . . . . .
Vv18g1335 Tc08_g003990 . . . .
Gorai.010G002100.1 Vv03g0214 . . . .
. . . . . . . .
.
Vv18g1336 Tc08_g004000 . . Gorai.009G192600.1
. Gorai.010G002000.1 . . . .
. . . . . . . .
. .
Vv18g1337 . . . . . . .
. . . . . . . .
. . . . .
Vv18g1338 . . . . . . .
. . . . . . . .
. . . . .
Vv18g1339 Tc08_g004010 Gorai.002G122200.1 .
Gorai.009G192500.1 . . . . . .
. . . Vv04g1359 Tc01_g001240 .
. Gorai.007G191800.1 Gorai.003G007000.1 .
Vv18g1340 Tc08_g004020 Gorai.002G121900.1 . .
. . . . . . . .
. Vv04g1360 Tc01_g001230 . .
Gorai.007G191700.1 Gorai.003G006800.1 .
Vv18g1341 Tc08_g004030 . . Gorai.009G192400.1
. . . . . . . .
. . . . . . . .
Vv18g1342 Tc08_g004040 Gorai.002G121800.1 .
Gorai.009G192300.1 . . . . . .
. . . . . . . .
. .
Vv18g1343 Tc08_g004050 Gorai.002G121700.1 . .
. . . . . . . .
. . . . . . . .
Vv18g1344 Tc08_g004130 . . . . .
. . . . . . . .
. . . . . .
Vv18g1345 . . Gorai.005G085300.1 Gorai.
009G250800.1 Gorai.009G250800.1 . . . .
. . . . . . . .
. . .
Vv18g1346 . . . . . . .
. . . . . . . .
. . . . .
Vv18g1347 . . . . . . .
. . . . . . . .
. . . . .
Vv18g1348 . . Gorai.005G085000.1 Gorai.

009G251000.1 Gorai.009G251000.1 . . . .
. . . . Vv04g1570
Tc01_g012050 . . . . .
Vv18g1349 . . Gorai.005G084900.1 . .
. . . . . . . .
. . . . . . .
Vv18g1350 . . . . . . .
. . . . . . . .
. . . . .
Vv18g1351 Tc08_g001420 . . . . .
. . . . . . . .
. . . . . .
Vv18g1352 . Gorai.002G119100.1 Gorai.005G084700.1
Gorai.009G251200.1 Gorai.009G251200.1 . .
. . . . . . . .
. . . . .
Vv18g1353 . . . . . . .
. . . . . . . .
. . . . .
Vv18g1354 . . Gorai.005G084600.1 Gorai.
009G251400.1 Gorai.009G251400.1 Gorai.010G000800.1 .
. . . . . . . .
. . . . .
Vv18g1355 . . . . . . .
. . . . . . . .
. . . . .
Vv18g1356 Tc08_g005510 . . . . .
. . . . . . . .
. . . . . .
Vv18g1357 . . Gorai.005G084500.1 . .
. . . . . . . .
. . . . . . .
Vv18g1358 . . . . . . .
. . . . . . . .
. . . . .
Vv18g1359 Tc08_g005220 . . . . .
Vv03g0234 Tc02_g004640 Gorai.002G199700.1
Gorai.005G255000.1 . Gorai.008G037200.1 Gorai.
012G156600.1 . . . . . . .
Vv18g1360 Tc08_g004870 . . . . .
. . . . . . . .
. . . . . .
Vv18g1361 . . . . . . .
. . . . . . . .
. . . . .
Vv18g1362 . . . . . . .
. . . . . . . .
. . . . .
Vv18g1363 . . . . . . .
. . . . . . . .

```
         .        .        .        .        .
Vv18g1364         .        .        .        .        .        .        .
         .        .        .        .        .        .        .        .
         .        .        .        .        .
Vv18g1365         .        .        .        .        .        .        .
         .        .        .        .        .        .        .        .
         .        .        .        .        .
Vv18g1366         .        .        .        .        .        .        .
         .        .        .        .        .        .        .        .
         .        .        .        .        .
Vv18g1367         .        .        .        .        .        .        .
         .        .        .        .        .        .        .        .
         .        .        .        .        .
Vv18g1368         .        .        .        .        .        .        .
         .        .        .        .        .        .        .        .
         .        .        .        .        .
Vv18g1369         .        .        .        Gorai.009G251500.1
Gorai.009G251500.1         .        .        .        .        .        .
         .        .        .        .        .        .        .        .
         .
Vv18g1370         .        .        .        .        .        .        .
         .        .        .        .        .        .        .        .
         .        .        .        .        .
Vv18g1371         .        .        .        .        .        .        .
         .        .        .        .        .        .        .        .
         .        .        .        .        .
Vv18g1372         .        .        .        .        .        .        .
         .        .        .        .        .        .        .        .
         .        .        .        .        .
Vv18g1373         .        .        .        .        .        .        .
         .        .        .        .        .        .        .        .
         .        .        .        .        .
Vv18g1374         .        .        .        .        .        .        .
         .        .        .        .        .        .        .        .
         .        .        .        .        .
Vv18g1375         .        .        .        .        .        .        .
         .        .        .        .        .        .        .        .
         .        .        .        .        .
Vv18g1376         .        .        Gorai.005G084100.1       .        .
         .        Vv04g1564         Tc01_g011950      .        .        .
         .        .        .        .        .        .        .        .
         .
Vv18g1377         .        .        .        Gorai.009G251600.1
Gorai.009G251600.1         .        .        .        .        .        .
         .        .        .        .        .        .        .        .
         .
Vv18g1378         .        .        .        .        .        .        .
         .        .        .        .        .        .        .        .
         .        .        .        .        .
Vv18g1379         .        .        .        .        .        .        .
```

```
        .       .       .       .       .       .       .       .
        .       .       .       .       .
Vv18g1380       .       .       Gorai.005G084000.1      .       .
        .       .       .       .       .       .       .       .
        .       .       .       .       .       .       .
Vv18g1381       Tc08_g001040    .       .       Gorai.009G251700.1
        Gorai.009G251700.1      .       .       .       .       .
        .       .       .       .       .       .       .       .
        .       .
Vv18g1382       .       .       .       .       .       .
Vv04g1556       Tc01_g038130    .       .       .       .       .
        .       .       .       .       .       .       .
Vv18g1383       .       .       Gorai.005G083600.1      .       .
        .       .       .       .       .       .       .       .
        .       .       .       .       .       .       .
Vv18g1384       .       .       Gorai.005G083500.1      .       .
        .       .       .       .       .       .       .       .
        .       .       .       .       .       .       .
Vv18g1385       .       .       Gorai.005G083300.1      Gorai.
009G251900.1    Gorai.009G251900.1      .       .       .       .
        .       .       .       .       .       .       .       .
        .       .       .
Vv18g1386       .       .       Gorai.005G083200.1      .       .
        .       Vv04g1545       Tc01_g011750    Gorai.007G065800.1
        .       .       .       .       .       .       .       .
        .       .       .
Vv18g1387       Tc08_g014600    .       .       .       .       .
        .       .       .       .       .       .       .       .
        .       .       .       .       .       .
Vv18g1388       .       .       .       .       .       .       .
        .       .       .       .       .       .       .       .
        .       .       .       .       .
Vv18g1389       .       .       Gorai.005G082800.1      .       .
        .       .       .       .       .       .       .       .
        .       .       .       .       .       .       .
Vv18g1390       .       .       Gorai.005G082700.1      .       .
        .       .       .       .       .       .       .       .
        .       .       .       .       .       .       .
Vv18g1391       .       .       .       .       .       .       .
        .       .       .       .       .       .       .       .
        .       .       .       .       .
Vv18g1392       Tc08_g004060    .       .       .       .       .
        .       .       .       .       .       .       .       .
        .       .       .       .       .       .
Vv18g1393       .       .       Gorai.005G082600.1      Gorai.
009G252200.1    Gorai.009G252200.1      .       .       .       .
        .       .       .       .       .       .       .       .
        .       .       .
Vv18g1394       Tc08_g004030    .       .       .       .       .
        Vv04g1525       Tc01_g011540    .       .       .       .
```

. . . . . . . .
Vv18g1395 . . Gorai.005G082500.1 . .
. . . . . . . .
. . . . . . .
Vv18g1396 . . . . . . .
. . . . . . . .
. . . . .
Vv18g1397 . . Gorai.005G082400.1 . .
. Vv04g1523 . . . . .
. . . . . . . .
Vv18g1398 . . . . . . .
. . . . . . . .
. . . . .
Vv18g1399 . . . . . . .
. . . . . . . .
. . . . .
Vv18g1400 . . . . . . .
. . . . . . . .
. . . . .
Vv18g1401 . . . . . . .
. . . . . . . .
. . . . .
Vv18g1402 . . . . . .
Vv04g1520 Tc01_g011470 . . . . .
. . . . . . .
Vv18g1403 . . . . . . .
. . . . . . . .
. . . . .
Vv18g1404 . . . . . . .
. . . . . . . .
. . . . .
Vv18g1405 . . . . . . .
. . . . . . . .
. . . . .
Vv18g1406 . . . Gorai.009G252400.1
Gorai.009G252400.1 . . . . . .
. . . . . . . .
.
Vv18g1407 . . . . . . .
. . . . . . . .
. . . . .
Vv18g1408 . . . . . . .
. . . . . . . .
. . . . .
Vv18g1409 . . . . . .
Vv04g1513 Tc01_g011420 Gorai.007G064800.1 . .
. Gorai.011G116600.1 . . . .
. . .
Vv18g1410 Tc08_g014730 . . . . .
. . . . . . . .

        .       .       .       .       .       .
Vv18g1411       .       .       .       .       .       .
Vv04g1511       Tc01_g011390    .       .       .       .       .
        .       .       .       .       .       .       .
Vv18g1412       Tc08_g003840    .       .       .       .       .
        .       .       .       .       .       .       .       .
        .       .       .       .       .       .
Vv18g1413       .       .       .       .       .       .       .
        .       .       .       .       .       .       .       .
        .       .       .       .       .
[truncated: 148,491 more chars]
